# A siphonous macroalgal genome suggests convergent functions of homeobox genes in algae and land plants

**DOI:** 10.1093/dnares/dsz002

**Published:** 2019-03-28

**Authors:** Asuka Arimoto, Koki Nishitsuji, Yoshimi Higa, Nana Arakaki, Kanako Hisata, Chuya Shinzato, Noriyuki Satoh, Eiichi Shoguchi

**Affiliations:** 1 Marine Genomics Unit, Okinawa Institute of Science and Technology Graduate University, Onna, Okinawa, Japan; 2 Onna Village Fisheries Cooperative, Onna, Okinawa, Japan; 3 DNA Sequencing Section, Okinawa Institute of Science and Technology Graduate University, Onna, Okinawa, Japan

**Keywords:** green seaweed genome, UTC clade, nuclear pore protein, TALE homeobox genes, segmental duplication

## Abstract

Genome evolution and development of unicellular, multinucleate macroalgae (siphonous algae) are poorly known, although various multicellular organisms have been studied extensively. To understand macroalgal developmental evolution, we assembled the ∼26 Mb genome of a siphonous green alga, *Caulerpa lentillifera*, with high contiguity, containing 9,311 protein-coding genes. Molecular phylogeny using 107 nuclear genes indicates that the diversification of the class Ulvophyceae, including *C. lentillifera*, occurred before the split of the Chlorophyceae and Trebouxiophyceae. Compared with other green algae, the TALE superclass of homeobox genes, which expanded in land plants, shows a series of lineage-specific duplications in this siphonous macroalga. Plant hormone signalling components were also expanded in a lineage-specific manner. Expanded transport regulators, which show spatially different expression, suggest that the structural patterning strategy of a multinucleate cell depends on diversification of nuclear pore proteins. These results not only imply functional convergence of duplicated genes among green plants, but also provide insight into evolutionary roots of green plants. Based on the present results, we propose cellular and molecular mechanisms involved in the structural differentiation in the siphonous alga.

## 1. Introduction

**Figure 1:**
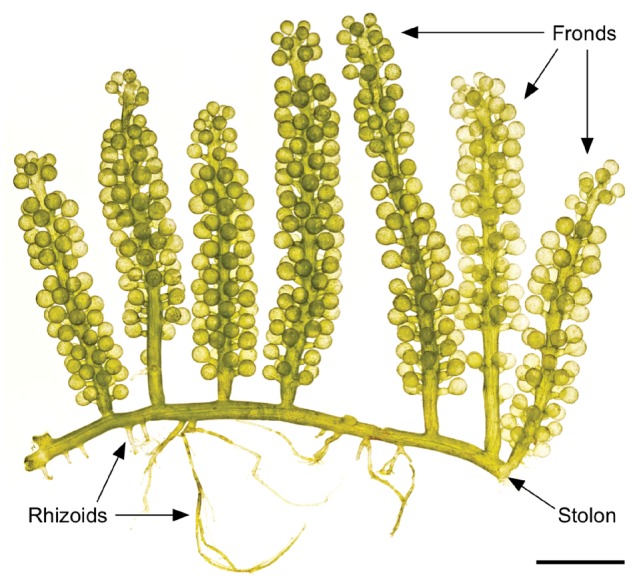
The siphonous alga, *Caulerpa lentillifera.* Cultivated *C. lentillifera* (photo by Dr Ken Maeda). The alga consists of many grape-like vesicles connected by stolons, and the entire alga comprises one cell with many nuclei. Scale bar, 10 mm.

The green alga, *Caulerpa lentillifera*, belongs to the family Caulerpaceae of the order Bryopsidales. Macroscopic morphologies observed in the Bryopsidales are composed of a large, multinucleated, single cell (Fig. 1).^1^ This type of body plan is termed siphonous, and multicellularity is not observed in this group. Some siphonous algae, including *C. lentillifera*, reach meters in size, likely being the largest single cells on earth. *C. lentillifera* possesses structures analogous to fronds (leaf-like), stolons (stem-like) and rhizoids (root-like) (Fig. 1). Frond morphologies are diverse in *Caulerpa*. *C*. *lentillifera* resembles clusters of green grapes (Fig. 1), and is commonly known as sea grapes, or umi-budo in Japanese. This alga is one of the major edible seaweeds in the subtropical/tropical Asia-Pacific region, especially cultivated for market in Okinawa, Japan.^2^

Some *Caulerpa* species become invasive, causing environmental disturbances in coastal waters worldwide.^3^ A recent report on microbial communities of marine sediments suggests that the relationship between *Caulerpa* and the microbial community is an important determinant of invasiveness.^4^ Cultivation and invasiveness depend upon asexual (vegetative) reproduction of macroscopic cells.^5^ Although multinucleate cells are also present in some clades of the Viridiplantae, including even land plants, homologies of molecular mechanisms associated with their development are little known.^6^^,^^7^

Inter- and intra-class relationships of ulvophytes, including six major orders (Bryopsidales, Cladophorales, Dasycladales, Trentepohliales, Ulotrichales and Ulvales), remain controversial,^8–10^ although all members of this class are likely included in the monophyletic UTC (classes Ulvophyceae, Trebouxiophyceae and Chlorophyceae) clade.^11^ Ulvophyceans are useful to understand evolutionary processes underlying cellularity and the diverse structural plans of green plants, because they display a variety of architectures, as well as having unicellular, multicellular and siphonous species. In addition, multicellular bodies composed of multinucleate cells are seen in the Cladophorales. It is thought that the hypothetical common ancestor was unicellular, and that siphonous body plans were not intermediate states in the development of multicellularity.^12^ These various types of morphogenesis have been studied extensively. For example, *Ulva*, which is multicellular, is one of the major experimental systems to explore mechanisms by which multicellularity evolved, as in *Volvox*.^13^ The draft genome of *Ulva mutabilis* has recently been published and provides insight into the evolution of multicellularity.^14^ The giant, mononuclear, unicellular *Acetabularia*, which belongs to the Dasycladales, has enabled elegant experiments for cutting and grafting of single cells to test whether the nucleus contains genetic materials.^15^*Caulerpa* likely provides a third experimental system among ulvophytes, since it is unicellular with multiple nuclei. As such, it should offer interesting comparisons with other major green plants, including land plants. However, no siphonous algal genome sequences were available for comparative genomic and developmental studies. Therefore, in order to explore evolution of plant structural plans and development, we decoded the genome of *C*. *lentillifera* and performed comparative genomic analyses using available green macroalgal genomes.

## 2. Materials and methods

### 2.1. Algal samples and nucleic acid extraction

*Caulerpa lentillifera*, which originated from a population in Okinawa and has been cultivated in aquaria at the Onna Village Fisheries Cooperative, Okinawa, Japan, was used in this study. It is cultivated under natural light and is harvested during the daytime. Frond specimens for DNA extraction were washed with flowing UV-sterilized seawater for 4 days after harvesting, and those for RNA extraction were rinsed with 0.22-µm-filtered seawater. Any debris was removed from washed samples using tweezers. Fronds and stolons were separated for differential gene expression analysis. Algal samples were immediately frozen in liquid nitrogen and stored at −80° C until nucleic acid extraction.

High-molecular-weight genomic DNA was prepared from isolated nuclei. Isolation of nuclei followed the protocol of Zhang et al.^16^ with some modifications. Frozen samples were ground with a mortar and pestle in liquid nitrogen and dissolved in pre-chilled nuclear isolation buffer [0.5 M sucrose, 10 mM Tris (pH 9.0), 80 mM KCl, 10 mM EDTA, 0.5% (v/v) Triton X-100, 5 mM dithiothreitol]. The purified nuclear pellet was dissolved in buffer G2 (Qiagen, 1014636). DNA was extracted using Qiagen Genomic-tips (10223, 10243). RNase and proteinase treatment and column purification followed manufacturer instructions. DNA was quantified using a dsDNA HS Assay Kit (ThermoFisher, Q32851) and DNA purity was verified with a NanoDrop 2000 Spectrophotometer (ThermoFisher). DNA integrity was checked with 0.7% (w/v) agarose gel electrophoresis.

Total RNA was extracted from ground macroscopic cells using Plant RNA reagent (ThermoFisher, 12322012) and a Qiagen RNeasy Plant Mini Kit (74904) with DNase treatment. RNA purity and quantity were verified with a NanoDrop 2000 Spectrophotometer. RNA integrity was confirmed with an Agilent 2100 Bioanalyzer.

### 2.2. Library preparation and sequencing

All sequencing libraries were prepared with protocols provided by the manufacturers, except for slight modifications described below. A whole-genome shotgun sequencing library was constructed using a KAPA Hyper Prep Kit (Kapa Biosystems, KK8502) with a PCR-free method. Genomic DNA was sheared to a target size of 550 bp using the Covaris M220 system. After Illumina-sequencing adapter ligation, fragmented DNA was size selected in 1.5% agarose gel cassettes (Sage Science, BDF1510) utilizing BluePippin (Sage Science). Two mate-pair libraries were prepared using a Nextera Mate Pair Library Prep Kit (Illumina, FC-132-1001). Gel-free and gel-based methods were used for 3-kb and 6-kb libraries, respectively. Size selection of gel-based libraries was performed with 0.75% agarose gel cassettes (Sage Science, BLF7510) and BluePippin. The library for RNA sequencing was constructed with a TruSeq Stranded mRNA Library Prep Kit (Illumina, RS-122-2101). Fragmentation of purified mRNA was performed using the manufacturer’s standard protocol. Short-read DNA and RNA libraries were sequenced using Illumina MiSeq and HiSeq platforms, respectively. BluePippin sizing of a long-read DNA library was performed using 0.75% agarose gel cassettes for adjustment of the read length to 20 kb before being loaded into a PacBio RSII sequencer.

### 2.3. Nuclear genome assembly and genome size estimation

In this study, we wished to study the *Caulerpa lentillifera* nuclear genome. To this end, we attempted to separate chloroplast genome sequences and possible contamination of bacterial sequences from nuclear sequences. First, adapter sequences and low-quality (<Q20) regions in Illumina data were removed with Trimmomatic 0.33^17^ and Sickle 1.33 (https://github.com/najoshi/sickle (6 October 2017, date last accessed)), respectively. Sequencing data from mate-pair libraries were filtered with NextClip 1.3.1.^18^

*(a) Chloroplast genome assembly and read removal:* A blastn search with default settings was performed to find PacBio reads that encode the *C*. *lentillifera rbcL* gene (accession number JN034416.1). We extracted a total of 3 Mb of longer sequences from rbcL-encoding reads. Extracted reads were assembled using sprai 0.9.9.19 (http://zombie.cb.k.u-tokyo.ac.jp/sprai/ (12 September 2016, date last accessed)) with default settings and the circularity of assembled contigs was automatically checked in the sprai pipeline. A single linear contig was constructed. To extend the contig, all PacBio reads encoding rbcL were aligned in the assembled contig using BLASR version 5.3.574e1c2^19^ and flanking sequences at both ends were extracted. These sequences were assembled with the first-round PacBio reads with the sprai assembler to obtain a finalized circular contig. Sequence accuracy of the contig was polished with Arrow software (https://github.com/PacificBiosciences/GenomicConsensus (24 July 2017, date last accessed)). All reads in the three genomic libraries that mapped onto the chloroplast genome using BWA 0.7.12,^20^ were removed.

*(b) Bacterial sequence assembly and read removal:* Remaining reads were assembled using MetaPlatanus 1.0.3 (http://platanus.bio.titech.ac.jp (4 July 2018, date last accessed)) to identify bacterial sequences. MetaPlatanus performs di-codon-based clustering by considering inter-/intra-cluster linkages. Using this method as well as GC content of each cluster, bacterial sequences were identified and removed.

*(c) The nuclear genome assembly:* The nuclear genome assembly without chloroplast and bacterial reads were generated with built-in programs in Platanus 1.2.4.^21^ Redundancy in primary assembly was removed using redundans 0.13c.^22^ After reduction, mis-assemblies were corrected using BIGMAC 5.1^23^ and contaminant sequences in the scaffolds were removed using BinSanity^24^ (commit version: f29c60). Cleaned scaffolds were treated with an iterative scaffolding pipeline employing three software packages, PBJelly in PBSuite 15.8.24,^25^ BESST 2.2.6^26^ and LINKS 1.8.5.^27^ The improved scaffolds were polished using BWA mapping of PCR-free Illumina reads and Pilon 1.22.^28^ BUSCO 3.0.2^29^ with a Eukaryota dataset and CEGMA 2.5^30^ were used to evaluate the final genome assembly. The genome size of *C. lentillifera* was calculated from k-mer histograms using Jellyfish 2.2.3^31^ and the GenomeScope web tool.^32^

### 2.4. Variant site detection and repeat analysis

The number of variant sites was determined using another sequencing library from a single specimen. After adapter and low-quality region removal, reads were mapped onto the assembled genome using BWA. Ambiguous variant sites were removed using VCFtools 0.1.15^33^ with ‘+/d = 3/q = 30’ options.

RepeatModeler 1.0.8 (http://www.repeatmasker.org/RepeatModeler (15 September 2017, date last accessed)) and RepeatMasker 4.0.6 (http://www.repeatmasker.org (15 September 2017, date last accessed)) were used to identify repeated elements in the assembled genome. Kimura substitution level of transposable elements (TEs) was predicted using utility scripts bundled with RepeatMasker.

### 2.5. Transcriptome assembly, gene prediction and annotation

Transcriptomes were assembled *de novo* using Trinity 2.1.1.^34^ In addition, STAR 2.5.2a^35^ and Trinity were combined to assemble a genome-guided transcriptome. The two types of transcriptomes were further integrated considering strand information and genome sequences using PASA 2.0.2.^36^ To create a training set for gene prediction, two additional datasets were generated. Preliminary gene models were prepared from RNA-seq mapping for genome-guided transcriptome assembly using BRAKER1 pipeline 1.9.^37^ Protein sequences in the UniProtKB/Swiss-Prot database were aligned with the genome using Exonerate 2.2.0^38^ with the ‘–percent 80’ option to find a putative conserved gene set. EVidenceModeler 1.1.1^39^ was used to create a training set with weight settings for results of PASA being 5, BRAKER1 being 2 and Exonerate being 1. Hint data about exon–exon junctions and repeat information for gene prediction were obtained with Bowtie 2 version 2.2.6^40^ combined with STAR and RepeatModeler/RepeatMasker pipeline, respectively. A final set of gene models was generated using AUGUSTUS 3.2.1^41^ with the softmasked genome and incorporated hint data after training.

Sequence similarities between *C*. *lentillifera* gene models and NCBI RefSeq protein database release 79 were explored using BLAST searches (*E*-value cut-off of 10^−5^). HMMER 3.1b2 (http://hmmer.org (4 March 2015, date last accessed)) and Pfam-A 29.0 were used with default parameters to detect protein domains in gene models. Results of BLAST and HMMER searches were incorporated into gene annotations, which are available on the genome browser at http://marinegenomics.oist.jp/gallery/. The results of the HMMER search were used to find homeodomains (Pfam accessions PF00046 and PF05920) in gene models of each species. Gene Ontology (GO) IDs were assigned using InterProScan 5.22-61.0 (https://www.ebi.ac.uk/interpro (15 March 2017, date last accessed)) with a database provided by the InterProScan distributer. Transcription factors (TF), transcription regulators (TR) and protein kinases were annotated using iTAK 1.7 alpha.^42^ Genes involved in plant hormone biosynthesis and signalling were predicted by reciprocal BLAST search analysis with an *E*-value cut-off of 10^−3^. Plant hormone-related genes in *Arabidopsis* (Ref. 43; http://www.genome.jp/kegg (23 November 2017, date last accessed); http://hormones.psc.riken.jp (6 February 2017, date last accessed.)) were employed as query sequences in the BLAST search.

### 2.6. Expression analysis

RNA-seq libraries for expression analysis were prepared from fronds and stolons of three different specimens. Expression levels of genes were based on the normalized count per million (CPM) from RNA-seq data. A trimmed mean of m-values (TMM) method was used for normalization. TMM normalization, calculation of CPM and detection of significant differences between tissues were performed using edgeR 3.20.1.^44^

### 2.7. Phylogenetic analysis

OrthoFinder 1.1.4^45^ was used to identify orthologous gene clusters with default settings. The longest protein sequences in each locus were used. Gene models of 16 genomes were employed for genome-based species tree reconstruction (Supplementary Table S1). An additional dataset was analysed to compare species tree topologies (Supplementary Table S1). Single-copy orthologous gene clusters were aligned with MAFFT 7.305^46^ under default settings and extracted gapless regions using trimAl 1.4.1^47^ with the ‘-nogaps’ option. IQ-TREE 1.5.3^48^ was used to find partitions in concatenated alignments and optimal substitution models for each partition. These optimal parameters were used for both maximum-likelihood (ML) and Bayesian analyses. A ML phylogenetic tree was inferred with 1,000 bootstrap replications using IQ-TREE. A Bayesian phylogenetic tree was created with MrBayes 3.2.6.^49^ The chain length for MCMC analysis was 1,000,000 and sampling frequency was each 1,000 generations. The first 25% of sampled trees were discarded as burn-in. Both trees were constructed using *Cyanidioschyzon merolae* as an outgroup. Topology of the tree was scored further using ASTRAL 5.5.6^50^ and BUCKy 1.4.4^51^ based on a ML method and a Bayesian method, respectively. Amino acid alignments of each single-copy gene cluster were re-used to assess concatenated tree topologies. ML trees for plant hormone-related genes were constructed as described previously.^52^ All generated trees were visualized using iTOL 3.5 (https://itol.embl.de (28 November 2017, date last accessed)).

### 2.8. Comparative gene family analysis and synteny

An UpSet plot of orthologous groups (OGs) assigned with OrthoFinder was visualized with UpSetR 1.3.3.^53^ The Dollop programme in PHYLIP 3.696^54^ was used to determine the presence or absence of OGs at ancestral nodes. Gains and losses of OGs in each clade were assumed from ancestral states based on least parsimony. The heat map of conserved OGs among green plants was visualized using the heat map function in R 3.6.0. Numbers of genes assigned within OGs were converted to robust *z*-scores defined by the median and interquartile range to calculate the relative abundances of genes.

Putative lost genes were determined by the following method. The initial dataset was a set of amino acid sequences that were not classified into the same OG with *Caulerpa lentillifera* gene models or transcriptomes that were translated to proteins. These query sequences were aligned to the *C*. *lentillifera* genome using Exonerate under default settings. Sequences aligned longer than 50% of query length were excluded as candidates of lost genes. OGs including at least one chlorophyte gene were collected. Collected sequences were assigned as *C*. *lentillifera* lost homologues. GO IDs of putative lost homologues were assigned based on Pfam domains encoded in query sequences using Pfam2GO (http://supfam.org/SUPERFAMILY/dcGO (25 October 2018, date last accessed)).

Genes located within 50 kb upstream or downstream of *BEL* or *KNOX* were used to trace the duplication history of the homeobox genes. Synteny among *BEL* and *KNOX* paralogous loci was estimated based on conserved OGs and/or protein domains. Paralogous conserved OGs were given by analyses using OrthoFinder as described above. Conserved protein domains in gene models were identified from the result of HMMER searches using default settings.

## 3. Results

### 3.1. Genome sequencing and annotation

We performed whole-genome shotgun sequencing of genomic DNA using Illumina short-read and PacBio long-read platforms (Supplementary Table S2) and assembled the sequences using a Platanus^21^-based hybrid pipeline (Supplementary Fig. S1). The *Caulerpa lentillifera* genome was estimated to be ∼26 Mb (Supplementary Fig. S2) and the resulting assembly comprised ∼29 Mb (scaffold and contig N50 lengths, 948 kb and 324 kb, respectively) (Table 1 and Supplementary Tables S3 and S4). Statistics regarding the assembly are comparable to those of the *Chlorella variabilis* and *Ostreococcus tauri* genomes (Table 1 and Supplementary Table S4). The heterozygosity rate estimated from the frequency of variant sites was ∼0.4%. The GC content of the genome was 40%, lower than in other green algae, most of which have GC contents close to 60% (Table 1 and Supplementary Table S4). Repetitive sequences constituted 6.7% of the genome, which is slightly higher than those in the genomes of *Coccomyxa subellipsoidea* (5.4%) and *Ostreococcus tauri* (5.3%) (Supplementary Table S4). Comparison of the substitution level of TEs among the four algal genomes showed a low frequency of low-substitution-rate TEs in *C*. *lentillifera* (Supplementary Fig. S3). The substitution level in the *C*. *lentillifera* genome is more similar to that of *Ostreococcus* than *Chlamydomonas* or *Chlorella*. The *C*. *lentillifera* genome contains 4.6% unknown repeats (Supplementary Table S5), which need to be further characterized in comparison with sequences of other ulvophytes.

**Table 1:** Assembly and annotation features of the *Caulerpa lentillifera* and other algal genomes

Genome features	*Caulerpa lentillifera*	*Chlamydomonas reinhardtii* ^58^	*Chlorella variabilis* ^60^	*Ostreococcus tauri* ^62^
Assembled genome size (Mb)	28.7	111.1	46.2	12.6
Number of scaffolds (≥500 bp)	185	54	414	103
Number of N50 scaffolds	14	7	12	7
N50 scaffold length (kb)	948	7,783	1,470	739
Longest scaffold (Mb)	1.29	9.73	3.12	1.08
Number of N50 contigs	28	141	438	243
N50 contig length (kb)	324	215	28	15
GC content (%)	40.4	64.1	67.1	59.2
Predicted protein-coding genes	9,311	17,741	9,791	7,664
Gene density (kb/gene)	3.2	6.2	4.7	1.6

RNA-seq reads (29.2 Gb) assembled with the PASA pipeline^36^ (Supplementary Fig. S1 and Table S2) were used to produce gene models with AUGUSTUS.^41^ A final set of *C. lentillifera* protein-coding gene models numbered 9,311 (Table 1 and Supplementary Table S4). Mapping of RNA-seq reads to scaffolds confirmed that at least 6,231 of 9,311 genes (∼67%) were expressed at the macroscopic stage of *C. lentillifera*.

BUSCO^29^ analysis, which was used to evaluate the genome assembly, showed that 86.4% of BUSCO single-copy orthologous queries were completely aligned with the assembled genome and 81.8% of the input queries were present in a single copy (Supplementary Table S4). These proportions of the *C*. *lentillifera* genome are comparable to those of other green algal genomes so far decoded, indicating the accuracy of the *C*. *lentillifera* genome assembly. The genome size and gene density of *C*. *lentillifera* (one gene/3.2 kb) appeared intermediate among the green algae (Table 1).

### 3.2. Phylogenetic analyses

**Figure 2:**
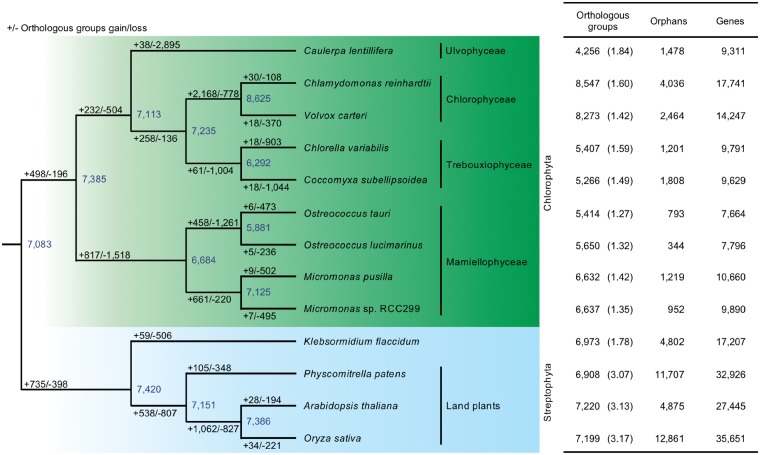
Evolution of gene families in green algae showing predicted gains and losses of orthologous gene clusters in each branch. This phylogenetic tree was constructed with the optimal maximum-likelihood method using a concatenation of 107 nuclear-gene-encoded protein alignments. Detailed information regarding the tree is shown in Supplementary Fig. S4. Numbers of assigned OGs, orphans, and genes are shown next to species names. Numbers in parentheses are average gene numbers in OGs.

To examine the phylogenetic position of the ulvophycean *Caulerpa lentillifera* among green algae, we constructed a molecular phylogenetic tree of algae and land plants, based on a comparison of 107 nuclear proteins from *C*. *lentillifera* and 15 published genomes (Fig. 2, Supplementary Fig. S4 and Table S1). Our analysis showed that the diversification of *C*. *lentillifera* occurred earlier than the split of the Chlorophyceae and Trebouxiophyceae with topological consistency among calculation methods. Some of the concordance factors from BUCKy were not high among the UTC clade due to limited taxonomic sampling. High-quality chlorophyte transcriptomic data were found in public databases (Supplementary Table S1). By including an additional five species in the potential UTC clade, we confirmed that the topology of the early split of the *Caulerpa* lineage is maintained in the clade (Supplementary Fig. S5). Therefore, an early branching of *Caulerpa* was evident among members of the UTC clade (Fig. 2, Supplementary Figs S4 and S5). Our present result (Supplementary Fig. S5), based on nuclear genes, failed to support the Ulvophyceae (*Caulerpa* and *Ulva*) as a monophyletic group.^55^^,^^56^

Based on a comparison of chloroplast genes, Fučíková et al.^11^ reported that siphonous ulvophytes, including *Caulerpa*, are sister to a part of the class Trebouxiophyceae. A mitochondrial multigene analysis supported a sister relationship between the Ulvophyceae and Chlorophyceae.^57^ However, our analysis demonstrated the earlier diversification of Ulvophyceae (Supplementary Figs S4 and S5) suggesting the paraphyly of Ulvophyceae to the other two clades. Specifically, our phylogenetic analysis was inconsistent with previous reports that used organelle-encoded genes.^11^^,^^57^ Genomic information from *C*. *lentillifera* will facilitate further exploration of the phylogeny of green plants. In addition, genomic data from lineages that have ancestral cytomorphological characters, such as Chlorocystidales, Oltmannsiellopsidales, Scotinosphaerales or Ignatiales,^10^^,^^12^ are needed to provide a more detailed evolutionary history of ulvophytes, because a recent analysis of the *Ulva mutabilis* genome indicated a sister relationship between *Ulva* and the Chlorophyceae.^14^

### 3.3. Comparative analysis of gene families and lineage-specific gene expansions

To understand the gene content of siphonous macroalga and to elucidate plant gene family evolution, we classified all *Caulerpa lentillifera* proteins and compared them using OrthoFinder,^45^ with those of five other green algae (*Chlamydomonas reinhardtii*,^58^*Volvox carteri*,^59^*Chlorella variabilis*,^60^*Coccomyxa subellipsoidea*^61^ and *Ostreococcus tauri*^62^) (Fig. 2, Supplementary Fig. S6 and Table S1). *C. lentillifera* had 4,256 of the classified OGs. In this comparative analysis, we used the term ‘OG’ to define a gene family. The classified OGs are likely sets of highly similar sequences that were derived from a single gene in the last common ancestor.^45^*C*. *lentillifera* OGs shared 3,846 of 8,547 *Chlamydomonas* OGs (45%), 3,779 of 8,273 *Volvox* OGs (46%), 3,470 of 5,407 *Chlorella* OGs (64%), 3,437 of 5,266 *Coccomyxa* OGs (65%) and 2,875 of 5,414 *Ostreococcus tauri* OGs (53%), respectively (Fig. 2 and Supplementary Fig. S6). On the other hand, 131 of 4,256 *C*. *lentillifera* OGs were not found in the five other green algal OGs (Supplementary Fig. S6).

Further analysis of gene acquisitions and losses in *C*. *lentillifera* was performed, based on phylogenetic relationships mentioned in Section 3.2 (Supplementary Fig. S4). This analysis showed that OG losses have occurred >76 times more frequently than OG acquisitions (Fig. 2). We annotated 2, 895 of putative OGs lost in *C*. *lentillifera*. Any green plant genes that were classified into 2,105 of the 2,895 OGs were not aligned on the *C*. *lentillifera* genome. These green plant genes did not show any similarity with the *C*. *lentillifera* transcriptome. Protein domains encoded in these putative lost genes in *C*. *lentillifera* were not uniformly conserved among other green plants (Supplementary Fig. S7a). On the other hand, 17 of 1,223 *C*. *lentillifera* lost protein-domains were conserved among the twelve green plants. Functions of lost protein domains are likely associated with intracellular locality (Supplementary Fig. S7b).

The average number of genes per OG in *C*. *lentillifera* was 1.84, which is higher than in other green algae. This suggests more lineage-specific gene expansions in *C*. *lentillifera* (Fig. 2). Heat map analyses of 14,946 OGs among 13 green plant genomes showed that the pattern of gene expansions in *C*. *lentillifera* did not overlap with those of other UTC algal genomes (Supplementary Fig. S8 and Dataset S1). This suggests that these gene expansions are specific to the *Caulerpa* lineage.

To further explore gene duplication in the *Caulerpa* lineage, we surveyed expanded OGs and performed GO analysis.^63^ We compared *C*. *lentillifera* with the five aforementioned algal taxa. In both categories of biological process and molecular function, ubiquitination-related GOs (GO: 0016567, GO: 0004842) were expanded in *C*. *lentillifera* (Supplementary Datasets S2 and S3). *C*. *lentillifera* genes for peptidase (GO: 0008233) and peroxidase activity (GO: 0004601) were more abundant than those of other algae (Supplementary Dataset S3). Analyses in cellular component categories also showed expansions of nuclear pore and COPI (coat protein complex I) vesicle coat (Supplementary Dataset S4). Interestingly, some nuclear pore-associated genes were expressed preferentially in fronds or stolons (Supplementary Fig. S9). For example, the *GLE1-like* RNA-export mediator was expressed preferentially in fronds.

### 3.4. Evolution of *Caulerpa* genes for core regulatory networks

**Figure 3:**
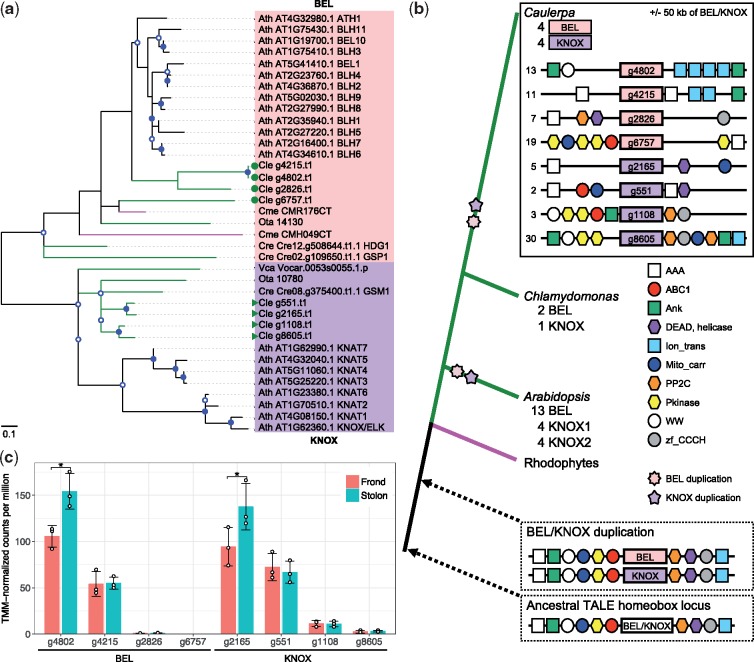
Molecular phylogenetic analysis showing lineage-specific evolution of TALE class homeobox proteins in green plants and the ancient plant genomic structure. (a) The maximum-likelihood tree is constructed using multiple alignments of homeodomains. Green symbols show four members of the KNOX class and four of the BEL class in the *Caulerpa* genome. Nodes with more than 50 and 70% bootstrap support are marked with open and closed circles, respectively. Green, magenta and black lines correspond to chlorophyte, rhodophyte and *Arabidopsis* sequences, respectively. Ath, *Arabidopsis thaliana*. Cle, *Caulerpa lentillifera*. Cme, *Cyanidioschyzon merolae*. Cre, *Chlamydomonas reinhardtii*. Ota, *Ostreococcus tauri*. Vca, *Volvox carteri*. (b) Origin and evolution of the plant BEL/KNOX family and candidate genes for exploring the duplication mechanism of the ancestral gene. Simplified gene order in the *Caulerpa* genome is shown above. Scaffold numbers are indicated at the left side. Potential ancestral genomic structures are surrounded by dotted lines. Circles, rectangles and hexagonal boxes in diagrams of genomic sequences indicate protein domains contained in each locus. The synteny diagrams are not drawn to scale. (c) Expression levels of TALE homeobox genes. * indicates *P*-value <0.05 and false discovery rate <0.05. Two genes, g4802 of *BEL*, and g2165 of *KNOX*, show the difference of gene expression level between frond and stolon. Error bars and white circles show standard deviation and TMM-normalized CPM observed in each replicate, respectively.

One of the most prominent characteristics of *Caulerpa* is its differentiated physical structure as a single cell. However, molecular mechanisms involved in development of the plant architecture without multicellularity remain unknown.^7^ To examine conservation and diversification of genes associated with core regulatory networks for morphology, we surveyed gene families for TF and TR. Comparisons with the plant database^42^ showed that TF gene families were comparatively conserved between them (Supplementary Dataset S5). One of the expanded gene families was the C3H class, the expansion of which has also been reported in the land plant lineage.^42^ In addition, homeobox genes of the HB-other class were abundant in the *C*. *lentillifera* genome (Supplementary Dataset S5). Molecular phylogeny showed that BEL and KNOX subclasses, which form heterodimeric TF complexes through gamete fusion,^64^ were expanded to eight genes in *C*. *lentillifera* (Fig. 3 and Supplementary Fig. S10). Such gene duplications have not been reported in other chlorophyte genomes (Fig. 3 and Supplementary Fig. S10), although the land plant lineage shows a greater number of duplications in this subclass.^42^ DNA-recognition sites of some expanded BEL proteins deviated from conserved motifs (Supplementary Fig. S11). Unexpectedly, comparative analysis of eight loci paralogous to *BEL* and *KNOX* showed syntenic relationships with other genes within each of the BEL and KNOX families (Fig. 3b). The syntenic genes included 10 OGs based on conserved domains (Fig. 3b and Supplementary Table S6). In addition, syntenic property appeared between *BEL* and *KNOX* (Fig. 3b). Because BEL and KNOX families belong to the TALE homeobox class, this suggests that the ancestral TALE TF gene has been expanded by segmental duplication (Fig. 3b).

To examine expression differences among eight genes of the BEL/KNOX family, we performed RNA-seq analysis in fronds and stolons of a macroscopic cell. g4802 of *BEL* and g2165 of *KNOX* showed distinct and different expression levels between frond and stolon (Fig. 3c). In addition, both had higher expression in stolons than the other six *BEL/KNOX* genes in the macroscopic stage (Fig. 3c). Only *BEL* genes, abundantly expressed in the macroscopic stage, have diversified DNA-recognition sites (Fig. 3c and Supplementary Fig. S11). These results suggest that the possible BEL/KNOX heterodimers may be core regulators involved in morphological differentiation of a macroscopic cell.

### 3.5. Evolution of *Caulerpa* genes for plant hormone signalling

In land plants, hormone signalling regulates growth, development and responses to environmental stress, although the evolutionary origins of the signalling machinery remain unknown.^65^ It is tempting to ask whether homologues of *Caulerpa lentillifera* proteins are involved in formation and growth of multinucleated, giant cells, and/or adaptive responses. Although the majority of gene families for biosynthesis and hormone signalling in land plants^65^ are not found in the *C*. *lentillifera* genome, most genes involved in abscisic acid (ABA) signalling in land plants were conserved (Supplementary Datasets S6 and S7). In addition, some components of ABA signalling are expanded in *C*. *lentillifera* (Supplementary Dataset S8). Phylogenetic analysis showed that genes for protein kinase have also been expanded in a lineage-specific manner, although the Chlorophyta has only one orthologue for *SnRK group 2 (SnRK2)*, an ABA signalling component (Supplementary Fig. S12a).

Interestingly, *Caulerpa*-specific genes similar to land plant *SnRK2* have been duplicated extensively, although the orthologues were likely lost in the land plant lineage. Additionally, 17 paralogs of *SnRKs* were tandemly clustered on scaffold 8 (Supplementary Fig. S12b). Extensive analyses of protein kinases showed that, in addition to protein kinases (class: CAMK_OST1L) including SnRKs, only the tyrosine kinase family (class: TKL_CTR1-DRK-1) has likely been expanded in the *C*. *lentillifera* genome (Supplementary Dataset S9). Another comprehensive survey of gene homologues for plant hormone biosynthesis clarified multiple tandem-duplicated genes of P450, which include a component of strigolactone (SL) biosynthesis (Supplementary Fig. S13). It is likely that a pathway involved in SL biosynthesis is also diversified in the *Caulerpa* lineage.

## 4. Discussion

**Figure 4:**
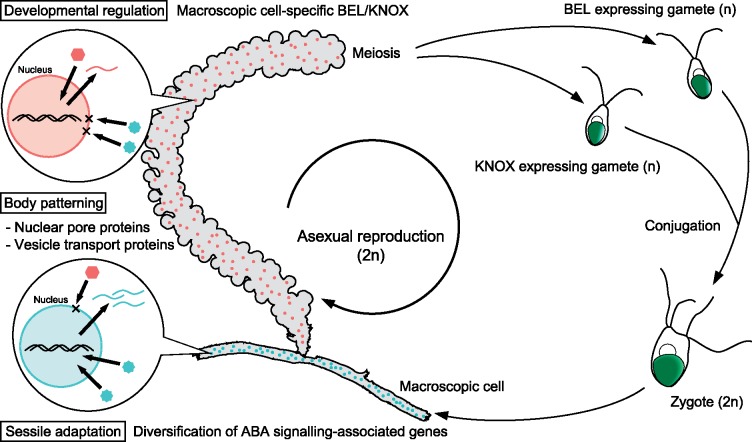
Hypothesized function of diversified gene families in siphonous macroalgae. In *Caulerpa*, divergent *BEL* and *KNOX* genes were formed by lineage-specific duplications, and they are differentially expressed in the macroscopic cell. By our prediction, *KNOX/BEL* genes of *Caulerpa* that have similar functions to those of *Chlamydomonas* are expressed specifically in gametes. In addition, nuclear pore-associated genes regulate RNA and/or protein transport in development of complex siphonous algae, as shown in balloons. Molecular localization is also controlled by vesicle transport proteins. These transporters generate molecular signatures in structure patterning without multicellularity. Expanded ABA signalling-associated genes are employed to adapt to environmental stress associated with a sessile lifestyle.

We decoded the genome of a siphonous ulvophyte, *Caulerpa lentillifera*. Based on results of present and previous studies, we hypothesize the following cellular and molecular mechanisms involved in the structural differentiation in the single cell (Fig. 4). First, we found expansion of some OGs in the *C*. *lentillifera* genome that may be involved in the siphonous body plan. Differential expression patterns of nuclear pore-associated genes, such as a *GLE1-like* RNA-export mediator, suggest that RNA localization is regulated not only by transportation of RNA in the cytoplasm,^66^ but also by nuclear position in the cell. The variations in expression profiles of transport proteins (Supplementary Fig. S9) imply that nuclei detect their own positions by unknown signals and express position-specific nuclear pore-associated genes (Fig. 4, left). Regulation of RNA transportation from nuclei by such genes function could play a role in different transcriptomic profiles in the structure, and structure-specific transcriptions may be regulated by nuclear import of TF using similar mechanisms (Fig. 4, left). It is likely that these phenomena enable structural patterning in siphonous algae without cell membrane boundaries, which are employed by multicellular organisms.

Interestingly, expanded OGs in the *C*. *lentillifera* genome included the TALE class of homeobox proteins, including BEL and KNOX. These TALE homeobox genes are located at distant loci not only in *C*. *lentillifera* but also in *Chlamydomonas* and *Arabidopsis*. Homeobox genes in land plant genomes have been expanded by segmental duplications.^67^ Although the evolutionary mechanism of such homeobox genes has been extensively studied in land plants, the mechanism of the first duplication has not been proposed. Our comparative analysis demonstrated the ancient homeobox locus and adjacent gene sets evolved by segmental duplication (Fig. 3b). The *C*. *lentillifera* genome thus provides the first suggestion of how the ancestral TALE TF gene was duplicated during eukaryote evolution (Fig. 3b). Ancestral BEL and KNOX may function in normal meiosis, mating and zygote development, as in *Chlamydomonas*.^68^ We hypothesize that less expressed *BEL/KNOX* genes in *C*. *lentillifera* macroscopic cells preserve ancestral functions in gametes (Fig. 4). On the other hand, some of duplicated homeobox genes in *C*. *lentillifera* may have promoted neofunctionalization and those genes may not regulate alternation of life cycles but morphogenesis as in the land plant lineage^64^^,^^69^ (Fig. 4). Differential expression of the homeobox genes in *C*. *lentillifera* can be interpreted as analogous to structural patterning through homeobox-protein antagonisms reported in land plants.^69^

In summary, our analyses illustrate the utility of the *C*. *lentillifera* genome for exploring possible evolutionary processes in green plants. Therefore, this siphonous algal genome will help to integrate knowledge from various model systems through comparisons with derived developmental traits and will accelerate understanding of apparent convergent evolution in plants.

## Data availability

All sequence data obtained in this study are accessible in the DDBJ/EMBL/NCBI database at the BioProject ID, PRJDB5734. A genome browser for assembled sequences, including annotation, is available at http://marinegenomics.oist.jp/gallery/.

## Supplementary Material

Supplementary Data
