# Supplementary material for: A siphonous macroalgal genome suggests convergent functions of homeobox genes in algae and land plants
Source: DNA Res. 2019 Mar 28;26(2):183–92. doi: 10.1093/dnares/dsz002 (PMC6476727; doi:10.1093/dnares/dsz002)
Supplement: Supplementary Data [file dsz002_supp.zip › dsz002-Suppl_data/dsz002_Suppl_Info.pdf]

Supplementary Figures

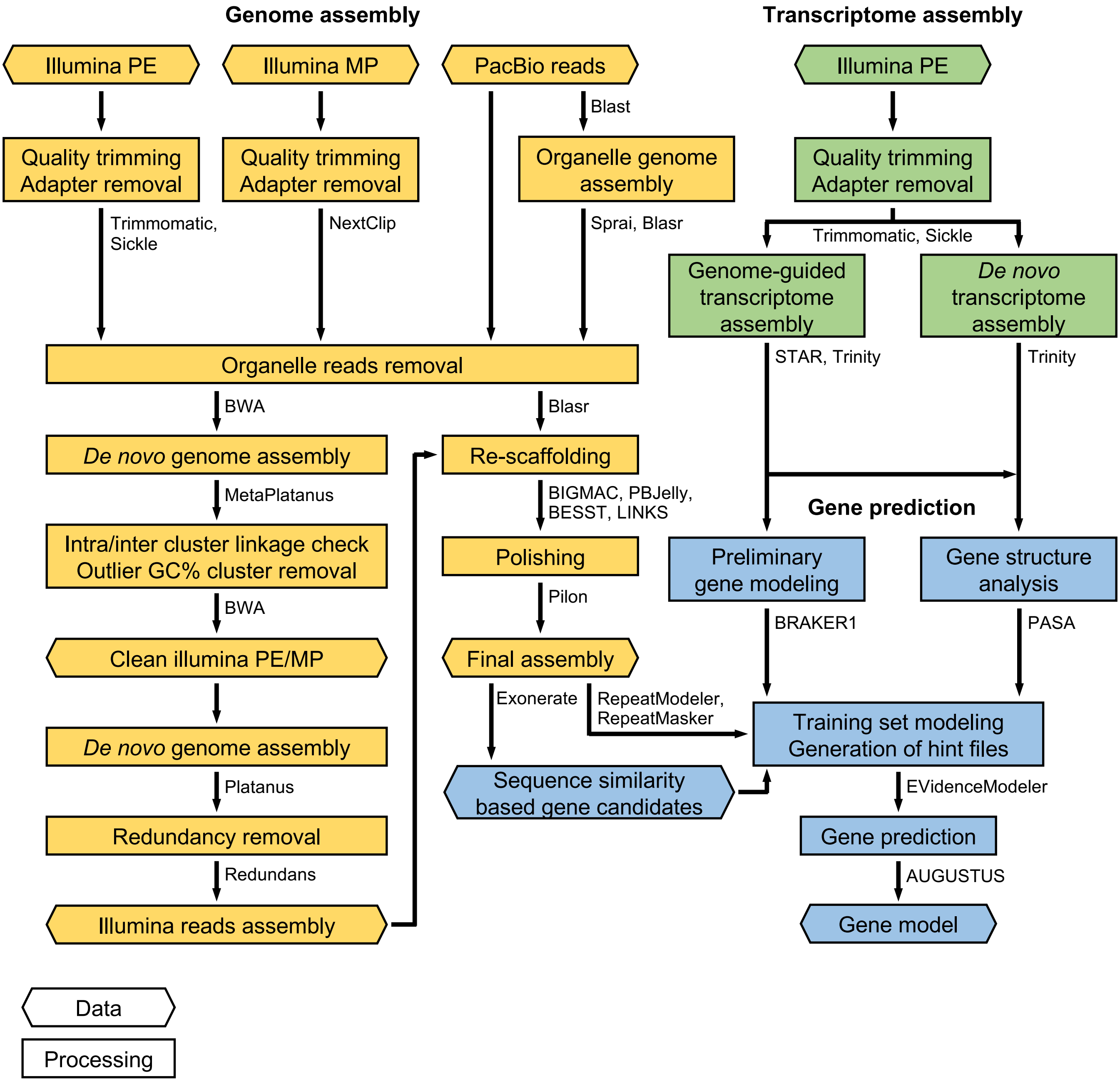

Supplementary Figure S1. The genome assembly and annotation pipeline in *Caulerpa lentillifera*. Hexagonal boxes represent input/output data, and square boxes indicate data processing steps. Yellow, green, and blue boxes correspond to genome assembly, transcriptome assembly, and gene prediction steps, respectively. Applications employed are indicated with arrows.

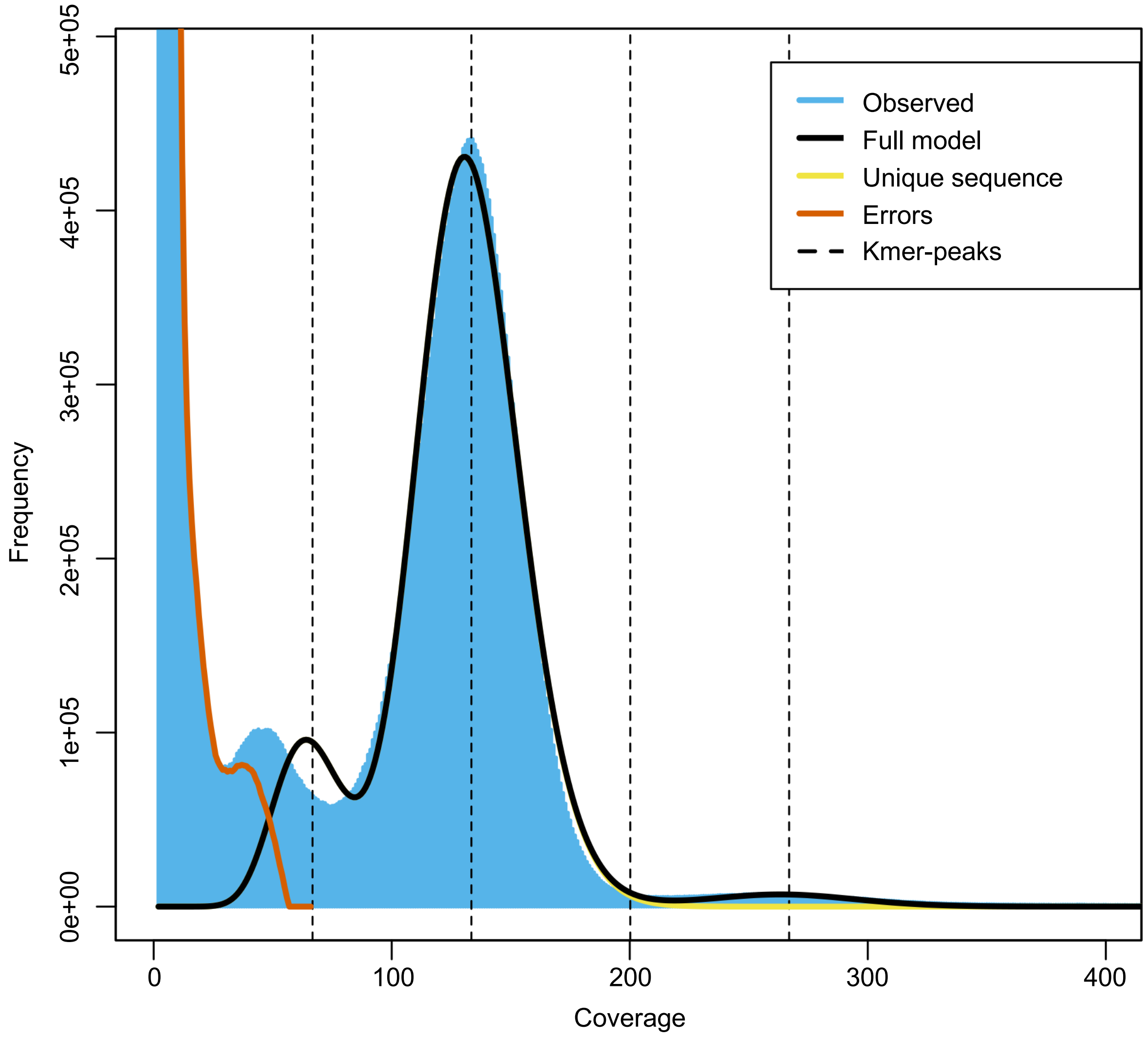

Supplementary Figure S2. K-mer analysis and genome size estimation of *Caulerpa lentillifera*. Paired-end sequences in *C. lentillifera* are analyzed using the GenomeScope web tool[22]. The major K-mer frequency peak of 21-mer occurs at 130x coverage, and the estimated genome size is 26.8 Mb, corresponding to the total length of the assembled scaffolds (28.7 Mb).

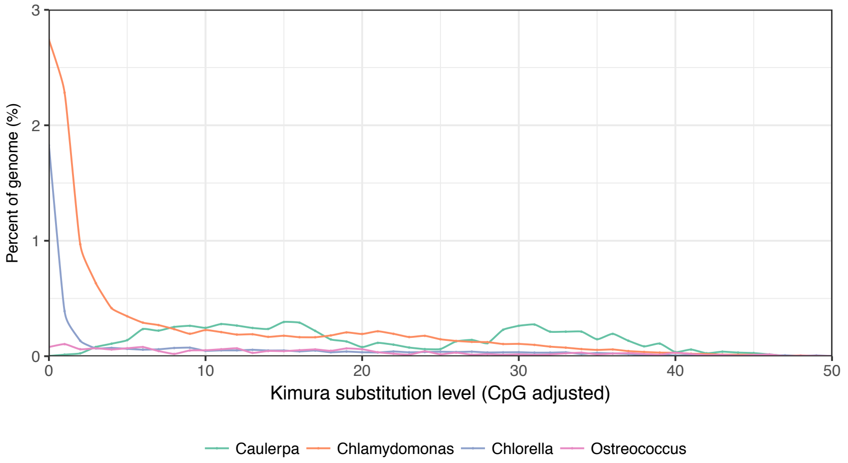

Supplementary Figure S3. Landscape of Kimura substitution level of four green algal genomes. The total percentages of transposable elements (TEs) for each of four green algae at a particular substitution level, analogous to the age of TEs, are represented (see Supplementary Methods). The *Caulerpa* genome shows a lower frequency of low-substitution-rate TEs than the *Chlamydomonas* or *Chlorella* genomes.

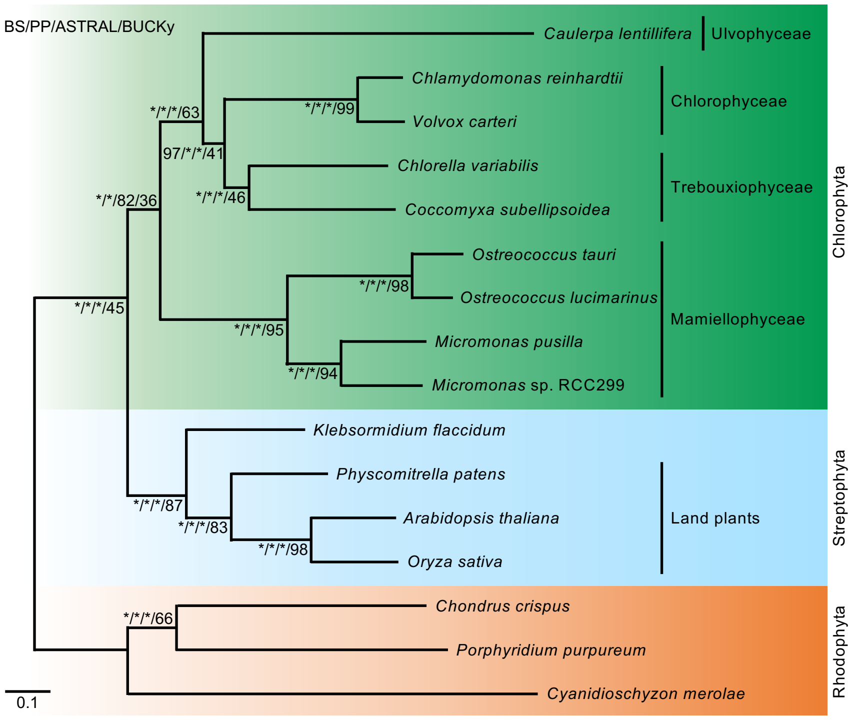

Supplementary Figure S4. Phylogenetic analysis of green plants. The phylogenetic position of *C. lentillifera* among algae and land plants. The phylogenetic tree was constructed with the optimal maximum-likelihood method using a concatenation of 107 nuclear-encoded protein alignments. Numbers at nodes represent bootstrap values, Bayesian posterior probabilities, ASTRAL score, and concordance factors, and each maximal value is indicated with an asterisk. The scale bar denotes the number of substitutions per site. The tree shows that the Ulvophyceae is the earliest branching group in the UTC clade.

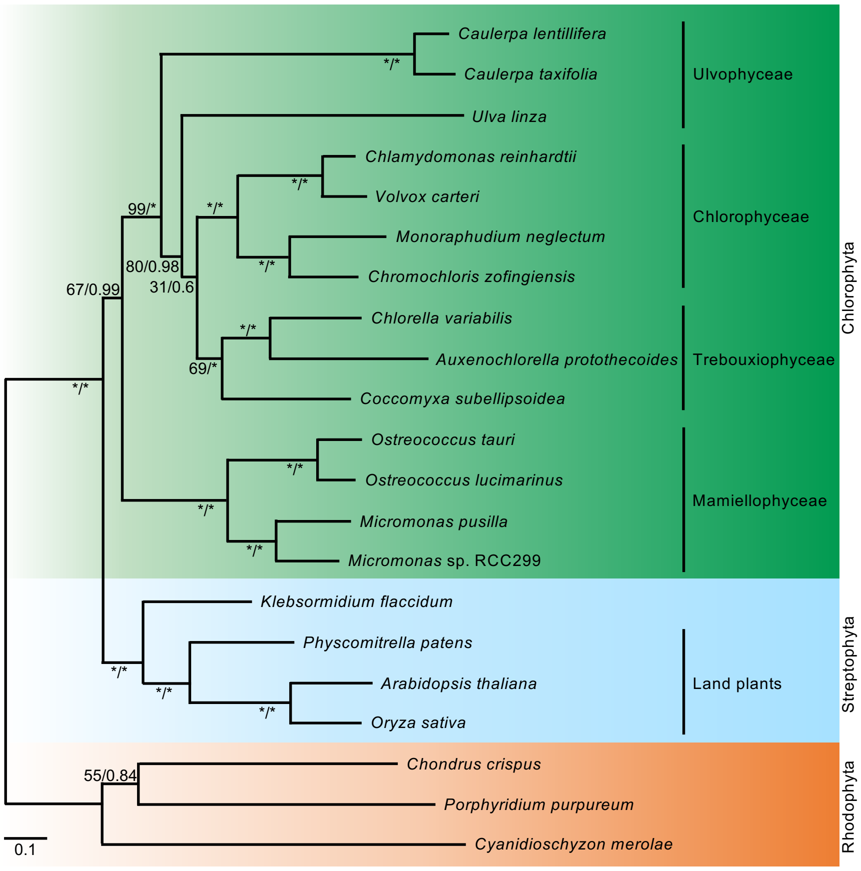

Supplementary Figure S5. Species tree inferred from an alternative dataset incorporating additional transcriptomes. The phylogenetic tree was constructed with the optimal maximum-likelihood method using a concatenation of 15 nuclear-encoded protein alignments. Numbers at nodes represent bootstrap values, Bayesian posterior probabilities. * indicates 100% bootstrap support or a posterior probability equal to 1. The scale bar denotes the number of substitutions per site. The topology of this alternative tree supports the species tree shown in Supplementary Fig. S4.

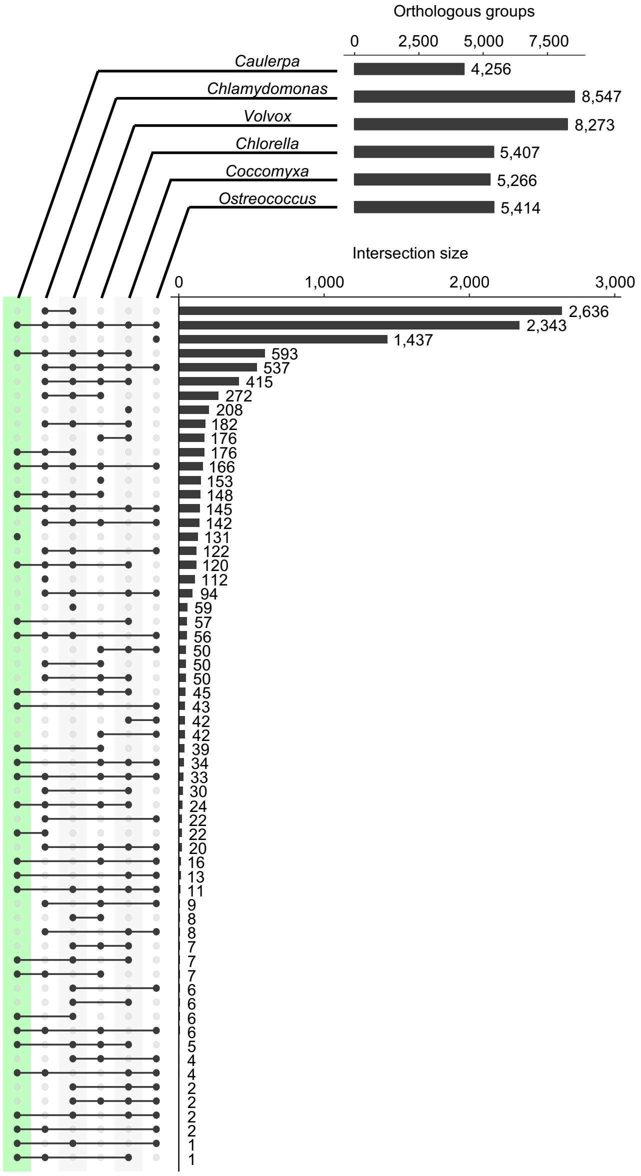

Supplementary Figure S6. Comparative analyses of genes and evolution of six green algal genomes. UpSet plot showing unique and shared orthologous groups among six chlorophyte genomes. The intersection shows the number of assigned orthologous groups in each genome.

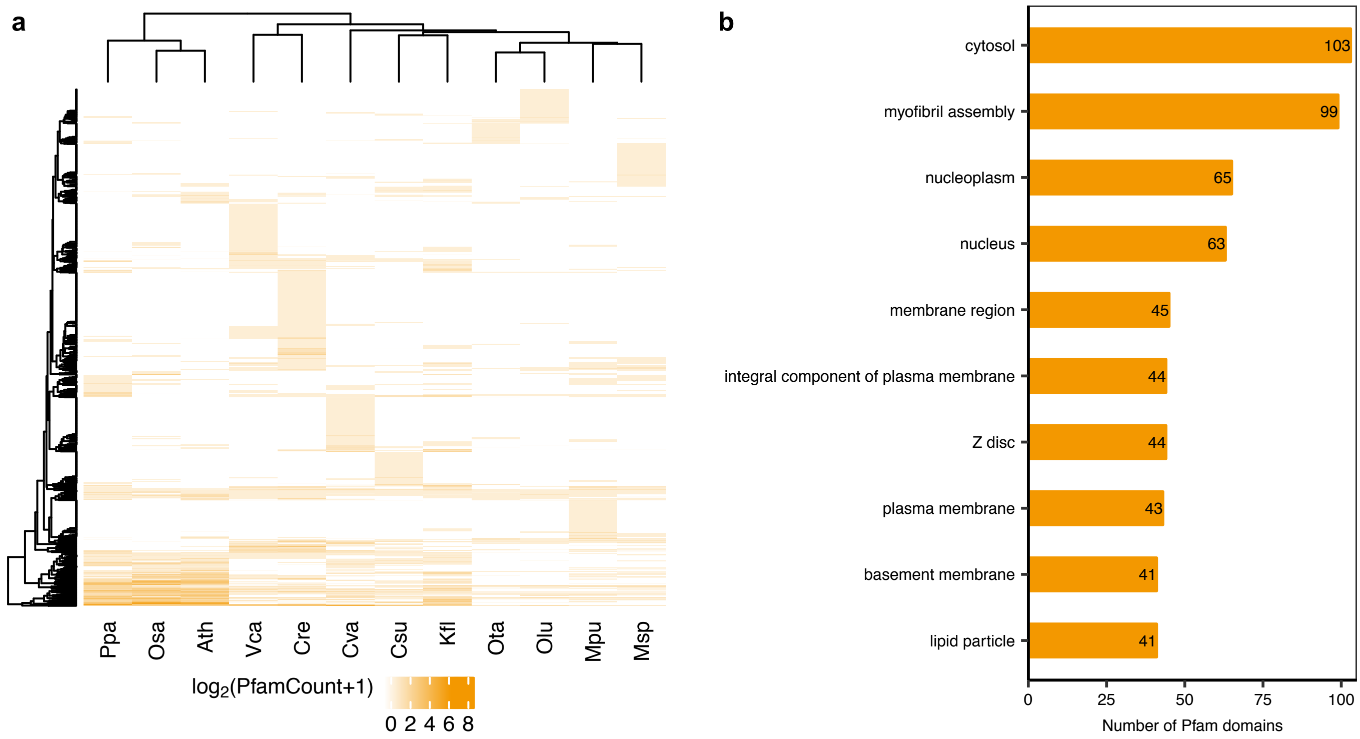

Supplementary Figure S7. Gene-loss analysis and functional annotation of lost genes in *Caulerpa*. (a) Retention pattern among twelve green plants of Pfam protein domains which are present in homologs thought to have been lost in *Caulerpa*. Ath, *Arabidopsis thaliana*. Cre, *Chlamydomonas reinhardtii*. Csu, *Coccomyxa subellipsoidea*. Cva, *Chlorella variabilis*. Kfl, *Klebsormidium flaccidum*. Msp, *Micromonas* sp. RCC299. Mpu, *Micromonas pusilla*. Olu, *Ostreococcus lucimarinus*. Ota, *Ostreococcus tauri*. Osa, *Oryza sativa*. Ppa, *Physcomitrella patens*. Vca, *Volvox carteri*. **(b)** Gene ontology terms assigned to putative lost homologs in *Caulerpa*. Gene ontology annotation is based on Pfam protein domains encoded in the genomes of twelve green plants.

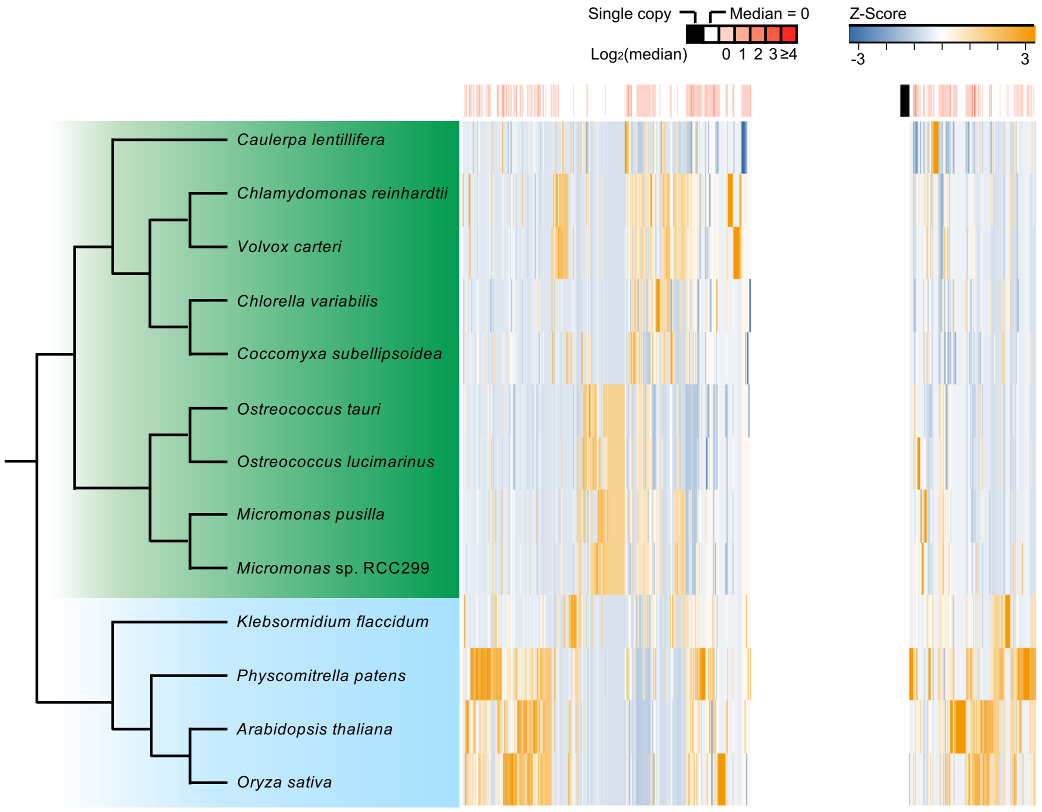

Supplementary Figure S8. Gene-family expansion profiles among green plants. A heat map of 14,946 orthologous groups (OGs) indicates lineage-specific gene expansions. The top row shows the median size of each OG. The black part of this row corresponds to putative single-copy OGs. A white window in the middle of a heat map represents OGs retained in only one to several species. Yellow and blue boxes in each row indicate expansion and collapse of the number of genes in each OG, respectively. The dendrogram shows phylogenetic relationships among thirteen green plants.

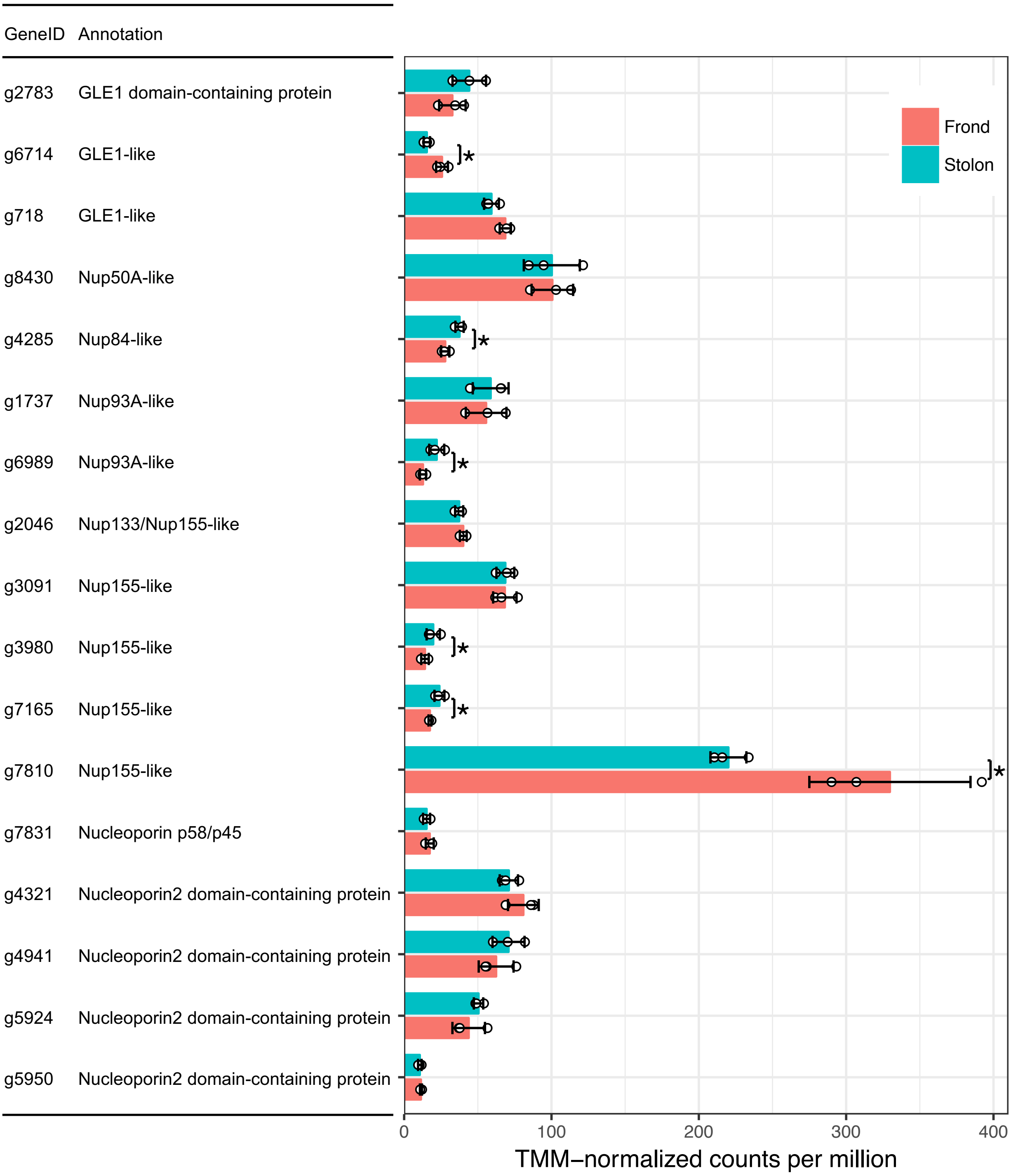

Supplementary Figure S9. Differential expression of nuclear pore-associated genes. Expression levels are shown in TMM-normalized counts per million (CPM). * indicates a p-value < 0.05 and a false discovery rate < 0.05. Six of seventeen genes were showed significant differentially expression. Error bars and white circles show standard deviation and TMM-normalized CPM observed in each replicate, respectively.

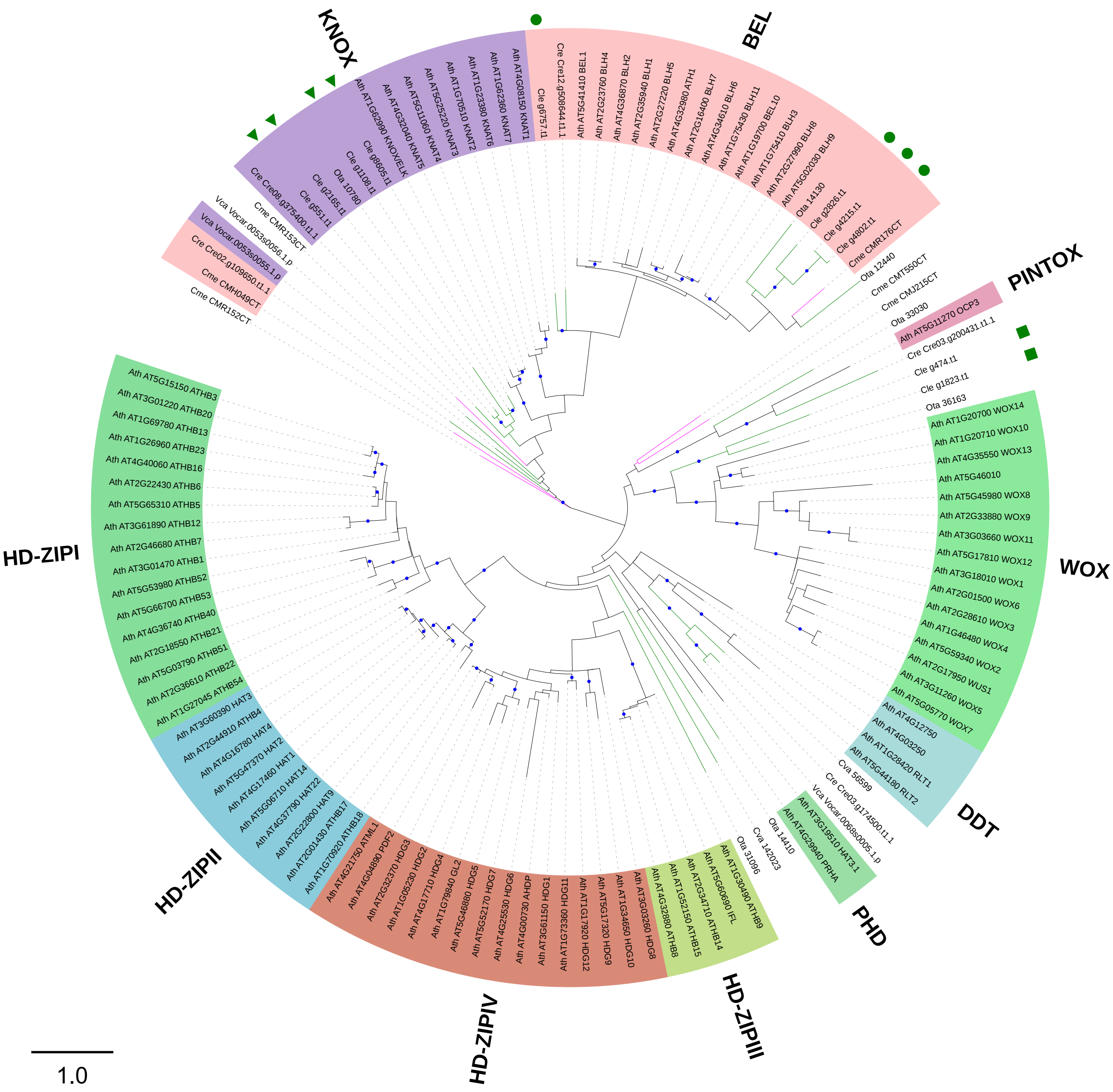

Supplementary Figure S10. Maximum-likelihood tree of homeodomain-containing proteins in the *Arabidopsis* and green algal genomes, using IQ-TREE. Green symbols show four of the KNOX class, four of the BEL class, and two of the WOX class in the *Caulerpa* genome, respectively. Analyzed proteins in Figure 3 and *Arabidopsis* proteins are highlighted in each subclass. Nodes with more than 50% bootstrap support are marked with closed circles. Green, magenta, and black lines correspond to chlorophytes, rhodophytes, and *Arabidopsis* sequences, respectively. The scale bar denotes the number of substitutions per site. Ath, *Arabidopsis thaliana*. Cle, *Caulerpa lentillifera*. Cme, *Cyanidioschyzon merolae*. Cre, *Chlamydomonas reinhardtii*. Ota, *Ostreococcus tauri*. Vca, *Volvox carteri*.

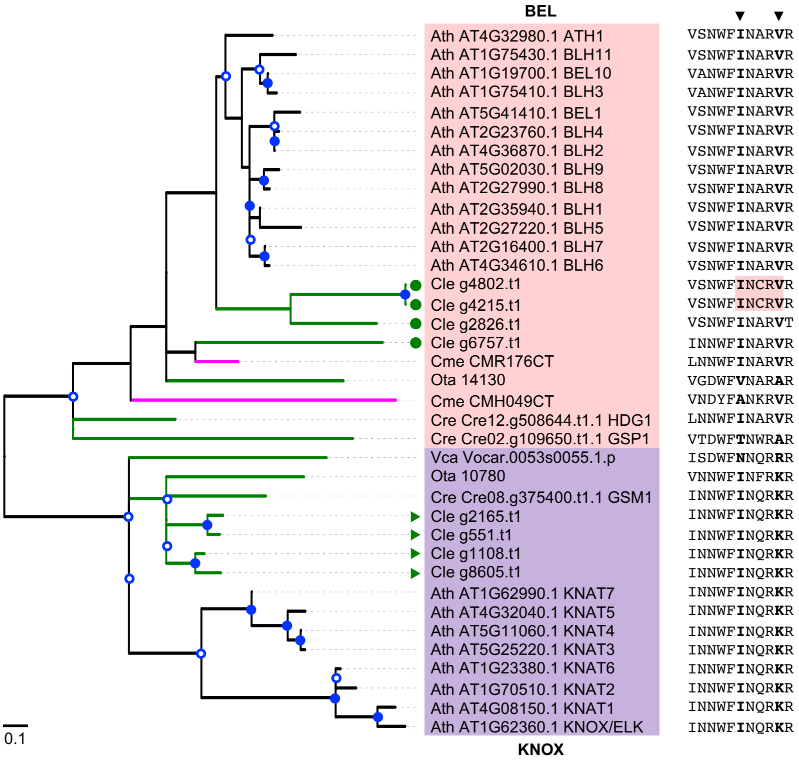

Supplementary Figure S11. Maximum-likelihood tree of TALE class homeodomain proteins in green plants and the variety of motifs in *Caulerpa*. The maximum-likelihood tree is constructed using multiple alignments of homeodomains. Green symbols show four members of the KNOX class and four of the BEL class in the *Caulerpa* genome, respectively. Nodes with more than 50% and 70% bootstrap support are marked with open and closed circles, respectively. Green, magenta, and black lines correspond to chlorophytes, rhodophytes, and *Arabidopsis* sequences, respectively. The alignment shows the DNA-recognition site of each homeodomain. Arrowheads indicate subgroup-specific motif sites. The box on alignment indicates diversified sites among *Caulerpa* proteins. The scale bar denotes the number of substitutions per site. Ath, *Arabidopsis thaliana*. Cle, *Caulerpa lentillifera*. Cme, *Cyanidioschyzon merolae*. Cre, *Chlamydomonas reinhardtii*. Ota, *Ostreococcus tauri*. Vca, *Volvox carteri*.

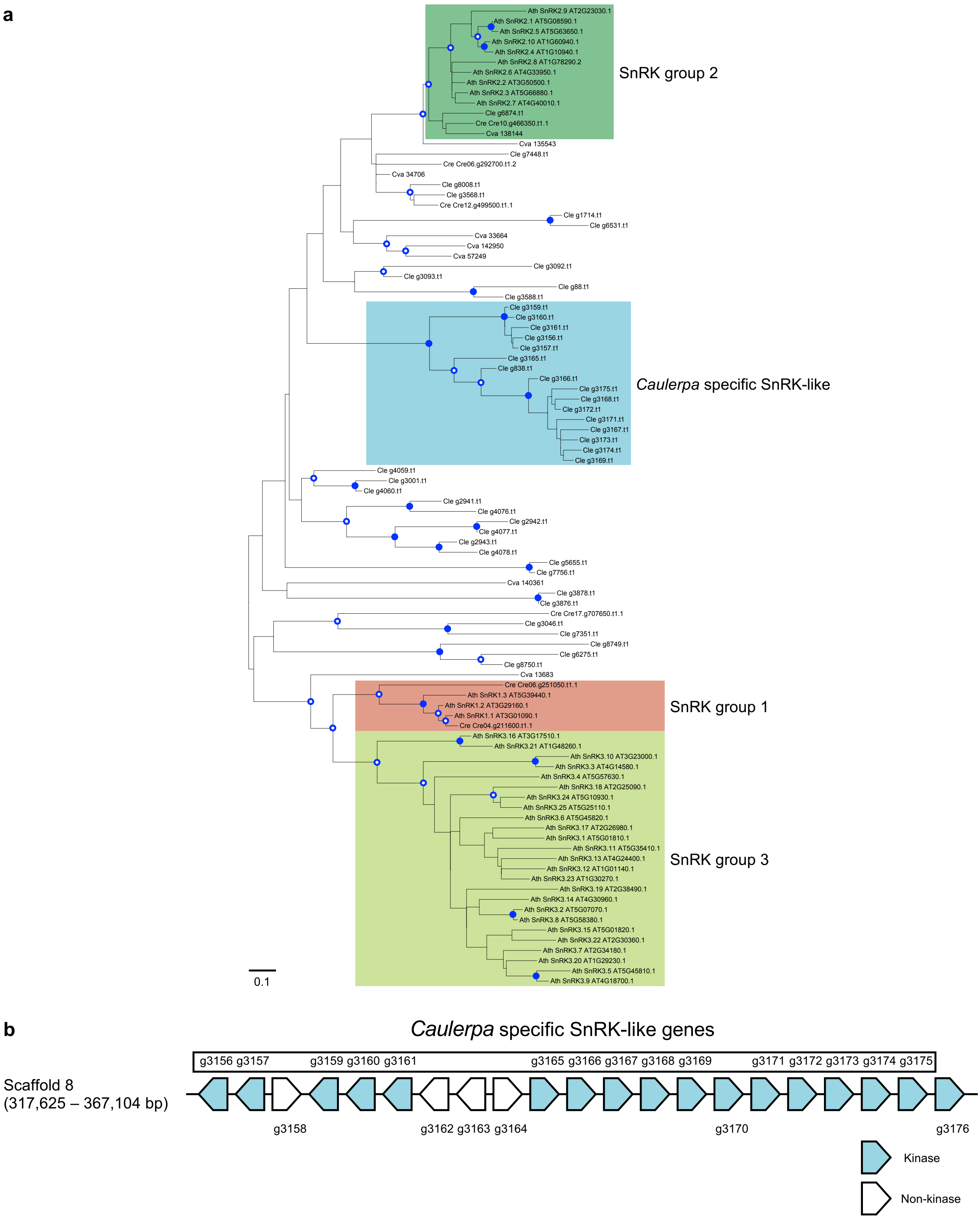

Supplementary Figure S12. Expansion of *SnRK* genes in the *Caulerpa lentillifera* genome. a, Maximum likelihood tree of the SnRK superfamily in the *Arabidopsis* and green algal genomes. Bootstrap support values ≥50% and 80% are marked with open and closed circles, respectively. The scale bar denotes the number of substitutions per site. The tree suggests that most *Caulerpa* proteins in the SnRK superfamily are derived from lineage-specific expansion. **b**, Tandem *SnRK-like* loci on Scaffold 8 of *C. lentillifera*. Seventeen *Caulerpa* specific SnRK-like genes are clustered in the assembled genome. Ath, *Arabidopsis thaliana*. Cle, *Caulerpa lentillifera*. Cre, *Chlamydomonas reinhardtii*. Cva, *Chlorella variabilis*.

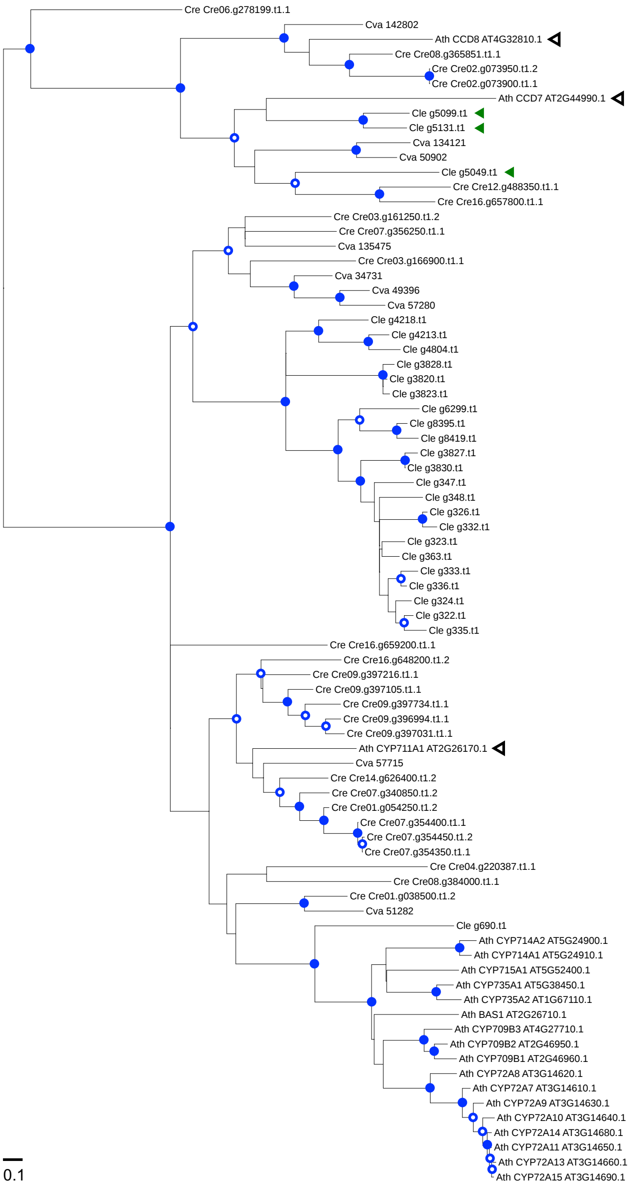

Supplementary Figure S13. Expansion of *P450* genes in the *Caulerpa lentillifera* genome. The maximum likelihood tree of the P450 strigolactone (SL) biosynthesis-associated superfamily in the *Arabidopsis* and green algal genomes. Bootstrap support values ≥50% and 80% are marked with open and closed circles, respectively. SL synthetic enzymes in *Arabidopsis* and putative orthologs in *Caulerpa* are marked with open and closed triangles, respectively. The scale bar denotes the number of substitutions per site. The tree suggests that most *Caulerpa* proteins in the P450 superfamily are derived from lineage-specific expansion. Ath, *Arabidopsis thaliana*. Cle, *Caulerpa lentillifera*. Cre, *Chlamydomonas reinhardtii*. Cva, *Chlorella variabilis*.

Supplementary Tables

Supplementary Table S1. Genomes and gene model versions used in the analysis

Species name	Abbreviations	Gene model version
Caulerpa lentillifera	Cle	This study
Chlamydomonas reinhardtii	Cre	JGI v5.5 (ref. 58)
Volvox carteri	Vca	JGI v2.1 (ref. 59)
Chlorella variabilis	Cva	JGI v1.0 (ref. 60)
Coccomyxa subellipsoidea	Csu	JGI v2.0 (ref. 61)
Ostreococcus tauri	Ota	JGI v2.0 (ref. 62)
Ostreococcus lucimarinus	Olu	JGI v2.0 (ref. 62)
Micromonas pusilla	Mpu	JGI v3.0 (ref. 70)
Micromonas sp. RCC299	Msp	JGI v3.0 (ref. 70)
Klebsormidium flaccidum	Kfl	Version 1.1 (ref. 71)
Physcomitrella patens	Ppa	JGI v3.3 (ref. 72)
Arabidopsis thaliana	Ath	Araport11 (ref. 73)
Oryza sativa	-	IRGSP-1.0 (ref. 74)
Cyanidioschyzon merolae	Cme	Ensembl Plants 34 (ref. 75)
Porphyridium purpureum	-	Updated 02/2012 (ref. 76)
Chondrus crispus	-	Ensembl Plants 34 (ref. 77)
Auxenochlorella protothecoides†	-	Version 1.0 (ref. 78)
Caulerpa taxifolia†	-	* (ref. 66)
Chromochloris zofingiensis†	-	Version 5.2.3.2 (ref. 79)
Monoraphidium neglectum†	-	Version 1.0 (ref. 80)
Ulva linza†	-	* (ref. 81)

* indicates de novo assembled transciptome.

† indicates that a dataset was only used for the analysis in Supplementary Figure S5.

Supplementary Table S2. Sequence data summary

				Raw data		Filtered data*			
		Insert size	Read length	Data amount	Coverage	Data amount	Coverage	Source	Experiment
DNA	Paired-end	1 kb	2x300 bp	19.7 Gb	735x	9.36 Gb	349x	Multi individuals	Genome assembly
	Mate-pair	3 kb	2x300 bp	9.31 Gb	347x	1.76 Gb	66x	Multi individuals	Genome assembly
	Mate-pair	6 kb	2x300 bp	1.50 Gb	56x	95.2 Mb	3.6x	Multi individuals	Genome assembly
	PacBio	-	-	2.06 Gb	77x	738 Mb	28x	Multi individuals	Genome assembly
	Paired-end	1 kb	2x300 bp	15.0 Gb	560x	3.89 Gb	145x	Single individual	Variant detection
RNA	Paired-end	200 bp	2x300 bp	29.2 Gb	-	25.9 Gb	-	Multi individuals	Gene modeling
	Paired-end	200 bp	2x150 bp	18.1 Gb	-	17.5 Gb	-	Individual A frond	Expression analysis
	Paired-end	200 bp	2x150 bp	18.1 Gb	-	17.5 Gb	-	Individual A stolon	Expression analysis
	Paired-end	200 bp	2x150 bp	19.2 Gb	-	18.6 Gb	-	Individual B frond	Expression analysis
	Paired-end	200 bp	2x150 bp	18.2 Gb	-	17.7 Gb	-	Individual B stolon	Expression analysis
	Paired-end	200 bp	2x150 bp	20.8 Gb	-	20.2 Gb	-	Individual C frond	Expression analysis
	Paired-end	200 bp	2x150 bp	17.3 Gb	-	16.9 Gb	-	Individual C stolon	Expression analysis

* Reads derived from chloroplast or prokaryotic genomes were removed. The filtering methods were described in the Methods section.

Supplementary Table S3. Summary of the *Caulerpa lentillifera* genome assembly

Minimum sequence length	Number of contigs	Number of scaffolds	Scaffold size	% Non-gap basepairs
1 Mb	2	11	12,330,917	99.66
500 kb	9	16	12,459,749	99.62
250 kb	28	4	1,440,551	99.97
100 kb	43	6	1,115,283	99.82
50 kb	22	6	412,175	98.00
25 kb	20	9	327,005	95.40
10 kb	35	12	196,762	96.99
5 kb	29	19	120,391	96.43
2.5 kb	40	33	118,240	96.18
1 kb	46	38	65,496	95.05
100 bp	303	268	67,691	99.98
Total	577	422	28,654,260	99.54

Supplementary Table S4. Genome assembly comparison of six green algae

Genome features	*Caulerpa lentillifera*	*Chlamydomonas reinhardtii*	*Volvox carteri*	*Chlorella variabilis*	*Coccomyxa subellipsoidea*	*Ostreococcus tauri*
Estimated genome size (Mb)	26.8	NA*	NA*	46.2	48.8	12.6
Estimation method	K-mer counting	NA*	NA*	PFGE	PFGE	PFGE
Sequencing technology	Illumina<br>PacBio	Sanger	Sanger	Sanger	Sanger	Sanger
Assembly version	This study	JGI v5.5 (ref. 58)	JGI v2.1 (ref. 59)	JGI v1.0 (ref. 60)	JGI v2.0 (ref. 61)	JGI v2.0 (ref. 62)
Assembled genome size (Mb)	28.7	111.1	131.2	46.2	49	12.6
Scaffolds (≥500 bp)						
Number	185	54	434	414	45	103
N50 (kb)	948	7,784	2,600	1,470	1,960	739
L50	14	7	15	12	9	7
Contigs (≥500 bp)						
Number	314	1,503	3,994	3,751	45	1,755
N50 (kb)	324	215	85	28	1,960	15
L50	28	141	410	438	9	243
% Gaps	0.5	3.6	4.4	8.5	0	0.3
% GC content	40.4	64.1	56.1	67.1	52.9	59.2
% Repeated sequences	6.7	21.2	26	10.9	5.4	5.3
BUSCO						
%Complete and single-copy	81.8	85.8	84.8	82.2	86.5	78.9
%Complete and duplicated	4.6	1.7	1.0	0.7	0.7	1.3
%Fragmented	3.3	0.7	1.7	5.0	2.6	3.6
%Missing	10.3	11.8	12.5	12.1	10.2	16.2
CEGMA						
%Complete	90.73	90.73	84.68	84.68	93.15	78.63
%Partial	93.95	94.35	93.55	89.11	95.16	82.66
Predicted protein-coding genes (loci)	9,311	17,741	14,247	9,791	9,629	7,664
% ESTs / RNA reads aligned to scaffolds	95	95	98	98	95	ND
% genes have EST / RNA-seq support	67	56	34	42	51	21
Average transcript length (bp)	2,381	3,267	3,221	1,416	3,250	1,175
Average exon frequency per gene	4.8	8.3	7.8	7.3	8.2	1.6

*Genome size estimation was not performed. Total assembled length is represented as the genome size.

NA, not applicable. ND, no data available. PFGE, pulsed-field gel electrophoresis.

Supplementary Table S5. Repeated sequences in the *Caulerpa lentillifera* genome

Class			Percentage in the assembly
DNA transposons			0.8929
		hAT	0.8123
		CMC	0.0122
		Helitron	0.0088
Retrotransposons			
	LTR		0.2487
		Gypsy	0.0686
		Copia	0.0346
		Pao	0.0188
	LINE		0.1525
		Tad1	0.1202
		L1	0.0088
		L2	0.0047
	SINE		0.0058
Others			
	Satellite		0.0096
	RNA		
		rRNA	0.0889
		tRNA	0.0166
	Simple repeat		0.5693
	Low complexity		0.0795
	Unknown		4.6115
Total			6.6770

Supplementary Table S6. Molecular signatures of segmental duplications of homeobox loci in *Caulerpa lentillifera*.

Scaffold ID	Gene ID	OG ID	Conserved protein domains	Pfam Accession
Scaffold 13	g4789	OG0000006	Ank_2,Ank_4,Ank_5	PF12796,PF13637,PF13857
Scaffold 13	g4792	OG0000922	WW	PF00397
Scaffold 13	g4802	OG0000116	Homeobox_KN	PF05920
Scaffold 13	g4810	OG0000062	Ion_trans	PF00520
Scaffold 13	g4811	OG0000062	Ion_trans	PF00520
Scaffold 13	g4812	OG0000062	Ion_trans	PF00520
Scaffold 13	g4814	OG0000062	Not_found	NA
Scaffold 13	g4815	OG0000006	Ank_2,Ank_4,Ank_5	PF12796,PF13637,PF13857
Scaffold 11	g4227	OG0000340	AAA	PF00004
Scaffold 11	g4215	OG0000116	Homeobox_KN	PF05920
Scaffold 11	g4212	OG0001292	AAA	PF00004
Scaffold 11	g4210	OG0000062	Ion_trans	PF00520
Scaffold 11	g4209	OG0000062	Ion_trans	PF00520
Scaffold 11	g4201	OG0000077	Ank_2,Ank_4,Ank_5	PF12796,PF13637,PF13857
Scaffold 7	g2808	OG0003843	AAA_33,AAA_18	PF13671,PF13238
Scaffold 7	g2814	OG0000813	PP2C	PF00481
Scaffold 7	g2817	OG0000072	DEAD,Helicase_C	PF00270,PF00271
Scaffold 7	g2826	OG0000116	Homeobox_KN	PF05920
Scaffold 7	g2843	OG0000332	zf-CCCH	PF00642
Scaffold 19	g6736	NA	Pkinase	PF00069
Scaffold 19	g6740	OG0000185	Mito_carr	PF00153
Scaffold 19	g6741	OG0000000	Pkinase	PF00069
Scaffold 19	g6745	OG0001600	Pkinase	PF00069
Scaffold 19	g6746	OG0000951	ABC1	PF03109
Scaffold 19	g6757	OG0000116	Homeobox_KN	PF05920
Scaffold 19	g6775	OG0000142	Pkinase	PF00069
Scaffold 19	g6776	OG0002024	AAA	PF00004
Scaffold 5	g2150	OG0001155	AAA_19	PF13245
Scaffold 5	g2165	OG0000116	Homeobox_KN	PF05920
Scaffold 5	g2169	OG0000363	DEAD,Helicase_C	PF00270,PF00271
Scaffold 5	g2184	OG0000075	Mito_carr	PF00153
Scaffold 2	g530	OG0000848	AAA_5	PF07728
Scaffold 2	g544	OG0000951	ABC1	PF03109
Scaffold 2	g545	OG0000634	Mito_carr	PF00153
Scaffold 2	g551	OG0000116	Homeobox_KN	PF05920
Scaffold 2	g552	OG0000850	AAA_31	PF13614
Scaffold 2	g564	OG0001741	DEAD,Helicase_C	PF00270,PF00271
Scaffold 3	g1120	OG0000244	WW	PF00397
Scaffold 3	g1119	OG0000000	Pkinase	PF00069
Scaffold 3	g1116	OG0007490	Pkinase	PF00069
Scaffold 3	g1114	OG0000132	ABC1	PF03109
Scaffold 3	g1112	OG0000006	Ank_2,Ank_4,Ank_5	PF12796,PF13637,PF13857
Scaffold 3	g1108	OG0000116	Homeobox_KN	PF05920
Scaffold 3	g1107	OG0000580	PP2C	PF00481
Scaffold 3	g1106	OG0000332	zf-CCCH	PF00642
Scaffold 30	g8588	OG0000006	Ank_2,Ank_4,Ank_5	PF12796,PF13637,PF13857
Scaffold 30	g8594	OG0000244	WW	PF00397
Scaffold 30	g8595	OG0000000	Pkinase	PF00069
Scaffold 30	g8598	OG0007490	Pkinase	PF00069
Scaffold 30	g8605	OG0000116	Homeobox_KN	PF05920
Scaffold 30	g8606	OG0000580	PP2C	PF00481
Scaffold 30	g8608	OG0000332	zf-CCCH	PF00642
Scaffold 30	g8609	OG0002561	Mito_carr	PF00153
Scaffold 30	g8620	OG0000047	PP2C	PF00481
Scaffold 30	g8621	OG0000006	Ank_2,Ank_4,Ank_5	PF12796,PF13637,PF13857
Scaffold 30	g8622	OG0000062	Ion_trans	PF00520

Supplementary References

70. Worden, A. Z., Lee, J. H., Mock, T., et al. 2009, Green evolution and dynamic adaptations revealed by genomes of the marine picoeukaryotes *Micromonas*, *Science*, **324**, 268-272.

71. Hori, K., Maruyama, F., Fujisawa, T., et al. 2014, *Klebsormidium flaccidum* genome reveals primary factors for plant terrestrial adaptation, *Nat. Commun.*, **5**, 3978.

72. Physcomitrella patens v3.3, DOE-JGI, http://phytozome.jgi.doe.gov (2015).

73. Cheng, C. Y., Krishnakumar, V., Chan, A. P., Thibaud-Nissen, F., Schobel, S. and Town, C. D. 2017, Araport11: a complete reannotation of the *Arabidopsis thaliana* reference genome, *Plant J.*, **89**, 789-804.

74. Sakai, H., Lee, S. S., Tanaka, T., et al. 2013, Rice Annotation Project Database (RAP-DB): an integrative and interactive database for rice genomics, *Plant Cell Physiol.*, **54**, e6.

75. Matsuzaki, M., Misumi, O., Shin-I, T., et al. 2004, Genome sequence of the ultrasmall unicellular red alga *Cyanidioschyzon merolae* 10D, *Nature*, **428**, 653-657.

76. Bhattacharya, D., Price, D. C., Chan, C. X., et al. 2013, Genome of the red alga *Porphyridium purpureum*, *Nat. Commun.*, **4**, 1941.

77. Collén, J., Porcel, B., Carré, W., et al. 2013, Genome structure and metabolic features in the red seaweed *Chondrus crispus* shed light on evolution of the Archaeplastida, *Proc. Natl. Acad. Sci. USA*, **110**, 5247-5252.

78. Gao, C., Wang, Y., Shen, Y., et al. 2014, Oil accumulation mechanisms of the oleaginous microalga *Chlorella protothecoides* revealed through its genome, transcriptomes, and proteomes, *BMC Genomics*, **15**, 582.

79. Roth, M. S., Cokus, S. J., Gallaher, S. D., et al. 2017, Chromosome-level genome assembly and transcriptome of the green alga *Chromochloris zofingiensis* illuminates astaxanthin production, *Proc. Natl. Acad. Sci. USA*, **114**, E4296-E4305.

80. Bogen, C., Al-Dilaimi, A., Albersmeier, A., et al. 2013, Reconstruction of the lipid metabolism for the microalga *Monoraphidium neglectum* from its genome sequence reveals characteristics suitable for biofuel production, *BMC Genomics*, **14**, 926.

81. Zhang, X., Ye, N., Liang, C., et al. 2012, De novo sequencing and analysis of the *Ulva linza* transcriptome to discover putative mechanisms associated with its successful colonization of coastal ecosystems, *BMC Genomics*, **13**, 565.
