# Supplementary material for: A siphonous macroalgal genome suggests convergent functions of homeobox genes in algae and land plants
Source: DNA Res. 2019 Mar 28;26(2):183–92. doi: 10.1093/dnares/dsz002 (PMC6476727; doi:10.1093/dnares/dsz002)
Supplement: Supplementary Data [file dsz002_Dataset_S1.pdf › dsz002-Suppl_data/dsz002_Dataset_S1.pdf]

g1119.t1 Caulerpa_lentillifera OG0000000
g1337.t1 Caulerpa_lentillifera OG0000000
g1383.t1 Caulerpa_lentillifera OG0000000
g1539.t1 Caulerpa_lentillifera OG0000000
g1888.t1 Caulerpa_lentillifera OG0000000
g1960.t1 Caulerpa_lentillifera OG0000000
g215.t1 Caulerpa_lentillifera OG0000000
g2284.t1 Caulerpa_lentillifera OG0000000
g2603.t1 Caulerpa_lentillifera OG0000000
g3013.t1 Caulerpa_lentillifera OG0000000
g3328.t1 Caulerpa_lentillifera OG0000000
g3634.t1 Caulerpa_lentillifera OG0000000
g391.t1 Caulerpa_lentillifera OG0000000
g4044.t1 Caulerpa_lentillifera OG0000000
g4161.t1 Caulerpa_lentillifera OG0000000
g4162.t1 Caulerpa_lentillifera OG0000000
g4640.t1 Caulerpa_lentillifera OG0000000
g4968.t1 Caulerpa_lentillifera OG0000000
g5271.t1 Caulerpa_lentillifera OG0000000
g5412.t1 Caulerpa_lentillifera OG0000000
g5487.t1 Caulerpa_lentillifera OG0000000
g5631.t1 Caulerpa_lentillifera OG0000000
g5632.t1 Caulerpa_lentillifera OG0000000
g5651.t1 Caulerpa_lentillifera OG0000000
g5719.t1 Caulerpa_lentillifera OG0000000
g5791.t1 Caulerpa_lentillifera OG0000000
g5816.t1 Caulerpa_lentillifera OG0000000
g5822.t1 Caulerpa_lentillifera OG0000000
g5861.t1 Caulerpa_lentillifera OG0000000
g6029.t1 Caulerpa_lentillifera OG0000000
g6622.t1 Caulerpa_lentillifera OG0000000
g6637.t1 Caulerpa_lentillifera OG0000000
g6673.t1 Caulerpa_lentillifera OG0000000
g6741.t1 Caulerpa_lentillifera OG0000000
g6781.t1 Caulerpa_lentillifera OG0000000
g6877.t1 Caulerpa_lentillifera OG0000000
g6966.t1 Caulerpa_lentillifera OG0000000
g6978.t1 Caulerpa_lentillifera OG0000000
g7757.t1 Caulerpa_lentillifera OG0000000
g7785.t1 Caulerpa_lentillifera OG0000000
g7786.t1 Caulerpa_lentillifera OG0000000
g7939.t1 Caulerpa_lentillifera OG0000000
g807.t1 Caulerpa_lentillifera OG0000000
g8153.t1 Caulerpa_lentillifera OG0000000
g8595.t1 Caulerpa_lentillifera OG0000000
g8856.t1 Caulerpa_lentillifera OG0000000
g923.t1 Caulerpa_lentillifera OG0000000
g963.t1 Caulerpa_lentillifera OG0000000
g993.t1 Caulerpa_lentillifera OG0000000
Cre02.g141926.t1.1 pacid=30784972 transcript=Cre02.g141926.t1.1 locus=Cre02.g141926 ID=Cre02.g141926.t1.1.v5.5 annot-version=v5.5 Chlamydomonas_reinhardtii OG0000000
Cre02.g116700.t1.2 pacid=30784996 transcript=Cre02.g116700.t1.2 locus=Cre02.g116700 ID=Cre02.g116700.t1.2.v5.5 annot-version=v5.5

Chlamydomonas_reinhardtii OG0000000
Cre02.g087850.t1.1 pacid=30784997 transcript=Cre02.g087850.t1.1
locus=Cre02.g087850 ID=Cre02.g087850.t1.1.v5.5 annot-version=v5.5
Chlamydomonas_reinhardtii OG0000000
Cre02.g141806.t1.1 pacid=30785206 transcript=Cre02.g141806.t1.1
locus=Cre02.g141806 ID=Cre02.g141806.t1.1.v5.5 annot-version=v5.5
Chlamydomonas_reinhardtii OG0000000
Cre02.g099750.t1.1 pacid=30785258 transcript=Cre02.g099750.t1.1
locus=Cre02.g099750 ID=Cre02.g099750.t1.1.v5.5 annot-version=v5.5
Chlamydomonas_reinhardtii OG0000000
Cre07.g351900.t1.1 pacid=30774544 transcript=Cre07.g351900.t1.1
locus=Cre07.g351900 ID=Cre07.g351900.t1.1.v5.5 annot-version=v5.5
Chlamydomonas_reinhardtii OG0000000
Cre02.g087800.t1.1 pacid=30785308 transcript=Cre02.g087800.t1.1
locus=Cre02.g087800 ID=Cre02.g087800.t1.1.v5.5 annot-version=v5.5
Chlamydomonas_reinhardtii OG0000000
Cre07.g316650.t1.1 pacid=30774550 transcript=Cre07.g316650.t1.1
locus=Cre07.g316650 ID=Cre07.g316650.t1.1.v5.5 annot-version=v5.5
Chlamydomonas_reinhardtii OG0000000
Cre02.g098550.t1.1 pacid=30785441 transcript=Cre02.g098550.t1.1
locus=Cre02.g098550 ID=Cre02.g098550.t1.1.v5.5 annot-version=v5.5
Chlamydomonas_reinhardtii OG0000000
Cre02.g102650.t1.1 pacid=30785461 transcript=Cre02.g102650.t1.1
locus=Cre02.g102650 ID=Cre02.g102650.t1.1.v5.5 annot-version=v5.5
Chlamydomonas_reinhardtii OG0000000
Cre02.g102900.t1.1 pacid=30785529 transcript=Cre02.g102900.t1.1
locus=Cre02.g102900 ID=Cre02.g102900.t1.1.v5.5 annot-version=v5.5
Chlamydomonas_reinhardtii OG0000000
Cre02.g145500.t1.2 pacid=30785547 transcript=Cre02.g145500.t1.2
locus=Cre02.g145500 ID=Cre02.g145500.t1.2.v5.5 annot-version=v5.5
Chlamydomonas_reinhardtii OG0000000
Cre02.g087900.t1.1 pacid=30785603 transcript=Cre02.g087900.t1.1
locus=Cre02.g087900 ID=Cre02.g087900.t1.1.v5.5 annot-version=v5.5
Chlamydomonas_reinhardtii OG0000000
Cre02.g095129.t1.1 pacid=30785662 transcript=Cre02.g095129.t1.1
locus=Cre02.g095129 ID=Cre02.g095129.t1.1.v5.5 annot-version=v5.5
Chlamydomonas_reinhardtii OG0000000
Cre02.g104201.t1.1 pacid=30785778 transcript=Cre02.g104201.t1.1
locus=Cre02.g104201 ID=Cre02.g104201.t1.1.v5.5 annot-version=v5.5
Chlamydomonas_reinhardtii OG0000000
Cre02.g087750.t1.2 pacid=30785802 transcript=Cre02.g087750.t1.2
locus=Cre02.g087750 ID=Cre02.g087750.t1.2.v5.5 annot-version=v5.5
Chlamydomonas_reinhardtii OG0000000
Cre02.g090700.t1.1 pacid=30785892 transcript=Cre02.g090700.t1.1
locus=Cre02.g090700 ID=Cre02.g090700.t1.1.v5.5 annot-version=v5.5
Chlamydomonas_reinhardtii OG0000000
Cre02.g097500.t1.1 pacid=30786040 transcript=Cre02.g097500.t1.1
locus=Cre02.g097500 ID=Cre02.g097500.t1.1.v5.5 annot-version=v5.5
Chlamydomonas_reinhardtii OG0000000
Cre02.g098600.t1.1 pacid=30786156 transcript=Cre02.g098600.t1.1
locus=Cre02.g098600 ID=Cre02.g098600.t1.1.v5.5 annot-version=v5.5
Chlamydomonas_reinhardtii OG0000000
Cre07.g346550.t1.1 pacid=30774640 transcript=Cre07.g346550.t1.1
locus=Cre07.g346550 ID=Cre07.g346550.t1.1.v5.5 annot-version=v5.5

Chlamydomonas_reinhardtii OG0000000
Cre02.g141966.t1.1 pacid=30786244 transcript=Cre02.g141966.t1.1
locus=Cre02.g141966 ID=Cre02.g141966.t1.1.v5.5 annot-version=v5.5
Chlamydomonas_reinhardtii OG0000000
Cre02.g110850.t1.2 pacid=30786285 transcript=Cre02.g110850.t1.2
locus=Cre02.g110850 ID=Cre02.g110850.t1.2.v5.5 annot-version=v5.5
Chlamydomonas_reinhardtii OG0000000
Cre02.g095099.t1.1 pacid=30786346 transcript=Cre02.g095099.t1.1
locus=Cre02.g095099 ID=Cre02.g095099.t1.1.v5.5 annot-version=v5.5
Chlamydomonas_reinhardtii OG0000000
Cre02.g104450.t1.1 pacid=30786402 transcript=Cre02.g104450.t1.1
locus=Cre02.g104450 ID=Cre02.g104450.t1.1.v5.5 annot-version=v5.5
Chlamydomonas_reinhardtii OG0000000
Cre02.g103900.t1.1 pacid=30786464 transcript=Cre02.g103900.t1.1
locus=Cre02.g103900 ID=Cre02.g103900.t1.1.v5.5 annot-version=v5.5
Chlamydomonas_reinhardtii OG0000000
Cre03.g144464.t1.1 pacid=30786554 transcript=Cre03.g144464.t1.1
locus=Cre03.g144464 ID=Cre03.g144464.t1.1.v5.5 annot-version=v5.5
Chlamydomonas_reinhardtii OG0000000
Cre03.g176400.t1.1 pacid=30787136 transcript=Cre03.g176400.t1.1
locus=Cre03.g176400 ID=Cre03.g176400.t1.1.v5.5 annot-version=v5.5
Chlamydomonas_reinhardtii OG0000000
Cre03.g144011.t1.1 pacid=30787156 transcript=Cre03.g144011.t1.1
locus=Cre03.g144011 ID=Cre03.g144011.t1.1.v5.5 annot-version=v5.5
Chlamydomonas_reinhardtii OG0000000
Cre03.g159016.t1.1 pacid=30787171 transcript=Cre03.g159016.t1.1
locus=Cre03.g159016 ID=Cre03.g159016.t1.1.v5.5 annot-version=v5.5
Chlamydomonas_reinhardtii OG0000000
Cre03.g194100.t1.2 pacid=30787204 transcript=Cre03.g194100.t1.2
locus=Cre03.g194100 ID=Cre03.g194100.t1.2.v5.5 annot-version=v5.5
Chlamydomonas_reinhardtii OG0000000
Cre03.g151300.t1.1 pacid=30787234 transcript=Cre03.g151300.t1.1
locus=Cre03.g151300 ID=Cre03.g151300.t1.1.v5.5 annot-version=v5.5
Chlamydomonas_reinhardtii OG0000000
Cre07.g325759.t1.1 pacid=30774745 transcript=Cre07.g325759.t1.1
locus=Cre07.g325759 ID=Cre07.g325759.t1.1.v5.5 annot-version=v5.5
Chlamydomonas_reinhardtii OG0000000
Cre03.g160900.t1.2 pacid=30787359 transcript=Cre03.g160900.t1.2
locus=Cre03.g160900 ID=Cre03.g160900.t1.2.v5.5 annot-version=v5.5
Chlamydomonas_reinhardtii OG0000000
Cre03.g159150.t1.1 pacid=30787438 transcript=Cre03.g159150.t1.1
locus=Cre03.g159150 ID=Cre03.g159150.t1.1.v5.5 annot-version=v5.5
Chlamydomonas_reinhardtii OG0000000
Cre07.g319850.t1.2 pacid=30774783 transcript=Cre07.g319850.t1.2
locus=Cre07.g319850 ID=Cre07.g319850.t1.2.v5.5 annot-version=v5.5
Chlamydomonas_reinhardtii OG0000000
Cre03.g144264.t1.1 pacid=30787682 transcript=Cre03.g144264.t1.1
locus=Cre03.g144264 ID=Cre03.g144264.t1.1.v5.5 annot-version=v5.5
Chlamydomonas_reinhardtii OG0000000
Cre03.g157250.t1.1 pacid=30787700 transcript=Cre03.g157250.t1.1
locus=Cre03.g157250 ID=Cre03.g157250.t1.1.v5.5 annot-version=v5.5
Chlamydomonas_reinhardtii OG0000000
Cre03.g175600.t1.1 pacid=30787823 transcript=Cre03.g175600.t1.1
locus=Cre03.g175600 ID=Cre03.g175600.t1.1.v5.5 annot-version=v5.5

Chlamydomonas_reinhardtii OG0000000
Cre03.g166750.t1.2 pacid=30787911 transcript=Cre03.g166750.t1.2
locus=Cre03.g166750 ID=Cre03.g166750.t1.2.v5.5 annot-version=v5.5
Chlamydomonas_reinhardtii OG0000000
Cre01.g064727.t1.1 pacid=30788461 transcript=Cre01.g064727.t1.1
locus=Cre01.g064727 ID=Cre01.g064727.t1.1.v5.5 annot-version=v5.5
Chlamydomonas_reinhardtii OG0000000
Cre01.g043850.t1.1 pacid=30788569 transcript=Cre01.g043850.t1.1
locus=Cre01.g043850 ID=Cre01.g043850.t1.1.v5.5 annot-version=v5.5
Chlamydomonas_reinhardtii OG0000000
Cre01.g011800.t1.1 pacid=30788849 transcript=Cre01.g011800.t1.1
locus=Cre01.g011800 ID=Cre01.g011800.t1.1.v5.5 annot-version=v5.5
Chlamydomonas_reinhardtii OG0000000
Cre01.g024650.t1.1 pacid=30788855 transcript=Cre01.g024650.t1.1
locus=Cre01.g024650 ID=Cre01.g024650.t1.1.v5.5 annot-version=v5.5
Chlamydomonas_reinhardtii OG0000000
Cre01.g024550.t1.1 pacid=30788929 transcript=Cre01.g024550.t1.1
locus=Cre01.g024550 ID=Cre01.g024550.t1.1.v5.5 annot-version=v5.5
Chlamydomonas_reinhardtii OG0000000
Cre07.g349000.t1.1 pacid=30774915 transcript=Cre07.g349000.t1.1
locus=Cre07.g349000 ID=Cre07.g349000.t1.1.v5.5 annot-version=v5.5
Chlamydomonas_reinhardtii OG0000000
Cre01.g064362.t1.1 pacid=30789078 transcript=Cre01.g064362.t1.1
locus=Cre01.g064362 ID=Cre01.g064362.t1.1.v5.5 annot-version=v5.5
Chlamydomonas_reinhardtii OG0000000
Cre01.g030100.t1.1 pacid=30789084 transcript=Cre01.g030100.t1.1
locus=Cre01.g030100 ID=Cre01.g030100.t1.1.v5.5 annot-version=v5.5
Chlamydomonas_reinhardtii OG0000000
Cre01.g036900.t1.1 pacid=30789136 transcript=Cre01.g036900.t1.1
locus=Cre01.g036900 ID=Cre01.g036900.t1.1.v5.5 annot-version=v5.5
Chlamydomonas_reinhardtii OG0000000
Cre01.g001200.t1.1 pacid=30789333 transcript=Cre01.g001200.t1.1
locus=Cre01.g001200 ID=Cre01.g001200.t1.1.v5.5 annot-version=v5.5
Chlamydomonas_reinhardtii OG0000000
Cre01.g024800.t1.1 pacid=30789335 transcript=Cre01.g024800.t1.1
locus=Cre01.g024800 ID=Cre01.g024800.t1.1.v5.5 annot-version=v5.5
Chlamydomonas_reinhardtii OG0000000
Cre01.g001700.t1.1 pacid=30789537 transcript=Cre01.g001700.t1.1
locus=Cre01.g001700 ID=Cre01.g001700.t1.1.v5.5 annot-version=v5.5
Chlamydomonas_reinhardtii OG0000000
Cre07.g320050.t1.1 pacid=30774974 transcript=Cre07.g320050.t1.1
locus=Cre07.g320050 ID=Cre07.g320050.t1.1.v5.5 annot-version=v5.5
Chlamydomonas_reinhardtii OG0000000
Cre01.g011850.t1.1 pacid=30789652 transcript=Cre01.g011850.t1.1
locus=Cre01.g011850 ID=Cre01.g011850.t1.1.v5.5 annot-version=v5.5
Chlamydomonas_reinhardtii OG0000000
Cre01.g032850.t1.1 pacid=30789653 transcript=Cre01.g032850.t1.1
locus=Cre01.g032850 ID=Cre01.g032850.t1.1.v5.5 annot-version=v5.5
Chlamydomonas_reinhardtii OG0000000
Cre01.g024750.t1.1 pacid=30789702 transcript=Cre01.g024750.t1.1
locus=Cre01.g024750 ID=Cre01.g024750.t1.1.v5.5 annot-version=v5.5
Chlamydomonas_reinhardtii OG0000000
Cre10.g462350.t1.1 pacid=30789743 transcript=Cre10.g462350.t1.1
locus=Cre10.g462350 ID=Cre10.g462350.t1.1.v5.5 annot-version=v5.5

Chlamydomonas_reinhardtii OG0000000
Cre07.g351825.t1.1 pacid=30775000 transcript=Cre07.g351825.t1.1
locus=Cre07.g351825 ID=Cre07.g351825.t1.1.v5.5 annot-version=v5.5
Chlamydomonas_reinhardtii OG0000000
Cre10.g424700.t1.1 pacid=30790099 transcript=Cre10.g424700.t1.1
locus=Cre10.g424700 ID=Cre10.g424700.t1.1.v5.5 annot-version=v5.5
Chlamydomonas_reinhardtii OG0000000
Cre10.g419450.t1.1 pacid=30790684 transcript=Cre10.g419450.t1.1
locus=Cre10.g419450 ID=Cre10.g419450.t1.1.v5.5 annot-version=v5.5
Chlamydomonas_reinhardtii OG0000000
Cre10.g423200.t1.1 pacid=30790707 transcript=Cre10.g423200.t1.1
locus=Cre10.g423200 ID=Cre10.g423200.t1.1.v5.5 annot-version=v5.5
Chlamydomonas_reinhardtii OG0000000
Cre10.g435750.t1.1 pacid=30790797 transcript=Cre10.g435750.t1.1
locus=Cre10.g435750 ID=Cre10.g435750.t1.1.v5.5 annot-version=v5.5
Chlamydomonas_reinhardtii OG0000000
Cre10.g454300.t1.1 pacid=30790816 transcript=Cre10.g454300.t1.1
locus=Cre10.g454300 ID=Cre10.g454300.t1.1.v5.5 annot-version=v5.5
Chlamydomonas_reinhardtii OG0000000
Cre07.g324866.t1.1 pacid=30775154 transcript=Cre07.g324866.t1.1
locus=Cre07.g324866 ID=Cre07.g324866.t1.1.v5.5 annot-version=v5.5
Chlamydomonas_reinhardtii OG0000000
Cre04.g223200.t1.1 pacid=30791312 transcript=Cre04.g223200.t1.1
locus=Cre04.g223200 ID=Cre04.g223200.t1.1.v5.5 annot-version=v5.5
Chlamydomonas_reinhardtii OG0000000
Cre12.g516750.t1.1 pacid=30791695 transcript=Cre12.g516750.t1.1
locus=Cre12.g516750 ID=Cre12.g516750.t1.1.v5.5 annot-version=v5.5
Chlamydomonas_reinhardtii OG0000000
Cre12.g525400.t1.1 pacid=30791697 transcript=Cre12.g525400.t1.1
locus=Cre12.g525400 ID=Cre12.g525400.t1.1.v5.5 annot-version=v5.5
Chlamydomonas_reinhardtii OG0000000
Cre12.g517600.t1.2 pacid=30791704 transcript=Cre12.g517600.t1.2
locus=Cre12.g517600 ID=Cre12.g517600.t1.2.v5.5 annot-version=v5.5
Chlamydomonas_reinhardtii OG0000000
Cre12.g552150.t1.1 pacid=30791763 transcript=Cre12.g552150.t1.1
locus=Cre12.g552150 ID=Cre12.g552150.t1.1.v5.5 annot-version=v5.5
Chlamydomonas_reinhardtii OG0000000
Cre12.g507200.t1.1 pacid=30791912 transcript=Cre12.g507200.t1.1
locus=Cre12.g507200 ID=Cre12.g507200.t1.1.v5.5 annot-version=v5.5
Chlamydomonas_reinhardtii OG0000000
Cre12.g534650.t1.1 pacid=30791998 transcript=Cre12.g534650.t1.1
locus=Cre12.g534650 ID=Cre12.g534650.t1.1.v5.5 annot-version=v5.5
Chlamydomonas_reinhardtii OG0000000
Cre12.g532050.t1.2 pacid=30792136 transcript=Cre12.g532050.t1.2
locus=Cre12.g532050 ID=Cre12.g532050.t1.2.v5.5 annot-version=v5.5
Chlamydomonas_reinhardtii OG0000000
Cre07.g314500.t1.1 pacid=30775259 transcript=Cre07.g314500.t1.1
locus=Cre07.g314500 ID=Cre07.g314500.t1.1.v5.5 annot-version=v5.5
Chlamydomonas_reinhardtii OG0000000
Cre07.g351800.t1.1 pacid=30775266 transcript=Cre07.g351800.t1.1
locus=Cre07.g351800 ID=Cre07.g351800.t1.1.v5.5 annot-version=v5.5
Chlamydomonas_reinhardtii OG0000000
Cre12.g516650.t1.1 pacid=30792461 transcript=Cre12.g516650.t1.1
locus=Cre12.g516650 ID=Cre12.g516650.t1.1.v5.5 annot-version=v5.5

Chlamydomonas_reinhardtii OG0000000
Cre12.g484800.t1.1 pacid=30792647 transcript=Cre12.g484800.t1.1
locus=Cre12.g484800 ID=Cre12.g484800.t1.1.v5.5 annot-version=v5.5
Chlamydomonas_reinhardtii OG0000000
Cre12.g545950.t1.1 pacid=30793000 transcript=Cre12.g545950.t1.1
locus=Cre12.g545950 ID=Cre12.g545950.t1.1.v5.5 annot-version=v5.5
Chlamydomonas_reinhardtii OG0000000
Cre12.g547200.t1.1 pacid=30793526 transcript=Cre12.g547200.t1.1
locus=Cre12.g547200 ID=Cre12.g547200.t1.1.v5.5 annot-version=v5.5
Chlamydomonas_reinhardtii OG0000000
Cre12.g516600.t1.1 pacid=30793603 transcript=Cre12.g516600.t1.1
locus=Cre12.g516600 ID=Cre12.g516600.t1.1.v5.5 annot-version=v5.5
Chlamydomonas_reinhardtii OG0000000
Cre11.g483100.t1.1 pacid=30775744 transcript=Cre11.g483100.t1.1
locus=Cre11.g483100 ID=Cre11.g483100.t1.1.v5.5 annot-version=v5.5
Chlamydomonas_reinhardtii OG0000000
Cre11.g482800.t1.1 pacid=30775892 transcript=Cre11.g482800.t1.1
locus=Cre11.g482800 ID=Cre11.g482800.t1.1.v5.5 annot-version=v5.5
Chlamydomonas_reinhardtii OG0000000
Cre11.g483000.t1.1 pacid=30775922 transcript=Cre11.g483000.t1.1
locus=Cre11.g483000 ID=Cre11.g483000.t1.1.v5.5 annot-version=v5.5
Chlamydomonas_reinhardtii OG0000000
Cre14.g628200.t1.1 pacid=30776190 transcript=Cre14.g628200.t1.1
locus=Cre14.g628200 ID=Cre14.g628200.t1.1.v5.5 annot-version=v5.5
Chlamydomonas_reinhardtii OG0000000
Cre08.g373300.t1.1 pacid=30773640 transcript=Cre08.g373300.t1.1
locus=Cre08.g373300 ID=Cre08.g373300.t1.1.v5.5 annot-version=v5.5
Chlamydomonas_reinhardtii OG0000000
Cre14.g625050.t1.1 pacid=30776708 transcript=Cre14.g625050.t1.1
locus=Cre14.g625050 ID=Cre14.g625050.t1.1.v5.5 annot-version=v5.5
Chlamydomonas_reinhardtii OG0000000
Cre16.g689550.t1.1 pacid=30776905 transcript=Cre16.g689550.t1.1
locus=Cre16.g689550 ID=Cre16.g689550.t1.1.v5.5 annot-version=v5.5
Chlamydomonas_reinhardtii OG0000000
Cre16.g679893.t1.1 pacid=30777060 transcript=Cre16.g679893.t1.1
locus=Cre16.g679893 ID=Cre16.g679893.t1.1.v5.5 annot-version=v5.5
Chlamydomonas_reinhardtii OG0000000
Cre16.g689300.t1.1 pacid=30777075 transcript=Cre16.g689300.t1.1
locus=Cre16.g689300 ID=Cre16.g689300.t1.1.v5.5 annot-version=v5.5
Chlamydomonas_reinhardtii OG0000000
Cre16.g654900.t1.1 pacid=30777367 transcript=Cre16.g654900.t1.1
locus=Cre16.g654900 ID=Cre16.g654900.t1.1.v5.5 annot-version=v5.5
Chlamydomonas_reinhardtii OG0000000
Cre16.g659400.t1.1 pacid=30777813 transcript=Cre16.g659400.t1.1
locus=Cre16.g659400 ID=Cre16.g659400.t1.1.v5.5 annot-version=v5.5
Chlamydomonas_reinhardtii OG0000000
Cre16.g666300.t1.1 pacid=30777967 transcript=Cre16.g666300.t1.1
locus=Cre16.g666300 ID=Cre16.g666300.t1.1.v5.5 annot-version=v5.5
Chlamydomonas_reinhardtii OG0000000
Cre16.g654850.t1.1 pacid=30777988 transcript=Cre16.g654850.t1.1
locus=Cre16.g654850 ID=Cre16.g654850.t1.1.v5.5 annot-version=v5.5
Chlamydomonas_reinhardtii OG0000000
Cre16.g695218.t1.2 pacid=30778057 transcript=Cre16.g695218.t1.2
locus=Cre16.g695218 ID=Cre16.g695218.t1.2.v5.5 annot-version=v5.5

Chlamydomonas_reinhardtii OG0000000
Cre16.g694550.t1.1 pacid=30778131 transcript=Cre16.g694550.t1.1
locus=Cre16.g694550 ID=Cre16.g694550.t1.1.v5.5 annot-version=v5.5
Chlamydomonas_reinhardtii OG0000000
Cre16.g683650.t1.2 pacid=30778189 transcript=Cre16.g683650.t1.2
locus=Cre16.g683650 ID=Cre16.g683650.t1.2.v5.5 annot-version=v5.5
Chlamydomonas_reinhardtii OG0000000
Cre42.g760447.t1.1 pacid=30778295 transcript=Cre42.g760447.t1.1
locus=Cre42.g760447 ID=Cre42.g760447.t1.1.v5.5 annot-version=v5.5
Chlamydomonas_reinhardtii OG0000000
Cre19.g751147.t3.1 pacid=30778334 transcript=Cre19.g751147.t3.1
locus=Cre19.g751147 ID=Cre19.g751147.t3.1.v5.5 annot-version=v5.5
Chlamydomonas_reinhardtii OG0000000
Cre19.g750647.t1.1 pacid=30778350 transcript=Cre19.g750647.t1.1
locus=Cre19.g750647 ID=Cre19.g750647.t1.1.v5.5 annot-version=v5.5
Chlamydomonas_reinhardtii OG0000000
Cre06.g301000.t1.2 pacid=30778863 transcript=Cre06.g301000.t1.2
locus=Cre06.g301000 ID=Cre06.g301000.t1.2.v5.5 annot-version=v5.5
Chlamydomonas_reinhardtii OG0000000
Cre08.g384900.t1.2 pacid=30773933 transcript=Cre08.g384900.t1.2
locus=Cre08.g384900 ID=Cre08.g384900.t1.2.v5.5 annot-version=v5.5
Chlamydomonas_reinhardtii OG0000000
Cre06.g253200.t1.1 pacid=30779646 transcript=Cre06.g253200.t1.1
locus=Cre06.g253200 ID=Cre06.g253200.t1.1.v5.5 annot-version=v5.5
Chlamydomonas_reinhardtii OG0000000
Cre06.g277300.t1.1 pacid=30779740 transcript=Cre06.g277300.t1.1
locus=Cre06.g277300 ID=Cre06.g277300.t1.1.v5.5 annot-version=v5.5
Chlamydomonas_reinhardtii OG0000000
Cre06.g293300.t1.1 pacid=30779758 transcript=Cre06.g293300.t1.1
locus=Cre06.g293300 ID=Cre06.g293300.t1.1.v5.5 annot-version=v5.5
Chlamydomonas_reinhardtii OG0000000
Cre09.g405900.t1.1 pacid=30780312 transcript=Cre09.g405900.t1.1
locus=Cre09.g405900 ID=Cre09.g405900.t1.1.v5.5 annot-version=v5.5
Chlamydomonas_reinhardtii OG0000000
Cre09.g387950.t1.1 pacid=30780350 transcript=Cre09.g387950.t1.1
locus=Cre09.g387950 ID=Cre09.g387950.t1.1.v5.5 annot-version=v5.5
Chlamydomonas_reinhardtii OG0000000
Cre08.g379500.t1.1 pacid=30774073 transcript=Cre08.g379500.t1.1
locus=Cre08.g379500 ID=Cre08.g379500.t1.1.v5.5 annot-version=v5.5
Chlamydomonas_reinhardtii OG0000000
Cre09.g404050.t1.1 pacid=30780528 transcript=Cre09.g404050.t1.1
locus=Cre09.g404050 ID=Cre09.g404050.t1.1.v5.5 annot-version=v5.5
Chlamydomonas_reinhardtii OG0000000
Cre09.g390300.t1.1 pacid=30780564 transcript=Cre09.g390300.t1.1
locus=Cre09.g390300 ID=Cre09.g390300.t1.1.v5.5 annot-version=v5.5
Chlamydomonas_reinhardtii OG0000000
Cre08.g385000.t1.2 pacid=30774083 transcript=Cre08.g385000.t1.2
locus=Cre08.g385000 ID=Cre08.g385000.t1.2.v5.5 annot-version=v5.5
Chlamydomonas_reinhardtii OG0000000
Cre09.g388875.t1.1 pacid=30780668 transcript=Cre09.g388875.t1.1
locus=Cre09.g388875 ID=Cre09.g388875.t1.1.v5.5 annot-version=v5.5
Chlamydomonas_reinhardtii OG0000000
Cre09.g393062.t1.1 pacid=30781189 transcript=Cre09.g393062.t1.1
locus=Cre09.g393062 ID=Cre09.g393062.t1.1.v5.5 annot-version=v5.5

Chlamydomonas_reinhardtii OG0000000
Cre09.g387023.t1.1 pacid=30781259 transcript=Cre09.g387023.t1.1
locus=Cre09.g387023 ID=Cre09.g387023.t1.1.v5.5 annot-version=v5.5
Chlamydomonas_reinhardtii OG0000000
Cre09.g390097.t1.1 pacid=30781315 transcript=Cre09.g390097.t1.1
locus=Cre09.g390097 ID=Cre09.g390097.t1.1.v5.5 annot-version=v5.5
Chlamydomonas_reinhardtii OG0000000
Cre09.g410332.t1.2 pacid=30781387 transcript=Cre09.g410332.t1.2
locus=Cre09.g410332 ID=Cre09.g410332.t1.2.v5.5 annot-version=v5.5
Chlamydomonas_reinhardtii OG0000000
Cre09.g388700.t1.1 pacid=30781464 transcript=Cre09.g388700.t1.1
locus=Cre09.g388700 ID=Cre09.g388700.t1.1.v5.5 annot-version=v5.5
Chlamydomonas_reinhardtii OG0000000
Cre17.g713025.t1.1 pacid=30781704 transcript=Cre17.g713025.t1.1
locus=Cre17.g713025 ID=Cre17.g713025.t1.1.v5.5 annot-version=v5.5
Chlamydomonas_reinhardtii OG0000000
Cre17.g741900.t1.1 pacid=30781725 transcript=Cre17.g741900.t1.1
locus=Cre17.g741900 ID=Cre17.g741900.t1.1.v5.5 annot-version=v5.5
Chlamydomonas_reinhardtii OG0000000
Cre17.g696900.t1.1 pacid=30781746 transcript=Cre17.g696900.t1.1
locus=Cre17.g696900 ID=Cre17.g696900.t1.1.v5.5 annot-version=v5.5
Chlamydomonas_reinhardtii OG0000000
Cre17.g696950.t1.1 pacid=30782029 transcript=Cre17.g696950.t1.1
locus=Cre17.g696950 ID=Cre17.g696950.t1.1.v5.5 annot-version=v5.5
Chlamydomonas_reinhardtii OG0000000
Cre07.g341251.t1.1 pacid=30774231 transcript=Cre07.g341251.t1.1
locus=Cre07.g341251 ID=Cre07.g341251.t1.1.v5.5 annot-version=v5.5
Chlamydomonas_reinhardtii OG0000000
Cre17.g743847.t1.1 pacid=30782211 transcript=Cre17.g743847.t1.1
locus=Cre17.g743847 ID=Cre17.g743847.t1.1.v5.5 annot-version=v5.5
Chlamydomonas_reinhardtii OG0000000
Cre17.g739250.t1.1 pacid=30782520 transcript=Cre17.g739250.t1.1
locus=Cre17.g739250 ID=Cre17.g739250.t1.1.v5.5 annot-version=v5.5
Chlamydomonas_reinhardtii OG0000000
Cre17.g736250.t1.2 pacid=30782655 transcript=Cre17.g736250.t1.2
locus=Cre17.g736250 ID=Cre17.g736250.t1.2.v5.5 annot-version=v5.5
Chlamydomonas_reinhardtii OG0000000
Cre17.g742400.t1.1 pacid=30782710 transcript=Cre17.g742400.t1.1
locus=Cre17.g742400 ID=Cre17.g742400.t1.1.v5.5 annot-version=v5.5
Chlamydomonas_reinhardtii OG0000000
Cre17.g702600.t1.1 pacid=30782718 transcript=Cre17.g702600.t1.1
locus=Cre17.g702600 ID=Cre17.g702600.t1.1.v5.5 annot-version=v5.5
Chlamydomonas_reinhardtii OG0000000
Cre17.g710000.t1.1 pacid=30782774 transcript=Cre17.g710000.t1.1
locus=Cre17.g710000 ID=Cre17.g710000.t1.1.v5.5 annot-version=v5.5
Chlamydomonas_reinhardtii OG0000000
Cre05.g236000.t1.1 pacid=30782953 transcript=Cre05.g236000.t1.1
locus=Cre05.g236000 ID=Cre05.g236000.t1.1.v5.5 annot-version=v5.5
Chlamydomonas_reinhardtii OG0000000
Cre05.g236216.t1.1 pacid=30783192 transcript=Cre05.g236216.t1.1
locus=Cre05.g236216 ID=Cre05.g236216.t1.1.v5.5 annot-version=v5.5
Chlamydomonas_reinhardtii OG0000000
Cre15.g635700.t1.1 pacid=30783583 transcript=Cre15.g635700.t1.1
locus=Cre15.g635700 ID=Cre15.g635700.t1.1.v5.5 annot-version=v5.5

Chlamydomonas_reinhardtii OG0000000
Cre07.g319350.t1.1 pacid=30774381 transcript=Cre07.g319350.t1.1
locus=Cre07.g319350 ID=Cre07.g319350.t1.1.v5.5 annot-version=v5.5
Chlamydomonas_reinhardtii OG0000000
Cre15.g641100.t1.1 pacid=30783593 transcript=Cre15.g641100.t1.1
locus=Cre15.g641100 ID=Cre15.g641100.t1.1.v5.5 annot-version=v5.5
Chlamydomonas_reinhardtii OG0000000
Cre15.g639503.t1.1 pacid=30783719 transcript=Cre15.g639503.t1.1
locus=Cre15.g639503 ID=Cre15.g639503.t1.1.v5.5 annot-version=v5.5
Chlamydomonas_reinhardtii OG0000000
Cre13.g574250.t1.1 pacid=30784021 transcript=Cre13.g574250.t1.1
locus=Cre13.g574250 ID=Cre13.g574250.t1.1.v5.5 annot-version=v5.5
Chlamydomonas_reinhardtii OG0000000
Cre13.g604350.t1.1 pacid=30784028 transcript=Cre13.g604350.t1.1
locus=Cre13.g604350 ID=Cre13.g604350.t1.1.v5.5 annot-version=v5.5
Chlamydomonas_reinhardtii OG0000000
Cre13.g591050.t1.2 pacid=30784208 transcript=Cre13.g591050.t1.2
locus=Cre13.g591050 ID=Cre13.g591050.t1.2.v5.5 annot-version=v5.5
Chlamydomonas_reinhardtii OG0000000
Cre13.g576600.t1.2 pacid=30784227 transcript=Cre13.g576600.t1.2
locus=Cre13.g576600 ID=Cre13.g576600.t1.2.v5.5 annot-version=v5.5
Chlamydomonas_reinhardtii OG0000000
Cre13.g603600.t1.1 pacid=30784239 transcript=Cre13.g603600.t1.1
locus=Cre13.g603600 ID=Cre13.g603600.t1.1.v5.5 annot-version=v5.5
Chlamydomonas_reinhardtii OG0000000
Cre13.g605000.t1.1 pacid=30784339 transcript=Cre13.g605000.t1.1
locus=Cre13.g605000 ID=Cre13.g605000.t1.1.v5.5 annot-version=v5.5
Chlamydomonas_reinhardtii OG0000000
Cre13.g576550.t2.1 pacid=30784419 transcript=Cre13.g576550.t2.1
locus=Cre13.g576550 ID=Cre13.g576550.t2.1.v5.5 annot-version=v5.5
Chlamydomonas_reinhardtii OG0000000
Cre13.g565200.t1.1 pacid=30784448 transcript=Cre13.g565200.t1.1
locus=Cre13.g565200 ID=Cre13.g565200.t1.1.v5.5 annot-version=v5.5
Chlamydomonas_reinhardtii OG0000000
Cre13.g574300.t1.1 pacid=30784526 transcript=Cre13.g574300.t1.1
locus=Cre13.g574300 ID=Cre13.g574300.t1.1.v5.5 annot-version=v5.5
Chlamydomonas_reinhardtii OG0000000
Cre13.g567200.t1.1 pacid=30784557 transcript=Cre13.g567200.t1.1
locus=Cre13.g567200 ID=Cre13.g567200.t1.1.v5.5 annot-version=v5.5
Chlamydomonas_reinhardtii OG0000000
Cre13.g574350.t1.2 pacid=30784619 transcript=Cre13.g574350.t1.2
locus=Cre13.g574350 ID=Cre13.g574350.t1.2.v5.5 annot-version=v5.5
Chlamydomonas_reinhardtii OG0000000
Cre13.g589500.t1.1 pacid=30784702 transcript=Cre13.g589500.t1.1
locus=Cre13.g589500 ID=Cre13.g589500.t1.1.v5.5 annot-version=v5.5
Chlamydomonas_reinhardtii OG0000000
Cre13.g566450.t1.2 pacid=30784705 transcript=Cre13.g566450.t1.2
locus=Cre13.g566450 ID=Cre13.g566450.t1.2.v5.5 annot-version=v5.5
Chlamydomonas_reinhardtii OG0000000
Cre02.g117813.t1.2 pacid=30784788 transcript=Cre02.g117813.t1.2
locus=Cre02.g117813 ID=Cre02.g117813.t1.2.v5.5 annot-version=v5.5
Chlamydomonas_reinhardtii OG0000000
Vocar.0030s0184.1.p pacid=32894602 transcript=Vocar.0030s0184.1
locus=Vocar.0030s0184 ID=Vocar.0030s0184.1.v2.1 annot-version=v2.1

Volvox_carteri	OG0000000
Vocar.0012s0118.1.p pacid=32894815 transcript=Vocar.0012s0118.1
locus=Vocar.0012s0118 ID=Vocar.0012s0118.1.v2.1 annot-version=v2.1
Volvox_carteri	OG0000000
Vocar.0012s0222.1.p pacid=32895084 transcript=Vocar.0012s0222.1
locus=Vocar.0012s0222 ID=Vocar.0012s0222.1.v2.1 annot-version=v2.1
Volvox_carteri	OG0000000
Vocar.0012s0089.1.p pacid=32895094 transcript=Vocar.0012s0089.1
locus=Vocar.0012s0089 ID=Vocar.0012s0089.1.v2.1 annot-version=v2.1
Volvox_carteri	OG0000000
Vocar.0001s0642.1.p pacid=32884385 transcript=Vocar.0001s0642.1
locus=Vocar.0001s0642 ID=Vocar.0001s0642.1.v2.1 annot-version=v2.1
Volvox_carteri	OG0000000
Vocar.0028s0037.1.p pacid=32895565 transcript=Vocar.0028s0037.1
locus=Vocar.0028s0037 ID=Vocar.0028s0037.1.v2.1 annot-version=v2.1
Volvox_carteri	OG0000000
Vocar.0028s0015.1.p pacid=32895620 transcript=Vocar.0028s0015.1
locus=Vocar.0028s0015 ID=Vocar.0028s0015.1.v2.1 annot-version=v2.1
Volvox_carteri	OG0000000
Vocar.0028s0112.1.p pacid=32895652 transcript=Vocar.0028s0112.1
locus=Vocar.0028s0112 ID=Vocar.0028s0112.1.v2.1 annot-version=v2.1
Volvox_carteri	OG0000000
Vocar.0075s0003.1.p pacid=32895796 transcript=Vocar.0075s0003.1
locus=Vocar.0075s0003 ID=Vocar.0075s0003.1.v2.1 annot-version=v2.1
Volvox_carteri	OG0000000
Vocar.0021s0146.1.p pacid=32895883 transcript=Vocar.0021s0146.1
locus=Vocar.0021s0146 ID=Vocar.0021s0146.1.v2.1 annot-version=v2.1
Volvox_carteri	OG0000000
Vocar.0071s0002.1.p pacid=32896145 transcript=Vocar.0071s0002.1
locus=Vocar.0071s0002 ID=Vocar.0071s0002.1.v2.1 annot-version=v2.1
Volvox_carteri	OG0000000
Vocar.0044s0024.1.p pacid=32896614 transcript=Vocar.0044s0024.1
locus=Vocar.0044s0024 ID=Vocar.0044s0024.1.v2.1 annot-version=v2.1
Volvox_carteri	OG0000000
Vocar.0001s1081.1.p pacid=32884536 transcript=Vocar.0001s1081.1
locus=Vocar.0001s1081 ID=Vocar.0001s1081.1.v2.1 annot-version=v2.1
Volvox_carteri	OG0000000
Vocar.0001s1519.1.p pacid=32884539 transcript=Vocar.0001s1519.1
locus=Vocar.0001s1519 ID=Vocar.0001s1519.1.v2.1 annot-version=v2.1
Volvox_carteri	OG0000000
Vocar.0035s0068.1.p pacid=32896812 transcript=Vocar.0035s0068.1
locus=Vocar.0035s0068 ID=Vocar.0035s0068.1.v2.1 annot-version=v2.1
Volvox_carteri	OG0000000
Vocar.0035s0070.1.p pacid=32896920 transcript=Vocar.0035s0070.1
locus=Vocar.0035s0070 ID=Vocar.0035s0070.1.v2.1 annot-version=v2.1
Volvox_carteri	OG0000000
Vocar.0035s0024.1.p pacid=32896933 transcript=Vocar.0035s0024.1
locus=Vocar.0035s0024 ID=Vocar.0035s0024.1.v2.1 annot-version=v2.1
Volvox_carteri	OG0000000
Vocar.0008s0103.1.p pacid=32896980 transcript=Vocar.0008s0103.1
locus=Vocar.0008s0103 ID=Vocar.0008s0103.1.v2.1 annot-version=v2.1
Volvox_carteri	OG0000000
Vocar.0008s0100.1.p pacid=32897061 transcript=Vocar.0008s0100.1
locus=Vocar.0008s0100 ID=Vocar.0008s0100.1.v2.1 annot-version=v2.1

Volvox_carteri	OG0000000
Vocar.0008s0128.1.p pacid=32897350 transcript=Vocar.0008s0128.1 locus=Vocar.0008s0128 ID=Vocar.0008s0128.1.v2.1 annot-version=v2.1
Volvox_carteri	OG0000000
Vocar.0039s0089.1.p pacid=32897813 transcript=Vocar.0039s0089.1 locus=Vocar.0039s0089 ID=Vocar.0039s0089.1.v2.1 annot-version=v2.1
Volvox_carteri	OG0000000
Vocar.0016s0280.1.p pacid=32898035 transcript=Vocar.0016s0280.1 locus=Vocar.0016s0280 ID=Vocar.0016s0280.1.v2.1 annot-version=v2.1
Volvox_carteri	OG0000000
Vocar.0016s0054.1.p pacid=32898183 transcript=Vocar.0016s0054.1 locus=Vocar.0016s0054 ID=Vocar.0016s0054.1.v2.1 annot-version=v2.1
Volvox_carteri	OG0000000
Vocar.0018s0276.1.p pacid=32898604 transcript=Vocar.0018s0276.1 locus=Vocar.0018s0276 ID=Vocar.0018s0276.1.v2.1 annot-version=v2.1
Volvox_carteri	OG0000000
Vocar.0018s0005.1.p pacid=32898641 transcript=Vocar.0018s0005.1 locus=Vocar.0018s0005 ID=Vocar.0018s0005.1.v2.1 annot-version=v2.1
Volvox_carteri	OG0000000
Vocar.0015s0377.1.p pacid=32898907 transcript=Vocar.0015s0377.1 locus=Vocar.0015s0377 ID=Vocar.0015s0377.1.v2.1 annot-version=v2.1
Volvox_carteri	OG0000000
Vocar.0015s0388.1.p pacid=32899235 transcript=Vocar.0015s0388.1 locus=Vocar.0015s0388 ID=Vocar.0015s0388.1.v2.1 annot-version=v2.1
Volvox_carteri	OG0000000
Vocar.0001s0157.1.p pacid=32884941 transcript=Vocar.0001s0157.1 locus=Vocar.0001s0157 ID=Vocar.0001s0157.1.v2.1 annot-version=v2.1
Volvox_carteri	OG0000000
Vocar.0001s0133.1.p pacid=32884998 transcript=Vocar.0001s0133.1 locus=Vocar.0001s0133 ID=Vocar.0001s0133.1.v2.1 annot-version=v2.1
Volvox_carteri	OG0000000
Vocar.0001s1577.1.p pacid=32885393 transcript=Vocar.0001s1577.1 locus=Vocar.0001s1577 ID=Vocar.0001s1577.1.v2.1 annot-version=v2.1
Volvox_carteri	OG0000000
Vocar.0001s1513.1.p pacid=32885426 transcript=Vocar.0001s1513.1 locus=Vocar.0001s1513 ID=Vocar.0001s1513.1.v2.1 annot-version=v2.1
Volvox_carteri	OG0000000
Vocar.0001s0647.1.p pacid=32885639 transcript=Vocar.0001s0647.1 locus=Vocar.0001s0647 ID=Vocar.0001s0647.1.v2.1 annot-version=v2.1
Volvox_carteri	OG0000000
Vocar.0001s0134.1.p pacid=32885705 transcript=Vocar.0001s0134.1 locus=Vocar.0001s0134 ID=Vocar.0001s0134.1.v2.1 annot-version=v2.1
Volvox_carteri	OG0000000
Vocar.0001s0307.1.p pacid=32885717 transcript=Vocar.0001s0307.1 locus=Vocar.0001s0307 ID=Vocar.0001s0307.1.v2.1 annot-version=v2.1
Volvox_carteri	OG0000000
Vocar.0001s1563.1.p pacid=32885930 transcript=Vocar.0001s1563.1 locus=Vocar.0001s1563 ID=Vocar.0001s1563.1.v2.1 annot-version=v2.1
Volvox_carteri	OG0000000
Vocar.0001s0132.1.p pacid=32885945 transcript=Vocar.0001s0132.1 locus=Vocar.0001s0132 ID=Vocar.0001s0132.1.v2.1 annot-version=v2.1
Volvox_carteri	OG0000000
Vocar.0040s0019.1.p pacid=32886204 transcript=Vocar.0040s0019.1 locus=Vocar.0040s0019 ID=Vocar.0040s0019.1.v2.1 annot-version=v2.1

Volvox_carteri   OG0000000
Vocar.0040s0006.1.p pacid=32886244 transcript=Vocar.0040s0006.1
locus=Vocar.0040s0006 ID=Vocar.0040s0006.1.v2.1 annot-version=v2.1
Volvox_carteri   OG0000000
Vocar.0040s0033.1.p pacid=32886294 transcript=Vocar.0040s0033.1
locus=Vocar.0040s0033 ID=Vocar.0040s0033.1.v2.1 annot-version=v2.1
Volvox_carteri   OG0000000
Vocar.0033s0172.1.p pacid=32886325 transcript=Vocar.0033s0172.1
locus=Vocar.0033s0172 ID=Vocar.0033s0172.1.v2.1 annot-version=v2.1
Volvox_carteri   OG0000000
Vocar.0065s0016.1.p pacid=32886854 transcript=Vocar.0065s0016.1
locus=Vocar.0065s0016 ID=Vocar.0065s0016.1.v2.1 annot-version=v2.1
Volvox_carteri   OG0000000
Vocar.0043s0007.1.p pacid=32886889 transcript=Vocar.0043s0007.1
locus=Vocar.0043s0007 ID=Vocar.0043s0007.1.v2.1 annot-version=v2.1
Volvox_carteri   OG0000000
Vocar.0027s0155.1.p pacid=32887180 transcript=Vocar.0027s0155.1
locus=Vocar.0027s0155 ID=Vocar.0027s0155.1.v2.1 annot-version=v2.1
Volvox_carteri   OG0000000
Vocar.0027s0079.1.p pacid=32887200 transcript=Vocar.0027s0079.1
locus=Vocar.0027s0079 ID=Vocar.0027s0079.1.v2.1 annot-version=v2.1
Volvox_carteri   OG0000000
Vocar.0027s0042.1.p pacid=32887232 transcript=Vocar.0027s0042.1
locus=Vocar.0027s0042 ID=Vocar.0027s0042.1.v2.1 annot-version=v2.1
Volvox_carteri   OG0000000
Vocar.0027s0171.1.p pacid=32887248 transcript=Vocar.0027s0171.1
locus=Vocar.0027s0171 ID=Vocar.0027s0171.1.v2.1 annot-version=v2.1
Volvox_carteri   OG0000000
Vocar.0007s0314.1.p pacid=32888009 transcript=Vocar.0007s0314.1
locus=Vocar.0007s0314 ID=Vocar.0007s0314.1.v2.1 annot-version=v2.1
Volvox_carteri   OG0000000
Vocar.0007s0186.1.p pacid=32888264 transcript=Vocar.0007s0186.1
locus=Vocar.0007s0186 ID=Vocar.0007s0186.1.v2.1 annot-version=v2.1
Volvox_carteri   OG0000000
Vocar.0014s0287.1.p pacid=32888399 transcript=Vocar.0014s0287.1
locus=Vocar.0014s0287 ID=Vocar.0014s0287.1.v2.1 annot-version=v2.1
Volvox_carteri   OG0000000
Vocar.0003s0101.1.p pacid=32888865 transcript=Vocar.0003s0101.1
locus=Vocar.0003s0101 ID=Vocar.0003s0101.1.v2.1 annot-version=v2.1
Volvox_carteri   OG0000000
Vocar.0003s0123.1.p pacid=32888963 transcript=Vocar.0003s0123.1
locus=Vocar.0003s0123 ID=Vocar.0003s0123.1.v2.1 annot-version=v2.1
Volvox_carteri   OG0000000
Vocar.0003s0182.1.p pacid=32889264 transcript=Vocar.0003s0182.1
locus=Vocar.0003s0182 ID=Vocar.0003s0182.1.v2.1 annot-version=v2.1
Volvox_carteri   OG0000000
Vocar.0004s0042.1.p pacid=32889564 transcript=Vocar.0004s0042.1
locus=Vocar.0004s0042 ID=Vocar.0004s0042.1.v2.1 annot-version=v2.1
Volvox_carteri   OG0000000
Vocar.0004s0127.1.p pacid=32889966 transcript=Vocar.0004s0127.1
locus=Vocar.0004s0127 ID=Vocar.0004s0127.1.v2.1 annot-version=v2.1
Volvox_carteri   OG0000000
Vocar.0024s0188.1.p pacid=32890386 transcript=Vocar.0024s0188.1
locus=Vocar.0024s0188 ID=Vocar.0024s0188.1.v2.1 annot-version=v2.1

Volvox_carteri   OG0000000
Vocar.0002s0669.1.p pacid=32890566 transcript=Vocar.0002s0669.1
locus=Vocar.0002s0669 ID=Vocar.0002s0669.1.v2.1 annot-version=v2.1
Volvox_carteri   OG0000000
Vocar.0002s0319.1.p pacid=32890748 transcript=Vocar.0002s0319.1
locus=Vocar.0002s0319 ID=Vocar.0002s0319.1.v2.1 annot-version=v2.1
Volvox_carteri   OG0000000
Vocar.0002s0324.1.p pacid=32890803 transcript=Vocar.0002s0324.1
locus=Vocar.0002s0324 ID=Vocar.0002s0324.1.v2.1 annot-version=v2.1
Volvox_carteri   OG0000000
Vocar.0002s0320.1.p pacid=32890946 transcript=Vocar.0002s0320.1
locus=Vocar.0002s0320 ID=Vocar.0002s0320.1.v2.1 annot-version=v2.1
Volvox_carteri   OG0000000
Vocar.0002s0322.1.p pacid=32891053 transcript=Vocar.0002s0322.1
locus=Vocar.0002s0322 ID=Vocar.0002s0322.1.v2.1 annot-version=v2.1
Volvox_carteri   OG0000000
Vocar.0002s0559.1.p pacid=32891055 transcript=Vocar.0002s0559.1
locus=Vocar.0002s0559 ID=Vocar.0002s0559.1.v2.1 annot-version=v2.1
Volvox_carteri   OG0000000
Vocar.0002s0434.1.p pacid=32891111 transcript=Vocar.0002s0434.1
locus=Vocar.0002s0434 ID=Vocar.0002s0434.1.v2.1 annot-version=v2.1
Volvox_carteri   OG0000000
Vocar.0076s0008.1.p pacid=32891300 transcript=Vocar.0076s0008.1
locus=Vocar.0076s0008 ID=Vocar.0076s0008.1.v2.1 annot-version=v2.1
Volvox_carteri   OG0000000
Vocar.0017s0168.1.p pacid=32891366 transcript=Vocar.0017s0168.1
locus=Vocar.0017s0168 ID=Vocar.0017s0168.1.v2.1 annot-version=v2.1
Volvox_carteri   OG0000000
Vocar.0017s0169.1.p pacid=32891456 transcript=Vocar.0017s0169.1
locus=Vocar.0017s0169 ID=Vocar.0017s0169.1.v2.1 annot-version=v2.1
Volvox_carteri   OG0000000
Vocar.0017s0004.1.p pacid=32891546 transcript=Vocar.0017s0004.1
locus=Vocar.0017s0004 ID=Vocar.0017s0004.1.v2.1 annot-version=v2.1
Volvox_carteri   OG0000000
Vocar.0066s0010.1.p pacid=32891753 transcript=Vocar.0066s0010.1
locus=Vocar.0066s0010 ID=Vocar.0066s0010.1.v2.1 annot-version=v2.1
Volvox_carteri   OG0000000
Vocar.0026s0031.1.p pacid=32892494 transcript=Vocar.0026s0031.1
locus=Vocar.0026s0031 ID=Vocar.0026s0031.1.v2.1 annot-version=v2.1
Volvox_carteri   OG0000000
Vocar.0026s0164.1.p pacid=32892554 transcript=Vocar.0026s0164.1
locus=Vocar.0026s0164 ID=Vocar.0026s0164.1.v2.1 annot-version=v2.1
Volvox_carteri   OG0000000
Vocar.0026s0032.1.p pacid=32892584 transcript=Vocar.0026s0032.1
locus=Vocar.0026s0032 ID=Vocar.0026s0032.1.v2.1 annot-version=v2.1
Volvox_carteri   OG0000000
Vocar.0019s0002.1.p pacid=32892870 transcript=Vocar.0019s0002.1
locus=Vocar.0019s0002 ID=Vocar.0019s0002.1.v2.1 annot-version=v2.1
Volvox_carteri   OG0000000
Vocar.0062s0015.1.p pacid=32892929 transcript=Vocar.0062s0015.1
locus=Vocar.0062s0015 ID=Vocar.0062s0015.1.v2.1 annot-version=v2.1
Volvox_carteri   OG0000000
Vocar.0001s1741.1.p pacid=32884170 transcript=Vocar.0001s1741.1
locus=Vocar.0001s1741 ID=Vocar.0001s1741.1.v2.1 annot-version=v2.1

Volvox_carteri   OG0000000
Vocar.0034s0092.1.p pacid=32893116 transcript=Vocar.0034s0092.1
locus=Vocar.0034s0092 ID=Vocar.0034s0092.1.v2.1 annot-version=v2.1
Volvox_carteri   OG0000000
Vocar.0009s0367.1.p pacid=32893164 transcript=Vocar.0009s0367.1
locus=Vocar.0009s0367 ID=Vocar.0009s0367.1.v2.1 annot-version=v2.1
Volvox_carteri   OG0000000
Vocar.0009s0012.1.p pacid=32893200 transcript=Vocar.0009s0012.1
locus=Vocar.0009s0012 ID=Vocar.0009s0012.1.v2.1 annot-version=v2.1
Volvox_carteri   OG0000000
Vocar.0009s0010.1.p pacid=32893522 transcript=Vocar.0009s0010.1
locus=Vocar.0009s0010 ID=Vocar.0009s0010.1.v2.1 annot-version=v2.1
Volvox_carteri   OG0000000
Vocar.0006s0113.1.p pacid=32894083 transcript=Vocar.0006s0113.1
locus=Vocar.0006s0113 ID=Vocar.0006s0113.1.v2.1 annot-version=v2.1
Volvox_carteri   OG0000000
Vocar.0006s0358.1.p pacid=32894171 transcript=Vocar.0006s0358.1
locus=Vocar.0006s0358 ID=Vocar.0006s0358.1.v2.1 annot-version=v2.1
Volvox_carteri   OG0000000
jgi|ChlNC64A_1|19632|e_gw1.3.731.1   Chlorella_variabilis
OG0000000
jgi|ChlNC64A_1|56862|estExt_fgenesh3_pg.C_30100
Chlorella_variabilis   OG0000000
jgi|ChlNC64A_1|29775|estExt_Genewise1.C_30315
Chlorella_variabilis   OG0000000
jgi|ChlNC64A_1|50349|fgenesh3_pg.C_scaffold_3000222
Chlorella_variabilis   OG0000000
jgi|ChlNC64A_1|140750|IGS.gm_3_00489   Chlorella_variabilis
OG0000000
jgi|ChlNC64A_1|29915|estExt_Genewise1.C_30619
Chlorella_variabilis   OG0000000
jgi|ChlNC64A_1|19742|e_gw1.3.413.1   Chlorella_variabilis
OG0000000
jgi|ChlNC64A_1|140910|IGS.gm_3_00649   Chlorella_variabilis
OG0000000
jgi|ChlNC64A_1|140943|IGS.gm_3_00682   Chlorella_variabilis
OG0000000
jgi|ChlNC64A_1|134744|IGS.gm_12_00130   Chlorella_variabilis
OG0000000
jgi|ChlNC64A_1|24126|e_gw1.12.323.1   Chlorella_variabilis
OG0000000
jgi|ChlNC64A_1|23988|e_gw1.12.249.1   Chlorella_variabilis
OG0000000
jgi|ChlNC64A_1|23857|e_gw1.12.401.1   Chlorella_variabilis
OG0000000
jgi|ChlNC64A_1|31062|estExt_Genewise1.C_100011
Chlorella_variabilis   OG0000000
jgi|ChlNC64A_1|133720|IGS.gm_10_00011   Chlorella_variabilis
OG0000000
jgi|ChlNC64A_1|133852|IGS.gm_10_00143   Chlorella_variabilis
OG0000000
jgi|ChlNC64A_1|134046|IGS.gm_10_00337   Chlorella_variabilis
OG0000000
jgi|ChlNC64A_1|141030|IGS.gm_30_00068   Chlorella_variabilis

OG0000000
jgi|ChlNC64A_1|133221|IGS.gm_1_00357	Chlorella_variabilis
OG0000000
jgi|ChlNC64A_1|25821|e_gw1.18.251.1	Chlorella_variabilis
OG0000000
jgi|ChlNC64A_1|137696|IGS.gm_2_00163	Chlorella_variabilis
OG0000000
jgi|ChlNC64A_1|137878|IGS.gm_2_00345	Chlorella_variabilis
OG0000000
jgi|ChlNC64A_1|12394|gw1.2.340.1	Chlorella_variabilis
OG0000000
jgi|ChlNC64A_1|138200|IGS.gm_2_00667	Chlorella_variabilis
OG0000000
jgi|ChlNC64A_1|133304|IGS.gm_1_00440	Chlorella_variabilis
OG0000000
jgi|ChlNC64A_1|50622|fgenesh3_pg.C_scaffold_4000121
Chlorella_variabilis	OG0000000
jgi|ChlNC64A_1|142162|IGS.gm_4_00303	Chlorella_variabilis
OG0000000
jgi|ChlNC64A_1|34388|estExt_Genewise1Plus.C_40254
Chlorella_variabilis	OG0000000
jgi|ChlNC64A_1|57116|estExt_fgenesh3_pg.C_40228
Chlorella_variabilis	OG0000000
jgi|ChlNC64A_1|142311|IGS.gm_4_00452	Chlorella_variabilis
OG0000000
jgi|ChlNC64A_1|142454|IGS.gm_4_00595	Chlorella_variabilis
OG0000000
jgi|ChlNC64A_1|58735|estExt_fgenesh3_pg.C_200038
Chlorella_variabilis	OG0000000
jgi|ChlNC64A_1|138454|IGS.gm_20_00203	Chlorella_variabilis
OG0000000
jgi|ChlNC64A_1|134359|IGS.gm_11_00205	Chlorella_variabilis
OG0000000
jgi|ChlNC64A_1|12761|gw1.11.217.1 Chlorella_variabilis
OG0000000
jgi|ChlNC64A_1|143982|IGS.gm_6_00556	Chlorella_variabilis
OG0000000
jgi|ChlNC64A_1|21563|e_gw1.6.136.1	Chlorella_variabilis
OG0000000
jgi|ChlNC64A_1|145300|IGS.gm_9_00107	Chlorella_variabilis
OG0000000
jgi|ChlNC64A_1|138610|IGS.gm_21_00089	Chlorella_variabilis
OG0000000
jgi|ChlNC64A_1|13714|gw1.21.137.1 Chlorella_variabilis
OG0000000
jgi|ChlNC64A_1|138721|IGS.gm_21_00200	Chlorella_variabilis
OG0000000
jgi|ChlNC64A_1|30642|estExt_Genewise1.C_70034
Chlorella_variabilis	OG0000000
jgi|ChlNC64A_1|133695|IGS.gm_1_00831	Chlorella_variabilis
OG0000000
jgi|ChlNC64A_1|14990|gw1.34.45.1	Chlorella_variabilis
OG0000000
jgi|ChlNC64A_1|13183|gw1.34.32.1	Chlorella_variabilis

OG0000000
jgi|ChlNC64A_1|36812|estExt_Genewise1Plus.C_220006
Chlorella_variabilis OG0000000
jgi|ChlNC64A_1|21329|e_gw1.5.644.1 Chlorella_variabilis
OG0000000
jgi|ChlNC64A_1|51171|fgenesh3_pg.C_scaffold_5000338
Chlorella_variabilis OG0000000
jgi|ChlNC64A_1|143307|IGS.gm_5_00636 Chlorella_variabilis
OG0000000
jgi|ChlNC64A_1|144818|IGS.gm_8_00146 Chlorella_variabilis
OG0000000
jgi|ChlNC64A_1|141510|IGS.gm_35_00022 Chlorella_variabilis
OG0000000
jgi|ChlNC64A_1|136568|IGS.gm_16_00291 Chlorella_variabilis
OG0000000
jgi|ChlNC64A_1|10735|gw1.32.73.1 Chlorella_variabilis
OG0000000
jgi|ChlNC64A_1|58145|estExt_fgenesh3_pg.C_130054
Chlorella_variabilis OG0000000
jgi|ChlNC64A_1|58181|estExt_fgenesh3_pg.C_130122
Chlorella_variabilis OG0000000
jgi|ChlNC64A_1|36848|estExt_Genewise1Plus.C_220151
Chlorella_variabilis OG0000000
jgi|ChlNC64A_1|13628|gw1.22.151.1 Chlorella_variabilis
OG0000000
jgi|ChlNC64A_1|139834|IGS.gm_27_00012 Chlorella_variabilis
OG0000000
jgi|ChlNC64A_1|137363|IGS.gm_19_00130 Chlorella_variabilis
OG0000000
jgi|ChlNC64A_1|13716|gw1.15.198.1 Chlorella_variabilis
OG0000000
jgi|ChlNC64A_1|136055|IGS.gm_15_00151 Chlorella_variabilis
OG0000000
jgi|ChlNC64A_1|54971|fgenesh3_pg.C_scaffold_28000001
Chlorella_variabilis OG0000000
jgi|ChlNC64A_1|29026|e_gw1.231.1.1 Chlorella_variabilis
OG0000000
jgi|ChlNC64A_1|55725|fgenesh3_pg.C_scaffold_130000001
Chlorella_variabilis OG0000000
jgi|ChlNC64A_1|59518|estExt_fgenesh3_pg.C_6530001
Chlorella_variabilis OG0000000
jgi|ChlNC64A_1|142667|IGS.gm_492_00001 Chlorella_variabilis
OG0000000
53391 pacid=27386673 transcript=53391
locus=estExt_fgenesh1_pm.C_70108 ID=53391.2.0.227 annot-version=v2.0
Coccomyxa_subellipsoidea OG0000000
14113 pacid=27387101 transcript=14113 locus=e_gw1.5.360.1
ID=14113.2.0.227 annot-version=v2.0 Coccomyxa_subellipsoidea
OG0000000
45216 pacid=27387656 transcript=45216 locus=fgenesh1_pg.22_#_101
ID=45216.2.0.227 annot-version=v2.0 Coccomyxa_subellipsoidea
OG0000000
45215 pacid=27387754 transcript=45215 locus=fgenesh1_pg.22_#_100
ID=45215.2.0.227 annot-version=v2.0 Coccomyxa_subellipsoidea

OG0000000
48923 pacid=27388344 transcript=48923
locus=estExt_fgenesh1_pg.C_190086 ID=48923.2.0.227 annot-version=v2.0	Coccomyxa_subellipsoidea	OG0000000
47864 pacid=27385908 transcript=47864
locus=estExt_fgenesh1_pg.C_100212 ID=47864.2.0.227 annot-version=v2.0	Coccomyxa_subellipsoidea	OG0000000
19713 pacid=27388403 transcript=19713 locus=e_gw1.19.278.1
ID=19713.2.0.227 annot-version=v2.0	Coccomyxa_subellipsoidea
OG0000000
66339 pacid=27388614 transcript=66339 locus=estExt_Genemark1.C_90142
ID=66339.2.0.227 annot-version=v2.0	Coccomyxa_subellipsoidea
OG0000000
42547 pacid=27385937 transcript=42547 locus=fgenesh1_pg.10_#_50
ID=42547.2.0.227 annot-version=v2.0	Coccomyxa_subellipsoidea
OG0000000
66336 pacid=27388722 transcript=66336 locus=estExt_Genemark1.C_90139
ID=66336.2.0.227 annot-version=v2.0	Coccomyxa_subellipsoidea
OG0000000
55865 pacid=27386012 transcript=55865 locus=Genemark1.4894_g
ID=55865.2.0.227 annot-version=v2.0	Coccomyxa_subellipsoidea
OG0000000
55214 pacid=27389397 transcript=55214 locus=Genemark1.158_g
ID=55214.2.0.227 annot-version=v2.0	Coccomyxa_subellipsoidea
OG0000000
45752 pacid=27389450 transcript=45752
locus=estExt_fgenesh1_pg.C_10096 ID=45752.2.0.227 annot-version=v2.0	Coccomyxa_subellipsoidea	OG0000000
45718 pacid=27389607 transcript=45718
locus=estExt_fgenesh1_pg.C_10022 ID=45718.2.0.227 annot-version=v2.0	Coccomyxa_subellipsoidea	OG0000000
11504 pacid=27389617 transcript=11504 locus=e_gw1.1.613.1
ID=11504.2.0.227 annot-version=v2.0	Coccomyxa_subellipsoidea
OG0000000
61634 pacid=27389958 transcript=61634 locus=Genemark1.2424_g
ID=61634.2.0.227 annot-version=v2.0	Coccomyxa_subellipsoidea
OG0000000
35653 pacid=27390068 transcript=35653 locus=fgenesh1_pm.4_#_55
ID=35653.2.0.227 annot-version=v2.0	Coccomyxa_subellipsoidea
OG0000000
67022 pacid=27386107 transcript=67022
locus=estExt_Genemark1.C_130006 ID=67022.2.0.227 annot-version=v2.0	Coccomyxa_subellipsoidea	OG0000000
64994 pacid=27390508 transcript=64994 locus=estExt_Genemark1.C_30467
ID=64994.2.0.227 annot-version=v2.0	Coccomyxa_subellipsoidea
OG0000000
46522 pacid=27390577 transcript=46522
locus=estExt_fgenesh1_pg.C_30366 ID=46522.2.0.227 annot-version=v2.0	Coccomyxa_subellipsoidea	OG0000000
52725 pacid=27390630 transcript=52725
locus=estExt_fgenesh1_pm.C_30265 ID=52725.2.0.227 annot-version=v2.0	Coccomyxa_subellipsoidea	OG0000000
46388 pacid=27390880 transcript=46388
locus=estExt_fgenesh1_pg.C_30135 ID=46388.2.0.227 annot-version=v2.0

Coccomyxa_subellipsoidea OG0000000
41270 pacid=27391061 transcript=41270 locus=fgenesh1_pg.6_#_73
ID=41270.2.0.227 annot-version=v2.0 Coccomyxa_subellipsoidea
OG0000000
62451 pacid=27391176 transcript=62451 locus=Genemark1.3234_g
ID=62451.2.0.227 annot-version=v2.0 Coccomyxa_subellipsoidea
OG0000000
14600 pacid=27391404 transcript=14600 locus=e_gw1.6.394.1
ID=14600.2.0.227 annot-version=v2.0 Coccomyxa_subellipsoidea
OG0000000
66889 pacid=27391672 transcript=66889
locus=estExt_Genemark1.C_120140 ID=66889.2.0.227 annot-version=v2.0
Coccomyxa_subellipsoidea OG0000000
56332 pacid=27392181 transcript=56332 locus=Genemark1.5361_g
ID=56332.2.0.227 annot-version=v2.0 Coccomyxa_subellipsoidea
OG0000000
15698 pacid=27392413 transcript=15698 locus=e_gw1.8.451.1
ID=15698.2.0.227 annot-version=v2.0 Coccomyxa_subellipsoidea
OG0000000
9882 pacid=27392433 transcript=9882 locus=gw1.8.460.1
ID=9882.2.0.227 annot-version=v2.0 Coccomyxa_subellipsoidea
OG0000000
15848 pacid=27392478 transcript=15848 locus=e_gw1.8.452.1
ID=15848.2.0.227 annot-version=v2.0 Coccomyxa_subellipsoidea
OG0000000
15794 pacid=27392619 transcript=15794 locus=e_gw1.8.275.1
ID=15794.2.0.227 annot-version=v2.0 Coccomyxa_subellipsoidea
OG0000000
8577 pacid=27392633 transcript=8577 locus=gw1.24.69.1
ID=8577.2.0.227 annot-version=v2.0 Coccomyxa_subellipsoidea
OG0000000
20761 pacid=27392697 transcript=20761 locus=e_gw1.24.64.1
ID=20761.2.0.227 annot-version=v2.0 Coccomyxa_subellipsoidea
OG0000000
39671 pacid=27393118 transcript=39671 locus=fgenesh1_pg.2_#_283
ID=39671.2.0.227 annot-version=v2.0 Coccomyxa_subellipsoidea
OG0000000
59252 pacid=27393323 transcript=59252 locus=Genemark1.1214_g
ID=59252.2.0.227 annot-version=v2.0 Coccomyxa_subellipsoidea
OG0000000
35053 pacid=27393659 transcript=35053 locus=fgenesh1_pm.2_#_63
ID=35053.2.0.227 annot-version=v2.0 Coccomyxa_subellipsoidea
OG0000000
64721 pacid=27393667 transcript=64721 locus=estExt_Genemark1.C_20640
ID=64721.2.0.227 annot-version=v2.0 Coccomyxa_subellipsoidea
OG0000000
20652 pacid=27386457 transcript=20652 locus=e_gw1.23.104.1
ID=20652.2.0.227 annot-version=v2.0 Coccomyxa_subellipsoidea
OG0000000
18717 pacid=27394052 transcript=18717 locus=e_gw1.16.320.1
ID=18717.2.0.227 annot-version=v2.0 Coccomyxa_subellipsoidea
OG0000000
26422 pacid=27394096 transcript=26422
locus=estExt_Genewise1.C_250101 ID=26422.2.0.227 annot-version=v2.0

Coccomyxa_subellipsoidea OG0000000
43870 pacid=27394899 transcript=43870 locus=fgenesh1_pg.15_#_51
ID=43870.2.0.227 annot-version=v2.0 Coccomyxa_subellipsoidea
OG0000000
43964 pacid=27394946 transcript=43964 locus=fgenesh1_pg.15_#_145
ID=43964.2.0.227 annot-version=v2.0 Coccomyxa_subellipsoidea
OG0000000
67360 pacid=27394956 transcript=67360
locus=estExt_Genemark1.C_150067 ID=67360.2.0.227 annot-version=v2.0
Coccomyxa_subellipsoidea OG0000000
3487_gw1.09.00.184.1 Ostreococcus_tauri OG0000000
3768_gw1.05.00.236.1 Ostreococcus_tauri OG0000000
3773_gw1.11.00.198.1 Ostreococcus_tauri OG0000000
4180_gw1.11.00.212.1 Ostreococcus_tauri OG0000000
4604_gw1.18.00.122.1 Ostreococcus_tauri OG0000000
21171_e_gw1.14.00.209.1 Ostreococcus_tauri OG0000000
29174_estExt_gwp_GeneWisePlus.C_Chr_12.00010034
Ostreococcus_tauri OG0000000
6503_gw1.04.00.438.1 Ostreococcus_tauri OG0000000
6516_gw1.05.00.448.1 Ostreococcus_tauri OG0000000
7730 pacid=27416395 transcript=7730 locus=gwEuk.5.459.1
ID=7730.2.0.231 annot-version=v2.0 Ostreococcus_lucimarinus
OG0000000
38832 pacid=27416572 transcript=38832 locus=e_gwEuk.5.319.1
ID=38832.2.0.231 annot-version=v2.0 Ostreococcus_lucimarinus
OG0000000
45682 pacid=27416704 transcript=45682
locus=estExt_GenewiseEukaryote.C_Chr_50114 ID=45682.2.0.231 annot-
version=v2.0 Ostreococcus_lucimarinus OG0000000
39208 pacid=27416887 transcript=39208 locus=e_gwEuk.12.171.1
ID=39208.2.0.231 annot-version=v2.0 Ostreococcus_lucimarinus
OG0000000
42011 pacid=27419112 transcript=42011 locus=e_gwEuk.17.220.1
ID=42011.2.0.231 annot-version=v2.0 Ostreococcus_lucimarinus
OG0000000
6189 pacid=27420147 transcript=6189 locus=gwEuk.20.241.1
ID=6189.2.0.231 annot-version=v2.0 Ostreococcus_lucimarinus
OG0000000
3085 pacid=27420640 transcript=3085 locus=gwEuk.11.188.1
ID=3085.2.0.231 annot-version=v2.0 Ostreococcus_lucimarinus
OG0000000
5051 pacid=27420655 transcript=5051 locus=gwEuk.11.262.1
ID=5051.2.0.231 annot-version=v2.0 Ostreococcus_lucimarinus
OG0000000
147656 pacid=27348873 transcript=147696 locus=gw1.12.840.1
ID=147696.3.0.228 annot-version=v3.0 Micromonas_pusilla
OG0000000
42056 pacid=27349251 transcript=42056
locus=MicpuC2.fgenesh1_pg.C_scaffold_12000005 ID=42056.3.0.228
annot-version=v3.0 Micromonas_pusilla OG0000000
20161 pacid=27340423 transcript=20161 locus=MicpuC2.e_gw1.10.198.1
ID=20161.3.0.228 annot-version=v3.0 Micromonas_pusilla
OG0000000
59318 pacid=27341179 transcript=59318 locus=MicpuC2.EuGene.

0000070167 ID=59318.3.0.228 annot-version=v3.0
Micromonas_pusilla OG0000000
134259 pacid=27341683 transcript=134299 locus=gw1.5.521.1
ID=134299.3.0.228 annot-version=v3.0 Micromonas_pusilla
OG0000000
154975 pacid=27341800 transcript=155015 locus=e_gw1.5.200.1
ID=155015.3.0.228 annot-version=v3.0 Micromonas_pusilla
OG0000000
132746 pacid=27343076 transcript=132786 locus=gw1.2.407.1
ID=132786.3.0.228 annot-version=v3.0 Micromonas_pusilla
OG0000000
8132 pacid=27344736 transcript=8132 locus=MicpuC2.gw1.17.154.1
ID=8132.3.0.228 annot-version=v3.0 Micromonas_pusilla
OG0000000
122206 pacid=27345210 transcript=122246 locus=fgenesh2_kg.
1_#_132_#_4276745:4 ID=122246.3.0.228 annot-version=v3.0
Micromonas_pusilla OG0000000
56536 pacid=27346885 transcript=56536 locus=MicpuC2.EuGene.
0000030690 ID=56536.3.0.228 annot-version=v3.0
Micromonas_pusilla OG0000000
5873 pacid=27346894 transcript=5873 locus=MicpuC2.gw1.3.628.1
ID=5873.3.0.228 annot-version=v3.0 Micromonas_pusilla
OG0000000
45380 pacid=27347098 transcript=45380
locus=MicpuC2.estExt_fgenesh1_pm.C_30089 ID=45380.3.0.228 annot-
version=v3.0 Micromonas_pusilla OG0000000
152406 pacid=27347124 transcript=152446 locus=e_gw1.3.1819.1
ID=152446.3.0.228 annot-version=v3.0 Micromonas_pusilla
OG0000000
7545 pacid=27347941 transcript=7545 locus=MicpuC2.gw1.6.401.1
ID=7545.3.0.228 annot-version=v3.0 Micromonas_pusilla
OG0000000
108286 pacid=27395459 transcript=108286
locus=est_cluster_kg.Chr_05__95__3199543:1 ID=108286.3.0.229 annot-
version=v3.0 Micromonas_sp._RCC299 OG0000000
108013 pacid=27398318 transcript=108013
locus=est_cluster_kg.Chr_03__170__3202253:1 ID=108013.3.0.229 annot-
version=v3.0 Micromonas_sp._RCC299 OG0000000
54846 pacid=27399236 transcript=54846 locus=EuGene.0100010073
ID=54846.3.0.229 annot-version=v3.0 Micromonas_sp._RCC299
OG0000000
81795 pacid=27395697 transcript=81795 locus=e_gw2.05.352.1
ID=81795.3.0.229 annot-version=v3.0 Micromonas_sp._RCC299
OG0000000
62964 pacid=27399726 transcript=62964 locus=EuGene.1200010136
ID=62964.3.0.229 annot-version=v3.0 Micromonas_sp._RCC299
OG0000000
108063 pacid=27400245 transcript=108063
locus=est_cluster_kg.Chr_04__41__3200498:1 ID=108063.3.0.229 annot-
version=v3.0 Micromonas_sp._RCC299 OG0000000
99814 pacid=27400720 transcript=99814
locus=fgenesh2_pg.C_Chr_04000009 ID=99814.3.0.229 annot-version=v3.0
Micromonas_sp._RCC299 OG0000000
64563 pacid=27400810 transcript=64563 locus=EuGene.1500010284

ID=64563.3.0.229 annot-version=v3.0	Micromonas_sp._RCC299
OG0000000
58573 pacid=27395858 transcript=58573 locus=EuGene.0500010306
ID=58573.3.0.229 annot-version=v3.0	Micromonas_sp._RCC299
OG0000000
63499 pacid=27402734 transcript=63499 locus=EuGene.1300010149
ID=63499.3.0.229 annot-version=v3.0	Micromonas_sp._RCC299
OG0000000
69373 pacid=27403725 transcript=69373 locus=gw2.14.195.1
ID=69373.3.0.229 annot-version=v3.0	Micromonas_sp._RCC299
OG0000000
59951 pacid=27404213 transcript=59951 locus=EuGene.0700010236
ID=59951.3.0.229 annot-version=v3.0	Micromonas_sp._RCC299
OG0000000
73365 pacid=27404397 transcript=73365 locus=gw2.07.434.1
ID=73365.3.0.229 annot-version=v3.0	Micromonas_sp._RCC299
OG0000000
59765 pacid=27404532 transcript=59765 locus=EuGene.0700010050
ID=59765.3.0.229 annot-version=v3.0	Micromonas_sp._RCC299
OG0000000
kfl00002_0140_v1.1	Klebsormidium_flaccidum	OG0000000
kfl00333_0130_v1.1	Klebsormidium_flaccidum	OG0000000
kfl00344_0150_v1.1	Klebsormidium_flaccidum	OG0000000
kfl00371_0060_v1.1	Klebsormidium_flaccidum	OG0000000
kfl00015_0490_v1.1	Klebsormidium_flaccidum	OG0000000
kfl00389_0150_v1.1	Klebsormidium_flaccidum	OG0000000
kfl00016_0130_v1.1	Klebsormidium_flaccidum	OG0000000
kfl00409_0120_v1.1	Klebsormidium_flaccidum	OG0000000
kfl00418_0010_v1.1	Klebsormidium_flaccidum	OG0000000
kfl00432_0080_v1.1	Klebsormidium_flaccidum	OG0000000
kfl00438_0120_v1.1	Klebsormidium_flaccidum	OG0000000
kfl00456_0030_v1.1	Klebsormidium_flaccidum	OG0000000
kfl00472_0010_v1.1	Klebsormidium_flaccidum	OG0000000
kfl00472_0020_v1.1	Klebsormidium_flaccidum	OG0000000
kfl00485_0050_v1.1	Klebsormidium_flaccidum	OG0000000
kfl00499_0010_v1.1	Klebsormidium_flaccidum	OG0000000
kfl00499_0040_v1.1	Klebsormidium_flaccidum	OG0000000
kfl00512_0120_v1.1	Klebsormidium_flaccidum	OG0000000
kfl00514_0040_v1.1	Klebsormidium_flaccidum	OG0000000
kfl00520_0010_v1.1	Klebsormidium_flaccidum	OG0000000
kfl00539_0030_v1.1	Klebsormidium_flaccidum	OG0000000
kfl00539_0040_v1.1	Klebsormidium_flaccidum	OG0000000
kfl00547_0050_v1.1	Klebsormidium_flaccidum	OG0000000
kfl00565_0080_v1.1	Klebsormidium_flaccidum	OG0000000
kfl00596_0060_v1.1	Klebsormidium_flaccidum	OG0000000
kfl00622_0040_v1.1	Klebsormidium_flaccidum	OG0000000
kfl00624_0020_v1.1	Klebsormidium_flaccidum	OG0000000
kfl00661_0010_v1.1	Klebsormidium_flaccidum	OG0000000
kfl00667_0030_v1.1	Klebsormidium_flaccidum	OG0000000
kfl00671_0010_v1.1	Klebsormidium_flaccidum	OG0000000
kfl00719_0050_v1.1	Klebsormidium_flaccidum	OG0000000
kfl00724_0030_v1.1	Klebsormidium_flaccidum	OG0000000
kfl00733_0040_v1.1	Klebsormidium_flaccidum	OG0000000
kfl00761_0040_v1.1	Klebsormidium_flaccidum	OG0000000

kfl00797_0025_v1.1	Klebsormidium_flaccidum	OG0000000
kfl00817_0030_v1.1	Klebsormidium_flaccidum	OG0000000
kfl00837_0020_v1.1	Klebsormidium_flaccidum	OG0000000
kfl00904_0030_v1.1	Klebsormidium_flaccidum	OG0000000
kfl00906_0020_v1.1	Klebsormidium_flaccidum	OG0000000
kfl01035_0040_v1.1	Klebsormidium_flaccidum	OG0000000
kfl01060_0040_v1.1	Klebsormidium_flaccidum	OG0000000
kfl01250_0010_v1.1	Klebsormidium_flaccidum	OG0000000
kfl01498_0010_v1.1	Klebsormidium_flaccidum	OG0000000
kfl00027_0130_v1.1	Klebsormidium_flaccidum	OG0000000
kfl00028_0080_v1.1	Klebsormidium_flaccidum	OG0000000
kfl00003_0680_v1.1	Klebsormidium_flaccidum	OG0000000
kfl00040_0260_v1.1	Klebsormidium_flaccidum	OG0000000
kfl00043_0050_v1.1	Klebsormidium_flaccidum	OG0000000
kfl00004_0060_v1.1	Klebsormidium_flaccidum	OG0000000
kfl00045_0420_v1.1	Klebsormidium_flaccidum	OG0000000
kfl00053_0200_v1.1	Klebsormidium_flaccidum	OG0000000
kfl00053_0340_v1.1	Klebsormidium_flaccidum	OG0000000
kfl00055_0120_v1.1	Klebsormidium_flaccidum	OG0000000
kfl00057_0480_v1.1	Klebsormidium_flaccidum	OG0000000
kfl00005_0130_v1.1	Klebsormidium_flaccidum	OG0000000
kfl00005_0200_v1.1	Klebsormidium_flaccidum	OG0000000
kfl00005_0240_v1.1	Klebsormidium_flaccidum	OG0000000
kfl00063_0270_v1.1	Klebsormidium_flaccidum	OG0000000
kfl00065_0090_v1.1	Klebsormidium_flaccidum	OG0000000
kfl00073_0300_v1.1	Klebsormidium_flaccidum	OG0000000
kfl00076_0260_v1.1	Klebsormidium_flaccidum	OG0000000
kfl00084_0110_v1.1	Klebsormidium_flaccidum	OG0000000
kfl00086_0290_v1.1	Klebsormidium_flaccidum	OG0000000
kfl00089_0170_v1.1	Klebsormidium_flaccidum	OG0000000
kfl00089_0190_v1.1	Klebsormidium_flaccidum	OG0000000
kfl00106_0040_v1.1	Klebsormidium_flaccidum	OG0000000
kfl00112_0050_v1.1	Klebsormidium_flaccidum	OG0000000
kfl00112_0210_v1.1	Klebsormidium_flaccidum	OG0000000
kfl00116_0170_v1.1	Klebsormidium_flaccidum	OG0000000
kfl00007_0440_v1.1	Klebsormidium_flaccidum	OG0000000
kfl00136_0140_v1.1	Klebsormidium_flaccidum	OG0000000
kfl00146_0140_v1.1	Klebsormidium_flaccidum	OG0000000
kfl00150_0120_v1.1	Klebsormidium_flaccidum	OG0000000
kfl00150_0200_v1.1	Klebsormidium_flaccidum	OG0000000
kfl00151_0210_v1.1	Klebsormidium_flaccidum	OG0000000
kfl00151_0220_v1.1	Klebsormidium_flaccidum	OG0000000
kfl00164_0070_v1.1	Klebsormidium_flaccidum	OG0000000
kfl00169_0040_v1.1	Klebsormidium_flaccidum	OG0000000
kfl00187_0080_v1.1	Klebsormidium_flaccidum	OG0000000
kfl00204_0070_v1.1	Klebsormidium_flaccidum	OG0000000
kfl00204_0090_v1.1	Klebsormidium_flaccidum	OG0000000
kfl00207_0010_v1.1	Klebsormidium_flaccidum	OG0000000
kfl00210_0010_v1.1	Klebsormidium_flaccidum	OG0000000
kfl00011_0065_v1.1	Klebsormidium_flaccidum	OG0000000
kfl00216_0120_v1.1	Klebsormidium_flaccidum	OG0000000
kfl00221_0080_v1.1	Klebsormidium_flaccidum	OG0000000
kfl00235_0170_v1.1	Klebsormidium_flaccidum	OG0000000
kfl00247_0200_v1.1	Klebsormidium_flaccidum	OG0000000

kfl00012_0320_v1.1	Klebsormidium_flaccidum	OG0000000
kfl00257_0190_v1.1	Klebsormidium_flaccidum	OG0000000
kfl00265_0130_v1.1	Klebsormidium_flaccidum	OG0000000
kfl00013_0260_v1.1	Klebsormidium_flaccidum	OG0000000
kfl00280_0190_v1.1	Klebsormidium_flaccidum	OG0000000
kfl00281_0090_v1.1	Klebsormidium_flaccidum	OG0000000
kfl00284_0070_v1.1	Klebsormidium_flaccidum	OG0000000
kfl00289_0140_v1.1	Klebsormidium_flaccidum	OG0000000
kfl00293_0090_v1.1	Klebsormidium_flaccidum	OG0000000
kfl00300_0120_v1.1	Klebsormidium_flaccidum	OG0000000
kfl00014_0010_v1.1	Klebsormidium_flaccidum	OG0000000
kfl00304_0130_v1.1	Klebsormidium_flaccidum	OG0000000
kfl00308_0120_v1.1	Klebsormidium_flaccidum	OG0000000
kfl00309_0080_v1.1	Klebsormidium_flaccidum	OG0000000
kfl00309_0190_v1.1	Klebsormidium_flaccidum	OG0000000
kfl00310_0150_v1.1	Klebsormidium_flaccidum	OG0000000
kfl00311_0040_v1.1	Klebsormidium_flaccidum	OG0000000
kfl00311_0050_v1.1	Klebsormidium_flaccidum	OG0000000
kfl00311_0060_v1.1	Klebsormidium_flaccidum	OG0000000
kfl00312_0060_v1.1	Klebsormidium_flaccidum	OG0000000
Pp3c7_11290V3.1.p pacid=32926000 transcript=Pp3c7_11290V3.1 locus=Pp3c7_11290 annot-version=v3.3	Physcomitrella_patens	OG0000000
Pp3c7_19750V3.1.p pacid=32926414 transcript=Pp3c7_19750V3.1 locus=Pp3c7_19750 annot-version=v3.3	Physcomitrella_patens	OG0000000
Pp3c15_24250V3.1.p pacid=32926673 transcript=Pp3c15_24250V3.1 locus=Pp3c15_24250 annot-version=v3.3	Physcomitrella_patens	OG0000000
Pp3c15_5330V3.1.p pacid=32926731 transcript=Pp3c15_5330V3.1 locus=Pp3c15_5330 annot-version=v3.3	Physcomitrella_patens	OG0000000
Pp3c15_17314V3.1.p pacid=32926942 transcript=Pp3c15_17314V3.1 locus=Pp3c15_17314 annot-version=v3.3	Physcomitrella_patens	OG0000000
Pp3c15_1540V3.1.p pacid=32927112 transcript=Pp3c15_1540V3.1 locus=Pp3c15_1540 annot-version=v3.3	Physcomitrella_patens	OG0000000
Pp3c15_16340V3.1.p pacid=32927114 transcript=Pp3c15_16340V3.1 locus=Pp3c15_16340 annot-version=v3.3	Physcomitrella_patens	OG0000000
Pp3c15_17640V3.1.p pacid=32927146 transcript=Pp3c15_17640V3.1 locus=Pp3c15_17640 annot-version=v3.3	Physcomitrella_patens	OG0000000
Pp3c15_17310V3.1.p pacid=32927839 transcript=Pp3c15_17310V3.1 locus=Pp3c15_17310 annot-version=v3.3	Physcomitrella_patens	OG0000000
Pp3c15_20810V3.1.p pacid=32929741 transcript=Pp3c15_20810V3.1 locus=Pp3c15_20810 annot-version=v3.3	Physcomitrella_patens	OG0000000
Pp3c15_3950V3.1.p pacid=32929766 transcript=Pp3c15_3950V3.1 locus=Pp3c15_3950 annot-version=v3.3	Physcomitrella_patens	OG0000000
Pp3c13_960V3.1.p pacid=32929858 transcript=Pp3c13_960V3.1

locus=Pp3c13_960 annot-version=v3.3	Physcomitrella_patens
OG0000000
Pp3c13_18000V3.1.p pacid=32929935 transcript=Pp3c13_18000V3.1
locus=Pp3c13_18000 annot-version=v3.3	Physcomitrella_patens
OG0000000
Pp3c13_19360V3.1.p pacid=32930177 transcript=Pp3c13_19360V3.1
locus=Pp3c13_19360 annot-version=v3.3	Physcomitrella_patens
OG0000000
Pp3c13_7450V3.1.p pacid=32930781 transcript=Pp3c13_7450V3.1
locus=Pp3c13_7450 annot-version=v3.3	Physcomitrella_patens
OG0000000
Pp3c10_2950V3.1.p pacid=32902635 transcript=Pp3c10_2950V3.1
locus=Pp3c10_2950 annot-version=v3.3	Physcomitrella_patens
OG0000000
Pp3c13_990V3.1.p pacid=32930929 transcript=Pp3c13_990V3.1
locus=Pp3c13_990 annot-version=v3.3	Physcomitrella_patens
OG0000000
Pp3c13_4780V3.1.p pacid=32931675 transcript=Pp3c13_4780V3.1
locus=Pp3c13_4780 annot-version=v3.3	Physcomitrella_patens
OG0000000
Pp3c10_1880V3.1.p pacid=32902732 transcript=Pp3c10_1880V3.1
locus=Pp3c10_1880 annot-version=v3.3	Physcomitrella_patens
OG0000000
Pp3c10_25470V3.1.p pacid=32902751 transcript=Pp3c10_25470V3.1
locus=Pp3c10_25470 annot-version=v3.3	Physcomitrella_patens
OG0000000
Pp3c13_11270V3.1.p pacid=32931880 transcript=Pp3c13_11270V3.1
locus=Pp3c13_11270 annot-version=v3.3	Physcomitrella_patens
OG0000000
Pp3c13_12310V3.1.p pacid=32932456 transcript=Pp3c13_12310V3.1
locus=Pp3c13_12310 annot-version=v3.3	Physcomitrella_patens
OG0000000
Pp3c22_12040V3.1.p pacid=32902823 transcript=Pp3c22_12040V3.1
locus=Pp3c22_12040 annot-version=v3.3	Physcomitrella_patens
OG0000000
Pp3c2_18630V3.1.p pacid=32932758 transcript=Pp3c2_18630V3.1
locus=Pp3c2_18630 annot-version=v3.3	Physcomitrella_patens
OG0000000
Pp3c2_18520V3.1.p pacid=32932897 transcript=Pp3c2_18520V3.1
locus=Pp3c2_18520 annot-version=v3.3	Physcomitrella_patens
OG0000000
Pp3c2_23960V3.1.p pacid=32933107 transcript=Pp3c2_23960V3.1
locus=Pp3c2_23960 annot-version=v3.3	Physcomitrella_patens
OG0000000
Pp3c2_24490V3.1.p pacid=32933170 transcript=Pp3c2_24490V3.1
locus=Pp3c2_24490 annot-version=v3.3	Physcomitrella_patens
OG0000000
Pp3c2_17640V3.1.p pacid=32933667 transcript=Pp3c2_17640V3.1
locus=Pp3c2_17640 annot-version=v3.3	Physcomitrella_patens
OG0000000
Pp3c2_16610V3.1.p pacid=32934335 transcript=Pp3c2_16610V3.1
locus=Pp3c2_16610 annot-version=v3.3	Physcomitrella_patens
OG0000000
Pp3c2_19850V3.1.p pacid=32934359 transcript=Pp3c2_19850V3.1

locus=Pp3c2_19850 annot-version=v3.3 Physcomitrella_patens
OG0000000
Pp3c2_27480V3.1.p pacid=32934422 transcript=Pp3c2_27480V3.1
locus=Pp3c2_27480 annot-version=v3.3 Physcomitrella_patens
OG0000000
Pp3c2_37290V3.1.p pacid=32934501 transcript=Pp3c2_37290V3.1
locus=Pp3c2_37290 annot-version=v3.3 Physcomitrella_patens
OG0000000
Pp3c22_12090V3.1.p pacid=32903030 transcript=Pp3c22_12090V3.1
locus=Pp3c22_12090 annot-version=v3.3 Physcomitrella_patens
OG0000000
Pp3c2_34580V3.1.p pacid=32935207 transcript=Pp3c2_34580V3.1
locus=Pp3c2_34580 annot-version=v3.3 Physcomitrella_patens
OG0000000
Pp3c2_18900V3.1.p pacid=32935882 transcript=Pp3c2_18900V3.1
locus=Pp3c2_18900 annot-version=v3.3 Physcomitrella_patens
OG0000000
Pp3c2_12320V3.1.p pacid=32935914 transcript=Pp3c2_12320V3.1
locus=Pp3c2_12320 annot-version=v3.3 Physcomitrella_patens
OG0000000
Pp3c2_34620V3.1.p pacid=32936275 transcript=Pp3c2_34620V3.1
locus=Pp3c2_34620 annot-version=v3.3 Physcomitrella_patens
OG0000000
Pp3c2_2220V3.1.p pacid=32936338 transcript=Pp3c2_2220V3.1
locus=Pp3c2_2220 annot-version=v3.3 Physcomitrella_patens
OG0000000
Pp3c2_30340V3.1.p pacid=32936593 transcript=Pp3c2_30340V3.1
locus=Pp3c2_30340 annot-version=v3.3 Physcomitrella_patens
OG0000000
Pp3c2_11910V3.1.p pacid=32937464 transcript=Pp3c2_11910V3.1
locus=Pp3c2_11910 annot-version=v3.3 Physcomitrella_patens
OG0000000
Pp3c2_34890V3.1.p pacid=32937589 transcript=Pp3c2_34890V3.1
locus=Pp3c2_34890 annot-version=v3.3 Physcomitrella_patens
OG0000000
Pp3c19_18380V3.1.p pacid=32938162 transcript=Pp3c19_18380V3.1
locus=Pp3c19_18380 annot-version=v3.3 Physcomitrella_patens
OG0000000
Pp3c19_11710V3.1.p pacid=32938172 transcript=Pp3c19_11710V3.1
locus=Pp3c19_11710 annot-version=v3.3 Physcomitrella_patens
OG0000000
Pp3c19_16570V3.1.p pacid=32938495 transcript=Pp3c19_16570V3.1
locus=Pp3c19_16570 annot-version=v3.3 Physcomitrella_patens
OG0000000
Pp3c19_20890V3.1.p pacid=32938647 transcript=Pp3c19_20890V3.1
locus=Pp3c19_20890 annot-version=v3.3 Physcomitrella_patens
OG0000000
Pp3c10_11000V3.1.p pacid=32899761 transcript=Pp3c10_11000V3.1
locus=Pp3c10_11000 annot-version=v3.3 Physcomitrella_patens
OG0000000
Pp3c19_9160V3.1.p pacid=32939575 transcript=Pp3c19_9160V3.1
locus=Pp3c19_9160 annot-version=v3.3 Physcomitrella_patens
OG0000000
Pp3c19_4760V3.1.p pacid=32939812 transcript=Pp3c19_4760V3.1

locus=Pp3c19_4760 annot-version=v3.3	Physcomitrella_patens
OG0000000
Pp3c3_24690V3.1.p pacid=32940694 transcript=Pp3c3_24690V3.1
locus=Pp3c3_24690 annot-version=v3.3	Physcomitrella_patens
OG0000000
Pp3c22_19310V3.1.p pacid=32903682 transcript=Pp3c22_19310V3.1
locus=Pp3c22_19310 annot-version=v3.3	Physcomitrella_patens
OG0000000
Pp3c22_8640V3.1.p pacid=32903695 transcript=Pp3c22_8640V3.1
locus=Pp3c22_8640 annot-version=v3.3	Physcomitrella_patens
OG0000000
Pp3c3_18160V3.1.p pacid=32941299 transcript=Pp3c3_18160V3.1
locus=Pp3c3_18160 annot-version=v3.3	Physcomitrella_patens
OG0000000
Pp3c3_36760V3.1.p pacid=32941656 transcript=Pp3c3_36760V3.1
locus=Pp3c3_36760 annot-version=v3.3	Physcomitrella_patens
OG0000000
Pp3c3_19490V3.1.p pacid=32942006 transcript=Pp3c3_19490V3.1
locus=Pp3c3_19490 annot-version=v3.3	Physcomitrella_patens
OG0000000
Pp3c3_35290V3.1.p pacid=32942521 transcript=Pp3c3_35290V3.1
locus=Pp3c3_35290 annot-version=v3.3	Physcomitrella_patens
OG0000000
Pp3c22_1220V3.1.p pacid=32903822 transcript=Pp3c22_1220V3.1
locus=Pp3c22_1220 annot-version=v3.3	Physcomitrella_patens
OG0000000
Pp3c3_11260V3.1.p pacid=32942858 transcript=Pp3c3_11260V3.1
locus=Pp3c3_11260 annot-version=v3.3	Physcomitrella_patens
OG0000000
Pp3c3_380V3.1.p pacid=32942958 transcript=Pp3c3_380V3.1
locus=Pp3c3_380 annot-version=v3.3	Physcomitrella_patens
OG0000000
Pp3c3_36680V3.1.p pacid=32942994 transcript=Pp3c3_36680V3.1
locus=Pp3c3_36680 annot-version=v3.3	Physcomitrella_patens
OG0000000
Pp3c3_10220V3.1.p pacid=32943678 transcript=Pp3c3_10220V3.1
locus=Pp3c3_10220 annot-version=v3.3	Physcomitrella_patens
OG0000000
Pp3c3_33210V3.1.p pacid=32943854 transcript=Pp3c3_33210V3.1
locus=Pp3c3_33210 annot-version=v3.3	Physcomitrella_patens
OG0000000
Pp3c3_36775V3.1.p pacid=32943949 transcript=Pp3c3_36775V3.1
locus=Pp3c3_36775 annot-version=v3.3	Physcomitrella_patens
OG0000000
Pp3c3_14110V3.1.p pacid=32944842 transcript=Pp3c3_14110V3.1
locus=Pp3c3_14110 annot-version=v3.3	Physcomitrella_patens
OG0000000
Pp3c3_19080V3.1.p pacid=32945104 transcript=Pp3c3_19080V3.1
locus=Pp3c3_19080 annot-version=v3.3	Physcomitrella_patens
OG0000000
Pp3c20_5180V3.1.p pacid=32945367 transcript=Pp3c20_5180V3.1
locus=Pp3c20_5180 annot-version=v3.3	Physcomitrella_patens
OG0000000
Pp3c20_14660V3.1.p pacid=32946263 transcript=Pp3c20_14660V3.1

locus=Pp3c20_14660 annot-version=v3.3   Physcomitrella_patens
OG0000000
Pp3c20_6870V3.1.p pacid=32946677 transcript=Pp3c20_6870V3.1
locus=Pp3c20_6870 annot-version=v3.3   Physcomitrella_patens
OG0000000
Pp3c22_19230V3.1.p pacid=32904223 transcript=Pp3c22_19230V3.1
locus=Pp3c22_19230 annot-version=v3.3   Physcomitrella_patens
OG0000000
Pp3c20_20260V3.1.p pacid=32946906 transcript=Pp3c20_20260V3.1
locus=Pp3c20_20260 annot-version=v3.3   Physcomitrella_patens
OG0000000
Pp3c20_16970V3.1.p pacid=32946988 transcript=Pp3c20_16970V3.1
locus=Pp3c20_16970 annot-version=v3.3   Physcomitrella_patens
OG0000000
Pp3c20_17290V3.1.p pacid=32947261 transcript=Pp3c20_17290V3.1
locus=Pp3c20_17290 annot-version=v3.3   Physcomitrella_patens
OG0000000
Pp3c20_5120V3.1.p pacid=32948108 transcript=Pp3c20_5120V3.1
locus=Pp3c20_5120 annot-version=v3.3   Physcomitrella_patens
OG0000000
Pp3c22_9330V3.1.p pacid=32904373 transcript=Pp3c22_9330V3.1
locus=Pp3c22_9330 annot-version=v3.3   Physcomitrella_patens
OG0000000
Pp3c23_21020V3.1.p pacid=32949447 transcript=Pp3c23_21020V3.1
locus=Pp3c23_21020 annot-version=v3.3   Physcomitrella_patens
OG0000000
Pp3c23_14220V3.1.p pacid=32949499 transcript=Pp3c23_14220V3.1
locus=Pp3c23_14220 annot-version=v3.3   Physcomitrella_patens
OG0000000
Pp3c23_17090V3.1.p pacid=32949892 transcript=Pp3c23_17090V3.1
locus=Pp3c23_17090 annot-version=v3.3   Physcomitrella_patens
OG0000000
Pp3c23_14970V3.1.p pacid=32950119 transcript=Pp3c23_14970V3.1
locus=Pp3c23_14970 annot-version=v3.3   Physcomitrella_patens
OG0000000
Pp3c23_14050V3.1.p pacid=32950310 transcript=Pp3c23_14050V3.1
locus=Pp3c23_14050 annot-version=v3.3   Physcomitrella_patens
OG0000000
Pp3c22_12350V3.1.p pacid=32904662 transcript=Pp3c22_12350V3.1
locus=Pp3c22_12350 annot-version=v3.3   Physcomitrella_patens
OG0000000
Pp3c23_22360V3.1.p pacid=32951209 transcript=Pp3c23_22360V3.1
locus=Pp3c23_22360 annot-version=v3.3   Physcomitrella_patens
OG0000000
Pp3c27_6910V3.1.p pacid=32951826 transcript=Pp3c27_6910V3.1
locus=Pp3c27_6910 annot-version=v3.3   Physcomitrella_patens
OG0000000
Pp3c27_5920V3.1.p pacid=32951875 transcript=Pp3c27_5920V3.1
locus=Pp3c27_5920 annot-version=v3.3   Physcomitrella_patens
OG0000000
Pp3c27_2280V3.1.p pacid=32952015 transcript=Pp3c27_2280V3.1
locus=Pp3c27_2280 annot-version=v3.3   Physcomitrella_patens
OG0000000
Pp3c27_5080V3.1.p pacid=32952360 transcript=Pp3c27_5080V3.1

locus=Pp3c27_5080 annot-version=v3.3 Physcomitrella_patens
OG0000000
Pp3c5_4350V3.1.p pacid=32952492 transcript=Pp3c5_4350V3.1
locus=Pp3c5_4350 annot-version=v3.3 Physcomitrella_patens
OG0000000
Pp3c5_4140V3.1.p pacid=32953135 transcript=Pp3c5_4140V3.1
locus=Pp3c5_4140 annot-version=v3.3 Physcomitrella_patens
OG0000000
Pp3c5_1950V3.1.p pacid=32953413 transcript=Pp3c5_1950V3.1
locus=Pp3c5_1950 annot-version=v3.3 Physcomitrella_patens
OG0000000
Pp3c5_6960V3.1.p pacid=32953577 transcript=Pp3c5_6960V3.1
locus=Pp3c5_6960 annot-version=v3.3 Physcomitrella_patens
OG0000000
Pp3c5_8240V3.1.p pacid=32954912 transcript=Pp3c5_8240V3.1
locus=Pp3c5_8240 annot-version=v3.3 Physcomitrella_patens
OG0000000
Pp3c5_8160V3.1.p pacid=32955041 transcript=Pp3c5_8160V3.1
locus=Pp3c5_8160 annot-version=v3.3 Physcomitrella_patens
OG0000000
Pp3c5_17270V3.1.p pacid=32955123 transcript=Pp3c5_17270V3.1
locus=Pp3c5_17270 annot-version=v3.3 Physcomitrella_patens
OG0000000
Pp3c5_4120V3.1.p pacid=32955378 transcript=Pp3c5_4120V3.1
locus=Pp3c5_4120 annot-version=v3.3 Physcomitrella_patens
OG0000000
Pp3c5_17880V3.1.p pacid=32955598 transcript=Pp3c5_17880V3.1
locus=Pp3c5_17880 annot-version=v3.3 Physcomitrella_patens
OG0000000
Pp3c5_18040V3.1.p pacid=32956088 transcript=Pp3c5_18040V3.1
locus=Pp3c5_18040 annot-version=v3.3 Physcomitrella_patens
OG0000000
Pp3c11_6870V3.1.p pacid=32956207 transcript=Pp3c11_6870V3.1
locus=Pp3c11_6870 annot-version=v3.3 Physcomitrella_patens
OG0000000
Pp3c11_13350V3.1.p pacid=32956605 transcript=Pp3c11_13350V3.1
locus=Pp3c11_13350 annot-version=v3.3 Physcomitrella_patens
OG0000000
Pp3c11_18470V3.1.p pacid=32956761 transcript=Pp3c11_18470V3.1
locus=Pp3c11_18470 annot-version=v3.3 Physcomitrella_patens
OG0000000
Pp3c11_11500V3.1.p pacid=32957305 transcript=Pp3c11_11500V3.1
locus=Pp3c11_11500 annot-version=v3.3 Physcomitrella_patens
OG0000000
Pp3c11_24230V3.1.p pacid=32957607 transcript=Pp3c11_24230V3.1
locus=Pp3c11_24230 annot-version=v3.3 Physcomitrella_patens
OG0000000
Pp3c11_6700V3.1.p pacid=32957788 transcript=Pp3c11_6700V3.1
locus=Pp3c11_6700 annot-version=v3.3 Physcomitrella_patens
OG0000000
Pp3c11_21790V3.1.p pacid=32958033 transcript=Pp3c11_21790V3.1
locus=Pp3c11_21790 annot-version=v3.3 Physcomitrella_patens
OG0000000
Pp3c11_8850V3.1.p pacid=32958106 transcript=Pp3c11_8850V3.1

locus=Pp3c11_8850 annot-version=v3.3 Physcomitrella_patens
OG0000000
Pp3c11_23980V3.1.p pacid=32958177 transcript=Pp3c11_23980V3.1
locus=Pp3c11_23980 annot-version=v3.3 Physcomitrella_patens
OG0000000
Pp3c11_24020V3.1.p pacid=32958911 transcript=Pp3c11_24020V3.1
locus=Pp3c11_24020 annot-version=v3.3 Physcomitrella_patens
OG0000000
Pp3c11_6330V3.1.p pacid=32959089 transcript=Pp3c11_6330V3.1
locus=Pp3c11_6330 annot-version=v3.3 Physcomitrella_patens
OG0000000
Pp3c11_12320V3.1.p pacid=32959639 transcript=Pp3c11_12320V3.1
locus=Pp3c11_12320 annot-version=v3.3 Physcomitrella_patens
OG0000000
Pp3c14_25010V3.1.p pacid=32959873 transcript=Pp3c14_25010V3.1
locus=Pp3c14_25010 annot-version=v3.3 Physcomitrella_patens
OG0000000
Pp3c14_6980V3.1.p pacid=32960231 transcript=Pp3c14_6980V3.1
locus=Pp3c14_6980 annot-version=v3.3 Physcomitrella_patens
OG0000000
Pp3c14_15710V3.1.p pacid=32960493 transcript=Pp3c14_15710V3.1
locus=Pp3c14_15710 annot-version=v3.3 Physcomitrella_patens
OG0000000
Pp3c14_1610V3.1.p pacid=32960903 transcript=Pp3c14_1610V3.1
locus=Pp3c14_1610 annot-version=v3.3 Physcomitrella_patens
OG0000000
Pp3c14_9730V3.1.p pacid=32960956 transcript=Pp3c14_9730V3.1
locus=Pp3c14_9730 annot-version=v3.3 Physcomitrella_patens
OG0000000
Pp3c14_24840V3.1.p pacid=32961306 transcript=Pp3c14_24840V3.1
locus=Pp3c14_24840 annot-version=v3.3 Physcomitrella_patens
OG0000000
Pp3c14_15220V3.1.p pacid=32961412 transcript=Pp3c14_15220V3.1
locus=Pp3c14_15220 annot-version=v3.3 Physcomitrella_patens
OG0000000
Pp3c14_20510V3.1.p pacid=32961550 transcript=Pp3c14_20510V3.1
locus=Pp3c14_20510 annot-version=v3.3 Physcomitrella_patens
OG0000000
Pp3c14_11590V3.1.p pacid=32961832 transcript=Pp3c14_11590V3.1
locus=Pp3c14_11590 annot-version=v3.3 Physcomitrella_patens
OG0000000
Pp3c14_24890V3.1.p pacid=32961992 transcript=Pp3c14_24890V3.1
locus=Pp3c14_24890 annot-version=v3.3 Physcomitrella_patens
OG0000000
Pp3c14_23270V3.1.p pacid=32962072 transcript=Pp3c14_23270V3.1
locus=Pp3c14_23270 annot-version=v3.3 Physcomitrella_patens
OG0000000
Pp3c14_400V3.1.p pacid=32962288 transcript=Pp3c14_400V3.1
locus=Pp3c14_400 annot-version=v3.3 Physcomitrella_patens
OG0000000
Pp3c14_25240V3.3.p pacid=32962422 transcript=Pp3c14_25240V3.3
locus=Pp3c14_25240 annot-version=v3.3 Physcomitrella_patens
OG0000000
Pp3c14_3550V3.1.p pacid=32962471 transcript=Pp3c14_3550V3.1

locus=Pp3c14_3550 annot-version=v3.3	Physcomitrella_patens
OG0000000
Pp3c14_150V3.1.p pacid=32962534 transcript=Pp3c14_150V3.1
locus=Pp3c14_150 annot-version=v3.3	Physcomitrella_patens
OG0000000
Pp3c14_18150V3.1.p pacid=32962839 transcript=Pp3c14_18150V3.1
locus=Pp3c14_18150 annot-version=v3.3	Physcomitrella_patens
OG0000000
Pp3c14_24970V3.1.p pacid=32963133 transcript=Pp3c14_24970V3.1
locus=Pp3c14_24970 annot-version=v3.3	Physcomitrella_patens
OG0000000
Pp3c8_25270V3.1.p pacid=32963410 transcript=Pp3c8_25270V3.1
locus=Pp3c8_25270 annot-version=v3.3	Physcomitrella_patens
OG0000000
Pp3c8_1070V3.1.p pacid=32963855 transcript=Pp3c8_1070V3.1
locus=Pp3c8_1070 annot-version=v3.3	Physcomitrella_patens
OG0000000
Pp3c8_3290V3.1.p pacid=32963920 transcript=Pp3c8_3290V3.1
locus=Pp3c8_3290 annot-version=v3.3	Physcomitrella_patens
OG0000000
Pp3c8_16440V3.1.p pacid=32964339 transcript=Pp3c8_16440V3.1
locus=Pp3c8_16440 annot-version=v3.3	Physcomitrella_patens
OG0000000
Pp3c8_6120V3.1.p pacid=32965178 transcript=Pp3c8_6120V3.1
locus=Pp3c8_6120 annot-version=v3.3	Physcomitrella_patens
OG0000000
Pp3c8_3330V3.1.p pacid=32965817 transcript=Pp3c8_3330V3.1
locus=Pp3c8_3330 annot-version=v3.3	Physcomitrella_patens
OG0000000
Pp3c8_22340V3.1.p pacid=32965829 transcript=Pp3c8_22340V3.1
locus=Pp3c8_22340 annot-version=v3.3	Physcomitrella_patens
OG0000000
Pp3c1_18280V3.1.p pacid=32966646 transcript=Pp3c1_18280V3.1
locus=Pp3c1_18280 annot-version=v3.3	Physcomitrella_patens
OG0000000
Pp3c1_32540V3.1.p pacid=32966789 transcript=Pp3c1_32540V3.1
locus=Pp3c1_32540 annot-version=v3.3	Physcomitrella_patens
OG0000000
Pp3c1_30900V3.1.p pacid=32966962 transcript=Pp3c1_30900V3.1
locus=Pp3c1_30900 annot-version=v3.3	Physcomitrella_patens
OG0000000
Pp3c1_21050V3.1.p pacid=32967395 transcript=Pp3c1_21050V3.1
locus=Pp3c1_21050 annot-version=v3.3	Physcomitrella_patens
OG0000000
Pp3c1_15730V3.1.p pacid=32967766 transcript=Pp3c1_15730V3.1
locus=Pp3c1_15730 annot-version=v3.3	Physcomitrella_patens
OG0000000
Pp3c1_20580V3.1.p pacid=32967805 transcript=Pp3c1_20580V3.1
locus=Pp3c1_20580 annot-version=v3.3	Physcomitrella_patens
OG0000000
Pp3c1_22880V3.1.p pacid=32968123 transcript=Pp3c1_22880V3.1
locus=Pp3c1_22880 annot-version=v3.3	Physcomitrella_patens
OG0000000
Pp3c1_30940V3.1.p pacid=32968163 transcript=Pp3c1_30940V3.1

locus=Pp3c1_30940 annot-version=v3.3 Physcomitrella_patens
OG0000000
Pp3c1_9320V3.1.p pacid=32968207 transcript=Pp3c1_9320V3.1
locus=Pp3c1_9320 annot-version=v3.3 Physcomitrella_patens
OG0000000
Pp3c1_11040V3.1.p pacid=32968355 transcript=Pp3c1_11040V3.1
locus=Pp3c1_11040 annot-version=v3.3 Physcomitrella_patens
OG0000000
Pp3c17_6060V3.1.p pacid=32906493 transcript=Pp3c17_6060V3.1
locus=Pp3c17_6060 annot-version=v3.3 Physcomitrella_patens
OG0000000
Pp3c1_22100V3.1.p pacid=32969289 transcript=Pp3c1_22100V3.1
locus=Pp3c1_22100 annot-version=v3.3 Physcomitrella_patens
OG0000000
Pp3c1_13200V3.1.p pacid=32969513 transcript=Pp3c1_13200V3.1
locus=Pp3c1_13200 annot-version=v3.3 Physcomitrella_patens
OG0000000
Pp3c1_23040V3.1.p pacid=32969542 transcript=Pp3c1_23040V3.1
locus=Pp3c1_23040 annot-version=v3.3 Physcomitrella_patens
OG0000000
Pp3c1_19980V3.1.p pacid=32969905 transcript=Pp3c1_19980V3.1
locus=Pp3c1_19980 annot-version=v3.3 Physcomitrella_patens
OG0000000
Pp3c1_9180V3.1.p pacid=32970035 transcript=Pp3c1_9180V3.1
locus=Pp3c1_9180 annot-version=v3.3 Physcomitrella_patens
OG0000000
Pp3c1_39210V3.1.p pacid=32970321 transcript=Pp3c1_39210V3.1
locus=Pp3c1_39210 annot-version=v3.3 Physcomitrella_patens
OG0000000
Pp3c1_10810V3.1.p pacid=32970419 transcript=Pp3c1_10810V3.1
locus=Pp3c1_10810 annot-version=v3.3 Physcomitrella_patens
OG0000000
Pp3c1_18390V3.1.p pacid=32970492 transcript=Pp3c1_18390V3.1
locus=Pp3c1_18390 annot-version=v3.3 Physcomitrella_patens
OG0000000
Pp3c1_23020V3.1.p pacid=32971160 transcript=Pp3c1_23020V3.1
locus=Pp3c1_23020 annot-version=v3.3 Physcomitrella_patens
OG0000000
Pp3c1_41480V3.1.p pacid=32971767 transcript=Pp3c1_41480V3.1
locus=Pp3c1_41480 annot-version=v3.3 Physcomitrella_patens
OG0000000
Pp3c1_30920V3.1.p pacid=32972029 transcript=Pp3c1_30920V3.1
locus=Pp3c1_30920 annot-version=v3.3 Physcomitrella_patens
OG0000000
Pp3c1_16810V3.1.p pacid=32972046 transcript=Pp3c1_16810V3.1
locus=Pp3c1_16810 annot-version=v3.3 Physcomitrella_patens
OG0000000
Pp3c12_3550V3.1.p pacid=32972168 transcript=Pp3c12_3550V3.1
locus=Pp3c12_3550 annot-version=v3.3 Physcomitrella_patens
OG0000000
Pp3c12_23490V3.1.p pacid=32972275 transcript=Pp3c12_23490V3.1
locus=Pp3c12_23490 annot-version=v3.3 Physcomitrella_patens
OG0000000
Pp3c12_5800V3.1.p pacid=32972796 transcript=Pp3c12_5800V3.1

locus=Pp3c12_5800 annot-version=v3.3	Physcomitrella_patens
OG0000000
Pp3c12_25650V3.1.p pacid=32972823 transcript=Pp3c12_25650V3.1
locus=Pp3c12_25650 annot-version=v3.3	Physcomitrella_patens
OG0000000
Pp3c12_2140V3.1.p pacid=32972855 transcript=Pp3c12_2140V3.1
locus=Pp3c12_2140 annot-version=v3.3	Physcomitrella_patens
OG0000000
Pp3c12_18960V3.1.p pacid=32972998 transcript=Pp3c12_18960V3.1
locus=Pp3c12_18960 annot-version=v3.3	Physcomitrella_patens
OG0000000
Pp3c12_23570V3.1.p pacid=32973121 transcript=Pp3c12_23570V3.1
locus=Pp3c12_23570 annot-version=v3.3	Physcomitrella_patens
OG0000000
Pp3c12_14020V3.1.p pacid=32973706 transcript=Pp3c12_14020V3.1
locus=Pp3c12_14020 annot-version=v3.3	Physcomitrella_patens
OG0000000
Pp3c12_24120V3.1.p pacid=32974153 transcript=Pp3c12_24120V3.1
locus=Pp3c12_24120 annot-version=v3.3	Physcomitrella_patens
OG0000000
Pp3c12_9140V3.1.p pacid=32974436 transcript=Pp3c12_9140V3.1
locus=Pp3c12_9140 annot-version=v3.3	Physcomitrella_patens
OG0000000
Pp3c12_17460V3.1.p pacid=32974678 transcript=Pp3c12_17460V3.1
locus=Pp3c12_17460 annot-version=v3.3	Physcomitrella_patens
OG0000000
Pp3c12_21860V3.1.p pacid=32975156 transcript=Pp3c12_21860V3.1
locus=Pp3c12_21860 annot-version=v3.3	Physcomitrella_patens
OG0000000
Pp3c6_18190V3.1.p pacid=32975484 transcript=Pp3c6_18190V3.1
locus=Pp3c6_18190 annot-version=v3.3	Physcomitrella_patens
OG0000000
Pp3c6_16200V3.1.p pacid=32975496 transcript=Pp3c6_16200V3.1
locus=Pp3c6_16200 annot-version=v3.3	Physcomitrella_patens
OG0000000
Pp3c17_19290V3.1.p pacid=32907113 transcript=Pp3c17_19290V3.1
locus=Pp3c17_19290 annot-version=v3.3	Physcomitrella_patens
OG0000000
Pp3c6_26620V3.1.p pacid=32975967 transcript=Pp3c6_26620V3.1
locus=Pp3c6_26620 annot-version=v3.3	Physcomitrella_patens
OG0000000
Pp3c6_26540V3.1.p pacid=32976180 transcript=Pp3c6_26540V3.1
locus=Pp3c6_26540 annot-version=v3.3	Physcomitrella_patens
OG0000000
Pp3c6_15470V3.1.p pacid=32976203 transcript=Pp3c6_15470V3.1
locus=Pp3c6_15470 annot-version=v3.3	Physcomitrella_patens
OG0000000
Pp3c17_14370V3.1.p pacid=32907169 transcript=Pp3c17_14370V3.1
locus=Pp3c17_14370 annot-version=v3.3	Physcomitrella_patens
OG0000000
Pp3c6_26930V3.1.p pacid=32976271 transcript=Pp3c6_26930V3.1
locus=Pp3c6_26930 annot-version=v3.3	Physcomitrella_patens
OG0000000
Pp3c6_15790V3.1.p pacid=32976394 transcript=Pp3c6_15790V3.1

locus=Pp3c6_15790 annot-version=v3.3	Physcomitrella_patens
OG0000000
Pp3c6_23170V3.1.p pacid=32976841 transcript=Pp3c6_23170V3.1
locus=Pp3c6_23170 annot-version=v3.3	Physcomitrella_patens
OG0000000
Pp3c6_19200V3.1.p pacid=32977118 transcript=Pp3c6_19200V3.1
locus=Pp3c6_19200 annot-version=v3.3	Physcomitrella_patens
OG0000000
Pp3c6_16360V3.1.p pacid=32977319 transcript=Pp3c6_16360V3.1
locus=Pp3c6_16360 annot-version=v3.3	Physcomitrella_patens
OG0000000
Pp3c6_6060V3.1.p pacid=32978612 transcript=Pp3c6_6060V3.1
locus=Pp3c6_6060 annot-version=v3.3	Physcomitrella_patens
OG0000000
Pp3c25_9800V3.1.p pacid=32979368 transcript=Pp3c25_9800V3.1
locus=Pp3c25_9800 annot-version=v3.3	Physcomitrella_patens
OG0000000
Pp3c25_1655V3.1.p pacid=32979730 transcript=Pp3c25_1655V3.1
locus=Pp3c25_1655 annot-version=v3.3	Physcomitrella_patens
OG0000000
Pp3c25_1650V3.1.p pacid=32980024 transcript=Pp3c25_1650V3.1
locus=Pp3c25_1650 annot-version=v3.3	Physcomitrella_patens
OG0000000
Pp3c25_10250V3.1.p pacid=32980546 transcript=Pp3c25_10250V3.1
locus=Pp3c25_10250 annot-version=v3.3	Physcomitrella_patens
OG0000000
Pp3c25_5290V3.1.p pacid=32980718 transcript=Pp3c25_5290V3.1
locus=Pp3c25_5290 annot-version=v3.3	Physcomitrella_patens
OG0000000
Pp3c18_11100V3.1.p pacid=32981329 transcript=Pp3c18_11100V3.1
locus=Pp3c18_11100 annot-version=v3.3	Physcomitrella_patens
OG0000000
Pp3c17_17580V3.1.p pacid=32907785 transcript=Pp3c17_17580V3.1
locus=Pp3c17_17580 annot-version=v3.3	Physcomitrella_patens
OG0000000
Pp3c18_5120V3.1.p pacid=32982069 transcript=Pp3c18_5120V3.1
locus=Pp3c18_5120 annot-version=v3.3	Physcomitrella_patens
OG0000000
Pp3c18_14390V3.1.p pacid=32982273 transcript=Pp3c18_14390V3.1
locus=Pp3c18_14390 annot-version=v3.3	Physcomitrella_patens
OG0000000
Pp3c18_12630V3.1.p pacid=32983284 transcript=Pp3c18_12630V3.1
locus=Pp3c18_12630 annot-version=v3.3	Physcomitrella_patens
OG0000000
Pp3c16_7870V3.1.p pacid=32983682 transcript=Pp3c16_7870V3.1
locus=Pp3c16_7870 annot-version=v3.3	Physcomitrella_patens
OG0000000
Pp3c16_17510V3.1.p pacid=32984047 transcript=Pp3c16_17510V3.1
locus=Pp3c16_17510 annot-version=v3.3	Physcomitrella_patens
OG0000000
Pp3c16_2720V3.1.p pacid=32984468 transcript=Pp3c16_2720V3.1
locus=Pp3c16_2720 annot-version=v3.3	Physcomitrella_patens
OG0000000
Pp3c16_12990V3.1.p pacid=32984658 transcript=Pp3c16_12990V3.1

locus=Pp3c16_12990 annot-version=v3.3   Physcomitrella_patens
OG0000000
Pp3c17_14330V3.1.p pacid=32908080 transcript=Pp3c17_14330V3.1
locus=Pp3c17_14330 annot-version=v3.3   Physcomitrella_patens
OG0000000
Pp3c10_6460V3.1.p pacid=32900221 transcript=Pp3c10_6460V3.1
locus=Pp3c10_6460 annot-version=v3.3   Physcomitrella_patens
OG0000000
Pp3c16_16080V3.1.p pacid=32984917 transcript=Pp3c16_16080V3.1
locus=Pp3c16_16080 annot-version=v3.3   Physcomitrella_patens
OG0000000
Pp3c16_23380V3.1.p pacid=32985309 transcript=Pp3c16_23380V3.1
locus=Pp3c16_23380 annot-version=v3.3   Physcomitrella_patens
OG0000000
Pp3c16_5720V3.1.p pacid=32985442 transcript=Pp3c16_5720V3.1
locus=Pp3c16_5720 annot-version=v3.3   Physcomitrella_patens
OG0000000
Pp3c16_16440V3.1.p pacid=32986267 transcript=Pp3c16_16440V3.1
locus=Pp3c16_16440 annot-version=v3.3   Physcomitrella_patens
OG0000000
Pp3c16_2580V3.1.p pacid=32986691 transcript=Pp3c16_2580V3.1
locus=Pp3c16_2580 annot-version=v3.3   Physcomitrella_patens
OG0000000
Pp3c24_20760V3.1.p pacid=32908723 transcript=Pp3c24_20760V3.1
locus=Pp3c24_20760 annot-version=v3.3   Physcomitrella_patens
OG0000000
Pp3c24_4410V3.1.p pacid=32909861 transcript=Pp3c24_4410V3.1
locus=Pp3c24_4410 annot-version=v3.3   Physcomitrella_patens
OG0000000
Pp3c24_12080V3.1.p pacid=32909868 transcript=Pp3c24_12080V3.1
locus=Pp3c24_12080 annot-version=v3.3   Physcomitrella_patens
OG0000000
Pp3c9_7920V3.1.p pacid=32911075 transcript=Pp3c9_7920V3.1
locus=Pp3c9_7920 annot-version=v3.3   Physcomitrella_patens
OG0000000
Pp3c10_1370V3.1.p pacid=32900682 transcript=Pp3c10_1370V3.1
locus=Pp3c10_1370 annot-version=v3.3   Physcomitrella_patens
OG0000000
Pp3c10_7650V3.1.p pacid=32900691 transcript=Pp3c10_7650V3.1
locus=Pp3c10_7650 annot-version=v3.3   Physcomitrella_patens
OG0000000
Pp3c9_5240V3.1.p pacid=32912341 transcript=Pp3c9_5240V3.1
locus=Pp3c9_5240 annot-version=v3.3   Physcomitrella_patens
OG0000000
Pp3c9_10730V3.1.p pacid=32913577 transcript=Pp3c9_10730V3.1
locus=Pp3c9_10730 annot-version=v3.3   Physcomitrella_patens
OG0000000
Pp3c9_16610V3.1.p pacid=32913774 transcript=Pp3c9_16610V3.1
locus=Pp3c9_16610 annot-version=v3.3   Physcomitrella_patens
OG0000000
Pp3c9_25280V3.1.p pacid=32913908 transcript=Pp3c9_25280V3.1
locus=Pp3c9_25280 annot-version=v3.3   Physcomitrella_patens
OG0000000
Pp3c9_10640V3.1.p pacid=32914072 transcript=Pp3c9_10640V3.1

locus=Pp3c9_10640 annot-version=v3.3	Physcomitrella_patens
OG0000000
Pp3c21_1520V3.1.p pacid=32914587 transcript=Pp3c21_1520V3.1
locus=Pp3c21_1520 annot-version=v3.3	Physcomitrella_patens
OG0000000
Pp3c10_19910V3.1.p pacid=32900933 transcript=Pp3c10_19910V3.1
locus=Pp3c10_19910 annot-version=v3.3	Physcomitrella_patens
OG0000000
Pp3c21_60V3.1.p pacid=32914923 transcript=Pp3c21_60V3.1
locus=Pp3c21_60 annot-version=v3.3	Physcomitrella_patens
OG0000000
Pp3c21_16810V3.1.p pacid=32915191 transcript=Pp3c21_16810V3.1
locus=Pp3c21_16810 annot-version=v3.3	Physcomitrella_patens
OG0000000
Pp3c21_11870V3.1.p pacid=32915257 transcript=Pp3c21_11870V3.1
locus=Pp3c21_11870 annot-version=v3.3	Physcomitrella_patens
OG0000000
Pp3c10_2340V3.1.p pacid=32899514 transcript=Pp3c10_2340V3.1
locus=Pp3c10_2340 annot-version=v3.3	Physcomitrella_patens
OG0000000
Pp3c21_18220V3.1.p pacid=32915927 transcript=Pp3c21_18220V3.1
locus=Pp3c21_18220 annot-version=v3.3	Physcomitrella_patens
OG0000000
Pp3c21_11170V3.1.p pacid=32915948 transcript=Pp3c21_11170V3.1
locus=Pp3c21_11170 annot-version=v3.3	Physcomitrella_patens
OG0000000
Pp3c26_8100V3.1.p pacid=32916901 transcript=Pp3c26_8100V3.1
locus=Pp3c26_8100 annot-version=v3.3	Physcomitrella_patens
OG0000000
Pp3c26_810V3.1.p pacid=32917017 transcript=Pp3c26_810V3.1
locus=Pp3c26_810 annot-version=v3.3	Physcomitrella_patens
OG0000000
Pp3c26_860V3.1.p pacid=32917421 transcript=Pp3c26_860V3.1
locus=Pp3c26_860 annot-version=v3.3	Physcomitrella_patens
OG0000000
Pp3c26_8120V3.1.p pacid=32917496 transcript=Pp3c26_8120V3.1
locus=Pp3c26_8120 annot-version=v3.3	Physcomitrella_patens
OG0000000
Pp3c26_12890V3.1.p pacid=32917631 transcript=Pp3c26_12890V3.1
locus=Pp3c26_12890 annot-version=v3.3	Physcomitrella_patens
OG0000000
Pp3c26_1050V3.1.p pacid=32917639 transcript=Pp3c26_1050V3.1
locus=Pp3c26_1050 annot-version=v3.3	Physcomitrella_patens
OG0000000
Pp3c26_950V3.1.p pacid=32917764 transcript=Pp3c26_950V3.1
locus=Pp3c26_950 annot-version=v3.3	Physcomitrella_patens
OG0000000
Pp3c26_3790V3.1.p pacid=32918205 transcript=Pp3c26_3790V3.1
locus=Pp3c26_3790 annot-version=v3.3	Physcomitrella_patens
OG0000000
Pp3c26_12800V3.1.p pacid=32918612 transcript=Pp3c26_12800V3.1
locus=Pp3c26_12800 annot-version=v3.3	Physcomitrella_patens
OG0000000
Pp3c4_20780V3.1.p pacid=32919002 transcript=Pp3c4_20780V3.1

locus=Pp3c4_20780 annot-version=v3.3	Physcomitrella_patens
OG0000000
Pp3c4_19720V3.1.p pacid=32919168 transcript=Pp3c4_19720V3.1
locus=Pp3c4_19720 annot-version=v3.3	Physcomitrella_patens
OG0000000
Pp3c4_2450V3.1.p pacid=32919501 transcript=Pp3c4_2450V3.1
locus=Pp3c4_2450 annot-version=v3.3	Physcomitrella_patens
OG0000000
Pp3c4_1480V3.1.p pacid=32920029 transcript=Pp3c4_1480V3.1
locus=Pp3c4_1480 annot-version=v3.3	Physcomitrella_patens
OG0000000
Pp3c4_18780V3.1.p pacid=32920071 transcript=Pp3c4_18780V3.1
locus=Pp3c4_18780 annot-version=v3.3	Physcomitrella_patens
OG0000000
Pp3c4_22080V3.1.p pacid=32920241 transcript=Pp3c4_22080V3.1
locus=Pp3c4_22080 annot-version=v3.3	Physcomitrella_patens
OG0000000
Pp3c4_18510V3.1.p pacid=32920342 transcript=Pp3c4_18510V3.1
locus=Pp3c4_18510 annot-version=v3.3	Physcomitrella_patens
OG0000000
Pp3c4_16210V3.1.p pacid=32920405 transcript=Pp3c4_16210V3.1
locus=Pp3c4_16210 annot-version=v3.3	Physcomitrella_patens
OG0000000
Pp3c4_18960V3.1.p pacid=32920696 transcript=Pp3c4_18960V3.1
locus=Pp3c4_18960 annot-version=v3.3	Physcomitrella_patens
OG0000000
Pp3c4_18720V3.1.p pacid=32920910 transcript=Pp3c4_18720V3.1
locus=Pp3c4_18720 annot-version=v3.3	Physcomitrella_patens
OG0000000
Pp3c4_140V3.1.p pacid=32920968 transcript=Pp3c4_140V3.1
locus=Pp3c4_140 annot-version=v3.3	Physcomitrella_patens
OG0000000
Pp3c10_14420V3.1.p pacid=32901570 transcript=Pp3c10_14420V3.1
locus=Pp3c10_14420 annot-version=v3.3	Physcomitrella_patens
OG0000000
Pp3c4_1620V3.1.p pacid=32921191 transcript=Pp3c4_1620V3.1
locus=Pp3c4_1620 annot-version=v3.3	Physcomitrella_patens
OG0000000
Pp3c10_15500V3.1.p pacid=32901634 transcript=Pp3c10_15500V3.1
locus=Pp3c10_15500 annot-version=v3.3	Physcomitrella_patens
OG0000000
Pp3c4_32350V3.1.p pacid=32921678 transcript=Pp3c4_32350V3.1
locus=Pp3c4_32350 annot-version=v3.3	Physcomitrella_patens
OG0000000
Pp3c4_19890V3.1.p pacid=32921754 transcript=Pp3c4_19890V3.1
locus=Pp3c4_19890 annot-version=v3.3	Physcomitrella_patens
OG0000000
Pp3c4_2350V3.1.p pacid=32922008 transcript=Pp3c4_2350V3.1
locus=Pp3c4_2350 annot-version=v3.3	Physcomitrella_patens
OG0000000
Pp3c4_11670V3.1.p pacid=32922068 transcript=Pp3c4_11670V3.1
locus=Pp3c4_11670 annot-version=v3.3	Physcomitrella_patens
OG0000000
Pp3c10_20260V3.1.p pacid=32901683 transcript=Pp3c10_20260V3.1

locus=Pp3c10_20260 annot-version=v3.3	Physcomitrella_patens
OG0000000
Pp3c4_23140V3.1.p pacid=32922492 transcript=Pp3c4_23140V3.1
locus=Pp3c4_23140 annot-version=v3.3	Physcomitrella_patens
OG0000000
Pp3c4_27350V3.1.p pacid=32922497 transcript=Pp3c4_27350V3.1
locus=Pp3c4_27350 annot-version=v3.3	Physcomitrella_patens
OG0000000
Pp3c4_28230V3.1.p pacid=32922609 transcript=Pp3c4_28230V3.1
locus=Pp3c4_28230 annot-version=v3.3	Physcomitrella_patens
OG0000000
Pp3c4_18360V3.1.p pacid=32922635 transcript=Pp3c4_18360V3.1
locus=Pp3c4_18360 annot-version=v3.3	Physcomitrella_patens
OG0000000
Pp3c4_12460V3.1.p pacid=32922663 transcript=Pp3c4_12460V3.1
locus=Pp3c4_12460 annot-version=v3.3	Physcomitrella_patens
OG0000000
Pp3c7_26460V3.1.p pacid=32923054 transcript=Pp3c7_26460V3.1
locus=Pp3c7_26460 annot-version=v3.3	Physcomitrella_patens
OG0000000
Pp3c7_18140V3.1.p pacid=32923164 transcript=Pp3c7_18140V3.1
locus=Pp3c7_18140 annot-version=v3.3	Physcomitrella_patens
OG0000000
Pp3c7_20780V3.1.p pacid=32923196 transcript=Pp3c7_20780V3.1
locus=Pp3c7_20780 annot-version=v3.3	Physcomitrella_patens
OG0000000
Pp3c7_1290V3.1.p pacid=32923209 transcript=Pp3c7_1290V3.1
locus=Pp3c7_1290 annot-version=v3.3	Physcomitrella_patens
OG0000000
Pp3c7_25560V3.1.p pacid=32923433 transcript=Pp3c7_25560V3.1
locus=Pp3c7_25560 annot-version=v3.3	Physcomitrella_patens
OG0000000
Pp3c7_14800V3.1.p pacid=32923901 transcript=Pp3c7_14800V3.1
locus=Pp3c7_14800 annot-version=v3.3	Physcomitrella_patens
OG0000000
Pp3c7_4070V3.1.p pacid=32924342 transcript=Pp3c7_4070V3.1
locus=Pp3c7_4070 annot-version=v3.3	Physcomitrella_patens
OG0000000
Pp3c7_3140V3.1.p pacid=32924589 transcript=Pp3c7_3140V3.1
locus=Pp3c7_3140 annot-version=v3.3	Physcomitrella_patens
OG0000000
Pp3c7_11260V3.1.p pacid=32924651 transcript=Pp3c7_11260V3.1
locus=Pp3c7_11260 annot-version=v3.3	Physcomitrella_patens
OG0000000
Pp3c7_14130V3.1.p pacid=32925172 transcript=Pp3c7_14130V3.1
locus=Pp3c7_14130 annot-version=v3.3	Physcomitrella_patens
OG0000000
Pp3c7_1440V3.1.p pacid=32925259 transcript=Pp3c7_1440V3.1
locus=Pp3c7_1440 annot-version=v3.3	Physcomitrella_patens
OG0000000
Pp3c7_23600V3.1.p pacid=32925421 transcript=Pp3c7_23600V3.1
locus=Pp3c7_23600 annot-version=v3.3	Physcomitrella_patens
OG0000000
Pp3c10_21660V3.1.p pacid=32902047 transcript=Pp3c10_21660V3.1

locus=Pp3c10_21660 annot-version=v3.3   Physcomitrella_patens
OG0000000
Pp3c10_6800V3.1.p pacid=32902099 transcript=Pp3c10_6800V3.1
locus=Pp3c10_6800 annot-version=v3.3   Physcomitrella_patens
OG0000000
AT2G35050.1 | kinase superfamily with octicosapeptide/Phox/Bem1p
domain-containing protein | Chr2:14769708-14774796 FORWARD
LENGTH=1257 | 201606   Arabidopsis_thaliana   OG0000000
AT2G35620.1 | Leucine-rich repeat protein kinase family protein |
Chr2:14961187-14964640 REVERSE LENGTH=589 | 201606
Arabidopsis_thaliana   OG0000000
AT2G37710.1 | receptor lectin kinase | Chr2:15814934-15816961
REVERSE LENGTH=675 | 201606   Arabidopsis_thaliana
OG0000000
AT1G10620.1 | Protein kinase superfamily protein |
Chr1:3509001-3511975 REVERSE LENGTH=718 | 201606
Arabidopsis_thaliana   OG0000000
AT2G39110.1 | Protein kinase superfamily protein |
Chr2:16319770-16321568 FORWARD LENGTH=435 | 201606
Arabidopsis_thaliana   OG0000000
AT2G39360.1 | Protein kinase superfamily protein |
Chr2:16437592-16440039 REVERSE LENGTH=815 | 201606
Arabidopsis_thaliana   OG0000000
AT2G39660.1 | botrytis-induced kinase1 | Chr2:16531943-16533601
FORWARD LENGTH=395 | 201606   Arabidopsis_thaliana
OG0000000
AT2G41910.1 | Protein kinase superfamily protein |
Chr2:17496956-17498077 FORWARD LENGTH=373 | 201606
Arabidopsis_thaliana   OG0000000
AT2G41970.1 | Protein kinase superfamily protein |
Chr2:17520517-17522304 REVERSE LENGTH=365 | 201606
Arabidopsis_thaliana   OG0000000
AT1G11050.1 | Protein kinase superfamily protein |
Chr1:3681892-3683769 FORWARD LENGTH=625 | 201606
Arabidopsis_thaliana   OG0000000
AT2G42550.1 | Protein kinase superfamily protein |
Chr2:17713196-17714230 FORWARD LENGTH=344 | 201606
Arabidopsis_thaliana   OG0000000
AT2G42640.1 | Mitogen activated protein kinase kinase kinase-like
protein | Chr2:17758532-17763708 REVERSE LENGTH=781 | 201606
Arabidopsis_thaliana   OG0000000
AT2G42960.1 | Protein kinase superfamily protein |
Chr2:17868597-17870630 REVERSE LENGTH=494 | 201606
Arabidopsis_thaliana   OG0000000
AT2G43230.1 | Protein kinase superfamily protein |
Chr2:17966475-17968446 FORWARD LENGTH=406 | 201606
Arabidopsis_thaliana   OG0000000
AT2G43690.1 | Concanavalin A-like lectin protein kinase family
protein | Chr2:18112589-18114583 FORWARD LENGTH=664 | 201606
Arabidopsis_thaliana   OG0000000
AT2G43700.1 | Concanavalin A-like lectin protein kinase family
protein | Chr2:18116523-18118499 FORWARD LENGTH=658 | 201606
Arabidopsis_thaliana   OG0000000
AT1G11280.1 | S-locus lectin protein kinase family protein |

Chr1:3787456-3790728 REVERSE LENGTH=830 | 201606
Arabidopsis_thaliana   OG0000000
AT1G11300.1 | G-type lectin S-receptor-like Serine/Threonine-kinase
| Chr1:3794389-3797346 FORWARD LENGTH=820 | 201606
Arabidopsis_thaliana   OG0000000
AT1G11303.1 | G-type lectin S-receptor-like Serine/Threonine-kinase
| Chr1:3797769-3800719 FORWARD LENGTH=820 | 201606
Arabidopsis_thaliana   OG0000000
AT1G11330.1 | S-locus lectin protein kinase family protein |
Chr1:3810372-3813416 FORWARD LENGTH=840 | 201606
Arabidopsis_thaliana   OG0000000
AT1G11340.1 | G-type lectin S-receptor-like Serine/Threonine-kinase
| Chr1:3814116-3817216 REVERSE LENGTH=833 | 201606
Arabidopsis_thaliana   OG0000000
AT1G11350.1 | S-domain-1 13 | Chr1:3817725-3820752 REVERSE
LENGTH=830 | 201606   Arabidopsis_thaliana   OG0000000
AT1G11410.1 | S-locus lectin protein kinase family protein |
Chr1:3841286-3844284 FORWARD LENGTH=845 | 201606
Arabidopsis_thaliana   OG0000000
AT2G45910.1 | U-box domain-containing protein kinase family protein
| Chr2:18894520-18898212 FORWARD LENGTH=834 | 201606
Arabidopsis_thaliana   OG0000000
AT2G45920.1 | U-box domain-containing protein |
Chr2:18899363-18901097 FORWARD LENGTH=400 | 201606
Arabidopsis_thaliana   OG0000000
AT2G47060.1 | Protein kinase superfamily protein |
Chr2:19333116-19334759 REVERSE LENGTH=365 | 201606
Arabidopsis_thaliana   OG0000000
AT2G48010.1 | receptor-like kinase in in flowers 3 |
Chr2:19641465-19643318 FORWARD LENGTH=617 | 201606
Arabidopsis_thaliana   OG0000000
AT3G01300.1 | Protein kinase superfamily protein | Chr3:90817-93335
REVERSE LENGTH=490 | 201606   Arabidopsis_thaliana
OG0000000
AT3G01490.1 | Protein kinase superfamily protein |
Chr3:191095-193258 REVERSE LENGTH=411 | 201606
Arabidopsis_thaliana   OG0000000
AT3G02810.1 | Protein kinase superfamily protein |
Chr3:608729-610785 REVERSE LENGTH=558 | 201606
Arabidopsis_thaliana   OG0000000
AT3G04690.1 | Malectin/receptor-like protein kinase family protein |
Chr3:1273386-1275938 REVERSE LENGTH=850 | 201606
Arabidopsis_thaliana   OG0000000
AT3G05140.1 | ROP binding protein kinases 2 | Chr3:1435817-1437800
REVERSE LENGTH=460 | 201606   Arabidopsis_thaliana
OG0000000
AT3G05990.1 | Leucine-rich repeat (LRR) family protein |
Chr3:1797116-1799732 REVERSE LENGTH=517 | 201606
Arabidopsis_thaliana   OG0000000
AT3G06620.1 | PAS domain-containing protein tyrosine kinase family
protein | Chr3:2062833-2067138 REVERSE LENGTH=773 | 201606
Arabidopsis_thaliana   OG0000000
AT3G06630.1 | protein kinase family protein | Chr3:2070388-2073791
REVERSE LENGTH=671 | 201606   Arabidopsis_thaliana

OG0000000
AT3G06640.1 | PAS domain-containing protein tyrosine kinase family protein | Chr3:2074491-2078317 REVERSE LENGTH=730 | 201606 Arabidopsis_thaliana OG0000000
AT3G07070.1 | Protein kinase superfamily protein | Chr3:2238455-2240074 FORWARD LENGTH=414 | 201606 Arabidopsis_thaliana OG0000000
AT3G08870.1 | Concanavalin A-like lectin protein kinase family protein | Chr3:2700500-2702581 REVERSE LENGTH=693 | 201606 Arabidopsis_thaliana OG0000000
AT3G09010.1 | Protein kinase superfamily protein | Chr3:2750285-2752086 FORWARD LENGTH=393 | 201606 Arabidopsis_thaliana OG0000000
AT3G09190.1 | Concanavalin A-like lectin family protein | Chr3:2821650-2822687 REVERSE LENGTH=345 | 201606 Arabidopsis_thaliana OG0000000
AT3G09780.1 | CRINKLY4 related 1 | Chr3:3000838-3003165 REVERSE LENGTH=775 | 201606 Arabidopsis_thaliana OG0000000
AT3G09830.1 | Protein kinase superfamily protein | Chr3:3017199-3018696 FORWARD LENGTH=418 | 201606 Arabidopsis_thaliana OG0000000
AT3G13690.1 | kinase with adenine nucleotide alpha hydrolases-like domain-containing protein | Chr3:4486920-4490011 FORWARD LENGTH=753 | 201606 Arabidopsis_thaliana OG0000000
AT3G14350.1 | STRUBBELIG-receptor family 7 | Chr3:4783115-4786999 REVERSE LENGTH=717 | 201606 Arabidopsis_thaliana OG0000000
AT3G15356.1 | Legume lectin family protein | Chr3:5174603-5175418 REVERSE LENGTH=271 | 201606 Arabidopsis_thaliana OG0000000
AT3G15890.1 | Protein kinase superfamily protein | Chr3:5374389-5376114 FORWARD LENGTH=361 | 201606 Arabidopsis_thaliana OG0000000
AT3G16030.1 | lectin protein kinase family protein | Chr3:5439609-5442802 FORWARD LENGTH=850 | 201606 Arabidopsis_thaliana OG0000000
AT3G16530.1 | Legume lectin family protein | Chr3:5624586-5625416 REVERSE LENGTH=276 | 201606 Arabidopsis_thaliana OG0000000
AT3G17410.1 | Protein kinase superfamily protein | Chr3:5956601-5958882 FORWARD LENGTH=364 | 201606 Arabidopsis_thaliana OG0000000
AT3G17420.1 | glyoxysomal protein kinase 1 | Chr3:5959462-5961313 REVERSE LENGTH=467 | 201606 Arabidopsis_thaliana OG0000000
AT3G18810.1 | Protein kinase superfamily protein | Chr3:6480701-6483593 REVERSE LENGTH=700 | 201606 Arabidopsis_thaliana OG0000000
AT3G19300.1 | Protein kinase superfamily protein | Chr3:6690242-6693210 REVERSE LENGTH=663 | 201606 Arabidopsis_thaliana OG0000000
AT3G20200.1 | kinase with adenine nucleotide alpha hydrolases-like domain-containing protein | Chr3:7047895-7051145 FORWARD LENGTH=780 | 201606 Arabidopsis_thaliana OG0000000

AT3G20530.1 | Protein kinase superfamily protein |
Chr3:7166318-7167806 FORWARD LENGTH=386 | 201606
Arabidopsis_thaliana OG0000000
AT3G21340.1 | Leucine-rich repeat protein kinase family protein |
Chr3:7511848-7515937 REVERSE LENGTH=899 | 201606
Arabidopsis_thaliana OG0000000
AT3G21630.1 | chitin elicitor receptor kinase 1 |
Chr3:7615543-7618530 REVERSE LENGTH=617 | 201606
Arabidopsis_thaliana OG0000000
AT3G22750.1 | Protein kinase superfamily protein |
Chr3:8037364-8039096 REVERSE LENGTH=378 | 201606
Arabidopsis_thaliana OG0000000
AT3G23750.1 | Leucine-rich repeat protein kinase family protein |
Chr3:8558332-8561263 FORWARD LENGTH=928 | 201606
Arabidopsis_thaliana OG0000000
AT3G24540.1 | Protein kinase superfamily protein |
Chr3:8952903-8955398 FORWARD LENGTH=554 | 201606
Arabidopsis_thaliana OG0000000
AT3G24550.1 | proline extensin-like receptor kinase 1 |
Chr3:8960411-8963303 FORWARD LENGTH=652 | 201606
Arabidopsis_thaliana OG0000000
AT3G24715.1 | kinase superfamily with octicosapeptide/Phox/Bem1p
domain-containing protein | Chr3:9025849-9029948 FORWARD LENGTH=1117
| 201606 Arabidopsis_thaliana OG0000000
AT3G24790.1 | Protein kinase superfamily protein |
Chr3:9052996-9054531 FORWARD LENGTH=363 | 201606
Arabidopsis_thaliana OG0000000
AT3G25490.1 | Protein kinase family protein | Chr3:9241725-9243113
FORWARD LENGTH=433 | 201606 Arabidopsis_thaliana
OG0000000
AT3G25560.1 | NSP-interacting kinase 2 | Chr3:9279682-9282560
REVERSE LENGTH=635 | 201606 Arabidopsis_thaliana
OG0000000
AT3G26940.1 | Protein kinase superfamily protein |
Chr3:9936707-9938936 REVERSE LENGTH=432 | 201606
Arabidopsis_thaliana OG0000000
AT3G27560.1 | Protein kinase superfamily protein |
Chr3:10210597-10212507 REVERSE LENGTH=356 | 201606
Arabidopsis_thaliana OG0000000
AT1G14370.1 | protein kinase 2A | Chr1:4915859-4917959 FORWARD
LENGTH=426 | 201606 Arabidopsis_thaliana OG0000000
AT3G28690.1 | Protein kinase superfamily protein |
Chr3:10756002-10757494 FORWARD LENGTH=376 | 201606
Arabidopsis_thaliana OG0000000
AT3G45330.1 | Concanavalin A-like lectin protein kinase family
protein | Chr3:16632440-16634488 REVERSE LENGTH=682 | 201606
Arabidopsis_thaliana OG0000000
AT3G45390.1 | LOW protein Arabidopsis_thaliana OG0000000
AT3G45410.1 | Concanavalin A-like lectin protein kinase family
protein | Chr3:16654019-16656013 REVERSE LENGTH=664 | 201606
Arabidopsis_thaliana OG0000000
AT3G45420.1 | Concanavalin A-like lectin protein kinase family
protein | Chr3:16657263-16659266 REVERSE LENGTH=667 | 201606
Arabidopsis_thaliana OG0000000

AT3G45430.1 | Concanavalin A-like lectin protein kinase family protein | Chr3:16660759-16662783 REVERSE LENGTH=674 | 201606 Arabidopsis_thaliana OG0000000
AT3G45440.1 | Concanavalin A-like lectin protein kinase family protein | Chr3:16664875-16666884 REVERSE LENGTH=669 | 201606 Arabidopsis_thaliana OG0000000
AT3G45670.1 | Protein kinase superfamily protein | Chr3:16765320-16766459 FORWARD LENGTH=379 | 201606 Arabidopsis_thaliana OG0000000
AT3G45790.1 | Protein kinase superfamily protein | Chr3:16825005-16826222 REVERSE LENGTH=376 | 201606 Arabidopsis_thaliana OG0000000
AT3G45860.1 | cysteine-rich RLK (RECEPTOR-like protein kinase) 4 | Chr3:16863401-16866041 REVERSE LENGTH=676 | 201606 Arabidopsis_thaliana OG0000000
AT3G45920.1 | Protein kinase superfamily protein | Chr3:16882182-16882871 FORWARD LENGTH=192 | 201606 Arabidopsis_thaliana OG0000000
AT3G46140.1 | Protein kinase superfamily protein | Chr3:16948090-16949220 FORWARD LENGTH=376 | 201606 Arabidopsis_thaliana OG0000000
AT3G46160.1 | Protein kinase superfamily protein | Chr3:16950955-16952136 FORWARD LENGTH=393 | 201606 Arabidopsis_thaliana OG0000000
AT3G46290.1 | hercules receptor kinase 1 | Chr3:17013009-17015501 FORWARD LENGTH=830 | 201606 Arabidopsis_thaliana OG0000000
AT3G46330.1 | Leucine-rich repeat protein kinase family protein | Chr3:17020887-17024884 REVERSE LENGTH=878 | 201606 Arabidopsis_thaliana OG0000000
AT3G46340.1 | Leucine-rich repeat protein kinase family protein | Chr3:17026658-17031842 FORWARD LENGTH=889 | 201606 Arabidopsis_thaliana OG0000000
AT3G46350.1 | LRR receptor-like Serine/Threonine-kinase | Chr3:17036427-17039547 FORWARD LENGTH=444 | 201606 Arabidopsis_thaliana OG0000000
AT3G46355.1 | LRR receptor-like Serine/Threonine-kinase | Chr3:17039609-17041680 FORWARD LENGTH=291 | 201606 Arabidopsis_thaliana OG0000000
AT3G46370.1 | Leucine-rich repeat protein kinase family protein | Chr3:17051955-17055514 FORWARD LENGTH=793 | 201606 Arabidopsis_thaliana OG0000000
AT3G46400.1 | Leucine-rich repeat protein kinase family protein | Chr3:17073196-17077328 FORWARD LENGTH=883 | 201606 Arabidopsis_thaliana OG0000000
AT3G46410.1 | Protein kinase superfamily protein | Chr3:17079093-17080684 FORWARD LENGTH=291 | 201606 Arabidopsis_thaliana OG0000000
AT3G46420.1 | Leucine-rich repeat protein kinase family protein | Chr3:17082108-17086534 FORWARD LENGTH=838 | 201606 Arabidopsis_thaliana OG0000000
AT3G46760.1 | Protein kinase superfamily protein | Chr3:17222027-17223040 FORWARD LENGTH=337 | 201606 Arabidopsis_thaliana OG0000000

AT3G46920.1 | kinase superfamily with octicosapeptide/Phox/Bem1p domain-containing protein | Chr3:17280430-17284659 REVERSE LENGTH=1089 | 201606 Arabidopsis_thaliana OG0000000
AT3G46930.1 | Protein kinase superfamily protein | Chr3:17285931-17288032 FORWARD LENGTH=480 | 201606 Arabidopsis_thaliana OG0000000
AT3G49060.1 | U-box domain-containing protein kinase family protein | Chr3:18187386-18191878 REVERSE LENGTH=805 | 201606 Arabidopsis_thaliana OG0000000
AT3G50720.1 | Protein kinase superfamily protein | Chr3:18847519-18849430 REVERSE LENGTH=377 | 201606 Arabidopsis_thaliana OG0000000
AT3G50730.1 | Protein kinase superfamily protein | Chr3:18851533-18853137 REVERSE LENGTH=371 | 201606 Arabidopsis_thaliana OG0000000
AT3G51550.1 | Malectin/receptor-like protein kinase family protein | Chr3:19117877-19120564 REVERSE LENGTH=895 | 201606 Arabidopsis_thaliana OG0000000
AT1G15530.1 | Concanavalin A-like lectin protein kinase family protein | Chr1:5339961-5341931 REVERSE LENGTH=656 | 201606 Arabidopsis_thaliana OG0000000
AT3G51990.1 | Protein kinase superfamily protein | Chr3:19287989-19289077 FORWARD LENGTH=362 | 201606 Arabidopsis_thaliana OG0000000
AT3G53380.1 | Concanavalin A-like lectin protein kinase family protein | Chr3:19789204-19791351 REVERSE LENGTH=715 | 201606 Arabidopsis_thaliana OG0000000
AT3G53810.1 | Concanavalin A-like lectin protein kinase family protein | Chr3:19933153-19935186 REVERSE LENGTH=677 | 201606 Arabidopsis_thaliana OG0000000
AT3G53840.1 | Protein kinase superfamily protein | Chr3:19945571-19947719 FORWARD LENGTH=639 | 201606 Arabidopsis_thaliana OG0000000
AT3G55450.1 | PBS1-like 1 | Chr3:20558129-20559963 FORWARD LENGTH=389 | 201606 Arabidopsis_thaliana OG0000000
AT3G55550.1 | Concanavalin A-like lectin protein kinase family protein | Chr3:20600019-20602073 REVERSE LENGTH=684 | 201606 Arabidopsis_thaliana OG0000000
AT3G55950.1 | CRINKLY4 related 3 | Chr3:20753903-20756347 REVERSE LENGTH=814 | 201606 Arabidopsis_thaliana OG0000000
AT1G16110.1 | wall associated kinase-like 6 | Chr1:5518381-5520470 FORWARD LENGTH=642 | 201606 Arabidopsis_thaliana OG0000000
AT1G16120.1 | wall associated kinase-like 1 | Chr1:5522639-5524983 FORWARD LENGTH=730 | 201606 Arabidopsis_thaliana OG0000000
AT1G16130.1 | wall associated kinase-like 2 | Chr1:5525634-5528047 FORWARD LENGTH=748 | 201606 Arabidopsis_thaliana OG0000000
AT1G16150.1 | wall associated kinase-like 4 | Chr1:5532415-5534877 FORWARD LENGTH=761 | 201606 Arabidopsis_thaliana OG0000000
AT1G16160.1 | wall associated kinase-like 5 | Chr1:5535913-5538269 FORWARD LENGTH=731 | 201606 Arabidopsis_thaliana

OG0000000
AT3G58640.1 | Mitogen activated protein kinase kinase kinase-like protein | Chr3:21687153-21692675 REVERSE LENGTH=809 | 201606 Arabidopsis_thaliana OG0000000
AT3G58690.1 | Protein kinase superfamily protein | Chr3:21709369-21711246 FORWARD LENGTH=400 | 201606 Arabidopsis_thaliana OG0000000
AT1G16260.1 | Wall-associated kinase family protein | Chr1:5559708-5562018 REVERSE LENGTH=720 | 201606 Arabidopsis_thaliana OG0000000
AT1G16270.1 | kinase superfamily with octicosapeptide/Phox/Bem1p domain-containing protein | Chr1:5563890-5568145 FORWARD LENGTH=1147 | 201606 Arabidopsis_thaliana OG0000000
AT3G59110.1 | Protein kinase superfamily protein | Chr3:21855673-21857847 FORWARD LENGTH=512 | 201606 Arabidopsis_thaliana OG0000000
AT3G59350.1 | Protein kinase superfamily protein | Chr3:21932930-21934883 FORWARD LENGTH=408 | 201606 Arabidopsis_thaliana OG0000000
AT3G59700.1 | lectin-receptor kinase | Chr3:22052146-22054131 FORWARD LENGTH=661 | 201606 Arabidopsis_thaliana
OG0000000
AT3G59730.1 | Concanavalin A-like lectin protein kinase family protein | Chr3:22064308-22065879 REVERSE LENGTH=523 | 201606 Arabidopsis_thaliana OG0000000
AT3G59740.1 | Concanavalin A-like lectin protein kinase family protein | Chr3:22067079-22069058 REVERSE LENGTH=659 | 201606 Arabidopsis_thaliana OG0000000
AT3G59750.1 | Concanavalin A-like lectin protein kinase family protein | Chr3:22069855-22071821 REVERSE LENGTH=626 | 201606 Arabidopsis_thaliana OG0000000
AT3G61390.1 | RING/U-box superfamily protein | Chr3:22716418-22717989 FORWARD LENGTH=373 | 201606 Arabidopsis_thaliana OG0000000
AT3G61410.1 | U-box kinase family protein | Chr3:22721112-22722452 FORWARD LENGTH=294 | 201606 Arabidopsis_thaliana
OG0000000
AT3G62220.1 | Protein kinase superfamily protein | Chr3:23029276-23030864 REVERSE LENGTH=361 | 201606 Arabidopsis_thaliana OG0000000
AT1G16670.1 | Protein kinase superfamily protein | Chr1:5697846-5699492 FORWARD LENGTH=390 | 201606 Arabidopsis_thaliana OG0000000
AT3G63260.1 | Protein kinase superfamily protein | Chr3:23373090-23374747 REVERSE LENGTH=391 | 201606 Arabidopsis_thaliana OG0000000
AT1G16760.1 | kinase with adenine nucleotide alpha hydrolases-like domain-containing protein | Chr1:5734234-5737307 FORWARD LENGTH=758 | 201606 Arabidopsis_thaliana OG0000000
AT4G00330.1 | calmodulin-binding receptor-like cytoplasmic kinase 2 | Chr4:142787-144427 REVERSE LENGTH=411 | 201606 Arabidopsis_thaliana OG0000000
AT4G00340.1 | receptor-like protein kinase 4 | Chr4:148958-151496 FORWARD LENGTH=818 | 201606 Arabidopsis_thaliana

OG0000000
AT4G00960.1 | Protein kinase superfamily protein |
Chr4:414361-416180 FORWARD LENGTH=379 | 201606
Arabidopsis_thaliana OG0000000
AT4G00970.1 | cysteine-rich RLK (RECEPTOR-like protein kinase) 41 |
Chr4:418437-421694 FORWARD LENGTH=665 | 201606
Arabidopsis_thaliana OG0000000
AT4G01330.1 | Protein kinase superfamily protein |
Chr4:550723-552847 FORWARD LENGTH=479 | 201606
Arabidopsis_thaliana OG0000000
AT4G02010.1 | Protein kinase superfamily protein |
Chr4:881457-885222 FORWARD LENGTH=725 | 201606
Arabidopsis_thaliana OG0000000
AT4G02410.1 | Concanavalin A-like lectin protein kinase family
protein | Chr4:1060086-1062110 REVERSE LENGTH=674 | 201606
Arabidopsis_thaliana OG0000000
AT4G02420.1 | Concanavalin A-like lectin protein kinase family
protein | Chr4:1064363-1066372 REVERSE LENGTH=669 | 201606
Arabidopsis_thaliana OG0000000
AT4G02630.1 | Protein kinase superfamily protein |
Chr4:1151683-1153161 FORWARD LENGTH=492 | 201606
Arabidopsis_thaliana OG0000000
AT4G03230.1 | G-type lectin S-receptor-like Serine/Threonine-kinase
| Chr4:1419278-1422828 REVERSE LENGTH=1010 | 201606
Arabidopsis_thaliana OG0000000
AT4G03390.1 | STRUBBELIG-receptor family 3 | Chr4:1490912-1494553
REVERSE LENGTH=776 | 201606 Arabidopsis_thaliana
OG0000000
AT4G04490.1 | cysteine-rich RLK (RECEPTOR-like protein kinase) 36 |
Chr4:2231957-2234638 REVERSE LENGTH=658 | 201606
Arabidopsis_thaliana OG0000000
AT4G04500.1 | cysteine-rich RLK (RECEPTOR-like protein kinase) 37 |
Chr4:2238411-2240767 FORWARD LENGTH=646 | 201606
Arabidopsis_thaliana OG0000000
AT4G04510.1 | cysteine-rich RLK (RECEPTOR-like protein kinase) 38 |
Chr4:2242122-2244656 FORWARD LENGTH=648 | 201606
Arabidopsis_thaliana OG0000000
AT4G04540.1 | cysteine-rich RLK (RECEPTOR-like protein kinase) 39 |
Chr4:2259580-2262138 FORWARD LENGTH=659 | 201606
Arabidopsis_thaliana OG0000000
AT4G04570.1 | cysteine-rich RLK (RECEPTOR-like protein kinase) 40 |
Chr4:2290045-2292717 FORWARD LENGTH=654 | 201606
Arabidopsis_thaliana OG0000000
AT4G04960.1 | Concanavalin A-like lectin protein kinase family
protein | Chr4:2533096-2535156 FORWARD LENGTH=686 | 201606
Arabidopsis_thaliana OG0000000
AT4G05200.1 | cysteine-rich RLK (RECEPTOR-like protein kinase) 25 |
Chr4:2679793-2682309 REVERSE LENGTH=675 | 201606
Arabidopsis_thaliana OG0000000
AT1G17540.1 | kinase with adenine nucleotide alpha hydrolases-like
domain-containing protein | Chr1:6029551-6032641 REVERSE LENGTH=728
| 201606 Arabidopsis_thaliana OG0000000
AT4G11460.1 | cysteine-rich RLK (RECEPTOR-like protein kinase) 30 |
Chr4:6964468-6967093 FORWARD LENGTH=700 | 201606

Arabidopsis_thaliana   OG0000000
AT4G11470.1 | cysteine-rich RLK (RECEPTOR-like protein kinase) 31 | Chr4:6967729-6970161 FORWARD LENGTH=666 | 201606
Arabidopsis_thaliana   OG0000000
AT4G11480.1 | cysteine-rich RLK (RECEPTOR-like protein kinase) 32 | Chr4:6971408-6973799 FORWARD LENGTH=656 | 201606
Arabidopsis_thaliana   OG0000000
AT4G11490.1 | cysteine-rich RLK (RECEPTOR-like protein kinase) 33 | Chr4:6978848-6981548 FORWARD LENGTH=636 | 201606
Arabidopsis_thaliana   OG0000000
AT4G11530.1 | cysteine-rich RLK (RECEPTOR-like protein kinase) 34 | Chr4:6987093-6989599 FORWARD LENGTH=676 | 201606
Arabidopsis_thaliana   OG0000000
AT4G11890.1 | Protein kinase superfamily protein | Chr4:7148269-7149772 FORWARD LENGTH=351 | 201606
Arabidopsis_thaliana   OG0000000
AT4G11900.1 | S-locus lectin protein kinase family protein | Chr4:7150241-7153542 REVERSE LENGTH=849 | 201606
Arabidopsis_thaliana   OG0000000
AT4G13190.1 | Protein kinase superfamily protein | Chr4:7659435-7661106 REVERSE LENGTH=389 | 201606
Arabidopsis_thaliana   OG0000000
AT1G17910.1 | Wall-associated kinase family protein | Chr1:6159126-6161615 FORWARD LENGTH=764 | 201606
Arabidopsis_thaliana   OG0000000
AT4G14780.1 | Protein kinase superfamily protein | Chr4:8492989-8494480 FORWARD LENGTH=364 | 201606
Arabidopsis_thaliana   OG0000000
AT1G18160.1 | Protein kinase superfamily protein | Chr1:6249126-6253835 FORWARD LENGTH=992 | 201606
Arabidopsis_thaliana   OG0000000
AT1G18390.1 | Serine/Threonine kinase family catalytic domain protein | Chr1:6325876-6329935 FORWARD LENGTH=648 | 201606
Arabidopsis_thaliana   OG0000000
AT4G17660.1 | Protein kinase superfamily protein | Chr4:9831401-9833006 FORWARD LENGTH=388 | 201606
Arabidopsis_thaliana   OG0000000
AT4G20450.1 | Leucine-rich repeat protein kinase family protein | Chr4:11024054-11029008 REVERSE LENGTH=898 | 201606
Arabidopsis_thaliana   OG0000000
AT4G21230.1 | cysteine-rich RLK (RECEPTOR-like protein kinase) 27 | Chr4:11319244-11321679 REVERSE LENGTH=642 | 201606
Arabidopsis_thaliana   OG0000000
AT4G21380.1 | receptor kinase 3 | Chr4:11389219-11393090 REVERSE LENGTH=850 | 201606   Arabidopsis_thaliana   OG0000000
AT4G21390.1 | S-locus lectin protein kinase family protein | Chr4:11394458-11397474 REVERSE LENGTH=849 | 201606
Arabidopsis_thaliana   OG0000000
AT4G21400.1 | cysteine-rich RLK (RECEPTOR-like protein kinase) 28 | Chr4:11399218-11401709 REVERSE LENGTH=683 | 201606
Arabidopsis_thaliana   OG0000000
AT4G21410.1 | cysteine-rich RLK (RECEPTOR-like protein kinase) 29 | Chr4:11402463-11404650 REVERSE LENGTH=554 | 201606
Arabidopsis_thaliana   OG0000000

AT4G22130.1 | STRUBBELIG-receptor family 8 | Chr4:11723733-11727331 FORWARD LENGTH=703 | 201606	Arabidopsis_thaliana	OG0000000
AT4G23050.1 | PAS domain-containing protein tyrosine kinase family protein | Chr4:12080112-12083708 FORWARD LENGTH=735 | 201606	Arabidopsis_thaliana	OG0000000
AT4G23130.1 | cysteine-rich RLK (RECEPTOR-like protein kinase) 5 | Chr4:12117688-12120134 REVERSE LENGTH=659 | 201606	Arabidopsis_thaliana	OG0000000
AT4G23140.1 | cysteine-rich RLK (RECEPTOR-like protein kinase) 6 | Chr4:12121397-12124037 FORWARD LENGTH=674 | 201606	Arabidopsis_thaliana	OG0000000
AT4G23150.1 | cysteine-rich RLK (RECEPTOR-like protein kinase) 7 | Chr4:12125731-12128301 FORWARD LENGTH=659 | 201606	Arabidopsis_thaliana	OG0000000
AT4G23160.1 | cysteine-rich RECEPTOR-like kinase | Chr4:12131325-12134086 FORWARD LENGTH=682 | 201606	Arabidopsis_thaliana	OG0000000
AT4G23180.1 | cysteine-rich RLK (RECEPTOR-like protein kinase) 10 | Chr4:12138171-12140780 FORWARD LENGTH=669 | 201606	Arabidopsis_thaliana	OG0000000
AT4G23190.1 | cysteine-rich RLK (RECEPTOR-like protein kinase) 11 | Chr4:12141197-12143710 REVERSE LENGTH=667 | 201606	Arabidopsis_thaliana	OG0000000
AT4G23200.1 | cysteine-rich RLK (RECEPTOR-like protein kinase) 12 | Chr4:12145380-12147934 REVERSE LENGTH=659 | 201606	Arabidopsis_thaliana	OG0000000
AT4G23210.1 | cysteine-rich RLK (RECEPTOR-like protein kinase) 13 | Chr4:12149154-12151418 REVERSE LENGTH=610 | 201606	Arabidopsis_thaliana	OG0000000
AT4G23220.1 | cysteine-rich RECEPTOR-like kinase | Chr4:12154091-12156881 REVERSE LENGTH=658 | 201606	Arabidopsis_thaliana	OG0000000
AT4G23230.1 | cysteine-rich RECEPTOR-like kinase | Chr4:12157827-12160279 REVERSE LENGTH=627 | 201606	Arabidopsis_thaliana	OG0000000
AT4G23240.1 | cysteine-rich RLK (RECEPTOR-like protein kinase) 16 | Chr4:12160502-12163193 REVERSE LENGTH=662 | 201606	Arabidopsis_thaliana	OG0000000
AT4G23250.1 | cysteine-rich receptor-like protein kinase 17 | Chr4:12164451-12167026 REVERSE LENGTH=686 | 201606	Arabidopsis_thaliana	OG0000000
AT4G23260.1 | cysteine-rich RLK (RECEPTOR-like protein kinase) 18 | Chr4:12167528-12170055 REVERSE LENGTH=659 | 201606	Arabidopsis_thaliana	OG0000000
AT4G23270.1 | cysteine-rich RLK (RECEPTOR-like protein kinase) 19 | Chr4:12171133-12173794 FORWARD LENGTH=645 | 201606	Arabidopsis_thaliana	OG0000000
AT4G23280.1 | cysteine-rich RLK (RECEPTOR-like protein kinase) 20 | Chr4:12174740-12177471 FORWARD LENGTH=666 | 201606	Arabidopsis_thaliana	OG0000000
AT4G23290.1 | cysteine-rich RLK (RECEPTOR-like protein kinase) 21 | Chr4:12177910-12180810 REVERSE LENGTH=647 | 201606	Arabidopsis_thaliana	OG0000000

AT4G23300.1 | cysteine-rich RLK (RECEPTOR-like protein kinase) 22 | Chr4:12182002-12184531 FORWARD LENGTH=660 | 201606 Arabidopsis_thaliana OG0000000
AT4G23310.1 | cysteine-rich RLK (RECEPTOR-like protein kinase) 23 | Chr4:12185737-12188763 FORWARD LENGTH=830 | 201606 Arabidopsis_thaliana OG0000000
AT4G23320.1 | cysteine-rich RLK (RECEPTOR-like protein kinase) 24 | Chr4:12189182-12191290 REVERSE LENGTH=507 | 201606 Arabidopsis_thaliana OG0000000
AT1G19090.1 | receptor-like serine/threonine kinase 2 | Chr1:6590350-6592615 FORWARD LENGTH=615 | 201606 Arabidopsis_thaliana OG0000000
AT4G24480.1 | Protein kinase superfamily protein | Chr4:12650410-12654755 FORWARD LENGTH=956 | 201606 Arabidopsis_thaliana OG0000000
AT4G25160.1 | U-box domain-containing protein kinase family protein | Chr4:12903360-12906669 REVERSE LENGTH=835 | 201606 Arabidopsis_thaliana OG0000000
AT1G19390.1 | Wall-associated kinase family protein | Chr1:6700772-6703368 REVERSE LENGTH=788 | 201606 Arabidopsis_thaliana OG0000000
AT4G27290.1 | S-locus lectin protein kinase family protein | Chr4:13666281-13669202 FORWARD LENGTH=783 | 201606 Arabidopsis_thaliana OG0000000
AT4G27300.1 | S-locus lectin protein kinase family protein | Chr4:13669308-13672348 REVERSE LENGTH=815 | 201606 Arabidopsis_thaliana OG0000000
AT4G28350.1 | Concanavalin A-like lectin protein kinase family protein | Chr4:14026577-14028622 FORWARD LENGTH=681 | 201606 Arabidopsis_thaliana OG0000000
AT4G28670.1 | cysteine-rich RECEPTOR-like kinase%2C putative (DUF26) | Chr4:14151387-14153935 FORWARD LENGTH=625 | 201606 Arabidopsis_thaliana OG0000000
AT4G29050.1 | Concanavalin A-like lectin protein kinase family protein | Chr4:14314870-14316879 REVERSE LENGTH=669 | 201606 Arabidopsis_thaliana OG0000000
AT4G29180.1 | root hair specific 16 | Chr4:14385631-14389524 FORWARD LENGTH=911 | 201606 Arabidopsis_thaliana OG0000000
AT4G29450.1 | Leucine-rich repeat protein kinase family protein | Chr4:14478837-14482626 REVERSE LENGTH=863 | 201606 Arabidopsis_thaliana OG0000000
AT4G29654.1 | Protein kinase superfamily protein | Chr4:14531601-14532454 REVERSE LENGTH=158 | 201606 Arabidopsis_thaliana OG0000000
AT4G29990.1 | Leucine-rich repeat transmembrane protein kinase protein | Chr4:14665802-14669438 REVERSE LENGTH=876 | 201606 Arabidopsis_thaliana OG0000000
AT4G30520.1 | Leucine-rich repeat protein kinase family protein | Chr4:14908193-14911040 REVERSE LENGTH=648 | 201606 Arabidopsis_thaliana OG0000000
AT4G31100.1 | wall-associated kinase | Chr4:15123862-15126426 FORWARD LENGTH=786 | 201606 Arabidopsis_thaliana OG0000000
AT4G31110.1 | Wall-associated kinase family protein |

Chr4:15127257-15129880 FORWARD LENGTH=793 | 201606
Arabidopsis_thaliana   OG0000000
AT4G31170.1 | Protein kinase superfamily protein |
Chr4:15153499-15154846 REVERSE LENGTH=412 | 201606
Arabidopsis_thaliana   OG0000000
AT4G31230.1 | kinase with adenine nucleotide alpha hydrolases-like
domain-containing protein | Chr4:15173071-15176109 REVERSE
LENGTH=764 | 201606   Arabidopsis_thaliana   OG0000000
AT4G32000.1 | Protein kinase superfamily protein |
Chr4:15474083-15476655 REVERSE LENGTH=418 | 201606
Arabidopsis_thaliana   OG0000000
AT4G32300.1 | S-domain-2 5 | Chr4:15599970-15602435 FORWARD
LENGTH=821 | 201606   Arabidopsis_thaliana   OG0000000
AT4G32710.1 | Protein kinase superfamily protein |
Chr4:15780248-15783242 FORWARD LENGTH=731 | 201606
Arabidopsis_thaliana   OG0000000
AT4G33430.1 | BRI1-associated receptor kinase |
Chr4:16086654-16090288 REVERSE LENGTH=615 | 201606
Arabidopsis_thaliana   OG0000000
AT4G34440.1 | Protein kinase superfamily protein |
Chr4:16466008-16468748 FORWARD LENGTH=670 | 201606
Arabidopsis_thaliana   OG0000000
AT4G34500.1 | Protein kinase superfamily protein |
Chr4:16488005-16490792 REVERSE LENGTH=437 | 201606
Arabidopsis_thaliana   OG0000000
AT4G35600.1 | Protein kinase superfamily protein |
Chr4:16896448-16898714 FORWARD LENGTH=419 | 201606
Arabidopsis_thaliana   OG0000000
AT4G35780.1 | ACT-like protein tyrosine kinase family protein |
Chr4:16946729-16950405 REVERSE LENGTH=570 | 201606
Arabidopsis_thaliana   OG0000000
AT1G20650.1 | Protein kinase superfamily protein |
Chr1:7158422-7160022 REVERSE LENGTH=381 | 201606
Arabidopsis_thaliana   OG0000000
AT4G38470.1 | ACT-like protein tyrosine kinase family protein |
Chr4:17999432-18003551 FORWARD LENGTH=575 | 201606
Arabidopsis_thaliana   OG0000000
AT4G38830.1 | cysteine-rich RLK (RECEPTOR-like protein kinase) 26 |
Chr4:18122339-18124943 FORWARD LENGTH=665 | 201606
Arabidopsis_thaliana   OG0000000
AT4G39110.1 | Malectin/receptor-like protein kinase family protein |
Chr4:18222483-18225119 REVERSE LENGTH=878 | 201606
Arabidopsis_thaliana   OG0000000
AT5G01020.1 | Protein kinase superfamily protein | Chr5:6309-8270
REVERSE LENGTH=410 | 201606   Arabidopsis_thaliana
OG0000000
AT5G01540.1 | lectin receptor kinase a4.1 | Chr5:211285-213333
REVERSE LENGTH=682 | 201606   Arabidopsis_thaliana
OG0000000
AT5G01550.1 | lectin receptor kinase a4.1 | Chr5:214517-216637
REVERSE LENGTH=706 | 201606   Arabidopsis_thaliana
OG0000000
AT5G01560.1 | lectin receptor kinase a4.3 | Chr5:218170-220245
REVERSE LENGTH=691 | 201606   Arabidopsis_thaliana

OG0000000
AT5G01850.1 | Protein kinase superfamily protein |
Chr5:332829-334180 FORWARD LENGTH=333 | 201606
Arabidopsis_thaliana OG0000000
AT5G02070.1 | Protein kinase family protein | Chr5:405895-408220
REVERSE LENGTH=657 | 201606 Arabidopsis_thaliana
OG0000000
AT5G02290.1 | Protein kinase superfamily protein |
Chr5:470387-472397 REVERSE LENGTH=389 | 201606
Arabidopsis_thaliana OG0000000
AT5G02800.1 | Protein kinase superfamily protein |
Chr5:635545-637374 REVERSE LENGTH=378 | 201606
Arabidopsis_thaliana OG0000000
AT5G03140.1 | Concanavalin A-like lectin protein kinase family
protein | Chr5:737750-739885 REVERSE LENGTH=711 | 201606
Arabidopsis_thaliana OG0000000
AT5G03320.1 | Protein kinase superfamily protein |
Chr5:802759-804242 FORWARD LENGTH=420 | 201606
Arabidopsis_thaliana OG0000000
AT5G03350.1 | Legume lectin family protein | Chr5:815804-816628
REVERSE LENGTH=274 | 201606 Arabidopsis_thaliana
OG0000000
AT5G03730.1 | Protein kinase superfamily protein |
Chr5:974958-979660 REVERSE LENGTH=821 | 201606
Arabidopsis_thaliana OG0000000
AT1G21210.1 | wall associated kinase 4 | Chr1:7424653-7427041
FORWARD LENGTH=738 | 201606 Arabidopsis_thaliana
OG0000000
AT1G21230.1 | wall associated kinase 5 | Chr1:7429980-7432346
FORWARD LENGTH=733 | 201606 Arabidopsis_thaliana
OG0000000
AT1G21240.1 | wall associated kinase 3 | Chr1:7434303-7436702
FORWARD LENGTH=741 | 201606 Arabidopsis_thaliana
OG0000000
AT1G21245.1 | Protein kinase superfamily protein |
Chr1:7436842-7437342 FORWARD LENGTH=166 | 201606
Arabidopsis_thaliana OG0000000
AT1G21250.1 | cell wall-associated kinase | Chr1:7439512-7441892
FORWARD LENGTH=735 | 201606 Arabidopsis_thaliana
OG0000000
AT1G21270.1 | wall-associated kinase 2 | Chr1:7444997-7447345
FORWARD LENGTH=732 | 201606 Arabidopsis_thaliana
OG0000000
AT5G06740.1 | Concanavalin A-like lectin protein kinase family
protein | Chr5:2084094-2086052 FORWARD LENGTH=652 | 201606
Arabidopsis_thaliana OG0000000
AT5G06820.1 | STRUBBELIG-receptor family 2 | Chr5:2112994-2116663
FORWARD LENGTH=735 | 201606 Arabidopsis_thaliana
OG0000000
AT1G21590.1 | kinase with adenine nucleotide alpha hydrolases-like
domain-containing protein | Chr1:7566613-7569694 REVERSE LENGTH=756
| 201606 Arabidopsis_thaliana OG0000000
AT5G10290.1 | leucine-rich repeat transmembrane protein kinase
family protein | Chr5:3235462-3238171 REVERSE LENGTH=613 | 201606

Arabidopsis_thaliana OG0000000
AT5G10520.1 | ROP binding protein kinases 1 | Chr5:3320584-3322649 REVERSE LENGTH=467 | 201606 Arabidopsis_thaliana OG0000000
AT5G10530.1 | Concanavalin A-like lectin protein kinase family protein | Chr5:3324978-3326933 REVERSE LENGTH=651 | 201606 Arabidopsis_thaliana OG0000000
AT5G11020.1 | Protein kinase superfamily protein | Chr5:3486439-3488983 REVERSE LENGTH=433 | 201606 Arabidopsis_thaliana OG0000000
AT5G11410.1 | Protein kinase superfamily protein | Chr5:3638431-3639883 REVERSE LENGTH=336 | 201606 Arabidopsis_thaliana OG0000000
AT5G11850.1 | Protein kinase superfamily protein | Chr5:3816632-3821024 REVERSE LENGTH=880 | 201606 Arabidopsis_thaliana OG0000000
AT5G12000.1 | kinase with adenine nucleotide alpha hydrolases-like domain-containing protein | Chr5:3874151-3876780 REVERSE LENGTH=701 | 201606 Arabidopsis_thaliana OG0000000
AT5G12090.1 | Protein kinase superfamily protein | Chr5:3909703-3910877 FORWARD LENGTH=369 | 201606 Arabidopsis_thaliana OG0000000
AT5G13160.1 | Protein kinase superfamily protein | Chr5:4176854-4179682 FORWARD LENGTH=456 | 201606 Arabidopsis_thaliana OG0000000
AT5G15080.1 | Protein kinase superfamily protein | Chr5:4886414-4888555 FORWARD LENGTH=493 | 201606 Arabidopsis_thaliana OG0000000
AT5G15730.1 | Protein kinase superfamily protein | Chr5:5131284-5133046 FORWARD LENGTH=434 | 201606 Arabidopsis_thaliana OG0000000
AT5G16000.1 | NSP-interacting kinase 1 | Chr5:5224264-5227003 FORWARD LENGTH=638 | 201606 Arabidopsis_thaliana OG0000000
AT5G16500.1 | Protein kinase superfamily protein | Chr5:5386733-5389003 REVERSE LENGTH=636 | 201606 Arabidopsis_thaliana OG0000000
AT5G16900.1 | Leucine-rich repeat protein kinase family protein | Chr5:5555254-5559715 FORWARD LENGTH=866 | 201606 Arabidopsis_thaliana OG0000000
AT5G18500.1 | Protein kinase superfamily protein | Chr5:6139263-6141283 FORWARD LENGTH=484 | 201606 Arabidopsis_thaliana OG0000000
AT5G18610.1 | Protein kinase superfamily protein | Chr5:6192736-6195371 FORWARD LENGTH=513 | 201606 Arabidopsis_thaliana OG0000000
AT5G18910.1 | Protein kinase superfamily protein | Chr5:6306994-6309396 REVERSE LENGTH=511 | 201606 Arabidopsis_thaliana OG0000000
AT1G22720.1 | Protein kinase superfamily protein | Chr1:8044232-8045665 REVERSE LENGTH=219 | 201606 Arabidopsis_thaliana OG0000000
AT5G23170.1 | Protein kinase superfamily protein | Chr5:7798411-7799436 REVERSE LENGTH=341 | 201606

Arabidopsis_thaliana   OG0000000
AT5G24010.1 | Protein kinase superfamily protein | Chr5:8113910-8116384 FORWARD LENGTH=824 | 201606   Arabidopsis_thaliana   OG0000000
AT5G24080.1 | Protein kinase superfamily protein | Chr5:8139334-8141839 REVERSE LENGTH=745 | 201606   Arabidopsis_thaliana   OG0000000
AT5G25440.1 | Protein kinase superfamily protein | Chr5:8854975-8856722 REVERSE LENGTH=313 | 201606   Arabidopsis_thaliana   OG0000000
AT5G26150.1 | protein kinase family protein | Chr5:9137461-9140108 REVERSE LENGTH=706 | 201606   Arabidopsis_thaliana   OG0000000
AT5G27790.1 | Protein kinase superfamily protein | Chr5:9840925-9842003 REVERSE LENGTH=327 | 201606   Arabidopsis_thaliana   OG0000000
AT5G28680.1 | Malectin/receptor-like protein kinase family protein | Chr5:10719437-10722013 REVERSE LENGTH=858 | 201606   Arabidopsis_thaliana   OG0000000
AT1G23540.1 | Protein kinase superfamily protein | Chr1:8346942-8349786 REVERSE LENGTH=720 | 201606   Arabidopsis_thaliana   OG0000000
AT5G35370.1 | S-locus lectin protein kinase family protein | Chr5:13588564-13591182 REVERSE LENGTH=872 | 201606   Arabidopsis_thaliana   OG0000000
AT5G35380.1 | kinase with adenine nucleotide alpha hydrolases-like domain-containing protein | Chr5:13593429-13596293 REVERSE LENGTH=731 | 201606   Arabidopsis_thaliana   OG0000000
AT5G35580.1 | Protein kinase superfamily protein | Chr5:13761980-13763851 FORWARD LENGTH=494 | 201606   Arabidopsis_thaliana   OG0000000
AT5G35960.1 | Protein kinase family protein | Chr5:14108524-14110536 REVERSE LENGTH=429 | 201606   Arabidopsis_thaliana   OG0000000
AT5G37790.1 | Protein kinase superfamily protein | Chr5:15008433-15011025 REVERSE LENGTH=552 | 201606   Arabidopsis_thaliana   OG0000000
AT5G38210.1 | Protein kinase family protein | Chr5:15261035-15265376 FORWARD LENGTH=686 | 201606   Arabidopsis_thaliana   OG0000000
AT5G38240.1 | Protein kinase family protein | Chr5:15277239-15279317 REVERSE LENGTH=642 | 201606   Arabidopsis_thaliana   OG0000000
AT5G38250.1 | Protein kinase family protein | Chr5:15280643-15282709 REVERSE LENGTH=620 | 201606   Arabidopsis_thaliana   OG0000000
AT5G38260.1 | Protein kinase superfamily protein | Chr5:15283692-15285837 REVERSE LENGTH=638 | 201606   Arabidopsis_thaliana   OG0000000
AT5G38560.1 | Protein kinase superfamily protein | Chr5:15439844-15443007 FORWARD LENGTH=681 | 201606   Arabidopsis_thaliana   OG0000000
AT5G38990.1 | Malectin/receptor-like protein kinase family protein | Chr5:15608824-15611466 FORWARD LENGTH=880 | 201606

Arabidopsis_thaliana   OG0000000
AT5G39000.1 | Malectin/receptor-like protein kinase family protein | Chr5:15611860-15614481 FORWARD LENGTH=873 | 201606
Arabidopsis_thaliana   OG0000000
AT5G39020.1 | Malectin/receptor-like protein kinase family protein | Chr5:15616917-15619358 FORWARD LENGTH=813 | 201606
Arabidopsis_thaliana   OG0000000
AT5G39030.1 | Protein kinase superfamily protein | Chr5:15620066-15622486 FORWARD LENGTH=806 | 201606
Arabidopsis_thaliana   OG0000000
AT1G24030.1 | Protein kinase superfamily protein | Chr1:8503394-8505195 FORWARD LENGTH=375 | 201606
Arabidopsis_thaliana   OG0000000
AT5G40380.1 | cysteine-rich RLK (RECEPTOR-like protein kinase) 42 | Chr5:16152121-16155038 FORWARD LENGTH=651 | 201606
Arabidopsis_thaliana   OG0000000
AT5G40540.1 | Protein kinase superfamily protein | Chr5:16237630-16239470 FORWARD LENGTH=353 | 201606
Arabidopsis_thaliana   OG0000000
AT5G41730.1 | Protein kinase family protein | Chr5:16684914-16687145 REVERSE LENGTH=711 | 201606   Arabidopsis_thaliana
OG0000000
AT5G42120.1 | Concanavalin A-like lectin protein kinase family protein | Chr5:16833073-16835148 REVERSE LENGTH=691 | 201606
Arabidopsis_thaliana   OG0000000
AT5G42440.1 | Protein kinase superfamily protein | Chr5:16973434-16974513 REVERSE LENGTH=359 | 201606
Arabidopsis_thaliana   OG0000000
AT5G45780.1 | Leucine-rich repeat protein kinase family protein | Chr5:18566946-18569625 REVERSE LENGTH=614 | 201606
Arabidopsis_thaliana   OG0000000
AT5G46080.1 | Protein kinase superfamily protein | Chr5:18689723-18690721 REVERSE LENGTH=332 | 201606
Arabidopsis_thaliana   OG0000000
AT1G24650.1 | Leucine-rich repeat protein kinase family protein | Chr1:8734570-8737315 FORWARD LENGTH=886 | 201606
Arabidopsis_thaliana   OG0000000
AT5G47070.1 | Protein kinase superfamily protein | Chr5:19118683-19120528 REVERSE LENGTH=410 | 201606
Arabidopsis_thaliana   OG0000000
AT5G47850.1 | CRINKLY4 related 4 | Chr5:19378803-19381058 REVERSE LENGTH=751 | 201606   Arabidopsis_thaliana   OG0000000
AT5G49470.1 | PAS domain-containing protein tyrosine kinase family protein | Chr5:20064669-20068311 FORWARD LENGTH=483 | 201606
Arabidopsis_thaliana   OG0000000
AT5G49760.1 | Leucine-rich repeat protein kinase family protein | Chr5:20216679-20221052 FORWARD LENGTH=953 | 201606
Arabidopsis_thaliana   OG0000000
AT5G49770.1 | Leucine-rich repeat protein kinase family protein | Chr5:20222860-20227267 FORWARD LENGTH=946 | 201606
Arabidopsis_thaliana   OG0000000
AT5G49780.1 | Leucine-rich repeat protein kinase family protein | Chr5:20229499-20233095 FORWARD LENGTH=857 | 201606
Arabidopsis_thaliana   OG0000000

AT5G50000.1 | Protein kinase superfamily protein |
Chr5:20342838-20345033 REVERSE LENGTH=385 | 201606
Arabidopsis_thaliana OG0000000
AT5G50180.1 | Protein kinase superfamily protein |
Chr5:20431116-20432883 FORWARD LENGTH=346 | 201606
Arabidopsis_thaliana OG0000000
AT1G25390.1 | Protein kinase superfamily protein |
Chr1:8906640-8908800 REVERSE LENGTH=629 | 201606
Arabidopsis_thaliana OG0000000
AT5G51270.1 | U-box domain-containing protein kinase family protein
| Chr5:20835137-20838262 REVERSE LENGTH=805 | 201606
Arabidopsis_thaliana OG0000000
AT1G25570.1 | Di-glucose binding protein with Leucine-rich repeat
domain-containing protein | Chr1:8992183-8995430 REVERSE LENGTH=628
| 201606 Arabidopsis_thaliana OG0000000
AT1G26150.1 | proline-rich extensin-like receptor kinase 10 |
Chr1:9039790-9042873 REVERSE LENGTH=762 | 201606
Arabidopsis_thaliana OG0000000
AT5G54380.1 | protein kinase family protein | Chr5:22077313-22079880
REVERSE LENGTH=855 | 201606 Arabidopsis_thaliana
OG0000000
AT5G54590.1 | Protein kinase superfamily protein |
Chr5:22180480-22181900 FORWARD LENGTH=261 | 201606
Arabidopsis_thaliana OG0000000
AT5G55830.1 | Concanavalin A-like lectin protein kinase family
protein | Chr5:22594655-22596700 FORWARD LENGTH=681 | 201606
Arabidopsis_thaliana OG0000000
AT5G56460.1 | Protein kinase superfamily protein |
Chr5:22865509-22867866 FORWARD LENGTH=408 | 201606
Arabidopsis_thaliana OG0000000
AT5G56790.1 | Protein kinase superfamily protein |
Chr5:22968610-22971391 FORWARD LENGTH=669 | 201606
Arabidopsis_thaliana OG0000000
AT5G56890.1 | Protein kinase superfamily protein |
Chr5:23010801-23015559 REVERSE LENGTH=1113 | 201606
Arabidopsis_thaliana OG0000000
AT5G57035.1 | U-box domain-containing protein kinase family protein
| Chr5:23080743-23083819 FORWARD LENGTH=789 | 201606
Arabidopsis_thaliana OG0000000
AT5G57610.1 | kinase superfamily with octicosapeptide/Phox/Bem1p
domain-containing protein | Chr5:23325307-23329099 FORWARD
LENGTH=1054 | 201606 Arabidopsis_thaliana OG0000000
AT5G57670.2 | Protein kinase superfamily protein |
Chr5:23360531-23363694 REVERSE LENGTH=579 | 201606
Arabidopsis_thaliana OG0000000
AT5G58940.1 | calmodulin-binding receptor-like cytoplasmic kinase 1
| Chr5:23798659-23800716 FORWARD LENGTH=470 | 201606
Arabidopsis_thaliana OG0000000
AT5G58950.1 | Protein kinase superfamily protein |
Chr5:23801136-23803025 REVERSE LENGTH=525 | 201606
Arabidopsis_thaliana OG0000000
AT5G59260.1 | Concanavalin A-like lectin protein kinase family
protein | Chr5:23907901-23909925 REVERSE LENGTH=674 | 201606
Arabidopsis_thaliana OG0000000

AT5G59270.1 | Concanavalin A-like lectin protein kinase family protein | Chr5:23911151-23913235 REVERSE LENGTH=668 | 201606
Arabidopsis_thaliana   OG0000000
AT5G59650.1 | Leucine-rich repeat protein kinase family protein | Chr5:24031346-24035100 FORWARD LENGTH=884 | 201606
Arabidopsis_thaliana   OG0000000
AT5G59660.1 | Leucine-rich repeat protein kinase family protein | Chr5:24035687-24039979 FORWARD LENGTH=852 | 201606
Arabidopsis_thaliana   OG0000000
AT5G59670.1 | Leucine-rich repeat protein kinase family protein | Chr5:24041538-24045478 FORWARD LENGTH=868 | 201606
Arabidopsis_thaliana   OG0000000
AT5G59680.1 | Leucine-rich repeat protein kinase family protein | Chr5:24046792-24050801 FORWARD LENGTH=887 | 201606
Arabidopsis_thaliana   OG0000000
AT5G59700.1 | Protein kinase superfamily protein | Chr5:24052613-24055102 REVERSE LENGTH=829 | 201606
Arabidopsis_thaliana   OG0000000
AT5G60080.1 | Protein kinase superfamily protein | Chr5:24193181-24194909 REVERSE LENGTH=377 | 201606
Arabidopsis_thaliana   OG0000000
AT5G60090.1 | Protein kinase superfamily protein | Chr5:24196082-24197725 REVERSE LENGTH=398 | 201606
Arabidopsis_thaliana   OG0000000
AT5G60270.1 | Concanavalin A-like lectin protein kinase family protein | Chr5:24257761-24259767 FORWARD LENGTH=668 | 201606
Arabidopsis_thaliana   OG0000000
AT5G60280.1 | Concanavalin A-like lectin protein kinase family protein | Chr5:24260563-24262536 FORWARD LENGTH=657 | 201606
Arabidopsis_thaliana   OG0000000
AT5G60300.1 | Concanavalin A-like lectin protein kinase family protein | Chr5:24264862-24267018 FORWARD LENGTH=718 | 201606
Arabidopsis_thaliana   OG0000000
AT5G60310.1 | Concanavalin A-like lectin protein kinase family protein | Chr5:24268011-24269982 FORWARD LENGTH=616 | 201606
Arabidopsis_thaliana   OG0000000
AT5G60320.1 | Concanavalin A-like lectin protein kinase family protein | Chr5:24270808-24272835 FORWARD LENGTH=675 | 201606
Arabidopsis_thaliana   OG0000000
AT5G61350.1 | Protein kinase superfamily protein | Chr5:24667973-24670501 FORWARD LENGTH=842 | 201606
Arabidopsis_thaliana   OG0000000
AT5G61550.1 | U-box domain-containing protein kinase family protein | Chr5:24748325-24751558 FORWARD LENGTH=845 | 201606
Arabidopsis_thaliana   OG0000000
AT5G61560.1 | U-box domain-containing protein kinase family protein | Chr5:24753476-24756506 FORWARD LENGTH=796 | 201606
Arabidopsis_thaliana   OG0000000
AT1G26970.1 | Protein kinase superfamily protein | Chr1:9359826-9361666 FORWARD LENGTH=412 | 201606
Arabidopsis_thaliana   OG0000000
AT5G62710.1 | Leucine-rich repeat protein kinase family protein | Chr5:25187438-25190325 FORWARD LENGTH=604 | 201606
Arabidopsis_thaliana   OG0000000

AT5G63710.1 | Leucine-rich repeat protein kinase family protein | Chr5:25499475-25502598 FORWARD LENGTH=614 | 201606 Arabidopsis_thaliana OG0000000
AT5G63940.1 | kinase with adenine nucleotide alpha hydrolases-like domain-containing protein | Chr5:25588254-25591229 FORWARD LENGTH=705 | 201606 Arabidopsis_thaliana OG0000000
AT1G27190.1 | Leucine-rich repeat protein kinase family protein | Chr1:9446923-9448728 REVERSE LENGTH=601 | 201606 Arabidopsis_thaliana OG0000000
AT5G65240.1 | Leucine-rich repeat protein kinase family protein | Chr5:26074980-26077650 REVERSE LENGTH=607 | 201606 Arabidopsis_thaliana OG0000000
AT5G65500.1 | U-box domain-containing protein kinase family protein | Chr5:26181093-26183997 REVERSE LENGTH=791 | 201606 Arabidopsis_thaliana OG0000000
AT5G65530.1 | Protein kinase superfamily protein | Chr5:26190844-26192826 REVERSE LENGTH=456 | 201606 Arabidopsis_thaliana OG0000000
AT5G65600.1 | Concanavalin A-like lectin protein kinase family protein | Chr5:26216126-26218153 REVERSE LENGTH=675 | 201606 Arabidopsis_thaliana OG0000000
AT5G66710.1 | Protein kinase superfamily protein | Chr5:26636609-26638564 FORWARD LENGTH=405 | 201606 Arabidopsis_thaliana OG0000000
AT5G66790.1 | Protein kinase superfamily protein | Chr5:26665181-26667387 FORWARD LENGTH=622 | 201606 Arabidopsis_thaliana OG0000000
AT1G28340.1 | receptor like protein 4 | Chr1:9940175-9943252 FORWARD LENGTH=626 | 201606 Arabidopsis_thaliana OG0000000
AT1G28390.1 | Protein kinase superfamily protein | Chr1:9966814-9968226 REVERSE LENGTH=470 | 201606 Arabidopsis_thaliana OG0000000
AT1G29720.1 | Leucine-rich repeat transmembrane protein kinase | Chr1:10393894-10399771 REVERSE LENGTH=1019 | 201606 Arabidopsis_thaliana OG0000000
AT1G29750.1 | receptor-like kinase in flowers 1 | Chr1:10414071-10419813 REVERSE LENGTH=1006 | 201606 Arabidopsis_thaliana OG0000000
AT1G30570.1 | hercules receptor kinase 2 | Chr1:10828933-10831482 FORWARD LENGTH=849 | 201606 Arabidopsis_thaliana OG0000000
AT1G31420.1 | Leucine-rich repeat protein kinase family protein | Chr1:11250360-11253516 FORWARD LENGTH=592 | 201606 Arabidopsis_thaliana OG0000000
AT1G34210.1 | somatic embryogenesis receptor-like kinase 2 | Chr1:12459078-12462752 FORWARD LENGTH=628 | 201606 Arabidopsis_thaliana OG0000000
AT1G48210.1 | Protein kinase superfamily protein | Chr1:17799551-17801798 FORWARD LENGTH=363 | 201606 Arabidopsis_thaliana OG0000000
AT1G48220.1 | Protein kinase superfamily protein | Chr1:17802863-17804882 FORWARD LENGTH=364 | 201606 Arabidopsis_thaliana OG0000000
AT1G04700.1 | PB1 domain-containing protein tyrosine kinase |

Chr1:1316919-1320653 FORWARD LENGTH=1042 | 201606
Arabidopsis_thaliana   OG0000000
AT1G49100.1 | Leucine-rich repeat protein kinase family protein |
Chr1:18166147-18170105 REVERSE LENGTH=888 | 201606
Arabidopsis_thaliana   OG0000000
AT1G49270.1 | Protein kinase superfamily protein |
Chr1:18227334-18230227 REVERSE LENGTH=699 | 201606
Arabidopsis_thaliana   OG0000000
AT1G49730.1 | Protein kinase superfamily protein |
Chr1:18402618-18405638 REVERSE LENGTH=693 | 201606
Arabidopsis_thaliana   OG0000000
AT1G51620.1 | Protein kinase superfamily protein |
Chr1:19140536-19141638 FORWARD LENGTH=262 | 201606
Arabidopsis_thaliana   OG0000000
AT1G51790.1 | Leucine-rich repeat protein kinase family protein |
Chr1:19206858-19210574 REVERSE LENGTH=882 | 201606
Arabidopsis_thaliana   OG0000000
AT1G51800.1 | Leucine-rich repeat protein kinase family protein |
Chr1:19214203-19217833 FORWARD LENGTH=894 | 201606
Arabidopsis_thaliana   OG0000000
AT1G51805.1 | Leucine-rich repeat protein kinase family protein |
Chr1:19221187-19225590 REVERSE LENGTH=884 | 201606
Arabidopsis_thaliana   OG0000000
AT1G51810.1 | Leucine-rich repeat protein kinase family protein |
Chr1:19227119-19230584 REVERSE LENGTH=744 | 201606
Arabidopsis_thaliana   OG0000000
AT1G51820.1 | Leucine-rich repeat protein kinase family protein |
Chr1:19237407-19241883 REVERSE LENGTH=885 | 201606
Arabidopsis_thaliana   OG0000000
AT1G51830.1 | Leucine-rich repeat protein kinase family protein |
Chr1:19243025-19246010 REVERSE LENGTH=693 | 201606
Arabidopsis_thaliana   OG0000000
AT1G51840.1 | kinase-like protein | Chr1:19249449-19250171 REVERSE
LENGTH=207 | 201606   Arabidopsis_thaliana   OG0000000
AT1G51850.1 | Leucine-rich repeat protein kinase family protein |
Chr1:19252964-19256783 REVERSE LENGTH=865 | 201606
Arabidopsis_thaliana   OG0000000
AT1G51860.1 | Leucine-rich repeat protein kinase family protein |
Chr1:19257634-19261479 REVERSE LENGTH=890 | 201606
Arabidopsis_thaliana   OG0000000
AT1G51870.1 | protein kinase family protein | Chr1:19262879-19267001
REVERSE LENGTH=837 | 201606   Arabidopsis_thaliana
OG0000000
AT1G51880.1 | root hair specific 6 | Chr1:19270193-19274068 REVERSE
LENGTH=872 | 201606   Arabidopsis_thaliana   OG0000000
AT1G51890.1 | Leucine-rich repeat protein kinase family protein |
Chr1:19274802-19278528 REVERSE LENGTH=876 | 201606
Arabidopsis_thaliana   OG0000000
AT1G51910.1 | Leucine-rich repeat protein kinase family protein |
Chr1:19284277-19288385 REVERSE LENGTH=876 | 201606
Arabidopsis_thaliana   OG0000000
AT1G51940.1 | protein kinase family protein / peptidoglycan-binding
LysM domain-containing protein | Chr1:19296092-19298941 REVERSE
LENGTH=651 | 201606   Arabidopsis_thaliana   OG0000000

AT1G52290.1 | Protein kinase superfamily protein |
Chr1:19470251-19472362 REVERSE LENGTH=509 | 201606
Arabidopsis_thaliana OG0000000
AT1G52540.1 | Protein kinase superfamily protein |
Chr1:19570298-19571884 REVERSE LENGTH=350 | 201606
Arabidopsis_thaliana OG0000000
AT1G53060.1 | Legume lectin family protein | Chr1:19776052-19776780
FORWARD LENGTH=242 | 201606 Arabidopsis_thaliana
OG0000000
AT1G53070.1 | Legume lectin family protein | Chr1:19778371-19779189
FORWARD LENGTH=272 | 201606 Arabidopsis_thaliana
OG0000000
AT1G53080.1 | Legume lectin family protein | Chr1:19781368-19782219
FORWARD LENGTH=283 | 201606 Arabidopsis_thaliana
OG0000000
AT1G53420.1 | Leucine-rich repeat transmembrane protein kinase |
Chr1:19926626-19931494 REVERSE LENGTH=953 | 201606
Arabidopsis_thaliana OG0000000
AT1G53430.1 | Leucine-rich repeat transmembrane protein kinase |
Chr1:19935298-19940959 FORWARD LENGTH=1038 | 201606
Arabidopsis_thaliana OG0000000
AT1G53440.1 | Leucine-rich repeat transmembrane protein kinase |
Chr1:19945959-19951562 FORWARD LENGTH=1035 | 201606
Arabidopsis_thaliana OG0000000
AT1G53730.1 | STRUBBELIG-receptor family 6 | Chr1:20061771-20065475
FORWARD LENGTH=719 | 201606 Arabidopsis_thaliana
OG0000000
AT1G54820.1 | Protein kinase superfamily protein |
Chr1:20447370-20450761 FORWARD LENGTH=458 | 201606
Arabidopsis_thaliana OG0000000
AT1G01450.1 | Protein kinase superfamily protein |
Chr1:164105-165517 REVERSE LENGTH=470 | 201606
Arabidopsis_thaliana OG0000000
AT1G55200.1 | kinase with adenine nucleotide alpha hydrolases-like
domain-containing protein | Chr1:20589309-20592049 REVERSE
LENGTH=676 | 201606 Arabidopsis_thaliana OG0000000
AT1G56030.1 | RING/U-box protein | Chr1:20957098-20958550 REVERSE
LENGTH=391 | 201606 Arabidopsis_thaliana OG0000000
AT1G56040.1 | HEAT/U-box protein | Chr1:20960356-20961870 REVERSE
LENGTH=420 | 201606 Arabidopsis_thaliana OG0000000
AT1G56120.1 | Leucine-rich repeat transmembrane protein kinase |
Chr1:20987288-20993072 REVERSE LENGTH=1047 | 201606
Arabidopsis_thaliana OG0000000
AT1G56130.1 | Leucine-rich repeat transmembrane protein kinase |
Chr1:20994931-20999977 REVERSE LENGTH=884 | 201606
Arabidopsis_thaliana OG0000000
AT1G56140.1 | Leucine-rich repeat transmembrane protein kinase |
Chr1:21001708-21007725 REVERSE LENGTH=1033 | 201606
Arabidopsis_thaliana OG0000000
AT1G56145.1 | Leucine-rich repeat transmembrane protein kinase |
Chr1:21008225-21013934 REVERSE LENGTH=1012 | 201606
Arabidopsis_thaliana OG0000000
AT1G56720.1 | Protein kinase superfamily protein |
Chr1:21263630-21265559 REVERSE LENGTH=492 | 201606

Arabidopsis_thaliana	OG0000000
AT1G05700.1 | Leucine-rich repeat transmembrane protein kinase protein | Chr1:1709796-1713245 FORWARD LENGTH=852 | 201606	Arabidopsis_thaliana	OG0000000
AT1G60800.1 | NSP-interacting kinase 3 | Chr1:22383601-22386931 REVERSE LENGTH=632 | 201606	Arabidopsis_thaliana	OG0000000
AT1G61360.1 | S-locus lectin protein kinase family protein | Chr1:22637867-22640974 REVERSE LENGTH=821 | 201606	Arabidopsis_thaliana	OG0000000
AT1G61370.1 | S-locus lectin protein kinase family protein | Chr1:22642096-22645147 REVERSE LENGTH=814 | 201606	Arabidopsis_thaliana	OG0000000
AT1G61380.1 | S-domain-1 29 | Chr1:22646277-22649401 REVERSE LENGTH=805 | 201606	Arabidopsis_thaliana	OG0000000
AT1G61390.1 | S-locus lectin protein kinase family protein | Chr1:22650338-22653639 REVERSE LENGTH=831 | 201606	Arabidopsis_thaliana	OG0000000
AT1G61400.1 | S-locus lectin protein kinase family protein | Chr1:22654638-22657774 REVERSE LENGTH=814 | 201606	Arabidopsis_thaliana	OG0000000
AT1G61420.1 | S-locus lectin protein kinase family protein | Chr1:22660557-22663596 REVERSE LENGTH=807 | 201606	Arabidopsis_thaliana	OG0000000
AT1G61430.1 | S-locus lectin protein kinase family protein | Chr1:22664669-22667769 REVERSE LENGTH=806 | 201606	Arabidopsis_thaliana	OG0000000
AT1G61440.1 | S-locus lectin protein kinase family protein | Chr1:22669245-22672323 REVERSE LENGTH=792 | 201606	Arabidopsis_thaliana	OG0000000
AT1G61460.1 | G-type lectin S-receptor-like Serine/Threonine-kinase | Chr1:22674268-22677188 REVERSE LENGTH=749 | 201606	Arabidopsis_thaliana	OG0000000
AT1G61480.1 | S-locus lectin protein kinase family protein | Chr1:22681420-22684404 REVERSE LENGTH=809 | 201606	Arabidopsis_thaliana	OG0000000
AT1G61490.1 | S-locus lectin protein kinase family protein | Chr1:22685154-22688267 REVERSE LENGTH=804 | 201606	Arabidopsis_thaliana	OG0000000
AT1G61500.1 | S-locus lectin protein kinase family protein | Chr1:22689729-22692881 REVERSE LENGTH=804 | 201606	Arabidopsis_thaliana	OG0000000
AT1G61550.1 | S-locus lectin protein kinase family protein | Chr1:22704866-22707826 REVERSE LENGTH=802 | 201606	Arabidopsis_thaliana	OG0000000
AT1G61590.1 | Protein kinase superfamily protein | Chr1:22723691-22726022 REVERSE LENGTH=424 | 201606	Arabidopsis_thaliana	OG0000000
AT1G61610.1 | S-locus lectin protein kinase family protein | Chr1:22733472-22736509 FORWARD LENGTH=842 | 201606	Arabidopsis_thaliana	OG0000000
AT1G61860.1 | Protein kinase superfamily protein | Chr1:22863079-22864619 REVERSE LENGTH=389 | 201606	Arabidopsis_thaliana	OG0000000

AT1G62400.1 | Protein kinase superfamily protein |
Chr1:23090108-23091529 FORWARD LENGTH=390 | 201606
Arabidopsis_thaliana   OG0000000
AT1G64300.1 | Protein kinase family protein | Chr1:23863543-23865776
FORWARD LENGTH=717 | 201606   Arabidopsis_thaliana
OG0000000
AT1G65790.1 | receptor kinase 1 | Chr1:24468932-24472329 FORWARD
LENGTH=843 | 201606   Arabidopsis_thaliana   OG0000000
AT1G65800.1 | receptor kinase 2 | Chr1:24473166-24476523 FORWARD
LENGTH=847 | 201606   Arabidopsis_thaliana   OG0000000
AT1G01540.1 | Protein kinase superfamily protein |
Chr1:195980-197973 FORWARD LENGTH=386 | 201606
Arabidopsis_thaliana   OG0000000
AT1G66150.1 | transmembrane kinase 1 | Chr1:24631503-24634415
FORWARD LENGTH=942 | 201606   Arabidopsis_thaliana
OG0000000
AT1G66460.1 | Protein kinase superfamily protein |
Chr1:24789894-24791988 REVERSE LENGTH=467 | 201606
Arabidopsis_thaliana   OG0000000
AT1G66880.1 | Protein kinase superfamily protein |
Chr1:24946928-24955438 FORWARD LENGTH=1296 | 201606
Arabidopsis_thaliana   OG0000000
AT1G66910.1 | Protein kinase superfamily protein |
Chr1:24961634-24963941 REVERSE LENGTH=666 | 201606
Arabidopsis_thaliana   OG0000000
AT1G66920.1 | Protein kinase superfamily protein |
Chr1:24965410-24967432 REVERSE LENGTH=609 | 201606
Arabidopsis_thaliana   OG0000000
AT1G66930.1 | Protein kinase superfamily protein |
Chr1:24970523-24973069 FORWARD LENGTH=674 | 201606
Arabidopsis_thaliana   OG0000000
AT1G66940.1 | kinase-like protein | Chr1:24973201-24974684 REVERSE
LENGTH=332 | 201606   Arabidopsis_thaliana   OG0000000
AT1G67000.1 | Protein kinase superfamily protein |
Chr1:25004217-25007604 REVERSE LENGTH=892 | 201606
Arabidopsis_thaliana   OG0000000
AT1G67520.1 | lectin protein kinase family protein |
Chr1:25303439-25305857 REVERSE LENGTH=587 | 201606
Arabidopsis_thaliana   OG0000000
AT1G67890.1 | PAS domain-containing protein tyrosine kinase family
protein | Chr1:25457345-25462436 FORWARD LENGTH=765 | 201606
Arabidopsis_thaliana   OG0000000
AT1G68690.1 | Protein kinase superfamily protein |
Chr1:25789192-25791886 FORWARD LENGTH=708 | 201606
Arabidopsis_thaliana   OG0000000
AT1G69270.1 | receptor-like protein kinase 1 |
Chr1:26040877-26042499 REVERSE LENGTH=540 | 201606
Arabidopsis_thaliana   OG0000000
AT1G69730.1 | Wall-associated kinase family protein |
Chr1:26228703-26231339 REVERSE LENGTH=792 | 201606
Arabidopsis_thaliana   OG0000000
AT1G69790.1 | Protein kinase superfamily protein |
Chr1:26266838-26268818 FORWARD LENGTH=387 | 201606
Arabidopsis_thaliana   OG0000000

AT1G69910.1 | Protein kinase superfamily protein |
Chr1:26330166-26332076 FORWARD LENGTH=636 | 201606
Arabidopsis_thaliana   OG0000000
AT1G70110.1 | Concanavalin A-like lectin protein kinase family
protein | Chr1:26406238-26408323 REVERSE LENGTH=666 | 201606
Arabidopsis_thaliana   OG0000000
AT1G70130.1 | Concanavalin A-like lectin protein kinase family
protein | Chr1:26409743-26411801 REVERSE LENGTH=656 | 201606
Arabidopsis_thaliana   OG0000000
AT1G70450.1 | Protein kinase superfamily protein |
Chr1:26552576-26554437 FORWARD LENGTH=394 | 201606
Arabidopsis_thaliana   OG0000000
AT1G70460.1 | root hair specific 10 | Chr1:26556155-26558994 FORWARD
LENGTH=710 | 201606   Arabidopsis_thaliana   OG0000000
AT1G70520.1 | cysteine-rich RLK (RECEPTOR-like protein kinase) 2 |
Chr1:26584888-26587334 REVERSE LENGTH=649 | 201606
Arabidopsis_thaliana   OG0000000
AT1G70530.1 | cysteine-rich RLK (RECEPTOR-like protein kinase) 3 |
Chr1:26588750-26591379 REVERSE LENGTH=646 | 201606
Arabidopsis_thaliana   OG0000000
AT1G06700.1 | Protein kinase superfamily protein |
Chr1:2052750-2054552 REVERSE LENGTH=361 | 201606
Arabidopsis_thaliana   OG0000000
AT1G70740.1 | Protein kinase superfamily protein |
Chr1:26673847-26675687 REVERSE LENGTH=425 | 201606
Arabidopsis_thaliana   OG0000000
AT1G71830.1 | somatic embryogenesis receptor-like kinase 1 |
Chr1:27018575-27021842 FORWARD LENGTH=625 | 201606
Arabidopsis_thaliana   OG0000000
AT1G06840.1 | Leucine-rich repeat protein kinase family protein |
Chr1:2097854-2103208 REVERSE LENGTH=953 | 201606
Arabidopsis_thaliana   OG0000000
AT1G72540.1 | Protein kinase superfamily protein |
Chr1:27314932-27316669 REVERSE LENGTH=450 | 201606
Arabidopsis_thaliana   OG0000000
AT1G72760.1 | Protein kinase superfamily protein |
Chr1:27385421-27388274 REVERSE LENGTH=697 | 201606
Arabidopsis_thaliana   OG0000000
AT1G73660.1 | protein tyrosine kinase family protein |
Chr1:27692247-27696718 REVERSE LENGTH=1030 | 201606
Arabidopsis_thaliana   OG0000000
AT1G74490.1 | Protein kinase superfamily protein |
Chr1:27994760-27996496 REVERSE LENGTH=399 | 201606
Arabidopsis_thaliana   OG0000000
AT1G76360.1 | Protein kinase superfamily protein |
Chr1:28643242-28646483 REVERSE LENGTH=484 | 201606
Arabidopsis_thaliana   OG0000000
AT1G76370.1 | Protein kinase superfamily protein |
Chr1:28648660-28650239 REVERSE LENGTH=381 | 201606
Arabidopsis_thaliana   OG0000000
AT1G77280.1 | kinase with adenine nucleotide alpha hydrolases-like
domain-containing protein | Chr1:29031468-29034574 REVERSE
LENGTH=753 | 201606   Arabidopsis_thaliana   OG0000000
AT1G07460.1 | Concanavalin A-like lectin family protein |

Chr1:2290201-2290977 FORWARD LENGTH=258 | 201606
Arabidopsis_thaliana OG0000000
AT1G78530.1 | Protein kinase superfamily protein |
Chr1:29539274-29540681 REVERSE LENGTH=355 | 201606
Arabidopsis_thaliana OG0000000
AT1G01670.1 | RING/U-box superfamily protein | Chr1:242943-245163
REVERSE LENGTH=365 | 201606 Arabidopsis_thaliana
OG0000000
AT1G78940.1 | kinase with adenine nucleotide alpha hydrolases-like
domain-containing protein | Chr1:29681056-29683678 REVERSE
LENGTH=680 | 201606 Arabidopsis_thaliana OG0000000
AT1G78980.1 | STRUBBELIG-receptor family 5 | Chr1:29707923-29711266
REVERSE LENGTH=699 | 201606 Arabidopsis_thaliana
OG0000000
AT1G07550.1 | Leucine-rich repeat protein kinase family protein |
Chr1:2322709-2326512 REVERSE LENGTH=864 | 201606
Arabidopsis_thaliana OG0000000
AT1G07560.1 | Leucine-rich repeat protein kinase family protein |
Chr1:2327320-2331096 FORWARD LENGTH=871 | 201606
Arabidopsis_thaliana OG0000000
AT1G07570.1 | Protein kinase superfamily protein |
Chr1:2331369-2333210 REVERSE LENGTH=410 | 201606
Arabidopsis_thaliana OG0000000
AT1G01680.1 | plant U-box 54 | Chr1:246411-248329 REVERSE LENGTH=308
| 201606 Arabidopsis_thaliana OG0000000
AT1G79570.1 | kinase superfamily with octicosapeptide/Phox/Bem1p
domain-containing protein | Chr1:29932856-29937540 REVERSE
LENGTH=1248 | 201606 Arabidopsis_thaliana OG0000000
AT1G79620.1 | Leucine-rich repeat protein kinase family protein |
Chr1:29957633-29962174 REVERSE LENGTH=971 | 201606
Arabidopsis_thaliana OG0000000
AT1G79670.1 | Wall-associated kinase family protein |
Chr1:29976887-29979337 REVERSE LENGTH=751 | 201606
Arabidopsis_thaliana OG0000000
AT1G79680.1 | WALL ASSOCIATED KINASE (WAK)-LIKE 10 |
Chr1:29980188-29982749 REVERSE LENGTH=769 | 201606
Arabidopsis_thaliana OG0000000
AT1G07650.1 | Leucine-rich repeat transmembrane protein kinase |
Chr1:2359817-2366423 REVERSE LENGTH=1014 | 201606
Arabidopsis_thaliana OG0000000
AT1G80640.1 | Protein kinase superfamily protein |
Chr1:30311979-30314238 FORWARD LENGTH=427 | 201606
Arabidopsis_thaliana OG0000000
AT2G01820.1 | Leucine-rich repeat protein kinase family protein |
Chr2:357664-360681 REVERSE LENGTH=943 | 201606
Arabidopsis_thaliana OG0000000
AT1G07870.1 | Protein kinase superfamily protein |
Chr1:2429933-2431843 REVERSE LENGTH=423 | 201606
Arabidopsis_thaliana OG0000000
AT2G02800.1 | protein kinase 2B | Chr2:796889-799250 REVERSE
LENGTH=426 | 201606 Arabidopsis_thaliana OG0000000
AT2G04300.1 | Leucine-rich repeat protein kinase family protein |
Chr2:1493009-1496914 FORWARD LENGTH=851 | 201606
Arabidopsis_thaliana OG0000000

AT2G05940.1 | Protein kinase superfamily protein | Chr2:2287514-2289270 REVERSE LENGTH=462 | 201606 Arabidopsis_thaliana OG0000000
AT2G07020.1 | kinase with adenine nucleotide alpha hydrolases-like domain-containing protein | Chr2:2908473-2911198 REVERSE LENGTH=700 | 201606 Arabidopsis_thaliana OG0000000
AT2G07180.1 | Protein kinase superfamily protein | Chr2:2981082-2983271 REVERSE LENGTH=442 | 201606 Arabidopsis_thaliana OG0000000
AT2G11520.1 | calmodulin-binding receptor-like cytoplasmic kinase 3 | Chr2:4619145-4621448 FORWARD LENGTH=510 | 201606 Arabidopsis_thaliana OG0000000
AT2G13790.1 | somatic embryogenesis receptor-like kinase 4 | Chr2:5741979-5746581 FORWARD LENGTH=620 | 201606 Arabidopsis_thaliana OG0000000
AT2G14440.1 | Leucine-rich repeat protein kinase family protein | Chr2:6143073-6147419 FORWARD LENGTH=869 | 201606 Arabidopsis_thaliana OG0000000
AT2G14510.1 | Leucine-rich repeat protein kinase family protein | Chr2:6171133-6175052 REVERSE LENGTH=868 | 201606 Arabidopsis_thaliana OG0000000
AT2G16750.1 | kinase with adenine nucleotide alpha hydrolases-like domain-containing protein | Chr2:7271786-7274446 FORWARD LENGTH=617 | 201606 Arabidopsis_thaliana OG0000000
AT2G17220.1 | Protein kinase superfamily protein | Chr2:7487866-7489768 REVERSE LENGTH=414 | 201606 Arabidopsis_thaliana OG0000000
AT2G17700.1 | ACT-like protein tyrosine kinase family protein | Chr2:7685778-7689278 REVERSE LENGTH=546 | 201606 Arabidopsis_thaliana OG0000000
AT1G08720.1 | Protein kinase superfamily protein | Chr1:2774089-2779077 FORWARD LENGTH=933 | 201606 Arabidopsis_thaliana OG0000000
AT2G18470.1 | roline-rich extensin-like receptor kinase 4 | Chr2:8005285-8007767 REVERSE LENGTH=633 | 201606 Arabidopsis_thaliana OG0000000
AT2G18890.1 | Protein kinase superfamily protein | Chr2:8184027-8186685 FORWARD LENGTH=392 | 201606 Arabidopsis_thaliana OG0000000
AT2G19130.1 | S-locus lectin protein kinase family protein | Chr2:8293789-8296275 FORWARD LENGTH=828 | 201606 Arabidopsis_thaliana OG0000000
AT2G19190.1 | FLG22-induced receptor-like kinase 1 | Chr2:8326067-8329893 REVERSE LENGTH=876 | 201606 Arabidopsis_thaliana OG0000000
AT2G19210.1 | Leucine-rich repeat transmembrane protein kinase protein | Chr2:8335639-8339307 REVERSE LENGTH=881 | 201606 Arabidopsis_thaliana OG0000000
AT2G19230.1 | Leucine-rich repeat transmembrane protein kinase protein | Chr2:8343452-8348431 REVERSE LENGTH=1025 | 201606 Arabidopsis_thaliana OG0000000
AT2G19410.1 | U-box domain-containing protein kinase family protein | Chr2:8404901-8409012 REVERSE LENGTH=801 | 201606 Arabidopsis_thaliana OG0000000

AT2G20300.1 | Protein kinase superfamily protein |
Chr2:8756475-8759845 REVERSE LENGTH=744 | 201606
Arabidopsis_thaliana   OG0000000
AT2G20850.1 | STRUBBELIG-receptor family 1 | Chr2:8975670-8979182
REVERSE LENGTH=775 | 201606   Arabidopsis_thaliana
OG0000000
AT2G21480.1 | Malectin/receptor-like protein kinase family protein |
Chr2:9202753-9205368 REVERSE LENGTH=871 | 201606
Arabidopsis_thaliana   OG0000000
AT2G23200.1 | Protein kinase superfamily protein |
Chr2:9879351-9881855 FORWARD LENGTH=834 | 201606
Arabidopsis_thaliana   OG0000000
AT2G23450.1 | Protein kinase superfamily protein |
Chr2:9988926-9991244 REVERSE LENGTH=708 | 201606
Arabidopsis_thaliana   OG0000000
AT2G23770.1 | protein kinase family protein / peptidoglycan-binding
LysM domain-containing protein | Chr2:10120242-10122080 REVERSE
LENGTH=612 | 201606   Arabidopsis_thaliana   OG0000000
AT2G23950.1 | Leucine-rich repeat protein kinase family protein |
Chr2:10187204-10189969 REVERSE LENGTH=634 | 201606
Arabidopsis_thaliana   OG0000000
AT2G24360.1 | Protein kinase superfamily protein |
Chr2:10364742-10366075 REVERSE LENGTH=411 | 201606
Arabidopsis_thaliana   OG0000000
AT2G24370.1 | kinase with adenine nucleotide alpha hydrolases-like
domain-containing protein | Chr2:10369979-10373063 REVERSE
LENGTH=788 | 201606   Arabidopsis_thaliana   OG0000000
AT2G25220.1 | Protein kinase superfamily protein |
Chr2:10742918-10745594 REVERSE LENGTH=432 | 201606
Arabidopsis_thaliana   OG0000000
AT1G09440.1 | Protein kinase superfamily protein |
Chr1:3045513-3047393 REVERSE LENGTH=466 | 201606
Arabidopsis_thaliana   OG0000000
AT2G26290.1 | root-specific kinase 1 | Chr2:11192237-11194259
REVERSE LENGTH=424 | 201606   Arabidopsis_thaliana
OG0000000
AT2G28250.1 | Protein kinase superfamily protein |
Chr2:12044004-12046339 FORWARD LENGTH=565 | 201606
Arabidopsis_thaliana   OG0000000
AT2G28590.1 | Protein kinase superfamily protein |
Chr2:12249835-12251490 FORWARD LENGTH=424 | 201606
Arabidopsis_thaliana   OG0000000
AT2G28930.1 | protein kinase 1B | Chr2:12424957-12426565 FORWARD
LENGTH=423 | 201606   Arabidopsis_thaliana   OG0000000
AT2G28940.1 | Protein kinase superfamily protein |
Chr2:12426853-12428314 REVERSE LENGTH=343 | 201606
Arabidopsis_thaliana   OG0000000
AT2G28960.1 | Leucine-rich repeat protein kinase family protein |
Chr2:12438058-12441645 REVERSE LENGTH=748 | 201606
Arabidopsis_thaliana   OG0000000
AT2G28970.1 | Leucine-rich repeat protein kinase family protein |
Chr2:12443919-12448163 FORWARD LENGTH=786 | 201606
Arabidopsis_thaliana   OG0000000
AT2G28990.1 | Leucine-rich repeat protein kinase family protein |

Chr2:12455055-12459541 FORWARD LENGTH=884 | 201606
Arabidopsis_thaliana   OG0000000
AT2G29000.1 | Leucine-rich repeat protein kinase family protein |
Chr2:12460781-12465037 FORWARD LENGTH=872 | 201606
Arabidopsis_thaliana   OG0000000
AT2G29220.1 | Concanavalin A-like lectin protein kinase family
protein | Chr2:12562781-12564664 REVERSE LENGTH=627 | 201606
Arabidopsis_thaliana   OG0000000
AT2G29250.1 | Concanavalin A-like lectin protein kinase family
protein | Chr2:12578909-12580780 REVERSE LENGTH=623 | 201606
Arabidopsis_thaliana   OG0000000
AT2G30730.1 | Protein kinase superfamily protein |
Chr2:13093145-13094677 FORWARD LENGTH=338 | 201606
Arabidopsis_thaliana   OG0000000
AT2G30740.1 | Protein kinase superfamily protein |
Chr2:13096399-13098285 FORWARD LENGTH=366 | 201606
Arabidopsis_thaliana   OG0000000
AT2G30940.1 | Protein kinase superfamily protein |
Chr2:13168533-13170285 FORWARD LENGTH=451 | 201606
Arabidopsis_thaliana   OG0000000
AT2G31010.1 | Protein kinase superfamily protein |
Chr2:13194939-13199642 FORWARD LENGTH=775 | 201606
Arabidopsis_thaliana   OG0000000
AT2G34290.1 | Protein kinase superfamily protein |
Chr2:14472633-14473430 REVERSE LENGTH=265 | 201606
Arabidopsis_thaliana   OG0000000
Os03t0241600-01 Serine/threonine protein kinase-related domain
containing protein.   Oryza_sativa   OG0000000
Os03t0258000-01 Similar to Resistance protein candidate (Fragment).
Oryza_sativa   OG0000000
Os03t0264300-00 Similar to Protein kinase domain containing protein,
expressed.   Oryza_sativa   OG0000000
Os03t0266800-01 Protein kinase, core domain containing protein.
Oryza_sativa   OG0000000
Os03t0269300-01 Protein kinase-like domain containing protein.
Oryza_sativa   OG0000000
Os03t0274800-01 Similar to Protein kinase APK1A, chloroplast
precursor (EC 2.7.1.-).   Oryza_sativa   OG0000000
Os03t0281500-01 Similar to Resistance protein candidate (Fragment).
Oryza_sativa   OG0000000
Os03t0283900-01 Similar to Serine/threonine-protein kinase PBS1 (EC
2.7.1.37) (AvrPphB susceptible protein 1). Oryza_sativa
OG0000000
Os03t0297800-01 Similar to Leucine Rich Repeat family protein,
expressed.   Oryza_sativa   OG0000000
Os03t0329700-01 Similar to predicted protein.   Oryza_sativa
OG0000000
Os03t0333200-01 Similar to Resistance protein candidate (Fragment).
Oryza_sativa   OG0000000
Os01t0113300-01 Similar to ARK protein (Fragment). Oryza_sativa
OG0000000
Os03t0364400-01 Similar to Phytosulfokine receptor-like protein.
Oryza_sativa   OG0000000
Os01t0113350-00 Hypothetical conserved gene.   Oryza_sativa

OG0000000
Os01t0113400-01 Similar to Receptor-like kinase.   Oryza_sativa
OG0000000
Os01t0247500-01 Protein kinase, core domain containing protein.
Oryza_sativa   OG0000000
Os03t0397700-01 Protein kinase, catalytic domain domain containing
protein. Oryza_sativa   OG0000000
Os03t0407900-01 Similar to Serine/threonine protein kinase-like.
Oryza_sativa   OG0000000
Os03t0422800-01 Similar to D-mannose binding lectin family protein,
expressed.   Oryza_sativa   OG0000000
Os03t0424200-01 Serine/threonine protein kinase-related domain
containing protein.   Oryza_sativa   OG0000000
Os01t0113500-01 Similar to Receptor-like kinase.   Oryza_sativa
OG0000000
Os03t0556600-01 Serine/threonine protein kinase-related domain
containing protein.   Oryza_sativa   OG0000000
Os03t0568800-01 Protein kinase, core domain containing protein.
Oryza_sativa   OG0000000
Os01t0253000-01 Similar to LpimPth3.   Oryza_sativa
OG0000000
Os01t0253100-01 Similar to Avr9/Cf-9 induced kinase 1.
Oryza_sativa   OG0000000
Os01t0113650-00 Hypothetical conserved gene.   Oryza_sativa
OG0000000
Os03t0588400-00 Similar to HT1 protein kinase.   Oryza_sativa
OG0000000
Os01t0113700-00 Similar to Receptor-like kinase.   Oryza_sativa
OG0000000
Os01t0113800-01 Protein kinase, core domain containing protein.
Oryza_sativa   OG0000000
Os03t0638800-01 Similar to ATP binding protein.   Oryza_sativa
OG0000000
Os03t0642600-00 Similar to Protein kinase domain containing protein,
expressed.   Oryza_sativa   OG0000000
Os01t0259200-01 Similar to serine/threonine protein kinase PBS1.
Oryza_sativa   OG0000000
Os01t0114100-01 Similar to Protein kinase RLK17.   Oryza_sativa
OG0000000
Os03t0717000-01 Similar to TMK protein precursor. Oryza_sativa
OG0000000
Os03t0719850-00 Similar to Protein kinase 2.   Oryza_sativa
OG0000000
Os01t0114400-00 Similar to Receptor-like kinase.   Oryza_sativa
OG0000000
Os03t0745700-00 Similar to ATP binding protein.   Oryza_sativa
OG0000000
Os03t0759600-00 Similar to protein kinase family protein.
Oryza_sativa   OG0000000
Os01t0114600-00 Similar to Receptor-like protein kinase.
Oryza_sativa   OG0000000
Os03t0772600-01 Similar to Lectin-like receptor kinase 7;2.
Oryza_sativa   OG0000000
Os03t0772700-00 Similar to lectin-like receptor kinase 7.

Oryza_sativa	OG0000000
Os01t0114700-00 Similar to Receptor-like kinase.	Oryza_sativa
OG0000000
Os01t0114900-00 Similar to LRK14.	Oryza_sativa	OG0000000
Os03t0816100-01 Similar to Protein kinase.	Oryza_sativa
OG0000000
Os03t0821900-01 Similar to Protein kinase APK1A, chloroplast
precursor (EC 2.7.1.-).	Oryza_sativa	OG0000000
Os03t0823000-01 Resistance protein candidate (Fragment).
Oryza_sativa	OG0000000
Os03t0825800-01 Protein kinase, core domain containing protein.
Oryza_sativa	OG0000000
Os03t0828800-01 Serine/threonine protein kinase-related domain
containing protein.	Oryza_sativa	OG0000000
Os03t0839700-00 Hypothetical conserved gene.	Oryza_sativa
OG0000000
Os03t0839900-01 Serine/threonine protein kinase-related domain
containing protein.	Oryza_sativa	OG0000000
Os03t0841100-01 Serine/threonine protein kinase-related domain
containing protein.	Oryza_sativa	OG0000000
Os03t0844100-01 Similar to Pti1 kinase-like protein.
Oryza_sativa	OG0000000
Os01t0115500-00 Similar to Receptor-like kinase.	Oryza_sativa
OG0000000
Os04t0103500-01 Serine/threonine protein kinase-related domain
containing protein.	Oryza_sativa	OG0000000
Os04t0103700-01 Serine/threonine protein kinase-related domain
containing protein.	Oryza_sativa	OG0000000
Os04t0109100-01 Concanavalin A-like lectin/glucanase domain
containing protein.	Oryza_sativa	OG0000000
Os04t0109400-00 Similar to H0201G08.3 protein.	Oryza_sativa
OG0000000
Os04t0109500-01 Legume lectin, beta domain containing protein.
Oryza_sativa	OG0000000
Os04t0110100-01 Concanavalin A-like lectin/glucanase domain
containing protein.	Oryza_sativa	OG0000000
Os04t0113100-00 Protein kinase, catalytic domain domain containing
protein.	Oryza_sativa	OG0000000
Os04t0123800-00 Legume lectin, beta chain domain containing protein.
	Oryza_sativa	OG0000000
Os04t0125700-01 Concanavalin A-like lectin/glucanase domain
containing protein.	Oryza_sativa	OG0000000
Os04t0127500-01 Serine/threonine protein kinase-related domain
containing protein.	Oryza_sativa	OG0000000
Os04t0138800-00 Similar to OSIGBa0140L04.2 protein.
Oryza_sativa	OG0000000
Os04t0140666-01 Similar to OSIGBa0124C14.6 protein.
Oryza_sativa	OG0000000
Os01t0115600-01 Similar to LRK14.	Oryza_sativa	OG0000000
Os04t0177000-00 Similar to Stress-induced receptor-like kinase 2.
Oryza_sativa	OG0000000
Os01t0115700-00 Similar to Receptor-like kinase.	Oryza_sativa
OG0000000
Os04t0197200-01 Protein kinase, core domain containing protein.

Oryza_sativa OG0000000
Os04t0220300-01 EGF-type aspartate/asparagine hydroxylation site domain containing protein. Oryza_sativa OG0000000
Os04t0225250-00 Similar to OSIGBa0148I18.4 protein. Oryza_sativa OG0000000
Os01t0115750-00 Similar to Receptor-like protein kinase. Oryza_sativa OG0000000
Os04t0275100-01 Serine/threonine protein kinase-related domain containing protein. Oryza_sativa OG0000000
Os01t0296000-01 Protein kinase, core domain containing protein. Oryza_sativa OG0000000
Os04t0288500-01 Protein kinase, catalytic domain domain containing protein. Oryza_sativa OG0000000
Os04t0292150-00 Similar to SHR5-receptor-like kinase (Fragment). Oryza_sativa OG0000000
Os04t0303100-00 Similar to H0215E01.10 protein. Oryza_sativa OG0000000
Os04t0307900-00 Similar to H0211A12.1 protein. Oryza_sativa OG0000000
Os04t0308100-01 Similar to Wall-associated kinase 4. Oryza_sativa OG0000000
Os04t0356600-01 Similar to OSIGBa0105P02.3 protein. Oryza_sativa OG0000000
Os04t0365100-01 Similar to OSIGBa0135K14.8 protein. Oryza_sativa OG0000000
Os04t0366000-00 Similar to OSIGBa0110B10.6 protein. Oryza_sativa OG0000000
Os04t0368000-01 Serine/threonine protein kinase-related domain containing protein. Oryza_sativa OG0000000
Os04t0368300-00 Similar to H0607F01.5 protein. Oryza_sativa OG0000000
Os04t0368800-00 Similar to H0607F01.6 protein. Oryza_sativa OG0000000
Os04t0369066-00 Similar to H0607F01.7 protein. Oryza_sativa OG0000000
Os04t0369600-00 Similar to OSIGBa0107E14.3 protein. Oryza_sativa OG0000000
Os04t0370100-01 Similar to OSIGBa0107E14.7 protein. Oryza_sativa OG0000000
Os04t0370900-01 Similar to H0607F01.5 protein. Oryza_sativa OG0000000
Os04t0371200-00 Similar to OSIGBa0134J07.8 protein. Oryza_sativa OG0000000
Os04t0372100-00 Similar to OSIGBa0107E14.7 protein. Oryza_sativa OG0000000
Os01t0116000-00 Similar to Receptor-like protein kinase. Oryza_sativa OG0000000
Os04t0393900-01 Similar to H0718E12.2 protein. Oryza_sativa OG0000000
Os04t0419700-00 Similar to H0525E10.7 protein. Oryza_sativa OG0000000
Os04t0419900-01 Similar to H0525E10.7 protein. Oryza_sativa OG0000000
Os04t0420033-00 Similar to H0525E10.1 protein. Oryza_sativa

OG0000000
Os04t0420300-00 Similar to H0525E10.1 protein.	Oryza_sativa
OG0000000
Os04t0420600-00 Similar to H0525E10.2 protein.	Oryza_sativa
OG0000000
Os04t0430000-01 Similar to Ser Thr specific protein kinase-like protein.	Oryza_sativa	OG0000000
Os04t0430400-01 Hypothetical conserved gene.	Oryza_sativa
OG0000000
Os01t0116200-01 Protein kinase, core domain containing protein.
Oryza_sativa	OG0000000
Os04t0439600-01 Regulator of chromosome condensation/beta-lactamase-inhibitor protein II domain containing protein.	Oryza_sativa
OG0000000
Os01t0310500-01 Protein kinase, catalytic domain domain containing protein.	Oryza_sativa	OG0000000
Os04t0457800-01 Similar to SERK1 (Fragment).	Oryza_sativa
OG0000000
Os01t0116400-01 Protein kinase, core domain containing protein.
Oryza_sativa	OG0000000
Os04t0503600-01 Similar to OSIGBa0112M24.5 protein.
Oryza_sativa	OG0000000
Os04t0506700-00 Similar to OSIGBa0145C12.7 protein.
Oryza_sativa	OG0000000
Os04t0509000-01 Similar to Pto kinase interactor 1.
Oryza_sativa	OG0000000
Os01t0116800-00 Similar to Receptor-like kinase.	Oryza_sativa
OG0000000
Os04t0517700-01 Similar to OSIGBa0145M07.8 protein.
Oryza_sativa	OG0000000
Os01t0323000-01 Similar to Ser Thr specific protein kinase-like protein.	Oryza_sativa	OG0000000
Os01t0323100-01 Similar to Pto kinase interactor 1.
Oryza_sativa	OG0000000
Os01t0116900-00 Protein kinase, catalytic domain domain containing protein.	Oryza_sativa	OG0000000
Os04t0531400-01 Similar to Lectin-like receptor kinase 7;2.
Oryza_sativa	OG0000000
Os04t0531500-01 Concanavalin A-like lectin/glucanase domain containing protein.	Oryza_sativa	OG0000000
Os04t0543000-01 Similar to Protein kinase.	Oryza_sativa
OG0000000
Os04t0563900-00 Similar to H0409D10.5 protein.	Oryza_sativa
OG0000000
Os04t0584100-01 Serine/threonine protein kinase domain containing protein.	Oryza_sativa	OG0000000
Os04t0584500-01 U box domain domain containing protein.
Oryza_sativa	OG0000000
Os01t0117100-00 Similar to Receptor kinase LRK10.	Oryza_sativa
OG0000000
Os04t0598600-00 Protein kinase, catalytic domain domain containing protein.	Oryza_sativa	OG0000000
Os04t0598800-01 Similar to Wall-associated kinase-like protein.
Oryza_sativa	OG0000000

Os04t0598900-01 Similar to Wall-associated kinase-like protein.
Oryza_sativa OG0000000
Os04t0599000-01 Similar to WAK53a - OsWAK receptor-like protein kinase. Oryza_sativa OG0000000
Os01t0117200-01 Similar to ARK protein (Fragment). Oryza_sativa OG0000000
Os04t0608900-01 Similar to Serine/thronine protein kinase-like protein. Oryza_sativa OG0000000
Os04t0610900-01 Similar to EDR1. Oryza_sativa OG0000000
Os04t0616200-01 Similar to H0525G02.9 protein. Oryza_sativa OG0000000
Os04t0616300-01 Similar to H0525G02.10 protein. Oryza_sativa OG0000000
Os04t0616500-01 Similar to H0525G02.11 protein. Oryza_sativa OG0000000
Os04t0616600-01 Serine/threonine protein kinase-related domain containing protein. Oryza_sativa OG0000000
Os04t0619400-01 Protein kinase, core domain containing protein.
Oryza_sativa OG0000000
Os04t0619600-01 Similar to Resistance protein candidate (Fragment).
Oryza_sativa OG0000000
Os01t0117300-00 Similar to Receptor kinase LRK10. Oryza_sativa OG0000000
Os04t0631800-00 Similar to H0105C05.10 protein. Oryza_sativa OG0000000
Os04t0632100-01 S-locus receptor-like kinase RLK13.
Oryza_sativa OG0000000
Os04t0632500-00 Protein kinase, catalytic domain domain containing protein. Oryza_sativa OG0000000
Os04t0632901-00 Similar to H0613A10.1 protein. Oryza_sativa OG0000000
Os04t0633200-00 Similar to H0613A10.1 protein. Oryza_sativa OG0000000
Os04t0633300-00 Similar to H0613A10.4 protein. Oryza_sativa OG0000000
Os04t0633600-00 Similar to H0613A10.6 protein. Oryza_sativa OG0000000
Os04t0633800-00 Protein kinase, catalytic domain domain containing protein. Oryza_sativa OG0000000
Os04t0633900-00 Similar to H0315F07.1 protein. Oryza_sativa OG0000000
Os04t0634000-01 Serine/threonine protein kinase-related domain containing protein. Oryza_sativa OG0000000
Os04t0634400-00 Similar to H0315F07.5 protein. Oryza_sativa OG0000000
Os01t0117400-00 Similar to Receptor-like kinase. Oryza_sativa OG0000000
Os04t0651500-01 Similar to OSIGBa0113E10.9 protein.
Oryza_sativa OG0000000
Os04t0654600-01 Protein kinase, core domain containing protein.
Oryza_sativa OG0000000
Os04t0654800-00 Similar to OSIGBa0147J19.7 protein.
Oryza_sativa OG0000000
Os04t0655000-00 Curculin-like (mannose-binding) lectin domain

containing protein.	Oryza_sativa	OG0000000
Os04t0655300-01 Protein kinase, core domain containing protein.
Oryza_sativa	OG0000000
Os04t0658700-00 Similar to OSIGBa0132E09-OSIGBa0108L24.8 protein.
Oryza_sativa	OG0000000
Os01t0117500-00 Similar to Receptor kinase LRK10.	Oryza_sativa
OG0000000
Os01t0117600-01 Protein kinase, catalytic domain domain containing
protein.	Oryza_sativa	OG0000000
Os04t0679200-01 Similar to H0801D08.12 protein.	Oryza_sativa
OG0000000
Os04t0685900-01 Similar to Receptor-like protein kinase-like protein
(Fragment).	Oryza_sativa	OG0000000
Os04t0686650-00 Similar to H0723C07.13 protein.	Oryza_sativa
OG0000000
Os04t0689400-01 Protein kinase, core domain containing protein.
Oryza_sativa	OG0000000
Os01t0117700-00 Similar to LRK14.	Oryza_sativa	OG0000000
Os05t0110900-01 Similar to Protein kinase APK1B, chloroplast
precursor (EC 2.7.1.-).	Oryza_sativa	OG0000000
Os05t0125200-01 Concanavalin A-like lectin/glucanase, subgroup
domain containing protein.	Oryza_sativa	OG0000000
Os05t0125300-00 Similar to Receptor protein kinase-like protein.
Oryza_sativa	OG0000000
Os05t0125400-00 Similar to protein kinase APK1B.	Oryza_sativa
OG0000000
Os05t0127300-01 Serine/threonine protein kinase domain containing
protein.	Oryza_sativa	OG0000000
Os05t0130100-00 Hypothetical conserved gene.	Oryza_sativa
OG0000000
Os05t0135100-00 Hypothetical conserved gene.	Oryza_sativa
OG0000000
Os05t0135800-01 Ser/Thr protein kinase, Negative regulator of innate
immunity	Oryza_sativa	OG0000000
Os01t0364100-00 Similar to OSIGBa0145M07.8 protein.
Oryza_sativa	OG0000000
Os01t0364400-00 Protein kinase, catalytic domain domain containing
protein.	Oryza_sativa	OG0000000
Os05t0165900-01 Serine/threonine protein kinase-related domain
containing protein.	Oryza_sativa	OG0000000
Os05t0166300-01 Serine/threonine protein kinase-related domain
containing protein.	Oryza_sativa	OG0000000
Os01t0364800-00 Protein kinase, catalytic domain domain containing
protein.	Oryza_sativa	OG0000000
Os05t0166600-00 Serine/threonine protein kinase-related domain
containing protein.	Oryza_sativa	OG0000000
Os01t0365000-00 Protein kinase, catalytic domain domain containing
protein.	Oryza_sativa	OG0000000
Os01t0366300-01 Similar to Receptor protein kinase.
Oryza_sativa	OG0000000
Os05t0207700-01 Similar to Serine/threonine-protein kinase PBS1 (EC
2.7.1.37) (AvrPphB susceptible protein 1).	Oryza_sativa
OG0000000
Os05t0218400-01 Similar to Somatic embryogenesis receptor kinase-

like protein.   Oryza_sativa   OG0000000
Os05t0231100-00 Hypothetical conserved gene.   Oryza_sativa
OG0000000
Os05t0246600-01 Leucine-rich repeat domain containing protein.
Oryza_sativa   OG0000000
Os05t0256500-00 Protein kinase, catalytic domain domain containing
protein. Oryza_sativa   OG0000000
Os05t0258400-00 Protein kinase, catalytic domain domain containing
protein. Oryza_sativa   OG0000000
Os05t0263100-00 Similar to SHR5-receptor-like kinase (Fragment).
Oryza_sativa   OG0000000
Os05t0280700-01 Similar to Resistance protein candidate (Fragment).
Oryza_sativa   OG0000000
Os05t0305900-01 Protein kinase, core domain containing protein.
Oryza_sativa   OG0000000
Os05t0317700-01 Similar to Resistance protein candidate (Fragment).
Oryza_sativa   OG0000000
Os05t0317900-01 Similar to Resistance protein candidate (Fragment).
Oryza_sativa   OG0000000
Os05t0318100-00 Similar to Protein kinase-related protein.
Oryza_sativa   OG0000000
Os05t0318700-01 Similar to Resistance protein candidate (Fragment).
Oryza_sativa   OG0000000
Os05t0319700-01 Similar to Protein kinase-like protein (Fragment).
Oryza_sativa   OG0000000
Os01t0384300-00 Similar to RKF3 (RECEPTOR-LIKE KINASE IN IN FLOWERS
3); kinase.   Oryza_sativa   OG0000000
Os05t0372100-01 Similar to protein kinase family protein.
Oryza_sativa   OG0000000
Os05t0387700-01 Protein kinase, core domain containing protein.
Oryza_sativa   OG0000000
Os05t0393100-01 Leucine-rich repeat, N-terminal domain containing
protein. Oryza_sativa   OG0000000
Os05t0398800-01 Similar to Serine/threonine-protein kinase PBS1 (EC
2.7.1.37) (AvrPphB susceptible protein 1). Oryza_sativa
OG0000000
Os05t0423500-01 Protein kinase, core domain containing protein.
Oryza_sativa   OG0000000
Os05t0436100-00 Similar to predicted protein.   Oryza_sativa
OG0000000
Os05t0463000-01 Similar to Receptor protein kinase-like protein.
Oryza_sativa   OG0000000
Os05t0481100-01 Similar to cDNA clone:J013034D11, full insert
sequence.   Oryza_sativa   OG0000000
Os05t0486100-01 Protein kinase, core domain containing protein.
Oryza_sativa   OG0000000
Os05t0493100-01 Similar to KI domain interacting kinase 1.
Oryza_sativa   OG0000000
Os05t0498900-01 Serine/threonine protein kinase domain containing
protein. Oryza_sativa   OG0000000
Os05t0501400-01 Similar to Receptor-like protein kinase 5.
Oryza_sativa   OG0000000
Os05t0516400-01 Similar to Hydroxyproline-rich glycoprotein DZ-HRGP
precursor.   Oryza_sativa   OG0000000

Os05t0524500-01 Protein kinase, core domain containing protein.
Oryza_sativa OG0000000
Os05t0525600-00 Protein kinase, catalytic domain domain containing protein. Oryza_sativa OG0000000
Os05t0550700-00 Protein kinase, catalytic domain domain containing protein. Oryza_sativa OG0000000
Os05t0550800-01 Serine/threonine protein kinase domain containing protein. Oryza_sativa OG0000000
Os05t0577700-01 Similar to ATP binding protein. Oryza_sativa
OG0000000
Os05t0585800-01 Protein of unknown function DUF1221 domain containing protein. Oryza_sativa OG0000000
Os05t0588300-01 Serine/threonine protein kinase-related domain containing protein. Oryza_sativa OG0000000
Os05t0589700-01 Similar to cDNA, clone Oryza_sativa
OG0000000
Os05t0591800-00 Protein kinase, catalytic domain domain containing protein. Oryza_sativa OG0000000
Os06t0126250-00 Similar to predicted protein. Oryza_sativa
OG0000000
Os06t0140800-01 Protein kinase, core domain containing protein.
Oryza_sativa OG0000000
Os06t0142500-00 Similar to Wall-associated kinase 3.
Oryza_sativa OG0000000
Os06t0159600-01 Tetratricopeptide-like helical domain containing protein. Oryza_sativa OG0000000
Os06t0164900-00 Similar to OSIGBa0147J19.7 protein.
Oryza_sativa OG0000000
Os06t0165500-00 Similar to OSIGBa0147J19.7 protein.
Oryza_sativa OG0000000
Os06t0166900-01 Protein kinase, core domain containing protein.
Oryza_sativa OG0000000
Os06t0168800-01 Similar to Protein kinase. Oryza_sativa
OG0000000
Os06t0170100-00 Similar to WAK80 - OsWAK receptor-like protein kinase. Oryza_sativa OG0000000
Os06t0181200-01 Serine/threonine protein kinase domain containing protein. Oryza_sativa OG0000000
Os06t0202900-01 Serine/threonine protein kinase domain containing protein. Oryza_sativa OG0000000
Os06t0210400-01 Concanavalin A-like lectin/glucanase, subgroup domain containing protein. Oryza_sativa OG0000000
Os01t0545500-01 Hypothetical conserved gene. Oryza_sativa
OG0000000
Os06t0225300-01 Similar to SERK1 (Fragment). Oryza_sativa
OG0000000
Os06t0241100-01 Protein kinase, core domain containing protein.
Oryza_sativa OG0000000
Os01t0548600-01 Protein kinase, catalytic domain domain containing protein. Oryza_sativa OG0000000
Os06t0274300-01 Similar to BRASSINOSTEROID INSENSITIVE 1-associated receptor kinase 1. Oryza_sativa OG0000000
Os06t0274500-01 Similar to SERK1 (Fragment). Oryza_sativa
OG0000000

Os06t0283300-01 Similar to nodulation receptor kinase.
Oryza_sativa OG0000000
Os06t0285400-01 Similar to Serine/threonine-specific kinase like protein. Oryza_sativa OG0000000
Os06t0328400-01 Protein kinase, catalytic domain domain containing protein. Oryza_sativa OG0000000
Os06t0334300-01 Similar to Resistance protein candidate (Fragment).
Oryza_sativa OG0000000
Os06t0486000-01 Protein kinase, core domain containing protein.
Oryza_sativa OG0000000
Os06t0488050-00 Similar to Protein kinase family protein.
Oryza_sativa OG0000000
Os06t0494100-01 Serine/threonine protein kinase-related domain containing protein. Oryza_sativa OG0000000
Os06t0496800-01 Similar to S-locus receptor kinase precursor.
Oryza_sativa OG0000000
Os06t0574200-01 Serine/threonine protein kinase-related domain containing protein. Oryza_sativa OG0000000
Os06t0574550-01 Protein kinase, catalytic domain domain containing protein. Oryza_sativa OG0000000
Os06t0575000-00 Similar to D-mannose binding lectin family protein.
Oryza_sativa OG0000000
Os06t0575400-01 Serine/threonine protein kinase-related domain containing protein. Oryza_sativa OG0000000
Os01t0568800-00 Protein kinase, catalytic domain domain containing protein. Oryza_sativa OG0000000
Os06t0602500-01 Serine/threonine protein kinase-related domain containing protein. Oryza_sativa OG0000000
Os06t0619600-00 Hypothetical conserved gene. Oryza_sativa
OG0000000
Os06t0620200-01 Protein kinase, catalytic domain domain containing protein. Oryza_sativa OG0000000
Os06t0636600-01 Protein kinase, core domain containing protein.
Oryza_sativa OG0000000
Os06t0646400-01 Tyrosine protein kinase domain containing protein.
Oryza_sativa OG0000000
Os06t0663200-01 Similar to Protein kinase APK1B, chloroplast precursor (EC 2.7.1.-). Oryza_sativa OG0000000
Os06t0663400-01 Serine/thronine protein kinase-like protein.
Oryza_sativa OG0000000
Os06t0676600-01 Protein kinase, core domain containing protein.
Oryza_sativa OG0000000
Os01t0581400-01 Serine/threonine protein kinase-related domain containing protein. Oryza_sativa OG0000000
Os06t0693000-01 Protein kinase, core domain containing protein.
Oryza_sativa OG0000000
Os06t0693200-01 Protein kinase, core domain containing protein.
Oryza_sativa OG0000000
Os06t0693900-00 Protein kinase, catalytic domain domain containing protein. Oryza_sativa OG0000000
Os06t0703000-01 Protein kinase, core domain containing protein.
Oryza_sativa OG0000000
Os06t0705200-00 Hypothetical conserved gene. Oryza_sativa
OG0000000

Os06t0706600-00 Similar to wall-associated kinase 3.
Oryza_sativa OG0000000
Os06t0714900-01 Serine/threonine protein kinase domain containing protein. Oryza_sativa OG0000000
Os06t0724900-01 Raf-Like MAPKKK, Mechanical tissue formation at the leaf lamina joint Oryza_sativa OG0000000
Os06t0727400-01 Similar to Protein kinase APK1A, chloroplast precursor (EC 2.7.1.-). Oryza_sativa OG0000000
Os01t0587400-01 Serine/threonine protein kinase-related domain containing protein. Oryza_sativa OG0000000
Os01t0588500-01 Similar to Avr9/Cf-9 induced kinase 1.
Oryza_sativa OG0000000
Os07t0129800-00 Similar to lectin-like receptor kinase 7.
Oryza_sativa OG0000000
Os07t0129900-00 Similar to lectin-like receptor kinase 7.
Oryza_sativa OG0000000
Os07t0130100-01 Similar to Resistance protein candidate (Fragment).
Oryza_sativa OG0000000
Os07t0130200-01 Similar to Resistance protein candidate (Fragment).
Oryza_sativa OG0000000
Os07t0130400-01 Similar to Lectin-like receptor kinase 7;2.
Oryza_sativa OG0000000
Os07t0130800-01 Protein kinase, catalytic domain domain containing protein. Oryza_sativa OG0000000
Os07t0131375-00 Protein kinase, catalytic domain domain containing protein. Oryza_sativa OG0000000
Os07t0131500-00 Similar to lectin-like receptor kinase 7.
Oryza_sativa OG0000000
Os07t0132500-01 Similar to Resistance protein candidate (Fragment).
Oryza_sativa OG0000000
Os07t0133000-01 Similar to lectin-like receptor kinase 7.
Oryza_sativa OG0000000
Os07t0133100-01 Concanavalin A-like lectin/glucanase, subgroup domain containing protein. Oryza_sativa OG0000000
Os07t0137800-00 Protein kinase, core domain containing protein.
Oryza_sativa OG0000000
Os07t0141100-01 Protein kinase, core domain containing protein.
Oryza_sativa OG0000000
Os07t0147600-01 Protein kinase, core domain containing protein.
Oryza_sativa OG0000000
Os07t0169300-00 EGF-type aspartate/asparagine hydroxylation site domain containing protein. Oryza_sativa OG0000000
Os07t0186200-01 S-domain recepter-like kinase, Abiotic stress tolerance Oryza_sativa OG0000000
Os07t0203925-00 Similar to ATP binding protein. Oryza_sativa OG0000000
Os07t0227300-00 Protein kinase, catalytic domain domain containing protein. Oryza_sativa OG0000000
Os01t0601200-01 Similar to Protein kinase family protein.
Oryza_sativa OG0000000
Os07t0248600-00 Similar to Protein kinase domain containing protein, expressed. Oryza_sativa OG0000000
Os07t0262600-00 Legume lectin, beta chain domain containing protein.
Oryza_sativa OG0000000

Os07t0262800-01 Similar to Resistance protein candidate (Fragment). Oryza_sativa OG0000000
Os01t0602800-01 Serine/threonine protein kinase-related domain containing protein. Oryza_sativa OG0000000
Os07t0283050-00 Concanavalin A-like lectin/glucanase, subgroup domain containing protein. Oryza_sativa OG0000000
Os07t0283125-00 Similar to lectin-like receptor kinase 7. Oryza_sativa OG0000000
Os07t0301500-00 Protein kinase, catalytic domain domain containing protein. Oryza_sativa OG0000000
Os07t0475900-01 Similar to kinase family protein. Oryza_sativa OG0000000
Os07t0487300-00 Protein kinase, catalytic domain domain containing protein. Oryza_sativa OG0000000
Os07t0488400-00 Protein kinase, catalytic domain domain containing protein. Oryza_sativa OG0000000
Os07t0493200-01 Protein kinase, catalytic domain domain containing protein. Oryza_sativa OG0000000
Os07t0493800-00 Protein kinase, catalytic domain domain containing protein. Oryza_sativa OG0000000
Os07t0494300-00 Protein kinase, catalytic domain domain containing protein. Oryza_sativa OG0000000
Os07t0494800-01 Serine/threonine protein kinase domain containing protein. Oryza_sativa OG0000000
Os07t0495150-00 Similar to Ser/thr protein kinase. Oryza_sativa OG0000000
Os07t0501800-01 Similar to Receptor-like protein kinase. Oryza_sativa OG0000000
Os07t0534100-00 Protein kinase, catalytic domain domain containing protein. Oryza_sativa OG0000000
Os07t0534300-01 Hypothetical conserved gene. Oryza_sativa OG0000000
Os07t0534700-01 Serine/threonine protein kinase-related domain containing protein. Oryza_sativa OG0000000
Os07t0535800-01 Similar to SRK15 protein (Fragment). Oryza_sativa OG0000000
Os07t0537000-01 Similar to Receptor protein kinase. Oryza_sativa OG0000000
Os07t0537500-01 Serine/threonine protein kinase-related domain containing protein. Oryza_sativa OG0000000
Os07t0537600-01 Hypothetical conserved gene. Oryza_sativa OG0000000
Os07t0537900-01 Similar to SRK3 gene. Oryza_sativa OG0000000
Os07t0538200-01 Serine/threonine protein kinase-related domain containing protein. Oryza_sativa OG0000000
Os07t0538400-01 Similar to Receptor-like protein kinase 4. Oryza_sativa OG0000000
Os07t0538700-01 Hypothetical conserved gene. Oryza_sativa OG0000000
Os07t0539700-01 Hypothetical conserved gene. Oryza_sativa OG0000000
Os07t0540100-01 Serine/threonine protein kinase-related domain containing protein. Oryza_sativa OG0000000

Os07t0540800-00 Protein kinase, catalytic domain domain containing protein. Oryza_sativa OG0000000
Os07t0541000-01 Similar to Receptor protein kinase. Oryza_sativa OG0000000
Os07t0541200-00 Similar to receptor-like serine-threonine protein kinase. Oryza_sativa OG0000000
Os07t0541400-01 Similar to Receptor protein kinase. Oryza_sativa OG0000000
Os07t0541500-01 Similar to KI domain interacting kinase 1. Oryza_sativa OG0000000
Os07t0541800-01 Similar to KI domain interacting kinase 1. Oryza_sativa OG0000000
Os07t0541900-01 Similar to KI domain interacting kinase 1. Oryza_sativa OG0000000
Os07t0542400-01 Similar to Receptor protein kinase. Oryza_sativa OG0000000
Os07t0542600-01 Similar to receptor-like protein kinase RK20-1. Oryza_sativa OG0000000
Os07t0550500-01 Hypothetical conserved gene. Oryza_sativa OG0000000
Os07t0550900-01 Similar to Receptor-like protein kinase 6. Oryza_sativa OG0000000
Os07t0551300-01 Similar to KI domain interacting kinase 1. Oryza_sativa OG0000000
Os07t0553633-01 Serine/threonine protein kinase-related domain containing protein. Oryza_sativa OG0000000
Os07t0568100-01 LRR protein kinase, Common symbiosis signaling (SYM) pathway Oryza_sativa OG0000000
Os07t0569550-00 Similar to protein kinase. Oryza_sativa OG0000000
Os07t0569800-01 Similar to protein kinase. Oryza_sativa OG0000000
Os07t0575600-01 Similar to Lectin receptor-type kinase. Oryza_sativa OG0000000
Os07t0575700-01 Similar to Lectin-like receptor kinase 7;2. Oryza_sativa OG0000000
Os07t0575750-00 Similar to Lectin receptor-type kinase. Oryza_sativa OG0000000
Os07t0613500-01 Similar to Protein kinase APK1B, chloroplast precursor (EC 2.7.1.-). Oryza_sativa OG0000000
Os07t0618400-01 Similar to Leucine-rich repeat transmembrane protein kinase 1 (Fragment). Oryza_sativa OG0000000
Os01t0621600-01 Serine/threonine protein kinase-related domain containing protein. Oryza_sativa OG0000000
Os07t0628700-01 Similar to Receptor protein kinase. Oryza_sativa OG0000000
Os07t0628900-01 Similar to KI domain interacting kinase 1. Oryza_sativa OG0000000
Os07t0668900-01 Similar to Serine/threonine-protein kinase PBS1 (EC 2.7.1.37) (AvrPphB susceptible protein 1). Oryza_sativa OG0000000
Os07t0686800-01 Similar to Serine/threonine protein kinase-like. Oryza_sativa OG0000000
Os07t0695300-01 Similar to Serine/threonine-protein kinase PBS1 (EC

2.7.1.37) (AvrPphB susceptible protein 1). Oryza_sativa
OG0000000
Os08t0109800-01 Regulator of chromosome condensation/beta-lactamase-inhibitor protein II domain containing protein. Oryza_sativa
OG0000000
Os01t0631700-01 Similar to Ser Thr specific protein kinase-like protein. Oryza_sativa OG0000000
Os08t0123900-00 Similar to cDNA, clone Oryza_sativa
OG0000000
Os08t0124000-01 Similar to Resistance protein candidate (Fragment). Oryza_sativa OG0000000
Os08t0124100-01 Concanavalin A-like lectin/glucanase, subgroup domain containing protein. Oryza_sativa OG0000000
Os08t0124500-01 Similar to Resistance protein candidate (Fragment). Oryza_sativa OG0000000
Os08t0124600-00 Similar to J065032N17, full insert sequence. Oryza_sativa OG0000000
Os08t0124700-01 Similar to Resistance protein candidate (Fragment). Oryza_sativa OG0000000
Os08t0125200-01 Similar to cDNA, clone Oryza_sativa
OG0000000
Os08t0125800-01 Hypothetical conserved gene. Oryza_sativa
OG0000000
Os08t0138700-01 Serine/threonine protein kinase-related domain containing protein. Oryza_sativa OG0000000
Os08t0174700-01 Similar to SERK1 (Fragment). Oryza_sativa
OG0000000
Os08t0176200-01 Serine/threonine protein kinase-related domain containing protein. Oryza_sativa OG0000000
Os08t0179000-01 Protein kinase, catalytic domain domain containing protein. Oryza_sativa OG0000000
Os01t0641000-01 Similar to Protein kinase. Oryza_sativa
OG0000000
Os08t0200500-01 Serine/threonine protein kinase domain containing protein. Oryza_sativa OG0000000
Os08t0201700-00 Protein kinase-like domain domain containing protein. Oryza_sativa OG0000000
Os08t0203201-00 Similar to SHR5-receptor-like kinase (Fragment). Oryza_sativa OG0000000
Os08t0203400-01 Protein kinase, core domain containing protein. Oryza_sativa OG0000000
Os08t0203700-01 Protein kinase, core domain containing protein. Oryza_sativa OG0000000
Os08t0224100-01 Similar to Serine/thronine protein kinase-like protein. Oryza_sativa OG0000000
Os08t0249100-01 Serine/threonine protein kinase-related domain containing protein. Oryza_sativa OG0000000
Os08t0285600-01 Similar to phytosulfokine receptor. Oryza_sativa OG0000000
Os01t0648600-00 Protein kinase, catalytic domain domain containing protein. Oryza_sativa OG0000000
Os08t0334300-00 Legume lectin, beta chain domain containing protein. Oryza_sativa OG0000000
Os08t0335500-01 Hypothetical conserved gene. Oryza_sativa

OG0000000
Os08t0343000-01 Protein kinase, core domain containing protein.
Oryza_sativa OG0000000
Os08t0365500-00 Similar to WAK80 - OsWAK receptor-like protein kinase. Oryza_sativa OG0000000
Os08t0365800-00 Hypothetical conserved gene. Oryza_sativa
OG0000000
Os08t0378300-00 Similar to cDNA clone:J013100G02, full insert sequence. Oryza_sativa OG0000000
Os08t0442700-01 Similar to SERK1 (Fragment). Oryza_sativa
OG0000000
Os08t0457400-01 Similar to Avr9/Cf-9 induced kinase 1.
Oryza_sativa OG0000000
Os08t0501000-00 Hypothetical conserved gene. Oryza_sativa
OG0000000
Os08t0501200-00 EGF-type aspartate/asparagine hydroxylation site domain containing protein. Oryza_sativa OG0000000
Os08t0501500-00 EGF-type aspartate/asparagine hydroxylation site domain containing protein. Oryza_sativa OG0000000
Os08t0501600-00 Similar to Wall associated kinase. Oryza_sativa
OG0000000
Os08t0514000-00 Protein kinase, catalytic domain domain containing protein. Oryza_sativa OG0000000
Os08t0514100-01 Legume lectin, beta chain domain containing protein.
Oryza_sativa OG0000000
Os01t0664200-01 Similar to Ser Thr specific protein kinase-like protein. Oryza_sativa OG0000000
Os08t0538300-01 LysM receptor-like kinase, Chitin elicitor signaling
Oryza_sativa OG0000000
Os01t0668400-00 Similar to predicted protein. Oryza_sativa
OG0000000
Os01t0668600-00 Curculin-like (mannose-binding) lectin domain containing protein. Oryza_sativa OG0000000
Os01t0668901-00 Protein kinase, catalytic domain domain containing protein. Oryza_sativa OG0000000
Os01t0669100-01 S-domain receptor-like kinase, OsPR1b-interacting factor, Resistance protein, Grain yield, Disease resistance
Oryza_sativa OG0000000
Os01t0670100-01 Serine/threonine protein kinase-related domain containing protein. Oryza_sativa OG0000000
Os09t0123300-01 Similar to Calmodulin-binding receptor-like kinase.
Oryza_sativa OG0000000
Os01t0670600-01 Protein kinase, catalytic domain domain containing protein. Oryza_sativa OG0000000
Os09t0251100-00 Protein kinase, catalytic domain domain containing protein. Oryza_sativa OG0000000
Os09t0268000-00 Similar to lectin-like receptor kinase 7.
Oryza_sativa OG0000000
Os09t0268100-00 Similar to lectin-like receptor kinase 7.
Oryza_sativa OG0000000
Os09t0314800-00 Similar to Receptor protein kinase PERK1.
Oryza_sativa OG0000000
Os09t0334800-01 Similar to protein kinase. Oryza_sativa
OG0000000

Os09t0339000-01 Protein kinase, core domain containing protein.
Oryza_sativa OG0000000
Os09t0341100-00 Similar to protein kinase. Oryza_sativa
OG0000000
Os09t0348400-00 Protein kinase, catalytic domain domain containing protein. Oryza_sativa OG0000000
Os09t0348600-00 Protein kinase, catalytic domain domain containing protein. Oryza_sativa OG0000000
Os09t0349100-00 Protein kinase, catalytic domain domain containing protein. Oryza_sativa OG0000000
Os09t0349800-00 Similar to OsD305. Oryza_sativa
OG0000000
Os09t0350900-01 Protein kinase, core domain containing protein.
Oryza_sativa OG0000000
Os09t0351700-00 Protein kinase, catalytic domain domain containing protein. Oryza_sativa OG0000000
Os09t0355400-01 Protein kinase, core domain containing protein.
Oryza_sativa OG0000000
Os09t0356000-00 Similar to OsD305. Oryza_sativa
OG0000000
Os09t0356800-01 Protein kinase, core domain containing protein.
Oryza_sativa OG0000000
Os09t0358000-00 Similar to OsD305. Oryza_sativa
OG0000000
Os09t0359500-01 Similar to OsD305. Oryza_sativa
OG0000000
Os09t0361100-01 Similar to Protein kinase. Oryza_sativa
OG0000000
Os09t0373800-00 Protein kinase, catalytic domain domain containing protein. Oryza_sativa OG0000000
Os09t0375600-01 Similar to Protein kinase family protein.
Oryza_sativa OG0000000
Os09t0400500-01 Serine/threonine protein kinase domain containing protein. Oryza_sativa OG0000000
Os09t0408900-00 Similar to kinase family protein. Oryza_sativa
OG0000000
Os09t0442100-01 Protein kinase, core domain containing protein.
Oryza_sativa OG0000000
Os09t0454900-01 Serine/threonine protein kinase-related domain containing protein. Oryza_sativa OG0000000
Os09t0458300-01 Leucine-rich repeat, N-terminal domain containing protein. Oryza_sativa OG0000000
Os09t0471200-00 Similar to WAK80 - OsWAK receptor-like protein kinase. Oryza_sativa OG0000000
Os09t0471400-01 Similar to WAK80 - OsWAK receptor-like protein kinase. Oryza_sativa OG0000000
Os09t0471550-01 EGF-like calcium-binding domain containing protein.
Oryza_sativa OG0000000
Os09t0471600-01 EGF-like calcium-binding domain containing protein.
Oryza_sativa OG0000000
Os09t0471800-01 Similar to WAK80 - OsWAK receptor-like protein kinase. Oryza_sativa OG0000000
Os09t0482640-01 Serine/threonine protein kinase-related domain containing protein. Oryza_sativa OG0000000

Os01t0690800-01 Protein kinase, core domain containing protein.
Oryza_sativa OG0000000
Os01t0691050-00 Protein kinase, catalytic domain domain containing protein. Oryza_sativa OG0000000
Os09t0511000-01 Similar to LysM receptor-like kinase.
Oryza_sativa OG0000000
Os09t0513600-01 Similar to predicted protein. Oryza_sativa
OG0000000
Os09t0531701-00 Similar to BRASSINOSTEROID INSENSITIVE 1-associated receptor kinase 1. Oryza_sativa OG0000000
Os09t0533600-01 Similar to Avr9/Cf-9 induced kinase 1.
Oryza_sativa OG0000000
Os09t0544300-01 ATMRK serine/threonine protein kinase-like domain containing protein. Oryza_sativa OG0000000
Os09t0550600-00 Similar to S-locus receptor-like kinase RLK14.
Oryza_sativa OG0000000
Os09t0551201-00 Hypothetical conserved gene. Oryza_sativa
OG0000000
Os09t0551251-00 Similar to H0105C05.10 protein. Oryza_sativa
OG0000000
Os09t0551400-00 Similar to S-locus receptor-like kinase RLK14.
Oryza_sativa OG0000000
Os09t0551500-00 Similar to S-locus receptor-like kinase RLK14.
Oryza_sativa OG0000000
Os09t0559900-01 Protein kinase, core domain containing protein.
Oryza_sativa OG0000000
Os09t0561000-01 Similar to Wall-associated kinase-like 2.
Oryza_sativa OG0000000
Os09t0561450-00 EGF-type aspartate/asparagine hydroxylation site domain containing protein. Oryza_sativa OG0000000
Os09t0561500-01 Wall-associated kinase, Rice blast resistance
Oryza_sativa OG0000000
Os09t0561600-01 Wall-associated receptor-like kinase, Regulation of early embryo sac development, Female gametophyte development, Positive regulation of rice blast resistance Oryza_sativa
OG0000000
Os09t0562600-01 Wall-associated kinase, Positive regulation of rice blast resistance Oryza_sativa OG0000000
Os09t0566550-01 Similar to serine/threonine protein kinase 1, CTR1.
Oryza_sativa OG0000000
Os09t0569800-00 Serine/threonine protein kinase-related domain containing protein. Oryza_sativa OG0000000
Os09t0570000-01 Serine/threonine protein kinase-related domain containing protein. Oryza_sativa OG0000000
Os09t0570100-01 Protein kinase, catalytic domain domain containing protein. Oryza_sativa OG0000000
Os09t0572600-01 Similar to Receptor protein kinase-like protein.
Oryza_sativa OG0000000
Os10t0100500-01 Serine/threonine protein kinase-related domain containing protein. Oryza_sativa OG0000000
Os10t0101000-01 Serine/threonine protein kinase domain containing protein. Oryza_sativa OG0000000
Os10t0103140-00 Similar to Protein kinase domain containing protein, expressed. Oryza_sativa OG0000000

Os10t0104800-01 Protein kinase, core domain containing protein.
Oryza_sativa OG0000000
Os10t0111400-00 Similar to Protein kinase domain containing protein, expressed. Oryza_sativa OG0000000
Os10t0111900-00 Similar to Protein kinase domain containing protein, expressed. Oryza_sativa OG0000000
Os10t0112700-01 Serine/threonine protein kinase-related domain containing protein. Oryza_sativa OG0000000
Os10t0116600-00 Serine/threonine protein kinase domain containing protein. Oryza_sativa OG0000000
Os10t0116701-00 Hypothetical conserved gene. Oryza_sativa OG0000000
Os10t0129200-00 Similar to OSIGBa0140L04.2 protein.
Oryza_sativa OG0000000
Os01t0136400-01 Protein kinase, core domain containing protein.
Oryza_sativa OG0000000
Os10t0136400-00 Similar to Protein kinase domain containing protein, expressed. Oryza_sativa OG0000000
Os10t0136500-01 Similar to SRK5 protein (Fragment).
Oryza_sativa OG0000000
Os10t0142600-00 Protein kinase, catalytic domain domain containing protein. Oryza_sativa OG0000000
Os10t0143866-00 Protein kinase, catalytic domain domain containing protein. Oryza_sativa OG0000000
Os10t0151100-00 Similar to Protein kinase domain containing protein, expressed. Oryza_sativa OG0000000
Os10t0151500-00 Similar to Protein kinase domain containing protein, expressed. Oryza_sativa OG0000000
Os10t0157200-00 Similar to Protein kinase domain containing protein. Oryza_sativa OG0000000
Os10t0162840-00 EGF-like calcium-binding domain containing protein.
Oryza_sativa OG0000000
Os10t0162844-00 Wall-associated kinase domain containing protein.
Oryza_sativa OG0000000
Os10t0174548-00 Similar to Calcium binding EGF domain containing protein. Oryza_sativa OG0000000
Os10t0174800-00 Similar to Protein kinase domain containing protein. Oryza_sativa OG0000000
Os10t0180800-01 Wall-associated kinase, Negative regulation of rice blast resistance Oryza_sativa OG0000000
Os10t0184871-00 Similar to Protein kinase domain containing protein, expressed. Oryza_sativa OG0000000
Os10t0200000-01 Protein kinase, core domain containing protein.
Oryza_sativa OG0000000
Os10t0326100-00 Similar to Protein kinase domain containing protein, expressed. Oryza_sativa OG0000000
Os10t0326200-00 Similar to Protein kinase domain containing protein. Oryza_sativa OG0000000
Os10t0326900-00 Similar to Protein kinase domain containing protein. Oryza_sativa OG0000000
Os10t0327000-00 Similar to Protein kinase domain containing protein. Oryza_sativa OG0000000
Os10t0342300-01 Serine/threonine protein kinase-related domain containing protein. Oryza_sativa OG0000000

Os01t0136800-01 Protein kinase, core domain containing protein.
Oryza_sativa OG0000000
Os10t0389800-01 Protein kinase, core domain containing protein.
Oryza_sativa OG0000000
Os10t0395000-01 Protein kinase, core domain containing protein.
Oryza_sativa OG0000000
Os01t0136900-00 Similar to Stress-induced receptor-like kinase 2.
Oryza_sativa OG0000000
Os10t0430900-01 Tyrosine protein kinase domain containing protein.
Oryza_sativa OG0000000
Os10t0431900-01 Serine/threonine protein kinase-related domain containing protein. Oryza_sativa OG0000000
Os10t0441900-01 Similar to Resistance protein candidate (Fragment).
Oryza_sativa OG0000000
Os10t0442000-01 Similar to Lectin-like receptor kinase 7;2.
Oryza_sativa OG0000000
Os10t0442800-01 Protein serine/threonine kinase (Protein kinase).
Oryza_sativa OG0000000
Os01t0137250-00 Similar to Stress-induced receptor-like kinase 2.
Oryza_sativa OG0000000
Os10t0497600-01 Serine/threonine protein kinase-related domain containing protein. Oryza_sativa OG0000000
Os10t0533150-00 Similar to BRASSINOSTEROID INSENSITIVE 1-associated receptor kinase 1. Oryza_sativa OG0000000
Os10t0533800-00 Concanavalin A-like lectin/glucanase, subgroup domain containing protein. Oryza_sativa OG0000000
Os10t0534500-01 Similar to Resistance protein candidate (Fragment).
Oryza_sativa OG0000000
Os01t0137400-00 Protein kinase, catalytic domain domain containing protein. Oryza_sativa OG0000000
Os10t0548300-01 Protein kinase, catalytic domain domain containing protein. Oryza_sativa OG0000000
Os10t0548700-01 Serine/threonine protein kinase-related domain containing protein. Oryza_sativa OG0000000
Os10t0548850-00 U box domain domain containing protein.
Oryza_sativa OG0000000
Os10t0552400-01 Zinc finger, RING/FYVE/PHD-type domain containing protein. Oryza_sativa OG0000000
Os01t0137500-01 Protein kinase, catalytic domain domain containing protein. Oryza_sativa OG0000000
Os10t0561500-01 Serine/threonine protein kinase-related domain containing protein. Oryza_sativa OG0000000
Os11t0108800-00 Similar to Protein kinase domain containing protein, expressed. Oryza_sativa OG0000000
Os11t0121400-01 Similar to Protein kinase APK1A, chloroplast precursor (EC 2.7.1.-). Oryza_sativa OG0000000
Os11t0133001-01 Similar to Ser/Thr protein kinase (Fragment).
Oryza_sativa OG0000000
Os11t0133100-00 Similar to D-mannose binding lectin family protein.
Oryza_sativa OG0000000
Os11t0133500-00 Serine/threonine protein kinase-related domain containing protein. Oryza_sativa OG0000000
Os11t0168600-01 Protein kinase, core domain containing protein.
Oryza_sativa OG0000000

Os11t0172350-00 Similar to Protein kinase domain containing protein.
Oryza_sativa OG0000000
Os01t0138400-01 Serine/threonine protein kinase domain containing protein. Oryza_sativa OG0000000
Os11t0194900-01 Protein kinase, core domain containing protein.
Oryza_sativa OG0000000
Os11t0212300-00 Similar to Protein kinase domain containing protein, expressed. Oryza_sativa OG0000000
Os11t0213000-01 Similar to Protein kinase domain containing protein, expressed. Oryza_sativa OG0000000
Os11t0222000-01 Regulator of chromosome condensation/beta-lactamase-inhibitor protein II domain containing protein. Oryza_sativa OG0000000
Os11t0225000-01 Serine/threonine protein kinase-related domain containing protein. Oryza_sativa OG0000000
Os11t0226201-00 Similar to Protein kinase domain containing protein, expressed. Oryza_sativa OG0000000
Os11t0245200-01 Similar to leucine-rich repeat family protein / protein kinase family protein. Oryza_sativa OG0000000
Os01t0738300-01 Protein kinase, core domain containing protein.
Oryza_sativa OG0000000
Os11t0274700-01 Hypothetical conserved gene. Oryza_sativa OG0000000
Os01t0741200-01 Similar to LysM type receptor kinase (Fragment).
Oryza_sativa OG0000000
Os11t0441900-01 Protein kinase, core domain containing protein.
Oryza_sativa OG0000000
Os11t0448000-00 Protein kinase, catalytic domain domain containing protein. Oryza_sativa OG0000000
Os11t0470500-00 Similar to Protein kinase domain containing protein, expressed. Oryza_sativa OG0000000
Os01t0747400-01 Protein kinase, core domain containing protein.
Oryza_sativa OG0000000
Os11t0549300-00 Similar to Protein kinase domain containing protein, expressed. Oryza_sativa OG0000000
Os11t0553500-00 Protein kinase, catalytic domain domain containing protein. Oryza_sativa OG0000000
Os11t0555600-01 Protein kinase, core domain containing protein.
Oryza_sativa OG0000000
Os11t0556400-00 Serine/threonine protein kinase-related domain containing protein. Oryza_sativa OG0000000
Os11t0557500-00 Protein kinase, core domain containing protein.
Oryza_sativa OG0000000
Os01t0140400-01 Similar to leucine-rich repeat protein-related.
Oryza_sativa OG0000000
Os11t0601500-00 Similar to Protein kinase domain containing protein.
Oryza_sativa OG0000000
Os01t0750600-01 Protein kinase-like domain containing protein.
Oryza_sativa OG0000000
Os11t0607200-01 Protein kinase, core domain containing protein.
Oryza_sativa OG0000000
Os11t0608700-00 Similar to Jacalin-like lectin domain containing protein. Oryza_sativa OG0000000
Os11t0616100-00 Similar to Protein kinase domain containing protein,

expressed.	Oryza_sativa	OG0000000
Os11t0644000-00 Similar to Protein kinase domain containing protein, expressed.	Oryza_sativa	OG0000000
Os11t0664100-00 Similar to Protein kinase domain containing protein.	Oryza_sativa	OG0000000
Os11t0666200-00 Similar to Protein kinase domain containing protein.	Oryza_sativa	OG0000000
Os11t0666300-00 Similar to Receptor kinase-like protein.	Oryza_sativa	OG0000000
Os11t0669200-00 Similar to Protein kinase domain containing protein, expressed.	Oryza_sativa	OG0000000
Os11t0681400-00 Similar to protein kinase.	Oryza_sativa	OG0000000
Os11t0690332-00 Similar to Protein kinase domain containing protein.	Oryza_sativa	OG0000000
Os11t0691100-00 Similar to Protein kinase.	Oryza_sativa	OG0000000
Os11t0691280-00 EGF-type aspartate/asparagine hydroxylation site domain containing protein.	Oryza_sativa	OG0000000
Os11t0691300-00 Similar to Protein kinase.	Oryza_sativa	OG0000000
Os11t0691500-00 Similar to Protein kinase domain containing protein.	Oryza_sativa	OG0000000
Os11t0691600-01 Similar to Protein kinase.	Oryza_sativa	OG0000000
Os11t0691650-00 Serine/threonine protein kinase domain containing protein.	Oryza_sativa	OG0000000
Os11t0694100-01 Similar to Protein kinase domain containing protein, expressed.	Oryza_sativa	OG0000000
Os12t0108100-01 Similar to Ser Thr specific protein kinase-like protein.	Oryza_sativa	OG0000000
Os12t0121100-01 Similar to Serine/threonine-protein kinase NAK.	Oryza_sativa	OG0000000
Os12t0130200-00 Similar to D-mannose binding lectin family protein, expressed.	Oryza_sativa	OG0000000
Os12t0130300-00 Similar to Resistance protein candidate (Fragment).	Oryza_sativa	OG0000000
Os12t0130800-00 Protein kinase-like domain containing protein.	Oryza_sativa	OG0000000
Os12t0163800-01 Similar to ATP binding protein.	Oryza_sativa	OG0000000
Os12t0180500-00 Similar to Protein kinase domain containing protein.	Oryza_sativa	OG0000000
Os12t0210400-01 Protein kinase, core domain containing protein.	Oryza_sativa	OG0000000
Os12t0266200-01 Similar to Fibrillin-2 precursor.	Oryza_sativa	OG0000000
Os01t0769700-01 Similar to Resistance protein candidate (Fragment).	Oryza_sativa	OG0000000
Os12t0427450-00 Similar to Protein kinase domain containing protein.	Oryza_sativa	OG0000000
Os12t0432000-00 Similar to Protein kinase domain containing protein.	Oryza_sativa	OG0000000
Os12t0478400-00 EGF-type aspartate/asparagine hydroxylation site

domain containing protein.	Oryza_sativa	OG0000000
Os12t0499700-00 Similar to Protein kinase domain containing protein.	Oryza_sativa	OG0000000
Os12t0527700-01 Serine/threonine protein kinase-related domain containing protein.	Oryza_sativa	OG0000000
Os12t0562500-00 Similar to Protein kinase-like protein (Fragment).	Oryza_sativa	OG0000000
Os12t0567500-00 Protein kinase, catalytic domain domain containing protein.	Oryza_sativa	OG0000000
Os12t0574700-00 Similar to lectin-like receptor kinase 7.	Oryza_sativa	OG0000000
Os01t0779300-01 Concanavalin A-like lectin/glucanase, subgroup domain containing protein.	Oryza_sativa	OG0000000
Os12t0594300-00 Hypothetical conserved gene.	Oryza_sativa	OG0000000
Os12t0595800-01 Similar to cDNA clone:J023098M23, full insert sequence.	Oryza_sativa	OG0000000
Os12t0605900-01 Similar to Kinase like protein.	Oryza_sativa	OG0000000
Os12t0606000-00 Similar to Protein kinase domain containing protein.	Oryza_sativa	OG0000000
Os12t0608500-01 Serine/threonine protein kinase-related domain containing protein.	Oryza_sativa	OG0000000
Os12t0608900-00 Protein of unknown function DUF26 domain containing protein.	Oryza_sativa	OG0000000
Os12t0614800-00 Similar to cDNA clone:002-112-E04, full insert sequence.	Oryza_sativa	OG0000000
Os12t0614851-00 Serine/threonine protein kinase domain containing protein.	Oryza_sativa	OG0000000
Os12t0615000-00 Tyrosine protein kinase domain containing protein.	Oryza_sativa	OG0000000
Os12t0615100-01 Similar to Protein kinase domain containing protein, expressed.	Oryza_sativa	OG0000000
Os12t0615300-01 Similar to Protein kinase domain containing protein, expressed.	Oryza_sativa	OG0000000
Os01t0783800-01 Serine/threonine protein kinase-related domain containing protein.	Oryza_sativa	OG0000000
Os01t0784200-00 Serine/threonine protein kinase-related domain containing protein.	Oryza_sativa	OG0000000
Os12t0638100-01 Similar to Receptor-like protein kinase.	Oryza_sativa	OG0000000
Os01t0784700-00 Serine/threonine protein kinase-related domain containing protein.	Oryza_sativa	OG0000000
Os12t0640700-01 Protein kinase-like domain containing protein.	Oryza_sativa	OG0000000
Os01t0789200-01 Similar to Serine/threonine-protein kinase PBS1 (EC 2.7.1.37) (AvrPphB susceptible protein 1).	Oryza_sativa	OG0000000
Os01t0810500-00 Hypothetical conserved gene.	Oryza_sativa	OG0000000
Os01t0810600-00 Similar to cDNA, clone	Oryza_sativa	OG0000000
Os01t0810800-01 Hypothetical conserved gene.	Oryza_sativa	OG0000000

Os01t0811001-00 Hypothetical conserved gene.	Oryza_sativa	OG0000000
Os01t0816600-01 Similar to predicted protein.	Oryza_sativa	OG0000000
Os01t0818600-01 Similar to Leucine-rich repeat transmembrane protein kinase.	Oryza_sativa	OG0000000
Os01t0819100-01 Similar to predicted protein.	Oryza_sativa	OG0000000
Os01t0870400-00 Similar to S-domain class receptor-like kinase3.	Oryza_sativa	OG0000000
Os01t0870500-01 Similar to S-domain class receptor-like kinase3.	Oryza_sativa	OG0000000
Os01t0871000-00 Similar to S-domain class receptor-like kinase3.	Oryza_sativa	OG0000000
Os01t0883000-00 Similar to protein kinase.	Oryza_sativa	OG0000000
Os01t0885700-01 Protein kinase-like domain containing protein.	Oryza_sativa	OG0000000
Os01t0889900-01 Serine/threonine protein kinase-related domain containing protein.	Oryza_sativa	OG0000000
Os01t0890100-00 Similar to S-domain class receptor-like kinase3.	Oryza_sativa	OG0000000
Os01t0890200-00 Similar to S-domain class receptor-like kinase3.	Oryza_sativa	OG0000000
Os01t0890600-00 Protein kinase, catalytic domain domain containing protein.	Oryza_sativa	OG0000000
Os01t0899000-01 Similar to Pti1 kinase-like protein.	Oryza_sativa	OG0000000
Os01t0929200-01 Serine/threonine protein kinase domain containing protein.	Oryza_sativa	OG0000000
Os01t0155200-00 Similar to H0525E10.8 protein.	Oryza_sativa	OG0000000
Os01t0155500-01 Protein kinase, catalytic domain domain containing protein.	Oryza_sativa	OG0000000
Os01t0936100-01 Similar to Protein kinase.	Oryza_sativa	OG0000000
Os01t0960400-01 Protein kinase, core domain containing protein.	Oryza_sativa	OG0000000
Os01t0973500-01 Similar to Protein kinase APK1A, chloroplast precursor (EC 2.7.1.-).	Oryza_sativa	OG0000000
Os01t0976900-00 Similar to kinase.	Oryza_sativa	OG0000000
Os02t0110600-01 Similar to protein kinase.	Oryza_sativa	OG0000000
Os02t0111600-01 Serine/threonine protein kinase-related domain containing protein.	Oryza_sativa	OG0000000
Os02t0118200-01 Similar to Protein kinase APK1B, chloroplast precursor (EC 2.7.1.-).	Oryza_sativa	OG0000000
Os02t0120100-01 ATMRK serine/threonine protein kinase-like domain containing protein.	Oryza_sativa	OG0000000
Os02t0151100-01 Hypothetical conserved gene.	Oryza_sativa	OG0000000
Os02t0152300-01 Serine/threonine protein kinase-related domain containing protein.	Oryza_sativa	OG0000000

Os02t0156000-00 Similar to H0201G08.2 protein.	Oryza_sativa	OG0000000
Os02t0165100-01 Protein kinase, core domain containing protein.	Oryza_sativa	OG0000000
Os02t0174200-01 ATMRK serine/threonine protein kinase-like domain containing protein.	Oryza_sativa	OG0000000
Os01t0171000-01 BRASSINOSTEROID INSENSITIVE 1-associated receptor kinase 1 precursor (EC 2.7.1.37) (BRI1-associated receptor kinase 1) (Somatic embryogenesis receptor-like kinase 3).	Oryza_sativa	OG0000000
Os02t0186500-01 Similar to Protein kinase-like protein.	Oryza_sativa	OG0000000
Os02t0190500-01 Serine/threonine protein kinase-related domain containing protein.	Oryza_sativa	OG0000000
Os02t0194400-01 Similar to Receptor-like kinase (Fragment).	Oryza_sativa	OG0000000
Os02t0194600-00 Protein kinase, catalytic domain domain containing protein.	Oryza_sativa	OG0000000
Os02t0218400-01 Serine/threonine protein kinase-related domain containing protein.	Oryza_sativa	OG0000000
Os02t0218600-00 Similar to Protein kinase.	Oryza_sativa	OG0000000
Os02t0220700-01 Hypothetical conserved gene.	Oryza_sativa	OG0000000
Os02t0234450-00 Hypothetical conserved gene.	Oryza_sativa	OG0000000
Os02t0236100-01 Leucine-rich repeat receptor-like kinase, Specification of root outer cell layers	Oryza_sativa	OG0000000
Os02t0241600-01 Protein kinase, core domain containing protein.	Oryza_sativa	OG0000000
Os02t0283800-01 Similar to SERK1 (Fragment).	Oryza_sativa	OG0000000
Os02t0298200-01 Similar to Resistance protein candidate (Fragment).	Oryza_sativa	OG0000000
Os02t0299000-00 Similar to lectin-like receptor kinase 7.	Oryza_sativa	OG0000000
Os02t0459600-00 Protein kinase, catalytic domain domain containing protein.	Oryza_sativa	OG0000000
Os02t0472700-00 Similar to receptor-like serine-threonine protein kinase.	Oryza_sativa	OG0000000
Os02t0513000-01 Similar to Receptor protein kinase-like protein.	Oryza_sativa	OG0000000
Os02t0527600-01 Similar to CTR1-like protein kinase.	Oryza_sativa	OG0000000
Os02t0549200-01 Similar to Ser Thr specific protein kinase-like protein.	Oryza_sativa	OG0000000
Os02t0553000-01 Protein kinase, catalytic domain domain containing protein.	Oryza_sativa	OG0000000
Os02t0565500-01 Similar to Pto kinase interactor 1.	Oryza_sativa	OG0000000
Os02t0594100-01 Similar to Protein kinase ATN1.	Oryza_sativa	OG0000000
Os01t0195200-01 Similar to Serine/threonine-protein kinase PBS1 (EC

2.7.1.37) (AvrPphB susceptible protein 1). Oryza_sativa
OG0000000
Os02t0623600-01 Similar to OSIGBa0145M07.8 protein.
Oryza_sativa OG0000000
Os02t0623932-00 Similar to WAK80 - OsWAK receptor-like protein
kinase. Oryza_sativa OG0000000
Os02t0624100-00 EGF-type aspartate/asparagine hydroxylation site
domain containing protein. Oryza_sativa OG0000000
Os02t0632100-00 Similar to WAK111 - OsWAK receptor-like protein
kinase. Oryza_sativa OG0000000
Os02t0632800-01 Wall-associated kinase, Positive regulation of rice
blast resistance Oryza_sativa OG0000000
Os02t0633066-00 Protein kinase, catalytic domain domain containing
protein. Oryza_sativa OG0000000
Os02t0639100-00 Similar to H0801D08.12 protein. Oryza_sativa
OG0000000
Os02t0640500-01 Lectin receptor-like kinase, Mediation of salt
sensitivity, Regulation of ethylene homeostasis Oryza_sativa
OG0000000
Os02t0650500-01 Similar to Protein kinase-like (Protein serine/
threonine kinase). Oryza_sativa OG0000000
Os02t0665500-01 Similar to U-box domain containing protein,
expressed. Oryza_sativa OG0000000
Os02t0670100-00 Similar to protein kinase APK1B. Oryza_sativa
OG0000000
Os02t0681700-01 Serine/threonine protein kinase-related domain
containing protein. Oryza_sativa OG0000000
Os01t0201200-01 Similar to Protein kinase. Oryza_sativa
OG0000000
Os02t0710500-01 Similar to Receptor protein kinase.
Oryza_sativa OG0000000
Os02t0712600-01 Concanavalin A-like lectin/glucanase domain
containing protein. Oryza_sativa OG0000000
Os02t0712700-01 Concanavalin A-like lectin/glucanase domain
containing protein. Oryza_sativa OG0000000
Os01t0110500-01 Protein kinase, core domain containing protein.
Oryza_sativa OG0000000
Os02t0743500-01 Similar to EDR1. Oryza_sativa OG0000000
Os02t0767400-01 Serine/threonine protein kinase-related domain
containing protein. Oryza_sativa OG0000000
Os02t0777400-01 Similar to ER (ERECTA); transmembrane receptor
protein kinase. Oryza_sativa OG0000000
Os02t0787200-01 Serine/threonine protein kinase-related domain
containing protein. Oryza_sativa OG0000000
Os02t0807200-00 Similar to Protein kinase family protein.
Oryza_sativa OG0000000
Os02t0807800-01 Hypothetical conserved gene. Oryza_sativa
OG0000000
Os02t0807900-01 Conserved hypothetical protein. Oryza_sativa
OG0000000
Os02t0808100-00 Similar to Protein kinase family protein.
Oryza_sativa OG0000000
Os02t0808300-01 EGF domain containing protein. Oryza_sativa
OG0000000

Os02t0811200-00 Protein kinase, core domain containing protein.
Oryza_sativa OG0000000
Os02t0815900-01 Protein kinase, core domain containing protein.
Oryza_sativa OG0000000
Os02t0819600-01 Serine/threonine protein kinase-related domain containing protein. Oryza_sativa OG0000000
Os02t0821400-01 Protein kinase, core domain containing protein.
Oryza_sativa OG0000000
Os02t0822900-01 Hypothetical conserved gene. Oryza_sativa
OG0000000
Os02t0833000-01 Similar to Serine/threonine-protein kinase PBS1 (EC 2.7.1.37) (AvrPphB susceptible protein 1). Oryza_sativa
OG0000000
Os03t0101200-00 Similar to U-box domain containing protein, expressed. Oryza_sativa OG0000000
Os03t0124200-01 Similar to Pto-like protein kinase F.
Oryza_sativa OG0000000
Os03t0125600-01 Ser Thr specific protein kinase-like protein.
Oryza_sativa OG0000000
Os03t0130900-01 Serine/threonine protein kinase domain containing protein. Oryza_sativa OG0000000
Os01t0223800-00 Hypothetical conserved gene. Oryza_sativa
OG0000000
Os01t0223900-01 Serine/threonine protein kinase-related domain containing protein. Oryza_sativa OG0000000
Os03t0148700-01 Similar to Calcium/calmodulin-regulated receptor-like kinase. Oryza_sativa OG0000000
Os01t0224000-00 Serine/threonine protein kinase-related domain containing protein. Oryza_sativa OG0000000
Os03t0159100-01 Similar to Protein kinase APK1B, chloroplast precursor (EC 2.7.1.-). Oryza_sativa OG0000000
Os03t0160100-01 Mitogen-activated protein kinase kinase kinase (MAPKKK), Disease resistance, Defense/stress response
Oryza_sativa OG0000000
Os03t0170400-01 Similar to serine/threonine-protein kinase NAK.
Oryza_sativa OG0000000
Os03t0179400-01 Drought-inducible receptor-like cytoplasmic kinase, Drought tolerance, Regulator of grain yield Oryza_sativa
OG0000000
Os01t0227200-01 Similar to Somatic embryogenesis receptor kinase-like protein. Oryza_sativa OG0000000
Os03t0183800-01 Similar to Leucine-rich repeat transmembrane protein kinase 1 (Fragment). Oryza_sativa OG0000000
Os03t0221700-02 Serine/threonine protein kinase-related domain containing protein. Oryza_sativa OG0000000
Os03t0225700-01 Wall-associated kinase, Biotic stress response
Oryza_sativa OG0000000
Os03t0226300-01 Similar to Pto kinase interactor 1.
Oryza_sativa OG0000000
Os03t0226901-00 Similar to C-5 cytosine-specific DNA methylase family protein, expressed. Oryza_sativa OG0000000
Os03t0227900-00 Serine/threonine protein kinase domain containing protein. Oryza_sativa OG0000000
CMR089CT pep chromosome:ASM9120v1:18:244665:245643:-1 gene:CMR089C

transcript:CMR089CT gene_biotype:protein_coding
transcript_biotype:protein_coding Cyanidioschyzon_merolae
OG0000000
CMS469CT pep chromosome:ASM9120v1:19:1178662:1181254:-1 gene:CMS469C
transcript:CMS469CT gene_biotype:protein_coding
transcript_biotype:protein_coding Cyanidioschyzon_merolae
OG0000000
CMO173CT pep chromosome:ASM9120v1:15:442486:446214:1 gene:CMO173C
transcript:CMO173CT gene_biotype:protein_coding
transcript_biotype:protein_coding Cyanidioschyzon_merolae
OG0000000
CMO051CT pep chromosome:ASM9120v1:15:141384:142371:-1 gene:CMO051C
transcript:CMO051CT gene_biotype:protein_coding
transcript_biotype:protein_coding Cyanidioschyzon_merolae
OG0000000
CMP015CT pep chromosome:ASM9120v1:16:31778:35803:1 gene:CMP015C
transcript:CMP015CT gene_biotype:protein_coding
transcript_biotype:protein_coding Cyanidioschyzon_merolae
OG0000000
CMG108CT pep chromosome:ASM9120v1:7:274540:275874:1 gene:CMG108C
transcript:CMG108CT gene_biotype:protein_coding
transcript_biotype:protein_coding Cyanidioschyzon_merolae
OG0000000
CMD015CT pep chromosome:ASM9120v1:4:44400:48741:-1 gene:CMD015C
transcript:CMD015CT gene_biotype:protein_coding
transcript_biotype:protein_coding Cyanidioschyzon_merolae
OG0000000
CMQ457CT pep chromosome:ASM9120v1:17:1179661:1182190:-1 gene:CMQ457C
transcript:CMQ457CT gene_biotype:protein_coding
transcript_biotype:protein_coding Cyanidioschyzon_merolae
OG0000000
evm.model.contig_2086.5 Porphyridium_purpureum   OG0000000
evm.model.contig_2090.17 Porphyridium_purpureum   OG0000000
evm.model.contig_2114.19 Porphyridium_purpureum   OG0000000
evm.model.contig_2171.4 Porphyridium_purpureum   OG0000000
evm.model.contig_2171.5 Porphyridium_purpureum   OG0000000
evm.model.contig_2173.1 Porphyridium_purpureum   OG0000000
evm.model.contig_2181.3 Porphyridium_purpureum   OG0000000
evm.model.contig_2184.2 Porphyridium_purpureum   OG0000000
evm.model.contig_2234.2 Porphyridium_purpureum   OG0000000
evm.model.contig_2286.14 Porphyridium_purpureum   OG0000000
evm.model.contig_2300.3 Porphyridium_purpureum   OG0000000
evm.model.contig_2301.1 Porphyridium_purpureum   OG0000000
evm.model.contig_2342.1 Porphyridium_purpureum   OG0000000
evm.model.contig_2413.1 Porphyridium_purpureum   OG0000000
evm.model.contig_2026.5 Porphyridium_purpureum   OG0000000
evm.model.contig_2439.2 Porphyridium_purpureum   OG0000000
evm.model.contig_2439.1 Porphyridium_purpureum   OG0000000
evm.model.contig_2440.1 Porphyridium_purpureum   OG0000000
evm.model.contig_2494.16 Porphyridium_purpureum   OG0000000
evm.model.contig_2538.4 Porphyridium_purpureum   OG0000000
evm.model.contig_2581.1 Porphyridium_purpureum   OG0000000
evm.model.contig_2606.1 Porphyridium_purpureum   OG0000000
evm.model.contig_2714.3 Porphyridium_purpureum   OG0000000

```
evm.model.contig_2719.1  Porphyridium_purpureum   OG0000000
evm.model.contig_3419.10 Porphyridium_purpureum   OG0000000
evm.model.contig_2033.10 Porphyridium_purpureum   OG0000000
evm.model.contig_3447.2  Porphyridium_purpureum   OG0000000
evm.model.contig_3450.22 Porphyridium_purpureum   OG0000000
evm.model.contig_3451.1  Porphyridium_purpureum   OG0000000
evm.model.contig_3478.12 Porphyridium_purpureum   OG0000000
evm.model.contig_3485.1  Porphyridium_purpureum   OG0000000
evm.model.contig_3550.1  Porphyridium_purpureum   OG0000000
evm.model.contig_3665.1  Porphyridium_purpureum   OG0000000
evm.model.contig_444.13  Porphyridium_purpureum   OG0000000
evm.model.contig_4505.2  Porphyridium_purpureum   OG0000000
evm.model.contig_4729.1  Porphyridium_purpureum   OG0000000
evm.model.contig_502.1   Porphyridium_purpureum   OG0000000
evm.model.contig_551.1   Porphyridium_purpureum   OG0000000
evm.model.contig_2059.9  Porphyridium_purpureum   OG0000000
evm.model.contig_2059.16 Porphyridium_purpureum   OG0000000
evm.model.contig_629.4   Porphyridium_purpureum   OG0000000
evm.model.contig_754.1   Porphyridium_purpureum   OG0000000
evm.model.contig_770.1   Porphyridium_purpureum   OG0000000
evm.model.contig_815.1   Porphyridium_purpureum   OG0000000
evm.model.contig_927.1   Porphyridium_purpureum   OG0000000
evm.model.contig_989.1   Porphyridium_purpureum   OG0000000
CDF32695 pep supercontig:ASM35022v2:HG001543:1:1979:-1
gene:CHC_T00008569001 transcript:CDF32695
gene_biotype:protein_coding transcript_biotype:protein_coding
description:Serine/threonine protein kinase (fragment) Endoplasmic
reticulum membrane       Chondrus_crispus OG0000000
CDF38761 pep supercontig:ASM35022v2:HG001967:295764:296951:1
gene:CHC_T00008638001 transcript:CDF38761
gene_biotype:protein_coding transcript_biotype:protein_coding
description:Serine/threonine protein kinase       Chondrus_crispus
OG0000000
CDF33041 pep supercontig:ASM35022v2:HG001631:245406:247655:1
gene:CHC_T00008949001 transcript:CDF33041
gene_biotype:protein_coding transcript_biotype:protein_coding
description:Serine/threonine protein kinase       Chondrus_crispus
OG0000000
CDF33038 pep supercontig:ASM35022v2:HG001631:128261:130840:1
gene:CHC_T00008793001 transcript:CDF33038
gene_biotype:protein_coding transcript_biotype:protein_coding
description:Serine/threonine protein kinase       Chondrus_crispus
OG0000000
CDF32887 pep supercontig:ASM35022v2:HG001593:190691:192827:1
gene:CHC_T00001680001 transcript:CDF32887
gene_biotype:protein_coding transcript_biotype:protein_coding
Chondrus_crispus OG0000000
CDF36802 pep supercontig:ASM35022v2:HG001803:52713:54917:1
gene:CHC_T00009163001 transcript:CDF36802
gene_biotype:protein_coding transcript_biotype:protein_coding
description:Serine/threonine-protein kinase       Chondrus_crispus
OG0000000
CDF40489 pep supercontig:ASM35022v2:HG002190:15168:17390:1
gene:CHC_T00008885001 transcript:CDF40489
```

gene_biotype:protein_coding transcript_biotype:protein_coding
description:Serine/threonine protein kinase Chondrus_crispus
OG0000000
CDF40784 pep supercontig:ASM35022v2:HG002242:342683:343587:-1
gene:CHC_T00009038001 transcript:CDF40784
gene_biotype:protein_coding transcript_biotype:protein_coding
description:Serine/threonine protein kinase Chondrus_crispus
OG0000000
CDF35240 pep supercontig:ASM35022v2:HG001725:87225:89678:-1
gene:CHC_T00008979001 transcript:CDF35240
gene_biotype:protein_coding transcript_biotype:protein_coding
description:Serine/threonine protein kinase Chondrus_crispus
OG0000000
CDF38243 pep supercontig:ASM35022v2:HG001906:24101:28749:1
gene:CHC_T00008623001 transcript:CDF38243
gene_biotype:protein_coding transcript_biotype:protein_coding
description:Serine/threonine protein kinase Chondrus_crispus
OG0000000
CDF40123 pep supercontig:ASM35022v2:HG002126:327420:328253:1
gene:CHC_T00006994001 transcript:CDF40123
gene_biotype:protein_coding transcript_biotype:protein_coding
Chondrus_crispus OG0000000
CDF34978 pep supercontig:ASM35022v2:HG001713:57816:60620:1
gene:CHC_T00009573001 transcript:CDF34978
gene_biotype:protein_coding transcript_biotype:protein_coding
description:Calcium-dependent Serine/threonine protein kinase
Chondrus_crispus OG0000000
CDF41159 pep supercontig:ASM35022v2:HG002310:330530:332307:-1
gene:CHC_T00008708001 transcript:CDF41159
gene_biotype:protein_coding transcript_biotype:protein_coding
description:Serine/threonine protein kinase Chondrus_crispus
OG0000000
CDF33185 pep supercontig:ASM35022v2:HG001636:172910:177047:-1
gene:CHC_T00009245001 transcript:CDF33185
gene_biotype:protein_coding transcript_biotype:protein_coding
description:Serine/threonine protein kinase Chondrus_crispus
OG0000000
CDF34034 pep supercontig:ASM35022v2:HG001669:188185:191878:-1
gene:CHC_T00008548001 transcript:CDF34034
gene_biotype:protein_coding transcript_biotype:protein_coding
description:Serine/threonine-protein kinase Chondrus_crispus
OG0000000
CDF41271 pep supercontig:ASM35022v2:HG002339:136479:137867:-1
gene:CHC_T00009536001 transcript:CDF41271
gene_biotype:protein_coding transcript_biotype:protein_coding
description:Serine/threonine protein kinase Chondrus_crispus
OG0000000
CDF39339 pep supercontig:ASM35022v2:HG002022:365842:368928:1
gene:CHC_T00009013001 transcript:CDF39339
gene_biotype:protein_coding transcript_biotype:protein_coding
description:Serine/threonine pseudokinase Chondrus_crispus
OG0000000
CDF32190 pep supercontig:ASM35022v2:HG001459:945659:946621:1
gene:CHC_T00009010001 transcript:CDF32190

gene_biotype:protein_coding transcript_biotype:protein_coding description:Cyclin-dependent kinase B2-1, CDKB2-1 Chondrus_crispus OG0000000
CDF38666 pep supercontig:ASM35022v2:HG001951:2501:3259:1 gene:CHC_T00009092001 transcript:CDF38666 gene_biotype:protein_coding transcript_biotype:protein_coding description:Serine/threonine protein kinase (fragment) Chondrus_crispus OG0000000
CDF35173 pep supercontig:ASM35022v2:HG001721:143027:146646:1 gene:CHC_T00009315001 transcript:CDF35173 gene_biotype:protein_coding transcript_biotype:protein_coding description:Serine/threonine protein kinase Chondrus_crispus OG0000000
g1406.t1 Caulerpa_lentillifera OG0000001
g2688.t1 Caulerpa_lentillifera OG0000001
g3352.t1 Caulerpa_lentillifera OG0000001
g3402.t1 Caulerpa_lentillifera OG0000001
g3756.t1 Caulerpa_lentillifera OG0000001
g4714.t1 Caulerpa_lentillifera OG0000001
g4736.t1 Caulerpa_lentillifera OG0000001
g5750.t1 Caulerpa_lentillifera OG0000001
g6210.t1 Caulerpa_lentillifera OG0000001
g6325.t1 Caulerpa_lentillifera OG0000001
g6450.t1 Caulerpa_lentillifera OG0000001
g6806.t1 Caulerpa_lentillifera OG0000001
g6814.t1 Caulerpa_lentillifera OG0000001
g7758.t1 Caulerpa_lentillifera OG0000001
g8895.t1 Caulerpa_lentillifera OG0000001
g8915.t1 Caulerpa_lentillifera OG0000001
g9178.t1 Caulerpa_lentillifera OG0000001
g967.t1 Caulerpa_lentillifera OG0000001
Cre02.g093850.t1.1 pacid=30785158 transcript=Cre02.g093850.t1.1 locus=Cre02.g093850 ID=Cre02.g093850.t1.1.v5.5 annot-version=v5.5 Chlamydomonas_reinhardtii OG0000001
Cre07.g318551.t1.1 pacid=30774539 transcript=Cre07.g318551.t1.1 locus=Cre07.g318551 ID=Cre07.g318551.t1.1.v5.5 annot-version=v5.5 Chlamydomonas_reinhardtii OG0000001
Cre02.g092150.t1.2 pacid=30785716 transcript=Cre02.g092150.t1.2 locus=Cre02.g092150 ID=Cre02.g092150.t1.2.v5.5 annot-version=v5.5 Chlamydomonas_reinhardtii OG0000001
Cre02.g115508.t1.1 pacid=30785984 transcript=Cre02.g115508.t1.1 locus=Cre02.g115508 ID=Cre02.g115508.t1.1.v5.5 annot-version=v5.5 Chlamydomonas_reinhardtii OG0000001
Cre03.g144324.t1.1 pacid=30786863 transcript=Cre03.g144324.t1.1 locus=Cre03.g144324 ID=Cre03.g144324.t1.1.v5.5 annot-version=v5.5 Chlamydomonas_reinhardtii OG0000001
Cre01.g017951.t1.1 pacid=30789190 transcript=Cre01.g017951.t1.1 locus=Cre01.g017951 ID=Cre01.g017951.t1.1.v5.5 annot-version=v5.5 Chlamydomonas_reinhardtii OG0000001
Cre01.g017550.t1.1 pacid=30789280 transcript=Cre01.g017550.t1.1 locus=Cre01.g017550 ID=Cre01.g017550.t1.1.v5.5 annot-version=v5.5 Chlamydomonas_reinhardtii OG0000001
Cre01.g054100.t1.1 pacid=30789315 transcript=Cre01.g054100.t1.1 locus=Cre01.g054100 ID=Cre01.g054100.t1.1.v5.5 annot-version=v5.5

Chlamydomonas_reinhardtii OG0000001
Cre10.g448250.t1.1 pacid=30790189 transcript=Cre10.g448250.t1.1
locus=Cre10.g448250 ID=Cre10.g448250.t1.1.v5.5 annot-version=v5.5
Chlamydomonas_reinhardtii OG0000001
Cre04.g216050.t1.1 pacid=30791135 transcript=Cre04.g216050.t1.1
locus=Cre04.g216050 ID=Cre04.g216050.t1.1.v5.5 annot-version=v5.5
Chlamydomonas_reinhardtii OG0000001
Cre04.g227600.t1.2 pacid=30791422 transcript=Cre04.g227600.t1.2
locus=Cre04.g227600 ID=Cre04.g227600.t1.2.v5.5 annot-version=v5.5
Chlamydomonas_reinhardtii OG0000001
Cre04.g231614.t1.1 pacid=30791516 transcript=Cre04.g231614.t1.1
locus=Cre04.g231614 ID=Cre04.g231614.t1.1.v5.5 annot-version=v5.5
Chlamydomonas_reinhardtii OG0000001
Cre12.g503600.t1.2 pacid=30791966 transcript=Cre12.g503600.t1.2
locus=Cre12.g503600 ID=Cre12.g503600.t1.2.v5.5 annot-version=v5.5
Chlamydomonas_reinhardtii OG0000001
Cre12.g502901.t1.1 pacid=30792638 transcript=Cre12.g502901.t1.1
locus=Cre12.g502901 ID=Cre12.g502901.t1.1.v5.5 annot-version=v5.5
Chlamydomonas_reinhardtii OG0000001
Cre12.g511950.t1.2 pacid=30793096 transcript=Cre12.g511950.t1.2
locus=Cre12.g511950 ID=Cre12.g511950.t1.2.v5.5 annot-version=v5.5
Chlamydomonas_reinhardtii OG0000001
Cre11.g467642.t1.1 pacid=30775476 transcript=Cre11.g467642.t1.1
locus=Cre11.g467642 ID=Cre11.g467642.t1.1.v5.5 annot-version=v5.5
Chlamydomonas_reinhardtii OG0000001
Cre11.g467638.t1.1 pacid=30775801 transcript=Cre11.g467638.t1.1
locus=Cre11.g467638 ID=Cre11.g467638.t1.1.v5.5 annot-version=v5.5
Chlamydomonas_reinhardtii OG0000001
Cre14.g622901.t1.1 pacid=30776777 transcript=Cre14.g622901.t1.1
locus=Cre14.g622901 ID=Cre14.g622901.t1.1.v5.5 annot-version=v5.5
Chlamydomonas_reinhardtii OG0000001
Cre16.g684800.t1.1 pacid=30777915 transcript=Cre16.g684800.t1.1
locus=Cre16.g684800 ID=Cre16.g684800.t1.1.v5.5 annot-version=v5.5
Chlamydomonas_reinhardtii OG0000001
Cre16.g682550.t1.1 pacid=30778132 transcript=Cre16.g682550.t1.1
locus=Cre16.g682550 ID=Cre16.g682550.t1.1.v5.5 annot-version=v5.5
Chlamydomonas_reinhardtii OG0000001
Cre06.g269200.t1.1 pacid=30778706 transcript=Cre06.g269200.t1.1
locus=Cre06.g269200 ID=Cre06.g269200.t1.1.v5.5 annot-version=v5.5
Chlamydomonas_reinhardtii OG0000001
Cre06.g275450.t1.1 pacid=30778920 transcript=Cre06.g275450.t1.1
locus=Cre06.g275450 ID=Cre06.g275450.t1.1.v5.5 annot-version=v5.5
Chlamydomonas_reinhardtii OG0000001
Cre06.g248900.t1.1 pacid=30779212 transcript=Cre06.g248900.t1.1
locus=Cre06.g248900 ID=Cre06.g248900.t1.1.v5.5 annot-version=v5.5
Chlamydomonas_reinhardtii OG0000001
Cre06.g311750.t1.1 pacid=30779538 transcript=Cre06.g311750.t1.1
locus=Cre06.g311750 ID=Cre06.g311750.t1.1.v5.5 annot-version=v5.5
Chlamydomonas_reinhardtii OG0000001
Cre17.g737702.t1.1 pacid=30781796 transcript=Cre17.g737702.t1.1
locus=Cre17.g737702 ID=Cre17.g737702.t1.1.v5.5 annot-version=v5.5
Chlamydomonas_reinhardtii OG0000001
Cre17.g737463.t1.1 pacid=30781799 transcript=Cre17.g737463.t1.1
locus=Cre17.g737463 ID=Cre17.g737463.t1.1.v5.5 annot-version=v5.5

Chlamydomonas_reinhardtii OG0000001
Cre17.g720300.t1.2 pacid=30782184 transcript=Cre17.g720300.t1.2
locus=Cre17.g720300 ID=Cre17.g720300.t1.2.v5.5 annot-version=v5.5
Chlamydomonas_reinhardtii OG0000001
Cre17.g737525.t1.1 pacid=30782208 transcript=Cre17.g737525.t1.1
locus=Cre17.g737525 ID=Cre17.g737525.t1.1.v5.5 annot-version=v5.5
Chlamydomonas_reinhardtii OG0000001
Cre17.g741150.t1.1 pacid=30782325 transcript=Cre17.g741150.t1.1
locus=Cre17.g741150 ID=Cre17.g741150.t1.1.v5.5 annot-version=v5.5
Chlamydomonas_reinhardtii OG0000001
Cre17.g737400.t1.1 pacid=30782542 transcript=Cre17.g737400.t1.1
locus=Cre17.g737400 ID=Cre17.g737400.t1.1.v5.5 annot-version=v5.5
Chlamydomonas_reinhardtii OG0000001
Cre17.g715200.t1.1 pacid=30782758 transcript=Cre17.g715200.t1.1
locus=Cre17.g715200 ID=Cre17.g715200.t1.1.v5.5 annot-version=v5.5
Chlamydomonas_reinhardtii OG0000001
Cre17.g737350.t1.1 pacid=30782821 transcript=Cre17.g737350.t1.1
locus=Cre17.g737350 ID=Cre17.g737350.t1.1.v5.5 annot-version=v5.5
Chlamydomonas_reinhardtii OG0000001
Cre13.g584450.t1.1 pacid=30784773 transcript=Cre13.g584450.t1.1
locus=Cre13.g584450 ID=Cre13.g584450.t1.1.v5.5 annot-version=v5.5
Chlamydomonas_reinhardtii OG0000001
Vocar.0030s0056.1.p pacid=32894604 transcript=Vocar.0030s0056.1
locus=Vocar.0030s0056 ID=Vocar.0030s0056.1.v2.1 annot-version=v2.1
Volvox_carteri OG0000001
Vocar.0012s0236.1.p pacid=32894924 transcript=Vocar.0012s0236.1
locus=Vocar.0012s0236 ID=Vocar.0012s0236.1.v2.1 annot-version=v2.1
Volvox_carteri OG0000001
Vocar.0070s0011.1.p pacid=32895836 transcript=Vocar.0070s0011.1
locus=Vocar.0070s0011 ID=Vocar.0070s0011.1.v2.1 annot-version=v2.1
Volvox_carteri OG0000001
Vocar.0021s0126.1.p pacid=32895979 transcript=Vocar.0021s0126.1
locus=Vocar.0021s0126 ID=Vocar.0021s0126.1.v2.1 annot-version=v2.1
Volvox_carteri OG0000001
Vocar.0016s0192.1.p pacid=32897922 transcript=Vocar.0016s0192.1
locus=Vocar.0016s0192 ID=Vocar.0016s0192.1.v2.1 annot-version=v2.1
Volvox_carteri OG0000001
Vocar.0025s0193.1.p pacid=32898296 transcript=Vocar.0025s0193.1
locus=Vocar.0025s0193 ID=Vocar.0025s0193.1.v2.1 annot-version=v2.1
Volvox_carteri OG0000001
Vocar.0015s0027.1.p pacid=32899041 transcript=Vocar.0015s0027.1
locus=Vocar.0015s0027 ID=Vocar.0015s0027.1.v2.1 annot-version=v2.1
Volvox_carteri OG0000001
Vocar.0001s1087.1.p pacid=32885057 transcript=Vocar.0001s1087.1
locus=Vocar.0001s1087 ID=Vocar.0001s1087.1.v2.1 annot-version=v2.1
Volvox_carteri OG0000001
Vocar.0001s1806.1.p pacid=32885899 transcript=Vocar.0001s1806.1
locus=Vocar.0001s1806 ID=Vocar.0001s1806.1.v2.1 annot-version=v2.1
Volvox_carteri OG0000001
Vocar.0033s0054.1.p pacid=32886500 transcript=Vocar.0033s0054.1
locus=Vocar.0033s0054 ID=Vocar.0033s0054.1.v2.1 annot-version=v2.1
Volvox_carteri OG0000001
Vocar.0007s0445.1.p pacid=32887931 transcript=Vocar.0007s0445.1
locus=Vocar.0007s0445 ID=Vocar.0007s0445.1.v2.1 annot-version=v2.1

Volvox_carteri   OG0000001
Vocar.0014s0168.1.p pacid=32888470 transcript=Vocar.0014s0168.1 locus=Vocar.0014s0168 ID=Vocar.0014s0168.1.v2.1 annot-version=v2.1
Volvox_carteri   OG0000001
Vocar.0041s0053.1.p pacid=32888743 transcript=Vocar.0041s0053.1 locus=Vocar.0041s0053 ID=Vocar.0041s0053.1.v2.1 annot-version=v2.1
Volvox_carteri   OG0000001
Vocar.0003s0057.1.p pacid=32888850 transcript=Vocar.0003s0057.1 locus=Vocar.0003s0057 ID=Vocar.0003s0057.1.v2.1 annot-version=v2.1
Volvox_carteri   OG0000001
Vocar.0004s0251.1.p pacid=32889438 transcript=Vocar.0004s0251.1 locus=Vocar.0004s0251 ID=Vocar.0004s0251.1.v2.1 annot-version=v2.1
Volvox_carteri   OG0000001
Vocar.0002s0147.1.p pacid=32890919 transcript=Vocar.0002s0147.1 locus=Vocar.0002s0147 ID=Vocar.0002s0147.1.v2.1 annot-version=v2.1
Volvox_carteri   OG0000001
Vocar.0002s0399.1.p pacid=32891011 transcript=Vocar.0002s0399.1 locus=Vocar.0002s0399 ID=Vocar.0002s0399.1.v2.1 annot-version=v2.1
Volvox_carteri   OG0000001
Vocar.0042s0111.1.p pacid=32892029 transcript=Vocar.0042s0111.1 locus=Vocar.0042s0111 ID=Vocar.0042s0111.1.v2.1 annot-version=v2.1
Volvox_carteri   OG0000001
Vocar.0036s0070.1.p pacid=32892272 transcript=Vocar.0036s0070.1 locus=Vocar.0036s0070 ID=Vocar.0036s0070.1.v2.1 annot-version=v2.1
Volvox_carteri   OG0000001
Vocar.0009s0341.1.p pacid=32893415 transcript=Vocar.0009s0341.1 locus=Vocar.0009s0341 ID=Vocar.0009s0341.1.v2.1 annot-version=v2.1
Volvox_carteri   OG0000001
Vocar.0006s0179.1.p pacid=32894162 transcript=Vocar.0006s0179.1 locus=Vocar.0006s0179 ID=Vocar.0006s0179.1.v2.1 annot-version=v2.1
Volvox_carteri   OG0000001
Vocar.0006s0161.1.p pacid=32894182 transcript=Vocar.0006s0161.1 locus=Vocar.0006s0161 ID=Vocar.0006s0161.1.v2.1 annot-version=v2.1
Volvox_carteri   OG0000001
jgi|ChlNC64A_1|140660|IGS.gm_3_00399   Chlorella_variabilis OG0000001
jgi|ChlNC64A_1|140893|IGS.gm_3_00632   Chlorella_variabilis OG0000001
jgi|ChlNC64A_1|134759|IGS.gm_12_00145   Chlorella_variabilis OG0000001
jgi|ChlNC64A_1|58999|estExt_fgenesh3_pg.C_240053 Chlorella_variabilis   OG0000001
jgi|ChlNC64A_1|133811|IGS.gm_10_00102   Chlorella_variabilis OG0000001
jgi|ChlNC64A_1|141046|IGS.gm_30_00084   Chlorella_variabilis OG0000001
jgi|ChlNC64A_1|136931|IGS.gm_18_00037   Chlorella_variabilis OG0000001
jgi|ChlNC64A_1|137597|IGS.gm_2_00064   Chlorella_variabilis OG0000001
jgi|ChlNC64A_1|137603|IGS.gm_2_00070   Chlorella_variabilis OG0000001
jgi|ChlNC64A_1|137605|IGS.gm_2_00072   Chlorella_variabilis OG0000001

jgi|ChlNC64A_1|56654|estExt_fgenesh3_pg.C_20040
Chlorella_variabilis	OG0000001
jgi|ChlNC64A_1|137615|IGS.gm_2_00082	Chlorella_variabilis
OG0000001
jgi|ChlNC64A_1|50123|fgenesh3_pg.C_scaffold_2000368
Chlorella_variabilis	OG0000001
jgi|ChlNC64A_1|142110|IGS.gm_4_00251	Chlorella_variabilis
OG0000001
jgi|ChlNC64A_1|35589|estExt_Genewise1Plus.C_110194
Chlorella_variabilis	OG0000001
jgi|ChlNC64A_1|52604|fgenesh3_pg.C_scaffold_11000180
Chlorella_variabilis	OG0000001
jgi|ChlNC64A_1|145350|IGS.gm_9_00157	Chlorella_variabilis
OG0000001
jgi|ChlNC64A_1|145500|IGS.gm_9_00307	Chlorella_variabilis
OG0000001
jgi|ChlNC64A_1|145501|IGS.gm_9_00308	Chlorella_variabilis
OG0000001
jgi|ChlNC64A_1|145502|IGS.gm_9_00309	Chlorella_variabilis
OG0000001
jgi|ChlNC64A_1|17626|gw1.9.412.1	Chlorella_variabilis
OG0000001
jgi|ChlNC64A_1|57778|estExt_fgenesh3_pg.C_90168
Chlorella_variabilis	OG0000001
jgi|ChlNC64A_1|138562|IGS.gm_21_00041	Chlorella_variabilis
OG0000001
jgi|ChlNC64A_1|57467|estExt_fgenesh3_pg.C_70027
Chlorella_variabilis	OG0000001
jgi|ChlNC64A_1|49746|fgenesh3_pg.C_scaffold_1000444
Chlorella_variabilis	OG0000001
jgi|ChlNC64A_1|15797|gw1.7.282.1	Chlorella_variabilis
OG0000001
jgi|ChlNC64A_1|141444|IGS.gm_34_00048	Chlorella_variabilis
OG0000001
jgi|ChlNC64A_1|144680|IGS.gm_8_00008	Chlorella_variabilis
OG0000001
jgi|ChlNC64A_1|58858|estExt_fgenesh3_pg.C_220049
Chlorella_variabilis	OG0000001
jgi|ChlNC64A_1|8973|gw1.8.98.1	Chlorella_variabilis
OG0000001
jgi|ChlNC64A_1|30865|estExt_Genewise1.C_80316
Chlorella_variabilis	OG0000001
jgi|ChlNC64A_1|53489|fgenesh3_pg.C_scaffold_16000001
Chlorella_variabilis	OG0000001
jgi|ChlNC64A_1|136507|IGS.gm_16_00230	Chlorella_variabilis
OG0000001
jgi|ChlNC64A_1|52984|fgenesh3_pg.C_scaffold_13000090
Chlorella_variabilis	OG0000001
jgi|ChlNC64A_1|136834|IGS.gm_17_00238	Chlorella_variabilis
OG0000001
jgi|ChlNC64A_1|136066|IGS.gm_15_00162	Chlorella_variabilis
OG0000001
jgi|ChlNC64A_1|25299|e_gw1.15.218.1	Chlorella_variabilis
OG0000001

jgi|ChlNC64A_1|136136|IGS.gm_15_00232	Chlorella_variabilis
OG0000001
jgi|ChlNC64A_1|141610|IGS.gm_36_00030	Chlorella_variabilis
OG0000001
jgi|ChlNC64A_1|143410|IGS.gm_57_00001	Chlorella_variabilis
OG0000001
65896 pacid=27386700 transcript=65896 locus=estExt_Genemark1.C_70071
ID=65896.2.0.227 annot-version=v2.0	Coccomyxa_subellipsoidea
OG0000001
66068 pacid=27386840 transcript=66068 locus=estExt_Genemark1.C_70380
ID=66068.2.0.227 annot-version=v2.0	Coccomyxa_subellipsoidea
OG0000001
20432 pacid=27387612 transcript=20432 locus=e_gw1.22.109.1
ID=20432.2.0.227 annot-version=v2.0	Coccomyxa_subellipsoidea
OG0000001
66439 pacid=27388467 transcript=66439 locus=estExt_Genemark1.C_90340
ID=66439.2.0.227 annot-version=v2.0	Coccomyxa_subellipsoidea
OG0000001
64013 pacid=27388627 transcript=64013 locus=Genemark1.4796_g
ID=64013.2.0.227 annot-version=v2.0	Coccomyxa_subellipsoidea
OG0000001
55314 pacid=27389413 transcript=55314 locus=Genemark1.258_g
ID=55314.2.0.227 annot-version=v2.0	Coccomyxa_subellipsoidea
OG0000001
13349 pacid=27389668 transcript=13349 locus=e_gw1.4.664.1
ID=13349.2.0.227 annot-version=v2.0	Coccomyxa_subellipsoidea
OG0000001
13805 pacid=27390051 transcript=13805 locus=e_gw1.4.291.1
ID=13805.2.0.227 annot-version=v2.0	Coccomyxa_subellipsoidea
OG0000001
61252 pacid=27390075 transcript=61252 locus=Genemark1.2042_g
ID=61252.2.0.227 annot-version=v2.0	Coccomyxa_subellipsoidea
OG0000001
60822 pacid=27390632 transcript=60822 locus=Genemark1.1633_g
ID=60822.2.0.227 annot-version=v2.0	Coccomyxa_subellipsoidea
OG0000001
47112 pacid=27391198 transcript=47112
locus=estExt_fgenesh1_pg.C_60158 ID=47112.2.0.227 annot-version=v2.0
	Coccomyxa_subellipsoidea	OG0000001
56455 pacid=27391895 transcript=56455 locus=Genemark1.5484_g
ID=56455.2.0.227 annot-version=v2.0	Coccomyxa_subellipsoidea
OG0000001
63353 pacid=27392297 transcript=63353 locus=Genemark1.4136_g
ID=63353.2.0.227 annot-version=v2.0	Coccomyxa_subellipsoidea
OG0000001
27330 pacid=27393070 transcript=27330
locus=estExt_Genewise1Plus.C_20732 ID=27330.2.0.227 annot-
version=v2.0	Coccomyxa_subellipsoidea	OG0000001
68225 pacid=27386392 transcript=68225
locus=estExt_Genemark1.C_230044 ID=68225.2.0.227 annot-version=v2.0
Coccomyxa_subellipsoidea	OG0000001
67732 pacid=27394334 transcript=67732
locus=estExt_Genemark1.C_180121 ID=67732.2.0.227 annot-version=v2.0
Coccomyxa_subellipsoidea	OG0000001

58198 pacid=27394612 transcript=58198 locus=Genemark1.7227_g
ID=58198.2.0.227 annot-version=v2.0	Coccomyxa_subellipsoidea
OG0000001
34125 pacid=27394617 transcript=34125 locus=fgenesh1_kg.
17_#_19_#_719_0_CBPA2243.g1_CBOZ_CBPA ID=34125.2.0.227 annot-
version=v2.0	Coccomyxa_subellipsoidea	OG0000001
14097_fgenesh1_pg.C_Chr_17.0001000004	Ostreococcus_tauri
OG0000001
14465_fgenesh1_pg.C_Chr_19.0001000037	Ostreococcus_tauri
OG0000001
14545_fgenesh1_pg.C_Chr_20.0001000060	Ostreococcus_tauri
OG0000001
20190_e_gw1.11.00.101.1	Ostreococcus_tauri	OG0000001
31679_0300010233 Ostreococcus_tauri	OG0000001
7232_gw1.06.00.431.1	Ostreococcus_tauri	OG0000001
33733_0700010086 Ostreococcus_tauri	OG0000001
35263_1000010283 Ostreococcus_tauri	OG0000001
9377_fgenesh1_pg.C_Chr_01.0001000143	Ostreococcus_tauri
OG0000001
32486 pacid=27413988 transcript=32486 locus=eugene.0700010083
ID=32486.2.0.231 annot-version=v2.0	Ostreococcus_lucimarinus
OG0000001
18438 pacid=27415966 transcript=18438
locus=fgenesh1_pg.C_Chr_16000003 ID=18438.2.0.231 annot-version=v2.0
Ostreococcus_lucimarinus	OG0000001
6692 pacid=27416350 transcript=6692 locus=gwEuk.10.348.1
ID=6692.2.0.231 annot-version=v2.0	Ostreococcus_lucimarinus
OG0000001
28825 pacid=27417750 transcript=28825 locus=eugene.0100010219
ID=28825.2.0.231 annot-version=v2.0	Ostreococcus_lucimarinus
OG0000001
86229 pacid=27418111 transcript=86229 locus=ost_01_021_090
ID=86229.2.0.231 annot-version=v2.0	Ostreococcus_lucimarinus
OG0000001
32267 pacid=27418165 transcript=32267 locus=eugene.0600010326
ID=32267.2.0.231 annot-version=v2.0	Ostreococcus_lucimarinus
OG0000001
10647 pacid=27418461 transcript=10647 locus=gwEuk.6.727.1
ID=10647.2.0.231 annot-version=v2.0	Ostreococcus_lucimarinus
OG0000001
7508 pacid=27413467 transcript=7508 locus=gwEuk.3.577.1
ID=7508.2.0.231 annot-version=v2.0	Ostreococcus_lucimarinus
OG0000001
5174 pacid=27419175 transcript=5174 locus=gwEuk.19.57.1
ID=5174.2.0.231 annot-version=v2.0	Ostreococcus_lucimarinus
OG0000001
1531 pacid=27420472 transcript=1531 locus=gwEuk.11.77.1
ID=1531.2.0.231 annot-version=v2.0	Ostreococcus_lucimarinus
OG0000001
164123 pacid=27348658 transcript=164163 locus=e_gw1.15.496.1
ID=164163.3.0.228 annot-version=v3.0	Micromonas_pusilla
OG0000001
9213 pacid=27339956 transcript=9213 locus=MicpuC2.gw1.13.348.1
ID=9213.3.0.228 annot-version=v3.0	Micromonas_pusilla

OG0000001
51282 pacid=27340133 transcript=51282 locus=MicpuC2.EuGene.
0000100353 ID=51282.3.0.228 annot-version=v3.0
Micromonas_pusilla OG0000001
178601 pacid=27341441 transcript=178641
locus=estExt_Genewise1Plus.C_5_t30232 ID=178641.3.0.228 annot-
version=v3.0 Micromonas_pusilla OG0000001
186515 pacid=27341510 transcript=186555 locus=gm1.3636_g
ID=186555.3.0.228 annot-version=v3.0 Micromonas_pusilla
OG0000001
147641 pacid=27341835 transcript=147681 locus=gw1.5.1456.1
ID=147681.3.0.228 annot-version=v3.0 Micromonas_pusilla
OG0000001
59838 pacid=27339144 transcript=59838 locus=MicpuC2.EuGene.
0000080032 ID=59838.3.0.228 annot-version=v3.0
Micromonas_pusilla OG0000001
158715 pacid=27339146 transcript=158755 locus=e_gw1.8.953.1
ID=158755.3.0.228 annot-version=v3.0 Micromonas_pusilla
OG0000001
50634 pacid=27345873 transcript=50634 locus=MicpuC2.EuGene.
0000010858 ID=50634.3.0.228 annot-version=v3.0
Micromonas_pusilla OG0000001
50694 pacid=27346037 transcript=50694 locus=MicpuC2.EuGene.
0000010918 ID=50694.3.0.228 annot-version=v3.0
Micromonas_pusilla OG0000001
57330 pacid=27346291 transcript=57330 locus=MicpuC2.EuGene.
0000040456 ID=57330.3.0.228 annot-version=v3.0
Micromonas_pusilla OG0000001
32570 pacid=27347501 transcript=32570
locus=MicpuC2.estExt_Genewise1Plus.C_30924 ID=32570.3.0.228 annot-
version=v3.0 Micromonas_pusilla OG0000001
168790 pacid=27347644 transcript=168830
locus=estExt_Genewise1.C_3_t40048 ID=168830.3.0.228 annot-
version=v3.0 Micromonas_pusilla OG0000001
2384 pacid=27347929 transcript=2384 locus=MicpuC2.gw1.6.88.1
ID=2384.3.0.228 annot-version=v3.0 Micromonas_pusilla
OG0000001
180896 pacid=27339619 transcript=180936
locus=estExt_Genewise1Plus.C_11_t10260 ID=180936.3.0.228 annot-
version=v3.0 Micromonas_pusilla OG0000001
107672 pacid=27396510 transcript=107672
locus=est_cluster_kg.Chr_02__36__3200519:1 ID=107672.3.0.229 annot-
version=v3.0 Micromonas_sp._RCC299 OG0000001
99058 pacid=27396622 transcript=99058
locus=fgenesh2_pg.C_Chr_02000434 ID=99058.3.0.229 annot-version=v3.0
Micromonas_sp._RCC299 OG0000001
71757 pacid=27396827 transcript=71757 locus=gw2.02.610.1
ID=71757.3.0.229 annot-version=v3.0 Micromonas_sp._RCC299
OG0000001
81826 pacid=27395423 transcript=81826 locus=e_gw2.05.838.1
ID=81826.3.0.229 annot-version=v3.0 Micromonas_sp._RCC299
OG0000001
78713 pacid=27396942 transcript=78713 locus=e_gw2.02.1066.1
ID=78713.3.0.229 annot-version=v3.0 Micromonas_sp._RCC299

OG0000001
89134 pacid=27397018 transcript=89134 locus=e_gw2.16.191.1
ID=89134.3.0.229 annot-version=v3.0	Micromonas_sp._RCC299
OG0000001
89053 pacid=27397164 transcript=89053 locus=e_gw2.16.246.1
ID=89053.3.0.229 annot-version=v3.0	Micromonas_sp._RCC299
OG0000001
109661 pacid=27397210 transcript=109661
locus=est_cluster_kg.Chr_16__58__3199908:1 ID=109661.3.0.229 annot-version=v3.0	Micromonas_sp._RCC299	OG0000001
88873 pacid=27397223 transcript=88873 locus=e_gw2.16.315.1
ID=88873.3.0.229 annot-version=v3.0	Micromonas_sp._RCC299
OG0000001
104507 pacid=27397226 transcript=104507
locus=fgenesh2_pg.C_Chr_16000037 ID=104507.3.0.229 annot-version=v3.0	Micromonas_sp._RCC299	OG0000001
89195 pacid=27397259 transcript=89195 locus=e_gw2.16.247.1
ID=89195.3.0.229 annot-version=v3.0	Micromonas_sp._RCC299
OG0000001
85925 pacid=27397385 transcript=85925 locus=e_gw2.10.653.1
ID=85925.3.0.229 annot-version=v3.0	Micromonas_sp._RCC299
OG0000001
108233 pacid=27395288 transcript=108233
locus=est_cluster_kg.Chr_05__42__3199878:1 ID=108233.3.0.229 annot-version=v3.0	Micromonas_sp._RCC299	OG0000001
74959 pacid=27395491 transcript=74959 locus=gw2.05.789.1
ID=74959.3.0.229 annot-version=v3.0	Micromonas_sp._RCC299
OG0000001
102887 pacid=27397700 transcript=102887
locus=fgenesh2_pg.C_Chr_10000373 ID=102887.3.0.229 annot-version=v3.0	Micromonas_sp._RCC299	OG0000001
62214 pacid=27397726 transcript=62214 locus=EuGene.1000010551
ID=62214.3.0.229 annot-version=v3.0	Micromonas_sp._RCC299
OG0000001
82305 pacid=27395520 transcript=82305 locus=e_gw2.05.584.1
ID=82305.3.0.229 annot-version=v3.0	Micromonas_sp._RCC299
OG0000001
79944 pacid=27397957 transcript=79944 locus=e_gw2.03.235.1
ID=79944.3.0.229 annot-version=v3.0	Micromonas_sp._RCC299
OG0000001
99220 pacid=27398639 transcript=99220
locus=fgenesh2_pg.C_Chr_03000001 ID=99220.3.0.229 annot-version=v3.0
	Micromonas_sp._RCC299	OG0000001
55526 pacid=27398709 transcript=55526 locus=EuGene.0100010753
ID=55526.3.0.229 annot-version=v3.0	Micromonas_sp._RCC299
OG0000001
55525 pacid=27398801 transcript=55525 locus=EuGene.0100010752
ID=55525.3.0.229 annot-version=v3.0	Micromonas_sp._RCC299
OG0000001
77261 pacid=27399047 transcript=77261 locus=e_gw2.01.999.1
ID=77261.3.0.229 annot-version=v3.0	Micromonas_sp._RCC299
OG0000001
77808 pacid=27399120 transcript=77808 locus=e_gw2.01.228.1
ID=77808.3.0.229 annot-version=v3.0	Micromonas_sp._RCC299

OG0000001
98569 pacid=27399127 transcript=98569
locus=fgenesh2_pg.C_Chr_01000126 ID=98569.3.0.229 annot-version=v3.0
Micromonas_sp._RCC299 OG0000001
55535 pacid=27399128 transcript=55535 locus=EuGene.0100010762
ID=55535.3.0.229 annot-version=v3.0 Micromonas_sp._RCC299
OG0000001
107462 pacid=27399147 transcript=107462
locus=est_cluster_kg.Chr_01__26__3202155:1 ID=107462.3.0.229 annot-version=v3.0 Micromonas_sp._RCC299 OG0000001
107620 pacid=27399272 transcript=107620
locus=est_cluster_kg.Chr_01__184__3201176:1 ID=107620.3.0.229 annot-version=v3.0 Micromonas_sp._RCC299 OG0000001
71845 pacid=27399316 transcript=71845 locus=gw2.01.645.1
ID=71845.3.0.229 annot-version=v3.0 Micromonas_sp._RCC299
OG0000001
71867 pacid=27399783 transcript=71867 locus=gw2.12.358.1
ID=71867.3.0.229 annot-version=v3.0 Micromonas_sp._RCC299
OG0000001
72677 pacid=27399791 transcript=72677 locus=gw2.12.397.1
ID=72677.3.0.229 annot-version=v3.0 Micromonas_sp._RCC299
OG0000001
62984 pacid=27399799 transcript=62984 locus=EuGene.1200010156
ID=62984.3.0.229 annot-version=v3.0 Micromonas_sp._RCC299
OG0000001
75476 pacid=27399976 transcript=75476 locus=gw2.12.528.1
ID=75476.3.0.229 annot-version=v3.0 Micromonas_sp._RCC299
OG0000001
58267 pacid=27400024 transcript=58267 locus=EuGene.0400010796
ID=58267.3.0.229 annot-version=v3.0 Micromonas_sp._RCC299
OG0000001
81207 pacid=27400485 transcript=81207 locus=e_gw2.04.521.1
ID=81207.3.0.229 annot-version=v3.0 Micromonas_sp._RCC299
OG0000001
57486 pacid=27400557 transcript=57486 locus=EuGene.0400010015
ID=57486.3.0.229 annot-version=v3.0 Micromonas_sp._RCC299
OG0000001
64639 pacid=27401073 transcript=64639 locus=EuGene.1500010360
ID=64639.3.0.229 annot-version=v3.0 Micromonas_sp._RCC299
OG0000001
109519 pacid=27401131 transcript=109519
locus=est_cluster_kg.Chr_15__1__3201171:1 ID=109519.3.0.229 annot-version=v3.0 Micromonas_sp._RCC299 OG0000001
85191 pacid=27401241 transcript=85191 locus=e_gw2.09.692.1
ID=85191.3.0.229 annot-version=v3.0 Micromonas_sp._RCC299
OG0000001
74314 pacid=27401292 transcript=74314 locus=gw2.09.601.1
ID=74314.3.0.229 annot-version=v3.0 Micromonas_sp._RCC299
OG0000001
61629 pacid=27401298 transcript=61629 locus=EuGene.0900010612
ID=61629.3.0.229 annot-version=v3.0 Micromonas_sp._RCC299
OG0000001
61018 pacid=27401303 transcript=61018 locus=EuGene.0900010001
ID=61018.3.0.229 annot-version=v3.0 Micromonas_sp._RCC299

OG0000001
75659 pacid=27401843 transcript=75659 locus=gw2.06.793.1
ID=75659.3.0.229 annot-version=v3.0 Micromonas_sp._RCC299
OG0000001
82436 pacid=27402101 transcript=82436 locus=e_gw2.06.592.1
ID=82436.3.0.229 annot-version=v3.0 Micromonas_sp._RCC299
OG0000001
83010 pacid=27402173 transcript=83010 locus=e_gw2.06.540.1
ID=83010.3.0.229 annot-version=v3.0 Micromonas_sp._RCC299
OG0000001
59673 pacid=27402217 transcript=59673 locus=EuGene.0600010675
ID=59673.3.0.229 annot-version=v3.0 Micromonas_sp._RCC299
OG0000001
59715 pacid=27402243 transcript=59715 locus=EuGene.0600010717
ID=59715.3.0.229 annot-version=v3.0 Micromonas_sp._RCC299
OG0000001
81822 pacid=27395990 transcript=81822 locus=e_gw2.05.732.1
ID=81822.3.0.229 annot-version=v3.0 Micromonas_sp._RCC299
OG0000001
65992 pacid=27402917 transcript=65992 locus=gw2.13.49.1
ID=65992.3.0.229 annot-version=v3.0 Micromonas_sp._RCC299
OG0000001
87686 pacid=27402958 transcript=87686 locus=e_gw2.13.510.1
ID=87686.3.0.229 annot-version=v3.0 Micromonas_sp._RCC299
OG0000001
84418 pacid=27403137 transcript=84418 locus=e_gw2.08.317.1
ID=84418.3.0.229 annot-version=v3.0 Micromonas_sp._RCC299
OG0000001
71908 pacid=27403182 transcript=71908 locus=gw2.08.417.1
ID=71908.3.0.229 annot-version=v3.0 Micromonas_sp._RCC299
OG0000001
78699 pacid=27396056 transcript=78699 locus=e_gw2.02.710.1
ID=78699.3.0.229 annot-version=v3.0 Micromonas_sp._RCC299
OG0000001
84421 pacid=27403595 transcript=84421 locus=e_gw2.08.188.1
ID=84421.3.0.229 annot-version=v3.0 Micromonas_sp._RCC299
OG0000001
84219 pacid=27403623 transcript=84219 locus=e_gw2.08.458.1
ID=84219.3.0.229 annot-version=v3.0 Micromonas_sp._RCC299
OG0000001
67619 pacid=27403724 transcript=67619 locus=gw2.14.106.1
ID=67619.3.0.229 annot-version=v3.0 Micromonas_sp._RCC299
OG0000001
88344 pacid=27403761 transcript=88344 locus=e_gw2.14.426.1
ID=88344.3.0.229 annot-version=v3.0 Micromonas_sp._RCC299
OG0000001
83283 pacid=27404319 transcript=83283 locus=e_gw2.07.575.1
ID=83283.3.0.229 annot-version=v3.0 Micromonas_sp._RCC299
OG0000001
101709 pacid=27404542 transcript=101709
locus=fgenesh2_pg.C_Chr_07000453 ID=101709.3.0.229 annot-
version=v3.0 Micromonas_sp._RCC299 OG0000001
108717 pacid=27404718 transcript=108717
locus=est_cluster_kg.Chr_07__187__3200613:1 ID=108717.3.0.229 annot-

version=v3.0   Micromonas_sp._RCC299   OG0000001
72501 pacid=27395361 transcript=72501 locus=gw2.05.575.1
ID=72501.3.0.229 annot-version=v3.0   Micromonas_sp._RCC299
OG0000001
86281 pacid=27404907 transcript=86281 locus=e_gw2.11.90.1
ID=86281.3.0.229 annot-version=v3.0   Micromonas_sp._RCC299
OG0000001
74345 pacid=27405298 transcript=74345 locus=gw2.11.558.1
ID=74345.3.0.229 annot-version=v3.0   Micromonas_sp._RCC299
OG0000001
kfl00349_0030_v1.1   Klebsormidium_flaccidum OG0000001
kfl00015_0310_v1.1   Klebsormidium_flaccidum OG0000001
kfl00407_0050_v1.1   Klebsormidium_flaccidum OG0000001
kfl00439_0010_v1.1   Klebsormidium_flaccidum OG0000001
kfl00441_0040_v1.1   Klebsormidium_flaccidum OG0000001
kfl00017_0430_v1.1   Klebsormidium_flaccidum OG0000001
kfl00478_0050_v1.1   Klebsormidium_flaccidum OG0000001
kfl00484_0040_v1.1   Klebsormidium_flaccidum OG0000001
kfl00538_0060_v1.1   Klebsormidium_flaccidum OG0000001
kfl00549_0060_v1.1   Klebsormidium_flaccidum OG0000001
kfl00596_0010_v1.1   Klebsormidium_flaccidum OG0000001
kfl00652_0030_v1.1   Klebsormidium_flaccidum OG0000001
kfl00680_0050_v1.1   Klebsormidium_flaccidum OG0000001
kfl00719_0060_v1.1   Klebsormidium_flaccidum OG0000001
kfl00738_0090_v1.1   Klebsormidium_flaccidum OG0000001
kfl00746_0010_v1.1   Klebsormidium_flaccidum OG0000001
kfl00794_0100_v1.1   Klebsormidium_flaccidum OG0000001
kfl00022_0520_v1.1   Klebsormidium_flaccidum OG0000001
kfl00982_0060_v1.1   Klebsormidium_flaccidum OG0000001
kfl01052_0030_v1.1   Klebsormidium_flaccidum OG0000001
kfl01056_0010_v1.1   Klebsormidium_flaccidum OG0000001
kfl01060_0010_v1.1   Klebsormidium_flaccidum OG0000001
kfl01060_0035_v1.1   Klebsormidium_flaccidum OG0000001
kfl01118_0040_v1.1   Klebsormidium_flaccidum OG0000001
kfl01223_0020_v1.1   Klebsormidium_flaccidum OG0000001
kfl01227_0010_v1.1   Klebsormidium_flaccidum OG0000001
kfl01227_0020_v1.1   Klebsormidium_flaccidum OG0000001
kfl00031_0510_v1.1   Klebsormidium_flaccidum OG0000001
kfl00035_0190_v1.1   Klebsormidium_flaccidum OG0000001
kfl00003_0570_v1.1   Klebsormidium_flaccidum OG0000001
kfl00003_0660_v1.1   Klebsormidium_flaccidum OG0000001
kfl00045_0400_v1.1   Klebsormidium_flaccidum OG0000001
kfl00050_0170_v1.1   Klebsormidium_flaccidum OG0000001
kfl00051_0040_v1.1   Klebsormidium_flaccidum OG0000001
kfl00054_0090_v1.1   Klebsormidium_flaccidum OG0000001
kfl00065_0330_v1.1   Klebsormidium_flaccidum OG0000001
kfl00070_0080_v1.1   Klebsormidium_flaccidum OG0000001
kfl00078_0010_v1.1   Klebsormidium_flaccidum OG0000001
kfl00081_0210_v1.1   Klebsormidium_flaccidum OG0000001
kfl00087_0220_v1.1   Klebsormidium_flaccidum OG0000001
kfl00089_0310_v1.1   Klebsormidium_flaccidum OG0000001
kfl00103_0010_v1.1   Klebsormidium_flaccidum OG0000001
kfl00112_0040_v1.1   Klebsormidium_flaccidum OG0000001
kfl00007_0350_v1.1   Klebsormidium_flaccidum OG0000001

kfl00007_0445_v1.1   Klebsormidium_flaccidum OG0000001
kfl00007_0610_v1.1   Klebsormidium_flaccidum OG0000001
kfl00007_0615_v1.1   Klebsormidium_flaccidum OG0000001
kfl00008_0140_v1.1   Klebsormidium_flaccidum OG0000001
kfl00139_0330_v1.1   Klebsormidium_flaccidum OG0000001
kfl00009_0150_v1.1   Klebsormidium_flaccidum OG0000001
kfl00157_0060_v1.1   Klebsormidium_flaccidum OG0000001
kfl00171_0060_v1.1   Klebsormidium_flaccidum OG0000001
kfl00176_0010_v1.1   Klebsormidium_flaccidum OG0000001
kfl00010_0400_v1.1   Klebsormidium_flaccidum OG0000001
kfl00196_0130_v1.1   Klebsormidium_flaccidum OG0000001
kfl00208_0190_v1.1   Klebsormidium_flaccidum OG0000001
kfl00214_0030_v1.1   Klebsormidium_flaccidum OG0000001
kfl00217_g21_v1.1   Klebsormidium_flaccidum OG0000001
kfl00242_0040_v1.1   Klebsormidium_flaccidum OG0000001
kfl00243_0020_v1.1   Klebsormidium_flaccidum OG0000001
kfl00249_0070_v1.1   Klebsormidium_flaccidum OG0000001
kfl00002_g1_v1.1 Klebsormidium_flaccidum OG0000001
kfl00279_0080_v1.1   Klebsormidium_flaccidum OG0000001
kfl00290_0090_v1.1   Klebsormidium_flaccidum OG0000001
kfl00304_0125_v1.1   Klebsormidium_flaccidum OG0000001
kfl00002_0120_v1.1   Klebsormidium_flaccidum OG0000001
kfl00320_0120_v1.1   Klebsormidium_flaccidum OG0000001
kfl00002_0130_v1.1   Klebsormidium_flaccidum OG0000001
kfl00325_0200_v1.1   Klebsormidium_flaccidum OG0000001
Pp3c7_5330V3.1.p pacid=32926438 transcript=Pp3c7_5330V3.1
locus=Pp3c7_5330 annot-version=v3.3   Physcomitrella_patens
OG0000001
Pp3c15_23400V3.1.p pacid=32927931 transcript=Pp3c15_23400V3.1
locus=Pp3c15_23400 annot-version=v3.3   Physcomitrella_patens
OG0000001
Pp3c15_7730V3.1.p pacid=32928486 transcript=Pp3c15_7730V3.1
locus=Pp3c15_7730 annot-version=v3.3   Physcomitrella_patens
OG0000001
Pp3c15_14980V3.1.p pacid=32928802 transcript=Pp3c15_14980V3.1
locus=Pp3c15_14980 annot-version=v3.3   Physcomitrella_patens
OG0000001
Pp3c13_6220V3.1.p pacid=32930445 transcript=Pp3c13_6220V3.1
locus=Pp3c13_6220 annot-version=v3.3   Physcomitrella_patens
OG0000001
Pp3c13_13360V3.1.p pacid=32930986 transcript=Pp3c13_13360V3.1
locus=Pp3c13_13360 annot-version=v3.3   Physcomitrella_patens
OG0000001
Pp3c13_13820V3.1.p pacid=32932167 transcript=Pp3c13_13820V3.1
locus=Pp3c13_13820 annot-version=v3.3   Physcomitrella_patens
OG0000001
Pp3c2_18570V3.1.p pacid=32933192 transcript=Pp3c2_18570V3.1
locus=Pp3c2_18570 annot-version=v3.3   Physcomitrella_patens
OG0000001
Pp3c22_13430V3.1.p pacid=32902946 transcript=Pp3c22_13430V3.1
locus=Pp3c22_13430 annot-version=v3.3   Physcomitrella_patens
OG0000001
Pp3c2_19460V3.1.p pacid=32934007 transcript=Pp3c2_19460V3.1
locus=Pp3c2_19460 annot-version=v3.3   Physcomitrella_patens

OG0000001
Pp3c2_22410V3.1.p pacid=32936286 transcript=Pp3c2_22410V3.1
locus=Pp3c2_22410 annot-version=v3.3 Physcomitrella_patens
OG0000001
Pp3c19_15110V3.1.p pacid=32938369 transcript=Pp3c19_15110V3.1
locus=Pp3c19_15110 annot-version=v3.3 Physcomitrella_patens
OG0000001
Pp3c19_9410V3.1.p pacid=32938427 transcript=Pp3c19_9410V3.1
locus=Pp3c19_9410 annot-version=v3.3 Physcomitrella_patens
OG0000001
Pp3c19_14630V3.1.p pacid=32938581 transcript=Pp3c19_14630V3.1
locus=Pp3c19_14630 annot-version=v3.3 Physcomitrella_patens
OG0000001
Pp3c19_8660V3.1.p pacid=32938769 transcript=Pp3c19_8660V3.1
locus=Pp3c19_8660 annot-version=v3.3 Physcomitrella_patens
OG0000001
Pp3c19_5680V3.1.p pacid=32939101 transcript=Pp3c19_5680V3.1
locus=Pp3c19_5680 annot-version=v3.3 Physcomitrella_patens
OG0000001
Pp3c19_18410V3.1.p pacid=32939120 transcript=Pp3c19_18410V3.1
locus=Pp3c19_18410 annot-version=v3.3 Physcomitrella_patens
OG0000001
Pp3c19_13129V3.1.p pacid=32939196 transcript=Pp3c19_13129V3.1
locus=Pp3c19_13129 annot-version=v3.3 Physcomitrella_patens
OG0000001
Pp3c19_5770V3.1.p pacid=32939729 transcript=Pp3c19_5770V3.1
locus=Pp3c19_5770 annot-version=v3.3 Physcomitrella_patens
OG0000001
Pp3c19_4590V3.1.p pacid=32939901 transcript=Pp3c19_4590V3.1
locus=Pp3c19_4590 annot-version=v3.3 Physcomitrella_patens
OG0000001
Pp3c19_19180V3.1.p pacid=32940159 transcript=Pp3c19_19180V3.1
locus=Pp3c19_19180 annot-version=v3.3 Physcomitrella_patens
OG0000001
Pp3c3_15220V3.1.p pacid=32940188 transcript=Pp3c3_15220V3.1
locus=Pp3c3_15220 annot-version=v3.3 Physcomitrella_patens
OG0000001
Pp3c3_17280V3.1.p pacid=32943375 transcript=Pp3c3_17280V3.1
locus=Pp3c3_17280 annot-version=v3.3 Physcomitrella_patens
OG0000001
Pp3c3_28520V3.1.p pacid=32943425 transcript=Pp3c3_28520V3.1
locus=Pp3c3_28520 annot-version=v3.3 Physcomitrella_patens
OG0000001
Pp3c3_29330V3.1.p pacid=32943497 transcript=Pp3c3_29330V3.1
locus=Pp3c3_29330 annot-version=v3.3 Physcomitrella_patens
OG0000001
Pp3c3_12560V3.1.p pacid=32943561 transcript=Pp3c3_12560V3.1
locus=Pp3c3_12560 annot-version=v3.3 Physcomitrella_patens
OG0000001
Pp3c3_36180V3.1.p pacid=32944434 transcript=Pp3c3_36180V3.1
locus=Pp3c3_36180 annot-version=v3.3 Physcomitrella_patens
OG0000001
Pp3c3_3780V3.1.p pacid=32944648 transcript=Pp3c3_3780V3.1
locus=Pp3c3_3780 annot-version=v3.3 Physcomitrella_patens

OG0000001
Pp3c20_20430V3.1.p pacid=32946254 transcript=Pp3c20_20430V3.1 locus=Pp3c20_20430 annot-version=v3.3 Physcomitrella_patens
OG0000001
Pp3c20_2070V3.1.p pacid=32947034 transcript=Pp3c20_2070V3.1 locus=Pp3c20_2070 annot-version=v3.3 Physcomitrella_patens
OG0000001
Pp3c23_19730V3.1.p pacid=32949890 transcript=Pp3c23_19730V3.1 locus=Pp3c23_19730 annot-version=v3.3 Physcomitrella_patens
OG0000001
Pp3c23_1160V3.1.p pacid=32950292 transcript=Pp3c23_1160V3.1 locus=Pp3c23_1160 annot-version=v3.3 Physcomitrella_patens
OG0000001
Pp3c23_17120V3.1.p pacid=32950466 transcript=Pp3c23_17120V3.1 locus=Pp3c23_17120 annot-version=v3.3 Physcomitrella_patens
OG0000001
Pp3c23_5250V3.1.p pacid=32950470 transcript=Pp3c23_5250V3.1 locus=Pp3c23_5250 annot-version=v3.3 Physcomitrella_patens
OG0000001
Pp3c23_19100V3.1.p pacid=32950884 transcript=Pp3c23_19100V3.1 locus=Pp3c23_19100 annot-version=v3.3 Physcomitrella_patens
OG0000001
Pp3c22_10630V3.1.p pacid=32904736 transcript=Pp3c22_10630V3.1 locus=Pp3c22_10630 annot-version=v3.3 Physcomitrella_patens
OG0000001
Pp3c5_19200V3.1.p pacid=32952727 transcript=Pp3c5_19200V3.1 locus=Pp3c5_19200 annot-version=v3.3 Physcomitrella_patens
OG0000001
Pp3c5_6590V3.1.p pacid=32953486 transcript=Pp3c5_6590V3.1 locus=Pp3c5_6590 annot-version=v3.3 Physcomitrella_patens
OG0000001
Pp3c5_8630V3.1.p pacid=32953567 transcript=Pp3c5_8630V3.1 locus=Pp3c5_8630 annot-version=v3.3 Physcomitrella_patens
OG0000001
Pp3c5_2390V3.1.p pacid=32955107 transcript=Pp3c5_2390V3.1 locus=Pp3c5_2390 annot-version=v3.3 Physcomitrella_patens
OG0000001
Pp3c22_21420V3.1.p pacid=32905155 transcript=Pp3c22_21420V3.1 locus=Pp3c22_21420 annot-version=v3.3 Physcomitrella_patens
OG0000001
Pp3c11_18980V3.1.p pacid=32956837 transcript=Pp3c11_18980V3.1 locus=Pp3c11_18980 annot-version=v3.3 Physcomitrella_patens
OG0000001
Pp3c11_20500V3.1.p pacid=32958169 transcript=Pp3c11_20500V3.1 locus=Pp3c11_20500 annot-version=v3.3 Physcomitrella_patens
OG0000001
Pp3c11_10500V3.1.p pacid=32958565 transcript=Pp3c11_10500V3.1 locus=Pp3c11_10500 annot-version=v3.3 Physcomitrella_patens
OG0000001
Pp3c11_5590V3.1.p pacid=32958736 transcript=Pp3c11_5590V3.1 locus=Pp3c11_5590 annot-version=v3.3 Physcomitrella_patens
OG0000001
Pp3c10_12760V3.1.p pacid=32899967 transcript=Pp3c10_12760V3.1 locus=Pp3c10_12760 annot-version=v3.3 Physcomitrella_patens

OG0000001
Pp3c14_2970V3.1.p pacid=32961399 transcript=Pp3c14_2970V3.1
locus=Pp3c14_2970 annot-version=v3.3 Physcomitrella_patens
OG0000001
Pp3c14_7280V3.1.p pacid=32961481 transcript=Pp3c14_7280V3.1
locus=Pp3c14_7280 annot-version=v3.3 Physcomitrella_patens
OG0000001
Pp3c14_13680V3.1.p pacid=32961540 transcript=Pp3c14_13680V3.1
locus=Pp3c14_13680 annot-version=v3.3 Physcomitrella_patens
OG0000001
Pp3c14_16840V3.1.p pacid=32963091 transcript=Pp3c14_16840V3.1
locus=Pp3c14_16840 annot-version=v3.3 Physcomitrella_patens
OG0000001
Pp3c8_15530V3.1.p pacid=32963440 transcript=Pp3c8_15530V3.1
locus=Pp3c8_15530 annot-version=v3.3 Physcomitrella_patens
OG0000001
Pp3c8_17530V3.1.p pacid=32963479 transcript=Pp3c8_17530V3.1
locus=Pp3c8_17530 annot-version=v3.3 Physcomitrella_patens
OG0000001
Pp3c8_2540V3.1.p pacid=32965030 transcript=Pp3c8_2540V3.1
locus=Pp3c8_2540 annot-version=v3.3 Physcomitrella_patens
OG0000001
Pp3c8_3600V3.1.p pacid=32965958 transcript=Pp3c8_3600V3.1
locus=Pp3c8_3600 annot-version=v3.3 Physcomitrella_patens
OG0000001
Pp3c8_14320V3.1.p pacid=32966339 transcript=Pp3c8_14320V3.1
locus=Pp3c8_14320 annot-version=v3.3 Physcomitrella_patens
OG0000001
Pp3c1_25110V3.1.p pacid=32967561 transcript=Pp3c1_25110V3.1
locus=Pp3c1_25110 annot-version=v3.3 Physcomitrella_patens
OG0000001
Pp3c1_18260V3.1.p pacid=32967921 transcript=Pp3c1_18260V3.1
locus=Pp3c1_18260 annot-version=v3.3 Physcomitrella_patens
OG0000001
Pp3c1_41620V3.1.p pacid=32968088 transcript=Pp3c1_41620V3.1
locus=Pp3c1_41620 annot-version=v3.3 Physcomitrella_patens
OG0000001
Pp3c1_16110V3.1.p pacid=32968387 transcript=Pp3c1_16110V3.1
locus=Pp3c1_16110 annot-version=v3.3 Physcomitrella_patens
OG0000001
Pp3c1_22560V3.1.p pacid=32969107 transcript=Pp3c1_22560V3.1
locus=Pp3c1_22560 annot-version=v3.3 Physcomitrella_patens
OG0000001
Pp3c1_10970V3.1.p pacid=32969678 transcript=Pp3c1_10970V3.1
locus=Pp3c1_10970 annot-version=v3.3 Physcomitrella_patens
OG0000001
Pp3c1_6700V3.1.p pacid=32969767 transcript=Pp3c1_6700V3.1
locus=Pp3c1_6700 annot-version=v3.3 Physcomitrella_patens
OG0000001
Pp3c1_11050V3.1.p pacid=32969979 transcript=Pp3c1_11050V3.1
locus=Pp3c1_11050 annot-version=v3.3 Physcomitrella_patens
OG0000001
Pp3c1_80V3.1.p pacid=32970385 transcript=Pp3c1_80V3.1 locus=Pp3c1_80
annot-version=v3.3 Physcomitrella_patens OG0000001

Pp3c1_17360V3.1.p pacid=32970459 transcript=Pp3c1_17360V3.1
locus=Pp3c1_17360 annot-version=v3.3 Physcomitrella_patens
OG0000001
Pp3c1_4210V3.1.p pacid=32971370 transcript=Pp3c1_4210V3.1
locus=Pp3c1_4210 annot-version=v3.3 Physcomitrella_patens
OG0000001
Pp3c12_25310V3.1.p pacid=32972668 transcript=Pp3c12_25310V3.1
locus=Pp3c12_25310 annot-version=v3.3 Physcomitrella_patens
OG0000001
Pp3c12_11940V3.1.p pacid=32972833 transcript=Pp3c12_11940V3.1
locus=Pp3c12_11940 annot-version=v3.3 Physcomitrella_patens
OG0000001
Pp3c12_22970V3.1.p pacid=32973100 transcript=Pp3c12_22970V3.1
locus=Pp3c12_22970 annot-version=v3.3 Physcomitrella_patens
OG0000001
Pp3c12_23280V3.1.p pacid=32973313 transcript=Pp3c12_23280V3.1
locus=Pp3c12_23280 annot-version=v3.3 Physcomitrella_patens
OG0000001
Pp3c12_23390V3.1.p pacid=32973743 transcript=Pp3c12_23390V3.1
locus=Pp3c12_23390 annot-version=v3.3 Physcomitrella_patens
OG0000001
Pp3c12_22990V3.1.p pacid=32974254 transcript=Pp3c12_22990V3.1
locus=Pp3c12_22990 annot-version=v3.3 Physcomitrella_patens
OG0000001
Pp3c12_16340V3.1.p pacid=32974491 transcript=Pp3c12_16340V3.1
locus=Pp3c12_16340 annot-version=v3.3 Physcomitrella_patens
OG0000001
Pp3c12_4430V3.1.p pacid=32974865 transcript=Pp3c12_4430V3.1
locus=Pp3c12_4430 annot-version=v3.3 Physcomitrella_patens
OG0000001
Pp3c6_19730V3.1.p pacid=32975492 transcript=Pp3c6_19730V3.1
locus=Pp3c6_19730 annot-version=v3.3 Physcomitrella_patens
OG0000001
Pp3c6_15930V3.1.p pacid=32975739 transcript=Pp3c6_15930V3.1
locus=Pp3c6_15930 annot-version=v3.3 Physcomitrella_patens
OG0000001
Pp3c6_21940V3.1.p pacid=32976116 transcript=Pp3c6_21940V3.1
locus=Pp3c6_21940 annot-version=v3.3 Physcomitrella_patens
OG0000001
Pp3c17_21540V3.1.p pacid=32907164 transcript=Pp3c17_21540V3.1
locus=Pp3c17_21540 annot-version=v3.3 Physcomitrella_patens
OG0000001
Pp3c6_22000V3.1.p pacid=32976486 transcript=Pp3c6_22000V3.1
locus=Pp3c6_22000 annot-version=v3.3 Physcomitrella_patens
OG0000001
Pp3c6_21440V3.1.p pacid=32976906 transcript=Pp3c6_21440V3.1
locus=Pp3c6_21440 annot-version=v3.3 Physcomitrella_patens
OG0000001
Pp3c6_16010V3.1.p pacid=32977038 transcript=Pp3c6_16010V3.1
locus=Pp3c6_16010 annot-version=v3.3 Physcomitrella_patens
OG0000001
Pp3c6_14960V3.1.p pacid=32978540 transcript=Pp3c6_14960V3.1
locus=Pp3c6_14960 annot-version=v3.3 Physcomitrella_patens
OG0000001

Pp3c25_12800V3.1.p pacid=32979127 transcript=Pp3c25_12800V3.1 locus=Pp3c25_12800 annot-version=v3.3 Physcomitrella_patens OG0000001
Pp3c25_4480V3.1.p pacid=32979348 transcript=Pp3c25_4480V3.1 locus=Pp3c25_4480 annot-version=v3.3 Physcomitrella_patens OG0000001
Pp3c25_470V3.1.p pacid=32980390 transcript=Pp3c25_470V3.1 locus=Pp3c25_470 annot-version=v3.3 Physcomitrella_patens OG0000001
Pp3c18_5730V3.1.p pacid=32981464 transcript=Pp3c18_5730V3.1 locus=Pp3c18_5730 annot-version=v3.3 Physcomitrella_patens OG0000001
Pp3c18_10870V3.1.p pacid=32981712 transcript=Pp3c18_10870V3.1 locus=Pp3c18_10870 annot-version=v3.3 Physcomitrella_patens OG0000001
Pp3c18_21010V3.1.p pacid=32981861 transcript=Pp3c18_21010V3.1 locus=Pp3c18_21010 annot-version=v3.3 Physcomitrella_patens OG0000001
Pp3c16_6190V3.1.p pacid=32983965 transcript=Pp3c16_6190V3.1 locus=Pp3c16_6190 annot-version=v3.3 Physcomitrella_patens OG0000001
Pp3c16_20190V3.1.p pacid=32986309 transcript=Pp3c16_20190V3.1 locus=Pp3c16_20190 annot-version=v3.3 Physcomitrella_patens OG0000001
Pp3c16_23370V3.1.p pacid=32986858 transcript=Pp3c16_23370V3.1 locus=Pp3c16_23370 annot-version=v3.3 Physcomitrella_patens OG0000001
Pp3c24_13300V3.1.p pacid=32908585 transcript=Pp3c24_13300V3.1 locus=Pp3c24_13300 annot-version=v3.3 Physcomitrella_patens OG0000001
Pp3c24_6450V3.1.p pacid=32909368 transcript=Pp3c24_6450V3.1 locus=Pp3c24_6450 annot-version=v3.3 Physcomitrella_patens OG0000001
Pp3c24_1080V3.1.p pacid=32909564 transcript=Pp3c24_1080V3.1 locus=Pp3c24_1080 annot-version=v3.3 Physcomitrella_patens OG0000001
Pp3c24_5390V3.1.p pacid=32910233 transcript=Pp3c24_5390V3.1 locus=Pp3c24_5390 annot-version=v3.3 Physcomitrella_patens OG0000001
Pp3c24_15950V3.1.p pacid=32910639 transcript=Pp3c24_15950V3.1 locus=Pp3c24_15950 annot-version=v3.3 Physcomitrella_patens OG0000001
Pp3c24_15190V3.1.p pacid=32910671 transcript=Pp3c24_15190V3.1 locus=Pp3c24_15190 annot-version=v3.3 Physcomitrella_patens OG0000001
Pp3c9_10550V3.1.p pacid=32911194 transcript=Pp3c9_10550V3.1 locus=Pp3c9_10550 annot-version=v3.3 Physcomitrella_patens OG0000001
Pp3c9_10110V3.1.p pacid=32913331 transcript=Pp3c9_10110V3.1 locus=Pp3c9_10110 annot-version=v3.3 Physcomitrella_patens OG0000001
Pp3c21_2960V3.1.p pacid=32915259 transcript=Pp3c21_2960V3.1 locus=Pp3c21_2960 annot-version=v3.3 Physcomitrella_patens OG0000001

Pp3c21_11200V3.1.p pacid=32915526 transcript=Pp3c21_11200V3.1 locus=Pp3c21_11200 annot-version=v3.3 Physcomitrella_patens OG0000001
Pp3c21_9500V3.1.p pacid=32916043 transcript=Pp3c21_9500V3.1 locus=Pp3c21_9500 annot-version=v3.3 Physcomitrella_patens OG0000001
Pp3c21_19160V3.1.p pacid=32916882 transcript=Pp3c21_19160V3.1 locus=Pp3c21_19160 annot-version=v3.3 Physcomitrella_patens OG0000001
Pp3c26_4970V3.1.p pacid=32917256 transcript=Pp3c26_4970V3.1 locus=Pp3c26_4970 annot-version=v3.3 Physcomitrella_patens OG0000001
Pp3c26_5010V3.1.p pacid=32918234 transcript=Pp3c26_5010V3.1 locus=Pp3c26_5010 annot-version=v3.3 Physcomitrella_patens OG0000001
Pp3c4_26070V3.1.p pacid=32918823 transcript=Pp3c4_26070V3.1 locus=Pp3c4_26070 annot-version=v3.3 Physcomitrella_patens OG0000001
Pp3c4_29120V3.1.p pacid=32918982 transcript=Pp3c4_29120V3.1 locus=Pp3c4_29120 annot-version=v3.3 Physcomitrella_patens OG0000001
Pp3c4_29300V3.1.p pacid=32919275 transcript=Pp3c4_29300V3.1 locus=Pp3c4_29300 annot-version=v3.3 Physcomitrella_patens OG0000001
Pp3c4_1760V3.1.p pacid=32919294 transcript=Pp3c4_1760V3.1 locus=Pp3c4_1760 annot-version=v3.3 Physcomitrella_patens OG0000001
Pp3c10_18600V3.1.p pacid=32901434 transcript=Pp3c10_18600V3.1 locus=Pp3c10_18600 annot-version=v3.3 Physcomitrella_patens OG0000001
Pp3c4_2690V3.1.p pacid=32920617 transcript=Pp3c4_2690V3.1 locus=Pp3c4_2690 annot-version=v3.3 Physcomitrella_patens OG0000001
Pp3c4_5010V3.1.p pacid=32921479 transcript=Pp3c4_5010V3.1 locus=Pp3c4_5010 annot-version=v3.3 Physcomitrella_patens OG0000001
Pp3c4_3520V3.1.p pacid=32921656 transcript=Pp3c4_3520V3.1 locus=Pp3c4_3520 annot-version=v3.3 Physcomitrella_patens OG0000001
Pp3c4_31950V3.1.p pacid=32922683 transcript=Pp3c4_31950V3.1 locus=Pp3c4_31950 annot-version=v3.3 Physcomitrella_patens OG0000001
Pp3c4_6580V3.1.p pacid=32922812 transcript=Pp3c4_6580V3.1 locus=Pp3c4_6580 annot-version=v3.3 Physcomitrella_patens OG0000001
Pp3c7_5570V3.1.p pacid=32922917 transcript=Pp3c7_5570V3.1 locus=Pp3c7_5570 annot-version=v3.3 Physcomitrella_patens OG0000001
Pp3c7_3320V3.1.p pacid=32923173 transcript=Pp3c7_3320V3.1 locus=Pp3c7_3320 annot-version=v3.3 Physcomitrella_patens OG0000001
Pp3c7_1830V3.1.p pacid=32923484 transcript=Pp3c7_1830V3.1 locus=Pp3c7_1830 annot-version=v3.3 Physcomitrella_patens OG0000001

Pp3c7_14840V3.1.p pacid=32923683 transcript=Pp3c7_14840V3.1 locus=Pp3c7_14840 annot-version=v3.3 Physcomitrella_patens OG0000001
Pp3c7_16420V3.1.p pacid=32924111 transcript=Pp3c7_16420V3.1 locus=Pp3c7_16420 annot-version=v3.3 Physcomitrella_patens OG0000001
Pp3c7_22100V3.1.p pacid=32924427 transcript=Pp3c7_22100V3.1 locus=Pp3c7_22100 annot-version=v3.3 Physcomitrella_patens OG0000001
Pp3c7_25750V3.1.p pacid=32924609 transcript=Pp3c7_25750V3.1 locus=Pp3c7_25750 annot-version=v3.3 Physcomitrella_patens OG0000001
AT2G34930.1 | disease resistance family protein / LRR family protein | Chr2:14737169-14739886 REVERSE LENGTH=905 | 201606 Arabidopsis_thaliana OG0000001
AT1G10850.1 | Leucine-rich repeat protein kinase family protein | Chr1:3612228-3614343 FORWARD LENGTH=663 | 201606 Arabidopsis_thaliana OG0000001
AT2G41820.1 | Leucine-rich repeat protein kinase family protein | Chr2:17447170-17449914 FORWARD LENGTH=890 | 201606 Arabidopsis_thaliana OG0000001
AT2G42800.1 | receptor like protein 29 | Chr2:17808157-17809545 REVERSE LENGTH=462 | 201606 Arabidopsis_thaliana OG0000001
AT1G11130.1 | Leucine-rich repeat protein kinase family protein | Chr1:3723135-3727178 FORWARD LENGTH=768 | 201606 Arabidopsis_thaliana OG0000001
AT2G45340.1 | Leucine-rich repeat protein kinase family protein | Chr2:18691739-18694466 FORWARD LENGTH=691 | 201606 Arabidopsis_thaliana OG0000001
AT3G02130.1 | receptor-like protein kinase 2 | Chr3:380726-384181 FORWARD LENGTH=1151 | 201606 Arabidopsis_thaliana OG0000001
AT3G02880.1 | Leucine-rich repeat protein kinase family protein | Chr3:634819-636982 FORWARD LENGTH=627 | 201606 Arabidopsis_thaliana OG0000001
AT1G12040.1 | leucine-rich repeat/extensin 1 | Chr1:4070160-4072394 FORWARD LENGTH=744 | 201606 Arabidopsis_thaliana OG0000001
AT3G05360.1 | receptor like protein 30 | Chr3:1530900-1533260 REVERSE LENGTH=786 | 201606 Arabidopsis_thaliana OG0000001
AT3G05370.1 | receptor like protein 31 | Chr3:1536134-1538716 REVERSE LENGTH=860 | 201606 Arabidopsis_thaliana OG0000001
AT3G05650.1 | receptor like protein 32 | Chr3:1645884-1648490 REVERSE LENGTH=868 | 201606 Arabidopsis_thaliana OG0000001
AT3G05660.1 | receptor like protein 33 | Chr3:1649258-1652001 REVERSE LENGTH=875 | 201606 Arabidopsis_thaliana OG0000001
AT3G08680.1 | Leucine-rich repeat protein kinase family protein | Chr3:2638591-2640590 FORWARD LENGTH=640 | 201606 Arabidopsis_thaliana OG0000001

AT1G12460.1 | Leucine-rich repeat protein kinase family protein | Chr1:4247703-4250444 FORWARD LENGTH=882 | 201606 Arabidopsis_thaliana OG0000001
AT3G11010.1 | receptor like protein 34 | Chr3:3450988-3453672 REVERSE LENGTH=894 | 201606 Arabidopsis_thaliana OG0000001
AT3G11080.1 | receptor like protein 35 | Chr3:3470481-3473312 FORWARD LENGTH=943 | 201606 Arabidopsis_thaliana OG0000001
AT3G11330.1 | plant intracellular ras group-related LRR 9 | Chr3:3552330-3554695 REVERSE LENGTH=499 | 201606 Arabidopsis_thaliana OG0000001
AT3G12145.1 | Leucine-rich repeat (LRR) family protein | Chr3:3874764-3876075 REVERSE LENGTH=325 | 201606 Arabidopsis_thaliana OG0000001
AT3G12610.1 | Leucine-rich repeat (LRR) family protein | Chr3:4006661-4007779 REVERSE LENGTH=372 | 201606 Arabidopsis_thaliana OG0000001
AT3G13065.1 | STRUBBELIG-receptor family 4 | Chr3:4187510-4190863 FORWARD LENGTH=687 | 201606 Arabidopsis_thaliana OG0000001
AT3G13380.1 | BRI1-like 3 | Chr3:4347240-4350734 FORWARD LENGTH=1164 | 201606 Arabidopsis_thaliana OG0000001
AT1G12970.1 | plant intracellular ras group-related LRR 3 | Chr1:4423727-4425632 FORWARD LENGTH=464 | 201606 Arabidopsis_thaliana OG0000001
AT3G15410.1 | Leucine-rich repeat (LRR) family protein | Chr3:5203380-5207279 FORWARD LENGTH=584 | 201606 Arabidopsis_thaliana OG0000001
AT1G13230.1 | Leucine-rich repeat (LRR) family protein | Chr1:4520679-4522439 FORWARD LENGTH=424 | 201606 Arabidopsis_thaliana OG0000001
AT3G17640.1 | Leucine-rich repeat (LRR) family protein | Chr3:6032393-6033583 FORWARD LENGTH=396 | 201606 Arabidopsis_thaliana OG0000001
AT3G19020.1 | Leucine-rich repeat (LRR) family protein | Chr3:6559174-6562044 REVERSE LENGTH=956 | 201606 Arabidopsis_thaliana OG0000001
AT3G19320.1 | Leucine-rich repeat (LRR) family protein | Chr3:6696395-6698073 REVERSE LENGTH=493 | 201606 Arabidopsis_thaliana OG0000001
AT3G19700.1 | Leucine-rich repeat protein kinase family protein | Chr3:6843662-6846791 FORWARD LENGTH=991 | 201606 Arabidopsis_thaliana OG0000001
AT3G20820.1 | Leucine-rich repeat (LRR) family protein | Chr3:7280930-7282027 FORWARD LENGTH=365 | 201606 Arabidopsis_thaliana OG0000001
AT3G22800.1 | Leucine-rich repeat (LRR) family protein | Chr3:8063063-8064475 REVERSE LENGTH=470 | 201606 Arabidopsis_thaliana OG0000001
AT3G23010.1 | receptor like protein 36 | Chr3:8174858-8176645 FORWARD LENGTH=595 | 201606 Arabidopsis_thaliana OG0000001
AT3G23110.1 | receptor like protein 37 | Chr3:8222364-8224871

REVERSE LENGTH=835 | 201606	Arabidopsis_thaliana
OG0000001
AT3G23120.1 | receptor like protein 38 | Chr3:8227222-8229576
REVERSE LENGTH=784 | 201606	Arabidopsis_thaliana
OG0000001
AT1G13910.1 | Leucine-rich repeat (LRR) family protein |
Chr1:4755955-4757814 FORWARD LENGTH=330 | 201606
Arabidopsis_thaliana	OG0000001
AT3G24240.1 | Leucine-rich repeat receptor-like protein kinase
family protein | Chr3:8780551-8784150 FORWARD LENGTH=1141 | 201606
Arabidopsis_thaliana	OG0000001
AT3G24480.1 | Leucine-rich repeat (LRR) family protein |
Chr3:8901154-8902638 REVERSE LENGTH=494 | 201606
Arabidopsis_thaliana	OG0000001
AT3G24900.1 | receptor like protein 39 | Chr3:9099183-9101837
REVERSE LENGTH=884 | 201606	Arabidopsis_thaliana
OG0000001
AT3G24982.1 | receptor like protein 40 | Chr3:9106157-9108937
REVERSE LENGTH=915 | 201606	Arabidopsis_thaliana
OG0000001
AT3G25010.1 | receptor like protein 41 | Chr3:9110103-9112748
REVERSE LENGTH=881 | 201606	Arabidopsis_thaliana
OG0000001
AT3G25020.1 | receptor like protein 42 | Chr3:9116868-9119540
REVERSE LENGTH=890 | 201606	Arabidopsis_thaliana
OG0000001
AT3G25670.1 | Leucine-rich repeat (LRR) family protein |
Chr3:9344532-9346301 REVERSE LENGTH=475 | 201606
Arabidopsis_thaliana	OG0000001
AT3G26500.1 | plant intracellular ras group-related LRR 2 |
Chr3:9708195-9709944 REVERSE LENGTH=471 | 201606
Arabidopsis_thaliana	OG0000001
AT1G14390.1 | Leucine-rich repeat protein kinase family protein |
Chr1:4924277-4926794 FORWARD LENGTH=728 | 201606
Arabidopsis_thaliana	OG0000001
AT3G28040.1 | Leucine-rich receptor-like protein kinase family
protein | Chr3:10435139-10438268 FORWARD LENGTH=1016 | 201606
Arabidopsis_thaliana	OG0000001
AT3G28890.1 | receptor like protein 43 | Chr3:10896706-10898841
REVERSE LENGTH=711 | 201606	Arabidopsis_thaliana
OG0000001
AT3G43740.1 | Leucine-rich repeat (LRR) family protein |
Chr3:15644127-15645446 FORWARD LENGTH=218 | 201606
Arabidopsis_thaliana	OG0000001
AT3G47090.1 | Leucine-rich repeat protein kinase family protein |
Chr3:17341512-17344645 REVERSE LENGTH=1009 | 201606
Arabidopsis_thaliana	OG0000001
AT3G47110.1 | Leucine-rich repeat protein kinase family protein |
Chr3:17347103-17350296 REVERSE LENGTH=1025 | 201606
Arabidopsis_thaliana	OG0000001
AT3G47570.1 | Leucine-rich repeat protein kinase family protein |
Chr3:17527611-17530748 FORWARD LENGTH=1010 | 201606
Arabidopsis_thaliana	OG0000001
AT3G47580.1 | Leucine-rich repeat protein kinase family protein |

Chr3:17532687-17535810 FORWARD LENGTH=1011 | 201606
Arabidopsis_thaliana OG0000001
AT3G49670.1 | Leucine-rich receptor-like protein kinase family protein | Chr3:18417741-18420836 FORWARD LENGTH=1002 | 201606
Arabidopsis_thaliana OG0000001
AT3G49750.1 | receptor like protein 44 | Chr3:18450604-18451428 REVERSE LENGTH=274 | 201606 Arabidopsis_thaliana
OG0000001
AT3G51740.1 | inflorescence meristem receptor-like kinase 2 | Chr3:19189248-19191842 FORWARD LENGTH=836 | 201606
Arabidopsis_thaliana OG0000001
AT3G53240.1 | receptor like protein 45 | Chr3:19735927-19739047 FORWARD LENGTH=891 | 201606 Arabidopsis_thaliana
OG0000001
AT3G53590.1 | LRR receptor-like Serine/Threonine-kinase | Chr3:19867379-19872542 REVERSE LENGTH=944 | 201606
Arabidopsis_thaliana OG0000001
AT3G56100.1 | meristematic receptor-like kinase | Chr3:20817074-20819517 REVERSE LENGTH=784 | 201606
Arabidopsis_thaliana OG0000001
AT3G56370.1 | Leucine-rich repeat protein kinase family protein | Chr3:20899403-20902390 REVERSE LENGTH=964 | 201606
Arabidopsis_thaliana OG0000001
AT3G59510.1 | Leucine-rich repeat (LRR) family protein | Chr3:21988453-21989712 REVERSE LENGTH=419 | 201606
Arabidopsis_thaliana OG0000001
AT4G03010.1 | RNI-like superfamily protein | Chr4:1329952-1331139 FORWARD LENGTH=395 | 201606 Arabidopsis_thaliana
OG0000001
AT4G04220.1 | receptor like protein 46 | Chr4:2033427-2035946 FORWARD LENGTH=811 | 201606 Arabidopsis_thaliana
OG0000001
AT1G17230.1 | Leucine-rich receptor-like protein kinase family protein | Chr1:5891375-5894855 FORWARD LENGTH=1101 | 201606
Arabidopsis_thaliana OG0000001
AT1G17240.1 | receptor like protein 2 | Chr1:5896528-5898717 REVERSE LENGTH=729 | 201606 Arabidopsis_thaliana OG0000001
AT1G17250.1 | receptor like protein 3 | Chr1:5901169-5903439 REVERSE LENGTH=756 | 201606 Arabidopsis_thaliana OG0000001
AT4G06744.1 | Leucine-rich repeat (LRR) family protein | Chr4:4070870-4072084 REVERSE LENGTH=404 | 201606
Arabidopsis_thaliana OG0000001
AT4G08850.1 | Leucine-rich repeat receptor-like protein kinase family protein | Chr4:5636693-5640496 REVERSE LENGTH=1045 | 201606
Arabidopsis_thaliana OG0000001
AT1G17750.1 | PEP1 receptor 2 | Chr1:6106656-6110008 FORWARD LENGTH=1088 | 201606 Arabidopsis_thaliana OG0000001
AT4G13340.1 | Leucine-rich repeat (LRR) family protein | Chr4:7758610-7760892 FORWARD LENGTH=760 | 201606
Arabidopsis_thaliana OG0000001
AT4G13810.1 | receptor like protein 47 | Chr4:8005062-8006936 REVERSE LENGTH=530 | 201606 Arabidopsis_thaliana
OG0000001
AT4G13820.1 | Leucine-rich repeat (LRR) family protein |

Chr4:8008535-8010694 REVERSE LENGTH=719 | 201606
Arabidopsis_thaliana   OG0000001
AT4G13880.1 | receptor like protein 48 | Chr4:8026151-8028614
FORWARD LENGTH=725 | 201606   Arabidopsis_thaliana
OG0000001
AT4G13920.1 | receptor like protein 50 | Chr4:8043861-8046536
FORWARD LENGTH=891 | 201606   Arabidopsis_thaliana
OG0000001
AT4G16162.1 | Leucine-rich repeat (LRR) family protein |
Chr4:9159766-9161605 REVERSE LENGTH=176 | 201606
Arabidopsis_thaliana   OG0000001
AT4G18670.1 | Leucine-rich repeat (LRR) family protein |
Chr4:10275918-10278491 REVERSE LENGTH=857 | 201606
Arabidopsis_thaliana   OG0000001
AT4G18760.1 | receptor like protein 51 | Chr4:10308163-10309458
REVERSE LENGTH=431 | 201606   Arabidopsis_thaliana
OG0000001
AT4G20140.1 | Leucine-rich repeat transmembrane protein kinase |
Chr4:10884220-10888045 FORWARD LENGTH=1249 | 201606
Arabidopsis_thaliana   OG0000001
AT4G20270.1 | Leucine-rich receptor-like protein kinase family
protein | Chr4:10949822-10952924 FORWARD LENGTH=992 | 201606
Arabidopsis_thaliana   OG0000001
AT4G20940.1 | Leucine-rich receptor-like protein kinase family
protein | Chr4:11202728-11206038 FORWARD LENGTH=1037 | 201606
Arabidopsis_thaliana   OG0000001
AT4G22730.1 | Leucine-rich repeat protein kinase family protein |
Chr4:11941384-11943696 FORWARD LENGTH=688 | 201606
Arabidopsis_thaliana   OG0000001
AT4G26050.1 | plant intracellular ras group-related LRR 8 |
Chr4:13210522-13213149 FORWARD LENGTH=383 | 201606
Arabidopsis_thaliana   OG0000001
AT4G26540.1 | Leucine-rich repeat receptor-like protein kinase
family protein | Chr4:13394673-13398028 REVERSE LENGTH=1091 | 201606
        Arabidopsis_thaliana   OG0000001
AT4G28380.1 | Leucine-rich repeat (LRR) family protein |
Chr4:14039756-14040931 REVERSE LENGTH=391 | 201606
Arabidopsis_thaliana   OG0000001
AT4G28490.1 | Leucine-rich receptor-like protein kinase family
protein | Chr4:14077894-14080965 FORWARD LENGTH=999 | 201606
Arabidopsis_thaliana   OG0000001
AT4G28560.1 | ROP-interactive CRIB motif-containing protein 7 |
Chr4:14116015-14117367 REVERSE LENGTH=450 | 201606
Arabidopsis_thaliana   OG0000001
AT4G28650.1 | Leucine-rich repeat transmembrane protein kinase
family protein | Chr4:14144155-14147276 REVERSE LENGTH=1013 | 201606
        Arabidopsis_thaliana   OG0000001
AT4G29240.1 | Leucine-rich repeat (LRR) family protein |
Chr4:14418826-14420073 FORWARD LENGTH=415 | 201606
Arabidopsis_thaliana   OG0000001
AT4G29880.1 | plant intracellular ras group-related LRR 7 |
Chr4:14607078-14608379 REVERSE LENGTH=373 | 201606
Arabidopsis_thaliana   OG0000001
AT4G33970.1 | Leucine-rich repeat (LRR) family protein |

Chr4:16279795-16281894 REVERSE LENGTH=699 | 201606
Arabidopsis_thaliana OG0000001
AT4G34220.1 | Leucine-rich repeat protein kinase family protein |
Chr4:16381653-16384054 REVERSE LENGTH=757 | 201606
Arabidopsis_thaliana OG0000001
AT4G35470.1 | plant intracellular ras group-related LRR 4 |
Chr4:16846531-16848448 FORWARD LENGTH=549 | 201606
Arabidopsis_thaliana OG0000001
AT4G36180.1 | Leucine-rich receptor-like protein kinase family
protein | Chr4:17120209-17123698 REVERSE LENGTH=1136 | 201606
Arabidopsis_thaliana OG0000001
AT4G37250.1 | Leucine-rich repeat protein kinase family protein |
Chr4:17527789-17530191 REVERSE LENGTH=768 | 201606
Arabidopsis_thaliana OG0000001
AT4G39270.1 | Leucine-rich repeat protein kinase family protein |
Chr4:18276874-18279710 FORWARD LENGTH=864 | 201606
Arabidopsis_thaliana OG0000001
AT4G39400.1 | Leucine-rich receptor-like protein kinase family
protein | Chr4:18324826-18328416 FORWARD LENGTH=1196 | 201606
Arabidopsis_thaliana OG0000001
AT5G01890.1 | Leucine-rich receptor-like protein kinase family
protein | Chr5:341661-344650 REVERSE LENGTH=967 | 201606
Arabidopsis_thaliana OG0000001
AT5G01950.1 | Leucine-rich repeat protein kinase family protein |
Chr5:365040-369532 REVERSE LENGTH=951 | 201606
Arabidopsis_thaliana OG0000001
AT5G05160.1 | Leucine-rich repeat protein kinase family protein |
Chr5:1528000-1530017 FORWARD LENGTH=640 | 201606
Arabidopsis_thaliana OG0000001
AT5G05850.1 | plant intracellular ras group-related LRR 1 |
Chr5:1762691-1764609 REVERSE LENGTH=506 | 201606
Arabidopsis_thaliana OG0000001
AT5G06860.1 | polygalacturonase inhibiting protein 1 |
Chr5:2132373-2133434 FORWARD LENGTH=330 | 201606
Arabidopsis_thaliana OG0000001
AT5G06870.1 | polygalacturonase inhibiting protein 2 |
Chr5:2133941-2135016 FORWARD LENGTH=330 | 201606
Arabidopsis_thaliana OG0000001
AT5G06940.1 | Leucine-rich repeat receptor-like protein kinase
family protein | Chr5:2148078-2150771 REVERSE LENGTH=872 | 201606
Arabidopsis_thaliana OG0000001
AT5G07150.1 | Leucine-rich repeat protein kinase family protein |
Chr5:2215820-2217983 FORWARD LENGTH=555 | 201606
Arabidopsis_thaliana OG0000001
AT5G07180.1 | ERECTA-like 2 | Chr5:2227787-2233232 REVERSE
LENGTH=967 | 201606 Arabidopsis_thaliana OG0000001
AT5G07280.1 | Leucine-rich repeat transmembrane protein kinase |
Chr5:2285088-2288666 FORWARD LENGTH=1192 | 201606
Arabidopsis_thaliana OG0000001
AT5G07910.1 | Leucine-rich repeat (LRR) family protein |
Chr5:2521937-2523769 REVERSE LENGTH=262 | 201606
Arabidopsis_thaliana OG0000001
AT5G10020.1 | Leucine-rich receptor-like protein kinase family
protein | Chr5:3133514-3136949 FORWARD LENGTH=1048 | 201606

Arabidopsis_thaliana	OG0000001
AT5G12940.1 | Leucine-rich repeat (LRR) family protein | Chr5:4087782-4088897 FORWARD LENGTH=371 | 201606	Arabidopsis_thaliana	OG0000001
AT5G14210.1 | Leucine-rich repeat protein kinase family protein | Chr5:4578503-4581374 REVERSE LENGTH=775 | 201606	Arabidopsis_thaliana	OG0000001
AT5G16590.1 | Leucine-rich repeat protein kinase family protein | Chr5:5431862-5433921 FORWARD LENGTH=625 | 201606	Arabidopsis_thaliana	OG0000001
AT5G20480.1 | EF-TU receptor | Chr5:6922497-6925679 FORWARD LENGTH=1031 | 201606	Arabidopsis_thaliana	OG0000001
AT5G21090.1 | Leucine-rich repeat (LRR) family protein | Chr5:7164758-7166904 FORWARD LENGTH=218 | 201606	Arabidopsis_thaliana	OG0000001
AT5G23400.1 | Leucine-rich repeat (LRR) family protein | Chr5:7880603-7882372 FORWARD LENGTH=589 | 201606	Arabidopsis_thaliana	OG0000001
AT5G24100.1 | Leucine-rich repeat protein kinase family protein | Chr5:8149216-8151191 FORWARD LENGTH=614 | 201606	Arabidopsis_thaliana	OG0000001
AT5G25550.1 | Leucine-rich repeat (LRR) family protein | Chr5:8894179-8895480 FORWARD LENGTH=433 | 201606	Arabidopsis_thaliana	OG0000001
AT5G25910.1 | receptor like protein 52 | Chr5:9038860-9041377 FORWARD LENGTH=811 | 201606	Arabidopsis_thaliana	OG0000001
AT5G25930.1 | kinase family with leucine-rich repeat domain-containing protein | Chr5:9050880-9053978 FORWARD LENGTH=1005 | 201606	Arabidopsis_thaliana	OG0000001
AT5G27060.1 | receptor like protein 53 | Chr5:9522534-9525407 REVERSE LENGTH=957 | 201606	Arabidopsis_thaliana	OG0000001
AT5G37450.1 | Leucine-rich repeat protein kinase family protein | Chr5:14852801-14857098 REVERSE LENGTH=935 | 201606	Arabidopsis_thaliana	OG0000001
AT5G39390.1 | Leucine-rich repeat protein kinase family protein | Chr5:15763715-15765469 REVERSE LENGTH=502 | 201606	Arabidopsis_thaliana	OG0000001
AT5G40170.1 | receptor like protein 54 | Chr5:16065179-16067557 REVERSE LENGTH=792 | 201606	Arabidopsis_thaliana	OG0000001
AT5G44700.1 | Leucine-rich repeat transmembrane protein kinase | Chr5:18033049-18036894 REVERSE LENGTH=1252 | 201606	Arabidopsis_thaliana	OG0000001
AT5G45770.1 | receptor like protein 55 | Chr5:18563568-18564845 FORWARD LENGTH=425 | 201606	Arabidopsis_thaliana	OG0000001
AT5G46330.1 | Leucine-rich receptor-like protein kinase family protein | Chr5:18791802-18795407 FORWARD LENGTH=1173 | 201606	Arabidopsis_thaliana	OG0000001
AT5G48940.1 | Leucine-rich repeat transmembrane protein kinase family protein | Chr5:19839785-19843744 FORWARD LENGTH=1135 | 201606	Arabidopsis_thaliana	OG0000001

AT5G49290.1 | receptor like protein 56 | Chr5:19980195-19983869 FORWARD LENGTH=908 | 201606 Arabidopsis_thaliana OG0000001
AT5G49660.1 | Leucine-rich repeat transmembrane protein kinase family protein | Chr5:20161401-20164534 REVERSE LENGTH=966 | 201606 Arabidopsis_thaliana OG0000001
AT5G49750.1 | Leucine-rich repeat (LRR) family protein | Chr5:20210878-20213734 FORWARD LENGTH=493 | 201606 Arabidopsis_thaliana OG0000001
AT5G51350.1 | Leucine-rich repeat transmembrane protein kinase family protein | Chr5:20867860-20870621 REVERSE LENGTH=895 | 201606 Arabidopsis_thaliana OG0000001
AT5G51560.1 | Leucine-rich repeat protein kinase family protein | Chr5:20945807-20948613 FORWARD LENGTH=680 | 201606 Arabidopsis_thaliana OG0000001
AT5G53320.1 | Leucine-rich repeat protein kinase family protein | Chr5:21636453-21638337 REVERSE LENGTH=601 | 201606 Arabidopsis_thaliana OG0000001
AT5G53890.1 | phytosylfokine-alpha receptor 2 | Chr5:21877235-21880345 FORWARD LENGTH=1036 | 201606 Arabidopsis_thaliana OG0000001
AT1G03440.1 | Leucine-rich repeat (LRR) family protein | Chr1:852681-853874 FORWARD LENGTH=397 | 201606 Arabidopsis_thaliana OG0000001
AT5G56040.1 | Leucine-rich receptor-like protein kinase family protein | Chr5:22695050-22697911 FORWARD LENGTH=953 | 201606 Arabidopsis_thaliana OG0000001
AT5G58150.1 | Leucine-rich repeat protein kinase family protein | Chr5:23530216-23532573 REVERSE LENGTH=785 | 201606 Arabidopsis_thaliana OG0000001
AT5G61240.1 | Leucine-rich repeat (LRR) family protein | Chr5:24629485-24631958 FORWARD LENGTH=326 | 201606 Arabidopsis_thaliana OG0000001
AT5G61480.1 | Leucine-rich repeat protein kinase family protein | Chr5:24724541-24727842 REVERSE LENGTH=1041 | 201606 Arabidopsis_thaliana OG0000001
AT5G62230.1 | ERECTA-like 1 | Chr5:24996433-25002130 FORWARD LENGTH=966 | 201606 Arabidopsis_thaliana OG0000001
AT5G63410.1 | Leucine-rich repeat protein kinase family protein | Chr5:25395173-25397768 REVERSE LENGTH=680 | 201606 Arabidopsis_thaliana OG0000001
AT5G63930.1 | Leucine-rich repeat protein kinase family protein | Chr5:25583006-25586392 FORWARD LENGTH=1102 | 201606 Arabidopsis_thaliana OG0000001
AT5G65700.1 | Leucine-rich receptor-like protein kinase family protein | Chr5:26281826-26284945 FORWARD LENGTH=1003 | 201606 Arabidopsis_thaliana OG0000001
AT5G65710.1 | HAESA-like 2 | Chr5:26292372-26295440 FORWARD LENGTH=993 | 201606 Arabidopsis_thaliana OG0000001
AT5G65830.1 | receptor like protein 57 | Chr5:26342396-26343235 REVERSE LENGTH=279 | 201606 Arabidopsis_thaliana OG0000001
AT5G66330.1 | Leucine-rich repeat (LRR) family protein | Chr5:26500531-26501787 REVERSE LENGTH=418 | 201606

Arabidopsis_thaliana	OG0000001
AT5G67280.1 | receptor-like kinase | Chr5:26842430-26845126 REVERSE LENGTH=751 | 201606	Arabidopsis_thaliana	OG0000001
AT1G28440.1 | HAESA-like 1 | Chr1:9996914-10000171 FORWARD LENGTH=996 | 201606	Arabidopsis_thaliana	OG0000001
AT1G29730.1 | Leucine-rich repeat transmembrane protein kinase | Chr1:10400710-10405874 REVERSE LENGTH=969 | 201606	Arabidopsis_thaliana	OG0000001
AT1G29740.1 | Leucine-rich repeat transmembrane protein kinase | Chr1:10407379-10412997 REVERSE LENGTH=1078 | 201606	Arabidopsis_thaliana	OG0000001
AT1G33590.1 | Leucine-rich repeat (LRR) family protein | Chr1:12177788-12179221 FORWARD LENGTH=477 | 201606	Arabidopsis_thaliana	OG0000001
AT1G33600.1 | Leucine-rich repeat (LRR) family protein | Chr1:12180776-12182212 FORWARD LENGTH=478 | 201606	Arabidopsis_thaliana	OG0000001
AT1G33610.1 | Leucine-rich repeat (LRR) family protein | Chr1:12188910-12190346 FORWARD LENGTH=478 | 201606	Arabidopsis_thaliana	OG0000001
AT1G33612.1 | Leucine-rich repeat (LRR) family protein | Chr1:12191312-12192679 FORWARD LENGTH=455 | 201606	Arabidopsis_thaliana	OG0000001
AT1G33670.1 | Leucine-rich repeat (LRR) family protein | Chr1:12201963-12203330 FORWARD LENGTH=455 | 201606	Arabidopsis_thaliana	OG0000001
AT1G34110.1 | Leucine-rich receptor-like protein kinase family protein | Chr1:12417331-12421246 REVERSE LENGTH=1072 | 201606	Arabidopsis_thaliana	OG0000001
AT1G34290.1 | receptor like protein 5 | Chr1:12498000-12498800 FORWARD LENGTH=266 | 201606	Arabidopsis_thaliana	OG0000001
AT1G34420.1 | leucine-rich repeat transmembrane protein kinase family protein | Chr1:12584587-12587570 FORWARD LENGTH=966 | 201606	Arabidopsis_thaliana	OG0000001
AT1G35710.1 | kinase family with leucine-rich repeat domain-containing protein | Chr1:13220940-13224386 FORWARD LENGTH=1120 | 201606	Arabidopsis_thaliana	OG0000001
AT1G45616.1 | receptor like protein 6 | Chr1:17183550-17186534 REVERSE LENGTH=994 | 201606	Arabidopsis_thaliana	OG0000001
AT1G47890.1 | receptor like protein 7 | Chr1:17643976-17647035 FORWARD LENGTH=1019 | 201606	Arabidopsis_thaliana	OG0000001
AT1G49490.1 | Leucine-rich repeat (LRR) family protein | Chr1:18317563-18320106 REVERSE LENGTH=847 | 201606	Arabidopsis_thaliana	OG0000001
AT1G49750.1 | Leucine-rich repeat (LRR) family protein | Chr1:18411177-18412779 REVERSE LENGTH=494 | 201606	Arabidopsis_thaliana	OG0000001
AT1G50610.1 | Leucine-rich repeat protein kinase family protein | Chr1:18742171-18744501 FORWARD LENGTH=686 | 201606	Arabidopsis_thaliana	OG0000001
AT1G54470.1 | resistance to peronospora parasitica protein |

Chr1:20344738-20347339 FORWARD LENGTH=457 | 201606
Arabidopsis_thaliana   OG0000001
AT1G54475.1 | LRR receptor-like Serine/Threonine-kinase |
Chr1:20348403-20349032 FORWARD LENGTH=209 | 201606
Arabidopsis_thaliana   OG0000001
AT1G55610.1 | BRI1 like | Chr1:20779874-20783374 REVERSE LENGTH=1166
| 201606 Arabidopsis_thaliana   OG0000001
AT1G58190.1 | receptor like protein 9 | Chr1:21540720-21544330
FORWARD LENGTH=932 | 201606   Arabidopsis_thaliana
OG0000001
AT1G60630.1 | Leucine-rich repeat protein kinase family protein |
Chr1:22334754-22336785 REVERSE LENGTH=652 | 201606
Arabidopsis_thaliana   OG0000001
AT1G62440.1 | leucine-rich repeat/extensin 2 |
Chr1:23112933-23115293 FORWARD LENGTH=786 | 201606
Arabidopsis_thaliana   OG0000001
AT1G62950.1 | leucine-rich repeat transmembrane protein kinase
family protein | Chr1:23315294-23318061 FORWARD LENGTH=890 | 201606
Arabidopsis_thaliana   OG0000001
AT1G64210.1 | Leucine-rich repeat protein kinase family protein |
Chr1:23831033-23832863 FORWARD LENGTH=587 | 201606
Arabidopsis_thaliana   OG0000001
AT1G65380.1 | Leucine-rich repeat (LRR) family protein |
Chr1:24286943-24289105 FORWARD LENGTH=720 | 201606
Arabidopsis_thaliana   OG0000001
AT1G66830.1 | Leucine-rich repeat protein kinase family protein |
Chr1:24930700-24932834 REVERSE LENGTH=685 | 201606
Arabidopsis_thaliana   OG0000001
AT1G67510.1 | Leucine-rich repeat protein kinase family protein |
Chr1:25297477-25300184 REVERSE LENGTH=719 | 201606
Arabidopsis_thaliana   OG0000001
AT1G68780.1 | RNI-like superfamily protein | Chr1:25831881-25833335
REVERSE LENGTH=432 | 201606   Arabidopsis_thaliana
OG0000001
AT1G69990.1 | Leucine-rich repeat protein kinase family protein |
Chr1:26360235-26362010 REVERSE LENGTH=591 | 201606
Arabidopsis_thaliana   OG0000001
AT1G71390.1 | receptor like protein 11 | Chr1:26906453-26908807
FORWARD LENGTH=784 | 201606   Arabidopsis_thaliana
OG0000001
AT1G71400.1 | receptor like protein 12 | Chr1:26909905-26912448
FORWARD LENGTH=847 | 201606   Arabidopsis_thaliana
OG0000001
AT1G72180.1 | Leucine-rich receptor-like protein kinase family
protein | Chr1:27164074-27167204 FORWARD LENGTH=977 | 201606
Arabidopsis_thaliana   OG0000001
AT1G72300.1 | Leucine-rich receptor-like protein kinase family
protein | Chr1:27217679-27220966 REVERSE LENGTH=1095 | 201606
Arabidopsis_thaliana   OG0000001
AT1G72460.1 | Leucine-rich repeat protein kinase family protein |
Chr1:27279510-27281533 FORWARD LENGTH=644 | 201606
Arabidopsis_thaliana   OG0000001
AT1G73066.1 | Leucine-rich repeat family protein |
Chr1:27481785-27483581 FORWARD LENGTH=598 | 201606

Arabidopsis_thaliana   OG0000001
AT1G73080.1 | PEP1 receptor 1 | Chr1:27484513-27488021 FORWARD LENGTH=1123 | 201606   Arabidopsis_thaliana   OG0000001
AT1G74170.1 | receptor like protein 13 | Chr1:27891555-27895441 REVERSE LENGTH=1000 | 201606   Arabidopsis_thaliana   OG0000001
AT1G74180.1 | receptor like protein 14 | Chr1:27897197-27900908 REVERSE LENGTH=976 | 201606   Arabidopsis_thaliana   OG0000001
AT1G74190.1 | receptor like protein 15 | Chr1:27902590-27906158 REVERSE LENGTH=965 | 201606   Arabidopsis_thaliana   OG0000001
AT1G74200.1 | receptor like protein 16 | Chr1:27907739-27908647 REVERSE LENGTH=302 | 201606   Arabidopsis_thaliana   OG0000001
AT1G74360.1 | Leucine-rich repeat protein kinase family protein | Chr1:27954299-27957911 FORWARD LENGTH=1106 | 201606   Arabidopsis_thaliana   OG0000001
AT1G75640.1 | Leucine-rich receptor-like protein kinase family protein | Chr1:28403600-28407022 REVERSE LENGTH=1140 | 201606   Arabidopsis_thaliana   OG0000001
AT1G75820.1 | Leucine-rich receptor-like protein kinase family protein | Chr1:28463631-28466652 REVERSE LENGTH=980 | 201606   Arabidopsis_thaliana   OG0000001
AT1G07390.1 | receptor like protein 1 | Chr1:2269893-2274654 FORWARD LENGTH=1034 | 201606   Arabidopsis_thaliana   OG0000001
AT1G80080.1 | Leucine-rich repeat (LRR) family protein | Chr1:30128073-30129563 REVERSE LENGTH=496 | 201606   Arabidopsis_thaliana   OG0000001
AT2G01950.1 | BRI1-like 2 | Chr2:440805-444236 REVERSE LENGTH=1143 | 201606   Arabidopsis_thaliana   OG0000001
AT2G02220.1 | phytosulfokin receptor 1 | Chr2:584098-587124 REVERSE LENGTH=1008 | 201606   Arabidopsis_thaliana   OG0000001
AT2G02780.1 | Leucine-rich repeat protein kinase family protein | Chr2:781932-784646 REVERSE LENGTH=742 | 201606   Arabidopsis_thaliana   OG0000001
AT2G13800.1 | somatic embryogenesis receptor-like kinase 5 | Chr2:5753276-5757065 FORWARD LENGTH=601 | 201606   Arabidopsis_thaliana   OG0000001
AT2G15042.1 | Leucine-rich repeat (LRR) family protein | Chr2:6511058-6512335 FORWARD LENGTH=425 | 201606   Arabidopsis_thaliana   OG0000001
AT2G15080.1 | receptor like protein 19 | Chr2:6533764-6536715 FORWARD LENGTH=983 | 201606   Arabidopsis_thaliana   OG0000001
AT2G15300.1 | Leucine-rich repeat protein kinase family protein | Chr2:6649630-6652010 FORWARD LENGTH=744 | 201606   Arabidopsis_thaliana   OG0000001
AT2G15320.1 | Leucine-rich repeat (LRR) family protein | Chr2:6666527-6667675 REVERSE LENGTH=382 | 201606   Arabidopsis_thaliana   OG0000001
AT2G15880.1 | Leucine-rich repeat (LRR) family protein | Chr2:6918039-6920319 REVERSE LENGTH=727 | 201606   Arabidopsis_thaliana   OG0000001

AT2G16250.1 | Leucine-rich repeat protein kinase family protein | Chr2:7039682-7042933 REVERSE LENGTH=915 | 201606 Arabidopsis_thaliana OG0000001
AT1G08590.1 | Leucine-rich receptor-like protein kinase family protein | Chr1:2718859-2721948 FORWARD LENGTH=1029 | 201606 Arabidopsis_thaliana OG0000001
AT2G17440.1 | plant intracellular ras group-related LRR 5 | Chr2:7571331-7573406 FORWARD LENGTH=526 | 201606 Arabidopsis_thaliana OG0000001
AT2G19330.1 | plant intracellular ras group-related LRR 6 | Chr2:8372947-8374453 FORWARD LENGTH=380 | 201606 Arabidopsis_thaliana OG0000001
AT2G19780.1 | Leucine-rich repeat (LRR) family protein | Chr2:8522831-8524039 REVERSE LENGTH=402 | 201606 Arabidopsis_thaliana OG0000001
AT2G23300.1 | Leucine-rich repeat protein kinase family protein | Chr2:9914608-9917130 FORWARD LENGTH=773 | 201606 Arabidopsis_thaliana OG0000001
AT2G24130.1 | Leucine-rich receptor-like protein kinase family protein | Chr2:10258148-10261220 FORWARD LENGTH=980 | 201606 Arabidopsis_thaliana OG0000001
AT2G24230.1 | Leucine-rich repeat protein kinase family protein | Chr2:10301979-10304540 REVERSE LENGTH=853 | 201606 Arabidopsis_thaliana OG0000001
AT2G25440.1 | receptor like protein 20 | Chr2:10826735-10829402 FORWARD LENGTH=671 | 201606 Arabidopsis_thaliana OG0000001
AT2G25470.1 | receptor like protein 21 | Chr2:10838420-10841881 FORWARD LENGTH=935 | 201606 Arabidopsis_thaliana OG0000001
AT2G25790.1 | Leucine-rich receptor-like protein kinase family protein | Chr2:11000631-11004031 FORWARD LENGTH=960 | 201606 Arabidopsis_thaliana OG0000001
AT2G26330.1 | Leucine-rich receptor-like protein kinase family protein | Chr2:11208367-11213895 REVERSE LENGTH=976 | 201606 Arabidopsis_thaliana OG0000001
AT2G26380.1 | Leucine-rich repeat (LRR) family protein | Chr2:11226615-11228057 REVERSE LENGTH=480 | 201606 Arabidopsis_thaliana OG0000001
AT2G27060.1 | Leucine-rich repeat protein kinase family protein | Chr2:11551288-11554577 FORWARD LENGTH=1020 | 201606 Arabidopsis_thaliana OG0000001
AT2G30105.1 | LRR/ubiquitin-like domain protein | Chr2:12849834-12851908 FORWARD LENGTH=374 | 201606 Arabidopsis_thaliana OG0000001
AT1G09970.1 | Leucine-rich receptor-like protein kinase family protein | Chr1:3252408-3255428 FORWARD LENGTH=976 | 201606 Arabidopsis_thaliana OG0000001
AT2G32660.1 | receptor like protein 22 | Chr2:13853897-13855666 REVERSE LENGTH=589 | 201606 Arabidopsis_thaliana OG0000001
AT2G32680.1 | receptor like protein 23 | Chr2:13859942-13862614 REVERSE LENGTH=890 | 201606 Arabidopsis_thaliana OG0000001

AT2G33020.1 | receptor like protein 24 | Chr2:14013874-14016516 REVERSE LENGTH=864 | 201606 Arabidopsis_thaliana OG0000001
AT2G33030.1 | receptor like protein 25 | Chr2:14017684-14018340 REVERSE LENGTH=218 | 201606 Arabidopsis_thaliana OG0000001
AT2G33050.1 | receptor like protein 26 | Chr2:14021870-14024272 FORWARD LENGTH=800 | 201606 Arabidopsis_thaliana OG0000001
AT2G33060.1 | receptor like protein 27 | Chr2:14025661-14028087 FORWARD LENGTH=808 | 201606 Arabidopsis_thaliana OG0000001
AT2G33080.1 | receptor like protein 28 | Chr2:14032015-14034237 FORWARD LENGTH=740 | 201606 Arabidopsis_thaliana OG0000001
AT2G33170.1 | Leucine-rich repeat receptor-like protein kinase family protein | Chr2:14056371-14059829 REVERSE LENGTH=1124 | 201606 Arabidopsis_thaliana OG0000001
Os03t0320800-01 Similar to Leucine Rich Repeat family protein, expressed. Oryza_sativa OG0000001
Os01t0239700-01 Similar to Leucine-rich receptor-like protein kinase. Oryza_sativa OG0000001
Os03t0335500-01 Protein kinase, core domain containing protein. Oryza_sativa OG0000001
Os03t0379801-00 Similar to NB-ARC domain containing protein. Oryza_sativa OG0000001
Os03t0400800-00 Similar to Leucine Rich Repeat family protein, expressed. Oryza_sativa OG0000001
Os03t0436600-00 Similar to polygalacturonase inhibitor 1. Oryza_sativa OG0000001
Os03t0440900-01 Similar to LRR protein. Oryza_sativa OG0000001
Os03t0637600-02 Leucine-rich repeat, plant specific containing protein. Oryza_sativa OG0000001
Os03t0724300-01 Tyrosine protein kinase domain containing protein. Oryza_sativa OG0000001
Os03t0756200-01 Protein kinase, core domain containing protein. Oryza_sativa OG0000001
Os03t0773300-01 Protein kinase, core domain containing protein. Oryza_sativa OG0000001
Os03t0773700-01 Similar to Receptor-like protein kinase 2. Oryza_sativa OG0000001
Os03t0795300-01 Similar to Extensin protein-like. Oryza_sativa OG0000001
Os01t0279800-01 Similar to LRR protein. Oryza_sativa OG0000001
Os04t0119800-01 Leucine-rich repeat domain containing protein. Oryza_sativa OG0000001
Os04t0122000-01 Leucine-rich repeat, N-terminal domain containing protein. Oryza_sativa OG0000001
Os04t0122200-00 Similar to OSIGBa0137A06.2 protein. Oryza_sativa OG0000001
Os04t0132500-01 Protein kinase, core domain containing protein. Oryza_sativa OG0000001

Os04t0226340-00 Conserved hypothetical protein.	Oryza_sativa	OG0000001
Os04t0226800-00 Similar to OSIGBa0135L04.4 protein.	Oryza_sativa	OG0000001
Os04t0227000-01 Leucine-rich repeat, N-terminal domain containing protein.	Oryza_sativa	OG0000001
Os04t0227200-00 Protein kinase, catalytic domain domain containing protein.	Oryza_sativa	OG0000001
Os04t0291900-00 Protein kinase, catalytic domain domain containing protein.	Oryza_sativa	OG0000001
Os04t0349700-01 Leucine-rich repeat, typical subtype containing protein.	Oryza_sativa	OG0000001
Os04t0472500-00 Serine/threonine protein kinase-related domain containing protein.	Oryza_sativa	OG0000001
Os04t0476700-01 Leucine-rich repeat, typical subtype containing protein.	Oryza_sativa	OG0000001
Os04t0480500-01 Leucine-rich repeat, N-terminal domain containing protein.	Oryza_sativa	OG0000001
Os04t0505200-01 Similar to Protein binding protein.	Oryza_sativa	OG0000001
Os04t0534166-00 Similar to OSIGBa0159I10.8 protein.	Oryza_sativa	OG0000001
Os04t0576900-01 Protein kinase, core domain containing protein.	Oryza_sativa	OG0000001
Os04t0605300-01 Leucine-rich repeat, typical subtype containing protein.	Oryza_sativa	OG0000001
Os04t0616400-01 Similar to H0525G02.12 protein.	Oryza_sativa	OG0000001
Os04t0618700-01 Protein kinase, core domain containing protein.	Oryza_sativa	OG0000001
Os04t0647900-01 Leucine-rich repeat, N-terminal domain containing protein.	Oryza_sativa	OG0000001
Os04t0648200-01 Leucine-rich repeat, plant specific containing protein.	Oryza_sativa	OG0000001
Os04t0648400-00 Similar to H0811D08.17 protein.	Oryza_sativa	OG0000001
Os04t0672100-01 Similar to Phytosulfokine receptor precursor (EC 2.7.1.37) (Phytosulfokine LRR receptor kinase).	Oryza_sativa	OG0000001
Os04t0672600-01 Leucine-rich repeat, N-terminal domain containing protein.	Oryza_sativa	OG0000001
Os04t0677200-01 Similar to H0402C08.6 protein.	Oryza_sativa	OG0000001
Os05t0104200-01 Polygalacturonase inhibiting protein, Inhibitor of fungal polygalacturonase, Sheath blight resistance	Oryza_sativa	OG0000001
Os05t0104600-01 Leucine-rich repeat (LRR) protein, Inhibitor of fungal polygalacturonase, Defence response	Oryza_sativa	OG0000001
Os05t0104700-01 Leucine-rich repeat (LRR) protein, Inhibitor of fungal polygalacturonase, Defence response	Oryza_sativa	OG0000001
Os01t0356900-01 Similar to Extensin-like protein.	Oryza_sativa	OG0000001

Os05t0154300-00 Hypothetical conserved gene.	Oryza_sativa	OG0000001
Os05t0169600-01 Serine/threonine protein kinase domain containing protein.	Oryza_sativa	OG0000001
Os05t0170300-00 Leucine-rich repeat domain containing protein.	Oryza_sativa	OG0000001
Os05t0170700-01 Leucine-rich repeat domain containing protein.	Oryza_sativa	OG0000001
Os05t0180300-01 Leucine-rich repeat, plant specific containing protein.	Oryza_sativa	OG0000001
Os05t0188700-01 Leucine-rich repeat domain containing protein.	Oryza_sativa	OG0000001
Os05t0256100-01 Similar to SHR5-receptor-like kinase (Fragment).	Oryza_sativa	OG0000001
Os05t0257100-01 Leucine-rich repeat, plant specific containing protein.	Oryza_sativa	OG0000001
Os05t0399800-01 Leucine-rich repeat, N-terminal domain containing protein.	Oryza_sativa	OG0000001
Os05t0406800-01 Leucine-rich repeat, N-terminal domain containing protein.	Oryza_sativa	OG0000001
Os05t0478300-01 Hypothetical conserved gene.	Oryza_sativa	OG0000001
Os05t0491400-01 Similar to LRR protein.	Oryza_sativa	OG0000001
Os05t0522600-01 Leucine-rich repeat, plant specific containing protein.	Oryza_sativa	OG0000001
Os05t0524600-00 Leucine-rich repeat domain containing protein.	Oryza_sativa	OG0000001
Os05t0525000-00 Protein kinase, catalytic domain domain containing protein.	Oryza_sativa	OG0000001
Os05t0525501-00 Serine-threonine/tyrosine-protein kinase domain containing protein.	Oryza_sativa	OG0000001
Os05t0530701-01 Leucine-rich repeat, plant specific containing protein.	Oryza_sativa	OG0000001
Os01t0515300-01 Similar to Leucine Rich Repeat family protein.	Oryza_sativa	OG0000001
Os01t0523100-00 Protein kinase, catalytic domain domain containing protein.	Oryza_sativa	OG0000001
Os05t0595800-00 Serine-threonine/tyrosine-protein kinase domain containing protein.	Oryza_sativa	OG0000001
Os06t0130100-01 Receptor-like kinase (RLK), Drought and salt stress tolerance	Oryza_sativa	OG0000001
Os06t0134700-01 Protein kinase, core domain containing protein.	Oryza_sativa	OG0000001
Os06t0140200-01 Leucine-rich repeat, plant specific containing protein.	Oryza_sativa	OG0000001
Os06t0140300-01 Leucine-rich repeat, N-terminal domain containing protein.	Oryza_sativa	OG0000001
Os06t0167500-01 Leucine-rich repeat, plant specific containing protein.	Oryza_sativa	OG0000001
Os06t0179800-01 Leucine-rich repeat, N-terminal domain containing protein.	Oryza_sativa	OG0000001
Os06t0185966-00 Similar to Glutathione peroxidase.	Oryza_sativa	OG0000001

Os06t0186100-00 Protein kinase, catalytic domain domain containing protein. Oryza_sativa OG0000001
Os06t0186300-00 Leucine-rich repeat domain containing protein. Oryza_sativa OG0000001
Os06t0203800-01 Receptor-like kinase, Heat tolerance Oryza_sativa OG0000001
Os06t0478600-01 Leucine-rich repeat, N-terminal domain containing protein. Oryza_sativa OG0000001
Os06t0557100-01 Serine/threonine protein kinase domain containing protein. Oryza_sativa OG0000001
Os06t0557400-00 Leucine-rich repeat domain containing protein. Oryza_sativa OG0000001
Os06t0557700-01 Protein kinase, catalytic domain domain containing protein. Oryza_sativa OG0000001
Os06t0581500-01 Protein kinase, core domain containing protein. Oryza_sativa OG0000001
Os06t0583600-00 Protein kinase, catalytic domain domain containing protein. Oryza_sativa OG0000001
Os06t0585950-00 Protein kinase, catalytic domain domain containing protein. Oryza_sativa OG0000001
Os06t0585982-00 Protein kinase, catalytic domain domain containing protein. Oryza_sativa OG0000001
Os06t0586150-00 Protein kinase, catalytic domain domain containing protein. Oryza_sativa OG0000001
Os06t0587000-00 Leucine-rich repeat domain containing protein. Oryza_sativa OG0000001
Os06t0587200-00 Protein kinase, catalytic domain domain containing protein. Oryza_sativa OG0000001
Os06t0587900-00 Hypothetical conserved gene. Oryza_sativa OG0000001
Os06t0588800-00 Protein kinase, catalytic domain domain containing protein. Oryza_sativa OG0000001
Os06t0589800-01 Protein kinase, core domain containing protein. Oryza_sativa OG0000001
Os06t0627500-01 Leucine-rich repeat, plant specific containing protein. Oryza_sativa OG0000001
Os06t0634500-01 Similar to kinase. Oryza_sativa OG0000001
Os06t0638500-00 Tyrosine protein kinase domain containing protein. Oryza_sativa OG0000001
Os01t0577600-01 Similar to predicted protein. Oryza_sativa OG0000001
Os06t0667000-01 Serine/threonine protein kinase domain containing protein. Oryza_sativa OG0000001
Os06t0691800-01 Protein kinase, core domain containing protein. Oryza_sativa OG0000001
Os06t0692100-01 Protein kinase, catalytic domain domain containing protein. Oryza_sativa OG0000001
Os06t0692300-00 Hypothetical conserved gene. Oryza_sativa OG0000001
Os06t0692500-00 Hypothetical conserved gene. Oryza_sativa OG0000001
Os06t0692600-01 Protein kinase, core domain containing protein. Oryza_sativa OG0000001

Os06t0704500-01 Leucine-rich repeat, plant specific containing protein. Oryza_sativa OG0000001
Os06t0717200-01 Serine/threonine protein kinase domain containing protein. Oryza_sativa OG0000001
Os07t0107800-01 Similar to Phytosulfokine receptor precursor (EC 2.7.1.37) (Phytosulfokine LRR receptor kinase). Oryza_sativa OG0000001
Os07t0121200-00 Similar to LRR receptor-like kinase. Oryza_sativa OG0000001
Os07t0134200-01 Receptor-like protein kinase 3. Oryza_sativa OG0000001
Os07t0134600-01 Serine/threonine protein kinase domain containing protein. Oryza_sativa OG0000001
Os07t0145400-01 Protein kinase, core domain containing protein. Oryza_sativa OG0000001
Os07t0152200-01 Similar to predicted protein. Oryza_sativa OG0000001
Os01t0594300-01 Similar to leucine-rich repeat family protein / extensin family protein. Oryza_sativa OG0000001
Os07t0176500-01 Leucine-rich repeat, plant specific containing protein. Oryza_sativa OG0000001
Os07t0207100-01 Protein kinase, core domain containing protein. Oryza_sativa OG0000001
Os01t0601625-00 Leucine rich repeat, N-terminal domain containing protein. Oryza_sativa OG0000001
Os01t0601675-01 Leucine-rich repeat, N-terminal domain containing protein. Oryza_sativa OG0000001
Os01t0601700-01 Leucine-rich repeat domain containing protein. Oryza_sativa OG0000001
Os07t0251900-01 Leucine-rich repeat, N-terminal domain containing protein. Oryza_sativa OG0000001
Os01t0603500-00 Similar to Cf2/Cf5-like disease resistance protein (Fragment). Oryza_sativa OG0000001
Os01t0603800-01 Similar to Triticum sp. (pAWJL3) leucine rich repeat region mRNA (Fragment). Oryza_sativa OG0000001
Os01t0604150-00 Leucine-rich repeat domain containing protein. Oryza_sativa OG0000001
Os07t0466500-00 Leucine-rich repeat domain containing protein. Oryza_sativa OG0000001
Os07t0468500-01 Leucine-rich repeat domain containing protein. Oryza_sativa OG0000001
Os07t0498400-00 Leucine-rich repeat, typical subtype domain containing protein. Oryza_sativa OG0000001
Os07t0568700-02 Polygalacturonase-inhibiting protein, Inhibitor of fungal polygalacturonase, Regulation of floral organ number Oryza_sativa OG0000001
Os07t0597200-01 Protein kinase, core domain containing protein. Oryza_sativa OG0000001
Os07t0602700-01 Protein kinase, core domain containing protein. Oryza_sativa OG0000001
Os07t0626500-01 Protein kinase, core domain containing protein. Oryza_sativa OG0000001
Os01t0623000-00 Hypothetical conserved gene. Oryza_sativa OG0000001

Os08t0117700-01 Serine/threonine protein kinase domain containing protein. Oryza_sativa OG0000001
Os08t0148300-01 Conserved hypothetical protein. Oryza_sativa OG0000001
Os08t0203100-01 Hypothetical conserved gene. Oryza_sativa OG0000001
Os08t0203300-01 Similar to SHR5-receptor-like kinase (Fragment). Oryza_sativa OG0000001
Os08t0203600-01 Hypothetical conserved gene. Oryza_sativa OG0000001
Os08t0247600-01 Leucine-rich repeat, N-terminal domain containing protein. Oryza_sativa OG0000001
Os08t0247700-00 Protein kinase, catalytic domain domain containing protein. Oryza_sativa OG0000001
Os08t0248100-01 Protein kinase, core domain containing protein. Oryza_sativa OG0000001
Os08t0266400-00 Leucine-rich repeat, N-terminal domain containing protein. Oryza_sativa OG0000001
Os08t0276400-01 Serine/threonine protein kinase domain containing protein. Oryza_sativa OG0000001
Os08t0322500-00 Hypothetical conserved gene. Oryza_sativa OG0000001
Os08t0331900-01 Similar to Leucine Rich Repeat family protein, expressed. Oryza_sativa OG0000001
Os08t0342300-01 BR receptor kinase, Brassinosteroid (BR) perception in the root Oryza_sativa OG0000001
Os01t0132100-00 Leucine-rich repeat domain containing protein. Oryza_sativa OG0000001
Os08t0376300-01 Similar to Leucine-rich receptor-like protein kinase. Oryza_sativa OG0000001
Os08t0427600-00 Similar to receptor kinase. Oryza_sativa OG0000001
Os08t0446200-01 Similar to Receptor-like protein kinase precursor (EC 2.7.1.37). Splice isoform INRPK1a. Oryza_sativa OG0000001
Os08t0446400-01 Leucine-rich repeat, N-terminal domain containing protein. Oryza_sativa OG0000001
Os08t0493800-01 Protein kinase, core domain containing protein. Oryza_sativa OG0000001
Os08t0505900-01 Leucine-rich repeat (LRR) protein, Inhibitor of fungal polygalacturonase, Defence response Oryza_sativa OG0000001
Os08t0510101-00 Similar to polygalacturonase inhibitor 1. Oryza_sativa OG0000001
Os08t0511700-00 Similar to PSR9 (Fragment). Oryza_sativa OG0000001
Os08t0541300-01 Leucine-rich repeat, N-terminal domain containing protein. Oryza_sativa OG0000001
Os09t0110100-01 Protein kinase, core domain containing protein. Oryza_sativa OG0000001
Os09t0131200-01 Similar to Elicitor-inducible LRR receptor-like protein EILP. Oryza_sativa OG0000001
Os09t0293500-01 BR receptor kinase, Brassinosteroid (BR) perception in the root Oryza_sativa OG0000001

Os09t0326100-01 Protein kinase, core domain containing protein.
Oryza_sativa OG0000001
Os09t0345300-01 Similar to Receptor-like serine/threonine kinase.
Oryza_sativa OG0000001
Os09t0376600-00 Tyrosine protein kinase domain containing protein.
Oryza_sativa OG0000001
Os09t0423200-00 Protein kinase, catalytic domain domain containing protein. Oryza_sativa OG0000001
Os09t0479200-01 Similar to Receptor protein kinase-like protein.
Oryza_sativa OG0000001
Os09t0479600-01 Similar to RXO1 disease resistance protein.
Oryza_sativa OG0000001
Os09t0491612-01 Leucine-rich repeat (LRR) protein, Inhibitor of fungal polygalacturonase, Defence response Oryza_sativa
OG0000001
Os01t0694000-01 Protein kinase, core domain containing protein.
Oryza_sativa OG0000001
Os01t0694100-01 Leucine-rich repeat domain containing protein.
Oryza_sativa OG0000001
Os10t0114400-01 Protein kinase, catalytic domain domain containing protein. Oryza_sativa OG0000001
Os10t0120300-01 Similar to Leucine Rich Repeat family protein, expressed. Oryza_sativa OG0000001
Os10t0155700-00 Protein kinase, catalytic domain domain containing protein. Oryza_sativa OG0000001
Os10t0155800-01 Protein kinase, core domain containing protein.
Oryza_sativa OG0000001
Os10t0336300-00 Leucine-rich repeat domain containing protein.
Oryza_sativa OG0000001
Os10t0337400-01 Similar to Taxa-1. Oryza_sativa
OG0000001
Os10t0360800-00 Protein kinase, catalytic domain domain containing protein. Oryza_sativa OG0000001
Os10t0374600-00 Hypothetical conserved gene. Oryza_sativa
OG0000001
Os10t0375000-01 Hypothetical conserved gene. Oryza_sativa
OG0000001
Os10t0375400-00 Leucine-rich repeat domain containing protein.
Oryza_sativa OG0000001
Os01t0711200-01 Tyrosine protein kinase domain containing protein.
Oryza_sativa OG0000001
Os10t0467900-01 Protein kinase, catalytic domain domain containing protein. Oryza_sativa OG0000001
Os10t0468500-01 Serine/threonine protein kinase-related domain containing protein. Oryza_sativa OG0000001
Os10t0468800-00 Hypothetical conserved gene. Oryza_sativa
OG0000001
Os10t0469000-01 Hypothetical conserved gene. Oryza_sativa
OG0000001
Os10t0469300-00 Leucine-rich repeat, N-terminal domain containing protein. Oryza_sativa OG0000001
Os10t0469600-01 Similar to Leucine Rich Repeat family protein, expressed. Oryza_sativa OG0000001
Os10t0469700-01 Leucine-rich repeat, typical subtype containing

protein. Oryza_sativa OG0000001
Os01t0718300-01 Receptor serine/threonine kinase, Organ development through controlling cell division and elongation Oryza_sativa OG0000001
Os10t0492400-01 Similar to Protein kinase domain containing protein, expressed. Oryza_sativa OG0000001
Os10t0527900-01 Leucine-rich repeat, N-terminal domain containing protein. Oryza_sativa OG0000001
Os10t0531700-00 Serine/threonine protein kinase-related domain containing protein. Oryza_sativa OG0000001
Os10t0572300-01 Leucine-rich repeat, typical subtype containing protein. Oryza_sativa OG0000001
Os11t0107700-01 Similar to Leucine Rich Repeat family protein, expressed. Oryza_sativa OG0000001
Os01t0729400-01 Similar to Too many mouths protein. Oryza_sativa OG0000001
Os11t0171800-01 Protein kinase, core domain containing protein. Oryza_sativa OG0000001
Os11t0172150-00 Similar to Leucine Rich Repeat family protein, expressed. Oryza_sativa OG0000001
Os11t0172300-01 Leucine-rich repeat domain containing protein. Oryza_sativa OG0000001
Os11t0173432-00 Protein kinase, catalytic domain domain containing protein. Oryza_sativa OG0000001
Os11t0173500-01 Leucine-rich repeat, typical subtype domain containing protein. Oryza_sativa OG0000001
Os11t0173550-01 Hypothetical conserved gene. Oryza_sativa OG0000001
Os11t0173800-01 Serine/threonine protein kinase-related domain containing protein. Oryza_sativa OG0000001
Os11t0173900-00 Similar to Leucine Rich Repeat family protein, expressed. Oryza_sativa OG0000001
Os11t0208900-01 Leucine rich repeat containing protein kinase. Oryza_sativa OG0000001
Os11t0228800-00 Similar to NBS-LRR-like protein. Oryza_sativa OG0000001
Os11t0232100-01 Protein kinase, core domain containing protein. Oryza_sativa OG0000001
Os11t0233000-00 Similar to Leucine Rich Repeat family protein. Oryza_sativa OG0000001
Os11t0233800-01 Serine/threonine protein kinase-related domain containing protein. Oryza_sativa OG0000001
Os11t0300600-01 Leucine-rich repeat, N-terminal domain containing protein. Oryza_sativa OG0000001
Os11t0308800-01 Hypothetical conserved gene. Oryza_sativa OG0000001
Os01t0742400-01 Hypothetical conserved gene. Oryza_sativa OG0000001
Os11t0490200-00 Protein kinase, catalytic domain domain containing protein. Oryza_sativa OG0000001
Os11t0514400-00 Similar to BRASSINOSTEROID INSENSITIVE 1-associated receptor kinase 1. Oryza_sativa OG0000001
Os11t0514500-01 Sorghum bicolor leucine-rich repeat-containing extracellular glycoprotein precursor. Oryza_sativa

OG0000001
Os11t0514700-00 Similar to BRASSINOSTEROID INSENSITIVE 1-associated receptor kinase 1. Oryza_sativa OG0000001
Os11t0558400-00 Leucine-rich repeat, N-terminal domain containing protein. Oryza_sativa OG0000001
Os11t0558900-01 Leucine-rich repeat, N-terminal domain containing protein. Oryza_sativa OG0000001
Os11t0559200-00 Protein kinase, core domain containing protein. Oryza_sativa OG0000001
Os11t0560900-00 Similar to Leucine Rich Repeat family protein. Oryza_sativa OG0000001
Os11t0561100-00 Similar to Leucine Rich Repeat family protein. Oryza_sativa OG0000001
Os11t0564900-00 Leucine-rich repeat domain containing protein. Oryza_sativa OG0000001
Os11t0565000-01 Leucine-rich repeat, N-terminal domain containing protein. Oryza_sativa OG0000001
Os11t0565920-00 Leucine-rich repeat domain containing protein. Oryza_sativa OG0000001
Os11t0567600-01 Leucine-rich repeat domain containing protein. Oryza_sativa OG0000001
Os11t0567800-01 Leucine-rich repeat-containing N-terminal, type 2 domain containing protein. Oryza_sativa OG0000001
Os11t0568800-00 Leucine-rich repeat, typical subtype domain containing protein. Oryza_sativa OG0000001
Os11t0569300-00 Serine/threonine protein kinase domain containing protein. Oryza_sativa OG0000001
Os11t0569500-01 Similar to Receptor kinase-like protein. Oryza_sativa OG0000001
Os11t0569600-01 Similar to Receptor kinase-like protein. Oryza_sativa OG0000001
Os11t0569733-00 Similar to Leucine Rich Repeat family protein. Oryza_sativa OG0000001
Os11t0569800-01 Protein kinase, catalytic domain domain containing protein. Oryza_sativa OG0000001
Os11t0570000-01 Similar to Receptor kinase-like protein. Oryza_sativa OG0000001
Os01t0750400-00 Leucine-rich repeat domain containing protein. Oryza_sativa OG0000001
Os11t0606200-01 Similar to Leucine Rich Repeat family protein, expressed. Oryza_sativa OG0000001
Os11t0606400-02 Similar to NB-ARC domain containing protein, expressed. Oryza_sativa OG0000001
Os11t0620500-01 Tyrosine protein kinase domain containing protein. Oryza_sativa OG0000001
Os11t0625900-01 Protein kinase, core domain containing protein. Oryza_sativa OG0000001
Os11t0628000-00 Protein kinase, catalytic domain domain containing protein. Oryza_sativa OG0000001
Os11t0640300-01 NB-ARC domain containing protein. Oryza_sativa OG0000001
Os11t0644100-00 Leucine-rich repeat, N-terminal domain containing protein. Oryza_sativa OG0000001
Os11t0657400-00 Similar to Extensin-like protein. Oryza_sativa

OG0000001
Os11t0688832-00 Similar to Leucine Rich Repeat family protein. Oryza_sativa OG0000001
Os11t0691800-01 Similar to Leucine Rich Repeat family protein, expressed. Oryza_sativa OG0000001
Os11t0692500-00 Protein kinase, catalytic domain domain containing protein. Oryza_sativa OG0000001
Os11t0695000-01 Similar to Leucine Rich Repeat family protein. Oryza_sativa OG0000001
Os11t0695800-01 Similar to Receptor kinase MRKc. Oryza_sativa OG0000001
Os12t0107700-00 Similar to Leucine Rich Repeat family protein, expressed. Oryza_sativa OG0000001
Os12t0182300-01 Protein kinase, core domain containing protein. Oryza_sativa OG0000001
Os12t0211500-01 Leucine-rich repeat, N-terminal domain containing protein. Oryza_sativa OG0000001
Os12t0215950-01 Similar to Leucine Rich Repeat family protein, expressed. Oryza_sativa OG0000001
Os12t0217400-00 Similar to Leucine Rich Repeat family protein, expressed. Oryza_sativa OG0000001
Os12t0218500-01 Hypothetical conserved gene. Oryza_sativa OG0000001
Os12t0221700-00 Hypothetical conserved gene. Oryza_sativa OG0000001
Os12t0222800-00 Leucine-rich repeat, N-terminal domain containing protein. Oryza_sativa OG0000001
Os12t0222900-01 Leucine-rich repeat, N-terminal domain containing protein. Oryza_sativa OG0000001
Os12t0235700-00 Serine/threonine protein kinase-related domain containing protein. Oryza_sativa OG0000001
Os12t0273900-00 Leucine-rich repeat domain containing protein. Oryza_sativa OG0000001
Os12t0498650-00 Protein kinase, catalytic domain domain containing protein. Oryza_sativa OG0000001
Os12t0542200-00 Conserved hypothetical protein. Oryza_sativa OG0000001
Os12t0620000-00 Similar to Leucine Rich Repeat family protein. Oryza_sativa OG0000001
Os12t0632800-00 Protein kinase, catalytic domain domain containing protein. Oryza_sativa OG0000001
Os12t0632900-01 Serine/threonine protein kinase-related domain containing protein. Oryza_sativa OG0000001
Os01t0809300-01 Leucine-rich repeat, N-terminal domain containing protein. Oryza_sativa OG0000001
Os01t0818700-01 Leucine-rich repeat, N-terminal domain containing protein. Oryza_sativa OG0000001
Os01t0821900-01 Protein kinase, core domain containing protein. Oryza_sativa OG0000001
Os01t0149700-00 Hypothetical conserved gene. Oryza_sativa OG0000001
Os01t0152000-01 Protein kinase, core domain containing protein. Oryza_sativa OG0000001
Os01t0878300-01 Protein kinase, core domain containing protein.

Oryza_sativa   OG0000001
Os01t0152600-00 Protein kinase, catalytic domain domain containing protein. Oryza_sativa   OG0000001
Os01t0152800-01 Protein kinase, core domain containing protein. Oryza_sativa   OG0000001
Os01t0891601-01 Leucine-rich repeat, N-terminal domain containing protein. Oryza_sativa   OG0000001
Os01t0891700-01 Leucine-rich repeat, N-terminal domain containing protein. Oryza_sativa   OG0000001
Os01t0892300-01 Leucine-rich repeat, plant specific containing protein. Oryza_sativa   OG0000001
Os01t0153000-00 Protein kinase, catalytic domain domain containing protein. Oryza_sativa   OG0000001
Os01t0917500-01 Hypothetical conserved gene.   Oryza_sativa   OG0000001
Os01t0957100-01 Protein kinase, core domain containing protein. Oryza_sativa   OG0000001
Os01t0158600-01 Leucine-rich repeat, N-terminal domain containing protein. Oryza_sativa   OG0000001
Os01t0160200-02 Leucine-rich repeat, N-terminal domain containing protein. Oryza_sativa   OG0000001
Os01t0160700-00 Leucine-rich repeat, N-terminal domain containing protein. Oryza_sativa   OG0000001
Os01t0161000-00 Hypothetical conserved gene.   Oryza_sativa   OG0000001
Os01t0161300-00 Hypothetical conserved gene.   Oryza_sativa   OG0000001
Os01t0162200-00 Leucine-rich repeat domain containing protein. Oryza_sativa   OG0000001
Os01t0162300-01 Leucine-rich repeat, plant specific containing protein. Oryza_sativa   OG0000001
Os01t0162500-01 Leucine-rich repeat-containing N-terminal, type 2 domain containing protein.   Oryza_sativa   OG0000001
Os02t0111800-02 Protein kinase, core domain containing protein. Oryza_sativa   OG0000001
Os01t0162800-00 Hypothetical conserved gene.   Oryza_sativa   OG0000001
Os01t0163000-01 Leucine-rich repeat, N-terminal domain containing protein. Oryza_sativa   OG0000001
Os02t0116700-01 Protein kinase, core domain containing protein. Oryza_sativa   OG0000001
Os02t0136900-01 Protein kinase, core domain containing protein. Oryza_sativa   OG0000001
Os02t0138000-00 Leucine-rich repeat, plant specific containing protein. Oryza_sativa   OG0000001
Os02t0153100-00 Protein kinase, core domain containing protein. Oryza_sativa   OG0000001
Os02t0153200-01 Protein kinase, core domain containing protein. Oryza_sativa   OG0000001
Os02t0153400-00 Protein kinase, core domain containing protein. Oryza_sativa   OG0000001
Os02t0153500-00 Protein kinase, core domain containing protein. Oryza_sativa   OG0000001
Os02t0153700-01 Serine/threonine protein kinase domain containing

protein. Oryza_sativa   OG0000001
Os02t0153900-01 Protein kinase, core domain containing protein.
Oryza_sativa   OG0000001
Os02t0154000-00 Protein kinase, core domain containing protein.
Oryza_sativa   OG0000001
Os02t0154200-01 Protein kinase, core domain containing protein.
Oryza_sativa   OG0000001
Os02t0155400-01 Leucine-rich repeat, N-terminal domain containing
protein. Oryza_sativa   OG0000001
Os02t0155600-00 Hypothetical conserved gene.   Oryza_sativa
OG0000001
Os02t0155700-00 Hypothetical conserved gene.   Oryza_sativa
OG0000001
Os02t0155900-00 Leucine-rich repeat domain containing protein.
Oryza_sativa   OG0000001
Os02t0156200-00 Leucine-rich repeat domain containing protein.
Oryza_sativa   OG0000001
Os02t0156400-01 Leucine-rich repeat, N-terminal domain containing
protein. Oryza_sativa   OG0000001
Os02t0156600-00 Hypothetical conserved gene.   Oryza_sativa
OG0000001
Os02t0156633-00 Hypothetical conserved gene.   Oryza_sativa
OG0000001
Os02t0156800-01 Leucine-rich repeat-containing N-terminal, type 2
domain containing protein.   Oryza_sativa   OG0000001
Os02t0157000-00 Leucine-rich repeat domain containing protein.
Oryza_sativa   OG0000001
Os02t0157100-00 Leucine-rich repeat domain containing protein.
Oryza_sativa   OG0000001
Os02t0157200-00 Leucine-rich repeat domain containing protein.
Oryza_sativa   OG0000001
Os02t0157400-01 Leucine-rich repeat-containing N-terminal, type 2
domain containing protein.   Oryza_sativa   OG0000001
Os02t0157501-00 Leucine-rich repeat domain containing protein.
Oryza_sativa   OG0000001
Os02t0161500-00 Leucine-rich repeat domain containing protein.
Oryza_sativa   OG0000001
Os02t0176100-01 Similar to predicted protein.   Oryza_sativa
OG0000001
Os01t0170300-01 Serine/threonine protein kinase-related domain
containing protein.   Oryza_sativa   OG0000001
Os02t0210700-01 Protein kinase, core domain containing protein.
Oryza_sativa   OG0000001
Os02t0211800-00 Hypothetical conserved gene.   Oryza_sativa
OG0000001
Os02t0211900-00 Hypothetical conserved gene.   Oryza_sativa
OG0000001
Os02t0215500-01 Protein kinase, catalytic domain domain containing
protein. Oryza_sativa   OG0000001
Os02t0215700-01 Protein kinase, catalytic domain domain containing
protein. Oryza_sativa   OG0000001
Os02t0215900-01 Protein kinase, catalytic domain domain containing
protein. Oryza_sativa   OG0000001
Os02t0216000-00 Hypothetical conserved gene.   Oryza_sativa

OG0000001
Os02t0227600-01 Leucine-rich repeat domain containing protein.
Oryza_sativa OG0000001
Os02t0227700-01 Serine/threonine protein kinase-related domain
containing protein. Oryza_sativa OG0000001
Os02t0227900-00 Leucine-rich repeat domain containing protein.
Oryza_sativa OG0000001
Os02t0228300-01 Protein kinase, core domain containing protein.
Oryza_sativa OG0000001
Os02t0231700-00 Protein kinase, catalytic domain domain containing
protein. Oryza_sativa OG0000001
Os02t0232500-01 Similar to Receptor-like serine/threonine kinase.
Oryza_sativa OG0000001
Os02t0277700-01 Leucine-rich repeat domain containing protein.
Oryza_sativa OG0000001
Os01t0180000-01 Pistil-specific extensin-like protein family
protein. Oryza_sativa OG0000001
Os02t0515125-00 Hypothetical conserved gene. Oryza_sativa
OG0000001
Os02t0593600-01 Leucine-rich repeat, typical subtype containing
protein. Oryza_sativa OG0000001
Os02t0603100-01 Similar to fasciated ear2. Oryza_sativa
OG0000001
Os02t0609900-00 Leucine-rich repeat, N-terminal domain containing
protein. Oryza_sativa OG0000001
Os02t0610000-00 Leucine-rich repeat domain containing protein.
Oryza_sativa OG0000001
Os02t0614966-00 Leucine-rich repeat domain containing protein.
Oryza_sativa OG0000001
Os02t0615400-00 Leucine-rich repeat domain containing protein.
Oryza_sativa OG0000001
Os02t0615500-01 Protein kinase, core domain containing protein.
Oryza_sativa OG0000001
Os02t0615800-01 Protein kinase, core domain containing protein.
Oryza_sativa OG0000001
Os02t0616100-01 Similar to protein binding protein.
Oryza_sativa OG0000001
Os02t0629400-01 Similar to Phytosulfokine receptor precursor (EC
2.7.1.37) (Phytosulfokine LRR receptor kinase). Oryza_sativa
OG0000001
Os02t0635600-01 Tyrosine protein kinase domain containing protein.
Oryza_sativa OG0000001
Os02t0647300-01 Leucine-rich repeat, plant specific containing
protein. Oryza_sativa OG0000001
Os02t0728500-01 Similar to BRASSINOSTEROID INSENSITIVE 1-associated
receptor kinase 1. Oryza_sativa OG0000001
Os01t0110800-01 Leucine-rich repeat, plant specific containing
protein. Oryza_sativa OG0000001
Os02t0826600-01 Leucine-rich repeat, typical subtype containing
protein. Oryza_sativa OG0000001
Os02t0830700-01 Leucine-rich repeat, N-terminal domain containing
protein. Oryza_sativa OG0000001
Os03t0127700-01 Serine/threonine protein kinase-related domain
containing protein. Oryza_sativa OG0000001

Os03t0145102-00 Similar to Leucine Rich Repeat family protein, expressed. Oryza_sativa OG0000001
Os03t0211900-01 Leucine-rich repeat, N-terminal domain containing protein. Oryza_sativa OG0000001
Os03t0212200-01 Leucine-rich repeat domain containing protein. Oryza_sativa OG0000001
Os03t0228800-01 Similar to LRK1 protein. Oryza_sativa OG0000001
CMS391CT pep chromosome:ASM9120v1:19:985659:986538:-1 gene:CMS391C transcript:CMS391CT gene_biotype:protein_coding transcript_biotype:protein_coding Cyanidioschyzon_merolae OG0000001
CMD073CT pep chromosome:ASM9120v1:4:181305:181665:-1 gene:CMD073C transcript:CMD073CT gene_biotype:protein_coding transcript_biotype:protein_coding Cyanidioschyzon_merolae OG0000001
CMK078CT pep chromosome:ASM9120v1:11:221643:225629:1 gene:CMK078C transcript:CMK078CT gene_biotype:protein_coding transcript_biotype:protein_coding Cyanidioschyzon_merolae OG0000001
evm.model.contig_2154.8 Porphyridium_purpureum OG0000001
evm.model.contig_3473.2 Porphyridium_purpureum OG0000001
evm.model.contig_3523.4 Porphyridium_purpureum OG0000001
evm.model.contig_4426.9 Porphyridium_purpureum OG0000001
evm.model.contig_4510.3 Porphyridium_purpureum OG0000001
evm.model.contig_639.2 Porphyridium_purpureum OG0000001
CDF32210 pep supercontig:ASM35022v2:HG001460:51741:52256:-1 gene:CHC_T00010170001 transcript:CDF32210 gene_biotype:protein_coding transcript_biotype:protein_coding description:Hypothetical leucine rich repeat-containing protein Chondrus_crispus OG0000001
CDF38814 pep supercontig:ASM35022v2:HG001969:31933:33143:1 gene:CHC_T00008541001 transcript:CDF38814 gene_biotype:protein_coding transcript_biotype:protein_coding description:Leucine rich repeat-containing protein Chondrus_crispus OG0000001
CDF34108 pep supercontig:ASM35022v2:HG001673:192259:194350:1 gene:CHC_T00008395001 transcript:CDF34108 gene_biotype:protein_coding transcript_biotype:protein_coding description:Leucine rich repeat-containing protein Chondrus_crispus OG0000001
CDF32119 pep supercontig:ASM35022v2:HG001459:235237:236511:-1 gene:CHC_T00008817001 transcript:CDF32119 gene_biotype:protein_coding transcript_biotype:protein_coding description:Leucine rich repeat protein kinase Chondrus_crispus OG0000001
CDF38927 pep supercontig:ASM35022v2:HG001976:57837:58766:1 gene:CHC_T00008388001 transcript:CDF38927 gene_biotype:protein_coding transcript_biotype:protein_coding description:Leucine-rich repeat-containing protein Chondrus_crispus OG0000001
g1317.t1 Caulerpa_lentillifera OG0000002
g1413.t1 Caulerpa_lentillifera OG0000002
g1702.t1 Caulerpa_lentillifera OG0000002

g1923.t1	Caulerpa_lentillifera	OG0000002
g2274.t1	Caulerpa_lentillifera	OG0000002
g282.t1	Caulerpa_lentillifera	OG0000002
g283.t1	Caulerpa_lentillifera	OG0000002
g285.t1	Caulerpa_lentillifera	OG0000002
g293.t1	Caulerpa_lentillifera	OG0000002
g302.t1	Caulerpa_lentillifera	OG0000002
g3181.t1	Caulerpa_lentillifera	OG0000002
g3190.t1	Caulerpa_lentillifera	OG0000002
g3192.t1	Caulerpa_lentillifera	OG0000002
g3207.t1	Caulerpa_lentillifera	OG0000002
g3208.t1	Caulerpa_lentillifera	OG0000002
g3218.t1	Caulerpa_lentillifera	OG0000002
g3224.t1	Caulerpa_lentillifera	OG0000002
g3537.t1	Caulerpa_lentillifera	OG0000002
g357.t1	Caulerpa_lentillifera	OG0000002
g361.t1	Caulerpa_lentillifera	OG0000002
g3874.t1	Caulerpa_lentillifera	OG0000002
g3911.t1	Caulerpa_lentillifera	OG0000002
g3934.t1	Caulerpa_lentillifera	OG0000002
g4261.t1	Caulerpa_lentillifera	OG0000002
g4269.t1	Caulerpa_lentillifera	OG0000002
g4354.t1	Caulerpa_lentillifera	OG0000002
g4356.t1	Caulerpa_lentillifera	OG0000002
g4360.t1	Caulerpa_lentillifera	OG0000002
g4362.t1	Caulerpa_lentillifera	OG0000002
g4363.t1	Caulerpa_lentillifera	OG0000002
g4364.t1	Caulerpa_lentillifera	OG0000002
g4369.t1	Caulerpa_lentillifera	OG0000002
g4378.t1	Caulerpa_lentillifera	OG0000002
g4857.t1	Caulerpa_lentillifera	OG0000002
g4901.t1	Caulerpa_lentillifera	OG0000002
g4950.t1	Caulerpa_lentillifera	OG0000002
g4992.t1	Caulerpa_lentillifera	OG0000002
g4994.t1	Caulerpa_lentillifera	OG0000002
g4997.t1	Caulerpa_lentillifera	OG0000002
g4998.t1	Caulerpa_lentillifera	OG0000002
g4999.t1	Caulerpa_lentillifera	OG0000002
g5000.t1	Caulerpa_lentillifera	OG0000002
g5001.t1	Caulerpa_lentillifera	OG0000002
g5002.t1	Caulerpa_lentillifera	OG0000002
g5004.t1	Caulerpa_lentillifera	OG0000002
g5005.t1	Caulerpa_lentillifera	OG0000002
g5007.t1	Caulerpa_lentillifera	OG0000002
g5008.t1	Caulerpa_lentillifera	OG0000002
g5014.t1	Caulerpa_lentillifera	OG0000002
g5494.t1	Caulerpa_lentillifera	OG0000002
g5642.t1	Caulerpa_lentillifera	OG0000002
g5949.t1	Caulerpa_lentillifera	OG0000002
g6508.t1	Caulerpa_lentillifera	OG0000002
g6509.t1	Caulerpa_lentillifera	OG0000002
g6862.t1	Caulerpa_lentillifera	OG0000002
g7767.t1	Caulerpa_lentillifera	OG0000002
g7947.t1	Caulerpa_lentillifera	OG0000002

g8192.t1 Caulerpa_lentillifera OG0000002
g8513.t1 Caulerpa_lentillifera OG0000002
g8740.t1 Caulerpa_lentillifera OG0000002
g893.t1 Caulerpa_lentillifera OG0000002
g9199.t1 Caulerpa_lentillifera OG0000002
g9200.t1 Caulerpa_lentillifera OG0000002
g9201.t1 Caulerpa_lentillifera OG0000002
g9202.t1 Caulerpa_lentillifera OG0000002
Cre01.g050500.t1.1 pacid=30788972 transcript=Cre01.g050500.t1.1 locus=Cre01.g050500 ID=Cre01.g050500.t1.1.v5.5 annot-version=v5.5 Chlamydomonas_reinhardtii OG0000002
Cre10.g437150.t1.2 pacid=30790568 transcript=Cre10.g437150.t1.2 locus=Cre10.g437150 ID=Cre10.g437150.t1.2.v5.5 annot-version=v5.5 Chlamydomonas_reinhardtii OG0000002
Cre12.g511400.t1.1 pacid=30791658 transcript=Cre12.g511400.t1.1 locus=Cre12.g511400 ID=Cre12.g511400.t1.1.v5.5 annot-version=v5.5 Chlamydomonas_reinhardtii OG0000002
Cre08.g358250.t1.2 pacid=30773680 transcript=Cre08.g358250.t1.2 locus=Cre08.g358250 ID=Cre08.g358250.t1.2.v5.5 annot-version=v5.5 Chlamydomonas_reinhardtii OG0000002
Cre16.g649800.t1.1 pacid=30777657 transcript=Cre16.g649800.t1.1 locus=Cre16.g649800 ID=Cre16.g649800.t1.1.v5.5 annot-version=v5.5 Chlamydomonas_reinhardtii OG0000002
Cre06.g298300.t1.1 pacid=30778826 transcript=Cre06.g298300.t1.1 locus=Cre06.g298300 ID=Cre06.g298300.t1.1.v5.5 annot-version=v5.5 Chlamydomonas_reinhardtii OG0000002
Vocar.0001s0609.1.p pacid=32884369 transcript=Vocar.0001s0609.1 locus=Vocar.0001s0609 ID=Vocar.0001s0609.1.v2.1 annot-version=v2.1 Volvox_carteri OG0000002
Vocar.0016s0194.1.p pacid=32897980 transcript=Vocar.0016s0194.1 locus=Vocar.0016s0194 ID=Vocar.0016s0194.1.v2.1 annot-version=v2.1 Volvox_carteri OG0000002
Vocar.0001s0238.1.p pacid=32885678 transcript=Vocar.0001s0238.1 locus=Vocar.0001s0238 ID=Vocar.0001s0238.1.v2.1 annot-version=v2.1 Volvox_carteri OG0000002
Vocar.0001s1331.1.p pacid=32886103 transcript=Vocar.0001s1331.1 locus=Vocar.0001s1331 ID=Vocar.0001s1331.1.v2.1 annot-version=v2.1 Volvox_carteri OG0000002
Vocar.0020s0117.1.p pacid=32886713 transcript=Vocar.0020s0117.1 locus=Vocar.0020s0117 ID=Vocar.0020s0117.1.v2.1 annot-version=v2.1 Volvox_carteri OG0000002
Vocar.0032s0094.1.p pacid=32893829 transcript=Vocar.0032s0094.1 locus=Vocar.0032s0094 ID=Vocar.0032s0094.1.v2.1 annot-version=v2.1 Volvox_carteri OG0000002
jgi|ChlNC64A_1|140648|IGS.gm_3_00387 Chlorella_variabilis OG0000002
jgi|ChlNC64A_1|140730|IGS.gm_3_00469 Chlorella_variabilis OG0000002
jgi|ChlNC64A_1|134967|IGS.gm_12_00353 Chlorella_variabilis OG0000002
jgi|ChlNC64A_1|137007|IGS.gm_18_00113 Chlorella_variabilis OG0000002
jgi|ChlNC64A_1|25863|e_gw1.18.246.1 Chlorella_variabilis OG0000002

jgi|ChlNC64A_1|137845|IGS.gm_2_00312	Chlorella_variabilis
OG0000002
jgi|ChlNC64A_1|142073|IGS.gm_4_00214	Chlorella_variabilis
OG0000002
jgi|ChlNC64A_1|50745|fgenesh3_pg.C_scaffold_4000244
Chlorella_variabilis	OG0000002
jgi|ChlNC64A_1|142373|IGS.gm_4_00514	Chlorella_variabilis
OG0000002
jgi|ChlNC64A_1|34493|estExt_Genewise1Plus.C_40493
Chlorella_variabilis	OG0000002
jgi|ChlNC64A_1|135726|IGS.gm_14_00259	Chlorella_variabilis
OG0000002
jgi|ChlNC64A_1|135872|IGS.gm_14_00405	Chlorella_variabilis
OG0000002
jgi|ChlNC64A_1|133694|IGS.gm_1_00830	Chlorella_variabilis
OG0000002
jgi|ChlNC64A_1|144903|IGS.gm_8_00231	Chlorella_variabilis
OG0000002
jgi|ChlNC64A_1|51958|fgenesh3_pg.C_scaffold_8000245
Chlorella_variabilis	OG0000002
jgi|ChlNC64A_1|58444|estExt_fgenesh3_pg.C_160078
Chlorella_variabilis	OG0000002
jgi|ChlNC64A_1|59068|estExt_fgenesh3_pg.C_250045
Chlorella_variabilis	OG0000002
jgi|ChlNC64A_1|54108|fgenesh3_pg.C_scaffold_19000133
Chlorella_variabilis	OG0000002
47692 pacid=27388559 transcript=47692
locus=estExt_fgenesh1_pg.C_90186 ID=47692.2.0.227 annot-version=v2.0
	Coccomyxa_subellipsoidea OG0000002
55901 pacid=27385954 transcript=55901 locus=Genemark1.4930_g
ID=55901.2.0.227 annot-version=v2.0	Coccomyxa_subellipsoidea
OG0000002
66323 pacid=27388818 transcript=66323 locus=estExt_Genemark1.C_90106
ID=66323.2.0.227 annot-version=v2.0	Coccomyxa_subellipsoidea
OG0000002
38919 pacid=27389602 transcript=38919 locus=fgenesh1_pg.1_#_112
ID=38919.2.0.227 annot-version=v2.0	Coccomyxa_subellipsoidea
OG0000002
61653 pacid=27390045 transcript=61653 locus=Genemark1.2443_g
ID=61653.2.0.227 annot-version=v2.0	Coccomyxa_subellipsoidea
OG0000002
46623 pacid=27390120 transcript=46623
locus=estExt_fgenesh1_pg.C_40150 ID=46623.2.0.227 annot-version=v2.0
	Coccomyxa_subellipsoidea OG0000002
40616 pacid=27390288 transcript=40616 locus=fgenesh1_pg.4_#_265
ID=40616.2.0.227 annot-version=v2.0	Coccomyxa_subellipsoidea
OG0000002
17988 pacid=27386124 transcript=17988 locus=e_gw1.13.41.1
ID=17988.2.0.227 annot-version=v2.0	Coccomyxa_subellipsoidea
OG0000002
62850 pacid=27391422 transcript=62850 locus=Genemark1.3633_g
ID=62850.2.0.227 annot-version=v2.0	Coccomyxa_subellipsoidea
OG0000002
17392 pacid=27391523 transcript=17392 locus=e_gw1.12.52.1

ID=17392.2.0.227 annot-version=v2.0	Coccomyxa_subellipsoidea
OG0000002
17399 pacid=27391859 transcript=17399 locus=e_gw1.12.284.1
ID=17399.2.0.227 annot-version=v2.0	Coccomyxa_subellipsoidea
OG0000002
56512 pacid=27392068 transcript=56512 locus=Genemark1.5541_g
ID=56512.2.0.227 annot-version=v2.0	Coccomyxa_subellipsoidea
OG0000002
64515 pacid=27393705 transcript=64515 locus=estExt_Genemark1.C_20240
ID=64515.2.0.227 annot-version=v2.0	Coccomyxa_subellipsoidea
OG0000002
59088 pacid=27393768 transcript=59088 locus=Genemark1.1050_g
ID=59088.2.0.227 annot-version=v2.0	Coccomyxa_subellipsoidea
OG0000002
68079 pacid=27395120 transcript=68079
locus=estExt_Genemark1.C_210108 ID=68079.2.0.227 annot-version=v2.0
Coccomyxa_subellipsoidea	OG0000002
20857_e_gw1.13.00.74.1	Ostreococcus_tauri	OG0000002
4632_gw1.17.00.141.1	Ostreococcus_tauri	OG0000002
23798_estExt_fgenesh1_pg.C_Chr_06.00010075	Ostreococcus_tauri
OG0000002
33612_0600010459 Ostreococcus_tauri	OG0000002
34108_0700010461 Ostreococcus_tauri	OG0000002
36704_1400010355 Ostreococcus_tauri	OG0000002
9597_fgenesh1_pg.C_Chr_01.0001000363	Ostreococcus_tauri
OG0000002
24879 pacid=27414387 transcript=24879
locus=estExt_fgenesh1_pg.C_Chr_70353 ID=24879.2.0.231 annot-
version=v2.0	Ostreococcus_lucimarinus	OG0000002
14352 pacid=27414729 transcript=14352
locus=fgenesh1_pg.C_Chr_2000295 ID=14352.2.0.231 annot-version=v2.0
Ostreococcus_lucimarinus	OG0000002
19174 pacid=27415127 transcript=19174
locus=fgenesh1_pg.C_Chr_21000017 ID=19174.2.0.231 annot-version=v2.0
	Ostreococcus_lucimarinus	OG0000002
2458 pacid=27415773 transcript=2458 locus=gwEuk.16.58.1
ID=2458.2.0.231 annot-version=v2.0	Ostreococcus_lucimarinus
OG0000002
29142 pacid=27417564 transcript=29142 locus=eugene.0100010536
ID=29142.2.0.231 annot-version=v2.0	Ostreococcus_lucimarinus
OG0000002
9534 pacid=27418301 transcript=9534 locus=gwEuk.6.625.1
ID=9534.2.0.231 annot-version=v2.0	Ostreococcus_lucimarinus
OG0000002
32043 pacid=27418533 transcript=32043 locus=eugene.0600010102
ID=32043.2.0.231 annot-version=v2.0	Ostreococcus_lucimarinus
OG0000002
89032 pacid=27418769 transcript=89032 locus=ost_13_013_053
ID=89032.2.0.231 annot-version=v2.0	Ostreococcus_lucimarinus
OG0000002
29618 pacid=27420272 transcript=29618 locus=eugene.2000010324
ID=29618.2.0.231 annot-version=v2.0	Ostreococcus_lucimarinus
OG0000002
190475 pacid=27340056 transcript=190515 locus=gm1.7596_g

ID=190515.3.0.228 annot-version=v3.0   Micromonas_pusilla
OG0000002
5717 pacid=27340799 transcript=5717 locus=MicpuC2.gw1.7.275.1
ID=5717.3.0.228 annot-version=v3.0   Micromonas_pusilla
OG0000002
63754 pacid=27342069 transcript=63754 locus=MicpuC2.est_orfs.
14_2737_4276191:1 ID=63754.3.0.228 annot-version=v3.0
Micromonas_pusilla   OG0000002
203382 pacid=27342837 transcript=203422 locus=fgenesh1_pm.2_#_468
ID=203422.3.0.228 annot-version=v3.0   Micromonas_pusilla
OG0000002
165030 pacid=27343295 transcript=165070 locus=e_gw1.16.389.1
ID=165070.3.0.228 annot-version=v3.0   Micromonas_pusilla
OG0000002
8438 pacid=27345195 transcript=8438 locus=MicpuC2.gw1.1.961.1
ID=8438.3.0.228 annot-version=v3.0   Micromonas_pusilla
OG0000002
168788 pacid=27347247 transcript=168828
locus=estExt_Genewise1.C_3_t40044 ID=168828.3.0.228 annot-
version=v3.0   Micromonas_pusilla   OG0000002
193917 pacid=27348087 transcript=193957
locus=estExt_Genemark1.C_6_t10200 ID=193957.3.0.228 annot-
version=v3.0   Micromonas_pusilla   OG0000002
58811 pacid=27395411 transcript=58811 locus=EuGene.0500010544
ID=58811.3.0.229 annot-version=v3.0   Micromonas_sp._RCC299
OG0000002
53601 pacid=27397017 transcript=53601
locus=est_orfs.Chr_16_3472_3200725:1 ID=53601.3.0.229 annot-
version=v3.0   Micromonas_sp._RCC299   OG0000002
79939 pacid=27397852 transcript=79939 locus=e_gw2.03.65.1
ID=79939.3.0.229 annot-version=v3.0   Micromonas_sp._RCC299
OG0000002
55164 pacid=27398837 transcript=55164 locus=EuGene.0100010391
ID=55164.3.0.229 annot-version=v3.0   Micromonas_sp._RCC299
OG0000002
64554 pacid=27401106 transcript=64554 locus=EuGene.1500010275
ID=64554.3.0.229 annot-version=v3.0   Micromonas_sp._RCC299
OG0000002
68304 pacid=27395867 transcript=68304 locus=gw2.05.221.1
ID=68304.3.0.229 annot-version=v3.0   Micromonas_sp._RCC299
OG0000002
71611 pacid=27402962 transcript=71611 locus=gw2.13.322.1
ID=71611.3.0.229 annot-version=v3.0   Micromonas_sp._RCC299
OG0000002
60068 pacid=27404342 transcript=60068 locus=EuGene.0700010353
ID=60068.3.0.229 annot-version=v3.0   Micromonas_sp._RCC299
OG0000002
kfl00412_0050_v1.1   Klebsormidium_flaccidum OG0000002
kfl00414_0100_v1.1   Klebsormidium_flaccidum OG0000002
kfl00485_0030_v1.1   Klebsormidium_flaccidum OG0000002
kfl00502_0050_v1.1   Klebsormidium_flaccidum OG0000002
kfl00651_0040_v1.1   Klebsormidium_flaccidum OG0000002
kfl00656_0040_v1.1   Klebsormidium_flaccidum OG0000002
kfl00020_0290_v1.1   Klebsormidium_flaccidum OG0000002

kfl00020_0390_v1.1 Klebsormidium_flaccidum OG0000002
kfl00735_g3_v1.1 Klebsormidium_flaccidum OG0000002
kfl00784_0030_v1.1 Klebsormidium_flaccidum OG0000002
kfl00021_0530_v1.1 Klebsormidium_flaccidum OG0000002
kfl00916_0020_v1.1 Klebsormidium_flaccidum OG0000002
kfl00957_0050_v1.1 Klebsormidium_flaccidum OG0000002
kfl01395_0010_v1.1 Klebsormidium_flaccidum OG0000002
kfl00041_0130_v1.1 Klebsormidium_flaccidum OG0000002
kfl00065_0080_v1.1 Klebsormidium_flaccidum OG0000002
kfl00005_0390_v1.1 Klebsormidium_flaccidum OG0000002
kfl00068_0080_v1.1 Klebsormidium_flaccidum OG0000002
kfl00068_0340_v1.1 Klebsormidium_flaccidum OG0000002
kfl00070_0140_v1.1 Klebsormidium_flaccidum OG0000002
kfl00005_0590_v1.1 Klebsormidium_flaccidum OG0000002
kfl00096_0090_v1.1 Klebsormidium_flaccidum OG0000002
kfl00098_0220_v1.1 Klebsormidium_flaccidum OG0000002
kfl00110_0360_v1.1 Klebsormidium_flaccidum OG0000002
kfl00131_0190_v1.1 Klebsormidium_flaccidum OG0000002
kfl00136_0255_v1.1 Klebsormidium_flaccidum OG0000002
kfl00146_0160_v1.1 Klebsormidium_flaccidum OG0000002
kfl00151_0160_v1.1 Klebsormidium_flaccidum OG0000002
kfl00162_0130_v1.1 Klebsormidium_flaccidum OG0000002
kfl00162_0170_v1.1 Klebsormidium_flaccidum OG0000002
kfl00163_0180_v1.1 Klebsormidium_flaccidum OG0000002
kfl00009_0560_v1.1 Klebsormidium_flaccidum OG0000002
kfl00009_0620_v1.1 Klebsormidium_flaccidum OG0000002
kfl00009_0670_v1.1 Klebsormidium_flaccidum OG0000002
kfl00009_0780_v1.1 Klebsormidium_flaccidum OG0000002
kfl00182_0140_v1.1 Klebsormidium_flaccidum OG0000002
kfl00183_0060_v1.1 Klebsormidium_flaccidum OG0000002
kfl00194_0240_v1.1 Klebsormidium_flaccidum OG0000002
kfl00230_0100_v1.1 Klebsormidium_flaccidum OG0000002
kfl00254_0030_v1.1 Klebsormidium_flaccidum OG0000002
kfl00271_0140_v1.1 Klebsormidium_flaccidum OG0000002
kfl00273_0050_v1.1 Klebsormidium_flaccidum OG0000002
kfl00281_0110_v1.1 Klebsormidium_flaccidum OG0000002
kfl00326_0100_v1.1 Klebsormidium_flaccidum OG0000002
Pp3c15_12010V3.1.p pacid=32927168 transcript=Pp3c15_12010V3.1 locus=Pp3c15_12010 annot-version=v3.3 Physcomitrella_patens OG0000002
Pp3c10_19570V3.1.p pacid=32902332 transcript=Pp3c10_19570V3.1 locus=Pp3c10_19570 annot-version=v3.3 Physcomitrella_patens OG0000002
Pp3c13_17120V3.1.p pacid=32930214 transcript=Pp3c13_17120V3.1 locus=Pp3c13_17120 annot-version=v3.3 Physcomitrella_patens OG0000002
Pp3c2_1560V3.1.p pacid=32933351 transcript=Pp3c2_1560V3.1 locus=Pp3c2_1560 annot-version=v3.3 Physcomitrella_patens OG0000002
Pp3c2_1940V3.1.p pacid=32934602 transcript=Pp3c2_1940V3.1 locus=Pp3c2_1940 annot-version=v3.3 Physcomitrella_patens OG0000002
Pp3c2_27580V3.1.p pacid=32934619 transcript=Pp3c2_27580V3.1 locus=Pp3c2_27580 annot-version=v3.3 Physcomitrella_patens

OG0000002
Pp3c2_36080V3.1.p pacid=32935939 transcript=Pp3c2_36080V3.1
locus=Pp3c2_36080 annot-version=v3.3 Physcomitrella_patens
OG0000002
Pp3c2_12230V3.1.p pacid=32937314 transcript=Pp3c2_12230V3.1
locus=Pp3c2_12230 annot-version=v3.3 Physcomitrella_patens
OG0000002
Pp3c19_930V3.1.p pacid=32938509 transcript=Pp3c19_930V3.1
locus=Pp3c19_930 annot-version=v3.3 Physcomitrella_patens
OG0000002
Pp3c3_24770V3.1.p pacid=32940540 transcript=Pp3c3_24770V3.1
locus=Pp3c3_24770 annot-version=v3.3 Physcomitrella_patens
OG0000002
Pp3c3_19290V3.1.p pacid=32941270 transcript=Pp3c3_19290V3.1
locus=Pp3c3_19290 annot-version=v3.3 Physcomitrella_patens
OG0000002
Pp3c22_3070V3.1.p pacid=32903707 transcript=Pp3c22_3070V3.1
locus=Pp3c22_3070 annot-version=v3.3 Physcomitrella_patens
OG0000002
Pp3c3_5760V3.1.p pacid=32941739 transcript=Pp3c3_5760V3.1
locus=Pp3c3_5760 annot-version=v3.3 Physcomitrella_patens
OG0000002
Pp3c22_3080V3.1.p pacid=32903826 transcript=Pp3c22_3080V3.1
locus=Pp3c22_3080 annot-version=v3.3 Physcomitrella_patens
OG0000002
Pp3c3_5764V3.1.p pacid=32943165 transcript=Pp3c3_5764V3.1
locus=Pp3c3_5764 annot-version=v3.3 Physcomitrella_patens
OG0000002
Pp3c3_2440V3.1.p pacid=32943906 transcript=Pp3c3_2440V3.1
locus=Pp3c3_2440 annot-version=v3.3 Physcomitrella_patens
OG0000002
Pp3c3_19780V3.1.p pacid=32944267 transcript=Pp3c3_19780V3.1
locus=Pp3c3_19780 annot-version=v3.3 Physcomitrella_patens
OG0000002
Pp3c20_15110V3.1.p pacid=32947953 transcript=Pp3c20_15110V3.1
locus=Pp3c20_15110 annot-version=v3.3 Physcomitrella_patens
OG0000002
Pp3c23_13490V3.1.p pacid=32949074 transcript=Pp3c23_13490V3.1
locus=Pp3c23_13490 annot-version=v3.3 Physcomitrella_patens
OG0000002
Pp3c27_5540V3.1.p pacid=32951699 transcript=Pp3c27_5540V3.1
locus=Pp3c27_5540 annot-version=v3.3 Physcomitrella_patens
OG0000002
Pp3c5_2770V3.1.p pacid=32953098 transcript=Pp3c5_2770V3.1
locus=Pp3c5_2770 annot-version=v3.3 Physcomitrella_patens
OG0000002
Pp3c5_2530V3.1.p pacid=32953388 transcript=Pp3c5_2530V3.1
locus=Pp3c5_2530 annot-version=v3.3 Physcomitrella_patens
OG0000002
Pp3c5_26320V3.1.p pacid=32954879 transcript=Pp3c5_26320V3.1
locus=Pp3c5_26320 annot-version=v3.3 Physcomitrella_patens
OG0000002
Pp3c5_10110V3.1.p pacid=32954904 transcript=Pp3c5_10110V3.1
locus=Pp3c5_10110 annot-version=v3.3 Physcomitrella_patens

OG0000002
Pp3c5_16950V3.1.p pacid=32955121 transcript=Pp3c5_16950V3.1
locus=Pp3c5_16950 annot-version=v3.3 Physcomitrella_patens
OG0000002
Pp3c22_3230V3.1.p pacid=32905138 transcript=Pp3c22_3230V3.1
locus=Pp3c22_3230 annot-version=v3.3 Physcomitrella_patens
OG0000002
Pp3c5_15090V3.1.p pacid=32955716 transcript=Pp3c5_15090V3.1
locus=Pp3c5_15090 annot-version=v3.3 Physcomitrella_patens
OG0000002
Pp3c5_7610V3.1.p pacid=32955957 transcript=Pp3c5_7610V3.1
locus=Pp3c5_7610 annot-version=v3.3 Physcomitrella_patens
OG0000002
Pp3c11_21930V3.1.p pacid=32956322 transcript=Pp3c11_21930V3.1
locus=Pp3c11_21930 annot-version=v3.3 Physcomitrella_patens
OG0000002
Pp3c11_2930V3.1.p pacid=32956978 transcript=Pp3c11_2930V3.1
locus=Pp3c11_2930 annot-version=v3.3 Physcomitrella_patens
OG0000002
Pp3c11_11830V3.1.p pacid=32957890 transcript=Pp3c11_11830V3.1
locus=Pp3c11_11830 annot-version=v3.3 Physcomitrella_patens
OG0000002
Pp3c11_7720V3.1.p pacid=32958137 transcript=Pp3c11_7720V3.1
locus=Pp3c11_7720 annot-version=v3.3 Physcomitrella_patens
OG0000002
Pp3c14_19310V3.1.p pacid=32960417 transcript=Pp3c14_19310V3.1
locus=Pp3c14_19310 annot-version=v3.3 Physcomitrella_patens
OG0000002
Pp3c14_15490V3.1.p pacid=32960790 transcript=Pp3c14_15490V3.1
locus=Pp3c14_15490 annot-version=v3.3 Physcomitrella_patens
OG0000002
Pp3c17_2130V3.1.p pacid=32905695 transcript=Pp3c17_2130V3.1
locus=Pp3c17_2130 annot-version=v3.3 Physcomitrella_patens
OG0000002
Pp3c14_16110V3.1.p pacid=32963317 transcript=Pp3c14_16110V3.1
locus=Pp3c14_16110 annot-version=v3.3 Physcomitrella_patens
OG0000002
Pp3c8_15500V3.1.p pacid=32963734 transcript=Pp3c8_15500V3.1
locus=Pp3c8_15500 annot-version=v3.3 Physcomitrella_patens
OG0000002
Pp3c17_24090V3.1.p pacid=32905926 transcript=Pp3c17_24090V3.1
locus=Pp3c17_24090 annot-version=v3.3 Physcomitrella_patens
OG0000002
Pp3c8_4280V3.1.p pacid=32964473 transcript=Pp3c8_4280V3.1
locus=Pp3c8_4280 annot-version=v3.3 Physcomitrella_patens
OG0000002
Pp3c8_15040V3.1.p pacid=32965525 transcript=Pp3c8_15040V3.1
locus=Pp3c8_15040 annot-version=v3.3 Physcomitrella_patens
OG0000002
Pp3c8_13830V3.1.p pacid=32965636 transcript=Pp3c8_13830V3.1
locus=Pp3c8_13830 annot-version=v3.3 Physcomitrella_patens
OG0000002
Pp3c17_23250V3.1.p pacid=32906627 transcript=Pp3c17_23250V3.1
locus=Pp3c17_23250 annot-version=v3.3 Physcomitrella_patens

OG0000002
Pp3c1_6490V3.1.p pacid=32971750 transcript=Pp3c1_6490V3.1
locus=Pp3c1_6490 annot-version=v3.3 Physcomitrella_patens
OG0000002
Pp3c12_14320V3.1.p pacid=32973707 transcript=Pp3c12_14320V3.1
locus=Pp3c12_14320 annot-version=v3.3 Physcomitrella_patens
OG0000002
Pp3c12_4690V3.1.p pacid=32974136 transcript=Pp3c12_4690V3.1
locus=Pp3c12_4690 annot-version=v3.3 Physcomitrella_patens
OG0000002
Pp3c12_2390V3.1.p pacid=32974749 transcript=Pp3c12_2390V3.1
locus=Pp3c12_2390 annot-version=v3.3 Physcomitrella_patens
OG0000002
Pp3c6_26210V3.1.p pacid=32976226 transcript=Pp3c6_26210V3.1
locus=Pp3c6_26210 annot-version=v3.3 Physcomitrella_patens
OG0000002
Pp3c6_15900V3.1.p pacid=32977104 transcript=Pp3c6_15900V3.1
locus=Pp3c6_15900 annot-version=v3.3 Physcomitrella_patens
OG0000002
Pp3c6_23550V3.1.p pacid=32977713 transcript=Pp3c6_23550V3.1
locus=Pp3c6_23550 annot-version=v3.3 Physcomitrella_patens
OG0000002
Pp3c6_3910V3.1.p pacid=32978122 transcript=Pp3c6_3910V3.1
locus=Pp3c6_3910 annot-version=v3.3 Physcomitrella_patens
OG0000002
Pp3c6_26920V3.1.p pacid=32978729 transcript=Pp3c6_26920V3.1
locus=Pp3c6_26920 annot-version=v3.3 Physcomitrella_patens
OG0000002
Pp3c17_11510V3.1.p pacid=32907515 transcript=Pp3c17_11510V3.1
locus=Pp3c17_11510 annot-version=v3.3 Physcomitrella_patens
OG0000002
Pp3c17_5040V3.1.p pacid=32907574 transcript=Pp3c17_5040V3.1
locus=Pp3c17_5040 annot-version=v3.3 Physcomitrella_patens
OG0000002
Pp3c17_13520V3.1.p pacid=32907679 transcript=Pp3c17_13520V3.1
locus=Pp3c17_13520 annot-version=v3.3 Physcomitrella_patens
OG0000002
Pp3c16_5890V3.1.p pacid=32984169 transcript=Pp3c16_5890V3.1
locus=Pp3c16_5890 annot-version=v3.3 Physcomitrella_patens
OG0000002
Pp3c16_280V3.1.p pacid=32985011 transcript=Pp3c16_280V3.1
locus=Pp3c16_280 annot-version=v3.3 Physcomitrella_patens
OG0000002
Pp3c16_5160V3.1.p pacid=32985060 transcript=Pp3c16_5160V3.1
locus=Pp3c16_5160 annot-version=v3.3 Physcomitrella_patens
OG0000002
Pp3c16_9240V3.1.p pacid=32985556 transcript=Pp3c16_9240V3.1
locus=Pp3c16_9240 annot-version=v3.3 Physcomitrella_patens
OG0000002
Pp3c16_4140V3.1.p pacid=32985889 transcript=Pp3c16_4140V3.1
locus=Pp3c16_4140 annot-version=v3.3 Physcomitrella_patens
OG0000002
Pp3c16_9420V3.1.p pacid=32986242 transcript=Pp3c16_9420V3.1
locus=Pp3c16_9420 annot-version=v3.3 Physcomitrella_patens

OG0000002
Pp3c24_8560V3.1.p pacid=32908988 transcript=Pp3c24_8560V3.1
locus=Pp3c24_8560 annot-version=v3.3 Physcomitrella_patens
OG0000002
Pp3c10_24680V3.1.p pacid=32900372 transcript=Pp3c10_24680V3.1
locus=Pp3c10_24680 annot-version=v3.3 Physcomitrella_patens
OG0000002
Pp3c24_4430V3.1.p pacid=32909511 transcript=Pp3c24_4430V3.1
locus=Pp3c24_4430 annot-version=v3.3 Physcomitrella_patens
OG0000002
Pp3c24_14870V3.1.p pacid=32910852 transcript=Pp3c24_14870V3.1
locus=Pp3c24_14870 annot-version=v3.3 Physcomitrella_patens
OG0000002
Pp3c9_6880V3.1.p pacid=32912411 transcript=Pp3c9_6880V3.1
locus=Pp3c9_6880 annot-version=v3.3 Physcomitrella_patens
OG0000002
Pp3c21_12360V3.1.p pacid=32915161 transcript=Pp3c21_12360V3.1
locus=Pp3c21_12360 annot-version=v3.3 Physcomitrella_patens
OG0000002
Pp3c26_11100V3.1.p pacid=32917687 transcript=Pp3c26_11100V3.1
locus=Pp3c26_11100 annot-version=v3.3 Physcomitrella_patens
OG0000002
Pp3c10_24670V3.1.p pacid=32901233 transcript=Pp3c10_24670V3.1
locus=Pp3c10_24670 annot-version=v3.3 Physcomitrella_patens
OG0000002
Pp3c26_10760V3.1.p pacid=32917768 transcript=Pp3c26_10760V3.1
locus=Pp3c26_10760 annot-version=v3.3 Physcomitrella_patens
OG0000002
Pp3c4_14490V3.1.p pacid=32919715 transcript=Pp3c4_14490V3.1
locus=Pp3c4_14490 annot-version=v3.3 Physcomitrella_patens
OG0000002
Pp3c4_14140V3.1.p pacid=32920133 transcript=Pp3c4_14140V3.1
locus=Pp3c4_14140 annot-version=v3.3 Physcomitrella_patens
OG0000002
Pp3c4_16600V3.1.p pacid=32921905 transcript=Pp3c4_16600V3.1
locus=Pp3c4_16600 annot-version=v3.3 Physcomitrella_patens
OG0000002
Pp3c4_4900V3.1.p pacid=32921931 transcript=Pp3c4_4900V3.1
locus=Pp3c4_4900 annot-version=v3.3 Physcomitrella_patens
OG0000002
Pp3c7_17210V3.1.p pacid=32922903 transcript=Pp3c7_17210V3.1
locus=Pp3c7_17210 annot-version=v3.3 Physcomitrella_patens
OG0000002
Pp3c7_22430V3.1.p pacid=32925734 transcript=Pp3c7_22430V3.1
locus=Pp3c7_22430 annot-version=v3.3 Physcomitrella_patens
OG0000002
Pp3c7_10060V3.1.p pacid=32925873 transcript=Pp3c7_10060V3.1
locus=Pp3c7_10060 annot-version=v3.3 Physcomitrella_patens
OG0000002
AT2G34400.1 | Pentatricopeptide repeat (PPR-like) superfamily
protein | Chr2:14516226-14518186 FORWARD LENGTH=621 | 201606
Arabidopsis_thaliana OG0000002
AT2G35130.1 | Tetratricopeptide repeat (TPR)-like superfamily
protein | Chr2:14807589-14810164 REVERSE LENGTH=591 | 201606

Arabidopsis_thaliana   OG0000002
AT2G36240.1 | pentatricopeptide (PPR) repeat-containing protein | Chr2:15195663-15197156 FORWARD LENGTH=497 | 201606
Arabidopsis_thaliana   OG0000002
AT2G37230.1 | Tetratricopeptide repeat (TPR)-like superfamily protein | Chr2:15637177-15639450 REVERSE LENGTH=757 | 201606
Arabidopsis_thaliana   OG0000002
AT2G38420.1 | Pentatricopeptide repeat (PPR) superfamily protein | Chr2:16091093-16092454 FORWARD LENGTH=453 | 201606
Arabidopsis_thaliana   OG0000002
AT2G39230.1 | LATERAL ORGAN JUNCTION | Chr2:16381647-16384250 FORWARD LENGTH=867 | 201606   Arabidopsis_thaliana
OG0000002
AT2G39620.1 | Pentatricopeptide repeat (PPR) superfamily protein | Chr2:16518968-16521478 REVERSE LENGTH=836 | 201606
Arabidopsis_thaliana   OG0000002
AT2G40720.1 | Tetratricopeptide repeat (TPR)-like superfamily protein | Chr2:16987269-16989851 FORWARD LENGTH=860 | 201606
Arabidopsis_thaliana   OG0000002
AT1G10910.1 | Pentatricopeptide repeat (PPR) superfamily protein | Chr1:3639908-3643974 FORWARD LENGTH=664 | 201606
Arabidopsis_thaliana   OG0000002
AT2G41720.1 | Tetratricopeptide repeat (TPR)-like superfamily protein | Chr2:17403744-17407127 REVERSE LENGTH=822 | 201606
Arabidopsis_thaliana   OG0000002
AT1G02060.1 | Tetratricopeptide repeat (TPR)-like superfamily protein | Chr1:360918-363050 REVERSE LENGTH=710 | 201606
Arabidopsis_thaliana   OG0000002
AT1G11290.1 | Pentatricopeptide repeat (PPR) superfamily protein | Chr1:3791454-3793883 REVERSE LENGTH=809 | 201606
Arabidopsis_thaliana   OG0000002
AT2G45350.1 | Pentatricopeptide repeat (PPR) superfamily protein | Chr2:18694816-18696657 REVERSE LENGTH=613 | 201606
Arabidopsis_thaliana   OG0000002
AT2G46050.1 | Pentatricopeptide repeat (PPR-like) superfamily protein | Chr2:18939262-18941034 FORWARD LENGTH=590 | 201606
Arabidopsis_thaliana   OG0000002
AT3G01580.1 | Tetratricopeptide repeat (TPR)-like superfamily protein | Chr3:223529-225511 REVERSE LENGTH=660 | 201606
Arabidopsis_thaliana   OG0000002
AT1G11710.1 | Pentatricopeptide repeat (PPR) superfamily protein | Chr1:3948886-3950859 FORWARD LENGTH=657 | 201606
Arabidopsis_thaliana   OG0000002
AT3G03580.1 | Tetratricopeptide repeat (TPR)-like superfamily protein | Chr3:860695-863343 REVERSE LENGTH=882 | 201606
Arabidopsis_thaliana   OG0000002
AT1G11900.1 | Tetratricopeptide repeat (TPR)-like superfamily protein | Chr1:4013166-4014630 REVERSE LENGTH=367 | 201606
Arabidopsis_thaliana   OG0000002
AT3G04130.1 | Tetratricopeptide repeat (TPR)-like superfamily protein | Chr3:1084136-1085662 FORWARD LENGTH=508 | 201606
Arabidopsis_thaliana   OG0000002
AT3G04760.1 | Pentatricopeptide repeat (PPR-like) superfamily protein | Chr3:1303884-1305692 REVERSE LENGTH=602 | 201606

Arabidopsis_thaliana   OG0000002
AT3G06430.1 | Tetratricopeptide repeat (TPR)-like superfamily protein | Chr3:1956658-1958240 REVERSE LENGTH=486 | 201606 Arabidopsis_thaliana   OG0000002
AT3G06920.1 | Tetratricopeptide repeat (TPR)-like superfamily protein | Chr3:2181717-2184449 FORWARD LENGTH=871 | 201606 Arabidopsis_thaliana   OG0000002
AT3G07290.1 | Pentatricopeptide repeat (PPR) superfamily protein | Chr3:2321740-2324382 REVERSE LENGTH=880 | 201606 Arabidopsis_thaliana   OG0000002
AT1G12300.1 | Tetratricopeptide repeat (TPR)-like superfamily protein | Chr1:4184163-4186076 REVERSE LENGTH=637 | 201606 Arabidopsis_thaliana   OG0000002
AT3G09060.1 | Pentatricopeptide repeat (PPR) superfamily protein | Chr3:2766367-2768430 REVERSE LENGTH=687 | 201606 Arabidopsis_thaliana   OG0000002
AT3G09650.1 | Tetratricopeptide repeat (TPR)-like superfamily protein | Chr3:2958704-2961040 FORWARD LENGTH=778 | 201606 Arabidopsis_thaliana   OG0000002
AT1G12615.1 | pentatricopeptide (PPR) repeat protein | Chr1:4294055-4294540 REVERSE LENGTH=161 | 201606 Arabidopsis_thaliana   OG0000002
AT1G12620.1 | Pentatricopeptide repeat (PPR) superfamily protein | Chr1:4294883-4296748 REVERSE LENGTH=621 | 201606 Arabidopsis_thaliana   OG0000002
AT1G12700.1 | RNA processing FACTOR | Chr1:4324419-4326227 REVERSE LENGTH=602 | 201606   Arabidopsis_thaliana   OG0000002
AT1G12775.1 | Pentatricopeptide repeat (PPR) superfamily protein | Chr1:4353906-4355840 FORWARD LENGTH=644 | 201606 Arabidopsis_thaliana   OG0000002
AT3G12770.1 | mitochondrial editing factor 22 | Chr3:4057027-4059193 REVERSE LENGTH=694 | 201606   Arabidopsis_thaliana OG0000002
AT3G13150.1 | Tetratricopeptide repeat (TPR)-like superfamily protein | Chr3:4227975-4229630 REVERSE LENGTH=551 | 201606 Arabidopsis_thaliana   OG0000002
AT3G13160.1 | Tetratricopeptide repeat (TPR)-like superfamily protein | Chr3:4229994-4231178 REVERSE LENGTH=394 | 201606 Arabidopsis_thaliana   OG0000002
AT3G13770.1 | Pentatricopeptide repeat (PPR) superfamily protein | Chr3:4519647-4521533 FORWARD LENGTH=628 | 201606 Arabidopsis_thaliana   OG0000002
AT3G14330.1 | Tetratricopeptide repeat (TPR)-like superfamily protein | Chr3:4779688-4782451 REVERSE LENGTH=710 | 201606 Arabidopsis_thaliana   OG0000002
AT3G14580.1 | Pentatricopeptide repeat (PPR) superfamily protein | Chr3:4903012-4904229 FORWARD LENGTH=405 | 201606 Arabidopsis_thaliana   OG0000002
AT1G13040.1 | Pentatricopeptide repeat (PPR-like) superfamily protein | Chr1:4447647-4449200 FORWARD LENGTH=517 | 201606 Arabidopsis_thaliana   OG0000002
AT3G15200.1 | Tetratricopeptide repeat (TPR)-like superfamily protein | Chr3:5117489-5119060 REVERSE LENGTH=523 | 201606 Arabidopsis_thaliana   OG0000002

AT3G15930.1 | Pentatricopeptide repeat (PPR) superfamily protein | Chr3:5387444-5389690 FORWARD LENGTH=687 | 201606 Arabidopsis_thaliana OG0000002
AT3G16010.1 | Pentatricopeptide repeat (PPR-like) superfamily protein | Chr3:5434142-5436244 FORWARD LENGTH=642 | 201606 Arabidopsis_thaliana OG0000002
AT3G16610.1 | pentatricopeptide (PPR) repeat-containing protein | Chr3:5656371-5658335 REVERSE LENGTH=654 | 201606 Arabidopsis_thaliana OG0000002
AT3G16710.1 | Pentatricopeptide repeat (PPR) superfamily protein | Chr3:5690020-5691543 FORWARD LENGTH=507 | 201606 Arabidopsis_thaliana OG0000002
AT3G16890.1 | pentatricopeptide (PPR) domain protein 40 | Chr3:5768401-5770380 REVERSE LENGTH=659 | 201606 Arabidopsis_thaliana OG0000002
AT3G17370.1 | Pentatricopeptide repeat (PPR) superfamily protein | Chr3:5949006-5949644 REVERSE LENGTH=212 | 201606 Arabidopsis_thaliana OG0000002
AT3G18020.1 | Pentatricopeptide repeat (PPR) superfamily protein | Chr3:6165449-6167515 FORWARD LENGTH=688 | 201606 Arabidopsis_thaliana OG0000002
AT3G18110.1 | Pentatricopeptide repeat (PPR) superfamily protein | Chr3:6204940-6209691 REVERSE LENGTH=1440 | 201606 Arabidopsis_thaliana OG0000002
AT3G18840.2 | LOW protein Arabidopsis_thaliana OG0000002
AT3G18970.1 | mitochondrial editing factor 20 | Chr3:6543699-6545117 REVERSE LENGTH=472 | 201606 Arabidopsis_thaliana OG0000002
AT1G13630.1 | Tetratricopeptide repeat (TPR)-like superfamily protein | Chr1:4669784-4672826 REVERSE LENGTH=806 | 201606 Arabidopsis_thaliana OG0000002
AT3G21470.1 | Pentatricopeptide repeat (PPR-like) superfamily protein | Chr3:7563503-7565074 FORWARD LENGTH=523 | 201606 Arabidopsis_thaliana OG0000002
AT3G22470.1 | Pentatricopeptide repeat (PPR) superfamily protein | Chr3:7966066-7967925 REVERSE LENGTH=619 | 201606 Arabidopsis_thaliana OG0000002
AT1G13800.1 | Tetratricopeptide repeat (TPR)-like superfamily protein | Chr1:4731056-4733707 REVERSE LENGTH=883 | 201606 Arabidopsis_thaliana OG0000002
AT3G22670.1 | Pentatricopeptide repeat (PPR) superfamily protein | Chr3:8017771-8019459 REVERSE LENGTH=562 | 201606 Arabidopsis_thaliana OG0000002
AT3G22690.1 | LOW protein Arabidopsis_thaliana OG0000002
AT3G23020.1 | Tetratricopeptide repeat (TPR)-like superfamily protein | Chr3:8177215-8179743 REVERSE LENGTH=842 | 201606 Arabidopsis_thaliana OG0000002
AT3G25060.1 | Tetratricopeptide repeat (TPR)-like superfamily protein | Chr3:9128516-9130321 FORWARD LENGTH=601 | 201606 Arabidopsis_thaliana OG0000002
AT1G14470.1 | Pentatricopeptide repeat (PPR) superfamily protein | Chr1:4954080-4955702 FORWARD LENGTH=540 | 201606 Arabidopsis_thaliana OG0000002
AT3G29230.1 | Tetratricopeptide repeat (TPR)-like superfamily

protein | Chr3:11188803-11190605 FORWARD LENGTH=600 | 201606
Arabidopsis_thaliana OG0000002
AT3G29290.1 | Pentatricopeptide repeat (PPR) superfamily protein | Chr3:11238421-11240125 FORWARD LENGTH=540 | 201606
Arabidopsis_thaliana OG0000002
AT1G02420.1 | Pentatricopeptide repeat (PPR) superfamily protein | Chr1:493683-495158 FORWARD LENGTH=491 | 201606
Arabidopsis_thaliana OG0000002
AT3G46610.1 | Pentatricopeptide repeat (PPR-like) superfamily protein | Chr3:17160224-17162221 REVERSE LENGTH=665 | 201606
Arabidopsis_thaliana OG0000002
AT3G48810.1 | Pentatricopeptide repeat (PPR) superfamily protein | Chr3:18097048-18099027 FORWARD LENGTH=659 | 201606
Arabidopsis_thaliana OG0000002
AT3G49170.1 | Tetratricopeptide repeat (TPR)-like superfamily protein | Chr3:18226954-18229600 REVERSE LENGTH=850 | 201606
Arabidopsis_thaliana OG0000002
AT3G49240.1 | Pentatricopeptide repeat (PPR) superfamily protein | Chr3:18256086-18257975 FORWARD LENGTH=629 | 201606
Arabidopsis_thaliana OG0000002
AT3G49710.1 | Pentatricopeptide repeat (PPR) superfamily protein | Chr3:18437845-18440010 FORWARD LENGTH=721 | 201606
Arabidopsis_thaliana OG0000002
AT3G49730.1 | Tetratricopeptide repeat (TPR)-like superfamily protein | Chr3:18445730-18447646 REVERSE LENGTH=638 | 201606
Arabidopsis_thaliana OG0000002
AT3G49740.1 | Tetratricopeptide repeat (TPR)-like superfamily protein | Chr3:18447788-18450001 FORWARD LENGTH=737 | 201606
Arabidopsis_thaliana OG0000002
AT3G53170.1 | LOW protein Arabidopsis_thaliana OG0000002
AT3G53700.1 | Pentatricopeptide repeat (PPR) superfamily protein | Chr3:19900303-19902567 FORWARD LENGTH=754 | 201606
Arabidopsis_thaliana OG0000002
AT3G54980.1 | Pentatricopeptide repeat (PPR) superfamily protein | Chr3:20370293-20372848 FORWARD LENGTH=851 | 201606
Arabidopsis_thaliana OG0000002
AT3G56550.1 | Pentatricopeptide repeat (PPR) superfamily protein | Chr3:20952896-20954641 REVERSE LENGTH=581 | 201606
Arabidopsis_thaliana OG0000002
AT3G59040.1 | Tetratricopeptide repeat (TPR)-like superfamily protein | Chr3:21821495-21824233 REVERSE LENGTH=583 | 201606
Arabidopsis_thaliana OG0000002
AT3G60050.1 | Pentatricopeptide repeat (PPR) superfamily protein | Chr3:22180231-22181652 REVERSE LENGTH=473 | 201606
Arabidopsis_thaliana OG0000002
AT1G16480.1 | Tetratricopeptide repeat (TPR)-like superfamily protein | Chr1:5625843-5628656 REVERSE LENGTH=937 | 201606
Arabidopsis_thaliana OG0000002
AT3G61170.1 | Tetratricopeptide repeat (TPR)-like superfamily protein | Chr3:22638691-22641237 REVERSE LENGTH=783 | 201606
Arabidopsis_thaliana OG0000002
AT3G61360.1 | Tetratricopeptide repeat (TPR)-like superfamily protein | Chr3:22704630-22706126 REVERSE LENGTH=498 | 201606
Arabidopsis_thaliana OG0000002

AT3G61520.1 | Pentatricopeptide repeat (PPR) superfamily protein | Chr3:22768974-22771274 REVERSE LENGTH=766 | 201606
Arabidopsis_thaliana   OG0000002
AT3G62470.1 | Pentatricopeptide repeat (PPR) superfamily protein | Chr3:23106600-23108399 REVERSE LENGTH=599 | 201606
Arabidopsis_thaliana   OG0000002
AT3G62540.1 | Pentatricopeptide repeat (PPR) superfamily protein | Chr3:23133514-23135313 REVERSE LENGTH=599 | 201606
Arabidopsis_thaliana   OG0000002
AT3G62890.1 | Pentatricopeptide repeat (PPR) superfamily protein | Chr3:23246168-23247973 FORWARD LENGTH=573 | 201606
Arabidopsis_thaliana   OG0000002
AT3G63370.1 | Tetratricopeptide repeat (TPR)-like superfamily protein | Chr3:23402080-23404962 FORWARD LENGTH=960 | 201606
Arabidopsis_thaliana   OG0000002
AT1G16830.1 | Pentatricopeptide repeat (PPR) superfamily protein | Chr1:5760793-5762619 FORWARD LENGTH=608 | 201606
Arabidopsis_thaliana   OG0000002
AT4G01030.1 | pentatricopeptide (PPR) repeat-containing protein | Chr4:448336-450642 REVERSE LENGTH=768 | 201606
Arabidopsis_thaliana   OG0000002
AT4G01400.1 | oligomeric golgi complex subunit-like protein | Chr4:575843-577243 REVERSE LENGTH=466 | 201606
Arabidopsis_thaliana   OG0000002
AT4G01570.1 | Tetratricopeptide repeat (TPR)-like superfamily protein | Chr4:679487-681904 FORWARD LENGTH=805 | 201606
Arabidopsis_thaliana   OG0000002
AT4G04370.1 | Tetratricopeptide repeat (TPR)-like superfamily protein | Chr4:2134060-2136249 REVERSE LENGTH=729 | 201606
Arabidopsis_thaliana   OG0000002
AT4G08210.1 | Pentatricopeptide repeat (PPR-like) superfamily protein | Chr4:5183813-5185873 REVERSE LENGTH=686 | 201606
Arabidopsis_thaliana   OG0000002
AT1G17630.1 | Pentatricopeptide repeat (PPR-like) superfamily protein | Chr1:6064525-6066720 FORWARD LENGTH=731 | 201606
Arabidopsis_thaliana   OG0000002
AT4G11690.1 | Pentatricopeptide repeat (PPR-like) superfamily protein | Chr4:7056254-7057954 FORWARD LENGTH=566 | 201606
Arabidopsis_thaliana   OG0000002
AT4G13650.1 | Pentatricopeptide repeat (PPR) superfamily protein | Chr4:7939611-7942898 REVERSE LENGTH=1064 | 201606
Arabidopsis_thaliana   OG0000002
AT4G14820.1 | Pentatricopeptide repeat (PPR) superfamily protein | Chr4:8507794-8510038 REVERSE LENGTH=722 | 201606
Arabidopsis_thaliana   OG0000002
AT4G16390.1 | pentatricopeptide (PPR) repeat-containing protein | Chr4:9257985-9260093 FORWARD LENGTH=702 | 201606
Arabidopsis_thaliana   OG0000002
AT1G18485.1 | Pentatricopeptide repeat (PPR) superfamily protein | Chr1:6363172-6366084 FORWARD LENGTH=970 | 201606
Arabidopsis_thaliana   OG0000002
AT4G18840.1 | Pentatricopeptide repeat (PPR-like) superfamily protein | Chr4:10338719-10340356 REVERSE LENGTH=545 | 201606
Arabidopsis_thaliana   OG0000002

AT4G19191.1 | Tetratricopeptide repeat (TPR)-like superfamily protein | Chr4:10496228-10498192 FORWARD LENGTH=654 | 201606 Arabidopsis_thaliana OG0000002
AT4G19440.1 | Tetratricopeptide repeat (TPR)-like superfamily protein | Chr4:10602006-10604522 REVERSE LENGTH=838 | 201606 Arabidopsis_thaliana OG0000002
AT4G19890.1 | Pentatricopeptide repeat (PPR-like) superfamily protein | Chr4:10786948-10789053 REVERSE LENGTH=701 | 201606 Arabidopsis_thaliana OG0000002
AT4G20090.1 | Pentatricopeptide repeat (PPR) superfamily protein | Chr4:10868400-10870382 REVERSE LENGTH=660 | 201606 Arabidopsis_thaliana OG0000002
AT4G20740.1 | Pentatricopeptide repeat (PPR-like) superfamily protein | Chr4:11126151-11128334 FORWARD LENGTH=727 | 201606 Arabidopsis_thaliana OG0000002
AT1G18900.1 | Pentatricopeptide repeat (PPR) superfamily protein | Chr1:6529778-6532360 FORWARD LENGTH=860 | 201606 Arabidopsis_thaliana OG0000002
AT4G22760.1 | Tetratricopeptide repeat (TPR)-like superfamily protein | Chr4:11960553-11962289 FORWARD LENGTH=578 | 201606 Arabidopsis_thaliana OG0000002
AT1G19290.1 | Pentatricopeptide repeat (PPR) superfamily protein | Chr1:6666249-6668963 FORWARD LENGTH=904 | 201606 Arabidopsis_thaliana OG0000002
AT4G26680.1 | Tetratricopeptide repeat (TPR)-like superfamily protein | Chr4:13454853-13456418 FORWARD LENGTH=521 | 201606 Arabidopsis_thaliana OG0000002
AT4G26800.1 | Pentatricopeptide repeat (PPR) superfamily protein | Chr4:13490251-13491458 FORWARD LENGTH=369 | 201606 Arabidopsis_thaliana OG0000002
AT4G28010.1 | Tetratricopeptide repeat (TPR)-like superfamily protein | Chr4:13930379-13932493 FORWARD LENGTH=704 | 201606 Arabidopsis_thaliana OG0000002
AT1G19720.1 | Pentatricopeptide repeat (PPR-like) superfamily protein | Chr1:6819926-6822610 REVERSE LENGTH=894 | 201606 Arabidopsis_thaliana OG0000002
AT4G30825.1 | Tetratricopeptide repeat (TPR)-like superfamily protein | Chr4:15009605-15012319 FORWARD LENGTH=904 | 201606 Arabidopsis_thaliana OG0000002
AT4G31070.1 | PPR superfamily protein | Chr4:15118696-15120570 REVERSE LENGTH=624 | 201606 Arabidopsis_thaliana OG0000002
AT4G31850.1 | proton gradient regulation 3 | Chr4:15403020-15406358 FORWARD LENGTH=1112 | 201606 Arabidopsis_thaliana OG0000002
AT4G33170.1 | Tetratricopeptide repeat (TPR)-like superfamily protein | Chr4:15995701-15998673 REVERSE LENGTH=990 | 201606 Arabidopsis_thaliana OG0000002
AT4G33990.1 | Tetratricopeptide repeat (TPR)-like superfamily protein | Chr4:16290141-16292612 REVERSE LENGTH=823 | 201606 Arabidopsis_thaliana OG0000002
AT4G34830.1 | Pentatricopeptide repeat (PPR) superfamily protein | Chr4:16599976-16605994 REVERSE LENGTH=1089 | 201606 Arabidopsis_thaliana OG0000002

AT1G20300.1 | Pentatricopeptide repeat (PPR) superfamily protein | Chr1:7029701-7031314 FORWARD LENGTH=537 | 201606 Arabidopsis_thaliana OG0000002
AT4G35130.1 | Tetratricopeptide repeat (TPR)-like superfamily protein | Chr4:16721084-16723498 REVERSE LENGTH=804 | 201606 Arabidopsis_thaliana OG0000002
AT4G36680.1 | Tetratricopeptide repeat (TPR)-like superfamily protein | Chr4:17292479-17293717 REVERSE LENGTH=412 | 201606 Arabidopsis_thaliana OG0000002
AT5G01110.1 | Tetratricopeptide repeat (TPR)-like superfamily protein | Chr5:42114-44303 REVERSE LENGTH=729 | 201606 Arabidopsis_thaliana OG0000002
AT5G02830.1 | Tetratricopeptide repeat (TPR)-like superfamily protein | Chr5:644458-648421 REVERSE LENGTH=852 | 201606 Arabidopsis_thaliana OG0000002
AT5G02860.1 | Pentatricopeptide repeat (PPR) superfamily protein | Chr5:654102-656561 FORWARD LENGTH=819 | 201606 Arabidopsis_thaliana OG0000002
AT5G04810.1 | pentatricopeptide (PPR) repeat-containing protein | Chr5:1390049-1393760 FORWARD LENGTH=952 | 201606 Arabidopsis_thaliana OG0000002
AT5G06400.1 | Pentatricopeptide repeat (PPR) superfamily protein | Chr5:1955959-1959051 FORWARD LENGTH=1030 | 201606 Arabidopsis_thaliana OG0000002
AT5G06540.1 | Pentatricopeptide repeat (PPR) superfamily protein | Chr5:1999181-2001049 REVERSE LENGTH=622 | 201606 Arabidopsis_thaliana OG0000002
AT5G08305.1 | Pentatricopeptide repeat (PPR) superfamily protein | Chr5:2670134-2671738 REVERSE LENGTH=534 | 201606 Arabidopsis_thaliana OG0000002
AT5G08310.1 | Tetratricopeptide repeat (TPR)-like superfamily protein | Chr5:2672756-2675254 REVERSE LENGTH=832 | 201606 Arabidopsis_thaliana OG0000002
AT5G08510.1 | Pentatricopeptide repeat (PPR) superfamily protein | Chr5:2753099-2754731 FORWARD LENGTH=511 | 201606 Arabidopsis_thaliana OG0000002
AT5G10690.1 | pentatricopeptide (PPR) repeat-containing protein / CBS domain-containing protein | Chr5:3374443-3377332 REVERSE LENGTH=580 | 201606 Arabidopsis_thaliana OG0000002
AT5G11310.1 | Pentatricopeptide repeat (PPR) superfamily protein | Chr5:3606490-3608409 FORWARD LENGTH=602 | 201606 Arabidopsis_thaliana OG0000002
AT5G12100.1 | pentatricopeptide (PPR) repeat-containing protein | Chr5:3911388-3913838 FORWARD LENGTH=816 | 201606 Arabidopsis_thaliana OG0000002
AT5G13270.1 | Pentatricopeptide repeat (PPR) superfamily protein | Chr5:4246954-4249212 REVERSE LENGTH=752 | 201606 Arabidopsis_thaliana OG0000002
AT5G13770.1 | Pentatricopeptide repeat (PPR-like) superfamily protein | Chr5:4445461-4447290 FORWARD LENGTH=609 | 201606 Arabidopsis_thaliana OG0000002
AT5G14080.1 | Tetratricopeptide repeat (TPR)-like superfamily protein | Chr5:4543265-4545256 REVERSE LENGTH=634 | 201606 Arabidopsis_thaliana OG0000002

AT5G14770.1 | PPR repeat protein | Chr5:4772881-4775703 REVERSE LENGTH=940 | 201606	Arabidopsis_thaliana	OG0000002
AT5G14820.1 | Pentatricopeptide repeat (PPR) superfamily protein | Chr5:4792072-4793868 REVERSE LENGTH=598 | 201606	Arabidopsis_thaliana	OG0000002
AT5G15010.1 | Tetratricopeptide repeat (TPR)-like superfamily protein | Chr5:4857241-4858959 FORWARD LENGTH=572 | 201606	Arabidopsis_thaliana	OG0000002
AT5G15340.1 | Pentatricopeptide repeat (PPR) superfamily protein | Chr5:4982273-4984144 REVERSE LENGTH=623 | 201606	Arabidopsis_thaliana	OG0000002
AT5G16420.1 | Pentatricopeptide repeat (PPR-like) superfamily protein | Chr5:5368034-5369641 FORWARD LENGTH=535 | 201606	Arabidopsis_thaliana	OG0000002
AT5G16640.1 | Pentatricopeptide repeat (PPR) superfamily protein | Chr5:5461031-5462545 FORWARD LENGTH=504 | 201606	Arabidopsis_thaliana	OG0000002
AT5G18390.1 | Pentatricopeptide repeat (PPR) superfamily protein | Chr5:6090954-6092333 FORWARD LENGTH=459 | 201606	Arabidopsis_thaliana	OG0000002
AT5G18475.1 | Pentatricopeptide repeat (PPR) superfamily protein | Chr5:6129255-6130775 REVERSE LENGTH=506 | 201606	Arabidopsis_thaliana	OG0000002
AT5G18950.1 | Tetratricopeptide repeat (TPR)-like superfamily protein | Chr5:6328519-6329970 REVERSE LENGTH=483 | 201606	Arabidopsis_thaliana	OG0000002
AT5G21222.1 | protein kinase family protein | Chr5:7209422-7213700 FORWARD LENGTH=831 | 201606	Arabidopsis_thaliana	OG0000002
AT1G22960.1 | Pentatricopeptide repeat (PPR) superfamily protein | Chr1:8128086-8130242 REVERSE LENGTH=718 | 201606	Arabidopsis_thaliana	OG0000002
AT5G24830.1 | Tetratricopeptide repeat (TPR)-like superfamily protein | Chr5:8531226-8533266 FORWARD LENGTH=593 | 201606	Arabidopsis_thaliana	OG0000002
AT5G25630.1 | Tetratricopeptide repeat (TPR)-like superfamily protein | Chr5:8947426-8949424 FORWARD LENGTH=574 | 201606	Arabidopsis_thaliana	OG0000002
AT5G27110.1 | Tetratricopeptide repeat (TPR)-like superfamily protein | Chr5:9538572-9540647 REVERSE LENGTH=691 | 201606	Arabidopsis_thaliana	OG0000002
AT5G27270.1 | Tetratricopeptide repeat (TPR)-like superfamily protein | Chr5:9605650-9609625 FORWARD LENGTH=1038 | 201606	Arabidopsis_thaliana	OG0000002
AT5G28370.1 | Pentatricopeptide repeat (PPR) superfamily protein | Chr5:10332375-10334558 REVERSE LENGTH=727 | 201606	Arabidopsis_thaliana	OG0000002
AT5G28460.1 | Pentatricopeptide repeat (PPR) superfamily protein | Chr5:10374927-10377227 FORWARD LENGTH=766 | 201606	Arabidopsis_thaliana	OG0000002
AT5G36300.1 | Tetratricopeptide repeat (TPR)-like superfamily protein | Chr5:14320668-14322398 FORWARD LENGTH=284 | 201606	Arabidopsis_thaliana	OG0000002
AT5G38730.1 | Tetratricopeptide repeat (TPR)-like superfamily

protein | Chr5:15510901-15512691 FORWARD LENGTH=596 | 201606
Arabidopsis_thaliana   OG0000002
AT5G39350.1 | Tetratricopeptide repeat (TPR)-like superfamily protein | Chr5:15750929-15752962 FORWARD LENGTH=677 | 201606
Arabidopsis_thaliana   OG0000002
AT5G39710.1 | Tetratricopeptide repeat (TPR)-like superfamily protein | Chr5:15895729-15897972 FORWARD LENGTH=747 | 201606
Arabidopsis_thaliana   OG0000002
AT5G39980.1 | Tetratricopeptide repeat (TPR)-like superfamily protein | Chr5:16001036-16003072 REVERSE LENGTH=678 | 201606
Arabidopsis_thaliana   OG0000002
AT5G40400.1 | Pentatricopeptide repeat (PPR) superfamily protein | Chr5:16166444-16168276 FORWARD LENGTH=610 | 201606
Arabidopsis_thaliana   OG0000002
AT5G40410.1 | Tetratricopeptide repeat (TPR)-like superfamily protein | Chr5:16171385-16173211 FORWARD LENGTH=608 | 201606
Arabidopsis_thaliana   OG0000002
AT5G41170.1 | Pentatricopeptide repeat (PPR-like) superfamily protein | Chr5:16478860-16480443 REVERSE LENGTH=527 | 201606
Arabidopsis_thaliana   OG0000002
AT5G42310.1 | Pentatricopeptide repeat (PPR-like) superfamily protein | Chr5:16915860-16918238 FORWARD LENGTH=709 | 201606
Arabidopsis_thaliana   OG0000002
AT5G43790.1 | Pentatricopeptide repeat (PPR) superfamily protein | Chr5:17592099-17593481 REVERSE LENGTH=460 | 201606
Arabidopsis_thaliana   OG0000002
AT5G43820.1 | Pentatricopeptide repeat (PPR) superfamily protein | Chr5:17618948-17620588 FORWARD LENGTH=546 | 201606
Arabidopsis_thaliana   OG0000002
AT5G46100.1 | Pentatricopeptide repeat (PPR) superfamily protein | Chr5:18694316-18695734 REVERSE LENGTH=472 | 201606
Arabidopsis_thaliana   OG0000002
AT5G46460.1 | Pentatricopeptide repeat (PPR) superfamily protein | Chr5:18840305-18842398 FORWARD LENGTH=697 | 201606
Arabidopsis_thaliana   OG0000002
AT5G46580.1 | pentatricopeptide (PPR) repeat-containing protein | Chr5:18897510-18899645 REVERSE LENGTH=711 | 201606
Arabidopsis_thaliana   OG0000002
AT5G46680.1 | Pentatricopeptide repeat (PPR-like) superfamily protein | Chr5:18941118-18942524 FORWARD LENGTH=468 | 201606
Arabidopsis_thaliana   OG0000002
AT5G47360.1 | Tetratricopeptide repeat (TPR)-like superfamily protein | Chr5:19214445-19215878 REVERSE LENGTH=477 | 201606
Arabidopsis_thaliana   OG0000002
AT5G47460.1 | Pentatricopeptide repeat (PPR) superfamily protein | Chr5:19252463-19254193 REVERSE LENGTH=576 | 201606
Arabidopsis_thaliana   OG0000002
AT5G48730.1 | Pentatricopeptide repeat (PPR) superfamily protein | Chr5:19763152-19765136 FORWARD LENGTH=508 | 201606
Arabidopsis_thaliana   OG0000002
AT5G48910.1 | Pentatricopeptide repeat (PPR) superfamily protein | Chr5:19832969-19834909 REVERSE LENGTH=646 | 201606
Arabidopsis_thaliana   OG0000002
AT5G50280.1 | Pentatricopeptide repeat (PPR) superfamily protein |

Chr5:20459238-20461504 FORWARD LENGTH=723 | 201606
Arabidopsis_thaliana   OG0000002
AT5G50990.1 | Tetratricopeptide repeat (TPR)-like superfamily
protein | Chr5:20739453-20741281 FORWARD LENGTH=534 | 201606
Arabidopsis_thaliana   OG0000002
AT5G52850.1 | Pentatricopeptide repeat (PPR) superfamily protein |
Chr5:21414935-21417616 REVERSE LENGTH=893 | 201606
Arabidopsis_thaliana   OG0000002
AT5G55740.1 | Tetratricopeptide repeat (TPR)-like superfamily
protein | Chr5:22561941-22564433 REVERSE LENGTH=830 | 201606
Arabidopsis_thaliana   OG0000002
AT5G55840.1 | PPR superfamily protein | Chr5:22598038-22601688
FORWARD LENGTH=1136 | 201606   Arabidopsis_thaliana
OG0000002
AT5G56310.1 | Pentatricopeptide repeat (PPR) superfamily protein |
Chr5:22802322-22803914 FORWARD LENGTH=530 | 201606
Arabidopsis_thaliana   OG0000002
AT5G57250.1 | Pentatricopeptide repeat (PPR) superfamily protein |
Chr5:23195609-23198524 REVERSE LENGTH=971 | 201606
Arabidopsis_thaliana   OG0000002
AT5G59200.1 | Tetratricopeptide repeat (TPR)-like superfamily
protein | Chr5:23888793-23890427 REVERSE LENGTH=544 | 201606
Arabidopsis_thaliana   OG0000002
AT5G59600.1 | Tetratricopeptide repeat (TPR)-like superfamily
protein | Chr5:24011315-24012919 REVERSE LENGTH=534 | 201606
Arabidopsis_thaliana   OG0000002
AT5G59900.1 | Pentatricopeptide repeat (PPR) superfamily protein |
Chr5:24123983-24126706 REVERSE LENGTH=907 | 201606
Arabidopsis_thaliana   OG0000002
AT5G61400.1 | Pentatricopeptide repeat (PPR) superfamily protein |
Chr5:24681550-24683514 FORWARD LENGTH=654 | 201606
Arabidopsis_thaliana   OG0000002
AT5G61800.1 | Pentatricopeptide repeat (PPR) superfamily protein |
Chr5:24830054-24831553 REVERSE LENGTH=499 | 201606
Arabidopsis_thaliana   OG0000002
AT5G61990.1 | Pentatricopeptide repeat (PPR) superfamily protein |
Chr5:24900186-24903110 REVERSE LENGTH=974 | 201606
Arabidopsis_thaliana   OG0000002
AT5G62370.1 | Tetratricopeptide repeat (TPR)-like superfamily
protein | Chr5:25041901-25044849 REVERSE LENGTH=982 | 201606
Arabidopsis_thaliana   OG0000002
AT5G64320.1 | Pentatricopeptide repeat (PPR) superfamily protein |
Chr5:25723247-25725439 REVERSE LENGTH=730 | 201606
Arabidopsis_thaliana   OG0000002
AT1G03560.1 | Pentatricopeptide repeat (PPR-like) superfamily
protein | Chr1:890428-892410 REVERSE LENGTH=660 | 201606
Arabidopsis_thaliana   OG0000002
AT5G65560.1 | Pentatricopeptide repeat (PPR) superfamily protein |
Chr5:26201012-26203759 REVERSE LENGTH=915 | 201606
Arabidopsis_thaliana   OG0000002
AT5G65570.1 | Tetratricopeptide repeat (TPR)-like superfamily
protein | Chr5:26203968-26206184 FORWARD LENGTH=738 | 201606
Arabidopsis_thaliana   OG0000002
AT5G65820.1 | Pentatricopeptide repeat (PPR) superfamily protein |

Chr5:26339876-26341789 REVERSE LENGTH=637 | 201606
Arabidopsis_thaliana   OG0000002
AT5G66520.1 | Tetratricopeptide repeat (TPR)-like superfamily
protein | Chr5:26551879-26553741 FORWARD LENGTH=620 | 201606
Arabidopsis_thaliana   OG0000002
AT5G67570.1 | Tetratricopeptide repeat (TPR)-like superfamily
protein | Chr5:26952352-26955480 FORWARD LENGTH=798 | 201606
Arabidopsis_thaliana   OG0000002
AT1G30290.1 | Tetratricopeptide repeat (TPR)-like superfamily
protein | Chr1:10670320-10672740 REVERSE LENGTH=806 | 201606
Arabidopsis_thaliana   OG0000002
AT1G30610.1 | pentatricopeptide (PPR) repeat-containing protein |
Chr1:10846676-10850517 FORWARD LENGTH=1006 | 201606
Arabidopsis_thaliana   OG0000002
AT1G31840.1 | Tetratricopeptide repeat (TPR)-like superfamily
protein | Chr1:11424006-11426528 FORWARD LENGTH=840 | 201606
Arabidopsis_thaliana   OG0000002
AT1G31920.1 | Tetratricopeptide repeat (TPR)-like superfamily
protein | Chr1:11461864-11463684 REVERSE LENGTH=606 | 201606
Arabidopsis_thaliana   OG0000002
AT1G32415.1 | pentatricopeptide (PPR) repeat-containing protein |
Chr1:11695611-11697896 FORWARD LENGTH=761 | 201606
Arabidopsis_thaliana   OG0000002
AT1G33350.1 | Pentatricopeptide repeat (PPR) superfamily protein |
Chr1:12090071-12091687 REVERSE LENGTH=538 | 201606
Arabidopsis_thaliana   OG0000002
AT1G04840.1 | Tetratricopeptide repeat (TPR)-like superfamily
protein | Chr1:1362867-1364962 REVERSE LENGTH=665 | 201606
Arabidopsis_thaliana   OG0000002
AT1G50270.1 | Pentatricopeptide repeat (PPR) superfamily protein |
Chr1:18622044-18623834 FORWARD LENGTH=596 | 201606
Arabidopsis_thaliana   OG0000002
AT1G51965.1 | ABA Overly-Sensitive 5 | Chr1:19312078-19314145
REVERSE LENGTH=653 | 201606   Arabidopsis_thaliana
OG0000002
AT1G52620.1 | Pentatricopeptide repeat (PPR) superfamily protein |
Chr1:19603828-19606287 FORWARD LENGTH=819 | 201606
Arabidopsis_thaliana   OG0000002
AT1G52640.1 | Pentatricopeptide repeat (PPR) superfamily protein |
Chr1:19608857-19610428 REVERSE LENGTH=523 | 201606
Arabidopsis_thaliana   OG0000002
AT1G53330.1 | Pentatricopeptide repeat (PPR) superfamily protein |
Chr1:19896027-19897442 FORWARD LENGTH=471 | 201606
Arabidopsis_thaliana   OG0000002
AT1G55630.1 | Pentatricopeptide repeat (PPR) superfamily protein |
Chr1:20791817-20793250 REVERSE LENGTH=477 | 201606
Arabidopsis_thaliana   OG0000002
AT1G55890.1 | Tetratricopeptide repeat (TPR)-like superfamily
protein | Chr1:20901364-20902560 FORWARD LENGTH=398 | 201606
Arabidopsis_thaliana   OG0000002
AT1G05670.1 | Pentatricopeptide repeat (PPR-like) superfamily
protein | Chr1:1698574-1700799 REVERSE LENGTH=741 | 201606
Arabidopsis_thaliana   OG0000002
AT1G05750.1 | Tetratricopeptide repeat (TPR)-like superfamily

protein | Chr1:1721523-1723025 FORWARD LENGTH=500 | 201606
Arabidopsis_thaliana   OG0000002
AT1G62260.1 | mitochondrial editing factor 9 |
Chr1:22997826-22999796 REVERSE LENGTH=656 | 201606
Arabidopsis_thaliana   OG0000002
AT1G62590.1 | pentatricopeptide (PPR) repeat-containing protein |
Chr1:23177294-23179198 REVERSE LENGTH=634 | 201606
Arabidopsis_thaliana   OG0000002
AT1G62670.1 | rna processing factor 2 | Chr1:23204773-23206665
REVERSE LENGTH=630 | 201606   Arabidopsis_thaliana
OG0000002
AT1G62680.1 | Pentatricopeptide repeat (PPR) superfamily protein |
Chr1:23208247-23209893 REVERSE LENGTH=548 | 201606
Arabidopsis_thaliana   OG0000002
AT1G62720.1 | Pentatricopeptide repeat (PPR-like) superfamily
protein | Chr1:23227574-23229031 FORWARD LENGTH=485 | 201606
Arabidopsis_thaliana   OG0000002
AT1G62910.1 | Pentatricopeptide repeat (PPR) superfamily protein |
Chr1:23299060-23300958 FORWARD LENGTH=632 | 201606
Arabidopsis_thaliana   OG0000002
AT1G62914.1 | pentatricopeptide (PPR) repeat-containing protein |
Chr1:23301576-23303162 FORWARD LENGTH=528 | 201606
Arabidopsis_thaliana   OG0000002
AT1G62930.1 | Tetratricopeptide repeat (TPR)-like superfamily
protein | Chr1:23306534-23308423 FORWARD LENGTH=629 | 201606
Arabidopsis_thaliana   OG0000002
AT1G63070.1 | pentatricopeptide (PPR) repeat-containing protein |
Chr1:23385324-23387167 REVERSE LENGTH=590 | 201606
Arabidopsis_thaliana   OG0000002
AT1G63080.1 | Pentatricopeptide repeat (PPR) superfamily protein |
Chr1:23388884-23390728 REVERSE LENGTH=614 | 201606
Arabidopsis_thaliana   OG0000002
AT1G63130.1 | Tetratricopeptide repeat (TPR)-like superfamily
protein | Chr1:23412854-23414746 FORWARD LENGTH=630 | 201606
Arabidopsis_thaliana   OG0000002
AT1G63150.1 | Tetratricopeptide repeat (TPR)-like superfamily
protein | Chr1:23419399-23421288 FORWARD LENGTH=629 | 201606
Arabidopsis_thaliana   OG0000002
AT1G63230.1 | Tetratricopeptide repeat (TPR)-like superfamily
protein | Chr1:23451144-23452201 FORWARD LENGTH=323 | 201606
Arabidopsis_thaliana   OG0000002
AT1G63320.1 | Pentatricopeptide repeat (PPR) superfamily protein |
Chr1:23488884-23489530 REVERSE LENGTH=189 | 201606
Arabidopsis_thaliana   OG0000002
AT1G63330.1 | Pentatricopeptide repeat (PPR) superfamily protein |
Chr1:23489840-23491519 FORWARD LENGTH=559 | 201606
Arabidopsis_thaliana   OG0000002
AT1G63400.1 | Pentatricopeptide repeat (PPR) superfamily protein |
Chr1:23507320-23509053 FORWARD LENGTH=577 | 201606
Arabidopsis_thaliana   OG0000002
AT1G63630.1 | Tetratricopeptide repeat (TPR)-like superfamily
protein | Chr1:23587250-23588220 FORWARD LENGTH=273 | 201606
Arabidopsis_thaliana   OG0000002
AT1G64100.1 | pentatricopeptide (PPR) repeat-containing protein |

Chr1:23791585-23793641 FORWARD LENGTH=666 | 201606
Arabidopsis_thaliana OG0000002
AT1G64580.1 | Pentatricopeptide repeat (PPR) superfamily protein | Chr1:23985078-23986649 REVERSE LENGTH=523 | 201606
Arabidopsis_thaliana OG0000002
AT1G64583.1 | Tetratricopeptide repeat (TPR)-like superfamily protein | Chr1:23987202-23988740 REVERSE LENGTH=512 | 201606
Arabidopsis_thaliana OG0000002
AT1G06140.1 | Pentatricopeptide repeat (PPR) superfamily protein | Chr1:1864796-1866472 FORWARD LENGTH=558 | 201606
Arabidopsis_thaliana OG0000002
AT1G06143.1 | basic helix loop helix DNA-binding superfamily protein | Chr1:1867129-1868862 REVERSE LENGTH=577 | 201606
Arabidopsis_thaliana OG0000002
AT1G66345.1 | Pentatricopeptide repeat (PPR) superfamily protein | Chr1:24737719-24739353 FORWARD LENGTH=544 | 201606
Arabidopsis_thaliana OG0000002
AT1G69350.1 | Tetratricopeptide repeat (TPR)-like superfamily protein | Chr1:26069882-26072245 FORWARD LENGTH=787 | 201606
Arabidopsis_thaliana OG0000002
AT1G06580.1 | Pentatricopeptide repeat (PPR) superfamily protein | Chr1:2014440-2015942 REVERSE LENGTH=500 | 201606
Arabidopsis_thaliana OG0000002
AT1G06710.1 | Tetratricopeptide repeat (TPR)-like superfamily protein | Chr1:2056999-2060242 REVERSE LENGTH=997 | 201606
Arabidopsis_thaliana OG0000002
AT1G71060.1 | Tetratricopeptide repeat (TPR)-like superfamily protein | Chr1:26805651-26807183 REVERSE LENGTH=510 | 201606
Arabidopsis_thaliana OG0000002
AT1G71420.1 | Tetratricopeptide repeat (TPR)-like superfamily protein | Chr1:26917822-26920059 REVERSE LENGTH=745 | 201606
Arabidopsis_thaliana OG0000002
AT1G74400.1 | Tetratricopeptide repeat (TPR)-like superfamily protein | Chr1:27963953-27965341 FORWARD LENGTH=462 | 201606
Arabidopsis_thaliana OG0000002
AT1G74580.1 | Pentatricopeptide repeat (PPR) superfamily protein | Chr1:28020777-28023068 FORWARD LENGTH=763 | 201606
Arabidopsis_thaliana OG0000002
AT1G74630.1 | Tetratricopeptide repeat (TPR)-like superfamily protein | Chr1:28030521-28032452 FORWARD LENGTH=643 | 201606
Arabidopsis_thaliana OG0000002
AT1G74750.1 | Pentatricopeptide repeat (PPR) superfamily protein | Chr1:28086800-28089367 FORWARD LENGTH=855 | 201606
Arabidopsis_thaliana OG0000002
AT1G74850.1 | plastid transcriptionally active 2 | Chr1:28119237-28122314 REVERSE LENGTH=862 | 201606
Arabidopsis_thaliana OG0000002
AT1G74900.1 | Pentatricopeptide repeat (PPR) superfamily protein | Chr1:28133933-28135381 FORWARD LENGTH=453 | 201606
Arabidopsis_thaliana OG0000002
AT1G76280.1 | Tetratricopeptide repeat (TPR)-like superfamily protein | Chr1:28617948-28622581 REVERSE LENGTH=773 | 201606
Arabidopsis_thaliana OG0000002
AT1G77170.1 | Tetratricopeptide repeat (TPR)-like superfamily

protein | Chr1:28998133-28999536 REVERSE LENGTH=467 | 201606
Arabidopsis_thaliana   OG0000002
AT1G77340.1 | Pentatricopeptide repeat (PPR) superfamily protein |
Chr1:29068620-29069828 REVERSE LENGTH=402 | 201606
Arabidopsis_thaliana   OG0000002
AT1G77360.1 | Tetratricopeptide repeat (TPR)-like superfamily
protein | Chr1:29071983-29073536 REVERSE LENGTH=517 | 201606
Arabidopsis_thaliana   OG0000002
AT1G77405.1 | Pentatricopeptide repeat (PPR) superfamily protein |
Chr1:29087145-29088521 FORWARD LENGTH=458 | 201606
Arabidopsis_thaliana   OG0000002
AT1G79080.1 | Pentatricopeptide repeat (PPR) superfamily protein |
Chr1:29747102-29748832 REVERSE LENGTH=576 | 201606
Arabidopsis_thaliana   OG0000002
AT1G07590.1 | Tetratricopeptide repeat (TPR)-like superfamily
protein | Chr1:2336649-2338481 REVERSE LENGTH=534 | 201606
Arabidopsis_thaliana   OG0000002
AT1G79540.1 | Pentatricopeptide repeat (PPR) superfamily protein |
Chr1:29920334-29922676 REVERSE LENGTH=780 | 201606
Arabidopsis_thaliana   OG0000002
AT1G80550.1 | Pentatricopeptide repeat (PPR) superfamily protein |
Chr1:30285358-30286704 REVERSE LENGTH=448 | 201606
Arabidopsis_thaliana   OG0000002
AT1G07740.1 | Tetratricopeptide repeat (TPR)-like superfamily
protein | Chr1:2399117-2400496 REVERSE LENGTH=459 | 201606
Arabidopsis_thaliana   OG0000002
AT2G01360.1 | pentatricopeptide (PPR) repeat protein |
Chr2:167931-168479 FORWARD LENGTH=182 | 201606
Arabidopsis_thaliana   OG0000002
AT2G01390.1 | Tetratricopeptide repeat (TPR)-like superfamily
protein | Chr2:172256-174137 FORWARD LENGTH=577 | 201606
Arabidopsis_thaliana   OG0000002
AT2G01510.1 | Tetratricopeptide repeat (TPR)-like superfamily
protein | Chr2:230752-232506 REVERSE LENGTH=584 | 201606
Arabidopsis_thaliana   OG0000002
AT2G01740.1 | Tetratricopeptide repeat (TPR)-like superfamily
protein | Chr2:326136-327815 REVERSE LENGTH=559 | 201606
Arabidopsis_thaliana   OG0000002
AT2G02150.1 | Tetratricopeptide repeat (TPR)-like superfamily
protein | Chr2:550340-552625 REVERSE LENGTH=761 | 201606
Arabidopsis_thaliana   OG0000002
AT2G02750.1 | Pentatricopeptide repeat (PPR) superfamily protein |
Chr2:771641-773482 REVERSE LENGTH=613 | 201606
Arabidopsis_thaliana   OG0000002
AT2G03880.1 | Pentatricopeptide repeat (PPR) superfamily protein |
Chr2:1181560-1183452 FORWARD LENGTH=630 | 201606
Arabidopsis_thaliana   OG0000002
AT2G04860.1 | Tetratricopeptide repeat (TPR)-like superfamily
protein | Chr2:1706787-1708865 REVERSE LENGTH=692 | 201606
Arabidopsis_thaliana   OG0000002
AT1G08070.1 | Tetratricopeptide repeat (TPR)-like superfamily
protein | Chr1:2514374-2516599 REVERSE LENGTH=741 | 201606
Arabidopsis_thaliana   OG0000002
AT2G06000.1 | Pentatricopeptide repeat (PPR) superfamily protein |

Chr2:2328000-2329610 REVERSE LENGTH=536 | 201606
Arabidopsis_thaliana   OG0000002
AT2G13600.1 | Pentatricopeptide repeat (PPR) superfamily protein |
Chr2:5671493-5673586 FORWARD LENGTH=697 | 201606
Arabidopsis_thaliana   OG0000002
AT2G15630.1 | Pentatricopeptide repeat (PPR) superfamily protein |
Chr2:6814521-6816404 FORWARD LENGTH=627 | 201606
Arabidopsis_thaliana   OG0000002
AT2G15980.1 | Tetratricopeptide repeat (TPR)-like superfamily
protein | Chr2:6951349-6952845 REVERSE LENGTH=498 | 201606
Arabidopsis_thaliana   OG0000002
AT2G16880.1 | Pentatricopeptide repeat (PPR) superfamily protein |
Chr2:7312262-7314493 REVERSE LENGTH=743 | 201606
Arabidopsis_thaliana   OG0000002
AT1G08610.1 | Pentatricopeptide repeat (PPR) superfamily protein |
Chr1:2733788-2735467 REVERSE LENGTH=559 | 201606
Arabidopsis_thaliana   OG0000002
AT2G17140.1 | Pentatricopeptide repeat (PPR) superfamily protein |
Chr2:7462820-7465740 FORWARD LENGTH=874 | 201606
Arabidopsis_thaliana   OG0000002
AT2G17210.1 | Tetratricopeptide repeat (TPR)-like superfamily
protein | Chr2:7485398-7487608 REVERSE LENGTH=736 | 201606
Arabidopsis_thaliana   OG0000002
AT2G17525.1 | Pentatricopeptide repeat (PPR) superfamily protein |
Chr2:7624178-7626058 FORWARD LENGTH=626 | 201606
Arabidopsis_thaliana   OG0000002
AT2G17670.1 | Tetratricopeptide repeat (TPR)-like superfamily
protein | Chr2:7674420-7675811 FORWARD LENGTH=463 | 201606
Arabidopsis_thaliana   OG0000002
AT2G18520.1 | Tetratricopeptide repeat (TPR)-like superfamily
protein | Chr2:8034036-8035292 REVERSE LENGTH=418 | 201606
Arabidopsis_thaliana   OG0000002
AT2G18940.1 | Tetratricopeptide repeat (TPR)-like superfamily
protein | Chr2:8203873-8206341 REVERSE LENGTH=822 | 201606
Arabidopsis_thaliana   OG0000002
AT2G19280.1 | Pentatricopeptide repeat (PPR) superfamily protein |
Chr2:8362672-8364753 FORWARD LENGTH=693 | 201606
Arabidopsis_thaliana   OG0000002
AT2G21090.1 | Pentatricopeptide repeat (PPR-like) superfamily
protein | Chr2:9045695-9047488 REVERSE LENGTH=597 | 201606
Arabidopsis_thaliana   OG0000002
AT1G09190.1 | Tetratricopeptide repeat (TPR)-like superfamily
protein | Chr1:2966263-2967717 REVERSE LENGTH=484 | 201606
Arabidopsis_thaliana   OG0000002
AT2G26790.1 | Pentatricopeptide repeat (PPR) superfamily protein |
Chr2:11425270-11427669 REVERSE LENGTH=799 | 201606
Arabidopsis_thaliana   OG0000002
AT1G09680.1 | Pentatricopeptide repeat (PPR) superfamily protein |
Chr1:3134107-3135930 REVERSE LENGTH=607 | 201606
Arabidopsis_thaliana   OG0000002
AT2G28050.1 | Pentatricopeptide repeat (PPR) superfamily protein |
Chr2:11938265-11939653 REVERSE LENGTH=462 | 201606
Arabidopsis_thaliana   OG0000002
AT1G09820.1 | Pentatricopeptide repeat (PPR-like) superfamily

protein | Chr1:3190594-3192414 REVERSE LENGTH=606 | 201606
Arabidopsis_thaliana OG0000002
AT2G29760.1 | Tetratricopeptide repeat (TPR)-like superfamily protein | Chr2:12712884-12715100 FORWARD LENGTH=738 | 201606
Arabidopsis_thaliana OG0000002
AT1G09900.1 | Pentatricopeptide repeat (PPR-like) superfamily protein | Chr1:3218133-3219929 FORWARD LENGTH=598 | 201606
Arabidopsis_thaliana OG0000002
AT2G31400.1 | s uncoupled 1 | Chr2:13387201-13390550 REVERSE LENGTH=918 | 201606 Arabidopsis_thaliana OG0000002
AT2G32630.1 | Pentatricopeptide repeat (PPR-like) superfamily protein | Chr2:13844834-13846708 FORWARD LENGTH=624 | 201606
Arabidopsis_thaliana OG0000002
Os03t0271300-01 Similar to Pentatricopeptide repeat protein PPR868-14. Oryza_sativa OG0000002
Os03t0284900-01 Annexin repeat, conserved site domain containing protein. Oryza_sativa OG0000002
Os03t0309800-01 Similar to PPR2. Oryza_sativa OG0000002
Os03t0314400-01 Pentatricopeptide repeat domain containing protein. Oryza_sativa OG0000002
Os03t0336000-01 Pentatricopeptide repeat domain containing protein. Oryza_sativa OG0000002
Os03t0363700-00 Pentatricopeptide repeat domain containing protein. Oryza_sativa OG0000002
Os03t0396200-01 Hypothetical conserved gene. Oryza_sativa OG0000002
Os03t0400901-00 Hypothetical conserved gene. Oryza_sativa OG0000002
Os03t0569800-01 Similar to ATP binding protein. Oryza_sativa OG0000002
Os03t0582100-01 Pentatricopeptide repeat domain containing protein. Oryza_sativa OG0000002
Os03t0597200-01 Pentatricopeptide repeat protein, Chloroplast development Oryza_sativa OG0000002
Os01t0263400-00 Pentatricopeptide repeat domain containing protein. Oryza_sativa OG0000002
Os03t0701400-01 Similar to Pentatricopeptide repeat protein PPR868-14. Oryza_sativa OG0000002
Os03t0712900-01 Pentatricopeptide repeat domain containing protein. Oryza_sativa OG0000002
Os03t0728200-00 Pentatricopeptide repeat domain containing protein. Oryza_sativa OG0000002
Os03t0736200-00 Pentatricopeptide repeat domain containing protein. Oryza_sativa OG0000002
Os03t0746400-01 Pentatricopeptide repeat domain containing protein. Oryza_sativa OG0000002
Os03t0761300-01 Pentatricopeptide repeat domain containing protein. Oryza_sativa OG0000002
Os03t0782900-01 Pentatricopeptide repeat domain containing protein. Oryza_sativa OG0000002
Os03t0795400-00 Hypothetical conserved gene. Oryza_sativa OG0000002
Os03t0807400-01 Pentatricopeptide repeat domain containing protein. Oryza_sativa OG0000002

Os03t0824100-01 Tetratricopeptide-like helical domain containing protein. Oryza_sativa OG0000002
Os03t0844000-01 Pentatricopeptide repeat domain containing protein. Oryza_sativa OG0000002
Os03t0856100-01 Pentatricopeptide repeat domain containing protein. Oryza_sativa OG0000002
Os03t0861900-01 Pentatricopeptide repeat domain containing protein. Oryza_sativa OG0000002
Os04t0110500-02 Pentatricopeptide repeat domain containing protein. Oryza_sativa OG0000002
Os04t0221300-01 Pentatricopeptide repeat domain containing protein. Oryza_sativa OG0000002
Os01t0299150-00 Pentatricopeptide repeat domain containing protein. Oryza_sativa OG0000002
Os04t0349600-01 Pentatricopeptide repeat domain containing protein. Oryza_sativa OG0000002
Os04t0350000-01 Similar to PPR protein. Oryza_sativa OG0000002
Os04t0351333-00 Similar to OSIGBa0144C23.4 protein. Oryza_sativa OG0000002
Os04t0401200-00 Similar to OSIGBa0155K12.4 protein. Oryza_sativa OG0000002
Os04t0432800-00 Similar to H0315E07.3 protein. Oryza_sativa OG0000002
Os04t0446100-00 Similar to OSIGBa0140O07.12 protein. Oryza_sativa OG0000002
Os04t0469400-01 Pentatricopeptide repeat domain containing protein. Oryza_sativa OG0000002
Os04t0477200-01 Pentatricopeptide repeat domain containing protein. Oryza_sativa OG0000002
Os04t0488500-01 Pentatricopeptide repeat domain containing protein. Oryza_sativa OG0000002
Os04t0520900-01 Pentatricopeptide repeat domain containing protein. Oryza_sativa OG0000002
Os04t0583101-01 Pentatricopeptide repeat domain containing protein. Oryza_sativa OG0000002
Os04t0618050-01 Pentatricopeptide repeat containing protein. Oryza_sativa OG0000002
Os04t0643700-01 Pentatricopeptide repeat domain containing protein. Oryza_sativa OG0000002
Os04t0675600-01 Hypothetical conserved gene. Oryza_sativa OG0000002
Os04t0676200-01 Pentatricopeptide repeat domain containing protein. Oryza_sativa OG0000002
Os04t0684500-01 Pentatricopeptide repeat domain containing protein. Oryza_sativa OG0000002
Os01t0355100-01 Pentatricopeptide repeat domain containing protein. Oryza_sativa OG0000002
Os05t0112900-01 Pentatricopeptide repeat domain containing protein. Oryza_sativa OG0000002
Os01t0357800-01 Pentatricopeptide repeat domain containing protein. Oryza_sativa OG0000002
Os05t0132000-00 Pentatricopeptide repeat domain containing protein. Oryza_sativa OG0000002

Os05t0153400-01 Pentatricopeptide repeat domain containing protein.
Oryza_sativa OG0000002
Os05t0207200-00 Similar to rf1 protein. Oryza_sativa
OG0000002
Os05t0212100-01 Asp/Glu racemase, active site domain containing
protein. Oryza_sativa OG0000002
Os05t0275000-01 Pentatricopeptide repeat domain containing protein.
Oryza_sativa OG0000002
Os05t0275100-00 Pentatricopeptide repeat domain containing protein.
Oryza_sativa OG0000002
Os05t0294600-01 Pentatricopeptide repeat domain containing protein.
Oryza_sativa OG0000002
Os05t0307200-00 Pentatricopeptide repeat domain containing protein.
Oryza_sativa OG0000002
Os05t0313600-00 Pentatricopeptide repeat domain containing protein.
Oryza_sativa OG0000002
Os05t0315100-00 Pentatricopeptide repeat domain containing protein.
Oryza_sativa OG0000002
Os05t0355200-00 Pentatricopeptide repeat domain containing protein.
Oryza_sativa OG0000002
Os05t0393900-00 Pentatricopeptide repeat domain containing protein.
Oryza_sativa OG0000002
Os01t0505500-00 Pentatricopeptide repeat domain containing protein.
Oryza_sativa OG0000002
Os01t0506100-01 Pentatricopeptide repeat domain containing protein.
Oryza_sativa OG0000002
Os05t0481800-01 Hypothetical conserved gene. Oryza_sativa
OG0000002
Os05t0548600-00 Pentatricopeptide repeat domain containing protein.
Oryza_sativa OG0000002
Os05t0583950-00 Hypothetical conserved gene. Oryza_sativa
OG0000002
Os05t0586900-01 Hypothetical conserved gene. Oryza_sativa
OG0000002
Os06t0111300-00 Hypothetical conserved gene. Oryza_sativa
OG0000002
Os06t0112000-01 Pentatricopeptide repeat domain containing protein.
Oryza_sativa OG0000002
Os06t0114366-00 Pentatricopeptide repeat domain containing protein.
Oryza_sativa OG0000002
Os06t0125300-01 Pentatricopeptide repeat domain containing protein.
Oryza_sativa OG0000002
Os06t0125600-01 Hypothetical conserved gene. Oryza_sativa
OG0000002
Os06t0143500-00 Pentatricopeptide repeat domain containing protein.
Oryza_sativa OG0000002
Os06t0152500-00 Hypothetical conserved gene. Oryza_sativa
OG0000002
Os06t0172000-01 Pentatricopeptide repeat domain containing protein.
Oryza_sativa OG0000002
Os06t0199100-01 Helix-turn-helix, AraC type, subdomain 2 domain
containing protein. Oryza_sativa OG0000002
Os06t0216400-01 Tetratricopeptide-like helical domain containing
protein. Oryza_sativa OG0000002

Os01t0546700-00 Hypothetical conserved gene.	Oryza_sativa
OG0000002
Os06t0228900-00 Pentatricopeptide repeat domain containing protein.
Oryza_sativa	OG0000002
Os06t0231100-00 Pentatricopeptide repeat domain containing protein.
Oryza_sativa	OG0000002
Os06t0231400-01 Pentatricopeptide repeat domain containing protein.
Oryza_sativa	OG0000002
Os06t0249500-00 Pentatricopeptide repeat domain containing protein.
Oryza_sativa	OG0000002
Os06t0308200-01 Similar to 60S ribosomal protein L15.
Oryza_sativa	OG0000002
Os06t0493801-00 Hypothetical conserved gene.	Oryza_sativa
OG0000002
Os06t0499301-00 Pentatricopeptide repeat domain containing protein.
Oryza_sativa	OG0000002
Os01t0559500-01 Similar to predicted protein.	Oryza_sativa
OG0000002
Os06t0565000-00 Hypothetical conserved gene.	Oryza_sativa
OG0000002
Os06t0690900-00 Pentatricopeptide repeat domain containing protein.
Oryza_sativa	OG0000002
Os06t0694600-00 Pentatricopeptide repeat domain containing protein.
Oryza_sativa	OG0000002
Os06t0700100-01 Pentatricopeptide repeat domain containing protein.
Oryza_sativa	OG0000002
Os06t0705350-00 Similar to pentatricopeptide (PPR) repeat-containing
protein. Oryza_sativa	OG0000002
Os06t0710800-01 Pentatricopeptide repeat domain containing protein.
Oryza_sativa	OG0000002
Os07t0101200-01 Pentatricopeptide repeat domain containing protein.
Oryza_sativa	OG0000002
Os07t0163900-00 Pentatricopeptide repeat domain containing protein.
Oryza_sativa	OG0000002
Os07t0179000-01 Pentatricopeptide repeat domain containing protein.
Oryza_sativa	OG0000002
Os07t0203900-00 Pentatricopeptide repeat domain containing protein.
Oryza_sativa	OG0000002
Os07t0213300-01 Pentatricopeptide repeat domain containing protein.
Oryza_sativa	OG0000002
Os07t0239600-01 Pentatricopeptide repeat domain containing protein.
Oryza_sativa	OG0000002
Os07t0249100-01 Pentatricopeptide repeat domain containing protein.
Oryza_sativa	OG0000002
Os07t0259400-00 Pentatricopeptide repeat domain containing protein.
Oryza_sativa	OG0000002
Os07t0260000-00 Pentatricopeptide repeat domain containing protein.
Oryza_sativa	OG0000002
Os07t0300200-01 Pentatricopeptide repeat domain containing protein.
Oryza_sativa	OG0000002
Os07t0436600-00 Pentatricopeptide repeat domain containing protein.
Oryza_sativa	OG0000002
Os07t0472100-00 Hypothetical conserved gene.	Oryza_sativa
OG0000002

Os01t0607800-01 Pentatricopeptide repeat domain containing protein.
Oryza_sativa OG0000002
Os07t0491500-01 Hypothetical conserved gene. Oryza_sativa
OG0000002
Os07t0513200-01 Pentatricopeptide repeat domain containing protein.
Oryza_sativa OG0000002
Os01t0611900-01 Pentatricopeptide repeat domain containing protein.
Oryza_sativa OG0000002
Os07t0548300-01 Hypothetical conserved gene. Oryza_sativa
OG0000002
Os07t0590600-00 Tetratricopeptide-like helical domain containing
protein. Oryza_sativa OG0000002
Os07t0615000-01 Similar to PPR repeat domain containing protein.
Oryza_sativa OG0000002
Os07t0621100-00 Pentatricopeptide repeat domain containing protein.
Oryza_sativa OG0000002
Os07t0635800-00 Pentatricopeptide repeat domain containing protein.
Oryza_sativa OG0000002
Os07t0636900-01 Asp/Glu racemase, active site domain containing
protein. Oryza_sativa OG0000002
Os07t0662700-01 Pentatricopeptide repeat domain containing protein.
Oryza_sativa OG0000002
Os07t0671200-00 Pentatricopeptide repeat domain containing protein.
Oryza_sativa OG0000002
Os07t0674300-01 Protein kinase, ATP binding site domain containing
protein. Oryza_sativa OG0000002
Os07t0688100-01 Pentatricopeptide repeat domain containing protein.
Oryza_sativa OG0000002
Os08t0107700-01 Similar to Rf1 protein, mitochondrial precursor (PPR
protein) (Fertility restorer) (Restorer for CMS). Oryza_sativa
OG0000002
Os08t0107800-01 Pentatricopeptide repeat domain containing protein.
Oryza_sativa OG0000002
Os08t0110200-01 Similar to Fertility restorer. Oryza_sativa
OG0000002
Os08t0162200-01 Pentatricopeptide repeat domain containing protein.
Oryza_sativa OG0000002
Os08t0191900-01 Pentatricopeptide repeat domain containing protein.
Oryza_sativa OG0000002
Os08t0248400-01 Pentatricopeptide repeat domain containing protein.
Oryza_sativa OG0000002
Os01t0646000-01 Pentatricopeptide repeat domain containing protein.
Oryza_sativa OG0000002
Os08t0290000-01 Pentatricopeptide repeat domain containing protein.
Oryza_sativa OG0000002
Os08t0300700-01 Pentatricopeptide repeat domain containing protein.
Oryza_sativa OG0000002
Os08t0369200-01 Pentatricopeptide repeat domain containing protein.
Oryza_sativa OG0000002
Os08t0402600-01 Pentatricopeptide repeat domain containing protein.
Oryza_sativa OG0000002
Os08t0481000-01 Pentatricopeptide repeat domain containing protein.
Oryza_sativa OG0000002
Os08t0525500-01 Pentatricopeptide repeat domain containing protein.

Oryza_sativa	OG0000002
Os08t0538800-01 Pentatricopeptide repeat domain containing protein.	Oryza_sativa	OG0000002
Os09t0110200-01 Pentatricopeptide repeat domain containing protein.	Oryza_sativa	OG0000002
Os01t0672166-00 Pentatricopeptide repeat domain containing protein.	Oryza_sativa	OG0000002
Os09t0251500-01 Pentatricopeptide repeat domain containing protein.	Oryza_sativa	OG0000002
Os01t0674700-00 Similar to EMB2279 (EMBRYO DEFECTIVE 2279).	Oryza_sativa	OG0000002
Os09t0407800-01 Pentatricopeptide repeat domain containing protein.	Oryza_sativa	OG0000002
Os09t0411600-01 Pentatricopeptide repeat domain containing protein.	Oryza_sativa	OG0000002
Os09t0417500-01 Pentatricopeptide repeat domain containing protein.	Oryza_sativa	OG0000002
Os09t0423300-01 Pentatricopeptide repeat domain containing protein.	Oryza_sativa	OG0000002
Os09t0431600-00 Cystathionine beta-synthase, core domain containing protein.	Oryza_sativa	OG0000002
Os09t0474051-01 Hypothetical conserved gene.	Oryza_sativa	OG0000002
Os09t0532500-01 Similar to peptidase S8 and S53, subtilisin, kexin, sedolisin.	Oryza_sativa	OG0000002
Os09t0532800-01 Similar to peptidase S8 and S53, subtilisin, kexin, sedolisin.	Oryza_sativa	OG0000002
Os09t0542800-01 Pentatricopeptide repeat domain containing protein.	Oryza_sativa	OG0000002
Os09t0555400-01 Pentatricopeptide repeat domain containing protein.	Oryza_sativa	OG0000002
Os10t0116000-00 Pentatricopeptide repeat domain containing protein.	Oryza_sativa	OG0000002
Os10t0147250-00 Hypothetical conserved gene.	Oryza_sativa	OG0000002
Os10t0181200-01 Similar to binding protein.	Oryza_sativa	OG0000002
Os10t0358700-01 Pentatricopeptide repeat domain containing protein.	Oryza_sativa	OG0000002
Os10t0363500-00 Pentatricopeptide repeat containing protein.	Oryza_sativa	OG0000002
Os10t0368902-00 Pentatricopeptide repeat domain containing protein.	Oryza_sativa	OG0000002
Os10t0421800-01 Pentatricopeptide repeat domain containing protein.	Oryza_sativa	OG0000002
Os10t0442700-01 Similar to Protein serine/threonine kinase (Protein kinase).	Oryza_sativa	OG0000002
Os01t0715900-01 Pentatricopeptide repeat domain containing protein.	Oryza_sativa	OG0000002
Os10t0476900-01 Pentatricopeptide repeat domain containing protein.	Oryza_sativa	OG0000002
Os10t0477200-01 Pentatricopeptide repeat domain containing protein.	Oryza_sativa	OG0000002
Os10t0484300-01 Pentatricopeptide repeat domain containing protein.

Oryza_sativa	OG0000002
Os10t0484900-00 Hypothetical conserved gene.	Oryza_sativa
OG0000002
Os10t0488900-01 Pentatricopeptide repeat domain containing protein.
Oryza_sativa	OG0000002
Os10t0495100-01 Similar to Fertility restorer.	Oryza_sativa
OG0000002
Os10t0495200-01 Similar to Fertility restorer.	Oryza_sativa
OG0000002
Os10t0495400-01 Hypothetical conserved gene.	Oryza_sativa
OG0000002
Os10t0497300-01 Pentatricopeptide repeat domain containing protein.
Oryza_sativa	OG0000002
Os10t0497366-00 Similar to Fertility restorer.	Oryza_sativa
OG0000002
Os10t0497432-01 Similar to PPR protein.	Oryza_sativa
OG0000002
Os10t0499500-01 Similar to Fertility restorer.	Oryza_sativa
OG0000002
Os10t0501700-00 Similar to Protein Rf1, mitochondrial.
Oryza_sativa	OG0000002
Os10t0506000-00 Pentatricopeptide repeat containing protein.
Oryza_sativa	OG0000002
Os10t0540100-01 Pentatricopeptide repeat domain containing protein.
Oryza_sativa	OG0000002
Os10t0578500-00 Hypothetical conserved gene.	Oryza_sativa
OG0000002
Os11t0103000-00 Pentatricopeptide repeat domain containing protein.
Oryza_sativa	OG0000002
Os11t0109800-00 Pentatricopeptide repeat domain containing protein.
Oryza_sativa	OG0000002
Os11t0114800-00 Pentatricopeptide repeat domain containing protein.
Oryza_sativa	OG0000002
Os11t0131400-00 Hypothetical conserved gene.	Oryza_sativa
OG0000002
Os11t0133200-00 Hypothetical conserved gene.	Oryza_sativa
OG0000002
Os11t0213500-01 Pentatricopeptide repeat protein, Mitochondrial nad7 transcript editing, Seed development	Oryza_sativa
OG0000002
Os11t0256100-00 Pentatricopeptide repeat domain containing protein.
Oryza_sativa	OG0000002
Os11t0433600-01 Tetratricopeptide-like helical domain containing protein.	Oryza_sativa	OG0000002
Os11t0482400-01 Pentatricopeptide repeat domain containing protein.
Oryza_sativa	OG0000002
Os11t0483950-00 Pentatricopeptide repeat domain containing protein.
Oryza_sativa	OG0000002
Os11t0587100-00 Pentatricopeptide repeat domain containing protein.
Oryza_sativa	OG0000002
Os11t0607100-01 Pentatricopeptide repeat domain containing protein.
Oryza_sativa	OG0000002
Os11t0659400-00 Hypothetical conserved gene.	Oryza_sativa
OG0000002

Os11t0673000-00 Pentatricopeptide repeat domain containing protein.
Oryza_sativa OG0000002
Os11t0680200-00 Pentatricopeptide repeat domain containing protein.
Oryza_sativa OG0000002
Os12t0102600-01 Similar to HCF152. Oryza_sativa
OG0000002
Os12t0109800-00 Hypothetical conserved gene. Oryza_sativa
OG0000002
Os12t0114400-01 Pentatricopeptide repeat domain containing protein.
Oryza_sativa OG0000002
Os12t0128200-01 Pentatricopeptide repeat domain containing protein.
Oryza_sativa OG0000002
Os12t0152600-01 Pentatricopeptide repeat domain containing protein.
Oryza_sativa OG0000002
Os01t0764950-01 Pentatricopeptide repeat domain containing protein.
Oryza_sativa OG0000002
Os12t0233200-00 Hypothetical conserved gene. Oryza_sativa
OG0000002
Os12t0283900-01 Heat shock protein DnaJ, conserved site domain
containing protein. Oryza_sativa OG0000002
Os12t0289800-00 Hypothetical conserved gene. Oryza_sativa
OG0000002
Os12t0407900-01 Pentatricopeptide repeat domain containing protein.
Oryza_sativa OG0000002
Os12t0456100-00 Hypothetical conserved gene. Oryza_sativa
OG0000002
Os12t0552300-00 Hypothetical conserved gene. Oryza_sativa
OG0000002
Os12t0557800-01 Pentatricopeptide repeat domain containing protein.
Oryza_sativa OG0000002
Os12t0562300-01 Pentatricopeptide repeat domain containing protein.
Oryza_sativa OG0000002
Os12t0615600-00 Hypothetical conserved gene. Oryza_sativa
OG0000002
Os01t0783100-01 Pentatricopeptide repeat domain containing protein.
Oryza_sativa OG0000002
Os12t0638900-01 Pentatricopeptide repeat domain containing protein.
Oryza_sativa OG0000002
Os01t0785700-01 Pentatricopeptide repeat domain containing protein.
Oryza_sativa OG0000002
Os01t0800400-01 Pentatricopeptide repeat domain containing protein.
Oryza_sativa OG0000002
Os01t0814300-01 Pentatricopeptide repeat domain containing protein.
Oryza_sativa OG0000002
Os01t0816000-00 Protein prenyltransferase domain containing protein.
Oryza_sativa OG0000002
Os01t0833000-01 Pentatricopeptide repeat domain containing protein.
Oryza_sativa OG0000002
Os01t0852900-01 Pentatricopeptide repeat domain containing protein.
Oryza_sativa OG0000002
Os01t0151900-01 Pentatricopeptide repeat domain containing protein.
Oryza_sativa OG0000002
Os01t0880900-01 Similar to Pentatricopeptide repeat protein
PPR868-14. Oryza_sativa OG0000002

Os01t0884800-00 Pentatricopeptide repeat domain containing protein.
Oryza_sativa OG0000002
Os01t0153250-00 Hypothetical conserved gene. Oryza_sativa
OG0000002
Os01t0908800-01 Pentatricopeptide repeat domain containing protein.
Oryza_sativa OG0000002
Os01t0912900-01 Pentatricopeptide repeat domain containing protein.
Oryza_sativa OG0000002
Os01t0913400-01 Nucleotide-binding, alpha-beta plait domain
containing protein. Oryza_sativa OG0000002
Os01t0914600-01 Pentatricopeptide repeat domain containing protein.
Oryza_sativa OG0000002
Os01t0977400-01 Pentatricopeptide repeat domain containing protein.
Oryza_sativa OG0000002
Os02t0110400-01 Sugar transporter, conserved site domain containing
protein. Oryza_sativa OG0000002
Os02t0116100-00 Hypothetical conserved gene. Oryza_sativa
OG0000002
Os02t0119700-01 Pentatricopeptide repeat domain containing protein.
Oryza_sativa OG0000002
Os02t0121900-01 Pentatricopeptide repeat domain containing protein.
Oryza_sativa OG0000002
Os02t0126500-01 Pentatricopeptide repeat domain containing protein.
Oryza_sativa OG0000002
Os02t0132100-00 Pentatricopeptide repeat domain containing protein.
Oryza_sativa OG0000002
Os02t0164600-01 Pentatricopeptide repeat domain containing protein.
Oryza_sativa OG0000002
Os02t0170000-01 Conserved hypothetical protein. Oryza_sativa
OG0000002
Os01t0170800-01 Pentatricopeptide repeat domain containing protein.
Oryza_sativa OG0000002
Os02t0191200-00 Pentatricopeptide repeat domain containing protein.
Oryza_sativa OG0000002
Os02t0226900-01 Pentatricopeptide repeat domain containing protein.
Oryza_sativa OG0000002
Os01t0176300-00 Pentatricopeptide repeat domain containing protein.
Oryza_sativa OG0000002
Os02t0266200-01 Pentatricopeptide repeat domain containing protein.
Oryza_sativa OG0000002
Os02t0304800-01 Hypothetical conserved gene. Oryza_sativa
OG0000002
Os02t0321000-01 Pentatricopeptide repeat domain containing protein.
Oryza_sativa OG0000002
Os02t0468500-00 Pentatricopeptide repeat domain containing protein.
Oryza_sativa OG0000002
Os02t0468800-01 Pentatricopeptide repeat domain containing protein.
Oryza_sativa OG0000002
Os02t0470000-01 Pentatricopeptide repeat domain containing protein.
Oryza_sativa OG0000002
Os02t0529900-01 Pentatricopeptide repeat domain containing protein.
Oryza_sativa OG0000002
Os02t0552100-01 Pentatricopeptide repeat domain containing protein.
Oryza_sativa OG0000002

Os02t0555100-01 Pentatricopeptide repeat domain containing protein.
Oryza_sativa OG0000002
Os02t0565400-01 Pentatricopeptide repeat domain containing protein.
Oryza_sativa OG0000002
Os02t0582300-01 Pentatricopeptide repeat domain containing protein.
Oryza_sativa OG0000002
Os02t0601600-01 Hypothetical conserved gene. Oryza_sativa
OG0000002
Os02t0625100-00 Hypothetical conserved gene. Oryza_sativa
OG0000002
Os02t0633200-01 Pentatricopeptide repeat domain containing protein.
Oryza_sativa OG0000002
Os01t0197450-00 Pentatricopeptide repeat domain containing protein.
Oryza_sativa OG0000002
Os02t0644600-01 Pentatricopeptide repeat domain containing protein.
Oryza_sativa OG0000002
Os02t0670700-01 Pentatricopeptide repeat domain containing protein.
Oryza_sativa OG0000002
Os02t0679200-01 Pentatricopeptide repeat domain containing protein.
Oryza_sativa OG0000002
Os02t0680500-01 Pentatricopeptide repeat domain containing protein.
Oryza_sativa OG0000002
Os02t0730900-00 Pentatricopeptide repeat domain containing protein.
Oryza_sativa OG0000002
Os02t0735800-01 Pentatricopeptide repeat domain containing protein.
Oryza_sativa OG0000002
Os02t0769900-01 Pentatricopeptide repeat domain containing protein.
Oryza_sativa OG0000002
Os02t0793200-01 Pentatricopeptide repeat domain containing protein.
Oryza_sativa OG0000002
Os02t0818800-01 Pentatricopeptide repeat domain containing protein.
Oryza_sativa OG0000002
Os02t0824000-00 Hypothetical conserved gene. Oryza_sativa
OG0000002
Os02t0829800-01 Pentatricopeptide repeat domain containing protein.
Oryza_sativa OG0000002
Os02t0830000-01 Pentatricopeptide repeat domain containing protein.
Oryza_sativa OG0000002
Os02t0833050-00 Pentatricopeptide repeat domain containing protein.
Oryza_sativa OG0000002
Os03t0115300-00 Pentatricopeptide repeat domain containing protein.
Oryza_sativa OG0000002
Os03t0119700-01 Pentatricopeptide repeat domain containing protein.
Oryza_sativa OG0000002
Os03t0162900-01 Pentatricopeptide repeat domain containing protein.
Oryza_sativa OG0000002
Os03t0165100-00 Pentatricopeptide repeat domain containing protein.
Oryza_sativa OG0000002
Os03t0169300-01 Pentatricopeptide repeat domain containing protein.
Oryza_sativa OG0000002
Os03t0185200-01 Pentatricopeptide repeat domain containing protein.
Oryza_sativa OG0000002
Os01t0228400-01 Pentatricopeptide repeat domain containing protein.
Oryza_sativa OG0000002

Os03t0201400-01 Pentatricopeptide repeat domain containing protein. Oryza_sativa OG0000002
Os03t0208600-01 Pentatricopeptide repeat domain containing protein. Oryza_sativa OG0000002
Os03t0211600-01 Pentatricopeptide repeat domain containing protein. Oryza_sativa OG0000002
Os03t0215900-00 Similar to 67kD chloroplastic RNA-binding protein, P67. Oryza_sativa OG0000002
Os03t0216300-01 Pentatricopeptide repeat domain containing protein. Oryza_sativa OG0000002
Os03t0216400-01 Pentatricopeptide repeat domain containing protein. Oryza_sativa OG0000002
Os03t0225300-01 Pentatricopeptide repeat domain containing protein. Oryza_sativa OG0000002
CMR044CT pep chromosome:ASM9120v1:18:121466:123685:1 gene:CMR044C transcript:CMR044CT gene_biotype:protein_coding transcript_biotype:protein_coding Cyanidioschyzon_merolae OG0000002
CMN222CT pep chromosome:ASM9120v1:14:541695:544391:1 gene:CMN222C transcript:CMN222CT gene_biotype:protein_coding transcript_biotype:protein_coding Cyanidioschyzon_merolae OG0000002
CMQ136CT pep chromosome:ASM9120v1:17:319974:322391:1 gene:CMQ136C transcript:CMQ136CT gene_biotype:protein_coding transcript_biotype:protein_coding Cyanidioschyzon_merolae OG0000002
CML043CT pep chromosome:ASM9120v1:12:91933:93993:1 gene:CML043C transcript:CML043CT gene_biotype:protein_coding transcript_biotype:protein_coding Cyanidioschyzon_merolae OG0000002
evm.model.contig_2095.6 Porphyridium_purpureum OG0000002
evm.model.contig_2343.9 Porphyridium_purpureum OG0000002
evm.model.contig_2350.5 Porphyridium_purpureum OG0000002
evm.model.contig_2421.9 Porphyridium_purpureum OG0000002
evm.model.contig_3420.3 Porphyridium_purpureum OG0000002
evm.model.contig_2033.25 Porphyridium_purpureum OG0000002
evm.model.contig_3543.3 Porphyridium_purpureum OG0000002
evm.model.contig_4456.11 Porphyridium_purpureum OG0000002
evm.model.contig_454.4 Porphyridium_purpureum OG0000002
evm.model.contig_4574.1 Porphyridium_purpureum OG0000002
evm.model.contig_510.2 Porphyridium_purpureum OG0000002
evm.model.contig_2059.20 Porphyridium_purpureum OG0000002
CDF32402 pep supercontig:ASM35022v2:HG001489:84237:86309:1 gene:CHC_T00008166001 transcript:CDF32402 gene_biotype:protein_coding transcript_biotype:protein_coding Chondrus_crispus OG0000002
CDF40327 pep supercontig:ASM35022v2:HG002154:352195:353835:-1 gene:CHC_T00007142001 transcript:CDF40327 gene_biotype:protein_coding transcript_biotype:protein_coding Chondrus_crispus OG0000002
CDF39354 pep supercontig:ASM35022v2:HG002023:14577:17115:-1 gene:CHC_T00000207001 transcript:CDF39354 gene_biotype:protein_coding transcript_biotype:protein_coding Chondrus_crispus OG0000002

CDF32813 pep supercontig:ASM35022v2:HG001592:55954:56616:1
gene:CHC_T00001427001 transcript:CDF32813
gene_biotype:protein_coding transcript_biotype:protein_coding
Chondrus_crispus OG0000002
CDF33629 pep supercontig:ASM35022v2:HG001651:199749:202319:-1
gene:CHC_T00002161001 transcript:CDF33629
gene_biotype:protein_coding transcript_biotype:protein_coding
Chondrus_crispus OG0000002
CDF33985 pep supercontig:ASM35022v2:HG001664:509570:510157:1
gene:CHC_T00002486001 transcript:CDF33985
gene_biotype:protein_coding transcript_biotype:protein_coding
Chondrus_crispus OG0000002
CDF40721 pep supercontig:ASM35022v2:HG002236:50850:53439:1
gene:CHC_T00007367001 transcript:CDF40721
gene_biotype:protein_coding transcript_biotype:protein_coding
Chondrus_crispus OG0000002
CDF77436 pep supercontig:ASM35022v2:HG001523:392637:394748:1
gene:CHC_T00007940001 transcript:CDF77436
gene_biotype:protein_coding transcript_biotype:protein_coding
Chondrus_crispus OG0000002
CDF35933 pep supercontig:ASM35022v2:HG001752:101707:104664:-1
gene:CHC_T00004314001 transcript:CDF35933
gene_biotype:protein_coding transcript_biotype:protein_coding
Chondrus_crispus OG0000002
CDF40902 pep supercontig:ASM35022v2:HG002266:4884:6904:-1
gene:CHC_T00000889001 transcript:CDF40902
gene_biotype:protein_coding transcript_biotype:protein_coding
Chondrus_crispus OG0000002
g7247.t1 Caulerpa_lentillifera OG0000003
Cre03.g183950.t1.2 pacid=30786839 transcript=Cre03.g183950.t1.2
locus=Cre03.g183950 ID=Cre03.g183950.t1.2.v5.5 annot-version=v5.5
Chlamydomonas_reinhardtii OG0000003
Cre03.g186800.t1.2 pacid=30787772 transcript=Cre03.g186800.t1.2
locus=Cre03.g186800 ID=Cre03.g186800.t1.2.v5.5 annot-version=v5.5
Chlamydomonas_reinhardtii OG0000003
Cre01.g045850.t1.1 pacid=30788778 transcript=Cre01.g045850.t1.1
locus=Cre01.g045850 ID=Cre01.g045850.t1.1.v5.5 annot-version=v5.5
Chlamydomonas_reinhardtii OG0000003
Cre01.g030968.t1.1 pacid=30789028 transcript=Cre01.g030968.t1.1
locus=Cre01.g030968 ID=Cre01.g030968.t1.1.v5.5 annot-version=v5.5
Chlamydomonas_reinhardtii OG0000003
Cre16.g673450.t1.1 pacid=30777482 transcript=Cre16.g673450.t1.1
locus=Cre16.g673450 ID=Cre16.g673450.t1.1.v5.5 annot-version=v5.5
Chlamydomonas_reinhardtii OG0000003
Vocar.0002s0673.1.p pacid=32890887 transcript=Vocar.0002s0673.1
locus=Vocar.0002s0673 ID=Vocar.0002s0673.1.v2.1 annot-version=v2.1
Volvox_carteri OG0000003
Vocar.0080s0004.1.p pacid=32891574 transcript=Vocar.0080s0004.1
locus=Vocar.0080s0004 ID=Vocar.0080s0004.1.v2.1 annot-version=v2.1
Volvox_carteri OG0000003
Vocar.0006s0199.1.p pacid=32894146 transcript=Vocar.0006s0199.1
locus=Vocar.0006s0199 ID=Vocar.0006s0199.1.v2.1 annot-version=v2.1
Volvox_carteri OG0000003
jgi|ChlNC64A_1|51454|fgenesh3_pg.C_scaffold_7000001

Chlorella_variabilis	OG0000003
jgi|ChlNC64A_1|141398|IGS.gm_34_00002	Chlorella_variabilis
OG0000003
jgi|ChlNC64A_1|142672|IGS.gm_5_00001	Chlorella_variabilis
OG0000003
jgi|ChlNC64A_1|142681|IGS.gm_5_00010	Chlorella_variabilis
OG0000003
jgi|ChlNC64A_1|142682|IGS.gm_5_00011	Chlorella_variabilis
OG0000003
jgi|ChlNC64A_1|141811|IGS.gm_38_00074	Chlorella_variabilis
OG0000003
jgi|ChlNC64A_1|134142|IGS.gm_100_00001	Chlorella_variabilis
OG0000003
jgi|ChlNC64A_1|141485|IGS.gm_342_00001	Chlorella_variabilis
OG0000003
jgi|ChlNC64A_1|144003|IGS.gm_621_00001	Chlorella_variabilis
OG0000003
jgi|ChlNC64A_1|144034|IGS.gm_664_00001	Chlorella_variabilis
OG0000003
jgi|ChlNC64A_1|139651|IGS.gm_26_00004	Chlorella_variabilis
OG0000003
68334 pacid=27392733 transcript=68334
locus=estExt_Genemark1.C_240104 ID=68334.2.0.227 annot-version=v2.0
Coccomyxa_subellipsoidea	OG0000003
22523_e_gw1.19.00.25.1	Ostreococcus_tauri	OG0000003
5129_gw1.19.00.26.1	Ostreococcus_tauri	OG0000003
29883_0000010004 Ostreococcus_tauri	OG0000003
29974_0000790001 Ostreococcus_tauri	OG0000003
5901_gw1.08.00.302.1	Ostreococcus_tauri	OG0000003
31077_0200010408 Ostreococcus_tauri	OG0000003
28449 pacid=27412885 transcript=28449 locus=eugene.1800010032
ID=28449.2.0.231 annot-version=v2.0	Ostreococcus_lucimarinus
OG0000003
165878 pacid=27343681 transcript=165918 locus=e_gw1.19.15.1
ID=165918.3.0.228 annot-version=v3.0	Micromonas_pusilla
OG0000003
129490 pacid=27343708 transcript=129530 locus=gw1.19.14.1
ID=129530.3.0.228 annot-version=v3.0	Micromonas_pusilla
OG0000003
54877 pacid=27343738 transcript=54877 locus=MicpuC2.EuGene.
0000190057 ID=54877.3.0.228 annot-version=v3.0
Micromonas_pusilla	OG0000003
124486 pacid=27343745 transcript=124526 locus=fgenesh2_kg.
19_#_47_#_4276671:16 ID=124526.3.0.228 annot-version=v3.0
Micromonas_pusilla	OG0000003
49347 pacid=27343782 transcript=49347
locus=MicpuC2.estExt_fgenesh1_pg.C_190011 ID=49347.3.0.228 annot-
version=v3.0	Micromonas_pusilla	OG0000003
124463 pacid=27343783 transcript=124503 locus=fgenesh2_kg.
19_#_24_#_4276671:1 ID=124503.3.0.228 annot-version=v3.0
Micromonas_pusilla	OG0000003
kfl00382_0010_v1.1	Klebsormidium_flaccidum	OG0000003
kfl00382_0020_v1.1	Klebsormidium_flaccidum	OG0000003
kfl00392_0010_v1.1	Klebsormidium_flaccidum	OG0000003

kfl00392_g2_v1.1 Klebsormidium_flaccidum	OG0000003
kfl00392_g3_v1.1 Klebsormidium_flaccidum	OG0000003
kfl00418_0080_v1.1	Klebsormidium_flaccidum	OG0000003
kfl00016_0510_v1.1	Klebsormidium_flaccidum	OG0000003
kfl00429_0030_v1.1	Klebsormidium_flaccidum	OG0000003
kfl00434_0110_v1.1	Klebsormidium_flaccidum	OG0000003
kfl00434_0120_v1.1	Klebsormidium_flaccidum	OG0000003
kfl00445_0130_v1.1	Klebsormidium_flaccidum	OG0000003
kfl00458_0070_v1.1	Klebsormidium_flaccidum	OG0000003
kfl00461_0010_v1.1	Klebsormidium_flaccidum	OG0000003
kfl00494_0020_v1.1	Klebsormidium_flaccidum	OG0000003
kfl00494_0120_v1.1	Klebsormidium_flaccidum	OG0000003
kfl00535_0110_v1.1	Klebsormidium_flaccidum	OG0000003
kfl00557_g2_v1.1 Klebsormidium_flaccidum	OG0000003
kfl00566_g1_v1.1 Klebsormidium_flaccidum	OG0000003
kfl00566_0010_v1.1	Klebsormidium_flaccidum	OG0000003
kfl00570_0080_v1.1	Klebsormidium_flaccidum	OG0000003
kfl00591_g14_v1.1	Klebsormidium_flaccidum	OG0000003
kfl00593_0100_v1.1	Klebsormidium_flaccidum	OG0000003
kfl00640_0010_v1.1	Klebsormidium_flaccidum	OG0000003
kfl00660_0030_v1.1	Klebsormidium_flaccidum	OG0000003
kfl00687_0010_v1.1	Klebsormidium_flaccidum	OG0000003
kfl00687_g2_v1.1 Klebsormidium_flaccidum	OG0000003
kfl00725_0060_v1.1	Klebsormidium_flaccidum	OG0000003
kfl00763_0010_v1.1	Klebsormidium_flaccidum	OG0000003
kfl00763_0020_v1.1	Klebsormidium_flaccidum	OG0000003
kfl00763_0030_v1.1	Klebsormidium_flaccidum	OG0000003
kfl00763_0035_v1.1	Klebsormidium_flaccidum	OG0000003
kfl00841_g1_v1.1 Klebsormidium_flaccidum	OG0000003
kfl00872_0040_v1.1	Klebsormidium_flaccidum	OG0000003
kfl00891_0010_v1.1	Klebsormidium_flaccidum	OG0000003
kfl00891_0020_v1.1	Klebsormidium_flaccidum	OG0000003
kfl00891_g3_v1.1 Klebsormidium_flaccidum	OG0000003
kfl00891_g4_v1.1 Klebsormidium_flaccidum	OG0000003
kfl00915_0010_v1.1	Klebsormidium_flaccidum	OG0000003
kfl00915_0020_v1.1	Klebsormidium_flaccidum	OG0000003
kfl00918_0040_v1.1	Klebsormidium_flaccidum	OG0000003
kfl00934_0020_v1.1	Klebsormidium_flaccidum	OG0000003
kfl00937_0010_v1.1	Klebsormidium_flaccidum	OG0000003
kfl00937_0020_v1.1	Klebsormidium_flaccidum	OG0000003
kfl00937_g3_v1.1 Klebsormidium_flaccidum	OG0000003
kfl00938_0020_v1.1	Klebsormidium_flaccidum	OG0000003
kfl00938_0030_v1.1	Klebsormidium_flaccidum	OG0000003
kfl00961_0010_v1.1	Klebsormidium_flaccidum	OG0000003
kfl00961_0015_v1.1	Klebsormidium_flaccidum	OG0000003
kfl00961_0020_v1.1	Klebsormidium_flaccidum	OG0000003
kfl00961_0030_v1.1	Klebsormidium_flaccidum	OG0000003
kfl00961_0040_v1.1	Klebsormidium_flaccidum	OG0000003
kfl00980_0030_v1.1	Klebsormidium_flaccidum	OG0000003
kfl00994_0030_v1.1	Klebsormidium_flaccidum	OG0000003
kfl00994_0035_v1.1	Klebsormidium_flaccidum	OG0000003
kfl01002_0010_v1.1	Klebsormidium_flaccidum	OG0000003
kfl01011_0020_v1.1	Klebsormidium_flaccidum	OG0000003
kfl01011_0030_v1.1	Klebsormidium_flaccidum	OG0000003

kfl01011_0040_v1.1	Klebsormidium_flaccidum	OG0000003
kfl01011_0060_v1.1	Klebsormidium_flaccidum	OG0000003
kfl01014_0020_v1.1	Klebsormidium_flaccidum	OG0000003
kfl01026_0010_v1.1	Klebsormidium_flaccidum	OG0000003
kfl01026_g2_v1.1 Klebsormidium_flaccidum	OG0000003
kfl01029_0020_v1.1	Klebsormidium_flaccidum	OG0000003
kfl01029_0030_v1.1	Klebsormidium_flaccidum	OG0000003
kfl01030_0010_v1.1	Klebsormidium_flaccidum	OG0000003
kfl01030_0020_v1.1	Klebsormidium_flaccidum	OG0000003
kfl01030_g4_v1.1 Klebsormidium_flaccidum	OG0000003
kfl01042_0010_v1.1	Klebsormidium_flaccidum	OG0000003
kfl01042_0020_v1.1	Klebsormidium_flaccidum	OG0000003
kfl01042_g3_v1.1 Klebsormidium_flaccidum	OG0000003
kfl01042_0030_v1.1	Klebsormidium_flaccidum	OG0000003
kfl01042_0035_v1.1	Klebsormidium_flaccidum	OG0000003
kfl01070_0010_v1.1	Klebsormidium_flaccidum	OG0000003
kfl01070_0040_v1.1	Klebsormidium_flaccidum	OG0000003
kfl01090_0010_v1.1	Klebsormidium_flaccidum	OG0000003
kfl01090_0020_v1.1	Klebsormidium_flaccidum	OG0000003
kfl01090_0030_v1.1	Klebsormidium_flaccidum	OG0000003
kfl01090_0050_v1.1	Klebsormidium_flaccidum	OG0000003
kfl01090_0060_v1.1	Klebsormidium_flaccidum	OG0000003
kfl01100_0010_v1.1	Klebsormidium_flaccidum	OG0000003
kfl01101_0020_v1.1	Klebsormidium_flaccidum	OG0000003
kfl01105_0010_v1.1	Klebsormidium_flaccidum	OG0000003
kfl01111_0020_v1.1	Klebsormidium_flaccidum	OG0000003
kfl01122_g1_v1.1 Klebsormidium_flaccidum	OG0000003
kfl01122_0010_v1.1	Klebsormidium_flaccidum	OG0000003
kfl01122_0020_v1.1	Klebsormidium_flaccidum	OG0000003
kfl01122_0025_v1.1	Klebsormidium_flaccidum	OG0000003
kfl01122_0030_v1.1	Klebsormidium_flaccidum	OG0000003
kfl01122_g6_v1.1 Klebsormidium_flaccidum	OG0000003
kfl01153_0010_v1.1	Klebsormidium_flaccidum	OG0000003
kfl01153_g2_v1.1 Klebsormidium_flaccidum	OG0000003
kfl01153_0030_v1.1	Klebsormidium_flaccidum	OG0000003
kfl01162_0020_v1.1	Klebsormidium_flaccidum	OG0000003
kfl01162_0030_v1.1	Klebsormidium_flaccidum	OG0000003
kfl01162_0040_v1.1	Klebsormidium_flaccidum	OG0000003
kfl01162_0050_v1.1	Klebsormidium_flaccidum	OG0000003
kfl01169_0020_v1.1	Klebsormidium_flaccidum	OG0000003
kfl01191_g1_v1.1 Klebsormidium_flaccidum	OG0000003
kfl01191_0020_v1.1	Klebsormidium_flaccidum	OG0000003
kfl01198_0020_v1.1	Klebsormidium_flaccidum	OG0000003
kfl01205_0010_v1.1	Klebsormidium_flaccidum	OG0000003
kfl01205_0020_v1.1	Klebsormidium_flaccidum	OG0000003
kfl01205_0030_v1.1	Klebsormidium_flaccidum	OG0000003
kfl01214_0010_v1.1	Klebsormidium_flaccidum	OG0000003
kfl01214_0020_v1.1	Klebsormidium_flaccidum	OG0000003
kfl01214_0030_v1.1	Klebsormidium_flaccidum	OG0000003
kfl01216_0010_v1.1	Klebsormidium_flaccidum	OG0000003
kfl01220_0010_v1.1	Klebsormidium_flaccidum	OG0000003
kfl01236_0010_v1.1	Klebsormidium_flaccidum	OG0000003
kfl01236_0020_v1.1	Klebsormidium_flaccidum	OG0000003
kfl01236_0030_v1.1	Klebsormidium_flaccidum	OG0000003

```
kfl01240_0010_v1.1	Klebsormidium_flaccidum	OG0000003
kfl01255_0020_v1.1	Klebsormidium_flaccidum	OG0000003
kfl01255_0030_v1.1	Klebsormidium_flaccidum	OG0000003
kfl01255_0040_v1.1	Klebsormidium_flaccidum	OG0000003
kfl01260_0010_v1.1	Klebsormidium_flaccidum	OG0000003
kfl01261_0010_v1.1	Klebsormidium_flaccidum	OG0000003
kfl01262_0015_v1.1	Klebsormidium_flaccidum	OG0000003
kfl01263_0010_v1.1	Klebsormidium_flaccidum	OG0000003
kfl01263_0030_v1.1	Klebsormidium_flaccidum	OG0000003
kfl01299_0010_v1.1	Klebsormidium_flaccidum	OG0000003
kfl01299_0020_v1.1	Klebsormidium_flaccidum	OG0000003
kfl01302_0010_v1.1	Klebsormidium_flaccidum	OG0000003
kfl01308_0030_v1.1	Klebsormidium_flaccidum	OG0000003
kfl01308_0040_v1.1	Klebsormidium_flaccidum	OG0000003
kfl01316_0010_v1.1	Klebsormidium_flaccidum	OG0000003
kfl01316_0020_v1.1	Klebsormidium_flaccidum	OG0000003
kfl01316_0030_v1.1	Klebsormidium_flaccidum	OG0000003
kfl01319_0020_v1.1	Klebsormidium_flaccidum	OG0000003
kfl01327_0010_v1.1	Klebsormidium_flaccidum	OG0000003
kfl01327_0020_v1.1	Klebsormidium_flaccidum	OG0000003
kfl01329_0010_v1.1	Klebsormidium_flaccidum	OG0000003
kfl01355_0010_v1.1	Klebsormidium_flaccidum	OG0000003
kfl01355_0020_v1.1	Klebsormidium_flaccidum	OG0000003
kfl01359_0010_v1.1	Klebsormidium_flaccidum	OG0000003
kfl01359_g2_v1.1 Klebsormidium_flaccidum	OG0000003
kfl01360_0010_v1.1	Klebsormidium_flaccidum	OG0000003
kfl01360_0020_v1.1	Klebsormidium_flaccidum	OG0000003
kfl01360_0025_v1.1	Klebsormidium_flaccidum	OG0000003
kfl01363_0010_v1.1	Klebsormidium_flaccidum	OG0000003
kfl01368_0010_v1.1	Klebsormidium_flaccidum	OG0000003
kfl01368_0020_v1.1	Klebsormidium_flaccidum	OG0000003
kfl01371_0010_v1.1	Klebsormidium_flaccidum	OG0000003
kfl01371_g2_v1.1 Klebsormidium_flaccidum	OG0000003
kfl01379_0010_v1.1	Klebsormidium_flaccidum	OG0000003
kfl01379_0020_v1.1	Klebsormidium_flaccidum	OG0000003
kfl01382_0010_v1.1	Klebsormidium_flaccidum	OG0000003
kfl01382_0020_v1.1	Klebsormidium_flaccidum	OG0000003
kfl01382_0025_v1.1	Klebsormidium_flaccidum	OG0000003
kfl01387_0020_v1.1	Klebsormidium_flaccidum	OG0000003
kfl01391_0010_v1.1	Klebsormidium_flaccidum	OG0000003
kfl01391_0020_v1.1	Klebsormidium_flaccidum	OG0000003
kfl01409_0010_v1.1	Klebsormidium_flaccidum	OG0000003
kfl01411_0010_v1.1	Klebsormidium_flaccidum	OG0000003
kfl01431_0010_v1.1	Klebsormidium_flaccidum	OG0000003
kfl01435_0010_v1.1	Klebsormidium_flaccidum	OG0000003
kfl01442_0010_v1.1	Klebsormidium_flaccidum	OG0000003
kfl01447_g1_v1.1 Klebsormidium_flaccidum	OG0000003
kfl01460_0010_v1.1	Klebsormidium_flaccidum	OG0000003
kfl01460_0020_v1.1	Klebsormidium_flaccidum	OG0000003
kfl01460_0030_v1.1	Klebsormidium_flaccidum	OG0000003
kfl01464_0010_v1.1	Klebsormidium_flaccidum	OG0000003
kfl01465_0010_v1.1	Klebsormidium_flaccidum	OG0000003
kfl01465_0020_v1.1	Klebsormidium_flaccidum	OG0000003
kfl01477_0010_v1.1	Klebsormidium_flaccidum	OG0000003
```

kfl01487_g1_v1.1 Klebsormidium_flaccidum	OG0000003
kfl01501_0010_v1.1	Klebsormidium_flaccidum	OG0000003
kfl01501_0020_v1.1	Klebsormidium_flaccidum	OG0000003
kfl01502_0010_v1.1	Klebsormidium_flaccidum	OG0000003
kfl01504_0020_v1.1	Klebsormidium_flaccidum	OG0000003
kfl01505_0010_v1.1	Klebsormidium_flaccidum	OG0000003
kfl01510_0010_v1.1	Klebsormidium_flaccidum	OG0000003
kfl01510_0020_v1.1	Klebsormidium_flaccidum	OG0000003
kfl01513_0020_v1.1	Klebsormidium_flaccidum	OG0000003
kfl01518_0010_v1.1	Klebsormidium_flaccidum	OG0000003
kfl01530_0010_v1.1	Klebsormidium_flaccidum	OG0000003
kfl01530_0015_v1.1	Klebsormidium_flaccidum	OG0000003
kfl01536_0015_v1.1	Klebsormidium_flaccidum	OG0000003
kfl01536_0020_v1.1	Klebsormidium_flaccidum	OG0000003
kfl01549_0010_v1.1	Klebsormidium_flaccidum	OG0000003
kfl01549_0020_v1.1	Klebsormidium_flaccidum	OG0000003
kfl01559_0020_v1.1	Klebsormidium_flaccidum	OG0000003
kfl01560_g1_v1.1 Klebsormidium_flaccidum	OG0000003
kfl01570_0010_v1.1	Klebsormidium_flaccidum	OG0000003
kfl01571_g1_v1.1 Klebsormidium_flaccidum	OG0000003
kfl01584_0020_v1.1	Klebsormidium_flaccidum	OG0000003
kfl01601_g1_v1.1 Klebsormidium_flaccidum	OG0000003
kfl01606_0010_v1.1	Klebsormidium_flaccidum	OG0000003
kfl01620_0010_v1.1	Klebsormidium_flaccidum	OG0000003
kfl01633_0010_v1.1	Klebsormidium_flaccidum	OG0000003
kfl01633_0020_v1.1	Klebsormidium_flaccidum	OG0000003
kfl01635_0010_v1.1	Klebsormidium_flaccidum	OG0000003
kfl01637_0010_v1.1	Klebsormidium_flaccidum	OG0000003
kfl01639_0020_v1.1	Klebsormidium_flaccidum	OG0000003
kfl01639_0030_v1.1	Klebsormidium_flaccidum	OG0000003
kfl01649_0010_v1.1	Klebsormidium_flaccidum	OG0000003
kfl01651_0010_v1.1	Klebsormidium_flaccidum	OG0000003
kfl01651_0020_v1.1	Klebsormidium_flaccidum	OG0000003
kfl01651_g3_v1.1 Klebsormidium_flaccidum	OG0000003
kfl01651_0030_v1.1	Klebsormidium_flaccidum	OG0000003
kfl01662_0010_v1.1	Klebsormidium_flaccidum	OG0000003
kfl01673_0010_v1.1	Klebsormidium_flaccidum	OG0000003
kfl01677_0010_v1.1	Klebsormidium_flaccidum	OG0000003
kfl01682_0010_v1.1	Klebsormidium_flaccidum	OG0000003
kfl01682_0015_v1.1	Klebsormidium_flaccidum	OG0000003
kfl01694_0010_v1.1	Klebsormidium_flaccidum	OG0000003
kfl01698_0010_v1.1	Klebsormidium_flaccidum	OG0000003
kfl01698_0020_v1.1	Klebsormidium_flaccidum	OG0000003
kfl01702_0010_v1.1	Klebsormidium_flaccidum	OG0000003
kfl01702_0020_v1.1	Klebsormidium_flaccidum	OG0000003
kfl01710_0010_v1.1	Klebsormidium_flaccidum	OG0000003
kfl01730_0010_v1.1	Klebsormidium_flaccidum	OG0000003
kfl01746_0010_v1.1	Klebsormidium_flaccidum	OG0000003
kfl01753_0010_v1.1	Klebsormidium_flaccidum	OG0000003
kfl01779_0010_v1.1	Klebsormidium_flaccidum	OG0000003
kfl01780_0010_v1.1	Klebsormidium_flaccidum	OG0000003
kfl01789_g1_v1.1 Klebsormidium_flaccidum	OG0000003
kfl01790_g1_v1.1 Klebsormidium_flaccidum	OG0000003
kfl01790_0010_v1.1	Klebsormidium_flaccidum	OG0000003

kfl01795_g1_v1.1 Klebsormidium_flaccidum OG0000003
kfl01802_0010_v1.1 Klebsormidium_flaccidum OG0000003
kfl00042_0010_v1.1 Klebsormidium_flaccidum OG0000003
kfl00048_0010_v1.1 Klebsormidium_flaccidum OG0000003
kfl00048_0020_v1.1 Klebsormidium_flaccidum OG0000003
kfl00048_0025_v1.1 Klebsormidium_flaccidum OG0000003
kfl00006_0950_v1.1 Klebsormidium_flaccidum OG0000003
kfl00157_0010_v1.1 Klebsormidium_flaccidum OG0000003
kfl00187_0010_v1.1 Klebsormidium_flaccidum OG0000003
kfl00187_0020_v1.1 Klebsormidium_flaccidum OG0000003
kfl00198_0010_v1.1 Klebsormidium_flaccidum OG0000003
kfl00198_0030_v1.1 Klebsormidium_flaccidum OG0000003
kfl00205_0200_v1.1 Klebsormidium_flaccidum OG0000003
kfl00205_0210_v1.1 Klebsormidium_flaccidum OG0000003
kfl00239_0190_v1.1 Klebsormidium_flaccidum OG0000003
Pp3c10_10296V3.1.p pacid=32899603 transcript=Pp3c10_10296V3.1 locus=Pp3c10_10296 annot-version=v3.3 Physcomitrella_patens OG0000003
Pp3c7_23370V3.1.p pacid=32926117 transcript=Pp3c7_23370V3.1 locus=Pp3c7_23370 annot-version=v3.3 Physcomitrella_patens OG0000003
Pp3c7_2330V3.1.p pacid=32926208 transcript=Pp3c7_2330V3.1 locus=Pp3c7_2330 annot-version=v3.3 Physcomitrella_patens OG0000003
Pp3c7_17660V3.1.p pacid=32926209 transcript=Pp3c7_17660V3.1 locus=Pp3c7_17660 annot-version=v3.3 Physcomitrella_patens OG0000003
Pp3c15_8430V3.1.p pacid=32926513 transcript=Pp3c15_8430V3.1 locus=Pp3c15_8430 annot-version=v3.3 Physcomitrella_patens OG0000003
Pp3c15_13080V3.1.p pacid=32926545 transcript=Pp3c15_13080V3.1 locus=Pp3c15_13080 annot-version=v3.3 Physcomitrella_patens OG0000003
Pp3c15_1610V3.1.p pacid=32927287 transcript=Pp3c15_1610V3.1 locus=Pp3c15_1610 annot-version=v3.3 Physcomitrella_patens OG0000003
Pp3c15_22580V3.1.p pacid=32927502 transcript=Pp3c15_22580V3.1 locus=Pp3c15_22580 annot-version=v3.3 Physcomitrella_patens OG0000003
Pp3c10_13830V3.1.p pacid=32902279 transcript=Pp3c10_13830V3.1 locus=Pp3c10_13830 annot-version=v3.3 Physcomitrella_patens OG0000003
Pp3c15_880V3.1.p pacid=32927744 transcript=Pp3c15_880V3.1 locus=Pp3c15_880 annot-version=v3.3 Physcomitrella_patens OG0000003
Pp3c15_13630V3.1.p pacid=32927793 transcript=Pp3c15_13630V3.1 locus=Pp3c15_13630 annot-version=v3.3 Physcomitrella_patens OG0000003
Pp3c15_20450V3.1.p pacid=32928000 transcript=Pp3c15_20450V3.1 locus=Pp3c15_20450 annot-version=v3.3 Physcomitrella_patens OG0000003
Pp3c15_10090V3.1.p pacid=32928043 transcript=Pp3c15_10090V3.1 locus=Pp3c15_10090 annot-version=v3.3 Physcomitrella_patens OG0000003

Pp3c15_7350V3.1.p pacid=32928101 transcript=Pp3c15_7350V3.1
locus=Pp3c15_7350 annot-version=v3.3 Physcomitrella_patens
OG0000003
Pp3c15_20720V3.1.p pacid=32928239 transcript=Pp3c15_20720V3.1
locus=Pp3c15_20720 annot-version=v3.3 Physcomitrella_patens
OG0000003
Pp3c15_4580V3.1.p pacid=32928396 transcript=Pp3c15_4580V3.1
locus=Pp3c15_4580 annot-version=v3.3 Physcomitrella_patens
OG0000003
Pp3c15_8400V3.1.p pacid=32928867 transcript=Pp3c15_8400V3.1
locus=Pp3c15_8400 annot-version=v3.3 Physcomitrella_patens
OG0000003
Pp3c15_21320V3.1.p pacid=32928879 transcript=Pp3c15_21320V3.1
locus=Pp3c15_21320 annot-version=v3.3 Physcomitrella_patens
OG0000003
Pp3c10_25900V3.1.p pacid=32902433 transcript=Pp3c10_25900V3.1
locus=Pp3c10_25900 annot-version=v3.3 Physcomitrella_patens
OG0000003
Pp3c15_7950V3.1.p pacid=32929508 transcript=Pp3c15_7950V3.1
locus=Pp3c15_7950 annot-version=v3.3 Physcomitrella_patens
OG0000003
Pp3c15_860V3.1.p pacid=32929621 transcript=Pp3c15_860V3.1
locus=Pp3c15_860 annot-version=v3.3 Physcomitrella_patens
OG0000003
Pp3c13_24320V3.1.p pacid=32930115 transcript=Pp3c13_24320V3.1
locus=Pp3c13_24320 annot-version=v3.3 Physcomitrella_patens
OG0000003
Pp3c13_150V3.1.p pacid=32930430 transcript=Pp3c13_150V3.1
locus=Pp3c13_150 annot-version=v3.3 Physcomitrella_patens
OG0000003
Pp3c13_9200V3.1.p pacid=32930538 transcript=Pp3c13_9200V3.1
locus=Pp3c13_9200 annot-version=v3.3 Physcomitrella_patens
OG0000003
Pp3c13_1920V3.1.p pacid=32930644 transcript=Pp3c13_1920V3.1
locus=Pp3c13_1920 annot-version=v3.3 Physcomitrella_patens
OG0000003
Pp3c13_210V3.1.p pacid=32930787 transcript=Pp3c13_210V3.1
locus=Pp3c13_210 annot-version=v3.3 Physcomitrella_patens
OG0000003
Pp3c13_14360V3.1.p pacid=32930815 transcript=Pp3c13_14360V3.1
locus=Pp3c13_14360 annot-version=v3.3 Physcomitrella_patens
OG0000003
Pp3c13_14630V3.1.p pacid=32931329 transcript=Pp3c13_14630V3.1
locus=Pp3c13_14630 annot-version=v3.3 Physcomitrella_patens
OG0000003
Pp3c13_22330V3.1.p pacid=32931396 transcript=Pp3c13_22330V3.1
locus=Pp3c13_22330 annot-version=v3.3 Physcomitrella_patens
OG0000003
Pp3c13_8170V3.1.p pacid=32931448 transcript=Pp3c13_8170V3.1
locus=Pp3c13_8170 annot-version=v3.3 Physcomitrella_patens
OG0000003
Pp3c13_200V3.1.p pacid=32931496 transcript=Pp3c13_200V3.1
locus=Pp3c13_200 annot-version=v3.3 Physcomitrella_patens
OG0000003

Pp3c10_7170V3.1.p pacid=32902713 transcript=Pp3c10_7170V3.1 locus=Pp3c10_7170 annot-version=v3.3 Physcomitrella_patens OG0000003
Pp3c13_7730V3.1.p pacid=32931761 transcript=Pp3c13_7730V3.1 locus=Pp3c13_7730 annot-version=v3.3 Physcomitrella_patens OG0000003
Pp3c13_3860V3.1.p pacid=32932409 transcript=Pp3c13_3860V3.1 locus=Pp3c13_3860 annot-version=v3.3 Physcomitrella_patens OG0000003
Pp3c13_21660V3.1.p pacid=32932451 transcript=Pp3c13_21660V3.1 locus=Pp3c13_21660 annot-version=v3.3 Physcomitrella_patens OG0000003
Pp3c2_35880V3.1.p pacid=32932795 transcript=Pp3c2_35880V3.1 locus=Pp3c2_35880 annot-version=v3.3 Physcomitrella_patens OG0000003
Pp3c2_13210V3.1.p pacid=32932888 transcript=Pp3c2_13210V3.1 locus=Pp3c2_13210 annot-version=v3.3 Physcomitrella_patens OG0000003
Pp3c2_31360V3.1.p pacid=32932895 transcript=Pp3c2_31360V3.1 locus=Pp3c2_31360 annot-version=v3.3 Physcomitrella_patens OG0000003
Pp3c2_13470V3.1.p pacid=32933101 transcript=Pp3c2_13470V3.1 locus=Pp3c2_13470 annot-version=v3.3 Physcomitrella_patens OG0000003
Pp3c2_13220V3.1.p pacid=32933449 transcript=Pp3c2_13220V3.1 locus=Pp3c2_13220 annot-version=v3.3 Physcomitrella_patens OG0000003
Pp3c2_32870V3.1.p pacid=32933722 transcript=Pp3c2_32870V3.1 locus=Pp3c2_32870 annot-version=v3.3 Physcomitrella_patens OG0000003
Pp3c2_37320V3.1.p pacid=32934031 transcript=Pp3c2_37320V3.1 locus=Pp3c2_37320 annot-version=v3.3 Physcomitrella_patens OG0000003
Pp3c2_8510V3.1.p pacid=32934216 transcript=Pp3c2_8510V3.1 locus=Pp3c2_8510 annot-version=v3.3 Physcomitrella_patens OG0000003
Pp3c2_17160V3.1.p pacid=32934544 transcript=Pp3c2_17160V3.1 locus=Pp3c2_17160 annot-version=v3.3 Physcomitrella_patens OG0000003
Pp3c2_17430V3.1.p pacid=32934566 transcript=Pp3c2_17430V3.1 locus=Pp3c2_17430 annot-version=v3.3 Physcomitrella_patens OG0000003
Pp3c2_25700V3.1.p pacid=32934870 transcript=Pp3c2_25700V3.1 locus=Pp3c2_25700 annot-version=v3.3 Physcomitrella_patens OG0000003
Pp3c2_34180V3.1.p pacid=32935041 transcript=Pp3c2_34180V3.1 locus=Pp3c2_34180 annot-version=v3.3 Physcomitrella_patens OG0000003
Pp3c2_5240V3.1.p pacid=32935108 transcript=Pp3c2_5240V3.1 locus=Pp3c2_5240 annot-version=v3.3 Physcomitrella_patens OG0000003
Pp3c2_27690V3.1.p pacid=32935231 transcript=Pp3c2_27690V3.1 locus=Pp3c2_27690 annot-version=v3.3 Physcomitrella_patens OG0000003

Pp3c2_13230V3.1.p pacid=32935793 transcript=Pp3c2_13230V3.1 locus=Pp3c2_13230 annot-version=v3.3 Physcomitrella_patens OG0000003
Pp3c2_37480V3.1.p pacid=32935902 transcript=Pp3c2_37480V3.1 locus=Pp3c2_37480 annot-version=v3.3 Physcomitrella_patens OG0000003
Pp3c2_13360V3.1.p pacid=32936117 transcript=Pp3c2_13360V3.1 locus=Pp3c2_13360 annot-version=v3.3 Physcomitrella_patens OG0000003
Pp3c2_30550V3.1.p pacid=32936536 transcript=Pp3c2_30550V3.1 locus=Pp3c2_30550 annot-version=v3.3 Physcomitrella_patens OG0000003
Pp3c2_29870V3.1.p pacid=32937441 transcript=Pp3c2_29870V3.1 locus=Pp3c2_29870 annot-version=v3.3 Physcomitrella_patens OG0000003
Pp3c2_10300V3.1.p pacid=32937550 transcript=Pp3c2_10300V3.1 locus=Pp3c2_10300 annot-version=v3.3 Physcomitrella_patens OG0000003
Pp3c2_11600V3.1.p pacid=32937634 transcript=Pp3c2_11600V3.1 locus=Pp3c2_11600 annot-version=v3.3 Physcomitrella_patens OG0000003
Pp3c19_10880V3.1.p pacid=32937740 transcript=Pp3c19_10880V3.1 locus=Pp3c19_10880 annot-version=v3.3 Physcomitrella_patens OG0000003
Pp3c19_11040V3.1.p pacid=32937906 transcript=Pp3c19_11040V3.1 locus=Pp3c19_11040 annot-version=v3.3 Physcomitrella_patens OG0000003
Pp3c19_2690V3.1.p pacid=32938562 transcript=Pp3c19_2690V3.1 locus=Pp3c19_2690 annot-version=v3.3 Physcomitrella_patens OG0000003
Pp3c19_1340V3.1.p pacid=32939008 transcript=Pp3c19_1340V3.1 locus=Pp3c19_1340 annot-version=v3.3 Physcomitrella_patens OG0000003
Pp3c19_13140V3.1.p pacid=32939956 transcript=Pp3c19_13140V3.1 locus=Pp3c19_13140 annot-version=v3.3 Physcomitrella_patens OG0000003
Pp3c19_10910V3.1.p pacid=32939977 transcript=Pp3c19_10910V3.1 locus=Pp3c19_10910 annot-version=v3.3 Physcomitrella_patens OG0000003
Pp3c22_5160V3.1.p pacid=32903600 transcript=Pp3c22_5160V3.1 locus=Pp3c22_5160 annot-version=v3.3 Physcomitrella_patens OG0000003
Pp3c3_29090V3.1.p pacid=32940604 transcript=Pp3c3_29090V3.1 locus=Pp3c3_29090 annot-version=v3.3 Physcomitrella_patens OG0000003
Pp3c3_12360V3.1.p pacid=32940674 transcript=Pp3c3_12360V3.1 locus=Pp3c3_12360 annot-version=v3.3 Physcomitrella_patens OG0000003
Pp3c3_10810V3.1.p pacid=32940732 transcript=Pp3c3_10810V3.1 locus=Pp3c3_10810 annot-version=v3.3 Physcomitrella_patens OG0000003
Pp3c3_18110V3.1.p pacid=32940784 transcript=Pp3c3_18110V3.1 locus=Pp3c3_18110 annot-version=v3.3 Physcomitrella_patens OG0000003

Pp3c3_1710V3.1.p pacid=32940915 transcript=Pp3c3_1710V3.1 locus=Pp3c3_1710 annot-version=v3.3 Physcomitrella_patens OG0000003
Pp3c3_10480V3.1.p pacid=32941174 transcript=Pp3c3_10480V3.1 locus=Pp3c3_10480 annot-version=v3.3 Physcomitrella_patens OG0000003
Pp3c3_24160V3.1.p pacid=32941198 transcript=Pp3c3_24160V3.1 locus=Pp3c3_24160 annot-version=v3.3 Physcomitrella_patens OG0000003
Pp3c3_18210V3.1.p pacid=32941556 transcript=Pp3c3_18210V3.1 locus=Pp3c3_18210 annot-version=v3.3 Physcomitrella_patens OG0000003
Pp3c3_30790V3.1.p pacid=32941903 transcript=Pp3c3_30790V3.1 locus=Pp3c3_30790 annot-version=v3.3 Physcomitrella_patens OG0000003
Pp3c3_16120V3.1.p pacid=32941939 transcript=Pp3c3_16120V3.1 locus=Pp3c3_16120 annot-version=v3.3 Physcomitrella_patens OG0000003
Pp3c3_24330V3.1.p pacid=32942073 transcript=Pp3c3_24330V3.1 locus=Pp3c3_24330 annot-version=v3.3 Physcomitrella_patens OG0000003
Pp3c3_32070V3.1.p pacid=32942215 transcript=Pp3c3_32070V3.1 locus=Pp3c3_32070 annot-version=v3.3 Physcomitrella_patens OG0000003
Pp3c3_10120V3.1.p pacid=32942244 transcript=Pp3c3_10120V3.1 locus=Pp3c3_10120 annot-version=v3.3 Physcomitrella_patens OG0000003
Pp3c3_24140V3.1.p pacid=32942639 transcript=Pp3c3_24140V3.1 locus=Pp3c3_24140 annot-version=v3.3 Physcomitrella_patens OG0000003
Pp3c3_36040V3.1.p pacid=32942811 transcript=Pp3c3_36040V3.1 locus=Pp3c3_36040 annot-version=v3.3 Physcomitrella_patens OG0000003
Pp3c3_11880V3.1.p pacid=32942823 transcript=Pp3c3_11880V3.1 locus=Pp3c3_11880 annot-version=v3.3 Physcomitrella_patens OG0000003
Pp3c3_2970V3.1.p pacid=32943147 transcript=Pp3c3_2970V3.1 locus=Pp3c3_2970 annot-version=v3.3 Physcomitrella_patens OG0000003
Pp3c3_33120V3.1.p pacid=32943391 transcript=Pp3c3_33120V3.1 locus=Pp3c3_33120 annot-version=v3.3 Physcomitrella_patens OG0000003
Pp3c20_3410V3.1.p pacid=32945318 transcript=Pp3c20_3410V3.1 locus=Pp3c20_3410 annot-version=v3.3 Physcomitrella_patens OG0000003
Pp3c20_550V3.1.p pacid=32945432 transcript=Pp3c20_550V3.1 locus=Pp3c20_550 annot-version=v3.3 Physcomitrella_patens OG0000003
Pp3c20_6760V3.1.p pacid=32945524 transcript=Pp3c20_6760V3.1 locus=Pp3c20_6760 annot-version=v3.3 Physcomitrella_patens OG0000003
Pp3c20_2910V3.1.p pacid=32946010 transcript=Pp3c20_2910V3.1 locus=Pp3c20_2910 annot-version=v3.3 Physcomitrella_patens OG0000003

Pp3c20_3370V3.1.p pacid=32946114 transcript=Pp3c20_3370V3.1 locus=Pp3c20_3370 annot-version=v3.3 Physcomitrella_patens OG0000003
Pp3c20_4370V3.1.p pacid=32946267 transcript=Pp3c20_4370V3.1 locus=Pp3c20_4370 annot-version=v3.3 Physcomitrella_patens OG0000003
Pp3c20_9430V3.1.p pacid=32946821 transcript=Pp3c20_9430V3.1 locus=Pp3c20_9430 annot-version=v3.3 Physcomitrella_patens OG0000003
Pp3c20_19290V3.1.p pacid=32946825 transcript=Pp3c20_19290V3.1 locus=Pp3c20_19290 annot-version=v3.3 Physcomitrella_patens OG0000003
Pp3c20_13200V3.1.p pacid=32947216 transcript=Pp3c20_13200V3.1 locus=Pp3c20_13200 annot-version=v3.3 Physcomitrella_patens OG0000003
Pp3c20_13450V3.1.p pacid=32947383 transcript=Pp3c20_13450V3.1 locus=Pp3c20_13450 annot-version=v3.3 Physcomitrella_patens OG0000003
Pp3c20_13440V3.1.p pacid=32947457 transcript=Pp3c20_13440V3.1 locus=Pp3c20_13440 annot-version=v3.3 Physcomitrella_patens OG0000003
Pp3c10_4830V3.1.p pacid=32899874 transcript=Pp3c10_4830V3.1 locus=Pp3c10_4830 annot-version=v3.3 Physcomitrella_patens OG0000003
Pp3c23_1320V3.1.p pacid=32948617 transcript=Pp3c23_1320V3.1 locus=Pp3c23_1320 annot-version=v3.3 Physcomitrella_patens OG0000003
Pp3c23_8160V3.1.p pacid=32948683 transcript=Pp3c23_8160V3.1 locus=Pp3c23_8160 annot-version=v3.3 Physcomitrella_patens OG0000003
Pp3c23_13740V3.1.p pacid=32948974 transcript=Pp3c23_13740V3.1 locus=Pp3c23_13740 annot-version=v3.3 Physcomitrella_patens OG0000003
Pp3c23_16480V3.1.p pacid=32949440 transcript=Pp3c23_16480V3.1 locus=Pp3c23_16480 annot-version=v3.3 Physcomitrella_patens OG0000003
Pp3c23_1620V3.1.p pacid=32949467 transcript=Pp3c23_1620V3.1 locus=Pp3c23_1620 annot-version=v3.3 Physcomitrella_patens OG0000003
Pp3c23_22280V3.1.p pacid=32949489 transcript=Pp3c23_22280V3.1 locus=Pp3c23_22280 annot-version=v3.3 Physcomitrella_patens OG0000003
Pp3c23_8930V3.1.p pacid=32949515 transcript=Pp3c23_8930V3.1 locus=Pp3c23_8930 annot-version=v3.3 Physcomitrella_patens OG0000003
Pp3c23_17680V3.1.p pacid=32949783 transcript=Pp3c23_17680V3.1 locus=Pp3c23_17680 annot-version=v3.3 Physcomitrella_patens OG0000003
Pp3c22_4240V3.1.p pacid=32904586 transcript=Pp3c22_4240V3.1 locus=Pp3c22_4240 annot-version=v3.3 Physcomitrella_patens OG0000003
Pp3c23_13580V3.1.p pacid=32950573 transcript=Pp3c23_13580V3.1 locus=Pp3c23_13580 annot-version=v3.3 Physcomitrella_patens OG0000003

Pp3c23_1350V3.1.p pacid=32950776 transcript=Pp3c23_1350V3.1 locus=Pp3c23_1350 annot-version=v3.3 Physcomitrella_patens OG0000003
Pp3c23_12170V3.1.p pacid=32950790 transcript=Pp3c23_12170V3.1 locus=Pp3c23_12170 annot-version=v3.3 Physcomitrella_patens OG0000003
Pp3c22_17610V3.1.p pacid=32904642 transcript=Pp3c22_17610V3.1 locus=Pp3c22_17610 annot-version=v3.3 Physcomitrella_patens OG0000003
Pp3c23_12080V3.1.p pacid=32951243 transcript=Pp3c23_12080V3.1 locus=Pp3c23_12080 annot-version=v3.3 Physcomitrella_patens OG0000003
Pp3c23_7880V3.1.p pacid=32951264 transcript=Pp3c23_7880V3.1 locus=Pp3c23_7880 annot-version=v3.3 Physcomitrella_patens OG0000003
Pp3c27_2220V3.1.p pacid=32951629 transcript=Pp3c27_2220V3.1 locus=Pp3c27_2220 annot-version=v3.3 Physcomitrella_patens OG0000003
Pp3c27_1230V3.1.p pacid=32952221 transcript=Pp3c27_1230V3.1 locus=Pp3c27_1230 annot-version=v3.3 Physcomitrella_patens OG0000003
Pp3c5_16880V3.1.p pacid=32952496 transcript=Pp3c5_16880V3.1 locus=Pp3c5_16880 annot-version=v3.3 Physcomitrella_patens OG0000003
Pp3c5_8600V3.1.p pacid=32952731 transcript=Pp3c5_8600V3.1 locus=Pp3c5_8600 annot-version=v3.3 Physcomitrella_patens OG0000003
Pp3c5_17950V3.1.p pacid=32953128 transcript=Pp3c5_17950V3.1 locus=Pp3c5_17950 annot-version=v3.3 Physcomitrella_patens OG0000003
Pp3c5_2320V3.1.p pacid=32953193 transcript=Pp3c5_2320V3.1 locus=Pp3c5_2320 annot-version=v3.3 Physcomitrella_patens OG0000003
Pp3c5_26530V3.1.p pacid=32953300 transcript=Pp3c5_26530V3.1 locus=Pp3c5_26530 annot-version=v3.3 Physcomitrella_patens OG0000003
Pp3c5_21060V3.1.p pacid=32953325 transcript=Pp3c5_21060V3.1 locus=Pp3c5_21060 annot-version=v3.3 Physcomitrella_patens OG0000003
Pp3c5_27270V3.1.p pacid=32953782 transcript=Pp3c5_27270V3.1 locus=Pp3c5_27270 annot-version=v3.3 Physcomitrella_patens OG0000003
Pp3c5_26460V3.1.p pacid=32953831 transcript=Pp3c5_26460V3.1 locus=Pp3c5_26460 annot-version=v3.3 Physcomitrella_patens OG0000003
Pp3c5_21350V3.1.p pacid=32954152 transcript=Pp3c5_21350V3.1 locus=Pp3c5_21350 annot-version=v3.3 Physcomitrella_patens OG0000003
Pp3c5_8260V3.1.p pacid=32954415 transcript=Pp3c5_8260V3.1 locus=Pp3c5_8260 annot-version=v3.3 Physcomitrella_patens OG0000003
Pp3c5_15080V3.1.p pacid=32954722 transcript=Pp3c5_15080V3.1 locus=Pp3c5_15080 annot-version=v3.3 Physcomitrella_patens OG0000003

Pp3c22_1640V3.1.p pacid=32905074 transcript=Pp3c22_1640V3.1 locus=Pp3c22_1640 annot-version=v3.3 Physcomitrella_patens OG0000003
Pp3c5_24700V3.1.p pacid=32954866 transcript=Pp3c5_24700V3.1 locus=Pp3c5_24700 annot-version=v3.3 Physcomitrella_patens OG0000003
Pp3c5_12890V3.1.p pacid=32955139 transcript=Pp3c5_12890V3.1 locus=Pp3c5_12890 annot-version=v3.3 Physcomitrella_patens OG0000003
Pp3c5_15560V3.1.p pacid=32955289 transcript=Pp3c5_15560V3.1 locus=Pp3c5_15560 annot-version=v3.3 Physcomitrella_patens OG0000003
Pp3c22_19380V3.1.p pacid=32905117 transcript=Pp3c22_19380V3.1 locus=Pp3c22_19380 annot-version=v3.3 Physcomitrella_patens OG0000003
Pp3c5_17440V3.1.p pacid=32955587 transcript=Pp3c5_17440V3.1 locus=Pp3c5_17440 annot-version=v3.3 Physcomitrella_patens OG0000003
Pp3c5_22520V3.1.p pacid=32955614 transcript=Pp3c5_22520V3.1 locus=Pp3c5_22520 annot-version=v3.3 Physcomitrella_patens OG0000003
Pp3c11_10980V3.1.p pacid=32956349 transcript=Pp3c11_10980V3.1 locus=Pp3c11_10980 annot-version=v3.3 Physcomitrella_patens OG0000003
Pp3c11_4460V3.1.p pacid=32956355 transcript=Pp3c11_4460V3.1 locus=Pp3c11_4460 annot-version=v3.3 Physcomitrella_patens OG0000003
Pp3c11_3600V3.1.p pacid=32956405 transcript=Pp3c11_3600V3.1 locus=Pp3c11_3600 annot-version=v3.3 Physcomitrella_patens OG0000003
Pp3c11_13320V3.1.p pacid=32956771 transcript=Pp3c11_13320V3.1 locus=Pp3c11_13320 annot-version=v3.3 Physcomitrella_patens OG0000003
Pp3c11_9370V3.1.p pacid=32957190 transcript=Pp3c11_9370V3.1 locus=Pp3c11_9370 annot-version=v3.3 Physcomitrella_patens OG0000003
Pp3c11_9050V3.1.p pacid=32957692 transcript=Pp3c11_9050V3.1 locus=Pp3c11_9050 annot-version=v3.3 Physcomitrella_patens OG0000003
Pp3c11_9140V3.1.p pacid=32957876 transcript=Pp3c11_9140V3.1 locus=Pp3c11_9140 annot-version=v3.3 Physcomitrella_patens OG0000003
Pp3c11_12670V3.1.p pacid=32957898 transcript=Pp3c11_12670V3.1 locus=Pp3c11_12670 annot-version=v3.3 Physcomitrella_patens OG0000003
Pp3c11_24550V3.1.p pacid=32957946 transcript=Pp3c11_24550V3.1 locus=Pp3c11_24550 annot-version=v3.3 Physcomitrella_patens OG0000003
Pp3c11_4720V3.1.p pacid=32958062 transcript=Pp3c11_4720V3.1 locus=Pp3c11_4720 annot-version=v3.3 Physcomitrella_patens OG0000003
Pp3c11_26070V3.1.p pacid=32959010 transcript=Pp3c11_26070V3.1 locus=Pp3c11_26070 annot-version=v3.3 Physcomitrella_patens OG0000003

Pp3c11_4730V3.1.p pacid=32959090 transcript=Pp3c11_4730V3.1 locus=Pp3c11_4730 annot-version=v3.3 Physcomitrella_patens OG0000003
Pp3c11_1220V3.1.p pacid=32959303 transcript=Pp3c11_1220V3.1 locus=Pp3c11_1220 annot-version=v3.3 Physcomitrella_patens OG0000003
Pp3c14_8840V3.1.p pacid=32959994 transcript=Pp3c14_8840V3.1 locus=Pp3c14_8840 annot-version=v3.3 Physcomitrella_patens OG0000003
Pp3c14_22220V3.1.p pacid=32960487 transcript=Pp3c14_22220V3.1 locus=Pp3c14_22220 annot-version=v3.3 Physcomitrella_patens OG0000003
Pp3c14_22620V3.1.p pacid=32960713 transcript=Pp3c14_22620V3.1 locus=Pp3c14_22620 annot-version=v3.3 Physcomitrella_patens OG0000003
Pp3c14_6702V3.1.p pacid=32961010 transcript=Pp3c14_6702V3.1 locus=Pp3c14_6702 annot-version=v3.3 Physcomitrella_patens OG0000003
Pp3c14_10030V3.1.p pacid=32961087 transcript=Pp3c14_10030V3.1 locus=Pp3c14_10030 annot-version=v3.3 Physcomitrella_patens OG0000003
Pp3c14_23500V3.1.p pacid=32961142 transcript=Pp3c14_23500V3.1 locus=Pp3c14_23500 annot-version=v3.3 Physcomitrella_patens OG0000003
Pp3c14_12819V3.1.p pacid=32961272 transcript=Pp3c14_12819V3.1 locus=Pp3c14_12819 annot-version=v3.3 Physcomitrella_patens OG0000003
Pp3c17_1440V3.1.p pacid=32905720 transcript=Pp3c17_1440V3.1 locus=Pp3c17_1440 annot-version=v3.3 Physcomitrella_patens OG0000003
Pp3c14_980V3.1.p pacid=32962014 transcript=Pp3c14_980V3.1 locus=Pp3c14_980 annot-version=v3.3 Physcomitrella_patens OG0000003
Pp3c14_990V3.1.p pacid=32962387 transcript=Pp3c14_990V3.1 locus=Pp3c14_990 annot-version=v3.3 Physcomitrella_patens OG0000003
Pp3c14_19920V3.1.p pacid=32962418 transcript=Pp3c14_19920V3.1 locus=Pp3c14_19920 annot-version=v3.3 Physcomitrella_patens OG0000003
Pp3c14_10000V3.1.p pacid=32962935 transcript=Pp3c14_10000V3.1 locus=Pp3c14_10000 annot-version=v3.3 Physcomitrella_patens OG0000003
Pp3c14_22010V3.1.p pacid=32963090 transcript=Pp3c14_22010V3.1 locus=Pp3c14_22010 annot-version=v3.3 Physcomitrella_patens OG0000003
Pp3s228_13V3.1.p pacid=32963322 transcript=Pp3s228_13V3.1 locus=Pp3s228_13 annot-version=v3.3 Physcomitrella_patens OG0000003
Pp3c8_1390V3.1.p pacid=32963860 transcript=Pp3c8_1390V3.1 locus=Pp3c8_1390 annot-version=v3.3 Physcomitrella_patens OG0000003
Pp3c8_17820V3.1.p pacid=32964160 transcript=Pp3c8_17820V3.1 locus=Pp3c8_17820 annot-version=v3.3 Physcomitrella_patens OG0000003

Pp3c8_8640V3.1.p pacid=32964933 transcript=Pp3c8_8640V3.1
locus=Pp3c8_8640 annot-version=v3.3 Physcomitrella_patens
OG0000003
Pp3c8_9221V3.1.p pacid=32965807 transcript=Pp3c8_9221V3.1
locus=Pp3c8_9221 annot-version=v3.3 Physcomitrella_patens
OG0000003
Pp3c8_19680V3.1.p pacid=32965828 transcript=Pp3c8_19680V3.1
locus=Pp3c8_19680 annot-version=v3.3 Physcomitrella_patens
OG0000003
Pp3c8_20130V3.1.p pacid=32965882 transcript=Pp3c8_20130V3.1
locus=Pp3c8_20130 annot-version=v3.3 Physcomitrella_patens
OG0000003
Pp3c8_9410V3.1.p pacid=32966071 transcript=Pp3c8_9410V3.1
locus=Pp3c8_9410 annot-version=v3.3 Physcomitrella_patens
OG0000003
Pp3c8_15150V3.1.p pacid=32966475 transcript=Pp3c8_15150V3.1
locus=Pp3c8_15150 annot-version=v3.3 Physcomitrella_patens
OG0000003
Pp3c17_23320V3.1.p pacid=32906201 transcript=Pp3c17_23320V3.1
locus=Pp3c17_23320 annot-version=v3.3 Physcomitrella_patens
OG0000003
Pp3c1_3840V3.1.p pacid=32966712 transcript=Pp3c1_3840V3.1
locus=Pp3c1_3840 annot-version=v3.3 Physcomitrella_patens
OG0000003
Pp3c1_34280V3.1.p pacid=32966760 transcript=Pp3c1_34280V3.1
locus=Pp3c1_34280 annot-version=v3.3 Physcomitrella_patens
OG0000003
Pp3c1_10940V3.1.p pacid=32966807 transcript=Pp3c1_10940V3.1
locus=Pp3c1_10940 annot-version=v3.3 Physcomitrella_patens
OG0000003
Pp3c1_9010V3.1.p pacid=32966989 transcript=Pp3c1_9010V3.1
locus=Pp3c1_9010 annot-version=v3.3 Physcomitrella_patens
OG0000003
Pp3c1_24910V3.1.p pacid=32967236 transcript=Pp3c1_24910V3.1
locus=Pp3c1_24910 annot-version=v3.3 Physcomitrella_patens
OG0000003
Pp3c1_13760V3.1.p pacid=32967372 transcript=Pp3c1_13760V3.1
locus=Pp3c1_13760 annot-version=v3.3 Physcomitrella_patens
OG0000003
Pp3c1_3600V3.1.p pacid=32968027 transcript=Pp3c1_3600V3.1
locus=Pp3c1_3600 annot-version=v3.3 Physcomitrella_patens
OG0000003
Pp3c1_21690V3.1.p pacid=32968190 transcript=Pp3c1_21690V3.1
locus=Pp3c1_21690 annot-version=v3.3 Physcomitrella_patens
OG0000003
Pp3c1_30050V3.1.p pacid=32968325 transcript=Pp3c1_30050V3.1
locus=Pp3c1_30050 annot-version=v3.3 Physcomitrella_patens
OG0000003
Pp3c1_38590V3.1.p pacid=32968399 transcript=Pp3c1_38590V3.1
locus=Pp3c1_38590 annot-version=v3.3 Physcomitrella_patens
OG0000003
Pp3c1_25590V3.1.p pacid=32969329 transcript=Pp3c1_25590V3.1
locus=Pp3c1_25590 annot-version=v3.3 Physcomitrella_patens
OG0000003

Pp3c1_34910V3.1.p pacid=32969391 transcript=Pp3c1_34910V3.1 locus=Pp3c1_34910 annot-version=v3.3	Physcomitrella_patens OG0000003
Pp3c1_29930V3.1.p pacid=32970091 transcript=Pp3c1_29930V3.1 locus=Pp3c1_29930 annot-version=v3.3	Physcomitrella_patens OG0000003
Pp3c17_5300V3.1.p pacid=32906633 transcript=Pp3c17_5300V3.1 locus=Pp3c17_5300 annot-version=v3.3	Physcomitrella_patens OG0000003
Pp3c17_19610V3.1.p pacid=32906649 transcript=Pp3c17_19610V3.1 locus=Pp3c17_19610 annot-version=v3.3	Physcomitrella_patens OG0000003
Pp3c1_34270V3.1.p pacid=32971183 transcript=Pp3c1_34270V3.1 locus=Pp3c1_34270 annot-version=v3.3	Physcomitrella_patens OG0000003
Pp3c17_16280V3.1.p pacid=32906718 transcript=Pp3c17_16280V3.1 locus=Pp3c17_16280 annot-version=v3.3	Physcomitrella_patens OG0000003
Pp3c1_34920V3.1.p pacid=32971939 transcript=Pp3c1_34920V3.1 locus=Pp3c1_34920 annot-version=v3.3	Physcomitrella_patens OG0000003
Pp3c1_9450V3.1.p pacid=32972004 transcript=Pp3c1_9450V3.1 locus=Pp3c1_9450 annot-version=v3.3	Physcomitrella_patens OG0000003
Pp3c12_19340V3.1.p pacid=32972118 transcript=Pp3c12_19340V3.1 locus=Pp3c12_19340 annot-version=v3.3	Physcomitrella_patens OG0000003
Pp3c12_25890V3.1.p pacid=32972142 transcript=Pp3c12_25890V3.1 locus=Pp3c12_25890 annot-version=v3.3	Physcomitrella_patens OG0000003
Pp3c12_4930V3.1.p pacid=32972307 transcript=Pp3c12_4930V3.1 locus=Pp3c12_4930 annot-version=v3.3	Physcomitrella_patens OG0000003
Pp3c12_12090V3.1.p pacid=32972557 transcript=Pp3c12_12090V3.1 locus=Pp3c12_12090 annot-version=v3.3	Physcomitrella_patens OG0000003
Pp3c12_23880V3.1.p pacid=32973343 transcript=Pp3c12_23880V3.1 locus=Pp3c12_23880 annot-version=v3.3	Physcomitrella_patens OG0000003
Pp3c12_9150V3.1.p pacid=32973380 transcript=Pp3c12_9150V3.1 locus=Pp3c12_9150 annot-version=v3.3	Physcomitrella_patens OG0000003
Pp3c17_21230V3.1.p pacid=32906909 transcript=Pp3c17_21230V3.1 locus=Pp3c17_21230 annot-version=v3.3	Physcomitrella_patens OG0000003
Pp3c12_7790V3.1.p pacid=32973629 transcript=Pp3c12_7790V3.1 locus=Pp3c12_7790 annot-version=v3.3	Physcomitrella_patens OG0000003
Pp3c12_15570V3.1.p pacid=32974179 transcript=Pp3c12_15570V3.1 locus=Pp3c12_15570 annot-version=v3.3	Physcomitrella_patens OG0000003
Pp3c12_12830V3.1.p pacid=32974801 transcript=Pp3c12_12830V3.1 locus=Pp3c12_12830 annot-version=v3.3	Physcomitrella_patens OG0000003

Pp3c12_12280V3.1.p pacid=32975072 transcript=Pp3c12_12280V3.1 locus=Pp3c12_12280 annot-version=v3.3 Physcomitrella_patens OG0000003
Pp3c12_9160V3.1.p pacid=32975221 transcript=Pp3c12_9160V3.1 locus=Pp3c12_9160 annot-version=v3.3 Physcomitrella_patens OG0000003
Pp3c6_3320V3.1.p pacid=32975409 transcript=Pp3c6_3320V3.1 locus=Pp3c6_3320 annot-version=v3.3 Physcomitrella_patens OG0000003
Pp3c6_12620V3.1.p pacid=32975560 transcript=Pp3c6_12620V3.1 locus=Pp3c6_12620 annot-version=v3.3 Physcomitrella_patens OG0000003
Pp3c6_800V3.1.p pacid=32975682 transcript=Pp3c6_800V3.1 locus=Pp3c6_800 annot-version=v3.3 Physcomitrella_patens OG0000003
Pp3c6_4070V3.1.p pacid=32975847 transcript=Pp3c6_4070V3.1 locus=Pp3c6_4070 annot-version=v3.3 Physcomitrella_patens OG0000003
Pp3c6_3350V3.1.p pacid=32976208 transcript=Pp3c6_3350V3.1 locus=Pp3c6_3350 annot-version=v3.3 Physcomitrella_patens OG0000003
Pp3c6_17530V3.1.p pacid=32976634 transcript=Pp3c6_17530V3.1 locus=Pp3c6_17530 annot-version=v3.3 Physcomitrella_patens OG0000003
Pp3c6_17090V3.1.p pacid=32977246 transcript=Pp3c6_17090V3.1 locus=Pp3c6_17090 annot-version=v3.3 Physcomitrella_patens OG0000003
Pp3c6_19020V3.1.p pacid=32977367 transcript=Pp3c6_19020V3.1 locus=Pp3c6_19020 annot-version=v3.3 Physcomitrella_patens OG0000003
Pp3c6_9080V3.1.p pacid=32977682 transcript=Pp3c6_9080V3.1 locus=Pp3c6_9080 annot-version=v3.3 Physcomitrella_patens OG0000003
Pp3c6_18150V3.1.p pacid=32977990 transcript=Pp3c6_18150V3.1 locus=Pp3c6_18150 annot-version=v3.3 Physcomitrella_patens OG0000003
Pp3c6_28960V3.1.p pacid=32978087 transcript=Pp3c6_28960V3.1 locus=Pp3c6_28960 annot-version=v3.3 Physcomitrella_patens OG0000003
Pp3c6_9670V3.1.p pacid=32978427 transcript=Pp3c6_9670V3.1 locus=Pp3c6_9670 annot-version=v3.3 Physcomitrella_patens OG0000003
Pp3c6_26380V3.1.p pacid=32978711 transcript=Pp3c6_26380V3.1 locus=Pp3c6_26380 annot-version=v3.3 Physcomitrella_patens OG0000003
Pp3c6_29850V3.1.p pacid=32978734 transcript=Pp3c6_29850V3.1 locus=Pp3c6_29850 annot-version=v3.3 Physcomitrella_patens OG0000003
Pp3c6_24190V3.1.p pacid=32978886 transcript=Pp3c6_24190V3.1 locus=Pp3c6_24190 annot-version=v3.3 Physcomitrella_patens OG0000003
Pp3c6_19310V3.1.p pacid=32978953 transcript=Pp3c6_19310V3.1 locus=Pp3c6_19310 annot-version=v3.3 Physcomitrella_patens OG0000003

Pp3c6_8900V3.1.p pacid=32978984 transcript=Pp3c6_8900V3.1 locus=Pp3c6_8900 annot-version=v3.3 Physcomitrella_patens OG0000003
Pp3c25_5170V3.1.p pacid=32979881 transcript=Pp3c25_5170V3.1 locus=Pp3c25_5170 annot-version=v3.3 Physcomitrella_patens OG0000003
Pp3c25_10820V3.1.p pacid=32980338 transcript=Pp3c25_10820V3.1 locus=Pp3c25_10820 annot-version=v3.3 Physcomitrella_patens OG0000003
Pp3c17_15370V3.1.p pacid=32907596 transcript=Pp3c17_15370V3.1 locus=Pp3c17_15370 annot-version=v3.3 Physcomitrella_patens OG0000003
Pp3c25_11001V3.1.p pacid=32980438 transcript=Pp3c25_11001V3.1 locus=Pp3c25_11001 annot-version=v3.3 Physcomitrella_patens OG0000003
Pp3c25_5410V3.1.p pacid=32980479 transcript=Pp3c25_5410V3.1 locus=Pp3c25_5410 annot-version=v3.3 Physcomitrella_patens OG0000003
Pp3s64_160V3.1.p pacid=32980916 transcript=Pp3s64_160V3.1 locus=Pp3s64_160 annot-version=v3.3 Physcomitrella_patens OG0000003
Pp3c18_6460V3.1.p pacid=32981122 transcript=Pp3c18_6460V3.1 locus=Pp3c18_6460 annot-version=v3.3 Physcomitrella_patens OG0000003
Pp3c18_20460V3.1.p pacid=32981131 transcript=Pp3c18_20460V3.1 locus=Pp3c18_20460 annot-version=v3.3 Physcomitrella_patens OG0000003
Pp3c17_2840V3.1.p pacid=32907752 transcript=Pp3c17_2840V3.1 locus=Pp3c17_2840 annot-version=v3.3 Physcomitrella_patens OG0000003
Pp3c18_6440V3.1.p pacid=32981926 transcript=Pp3c18_6440V3.1 locus=Pp3c18_6440 annot-version=v3.3 Physcomitrella_patens OG0000003
Pp3c18_14670V3.1.p pacid=32981998 transcript=Pp3c18_14670V3.1 locus=Pp3c18_14670 annot-version=v3.3 Physcomitrella_patens OG0000003
Pp3c18_10510V3.1.p pacid=32982102 transcript=Pp3c18_10510V3.1 locus=Pp3c18_10510 annot-version=v3.3 Physcomitrella_patens OG0000003
Pp3c18_6600V3.1.p pacid=32982126 transcript=Pp3c18_6600V3.1 locus=Pp3c18_6600 annot-version=v3.3 Physcomitrella_patens OG0000003
Pp3c18_10520V3.1.p pacid=32982128 transcript=Pp3c18_10520V3.1 locus=Pp3c18_10520 annot-version=v3.3 Physcomitrella_patens OG0000003
Pp3c18_20660V3.1.p pacid=32982130 transcript=Pp3c18_20660V3.1 locus=Pp3c18_20660 annot-version=v3.3 Physcomitrella_patens OG0000003
Pp3c18_20830V3.1.p pacid=32982407 transcript=Pp3c18_20830V3.1 locus=Pp3c18_20830 annot-version=v3.3 Physcomitrella_patens OG0000003
Pp3c18_9450V3.1.p pacid=32982553 transcript=Pp3c18_9450V3.1 locus=Pp3c18_9450 annot-version=v3.3 Physcomitrella_patens OG0000003

Pp3c18_5410V3.1.p pacid=32982652 transcript=Pp3c18_5410V3.1 locus=Pp3c18_5410 annot-version=v3.3 Physcomitrella_patens OG0000003
Pp3c17_23810V3.1.p pacid=32907938 transcript=Pp3c17_23810V3.1 locus=Pp3c17_23810 annot-version=v3.3 Physcomitrella_patens OG0000003
Pp3c18_3680V3.1.p pacid=32983302 transcript=Pp3c18_3680V3.1 locus=Pp3c18_3680 annot-version=v3.3 Physcomitrella_patens OG0000003
Pp3c18_1850V3.1.p pacid=32983409 transcript=Pp3c18_1850V3.1 locus=Pp3c18_1850 annot-version=v3.3 Physcomitrella_patens OG0000003
Pp3c16_19670V3.1.p pacid=32984520 transcript=Pp3c16_19670V3.1 locus=Pp3c16_19670 annot-version=v3.3 Physcomitrella_patens OG0000003
Pp3c16_3110V3.1.p pacid=32984593 transcript=Pp3c16_3110V3.1 locus=Pp3c16_3110 annot-version=v3.3 Physcomitrella_patens OG0000003
Pp3c16_14760V3.1.p pacid=32984675 transcript=Pp3c16_14760V3.1 locus=Pp3c16_14760 annot-version=v3.3 Physcomitrella_patens OG0000003
Pp3c17_22130V3.1.p pacid=32908074 transcript=Pp3c17_22130V3.1 locus=Pp3c17_22130 annot-version=v3.3 Physcomitrella_patens OG0000003
Pp3c16_770V3.1.p pacid=32984882 transcript=Pp3c16_770V3.1 locus=Pp3c16_770 annot-version=v3.3 Physcomitrella_patens OG0000003
Pp3c16_17250V3.1.p pacid=32985074 transcript=Pp3c16_17250V3.1 locus=Pp3c16_17250 annot-version=v3.3 Physcomitrella_patens OG0000003
Pp3c16_13680V3.1.p pacid=32985103 transcript=Pp3c16_13680V3.1 locus=Pp3c16_13680 annot-version=v3.3 Physcomitrella_patens OG0000003
Pp3c16_15820V3.1.p pacid=32985112 transcript=Pp3c16_15820V3.1 locus=Pp3c16_15820 annot-version=v3.3 Physcomitrella_patens OG0000003
Pp3c16_7680V3.1.p pacid=32985131 transcript=Pp3c16_7680V3.1 locus=Pp3c16_7680 annot-version=v3.3 Physcomitrella_patens OG0000003
Pp3c16_19660V3.1.p pacid=32985132 transcript=Pp3c16_19660V3.1 locus=Pp3c16_19660 annot-version=v3.3 Physcomitrella_patens OG0000003
Pp3c16_5250V3.1.p pacid=32985209 transcript=Pp3c16_5250V3.1 locus=Pp3c16_5250 annot-version=v3.3 Physcomitrella_patens OG0000003
Pp3c16_5260V3.1.p pacid=32985508 transcript=Pp3c16_5260V3.1 locus=Pp3c16_5260 annot-version=v3.3 Physcomitrella_patens OG0000003
Pp3c16_19510V3.1.p pacid=32986004 transcript=Pp3c16_19510V3.1 locus=Pp3c16_19510 annot-version=v3.3 Physcomitrella_patens OG0000003
Pp3c16_4540V3.1.p pacid=32986319 transcript=Pp3c16_4540V3.1 locus=Pp3c16_4540 annot-version=v3.3 Physcomitrella_patens OG0000003

Pp3c16_70V3.1.p pacid=32986388 transcript=Pp3c16_70V3.1 locus=Pp3c16_70 annot-version=v3.3 Physcomitrella_patens OG0000003
Pp3c16_21500V3.1.p pacid=32986580 transcript=Pp3c16_21500V3.1 locus=Pp3c16_21500 annot-version=v3.3 Physcomitrella_patens OG0000003
Pp3c17_23340V3.1.p pacid=32908281 transcript=Pp3c17_23340V3.1 locus=Pp3c17_23340 annot-version=v3.3 Physcomitrella_patens OG0000003
Pp3c17_12750V3.1.p pacid=32908391 transcript=Pp3c17_12750V3.1 locus=Pp3c17_12750 annot-version=v3.3 Physcomitrella_patens OG0000003
Pp3c17_12640V3.1.p pacid=32908429 transcript=Pp3c17_12640V3.1 locus=Pp3c17_12640 annot-version=v3.3 Physcomitrella_patens OG0000003
Pp3c24_16280V3.1.p pacid=32909220 transcript=Pp3c24_16280V3.1 locus=Pp3c24_16280 annot-version=v3.3 Physcomitrella_patens OG0000003
Pp3c24_850V3.1.p pacid=32909387 transcript=Pp3c24_850V3.1 locus=Pp3c24_850 annot-version=v3.3 Physcomitrella_patens OG0000003
Pp3c24_16290V3.1.p pacid=32910785 transcript=Pp3c24_16290V3.1 locus=Pp3c24_16290 annot-version=v3.3 Physcomitrella_patens OG0000003
Pp3c24_9180V3.1.p pacid=32910946 transcript=Pp3c24_9180V3.1 locus=Pp3c24_9180 annot-version=v3.3 Physcomitrella_patens OG0000003
Pp3c9_10660V3.1.p pacid=32911571 transcript=Pp3c9_10660V3.1 locus=Pp3c9_10660 annot-version=v3.3 Physcomitrella_patens OG0000003
Pp3c9_16900V3.1.p pacid=32911636 transcript=Pp3c9_16900V3.1 locus=Pp3c9_16900 annot-version=v3.3 Physcomitrella_patens OG0000003
Pp3c9_8540V3.1.p pacid=32911676 transcript=Pp3c9_8540V3.1 locus=Pp3c9_8540 annot-version=v3.3 Physcomitrella_patens OG0000003
Pp3c9_2660V3.1.p pacid=32911719 transcript=Pp3c9_2660V3.1 locus=Pp3c9_2660 annot-version=v3.3 Physcomitrella_patens OG0000003
Pp3c9_7160V3.1.p pacid=32911921 transcript=Pp3c9_7160V3.1 locus=Pp3c9_7160 annot-version=v3.3 Physcomitrella_patens OG0000003
Pp3c9_20430V3.1.p pacid=32912205 transcript=Pp3c9_20430V3.1 locus=Pp3c9_20430 annot-version=v3.3 Physcomitrella_patens OG0000003
Pp3c9_7240V3.1.p pacid=32912740 transcript=Pp3c9_7240V3.1 locus=Pp3c9_7240 annot-version=v3.3 Physcomitrella_patens OG0000003
Pp3c10_20880V3.1.p pacid=32899376 transcript=Pp3c10_20880V3.1 locus=Pp3c10_20880 annot-version=v3.3 Physcomitrella_patens OG0000003
Pp3c9_20440V3.1.p pacid=32912836 transcript=Pp3c9_20440V3.1 locus=Pp3c9_20440 annot-version=v3.3 Physcomitrella_patens OG0000003

Pp3c9_1850V3.1.p pacid=32912903 transcript=Pp3c9_1850V3.1 locus=Pp3c9_1850 annot-version=v3.3 Physcomitrella_patens OG0000003
Pp3c9_2910V3.1.p pacid=32913074 transcript=Pp3c9_2910V3.1 locus=Pp3c9_2910 annot-version=v3.3 Physcomitrella_patens OG0000003
Pp3c9_24680V3.1.p pacid=32913214 transcript=Pp3c9_24680V3.1 locus=Pp3c9_24680 annot-version=v3.3 Physcomitrella_patens OG0000003
Pp3c9_1860V3.1.p pacid=32913588 transcript=Pp3c9_1860V3.1 locus=Pp3c9_1860 annot-version=v3.3 Physcomitrella_patens OG0000003
Pp3c9_8550V3.1.p pacid=32913602 transcript=Pp3c9_8550V3.1 locus=Pp3c9_8550 annot-version=v3.3 Physcomitrella_patens OG0000003
Pp3c9_2918V3.1.p pacid=32913628 transcript=Pp3c9_2918V3.1 locus=Pp3c9_2918 annot-version=v3.3 Physcomitrella_patens OG0000003
Pp3c9_25800V3.1.p pacid=32914308 transcript=Pp3c9_25800V3.1 locus=Pp3c9_25800 annot-version=v3.3 Physcomitrella_patens OG0000003
Pp3c21_14950V3.1.p pacid=32914521 transcript=Pp3c21_14950V3.1 locus=Pp3c21_14950 annot-version=v3.3 Physcomitrella_patens OG0000003
Pp3c21_14170V3.1.p pacid=32914592 transcript=Pp3c21_14170V3.1 locus=Pp3c21_14170 annot-version=v3.3 Physcomitrella_patens OG0000003
Pp3c21_12890V3.1.p pacid=32914957 transcript=Pp3c21_12890V3.1 locus=Pp3c21_12890 annot-version=v3.3 Physcomitrella_patens OG0000003
Pp3c10_25320V3.1.p pacid=32899510 transcript=Pp3c10_25320V3.1 locus=Pp3c10_25320 annot-version=v3.3 Physcomitrella_patens OG0000003
Pp3c21_4820V3.1.p pacid=32915028 transcript=Pp3c21_4820V3.1 locus=Pp3c21_4820 annot-version=v3.3 Physcomitrella_patens OG0000003
Pp3c21_20990V3.1.p pacid=32915774 transcript=Pp3c21_20990V3.1 locus=Pp3c21_20990 annot-version=v3.3 Physcomitrella_patens OG0000003
Pp3c21_16230V3.1.p pacid=32916325 transcript=Pp3c21_16230V3.1 locus=Pp3c21_16230 annot-version=v3.3 Physcomitrella_patens OG0000003
Pp3c21_14390V3.1.p pacid=32916470 transcript=Pp3c21_14390V3.1 locus=Pp3c21_14390 annot-version=v3.3 Physcomitrella_patens OG0000003
Pp3c26_880V3.1.p pacid=32917304 transcript=Pp3c26_880V3.1 locus=Pp3c26_880 annot-version=v3.3 Physcomitrella_patens OG0000003
Pp3c10_8280V3.1.p pacid=32901220 transcript=Pp3c10_8280V3.1 locus=Pp3c10_8280 annot-version=v3.3 Physcomitrella_patens OG0000003
Pp3c26_14020V3.1.p pacid=32917604 transcript=Pp3c26_14020V3.1 locus=Pp3c26_14020 annot-version=v3.3 Physcomitrella_patens OG0000003

Pp3c26_871V3.1.p pacid=32917777 transcript=Pp3c26_871V3.1 locus=Pp3c26_871 annot-version=v3.3 Physcomitrella_patens OG0000003
Pp3c26_1660V3.1.p pacid=32917779 transcript=Pp3c26_1660V3.1 locus=Pp3c26_1660 annot-version=v3.3 Physcomitrella_patens OG0000003
Pp3c26_790V3.1.p pacid=32917787 transcript=Pp3c26_790V3.1 locus=Pp3c26_790 annot-version=v3.3 Physcomitrella_patens OG0000003
Pp3c26_900V3.1.p pacid=32917977 transcript=Pp3c26_900V3.1 locus=Pp3c26_900 annot-version=v3.3 Physcomitrella_patens OG0000003
Pp3c26_13980V3.1.p pacid=32918039 transcript=Pp3c26_13980V3.1 locus=Pp3c26_13980 annot-version=v3.3 Physcomitrella_patens OG0000003
Pp3c26_3260V3.1.p pacid=32918060 transcript=Pp3c26_3260V3.1 locus=Pp3c26_3260 annot-version=v3.3 Physcomitrella_patens OG0000003
Pp3c26_1080V3.1.p pacid=32918446 transcript=Pp3c26_1080V3.1 locus=Pp3c26_1080 annot-version=v3.3 Physcomitrella_patens OG0000003
Pp3c26_13990V3.1.p pacid=32918482 transcript=Pp3c26_13990V3.1 locus=Pp3c26_13990 annot-version=v3.3 Physcomitrella_patens OG0000003
Pp3c10_10414V3.1.p pacid=32901321 transcript=Pp3c10_10414V3.1 locus=Pp3c10_10414 annot-version=v3.3 Physcomitrella_patens OG0000003
Pp3c10_20570V3.1.p pacid=32899550 transcript=Pp3c10_20570V3.1 locus=Pp3c10_20570 annot-version=v3.3 Physcomitrella_patens OG0000003
Pp3s38_160V3.1.p pacid=32918797 transcript=Pp3s38_160V3.1 locus=Pp3s38_160 annot-version=v3.3 Physcomitrella_patens OG0000003
Pp3c4_10670V3.1.p pacid=32918864 transcript=Pp3c4_10670V3.1 locus=Pp3c4_10670 annot-version=v3.3 Physcomitrella_patens OG0000003
Pp3c4_29350V3.1.p pacid=32919053 transcript=Pp3c4_29350V3.1 locus=Pp3c4_29350 annot-version=v3.3 Physcomitrella_patens OG0000003
Pp3c4_31000V3.1.p pacid=32919220 transcript=Pp3c4_31000V3.1 locus=Pp3c4_31000 annot-version=v3.3 Physcomitrella_patens OG0000003
Pp3c4_7683V3.1.p pacid=32919297 transcript=Pp3c4_7683V3.1 locus=Pp3c4_7683 annot-version=v3.3 Physcomitrella_patens OG0000003
Pp3c4_960V3.1.p pacid=32919364 transcript=Pp3c4_960V3.1 locus=Pp3c4_960 annot-version=v3.3 Physcomitrella_patens OG0000003
Pp3c4_28400V3.1.p pacid=32919517 transcript=Pp3c4_28400V3.1 locus=Pp3c4_28400 annot-version=v3.3 Physcomitrella_patens OG0000003
Pp3c4_18120V3.1.p pacid=32919679 transcript=Pp3c4_18120V3.1 locus=Pp3c4_18120 annot-version=v3.3 Physcomitrella_patens OG0000003

Pp3c4_16340V3.1.p pacid=32919731 transcript=Pp3c4_16340V3.1 locus=Pp3c4_16340 annot-version=v3.3 Physcomitrella_patens OG0000003
Pp3c4_18500V3.1.p pacid=32919742 transcript=Pp3c4_18500V3.1 locus=Pp3c4_18500 annot-version=v3.3 Physcomitrella_patens OG0000003
Pp3c4_12770V3.1.p pacid=32919793 transcript=Pp3c4_12770V3.1 locus=Pp3c4_12770 annot-version=v3.3 Physcomitrella_patens OG0000003
Pp3c4_28420V3.1.p pacid=32919932 transcript=Pp3c4_28420V3.1 locus=Pp3c4_28420 annot-version=v3.3 Physcomitrella_patens OG0000003
Pp3c4_5560V3.1.p pacid=32919967 transcript=Pp3c4_5560V3.1 locus=Pp3c4_5560 annot-version=v3.3 Physcomitrella_patens OG0000003
Pp3c4_10620V3.1.p pacid=32920753 transcript=Pp3c4_10620V3.1 locus=Pp3c4_10620 annot-version=v3.3 Physcomitrella_patens OG0000003
Pp3c4_8530V3.1.p pacid=32920873 transcript=Pp3c4_8530V3.1 locus=Pp3c4_8530 annot-version=v3.3 Physcomitrella_patens OG0000003
Pp3c4_15420V3.1.p pacid=32920902 transcript=Pp3c4_15420V3.1 locus=Pp3c4_15420 annot-version=v3.3 Physcomitrella_patens OG0000003
Pp3c4_23690V3.1.p pacid=32920986 transcript=Pp3c4_23690V3.1 locus=Pp3c4_23690 annot-version=v3.3 Physcomitrella_patens OG0000003
Pp3c4_27890V3.1.p pacid=32921201 transcript=Pp3c4_27890V3.1 locus=Pp3c4_27890 annot-version=v3.3 Physcomitrella_patens OG0000003
Pp3c4_19620V3.1.p pacid=32921242 transcript=Pp3c4_19620V3.1 locus=Pp3c4_19620 annot-version=v3.3 Physcomitrella_patens OG0000003
Pp3c4_14498V3.1.p pacid=32921478 transcript=Pp3c4_14498V3.1 locus=Pp3c4_14498 annot-version=v3.3 Physcomitrella_patens OG0000003
Pp3c4_8550V3.1.p pacid=32921873 transcript=Pp3c4_8550V3.1 locus=Pp3c4_8550 annot-version=v3.3 Physcomitrella_patens OG0000003
Pp3c4_1500V3.1.p pacid=32921885 transcript=Pp3c4_1500V3.1 locus=Pp3c4_1500 annot-version=v3.3 Physcomitrella_patens OG0000003
Pp3c4_1530V3.1.p pacid=32921902 transcript=Pp3c4_1530V3.1 locus=Pp3c4_1530 annot-version=v3.3 Physcomitrella_patens OG0000003
Pp3c10_13350V3.1.p pacid=32901677 transcript=Pp3c10_13350V3.1 locus=Pp3c10_13350 annot-version=v3.3 Physcomitrella_patens OG0000003
Pp3c4_20260V3.1.p pacid=32922299 transcript=Pp3c4_20260V3.1 locus=Pp3c4_20260 annot-version=v3.3 Physcomitrella_patens OG0000003
Pp3c4_5910V3.1.p pacid=32922345 transcript=Pp3c4_5910V3.1 locus=Pp3c4_5910 annot-version=v3.3 Physcomitrella_patens OG0000003

Pp3c4_28390V3.1.p pacid=32922377 transcript=Pp3c4_28390V3.1
locus=Pp3c4_28390 annot-version=v3.3 Physcomitrella_patens
OG0000003
Pp3c4_7430V3.1.p pacid=32922477 transcript=Pp3c4_7430V3.1
locus=Pp3c4_7430 annot-version=v3.3 Physcomitrella_patens
OG0000003
Pp3c4_780V3.1.p pacid=32922710 transcript=Pp3c4_780V3.1
locus=Pp3c4_780 annot-version=v3.3 Physcomitrella_patens
OG0000003
Pp3c7_3830V3.1.p pacid=32923077 transcript=Pp3c7_3830V3.1
locus=Pp3c7_3830 annot-version=v3.3 Physcomitrella_patens
OG0000003
Pp3c7_7880V3.1.p pacid=32923808 transcript=Pp3c7_7880V3.1
locus=Pp3c7_7880 annot-version=v3.3 Physcomitrella_patens
OG0000003
Pp3c7_15950V3.1.p pacid=32924543 transcript=Pp3c7_15950V3.1
locus=Pp3c7_15950 annot-version=v3.3 Physcomitrella_patens
OG0000003
Pp3c7_3400V3.1.p pacid=32924718 transcript=Pp3c7_3400V3.1
locus=Pp3c7_3400 annot-version=v3.3 Physcomitrella_patens
OG0000003
AT3G20980.1 | Gag-Pol-related retrotransposon family protein |
Chr3:7355963-7357966 FORWARD LENGTH=405 | 201606
Arabidopsis_thaliana OG0000003
AT3G21000.1 | Gag-Pol-related retrotransposon family protein |
Chr3:7363921-7365138 FORWARD LENGTH=405 | 201606
Arabidopsis_thaliana OG0000003
AT1G21280.1 | Copia-like polyprotein/retrotransposon |
Chr1:7447690-7448403 REVERSE LENGTH=237 | 201606
Arabidopsis_thaliana OG0000003
AT5G48050.1 | Copia-like polyprotein/retrotransposon |
Chr5:19472661-19473770 REVERSE LENGTH=369 | 201606
Arabidopsis_thaliana OG0000003
AT1G34070.1 | Copia-like polyprotein/retrotransposon |
Chr1:12402283-12403209 FORWARD LENGTH=308 | 201606
Arabidopsis_thaliana OG0000003
AT1G48720.1 | Copia-like polyprotein/retrotransposon |
Chr1:18018148-18018441 FORWARD LENGTH=97 | 201606
Arabidopsis_thaliana OG0000003
Os04t0313600-00 Zinc finger, CCHC-type domain containing protein.
Oryza_sativa OG0000003
Os04t0324550-01 Zinc finger, CCHC-type domain containing protein.
Oryza_sativa OG0000003
Os05t0380700-01 Conserved hypothetical protein. Oryza_sativa
OG0000003
Os05t0442550-01 Similar to H0801D08.16 protein. Oryza_sativa
OG0000003
Os06t0684500-01 Conserved hypothetical protein. Oryza_sativa
OG0000003
Os08t0125300-01 Integrase, catalytic core domain containing protein.
Oryza_sativa OG0000003
Os10t0432200-01 Conserved hypothetical protein. Oryza_sativa
OG0000003
Os11t0656500-01 Integrase, catalytic core domain containing protein.

Oryza_sativa	OG0000003
Os11t0678200-01 Conserved hypothetical protein.	Oryza_sativa
OG0000003
Os11t0678301-00 Hypothetical conserved gene.	Oryza_sativa
OG0000003
Os01t0909600-01 Integrase, catalytic core domain containing protein.
Oryza_sativa	OG0000003
Os02t0671800-01 Integrase, catalytic core domain containing protein.
Oryza_sativa	OG0000003
evm.model.contig_2091.1	Porphyridium_purpureum	OG0000003
evm.model.contig_2091.3	Porphyridium_purpureum	OG0000003
evm.model.contig_2166.3	Porphyridium_purpureum	OG0000003
evm.model.contig_2182.1	Porphyridium_purpureum	OG0000003
evm.model.contig_2212.2	Porphyridium_purpureum	OG0000003
evm.model.contig_2214.1	Porphyridium_purpureum	OG0000003
evm.model.contig_2237.1	Porphyridium_purpureum	OG0000003
evm.model.contig_2245.1	Porphyridium_purpureum	OG0000003
evm.model.contig_2292.1	Porphyridium_purpureum	OG0000003
evm.model.contig_2292.2	Porphyridium_purpureum	OG0000003
evm.model.contig_2292.3	Porphyridium_purpureum	OG0000003
evm.model.contig_2311.1	Porphyridium_purpureum	OG0000003
evm.model.contig_2322.1	Porphyridium_purpureum	OG0000003
evm.model.contig_2026.2	Porphyridium_purpureum	OG0000003
evm.model.contig_2395.1	Porphyridium_purpureum	OG0000003
evm.model.contig_2026.1	Porphyridium_purpureum	OG0000003
evm.model.contig_2419.2	Porphyridium_purpureum	OG0000003
evm.model.contig_2441.1	Porphyridium_purpureum	OG0000003
evm.model.contig_2445.7	Porphyridium_purpureum	OG0000003
evm.model.contig_2449.3	Porphyridium_purpureum	OG0000003
evm.model.contig_2449.1	Porphyridium_purpureum	OG0000003
evm.model.contig_2449.2	Porphyridium_purpureum	OG0000003
evm.model.contig_2450.1	Porphyridium_purpureum	OG0000003
evm.model.contig_2497.2	Porphyridium_purpureum	OG0000003
evm.model.contig_2530.1	Porphyridium_purpureum	OG0000003
evm.model.contig_2531.1	Porphyridium_purpureum	OG0000003
evm.model.contig_2541.1	Porphyridium_purpureum	OG0000003
evm.model.contig_3460.4	Porphyridium_purpureum	OG0000003
evm.model.contig_3496.2	Porphyridium_purpureum	OG0000003
evm.model.contig_3513.1	Porphyridium_purpureum	OG0000003
evm.model.contig_3517.1	Porphyridium_purpureum	OG0000003
evm.model.contig_3533.1	Porphyridium_purpureum	OG0000003
evm.model.contig_3565.4	Porphyridium_purpureum	OG0000003
evm.model.contig_3584.2	Porphyridium_purpureum	OG0000003
evm.model.contig_3584.1	Porphyridium_purpureum	OG0000003
evm.model.contig_3644.2	Porphyridium_purpureum	OG0000003
evm.model.contig_3644.1	Porphyridium_purpureum	OG0000003
evm.model.contig_3711.1	Porphyridium_purpureum	OG0000003
evm.model.contig_3718.1	Porphyridium_purpureum	OG0000003
evm.model.contig_3730.1	Porphyridium_purpureum	OG0000003
evm.model.contig_3732.1	Porphyridium_purpureum	OG0000003
evm.model.contig_3735.2	Porphyridium_purpureum	OG0000003
evm.model.contig_3737.1	Porphyridium_purpureum	OG0000003
evm.model.contig_3747.1	Porphyridium_purpureum	OG0000003
evm.model.contig_3776.2	Porphyridium_purpureum	OG0000003

```
evm.model.contig_3813.1  Porphyridium_purpureum   OG0000003
evm.model.contig_2047.1  Porphyridium_purpureum   OG0000003
evm.model.contig_4504.1  Porphyridium_purpureum   OG0000003
evm.model.contig_4504.2  Porphyridium_purpureum   OG0000003
evm.model.contig_4508.4  Porphyridium_purpureum   OG0000003
evm.model.contig_4533.1  Porphyridium_purpureum   OG0000003
evm.model.contig_4534.3  Porphyridium_purpureum   OG0000003
evm.model.contig_4534.5  Porphyridium_purpureum   OG0000003
evm.model.contig_4568.1  Porphyridium_purpureum   OG0000003
evm.model.contig_4573.1  Porphyridium_purpureum   OG0000003
evm.model.contig_459.1   Porphyridium_purpureum   OG0000003
evm.model.contig_4617.1  Porphyridium_purpureum   OG0000003
evm.model.contig_4650.2  Porphyridium_purpureum   OG0000003
evm.model.contig_577.2   Porphyridium_purpureum   OG0000003
evm.model.contig_577.4   Porphyridium_purpureum   OG0000003
evm.model.contig_577.3   Porphyridium_purpureum   OG0000003
evm.model.contig_694.1   Porphyridium_purpureum   OG0000003
evm.model.contig_795.1   Porphyridium_purpureum   OG0000003
evm.model.contig_845.1   Porphyridium_purpureum   OG0000003
evm.model.contig_853.1   Porphyridium_purpureum   OG0000003
evm.model.contig_867.1   Porphyridium_purpureum   OG0000003
evm.model.contig_2078.1  Porphyridium_purpureum   OG0000003
CDF40674 pep supercontig:ASM35022v2:HG002227:321469:322023:-1
gene:CHC_T00007046001 transcript:CDF40674
gene_biotype:protein_coding transcript_biotype:protein_coding
Chondrus_crispus OG0000003
CDF33464 pep supercontig:ASM35022v2:HG001647:90879:93754:-1
gene:CHC_T00002257001 transcript:CDF33464
gene_biotype:protein_coding transcript_biotype:protein_coding
Chondrus_crispus OG0000003
CDF38282 pep supercontig:ASM35022v2:HG001914:209811:210599:1
gene:CHC_T00006108001 transcript:CDF38282
gene_biotype:protein_coding transcript_biotype:protein_coding
Chondrus_crispus OG0000003
CDF38077 pep supercontig:ASM35022v2:HG001898:338494:339449:1
gene:CHC_T00006266001 transcript:CDF38077
gene_biotype:protein_coding transcript_biotype:protein_coding
Chondrus_crispus OG0000003
CDF40831 pep supercontig:ASM35022v2:HG002253:9662:12323:-1
gene:CHC_T00000876001 transcript:CDF40831
gene_biotype:protein_coding transcript_biotype:protein_coding
Chondrus_crispus OG0000003
CDF38369 pep supercontig:ASM35022v2:HG001921:64425:67034:1
gene:CHC_T00000970001 transcript:CDF38369
gene_biotype:protein_coding transcript_biotype:protein_coding
Chondrus_crispus OG0000003
CDF38368 pep supercontig:ASM35022v2:HG001921:33508:36090:1
gene:CHC_T00000969001 transcript:CDF38368
gene_biotype:protein_coding transcript_biotype:protein_coding
Chondrus_crispus OG0000003
CDF32625 pep supercontig:ASM35022v2:HG001524:275944:277664:-1
gene:CHC_T00008234001 transcript:CDF32625
gene_biotype:protein_coding transcript_biotype:protein_coding
Chondrus_crispus OG0000003
```

CDF38468 pep supercontig:ASM35022v2:HG001933:14984:15880:1
gene:CHC_T00001045001 transcript:CDF38468
gene_biotype:protein_coding transcript_biotype:protein_coding
Chondrus_crispus OG0000003
CDF36552 pep supercontig:ASM35022v2:HG001786:119771:120547:1
gene:CHC_T00005007001 transcript:CDF36552
gene_biotype:protein_coding transcript_biotype:protein_coding
Chondrus_crispus OG0000003
CDF36551 pep supercontig:ASM35022v2:HG001786:83234:84013:1
gene:CHC_T00005006001 transcript:CDF36551
gene_biotype:protein_coding transcript_biotype:protein_coding
Chondrus_crispus OG0000003
CDF34776 pep supercontig:ASM35022v2:HG001706:572879:573484:-1
gene:CHC_T00003690001 transcript:CDF34776
gene_biotype:protein_coding transcript_biotype:protein_coding
Chondrus_crispus OG0000003
CDF36411 pep supercontig:ASM35022v2:HG001777:113166:114650:1
gene:CHC_T00004839001 transcript:CDF36411
gene_biotype:protein_coding transcript_biotype:protein_coding
Chondrus_crispus OG0000003
CDF37987 pep supercontig:ASM35022v2:HG001890:448746:451390:1
gene:CHC_T00005980001 transcript:CDF37987
gene_biotype:protein_coding transcript_biotype:protein_coding
Chondrus_crispus OG0000003
CDF35378 pep supercontig:ASM35022v2:HG001729:304175:304921:1
gene:CHC_T00003431001 transcript:CDF35378
gene_biotype:protein_coding transcript_biotype:protein_coding
Chondrus_crispus OG0000003
CDF38270 pep supercontig:ASM35022v2:HG001912:67894:69275:-1
gene:CHC_T00000802001 transcript:CDF38270
gene_biotype:protein_coding transcript_biotype:protein_coding
Chondrus_crispus OG0000003
CDF32368 pep supercontig:ASM35022v2:HG001488:117:2390:1
gene:CHC_T00001006001 transcript:CDF32368
gene_biotype:protein_coding transcript_biotype:protein_coding
Chondrus_crispus OG0000003
CDF77536 pep supercontig:ASM35022v2:HG001843:262967:267064:-1
gene:CHC_T00010200001 transcript:CDF77536
gene_biotype:protein_coding transcript_biotype:protein_coding
description:Retrovirus-related Gag-Pol polyprotein Chondrus_crispus
OG0000003
CDF77535 pep supercontig:ASM35022v2:HG001843:254609:258727:-1
gene:CHC_T00010272001 transcript:CDF77535
gene_biotype:protein_coding transcript_biotype:protein_coding
description:RNA-dependent DNA polymerase Chondrus_crispus
OG0000003
CDF77556 pep supercontig:ASM35022v2:HG001843:386909:391006:-1
gene:CHC_T00010154001 transcript:CDF77556
gene_biotype:protein_coding transcript_biotype:protein_coding
description:Retrovirus-related Gag-Pol polyprotein Chondrus_crispus
OG0000003
CDF77534 pep supercontig:ASM35022v2:HG001843:248401:249313:-1
gene:CHC_T00005394001 transcript:CDF77534
gene_biotype:protein_coding transcript_biotype:protein_coding

Chondrus_crispus OG0000003
CDF34681 pep supercontig:ASM35022v2:HG001705:137325:137987:1
gene:CHC_T00003400001 transcript:CDF34681
gene_biotype:protein_coding transcript_biotype:protein_coding
Chondrus_crispus OG0000003
CDF39400 pep supercontig:ASM35022v2:HG002030:6038:6136:-1
gene:CHC_T00000262001 transcript:CDF39400
gene_biotype:protein_coding transcript_biotype:protein_coding
Chondrus_crispus OG0000003
CDF37707 pep supercontig:ASM35022v2:HG001863:69574:71847:1
gene:CHC_T00005954001 transcript:CDF37707
gene_biotype:protein_coding transcript_biotype:protein_coding
Chondrus_crispus OG0000003
CDF37235 pep supercontig:ASM35022v2:HG001820:86737:87525:-1
gene:CHC_T00005357001 transcript:CDF37235
gene_biotype:protein_coding transcript_biotype:protein_coding
Chondrus_crispus OG0000003
CDF33697 pep supercontig:ASM35022v2:HG001654:218199:218747:1
gene:CHC_T00002395001 transcript:CDF33697
gene_biotype:protein_coding transcript_biotype:protein_coding
Chondrus_crispus OG0000003
CDF37501 pep supercontig:ASM35022v2:HG001844:79076:79864:1
gene:CHC_T00005785001 transcript:CDF37501
gene_biotype:protein_coding transcript_biotype:protein_coding
Chondrus_crispus OG0000003
CDF36216 pep supercontig:ASM35022v2:HG001766:93225:93944:-1
gene:CHC_T00004607001 transcript:CDF36216
gene_biotype:protein_coding transcript_biotype:protein_coding
Chondrus_crispus OG0000003
CDF40429 pep supercontig:ASM35022v2:HG002176:66617:68476:1
gene:CHC_T00007193001 transcript:CDF40429
gene_biotype:protein_coding transcript_biotype:protein_coding
Chondrus_crispus OG0000003
CDF40427 pep supercontig:ASM35022v2:HG002176:23607:25556:-1
gene:CHC_T00007192001 transcript:CDF40427
gene_biotype:protein_coding transcript_biotype:protein_coding
Chondrus_crispus OG0000003
CDF39895 pep supercontig:ASM35022v2:HG002088:25731:26237:-1
gene:CHC_T00000484001 transcript:CDF39895
gene_biotype:protein_coding transcript_biotype:protein_coding
Chondrus_crispus OG0000003
CDF38989 pep supercontig:ASM35022v2:HG001983:594624:595247:1
gene:CHC_T00006701001 transcript:CDF38989
gene_biotype:protein_coding transcript_biotype:protein_coding
Chondrus_crispus OG0000003
CDF39894 pep supercontig:ASM35022v2:HG002088:19154:23268:-1
gene:CHC_T00000483001 transcript:CDF39894
gene_biotype:protein_coding transcript_biotype:protein_coding
Chondrus_crispus OG0000003
CDF39893 pep supercontig:ASM35022v2:HG002088:12966:17080:-1
gene:CHC_T00000482001 transcript:CDF39893
gene_biotype:protein_coding transcript_biotype:protein_coding
Chondrus_crispus OG0000003
CDF41419 pep supercontig:ASM35022v2:HG002379:75414:76076:-1

gene:CHC_T00008001001 transcript:CDF41419
gene_biotype:protein_coding transcript_biotype:protein_coding
Chondrus_crispus OG0000003
CDF39892 pep supercontig:ASM35022v2:HG002088:6778:10892:-1
gene:CHC_T00000481001 transcript:CDF39892
gene_biotype:protein_coding transcript_biotype:protein_coding
Chondrus_crispus OG0000003
CDF39896 pep supercontig:ASM35022v2:HG002088:29261:32257:-1
gene:CHC_T00000485001 transcript:CDF39896
gene_biotype:protein_coding transcript_biotype:protein_coding
Chondrus_crispus OG0000003
CDF39897 pep supercontig:ASM35022v2:HG002088:33160:36846:-1
gene:CHC_T00000486001 transcript:CDF39897
gene_biotype:protein_coding transcript_biotype:protein_coding
Chondrus_crispus OG0000003
CDF34864 pep supercontig:ASM35022v2:HG001707:386741:389221:-1
gene:CHC_T00003213001 transcript:CDF34864
gene_biotype:protein_coding transcript_biotype:protein_coding
Chondrus_crispus OG0000003
CDF39842 pep supercontig:ASM35022v2:HG002084:19280:21190:1
gene:CHC_T00000447001 transcript:CDF39842
gene_biotype:protein_coding transcript_biotype:protein_coding
Chondrus_crispus OG0000003
CDF39891 pep supercontig:ASM35022v2:HG002088:595:4704:-1
gene:CHC_T00000480001 transcript:CDF39891
gene_biotype:protein_coding transcript_biotype:protein_coding
Chondrus_crispus OG0000003
CDF38524 pep supercontig:ASM35022v2:HG001940:179017:179787:-1
gene:CHC_T00006244001 transcript:CDF38524
gene_biotype:protein_coding transcript_biotype:protein_coding
Chondrus_crispus OG0000003
CDF33638 pep supercontig:ASM35022v2:HG001652:119137:119799:-1
gene:CHC_T00002368001 transcript:CDF33638
gene_biotype:protein_coding transcript_biotype:protein_coding
Chondrus_crispus OG0000003
CDF33643 pep supercontig:ASM35022v2:HG001652:164093:164608:-1
gene:CHC_T00002373001 transcript:CDF33643
gene_biotype:protein_coding transcript_biotype:protein_coding
Chondrus_crispus OG0000003
CDF39664 pep supercontig:ASM35022v2:HG002064:41458:44148:-1
gene:CHC_T00000348001 transcript:CDF39664
gene_biotype:protein_coding transcript_biotype:protein_coding
Chondrus_crispus OG0000003
CDF33176 pep supercontig:ASM35022v2:HG001635:210183:210968:1
gene:CHC_T00002022001 transcript:CDF33176
gene_biotype:protein_coding transcript_biotype:protein_coding
Chondrus_crispus OG0000003
CDF39395 pep supercontig:ASM35022v2:HG002028:19505:21067:1
gene:CHC_T00000259001 transcript:CDF39395
gene_biotype:protein_coding transcript_biotype:protein_coding
Chondrus_crispus OG0000003
CDF40943 pep supercontig:ASM35022v2:HG002277:5986:6444:-1
gene:CHC_T00000903001 transcript:CDF40943
gene_biotype:protein_coding transcript_biotype:protein_coding

Chondrus_crispus OG0000003
CDF35155 pep supercontig:ASM35022v2:HG001719:188752:190392:1
gene:CHC_T00003582001 transcript:CDF35155
gene_biotype:protein_coding transcript_biotype:protein_coding
Chondrus_crispus OG0000003
CDF34544 pep supercontig:ASM35022v2:HG001696:168334:168666:1
gene:CHC_T00003178001 transcript:CDF34544
gene_biotype:protein_coding transcript_biotype:protein_coding
Chondrus_crispus OG0000003
CDF32156 pep supercontig:ASM35022v2:HG001459:379719:380624:-1
gene:CHC_T00001384001 transcript:CDF32156
gene_biotype:protein_coding transcript_biotype:protein_coding
Chondrus_crispus OG0000003
CDF39993 pep supercontig:ASM35022v2:HG002102:393:2714:1
gene:CHC_T00000622001 transcript:CDF39993
gene_biotype:protein_coding transcript_biotype:protein_coding
Chondrus_crispus OG0000003
CDF34349 pep supercontig:ASM35022v2:HG001687:20683:22430:1
gene:CHC_T00002976001 transcript:CDF34349
gene_biotype:protein_coding transcript_biotype:protein_coding
Chondrus_crispus OG0000003
CDF34531 pep supercontig:ASM35022v2:HG001695:555952:557901:1
gene:CHC_T00002896001 transcript:CDF34531
gene_biotype:protein_coding transcript_biotype:protein_coding
Chondrus_crispus OG0000003
CDF35900 pep supercontig:ASM35022v2:HG001750:338773:339507:-1
gene:CHC_T00004027001 transcript:CDF35900
gene_biotype:protein_coding transcript_biotype:protein_coding
Chondrus_crispus OG0000003
CDF35902 pep supercontig:ASM35022v2:HG001750:463383:464767:1
gene:CHC_T00004029001 transcript:CDF35902
gene_biotype:protein_coding transcript_biotype:protein_coding
Chondrus_crispus OG0000003
CDF36102 pep supercontig:ASM35022v2:HG001760:50313:52406:-1
gene:CHC_T00004214001 transcript:CDF36102
gene_biotype:protein_coding transcript_biotype:protein_coding
Chondrus_crispus OG0000003
CDF36118 pep supercontig:ASM35022v2:HG001760:483442:484176:-1
gene:CHC_T00004227001 transcript:CDF36118
gene_biotype:protein_coding transcript_biotype:protein_coding
Chondrus_crispus OG0000003
CDF36119 pep supercontig:ASM35022v2:HG001760:524912:527352:1
gene:CHC_T00004228001 transcript:CDF36119
gene_biotype:protein_coding transcript_biotype:protein_coding
Chondrus_crispus OG0000003
CDF35678 pep supercontig:ASM35022v2:HG001740:64477:66257:1
gene:CHC_T00004196001 transcript:CDF35678
gene_biotype:protein_coding transcript_biotype:protein_coding
Chondrus_crispus OG0000003
CDF37816 pep supercontig:ASM35022v2:HG001871:451945:454159:1
gene:CHC_T00005743001 transcript:CDF37816
gene_biotype:protein_coding transcript_biotype:protein_coding
Chondrus_crispus OG0000003
CDF38072 pep supercontig:ASM35022v2:HG001897:94545:96047:-1

gene:CHC_T00000499001 transcript:CDF38072
gene_biotype:protein_coding transcript_biotype:protein_coding
Chondrus_crispus OG0000003
CDF37815 pep supercontig:ASM35022v2:HG001871:436437:438479:-1
gene:CHC_T00005742001 transcript:CDF37815
gene_biotype:protein_coding transcript_biotype:protein_coding
Chondrus_crispus OG0000003
CDF37845 pep supercontig:ASM35022v2:HG001878:49124:51037:1
gene:CHC_T00000007001 transcript:CDF37845
gene_biotype:protein_coding transcript_biotype:protein_coding
Chondrus_crispus OG0000003
CDF40005 pep supercontig:ASM35022v2:HG002103:268442:269275:1
gene:CHC_T00006955001 transcript:CDF40005
gene_biotype:protein_coding transcript_biotype:protein_coding
Chondrus_crispus OG0000003
CDF40188 pep supercontig:ASM35022v2:HG002137:24123:24785:1
gene:CHC_T00000680001 transcript:CDF40188
gene_biotype:protein_coding transcript_biotype:protein_coding
Chondrus_crispus OG0000003
g1208.t1 Caulerpa_lentillifera OG0000004
g1498.t1 Caulerpa_lentillifera OG0000004
g1953.t1 Caulerpa_lentillifera OG0000004
g2528.t1 Caulerpa_lentillifera OG0000004
g2952.t1 Caulerpa_lentillifera OG0000004
g3600.t1 Caulerpa_lentillifera OG0000004
g4068.t1 Caulerpa_lentillifera OG0000004
g4266.t1 Caulerpa_lentillifera OG0000004
g4569.t1 Caulerpa_lentillifera OG0000004
g6732.t1 Caulerpa_lentillifera OG0000004
g6754.t1 Caulerpa_lentillifera OG0000004
g6906.t1 Caulerpa_lentillifera OG0000004
g6918.t1 Caulerpa_lentillifera OG0000004
g6919.t1 Caulerpa_lentillifera OG0000004
g8282.t1 Caulerpa_lentillifera OG0000004
g856.t1 Caulerpa_lentillifera OG0000004
g96.t1 Caulerpa_lentillifera OG0000004
Cre02.g085701.t1.1 pacid=30785514 transcript=Cre02.g085701.t1.1
locus=Cre02.g085701 ID=Cre02.g085701.t1.1.v5.5 annot-version=v5.5
Chlamydomonas_reinhardtii OG0000004
Cre08.g371400.t1.1 pacid=30773476 transcript=Cre08.g371400.t1.1
locus=Cre08.g371400 ID=Cre08.g371400.t1.1.v5.5 annot-version=v5.5
Chlamydomonas_reinhardtii OG0000004
Cre01.g051700.t1.1 pacid=30788781 transcript=Cre01.g051700.t1.1
locus=Cre01.g051700 ID=Cre01.g051700.t1.1.v5.5 annot-version=v5.5
Chlamydomonas_reinhardtii OG0000004
Cre10.g422050.t1.2 pacid=30790299 transcript=Cre10.g422050.t1.2
locus=Cre10.g422050 ID=Cre10.g422050.t1.2.v5.5 annot-version=v5.5
Chlamydomonas_reinhardtii OG0000004
Cre10.g442750.t1.2 pacid=30790553 transcript=Cre10.g442750.t1.2
locus=Cre10.g442750 ID=Cre10.g442750.t1.2.v5.5 annot-version=v5.5
Chlamydomonas_reinhardtii OG0000004
Cre10.g442450.t1.2 pacid=30790741 transcript=Cre10.g442450.t1.2
locus=Cre10.g442450 ID=Cre10.g442450.t1.2.v5.5 annot-version=v5.5
Chlamydomonas_reinhardtii OG0000004

Cre07.g350867.t1.1 pacid=30775157 transcript=Cre07.g350867.t1.1 locus=Cre07.g350867 ID=Cre07.g350867.t1.1.v5.5 annot-version=v5.5
Chlamydomonas_reinhardtii OG0000004
Cre12.g544327.t2.1 pacid=30791688 transcript=Cre12.g544327.t2.1 locus=Cre12.g544327 ID=Cre12.g544327.t2.1.v5.5 annot-version=v5.5
Chlamydomonas_reinhardtii OG0000004
Cre12.g501450.t1.2 pacid=30793221 transcript=Cre12.g501450.t1.2 locus=Cre12.g501450 ID=Cre12.g501450.t1.2.v5.5 annot-version=v5.5
Chlamydomonas_reinhardtii OG0000004
Cre11.g476250.t1.2 pacid=30775659 transcript=Cre11.g476250.t1.2 locus=Cre11.g476250 ID=Cre11.g476250.t1.2.v5.5 annot-version=v5.5
Chlamydomonas_reinhardtii OG0000004
Cre16.g651400.t1.2 pacid=30776843 transcript=Cre16.g651400.t1.2 locus=Cre16.g651400 ID=Cre16.g651400.t1.2.v5.5 annot-version=v5.5
Chlamydomonas_reinhardtii OG0000004
Cre16.g680117.t1.1 pacid=30776894 transcript=Cre16.g680117.t1.1 locus=Cre16.g680117 ID=Cre16.g680117.t1.1.v5.5 annot-version=v5.5
Chlamydomonas_reinhardtii OG0000004
Cre16.g649833.t1.1 pacid=30777038 transcript=Cre16.g649833.t1.1 locus=Cre16.g649833 ID=Cre16.g649833.t1.1.v5.5 annot-version=v5.5
Chlamydomonas_reinhardtii OG0000004
Cre06.g288450.t1.2 pacid=30778465 transcript=Cre06.g288450.t1.2 locus=Cre06.g288450 ID=Cre06.g288450.t1.2.v5.5 annot-version=v5.5
Chlamydomonas_reinhardtii OG0000004
Cre13.g580750.t1.1 pacid=30784244 transcript=Cre13.g580750.t1.1 locus=Cre13.g580750 ID=Cre13.g580750.t1.1.v5.5 annot-version=v5.5
Chlamydomonas_reinhardtii OG0000004
Vocar.0011s0053.1.p pacid=32895230 transcript=Vocar.0011s0053.1 locus=Vocar.0011s0053 ID=Vocar.0011s0053.1.v2.1 annot-version=v2.1
Volvox_carteri OG0000004
Vocar.0001s0082.1.p pacid=32884580 transcript=Vocar.0001s0082.1 locus=Vocar.0001s0082 ID=Vocar.0001s0082.1.v2.1 annot-version=v2.1
Volvox_carteri OG0000004
Vocar.0001s0398.1.p pacid=32884606 transcript=Vocar.0001s0398.1 locus=Vocar.0001s0398 ID=Vocar.0001s0398.1.v2.1 annot-version=v2.1
Volvox_carteri OG0000004
Vocar.0081s0005.1.p pacid=32897469 transcript=Vocar.0081s0005.1 locus=Vocar.0081s0005 ID=Vocar.0081s0005.1.v2.1 annot-version=v2.1
Volvox_carteri OG0000004
Vocar.0001s1405.1.p pacid=32885571 transcript=Vocar.0001s1405.1 locus=Vocar.0001s1405 ID=Vocar.0001s1405.1.v2.1 annot-version=v2.1
Volvox_carteri OG0000004
Vocar.0001s1218.1.p pacid=32885771 transcript=Vocar.0001s1218.1 locus=Vocar.0001s1218 ID=Vocar.0001s1218.1.v2.1 annot-version=v2.1
Volvox_carteri OG0000004
Vocar.0027s0070.1.p pacid=32887136 transcript=Vocar.0027s0070.1 locus=Vocar.0027s0070 ID=Vocar.0027s0070.1.v2.1 annot-version=v2.1
Volvox_carteri OG0000004
Vocar.0007s0009.1.p pacid=32888037 transcript=Vocar.0007s0009.1 locus=Vocar.0007s0009 ID=Vocar.0007s0009.1.v2.1 annot-version=v2.1
Volvox_carteri OG0000004
Vocar.0004s0407.1.p pacid=32889875 transcript=Vocar.0004s0407.1 locus=Vocar.0004s0407 ID=Vocar.0004s0407.1.v2.1 annot-version=v2.1
Volvox_carteri OG0000004

Vocar.0058s0022.1.p pacid=32890224 transcript=Vocar.0058s0022.1 locus=Vocar.0058s0022 ID=Vocar.0058s0022.1.v2.1 annot-version=v2.1 Volvox_carteri OG0000004
Vocar.0009s0104.1.p pacid=32893138 transcript=Vocar.0009s0104.1 locus=Vocar.0009s0104 ID=Vocar.0009s0104.1.v2.1 annot-version=v2.1 Volvox_carteri OG0000004
Vocar.0032s0086.1.p pacid=32893795 transcript=Vocar.0032s0086.1 locus=Vocar.0032s0086 ID=Vocar.0032s0086.1.v2.1 annot-version=v2.1 Volvox_carteri OG0000004
Vocar.0032s0095.1.p pacid=32893807 transcript=Vocar.0032s0095.1 locus=Vocar.0032s0095 ID=Vocar.0032s0095.1.v2.1 annot-version=v2.1 Volvox_carteri OG0000004
Vocar.0006s0216.1.p pacid=32894307 transcript=Vocar.0006s0216.1 locus=Vocar.0006s0216 ID=Vocar.0006s0216.1.v2.1 annot-version=v2.1 Volvox_carteri OG0000004
jgi|ChlNC64A_1|18021|gw1.10.381.1 Chlorella_variabilis OG0000004
jgi|ChlNC64A_1|137622|IGS.gm_2_00089 Chlorella_variabilis OG0000004
jgi|ChlNC64A_1|15464|gw1.2.508.1 Chlorella_variabilis OG0000004
jgi|ChlNC64A_1|15680|gw1.2.515.1 Chlorella_variabilis OG0000004
jgi|ChlNC64A_1|135820|IGS.gm_14_00353 Chlorella_variabilis OG0000004
jgi|ChlNC64A_1|143853|IGS.gm_6_00427 Chlorella_variabilis OG0000004
jgi|ChlNC64A_1|51582|fgenesh3_pg.C_scaffold_7000129 Chlorella_variabilis OG0000004
jgi|ChlNC64A_1|133669|IGS.gm_1_00805 Chlorella_variabilis OG0000004
jgi|ChlNC64A_1|144831|IGS.gm_8_00159 Chlorella_variabilis OG0000004
jgi|ChlNC64A_1|136184|IGS.gm_15_00280 Chlorella_variabilis OG0000004
jgi|ChlNC64A_1|55921|fgenesh3_pg.C_scaffold_788000001 Chlorella_variabilis OG0000004
65365 pacid=27387290 transcript=65365 locus=estExt_Genemark1.C_50077 ID=65365.2.0.227 annot-version=v2.0 Coccomyxa_subellipsoidea OG0000004
25926 pacid=27388364 transcript=25926 locus=estExt_Genewise1.C_190294 ID=25926.2.0.227 annot-version=v2.0 Coccomyxa_subellipsoidea OG0000004
35426 pacid=27390527 transcript=35426 locus=fgenesh1_pm.3_#_101 ID=35426.2.0.227 annot-version=v2.0 Coccomyxa_subellipsoidea OG0000004
40298 pacid=27390709 transcript=40298 locus=fgenesh1_pg.3_#_359 ID=40298.2.0.227 annot-version=v2.0 Coccomyxa_subellipsoidea OG0000004
60820 pacid=27390811 transcript=60820 locus=Genemark1.1631_g ID=60820.2.0.227 annot-version=v2.0 Coccomyxa_subellipsoidea OG0000004
23409 pacid=27391053 transcript=23409 locus=estExt_Genewise1.C_60493 ID=23409.2.0.227 annot-version=v2.0 Coccomyxa_subellipsoidea

OG0000004
62669 pacid=27391441 transcript=62669 locus=Genemark1.3452_g
ID=62669.2.0.227 annot-version=v2.0 Coccomyxa_subellipsoidea
OG0000004
39634 pacid=27393100 transcript=39634 locus=fgenesh1_pg.2_#_246
ID=39634.2.0.227 annot-version=v2.0 Coccomyxa_subellipsoidea
OG0000004
59218 pacid=27393297 transcript=59218 locus=Genemark1.1180_g
ID=59218.2.0.227 annot-version=v2.0 Coccomyxa_subellipsoidea
OG0000004
59336 pacid=27393737 transcript=59336 locus=Genemark1.1298_g
ID=59336.2.0.227 annot-version=v2.0 Coccomyxa_subellipsoidea
OG0000004
58012 pacid=27393905 transcript=58012 locus=Genemark1.7041_g
ID=58012.2.0.227 annot-version=v2.0 Coccomyxa_subellipsoidea
OG0000004
10773_fgenesh1_pg.C_Chr_04.0001000284 Ostreococcus_tauri
OG0000004
16814_e_gw1.04.00.539.1 Ostreococcus_tauri OG0000004
30007_0000940002 Ostreococcus_tauri OG0000004
33582_0600010429 Ostreococcus_tauri OG0000004
35127_1000010147 Ostreococcus_tauri OG0000004
35225_1000010245 Ostreococcus_tauri OG0000004
35304_1000010324 Ostreococcus_tauri OG0000004
36236_1300010208 Ostreococcus_tauri OG0000004
36623_1400010274 Ostreococcus_tauri OG0000004
29739 pacid=27415180 transcript=29739 locus=eugene.2100010113
ID=29739.2.0.231 annot-version=v2.0 Ostreococcus_lucimarinus
OG0000004
9668 pacid=27415538 transcript=9668 locus=gwEuk.4.648.1
ID=9668.2.0.231 annot-version=v2.0 Ostreococcus_lucimarinus
OG0000004
31341 pacid=27415597 transcript=31341 locus=eugene.0400010417
ID=31341.2.0.231 annot-version=v2.0 Ostreococcus_lucimarinus
OG0000004
28002 pacid=27415985 transcript=28002 locus=eugene.1600010058
ID=28002.2.0.231 annot-version=v2.0 Ostreococcus_lucimarinus
OG0000004
26278 pacid=27416162 transcript=26278 locus=eugene.1000010312
ID=26278.2.0.231 annot-version=v2.0 Ostreococcus_lucimarinus
OG0000004
38024 pacid=27416375 transcript=38024 locus=e_gwEuk.10.493.1
ID=38024.2.0.231 annot-version=v2.0 Ostreococcus_lucimarinus
OG0000004
26734 pacid=27416911 transcript=26734 locus=eugene.1200010079
ID=26734.2.0.231 annot-version=v2.0 Ostreococcus_lucimarinus
OG0000004
28766 pacid=27417578 transcript=28766 locus=eugene.0100010160
ID=28766.2.0.231 annot-version=v2.0 Ostreococcus_lucimarinus
OG0000004
32341 pacid=27418550 transcript=32341 locus=eugene.0600010400
ID=32341.2.0.231 annot-version=v2.0 Ostreococcus_lucimarinus
OG0000004
27170 pacid=27418730 transcript=27170 locus=eugene.1300010192

ID=27170.2.0.231 annot-version=v2.0	Ostreococcus_lucimarinus
OG0000004
29546 pacid=27420121 transcript=29546 locus=eugene.2000010252
ID=29546.2.0.231 annot-version=v2.0	Ostreococcus_lucimarinus
OG0000004
194508 pacid=27338766 transcript=194548
locus=estExt_Genemark1.C_8_t20010 ID=194548.3.0.228 annot-
version=v3.0	Micromonas_pusilla	OG0000004
60097 pacid=27338838 transcript=60097 locus=MicpuC2.EuGene.
0000080291 ID=60097.3.0.228 annot-version=v3.0
Micromonas_pusilla	OG0000004
157499 pacid=27340842 transcript=157539 locus=e_gw1.7.5.1
ID=157539.3.0.228 annot-version=v3.0	Micromonas_pusilla
OG0000004
36262 pacid=27342095 transcript=36262
locus=MicpuC2.estExt_Genewise1Plus.C_140023 ID=36262.3.0.228 annot-
version=v3.0	Micromonas_pusilla	OG0000004
55314 pacid=27342707 transcript=55314 locus=MicpuC2.EuGene.
0000020343 ID=55314.3.0.228 annot-version=v3.0
Micromonas_pusilla	OG0000004
53805 pacid=27343534 transcript=53805 locus=MicpuC2.EuGene.
0000160081 ID=53805.3.0.228 annot-version=v3.0
Micromonas_pusilla	OG0000004
139203 pacid=27344801 transcript=139243 locus=gw1.17.258.1
ID=139243.3.0.228 annot-version=v3.0	Micromonas_pusilla
OG0000004
183490 pacid=27345835 transcript=183530 locus=gm1.611_g
ID=183530.3.0.228 annot-version=v3.0	Micromonas_pusilla
OG0000004
50363 pacid=27345975 transcript=50363 locus=MicpuC2.EuGene.
0000010587 ID=50363.3.0.228 annot-version=v3.0
Micromonas_pusilla	OG0000004
57582 pacid=27346755 transcript=57582 locus=MicpuC2.EuGene.
0000040708 ID=57582.3.0.228 annot-version=v3.0
Micromonas_pusilla	OG0000004
125101 pacid=27347207 transcript=125141
locus=estExt_fgenesh3_kg.C_30036 ID=125141.3.0.228 annot-
version=v3.0	Micromonas_pusilla	OG0000004
9885 pacid=27339523 transcript=9885 locus=MicpuC2.gw1.11.434.1
ID=9885.3.0.228 annot-version=v3.0	Micromonas_pusilla
OG0000004
9869 pacid=27347821 transcript=9869 locus=MicpuC2.gw1.6.546.1
ID=9869.3.0.228 annot-version=v3.0	Micromonas_pusilla
OG0000004
74929 pacid=27397524 transcript=74929 locus=gw2.10.607.1
ID=74929.3.0.229 annot-version=v3.0	Micromonas_sp._RCC299
OG0000004
57350 pacid=27398163 transcript=57350 locus=EuGene.0300010778
ID=57350.3.0.229 annot-version=v3.0	Micromonas_sp._RCC299
OG0000004
105261 pacid=27398339 transcript=105261
locus=estExt_fgenesh2_pg.C_Chr_030158 ID=105261.3.0.229 annot-
version=v3.0	Micromonas_sp._RCC299	OG0000004
108141 pacid=27400436 transcript=108141

locus=est_cluster_kg.Chr_04__119__3201493:1 ID=108141.3.0.229 annot-version=v3.0	Micromonas_sp._RCC299	OG0000004
61535 pacid=27401227 transcript=61535 locus=EuGene.0900010518 ID=61535.3.0.229 annot-version=v3.0	Micromonas_sp._RCC299	OG0000004
101076 pacid=27402408 transcript=101076 locus=fgenesh2_pg.C_Chr_06000277 ID=101076.3.0.229 annot-version=v3.0	Micromonas_sp._RCC299	OG0000004
109335 pacid=27402848 transcript=109335 locus=est_cluster_kg.Chr_13__18__3201321:1 ID=109335.3.0.229 annot-version=v3.0	Micromonas_sp._RCC299	OG0000004
60573 pacid=27403259 transcript=60573 locus=EuGene.0800010141 ID=60573.3.0.229 annot-version=v3.0	Micromonas_sp._RCC299	OG0000004
56207 pacid=27396137 transcript=56207 locus=EuGene.0200010584 ID=56207.3.0.229 annot-version=v3.0	Micromonas_sp._RCC299	OG0000004
50956 pacid=27404170 transcript=50956 locus=est_orfs.Chr_07_827_3202687:1 ID=50956.3.0.229 annot-version=v3.0	Micromonas_sp._RCC299	OG0000004
101390 pacid=27404357 transcript=101390 locus=fgenesh2_pg.C_Chr_07000134 ID=101390.3.0.229 annot-version=v3.0	Micromonas_sp._RCC299	OG0000004
62527 pacid=27404788 transcript=62527 locus=EuGene.1100010313 ID=62527.3.0.229 annot-version=v3.0	Micromonas_sp._RCC299	OG0000004
62302 pacid=27405094 transcript=62302 locus=EuGene.1100010088 ID=62302.3.0.229 annot-version=v3.0	Micromonas_sp._RCC299	OG0000004
62567 pacid=27405319 transcript=62567 locus=EuGene.1100010353 ID=62567.3.0.229 annot-version=v3.0	Micromonas_sp._RCC299	OG0000004
kfl00453_g5_v1.1 Klebsormidium_flaccidum	OG0000004
kfl00482_0050_v1.1	Klebsormidium_flaccidum	OG0000004
kfl00556_0030_v1.1	Klebsormidium_flaccidum	OG0000004
kfl00775_0030_v1.1	Klebsormidium_flaccidum	OG0000004
kfl00843_0040_v1.1	Klebsormidium_flaccidum	OG0000004
kfl00027_0540_v1.1	Klebsormidium_flaccidum	OG0000004
kfl00003_0180_v1.1	Klebsormidium_flaccidum	OG0000004
kfl00003_0200_v1.1	Klebsormidium_flaccidum	OG0000004
kfl00034_0310_v1.1	Klebsormidium_flaccidum	OG0000004
kfl00037_0250_v1.1	Klebsormidium_flaccidum	OG0000004
kfl00066_0030_v1.1	Klebsormidium_flaccidum	OG0000004
kfl00079_0070_v1.1	Klebsormidium_flaccidum	OG0000004
kfl00113_0130_v1.1	Klebsormidium_flaccidum	OG0000004
kfl00141_0100_v1.1	Klebsormidium_flaccidum	OG0000004
kfl00154_0250_v1.1	Klebsormidium_flaccidum	OG0000004
kfl00158_0180_v1.1	Klebsormidium_flaccidum	OG0000004
kfl00166_0070_v1.1	Klebsormidium_flaccidum	OG0000004
kfl00190_0050_v1.1	Klebsormidium_flaccidum	OG0000004
kfl00212_0070_v1.1	Klebsormidium_flaccidum	OG0000004
kfl00237_0130_v1.1	Klebsormidium_flaccidum	OG0000004
kfl00255_0100_v1.1	Klebsormidium_flaccidum	OG0000004
kfl00326_0120_v1.1	Klebsormidium_flaccidum	OG0000004

Pp3c15_2690V3.1.p pacid=32929548 transcript=Pp3c15_2690V3.1
locus=Pp3c15_2690 annot-version=v3.3   Physcomitrella_patens
OG0000004
Pp3c13_4710V3.1.p pacid=32930075 transcript=Pp3c13_4710V3.1
locus=Pp3c13_4710 annot-version=v3.3   Physcomitrella_patens
OG0000004
Pp3c13_19370V3.1.p pacid=32931234 transcript=Pp3c13_19370V3.1
locus=Pp3c13_19370 annot-version=v3.3   Physcomitrella_patens
OG0000004
Pp3c13_21890V3.1.p pacid=32931735 transcript=Pp3c13_21890V3.1
locus=Pp3c13_21890 annot-version=v3.3   Physcomitrella_patens
OG0000004
Pp3c2_17560V3.1.p pacid=32934608 transcript=Pp3c2_17560V3.1
locus=Pp3c2_17560 annot-version=v3.3   Physcomitrella_patens
OG0000004
Pp3c2_5960V3.1.p pacid=32934735 transcript=Pp3c2_5960V3.1
locus=Pp3c2_5960 annot-version=v3.3   Physcomitrella_patens
OG0000004
Pp3c2_28540V3.1.p pacid=32935539 transcript=Pp3c2_28540V3.1
locus=Pp3c2_28540 annot-version=v3.3   Physcomitrella_patens
OG0000004
Pp3c19_21510V3.1.p pacid=32938029 transcript=Pp3c19_21510V3.1
locus=Pp3c19_21510 annot-version=v3.3   Physcomitrella_patens
OG0000004
Pp3c19_14050V3.1.p pacid=32939353 transcript=Pp3c19_14050V3.1
locus=Pp3c19_14050 annot-version=v3.3   Physcomitrella_patens
OG0000004
Pp3c19_14040V3.1.p pacid=32939534 transcript=Pp3c19_14040V3.1
locus=Pp3c19_14040 annot-version=v3.3   Physcomitrella_patens
OG0000004
Pp3c3_13100V3.1.p pacid=32941589 transcript=Pp3c3_13100V3.1
locus=Pp3c3_13100 annot-version=v3.3   Physcomitrella_patens
OG0000004
Pp3c22_1990V3.1.p pacid=32903751 transcript=Pp3c22_1990V3.1
locus=Pp3c22_1990 annot-version=v3.3   Physcomitrella_patens
OG0000004
Pp3c3_10570V3.1.p pacid=32942518 transcript=Pp3c3_10570V3.1
locus=Pp3c3_10570 annot-version=v3.3   Physcomitrella_patens
OG0000004
Pp3c22_15530V3.1.p pacid=32904107 transcript=Pp3c22_15530V3.1
locus=Pp3c22_15530 annot-version=v3.3   Physcomitrella_patens
OG0000004
Pp3c20_11600V3.1.p pacid=32946537 transcript=Pp3c20_11600V3.1
locus=Pp3c20_11600 annot-version=v3.3   Physcomitrella_patens
OG0000004
Pp3c20_12240V3.1.p pacid=32946599 transcript=Pp3c20_12240V3.1
locus=Pp3c20_12240 annot-version=v3.3   Physcomitrella_patens
OG0000004
Pp3c23_21240V3.1.p pacid=32949016 transcript=Pp3c23_21240V3.1
locus=Pp3c23_21240 annot-version=v3.3   Physcomitrella_patens
OG0000004
Pp3c23_1651V3.1.p pacid=32950122 transcript=Pp3c23_1651V3.1
locus=Pp3c23_1651 annot-version=v3.3   Physcomitrella_patens
OG0000004

Pp3c23_10580V3.1.p pacid=32950708 transcript=Pp3c23_10580V3.1 locus=Pp3c23_10580 annot-version=v3.3 Physcomitrella_patens OG0000004
Pp3c5_4170V3.1.p pacid=32952777 transcript=Pp3c5_4170V3.1 locus=Pp3c5_4170 annot-version=v3.3 Physcomitrella_patens OG0000004
Pp3c22_17730V3.1.p pacid=32904926 transcript=Pp3c22_17730V3.1 locus=Pp3c22_17730 annot-version=v3.3 Physcomitrella_patens OG0000004
Pp3c5_17300V3.1.p pacid=32953776 transcript=Pp3c5_17300V3.1 locus=Pp3c5_17300 annot-version=v3.3 Physcomitrella_patens OG0000004
Pp3c11_26010V3.1.p pacid=32956903 transcript=Pp3c11_26010V3.1 locus=Pp3c11_26010 annot-version=v3.3 Physcomitrella_patens OG0000004
Pp3c22_15440V3.1.p pacid=32905317 transcript=Pp3c22_15440V3.1 locus=Pp3c22_15440 annot-version=v3.3 Physcomitrella_patens OG0000004
Pp3c11_13800V3.1.p pacid=32957660 transcript=Pp3c11_13800V3.1 locus=Pp3c11_13800 annot-version=v3.3 Physcomitrella_patens OG0000004
Pp3c10_8120V3.1.p pacid=32899960 transcript=Pp3c10_8120V3.1 locus=Pp3c10_8120 annot-version=v3.3 Physcomitrella_patens OG0000004
Pp3c14_14600V3.1.p pacid=32960566 transcript=Pp3c14_14600V3.1 locus=Pp3c14_14600 annot-version=v3.3 Physcomitrella_patens OG0000004
Pp3c17_13190V3.1.p pacid=32905771 transcript=Pp3c17_13190V3.1 locus=Pp3c17_13190 annot-version=v3.3 Physcomitrella_patens OG0000004
Pp3c14_25422V3.1.p pacid=32962914 transcript=Pp3c14_25422V3.1 locus=Pp3c14_25422 annot-version=v3.3 Physcomitrella_patens OG0000004
Pp3c1_170V3.1.p pacid=32967228 transcript=Pp3c1_170V3.1 locus=Pp3c1_170 annot-version=v3.3 Physcomitrella_patens OG0000004
Pp3c1_32230V3.1.p pacid=32967866 transcript=Pp3c1_32230V3.1 locus=Pp3c1_32230 annot-version=v3.3 Physcomitrella_patens OG0000004
Pp3c1_9560V3.1.p pacid=32968475 transcript=Pp3c1_9560V3.1 locus=Pp3c1_9560 annot-version=v3.3 Physcomitrella_patens OG0000004
Pp3c1_42450V3.1.p pacid=32968653 transcript=Pp3c1_42450V3.1 locus=Pp3c1_42450 annot-version=v3.3 Physcomitrella_patens OG0000004
Pp3c1_5780V3.1.p pacid=32969467 transcript=Pp3c1_5780V3.1 locus=Pp3c1_5780 annot-version=v3.3 Physcomitrella_patens OG0000004
Pp3c1_20780V3.1.p pacid=32971249 transcript=Pp3c1_20780V3.1 locus=Pp3c1_20780 annot-version=v3.3 Physcomitrella_patens OG0000004
Pp3c1_34410V3.1.p pacid=32971445 transcript=Pp3c1_34410V3.1 locus=Pp3c1_34410 annot-version=v3.3 Physcomitrella_patens OG0000004

Pp3c12_7720V3.1.p pacid=32972656 transcript=Pp3c12_7720V3.1 locus=Pp3c12_7720 annot-version=v3.3 Physcomitrella_patens OG0000004
Pp3c6_7790V3.1.p pacid=32975290 transcript=Pp3c6_7790V3.1 locus=Pp3c6_7790 annot-version=v3.3 Physcomitrella_patens OG0000004
Pp3c6_19030V3.1.p pacid=32975572 transcript=Pp3c6_19030V3.1 locus=Pp3c6_19030 annot-version=v3.3 Physcomitrella_patens OG0000004
Pp3c6_21080V3.1.p pacid=32978503 transcript=Pp3c6_21080V3.1 locus=Pp3c6_21080 annot-version=v3.3 Physcomitrella_patens OG0000004
Pp3c25_6520V3.1.p pacid=32979042 transcript=Pp3c25_6520V3.1 locus=Pp3c25_6520 annot-version=v3.3 Physcomitrella_patens OG0000004
Pp3c25_15200V3.1.p pacid=32979964 transcript=Pp3c25_15200V3.1 locus=Pp3c25_15200 annot-version=v3.3 Physcomitrella_patens OG0000004
Pp3c25_7040V3.1.p pacid=32980417 transcript=Pp3c25_7040V3.1 locus=Pp3c25_7040 annot-version=v3.3 Physcomitrella_patens OG0000004
Pp3s52_10V3.1.p pacid=32980906 transcript=Pp3s52_10V3.1 locus=Pp3s52_10 annot-version=v3.3 Physcomitrella_patens OG0000004
Pp3c18_5860V3.1.p pacid=32981732 transcript=Pp3c18_5860V3.1 locus=Pp3c18_5860 annot-version=v3.3 Physcomitrella_patens OG0000004
Pp3c18_7090V3.1.p pacid=32982116 transcript=Pp3c18_7090V3.1 locus=Pp3c18_7090 annot-version=v3.3 Physcomitrella_patens OG0000004
Pp3c18_7100V3.1.p pacid=32982558 transcript=Pp3c18_7100V3.1 locus=Pp3c18_7100 annot-version=v3.3 Physcomitrella_patens OG0000004
Pp3c16_18860V3.1.p pacid=32984201 transcript=Pp3c16_18860V3.1 locus=Pp3c16_18860 annot-version=v3.3 Physcomitrella_patens OG0000004
Pp3c16_18430V3.1.p pacid=32985039 transcript=Pp3c16_18430V3.1 locus=Pp3c16_18430 annot-version=v3.3 Physcomitrella_patens OG0000004
Pp3c24_11700V3.1.p pacid=32910497 transcript=Pp3c24_11700V3.1 locus=Pp3c24_11700 annot-version=v3.3 Physcomitrella_patens OG0000004
Pp3c24_7770V3.1.p pacid=32910767 transcript=Pp3c24_7770V3.1 locus=Pp3c24_7770 annot-version=v3.3 Physcomitrella_patens OG0000004
Pp3c9_3390V3.1.p pacid=32913020 transcript=Pp3c9_3390V3.1 locus=Pp3c9_3390 annot-version=v3.3 Physcomitrella_patens OG0000004
Pp3c9_2330V3.1.p pacid=32913565 transcript=Pp3c9_2330V3.1 locus=Pp3c9_2330 annot-version=v3.3 Physcomitrella_patens OG0000004
Pp3c21_14400V3.1.p pacid=32916055 transcript=Pp3c21_14400V3.1 locus=Pp3c21_14400 annot-version=v3.3 Physcomitrella_patens OG0000004

Pp3c21_11960V3.1.p pacid=32916700 transcript=Pp3c21_11960V3.1 locus=Pp3c21_11960 annot-version=v3.3 Physcomitrella_patens OG0000004
Pp3c21_17280V3.1.p pacid=32916709 transcript=Pp3c21_17280V3.1 locus=Pp3c21_17280 annot-version=v3.3 Physcomitrella_patens OG0000004
Pp3c26_7240V3.1.p pacid=32917110 transcript=Pp3c26_7240V3.1 locus=Pp3c26_7240 annot-version=v3.3 Physcomitrella_patens OG0000004
Pp3c26_11650V3.1.p pacid=32917258 transcript=Pp3c26_11650V3.1 locus=Pp3c26_11650 annot-version=v3.3 Physcomitrella_patens OG0000004
Pp3c4_30240V3.1.p pacid=32922541 transcript=Pp3c4_30240V3.1 locus=Pp3c4_30240 annot-version=v3.3 Physcomitrella_patens OG0000004
Pp3c4_17150V3.1.p pacid=32922805 transcript=Pp3c4_17150V3.1 locus=Pp3c4_17150 annot-version=v3.3 Physcomitrella_patens OG0000004
Pp3c7_560V3.1.p pacid=32923348 transcript=Pp3c7_560V3.1 locus=Pp3c7_560 annot-version=v3.3 Physcomitrella_patens OG0000004
AT2G34990.1 | RING/U-box superfamily protein | Chr2:14750260-14751168 REVERSE LENGTH=302 | 201606 Arabidopsis_thaliana OG0000004
AT2G35000.1 | RING/U-box superfamily protein | Chr2:14751809-14752945 REVERSE LENGTH=378 | 201606 Arabidopsis_thaliana OG0000004
AT2G35420.1 | RING/U-box superfamily protein | Chr2:14899715-14900479 REVERSE LENGTH=254 | 201606 Arabidopsis_thaliana OG0000004
AT2G35910.1 | RING/U-box superfamily protein | Chr2:15073225-15073878 REVERSE LENGTH=217 | 201606 Arabidopsis_thaliana OG0000004
AT2G37150.1 | RING/U-box superfamily protein | Chr2:15603748-15605988 REVERSE LENGTH=546 | 201606 Arabidopsis_thaliana OG0000004
AT2G37580.1 | RING/U-box superfamily protein | Chr2:15764745-15765452 FORWARD LENGTH=235 | 201606 Arabidopsis_thaliana OG0000004
AT2G39100.1 | RING/U-box superfamily protein | Chr2:16317462-16318869 FORWARD LENGTH=296 | 201606 Arabidopsis_thaliana OG0000004
AT2G39720.1 | RING-H2 finger C2A | Chr2:16567506-16568711 REVERSE LENGTH=401 | 201606 Arabidopsis_thaliana OG0000004
AT2G40830.1 | RING-H2 finger C1A | Chr2:17043642-17044628 FORWARD LENGTH=328 | 201606 Arabidopsis_thaliana OG0000004
AT2G42350.1 | RING/U-box superfamily protein | Chr2:17639245-17639898 FORWARD LENGTH=217 | 201606 Arabidopsis_thaliana OG0000004
AT2G42360.1 | RING/U-box superfamily protein | Chr2:17640907-17641617 FORWARD LENGTH=236 | 201606 Arabidopsis_thaliana OG0000004
AT2G44330.1 | RING/U-box superfamily protein | Chr2:18310621-18311163 FORWARD LENGTH=180 | 201606

Arabidopsis_thaliana   OG0000004
AT2G44578.1 | RING/U-box superfamily protein | Chr2:18400864-18401301 REVERSE LENGTH=145 | 201606
Arabidopsis_thaliana   OG0000004
AT2G44581.1 | RING/U-box superfamily protein | Chr2:18397996-18398433 REVERSE LENGTH=145 | 201606
Arabidopsis_thaliana   OG0000004
AT2G46160.1 | RING/U-box superfamily protein | Chr2:18963109-18963753 FORWARD LENGTH=214 | 201606
Arabidopsis_thaliana   OG0000004
AT2G46493.1 | RING/U-box superfamily protein | Chr2:19080254-19081242 REVERSE LENGTH=184 | 201606
Arabidopsis_thaliana   OG0000004
AT2G46494.1 | RING/U-box superfamily protein | Chr2:19082344-19083811 REVERSE LENGTH=362 | 201606
Arabidopsis_thaliana   OG0000004
AT2G46495.1 | RING/U-box superfamily protein | Chr2:19084134-19085704 REVERSE LENGTH=372 | 201606
Arabidopsis_thaliana   OG0000004
AT2G47560.1 | RING/U-box superfamily protein | Chr2:19511934-19512617 REVERSE LENGTH=227 | 201606
Arabidopsis_thaliana   OG0000004
AT3G02290.1 | RING/U-box superfamily protein | Chr3:459385-460688 FORWARD LENGTH=231 | 201606   Arabidopsis_thaliana
OG0000004
AT3G02340.1 | RING/U-box superfamily protein | Chr3:477032-478261 FORWARD LENGTH=409 | 201606   Arabidopsis_thaliana
OG0000004
AT3G03550.1 | RING/U-box superfamily protein | Chr3:850391-851461 REVERSE LENGTH=356 | 201606   Arabidopsis_thaliana
OG0000004
AT3G05200.1 | RING/U-box superfamily protein | Chr3:1477377-1478573 FORWARD LENGTH=398 | 201606   Arabidopsis_thaliana
OG0000004
AT3G10815.1 | RING/U-box superfamily protein | Chr3:3385009-3385608 REVERSE LENGTH=199 | 201606   Arabidopsis_thaliana
OG0000004
AT3G10910.1 | RING/U-box superfamily protein | Chr3:3413068-3413613 REVERSE LENGTH=181 | 201606   Arabidopsis_thaliana
OG0000004
AT3G11110.1 | RING/U-box superfamily protein | Chr3:3479979-3480455 FORWARD LENGTH=158 | 201606   Arabidopsis_thaliana
OG0000004
AT1G12760.1 | Zinc finger%2C C3HC4 type (RING finger) family protein | Chr1:4348728-4350512 FORWARD LENGTH=408 | 201606
Arabidopsis_thaliana   OG0000004
AT3G13228.1 | RING/U-box superfamily protein | Chr3:4267075-4268052 FORWARD LENGTH=325 | 201606   Arabidopsis_thaliana
OG0000004
AT3G13430.1 | RING/U-box superfamily protein | Chr3:4367754-4368701 FORWARD LENGTH=315 | 201606   Arabidopsis_thaliana
OG0000004
AT3G14320.1 | Zinc finger%2C C3HC4 type (RING finger) family protein | Chr3:4777731-4778345 REVERSE LENGTH=204 | 201606

Arabidopsis_thaliana OG0000004
AT3G14970.1 | RING/U-box superfamily protein | Chr3:5039071-5039733 REVERSE LENGTH=220 | 201606 Arabidopsis_thaliana OG0000004
AT3G15070.1 | RING/U-box superfamily protein | Chr3:5070472-5072344 REVERSE LENGTH=486 | 201606 Arabidopsis_thaliana OG0000004
AT3G15740.1 | RING/U-box superfamily protein | Chr3:5333879-5334553 REVERSE LENGTH=224 | 201606 Arabidopsis_thaliana OG0000004
AT3G16720.1 | TOXICOS EN LEVADURA 2 | Chr3:5692880-5693794 FORWARD LENGTH=304 | 201606 Arabidopsis_thaliana OG0000004
AT3G18773.1 | RING/U-box superfamily protein | Chr3:6466304-6466966 FORWARD LENGTH=220 | 201606 Arabidopsis_thaliana OG0000004
AT3G18930.1 | RING/U-box superfamily protein | Chr3:6524110-6525345 REVERSE LENGTH=411 | 201606 Arabidopsis_thaliana OG0000004
AT3G19140.1 | RING/U-box superfamily protein | Chr3:6614910-6615335 REVERSE LENGTH=141 | 201606 Arabidopsis_thaliana OG0000004
AT3G19910.1 | RING/U-box superfamily protein | Chr3:6926497-6929324 FORWARD LENGTH=340 | 201606 Arabidopsis_thaliana OG0000004
AT3G19950.1 | RING/U-box superfamily protein | Chr3:6942853-6943839 FORWARD LENGTH=328 | 201606 Arabidopsis_thaliana OG0000004
AT3G20395.1 | RING/U-box superfamily protein | Chr3:7112020-7113792 REVERSE LENGTH=223 | 201606 Arabidopsis_thaliana OG0000004
AT1G14200.1 | RING/U-box superfamily protein | Chr1:4854532-4855071 REVERSE LENGTH=179 | 201606 Arabidopsis_thaliana OG0000004
AT3G28620.1 | Zinc finger%2C C3HC4 type (RING finger) family protein | Chr3:10727826-10728461 FORWARD LENGTH=211 | 201606 Arabidopsis_thaliana OG0000004
AT3G30460.1 | RING/U-box superfamily protein | Chr3:12104433-12104876 FORWARD LENGTH=147 | 201606 Arabidopsis_thaliana OG0000004
AT3G43430.1 | RING/U-box superfamily protein | Chr3:15354758-15355261 REVERSE LENGTH=167 | 201606 Arabidopsis_thaliana OG0000004
AT3G46620.1 | zinc finger (C3HC4-type RING finger) family protein | Chr3:17178837-17180024 REVERSE LENGTH=395 | 201606 Arabidopsis_thaliana OG0000004
AT3G47180.1 | RING/U-box superfamily protein | Chr3:17372821-17373453 REVERSE LENGTH=210 | 201606 Arabidopsis_thaliana OG0000004
AT1G15100.1 | RING-H2 finger A2A | Chr1:5193703-5194170 REVERSE LENGTH=155 | 201606 Arabidopsis_thaliana OG0000004
AT3G51325.1 | RING/U-box superfamily protein | Chr3:19052259-19052531 FORWARD LENGTH=90 | 201606 Arabidopsis_thaliana OG0000004
AT3G55530.1 | RING/U-box superfamily protein |

Chr3:20595300-20597188 REVERSE LENGTH=273 | 201606
Arabidopsis_thaliana OG0000004
AT3G56580.1 | RING/U-box superfamily protein |
Chr3:20962563-20963525 FORWARD LENGTH=320 | 201606
Arabidopsis_thaliana OG0000004
AT3G58720.1 | RING/U-box superfamily protein |
Chr3:21716920-21717892 REVERSE LENGTH=238 | 201606
Arabidopsis_thaliana OG0000004
AT3G60080.1 | RING/U-box superfamily protein |
Chr3:22187635-22188555 FORWARD LENGTH=306 | 201606
Arabidopsis_thaliana OG0000004
AT3G60220.1 | TOXICOS EN LEVADURA 4 | Chr3:22254790-22255794 REVERSE
LENGTH=334 | 201606 Arabidopsis_thaliana OG0000004
AT3G60966.1 | RING/U-box superfamily protein |
Chr3:22552718-22553137 FORWARD LENGTH=139 | 201606
Arabidopsis_thaliana OG0000004
AT3G61180.1 | RING/U-box superfamily protein |
Chr3:22645680-22647290 FORWARD LENGTH=379 | 201606
Arabidopsis_thaliana OG0000004
AT3G61460.1 | brassinosteroid-responsive RING-H2 |
Chr3:22741701-22742213 REVERSE LENGTH=170 | 201606
Arabidopsis_thaliana OG0000004
AT3G61550.1 | RING/U-box superfamily protein |
Chr3:22776444-22777082 FORWARD LENGTH=212 | 201606
Arabidopsis_thaliana OG0000004
AT3G62690.1 | AtL5 | Chr3:23185829-23186602 REVERSE LENGTH=257 |
201606 Arabidopsis_thaliana OG0000004
AT3G63530.1 | RING/U-box superfamily protein |
Chr3:23456407-23457787 REVERSE LENGTH=248 | 201606
Arabidopsis_thaliana OG0000004
AT4G00070.1 | RING/U-box superfamily protein | Chr4:29672-31426
REVERSE LENGTH=179 | 201606 Arabidopsis_thaliana
OG0000004
AT4G00305.1 | RING/U-box superfamily protein | Chr4:131550-131930
FORWARD LENGTH=126 | 201606 Arabidopsis_thaliana
OG0000004
AT4G00335.1 | RING-H2 finger B1A | Chr4:146866-147962 FORWARD
LENGTH=190 | 201606 Arabidopsis_thaliana OG0000004
AT4G05350.1 | RING/U-box superfamily protein | Chr4:2726910-2727530
REVERSE LENGTH=206 | 201606 Arabidopsis_thaliana
OG0000004
AT4G09100.1 | RING/U-box superfamily protein | Chr4:5811256-5811654
FORWARD LENGTH=132 | 201606 Arabidopsis_thaliana
OG0000004
AT4G09110.1 | RING/U-box superfamily protein | Chr4:5812488-5813396
FORWARD LENGTH=302 | 201606 Arabidopsis_thaliana
OG0000004
AT4G09120.1 | RING/U-box superfamily protein | Chr4:5813998-5815035
FORWARD LENGTH=345 | 201606 Arabidopsis_thaliana
OG0000004
AT4G09130.1 | RING/U-box superfamily protein | Chr4:5815849-5816922
FORWARD LENGTH=357 | 201606 Arabidopsis_thaliana
OG0000004
AT4G09560.1 | Protease-associated (PA) RING/U-box zinc finger family

protein | Chr4:6041652-6043681 REVERSE LENGTH=448 | 201606
Arabidopsis_thaliana   OG0000004
AT4G10150.1 | RING/U-box superfamily protein | Chr4:6328136-6329558
FORWARD LENGTH=236 | 201606   Arabidopsis_thaliana
OG0000004
AT4G10160.1 | RING/U-box superfamily protein | Chr4:6336023-6337301
FORWARD LENGTH=225 | 201606   Arabidopsis_thaliana
OG0000004
AT4G11360.1 | RING-H2 finger A1B | Chr4:6906066-6906539 FORWARD
LENGTH=157 | 201606   Arabidopsis_thaliana   OG0000004
AT4G11370.1 | RING-H2 finger A1A | Chr4:6907777-6908256 FORWARD
LENGTH=159 | 201606   Arabidopsis_thaliana   OG0000004
AT4G11680.1 | Zinc finger%2C C3HC4 type (RING finger) family protein
| Chr4:7053737-7055516 REVERSE LENGTH=390 | 201606
Arabidopsis_thaliana   OG0000004
AT4G12140.1 | RING/U-box superfamily protein | Chr4:7266546-7267154
REVERSE LENGTH=202 | 201606   Arabidopsis_thaliana
OG0000004
AT4G12150.1 | RING/U-box superfamily protein | Chr4:7268837-7270267
REVERSE LENGTH=357 | 201606   Arabidopsis_thaliana
OG0000004
AT4G12190.1 | RING/U-box superfamily protein | Chr4:7278545-7278760
REVERSE LENGTH=71 | 201606   Arabidopsis_thaliana
OG0000004
AT4G12210.1 | RING/U-box superfamily protein | Chr4:7281308-7281919
REVERSE LENGTH=203 | 201606   Arabidopsis_thaliana
OG0000004
AT1G17970.1 | RING/U-box superfamily protein | Chr1:6185032-6187202
FORWARD LENGTH=368 | 201606   Arabidopsis_thaliana
OG0000004
AT4G14220.1 | RING-H2 group F1A | Chr4:8196012-8198240 FORWARD
LENGTH=371 | 201606   Arabidopsis_thaliana   OG0000004
AT4G15975.1 | RING/U-box superfamily protein | Chr4:9052313-9053020
FORWARD LENGTH=235 | 201606   Arabidopsis_thaliana
OG0000004
AT4G17245.1 | RING/U-box superfamily protein | Chr4:9669383-9669883
FORWARD LENGTH=166 | 201606   Arabidopsis_thaliana
OG0000004
AT4G17905.1 | RING/U-box superfamily protein | Chr4:9948853-9949785
REVERSE LENGTH=310 | 201606   Arabidopsis_thaliana
OG0000004
AT4G17920.1 | RING/U-box superfamily protein | Chr4:9963221-9964090
REVERSE LENGTH=289 | 201606   Arabidopsis_thaliana
OG0000004
AT4G18110.1 | RING/U-box superfamily protein |
Chr4:10037077-10037718 REVERSE LENGTH=213 | 201606
Arabidopsis_thaliana   OG0000004
AT1G18760.1 | Zinc finger%2C C3HC4 type (RING finger) family protein
| Chr1:6471150-6471824 REVERSE LENGTH=224 | 201606
Arabidopsis_thaliana   OG0000004
AT1G18770.1 | RING/U-box superfamily protein | Chr1:6473370-6474048
REVERSE LENGTH=106 | 201606   Arabidopsis_thaliana
OG0000004
AT1G18780.1 | RING/U-box superfamily protein | Chr1:6476258-6477235

REVERSE LENGTH=325 | 201606	Arabidopsis_thaliana
OG0000004
AT4G23450.1 | RING/U-box superfamily protein |
Chr4:12241113-12241837 REVERSE LENGTH=153 | 201606
Arabidopsis_thaliana	OG0000004
AT4G24015.1 | RING/U-box superfamily protein |
Chr4:12469887-12471197 REVERSE LENGTH=174 | 201606
Arabidopsis_thaliana	OG0000004
AT4G26400.1 | RING/U-box superfamily protein |
Chr4:13344953-13346023 REVERSE LENGTH=356 | 201606
Arabidopsis_thaliana	OG0000004
AT4G26580.1 | RING/U-box superfamily protein |
Chr4:13411705-13412889 REVERSE LENGTH=335 | 201606
Arabidopsis_thaliana	OG0000004
AT4G28890.1 | RING/U-box superfamily protein |
Chr4:14256437-14257735 REVERSE LENGTH=432 | 201606
Arabidopsis_thaliana	OG0000004
AT4G30370.1 | RING/U-box superfamily protein |
Chr4:14858743-14859273 REVERSE LENGTH=176 | 201606
Arabidopsis_thaliana	OG0000004
AT4G30400.1 | RING/U-box superfamily protein |
Chr4:14867068-14868486 FORWARD LENGTH=472 | 201606
Arabidopsis_thaliana	OG0000004
AT4G31450.1 | RING/U-box superfamily protein |
Chr4:15255955-15257977 REVERSE LENGTH=497 | 201606
Arabidopsis_thaliana	OG0000004
AT4G32600.1 | C3H4 type zinc finger protein | Chr4:15724010-15725737
FORWARD LENGTH=453 | 201606	Arabidopsis_thaliana
OG0000004
AT4G33565.1 | RING/U-box superfamily protein |
Chr4:16136821-16137924 FORWARD LENGTH=367 | 201606
Arabidopsis_thaliana	OG0000004
AT4G34040.1 | RING/U-box superfamily protein |
Chr4:16304638-16307503 REVERSE LENGTH=666 | 201606
Arabidopsis_thaliana	OG0000004
AT4G35480.1 | RING-H2 finger A3B | Chr4:16852233-16852835 REVERSE
LENGTH=200 | 201606	Arabidopsis_thaliana	OG0000004
AT4G35840.1 | RING/U-box superfamily protein |
Chr4:16981083-16982266 FORWARD LENGTH=236 | 201606
Arabidopsis_thaliana	OG0000004
AT4G38140.1 | RING/U-box superfamily protein |
Chr4:17899868-17900305 REVERSE LENGTH=145 | 201606
Arabidopsis_thaliana	OG0000004
AT4G40070.1 | RING/U-box superfamily protein |
Chr4:18576533-18577504 FORWARD LENGTH=323 | 201606
Arabidopsis_thaliana	OG0000004
AT1G20823.1 | RING/U-box superfamily protein | Chr1:7238880-7239473
FORWARD LENGTH=197 | 201606	Arabidopsis_thaliana
OG0000004
AT5G01880.1 | RING/U-box superfamily protein | Chr5:339017-339496
FORWARD LENGTH=159 | 201606	Arabidopsis_thaliana
OG0000004
AT5G01980.1 | RING/U-box superfamily protein | Chr5:375542-377023
FORWARD LENGTH=493 | 201606	Arabidopsis_thaliana

OG0000004
AT5G02750.1 | RING/U-box superfamily protein | Chr5:620101-620952 FORWARD LENGTH=283 | 201606	Arabidopsis_thaliana	OG0000004
AT5G05280.1 | RING/U-box superfamily protein | Chr5:1565509-1566039 REVERSE LENGTH=176 | 201606	Arabidopsis_thaliana	OG0000004
AT5G05810.1 | RING/U-box superfamily protein | Chr5:1746776-1747999 FORWARD LENGTH=407 | 201606	Arabidopsis_thaliana	OG0000004
AT5G05910.1 | RING/U-box superfamily protein | Chr5:1776858-1777313 FORWARD LENGTH=151 | 201606	Arabidopsis_thaliana	OG0000004
AT5G06490.1 | RING/U-box superfamily protein | Chr5:1977996-1978589 REVERSE LENGTH=197 | 201606	Arabidopsis_thaliana	OG0000004
AT5G07040.1 | RING/U-box superfamily protein | Chr5:2190344-2190823 FORWARD LENGTH=159 | 201606	Arabidopsis_thaliana	OG0000004
AT5G07225.1 | RING/U-box superfamily protein | Chr5:2268642-2270227 REVERSE LENGTH=234 | 201606	Arabidopsis_thaliana	OG0000004
AT5G08139.1 | RING/U-box superfamily protein | Chr5:2616487-2617617 FORWARD LENGTH=376 | 201606	Arabidopsis_thaliana	OG0000004
AT5G10380.1 | RING/U-box superfamily protein | Chr5:3267819-3268724 FORWARD LENGTH=301 | 201606	Arabidopsis_thaliana	OG0000004
AT5G10650.1 | RING/U-box superfamily protein | Chr5:3365237-3367263 REVERSE LENGTH=525 | 201606	Arabidopsis_thaliana	OG0000004
AT1G21960.1 | RING/U-box superfamily protein | Chr1:7725972-7726586 FORWARD LENGTH=204 | 201606	Arabidopsis_thaliana	OG0000004
AT5G15790.1 | RING/U-box superfamily protein | Chr5:5149800-5150964 FORWARD LENGTH=232 | 201606	Arabidopsis_thaliana	OG0000004
AT5G15820.1 | RING/U-box superfamily protein | Chr5:5161787-5162833 FORWARD LENGTH=348 | 201606	Arabidopsis_thaliana	OG0000004
AT5G17600.1 | RING/U-box superfamily protein | Chr5:5800029-5801117 REVERSE LENGTH=362 | 201606	Arabidopsis_thaliana	OG0000004
AT1G22500.1 | RING/U-box superfamily protein | Chr1:7949581-7950726 FORWARD LENGTH=381 | 201606	Arabidopsis_thaliana	OG0000004
AT1G22670.1 | Protease-associated (PA) RING/U-box zinc finger family protein | Chr1:8021854-8023516 REVERSE LENGTH=422 | 201606	Arabidopsis_thaliana	OG0000004
AT5G20885.1 | RING/U-box superfamily protein | Chr5:7084133-7084663 REVERSE LENGTH=176 | 201606	Arabidopsis_thaliana	OG0000004
AT5G20910.1 | RING/U-box superfamily protein | Chr5:7092663-7094310 REVERSE LENGTH=310 | 201606	Arabidopsis_thaliana

OG0000004
AT5G24870.1 | RING/U-box superfamily protein | Chr5:8545008-8546923
REVERSE LENGTH=520 | 201606 Arabidopsis_thaliana
OG0000004
AT5G27420.1 | carbon/nitrogen insensitive 1 | Chr5:9684119-9685225
FORWARD LENGTH=368 | 201606 Arabidopsis_thaliana
OG0000004
AT5G36001.1 | RING/U-box superfamily protein |
Chr5:14142050-14143143 FORWARD LENGTH=322 | 201606
Arabidopsis_thaliana OG0000004
AT5G37200.1 | RING/U-box superfamily protein |
Chr5:14727832-14728485 FORWARD LENGTH=217 | 201606
Arabidopsis_thaliana OG0000004
AT5G37220.1 | RING/U-box superfamily protein |
Chr5:14733877-14734521 FORWARD LENGTH=214 | 201606
Arabidopsis_thaliana OG0000004
AT5G37230.1 | RING/U-box superfamily protein |
Chr5:14735154-14735780 FORWARD LENGTH=208 | 201606
Arabidopsis_thaliana OG0000004
AT5G37250.1 | RING/U-box superfamily protein |
Chr5:14750393-14750971 FORWARD LENGTH=192 | 201606
Arabidopsis_thaliana OG0000004
AT5G37270.1 | RING/U-box superfamily protein |
Chr5:14757933-14758559 REVERSE LENGTH=208 | 201606
Arabidopsis_thaliana OG0000004
AT5G37280.1 | RING/U-box superfamily protein |
Chr5:14761275-14761925 REVERSE LENGTH=216 | 201606
Arabidopsis_thaliana OG0000004
AT5G38895.1 | RING/U-box superfamily protein |
Chr5:15572084-15573315 FORWARD LENGTH=221 | 201606
Arabidopsis_thaliana OG0000004
AT1G23980.1 | RING/U-box superfamily protein | Chr1:8484879-8485988
REVERSE LENGTH=369 | 201606 Arabidopsis_thaliana
OG0000004
AT5G40250.1 | RING/U-box superfamily protein |
Chr5:16086056-16087186 FORWARD LENGTH=376 | 201606
Arabidopsis_thaliana OG0000004
AT5G41350.1 | RING/U-box superfamily protein |
Chr5:16542026-16543092 REVERSE LENGTH=212 | 201606
Arabidopsis_thaliana OG0000004
AT5G41400.1 | RING/U-box superfamily protein |
Chr5:16569584-16570114 REVERSE LENGTH=176 | 201606
Arabidopsis_thaliana OG0000004
AT5G41430.1 | RING/U-box superfamily protein |
Chr5:16586119-16586604 REVERSE LENGTH=161 | 201606
Arabidopsis_thaliana OG0000004
AT5G41440.1 | RING/U-box superfamily protein |
Chr5:16587235-16587609 REVERSE LENGTH=124 | 201606
Arabidopsis_thaliana OG0000004
AT5G41450.1 | RING/U-box superfamily protein |
Chr5:16588600-16589094 REVERSE LENGTH=164 | 201606
Arabidopsis_thaliana OG0000004
AT5G42200.1 | RING/U-box superfamily protein |
Chr5:16860523-16861014 FORWARD LENGTH=163 | 201606

Arabidopsis_thaliana   OG0000004
AT5G42940.1 | RING/U-box superfamily protein |
Chr5:17216649-17219171 REVERSE LENGTH=691 | 201606
Arabidopsis_thaliana   OG0000004
AT5G43200.1 | Zinc finger%2C C3HC4 type (RING finger) family protein
| Chr5:17346141-17346764 REVERSE LENGTH=207 | 201606
Arabidopsis_thaliana   OG0000004
AT5G43420.1 | RING/U-box superfamily protein |
Chr5:17451790-17452917 FORWARD LENGTH=375 | 201606
Arabidopsis_thaliana   OG0000004
AT5G45290.1 | RING/U-box superfamily protein |
Chr5:18350011-18352092 REVERSE LENGTH=545 | 201606
Arabidopsis_thaliana   OG0000004
AT1G24580.1 | RING/U-box superfamily protein | Chr1:8710178-8710573
FORWARD LENGTH=131 | 201606   Arabidopsis_thaliana
OG0000004
AT5G46650.1 | RING/U-box superfamily protein |
Chr5:18930443-18931312 FORWARD LENGTH=289 | 201606
Arabidopsis_thaliana   OG0000004
AT5G47610.1 | RING/U-box superfamily protein |
Chr5:19301399-19301899 REVERSE LENGTH=166 | 201606
Arabidopsis_thaliana   OG0000004
AT5G52140.1 | RING/U-box superfamily protein |
Chr5:21184566-21186872 REVERSE LENGTH=280 | 201606
Arabidopsis_thaliana   OG0000004
AT5G52150.1 | RING/U-box superfamily protein |
Chr5:21189988-21191303 REVERSE LENGTH=200 | 201606
Arabidopsis_thaliana   OG0000004
AT5G52155.1 | E3 ubiquitin ligase BIG BROTHER-like protein |
Chr5:21192235-21194021 REVERSE LENGTH=182 | 201606
Arabidopsis_thaliana   OG0000004
AT5G53110.1 | RING/U-box superfamily protein |
Chr5:21529022-21533008 FORWARD LENGTH=382 | 201606
Arabidopsis_thaliana   OG0000004
AT5G53910.1 | RING/U-box superfamily protein |
Chr5:21890643-21891335 FORWARD LENGTH=230 | 201606
Arabidopsis_thaliana   OG0000004
AT5G54990.1 | RING/U-box superfamily protein |
Chr5:22317505-22318185 FORWARD LENGTH=226 | 201606
Arabidopsis_thaliana   OG0000004
AT5G55970.1 | RING/U-box superfamily protein |
Chr5:22668019-22669312 FORWARD LENGTH=343 | 201606
Arabidopsis_thaliana   OG0000004
AT5G56340.1 | RING/U-box superfamily protein |
Chr5:22818254-22819444 FORWARD LENGTH=396 | 201606
Arabidopsis_thaliana   OG0000004
AT5G57750.1 | RING/U-box superfamily protein |
Chr5:23399668-23400300 FORWARD LENGTH=210 | 201606
Arabidopsis_thaliana   OG0000004
AT5G58580.1 | TOXICOS EN LEVADURA 63 | Chr5:23676906-23677832
REVERSE LENGTH=308 | 201606   Arabidopsis_thaliana
OG0000004
AT5G59550.1 | zinc finger (C3HC4-type RING finger) family protein |
Chr5:23998422-23999645 REVERSE LENGTH=407 | 201606

Arabidopsis_thaliana   OG0000004
AT1G26800.1 | RING/U-box superfamily protein | Chr1:9285576-9286190 REVERSE LENGTH=204 | 201606   Arabidopsis_thaliana
OG0000004
AT5G60820.1 | RING/U-box superfamily protein |
Chr5:24469636-24470895 FORWARD LENGTH=419 | 201606
Arabidopsis_thaliana   OG0000004
AT5G64920.1 | COP1-interacting protein 8 | Chr5:25944338-25945342 REVERSE LENGTH=334 | 201606   Arabidopsis_thaliana
OG0000004
AT5G66070.1 | RING/U-box superfamily protein |
Chr5:26421923-26423033 FORWARD LENGTH=221 | 201606
Arabidopsis_thaliana   OG0000004
AT5G66160.1 | receptor homology region transmembrane domain ring H2 motif protein 1 | Chr5:26445198-26446878 FORWARD LENGTH=310 | 201606
Arabidopsis_thaliana   OG0000004
AT5G67120.1 | RING/U-box superfamily protein |
Chr5:26787830-26789737 REVERSE LENGTH=272 | 201606
Arabidopsis_thaliana   OG0000004
AT1G28040.1 | RING/U-box superfamily protein | Chr1:9773580-9774910 REVERSE LENGTH=299 | 201606   Arabidopsis_thaliana
OG0000004
AT1G32361.1 | RING/U-box superfamily protein |
Chr1:11675531-11676529 FORWARD LENGTH=332 | 201606
Arabidopsis_thaliana   OG0000004
AT1G33480.1 | RING/U-box superfamily protein |
Chr1:12148758-12150121 REVERSE LENGTH=261 | 201606
Arabidopsis_thaliana   OG0000004
AT1G35330.1 | RING/U-box superfamily protein |
Chr1:12965043-12966113 FORWARD LENGTH=328 | 201606
Arabidopsis_thaliana   OG0000004
AT1G04360.1 | RING/U-box superfamily protein | Chr1:1167507-1168652 REVERSE LENGTH=381 | 201606   Arabidopsis_thaliana
OG0000004
AT1G35625.1 | RING/U-box superfamily protein |
Chr1:13158466-13160151 REVERSE LENGTH=318 | 201606
Arabidopsis_thaliana   OG0000004
AT1G35630.1 | Protease-associated (PA) RING/U-box zinc finger family protein | Chr1:13163041-13164484 REVERSE LENGTH=318 | 201606
Arabidopsis_thaliana   OG0000004
AT1G36950.1 | RING/U-box superfamily protein |
Chr1:14009673-14010822 REVERSE LENGTH=226 | 201606
Arabidopsis_thaliana   OG0000004
AT1G45180.1 | RING/U-box superfamily protein |
Chr1:17099335-17101683 FORWARD LENGTH=645 | 201606
Arabidopsis_thaliana   OG0000004
AT1G04790.1 | RING/U-box superfamily protein | Chr1:1345469-1348143 FORWARD LENGTH=634 | 201606   Arabidopsis_thaliana
OG0000004
AT1G49200.1 | RING/U-box superfamily protein |
Chr1:18198298-18198978 FORWARD LENGTH=226 | 201606
Arabidopsis_thaliana   OG0000004
AT1G49210.1 | RING/U-box superfamily protein |
Chr1:18201994-18202671 FORWARD LENGTH=225 | 201606

Arabidopsis_thaliana   OG0000004
AT1G49220.1 | RING/U-box superfamily protein |
Chr1:18205946-18206701 FORWARD LENGTH=251 | 201606
Arabidopsis_thaliana   OG0000004
AT1G49230.1 | RING/U-box superfamily protein |
Chr1:18209320-18209979 FORWARD LENGTH=219 | 201606
Arabidopsis_thaliana   OG0000004
AT1G49850.1 | RING/U-box superfamily protein |
Chr1:18455326-18456444 REVERSE LENGTH=250 | 201606
Arabidopsis_thaliana   OG0000004
AT1G50350.1 | E3 ubiquitin-protein ligase RING1-like protein |
Chr1:18648756-18649227 FORWARD LENGTH=133 | 201606
Arabidopsis_thaliana   OG0000004
AT1G51930.1 | RING/U-box superfamily protein |
Chr1:19294838-19295236 REVERSE LENGTH=132 | 201606
Arabidopsis_thaliana   OG0000004
AT1G53010.1 | RING/U-box superfamily protein |
Chr1:19747847-19748383 FORWARD LENGTH=178 | 201606
Arabidopsis_thaliana   OG0000004
AT1G53190.1 | RING/U-box superfamily protein |
Chr1:19838072-19840003 FORWARD LENGTH=494 | 201606
Arabidopsis_thaliana   OG0000004
AT1G53820.1 | RING/U-box superfamily protein |
Chr1:20091491-20092423 FORWARD LENGTH=310 | 201606
Arabidopsis_thaliana   OG0000004
AT1G55530.1 | RING/U-box superfamily protein |
Chr1:20729472-20730527 REVERSE LENGTH=351 | 201606
Arabidopsis_thaliana   OG0000004
AT1G57730.1 | RING/U-box superfamily protein |
Chr1:21380858-21381382 REVERSE LENGTH=174 | 201606
Arabidopsis_thaliana   OG0000004
AT1G60360.1 | RING/U-box superfamily protein |
Chr1:22242748-22243731 REVERSE LENGTH=327 | 201606
Arabidopsis_thaliana   OG0000004
AT1G63170.1 | Zinc finger%2C C3HC4 type (RING finger) family protein
| Chr1:23425574-23427073 FORWARD LENGTH=381 | 201606
Arabidopsis_thaliana   OG0000004
AT1G63840.1 | RING/U-box superfamily protein |
Chr1:23689991-23690491 REVERSE LENGTH=166 | 201606
Arabidopsis_thaliana   OG0000004
AT1G68070.1 | Zinc finger%2C C3HC4 type (RING finger) family protein
| Chr1:25515412-25516767 REVERSE LENGTH=343 | 201606
Arabidopsis_thaliana   OG0000004
AT1G68180.1 | RING/U-box superfamily protein |
Chr1:25554816-25555625 FORWARD LENGTH=235 | 201606
Arabidopsis_thaliana   OG0000004
AT1G69085.1 | E3 ubiquitin-protein ligase | Chr1:25975120-25975793
FORWARD LENGTH=117 | 201606   Arabidopsis_thaliana
OG0000004
AT1G70910.1 | RING/U-box superfamily protein |
Chr1:26734155-26734640 FORWARD LENGTH=161 | 201606
Arabidopsis_thaliana   OG0000004
AT1G71980.1 | Protease-associated (PA) RING/U-box zinc finger family
protein | Chr1:27098250-27099881 FORWARD LENGTH=448 | 201606

Arabidopsis_thaliana OG0000004
AT1G72200.1 | RING/U-box superfamily protein |
Chr1:27169935-27171149 REVERSE LENGTH=404 | 201606
Arabidopsis_thaliana OG0000004
AT1G72220.1 | RING/U-box superfamily protein |
Chr1:27184388-27185629 REVERSE LENGTH=413 | 201606
Arabidopsis_thaliana OG0000004
AT1G72310.1 | RING/U-box superfamily protein |
Chr1:27226405-27227379 FORWARD LENGTH=324 | 201606
Arabidopsis_thaliana OG0000004
AT1G73760.1 | RING/U-box superfamily protein |
Chr1:27739366-27741161 REVERSE LENGTH=367 | 201606
Arabidopsis_thaliana OG0000004
AT1G74410.1 | RING/U-box superfamily protein |
Chr1:27965723-27967681 FORWARD LENGTH=223 | 201606
Arabidopsis_thaliana OG0000004
AT1G74620.1 | RING/U-box superfamily protein |
Chr1:28028252-28029001 FORWARD LENGTH=249 | 201606
Arabidopsis_thaliana OG0000004
AT1G76410.1 | RING/U-box superfamily protein |
Chr1:28668915-28669472 FORWARD LENGTH=185 | 201606
Arabidopsis_thaliana OG0000004
AT1G80400.1 | RING/U-box superfamily protein |
Chr1:30225864-30227360 FORWARD LENGTH=407 | 201606
Arabidopsis_thaliana OG0000004
AT2G01150.1 | RING-H2 finger protein 2B | Chr2:100703-101146 FORWARD
LENGTH=147 | 201606 Arabidopsis_thaliana OG0000004
AT2G01735.1 | RING-finger protein for embryogenesi |
Chr2:324499-325895 FORWARD LENGTH=359 | 201606
Arabidopsis_thaliana OG0000004
AT2G03000.1 | RING/U-box superfamily protein | Chr2:875233-877207
FORWARD LENGTH=535 | 201606 Arabidopsis_thaliana
OG0000004
AT2G04240.1 | RING/U-box superfamily protein | Chr2:1461816-1462304
REVERSE LENGTH=162 | 201606 Arabidopsis_thaliana
OG0000004
AT2G15530.1 | RING/U-box superfamily protein | Chr2:6774150-6777068
FORWARD LENGTH=704 | 201606 Arabidopsis_thaliana
OG0000004
AT2G15580.1 | RING/U-box superfamily protein | Chr2:6797687-6798815
FORWARD LENGTH=196 | 201606 Arabidopsis_thaliana
OG0000004
AT2G17450.1 | RING-H2 finger A3A | Chr2:7576640-7577197 REVERSE
LENGTH=185 | 201606 Arabidopsis_thaliana OG0000004
AT2G17730.1 | NEP-interacting protein 2 | Chr2:7704142-7705312
FORWARD LENGTH=241 | 201606 Arabidopsis_thaliana
OG0000004
AT2G18650.1 | RING/U-box superfamily protein | Chr2:8086860-8088131
REVERSE LENGTH=423 | 201606 Arabidopsis_thaliana
OG0000004
AT2G18670.1 | RING/U-box superfamily protein | Chr2:8093469-8094452
FORWARD LENGTH=181 | 201606 Arabidopsis_thaliana
OG0000004
AT2G20030.1 | RING/U-box superfamily protein | Chr2:8647813-8648985

FORWARD LENGTH=390 | 201606 Arabidopsis_thaliana
OG0000004
AT2G24480.1 | Zinc finger%2C C3HC4 type (RING finger) family protein
| Chr2:10397588-10398184 FORWARD LENGTH=198 | 201606
Arabidopsis_thaliana OG0000004
AT2G25410.1 | RING/U-box superfamily protein |
Chr2:10814470-10815917 FORWARD LENGTH=377 | 201606
Arabidopsis_thaliana OG0000004
AT2G27940.1 | RING/U-box superfamily protein |
Chr2:11897963-11898742 FORWARD LENGTH=237 | 201606
Arabidopsis_thaliana OG0000004
AT2G28920.1 | RING/U-box superfamily protein |
Chr2:12418017-12418454 FORWARD LENGTH=145 | 201606
Arabidopsis_thaliana OG0000004
AT2G29840.1 | RING/U-box superfamily protein |
Chr2:12732387-12733983 REVERSE LENGTH=293 | 201606
Arabidopsis_thaliana OG0000004
AT2G34000.1 | RING/U-box superfamily protein |
Chr2:14365177-14365632 FORWARD LENGTH=151 | 201606
Arabidopsis_thaliana OG0000004
Os03t0271600-01 Zinc finger, RING/FYVE/PHD-type domain containing
protein. Oryza_sativa OG0000004
Os03t0272300-01 Similar to protein binding protein.
Oryza_sativa OG0000004
Os03t0286100-00 Similar to protein binding protein.
Oryza_sativa OG0000004
Os03t0324900-01 Zinc finger, RING/FYVE/PHD-type domain containing
protein. Oryza_sativa OG0000004
Os03t0340100-00 Similar to Zinc finger, C3HC4 type family protein.
Oryza_sativa OG0000004
Os03t0351800-01 Zinc finger, RING/FYVE/PHD-type domain containing
protein. Oryza_sativa OG0000004
Os03t0377600-01 Zinc finger, RING/FYVE/PHD-type domain containing
protein. Oryza_sativa OG0000004
Os03t0380700-00 Similar to Zinc finger, C3HC4 type family protein.
Oryza_sativa OG0000004
Os03t0391100-00 Zinc finger, RING-type domain containing protein.
Oryza_sativa OG0000004
Os03t0398250-00 Zinc finger, RING/FYVE/PHD-type domain containing
protein. Oryza_sativa OG0000004
Os03t0398600-00 Hypothetical conserved gene. Oryza_sativa
OG0000004
Os03t0598700-01 Zinc finger, RING/FYVE/PHD-type domain containing
protein. Oryza_sativa OG0000004
Os03t0648600-00 Similar to Zinc finger, C3HC4 type family protein,
expressed. Oryza_sativa OG0000004
Os01t0266100-01 Similar to RING finger family protein.
Oryza_sativa OG0000004
Os03t0788100-01 Similar to RING-H2 finger protein ATL1P (RING-H2
finger protein ATL3). Oryza_sativa OG0000004
Os03t0812200-01 Zinc finger, RING/FYVE/PHD-type domain containing
protein. Oryza_sativa OG0000004
Os01t0276600-01 Zinc finger, RING/FYVE/PHD-type domain containing
protein. Oryza_sativa OG0000004

Os04t0185500-01 Similar to Protein containing C-terminal RING-finger. Oryza_sativa OG0000004
Os04t0243700-01 Zinc finger, RING/FYVE/PHD-type domain containing protein. Oryza_sativa OG0000004
Os04t0419550-01 Zinc finger, RING-type domain containing protein. Oryza_sativa OG0000004
Os04t0450300-00 Similar to H0818E04.18 protein. Oryza_sativa OG0000004
Os04t0450400-01 Similar to H0818E04.19 protein. Oryza_sativa OG0000004
Os01t0311100-00 Similar to RING-H2 finger protein ATL2L. Oryza_sativa OG0000004
Os01t0311400-00 Zinc finger, RING/FYVE/PHD-type domain containing protein. Oryza_sativa OG0000004
Os04t0487400-00 Zinc finger, RING-type domain containing protein. Oryza_sativa OG0000004
Os04t0511600-01 Similar to RING-H2 finger protein ATL3G. Oryza_sativa OG0000004
Os04t0568900-01 Similar to RING finger protein 6 (RING-H2 protein). Oryza_sativa OG0000004
Os04t0571200-01 Similar to OSIGBa0111L12.9 protein. Oryza_sativa OG0000004
Os04t0571800-01 Zinc finger, RING/FYVE/PHD-type domain containing protein. Oryza_sativa OG0000004
Os04t0580800-01 Zinc finger, RING/FYVE/PHD-type domain containing protein. Oryza_sativa OG0000004
Os04t0580866-00 Hypothetical conserved gene. Oryza_sativa OG0000004
Os04t0585000-01 Similar to RING-H2 finger protein ATL1R. Oryza_sativa OG0000004
Os04t0586700-01 Zinc finger, RING/FYVE/PHD-type domain containing protein. Oryza_sativa OG0000004
Os04t0590900-01 Similar to RING-H2 finger protein ATL1B. Oryza_sativa OG0000004
Os04t0648800-01 C3HC4 RING domain-containing E3 ubiquitin ligase, Modulation of heading date by physically interacting with Hd1 Oryza_sativa OG0000004
Os05t0110000-01 Zinc finger, RING/FYVE/PHD-type domain containing protein. Oryza_sativa OG0000004
Os05t0163400-01 Zinc finger, RING/FYVE/PHD-type domain containing protein. Oryza_sativa OG0000004
Os05t0164200-01 Zinc finger, RING/FYVE/PHD-type domain containing protein. Oryza_sativa OG0000004
Os05t0179000-01 Zinc finger, RING/FYVE/PHD-type domain containing protein. Oryza_sativa OG0000004
Os05t0359500-00 Zinc finger, RING-type domain containing protein. Oryza_sativa OG0000004
Os05t0360400-01 Zinc finger, RING/FYVE/PHD-type domain containing protein. Oryza_sativa OG0000004
Os05t0389600-01 Zinc finger, RING/FYVE/PHD-type domain containing protein. Oryza_sativa OG0000004
Os05t0408800-00 Similar to zinc finger, C3HC4 type family protein. Oryza_sativa OG0000004
Os05t0426066-00 Hypothetical conserved gene. Oryza_sativa

OG0000004
Os05t0439000-01 Similar to RING-H2 finger protein ATL1O (RING-H2 finger protein ATL11).	Oryza_sativa	OG0000004
Os05t0468900-01 Zinc finger, RING/FYVE/PHD-type domain containing protein.	Oryza_sativa	OG0000004
Os05t0478000-01 Zinc finger, RING/FYVE/PHD-type domain containing protein.	Oryza_sativa	OG0000004
Os05t0488800-01 Zinc finger, RING/FYVE/PHD-type domain containing protein.	Oryza_sativa	OG0000004
Os05t0495000-00 Hypothetical conserved gene.	Oryza_sativa	OG0000004
Os05t0526600-00 Zinc finger, RING/FYVE/PHD-type domain containing protein.	Oryza_sativa	OG0000004
Os05t0550000-00 Similar to protein binding protein.	Oryza_sativa	OG0000004
Os05t0564300-01 Zinc finger, RING/FYVE/PHD-type domain containing protein.	Oryza_sativa	OG0000004
Os05t0596500-01 Similar to RING-H2 finger protein ATL3I (YGHL1-C3HC4 RING fusion protein).	Oryza_sativa	OG0000004
Os06t0101300-01 Zinc finger, RING/FYVE/PHD-type domain containing protein.	Oryza_sativa	OG0000004
Os06t0109100-00 Hypothetical conserved gene.	Oryza_sativa	OG0000004
Os06t0125800-01 Zinc finger, RING/FYVE/PHD-type domain containing protein.	Oryza_sativa	OG0000004
Os01t0535100-00 Zinc finger, RING-type domain containing protein.	Oryza_sativa	OG0000004
Os01t0535300-00 Zinc finger, RING-type domain containing protein.	Oryza_sativa	OG0000004
Os06t0167200-01 Similar to RING-H2 finger protein ATL1R (RING-H2 finger protein ATL8).	Oryza_sativa	OG0000004
Os06t0187300-00 Similar to RING-H2 finger protein ATL2C.	Oryza_sativa	OG0000004
Os06t0192800-01 Similar to RING-H2 finger protein ATL1R (RING-H2 finger protein ATL8).	Oryza_sativa	OG0000004
Os06t0218300-01 Zinc finger, RING-type domain containing protein.	Oryza_sativa	OG0000004
Os06t0231600-01 Similar to RING-H2 finger protein ATL1Q.	Oryza_sativa	OG0000004
Os06t0233200-01 Similar to RING-H2 finger protein ATL1R (RING-H2 finger protein ATL8).	Oryza_sativa	OG0000004
Os06t0271600-00 Zinc finger, RING/FYVE/PHD-type domain containing protein.	Oryza_sativa	OG0000004
Os06t0534500-00 Zinc finger, RING-type domain containing protein.	Oryza_sativa	OG0000004
Os06t0534800-01 Zinc finger, RING-type domain containing protein.	Oryza_sativa	OG0000004
Os06t0534900-00 Hypothetical conserved gene.	Oryza_sativa	OG0000004
Os06t0534950-00 Similar to zinc finger, C3HC4 type family protein.	Oryza_sativa	OG0000004
Os06t0535200-01 Zinc finger, RING/FYVE/PHD-type domain containing protein.	Oryza_sativa	OG0000004
Os06t0535400-01 Similar to E3 ubiquitin ligase EL5 (EC 6.3.2.-).

Oryza_sativa OG0000004
Os06t0535900-01 Arbuscular mycorrhizal specific marker 29. Oryza_sativa OG0000004
Os06t0536100-00 Hypothetical conserved gene. Oryza_sativa OG0000004
Os06t0536600-00 Zinc finger, RING-type domain containing protein. Oryza_sativa OG0000004
Os06t0537300-00 Zinc finger, RING-type domain containing protein. Oryza_sativa OG0000004
Os06t0537500-00 Zinc finger, RING-type domain containing protein. Oryza_sativa OG0000004
Os06t0537600-01 Zinc finger, RING/FYVE/PHD-type domain containing protein. Oryza_sativa OG0000004
Os06t0540050-00 Hypothetical conserved gene. Oryza_sativa OG0000004
Os06t0540200-00 Zinc finger, RING-type domain containing protein. Oryza_sativa OG0000004
Os06t0633500-01 Zinc finger, RING/FYVE/PHD-type domain containing protein. Oryza_sativa OG0000004
Os06t0666500-00 Zinc finger, RING/FYVE/PHD-type domain containing protein. Oryza_sativa OG0000004
Os06t0687200-01 Zinc finger, RING/FYVE/PHD-type domain containing protein. Oryza_sativa OG0000004
Os06t0695600-01 Zinc finger, RING/FYVE/PHD-type domain containing protein. Oryza_sativa OG0000004
Os06t0695900-01 Zinc finger, RING-type domain containing protein. Oryza_sativa OG0000004
Os06t0717600-01 Zinc finger, RING/FYVE/PHD-type domain containing protein. Oryza_sativa OG0000004
Os07t0159600-00 Similar to RING-H2 finger protein ATL2I. Oryza_sativa OG0000004
Os07t0194675-00 Zinc finger, RING/FYVE/PHD-type domain containing protein. Oryza_sativa OG0000004
Os07t0421800-01 Hypothetical conserved gene. Oryza_sativa OG0000004
Os07t0463400-01 Similar to RES protein. Oryza_sativa OG0000004
Os07t0479100-01 Zinc finger, RING/FYVE/PHD-type domain containing protein. Oryza_sativa OG0000004
Os07t0631200-01 Zinc finger, RING/FYVE/PHD-type domain containing protein. Oryza_sativa OG0000004
Os07t0686300-01 Zinc finger, RING/FYVE/PHD-type domain containing protein. Oryza_sativa OG0000004
Os08t0104300-01 Similar to RING finger protein 13. Oryza_sativa OG0000004
Os08t0157400-01 Zinc finger, RING/FYVE/PHD-type domain containing protein. Oryza_sativa OG0000004
Os08t0241400-00 Similar to protein binding protein. Oryza_sativa OG0000004
Os08t0445000-01 Similar to RING-H2 finger protein ATL5A. Oryza_sativa OG0000004
Os08t0464400-01 Zinc finger, RING/FYVE/PHD-type domain containing protein. Oryza_sativa OG0000004
Os08t0484200-01 Zinc finger, RING/FYVE/PHD-type domain containing

protein. Oryza_sativa	OG0000004
Os08t0487500-01 Zinc finger, RING/FYVE/PHD-type domain containing protein. Oryza_sativa	OG0000004
Os08t0492500-01 Zinc finger, RING/FYVE/PHD-type domain containing protein. Oryza_sativa	OG0000004
Os08t0548300-01 Zinc finger, RING/FYVE/PHD-type domain containing protein. Oryza_sativa	OG0000004
Os08t0550400-01 Similar to RING-H2 finger protein ATL5P. Oryza_sativa	OG0000004
Os01t0667700-01 Zinc finger, RING/FYVE/PHD-type domain containing protein. Oryza_sativa	OG0000004
Os08t0563500-00 Hypothetical conserved gene.	Oryza_sativa	OG0000004
Os09t0242800-01 Zinc finger, RING/FYVE/PHD-type domain containing protein. Oryza_sativa	OG0000004
Os09t0243200-01 Zinc finger, RING/FYVE/PHD-type domain containing protein. Oryza_sativa	OG0000004
Os01t0673900-01 Zinc finger, RING-type domain containing protein. Oryza_sativa	OG0000004
Os09t0323100-01 RING finger ubiquitin E3 ligase, Heat tolerance, Modulation of hydrogen peroxide-induced stomatal closure Oryza_sativa	OG0000004
Os09t0344900-01 Zinc finger, RING/FYVE/PHD-type domain containing protein. Oryza_sativa	OG0000004
Os09t0376700-01 ATL family protein containing RING-H2 finger motif Oryza_sativa	OG0000004
Os09t0434200-01 Similar to ABI3-interacting protein 2-1 protein. Oryza_sativa	OG0000004
Os09t0446275-00 Zinc finger, RING-type domain containing protein. Oryza_sativa	OG0000004
Os09t0468300-00 Hypothetical conserved gene.	Oryza_sativa	OG0000004
Os09t0468800-00 Similar to RING-H2 finger protein ATL5F. Oryza_sativa	OG0000004
Os09t0478600-01 Zinc finger, RING/FYVE/PHD-type domain containing protein. Oryza_sativa	OG0000004
Os01t0690333-00 Similar to Zinc finger, C3HC4 type family protein. Oryza_sativa	OG0000004
Os09t0525400-01 Similar to RING finger protein 13 (C-RZF). Oryza_sativa	OG0000004
Os09t0535100-01 Zinc finger, RING/FYVE/PHD-type domain containing protein. Oryza_sativa	OG0000004
Os09t0535500-01 Similar to RING-H2 finger protein ATL1R (RING-H2 finger protein ATL8).	Oryza_sativa	OG0000004
Os09t0542600-01 Similar to RING-H2 finger protein ATL1I. Oryza_sativa	OG0000004
Os09t0554200-01 Zinc finger, RING/FYVE/PHD-type domain containing protein. Oryza_sativa	OG0000004
Os10t0142100-01 Zinc finger, RING/FYVE/PHD-type domain containing protein. Oryza_sativa	OG0000004
Os01t0703300-01 Zinc finger, RING/FYVE/PHD-type domain containing protein. Oryza_sativa	OG0000004
Os10t0438800-01 Similar to RING-H2 finger protein ATL2E. Oryza_sativa	OG0000004

Os10t0487400-01 Zinc finger, RING/FYVE/PHD-type domain containing protein. Oryza_sativa OG0000004
Os10t0544600-01 Zinc finger, RING/FYVE/PHD-type domain containing protein. Oryza_sativa OG0000004
Os10t0546900-00 Similar to Zinc finger, C3HC4 type family protein, expressed. Oryza_sativa OG0000004
Os10t0566400-01 Similar to RING-H2 finger protein ATL3J. Oryza_sativa OG0000004
Os10t0574400-01 Zinc finger, RING-type domain containing protein. Oryza_sativa OG0000004
Os11t0113900-00 Similar to Zinc finger, C3HC4 type family protein. Oryza_sativa OG0000004
Os11t0114000-00 Similar to Zinc finger, C3HC4 type family protein. Oryza_sativa OG0000004
Os11t0119200-01 Zinc finger, RING/FYVE/PHD-type domain containing protein. Oryza_sativa OG0000004
Os11t0142800-00 Similar to Zinc finger, C3HC4 type family protein. Oryza_sativa OG0000004
Os11t0144500-01 Zinc finger, RING/FYVE/PHD-type domain containing protein. Oryza_sativa OG0000004
Os11t0146750-00 Hypothetical conserved gene. Oryza_sativa OG0000004
Os11t0150450-01 Similar to Zinc finger, C3HC4 type family protein, expressed. Oryza_sativa OG0000004
Os01t0736600-01 Zinc finger, RING/FYVE/PHD-type domain containing protein. Oryza_sativa OG0000004
Os11t0294600-01 Zinc finger, RING/FYVE/PHD-type domain containing protein. Oryza_sativa OG0000004
Os11t0582100-01 Zinc finger, RING/FYVE/PHD-type domain containing protein. Oryza_sativa OG0000004
Os11t0610600-00 Zinc finger, RING-type domain containing protein. Oryza_sativa OG0000004
Os11t0629300-01 Similar to RING-H2 finger protein RHB1a. Oryza_sativa OG0000004
Os11t0649801-00 Similar to E3 ubiquitin-protein ligase EL5. Oryza_sativa OG0000004
Os01t0755700-01 Zinc finger, RING/FYVE/PHD-type domain containing protein. Oryza_sativa OG0000004
Os12t0108350-00 Similar to Zinc finger, C3HC4 type family protein. Oryza_sativa OG0000004
Os12t0113700-00 Similar to Zinc finger, C3HC4 type family protein, expressed. Oryza_sativa OG0000004
Os12t0113800-00 Similar to Zinc finger, C3HC4 type family protein. Oryza_sativa OG0000004
Os12t0118700-01 Zinc finger, RING/FYVE/PHD-type domain containing protein. Oryza_sativa OG0000004
Os12t0134500-00 Similar to Zinc finger, C3HC4 type family protein. Oryza_sativa OG0000004
Os12t0135300-00 Similar to Kelch motif family protein. Oryza_sativa OG0000004
Os12t0139100-00 Similar to Zinc finger, C3HC4 type family protein. Oryza_sativa OG0000004
Os12t0140200-00 Zinc finger, RING/FYVE/PHD-type domain containing protein. Oryza_sativa OG0000004

Os12t0140600-01 Zinc finger, RING/FYVE/PHD-type domain containing protein. Oryza_sativa OG0000004
Os12t0140700-01 Zinc finger, RING/FYVE/PHD-type domain containing protein. Oryza_sativa OG0000004
Os12t0143750-01 Zinc finger, RING/FYVE/PHD-type domain containing protein. Oryza_sativa OG0000004
Os12t0432600-01 Zinc finger, RING/FYVE/PHD-type domain containing protein. Oryza_sativa OG0000004
Os12t0580700-01 Similar to RING-H2 finger protein ATL2N. Oryza_sativa OG0000004
Os12t0620100-00 Zinc finger, RING-type domain containing protein. Oryza_sativa OG0000004
Os01t0802000-01 Zinc finger, RING/FYVE/PHD-type domain containing protein. Oryza_sativa OG0000004
Os01t0822800-01 Similar to RING-H2 finger protein ATL3C. Oryza_sativa OG0000004
Os01t0830900-00 Hypothetical conserved gene. Oryza_sativa OG0000004
Os01t0844700-00 Similar to protein binding protein. Oryza_sativa OG0000004
Os01t0866000-01 Similar to E3 ubiquitin ligase EL5 (EC 6.3.2.-). Oryza_sativa OG0000004
Os01t0926200-01 Similar to RING-H2 finger protein RHF1a (Fragment). Oryza_sativa OG0000004
Os01t0159300-01 Zinc finger, RING/FYVE/PHD-type domain containing protein. Oryza_sativa OG0000004
Os01t0972000-01 Zinc finger, RING/FYVE/PHD-type domain containing protein. Oryza_sativa OG0000004
Os02t0106600-01 Zinc finger, RING/FYVE/PHD-type domain containing protein. Oryza_sativa OG0000004
Os02t0150700-01 Zinc finger, RING-type domain containing protein. Oryza_sativa OG0000004
Os02t0178600-01 Similar to E3 ubiquitin ligase EL5 (EC 6.3.2.-). Oryza_sativa OG0000004
Os02t0191500-01 Similar to Goliath homolog precursor (RING finger protein 130). Oryza_sativa OG0000004
Os02t0248100-00 Hypothetical conserved gene. Oryza_sativa OG0000004
Os02t0248200-00 Zinc finger, RING-type domain containing protein. Oryza_sativa OG0000004
Os02t0248440-00 Zinc finger, RING-type domain containing protein. Oryza_sativa OG0000004
Os02t0249200-00 Similar to RING-H2 finger protein ATL5H. Oryza_sativa OG0000004
Os02t0249300-00 Zinc finger, RING/FYVE/PHD-type domain containing protein. Oryza_sativa OG0000004
Os01t0178700-01 Similar to Protein binding protein. Oryza_sativa OG0000004
Os02t0475101-00 Similar to RING-H2 finger protein ATL5A. Oryza_sativa OG0000004
Os02t0528000-00 Zinc finger, RING-type domain containing protein. Oryza_sativa OG0000004
Os02t0541500-00 Similar to predicted protein. Oryza_sativa OG0000004

Os02t0557300-01 Similar to RING-H2 finger protein ATL1G.
Oryza_sativa OG0000004
Os02t0559800-01 RING-H2 type E3 ubiquitin ligase, Root development, Maintenance of cell viability after the initiation of root primordial formation, Defense response Oryza_sativa OG0000004
Os02t0560200-01 RING-H2 type E3 ubiquitin ligase, Root development, Maintenance of cell viability after the initiation of root primordial formation, Defense response Oryza_sativa OG0000004
Os02t0560600-01 RING-H2 type E3 ubiquitin ligase, Root development, Maintenance of cell viability after the initiation of root primordial formation, Defense response Oryza_sativa OG0000004
Os02t0561000-01 RING-H2 type E3 ubiquitin ligase, Root development, Maintenance of cell viability after the initiation of root primordial formation, Defense response Oryza_sativa OG0000004
Os02t0561400-01 RING-H2 type E3 ubiquitin ligase, Root development, Maintenance of cell viability after the initiation of root primordial formation, Defense response Oryza_sativa OG0000004
Os02t0561800-01 RING-H2 type E3 ubiquitin ligase, Root development, Maintenance of cell viability after the initiation of root primordial formation, Defense response Oryza_sativa OG0000004
Os02t0561900-00 Similar to E3 ubiquitin-protein ligase EL5.
Oryza_sativa OG0000004
Os02t0572200-01 Similar to RING-H2 finger protein ATL3I (YGHL1-C3HC4 RING fusion protein). Oryza_sativa OG0000004
Os02t0572300-00 Similar to RING-H2 finger protein ATL3B.
Oryza_sativa OG0000004
Os02t0645000-01 Zinc finger, RING/FYVE/PHD-type domain containing protein. Oryza_sativa OG0000004
Os02t0666900-00 Zinc finger, RING-type domain containing protein.
Oryza_sativa OG0000004
Os02t0674700-00 Similar to protein binding protein.
Oryza_sativa OG0000004
Os02t0676500-00 Similar to OSIGBa0127A14.6 protein.
Oryza_sativa OG0000004
Os02t0682300-01 Zinc finger, RING/FYVE/PHD-type domain containing protein. Oryza_sativa OG0000004
Os02t0686100-00 Similar to RING-H2 finger protein ATL1B.
Oryza_sativa OG0000004
Os02t0692000-01 Similar to RING finger family protein.
Oryza_sativa OG0000004
Os02t0727700-01 Zinc finger, RING/FYVE/PHD-type domain containing protein. Oryza_sativa OG0000004
Os02t0730050-00 Zinc finger, RING-type domain containing protein.
Oryza_sativa OG0000004
Os02t0735900-01 Zinc finger, RING/FYVE/PHD-type domain containing protein. Oryza_sativa OG0000004
Os02t0743100-00 Zinc finger, RING/FYVE/PHD-type domain containing protein. Oryza_sativa OG0000004

Os02t0743700-01 Similar to RING-H2 finger protein ATL1Q.
Oryza_sativa OG0000004
Os02t0759400-01 Zinc finger, RING/FYVE/PHD-type domain containing protein. Oryza_sativa OG0000004
Os02t0767600-00 Similar to RHC1A. Oryza_sativa OG0000004
Os01t0212700-01 Similar to RING-H2 finger protein ATL2K.
Oryza_sativa OG0000004
Os02t0790600-01 Similar to RING-H2 finger protein ATL1R (RING-H2 finger protein ATL8). Oryza_sativa OG0000004
Os01t0213100-00 Similar to Zinc finger, C3HC4 type family protein.
Oryza_sativa OG0000004
Os01t0213400-01 Zinc finger, RING/FYVE/PHD-type domain containing protein. Oryza_sativa OG0000004
Os02t0798200-01 Zinc finger, RING/FYVE/PHD-type domain containing protein. Oryza_sativa OG0000004
Os02t0798600-01 Similar to Zinc finger, C3HC4 type family protein.
Oryza_sativa OG0000004
Os02t0820200-01 Zinc finger, RING/FYVE/PHD-type domain containing protein. Oryza_sativa OG0000004
Os02t0832150-00 Similar to zinc finger (C3HC4-type RING finger) family protein. Oryza_sativa OG0000004
Os03t0149700-00 Zinc finger, RING/FYVE/PHD-type domain containing protein. Oryza_sativa OG0000004
Os03t0149800-01 Similar to RING-H2 finger protein ATL1D.
Oryza_sativa OG0000004
Os03t0167500-01 Splicing isoform of OsRMR1 Oryza_sativa
OG0000004
Os03t0173900-01 Zinc finger, RING/FYVE/PHD-type domain containing protein. Oryza_sativa OG0000004
Os03t0188200-01 Zinc finger, RING/FYVE/PHD-type domain containing protein. Oryza_sativa OG0000004
Os03t0206900-01 Zinc finger, RING/FYVE/PHD-type domain containing protein. Oryza_sativa OG0000004
CMT443CT pep chromosome:ASM9120v1:20:1104160:1105344:1 gene:CMT443C transcript:CMT443CT gene_biotype:protein_coding transcript_biotype:protein_coding Cyanidioschyzon_merolae
OG0000004
CMI144CT pep chromosome:ASM9120v1:9:399174:400032:-1 gene:CMI144C transcript:CMI144CT gene_biotype:protein_coding transcript_biotype:protein_coding Cyanidioschyzon_merolae
OG0000004
CMG008CT pep chromosome:ASM9120v1:7:25214:26308:1 gene:CMG008C transcript:CMG008CT gene_biotype:protein_coding transcript_biotype:protein_coding Cyanidioschyzon_merolae
OG0000004
CMS095CT pep chromosome:ASM9120v1:19:239943:240507:-1 gene:CMS095C transcript:CMS095CT gene_biotype:protein_coding transcript_biotype:protein_coding Cyanidioschyzon_merolae
OG0000004
CMM236CT pep chromosome:ASM9120v1:13:587189:589609:1 gene:CMM236C transcript:CMM236CT gene_biotype:protein_coding transcript_biotype:protein_coding Cyanidioschyzon_merolae
OG0000004
CMM012CT pep chromosome:ASM9120v1:13:26513:27610:1 gene:CMM012C

transcript:CMM012CT gene_biotype:protein_coding
transcript_biotype:protein_coding Cyanidioschyzon_merolae
OG0000004
CMO162CT pep chromosome:ASM9120v1:15:419247:421374:-1 gene:CMO162C
transcript:CMO162CT gene_biotype:protein_coding
transcript_biotype:protein_coding Cyanidioschyzon_merolae
OG0000004
evm.model.contig_2102.17 Porphyridium_purpureum OG0000004
evm.model.contig_2210.2 Porphyridium_purpureum OG0000004
evm.model.contig_2281.4 Porphyridium_purpureum OG0000004
evm.model.contig_2025.32 Porphyridium_purpureum OG0000004
evm.model.contig_2287.2 Porphyridium_purpureum OG0000004
evm.model.contig_2500.15 Porphyridium_purpureum OG0000004
evm.model.contig_3384.9 Porphyridium_purpureum OG0000004
evm.model.contig_3394.2 Porphyridium_purpureum OG0000004
evm.model.contig_3435.1 Porphyridium_purpureum OG0000004
evm.model.contig_3497.6 Porphyridium_purpureum OG0000004
evm.model.contig_438.4 Porphyridium_purpureum OG0000004
evm.model.contig_4403.15 Porphyridium_purpureum OG0000004
evm.model.contig_4405.7 Porphyridium_purpureum OG0000004
evm.model.contig_4407.10 Porphyridium_purpureum OG0000004
evm.model.contig_4437.12 Porphyridium_purpureum OG0000004
evm.model.contig_4448.17 Porphyridium_purpureum OG0000004
evm.model.contig_4465.8 Porphyridium_purpureum OG0000004
evm.model.contig_4471.2 Porphyridium_purpureum OG0000004
evm.model.contig_465.2 Porphyridium_purpureum OG0000004
evm.model.contig_474.3 Porphyridium_purpureum OG0000004
evm.model.contig_481.14 Porphyridium_purpureum OG0000004
evm.model.contig_536.2 Porphyridium_purpureum OG0000004
evm.model.contig_591.1 Porphyridium_purpureum OG0000004
evm.model.contig_606.5 Porphyridium_purpureum OG0000004
evm.model.contig_2017.1 Porphyridium_purpureum OG0000004
evm.model.contig_2070.1 Porphyridium_purpureum OG0000004
CDF33945 pep supercontig:ASM35022v2:HG001663:39279:40645:1
gene:CHC_T00002618001 transcript:CDF33945
gene_biotype:protein_coding transcript_biotype:protein_coding
Chondrus_crispus OG0000004
CDF35579 pep supercontig:ASM35022v2:HG001735:172382:173012:-1
gene:CHC_T00004102001 transcript:CDF35579
gene_biotype:protein_coding transcript_biotype:protein_coding
Chondrus_crispus OG0000004
CDF35458 pep supercontig:ASM35022v2:HG001731:126451:126960:-1
gene:CHC_T00003966001 transcript:CDF35458
gene_biotype:protein_coding transcript_biotype:protein_coding
Chondrus_crispus OG0000004
CDF33605 pep supercontig:ASM35022v2:HG001651:31908:34656:-1
gene:CHC_T00002144001 transcript:CDF33605
gene_biotype:protein_coding transcript_biotype:protein_coding
Chondrus_crispus OG0000004
CDF38618 pep supercontig:ASM35022v2:HG001949:95535:97054:1
gene:CHC_T00006427001 transcript:CDF38618
gene_biotype:protein_coding transcript_biotype:protein_coding
Chondrus_crispus OG0000004
CDF41325 pep supercontig:ASM35022v2:HG002355:106318:107130:-1

```
gene:CHC_T00007841001 transcript:CDF41325
gene_biotype:protein_coding transcript_biotype:protein_coding
Chondrus_crispus OG0000004
CDF40180 pep supercontig:ASM35022v2:HG002134:382606:384552:1
gene:CHC_T00007036001 transcript:CDF40180
gene_biotype:protein_coding transcript_biotype:protein_coding
Chondrus_crispus OG0000004
CDF41286 pep supercontig:ASM35022v2:HG002339:232536:234399:-1
gene:CHC_T00007799001 transcript:CDF41286
gene_biotype:protein_coding transcript_biotype:protein_coding
Chondrus_crispus OG0000004
CDF36689 pep supercontig:ASM35022v2:HG001797:53444:54363:-1
gene:CHC_T00005071001 transcript:CDF36689
gene_biotype:protein_coding transcript_biotype:protein_coding
Chondrus_crispus OG0000004
CDF32131 pep supercontig:ASM35022v2:HG001459:265102:267154:1
gene:CHC_T00001366001 transcript:CDF32131
gene_biotype:protein_coding transcript_biotype:protein_coding
Chondrus_crispus OG0000004
CDF34124 pep supercontig:ASM35022v2:HG001674:17474:18734:1
gene:CHC_T00002816001 transcript:CDF34124
gene_biotype:protein_coding transcript_biotype:protein_coding
Chondrus_crispus OG0000004
g322.t1  Caulerpa_lentillifera    OG0000005
g323.t1  Caulerpa_lentillifera    OG0000005
g324.t1  Caulerpa_lentillifera    OG0000005
g325.t1  Caulerpa_lentillifera    OG0000005
g326.t1  Caulerpa_lentillifera    OG0000005
g332.t1  Caulerpa_lentillifera    OG0000005
g333.t1  Caulerpa_lentillifera    OG0000005
g334.t1  Caulerpa_lentillifera    OG0000005
g335.t1  Caulerpa_lentillifera    OG0000005
g336.t1  Caulerpa_lentillifera    OG0000005
g342.t1  Caulerpa_lentillifera    OG0000005
g347.t1  Caulerpa_lentillifera    OG0000005
g348.t1  Caulerpa_lentillifera    OG0000005
g362.t1  Caulerpa_lentillifera    OG0000005
g363.t1  Caulerpa_lentillifera    OG0000005
g3820.t1 Caulerpa_lentillifera    OG0000005
g3823.t1 Caulerpa_lentillifera    OG0000005
g3827.t1 Caulerpa_lentillifera    OG0000005
g3828.t1 Caulerpa_lentillifera    OG0000005
g3830.t1 Caulerpa_lentillifera    OG0000005
g4213.t1 Caulerpa_lentillifera    OG0000005
g4218.t1 Caulerpa_lentillifera    OG0000005
g4804.t1 Caulerpa_lentillifera    OG0000005
g499.t1  Caulerpa_lentillifera    OG0000005
g500.t1  Caulerpa_lentillifera    OG0000005
g6299.t1 Caulerpa_lentillifera    OG0000005
g690.t1  Caulerpa_lentillifera    OG0000005
g8395.t1 Caulerpa_lentillifera    OG0000005
g8419.t1 Caulerpa_lentillifera    OG0000005
Cre03.g161250.t1.2 pacid=30786699 transcript=Cre03.g161250.t1.2
locus=Cre03.g161250 ID=Cre03.g161250.t1.2.v5.5 annot-version=v5.5
```

Chlamydomonas_reinhardtii OG0000005
Cre07.g354450.t1.2 pacid=30774717 transcript=Cre07.g354450.t1.2
locus=Cre07.g354450 ID=Cre07.g354450.t1.2.v5.5 annot-version=v5.5
Chlamydomonas_reinhardtii OG0000005
Cre03.g166900.t1.1 pacid=30787369 transcript=Cre03.g166900.t1.1
locus=Cre03.g166900 ID=Cre03.g166900.t1.1.v5.5 annot-version=v5.5
Chlamydomonas_reinhardtii OG0000005
Cre07.g356250.t1.1 pacid=30774793 transcript=Cre07.g356250.t1.1
locus=Cre07.g356250 ID=Cre07.g356250.t1.1.v5.5 annot-version=v5.5
Chlamydomonas_reinhardtii OG0000005
Cre07.g354350.t1.1 pacid=30774885 transcript=Cre07.g354350.t1.1
locus=Cre07.g354350 ID=Cre07.g354350.t1.1.v5.5 annot-version=v5.5
Chlamydomonas_reinhardtii OG0000005
Cre01.g038500.t1.2 pacid=30788986 transcript=Cre01.g038500.t1.2
locus=Cre01.g038500 ID=Cre01.g038500.t1.2.v5.5 annot-version=v5.5
Chlamydomonas_reinhardtii OG0000005
Cre01.g054250.t1.2 pacid=30789532 transcript=Cre01.g054250.t1.2
locus=Cre01.g054250 ID=Cre01.g054250.t1.2.v5.5 annot-version=v5.5
Chlamydomonas_reinhardtii OG0000005
Cre04.g220387.t1.1 pacid=30790962 transcript=Cre04.g220387.t1.1
locus=Cre04.g220387 ID=Cre04.g220387.t1.1.v5.5 annot-version=v5.5
Chlamydomonas_reinhardtii OG0000005
Cre07.g340850.t1.2 pacid=30775252 transcript=Cre07.g340850.t1.2
locus=Cre07.g340850 ID=Cre07.g340850.t1.2.v5.5 annot-version=v5.5
Chlamydomonas_reinhardtii OG0000005
Cre14.g626400.t1.2 pacid=30776600 transcript=Cre14.g626400.t1.2
locus=Cre14.g626400 ID=Cre14.g626400.t1.2.v5.5 annot-version=v5.5
Chlamydomonas_reinhardtii OG0000005
Cre16.g648200.t1.2 pacid=30777983 transcript=Cre16.g648200.t1.2
locus=Cre16.g648200 ID=Cre16.g648200.t1.2.v5.5 annot-version=v5.5
Chlamydomonas_reinhardtii OG0000005
Cre16.g659200.t1.1 pacid=30778122 transcript=Cre16.g659200.t1.1
locus=Cre16.g659200 ID=Cre16.g659200.t1.1.v5.5 annot-version=v5.5
Chlamydomonas_reinhardtii OG0000005
Cre08.g384000.t1.1 pacid=30773909 transcript=Cre08.g384000.t1.1
locus=Cre08.g384000 ID=Cre08.g384000.t1.1.v5.5 annot-version=v5.5
Chlamydomonas_reinhardtii OG0000005
Cre09.g397216.t1.1 pacid=30780342 transcript=Cre09.g397216.t1.1
locus=Cre09.g397216 ID=Cre09.g397216.t1.1.v5.5 annot-version=v5.5
Chlamydomonas_reinhardtii OG0000005
Cre09.g397105.t1.1 pacid=30780474 transcript=Cre09.g397105.t1.1
locus=Cre09.g397105 ID=Cre09.g397105.t1.1.v5.5 annot-version=v5.5
Chlamydomonas_reinhardtii OG0000005
Cre09.g397734.t1.1 pacid=30780569 transcript=Cre09.g397734.t1.1
locus=Cre09.g397734 ID=Cre09.g397734.t1.1.v5.5 annot-version=v5.5
Chlamydomonas_reinhardtii OG0000005
Cre09.g396994.t1.1 pacid=30781008 transcript=Cre09.g396994.t1.1
locus=Cre09.g396994 ID=Cre09.g396994.t1.1.v5.5 annot-version=v5.5
Chlamydomonas_reinhardtii OG0000005
Cre09.g397031.t1.1 pacid=30781486 transcript=Cre09.g397031.t1.1
locus=Cre09.g397031 ID=Cre09.g397031.t1.1.v5.5 annot-version=v5.5
Chlamydomonas_reinhardtii OG0000005
Cre07.g354400.t1.1 pacid=30774320 transcript=Cre07.g354400.t1.1
locus=Cre07.g354400 ID=Cre07.g354400.t1.1.v5.5 annot-version=v5.5

Chlamydomonas_reinhardtii OG0000005
Vocar.0008s0122.1.p pacid=32897012 transcript=Vocar.0008s0122.1 locus=Vocar.0008s0122 ID=Vocar.0008s0122.1.v2.1 annot-version=v2.1 Volvox_carteri OG0000005
Vocar.0001s0733.1.p pacid=32885440 transcript=Vocar.0001s0733.1 locus=Vocar.0001s0733 ID=Vocar.0001s0733.1.v2.1 annot-version=v2.1 Volvox_carteri OG0000005
Vocar.0040s0108.1.p pacid=32886198 transcript=Vocar.0040s0108.1 locus=Vocar.0040s0108 ID=Vocar.0040s0108.1.v2.1 annot-version=v2.1 Volvox_carteri OG0000005
Vocar.0051s0007.1.p pacid=32886995 transcript=Vocar.0051s0007.1 locus=Vocar.0051s0007 ID=Vocar.0051s0007.1.v2.1 annot-version=v2.1 Volvox_carteri OG0000005
Vocar.0005s0294.1.p pacid=32883813 transcript=Vocar.0005s0294.1 locus=Vocar.0005s0294 ID=Vocar.0005s0294.1.v2.1 annot-version=v2.1 Volvox_carteri OG0000005
Vocar.0004s0044.1.p pacid=32889635 transcript=Vocar.0004s0044.1 locus=Vocar.0004s0044 ID=Vocar.0004s0044.1.v2.1 annot-version=v2.1 Volvox_carteri OG0000005
Vocar.0067s0014.1.p pacid=32884137 transcript=Vocar.0067s0014.1 locus=Vocar.0067s0014 ID=Vocar.0067s0014.1.v2.1 annot-version=v2.1 Volvox_carteri OG0000005
Vocar.0009s0201.1.p pacid=32893308 transcript=Vocar.0009s0201.1 locus=Vocar.0009s0201 ID=Vocar.0009s0201.1.v2.1 annot-version=v2.1 Volvox_carteri OG0000005
jgi|ChlNC64A_1|49396|fgenesh3_pg.C_scaffold_1000094 Chlorella_variabilis OG0000005
jgi|ChlNC64A_1|135475|IGS.gm_14_00008 Chlorella_variabilis OG0000005
jgi|ChlNC64A_1|51282|fgenesh3_pg.C_scaffold_6000096 Chlorella_variabilis OG0000005
jgi|ChlNC64A_1|57715|estExt_fgenesh3_pg.C_90039 Chlorella_variabilis OG0000005
jgi|ChlNC64A_1|34731|estExt_Genewise1Plus.C_50379 Chlorella_variabilis OG0000005
jgi|ChlNC64A_1|57280|estExt_fgenesh3_pg.C_50207 Chlorella_variabilis OG0000005
63249 pacid=27386838 transcript=63249 locus=Genemark1.4032_g ID=63249.2.0.227 annot-version=v2.0 Coccomyxa_subellipsoidea OG0000005
65414 pacid=27387223 transcript=65414 locus=estExt_Genemark1.C_50176 ID=65414.2.0.227 annot-version=v2.0 Coccomyxa_subellipsoidea OG0000005
42612 pacid=27385820 transcript=42612 locus=fgenesh1_pg.10_#_115 ID=42612.2.0.227 annot-version=v2.0 Coccomyxa_subellipsoidea OG0000005
65415 pacid=27387534 transcript=65415 locus=estExt_Genemark1.C_50178 ID=65415.2.0.227 annot-version=v2.0 Coccomyxa_subellipsoidea OG0000005
9001 pacid=27387596 transcript=9001 locus=gw1.22.177.1 ID=9001.2.0.227 annot-version=v2.0 Coccomyxa_subellipsoidea OG0000005
42702 pacid=27385854 transcript=42702 locus=fgenesh1_pg.10_#_205 ID=42702.2.0.227 annot-version=v2.0 Coccomyxa_subellipsoidea

OG0000005
67174 pacid=27387899 transcript=67174
locus=estExt_Genemark1.C_140035 ID=67174.2.0.227 annot-version=v2.0
Coccomyxa_subellipsoidea OG0000005
67237 pacid=27387912 transcript=67237
locus=estExt_Genemark1.C_140156 ID=67237.2.0.227 annot-version=v2.0
Coccomyxa_subellipsoidea OG0000005
67847 pacid=27388428 transcript=67847
locus=estExt_Genemark1.C_190065 ID=67847.2.0.227 annot-version=v2.0
Coccomyxa_subellipsoidea OG0000005
48278 pacid=27386088 transcript=48278
locus=estExt_fgenesh1_pg.C_130097 ID=48278.2.0.227 annot-
version=v2.0 Coccomyxa_subellipsoidea OG0000005
39976 pacid=27390645 transcript=39976 locus=fgenesh1_pg.3_#_37
ID=39976.2.0.227 annot-version=v2.0 Coccomyxa_subellipsoidea
OG0000005
56773 pacid=27391459 transcript=56773 locus=Genemark1.5802_g
ID=56773.2.0.227 annot-version=v2.0 Coccomyxa_subellipsoidea
OG0000005
43257 pacid=27391483 transcript=43257 locus=fgenesh1_pg.12_#_196
ID=43257.2.0.227 annot-version=v2.0 Coccomyxa_subellipsoidea
OG0000005
66732 pacid=27392045 transcript=66732
locus=estExt_Genemark1.C_110173 ID=66732.2.0.227 annot-version=v2.0
Coccomyxa_subellipsoidea OG0000005
63452 pacid=27392618 transcript=63452 locus=Genemark1.4235_g
ID=63452.2.0.227 annot-version=v2.0 Coccomyxa_subellipsoidea
OG0000005
59547 pacid=27392896 transcript=59547 locus=Genemark1.7863_g
ID=59547.2.0.227 annot-version=v2.0 Coccomyxa_subellipsoidea
OG0000005
44844 pacid=27393013 transcript=44844 locus=fgenesh1_pg.20_#_43
ID=44844.2.0.227 annot-version=v2.0 Coccomyxa_subellipsoidea
OG0000005
49034 pacid=27393016 transcript=49034
locus=estExt_fgenesh1_pg.C_200130 ID=49034.2.0.227 annot-
version=v2.0 Coccomyxa_subellipsoidea OG0000005
39702 pacid=27393287 transcript=39702 locus=fgenesh1_pg.2_#_314
ID=39702.2.0.227 annot-version=v2.0 Coccomyxa_subellipsoidea
OG0000005
59196 pacid=27393454 transcript=59196 locus=Genemark1.1158_g
ID=59196.2.0.227 annot-version=v2.0 Coccomyxa_subellipsoidea
OG0000005
57964 pacid=27394053 transcript=57964 locus=Genemark1.6993_g
ID=57964.2.0.227 annot-version=v2.0 Coccomyxa_subellipsoidea
OG0000005
18864 pacid=27394064 transcript=18864 locus=e_gw1.16.81.1
ID=18864.2.0.227 annot-version=v2.0 Coccomyxa_subellipsoidea
OG0000005
19124 pacid=27394623 transcript=19124 locus=e_gw1.17.75.1
ID=19124.2.0.227 annot-version=v2.0 Coccomyxa_subellipsoidea
OG0000005
58178 pacid=27394704 transcript=58178 locus=Genemark1.7207_g
ID=58178.2.0.227 annot-version=v2.0 Coccomyxa_subellipsoidea

OG0000005
48580 pacid=27394921 transcript=48580
locus=estExt_fgenesh1_pg.C_150193 ID=48580.2.0.227 annot-version=v2.0	Coccomyxa_subellipsoidea	OG0000005
10325_fgenesh1_pg.C_Chr_03.0001000270	Ostreococcus_tauri
OG0000005
23029_estExt_fgenesh1_pm.C_Chr_13.00010043	Ostreococcus_tauri
OG0000005
30690 pacid=27413216 transcript=30690 locus=eugene.0300010366
ID=30690.2.0.231 annot-version=v2.0	Ostreococcus_lucimarinus
OG0000005
52070 pacid=27349094 transcript=52070 locus=MicpuC2.EuGene.
0000120135 ID=52070.3.0.228 annot-version=v3.0
Micromonas_pusilla	OG0000005
93908 pacid=27400515 transcript=93908
locus=estExt_Genewise2Plus.C_Chr_040155 ID=93908.3.0.229 annot-version=v3.0	Micromonas_sp._RCC299	OG0000005
kfl00437_0060_v1.1	Klebsormidium_flaccidum	OG0000005
kfl00468_0120_v1.1	Klebsormidium_flaccidum	OG0000005
kfl00471_0090_v1.1	Klebsormidium_flaccidum	OG0000005
kfl00473_0030_v1.1	Klebsormidium_flaccidum	OG0000005
kfl00473_0040_v1.1	Klebsormidium_flaccidum	OG0000005
kfl00018_0070_v1.1	Klebsormidium_flaccidum	OG0000005
kfl00588_0070_v1.1	Klebsormidium_flaccidum	OG0000005
kfl00035_0080_v1.1	Klebsormidium_flaccidum	OG0000005
kfl00038_0230_v1.1	Klebsormidium_flaccidum	OG0000005
kfl00043_0360_v1.1	Klebsormidium_flaccidum	OG0000005
kfl00161_0090_v1.1	Klebsormidium_flaccidum	OG0000005
kfl00010_0080_v1.1	Klebsormidium_flaccidum	OG0000005
kfl00188_0250_v1.1	Klebsormidium_flaccidum	OG0000005
kfl00191_0130_v1.1	Klebsormidium_flaccidum	OG0000005
kfl00209_0130_v1.1	Klebsormidium_flaccidum	OG0000005
kfl00299_0120_v1.1	Klebsormidium_flaccidum	OG0000005
Pp3c15_21450V3.1.p pacid=32928913 transcript=Pp3c15_21450V3.1
locus=Pp3c15_21450 annot-version=v3.3	Physcomitrella_patens
OG0000005
Pp3c13_14870V3.1.p pacid=32930183 transcript=Pp3c13_14870V3.1
locus=Pp3c13_14870 annot-version=v3.3	Physcomitrella_patens
OG0000005
Pp3c2_30400V3.1.p pacid=32934078 transcript=Pp3c2_30400V3.1
locus=Pp3c2_30400 annot-version=v3.3	Physcomitrella_patens
OG0000005
Pp3c2_30580V3.1.p pacid=32934555 transcript=Pp3c2_30580V3.1
locus=Pp3c2_30580 annot-version=v3.3	Physcomitrella_patens
OG0000005
Pp3c2_24000V3.1.p pacid=32934842 transcript=Pp3c2_24000V3.1
locus=Pp3c2_24000 annot-version=v3.3	Physcomitrella_patens
OG0000005
Pp3c2_37410V3.1.p pacid=32935303 transcript=Pp3c2_37410V3.1
locus=Pp3c2_37410 annot-version=v3.3	Physcomitrella_patens
OG0000005
Pp3c3_20750V3.1.p pacid=32940380 transcript=Pp3c3_20750V3.1
locus=Pp3c3_20750 annot-version=v3.3	Physcomitrella_patens
OG0000005

Pp3c3_17840V3.1.p pacid=32940458 transcript=Pp3c3_17840V3.1
locus=Pp3c3_17840 annot-version=v3.3 Physcomitrella_patens
OG0000005
Pp3c3_3050V3.1.p pacid=32941641 transcript=Pp3c3_3050V3.1
locus=Pp3c3_3050 annot-version=v3.3 Physcomitrella_patens
OG0000005
Pp3c3_26120V3.1.p pacid=32942623 transcript=Pp3c3_26120V3.1
locus=Pp3c3_26120 annot-version=v3.3 Physcomitrella_patens
OG0000005
Pp3c3_32210V3.1.p pacid=32943066 transcript=Pp3c3_32210V3.1
locus=Pp3c3_32210 annot-version=v3.3 Physcomitrella_patens
OG0000005
Pp3c22_19580V3.1.p pacid=32904236 transcript=Pp3c22_19580V3.1
locus=Pp3c22_19580 annot-version=v3.3 Physcomitrella_patens
OG0000005
Pp3c23_6470V3.1.p pacid=32949734 transcript=Pp3c23_6470V3.1
locus=Pp3c23_6470 annot-version=v3.3 Physcomitrella_patens
OG0000005
Pp3c22_19010V3.1.p pacid=32904654 transcript=Pp3c22_19010V3.1
locus=Pp3c22_19010 annot-version=v3.3 Physcomitrella_patens
OG0000005
Pp3c23_6540V3.1.p pacid=32950991 transcript=Pp3c23_6540V3.1
locus=Pp3c23_6540 annot-version=v3.3 Physcomitrella_patens
OG0000005
Pp3c27_5310V3.1.p pacid=32951683 transcript=Pp3c27_5310V3.1
locus=Pp3c27_5310 annot-version=v3.3 Physcomitrella_patens
OG0000005
Pp3c27_4440V3.1.p pacid=32951824 transcript=Pp3c27_4440V3.1
locus=Pp3c27_4440 annot-version=v3.3 Physcomitrella_patens
OG0000005
Pp3c27_5460V3.1.p pacid=32952082 transcript=Pp3c27_5460V3.1
locus=Pp3c27_5460 annot-version=v3.3 Physcomitrella_patens
OG0000005
Pp3c27_8510V3.1.p pacid=32952144 transcript=Pp3c27_8510V3.1
locus=Pp3c27_8510 annot-version=v3.3 Physcomitrella_patens
OG0000005
Pp3c27_4400V3.1.p pacid=32952197 transcript=Pp3c27_4400V3.1
locus=Pp3c27_4400 annot-version=v3.3 Physcomitrella_patens
OG0000005
Pp3c5_7340V3.1.p pacid=32954132 transcript=Pp3c5_7340V3.1
locus=Pp3c5_7340 annot-version=v3.3 Physcomitrella_patens
OG0000005
Pp3c5_14800V3.1.p pacid=32955063 transcript=Pp3c5_14800V3.1
locus=Pp3c5_14800 annot-version=v3.3 Physcomitrella_patens
OG0000005
Pp3c11_470V3.1.p pacid=32956947 transcript=Pp3c11_470V3.1
locus=Pp3c11_470 annot-version=v3.3 Physcomitrella_patens
OG0000005
Pp3c11_13670V3.1.p pacid=32957127 transcript=Pp3c11_13670V3.1
locus=Pp3c11_13670 annot-version=v3.3 Physcomitrella_patens
OG0000005
Pp3c11_6580V3.1.p pacid=32957148 transcript=Pp3c11_6580V3.1
locus=Pp3c11_6580 annot-version=v3.3 Physcomitrella_patens
OG0000005

Pp3c11_7340V3.1.p pacid=32958109 transcript=Pp3c11_7340V3.1
locus=Pp3c11_7340 annot-version=v3.3 Physcomitrella_patens
OG0000005
Pp3c8_21260V3.1.p pacid=32966358 transcript=Pp3c8_21260V3.1
locus=Pp3c8_21260 annot-version=v3.3 Physcomitrella_patens
OG0000005
Pp3s124_100V3.1.p pacid=32966525 transcript=Pp3s124_100V3.1
locus=Pp3s124_100 annot-version=v3.3 Physcomitrella_patens
OG0000005
Pp3c1_38740V3.1.p pacid=32967237 transcript=Pp3c1_38740V3.1
locus=Pp3c1_38740 annot-version=v3.3 Physcomitrella_patens
OG0000005
Pp3c1_33340V3.1.p pacid=32967494 transcript=Pp3c1_33340V3.1
locus=Pp3c1_33340 annot-version=v3.3 Physcomitrella_patens
OG0000005
Pp3c1_1880V3.1.p pacid=32968205 transcript=Pp3c1_1880V3.1
locus=Pp3c1_1880 annot-version=v3.3 Physcomitrella_patens
OG0000005
Pp3c1_23870V3.1.p pacid=32969269 transcript=Pp3c1_23870V3.1
locus=Pp3c1_23870 annot-version=v3.3 Physcomitrella_patens
OG0000005
Pp3c1_39440V3.1.p pacid=32969914 transcript=Pp3c1_39440V3.1
locus=Pp3c1_39440 annot-version=v3.3 Physcomitrella_patens
OG0000005
Pp3c12_25250V3.1.p pacid=32972818 transcript=Pp3c12_25250V3.1
locus=Pp3c12_25250 annot-version=v3.3 Physcomitrella_patens
OG0000005
Pp3c12_8440V3.1.p pacid=32973071 transcript=Pp3c12_8440V3.1
locus=Pp3c12_8440 annot-version=v3.3 Physcomitrella_patens
OG0000005
Pp3c12_6560V3.1.p pacid=32974501 transcript=Pp3c12_6560V3.1
locus=Pp3c12_6560 annot-version=v3.3 Physcomitrella_patens
OG0000005
Pp3c6_19010V3.1.p pacid=32977132 transcript=Pp3c6_19010V3.1
locus=Pp3c6_19010 annot-version=v3.3 Physcomitrella_patens
OG0000005
Pp3c25_15450V3.1.p pacid=32979545 transcript=Pp3c25_15450V3.1
locus=Pp3c25_15450 annot-version=v3.3 Physcomitrella_patens
OG0000005
Pp3c18_21260V3.1.p pacid=32983148 transcript=Pp3c18_21260V3.1
locus=Pp3c18_21260 annot-version=v3.3 Physcomitrella_patens
OG0000005
Pp3c16_21250V3.1.p pacid=32984212 transcript=Pp3c16_21250V3.1
locus=Pp3c16_21250 annot-version=v3.3 Physcomitrella_patens
OG0000005
Pp3c16_5820V3.1.p pacid=32984635 transcript=Pp3c16_5820V3.1
locus=Pp3c16_5820 annot-version=v3.3 Physcomitrella_patens
OG0000005
Pp3c17_21950V3.1.p pacid=32908075 transcript=Pp3c17_21950V3.1
locus=Pp3c17_21950 annot-version=v3.3 Physcomitrella_patens
OG0000005
Pp3c16_630V3.1.p pacid=32985015 transcript=Pp3c16_630V3.1
locus=Pp3c16_630 annot-version=v3.3 Physcomitrella_patens
OG0000005

Pp3c17_14010V3.1.p pacid=32908498 transcript=Pp3c17_14010V3.1 locus=Pp3c17_14010 annot-version=v3.3	Physcomitrella_patens OG0000005
Pp3c24_17830V3.1.p pacid=32909342 transcript=Pp3c24_17830V3.1 locus=Pp3c24_17830 annot-version=v3.3	Physcomitrella_patens OG0000005
Pp3c24_15550V3.1.p pacid=32910563 transcript=Pp3c24_15550V3.1 locus=Pp3c24_15550 annot-version=v3.3	Physcomitrella_patens OG0000005
Pp3c9_20580V3.1.p pacid=32913384 transcript=Pp3c9_20580V3.1 locus=Pp3c9_20580 annot-version=v3.3	Physcomitrella_patens OG0000005
Pp3c21_19760V3.1.p pacid=32914908 transcript=Pp3c21_19760V3.1 locus=Pp3c21_19760 annot-version=v3.3	Physcomitrella_patens OG0000005
Pp3c21_10970V3.1.p pacid=32915206 transcript=Pp3c21_10970V3.1 locus=Pp3c21_10970 annot-version=v3.3	Physcomitrella_patens OG0000005
Pp3c21_9750V3.1.p pacid=32916792 transcript=Pp3c21_9750V3.1 locus=Pp3c21_9750 annot-version=v3.3	Physcomitrella_patens OG0000005
Pp3c26_6560V3.1.p pacid=32918162 transcript=Pp3c26_6560V3.1 locus=Pp3c26_6560 annot-version=v3.3	Physcomitrella_patens OG0000005
Pp3c4_30340V3.1.p pacid=32922788 transcript=Pp3c4_30340V3.1 locus=Pp3c4_30340 annot-version=v3.3	Physcomitrella_patens OG0000005
Pp3c4_30670V3.1.p pacid=32922810 transcript=Pp3c4_30670V3.1 locus=Pp3c4_30670 annot-version=v3.3	Physcomitrella_patens OG0000005
Pp3c7_17090V3.1.p pacid=32924268 transcript=Pp3c7_17090V3.1 locus=Pp3c7_17090 annot-version=v3.3	Physcomitrella_patens OG0000005
Pp3c7_3710V3.1.p pacid=32924505 transcript=Pp3c7_3710V3.1 locus=Pp3c7_3710 annot-version=v3.3	Physcomitrella_patens OG0000005
Pp3c7_22790V3.1.p pacid=32925322 transcript=Pp3c7_22790V3.1 locus=Pp3c7_22790 annot-version=v3.3	Physcomitrella_patens OG0000005
AT2G40890.1 | cytochrome P450%2C family 98%2C subfamily A%2C polypeptide 3 | Chr2:17058291-17060532 REVERSE LENGTH=508 | 201606 Arabidopsis_thaliana	OG0000005
AT2G42250.1 | cytochrome P450%2C family 712%2C subfamily A%2C polypeptide 1 | Chr2:17600075-17601709 REVERSE LENGTH=514 | 201606 Arabidopsis_thaliana	OG0000005
AT2G45550.1 | cytochrome P450%2C family 76%2C subfamily C%2C polypeptide 4 | Chr2:18773541-18775654 REVERSE LENGTH=511 | 201606 Arabidopsis_thaliana	OG0000005
AT2G45560.1 | cytochrome P450%2C family 76%2C subfamily C%2C polypeptide 1 | Chr2:18776391-18778354 REVERSE LENGTH=512 | 201606 Arabidopsis_thaliana	OG0000005
AT2G45570.1 | cytochrome P450%2C family 76%2C subfamily C%2C polypeptide 2 | Chr2:18779935-18781922 REVERSE LENGTH=512 | 201606 Arabidopsis_thaliana	OG0000005

AT2G45580.1 | cytochrome P450%2C family 76%2C subfamily C%2C polypeptide 3 | Chr2:18782388-18784286 REVERSE LENGTH=515 | 201606 Arabidopsis_thaliana OG0000005
AT2G46660.1 | cytochrome P450%2C family 78%2C subfamily A%2C polypeptide 6 | Chr2:19153602-19155417 REVERSE LENGTH=530 | 201606 Arabidopsis_thaliana OG0000005
AT2G46950.1 | cytochrome P450%2C family 709%2C subfamily B%2C polypeptide 2 | Chr2:19289466-19291541 REVERSE LENGTH=572 | 201606 Arabidopsis_thaliana OG0000005
AT2G46960.1 | cytochrome P450%2C family 709%2C subfamily B%2C polypeptide 1 | Chr2:19292295-19293765 REVERSE LENGTH=403 | 201606 Arabidopsis_thaliana OG0000005
AT1G11600.1 | cytochrome P450%2C family 77%2C subfamily B%2C polypeptide 1 | Chr1:3902090-3903622 FORWARD LENGTH=510 | 201606 Arabidopsis_thaliana OG0000005
AT1G11610.1 | cytochrome P450%2C family 71%2C subfamily A%2C polypeptide 18 | Chr1:3907461-3909291 REVERSE LENGTH=497 | 201606 Arabidopsis_thaliana OG0000005
AT3G03470.1 | cytochrome P450%2C family 87%2C subfamily A%2C polypeptide 9 | Chr3:824692-826345 REVERSE LENGTH=511 | 201606 Arabidopsis_thaliana OG0000005
AT3G10560.1 | Cytochrome P450 superfamily protein | Chr3:3299939-3301483 FORWARD LENGTH=514 | 201606 Arabidopsis_thaliana OG0000005
AT3G10570.1 | cytochrome P450%2C family 77%2C subfamily A%2C polypeptide 6 | Chr3:3302156-3303697 FORWARD LENGTH=513 | 201606 Arabidopsis_thaliana OG0000005
AT3G14610.1 | cytochrome P450%2C family 72%2C subfamily A%2C polypeptide 7 | Chr3:4912565-4914503 FORWARD LENGTH=512 | 201606 Arabidopsis_thaliana OG0000005
AT3G14620.1 | cytochrome P450%2C family 72%2C subfamily A%2C polypeptide 8 | Chr3:4914978-4916853 FORWARD LENGTH=515 | 201606 Arabidopsis_thaliana OG0000005
AT3G14630.1 | cytochrome P450%2C family 72%2C subfamily A%2C polypeptide 9 | Chr3:4917498-4919409 FORWARD LENGTH=508 | 201606 Arabidopsis_thaliana OG0000005
AT3G14640.1 | cytochrome P450%2C family 72%2C subfamily A%2C polypeptide 10 | Chr3:4919856-4921787 FORWARD LENGTH=514 | 201606 Arabidopsis_thaliana OG0000005
AT3G14650.1 | cytochrome P450%2C family 72%2C subfamily A%2C polypeptide 11 | Chr3:4922206-4924165 FORWARD LENGTH=512 | 201606 Arabidopsis_thaliana OG0000005
AT3G14660.1 | cytochrome P450%2C family 72%2C subfamily A%2C polypeptide 13 | Chr3:4924960-4926911 FORWARD LENGTH=512 | 201606 Arabidopsis_thaliana OG0000005
AT3G14680.1 | cytochrome P450%2C family 72%2C subfamily A%2C polypeptide 14 | Chr3:4934478-4936462 FORWARD LENGTH=512 | 201606 Arabidopsis_thaliana OG0000005
AT3G14690.1 | cytochrome P450%2C family 72%2C subfamily A%2C polypeptide 15 | Chr3:4937410-4939310 FORWARD LENGTH=512 | 201606 Arabidopsis_thaliana OG0000005
AT1G13080.1 | cytochrome P450%2C family 71%2C subfamily B%2C polypeptide 2 | Chr1:4459212-4460807 FORWARD LENGTH=502 | 201606 Arabidopsis_thaliana OG0000005

AT1G13090.1 | cytochrome P450%2C family 71%2C subfamily B%2C polypeptide 28 | Chr1:4461846-4462742 FORWARD LENGTH=298 | 201606 Arabidopsis_thaliana OG0000005
AT1G13100.1 | cytochrome P450%2C family 71%2C subfamily B%2C polypeptide 29 | Chr1:4463983-4465538 FORWARD LENGTH=490 | 201606 Arabidopsis_thaliana OG0000005
AT1G13110.1 | cytochrome P450%2C family 71 subfamily B%2C polypeptide 7 | Chr1:4467272-4468857 FORWARD LENGTH=504 | 201606 Arabidopsis_thaliana OG0000005
AT3G20080.1 | cytochrome P450%2C family 705%2C subfamily A%2C polypeptide 15 | Chr3:7008813-7010463 FORWARD LENGTH=523 | 201606 Arabidopsis_thaliana OG0000005
AT3G20090.1 | cytochrome P450%2C family 705%2C subfamily A%2C polypeptide 18 | Chr3:7017046-7018287 FORWARD LENGTH=386 | 201606 Arabidopsis_thaliana OG0000005
AT3G20100.1 | cytochrome P450%2C family 705%2C subfamily A%2C polypeptide 19 | Chr3:7019014-7020649 FORWARD LENGTH=513 | 201606 Arabidopsis_thaliana OG0000005
AT3G20110.1 | cytochrome P450%2C family 705%2C subfamily A%2C polypeptide 20 | Chr3:7021495-7023113 FORWARD LENGTH=510 | 201606 Arabidopsis_thaliana OG0000005
AT3G20120.1 | cytochrome P450%2C family 705%2C subfamily A%2C polypeptide 21 | Chr3:7024576-7025789 FORWARD LENGTH=378 | 201606 Arabidopsis_thaliana OG0000005
AT3G20130.1 | cytochrome P450%2C family 705%2C subfamily A%2C polypeptide 22 | Chr3:7026982-7028613 FORWARD LENGTH=515 | 201606 Arabidopsis_thaliana OG0000005
AT3G20140.1 | cytochrome P450%2C family 705%2C subfamily A%2C polypeptide 23 | Chr3:7029175-7030787 FORWARD LENGTH=510 | 201606 Arabidopsis_thaliana OG0000005
AT3G20935.1 | cytochrome P450%2C family 705%2C subfamily A%2C polypeptide 28 | Chr3:7334770-7335912 FORWARD LENGTH=348 | 201606 Arabidopsis_thaliana OG0000005
AT3G20940.1 | cytochrome P450%2C family 705%2C subfamily A%2C polypeptide 30 | Chr3:7339732-7341518 FORWARD LENGTH=523 | 201606 Arabidopsis_thaliana OG0000005
AT3G20950.1 | cytochrome P450%2C family 705%2C subfamily A%2C polypeptide 32 | Chr3:7342675-7344744 FORWARD LENGTH=526 | 201606 Arabidopsis_thaliana OG0000005
AT3G20960.1 | cytochrome P450%2C family 705%2C subfamily A%2C polypeptide 33 | Chr3:7345672-7347014 FORWARD LENGTH=418 | 201606 Arabidopsis_thaliana OG0000005
AT1G13710.1 | cytochrome P450%2C family 78%2C subfamily A%2C polypeptide 5 | Chr1:4702932-4704592 REVERSE LENGTH=517 | 201606 Arabidopsis_thaliana OG0000005
AT3G25180.1 | cytochrome P450%2C family 82%2C subfamily G%2C polypeptide 1 | Chr3:9167443-9169270 REVERSE LENGTH=515 | 201606 Arabidopsis_thaliana OG0000005
AT3G26150.1 | cytochrome P450%2C family 71%2C subfamily B%2C polypeptide 16 | Chr3:9565627-9567212 REVERSE LENGTH=502 | 201606 Arabidopsis_thaliana OG0000005
AT3G26160.1 | cytochrome P450%2C family 71%2C subfamily B%2C polypeptide 17 | Chr3:9568280-9569871 REVERSE LENGTH=502 | 201606 Arabidopsis_thaliana OG0000005

AT3G26170.1 | cytochrome P450%2C family 71%2C subfamily B%2C polypeptide 19 | Chr3:9573542-9575129 REVERSE LENGTH=502 | 201606 Arabidopsis_thaliana OG0000005
AT3G26180.1 | cytochrome P450%2C family 71%2C subfamily B%2C polypeptide 20 | Chr3:9578407-9579993 REVERSE LENGTH=502 | 201606 Arabidopsis_thaliana OG0000005
AT3G26190.1 | cytochrome P450%2C family 71%2C subfamily B%2C polypeptide 21 | Chr3:9583475-9585083 REVERSE LENGTH=499 | 201606 Arabidopsis_thaliana OG0000005
AT3G26200.1 | cytochrome P450%2C family 71%2C subfamily B%2C polypeptide 22 | Chr3:9589347-9590972 FORWARD LENGTH=500 | 201606 Arabidopsis_thaliana OG0000005
AT3G26210.1 | cytochrome P450%2C family 71%2C subfamily B%2C polypeptide 23 | Chr3:9593329-9595202 REVERSE LENGTH=501 | 201606 Arabidopsis_thaliana OG0000005
AT3G26220.1 | cytochrome P450%2C family 71%2C subfamily B%2C polypeptide 3 | Chr3:9596208-9597828 REVERSE LENGTH=501 | 201606 Arabidopsis_thaliana OG0000005
AT3G26230.1 | cytochrome P450%2C family 71%2C subfamily B%2C polypeptide 24 | Chr3:9598200-9599892 REVERSE LENGTH=498 | 201606 Arabidopsis_thaliana OG0000005
AT3G26270.1 | cytochrome P450%2C family 71%2C subfamily B%2C polypeptide 25 | Chr3:9627602-9629195 REVERSE LENGTH=501 | 201606 Arabidopsis_thaliana OG0000005
AT3G26280.1 | cytochrome P450%2C family 71%2C subfamily B%2C polypeptide 4 | Chr3:9630358-9631970 REVERSE LENGTH=504 | 201606 Arabidopsis_thaliana OG0000005
AT3G26290.1 | cytochrome P450%2C family 71%2C subfamily B%2C polypeptide 26 | Chr3:9632770-9634439 REVERSE LENGTH=500 | 201606 Arabidopsis_thaliana OG0000005
AT3G26300.1 | cytochrome P450%2C family 71%2C subfamily B%2C polypeptide 34 | Chr3:9639199-9640866 REVERSE LENGTH=500 | 201606 Arabidopsis_thaliana OG0000005
AT3G26310.1 | cytochrome P450%2C family 71%2C subfamily B%2C polypeptide 35 | Chr3:9641089-9642779 REVERSE LENGTH=500 | 201606 Arabidopsis_thaliana OG0000005
AT3G26320.1 | cytochrome P450%2C family 71%2C subfamily B%2C polypeptide 36 | Chr3:9644383-9646064 REVERSE LENGTH=500 | 201606 Arabidopsis_thaliana OG0000005
AT3G26330.1 | cytochrome P450%2C family 71%2C subfamily B%2C polypeptide 37 | Chr3:9646873-9648536 REVERSE LENGTH=500 | 201606 Arabidopsis_thaliana OG0000005
AT3G26830.1 | Cytochrome P450 superfamily protein | Chr3:9887990-9889560 FORWARD LENGTH=490 | 201606 Arabidopsis_thaliana OG0000005
AT3G28740.1 | Cytochrome P450 superfamily protein | Chr3:10788764-10790552 REVERSE LENGTH=509 | 201606 Arabidopsis_thaliana OG0000005
AT3G32047.1 | Cytochrome P450 superfamily protein | Chr3:13061048-13062710 FORWARD LENGTH=502 | 201606 Arabidopsis_thaliana OG0000005
AT3G44250.1 | cytochrome P450%2C family 71%2C subfamily B%2C polypeptide 38 | Chr3:15948505-15950224 REVERSE LENGTH=499 | 201606 Arabidopsis_thaliana OG0000005

AT3G48270.1 | cytochrome P450%2C family 71%2C subfamily A%2C polypeptide 26 | Chr3:17876571-17878173 FORWARD LENGTH=489 | 201606 Arabidopsis_thaliana OG0000005
AT3G48280.1 | cytochrome P450%2C family 71%2C subfamily A%2C polypeptide 25 | Chr3:17879594-17881164 FORWARD LENGTH=490 | 201606 Arabidopsis_thaliana OG0000005
AT3G48290.1 | cytochrome P450%2C family 71%2C subfamily A%2C polypeptide 24 | Chr3:17882590-17884134 FORWARD LENGTH=488 | 201606 Arabidopsis_thaliana OG0000005
AT3G48300.1 | cytochrome P450%2C family 71%2C subfamily A%2C polypeptide 23 | Chr3:17885524-17887118 FORWARD LENGTH=483 | 201606 Arabidopsis_thaliana OG0000005
AT3G48310.1 | cytochrome P450%2C family 71%2C subfamily A%2C polypeptide 22 | Chr3:17888192-17889749 FORWARD LENGTH=490 | 201606 Arabidopsis_thaliana OG0000005
AT3G48320.1 | cytochrome P450%2C family 71%2C subfamily A%2C polypeptide 21 | Chr3:17891241-17892804 FORWARD LENGTH=490 | 201606 Arabidopsis_thaliana OG0000005
AT3G52970.1 | cytochrome P450%2C family 76%2C subfamily G%2C polypeptide 1 | Chr3:19641400-19643259 REVERSE LENGTH=516 | 201606 Arabidopsis_thaliana OG0000005
AT3G53280.1 | cytochrome p450 71b5 | Chr3:19755749-19757466 FORWARD LENGTH=498 | 201606 Arabidopsis_thaliana OG0000005
AT3G53290.1 | cytochrome P450%2C family 71%2C subfamily B%2C polypeptide 30 pseudogene | Chr3:19758157-19759603 FORWARD LENGTH=407 | 201606 Arabidopsis_thaliana OG0000005
AT3G53300.1 | cytochrome P450%2C family 71%2C subfamily B%2C polypeptide 31 | Chr3:19760525-19762234 FORWARD LENGTH=498 | 201606 Arabidopsis_thaliana OG0000005
AT3G53305.1 | cytochrome P450%2C family 71%2C subfamily B%2C polypeptide 32 | Chr3:19763618-19765268 FORWARD LENGTH=338 | 201606 Arabidopsis_thaliana OG0000005
AT1G16400.1 | cytochrome P450%2C family 79%2C subfamily F%2C polypeptide 2 | Chr1:5605231-5607281 FORWARD LENGTH=537 | 201606 Arabidopsis_thaliana OG0000005
AT1G16410.1 | cytochrome p450 79f1 | Chr1:5608862-5611118 FORWARD LENGTH=538 | 201606 Arabidopsis_thaliana OG0000005
AT3G61040.1 | cytochrome P450%2C family 76%2C subfamily C%2C polypeptide 7 | Chr3:22594074-22596125 REVERSE LENGTH=498 | 201606 Arabidopsis_thaliana OG0000005
AT3G61880.1 | cytochrome p450 78a9 | Chr3:22906114-22907890 REVERSE LENGTH=534 | 201606 Arabidopsis_thaliana OG0000005
AT1G17060.1 | cytochrome p450 72c1 | Chr1:5832989-5835255 REVERSE LENGTH=313 | 201606 Arabidopsis_thaliana OG0000005
AT1G01190.1 | cytochrome P450%2C family 78%2C subfamily A%2C polypeptide 8 | Chr1:83045-84864 REVERSE LENGTH=535 | 201606 Arabidopsis_thaliana OG0000005
AT4G12300.1 | cytochrome P450%2C family 706%2C subfamily A%2C polypeptide 4 | Chr4:7308016-7309692 REVERSE LENGTH=516 | 201606 Arabidopsis_thaliana OG0000005
AT4G12310.1 | cytochrome P450%2C family 706%2C subfamily A%2C polypeptide 5 | Chr4:7310598-7312522 REVERSE LENGTH=520 | 201606 Arabidopsis_thaliana OG0000005
AT4G12320.1 | cytochrome P450%2C family 706%2C subfamily A%2C

polypeptide 6 | Chr4:7314939-7316647 REVERSE LENGTH=518 | 201606
Arabidopsis_thaliana   OG0000005
AT4G12330.1 | cytochrome P450%2C family 706%2C subfamily A%2C
polypeptide 7 | Chr4:7317776-7319658 REVERSE LENGTH=518 | 201606
Arabidopsis_thaliana   OG0000005
AT4G13290.1 | cytochrome P450%2C family 71%2C subfamily A%2C
polypeptide 19 | Chr4:7740681-7742670 FORWARD LENGTH=490 | 201606
Arabidopsis_thaliana   OG0000005
AT4G13310.1 | cytochrome P450%2C family 71%2C subfamily A%2C
polypeptide 20 | Chr4:7750453-7753049 FORWARD LENGTH=497 | 201606
Arabidopsis_thaliana   OG0000005
AT4G13770.1 | cytochrome P450%2C family 83%2C subfamily A%2C
polypeptide 1 | Chr4:7990682-7992282 REVERSE LENGTH=502 | 201606
Arabidopsis_thaliana   OG0000005
AT4G15330.1 | cytochrome P450%2C family 705%2C subfamily A%2C
polypeptide 1 | Chr4:8751523-8753134 REVERSE LENGTH=513 | 201606
Arabidopsis_thaliana   OG0000005
AT4G15350.1 | cytochrome P450%2C family 705%2C subfamily A%2C
polypeptide 2 | Chr4:8762953-8764563 FORWARD LENGTH=509 | 201606
Arabidopsis_thaliana   OG0000005
AT4G15360.1 | cytochrome P450%2C family 705%2C subfamily A%2C
polypeptide 3 | Chr4:8770185-8771852 FORWARD LENGTH=527 | 201606
Arabidopsis_thaliana   OG0000005
AT4G15380.1 | cytochrome P450%2C family 705%2C subfamily A%2C
polypeptide 4 | Chr4:8788762-8790391 FORWARD LENGTH=517 | 201606
Arabidopsis_thaliana   OG0000005
AT4G20240.1 | cytochrome P450%2C family 71%2C subfamily A%2C
polypeptide 27 | Chr4:10931745-10934212 REVERSE LENGTH=451 | 201606
Arabidopsis_thaliana   OG0000005
AT4G22690.1 | cytochrome P450%2C family 706%2C subfamily A%2C
polypeptide 1 | Chr4:11929847-11931520 FORWARD LENGTH=557 | 201606
Arabidopsis_thaliana   OG0000005
AT4G22710.1 | cytochrome P450%2C family 706%2C subfamily A%2C
polypeptide 2 | Chr4:11935038-11936618 FORWARD LENGTH=526 | 201606
Arabidopsis_thaliana   OG0000005
AT4G27710.1 | cytochrome P450%2C family 709%2C subfamily B%2C
polypeptide 3 | Chr4:13828520-13830417 FORWARD LENGTH=518 | 201606
Arabidopsis_thaliana   OG0000005
AT4G31500.1 | cytochrome P450%2C family 83%2C subfamily B%2C
polypeptide 1 | Chr4:15273677-15275271 REVERSE LENGTH=499 | 201606
Arabidopsis_thaliana   OG0000005
AT4G31940.1 | cytochrome P450%2C family 82%2C subfamily C%2C
polypeptide 4 | Chr4:15452040-15453966 FORWARD LENGTH=524 | 201606
Arabidopsis_thaliana   OG0000005
AT4G31950.1 | cytochrome P450%2C family 82%2C subfamily C%2C
polypeptide 3 | Chr4:15455163-15457090 FORWARD LENGTH=512 | 201606
Arabidopsis_thaliana   OG0000005
AT4G31970.1 | cytochrome P450%2C family 82%2C subfamily C%2C
polypeptide 2 | Chr4:15462408-15464358 FORWARD LENGTH=523 | 201606
Arabidopsis_thaliana   OG0000005
AT4G36220.1 | ferulic acid 5-hydroxylase 1 | Chr4:17137584-17139619
REVERSE LENGTH=520 | 201606   Arabidopsis_thaliana
OG0000005
AT4G37310.1 | cytochrome P450%2C family 81%2C subfamily H%2C

polypeptide 1 | Chr4:17556152-17558833 REVERSE LENGTH=518 | 201606
Arabidopsis_thaliana   OG0000005
AT4G37320.1 | cytochrome P450%2C family 81%2C subfamily D%2C
polypeptide 5 | Chr4:17559742-17561690 REVERSE LENGTH=495 | 201606
Arabidopsis_thaliana   OG0000005
AT4G37330.1 | cytochrome P450%2C family 81%2C subfamily D%2C
polypeptide 4 | Chr4:17562547-17564569 REVERSE LENGTH=492 | 201606
Arabidopsis_thaliana   OG0000005
AT4G37340.1 | cytochrome P450%2C family 81%2C subfamily D%2C
polypeptide 3 | Chr4:17564953-17566706 REVERSE LENGTH=500 | 201606
Arabidopsis_thaliana   OG0000005
AT4G37360.1 | cytochrome P450%2C family 81%2C subfamily D%2C
polypeptide 2 | Chr4:17567124-17568858 REVERSE LENGTH=499 | 201606
Arabidopsis_thaliana   OG0000005
AT4G37370.1 | cytochrome P450%2C family 81%2C subfamily D%2C
polypeptide 8 | Chr4:17569971-17571678 REVERSE LENGTH=497 | 201606
Arabidopsis_thaliana   OG0000005
AT4G37400.1 | cytochrome P450%2C family 81%2C subfamily F%2C
polypeptide 3 | Chr4:17584096-17586197 FORWARD LENGTH=501 | 201606
Arabidopsis_thaliana   OG0000005
AT4G37410.1 | cytochrome P450%2C family 81%2C subfamily F%2C
polypeptide 4 | Chr4:17590848-17592780 FORWARD LENGTH=501 | 201606
Arabidopsis_thaliana   OG0000005
AT4G37430.1 | cytochrome P450%2C family 91%2C subfamily A%2C
polypeptide 2 | Chr4:17597242-17598829 FORWARD LENGTH=500 | 201606
Arabidopsis_thaliana   OG0000005
AT5G04330.1 | Cytochrome P450 superfamily protein |
Chr5:1212695-1214310 REVERSE LENGTH=512 | 201606
Arabidopsis_thaliana   OG0000005
AT5G04630.1 | cytochrome P450%2C family 77%2C subfamily A%2C
polypeptide 9 | Chr5:1330578-1332107 FORWARD LENGTH=509 | 201606
Arabidopsis_thaliana   OG0000005
AT5G04660.1 | cytochrome P450%2C family 77%2C subfamily A%2C
polypeptide 4 | Chr5:1336049-1337587 FORWARD LENGTH=512 | 201606
Arabidopsis_thaliana   OG0000005
AT5G05260.1 | phenylalanine N-monooxygenase-like protein |
Chr5:1559778-1561783 REVERSE LENGTH=497 | 201606
Arabidopsis_thaliana   OG0000005
AT5G06900.1 | cytochrome P450%2C family 93%2C subfamily D%2C
polypeptide 1 | Chr5:2136160-2137925 REVERSE LENGTH=507 | 201606
Arabidopsis_thaliana   OG0000005
AT5G06905.1 | cytochrome P450%2C family 712%2C subfamily A%2C
polypeptide 2 | Chr5:2138438-2140078 REVERSE LENGTH=521 | 201606
Arabidopsis_thaliana   OG0000005
AT5G07990.1 | Cytochrome P450 superfamily protein |
Chr5:2560437-2562859 FORWARD LENGTH=513 | 201606
Arabidopsis_thaliana   OG0000005
AT5G09970.1 | cytochrome P450%2C family 78%2C subfamily A%2C
polypeptide 7 | Chr5:3112241-3113987 FORWARD LENGTH=536 | 201606
Arabidopsis_thaliana   OG0000005
AT5G10600.1 | cytochrome P450%2C family 81%2C subfamily K%2C
polypeptide 2 | Chr5:3351227-3352777 FORWARD LENGTH=516 | 201606
Arabidopsis_thaliana   OG0000005
AT5G10610.1 | cytochrome P450%2C family 81%2C subfamily K%2C

polypeptide 1 | Chr5:3353518-3355020 FORWARD LENGTH=500 | 201606 Arabidopsis_thaliana OG0000005
AT5G24900.1 | cytochrome P450%2C family 714%2C subfamily A%2C polypeptide 2 | Chr5:8563853-8566771 REVERSE LENGTH=525 | 201606 Arabidopsis_thaliana OG0000005
AT5G24910.1 | cytochrome P450%2C family 714%2C subfamily A%2C polypeptide 1 | Chr5:8567674-8570260 REVERSE LENGTH=532 | 201606 Arabidopsis_thaliana OG0000005
AT5G24950.1 | cytochrome P450%2C family 71%2C subfamily A%2C polypeptide 15 | Chr5:8595209-8597761 REVERSE LENGTH=496 | 201606 Arabidopsis_thaliana OG0000005
AT5G24960.1 | cytochrome P450%2C family 71%2C subfamily A%2C polypeptide 14 | Chr5:8599988-8603194 REVERSE LENGTH=497 | 201606 Arabidopsis_thaliana OG0000005
AT5G25120.1 | cytochrome p450%2C family 71%2C subfamily B%2C polypeptide 11 | Chr5:8662851-8664432 FORWARD LENGTH=496 | 201606 Arabidopsis_thaliana OG0000005
AT5G25130.1 | cytochrome P450%2C family 71%2C subfamily B%2C polypeptide 12 | Chr5:8668521-8670104 FORWARD LENGTH=496 | 201606 Arabidopsis_thaliana OG0000005
AT5G25140.1 | cytochrome P450%2C family 71%2C subfamily B%2C polypeptide 13 | Chr5:8672989-8674557 FORWARD LENGTH=496 | 201606 Arabidopsis_thaliana OG0000005
AT5G25180.1 | cytochrome P450%2C family 71%2C subfamily B%2C polypeptide 14 | Chr5:8694630-8696221 REVERSE LENGTH=496 | 201606 Arabidopsis_thaliana OG0000005
AT5G35715.1 | cytochrome P450%2C family 71%2C subfamily B%2C polypeptide 8 | Chr5:13881415-13882937 FORWARD LENGTH=442 | 201606 Arabidopsis_thaliana OG0000005
AT5G35917.1 | cytochrome P450%2C family 79%2C subfamily A%2C polypeptide 3 pseudogene | Chr5:14049123-14051128 FORWARD LENGTH=490 | 201606 Arabidopsis_thaliana OG0000005
AT5G35920.1 | cytochrome P450%2C family 79%2C subfamily A%2C polypeptide 4 pseudogene | Chr5:14056437-14056904 FORWARD LENGTH=155 | 201606 Arabidopsis_thaliana OG0000005
AT5G36220.1 | cytochrome p450 81d1 | Chr5:14253827-14256015 REVERSE LENGTH=502 | 201606 Arabidopsis_thaliana OG0000005
AT5G38450.1 | cytochrome P450%2C family 735%2C subfamily A%2C polypeptide 1 | Chr5:15394000-15397233 REVERSE LENGTH=518 | 201606 Arabidopsis_thaliana OG0000005
AT5G42580.1 | cytochrome P450%2C family 705%2C subfamily A%2C polypeptide 12 | Chr5:17023646-17025229 REVERSE LENGTH=499 | 201606 Arabidopsis_thaliana OG0000005
AT5G42590.1 | cytochrome P450%2C family 71%2C subfamily A%2C polypeptide 16 | Chr5:17031217-17033640 REVERSE LENGTH=497 | 201606 Arabidopsis_thaliana OG0000005
AT5G44620.1 | cytochrome P450%2C family 706%2C subfamily A%2C polypeptide 3 | Chr5:17997908-17999539 REVERSE LENGTH=519 | 201606 Arabidopsis_thaliana OG0000005
AT5G47990.1 | cytochrome P450%2C family 705%2C subfamily A%2C polypeptide 5 | Chr5:19434827-19436444 FORWARD LENGTH=511 | 201606 Arabidopsis_thaliana OG0000005
AT5G52400.1 | cytochrome P450%2C family 715%2C subfamily A%2C polypeptide 1 | Chr5:21272949-21275509 FORWARD LENGTH=519 | 201606

Arabidopsis_thaliana   OG0000005
AT5G57220.1 | cytochrome P450%2C family 81%2C subfamily F%2C polypeptide 2 | Chr5:23187911-23189681 FORWARD LENGTH=491 | 201606
Arabidopsis_thaliana   OG0000005
AT5G57260.1 | cytochrome P450%2C family 71%2C subfamily B%2C polypeptide 10 | Chr5:23198694-23200273 REVERSE LENGTH=502 | 201606
Arabidopsis_thaliana   OG0000005
AT5G61320.1 | cytochrome P450%2C family 89%2C subfamily A%2C polypeptide 3 | Chr5:24655098-24656638 REVERSE LENGTH=497 | 201606
Arabidopsis_thaliana   OG0000005
AT1G01280.1 | cytochrome P450%2C family 703%2C subfamily A%2C polypeptide 2 | Chr1:112290-113905 FORWARD LENGTH=510 | 201606
Arabidopsis_thaliana   OG0000005
AT5G67310.1 | cytochrome P450%2C family 81%2C subfamily G%2C polypeptide 1 | Chr5:26855313-26856924 REVERSE LENGTH=507 | 201606
Arabidopsis_thaliana   OG0000005
AT1G28430.1 | cytochrome P450%2C family 705%2C subfamily A%2C polypeptide 24 | Chr1:9992986-9994642 REVERSE LENGTH=521 | 201606
Arabidopsis_thaliana   OG0000005
AT1G33720.1 | cytochrome P450%2C family 76%2C subfamily C%2C polypeptide 6 | Chr1:12220957-12223981 REVERSE LENGTH=511 | 201606
Arabidopsis_thaliana   OG0000005
AT1G33730.1 | cytochrome P450%2C family 76%2C subfamily C%2C polypeptide 5 | Chr1:12227279-12228460 FORWARD LENGTH=368 | 201606
Arabidopsis_thaliana   OG0000005
AT1G50520.1 | cytochrome P450%2C family 705%2C subfamily A%2C polypeptide 27 | Chr1:18719381-18721070 FORWARD LENGTH=533 | 201606
Arabidopsis_thaliana   OG0000005
AT1G50560.1 | cytochrome P450%2C family 705%2C subfamily A%2C polypeptide 25 | Chr1:18724275-18725916 FORWARD LENGTH=519 | 201606
Arabidopsis_thaliana   OG0000005
AT1G58260.1 | cytochrome p450 79c2 | Chr1:21605752-21607995 FORWARD LENGTH=530 | 201606   Arabidopsis_thaliana   OG0000005
AT1G58265.1 | Cytochrome P450 superfamily protein | Chr1:21610909-21611388 FORWARD LENGTH=159 | 201606
Arabidopsis_thaliana   OG0000005
AT1G64900.1 | cytochrome P450%2C family 89%2C subfamily A%2C polypeptide 2 | Chr1:24113283-24114803 FORWARD LENGTH=506 | 201606
Arabidopsis_thaliana   OG0000005
AT1G64930.1 | cytochrome P450%2C family 87%2C subfamily A%2C polypeptide 7 | Chr1:24120926-24122461 FORWARD LENGTH=511 | 201606
Arabidopsis_thaliana   OG0000005
AT1G64940.1 | cytochrome P450%2C family 87%2C subfamily A%2C polypeptide 6 | Chr1:24123789-24125324 FORWARD LENGTH=511 | 201606
Arabidopsis_thaliana   OG0000005
AT1G64950.1 | cytochrome P450%2C family 89%2C subfamily A%2C polypeptide 5 | Chr1:24127587-24129119 FORWARD LENGTH=510 | 201606
Arabidopsis_thaliana   OG0000005
AT1G66540.1 | Cytochrome P450 superfamily protein | Chr1:24824837-24826502 FORWARD LENGTH=386 | 201606
Arabidopsis_thaliana   OG0000005
AT1G67110.1 | cytochrome P450%2C family 735%2C subfamily A%2C polypeptide 2 | Chr1:25061836-25065355 REVERSE LENGTH=512 | 201606
Arabidopsis_thaliana   OG0000005

AT1G74110.1 | cytochrome P450%2C family 78%2C subfamily A%2C polypeptide 10 | Chr1:27866667-27868368 REVERSE LENGTH=537 | 201606 Arabidopsis_thaliana OG0000005
AT1G74540.1 | cytochrome P450%2C family 98%2C subfamily A%2C polypeptide 8 | Chr1:28013362-28014855 FORWARD LENGTH=497 | 201606 Arabidopsis_thaliana OG0000005
AT1G74550.1 | cytochrome P450%2C family 98%2C subfamily A%2C polypeptide 9 | Chr1:28016086-28017549 FORWARD LENGTH=487 | 201606 Arabidopsis_thaliana OG0000005
AT1G75130.1 | cytochrome P450%2C family 721%2C subfamily A%2C polypeptide 1 | Chr1:28200073-28201911 REVERSE LENGTH=505 | 201606 Arabidopsis_thaliana OG0000005
AT1G79370.1 | cytochrome P450%2C family 79%2C subfamily C%2C polypeptide 1 | Chr1:29857934-29860163 FORWARD LENGTH=546 | 201606 Arabidopsis_thaliana OG0000005
AT2G02580.1 | cytochrome P450%2C family 71%2C subfamily B%2C polypeptide 9 | Chr2:701985-703661 FORWARD LENGTH=500 | 201606 Arabidopsis_thaliana OG0000005
AT2G05180.1 | cytochrome P450%2C family 705%2C subfamily A%2C polypeptide 6 | Chr2:1875390-1876794 FORWARD LENGTH=442 | 201606 Arabidopsis_thaliana OG0000005
AT2G12190.1 | Cytochrome P450 superfamily protein | Chr2:4891807-4893345 REVERSE LENGTH=512 | 201606 Arabidopsis_thaliana OG0000005
AT2G14100.1 | cytochrome P450%2C family 705%2C subfamily A%2C polypeptide 13 | Chr2:5934733-5936371 REVERSE LENGTH=518 | 201606 Arabidopsis_thaliana OG0000005
AT2G23190.1 | cytochrome P450%2C family 81%2C subfamily D%2C polypeptide 7 | Chr2:9877058-9879007 FORWARD LENGTH=543 | 201606 Arabidopsis_thaliana OG0000005
AT2G23220.1 | cytochrome P450%2C family 81%2C subfamily D%2C polypeptide 6 | Chr2:9884550-9886752 FORWARD LENGTH=515 | 201606 Arabidopsis_thaliana OG0000005
AT2G24180.1 | cytochrome p450 71b6 | Chr2:10281890-10283589 FORWARD LENGTH=503 | 201606 Arabidopsis_thaliana OG0000005
AT2G25160.1 | cytochrome P450%2C family 82%2C subfamily F%2C polypeptide 1 | Chr2:10709236-10711211 REVERSE LENGTH=527 | 201606 Arabidopsis_thaliana OG0000005
AT2G26170.1 | cytochrome P450%2C family 711%2C subfamily A%2C polypeptide 1 | Chr2:11140851-11143270 FORWARD LENGTH=522 | 201606 Arabidopsis_thaliana OG0000005
AT2G26710.1 | Cytochrome P450 superfamily protein | Chr2:11380700-11383413 FORWARD LENGTH=520 | 201606 Arabidopsis_thaliana OG0000005
AT2G27000.1 | cytochrome P450%2C family 705%2C subfamily A%2C polypeptide 8 | Chr2:11523475-11525095 REVERSE LENGTH=514 | 201606 Arabidopsis_thaliana OG0000005
AT2G27010.1 | cytochrome P450%2C family 705%2C subfamily A%2C polypeptide 9 | Chr2:11526236-11527854 REVERSE LENGTH=498 | 201606 Arabidopsis_thaliana OG0000005
AT2G30750.1 | cytochrome P450 family 71 polypeptide | Chr2:13099486-13101371 REVERSE LENGTH=497 | 201606 Arabidopsis_thaliana OG0000005
AT2G30770.1 | cytochrome P450 family 71 polypeptide |

Chr2:13109909-13111988 REVERSE LENGTH=497 | 201606
Arabidopsis_thaliana OG0000005
Os03t0248200-01 Similar to Cytochrome P450 family protein, expressed. Oryza_sativa OG0000005
Os03t0248300-01 Similar to Cytochrome P450 76C4 (EC 1.14.-.-). Oryza_sativa OG0000005
Os03t0332100-00 Similar to Cytochrome P450 CYP714B3. Oryza_sativa OG0000005
Os03t0370900-01 Similar to Cytochrome P450. Oryza_sativa OG0000005
Os03t0371350-00 Similar to Cytochrome P450 family protein, expressed. Oryza_sativa OG0000005
Os03t0371400-01 Cytochrome P450 family protein. Oryza_sativa OG0000005
Os03t0417700-01 Similar to Phytochrome P450. Oryza_sativa OG0000005
Os03t0568400-00 Similar to Cytochrome P450 family protein. Oryza_sativa OG0000005
Os03t0593600-00 Similar to Cytochrome P450 family protein, expressed. Oryza_sativa OG0000005
Os03t0594100-01 Cytochrome P450 family protein. Oryza_sativa OG0000005
Os03t0594900-01 Cytochrome P450 family protein. Oryza_sativa OG0000005
Os03t0603100-00 Similar to Phytochrome P450. Oryza_sativa OG0000005
Os03t0741500-01 Similar to Cytochrome P450 family protein, expressed. Oryza_sativa OG0000005
Os03t0760000-01 Cytochrome P450 family protein. Oryza_sativa OG0000005
Os03t0760200-01 Cytochrome P450 family protein. Oryza_sativa OG0000005
Os03t0760500-01 Cytochrome P450 family protein. Oryza_sativa OG0000005
Os03t0850200-00 Similar to Cytochrome P450. Oryza_sativa OG0000005
Os04t0101400-01 Flavone synthase II (FNSII), Biosynthesis of tricin O-linked conjugates Oryza_sativa OG0000005
Os04t0128001-00 Similar to OSIGBa0126J24.7 protein. Oryza_sativa OG0000005
Os04t0128400-00 Similar to OSIGBa0126J24.7 protein. Oryza_sativa OG0000005
Os04t0174100-00 Similar to OSIGBa0114I04.1 protein. Oryza_sativa OG0000005
Os04t0178400-01 Similar to Cytochrome P450 CYP99A1 (EC 1.14.-.-) (Fragment). Oryza_sativa OG0000005
Os04t0180400-01 Similar to Cytochrome P450 99A2. Oryza_sativa OG0000005
Os04t0255600-00 Cytochrome P450 domain containing protein. Oryza_sativa OG0000005
Os04t0407900-01 Similar to Cytochrom P450-like protein. Oryza_sativa OG0000005
Os04t0480600-01 Similar to Cytochrome P450 71A1 (EC 1.14.-.-) (CYPLXXIA1) (ARP-2). Oryza_sativa OG0000005

Os04t0480700-01 Cytochrome P450 family protein.	Oryza_sativa
OG0000005
Os01t0349800-01 Similar to Cytochrome P450.	Oryza_sativa
OG0000005
Os01t0377000-01 Cytochrome P450 family protein.	Oryza_sativa
OG0000005
Os05t0361000-00 Similar to cDNA, clone	Oryza_sativa
OG0000005
Os05t0372300-01 Cytochrome P450 family protein.	Oryza_sativa
OG0000005
Os01t0388000-01 Similar to Cytochrome P450 monooxygenase CYP72A5 (Fragment).	Oryza_sativa	OG0000005
Os05t0405500-01 Similar to Cytochrome P450 CYP721B4.
Oryza_sativa	OG0000005
Os05t0405600-01 Similar to Cytochrome P450 CYP721B4.
Oryza_sativa	OG0000005
Os05t0424300-01 Cytochrome P450 family protein.	Oryza_sativa
OG0000005
Os05t0482400-01 Cytochrome P450 monooxygenase, Gibberellin (GA)-deactivating enzyme, Modulation of internode elongation
Oryza_sativa	OG0000005
Os05t0494000-01 Similar to Cytochrome P450 98A1 (EC 1.14.-.-).
Oryza_sativa	OG0000005
Os05t0515200-01 Cytochrome P450 family protein.	Oryza_sativa
OG0000005
Os06t0102100-01 Cytochrome P450 93G2 protein (CYP93G2), flavanone 2-hydroxylase (F2H), C-glycosylflavone biosynthesis	Oryza_sativa
OG0000005
Os06t0191700-00 Similar to Cytochrome P450.	Oryza_sativa
OG0000005
Os06t0191800-01 Cytochrome P450 family protein.	Oryza_sativa
OG0000005
Os01t0544200-01 Similar to P450.	Oryza_sativa	OG0000005
Os06t0267400-00 Similar to Cytochrome P450.	Oryza_sativa
OG0000005
Os06t0294600-02 Cytochrome P450 family protein.	Oryza_sativa
OG0000005
Os06t0325900-00 Similar to Bx2-like protein.	Oryza_sativa
OG0000005
Os06t0328900-01 Cytochrome P450 family protein.	Oryza_sativa
OG0000005
Os06t0349700-00 Cytochrome P450 family protein.	Oryza_sativa
OG0000005
Os06t0497200-00 Hypothetical conserved gene.	Oryza_sativa
OG0000005
Os06t0497350-00 Similar to Cytochrome P450 CYP71K14.
Oryza_sativa	OG0000005
Os06t0500700-01 Cytochrome P450 family protein.	Oryza_sativa
OG0000005
Os06t0501900-01 Cytochrome P450 family protein.	Oryza_sativa
OG0000005
Os01t0561600-01 Cytochrome P450 family protein.	Oryza_sativa
OG0000005
Os06t0565100-00 Cytochrome P450 family protein.	Oryza_sativa

OG0000005
Os06t0599200-01 Cytochrome P450 family protein.	Oryza_sativa
OG0000005
Os06t0600400-01 Cytochrome P450 family protein.	Oryza_sativa
OG0000005
Os06t0613600-01 Cytochrome P450 family protein.	Oryza_sativa
OG0000005
Os06t0632300-00 Similar to Transposon protein.	Oryza_sativa
OG0000005
Os06t0639800-01 Cytochrome P450 family protein.	Oryza_sativa
OG0000005
Os06t0640100-00 Similar to Cytochrome P450 CYP71Y10.
Oryza_sativa	OG0000005
Os06t0640800-00 Similar to Cytochrome P450 CYP71Y10.
Oryza_sativa	OG0000005
Os06t0641200-00 Similar to Cytochrome P450 CYP71Y10.
Oryza_sativa	OG0000005
Os06t0641500-00 Similar to Cytochrome P450 CYP71Y10.
Oryza_sativa	OG0000005
Os06t0641600-00 Similar to Cytochrome P450 family protein.
Oryza_sativa	OG0000005
Os06t0641800-00 Cytochrome P450 family protein.	Oryza_sativa
OG0000005
Os06t0642200-00 Similar to Cytochrome P450 family protein.
Oryza_sativa	OG0000005
Os06t0642600-00 Similar to Cytochrome P450 CYP71Y10.
Oryza_sativa	OG0000005
Os06t0671300-01 Cytochrome P450 family protein.	Oryza_sativa
OG0000005
Os06t0680700-01 Cytochrome P450 family protein.	Oryza_sativa
OG0000005
Os07t0217600-01 Cytochrome P450 family protein.	Oryza_sativa
OG0000005
Os07t0218700-01 Cytochrome P450 family protein.	Oryza_sativa
OG0000005
Os01t0602200-01 Cytochrome P450 monooxygenase, Tolerance to
acetolactate synthase-inhibiting herbicides (pseudogene)
Oryza_sativa	OG0000005
Os01t0602400-01 Similar to Cytochrome P450 monooxygenase CYP72A5
(Fragment).	Oryza_sativa	OG0000005
Os01t0602500-01 Cytochrome P450 family protein.	Oryza_sativa
OG0000005
Os07t0291750-00 Similar to CYP71B26; electron carrier/ heme
binding / iron ion binding / monooxygenase/ oxygen binding.
Oryza_sativa	OG0000005
Os07t0293000-00 Hypothetical conserved gene.	Oryza_sativa
OG0000005
Os07t0418500-01 Similar to Cytochrome P450.	Oryza_sativa
OG0000005
Os07t0419000-00 Similar to Cytochrome P450.	Oryza_sativa
OG0000005
Os07t0419100-00 Cytochrome P450 family protein.	Oryza_sativa
OG0000005
Os07t0451300-01 Cytochrome P450 family protein.	Oryza_sativa

OG0000005
Os07t0603700-01 CYP78A13 (Cytochrome P450), Size control between embryo and endosperm	Oryza_sativa	OG0000005
Os07t0635200-00 Cytochrome P450, E-class, group I domain containing protein.	Oryza_sativa	OG0000005
Os07t0635300-01 Similar to Cytochrome P450.	Oryza_sativa	OG0000005
Os07t0635500-01 Similar to Cytochrome P450.	Oryza_sativa	OG0000005
Os07t0635700-00 Cytochrome P450 family protein.	Oryza_sativa	OG0000005
Os07t0647200-00 Similar to Cytochrome P450 CYP734A7.	Oryza_sativa	OG0000005
Os01t0627400-01 Similar to Cytochrome P450 monooxygenase CYP72A5 (Fragment).	Oryza_sativa	OG0000005
Os01t0627500-01 Cytochrome P450 family protein.	Oryza_sativa	OG0000005
Os01t0627600-01 Similar to Cytochrome P450 monooxygenase.	Oryza_sativa	OG0000005
Os07t0681300-00 Cytochrome P450 family protein.	Oryza_sativa	OG0000005
Os01t0627800-01 Similar to Cytochrome P450 monooxygenase CYP72A5 (Fragment).	Oryza_sativa	OG0000005
Os01t0627900-01 Similar to Cytochrome P450.	Oryza_sativa	OG0000005
Os01t0627933-00 Similar to Cytochrome P450.	Oryza_sativa	OG0000005
Os01t0628700-01 Cytochrome P450.	Oryza_sativa	OG0000005
Os01t0628900-01 Cytochrome P450 family protein.	Oryza_sativa	OG0000005
Os08t0105400-00 Similar to P450.	Oryza_sativa	OG0000005
Os08t0105600-00 Cytochrome P450 domain containing protein.	Oryza_sativa	OG0000005
Os08t0105700-01 Similar to Bx2-like protein.	Oryza_sativa	OG0000005
Os08t0105800-01 Cytochrome P450 family protein.	Oryza_sativa	OG0000005
Os08t0106000-00 Similar to Bx2-like protein.	Oryza_sativa	OG0000005
Os08t0106300-00 Similar to Cytochrome P450.	Oryza_sativa	OG0000005
Os08t0131100-01 Similar to Cytochrome P450.	Oryza_sativa	OG0000005
Os08t0152300-00 Similar to P450.	Oryza_sativa	OG0000005
Os08t0152400-00 Similar to P450.	Oryza_sativa	OG0000005
Os08t0226166-00 Similar to Cytochrome P450 CYP76H18.	Oryza_sativa	OG0000005
Os08t0226300-00 Similar to Cytochrome P450 family protein.	Oryza_sativa	OG0000005
Os08t0456200-00 Similar to flavonoid 3-monooxygenase.	Oryza_sativa	OG0000005
Os08t0465700-01 Cytochrome P450 family protein.	Oryza_sativa	OG0000005
Os08t0507000-00 Similar to Cyt-P450 monooxygenase.	Oryza_sativa

OG0000005
Os08t0507100-00 Cytochrome P450 family protein.	Oryza_sativa
OG0000005
Os08t0507702-01 Cytochrome P450 family protein.	Oryza_sativa
OG0000005
Os08t0508000-01 Cytochrome P450 family protein.	Oryza_sativa
OG0000005
Os08t0547300-01 Similar to Cytochrome P450 78A1 (EC 1.14.-.-)
(CYPLXXVIII).	Oryza_sativa	OG0000005
Os08t0547900-01 Similar to Flavonoid 3-monooxygenase.
Oryza_sativa	OG0000005
Os09t0263933-00 Similar to flavonoid 3-monooxygenase.
Oryza_sativa	OG0000005
Os09t0264051-00 Similar to flavonoid 3-monooxygenase.
Oryza_sativa	OG0000005
Os09t0264400-01 Cytochrome P450 family protein.	Oryza_sativa
OG0000005
Os09t0275400-01 Cytochrome P450 family protein.	Oryza_sativa
OG0000005
Os09t0441100-01 Similar to Cytochrome P450 monooxygenase CYP92A1.
Oryza_sativa	OG0000005
Os09t0441400-01 Similar to Elicitor-inducible cytochrome P450.
Oryza_sativa	OG0000005
Os09t0441600-00 Similar to flavonoid 3-monooxygenase.
Oryza_sativa	OG0000005
Os09t0441625-00 Similar to flavonoid 3-monooxygenase.
Oryza_sativa	OG0000005
Os09t0441700-00 Similar to flavonoid 3-monooxygenase.
Oryza_sativa	OG0000005
Os09t0447300-01 Cytochrome P450 family protein.	Oryza_sativa
OG0000005
Os09t0447500-01 Cytochrome P450 family protein.	Oryza_sativa
OG0000005
Os09t0528700-00 Similar to Cytochrome p450 (CYP78A9).
Oryza_sativa	OG0000005
Os09t0530275-00 Cytochrome P450 domain containing protein.
Oryza_sativa	OG0000005
Os09t0530300-01 Cytochrome P450 family protein.	Oryza_sativa
OG0000005
Os01t0700500-01 Similar to Flavonoid 3-monooxygenase.
Oryza_sativa	OG0000005
Os01t0700900-01 Similar to Cytochrome P450 monooxygenase.
Oryza_sativa	OG0000005
Os01t0701300-00 Similar to Cytochrome P450 monooxygenase.
Oryza_sativa	OG0000005
Os01t0701400-00 Similar to Cytochrome P450 monooxygenase.
Oryza_sativa	OG0000005
Os10t0139700-01 Cytochrome P450 family protein.	Oryza_sativa
OG0000005
Os01t0701500-00 Cytochrome P450 family protein.	Oryza_sativa
OG0000005
Os10t0144700-00 Cytochrome P450 domain containing protein.
Oryza_sativa	OG0000005
Os10t0164500-00 Similar to Cytochrome P450 family protein,

expressed.	Oryza_sativa	OG0000005
Os10t0165300-01 Cytochrome P450 family protein.	Oryza_sativa
OG0000005
Os10t0166600-01 Cytochrome P450 family protein.	Oryza_sativa
OG0000005
Os10t0167200-00 Similar to Cytochrome P450 family protein.
Oryza_sativa	OG0000005
Os10t0167250-00 Similar to Cytochrome P450 CYP76H18.
Oryza_sativa	OG0000005
Os10t0171300-01 Cytochrome P450 family protein.	Oryza_sativa
OG0000005
Os10t0171500-01 Similar to Cytochrome P450 CYP76H18.
Oryza_sativa	OG0000005
Os10t0172100-00 Similar to Cytochrome P450 family protein,
expressed.	Oryza_sativa	OG0000005
Os10t0196100-00 Similar to Cytochrome P450 98A1.	Oryza_sativa
OG0000005
Os10t0317900-01 Chrysoeriol 5'-Hydroxylase, Flavonoid B-ring
hydroxylase, Tricin biosynthesis	Oryza_sativa	OG0000005
Os10t0320100-01 Similar to Flavonoid 3'-monooxygenase (EC
1.14.13.21) (Flavonoid 3'-hydroxylase) (Cytochrome P450 75B2).
Oryza_sativa	OG0000005
Os10t0351200-00 Similar to Cytochrome P450 family protein,
expressed.	Oryza_sativa	OG0000005
Os10t0403000-01 Cytochrome P450 protein, CYP78A11, Regulation of
leaf initiation rate and vegetative-reproductive phase change
Oryza_sativa	OG0000005
Os10t0439800-00 Similar to H0813E03.1 protein.	Oryza_sativa
OG0000005
Os10t0439924-01 Cytochrome P450 family protein.	Oryza_sativa
OG0000005
Os10t0512400-01 Cytochrome P450 family protein.	Oryza_sativa
OG0000005
Os10t0513400-00 Cytochrome P450 family protein.	Oryza_sativa
OG0000005
Os10t0513900-01 Cytochrome P450 family protein.	Oryza_sativa
OG0000005
Os10t0514300-00 Similar to Transposon protein.	Oryza_sativa
OG0000005
Os10t0514450-00 Similar to Cytochrome P450 family protein.
Oryza_sativa	OG0000005
Os10t0514500-00 Similar to transposon protein.	Oryza_sativa
OG0000005
Os10t0514700-00 Similar to Cytochrome P450 family protein.
Oryza_sativa	OG0000005
Os10t0514901-00 Similar to Cytochrome P450 family protein.
Oryza_sativa	OG0000005
Os10t0515200-01 Cytochrome P450 family protein.	Oryza_sativa
OG0000005
Os10t0515400-00 Similar to Transposon protein.	Oryza_sativa
OG0000005
Os10t0515900-01 Cytochrome P450 family protein.	Oryza_sativa
OG0000005
Os01t0728300-01 Similar to Cytochrome P450 monooxygenase CYP72A5

(Fragment).   Oryza_sativa   OG0000005
Os01t0728350-00 Similar to Cytochrome P450 monooxygenase CYP72A5.
Oryza_sativa   OG0000005
Os11t0119700-00 Hypothetical conserved gene.   Oryza_sativa
OG0000005
Os11t0465200-01 Similar to Bx2-like protein.   Oryza_sativa
OG0000005
Os11t0489250-00 Similar to Cytochrome P450 family protein,
expressed.   Oryza_sativa   OG0000005
Os11t0635300-00 Similar to Cytochrome P450 family protein.
Oryza_sativa   OG0000005
Os11t0635500-01 Cytochrome P450 family protein.   Oryza_sativa
OG0000005
Os12t0118900-01 Cytochrome P450 family protein.   Oryza_sativa
OG0000005
Os12t0119000-01 Cytochrome P450 family protein.   Oryza_sativa
OG0000005
Os12t0199800-01 Similar to Cyt-P450 monooxygenase. Oryza_sativa
OG0000005
Os12t0268000-01 Similar to Cytochrome P450 71A1 (EC 1.14.-.-)
(CYPLXXIA1) (ARP-2).   Oryza_sativa   OG0000005
Os12t0512800-01 Cytochrome P450 71E1 (EC 1.14.13.68) (4-
hydroxyphenylacetaldehyde oxime monooxygenase).   Oryza_sativa
OG0000005
Os12t0582000-00 Similar to Cytochrome P450 family protein.
Oryza_sativa   OG0000005
Os12t0582666-00 Cytochrome P450 family protein.   Oryza_sativa
OG0000005
Os12t0582700-01 Similar to Cytochrome P450.   Oryza_sativa
OG0000005
Os12t0640200-00 Similar to Cytochrome P450 family protein,
expressed.   Oryza_sativa   OG0000005
Os01t0804900-01 Similar to Cytochrome P450.   Oryza_sativa
OG0000005
Os01t0957600-01 Similar to cDNA, clone   Oryza_sativa
OG0000005
Os01t0957800-01 Cytochrome P450 domain containing protein.
Oryza_sativa   OG0000005
Os02t0108800-01 Cytochrome P450 domain containing protein.
Oryza_sativa   OG0000005
Os02t0173100-01 Similar to cytochrome P450.   Oryza_sativa
OG0000005
Os02t0184600-00 Similar to Cytochrome P450 CYP71K14.
Oryza_sativa   OG0000005
Os02t0184700-00 Cytochrome P450 family protein.   Oryza_sativa
OG0000005
Os02t0184900-01 Cytochrome P450 family protein.   Oryza_sativa
OG0000005
Os02t0185200-01 Cytochrome P450 family protein.   Oryza_sativa
OG0000005
Os02t0185300-01 Cytochrome P450 family protein.   Oryza_sativa
OG0000005
Os02t0185500-00 Similar to cDNA, clone   Oryza_sativa
OG0000005

Os02t0185900-01 Hypothetical conserved gene.	Oryza_sativa	OG0000005
Os02t0186200-00 Similar to Cytochrome P450 CYP71K14.	Oryza_sativa	OG0000005
Os02t0186800-00 Cytochrome P450 family protein.	Oryza_sativa	OG0000005
Os02t0186900-01 Cytochrome P450 family protein.	Oryza_sativa	OG0000005
Os02t0187000-00 Similar to Cytochrome P450 CYP71K14.	Oryza_sativa	OG0000005
Os02t0204700-00 Cytochrome P450 family protein.	Oryza_sativa	OG0000005
Os02t0217400-01 Cytochrome P450 family protein.	Oryza_sativa	OG0000005
Os02t0221900-01 Cytochrome P450 family protein.	Oryza_sativa	OG0000005
Os02t0278400-01 Cytochrome P450 family protein.	Oryza_sativa	OG0000005
Os02t0467000-00 Similar to trans-cinnamate 4-monooxygenase.	Oryza_sativa	OG0000005
Os02t0467600-01 Similar to Cinnamate 4-hydroxylase CYP73.	Oryza_sativa	OG0000005
Os02t0500700-00 Similar to Cytochrome P450 family protein.	Oryza_sativa	OG0000005
Os02t0503100-00 Similar to flavonoid 3-monooxygenase.	Oryza_sativa	OG0000005
Os02t0503700-00 Similar to Cytochrome P450 CYP81L6.	Oryza_sativa	OG0000005
Os02t0503850-00 Similar to Cytochrome P450 CYP81L6.	Oryza_sativa	OG0000005
Os02t0503900-01 Cytochrome P450 family protein.	Oryza_sativa	OG0000005
Os02t0529800-00 Cytochrome P450 family protein.	Oryza_sativa	OG0000005
Os02t0569000-01 Cytochrome P450 family protein.	Oryza_sativa	OG0000005
Os02t0569400-01 Cytochrome P450 family protein.	Oryza_sativa	OG0000005
Os02t0569900-01 Cytochrome P450 family protein.	Oryza_sativa	OG0000005
Os02t0570500-01 Similar to Cytochrome P450 family protein, expressed.	Oryza_sativa	OG0000005
Os02t0570700-01 Cytochrome P450 family protein.	Oryza_sativa	OG0000005
Os02t0571900-01 Cytochrome P450 family protein.	Oryza_sativa	OG0000005
Os02t0824100-00 Cytochrome P450 family protein.	Oryza_sativa	OG0000005
Os03t0112900-00 Similar to Aldehyde 5-hydroxylase.	Oryza_sativa	OG0000005
Os03t0134500-00 Cytochrome p450 (CYP78A9).	Oryza_sativa	OG0000005
Os01t0227400-01 Similar to Cytochrome P450 71A1 (EC 1.14.-.-) (CYPLXXIA1) (ARP-2).	Oryza_sativa	OG0000005

Os01t0227500-01 Cytochrome P450 family protein.   Oryza_sativa
OG0000005
Os01t0227700-01 Cytochrome P450 family protein.   Oryza_sativa
OG0000005
Os01t0227800-01 Similar to cDNA, clone   Oryza_sativa
OG0000005
CMD096CT pep chromosome:ASM9120v1:4:232372:234060:1 gene:CMD096C
transcript:CMD096CT gene_biotype:protein_coding
transcript_biotype:protein_coding Cyanidioschyzon_merolae
OG0000005
evm.model.contig_2494.9 Porphyridium_purpureum   OG0000005
evm.model.contig_3385.7 Porphyridium_purpureum   OG0000005
evm.model.contig_2032.24 Porphyridium_purpureum   OG0000005
evm.model.contig_458.12 Porphyridium_purpureum   OG0000005
evm.model.contig_2058.6 Porphyridium_purpureum   OG0000005
CDF32288 pep supercontig:ASM35022v2:HG001469:14231:15838:-1
gene:CHC_T00009041001 transcript:CDF32288
gene_biotype:protein_coding transcript_biotype:protein_coding
description:cytochrome P450 family 809A-CYP809A1   Chondrus_crispus
OG0000005
CDF39295 pep supercontig:ASM35022v2:HG002021:45714:47315:1
gene:CHC_T00008350001 transcript:CDF39295
gene_biotype:protein_coding transcript_biotype:protein_coding
description:cytochrome P450 family 807A-CYP807A1   Chondrus_crispus
OG0000005
CDF37857 pep supercontig:ASM35022v2:HG001881:98999:101119:-1
gene:CHC_T00009499001 transcript:CDF37857
gene_biotype:protein_coding transcript_biotype:protein_coding
description:cytochrome P450 family 808A-CYP808A1   Chondrus_crispus
OG0000005
CDF35351 pep supercontig:ASM35022v2:HG001729:170839:173372:-1
gene:CHC_T00008799001 transcript:CDF35351
gene_biotype:protein_coding transcript_biotype:protein_coding
description:cytochrome P450 family 805C-CYP805C1   Chondrus_crispus
OG0000005
CDF35349 pep supercontig:ASM35022v2:HG001729:165249:166778:1
gene:CHC_T00008302001 transcript:CDF35349
gene_biotype:protein_coding transcript_biotype:protein_coding
description:cytochrome P450 family 805A-CYP805A1   Chondrus_crispus
OG0000005
CDF40484 pep supercontig:ASM35022v2:HG002186:10341:12448:-1
gene:CHC_T00008663001 transcript:CDF40484
gene_biotype:protein_coding transcript_biotype:protein_coding
description:cytochrome P450 family 808A-CYP808A2   Chondrus_crispus
OG0000005
CDF34928 pep supercontig:ASM35022v2:HG001711:27700:29459:1
gene:CHC_T00008616001 transcript:CDF34928
gene_biotype:protein_coding transcript_biotype:protein_coding
description:cytochrome P450 family 808G-CYP808G1   Chondrus_crispus
OG0000005
CDF34019 pep supercontig:ASM35022v2:HG001668:124174:125998:-1
gene:CHC_T00008813001 transcript:CDF34019
gene_biotype:protein_coding transcript_biotype:protein_coding
description:cytochrome P450 family 808H-CYP808H1   Chondrus_crispus

OG0000005
CDF34023 pep supercontig:ASM35022v2:HG001668:203097:204617:-1
gene:CHC_T00009062001 transcript:CDF34023
gene_biotype:protein_coding transcript_biotype:protein_coding
description:cytochrome P450 family 808F-CYP808F1	Chondrus_crispus
OG0000005
CDF34021 pep supercontig:ASM35022v2:HG001668:199198:200752:-1
gene:CHC_T00010004001 transcript:CDF34021
gene_biotype:protein_coding transcript_biotype:protein_coding
description:cytochrome P450 family 808C-CYP808C1	Chondrus_crispus
OG0000005
CDF34839 pep supercontig:ASM35022v2:HG001707:177752:179293:-1
gene:CHC_T00010245001 transcript:CDF34839
gene_biotype:protein_coding transcript_biotype:protein_coding
description:cytochrome P450 family 808B-CYP808B1	Chondrus_crispus
OG0000005
CDF37211 pep supercontig:ASM35022v2:HG001819:8535:10040:1
gene:CHC_T00009144001 transcript:CDF37211
gene_biotype:protein_coding transcript_biotype:protein_coding
description:cytochrome P450 family 805B-CYP805B1	Chondrus_crispus
OG0000005
CDF32186 pep supercontig:ASM35022v2:HG001459:852886:854451:-1
gene:CHC_T00009131001 transcript:CDF32186
gene_biotype:protein_coding transcript_biotype:protein_coding
description:cytochrome P450 family 808D-CYP808D1	Chondrus_crispus
OG0000005
CDF32414 pep supercontig:ASM35022v2:HG001492:108:2634:-1
gene:CHC_T00008672001 transcript:CDF32414
gene_biotype:protein_coding transcript_biotype:protein_coding
description:cytochrome P450 family 808E-CYP808E1	Chondrus_crispus
OG0000005
g1011.t1 Caulerpa_lentillifera	OG0000006
g1091.t1 Caulerpa_lentillifera	OG0000006
g1112.t1 Caulerpa_lentillifera	OG0000006
g1179.t1 Caulerpa_lentillifera	OG0000006
g1233.t1 Caulerpa_lentillifera	OG0000006
g1325.t1 Caulerpa_lentillifera	OG0000006
g1395.t1 Caulerpa_lentillifera	OG0000006
g1551.t1 Caulerpa_lentillifera	OG0000006
g1850.t1 Caulerpa_lentillifera	OG0000006
g1851.t1 Caulerpa_lentillifera	OG0000006
g1891.t1 Caulerpa_lentillifera	OG0000006
g2266.t1 Caulerpa_lentillifera	OG0000006
g2267.t1 Caulerpa_lentillifera	OG0000006
g2326.t1 Caulerpa_lentillifera	OG0000006
g2672.t1 Caulerpa_lentillifera	OG0000006
g2673.t1 Caulerpa_lentillifera	OG0000006
g2758.t1 Caulerpa_lentillifera	OG0000006
g2785.t1 Caulerpa_lentillifera	OG0000006
g2975.t1 Caulerpa_lentillifera	OG0000006
g3040.t1 Caulerpa_lentillifera	OG0000006
g3041.t1 Caulerpa_lentillifera	OG0000006
g3295.t1 Caulerpa_lentillifera	OG0000006
g3377.t1 Caulerpa_lentillifera	OG0000006

g3436.t1	Caulerpa_lentillifera	OG0000006
g353.t1	Caulerpa_lentillifera	OG0000006
g354.t1	Caulerpa_lentillifera	OG0000006
g356.t1	Caulerpa_lentillifera	OG0000006
g3721.t1	Caulerpa_lentillifera	OG0000006
g3722.t1	Caulerpa_lentillifera	OG0000006
g3767.t1	Caulerpa_lentillifera	OG0000006
g3770.t1	Caulerpa_lentillifera	OG0000006
g378.t1	Caulerpa_lentillifera	OG0000006
g3994.t1	Caulerpa_lentillifera	OG0000006
g4037.t1	Caulerpa_lentillifera	OG0000006
g4038.t1	Caulerpa_lentillifera	OG0000006
g4096.t1	Caulerpa_lentillifera	OG0000006
g4292.t1	Caulerpa_lentillifera	OG0000006
g4325.t1	Caulerpa_lentillifera	OG0000006
g4372.t1	Caulerpa_lentillifera	OG0000006
g4374.t1	Caulerpa_lentillifera	OG0000006
g4375.t1	Caulerpa_lentillifera	OG0000006
g439.t1	Caulerpa_lentillifera	OG0000006
g440.t1	Caulerpa_lentillifera	OG0000006
g4705.t1	Caulerpa_lentillifera	OG0000006
g4788.t1	Caulerpa_lentillifera	OG0000006
g4789.t1	Caulerpa_lentillifera	OG0000006
g4815.t1	Caulerpa_lentillifera	OG0000006
g5021.t1	Caulerpa_lentillifera	OG0000006
g5022.t1	Caulerpa_lentillifera	OG0000006
g5114.t1	Caulerpa_lentillifera	OG0000006
g5117.t1	Caulerpa_lentillifera	OG0000006
g5118.t1	Caulerpa_lentillifera	OG0000006
g5121.t1	Caulerpa_lentillifera	OG0000006
g5122.t1	Caulerpa_lentillifera	OG0000006
g5161.t1	Caulerpa_lentillifera	OG0000006
g5163.t1	Caulerpa_lentillifera	OG0000006
g5164.t1	Caulerpa_lentillifera	OG0000006
g5184.t1	Caulerpa_lentillifera	OG0000006
g5190.t1	Caulerpa_lentillifera	OG0000006
g5193.t1	Caulerpa_lentillifera	OG0000006
g5232.t1	Caulerpa_lentillifera	OG0000006
g5243.t1	Caulerpa_lentillifera	OG0000006
g5314.t1	Caulerpa_lentillifera	OG0000006
g5337.t1	Caulerpa_lentillifera	OG0000006
g5338.t1	Caulerpa_lentillifera	OG0000006
g5360.t1	Caulerpa_lentillifera	OG0000006
g5363.t1	Caulerpa_lentillifera	OG0000006
g5364.t1	Caulerpa_lentillifera	OG0000006
g5365.t1	Caulerpa_lentillifera	OG0000006
g5366.t1	Caulerpa_lentillifera	OG0000006
g5367.t1	Caulerpa_lentillifera	OG0000006
g5368.t1	Caulerpa_lentillifera	OG0000006
g5372.t1	Caulerpa_lentillifera	OG0000006
g5382.t1	Caulerpa_lentillifera	OG0000006
g5383.t1	Caulerpa_lentillifera	OG0000006
g5391.t1	Caulerpa_lentillifera	OG0000006
g5403.t1	Caulerpa_lentillifera	OG0000006

g5415.t1	Caulerpa_lentillifera	OG0000006
g5433.t1	Caulerpa_lentillifera	OG0000006
g5434.t1	Caulerpa_lentillifera	OG0000006
g5436.t1	Caulerpa_lentillifera	OG0000006
g5478.t1	Caulerpa_lentillifera	OG0000006
g5656.t1	Caulerpa_lentillifera	OG0000006
g5986.t1	Caulerpa_lentillifera	OG0000006
g5987.t1	Caulerpa_lentillifera	OG0000006
g6104.t1	Caulerpa_lentillifera	OG0000006
g6228.t1	Caulerpa_lentillifera	OG0000006
g6271.t1	Caulerpa_lentillifera	OG0000006
g6358.t1	Caulerpa_lentillifera	OG0000006
g6623.t1	Caulerpa_lentillifera	OG0000006
g6624.t1	Caulerpa_lentillifera	OG0000006
g6625.t1	Caulerpa_lentillifera	OG0000006
g6626.t1	Caulerpa_lentillifera	OG0000006
g6627.t1	Caulerpa_lentillifera	OG0000006
g6638.t1	Caulerpa_lentillifera	OG0000006
g6640.t1	Caulerpa_lentillifera	OG0000006
g6641.t1	Caulerpa_lentillifera	OG0000006
g6643.t1	Caulerpa_lentillifera	OG0000006
g6644.t1	Caulerpa_lentillifera	OG0000006
g6645.t1	Caulerpa_lentillifera	OG0000006
g6669.t1	Caulerpa_lentillifera	OG0000006
g6670.t1	Caulerpa_lentillifera	OG0000006
g6671.t1	Caulerpa_lentillifera	OG0000006
g6674.t1	Caulerpa_lentillifera	OG0000006
g6675.t1	Caulerpa_lentillifera	OG0000006
g6821.t1	Caulerpa_lentillifera	OG0000006
g6823.t1	Caulerpa_lentillifera	OG0000006
g7059.t1	Caulerpa_lentillifera	OG0000006
g7060.t1	Caulerpa_lentillifera	OG0000006
g72.t1	Caulerpa_lentillifera	OG0000006
g7106.t1	Caulerpa_lentillifera	OG0000006
g7107.t1	Caulerpa_lentillifera	OG0000006
g73.t1	Caulerpa_lentillifera	OG0000006
g7278.t1	Caulerpa_lentillifera	OG0000006
g7349.t1	Caulerpa_lentillifera	OG0000006
g7528.t1	Caulerpa_lentillifera	OG0000006
g7558.t1	Caulerpa_lentillifera	OG0000006
g7559.t1	Caulerpa_lentillifera	OG0000006
g7620.t1	Caulerpa_lentillifera	OG0000006
g7748.t1	Caulerpa_lentillifera	OG0000006
g7750.t1	Caulerpa_lentillifera	OG0000006
g7751.t1	Caulerpa_lentillifera	OG0000006
g8080.t1	Caulerpa_lentillifera	OG0000006
g8082.t1	Caulerpa_lentillifera	OG0000006
g8083.t1	Caulerpa_lentillifera	OG0000006
g8096.t1	Caulerpa_lentillifera	OG0000006
g8142.t1	Caulerpa_lentillifera	OG0000006
g8144.t1	Caulerpa_lentillifera	OG0000006
g8145.t1	Caulerpa_lentillifera	OG0000006
g8265.t1	Caulerpa_lentillifera	OG0000006
g8527.t1	Caulerpa_lentillifera	OG0000006

g8588.t1 Caulerpa_lentillifera OG0000006
g8621.t1 Caulerpa_lentillifera OG0000006
g8699.t1 Caulerpa_lentillifera OG0000006
g8700.t1 Caulerpa_lentillifera OG0000006
g8777.t1 Caulerpa_lentillifera OG0000006
g8798.t1 Caulerpa_lentillifera OG0000006
g8805.t1 Caulerpa_lentillifera OG0000006
g8807.t1 Caulerpa_lentillifera OG0000006
g8809.t1 Caulerpa_lentillifera OG0000006
g8839.t1 Caulerpa_lentillifera OG0000006
g8848.t1 Caulerpa_lentillifera OG0000006
g8938.t1 Caulerpa_lentillifera OG0000006
g9020.t1 Caulerpa_lentillifera OG0000006
g9037.t1 Caulerpa_lentillifera OG0000006
g9040.t1 Caulerpa_lentillifera OG0000006
g9073.t1 Caulerpa_lentillifera OG0000006
g9113.t1 Caulerpa_lentillifera OG0000006
g919.t1 Caulerpa_lentillifera OG0000006
g9209.t1 Caulerpa_lentillifera OG0000006
g94.t1 Caulerpa_lentillifera OG0000006
g95.t1 Caulerpa_lentillifera OG0000006
g957.t1 Caulerpa_lentillifera OG0000006
g958.t1 Caulerpa_lentillifera OG0000006
g991.t1 Caulerpa_lentillifera OG0000006
Cre02.g083050.t1.1 pacid=30785434 transcript=Cre02.g083050.t1.1 locus=Cre02.g083050 ID=Cre02.g083050.t1.1.v5.5 annot-version=v5.5 Chlamydomonas_reinhardtii OG0000006
Cre02.g099300.t1.2 pacid=30785454 transcript=Cre02.g099300.t1.2 locus=Cre02.g099300 ID=Cre02.g099300.t1.2.v5.5 annot-version=v5.5 Chlamydomonas_reinhardtii OG0000006
Cre02.g104600.t1.2 pacid=30785753 transcript=Cre02.g104600.t1.2 locus=Cre02.g104600 ID=Cre02.g104600.t1.2.v5.5 annot-version=v5.5 Chlamydomonas_reinhardtii OG0000006
Cre02.g116400.t1.1 pacid=30786151 transcript=Cre02.g116400.t1.1 locus=Cre02.g116400 ID=Cre02.g116400.t1.1.v5.5 annot-version=v5.5 Chlamydomonas_reinhardtii OG0000006
Cre02.g117550.t1.2 pacid=30786416 transcript=Cre02.g117550.t1.2 locus=Cre02.g117550 ID=Cre02.g117550.t1.2.v5.5 annot-version=v5.5 Chlamydomonas_reinhardtii OG0000006
Cre02.g095120.t1.1 pacid=30786455 transcript=Cre02.g095120.t1.1 locus=Cre02.g095120 ID=Cre02.g095120.t1.1.v5.5 annot-version=v5.5 Chlamydomonas_reinhardtii OG0000006
Cre03.g146867.t1.1 pacid=30786716 transcript=Cre03.g146867.t1.1 locus=Cre03.g146867 ID=Cre03.g146867.t1.1.v5.5 annot-version=v5.5 Chlamydomonas_reinhardtii OG0000006
Cre03.g177550.t1.2 pacid=30787416 transcript=Cre03.g177550.t1.2 locus=Cre03.g177550 ID=Cre03.g177550.t1.2.v5.5 annot-version=v5.5 Chlamydomonas_reinhardtii OG0000006
Cre03.g166000.t1.1 pacid=30787612 transcript=Cre03.g166000.t1.1 locus=Cre03.g166000 ID=Cre03.g166000.t1.1.v5.5 annot-version=v5.5 Chlamydomonas_reinhardtii OG0000006
Cre01.g008500.t1.2 pacid=30788844 transcript=Cre01.g008500.t1.2 locus=Cre01.g008500 ID=Cre01.g008500.t1.2.v5.5 annot-version=v5.5 Chlamydomonas_reinhardtii OG0000006

Cre01.g052150.t1.1 pacid=30789444 transcript=Cre01.g052150.t1.1 locus=Cre01.g052150 ID=Cre01.g052150.t1.1.v5.5 annot-version=v5.5 Chlamydomonas_reinhardtii OG0000006
Cre01.g001650.t1.2 pacid=30789638 transcript=Cre01.g001650.t1.2 locus=Cre01.g001650 ID=Cre01.g001650.t1.2.v5.5 annot-version=v5.5 Chlamydomonas_reinhardtii OG0000006
Cre10.g422750.t1.1 pacid=30789888 transcript=Cre10.g422750.t1.1 locus=Cre10.g422750 ID=Cre10.g422750.t1.1.v5.5 annot-version=v5.5 Chlamydomonas_reinhardtii OG0000006
Cre10.g449200.t1.1 pacid=30790055 transcript=Cre10.g449200.t1.1 locus=Cre10.g449200 ID=Cre10.g449200.t1.1.v5.5 annot-version=v5.5 Chlamydomonas_reinhardtii OG0000006
Cre04.g217908.t1.1 pacid=30791256 transcript=Cre04.g217908.t1.1 locus=Cre04.g217908 ID=Cre04.g217908.t1.1.v5.5 annot-version=v5.5 Chlamydomonas_reinhardtii OG0000006
Cre04.g225800.t1.2 pacid=30791284 transcript=Cre04.g225800.t1.2 locus=Cre04.g225800 ID=Cre04.g225800.t1.2.v5.5 annot-version=v5.5 Chlamydomonas_reinhardtii OG0000006
Cre12.g483600.t1.2 pacid=30791921 transcript=Cre12.g483600.t1.2 locus=Cre12.g483600 ID=Cre12.g483600.t1.2.v5.5 annot-version=v5.5 Chlamydomonas_reinhardtii OG0000006
Cre12.g484500.t1.2 pacid=30792787 transcript=Cre12.g484500.t1.2 locus=Cre12.g484500 ID=Cre12.g484500.t1.2.v5.5 annot-version=v5.5 Chlamydomonas_reinhardtii OG0000006
Cre12.g520000.t1.1 pacid=30793330 transcript=Cre12.g520000.t1.1 locus=Cre12.g520000 ID=Cre12.g520000.t1.1.v5.5 annot-version=v5.5 Chlamydomonas_reinhardtii OG0000006
Cre11.g482001.t1.1 pacid=30775566 transcript=Cre11.g482001.t1.1 locus=Cre11.g482001 ID=Cre11.g482001.t1.1.v5.5 annot-version=v5.5 Chlamydomonas_reinhardtii OG0000006
Cre11.g481866.t1.1 pacid=30775777 transcript=Cre11.g481866.t1.1 locus=Cre11.g481866 ID=Cre11.g481866.t1.1.v5.5 annot-version=v5.5 Chlamydomonas_reinhardtii OG0000006
Cre14.g614850.t1.1 pacid=30776426 transcript=Cre14.g614850.t1.1 locus=Cre14.g614850 ID=Cre14.g614850.t1.1.v5.5 annot-version=v5.5 Chlamydomonas_reinhardtii OG0000006
Cre14.g625500.t1.1 pacid=30776487 transcript=Cre14.g625500.t1.1 locus=Cre14.g625500 ID=Cre14.g625500.t1.1.v5.5 annot-version=v5.5 Chlamydomonas_reinhardtii OG0000006
Cre08.g366101.t1.1 pacid=30773700 transcript=Cre08.g366101.t1.1 locus=Cre08.g366101 ID=Cre08.g366101.t1.1.v5.5 annot-version=v5.5 Chlamydomonas_reinhardtii OG0000006
Cre16.g679221.t1.1 pacid=30777190 transcript=Cre16.g679221.t1.1 locus=Cre16.g679221 ID=Cre16.g679221.t1.1.v5.5 annot-version=v5.5 Chlamydomonas_reinhardtii OG0000006
Cre16.g662951.t1.2 pacid=30777372 transcript=Cre16.g662951.t1.2 locus=Cre16.g662951 ID=Cre16.g662951.t1.2.v5.5 annot-version=v5.5 Chlamydomonas_reinhardtii OG0000006
Cre06.g261950.t1.2 pacid=30778774 transcript=Cre06.g261950.t1.2 locus=Cre06.g261950 ID=Cre06.g261950.t1.2.v5.5 annot-version=v5.5 Chlamydomonas_reinhardtii OG0000006
Cre06.g304150.t1.1 pacid=30779210 transcript=Cre06.g304150.t1.1 locus=Cre06.g304150 ID=Cre06.g304150.t1.1.v5.5 annot-version=v5.5 Chlamydomonas_reinhardtii OG0000006

Cre06.g287650.t1.2 pacid=30779354 transcript=Cre06.g287650.t1.2 locus=Cre06.g287650 ID=Cre06.g287650.t1.2.v5.5 annot-version=v5.5 Chlamydomonas_reinhardtii OG0000006
Cre06.g283300.t1.2 pacid=30779621 transcript=Cre06.g283300.t1.2 locus=Cre06.g283300 ID=Cre06.g283300.t1.2.v5.5 annot-version=v5.5 Chlamydomonas_reinhardtii OG0000006
Cre09.g396549.t1.1 pacid=30780390 transcript=Cre09.g396549.t1.1 locus=Cre09.g396549 ID=Cre09.g396549.t1.1.v5.5 annot-version=v5.5 Chlamydomonas_reinhardtii OG0000006
Cre09.g396624.t1.1 pacid=30780844 transcript=Cre09.g396624.t1.1 locus=Cre09.g396624 ID=Cre09.g396624.t1.1.v5.5 annot-version=v5.5 Chlamydomonas_reinhardtii OG0000006
Cre17.g720700.t1.1 pacid=30781758 transcript=Cre17.g720700.t1.1 locus=Cre17.g720700 ID=Cre17.g720700.t1.1.v5.5 annot-version=v5.5 Chlamydomonas_reinhardtii OG0000006
Cre17.g746697.t1.1 pacid=30781947 transcript=Cre17.g746697.t1.1 locus=Cre17.g746697 ID=Cre17.g746697.t1.1.v5.5 annot-version=v5.5 Chlamydomonas_reinhardtii OG0000006
Cre17.g735350.t1.1 pacid=30782661 transcript=Cre17.g735350.t1.1 locus=Cre17.g735350 ID=Cre17.g735350.t1.1.v5.5 annot-version=v5.5 Chlamydomonas_reinhardtii OG0000006
Cre05.g245500.t1.1 pacid=30783005 transcript=Cre05.g245500.t1.1 locus=Cre05.g245500 ID=Cre05.g245500.t1.1.v5.5 annot-version=v5.5 Chlamydomonas_reinhardtii OG0000006
Cre05.g231100.t1.1 pacid=30783178 transcript=Cre05.g231100.t1.1 locus=Cre05.g231100 ID=Cre05.g231100.t1.1.v5.5 annot-version=v5.5 Chlamydomonas_reinhardtii OG0000006
Cre05.g248100.t1.2 pacid=30783343 transcript=Cre05.g248100.t1.2 locus=Cre05.g248100 ID=Cre05.g248100.t1.2.v5.5 annot-version=v5.5 Chlamydomonas_reinhardtii OG0000006
Cre05.g245700.t1.1 pacid=30783394 transcript=Cre05.g245700.t1.1 locus=Cre05.g245700 ID=Cre05.g245700.t1.1.v5.5 annot-version=v5.5 Chlamydomonas_reinhardtii OG0000006
Cre05.g236300.t1.1 pacid=30783471 transcript=Cre05.g236300.t1.1 locus=Cre05.g236300 ID=Cre05.g236300.t1.1.v5.5 annot-version=v5.5 Chlamydomonas_reinhardtii OG0000006
Cre05.g238052.t1.1 pacid=30783482 transcript=Cre05.g238052.t1.1 locus=Cre05.g238052 ID=Cre05.g238052.t1.1.v5.5 annot-version=v5.5 Chlamydomonas_reinhardtii OG0000006
Cre15.g634600.t1.1 pacid=30783682 transcript=Cre15.g634600.t1.1 locus=Cre15.g634600 ID=Cre15.g634600.t1.1.v5.5 annot-version=v5.5 Chlamydomonas_reinhardtii OG0000006
Cre15.g641527.t1.1 pacid=30783759 transcript=Cre15.g641527.t1.1 locus=Cre15.g641527 ID=Cre15.g641527.t1.1.v5.5 annot-version=v5.5 Chlamydomonas_reinhardtii OG0000006
Cre13.g573550.t1.1 pacid=30784497 transcript=Cre13.g573550.t1.1 locus=Cre13.g573550 ID=Cre13.g573550.t1.1.v5.5 annot-version=v5.5 Chlamydomonas_reinhardtii OG0000006
Cre13.g571520.t1.1 pacid=30784785 transcript=Cre13.g571520.t1.1 locus=Cre13.g571520 ID=Cre13.g571520.t1.1.v5.5 annot-version=v5.5 Chlamydomonas_reinhardtii OG0000006
Vocar.0001s1157.1.p pacid=32884361 transcript=Vocar.0001s1157.1 locus=Vocar.0001s1157 ID=Vocar.0001s1157.1.v2.1 annot-version=v2.1 Volvox_carteri OG0000006

Vocar.0070s0019.1.p pacid=32895831 transcript=Vocar.0070s0019.1 locus=Vocar.0070s0019 ID=Vocar.0070s0019.1.v2.1 annot-version=v2.1
Volvox_carteri OG0000006
Vocar.0079s0015.1.p pacid=32896113 transcript=Vocar.0079s0015.1 locus=Vocar.0079s0015 ID=Vocar.0079s0015.1.v2.1 annot-version=v2.1
Volvox_carteri OG0000006
Vocar.0013s0073.1.p pacid=32896231 transcript=Vocar.0013s0073.1 locus=Vocar.0013s0073 ID=Vocar.0013s0073.1.v2.1 annot-version=v2.1
Volvox_carteri OG0000006
Vocar.0001s1163.1.p pacid=32884554 transcript=Vocar.0001s1163.1 locus=Vocar.0001s1163 ID=Vocar.0001s1163.1.v2.1 annot-version=v2.1
Volvox_carteri OG0000006
Vocar.0008s0266.1.p pacid=32897256 transcript=Vocar.0008s0266.1 locus=Vocar.0008s0266 ID=Vocar.0008s0266.1.v2.1 annot-version=v2.1
Volvox_carteri OG0000006
Vocar.0039s0012.1.p pacid=32897844 transcript=Vocar.0039s0012.1 locus=Vocar.0039s0012 ID=Vocar.0039s0012.1.v2.1 annot-version=v2.1
Volvox_carteri OG0000006
Vocar.0015s0366.1.p pacid=32898851 transcript=Vocar.0015s0366.1 locus=Vocar.0015s0366 ID=Vocar.0015s0366.1.v2.1 annot-version=v2.1
Volvox_carteri OG0000006
Vocar.0001s0358.1.p pacid=32885530 transcript=Vocar.0001s0358.1 locus=Vocar.0001s0358 ID=Vocar.0001s0358.1.v2.1 annot-version=v2.1
Volvox_carteri OG0000006
Vocar.0001s1474.1.p pacid=32885693 transcript=Vocar.0001s1474.1 locus=Vocar.0001s1474 ID=Vocar.0001s1474.1.v2.1 annot-version=v2.1
Volvox_carteri OG0000006
Vocar.0001s1154.1.p pacid=32885816 transcript=Vocar.0001s1154.1 locus=Vocar.0001s1154 ID=Vocar.0001s1154.1.v2.1 annot-version=v2.1
Volvox_carteri OG0000006
Vocar.0063s0043.1.p pacid=32883471 transcript=Vocar.0063s0043.1 locus=Vocar.0063s0043 ID=Vocar.0063s0043.1.v2.1 annot-version=v2.1
Volvox_carteri OG0000006
Vocar.0033s0012.1.p pacid=32886422 transcript=Vocar.0033s0012.1 locus=Vocar.0033s0012 ID=Vocar.0033s0012.1.v2.1 annot-version=v2.1
Volvox_carteri OG0000006
Vocar.0004s0503.1.p pacid=32889619 transcript=Vocar.0004s0503.1 locus=Vocar.0004s0503 ID=Vocar.0004s0503.1.v2.1 annot-version=v2.1
Volvox_carteri OG0000006
Vocar.0031s0100.1.p pacid=32890159 transcript=Vocar.0031s0100.1 locus=Vocar.0031s0100 ID=Vocar.0031s0100.1.v2.1 annot-version=v2.1
Volvox_carteri OG0000006
Vocar.0002s0574.1.p pacid=32890603 transcript=Vocar.0002s0574.1 locus=Vocar.0002s0574 ID=Vocar.0002s0574.1.v2.1 annot-version=v2.1
Volvox_carteri OG0000006
Vocar.0002s0076.1.p pacid=32890727 transcript=Vocar.0002s0076.1 locus=Vocar.0002s0076 ID=Vocar.0002s0076.1.v2.1 annot-version=v2.1
Volvox_carteri OG0000006
Vocar.0002s0487.1.p pacid=32890754 transcript=Vocar.0002s0487.1 locus=Vocar.0002s0487 ID=Vocar.0002s0487.1.v2.1 annot-version=v2.1
Volvox_carteri OG0000006
Vocar.0002s0577.1.p pacid=32891095 transcript=Vocar.0002s0577.1 locus=Vocar.0002s0577 ID=Vocar.0002s0577.1.v2.1 annot-version=v2.1
Volvox_carteri OG0000006

Vocar.0022s0117.1.p pacid=32891776 transcript=Vocar.0022s0117.1 locus=Vocar.0022s0117 ID=Vocar.0022s0117.1.v2.1 annot-version=v2.1 Volvox_carteri OG0000006
Vocar.0009s0143.1.p pacid=32893326 transcript=Vocar.0009s0143.1 locus=Vocar.0009s0143 ID=Vocar.0009s0143.1.v2.1 annot-version=v2.1 Volvox_carteri OG0000006
Vocar.0009s0182.1.p pacid=32893453 transcript=Vocar.0009s0182.1 locus=Vocar.0009s0182 ID=Vocar.0009s0182.1.v2.1 annot-version=v2.1 Volvox_carteri OG0000006
Vocar.0009s0020.1.p pacid=32893457 transcript=Vocar.0009s0020.1 locus=Vocar.0009s0020 ID=Vocar.0009s0020.1.v2.1 annot-version=v2.1 Volvox_carteri OG0000006
Vocar.0009s0045.1.p pacid=32893463 transcript=Vocar.0009s0045.1 locus=Vocar.0009s0045 ID=Vocar.0009s0045.1.v2.1 annot-version=v2.1 Volvox_carteri OG0000006
Vocar.0009s0176.1.p pacid=32893523 transcript=Vocar.0009s0176.1 locus=Vocar.0009s0176 ID=Vocar.0009s0176.1.v2.1 annot-version=v2.1 Volvox_carteri OG0000006
Vocar.0006s0107.1.p pacid=32894092 transcript=Vocar.0006s0107.1 locus=Vocar.0006s0107 ID=Vocar.0006s0107.1.v2.1 annot-version=v2.1 Volvox_carteri OG0000006
jgi|ChlNC64A_1|12983|gw1.3.429.1 Chlorella_variabilis OG0000006
jgi|ChlNC64A_1|17581|gw1.3.770.1 Chlorella_variabilis OG0000006
jgi|ChlNC64A_1|18784|e_gw1.1.335.1 Chlorella_variabilis OG0000006
jgi|ChlNC64A_1|13829|gw1.24.226.1 Chlorella_variabilis OG0000006
jgi|ChlNC64A_1|133728|IGS.gm_10_00019 Chlorella_variabilis OG0000006
jgi|ChlNC64A_1|133822|IGS.gm_10_00113 Chlorella_variabilis OG0000006
jgi|ChlNC64A_1|133163|IGS.gm_1_00299 Chlorella_variabilis OG0000006
jgi|ChlNC64A_1|7157|gw1.10.25.1 Chlorella_variabilis OG0000006
jgi|ChlNC64A_1|133973|IGS.gm_10_00264 Chlorella_variabilis OG0000006
jgi|ChlNC64A_1|19334|e_gw1.2.298.1 Chlorella_variabilis OG0000006
jgi|ChlNC64A_1|12772|gw1.4.327.1 Chlorella_variabilis OG0000006
jgi|ChlNC64A_1|57095|estExt_fgenesh3_pg.C_40188 Chlorella_variabilis OG0000006
jgi|ChlNC64A_1|141821|IGS.gm_39_00001 Chlorella_variabilis OG0000006
jgi|ChlNC64A_1|134585|IGS.gm_11_00431 Chlorella_variabilis OG0000006
jgi|ChlNC64A_1|21705|e_gw1.6.190.1 Chlorella_variabilis OG0000006
jgi|ChlNC64A_1|14469|gw1.6.270.1 Chlorella_variabilis OG0000006
jgi|ChlNC64A_1|143805|IGS.gm_6_00379 Chlorella_variabilis

OG0000006
jgi|ChlNC64A_1|145427|IGS.gm_9_00234 Chlorella_variabilis
OG0000006
jgi|ChlNC64A_1|14335|gw1.21.143.1 Chlorella_variabilis
OG0000006
jgi|ChlNC64A_1|144464|IGS.gm_7_00406 Chlorella_variabilis
OG0000006
jgi|ChlNC64A_1|9728|gw1.7.75.1 Chlorella_variabilis
OG0000006
jgi|ChlNC64A_1|144591|IGS.gm_7_00533 Chlorella_variabilis
OG0000006
jgi|ChlNC64A_1|14719|gw1.5.482.1 Chlorella_variabilis
OG0000006
jgi|ChlNC64A_1|51747|fgenesh3_pg.C_scaffold_8000034
Chlorella_variabilis OG0000006
jgi|ChlNC64A_1|22241|e_gw1.8.472.1 Chlorella_variabilis
OG0000006
jgi|ChlNC64A_1|141505|IGS.gm_35_00017 Chlorella_variabilis
OG0000006
jgi|ChlNC64A_1|17618|gw1.22.199.1 Chlorella_variabilis
OG0000006
jgi|ChlNC64A_1|28265|e_gw1.32.90.1 Chlorella_variabilis
OG0000006
jgi|ChlNC64A_1|135171|IGS.gm_13_00099 Chlorella_variabilis
OG0000006
jgi|ChlNC64A_1|13257|gw1.13.163.1 Chlorella_variabilis
OG0000006
jgi|ChlNC64A_1|141330|IGS.gm_33_00040 Chlorella_variabilis
OG0000006
jgi|ChlNC64A_1|55321|fgenesh3_pg.C_scaffold_33000044
Chlorella_variabilis OG0000006
jgi|ChlNC64A_1|139888|IGS.gm_27_00066 Chlorella_variabilis
OG0000006
jgi|ChlNC64A_1|137272|IGS.gm_19_00039 Chlorella_variabilis
OG0000006
jgi|ChlNC64A_1|54058|fgenesh3_pg.C_scaffold_19000083
Chlorella_variabilis OG0000006
jgi|ChlNC64A_1|14566|gw1.19.182.1 Chlorella_variabilis
OG0000006
jgi|ChlNC64A_1|136194|IGS.gm_15_00290 Chlorella_variabilis
OG0000006
jgi|ChlNC64A_1|139979|IGS.gm_28_00010 Chlorella_variabilis
OG0000006
jgi|ChlNC64A_1|139989|IGS.gm_28_00020 Chlorella_variabilis
OG0000006
jgi|ChlNC64A_1|139993|IGS.gm_28_00024 Chlorella_variabilis
OG0000006
jgi|ChlNC64A_1|139999|IGS.gm_28_00030 Chlorella_variabilis
OG0000006
jgi|ChlNC64A_1|140002|IGS.gm_28_00033 Chlorella_variabilis
OG0000006
jgi|ChlNC64A_1|140003|IGS.gm_28_00034 Chlorella_variabilis
OG0000006
jgi|ChlNC64A_1|59197|estExt_fgenesh3_pg.C_280067

Chlorella_variabilis OG0000006
jgi|ChlNC64A_1|142585|IGS.gm_43_00017 Chlorella_variabilis
OG0000006
54237 pacid=27387874 transcript=54237
locus=estExt_fgenesh1_pm.C_140072 ID=54237.2.0.227 annot-
version=v2.0 Coccomyxa_subellipsoidea OG0000006
58605 pacid=27388381 transcript=58605 locus=Genemark1.7634_g
ID=58605.2.0.227 annot-version=v2.0 Coccomyxa_subellipsoidea
OG0000006
34884 pacid=27389547 transcript=34884 locus=fgenesh1_pm.1_#_225
ID=34884.2.0.227 annot-version=v2.0 Coccomyxa_subellipsoidea
OG0000006
7444 pacid=27394298 transcript=7444 locus=gw1.18.197.1
ID=7444.2.0.227 annot-version=v2.0 Coccomyxa_subellipsoidea
OG0000006
13370_fgenesh1_pg.C_Chr_13.0001000151 Ostreococcus_tauri
OG0000006
16947_e_gw1.04.00.150.1 Ostreococcus_tauri OG0000006
20423_e_gw1.12.00.354.1 Ostreococcus_tauri OG0000006
22642_e_gw1.20.00.71.1 Ostreococcus_tauri OG0000006
23631_estExt_fgenesh1_pg.C_Chr_04.00010248 Ostreococcus_tauri
OG0000006
24148_estExt_fgenesh1_pg.C_Chr_09.00010315 Ostreococcus_tauri
OG0000006
5339_gw1.05.00.352.1 Ostreococcus_tauri OG0000006
26354_estExt_genewise1.C_Chr_09.00010468 Ostreococcus_tauri
OG0000006
32205_0400010151 Ostreococcus_tauri OG0000006
6875_gw1.13.00.309.1 Ostreococcus_tauri OG0000006
6973_gw1.04.00.470.1 Ostreococcus_tauri OG0000006
33280_0600010127 Ostreococcus_tauri OG0000006
34162_0800010049 Ostreococcus_tauri OG0000006
34220_0800010107 Ostreococcus_tauri OG0000006
36031_1300010003 Ostreococcus_tauri OG0000006
36356_1400010007 Ostreococcus_tauri OG0000006
3710 pacid=27415246 transcript=3710 locus=gwEuk.4.264.1
ID=3710.2.0.231 annot-version=v2.0 Ostreococcus_lucimarinus
OG0000006
37212 pacid=27415487 transcript=37212 locus=e_gwEuk.4.582.1
ID=37212.2.0.231 annot-version=v2.0 Ostreococcus_lucimarinus
OG0000006
9151 pacid=27415573 transcript=9151 locus=gwEuk.4.608.1
ID=9151.2.0.231 annot-version=v2.0 Ostreococcus_lucimarinus
OG0000006
5133 pacid=27416598 transcript=5133 locus=gwEuk.5.291.1
ID=5133.2.0.231 annot-version=v2.0 Ostreococcus_lucimarinus
OG0000006
8694 pacid=27416985 transcript=8694 locus=gwEuk.12.369.1
ID=8694.2.0.231 annot-version=v2.0 Ostreococcus_lucimarinus
OG0000006
26723 pacid=27417062 transcript=26723 locus=eugene.1200010068
ID=26723.2.0.231 annot-version=v2.0 Ostreococcus_lucimarinus
OG0000006
15689 pacid=27418194 transcript=15689

locus=fgenesh1_pg.C_Chr_6000076 ID=15689.2.0.231 annot-version=v2.0
Ostreococcus_lucimarinus OG0000006
7246 pacid=27418650 transcript=7246 locus=gwEuk.13.336.1
ID=7246.2.0.231 annot-version=v2.0 Ostreococcus_lucimarinus
OG0000006
8728 pacid=27418770 transcript=8728 locus=gwEuk.13.387.1
ID=8728.2.0.231 annot-version=v2.0 Ostreococcus_lucimarinus
OG0000006
42708 pacid=27419233 transcript=42708 locus=e_gwEuk.8.401.1
ID=42708.2.0.231 annot-version=v2.0 Ostreococcus_lucimarinus
OG0000006
24893 pacid=27419555 transcript=24893
locus=estExt_fgenesh1_pg.C_Chr_80033 ID=24893.2.0.231 annot-version=v2.0 Ostreococcus_lucimarinus OG0000006
8045 pacid=27419587 transcript=8045 locus=gwEuk.8.446.1
ID=8045.2.0.231 annot-version=v2.0 Ostreococcus_lucimarinus
OG0000006
25814 pacid=27420225 transcript=25814
locus=estExt_fgenesh1_pg.C_Chr_200005 ID=25814.2.0.231 annot-version=v2.0 Ostreococcus_lucimarinus OG0000006
43531 pacid=27420330 transcript=43531 locus=e_gwEuk.20.444.1
ID=43531.2.0.231 annot-version=v2.0 Ostreococcus_lucimarinus
OG0000006
8897 pacid=27413717 transcript=8897 locus=gwEuk.9.522.1
ID=8897.2.0.231 annot-version=v2.0 Ostreococcus_lucimarinus
OG0000006
49015 pacid=27348675 transcript=49015
locus=MicpuC2.estExt_fgenesh1_pg.C_150201 ID=49015.3.0.228 annot-version=v3.0 Micromonas_pusilla OG0000006
9003 pacid=27340477 transcript=9003 locus=MicpuC2.gw1.10.423.1
ID=9003.3.0.228 annot-version=v3.0 Micromonas_pusilla
OG0000006
142920 pacid=27340493 transcript=142960 locus=gw1.10.691.1
ID=142960.3.0.228 annot-version=v3.0 Micromonas_pusilla
OG0000006
20210 pacid=27340514 transcript=20210 locus=MicpuC2.e_gw1.10.406.1
ID=20210.3.0.228 annot-version=v3.0 Micromonas_pusilla
OG0000006
18587 pacid=27341114 transcript=18587 locus=MicpuC2.e_gw1.7.301.1
ID=18587.3.0.228 annot-version=v3.0 Micromonas_pusilla
OG0000006
157322 pacid=27341143 transcript=157362 locus=e_gw1.7.143.1
ID=157362.3.0.228 annot-version=v3.0 Micromonas_pusilla
OG0000006
125232 pacid=27341432 transcript=125272
locus=estExt_fgenesh3_kg.C_50016 ID=125272.3.0.228 annot-version=v3.0 Micromonas_pusilla OG0000006
8385 pacid=27341562 transcript=8385 locus=MicpuC2.gw1.5.606.1
ID=8385.3.0.228 annot-version=v3.0 Micromonas_pusilla
OG0000006
67917 pacid=27341569 transcript=67917 locus=MicpuC2.est_orfs.5_6900_4275102:1 ID=67917.3.0.228 annot-version=v3.0
Micromonas_pusilla OG0000006
58300 pacid=27341760 transcript=58300 locus=MicpuC2.EuGene.

0000050605 ID=58300.3.0.228 annot-version=v3.0
Micromonas_pusilla OG0000006
142517 pacid=27342356 transcript=142557 locus=gw1.14.488.1
ID=142557.3.0.228 annot-version=v3.0 Micromonas_pusilla
OG0000006
192768 pacid=27342432 transcript=192808
locus=estExt_Genemark1.C_20257 ID=192808.3.0.228 annot-version=v3.0
Micromonas_pusilla OG0000006
54848 pacid=27343683 transcript=54848 locus=MicpuC2.EuGene.
0000190028 ID=54848.3.0.228 annot-version=v3.0
Micromonas_pusilla OG0000006
54852 pacid=27343726 transcript=54852 locus=MicpuC2.EuGene.
0000190032 ID=54852.3.0.228 annot-version=v3.0
Micromonas_pusilla OG0000006
182838 pacid=27343739 transcript=182878
locus=estExt_Genewise1Plus.C_190037 ID=182878.3.0.228 annot-
version=v3.0 Micromonas_pusilla OG0000006
19380 pacid=27339151 transcript=19380 locus=MicpuC2.e_gw1.8.485.1
ID=19380.3.0.228 annot-version=v3.0 Micromonas_pusilla
OG0000006
142411 pacid=27343760 transcript=142451 locus=gw1.19.99.1
ID=142451.3.0.228 annot-version=v3.0 Micromonas_pusilla
OG0000006
54846 pacid=27343764 transcript=54846 locus=MicpuC2.EuGene.
0000190026 ID=54846.3.0.228 annot-version=v3.0
Micromonas_pusilla OG0000006
129016 pacid=27343780 transcript=129056 locus=gw1.19.10.1
ID=129056.3.0.228 annot-version=v3.0 Micromonas_pusilla
OG0000006
23576 pacid=27344767 transcript=23576 locus=MicpuC2.e_gw1.17.174.1
ID=23576.3.0.228 annot-version=v3.0 Micromonas_pusilla
OG0000006
50878 pacid=27345149 transcript=50878 locus=MicpuC2.EuGene.
0000011102 ID=50878.3.0.228 annot-version=v3.0
Micromonas_pusilla OG0000006
61074 pacid=27345165 transcript=61074 locus=MicpuC2.est_orfs.
1_57_4271379:2 ID=61074.3.0.228 annot-version=v3.0
Micromonas_pusilla OG0000006
174587 pacid=27345527 transcript=174627
locus=estExt_Genewise1Plus.C_1_t10267 ID=174627.3.0.228 annot-
version=v3.0 Micromonas_pusilla OG0000006
70193 pacid=27345597 transcript=70193
locus=MicpuC2.AZW_fgenesh1_pg.C_scaffold_1000636 ID=70193.3.0.228
annot-version=v3.0 Micromonas_pusilla OG0000006
7046 pacid=27345905 transcript=7046 locus=MicpuC2.gw1.1.780.1
ID=7046.3.0.228 annot-version=v3.0 Micromonas_pusilla
OG0000006
11584 pacid=27346316 transcript=11584 locus=MicpuC2.gw1.4.954.1
ID=11584.3.0.228 annot-version=v3.0 Micromonas_pusilla
OG0000006
153905 pacid=27346579 transcript=153945 locus=e_gw1.4.1416.1
ID=153945.3.0.228 annot-version=v3.0 Micromonas_pusilla
OG0000006
138568 pacid=27347083 transcript=138608 locus=gw1.3.1034.1

ID=138608.3.0.228 annot-version=v3.0	Micromonas_pusilla
OG0000006
15020 pacid=27347146 transcript=15020 locus=MicpuC2.e_gw1.3.900.1
ID=15020.3.0.228 annot-version=v3.0	Micromonas_pusilla
OG0000006
38495 pacid=27347537 transcript=38495
locus=MicpuC2.fgenesh1_pg.C_scaffold_3000002 ID=38495.3.0.228 annot-version=v3.0	Micromonas_pusilla	OG0000006
121510 pacid=27348135 transcript=121550 locus=fgenesh3_kg.
6_#_20_#_isotig02061 ID=121550.3.0.228 annot-version=v3.0
Micromonas_pusilla	OG0000006
59077 pacid=27348340 transcript=59077 locus=MicpuC2.EuGene.
0000060586 ID=59077.3.0.228 annot-version=v3.0
Micromonas_pusilla	OG0000006
69733 pacid=27348391 transcript=69733 locus=MicpuC2.est_orfs.
6_8716_4278309:2 ID=69733.3.0.228 annot-version=v3.0
Micromonas_pusilla	OG0000006
59076 pacid=27348413 transcript=59076 locus=MicpuC2.EuGene.
0000060585 ID=59076.3.0.228 annot-version=v3.0
Micromonas_pusilla	OG0000006
22834 pacid=27348500 transcript=22834 locus=MicpuC2.e_gw1.15.239.1
ID=22834.3.0.228 annot-version=v3.0	Micromonas_pusilla
OG0000006
99206 pacid=27396479 transcript=99206
locus=fgenesh2_pg.C_Chr_02000582 ID=99206.3.0.229 annot-version=v3.0
Micromonas_sp._RCC299	OG0000006
74026 pacid=27396724 transcript=74026 locus=gw2.02.819.1
ID=74026.3.0.229 annot-version=v3.0	Micromonas_sp._RCC299
OG0000006
77042 pacid=27396794 transcript=77042 locus=gw2.02.1092.1
ID=77042.3.0.229 annot-version=v3.0	Micromonas_sp._RCC299
OG0000006
88925 pacid=27397187 transcript=88925 locus=e_gw2.16.198.1
ID=88925.3.0.229 annot-version=v3.0	Micromonas_sp._RCC299
OG0000006
56707 pacid=27397921 transcript=56707 locus=EuGene.0300010135
ID=56707.3.0.229 annot-version=v3.0	Micromonas_sp._RCC299
OG0000006
79900 pacid=27398168 transcript=79900 locus=e_gw2.03.745.1
ID=79900.3.0.229 annot-version=v3.0	Micromonas_sp._RCC299
OG0000006
99299 pacid=27398245 transcript=99299
locus=fgenesh2_pg.C_Chr_03000080 ID=99299.3.0.229 annot-version=v3.0
Micromonas_sp._RCC299	OG0000006
56643 pacid=27398282 transcript=56643 locus=EuGene.0300010071
ID=56643.3.0.229 annot-version=v3.0	Micromonas_sp._RCC299
OG0000006
77288 pacid=27399190 transcript=77288 locus=e_gw2.01.675.1
ID=77288.3.0.229 annot-version=v3.0	Micromonas_sp._RCC299
OG0000006
82253 pacid=27395683 transcript=82253 locus=e_gw2.05.954.1
ID=82253.3.0.229 annot-version=v3.0	Micromonas_sp._RCC299
OG0000006
63054 pacid=27399729 transcript=63054 locus=EuGene.1200010226

ID=63054.3.0.229 annot-version=v3.0	Micromonas_sp._RCC299	OG0000006
73919 pacid=27399769 transcript=73919 locus=gw2.12.448.1 ID=73919.3.0.229 annot-version=v3.0	Micromonas_sp._RCC299	OG0000006
73069 pacid=27400089 transcript=73069 locus=gw2.04.631.1 ID=73069.3.0.229 annot-version=v3.0	Micromonas_sp._RCC299	OG0000006
88847 pacid=27400804 transcript=88847 locus=e_gw2.15.285.1 ID=88847.3.0.229 annot-version=v3.0	Micromonas_sp._RCC299	OG0000006
74023 pacid=27400851 transcript=74023 locus=gw2.15.283.1 ID=74023.3.0.229 annot-version=v3.0	Micromonas_sp._RCC299	OG0000006
61039 pacid=27401313 transcript=61039 locus=EuGene.0900010022 ID=61039.3.0.229 annot-version=v3.0	Micromonas_sp._RCC299	OG0000006
108957 pacid=27401397 transcript=108957 locus=est_cluster_kg.Chr_09__121__3199135:1 ID=108957.3.0.229 annot-version=v3.0	Micromonas_sp._RCC299	OG0000006
61158 pacid=27401731 transcript=61158 locus=EuGene.0900010141 ID=61158.3.0.229 annot-version=v3.0	Micromonas_sp._RCC299	OG0000006
105748 pacid=27401786 transcript=105748 locus=estExt_fgenesh2_pg.C_Chr_060304 ID=105748.3.0.229 annot-version=v3.0	Micromonas_sp._RCC299	OG0000006
73989 pacid=27402052 transcript=73989 locus=gw2.06.651.1 ID=73989.3.0.229 annot-version=v3.0	Micromonas_sp._RCC299	OG0000006
76461 pacid=27402420 transcript=76461 locus=gw2.06.860.1 ID=76461.3.0.229 annot-version=v3.0	Micromonas_sp._RCC299	OG0000006
108468 pacid=27402473 transcript=108468 locus=est_cluster_kg.Chr_06__112__3202508:1 ID=108468.3.0.229 annot-version=v3.0	Micromonas_sp._RCC299	OG0000006
73997 pacid=27402754 transcript=73997 locus=gw2.13.437.1 ID=73997.3.0.229 annot-version=v3.0	Micromonas_sp._RCC299	OG0000006
63626 pacid=27402859 transcript=63626 locus=EuGene.1300010276 ID=63626.3.0.229 annot-version=v3.0	Micromonas_sp._RCC299	OG0000006
74009 pacid=27396022 transcript=74009 locus=gw2.02.817.1 ID=74009.3.0.229 annot-version=v3.0	Micromonas_sp._RCC299	OG0000006
76994 pacid=27402911 transcript=76994 locus=gw2.13.578.1 ID=76994.3.0.229 annot-version=v3.0	Micromonas_sp._RCC299	OG0000006
106453 pacid=27402945 transcript=106453 locus=estExt_fgenesh2_pg.C_Chr_130300 ID=106453.3.0.229 annot-version=v3.0	Micromonas_sp._RCC299	OG0000006
53076 pacid=27403191 transcript=53076 locus=est_orfs.Chr_08_2947_3200577:1 ID=53076.3.0.229 annot-version=v3.0	Micromonas_sp._RCC299	OG0000006
73744 pacid=27396064 transcript=73744 locus=gw2.02.796.1

ID=73744.3.0.229 annot-version=v3.0	Micromonas_sp._RCC299
OG0000006
60434 pacid=27403354 transcript=60434 locus=EuGene.0800010002
ID=60434.3.0.229 annot-version=v3.0	Micromonas_sp._RCC299
OG0000006
73223 pacid=27403618 transcript=73223 locus=gw2.08.481.1
ID=73223.3.0.229 annot-version=v3.0	Micromonas_sp._RCC299
OG0000006
88051 pacid=27403880 transcript=88051 locus=e_gw2.14.382.1
ID=88051.3.0.229 annot-version=v3.0	Micromonas_sp._RCC299
OG0000006
60367 pacid=27404267 transcript=60367 locus=EuGene.0700010652
ID=60367.3.0.229 annot-version=v3.0	Micromonas_sp._RCC299
OG0000006
86471 pacid=27404782 transcript=86471 locus=e_gw2.11.665.1
ID=86471.3.0.229 annot-version=v3.0	Micromonas_sp._RCC299
OG0000006
72498 pacid=27405083 transcript=72498 locus=gw2.11.425.1
ID=72498.3.0.229 annot-version=v3.0	Micromonas_sp._RCC299
OG0000006
74266 pacid=27405297 transcript=74266 locus=gw2.11.553.1
ID=74266.3.0.229 annot-version=v3.0	Micromonas_sp._RCC299
OG0000006
kfl00354_0020_v1.1	Klebsormidium_flaccidum	OG0000006
kfl00383_0140_v1.1	Klebsormidium_flaccidum	OG0000006
kfl00019_g43_v1.1	Klebsormidium_flaccidum	OG0000006
kfl00652_0040_v1.1	Klebsormidium_flaccidum	OG0000006
kfl00708_0040_v1.1	Klebsormidium_flaccidum	OG0000006
kfl00765_0050_v1.1	Klebsormidium_flaccidum	OG0000006
kfl01094_0020_v1.1	Klebsormidium_flaccidum	OG0000006
kfl01129_0020_v1.1	Klebsormidium_flaccidum	OG0000006
kfl01326_0010_v1.1	Klebsormidium_flaccidum	OG0000006
kfl00049_0220_v1.1	Klebsormidium_flaccidum	OG0000006
kfl00054_0290_v1.1	Klebsormidium_flaccidum	OG0000006
kfl00005_0630_v1.1	Klebsormidium_flaccidum	OG0000006
kfl00007_0120_v1.1	Klebsormidium_flaccidum	OG0000006
kfl00001_0580_v1.1	Klebsormidium_flaccidum	OG0000006
kfl00170_0090_v1.1	Klebsormidium_flaccidum	OG0000006
kfl00009_0600_v1.1	Klebsormidium_flaccidum	OG0000006
kfl00226_0160_v1.1	Klebsormidium_flaccidum	OG0000006
kfl00231_0200_v1.1	Klebsormidium_flaccidum	OG0000006
kfl00236_0100_v1.1	Klebsormidium_flaccidum	OG0000006
kfl00255_0150_v1.1	Klebsormidium_flaccidum	OG0000006
kfl00261_0120_v1.1	Klebsormidium_flaccidum	OG0000006
Pp3c2_27120V3.1.p pacid=32934058 transcript=Pp3c2_27120V3.1
locus=Pp3c2_27120 annot-version=v3.3	Physcomitrella_patens
OG0000006
Pp3c3_11090V3.1.p pacid=32944642 transcript=Pp3c3_11090V3.1
locus=Pp3c3_11090 annot-version=v3.3	Physcomitrella_patens
OG0000006
Pp3c3_30370V3.1.p pacid=32944838 transcript=Pp3c3_30370V3.1
locus=Pp3c3_30370 annot-version=v3.3	Physcomitrella_patens
OG0000006
Pp3c20_1240V3.1.p pacid=32946343 transcript=Pp3c20_1240V3.1

locus=Pp3c20_1240 annot-version=v3.3	Physcomitrella_patens
OG0000006
Pp3c20_7810V3.1.p pacid=32946448 transcript=Pp3c20_7810V3.1
locus=Pp3c20_7810 annot-version=v3.3	Physcomitrella_patens
OG0000006
Pp3c23_12540V3.1.p pacid=32950086 transcript=Pp3c23_12540V3.1
locus=Pp3c23_12540 annot-version=v3.3	Physcomitrella_patens
OG0000006
Pp3c23_6190V3.1.p pacid=32950826 transcript=Pp3c23_6190V3.1
locus=Pp3c23_6190 annot-version=v3.3	Physcomitrella_patens
OG0000006
Pp3c23_21680V3.1.p pacid=32950948 transcript=Pp3c23_21680V3.1
locus=Pp3c23_21680 annot-version=v3.3	Physcomitrella_patens
OG0000006
Pp3c8_14440V3.1.p pacid=32963879 transcript=Pp3c8_14440V3.1
locus=Pp3c8_14440 annot-version=v3.3	Physcomitrella_patens
OG0000006
Pp3c8_640V3.1.p pacid=32965877 transcript=Pp3c8_640V3.1
locus=Pp3c8_640 annot-version=v3.3	Physcomitrella_patens
OG0000006
Pp3c6_3070V3.1.p pacid=32975837 transcript=Pp3c6_3070V3.1
locus=Pp3c6_3070 annot-version=v3.3	Physcomitrella_patens
OG0000006
Pp3c6_21850V3.1.p pacid=32978492 transcript=Pp3c6_21850V3.1
locus=Pp3c6_21850 annot-version=v3.3	Physcomitrella_patens
OG0000006
Pp3c25_10520V3.1.p pacid=32979921 transcript=Pp3c25_10520V3.1
locus=Pp3c25_10520 annot-version=v3.3	Physcomitrella_patens
OG0000006
Pp3c10_23790V3.1.p pacid=32900289 transcript=Pp3c10_23790V3.1
locus=Pp3c10_23790 annot-version=v3.3	Physcomitrella_patens
OG0000006
Pp3c26_10740V3.1.p pacid=32917107 transcript=Pp3c26_10740V3.1
locus=Pp3c26_10740 annot-version=v3.3	Physcomitrella_patens
OG0000006
Pp3c26_6440V3.1.p pacid=32918146 transcript=Pp3c26_6440V3.1
locus=Pp3c26_6440 annot-version=v3.3	Physcomitrella_patens
OG0000006
Pp3c10_11210V3.1.p pacid=32901295 transcript=Pp3c10_11210V3.1
locus=Pp3c10_11210 annot-version=v3.3	Physcomitrella_patens
OG0000006
Pp3c4_19240V3.1.p pacid=32919641 transcript=Pp3c4_19240V3.1
locus=Pp3c4_19240 annot-version=v3.3	Physcomitrella_patens
OG0000006
Pp3c4_24490V3.1.p pacid=32921373 transcript=Pp3c4_24490V3.1
locus=Pp3c4_24490 annot-version=v3.3	Physcomitrella_patens
OG0000006
Pp3c7_27080V3.1.p pacid=32923776 transcript=Pp3c7_27080V3.1
locus=Pp3c7_27080 annot-version=v3.3	Physcomitrella_patens
OG0000006
Pp3c7_14210V3.1.p pacid=32924467 transcript=Pp3c7_14210V3.1
locus=Pp3c7_14210 annot-version=v3.3	Physcomitrella_patens
OG0000006
AT1G10340.1 | Ankyrin repeat family protein | Chr1:3390475-3392481

REVERSE LENGTH=578 | 201606	Arabidopsis_thaliana
OG0000006
AT3G04140.1 | Ankyrin repeat family protein | Chr3:1087063-1089106
FORWARD LENGTH=656 | 201606	Arabidopsis_thaliana
OG0000006
AT3G04710.1 | ankyrin repeat family protein | Chr3:1278229-1280942
FORWARD LENGTH=456 | 201606	Arabidopsis_thaliana
OG0000006
AT3G09550.1 | Ankyrin repeat family protein | Chr3:2932007-2934199
FORWARD LENGTH=607 | 201606	Arabidopsis_thaliana
OG0000006
AT3G09890.1 | Ankyrin repeat family protein | Chr3:3032678-3034158
FORWARD LENGTH=206 | 201606	Arabidopsis_thaliana
OG0000006
AT3G12360.1 | Ankyrin repeat family protein | Chr3:3934146-3936495
FORWARD LENGTH=590 | 201606	Arabidopsis_thaliana
OG0000006
AT1G14480.1 | Ankyrin repeat family protein | Chr1:4956404-4957888
FORWARD LENGTH=441 | 201606	Arabidopsis_thaliana
OG0000006
AT1G14500.1 | Ankyrin repeat family protein | Chr1:4960375-4961780
FORWARD LENGTH=436 | 201606	Arabidopsis_thaliana
OG0000006
AT3G52830.1 | ankyrin repeat protein | Chr3:19578005-19578265
FORWARD LENGTH=86 | 201606	Arabidopsis_thaliana
OG0000006
AT4G10720.1 | Ankyrin repeat family protein | Chr4:6607879-6609358
FORWARD LENGTH=445 | 201606	Arabidopsis_thaliana
OG0000006
AT4G19150.1 | Ankyrin repeat family protein | Chr4:10471578-10472677
REVERSE LENGTH=243 | 201606	Arabidopsis_thaliana
OG0000006
AT5G02620.1 | ankyrin-like1 | Chr5:589666-591536 FORWARD LENGTH=524
| 201606 Arabidopsis_thaliana	OG0000006
AT5G07270.1 | hypothetical protein | Chr5:2280821-2283384 FORWARD
LENGTH=513 | 201606	Arabidopsis_thaliana	OG0000006
AT5G12320.1 | ankyrin repeat family protein | Chr5:3982762-3983899
FORWARD LENGTH=144 | 201606	Arabidopsis_thaliana
OG0000006
AT5G15500.1 | Ankyrin repeat family protein | Chr5:5031791-5032846
REVERSE LENGTH=351 | 201606	Arabidopsis_thaliana
OG0000006
AT5G40160.1 | Ankyrin repeat family protein | Chr5:16062726-16064301
REVERSE LENGTH=315 | 201606	Arabidopsis_thaliana
OG0000006
AT5G50140.1 | Ankyrin repeat family protein | Chr5:20395856-20398197
FORWARD LENGTH=535 | 201606	Arabidopsis_thaliana
OG0000006
AT5G51160.1 | Ankyrin repeat family protein | Chr5:20792280-20793681
FORWARD LENGTH=442 | 201606	Arabidopsis_thaliana
OG0000006
AT5G54610.1 | ankyrin | Chr5:22184781-22186481 REVERSE LENGTH=426 |
201606	Arabidopsis_thaliana	OG0000006
AT5G54620.1 | Ankyrin repeat family protein | Chr5:22187761-22189746

REVERSE LENGTH=431 | 201606 Arabidopsis_thaliana
OG0000006
AT5G54700.1 | Ankyrin repeat family protein | Chr5:22223096-22225509
REVERSE LENGTH=480 | 201606 Arabidopsis_thaliana
OG0000006
AT5G54710.1 | Ankyrin repeat family protein | Chr5:22227665-22230500
REVERSE LENGTH=598 | 201606 Arabidopsis_thaliana
OG0000006
AT5G57740.1 | hypothetical protein | Chr5:23394789-23397145 REVERSE
LENGTH=508 | 201606 Arabidopsis_thaliana OG0000006
AT5G60070.1 | ankyrin repeat family protein | Chr5:24190440-24192570
REVERSE LENGTH=548 | 201606 Arabidopsis_thaliana
OG0000006
AT5G66055.1 | ankyrin repeat protein | Chr5:26417425-26419234
REVERSE LENGTH=435 | 201606 Arabidopsis_thaliana
OG0000006
AT1G34046.1 | Ankyrin-repeat containing protein |
Chr1:12388673-12389259 FORWARD LENGTH=97 | 201606
Arabidopsis_thaliana OG0000006
AT1G34050.1 | Ankyrin repeat family protein | Chr1:12393495-12396006
FORWARD LENGTH=573 | 201606 Arabidopsis_thaliana
OG0000006
AT1G05640.1 | Ankyrin repeat family protein | Chr1:1687436-1689501
REVERSE LENGTH=627 | 201606 Arabidopsis_thaliana
OG0000006
AT1G07710.1 | Ankyrin repeat family protein | Chr1:2386275-2387986
REVERSE LENGTH=543 | 201606 Arabidopsis_thaliana
OG0000006
AT2G03430.1 | Ankyrin repeat family protein | Chr2:1036192-1037536
REVERSE LENGTH=240 | 201606 Arabidopsis_thaliana
OG0000006
AT2G24600.1 | Ankyrin repeat family protein | Chr2:10452430-10454414
REVERSE LENGTH=548 | 201606 Arabidopsis_thaliana
OG0000006
AT2G28840.1 | hypothetical protein | Chr2:12378542-12380474 FORWARD
LENGTH=456 | 201606 Arabidopsis_thaliana OG0000006
AT2G31820.1 | Ankyrin repeat family protein | Chr2:13530350-13532562
FORWARD LENGTH=662 | 201606 Arabidopsis_thaliana
OG0000006
Os03t0275300-01 Hypothetical conserved gene. Oryza_sativa
OG0000006
Os03t0281000-01 Similar to Ankyrin-like protein. Oryza_sativa
OG0000006
Os03t0281100-00 Ankyrin repeat containing protein. Oryza_sativa
OG0000006
Os03t0621400-01 Hypothetical conserved gene. Oryza_sativa
OG0000006
Os03t0655500-00 Ankyrin domain containing protein. Oryza_sativa
OG0000006
Os03t0679800-01 Similar to TPR Domain containing protein.
Oryza_sativa OG0000006
Os03t0680100-00 Similar to TPR Domain containing protein.
Oryza_sativa OG0000006
Os03t0680200-00 Ankyrin repeat domain containing protein.

Oryza_sativa	OG0000006
Os03t0680300-00 Similar to TPR Domain containing protein.	Oryza_sativa	OG0000006
Os03t0680400-00 Ankyrin repeat domain containing protein.	Oryza_sativa	OG0000006
Os03t0680500-01 Ankyrin repeat domain containing protein.	Oryza_sativa	OG0000006
Os03t0680566-00 Tetratricopeptide TPR-1 domain containing protein.	Oryza_sativa	OG0000006
Os03t0680600-00 Ankyrin repeat domain containing protein.	Oryza_sativa	OG0000006
Os03t0758400-01 Ankyrin repeat containing protein.	Oryza_sativa	OG0000006
Os05t0103600-01 Similar to Ankyrin-like protein.	Oryza_sativa	OG0000006
Os05t0112000-01 E3 ubiquitin ligase, XA21-mediated disease resistance	Oryza_sativa	OG0000006
Os06t0128700-01 Ankyrin repeat domain containing protein.	Oryza_sativa	OG0000006
Os07t0532500-01 Ankyrin repeat containing protein.	Oryza_sativa	OG0000006
Os08t0233400-01 Ankyrin repeat domain containing protein.	Oryza_sativa	OG0000006
Os08t0258200-01 Ankyrin domain containing protein.	Oryza_sativa	OG0000006
Os08t0325267-00 Similar to TPR Domain containing protein, expressed.	Oryza_sativa	OG0000006
Os08t0542700-01 Ankyrin repeat containing protein.	Oryza_sativa	OG0000006
Os09t0123500-01 Tetratricopeptide-like helical domain containing protein.	Oryza_sativa	OG0000006
Os09t0124100-00 Ankyrin repeat domain containing protein.	Oryza_sativa	OG0000006
Os09t0124800-01 Tetratricopeptide-like helical domain containing protein.	Oryza_sativa	OG0000006
Os09t0518500-01 Ankyrin repeat containing protein.	Oryza_sativa	OG0000006
Os10t0521600-01 Similar to Ankyrin-1 (Erythrocyte ankyrin).	Oryza_sativa	OG0000006
Os10t0580700-01 Ankyrin repeat containing protein.	Oryza_sativa	OG0000006
Os11t0146200-01 Ankyrin repeat domain containing protein.	Oryza_sativa	OG0000006
Os11t0251200-00 Ankyrin repeat domain containing protein.	Oryza_sativa	OG0000006
Os11t0252400-01 Ankyrin repeat containing protein.	Oryza_sativa	OG0000006
Os11t0253100-00 Ankyrin repeat domain containing protein.	Oryza_sativa	OG0000006
Os11t0269500-00 Similar to HAT family dimerisation domain containing protein.	Oryza_sativa	OG0000006
Os12t0599900-01 Tetratricopeptide-like helical domain containing protein.	Oryza_sativa	OG0000006
Os12t0600100-01 Tetratricopeptide-like helical domain containing

protein. Oryza_sativa   OG0000006
Os12t0634900-01 Ankyrin repeat domain containing protein.
Oryza_sativa   OG0000006
Os12t0636100-01 Tetratricopeptide-like helical domain containing
protein. Oryza_sativa   OG0000006
Os01t0829100-01 Ankyrin domain containing protein. Oryza_sativa
OG0000006
Os01t0837000-01 Ankyrin repeat containing protein. Oryza_sativa
OG0000006
Os01t0974400-01 Zinc finger, RING/FYVE/PHD-type domain containing
protein. Oryza_sativa   OG0000006
Os01t0171100-00 Ankyrin repeat domain containing protein.
Oryza_sativa   OG0000006
Os01t0175400-01 Ankyrin repeat containing protein. Oryza_sativa
OG0000006
Os02t0491900-00 Ankyrin repeat domain containing protein.
Oryza_sativa   OG0000006
Os02t0492000-01 Ankyrin repeat containing protein. Oryza_sativa
OG0000006
Os02t0492600-00 Tetratricopeptide-like helical domain containing
protein. Oryza_sativa   OG0000006
Os02t0492900-01 Similar to Ankyrin-like protein-like protein.
Oryza_sativa   OG0000006
Os02t0493050-00 Ankyrin repeat domain containing protein.
Oryza_sativa   OG0000006
Os02t0493300-01 Tetratricopeptide-like helical domain containing
protein. Oryza_sativa   OG0000006
Os02t0494000-00 Ankyrin repeat domain containing protein.
Oryza_sativa   OG0000006
Os02t0494400-01 Similar to TPR Domain containing protein, expressed.
Oryza_sativa   OG0000006
Os01t0188900-01 Similar to Ankyrin-like protein-like protein.
Oryza_sativa   OG0000006
Os01t0189100-01 Ankyrin domain containing protein. Oryza_sativa
OG0000006
Os02t0791200-01 Ankyrin domain containing protein. Oryza_sativa
OG0000006
Os03t0135600-01 Similar to Ankyrin repeat protein. Oryza_sativa
OG0000006
Os03t0145800-01 Ankyrin repeat containing protein. Oryza_sativa
OG0000006
CMS172CT pep chromosome:ASM9120v1:19:451401:454911:-1 gene:CMS172C
transcript:CMS172CT gene_biotype:protein_coding
transcript_biotype:protein_coding Cyanidioschyzon_merolae
OG0000006
CMQ294CT pep chromosome:ASM9120v1:17:759987:761877:-1 gene:CMQ294C
transcript:CMQ294CT gene_biotype:protein_coding
transcript_biotype:protein_coding Cyanidioschyzon_merolae
OG0000006
CMS255CT pep chromosome:ASM9120v1:19:653859:654615:-1 gene:CMS255C
transcript:CMS255CT gene_biotype:protein_coding
transcript_biotype:protein_coding Cyanidioschyzon_merolae
OG0000006
CMT367CT pep chromosome:ASM9120v1:20:914095:914641:-1 gene:CMT367C

transcript:CMT367CT gene_biotype:protein_coding
transcript_biotype:protein_coding Cyanidioschyzon_merolae
OG0000006
CME092CT pep chromosome:ASM9120v1:5:255785:258518:-1 gene:CME092C
transcript:CME092CT gene_biotype:protein_coding
transcript_biotype:protein_coding Cyanidioschyzon_merolae
OG0000006
CMT161CT pep chromosome:ASM9120v1:20:418162:418849:-1 gene:CMT161C
transcript:CMT161CT gene_biotype:protein_coding
transcript_biotype:protein_coding Cyanidioschyzon_merolae
OG0000006
evm.model.contig_2122.19 Porphyridium_purpureum OG0000006
evm.model.contig_2149.19 Porphyridium_purpureum OG0000006
evm.model.contig_2180.4 Porphyridium_purpureum OG0000006
evm.model.contig_2181.1 Porphyridium_purpureum OG0000006
evm.model.contig_2211.5 Porphyridium_purpureum OG0000006
evm.model.contig_2295.4 Porphyridium_purpureum OG0000006
evm.model.contig_2301.4 Porphyridium_purpureum OG0000006
evm.model.contig_2349.5 Porphyridium_purpureum OG0000006
evm.model.contig_2360.1 Porphyridium_purpureum OG0000006
evm.model.contig_2688.2 Porphyridium_purpureum OG0000006
evm.model.contig_3400.5 Porphyridium_purpureum OG0000006
evm.model.contig_3423.7 Porphyridium_purpureum OG0000006
evm.model.contig_3435.6 Porphyridium_purpureum OG0000006
evm.model.contig_3451.14 Porphyridium_purpureum OG0000006
evm.model.contig_3451.13 Porphyridium_purpureum OG0000006
evm.model.contig_3452.5 Porphyridium_purpureum OG0000006
evm.model.contig_3460.3 Porphyridium_purpureum OG0000006
evm.model.contig_3524.10 Porphyridium_purpureum OG0000006
evm.model.contig_3579.3 Porphyridium_purpureum OG0000006
evm.model.contig_3618.1 Porphyridium_purpureum OG0000006
evm.model.contig_3627.1 Porphyridium_purpureum OG0000006
evm.model.contig_3722.1 Porphyridium_purpureum OG0000006
evm.model.contig_3805.1 Porphyridium_purpureum OG0000006
evm.model.contig_3819.1 Porphyridium_purpureum OG0000006
evm.model.contig_4416.13 Porphyridium_purpureum OG0000006
evm.model.contig_4417.2 Porphyridium_purpureum OG0000006
evm.model.contig_4428.8 Porphyridium_purpureum OG0000006
evm.model.contig_4431.8 Porphyridium_purpureum OG0000006
evm.model.contig_4436.2 Porphyridium_purpureum OG0000006
evm.model.contig_4448.8 Porphyridium_purpureum OG0000006
evm.model.contig_4489.1 Porphyridium_purpureum OG0000006
evm.model.contig_4493.6 Porphyridium_purpureum OG0000006
evm.model.contig_4539.1 Porphyridium_purpureum OG0000006
evm.model.contig_454.17 Porphyridium_purpureum OG0000006
evm.model.contig_4593.3 Porphyridium_purpureum OG0000006
evm.model.contig_4621.1 Porphyridium_purpureum OG0000006
evm.model.contig_523.1 Porphyridium_purpureum OG0000006
evm.model.contig_527.18 Porphyridium_purpureum OG0000006
evm.model.contig_527.16 Porphyridium_purpureum OG0000006
evm.model.contig_527.17 Porphyridium_purpureum OG0000006
evm.model.contig_584.2 Porphyridium_purpureum OG0000006
evm.model.contig_590.3 Porphyridium_purpureum OG0000006
CDF35071 pep supercontig:ASM35022v2:HG001717:101590:102932:1

gene:CHC_T00003558001 transcript:CDF35071
gene_biotype:protein_coding transcript_biotype:protein_coding
Chondrus_crispus OG0000006
CDF34295 pep supercontig:ASM35022v2:HG001684:187484:190291:1
gene:CHC_T00003015001 transcript:CDF34295
gene_biotype:protein_coding transcript_biotype:protein_coding
Chondrus_crispus OG0000006
CDF39470 pep supercontig:ASM35022v2:HG002041:20967:21530:1
gene:CHC_T00000297001 transcript:CDF39470
gene_biotype:protein_coding transcript_biotype:protein_coding
Chondrus_crispus OG0000006
CDF33838 pep supercontig:ASM35022v2:HG001659:87806:88765:1
gene:CHC_T00002543001 transcript:CDF33838
gene_biotype:protein_coding transcript_biotype:protein_coding
Chondrus_crispus OG0000006
CDF39250 pep supercontig:ASM35022v2:HG002013:13291:19019:1
gene:CHC_T00000180001 transcript:CDF39250
gene_biotype:protein_coding transcript_biotype:protein_coding
Chondrus_crispus OG0000006
CDF39229 pep supercontig:ASM35022v2:HG002012:349776:352775:-1
gene:CHC_T00006488001 transcript:CDF39229
gene_biotype:protein_coding transcript_biotype:protein_coding
Chondrus_crispus OG0000006
CDF38499 pep supercontig:ASM35022v2:HG001939:46582:48341:1
gene:CHC_T00001108001 transcript:CDF38499
gene_biotype:protein_coding transcript_biotype:protein_coding
Chondrus_crispus OG0000006
g1295.t1 Caulerpa_lentillifera OG0000007
g1714.t1 Caulerpa_lentillifera OG0000007
g1839.t1 Caulerpa_lentillifera OG0000007
g2272.t1 Caulerpa_lentillifera OG0000007
g2447.t1 Caulerpa_lentillifera OG0000007
g2571.t1 Caulerpa_lentillifera OG0000007
g2894.t1 Caulerpa_lentillifera OG0000007
g2941.t1 Caulerpa_lentillifera OG0000007
g2942.t1 Caulerpa_lentillifera OG0000007
g2943.t1 Caulerpa_lentillifera OG0000007
g3046.t1 Caulerpa_lentillifera OG0000007
g3156.t1 Caulerpa_lentillifera OG0000007
g3157.t1 Caulerpa_lentillifera OG0000007
g3161.t1 Caulerpa_lentillifera OG0000007
g3165.t1 Caulerpa_lentillifera OG0000007
g3168.t1 Caulerpa_lentillifera OG0000007
g3172.t1 Caulerpa_lentillifera OG0000007
g3173.t1 Caulerpa_lentillifera OG0000007
g3878.t1 Caulerpa_lentillifera OG0000007
g4076.t1 Caulerpa_lentillifera OG0000007
g4077.t1 Caulerpa_lentillifera OG0000007
g4078.t1 Caulerpa_lentillifera OG0000007
g4130.t1 Caulerpa_lentillifera OG0000007
g4283.t1 Caulerpa_lentillifera OG0000007
g4379.t1 Caulerpa_lentillifera OG0000007
g4519.t1 Caulerpa_lentillifera OG0000007
g4897.t1 Caulerpa_lentillifera OG0000007

g5098.t1 Caulerpa_lentillifera OG0000007
g5561.t1 Caulerpa_lentillifera OG0000007
g5655.t1 Caulerpa_lentillifera OG0000007
g5762.t1 Caulerpa_lentillifera OG0000007
g5799.t1 Caulerpa_lentillifera OG0000007
g5827.t1 Caulerpa_lentillifera OG0000007
g5853.t1 Caulerpa_lentillifera OG0000007
g5899.t1 Caulerpa_lentillifera OG0000007
g5917.t1 Caulerpa_lentillifera OG0000007
g5918.t1 Caulerpa_lentillifera OG0000007
g5958.t1 Caulerpa_lentillifera OG0000007
g6005.t1 Caulerpa_lentillifera OG0000007
g6275.t1 Caulerpa_lentillifera OG0000007
g6368.t1 Caulerpa_lentillifera OG0000007
g6531.t1 Caulerpa_lentillifera OG0000007
g6926.t1 Caulerpa_lentillifera OG0000007
g6927.t1 Caulerpa_lentillifera OG0000007
g6937.t1 Caulerpa_lentillifera OG0000007
g6958.t1 Caulerpa_lentillifera OG0000007
g6984.t1 Caulerpa_lentillifera OG0000007
g7159.t1 Caulerpa_lentillifera OG0000007
g721.t1 Caulerpa_lentillifera OG0000007
g7351.t1 Caulerpa_lentillifera OG0000007
g7595.t1 Caulerpa_lentillifera OG0000007
g7756.t1 Caulerpa_lentillifera OG0000007
g8202.t1 Caulerpa_lentillifera OG0000007
g8212.t1 Caulerpa_lentillifera OG0000007
g838.t1 Caulerpa_lentillifera OG0000007
g88.t1 Caulerpa_lentillifera OG0000007
g8749.t1 Caulerpa_lentillifera OG0000007
g8750.t1 Caulerpa_lentillifera OG0000007
g9003.t1 Caulerpa_lentillifera OG0000007
Cre02.g074370.t1.2 pacid=30785249 transcript=Cre02.g074370.t1.2 locus=Cre02.g074370 ID=Cre02.g074370.t1.2.v5.5 annot-version=v5.5 Chlamydomonas_reinhardtii OG0000007
Cre02.g114750.t1.2 pacid=30785389 transcript=Cre02.g114750.t1.2 locus=Cre02.g114750 ID=Cre02.g114750.t1.2.v5.5 annot-version=v5.5 Chlamydomonas_reinhardtii OG0000007
Cre02.g106650.t1.1 pacid=30785987 transcript=Cre02.g106650.t1.1 locus=Cre02.g106650 ID=Cre02.g106650.t1.1.v5.5 annot-version=v5.5 Chlamydomonas_reinhardtii OG0000007
Cre03.g153150.t1.2 pacid=30786676 transcript=Cre03.g153150.t1.2 locus=Cre03.g153150 ID=Cre03.g153150.t1.2.v5.5 annot-version=v5.5 Chlamydomonas_reinhardtii OG0000007
Cre03.g144484.t1.1 pacid=30787570 transcript=Cre03.g144484.t1.1 locus=Cre03.g144484 ID=Cre03.g144484.t1.1.v5.5 annot-version=v5.5 Chlamydomonas_reinhardtii OG0000007
Cre07.g328900.t1.2 pacid=30774791 transcript=Cre07.g328900.t1.2 locus=Cre07.g328900 ID=Cre07.g328900.t1.2.v5.5 annot-version=v5.5 Chlamydomonas_reinhardtii OG0000007
Cre01.g009500.t1.2 pacid=30788572 transcript=Cre01.g009500.t1.2 locus=Cre01.g009500 ID=Cre01.g009500.t1.2.v5.5 annot-version=v5.5 Chlamydomonas_reinhardtii OG0000007
Cre01.g003524.t1.1 pacid=30788840 transcript=Cre01.g003524.t1.1

locus=Cre01.g003524 ID=Cre01.g003524.t1.1.v5.5 annot-version=v5.5
Chlamydomonas_reinhardtii OG0000007
Cre10.g428650.t1.2 pacid=30789786 transcript=Cre10.g428650.t1.2
locus=Cre10.g428650 ID=Cre10.g428650.t1.2.v5.5 annot-version=v5.5
Chlamydomonas_reinhardtii OG0000007
Cre10.g457700.t1.2 pacid=30790742 transcript=Cre10.g457700.t1.2
locus=Cre10.g457700 ID=Cre10.g457700.t1.2.v5.5 annot-version=v5.5
Chlamydomonas_reinhardtii OG0000007
Cre10.g418900.t1.2 pacid=30790950 transcript=Cre10.g418900.t1.2
locus=Cre10.g418900 ID=Cre10.g418900.t1.2.v5.5 annot-version=v5.5
Chlamydomonas_reinhardtii OG0000007
Cre04.g211600.t1.1 pacid=30791133 transcript=Cre04.g211600.t1.1
locus=Cre04.g211600 ID=Cre04.g211600.t1.1.v5.5 annot-version=v5.5
Chlamydomonas_reinhardtii OG0000007
Cre08.g382800.t1.2 pacid=30773557 transcript=Cre08.g382800.t1.2
locus=Cre08.g382800 ID=Cre08.g382800.t1.2.v5.5 annot-version=v5.5
Chlamydomonas_reinhardtii OG0000007
Cre12.g486350.t1.1 pacid=30793556 transcript=Cre12.g486350.t1.1
locus=Cre12.g486350 ID=Cre12.g486350.t1.1.v5.5 annot-version=v5.5
Chlamydomonas_reinhardtii OG0000007
Cre16.g665364.t1.1 pacid=30778178 transcript=Cre16.g665364.t1.1
locus=Cre16.g665364 ID=Cre16.g665364.t1.1.v5.5 annot-version=v5.5
Chlamydomonas_reinhardtii OG0000007
Cre19.g750597.t1.1 pacid=30778356 transcript=Cre19.g750597.t1.1
locus=Cre19.g750597 ID=Cre19.g750597.t1.1.v5.5 annot-version=v5.5
Chlamydomonas_reinhardtii OG0000007
Cre06.g296200.t1.2 pacid=30778462 transcript=Cre06.g296200.t1.2
locus=Cre06.g296200 ID=Cre06.g296200.t1.2.v5.5 annot-version=v5.5
Chlamydomonas_reinhardtii OG0000007
Cre06.g265550.t1.2 pacid=30778926 transcript=Cre06.g265550.t1.2
locus=Cre06.g265550 ID=Cre06.g265550.t1.2.v5.5 annot-version=v5.5
Chlamydomonas_reinhardtii OG0000007
Cre06.g251050.t1.1 pacid=30780075 transcript=Cre06.g251050.t1.1
locus=Cre06.g251050 ID=Cre06.g251050.t1.1.v5.5 annot-version=v5.5
Chlamydomonas_reinhardtii OG0000007
Cre17.g707650.t1.1 pacid=30781944 transcript=Cre17.g707650.t1.1
locus=Cre17.g707650 ID=Cre17.g707650.t1.1.v5.5 annot-version=v5.5
Chlamydomonas_reinhardtii OG0000007
Cre17.g705000.t1.2 pacid=30782154 transcript=Cre17.g705000.t1.2
locus=Cre17.g705000 ID=Cre17.g705000.t1.2.v5.5 annot-version=v5.5
Chlamydomonas_reinhardtii OG0000007
Cre17.g705350.t1.1 pacid=30782435 transcript=Cre17.g705350.t1.1
locus=Cre17.g705350 ID=Cre17.g705350.t1.1.v5.5 annot-version=v5.5
Chlamydomonas_reinhardtii OG0000007
Cre13.g570250.t1.1 pacid=30784374 transcript=Cre13.g570250.t1.1
locus=Cre13.g570250 ID=Cre13.g570250.t1.1.v5.5 annot-version=v5.5
Chlamydomonas_reinhardtii OG0000007
Cre13.g564500.t1.1 pacid=30784680 transcript=Cre13.g564500.t1.1
locus=Cre13.g564500 ID=Cre13.g564500.t1.1.v5.5 annot-version=v5.5
Chlamydomonas_reinhardtii OG0000007
Cre13.g571700.t1.1 pacid=30784725 transcript=Cre13.g571700.t1.1
locus=Cre13.g571700 ID=Cre13.g571700.t1.1.v5.5 annot-version=v5.5
Chlamydomonas_reinhardtii OG0000007
Vocar.0001s0337.1.p pacid=32884400 transcript=Vocar.0001s0337.1

locus=Vocar.0001s0337 ID=Vocar.0001s0337.1.v2.1 annot-version=v2.1
Volvox_carteri OG0000007
Vocar.0011s0105.1.p pacid=32895481 transcript=Vocar.0011s0105.1
locus=Vocar.0011s0105 ID=Vocar.0011s0105.1.v2.1 annot-version=v2.1
Volvox_carteri OG0000007
Vocar.0028s0045.1.p pacid=32895727 transcript=Vocar.0028s0045.1
locus=Vocar.0028s0045 ID=Vocar.0028s0045.1.v2.1 annot-version=v2.1
Volvox_carteri OG0000007
Vocar.0079s0005.1.p pacid=32896114 transcript=Vocar.0079s0005.1
locus=Vocar.0079s0005 ID=Vocar.0079s0005.1.v2.1 annot-version=v2.1
Volvox_carteri OG0000007
Vocar.0016s0155.1.p pacid=32897902 transcript=Vocar.0016s0155.1
locus=Vocar.0016s0155 ID=Vocar.0016s0155.1.v2.1 annot-version=v2.1
Volvox_carteri OG0000007
Vocar.0016s0075.1.p pacid=32898161 transcript=Vocar.0016s0075.1
locus=Vocar.0016s0075 ID=Vocar.0016s0075.1.v2.1 annot-version=v2.1
Volvox_carteri OG0000007
Vocar.0025s0127.1.p pacid=32898443 transcript=Vocar.0025s0127.1
locus=Vocar.0025s0127 ID=Vocar.0025s0127.1.v2.1 annot-version=v2.1
Volvox_carteri OG0000007
Vocar.0001s1714.1.p pacid=32885416 transcript=Vocar.0001s1714.1
locus=Vocar.0001s1714 ID=Vocar.0001s1714.1.v2.1 annot-version=v2.1
Volvox_carteri OG0000007
Vocar.0001s0327.1.p pacid=32885814 transcript=Vocar.0001s0327.1
locus=Vocar.0001s0327 ID=Vocar.0001s0327.1.v2.1 annot-version=v2.1
Volvox_carteri OG0000007
Vocar.0020s0139.1.p pacid=32886630 transcript=Vocar.0020s0139.1
locus=Vocar.0020s0139 ID=Vocar.0020s0139.1.v2.1 annot-version=v2.1
Volvox_carteri OG0000007
Vocar.0005s0229.1.p pacid=32883627 transcript=Vocar.0005s0229.1
locus=Vocar.0005s0229 ID=Vocar.0005s0229.1.v2.1 annot-version=v2.1
Volvox_carteri OG0000007
Vocar.0038s0050.1.p pacid=32887681 transcript=Vocar.0038s0050.1
locus=Vocar.0038s0050 ID=Vocar.0038s0050.1.v2.1 annot-version=v2.1
Volvox_carteri OG0000007
Vocar.0004s0220.1.p pacid=32889487 transcript=Vocar.0004s0220.1
locus=Vocar.0004s0220 ID=Vocar.0004s0220.1.v2.1 annot-version=v2.1
Volvox_carteri OG0000007
Vocar.0004s0229.1.p pacid=32889997 transcript=Vocar.0004s0229.1
locus=Vocar.0004s0229 ID=Vocar.0004s0229.1.v2.1 annot-version=v2.1
Volvox_carteri OG0000007
Vocar.0002s0537.1.p pacid=32891181 transcript=Vocar.0002s0537.1
locus=Vocar.0002s0537 ID=Vocar.0002s0537.1.v2.1 annot-version=v2.1
Volvox_carteri OG0000007
Vocar.0017s0028.1.p pacid=32891414 transcript=Vocar.0017s0028.1
locus=Vocar.0017s0028 ID=Vocar.0017s0028.1.v2.1 annot-version=v2.1
Volvox_carteri OG0000007
Vocar.0017s0101.1.p pacid=32891559 transcript=Vocar.0017s0101.1
locus=Vocar.0017s0101 ID=Vocar.0017s0101.1.v2.1 annot-version=v2.1
Volvox_carteri OG0000007
Vocar.0022s0038.1.p pacid=32891790 transcript=Vocar.0022s0038.1
locus=Vocar.0022s0038 ID=Vocar.0022s0038.1.v2.1 annot-version=v2.1
Volvox_carteri OG0000007
Vocar.0019s0119.1.p pacid=32892620 transcript=Vocar.0019s0119.1

locus=Vocar.0019s0119 ID=Vocar.0019s0119.1.v2.1 annot-version=v2.1
Volvox_carteri OG0000007
Vocar.0006s0095.1.p pacid=32894153 transcript=Vocar.0006s0095.1
locus=Vocar.0006s0095 ID=Vocar.0006s0095.1.v2.1 annot-version=v2.1
Volvox_carteri OG0000007
jgi|ChlNC64A_1|140361|IGS.gm_3_00100 Chlorella_variabilis
OG0000007
jgi|ChlNC64A_1|140646|IGS.gm_3_00385 Chlorella_variabilis
OG0000007
jgi|ChlNC64A_1|139412|IGS.gm_24_00192 Chlorella_variabilis
OG0000007
jgi|ChlNC64A_1|20239|e_gw1.4.103.1 Chlorella_variabilis
OG0000007
jgi|ChlNC64A_1|143521|IGS.gm_6_00095 Chlorella_variabilis
OG0000007
jgi|ChlNC64A_1|143714|IGS.gm_6_00288 Chlorella_variabilis
OG0000007
jgi|ChlNC64A_1|35323|estExt_Genewise1Plus.C_90253
Chlorella_variabilis OG0000007
jgi|ChlNC64A_1|22745|e_gw1.9.373.1 Chlorella_variabilis
OG0000007
jgi|ChlNC64A_1|30247|estExt_Genewise1.C_50021
Chlorella_variabilis OG0000007
jgi|ChlNC64A_1|57611|estExt_fgenesh3_pg.C_80094
Chlorella_variabilis OG0000007
jgi|ChlNC64A_1|22362|e_gw1.8.438.1 Chlorella_variabilis
OG0000007
jgi|ChlNC64A_1|37582|estExt_Genewise1Plus.C_350059
Chlorella_variabilis OG0000007
jgi|ChlNC64A_1|141552|IGS.gm_35_00064 Chlorella_variabilis
OG0000007
jgi|ChlNC64A_1|33257|estExt_Genewise1.C_350064
Chlorella_variabilis OG0000007
jgi|ChlNC64A_1|36351|estExt_Genewise1Plus.C_160110
Chlorella_variabilis OG0000007
jgi|ChlNC64A_1|136678|IGS.gm_17_00082 Chlorella_variabilis
OG0000007
jgi|ChlNC64A_1|32147|estExt_Genewise1.C_170148
Chlorella_variabilis OG0000007
jgi|ChlNC64A_1|136852|IGS.gm_17_00256 Chlorella_variabilis
OG0000007
jgi|ChlNC64A_1|8755|gw1.15.55.1 Chlorella_variabilis
OG0000007
jgi|ChlNC64A_1|10014|gw1.40.8.1 Chlorella_variabilis
OG0000007
29600 pacid=27385789 transcript=29600
locus=estExt_Genewise1Plus.C_100192 ID=29600.2.0.227 annot-
version=v2.0 Coccomyxa_subellipsoidea OG0000007
14400 pacid=27387438 transcript=14400 locus=e_gw1.5.212.1
ID=14400.2.0.227 annot-version=v2.0 Coccomyxa_subellipsoidea
OG0000007
47731 pacid=27388535 transcript=47731
locus=estExt_fgenesh1_pg.C_90258 ID=47731.2.0.227 annot-version=v2.0
Coccomyxa_subellipsoidea OG0000007

11155 pacid=27389065 transcript=11155 locus=e_gw1.1.209.1
ID=11155.2.0.227 annot-version=v2.0 Coccomyxa_subellipsoidea
OG0000007
27599 pacid=27390428 transcript=27599
locus=estExt_Genewise1Plus.C_30441 ID=27599.2.0.227 annot-
version=v2.0 Coccomyxa_subellipsoidea OG0000007
27662 pacid=27390885 transcript=27662
locus=estExt_Genewise1Plus.C_30548 ID=27662.2.0.227 annot-
version=v2.0 Coccomyxa_subellipsoidea OG0000007
29803 pacid=27392057 transcript=29803
locus=estExt_Genewise1Plus.C_110098 ID=29803.2.0.227 annot-
version=v2.0 Coccomyxa_subellipsoidea OG0000007
16775 pacid=27385702 transcript=16775 locus=e_gw1.10.111.1
ID=16775.2.0.227 annot-version=v2.0 Coccomyxa_subellipsoidea
OG0000007
68309 pacid=27392644 transcript=68309
locus=estExt_Genemark1.C_240049 ID=68309.2.0.227 annot-version=v2.0
Coccomyxa_subellipsoidea OG0000007
20026 pacid=27392945 transcript=20026 locus=e_gw1.20.89.1
ID=20026.2.0.227 annot-version=v2.0 Coccomyxa_subellipsoidea
OG0000007
46159 pacid=27393673 transcript=46159
locus=estExt_fgenesh1_pg.C_20259 ID=46159.2.0.227 annot-version=v2.0
Coccomyxa_subellipsoidea OG0000007
28987 pacid=27386537 transcript=28987
locus=estExt_Genewise1Plus.C_70371 ID=28987.2.0.227 annot-
version=v2.0 Coccomyxa_subellipsoidea OG0000007
54361 pacid=27394994 transcript=54361
locus=estExt_fgenesh1_pm.C_150088 ID=54361.2.0.227 annot-
version=v2.0 Coccomyxa_subellipsoidea OG0000007
20203 pacid=27395183 transcript=20203 locus=e_gw1.21.89.1
ID=20203.2.0.227 annot-version=v2.0 Coccomyxa_subellipsoidea
OG0000007
2136_gw1.15.00.85.1 Ostreococcus_tauri OG0000007
13089_fgenesh1_pg.C_Chr_12.0001000108 Ostreococcus_tauri
OG0000007
3116_gw1.07.00.204.1 Ostreococcus_tauri OG0000007
3137_gw1.13.00.175.1 Ostreococcus_tauri OG0000007
14532_fgenesh1_pg.C_Chr_20.0001000047 Ostreococcus_tauri
OG0000007
17342_e_gw1.05.00.357.1 Ostreococcus_tauri OG0000007
28423_estExt_gwp_GeneWisePlus.C_Chr_06.00010200
Ostreococcus_tauri OG0000007
6377_gw1.20.00.79.1 Ostreococcus_tauri OG0000007
31789_0300010343 Ostreococcus_tauri OG0000007
34881_0900010347 Ostreococcus_tauri OG0000007
38920 pacid=27416413 transcript=38920 locus=e_gwEuk.5.316.1
ID=38920.2.0.231 annot-version=v2.0 Ostreococcus_lucimarinus
OG0000007
39356 pacid=27417106 transcript=39356 locus=e_gwEuk.12.165.1
ID=39356.2.0.231 annot-version=v2.0 Ostreococcus_lucimarinus
OG0000007
49728 pacid=27418220 transcript=49728
locus=estExt_Genewise_ext.C_Chr_60025 ID=49728.2.0.231 annot-

version=v2.0	Ostreococcus_lucimarinus	OG0000007
12650 pacid=27418398 transcript=12650
locus=fgenesh1_pm.C_Chr_6000070 ID=12650.2.0.231 annot-version=v2.0
Ostreococcus_lucimarinus	OG0000007
41712 pacid=27418878 transcript=41712 locus=e_gwEuk.13.203.1
ID=41712.2.0.231 annot-version=v2.0	Ostreococcus_lucimarinus
OG0000007
24253 pacid=27413490 transcript=24253
locus=estExt_fgenesh1_pg.C_Chr_30268 ID=24253.2.0.231 annot-
version=v2.0	Ostreococcus_lucimarinus	OG0000007
4341 pacid=27419151 transcript=4341 locus=gwEuk.19.44.1
ID=4341.2.0.231 annot-version=v2.0	Ostreococcus_lucimarinus
OG0000007
3082 pacid=27419192 transcript=3082 locus=gwEuk.19.29.1
ID=3082.2.0.231 annot-version=v2.0	Ostreococcus_lucimarinus
OG0000007
2798 pacid=27419714 transcript=2798 locus=gwEuk.14.136.1
ID=2798.2.0.231 annot-version=v2.0	Ostreococcus_lucimarinus
OG0000007
42946 pacid=27419863 transcript=42946 locus=e_gwEuk.14.119.1
ID=42946.2.0.231 annot-version=v2.0	Ostreococcus_lucimarinus
OG0000007
5956 pacid=27340615 transcript=5956 locus=MicpuC2.gw1.10.292.1
ID=5956.3.0.228 annot-version=v3.0	Micromonas_pusilla
OG0000007
58417 pacid=27341574 transcript=58417 locus=MicpuC2.EuGene.
0000050722 ID=58417.3.0.228 annot-version=v3.0
Micromonas_pusilla	OG0000007
34898 pacid=27344450 transcript=34898
locus=MicpuC2.estExt_Genewise1Plus.C_90192 ID=34898.3.0.228 annot-
version=v3.0	Micromonas_pusilla	OG0000007
165151 pacid=27344932 transcript=165191 locus=e_gw1.17.3.1
ID=165191.3.0.228 annot-version=v3.0	Micromonas_pusilla
OG0000007
126004 pacid=27345409 transcript=126044
locus=estExt_fgenesh2_kg.C_10200 ID=126044.3.0.228 annot-
version=v3.0	Micromonas_pusilla	OG0000007
154615 pacid=27346235 transcript=154655 locus=e_gw1.4.726.1
ID=154655.3.0.228 annot-version=v3.0	Micromonas_pusilla
OG0000007
33041 pacid=27346692 transcript=33041
locus=MicpuC2.estExt_Genewise1Plus.C_40546 ID=33041.3.0.228 annot-
version=v3.0	Micromonas_pusilla	OG0000007
153455 pacid=27346763 transcript=153495 locus=e_gw1.4.352.1
ID=153495.3.0.228 annot-version=v3.0	Micromonas_pusilla
OG0000007
35658 pacid=27339530 transcript=35658
locus=MicpuC2.estExt_Genewise1Plus.C_110349 ID=35658.3.0.228 annot-
version=v3.0	Micromonas_pusilla	OG0000007
97075 pacid=27396855 transcript=97075
locus=fgenesh2_pm.C_Chr_02000168 ID=97075.3.0.229 annot-version=v3.0
	Micromonas_sp._RCC299	OG0000007
113565 pacid=27398836 transcript=113565 locus=MSI_EuGene.0100010825
ID=113565.3.0.229 annot-version=v3.0	Micromonas_sp._RCC299

OG0000007
89488 pacid=27399002 transcript=89488
locus=estExt_Genewise2.C_Chr_010463 ID=89488.3.0.229 annot-version=v3.0	Micromonas_sp._RCC299	OG0000007
55132 pacid=27399017 transcript=55132 locus=EuGene.0100010359
ID=55132.3.0.229 annot-version=v3.0	Micromonas_sp._RCC299
OG0000007
68014 pacid=27399925 transcript=68014 locus=gw2.12.190.1
ID=68014.3.0.229 annot-version=v3.0	Micromonas_sp._RCC299
OG0000007
71523 pacid=27400023 transcript=71523 locus=gw2.04.495.1
ID=71523.3.0.229 annot-version=v3.0	Micromonas_sp._RCC299
OG0000007
58196 pacid=27400330 transcript=58196 locus=EuGene.0400010725
ID=58196.3.0.229 annot-version=v3.0	Micromonas_sp._RCC299
OG0000007
85139 pacid=27401295 transcript=85139 locus=e_gw2.09.239.1
ID=85139.3.0.229 annot-version=v3.0	Micromonas_sp._RCC299
OG0000007
82799 pacid=27401902 transcript=82799 locus=e_gw2.06.324.1
ID=82799.3.0.229 annot-version=v3.0	Micromonas_sp._RCC299
OG0000007
60958 pacid=27403105 transcript=60958 locus=EuGene.0800010526
ID=60958.3.0.229 annot-version=v3.0	Micromonas_sp._RCC299
OG0000007
56000 pacid=27396240 transcript=56000 locus=EuGene.0200010377
ID=56000.3.0.229 annot-version=v3.0	Micromonas_sp._RCC299
OG0000007
kfl00365_0070_v1.1	Klebsormidium_flaccidum	OG0000007
kfl00389_0060_v1.1	Klebsormidium_flaccidum	OG0000007
kfl00445_0090_v1.1	Klebsormidium_flaccidum	OG0000007
kfl00450_0060_v1.1	Klebsormidium_flaccidum	OG0000007
kfl00568_0080_v1.1	Klebsormidium_flaccidum	OG0000007
kfl00573_0060_v1.1	Klebsormidium_flaccidum	OG0000007
kfl00709_g8_v1.1 Klebsormidium_flaccidum	OG0000007
kfl00728_0060_v1.1	Klebsormidium_flaccidum	OG0000007
kfl00778_0050_v1.1	Klebsormidium_flaccidum	OG0000007
kfl00822_0030_v1.1	Klebsormidium_flaccidum	OG0000007
kfl00031_0370_v1.1	Klebsormidium_flaccidum	OG0000007
kfl00033_0150_v1.1	Klebsormidium_flaccidum	OG0000007
kfl00034_0110_v1.1	Klebsormidium_flaccidum	OG0000007
kfl00036_0390_v1.1	Klebsormidium_flaccidum	OG0000007
kfl00060_0060_v1.1	Klebsormidium_flaccidum	OG0000007
kfl00097_0270_v1.1	Klebsormidium_flaccidum	OG0000007
kfl00125_0250_v1.1	Klebsormidium_flaccidum	OG0000007
kfl00187_0160_v1.1	Klebsormidium_flaccidum	OG0000007
kfl00187_0240_v1.1	Klebsormidium_flaccidum	OG0000007
kfl00234_0080_v1.1	Klebsormidium_flaccidum	OG0000007
kfl00013_0520_v1.1	Klebsormidium_flaccidum	OG0000007
Pp3c15_5120V3.1.p pacid=32926634 transcript=Pp3c15_5120V3.1
locus=Pp3c15_5120 annot-version=v3.3	Physcomitrella_patens
OG0000007
Pp3c15_1210V3.1.p pacid=32929324 transcript=Pp3c15_1210V3.1
locus=Pp3c15_1210 annot-version=v3.3	Physcomitrella_patens

OG0000007
Pp3c15_3590V3.1.p pacid=32929782 transcript=Pp3c15_3590V3.1
locus=Pp3c15_3590 annot-version=v3.3 Physcomitrella_patens
OG0000007
Pp3c13_4100V3.1.p pacid=32930305 transcript=Pp3c13_4100V3.1
locus=Pp3c13_4100 annot-version=v3.3 Physcomitrella_patens
OG0000007
Pp3c13_2340V3.1.p pacid=32931097 transcript=Pp3c13_2340V3.1
locus=Pp3c13_2340 annot-version=v3.3 Physcomitrella_patens
OG0000007
Pp3c2_14270V3.1.p pacid=32933292 transcript=Pp3c2_14270V3.1
locus=Pp3c2_14270 annot-version=v3.3 Physcomitrella_patens
OG0000007
Pp3c2_5790V3.1.p pacid=32933841 transcript=Pp3c2_5790V3.1
locus=Pp3c2_5790 annot-version=v3.3 Physcomitrella_patens
OG0000007
Pp3c2_4720V3.1.p pacid=32934214 transcript=Pp3c2_4720V3.1
locus=Pp3c2_4720 annot-version=v3.3 Physcomitrella_patens
OG0000007
Pp3c2_13790V3.1.p pacid=32935959 transcript=Pp3c2_13790V3.1
locus=Pp3c2_13790 annot-version=v3.3 Physcomitrella_patens
OG0000007
Pp3c19_20580V3.1.p pacid=32938303 transcript=Pp3c19_20580V3.1
locus=Pp3c19_20580 annot-version=v3.3 Physcomitrella_patens
OG0000007
Pp3c3_5970V3.1.p pacid=32940746 transcript=Pp3c3_5970V3.1
locus=Pp3c3_5970 annot-version=v3.3 Physcomitrella_patens
OG0000007
Pp3c3_5570V3.1.p pacid=32941476 transcript=Pp3c3_5570V3.1
locus=Pp3c3_5570 annot-version=v3.3 Physcomitrella_patens
OG0000007
Pp3c3_5990V3.1.p pacid=32942766 transcript=Pp3c3_5990V3.1
locus=Pp3c3_5990 annot-version=v3.3 Physcomitrella_patens
OG0000007
Pp3c3_37890V3.1.p pacid=32944916 transcript=Pp3c3_37890V3.1
locus=Pp3c3_37890 annot-version=v3.3 Physcomitrella_patens
OG0000007
Pp3c20_4100V3.1.p pacid=32945505 transcript=Pp3c20_4100V3.1
locus=Pp3c20_4100 annot-version=v3.3 Physcomitrella_patens
OG0000007
Pp3c20_12010V3.1.p pacid=32946771 transcript=Pp3c20_12010V3.1
locus=Pp3c20_12010 annot-version=v3.3 Physcomitrella_patens
OG0000007
Pp3c20_4170V3.1.p pacid=32948019 transcript=Pp3c20_4170V3.1
locus=Pp3c20_4170 annot-version=v3.3 Physcomitrella_patens
OG0000007
Pp3c23_18930V3.1.p pacid=32950555 transcript=Pp3c23_18930V3.1
locus=Pp3c23_18930 annot-version=v3.3 Physcomitrella_patens
OG0000007
Pp3c23_18880V3.1.p pacid=32950805 transcript=Pp3c23_18880V3.1
locus=Pp3c23_18880 annot-version=v3.3 Physcomitrella_patens
OG0000007
Pp3c27_2930V3.1.p pacid=32951772 transcript=Pp3c27_2930V3.1
locus=Pp3c27_2930 annot-version=v3.3 Physcomitrella_patens

OG0000007
Pp3c5_7750V3.1.p pacid=32954381 transcript=Pp3c5_7750V3.1 locus=Pp3c5_7750 annot-version=v3.3 Physcomitrella_patens OG0000007
Pp3c11_20920V3.1.p pacid=32956462 transcript=Pp3c11_20920V3.1 locus=Pp3c11_20920 annot-version=v3.3 Physcomitrella_patens OG0000007
Pp3c11_5760V3.1.p pacid=32956551 transcript=Pp3c11_5760V3.1 locus=Pp3c11_5760 annot-version=v3.3 Physcomitrella_patens OG0000007
Pp3c22_18850V3.1.p pacid=32905289 transcript=Pp3c22_18850V3.1 locus=Pp3c22_18850 annot-version=v3.3 Physcomitrella_patens OG0000007
Pp3c11_5820V3.1.p pacid=32957965 transcript=Pp3c11_5820V3.1 locus=Pp3c11_5820 annot-version=v3.3 Physcomitrella_patens OG0000007
Pp3c11_25550V3.1.p pacid=32958204 transcript=Pp3c11_25550V3.1 locus=Pp3c11_25550 annot-version=v3.3 Physcomitrella_patens OG0000007
Pp3c11_4640V3.1.p pacid=32959137 transcript=Pp3c11_4640V3.1 locus=Pp3c11_4640 annot-version=v3.3 Physcomitrella_patens OG0000007
Pp3c14_7960V3.1.p pacid=32962857 transcript=Pp3c14_7960V3.1 locus=Pp3c14_7960 annot-version=v3.3 Physcomitrella_patens OG0000007
Pp3c17_2480V3.1.p pacid=32905868 transcript=Pp3c17_2480V3.1 locus=Pp3c17_2480 annot-version=v3.3 Physcomitrella_patens OG0000007
Pp3c8_690V3.1.p pacid=32965098 transcript=Pp3c8_690V3.1 locus=Pp3c8_690 annot-version=v3.3 Physcomitrella_patens OG0000007
Pp3c1_23490V3.1.p pacid=32966856 transcript=Pp3c1_23490V3.1 locus=Pp3c1_23490 annot-version=v3.3 Physcomitrella_patens OG0000007
Pp3c1_37450V3.1.p pacid=32967417 transcript=Pp3c1_37450V3.1 locus=Pp3c1_37450 annot-version=v3.3 Physcomitrella_patens OG0000007
Pp3c12_210V3.1.p pacid=32973292 transcript=Pp3c12_210V3.1 locus=Pp3c12_210 annot-version=v3.3 Physcomitrella_patens OG0000007
Pp3c12_21850V3.1.p pacid=32973633 transcript=Pp3c12_21850V3.1 locus=Pp3c12_21850 annot-version=v3.3 Physcomitrella_patens OG0000007
Pp3c12_21880V3.1.p pacid=32974473 transcript=Pp3c12_21880V3.1 locus=Pp3c12_21880 annot-version=v3.3 Physcomitrella_patens OG0000007
Pp3c12_190V3.1.p pacid=32975193 transcript=Pp3c12_190V3.1 locus=Pp3c12_190 annot-version=v3.3 Physcomitrella_patens OG0000007
Pp3c6_50V3.1.p pacid=32978421 transcript=Pp3c6_50V3.1 locus=Pp3c6_50 annot-version=v3.3 Physcomitrella_patens OG0000007
Pp3c18_12780V3.1.p pacid=32982734 transcript=Pp3c18_12780V3.1 locus=Pp3c18_12780 annot-version=v3.3 Physcomitrella_patens OG0000007

Pp3c10_7990V3.1.p pacid=32899433 transcript=Pp3c10_7990V3.1 locus=Pp3c10_7990 annot-version=v3.3 Physcomitrella_patens OG0000007
Pp3c16_15230V3.1.p pacid=32985114 transcript=Pp3c16_15230V3.1 locus=Pp3c16_15230 annot-version=v3.3 Physcomitrella_patens OG0000007
Pp3c9_5860V3.1.p pacid=32911605 transcript=Pp3c9_5860V3.1 locus=Pp3c9_5860 annot-version=v3.3 Physcomitrella_patens OG0000007
Pp3c9_4620V3.1.p pacid=32913651 transcript=Pp3c9_4620V3.1 locus=Pp3c9_4620 annot-version=v3.3 Physcomitrella_patens OG0000007
Pp3c9_21410V3.1.p pacid=32913788 transcript=Pp3c9_21410V3.1 locus=Pp3c9_21410 annot-version=v3.3 Physcomitrella_patens OG0000007
Pp3c10_7160V3.1.p pacid=32900983 transcript=Pp3c10_7160V3.1 locus=Pp3c10_7160 annot-version=v3.3 Physcomitrella_patens OG0000007
Pp3c21_15330V3.1.p pacid=32915929 transcript=Pp3c21_15330V3.1 locus=Pp3c21_15330 annot-version=v3.3 Physcomitrella_patens OG0000007
Pp3c26_4540V3.1.p pacid=32918607 transcript=Pp3c26_4540V3.1 locus=Pp3c26_4540 annot-version=v3.3 Physcomitrella_patens OG0000007
Pp3c4_25010V3.1.p pacid=32919704 transcript=Pp3c4_25010V3.1 locus=Pp3c4_25010 annot-version=v3.3 Physcomitrella_patens OG0000007
Pp3c4_7390V3.1.p pacid=32919729 transcript=Pp3c4_7390V3.1 locus=Pp3c4_7390 annot-version=v3.3 Physcomitrella_patens OG0000007
Pp3c10_8000V3.1.p pacid=32901692 transcript=Pp3c10_8000V3.1 locus=Pp3c10_8000 annot-version=v3.3 Physcomitrella_patens OG0000007
Pp3c7_25180V3.1.p pacid=32925156 transcript=Pp3c7_25180V3.1 locus=Pp3c7_25180 annot-version=v3.3 Physcomitrella_patens OG0000007
Pp3c7_22440V3.1.p pacid=32925272 transcript=Pp3c7_22440V3.1 locus=Pp3c7_22440 annot-version=v3.3 Physcomitrella_patens OG0000007
Pp3c7_22710V3.1.p pacid=32925774 transcript=Pp3c7_22710V3.1 locus=Pp3c7_22710 annot-version=v3.3 Physcomitrella_patens OG0000007
AT2G35890.1 | calcium-dependent protein kinase 25 | Chr2:15067175-15069136 REVERSE LENGTH=520 | 201606 Arabidopsis_thaliana OG0000007
AT2G38490.1 | CBL-interacting Serine/Threonine-kinase | Chr2:16113909-16115204 REVERSE LENGTH=431 | 201606 Arabidopsis_thaliana OG0000007
AT2G38910.1 | calcium-dependent protein kinase 20 | Chr2:16245214-16247483 REVERSE LENGTH=583 | 201606 Arabidopsis_thaliana OG0000007
AT2G41140.1 | CDPK-related kinase 1 | Chr2:17150492-17153378 FORWARD LENGTH=576 | 201606 Arabidopsis_thaliana OG0000007
AT2G41860.1 | calcium-dependent protein kinase 14 |

Chr2:17467646-17469786 REVERSE LENGTH=530 | 201606
Arabidopsis_thaliana OG0000007
AT2G46700.1 | CDPK-related kinase 3 | Chr2:19182968-19186430 REVERSE
LENGTH=595 | 201606 Arabidopsis_thaliana OG0000007
AT3G01090.1 | SNF1 kinase homolog 10 | Chr3:31437-33977 REVERSE
LENGTH=512 | 201606 Arabidopsis_thaliana OG0000007
AT3G04530.1 | phosphoenolpyruvate carboxylase kinase 2 |
Chr3:1221546-1222456 FORWARD LENGTH=278 | 201606
Arabidopsis_thaliana OG0000007
AT3G10660.1 | calmodulin-domain protein kinase cdpk isoform 2 |
Chr3:3331599-3334268 REVERSE LENGTH=646 | 201606
Arabidopsis_thaliana OG0000007
AT1G12580.1 | phosphoenolpyruvate carboxylase-related kinase 1 |
Chr1:4283635-4285675 FORWARD LENGTH=522 | 201606
Arabidopsis_thaliana OG0000007
AT1G12680.1 | phosphoenolpyruvate carboxylase-related kinase 2 |
Chr1:4320123-4322269 REVERSE LENGTH=470 | 201606
Arabidopsis_thaliana OG0000007
AT1G01140.1 | CBL-interacting protein kinase 9 | Chr1:64398-67512
REVERSE LENGTH=447 | 201606 Arabidopsis_thaliana
OG0000007
AT3G17510.1 | CBL-interacting protein kinase 1 |
Chr3:5989309-5992627 REVERSE LENGTH=444 | 201606
Arabidopsis_thaliana OG0000007
AT3G19100.1 | Protein kinase superfamily protein |
Chr3:6605681-6608980 FORWARD LENGTH=599 | 201606
Arabidopsis_thaliana OG0000007
AT3G20410.1 | calmodulin-domain protein kinase 9 |
Chr3:7116388-7118824 FORWARD LENGTH=541 | 201606
Arabidopsis_thaliana OG0000007
AT3G23000.1 | CBL-interacting protein kinase 7 |
Chr3:8172654-8173943 FORWARD LENGTH=429 | 201606
Arabidopsis_thaliana OG0000007
AT3G29160.1 | SNF1 kinase homolog 11 | Chr3:11128893-11131510
REVERSE LENGTH=512 | 201606 Arabidopsis_thaliana
OG0000007
AT3G45240.1 | geminivirus rep interacting kinase 1 |
Chr3:16570774-16572902 REVERSE LENGTH=396 | 201606
Arabidopsis_thaliana OG0000007
AT3G49370.1 | Calcium-dependent protein kinase (CDPK) family protein
| Chr3:18304954-18307906 REVERSE LENGTH=594 | 201606
Arabidopsis_thaliana OG0000007
AT3G50530.1 | CDPK-related kinase | Chr3:18753833-18756487 FORWARD
LENGTH=601 | 201606 Arabidopsis_thaliana OG0000007
AT3G51850.1 | calcium-dependent protein kinase 13 |
Chr3:19232667-19235526 FORWARD LENGTH=528 | 201606
Arabidopsis_thaliana OG0000007
AT3G56760.1 | Protein kinase superfamily protein |
Chr3:21020661-21023756 REVERSE LENGTH=577 | 201606
Arabidopsis_thaliana OG0000007
AT3G57530.1 | calcium-dependent protein kinase 32 |
Chr3:21296898-21299351 REVERSE LENGTH=538 | 201606
Arabidopsis_thaliana OG0000007
AT4G04695.1 | calcium-dependent protein kinase 31 |

Chr4:2381634-2383996 REVERSE LENGTH=484 | 201606
Arabidopsis_thaliana   OG0000007
AT4G04700.1 | calcium-dependent protein kinase 27 |
Chr4:2385276-2387986 REVERSE LENGTH=485 | 201606
Arabidopsis_thaliana   OG0000007
AT4G04710.1 | calcium-dependent kinase-like protein |
Chr4:2390497-2392887 REVERSE LENGTH=498 | 201606
Arabidopsis_thaliana   OG0000007
AT4G04720.1 | calcium-dependent protein kinase 21 |
Chr4:2394817-2397631 REVERSE LENGTH=531 | 201606
Arabidopsis_thaliana   OG0000007
AT4G04740.1 | calcium-dependent protein kinase 23 |
Chr4:2405406-2408493 REVERSE LENGTH=520 | 201606
Arabidopsis_thaliana   OG0000007
AT4G09570.1 | calcium-dependent protein kinase 4 |
Chr4:6049560-6052184 FORWARD LENGTH=501 | 201606
Arabidopsis_thaliana   OG0000007
AT4G14580.1 | CBL-interacting protein kinase 4 |
Chr4:8367887-8369167 REVERSE LENGTH=426 | 201606
Arabidopsis_thaliana   OG0000007
AT4G18700.1 | CBL-interacting protein kinase 12 |
Chr4:10289110-10290579 REVERSE LENGTH=489 | 201606
Arabidopsis_thaliana   OG0000007
AT4G21940.1 | calcium-dependent protein kinase 15 |
Chr4:11640847-11643387 FORWARD LENGTH=554 | 201606
Arabidopsis_thaliana   OG0000007
AT1G18890.1 | calcium-dependent protein kinase 1 |
Chr1:6523468-6525736 REVERSE LENGTH=545 | 201606
Arabidopsis_thaliana   OG0000007
AT4G23650.1 | calcium-dependent protein kinase 6 |
Chr4:12324967-12327415 REVERSE LENGTH=529 | 201606
Arabidopsis_thaliana   OG0000007
AT4G24400.1 | CBL-interacting protein kinase 8 |
Chr4:12617379-12620481 FORWARD LENGTH=445 | 201606
Arabidopsis_thaliana   OG0000007
AT4G30960.1 | SOS3-interacting protein 3 | Chr4:15067400-15068725
FORWARD LENGTH=441 | 201606   Arabidopsis_thaliana
OG0000007
AT4G35310.1 | calmodulin-domain protein kinase 5 |
Chr4:16802436-16804628 FORWARD LENGTH=556 | 201606
Arabidopsis_thaliana   OG0000007
AT4G36070.1 | calcium-dependent protein kinase 18 |
Chr4:17056907-17059595 REVERSE LENGTH=534 | 201606
Arabidopsis_thaliana   OG0000007
AT4G38230.1 | calcium-dependent protein kinase 26 |
Chr4:17928994-17930486 REVERSE LENGTH=340 | 201606
Arabidopsis_thaliana   OG0000007
AT5G01810.1 | CBL-interacting protein kinase 15 | Chr5:310460-311725
FORWARD LENGTH=421 | 201606   Arabidopsis_thaliana
OG0000007
AT5G01820.1 | serine/threonine protein kinase 1 | Chr5:313423-314751
REVERSE LENGTH=442 | 201606   Arabidopsis_thaliana
OG0000007
AT5G04870.1 | calcium dependent protein kinase 1 |

Chr5:1417015-1419877 REVERSE LENGTH=610 | 201606
Arabidopsis_thaliana OG0000007
AT5G07070.1 | CBL-interacting protein kinase 2 |
Chr5:2196743-2198113 REVERSE LENGTH=456 | 201606
Arabidopsis_thaliana OG0000007
AT5G10930.1 | CBL-interacting protein kinase 5 |
Chr5:3445569-3446906 REVERSE LENGTH=445 | 201606
Arabidopsis_thaliana OG0000007
AT5G12180.1 | calcium-dependent protein kinase 17 |
Chr5:3937136-3939323 FORWARD LENGTH=528 | 201606
Arabidopsis_thaliana OG0000007
AT5G12480.1 | calmodulin-domain protein kinase 7 |
Chr5:4047817-4050035 REVERSE LENGTH=535 | 201606
Arabidopsis_thaliana OG0000007
AT5G19360.1 | calcium-dependent protein kinase 34 |
Chr5:6521716-6523780 REVERSE LENGTH=523 | 201606
Arabidopsis_thaliana OG0000007
AT5G19450.1 | calcium-dependent protein kinase 19 |
Chr5:6558672-6561471 REVERSE LENGTH=533 | 201606
Arabidopsis_thaliana OG0000007
AT5G21326.1 | Ca2+regulated serine-threonine protein kinase family protein | Chr5:7218081-7221743 FORWARD LENGTH=439 | 201606
Arabidopsis_thaliana OG0000007
AT5G23580.1 | calmodulin-like domain protein kinase 9 |
Chr5:7950388-7952433 REVERSE LENGTH=490 | 201606
Arabidopsis_thaliana OG0000007
AT5G24430.1 | Calcium-dependent protein kinase (CDPK) family protein | Chr5:8339390-8342913 REVERSE LENGTH=594 | 201606
Arabidopsis_thaliana OG0000007
AT5G25110.1 | CBL-interacting protein kinase 25 |
Chr5:8657740-8659206 REVERSE LENGTH=488 | 201606
Arabidopsis_thaliana OG0000007
AT5G35410.1 | Protein kinase superfamily protein |
Chr5:13634933-13638062 FORWARD LENGTH=446 | 201606
Arabidopsis_thaliana OG0000007
AT5G39440.1 | SNF1-related protein kinase 1.3 |
Chr5:15781907-15784699 FORWARD LENGTH=494 | 201606
Arabidopsis_thaliana OG0000007
AT5G45810.1 | CBL-interacting protein kinase 19 |
Chr5:18584942-18586393 FORWARD LENGTH=483 | 201606
Arabidopsis_thaliana OG0000007
AT5G45820.1 | CBL-interacting protein kinase 20 |
Chr5:18587081-18588400 REVERSE LENGTH=439 | 201606
Arabidopsis_thaliana OG0000007
AT5G57630.1 | CBL-interacting protein kinase 21 |
Chr5:23341092-23343143 REVERSE LENGTH=416 | 201606
Arabidopsis_thaliana OG0000007
AT5G58380.1 | SOS3-interacting protein 1 | Chr5:23597092-23598531 REVERSE LENGTH=479 | 201606 Arabidopsis_thaliana
OG0000007
AT5G60550.1 | geminivirus rep interacting kinase 2 |
Chr5:24340135-24342356 FORWARD LENGTH=407 | 201606
Arabidopsis_thaliana OG0000007
AT5G66210.1 | calcium-dependent protein kinase 28 |

Chr5:26456681-26459434 REVERSE LENGTH=523 | 201606
Arabidopsis_thaliana   OG0000007
AT1G29230.1 | CBL-interacting protein kinase 18 |
Chr1:10214860-10216422 FORWARD LENGTH=520 | 201606
Arabidopsis_thaliana   OG0000007
AT1G30270.1 | CBL-interacting protein kinase 23 |
Chr1:10655270-10658524 FORWARD LENGTH=482 | 201606
Arabidopsis_thaliana   OG0000007
AT1G35670.1 | calcium-dependent protein kinase 2 |
Chr1:13205456-13208058 FORWARD LENGTH=495 | 201606
Arabidopsis_thaliana   OG0000007
AT1G48260.1 | CBL-interacting protein kinase 17 |
Chr1:17814226-17817226 REVERSE LENGTH=432 | 201606
Arabidopsis_thaliana   OG0000007
AT1G49580.1 | Calcium-dependent protein kinase (CDPK) family protein
| Chr1:18351611-18354384 FORWARD LENGTH=606 | 201606
Arabidopsis_thaliana   OG0000007
AT1G50700.1 | calcium-dependent protein kinase 33 |
Chr1:18782214-18784385 FORWARD LENGTH=521 | 201606
Arabidopsis_thaliana   OG0000007
AT1G61950.1 | calcium-dependent protein kinase 19 |
Chr1:22899417-22901941 FORWARD LENGTH=551 | 201606
Arabidopsis_thaliana   OG0000007
AT1G74740.1 | calcium-dependent protein kinase 30 |
Chr1:28080199-28082476 REVERSE LENGTH=541 | 201606
Arabidopsis_thaliana   OG0000007
AT1G76040.1 | calcium-dependent protein kinase 29 |
Chr1:28538906-28540448 FORWARD LENGTH=323 | 201606
Arabidopsis_thaliana   OG0000007
AT2G17290.1 | Calcium-dependent protein kinase family protein |
Chr2:7517005-7519239 FORWARD LENGTH=544 | 201606
Arabidopsis_thaliana   OG0000007
AT1G08650.1 | phosphoenolpyruvate carboxylase kinase 1 |
Chr1:2752206-2753232 FORWARD LENGTH=284 | 201606
Arabidopsis_thaliana   OG0000007
AT2G17890.1 | calcium-dependent protein kinase 16 |
Chr2:7769885-7772627 REVERSE LENGTH=571 | 201606
Arabidopsis_thaliana   OG0000007
AT2G25090.1 | CBL-interacting protein kinase 16 |
Chr2:10670542-10672610 REVERSE LENGTH=469 | 201606
Arabidopsis_thaliana   OG0000007
AT2G26980.1 | CBL-interacting protein kinase 3 |
Chr2:11515671-11518205 REVERSE LENGTH=382 | 201606
Arabidopsis_thaliana   OG0000007
AT2G30360.1 | SOS3-interacting protein 4 | Chr2:12937265-12938572
REVERSE LENGTH=435 | 201606   Arabidopsis_thaliana
OG0000007
AT2G31500.1 | calcium-dependent protein kinase 24 |
Chr2:13414016-13416324 FORWARD LENGTH=582 | 201606
Arabidopsis_thaliana   OG0000007
AT2G34180.1 | CBL-interacting protein kinase 13 |
Chr2:14430761-14432269 REVERSE LENGTH=502 | 201606
Arabidopsis_thaliana   OG0000007
Os03t0289100-01 Serine/threonine protein kinase, Carbohydrate

metabolism	Oryza_sativa	OG0000007
Os03t0319400-01 Serine/threonine protein kinase, Cold stress tolerance, Abiotic stresses	Oryza_sativa	OG0000007
Os03t0339900-01 Similar to Serine/threonine protein kinase.	Oryza_sativa	OG0000007
Os03t0634400-01 Serine/threonine protein kinase domain containing protein.	Oryza_sativa	OG0000007
Os03t0688300-01 Calcium-dependent protein kinase, Positive regulator of drought stress tolerance and spikelet fertility	Oryza_sativa	OG0000007
Os03t0711300-01 Serine/threonine protein kinase domain containing protein.	Oryza_sativa	OG0000007
Os03t0788500-01 Similar to Calcium-dependent protein kinase 2.	Oryza_sativa	OG0000007
Os03t0789000-00 Similar to Calcium-dependent protein kinase.	Oryza_sativa	OG0000007
Os03t0808600-01 Similar to Calcium-dependent protein kinase.	Oryza_sativa	OG0000007
Os01t0292200-01 Serine/threonine protein kinase domain containing protein.	Oryza_sativa	OG0000007
Os04t0560600-01 Calcium-dependent protein kinase, Salt-stress tolerance, Blast disease resistance	Oryza_sativa	OG0000007
Os04t0584600-01 Similar to Calcium dependent protein kinase.	Oryza_sativa	OG0000007
Os05t0136200-01 Serine/threonine protein kinase domain containing protein.	Oryza_sativa	OG0000007
Os05t0208100-01 Similar to CBL-interacting serine/threonine-protein kinase 15 (EC 2.7.1.37) (Serine/threonine-protein kinase ATPK10) (SOS2-like protein kinase PKS3) (SOS-interacting protein 2) (SNF1-related kinase 3.1).	Oryza_sativa	OG0000007
Os05t0332300-01 Similar to CBL-interacting protein kinase 2.	Oryza_sativa	OG0000007
Os05t0333100-00 Similar to CBL-interacting protein kinase 22.	Oryza_sativa	OG0000007
Os05t0334750-00 Similar to CBL-interacting protein kinase 22.	Oryza_sativa	OG0000007
Os05t0467000-01 Similar to Calcium-dependent protein kinase.	Oryza_sativa	OG0000007
Os05t0476350-00 Similar to CBL-interacting protein kinase 28.	Oryza_sativa	OG0000007
Os05t0489900-01 Ca/calmodulin-dependent protein kinase, Calcium/calmodulin-dependent protein kinase, Bacterial and fungal symbioses, Common symbiosis signaling (SYM) pathway	Oryza_sativa	OG0000007
Os05t0514200-01 OsPK4.	Oryza_sativa	OG0000007
Os05t0530500-01 Similar to OSK1.	Oryza_sativa	OG0000007
Os05t0585500-01 Similar to Calcium-dependent protein kinase.	Oryza_sativa	OG0000007
Os01t0536000-01 Similar to CBL-interacting serine/threonine-protein kinase 24 (EC 2.7.1.37) (SNF1-related kinase 3.11) (SALT OVERLY SENSITIVE 2 protein).	Oryza_sativa	OG0000007
Os06t0543400-01 Similar to CBL-interacting serine/threonine-protein kinase 11 (EC 2.7.1.37) (SOS2-like protein kinase PKS5) (SOS-

interacting protein 4) (SNF1- related kinase 3.22).
Oryza_sativa OG0000007
Os06t0606000-01 Similar to CBL-interacting serine/threonine-protein kinase 24 (EC 2.7.1.37) (SNF1-related kinase 3.11) (SALT OVERLY SENSITIVE 2 protein). Oryza_sativa OG0000007
Os06t0714200-01 Similar to calcium dependent protein kinase1.
Oryza_sativa OG0000007
Os07t0150700-01 Serine/threonine protein kinase, Pollination and drought stress responses, Reguration of potassium uptake by CBL1-CIPK23 complex Oryza_sativa OG0000007
Os07t0161600-01 Similar to Calmodulin-domain protein kinase CDPK isoform 2 (Calmodulin-domain protein kinase CDPK isoform 2; 13089-15758) (Calcium-dependent protein kinase isoform 2).
Oryza_sativa OG0000007
Os07t0194100-01 Similar to OSK2 (Fragment). Oryza_sativa OG0000007
Os07t0409900-01 Plasma membrane-associated calcium-dependent protein kinase, Direct phosphorylation and activation of MAPK, Negative reguration of rice immunity Oryza_sativa OG0000007
Os07t0515100-01 Calcium-dependent protein kinase, isoform 2 (EC 2.7.1.-) (CDPK 2). Oryza_sativa OG0000007
Os07t0568600-01 Similar to Calcium-dependent protein kinase.
Oryza_sativa OG0000007
Os07t0637000-01 Similar to CBL-interacting protein kinase 21.
Oryza_sativa OG0000007
Os01t0622600-01 Similar to calcium-dependent protein kinase, isoform 2. Oryza_sativa OG0000007
Os07t0678300-01 Similar to CBL-interacting protein kinase 29.
Oryza_sativa OG0000007
Os07t0678600-01 Similar to Serine/threonine protein kinase.
Oryza_sativa OG0000007
Os07t0687000-00 Serine/threonine protein kinase, Abiotic stresses
Oryza_sativa OG0000007
Os08t0441100-01 Similar to PnC401 homologue. Oryza_sativa OG0000007
Os08t0484600-01 Serine/threonine protein kinase, Carbohydrate metabolism Oryza_sativa OG0000007
Os08t0540400-01 Similar to Calcium-dependent protein kinase.
Oryza_sativa OG0000007
Os09t0418000-01 Similar to CBL-interacting protein kinase 16.
Oryza_sativa OG0000007
Os09t0418500-00 Similar to CBL-interacting protein kinase 14.
Oryza_sativa OG0000007
Os09t0466900-01 Similar to Calcium-dependent protein kinase.
Oryza_sativa OG0000007
Os09t0514200-01 Similar to Calcium-dependent protein kinase.
Oryza_sativa OG0000007
Os10t0510700-00 Similar to calcium dependent protein kinase1.
Oryza_sativa OG0000007
Os10t0539600-01 Similar to Calcium-dependent protein kinase 3.
Oryza_sativa OG0000007
Os11t0113700-01 Serine/threonine protein kinase, Abiotic stresses
Oryza_sativa OG0000007
Os11t0134300-01 Similar to Serine/threonine kinase.

Oryza_sativa OG0000007
Os11t0136600-00 Similar to Calcium-dependent protein kinase (Fragment). Oryza_sativa OG0000007
Os11t0171500-01 Similar to Calcium-dependent protein kinase SK5 (EC 2.7.1.-) (CDPK). Oryza_sativa OG0000007
Os12t0113500-01 Similar to CBL-interacting protein kinase 14. Oryza_sativa OG0000007
Os01t0759200-01 Similar to PnC401 homologue. Oryza_sativa OG0000007
Os01t0759400-02 OsPK7. Oryza_sativa OG0000007
Os12t0132200-01 Similar to CBL-interacting protein kinase 32. Oryza_sativa OG0000007
Os12t0133500-00 Similar to Calcium-dependent protein kinase (Fragment). Oryza_sativa OG0000007
Os12t0169800-00 Similar to Calcium-dependent protein kinase SK5 (EC 2.7.1.-) (CDPK). Oryza_sativa OG0000007
Os12t0486600-00 Similar to J013164K19, full insert sequence (Fragment). Oryza_sativa OG0000007
Os12t0487250-01 Arbuscular mycorrhizal specific marker 26. Oryza_sativa OG0000007
Os12t0603700-00 Similar to CBL-interacting protein kinase 4. Oryza_sativa OG0000007
Os01t0808400-01 Similar to Calcium-dependent protein kinase (Fragment). Oryza_sativa OG0000007
Os01t0824600-01 Serine/threonine protein kinase domain containing protein. Oryza_sativa OG0000007
Os01t0832300-00 Similar to Calcium-dependent protein kinase. Oryza_sativa OG0000007
Os02t0126400-01 Calcium-dependent protein kinase, Positive regulator of the salt and drought stress responses Oryza_sativa OG0000007
Os02t0161000-01 Similar to Serine/threonine protein kinase-like protein. Oryza_sativa OG0000007
Os02t0178000-01 Similar to SNF1 related protein kinase-like protein. Oryza_sativa OG0000007
Os02t0625300-00 Similar to PEPC kinase. Oryza_sativa OG0000007
Os02t0685900-00 Similar to Calcium dependent protein kinase. Oryza_sativa OG0000007
Os01t0206300-00 Similar to CBL-interacting protein kinase 18. Oryza_sativa OG0000007
Os01t0206700-01 Similar to Serine/threonine protein kinase (CBL-interacting protein kinase 20). Oryza_sativa OG0000007
Os02t0807000-01 Similar to Phosphoenolpyruvate carboxylase kinase 1. Oryza_sativa OG0000007
Os02t0832000-01 Similar to Calmodulin-like-domain protein kinase CPK2. Oryza_sativa OG0000007
Os03t0126800-00 Similar to CBL-interacting protein kinase 9. Oryza_sativa OG0000007
Os03t0128700-01 Calcium-dependent protein kinase, isoform 11 (EC 2.7.1.-) (CDPK 11). Oryza_sativa OG0000007
CMF051CT pep chromosome:ASM9120v1:6:125666:126958:-1 gene:CMF051C transcript:CMF051CT gene_biotype:protein_coding transcript_biotype:protein_coding Cyanidioschyzon_merolae

OG0000007
CML025CT pep chromosome:ASM9120v1:12:47346:52496:1 gene:CML025C transcript:CML025CT gene_biotype:protein_coding transcript_biotype:protein_coding Cyanidioschyzon_merolae OG0000007
CMT325CT pep chromosome:ASM9120v1:20:823395:825080:1 gene:CMT325C transcript:CMT325CT gene_biotype:protein_coding transcript_biotype:protein_coding Cyanidioschyzon_merolae OG0000007
CMO207CT pep chromosome:ASM9120v1:15:533007:534923:1 gene:CMO207C transcript:CMO207CT gene_biotype:protein_coding transcript_biotype:protein_coding Cyanidioschyzon_merolae OG0000007
CMF139CT pep chromosome:ASM9120v1:6:389730:391334:1 gene:CMF139C transcript:CMF139CT gene_biotype:protein_coding transcript_biotype:protein_coding Cyanidioschyzon_merolae OG0000007

evm.model.contig_2085.10	Porphyridium_purpureum	OG0000007
evm.model.contig_2102.9	Porphyridium_purpureum	OG0000007
evm.model.contig_2107.2	Porphyridium_purpureum	OG0000007
evm.model.contig_2132.3	Porphyridium_purpureum	OG0000007
evm.model.contig_2139.4	Porphyridium_purpureum	OG0000007
evm.model.contig_2200.1	Porphyridium_purpureum	OG0000007
evm.model.contig_2025.1	Porphyridium_purpureum	OG0000007
evm.model.contig_2273.29	Porphyridium_purpureum	OG0000007
evm.model.contig_2273.3	Porphyridium_purpureum	OG0000007
evm.model.contig_2279.15	Porphyridium_purpureum	OG0000007
evm.model.contig_2286.12	Porphyridium_purpureum	OG0000007
evm.model.contig_2296.12	Porphyridium_purpureum	OG0000007
evm.model.contig_2316.6	Porphyridium_purpureum	OG0000007
evm.model.contig_2320.1	Porphyridium_purpureum	OG0000007
evm.model.contig_2397.1	Porphyridium_purpureum	OG0000007
evm.model.contig_2500.4	Porphyridium_purpureum	OG0000007
evm.model.contig_3437.8	Porphyridium_purpureum	OG0000007
evm.model.contig_2033.15	Porphyridium_purpureum	OG0000007
evm.model.contig_3451.8	Porphyridium_purpureum	OG0000007
evm.model.contig_3468.9	Porphyridium_purpureum	OG0000007
evm.model.contig_3497.1	Porphyridium_purpureum	OG0000007
evm.model.contig_3531.1	Porphyridium_purpureum	OG0000007
evm.model.contig_3557.1	Porphyridium_purpureum	OG0000007
evm.model.contig_3589.1	Porphyridium_purpureum	OG0000007
evm.model.contig_3638.1	Porphyridium_purpureum	OG0000007
evm.model.contig_3678.1	Porphyridium_purpureum	OG0000007
evm.model.contig_3682.1	Porphyridium_purpureum	OG0000007
evm.model.contig_3706.1	Porphyridium_purpureum	OG0000007
evm.model.contig_3706.2	Porphyridium_purpureum	OG0000007
evm.model.contig_3806.1	Porphyridium_purpureum	OG0000007
evm.model.contig_3817.2	Porphyridium_purpureum	OG0000007
evm.model.contig_2040.7	Porphyridium_purpureum	OG0000007
evm.model.contig_429.14	Porphyridium_purpureum	OG0000007
evm.model.contig_431.4	Porphyridium_purpureum	OG0000007
evm.model.contig_4408.22	Porphyridium_purpureum	OG0000007
evm.model.contig_4408.9	Porphyridium_purpureum	OG0000007
evm.model.contig_441.14	Porphyridium_purpureum	OG0000007

evm.model.contig_4442.4 Porphyridium_purpureum   OG0000007
evm.model.contig_4468.4 Porphyridium_purpureum   OG0000007
evm.model.contig_4476.12 Porphyridium_purpureum   OG0000007
evm.model.contig_4476.17 Porphyridium_purpureum   OG0000007
evm.model.contig_2051.31 Porphyridium_purpureum   OG0000007
evm.model.contig_4487.6 Porphyridium_purpureum   OG0000007
evm.model.contig_4499.1 Porphyridium_purpureum   OG0000007
evm.model.contig_4515.1 Porphyridium_purpureum   OG0000007
evm.model.contig_4563.1 Porphyridium_purpureum   OG0000007
evm.model.contig_461.1   Porphyridium_purpureum   OG0000007
evm.model.contig_494.1   Porphyridium_purpureum   OG0000007
evm.model.contig_497.8   Porphyridium_purpureum   OG0000007
evm.model.contig_507.6   Porphyridium_purpureum   OG0000007
evm.model.contig_2056.5 Porphyridium_purpureum   OG0000007
evm.model.contig_526.5   Porphyridium_purpureum   OG0000007
evm.model.contig_527.13 Porphyridium_purpureum   OG0000007
evm.model.contig_543.2   Porphyridium_purpureum   OG0000007
evm.model.contig_559.5   Porphyridium_purpureum   OG0000007
evm.model.contig_599.2   Porphyridium_purpureum   OG0000007
evm.model.contig_662.2   Porphyridium_purpureum   OG0000007
evm.model.contig_676.1   Porphyridium_purpureum   OG0000007
evm.model.contig_731.2   Porphyridium_purpureum   OG0000007
evm.model.contig_731.1   Porphyridium_purpureum   OG0000007
evm.model.contig_774.1   Porphyridium_purpureum   OG0000007
evm.model.contig_861.1   Porphyridium_purpureum   OG0000007
evm.model.contig_2065.8 Porphyridium_purpureum   OG0000007
CDF35219 pep supercontig:ASM35022v2:HG001723:111702:113117:1 gene:CHC_T00009502001 transcript:CDF35219 gene_biotype:protein_coding transcript_biotype:protein_coding description:Serine/threonine-protein kinase   Chondrus_crispus OG0000007
CDF32209 pep supercontig:ASM35022v2:HG001460:49000:50816:1 gene:CHC_T00009300001 transcript:CDF32209 gene_biotype:protein_coding transcript_biotype:protein_coding description:SNF1-related serine threonine protein kinase catalytic subunit alpha KIN10   Chondrus_crispus OG0000007
CDF32379 pep supercontig:ASM35022v2:HG001489:17866:19566:-1 gene:CHC_T00008303001 transcript:CDF32379 gene_biotype:protein_coding transcript_biotype:protein_coding description:Serine/threonine protein kinase   Chondrus_crispus OG0000007
CDF34432 pep supercontig:ASM35022v2:HG001693:15088:16530:-1 gene:CHC_T00008532001 transcript:CDF34432 gene_biotype:protein_coding transcript_biotype:protein_coding description:Serine/threonine-protein kinase   Chondrus_crispus OG0000007
CDF39730 pep supercontig:ASM35022v2:HG002070:265057:266919:-1 gene:CHC_T00008896001 transcript:CDF39730 gene_biotype:protein_coding transcript_biotype:protein_coding description:Protein kinase   Chondrus_crispus OG0000007
CDF36388 pep supercontig:ASM35022v2:HG001775:147141:148181:-1 gene:CHC_T00009344001 transcript:CDF36388 gene_biotype:protein_coding transcript_biotype:protein_coding description:Serine/threonine protein kinase   Chondrus_crispus

OG0000007
CDF36810 pep supercontig:ASM35022v2:HG001803:73865:76216:-1
gene:CHC_T00008389001 transcript:CDF36810
gene_biotype:protein_coding transcript_biotype:protein_coding
description:Serine/threonine-protein kinase Chondrus_crispus
OG0000007
CDF34320 pep supercontig:ASM35022v2:HG001685:152480:153709:1
gene:CHC_T00008731001 transcript:CDF34320
gene_biotype:protein_coding transcript_biotype:protein_coding
description:Serine/threnine protein kinase Chondrus_crispus
OG0000007
CDF32305 pep supercontig:ASM35022v2:HG001469:223964:226348:1
gene:CHC_T00008462001 transcript:CDF32305
gene_biotype:protein_coding transcript_biotype:protein_coding
description:Serine/threonine protein kinase Chondrus_crispus
OG0000007
CDF37143 pep supercontig:ASM35022v2:HG001818:119250:121652:1
gene:CHC_T00008772001 transcript:CDF37143
gene_biotype:protein_coding transcript_biotype:protein_coding
description:Serine/threonine protein kinase Chondrus_crispus
OG0000007
CDF40084 pep supercontig:ASM35022v2:HG002125:23036:25207:1
gene:CHC_T00008682001 transcript:CDF40084
gene_biotype:protein_coding transcript_biotype:protein_coding
description:Serine/threonine protein kinase Chondrus_crispus
OG0000007
CDF32686 pep supercontig:ASM35022v2:HG001539:129327:131060:-1
gene:CHC_T00009473001 transcript:CDF32686
gene_biotype:protein_coding transcript_biotype:protein_coding
description:Serine/threonine protein kinase Chondrus_crispus
OG0000007
CDF34704 pep supercontig:ASM35022v2:HG001706:319647:320984:1
gene:CHC_T00008314001 transcript:CDF34704
gene_biotype:protein_coding transcript_biotype:protein_coding
description:Serine/threonine protein kinase Mitochondrial
Chondrus_crispus OG0000007
CDF34762 pep supercontig:ASM35022v2:HG001706:470007:472476:1
gene:CHC_T00010324001 transcript:CDF34762
gene_biotype:protein_coding transcript_biotype:protein_coding
description:Serine/threonine protein kinase Membrane
Chondrus_crispus OG0000007
CDF37978 pep supercontig:ASM35022v2:HG001890:217510:221117:1
gene:CHC_T00008782001 transcript:CDF37978
gene_biotype:protein_coding transcript_biotype:protein_coding
description:Possible calcium-dependent tyrosine protein kinase
Chondrus_crispus OG0000007
CDF34680 pep supercontig:ASM35022v2:HG001705:57452:58626:-1
gene:CHC_T00008284001 transcript:CDF34680
gene_biotype:protein_coding transcript_biotype:protein_coding
description:Serine/threonine protein kinase Chondrus_crispus
OG0000007
CDF39401 pep supercontig:ASM35022v2:HG002030:47290:49431:-1
gene:CHC_T00009203001 transcript:CDF39401
gene_biotype:protein_coding transcript_biotype:protein_coding

description:Serine/threonine protein kinase	Chondrus_crispus OG0000007
CDF37384 pep supercontig:ASM35022v2:HG001833:113876:115335:-1 gene:CHC_T00008833001 transcript:CDF37384 gene_biotype:protein_coding transcript_biotype:protein_coding description:Calcium-dependent protein kinase	Chondrus_crispus OG0000007
CDF37382 pep supercontig:ASM35022v2:HG001833:110004:111615:1 gene:CHC_T00009242001 transcript:CDF37382 gene_biotype:protein_coding transcript_biotype:protein_coding description:Protein-serine/threonine kinases Rhodoplastic Chondrus_crispus OG0000007
CDF41350 pep supercontig:ASM35022v2:HG002355:259008:260300:1 gene:CHC_T00008944001 transcript:CDF41350 gene_biotype:protein_coding transcript_biotype:protein_coding description:Serine/threonine protein kinase	Chondrus_crispus OG0000007
CDF33422 pep supercontig:ASM35022v2:HG001644:182361:183878:-1 gene:CHC_T00008435001 transcript:CDF33422 gene_biotype:protein_coding transcript_biotype:protein_coding description:Serine/threonine protein kinase	Chondrus_crispus OG0000007
CDF33575 pep supercontig:ASM35022v2:HG001650:95612:96918:-1 gene:CHC_T00009299001 transcript:CDF33575 gene_biotype:protein_coding transcript_biotype:protein_coding description:Serine/threonine protein kinase	Chondrus_crispus OG0000007
CDF34043 pep supercontig:ASM35022v2:HG001669:218502:220409:-1 gene:CHC_T00009286001 transcript:CDF34043 gene_biotype:protein_coding transcript_biotype:protein_coding description:Serine/threonine protein kinase	Chondrus_crispus OG0000007
CDF39392 pep supercontig:ASM35022v2:HG002026:44909:47108:1 gene:CHC_T00009439001 transcript:CDF39392 gene_biotype:protein_coding transcript_biotype:protein_coding description:Serine/threonine protein kinase	Chondrus_crispus OG0000007
CDF35627 pep supercontig:ASM35022v2:HG001738:27842:29389:1 gene:CHC_T00008538001 transcript:CDF35627 gene_biotype:protein_coding transcript_biotype:protein_coding description:Serine/threonine protein kinase	Chondrus_crispus OG0000007
CDF34183 pep supercontig:ASM35022v2:HG001676:22590:26850:-1 gene:CHC_T00008625001 transcript:CDF34183 gene_biotype:protein_coding transcript_biotype:protein_coding description:Serine/threonine protein kinase	Chondrus_crispus OG0000007
CDF39603 pep supercontig:ASM35022v2:HG002055:18121:20815:-1 gene:CHC_T00008503001 transcript:CDF39603 gene_biotype:protein_coding transcript_biotype:protein_coding description:Serine/threonine-protein kinase	Chondrus_crispus OG0000007
CDF39447 pep supercontig:ASM35022v2:HG002032:44852:48849:-1 gene:CHC_T00008716001 transcript:CDF39447

gene_biotype:protein_coding transcript_biotype:protein_coding
description:Serine/threonine protein kinase	Chondrus_crispus
OG0000007
CDF36873 pep supercontig:ASM35022v2:HG001807:432950:434748:-1
gene:CHC_T00008378001 transcript:CDF36873
gene_biotype:protein_coding transcript_biotype:protein_coding
description:Serine/threonine protein kinase	Chondrus_crispus
OG0000007
CDF34350 pep supercontig:ASM35022v2:HG001687:36123:38263:-1
gene:CHC_T00008624001 transcript:CDF34350
gene_biotype:protein_coding transcript_biotype:protein_coding
description:Serine/threonine protein kinase	Chondrus_crispus
OG0000007
CDF36458 pep supercontig:ASM35022v2:HG001780:356192:358232:1
gene:CHC_T00008604001 transcript:CDF36458
gene_biotype:protein_coding transcript_biotype:protein_coding
description:Serine/threonine protein kinase	Chondrus_crispus
OG0000007
CDF36461 pep supercontig:ASM35022v2:HG001780:379640:384204:1
gene:CHC_T00008700001 transcript:CDF36461
gene_biotype:protein_coding transcript_biotype:protein_coding
description:Serine/threonine protein kinase	Chondrus_crispus
OG0000007
CDF35038 pep supercontig:ASM35022v2:HG001715:116071:117284:1
gene:CHC_T00003488001 transcript:CDF35038
gene_biotype:protein_coding transcript_biotype:protein_coding
Chondrus_crispus OG0000007
CDF38320 pep supercontig:ASM35022v2:HG001916:71059:74400:1
gene:CHC_T00008959001 transcript:CDF38320
gene_biotype:protein_coding transcript_biotype:protein_coding
description:Serine/threonine protein kinase	Chondrus_crispus
OG0000007
CDF35536 pep supercontig:ASM35022v2:HG001734:39806:40525:1
gene:CHC_T00004049001 transcript:CDF35536
gene_biotype:protein_coding transcript_biotype:protein_coding
Chondrus_crispus OG0000007
CDF36602 pep supercontig:ASM35022v2:HG001791:138821:140694:1
gene:CHC_T00009517001 transcript:CDF36602
gene_biotype:protein_coding transcript_biotype:protein_coding
description:Serine/threonine protein kinase Rhodoplastic
Chondrus_crispus OG0000007
g1014.t1 Caulerpa_lentillifera	OG0000008
g1482.t1 Caulerpa_lentillifera	OG0000008
g2059.t1 Caulerpa_lentillifera	OG0000008
g2271.t1 Caulerpa_lentillifera	OG0000008
g2878.t1 Caulerpa_lentillifera	OG0000008
g3163.t1 Caulerpa_lentillifera	OG0000008
g3975.t1 Caulerpa_lentillifera	OG0000008
g4153.t1 Caulerpa_lentillifera	OG0000008
g4342.t1 Caulerpa_lentillifera	OG0000008
g4380.t1 Caulerpa_lentillifera	OG0000008
g4444.t1 Caulerpa_lentillifera	OG0000008
g4587.t1 Caulerpa_lentillifera	OG0000008
g4708.t1 Caulerpa_lentillifera	OG0000008

g4969.t1 Caulerpa_lentillifera OG0000008
g5477.t1 Caulerpa_lentillifera OG0000008
g5493.t1 Caulerpa_lentillifera OG0000008
g5870.t1 Caulerpa_lentillifera OG0000008
g6003.t1 Caulerpa_lentillifera OG0000008
g6198.t1 Caulerpa_lentillifera OG0000008
g6280.t1 Caulerpa_lentillifera OG0000008
g6475.t1 Caulerpa_lentillifera OG0000008
g6760.t1 Caulerpa_lentillifera OG0000008
g722.t1 Caulerpa_lentillifera OG0000008
g7530.t1 Caulerpa_lentillifera OG0000008
g7534.t1 Caulerpa_lentillifera OG0000008
g7537.t1 Caulerpa_lentillifera OG0000008
g7943.t1 Caulerpa_lentillifera OG0000008
g8141.t1 Caulerpa_lentillifera OG0000008
g8487.t1 Caulerpa_lentillifera OG0000008
g8765.t1 Caulerpa_lentillifera OG0000008
g8766.t1 Caulerpa_lentillifera OG0000008
g8912.t1 Caulerpa_lentillifera OG0000008
g989.t1 Caulerpa_lentillifera OG0000008
Cre03.g168800.t1.1 pacid=30787095 transcript=Cre03.g168800.t1.1 locus=Cre03.g168800 ID=Cre03.g168800.t1.1.v5.5 annot-version=v5.5 Chlamydomonas_reinhardtii OG0000008
Cre03.g200600.t1.1 pacid=30787869 transcript=Cre03.g200600.t1.1 locus=Cre03.g200600 ID=Cre03.g200600.t1.1.v5.5 annot-version=v5.5 Chlamydomonas_reinhardtii OG0000008
Cre03.g164050.t1.1 pacid=30788019 transcript=Cre03.g164050.t1.1 locus=Cre03.g164050 ID=Cre03.g164050.t1.1.v5.5 annot-version=v5.5 Chlamydomonas_reinhardtii OG0000008
Cre03.g145107.t1.1 pacid=30788181 transcript=Cre03.g145107.t1.1 locus=Cre03.g145107 ID=Cre03.g145107.t1.1.v5.5 annot-version=v5.5 Chlamydomonas_reinhardtii OG0000008
Cre03.g202000.t1.2 pacid=30788183 transcript=Cre03.g202000.t1.2 locus=Cre03.g202000 ID=Cre03.g202000.t1.2.v5.5 annot-version=v5.5 Chlamydomonas_reinhardtii OG0000008
Cre01.g055600.t1.1 pacid=30788322 transcript=Cre01.g055600.t1.1 locus=Cre01.g055600 ID=Cre01.g055600.t1.1.v5.5 annot-version=v5.5 Chlamydomonas_reinhardtii OG0000008
Cre01.g036800.t1.1 pacid=30789295 transcript=Cre01.g036800.t1.1 locus=Cre01.g036800 ID=Cre01.g036800.t1.1.v5.5 annot-version=v5.5 Chlamydomonas_reinhardtii OG0000008
Cre01.g049650.t1.1 pacid=30789661 transcript=Cre01.g049650.t1.1 locus=Cre01.g049650 ID=Cre01.g049650.t1.1.v5.5 annot-version=v5.5 Chlamydomonas_reinhardtii OG0000008
Cre10.g456350.t1.2 pacid=30789816 transcript=Cre10.g456350.t1.2 locus=Cre10.g456350 ID=Cre10.g456350.t1.2.v5.5 annot-version=v5.5 Chlamydomonas_reinhardtii OG0000008
Cre10.g427750.t1.2 pacid=30790017 transcript=Cre10.g427750.t1.2 locus=Cre10.g427750 ID=Cre10.g427750.t1.2.v5.5 annot-version=v5.5 Chlamydomonas_reinhardtii OG0000008
Cre10.g447500.t1.1 pacid=30790036 transcript=Cre10.g447500.t1.1 locus=Cre10.g447500 ID=Cre10.g447500.t1.1.v5.5 annot-version=v5.5 Chlamydomonas_reinhardtii OG0000008
Cre10.g418950.t1.1 pacid=30790133 transcript=Cre10.g418950.t1.1

locus=Cre10.g418950 ID=Cre10.g418950.t1.1.v5.5 annot-version=v5.5
Chlamydomonas_reinhardtii OG0000008
Cre12.g522550.t1.2 pacid=30791919 transcript=Cre12.g522550.t1.2
locus=Cre12.g522550 ID=Cre12.g522550.t1.2.v5.5 annot-version=v5.5
Chlamydomonas_reinhardtii OG0000008
Cre16.g656700.t1.2 pacid=30777807 transcript=Cre16.g656700.t1.2
locus=Cre16.g656700 ID=Cre16.g656700.t1.2.v5.5 annot-version=v5.5
Chlamydomonas_reinhardtii OG0000008
Cre16.g672497.t1.1 pacid=30777944 transcript=Cre16.g672497.t1.1
locus=Cre16.g672497 ID=Cre16.g672497.t1.1.v5.5 annot-version=v5.5
Chlamydomonas_reinhardtii OG0000008
Cre16.g657979.t1.2 pacid=30778039 transcript=Cre16.g657979.t1.2
locus=Cre16.g657979 ID=Cre16.g657979.t1.2.v5.5 annot-version=v5.5
Chlamydomonas_reinhardtii OG0000008
Cre19.g750197.t1.1 pacid=30778331 transcript=Cre19.g750197.t1.1
locus=Cre19.g750197 ID=Cre19.g750197.t1.1.v5.5 annot-version=v5.5
Chlamydomonas_reinhardtii OG0000008
Cre06.g278125.t1.1 pacid=30779486 transcript=Cre06.g278125.t1.1
locus=Cre06.g278125 ID=Cre06.g278125.t1.1.v5.5 annot-version=v5.5
Chlamydomonas_reinhardtii OG0000008
Cre09.g415450.t1.2 pacid=30780526 transcript=Cre09.g415450.t1.2
locus=Cre09.g415450 ID=Cre09.g415450.t1.2.v5.5 annot-version=v5.5
Chlamydomonas_reinhardtii OG0000008
Cre17.g741350.t1.2 pacid=30782011 transcript=Cre17.g741350.t1.2
locus=Cre17.g741350 ID=Cre17.g741350.t1.2.v5.5 annot-version=v5.5
Chlamydomonas_reinhardtii OG0000008
Cre17.g730950.t1.2 pacid=30782448 transcript=Cre17.g730950.t1.2
locus=Cre17.g730950 ID=Cre17.g730950.t1.2.v5.5 annot-version=v5.5
Chlamydomonas_reinhardtii OG0000008
Cre17.g735200.t1.2 pacid=30782810 transcript=Cre17.g735200.t1.2
locus=Cre17.g735200 ID=Cre17.g735200.t1.2.v5.5 annot-version=v5.5
Chlamydomonas_reinhardtii OG0000008
Cre05.g235500.t1.1 pacid=30783101 transcript=Cre05.g235500.t1.1
locus=Cre05.g235500 ID=Cre05.g235500.t1.1.v5.5 annot-version=v5.5
Chlamydomonas_reinhardtii OG0000008
Cre05.g233100.t2.1 pacid=30783379 transcript=Cre05.g233100.t2.1
locus=Cre05.g233100 ID=Cre05.g233100.t2.1.v5.5 annot-version=v5.5
Chlamydomonas_reinhardtii OG0000008
Cre13.g586500.t1.2 pacid=30784503 transcript=Cre13.g586500.t1.2
locus=Cre13.g586500 ID=Cre13.g586500.t1.2.v5.5 annot-version=v5.5
Chlamydomonas_reinhardtii OG0000008
Cre13.g602400.t1.2 pacid=30784609 transcript=Cre13.g602400.t1.2
locus=Cre13.g602400 ID=Cre13.g602400.t1.2.v5.5 annot-version=v5.5
Chlamydomonas_reinhardtii OG0000008
Cre13.g568450.t1.1 pacid=30784632 transcript=Cre13.g568450.t1.1
locus=Cre13.g568450 ID=Cre13.g568450.t1.1.v5.5 annot-version=v5.5
Chlamydomonas_reinhardtii OG0000008
Cre13.g588600.t1.1 pacid=30784693 transcript=Cre13.g588600.t1.1
locus=Cre13.g588600 ID=Cre13.g588600.t1.1.v5.5 annot-version=v5.5
Chlamydomonas_reinhardtii OG0000008
Cre02.g073750.t1.2 pacid=30784795 transcript=Cre02.g073750.t1.2
locus=Cre02.g073750 ID=Cre02.g073750.t1.2.v5.5 annot-version=v5.5
Chlamydomonas_reinhardtii OG0000008
Vocar.0012s0070.1.p pacid=32894793 transcript=Vocar.0012s0070.1

locus=Vocar.0012s0070 ID=Vocar.0012s0070.1.v2.1 annot-version=v2.1
Volvox_carteri OG0000008
Vocar.0001s0029.1.p pacid=32884399 transcript=Vocar.0001s0029.1
locus=Vocar.0001s0029 ID=Vocar.0001s0029.1.v2.1 annot-version=v2.1
Volvox_carteri OG0000008
Vocar.0011s0337.1.p pacid=32895494 transcript=Vocar.0011s0337.1
locus=Vocar.0011s0337 ID=Vocar.0011s0337.1.v2.1 annot-version=v2.1
Volvox_carteri OG0000008
Vocar.0070s0026.1.p pacid=32895816 transcript=Vocar.0070s0026.1
locus=Vocar.0070s0026 ID=Vocar.0070s0026.1.v2.1 annot-version=v2.1
Volvox_carteri OG0000008
Vocar.0056s0026.1.p pacid=32896698 transcript=Vocar.0056s0026.1
locus=Vocar.0056s0026 ID=Vocar.0056s0026.1.v2.1 annot-version=v2.1
Volvox_carteri OG0000008
Vocar.0035s0012.1.p pacid=32896912 transcript=Vocar.0035s0012.1
locus=Vocar.0035s0012 ID=Vocar.0035s0012.1.v2.1 annot-version=v2.1
Volvox_carteri OG0000008
Vocar.0001s0451.1.p pacid=32884565 transcript=Vocar.0001s0451.1
locus=Vocar.0001s0451 ID=Vocar.0001s0451.1.v2.1 annot-version=v2.1
Volvox_carteri OG0000008
Vocar.0037s0098.1.p pacid=32897524 transcript=Vocar.0037s0098.1
locus=Vocar.0037s0098 ID=Vocar.0037s0098.1.v2.1 annot-version=v2.1
Volvox_carteri OG0000008
Vocar.0016s0074.1.p pacid=32898203 transcript=Vocar.0016s0074.1
locus=Vocar.0016s0074 ID=Vocar.0016s0074.1.v2.1 annot-version=v2.1
Volvox_carteri OG0000008
Vocar.0018s0229.1.p pacid=32898579 transcript=Vocar.0018s0229.1
locus=Vocar.0018s0229 ID=Vocar.0018s0229.1.v2.1 annot-version=v2.1
Volvox_carteri OG0000008
Vocar.0047s0017.1.p pacid=32899314 transcript=Vocar.0047s0017.1
locus=Vocar.0047s0017 ID=Vocar.0047s0017.1.v2.1 annot-version=v2.1
Volvox_carteri OG0000008
Vocar.0001s0710.1.p pacid=32884970 transcript=Vocar.0001s0710.1
locus=Vocar.0001s0710 ID=Vocar.0001s0710.1.v2.1 annot-version=v2.1
Volvox_carteri OG0000008
Vocar.0001s1640.1.p pacid=32885081 transcript=Vocar.0001s1640.1
locus=Vocar.0001s1640 ID=Vocar.0001s1640.1.v2.1 annot-version=v2.1
Volvox_carteri OG0000008
Vocar.0001s0581.1.p pacid=32885279 transcript=Vocar.0001s0581.1
locus=Vocar.0001s0581 ID=Vocar.0001s0581.1.v2.1 annot-version=v2.1
Volvox_carteri OG0000008
Vocar.0001s0708.1.p pacid=32886121 transcript=Vocar.0001s0708.1
locus=Vocar.0001s0708 ID=Vocar.0001s0708.1.v2.1 annot-version=v2.1
Volvox_carteri OG0000008
Vocar.0040s0077.1.p pacid=32886214 transcript=Vocar.0040s0077.1
locus=Vocar.0040s0077 ID=Vocar.0040s0077.1.v2.1 annot-version=v2.1
Volvox_carteri OG0000008
Vocar.0033s0161.1.p pacid=32886460 transcript=Vocar.0033s0161.1
locus=Vocar.0033s0161 ID=Vocar.0033s0161.1.v2.1 annot-version=v2.1
Volvox_carteri OG0000008
Vocar.0005s0215.1.p pacid=32883553 transcript=Vocar.0005s0215.1
locus=Vocar.0005s0215 ID=Vocar.0005s0215.1.v2.1 annot-version=v2.1
Volvox_carteri OG0000008
Vocar.0029s0025.1.p pacid=32887397 transcript=Vocar.0029s0025.1

locus=Vocar.0029s0025 ID=Vocar.0029s0025.1.v2.1 annot-version=v2.1
Volvox_carteri   OG0000008
Vocar.0014s0193.1.p pacid=32888597 transcript=Vocar.0014s0193.1
locus=Vocar.0014s0193 ID=Vocar.0014s0193.1.v2.1 annot-version=v2.1
Volvox_carteri   OG0000008
Vocar.0003s0204.1.p pacid=32888854 transcript=Vocar.0003s0204.1
locus=Vocar.0003s0204 ID=Vocar.0003s0204.1.v2.1 annot-version=v2.1
Volvox_carteri   OG0000008
Vocar.0003s0405.1.p pacid=32889320 transcript=Vocar.0003s0405.1
locus=Vocar.0003s0405 ID=Vocar.0003s0405.1.v2.1 annot-version=v2.1
Volvox_carteri   OG0000008
Vocar.0003s0462.1.p pacid=32889369 transcript=Vocar.0003s0462.1
locus=Vocar.0003s0462 ID=Vocar.0003s0462.1.v2.1 annot-version=v2.1
Volvox_carteri   OG0000008
Vocar.0004s0058.1.p pacid=32889465 transcript=Vocar.0004s0058.1
locus=Vocar.0004s0058 ID=Vocar.0004s0058.1.v2.1 annot-version=v2.1
Volvox_carteri   OG0000008
Vocar.0004s0057.1.p pacid=32889860 transcript=Vocar.0004s0057.1
locus=Vocar.0004s0057 ID=Vocar.0004s0057.1.v2.1 annot-version=v2.1
Volvox_carteri   OG0000008
Vocar.0005s0163.1.p pacid=32883867 transcript=Vocar.0005s0163.1
locus=Vocar.0005s0163 ID=Vocar.0005s0163.1.v2.1 annot-version=v2.1
Volvox_carteri   OG0000008
Vocar.0002s0592.1.p pacid=32890992 transcript=Vocar.0002s0592.1
locus=Vocar.0002s0592 ID=Vocar.0002s0592.1.v2.1 annot-version=v2.1
Volvox_carteri   OG0000008
Vocar.0017s0144.1.p pacid=32891499 transcript=Vocar.0017s0144.1
locus=Vocar.0017s0144 ID=Vocar.0017s0144.1.v2.1 annot-version=v2.1
Volvox_carteri   OG0000008
Vocar.0009s0040.1.p pacid=32893478 transcript=Vocar.0009s0040.1
locus=Vocar.0009s0040 ID=Vocar.0009s0040.1.v2.1 annot-version=v2.1
Volvox_carteri   OG0000008
Vocar.0006s0438.1.p pacid=32894050 transcript=Vocar.0006s0438.1
locus=Vocar.0006s0438 ID=Vocar.0006s0438.1.v2.1 annot-version=v2.1
Volvox_carteri   OG0000008
Vocar.0006s0081.1.p pacid=32894079 transcript=Vocar.0006s0081.1
locus=Vocar.0006s0081 ID=Vocar.0006s0081.1.v2.1 annot-version=v2.1
Volvox_carteri   OG0000008
Vocar.0030s0142.1.p pacid=32894504 transcript=Vocar.0030s0142.1
locus=Vocar.0030s0142 ID=Vocar.0030s0142.1.v2.1 annot-version=v2.1
Volvox_carteri   OG0000008
jgi|ChlNC64A_1|37158|estExt_Genewise1Plus.C_260042
Chlorella_variabilis   OG0000008
jgi|ChlNC64A_1|134787|IGS.gm_12_00173   Chlorella_variabilis
OG0000008
jgi|ChlNC64A_1|133904|IGS.gm_10_00195   Chlorella_variabilis
OG0000008
jgi|ChlNC64A_1|14136|gw1.10.262.1 Chlorella_variabilis
OG0000008
jgi|ChlNC64A_1|10834|gw1.10.139.1 Chlorella_variabilis
OG0000008
jgi|ChlNC64A_1|10695|gw1.10.136.1 Chlorella_variabilis
OG0000008
jgi|ChlNC64A_1|9136|gw1.20.54.1   Chlorella_variabilis

OG0000008
jgi|ChlNC64A_1|135529|IGS.gm_14_00062	Chlorella_variabilis
OG0000008
jgi|ChlNC64A_1|36044|estExt_Genewise1Plus.C_140167
Chlorella_variabilis	OG0000008
jgi|ChlNC64A_1|10962|gw1.14.177.1 Chlorella_variabilis
OG0000008
jgi|ChlNC64A_1|21604|e_gw1.6.142.1	Chlorella_variabilis
OG0000008
jgi|ChlNC64A_1|10198|gw1.6.120.1	Chlorella_variabilis
OG0000008
jgi|ChlNC64A_1|145586|IGS.gm_9_00393	Chlorella_variabilis
OG0000008
jgi|ChlNC64A_1|26558|e_gw1.21.92.1	Chlorella_variabilis
OG0000008
jgi|ChlNC64A_1|48708|fgenesh3_pm.C_scaffold_7000003
Chlorella_variabilis	OG0000008
jgi|ChlNC64A_1|8821|gw1.7.60.1	Chlorella_variabilis
OG0000008
jgi|ChlNC64A_1|142719|IGS.gm_5_00048	Chlorella_variabilis
OG0000008
jgi|ChlNC64A_1|9849|gw1.5.202.1	Chlorella_variabilis
OG0000008
jgi|ChlNC64A_1|59403|estExt_fgenesh3_pg.C_380016
Chlorella_variabilis	OG0000008
jgi|ChlNC64A_1|137436|IGS.gm_19_00203	Chlorella_variabilis
OG0000008
jgi|ChlNC64A_1|26240|e_gw1.19.55.1	Chlorella_variabilis
OG0000008
jgi|ChlNC64A_1|136717|IGS.gm_17_00121	Chlorella_variabilis
OG0000008
jgi|ChlNC64A_1|28927|e_gw1.91.2.1 Chlorella_variabilis
OG0000008
15326 pacid=27386654 transcript=15326 locus=e_gw1.7.245.1
ID=15326.2.0.227 annot-version=v2.0	Coccomyxa_subellipsoidea
OG0000008
15452 pacid=27386988 transcript=15452 locus=e_gw1.7.246.1
ID=15452.2.0.227 annot-version=v2.0	Coccomyxa_subellipsoidea
OG0000008
14339 pacid=27387103 transcript=14339 locus=e_gw1.5.274.1
ID=14339.2.0.227 annot-version=v2.0	Coccomyxa_subellipsoidea
OG0000008
16480 pacid=27385801 transcript=16480 locus=e_gw1.10.216.1
ID=16480.2.0.227 annot-version=v2.0	Coccomyxa_subellipsoidea
OG0000008
38523 pacid=27387768 transcript=38523 locus=fgenesh1_pm.22_#_26
ID=38523.2.0.227 annot-version=v2.0	Coccomyxa_subellipsoidea
OG0000008
43593 pacid=27387936 transcript=43593 locus=fgenesh1_pg.14_#_8
ID=43593.2.0.227 annot-version=v2.0	Coccomyxa_subellipsoidea
OG0000008
38247 pacid=27388324 transcript=38247 locus=fgenesh1_pm.19_#_69
ID=38247.2.0.227 annot-version=v2.0	Coccomyxa_subellipsoidea
OG0000008

16329 pacid=27388536 transcript=16329 locus=e_gw1.9.386.1
ID=16329.2.0.227 annot-version=v2.0	Coccomyxa_subellipsoidea
OG0000008
29293 pacid=27388610 transcript=29293
locus=estExt_Genewise1Plus.C_90082 ID=29293.2.0.227 annot-
version=v2.0	Coccomyxa_subellipsoidea	OG0000008
36803 pacid=27388624 transcript=36803 locus=fgenesh1_pm.9_#_60
ID=36803.2.0.227 annot-version=v2.0	Coccomyxa_subellipsoidea
OG0000008
52195 pacid=27389341 transcript=52195
locus=estExt_fgenesh1_pm.C_10192 ID=52195.2.0.227 annot-version=v2.0
	Coccomyxa_subellipsoidea	OG0000008
13773 pacid=27390064 transcript=13773 locus=e_gw1.4.454.1
ID=13773.2.0.227 annot-version=v2.0	Coccomyxa_subellipsoidea
OG0000008
12590 pacid=27390583 transcript=12590 locus=e_gw1.3.402.1
ID=12590.2.0.227 annot-version=v2.0	Coccomyxa_subellipsoidea
OG0000008
62751 pacid=27391012 transcript=62751 locus=Genemark1.3534_g
ID=62751.2.0.227 annot-version=v2.0	Coccomyxa_subellipsoidea
OG0000008
17907 pacid=27386234 transcript=17907 locus=e_gw1.13.99.1
ID=17907.2.0.227 annot-version=v2.0	Coccomyxa_subellipsoidea
OG0000008
37341 pacid=27391824 transcript=37341 locus=fgenesh1_pm.12_#_84
ID=37341.2.0.227 annot-version=v2.0	Coccomyxa_subellipsoidea
OG0000008
19904 pacid=27393007 transcript=19904 locus=e_gw1.20.167.1
ID=19904.2.0.227 annot-version=v2.0	Coccomyxa_subellipsoidea
OG0000008
35111 pacid=27393320 transcript=35111 locus=fgenesh1_pm.2_#_121
ID=35111.2.0.227 annot-version=v2.0	Coccomyxa_subellipsoidea
OG0000008
27133 pacid=27393612 transcript=27133
locus=estExt_Genewise1Plus.C_20388 ID=27133.2.0.227 annot-
version=v2.0	Coccomyxa_subellipsoidea	OG0000008
20645 pacid=27386456 transcript=20645 locus=e_gw1.23.81.1
ID=20645.2.0.227 annot-version=v2.0	Coccomyxa_subellipsoidea
OG0000008
19236 pacid=27394451 transcript=19236 locus=e_gw1.18.25.1
ID=19236.2.0.227 annot-version=v2.0	Coccomyxa_subellipsoidea
OG0000008
146_gw1.11.00.12.1	Ostreococcus_tauri	OG0000008
3483_gw1.05.00.217.1	Ostreococcus_tauri	OG0000008
16888_e_gw1.04.00.280.1	Ostreococcus_tauri	OG0000008
18431_e_gw1.07.00.234.1	Ostreococcus_tauri	OG0000008
18833_e_gw1.08.00.93.1	Ostreococcus_tauri	OG0000008
18869_e_gw1.08.00.243.1	Ostreococcus_tauri	OG0000008
18966_e_gw1.08.00.215.1	Ostreococcus_tauri	OG0000008
19771_e_gw1.10.00.249.1	Ostreococcus_tauri	OG0000008
4415_gw1.04.00.306.1	Ostreococcus_tauri	OG0000008
20262_e_gw1.11.00.187.1	Ostreococcus_tauri	OG0000008
26160_estExt_genewise1.C_Chr_08.00010238	Ostreococcus_tauri
OG0000008

34760_0900010226 Ostreococcus_tauri OG0000008
1052_gw1.04.00.97.1 Ostreococcus_tauri OG0000008
32718 pacid=27414313 transcript=32718 locus=eugene.0700010315
ID=32718.2.0.231 annot-version=v2.0 Ostreococcus_lucimarinus
OG0000008
37187 pacid=27415588 transcript=37187 locus=e_gwEuk.4.59.1
ID=37187.2.0.231 annot-version=v2.0 Ostreococcus_lucimarinus
OG0000008
30968 pacid=27415717 transcript=30968 locus=eugene.0400010044
ID=30968.2.0.231 annot-version=v2.0 Ostreococcus_lucimarinus
OG0000008
45426 pacid=27415748 transcript=45426
locus=estExt_GenewiseEukaryote.C_Chr_40085 ID=45426.2.0.231 annot-
version=v2.0 Ostreococcus_lucimarinus OG0000008
38355 pacid=27416185 transcript=38355 locus=e_gwEuk.10.231.1
ID=38355.2.0.231 annot-version=v2.0 Ostreococcus_lucimarinus
OG0000008
4049 pacid=27416729 transcript=4049 locus=gwEuk.5.219.1
ID=4049.2.0.231 annot-version=v2.0 Ostreococcus_lucimarinus
OG0000008
42772 pacid=27419393 transcript=42772 locus=e_gwEuk.8.195.1
ID=42772.2.0.231 annot-version=v2.0 Ostreococcus_lucimarinus
OG0000008
4140 pacid=27419461 transcript=4140 locus=gwEuk.8.250.1
ID=4140.2.0.231 annot-version=v2.0 Ostreococcus_lucimarinus
OG0000008
42344 pacid=27419494 transcript=42344 locus=e_gwEuk.8.174.1
ID=42344.2.0.231 annot-version=v2.0 Ostreococcus_lucimarinus
OG0000008
4231 pacid=27419614 transcript=4231 locus=gwEuk.8.260.1
ID=4231.2.0.231 annot-version=v2.0 Ostreococcus_lucimarinus
OG0000008
2187 pacid=27413573 transcript=2187 locus=gwEuk.9.124.1
ID=2187.2.0.231 annot-version=v2.0 Ostreococcus_lucimarinus
OG0000008
44108 pacid=27420533 transcript=44108 locus=e_gwEuk.11.206.1
ID=44108.2.0.231 annot-version=v2.0 Ostreococcus_lucimarinus
OG0000008
25198 pacid=27420668 transcript=25198
locus=estExt_fgenesh1_pg.C_Chr_110031 ID=25198.2.0.231 annot-
version=v2.0 Ostreococcus_lucimarinus OG0000008
206546 pacid=27348918 transcript=206586 locus=fgenesh1_pm.12_#_18
ID=206586.3.0.228 annot-version=v3.0 Micromonas_pusilla
OG0000008
45791 pacid=27340150 transcript=45791
locus=MicpuC2.estExt_fgenesh1_pm.C_100003 ID=45791.3.0.228 annot-
version=v3.0 Micromonas_pusilla OG0000008
180632 pacid=27340280 transcript=180672
locus=estExt_Genewise1Plus.C_10_t20010 ID=180672.3.0.228 annot-
version=v3.0 Micromonas_pusilla OG0000008
160362 pacid=27340355 transcript=160402 locus=e_gw1.10.500.1
ID=160402.3.0.228 annot-version=v3.0 Micromonas_pusilla
OG0000008
11973 pacid=27340847 transcript=11973 locus=MicpuC2.gw1.7.652.1

ID=11973.3.0.228 annot-version=v3.0   Micromonas_pusilla
OG0000008
17223 pacid=27341341 transcript=17223 locus=MicpuC2.e_gw1.5.792.1
ID=17223.3.0.228 annot-version=v3.0   Micromonas_pusilla
OG0000008
156054 pacid=27341471 transcript=156094 locus=e_gw1.5.1168.1
ID=156094.3.0.228 annot-version=v3.0   Micromonas_pusilla
OG0000008
186426 pacid=27341580 transcript=186466 locus=gm1.3547_g
ID=186466.3.0.228 annot-version=v3.0   Micromonas_pusilla
OG0000008
143994 pacid=27341929 transcript=144034 locus=gw1.5.1225.1
ID=144034.3.0.228 annot-version=v3.0   Micromonas_pusilla
OG0000008
49108 pacid=27343528 transcript=49108
locus=MicpuC2.estExt_fgenesh1_pg.C_160133 ID=49108.3.0.228 annot-
version=v3.0   Micromonas_pusilla   OG0000008
70835 pacid=27343543 transcript=70835 locus=MicpuC2.SAR_EuGene.
0000160338 ID=70835.3.0.228 annot-version=v3.0
Micromonas_pusilla   OG0000008
36912 pacid=27343593 transcript=36912
locus=MicpuC2.estExt_Genewise1Plus.C_160310 ID=36912.3.0.228 annot-
version=v3.0   Micromonas_pusilla   OG0000008
188217 pacid=27338693 transcript=188257 locus=gm1.5338_g
ID=188257.3.0.228 annot-version=v3.0   Micromonas_pusilla
OG0000008
71089 pacid=27343861 transcript=71089 locus=MicpuC2.PR_1800000003
ID=71089.3.0.228 annot-version=v3.0   Micromonas_pusilla
OG0000008
165805 pacid=27343911 transcript=165845 locus=e_gw1.18.171.1
ID=165845.3.0.228 annot-version=v3.0   Micromonas_pusilla
OG0000008
182567 pacid=27344630 transcript=182607
locus=estExt_Genewise1Plus.C_170064 ID=182607.3.0.228 annot-
version=v3.0   Micromonas_pusilla   OG0000008
202597 pacid=27344992 transcript=202637 locus=fgenesh1_pm.1_#_269
ID=202637.3.0.228 annot-version=v3.0   Micromonas_pusilla
OG0000008
50310 pacid=27345241 transcript=50310 locus=MicpuC2.EuGene.
0000010534 ID=50310.3.0.228 annot-version=v3.0
Micromonas_pusilla   OG0000008
15619 pacid=27347297 transcript=15619 locus=MicpuC2.e_gw1.3.1038.1
ID=15619.3.0.228 annot-version=v3.0   Micromonas_pusilla
OG0000008
152270 pacid=27347363 transcript=152310 locus=e_gw1.3.696.1
ID=152310.3.0.228 annot-version=v3.0   Micromonas_pusilla
OG0000008
39045 pacid=27347555 transcript=39045
locus=MicpuC2.fgenesh1_pg.C_scaffold_3000552 ID=39045.3.0.228 annot-
version=v3.0   Micromonas_pusilla   OG0000008
152122 pacid=27347758 transcript=152162 locus=e_gw1.3.1715.1
ID=152162.3.0.228 annot-version=v3.0   Micromonas_pusilla
OG0000008
17639 pacid=27348415 transcript=17639 locus=MicpuC2.e_gw1.6.244.1

ID=17639.3.0.228 annot-version=v3.0   Micromonas_pusilla
OG0000008
58933 pacid=27395452 transcript=58933 locus=EuGene.0500010666
ID=58933.3.0.229 annot-version=v3.0   Micromonas_sp._RCC299
OG0000008
67858 pacid=27397405 transcript=67858 locus=gw2.10.148.1
ID=67858.3.0.229 annot-version=v3.0   Micromonas_sp._RCC299
OG0000008
58849 pacid=27395290 transcript=58849 locus=EuGene.0500010582
ID=58849.3.0.229 annot-version=v3.0   Micromonas_sp._RCC299
OG0000008
81659 pacid=27395521 transcript=81659 locus=e_gw2.05.224.1
ID=81659.3.0.229 annot-version=v3.0   Micromonas_sp._RCC299
OG0000008
67788 pacid=27398203 transcript=67788 locus=gw2.03.202.1
ID=67788.3.0.229 annot-version=v3.0   Micromonas_sp._RCC299
OG0000008
113650 pacid=27395301 transcript=113650 locus=AZW_EuGene0500010316
ID=113650.3.0.229 annot-version=v3.0   Micromonas_sp._RCC299
OG0000008
77016 pacid=27399412 transcript=77016 locus=gw2.01.1115.1
ID=77016.3.0.229 annot-version=v3.0   Micromonas_sp._RCC299
OG0000008
62851 pacid=27399599 transcript=62851 locus=EuGene.1200010023
ID=62851.3.0.229 annot-version=v3.0   Micromonas_sp._RCC299
OG0000008
63076 pacid=27399654 transcript=63076 locus=EuGene.1200010248
ID=63076.3.0.229 annot-version=v3.0   Micromonas_sp._RCC299
OG0000008
87350 pacid=27399780 transcript=87350 locus=e_gw2.12.68.1
ID=87350.3.0.229 annot-version=v3.0   Micromonas_sp._RCC299
OG0000008
80653 pacid=27400062 transcript=80653 locus=e_gw2.04.813.1
ID=80653.3.0.229 annot-version=v3.0   Micromonas_sp._RCC299
OG0000008
81000 pacid=27400265 transcript=81000 locus=e_gw2.04.341.1
ID=81000.3.0.229 annot-version=v3.0   Micromonas_sp._RCC299
OG0000008
81350 pacid=27400776 transcript=81350 locus=e_gw2.04.185.1
ID=81350.3.0.229 annot-version=v3.0   Micromonas_sp._RCC299
OG0000008
84915 pacid=27401188 transcript=84915 locus=e_gw2.09.303.1
ID=84915.3.0.229 annot-version=v3.0   Micromonas_sp._RCC299
OG0000008
68453 pacid=27401382 transcript=68453 locus=gw2.09.193.1
ID=68453.3.0.229 annot-version=v3.0   Micromonas_sp._RCC299
OG0000008
85360 pacid=27401670 transcript=85360 locus=e_gw2.09.274.1
ID=85360.3.0.229 annot-version=v3.0   Micromonas_sp._RCC299
OG0000008
97339 pacid=27395901 transcript=97339
locus=fgenesh2_pm.C_Chr_05000015 ID=97339.3.0.229 annot-version=v3.0
        Micromonas_sp._RCC299   OG0000008
60763 pacid=27403123 transcript=60763 locus=EuGene.0800010331

ID=60763.3.0.229 annot-version=v3.0	Micromonas_sp._RCC299
OG0000008
84595 pacid=27403363 transcript=84595 locus=e_gw2.08.74.1
ID=84595.3.0.229 annot-version=v3.0	Micromonas_sp._RCC299
OG0000008
69676 pacid=27403902 transcript=69676 locus=gw2.14.210.1
ID=69676.3.0.229 annot-version=v3.0	Micromonas_sp._RCC299
OG0000008
58932 pacid=27395353 transcript=58932 locus=EuGene.0500010665
ID=58932.3.0.229 annot-version=v3.0	Micromonas_sp._RCC299
OG0000008
51001 pacid=27404094 transcript=51001
locus=est_orfs.Chr_07_872_3200126:1 ID=51001.3.0.229 annot-
version=v3.0	Micromonas_sp._RCC299	OG0000008
68300 pacid=27404160 transcript=68300 locus=gw2.07.196.1
ID=68300.3.0.229 annot-version=v3.0	Micromonas_sp._RCC299
OG0000008
60323 pacid=27404372 transcript=60323 locus=EuGene.0700010608
ID=60323.3.0.229 annot-version=v3.0	Micromonas_sp._RCC299
OG0000008
83857 pacid=27404401 transcript=83857 locus=e_gw2.07.63.1
ID=83857.3.0.229 annot-version=v3.0	Micromonas_sp._RCC299
OG0000008
98046 pacid=27404791 transcript=98046
locus=fgenesh2_pm.C_Chr_11000029 ID=98046.3.0.229 annot-version=v3.0
	Micromonas_sp._RCC299	OG0000008
kfl00334_0110_v1.1	Klebsormidium_flaccidum	OG0000008
kfl00356_0150_v1.1	Klebsormidium_flaccidum	OG0000008
kfl00364_0070_v1.1	Klebsormidium_flaccidum	OG0000008
kfl00364_0100_v1.1	Klebsormidium_flaccidum	OG0000008
kfl00407_0100_v1.1	Klebsormidium_flaccidum	OG0000008
kfl00447_0060_v1.1	Klebsormidium_flaccidum	OG0000008
kfl00017_0280_v1.1	Klebsormidium_flaccidum	OG0000008
kfl00018_0290_v1.1	Klebsormidium_flaccidum	OG0000008
kfl00519_0020_v1.1	Klebsormidium_flaccidum	OG0000008
kfl00520_0080_v1.1	Klebsormidium_flaccidum	OG0000008
kfl00545_0010_v1.1	Klebsormidium_flaccidum	OG0000008
kfl00019_0100_v1.1	Klebsormidium_flaccidum	OG0000008
kfl00630_0060_v1.1	Klebsormidium_flaccidum	OG0000008
kfl00661_0020_v1.1	Klebsormidium_flaccidum	OG0000008
kfl00691_0055_v1.1	Klebsormidium_flaccidum	OG0000008
kfl00714_0060_v1.1	Klebsormidium_flaccidum	OG0000008
kfl00785_0060_v1.1	Klebsormidium_flaccidum	OG0000008
kfl01264_0010_v1.1	Klebsormidium_flaccidum	OG0000008
kfl00041_0070_v1.1	Klebsormidium_flaccidum	OG0000008
kfl00004_0490_v1.1	Klebsormidium_flaccidum	OG0000008
kfl00089_0140_v1.1	Klebsormidium_flaccidum	OG0000008
kfl00124_0330_v1.1	Klebsormidium_flaccidum	OG0000008
kfl00009_0160_v1.1	Klebsormidium_flaccidum	OG0000008
kfl00154_0070_v1.1	Klebsormidium_flaccidum	OG0000008
kfl00163_0120_v1.1	Klebsormidium_flaccidum	OG0000008
kfl00179_0290_v1.1	Klebsormidium_flaccidum	OG0000008
kfl00199_0060_v1.1	Klebsormidium_flaccidum	OG0000008
kfl00229_0050_v1.1	Klebsormidium_flaccidum	OG0000008

kfl00274_0150_v1.1   Klebsormidium_flaccidum OG0000008
kfl00317_0030_v1.1   Klebsormidium_flaccidum OG0000008
Pp3c7_11530V3.1.p pacid=32926173 transcript=Pp3c7_11530V3.1
locus=Pp3c7_11530 annot-version=v3.3   Physcomitrella_patens
OG0000008
Pp3c15_1832V3.1.p pacid=32927285 transcript=Pp3c15_1832V3.1
locus=Pp3c15_1832 annot-version=v3.3   Physcomitrella_patens
OG0000008
Pp3c15_1830V3.1.p pacid=32927336 transcript=Pp3c15_1830V3.1
locus=Pp3c15_1830 annot-version=v3.3   Physcomitrella_patens
OG0000008
Pp3c15_3730V3.1.p pacid=32928474 transcript=Pp3c15_3730V3.1
locus=Pp3c15_3730 annot-version=v3.3   Physcomitrella_patens
OG0000008
Pp3c13_14170V3.1.p pacid=32931089 transcript=Pp3c13_14170V3.1
locus=Pp3c13_14170 annot-version=v3.3   Physcomitrella_patens
OG0000008
Pp3c13_15600V3.1.p pacid=32932172 transcript=Pp3c13_15600V3.1
locus=Pp3c13_15600 annot-version=v3.3   Physcomitrella_patens
OG0000008
Pp3c2_28160V3.1.p pacid=32932652 transcript=Pp3c2_28160V3.1
locus=Pp3c2_28160 annot-version=v3.3   Physcomitrella_patens
OG0000008
Pp3c2_7980V3.1.p pacid=32934498 transcript=Pp3c2_7980V3.1
locus=Pp3c2_7980 annot-version=v3.3   Physcomitrella_patens
OG0000008
Pp3c2_29920V3.1.p pacid=32934666 transcript=Pp3c2_29920V3.1
locus=Pp3c2_29920 annot-version=v3.3   Physcomitrella_patens
OG0000008
Pp3c2_28162V3.1.p pacid=32934732 transcript=Pp3c2_28162V3.1
locus=Pp3c2_28162 annot-version=v3.3   Physcomitrella_patens
OG0000008
Pp3c2_9150V3.1.p pacid=32935098 transcript=Pp3c2_9150V3.1
locus=Pp3c2_9150 annot-version=v3.3   Physcomitrella_patens
OG0000008
Pp3c2_3070V3.1.p pacid=32936641 transcript=Pp3c2_3070V3.1
locus=Pp3c2_3070 annot-version=v3.3   Physcomitrella_patens
OG0000008
Pp3c2_19380V3.1.p pacid=32937144 transcript=Pp3c2_19380V3.1
locus=Pp3c2_19380 annot-version=v3.3   Physcomitrella_patens
OG0000008
Pp3c3_9850V3.1.p pacid=32940174 transcript=Pp3c3_9850V3.1
locus=Pp3c3_9850 annot-version=v3.3   Physcomitrella_patens
OG0000008
Pp3c3_10820V3.1.p pacid=32940198 transcript=Pp3c3_10820V3.1
locus=Pp3c3_10820 annot-version=v3.3   Physcomitrella_patens
OG0000008
Pp3c3_26490V3.1.p pacid=32940284 transcript=Pp3c3_26490V3.1
locus=Pp3c3_26490 annot-version=v3.3   Physcomitrella_patens
OG0000008
Pp3c3_17710V3.1.p pacid=32940866 transcript=Pp3c3_17710V3.1
locus=Pp3c3_17710 annot-version=v3.3   Physcomitrella_patens
OG0000008
Pp3c3_10920V3.1.p pacid=32941342 transcript=Pp3c3_10920V3.1

locus=Pp3c3_10920 annot-version=v3.3	Physcomitrella_patens
OG0000008
Pp3c3_21330V3.1.p pacid=32942953 transcript=Pp3c3_21330V3.1
locus=Pp3c3_21330 annot-version=v3.3	Physcomitrella_patens
OG0000008
Pp3c3_10921V3.1.p pacid=32944951 transcript=Pp3c3_10921V3.1
locus=Pp3c3_10921 annot-version=v3.3	Physcomitrella_patens
OG0000008
Pp3c20_5990V3.1.p pacid=32947249 transcript=Pp3c20_5990V3.1
locus=Pp3c20_5990 annot-version=v3.3	Physcomitrella_patens
OG0000008
Pp3c27_5070V3.1.p pacid=32951374 transcript=Pp3c27_5070V3.1
locus=Pp3c27_5070 annot-version=v3.3	Physcomitrella_patens
OG0000008
Pp3c27_4630V3.1.p pacid=32951421 transcript=Pp3c27_4630V3.1
locus=Pp3c27_4630 annot-version=v3.3	Physcomitrella_patens
OG0000008
Pp3c27_850V3.1.p pacid=32952292 transcript=Pp3c27_850V3.1
locus=Pp3c27_850 annot-version=v3.3	Physcomitrella_patens
OG0000008
Pp3c22_20430V3.1.p pacid=32904895 transcript=Pp3c22_20430V3.1
locus=Pp3c22_20430 annot-version=v3.3	Physcomitrella_patens
OG0000008
Pp3c5_5310V3.1.p pacid=32953822 transcript=Pp3c5_5310V3.1
locus=Pp3c5_5310 annot-version=v3.3	Physcomitrella_patens
OG0000008
Pp3c5_1180V3.1.p pacid=32953880 transcript=Pp3c5_1180V3.1
locus=Pp3c5_1180 annot-version=v3.3	Physcomitrella_patens
OG0000008
Pp3c5_1290V3.1.p pacid=32954036 transcript=Pp3c5_1290V3.1
locus=Pp3c5_1290 annot-version=v3.3	Physcomitrella_patens
OG0000008
Pp3c5_4080V3.1.p pacid=32954950 transcript=Pp3c5_4080V3.1
locus=Pp3c5_4080 annot-version=v3.3	Physcomitrella_patens
OG0000008
Pp3c11_17850V3.1.p pacid=32956305 transcript=Pp3c11_17850V3.1
locus=Pp3c11_17850 annot-version=v3.3	Physcomitrella_patens
OG0000008
Pp3c11_18240V3.1.p pacid=32956359 transcript=Pp3c11_18240V3.1
locus=Pp3c11_18240 annot-version=v3.3	Physcomitrella_patens
OG0000008
Pp3c11_6950V3.1.p pacid=32957188 transcript=Pp3c11_6950V3.1
locus=Pp3c11_6950 annot-version=v3.3	Physcomitrella_patens
OG0000008
Pp3c11_8020V3.1.p pacid=32959030 transcript=Pp3c11_8020V3.1
locus=Pp3c11_8020 annot-version=v3.3	Physcomitrella_patens
OG0000008
Pp3c11_5890V3.1.p pacid=32959566 transcript=Pp3c11_5890V3.1
locus=Pp3c11_5890 annot-version=v3.3	Physcomitrella_patens
OG0000008
Pp3c14_1560V3.1.p pacid=32959803 transcript=Pp3c14_1560V3.1
locus=Pp3c14_1560 annot-version=v3.3	Physcomitrella_patens
OG0000008
Pp3c14_12630V3.1.p pacid=32960095 transcript=Pp3c14_12630V3.1

locus=Pp3c14_12630 annot-version=v3.3   Physcomitrella_patens
OG0000008
Pp3c14_15480V3.1.p pacid=32962149 transcript=Pp3c14_15480V3.1
locus=Pp3c14_15480 annot-version=v3.3   Physcomitrella_patens
OG0000008
Pp3c14_19550V3.1.p pacid=32962927 transcript=Pp3c14_19550V3.1
locus=Pp3c14_19550 annot-version=v3.3   Physcomitrella_patens
OG0000008
Pp3c1_7630V3.1.p pacid=32966579 transcript=Pp3c1_7630V3.1
locus=Pp3c1_7630 annot-version=v3.3   Physcomitrella_patens
OG0000008
Pp3c1_18150V3.1.p pacid=32969823 transcript=Pp3c1_18150V3.1
locus=Pp3c1_18150 annot-version=v3.3   Physcomitrella_patens
OG0000008
Pp3c1_6090V3.1.p pacid=32970905 transcript=Pp3c1_6090V3.1
locus=Pp3c1_6090 annot-version=v3.3   Physcomitrella_patens
OG0000008
Pp3c17_21780V3.1.p pacid=32906732 transcript=Pp3c17_21780V3.1
locus=Pp3c17_21780 annot-version=v3.3   Physcomitrella_patens
OG0000008
Pp3c1_10680V3.1.p pacid=32971866 transcript=Pp3c1_10680V3.1
locus=Pp3c1_10680 annot-version=v3.3   Physcomitrella_patens
OG0000008
Pp3c1_32120V3.1.p pacid=32972082 transcript=Pp3c1_32120V3.1
locus=Pp3c1_32120 annot-version=v3.3   Physcomitrella_patens
OG0000008
Pp3c17_15260V3.1.p pacid=32906876 transcript=Pp3c17_15260V3.1
locus=Pp3c17_15260 annot-version=v3.3   Physcomitrella_patens
OG0000008
Pp3c12_14710V3.1.p pacid=32975191 transcript=Pp3c12_14710V3.1
locus=Pp3c12_14710 annot-version=v3.3   Physcomitrella_patens
OG0000008
Pp3c6_19520V3.1.p pacid=32975720 transcript=Pp3c6_19520V3.1
locus=Pp3c6_19520 annot-version=v3.3   Physcomitrella_patens
OG0000008
Pp3c17_5100V3.1.p pacid=32907198 transcript=Pp3c17_5100V3.1
locus=Pp3c17_5100 annot-version=v3.3   Physcomitrella_patens
OG0000008
Pp3c6_21870V3.1.p pacid=32977393 transcript=Pp3c6_21870V3.1
locus=Pp3c6_21870 annot-version=v3.3   Physcomitrella_patens
OG0000008
Pp3c16_4640V3.1.p pacid=32983830 transcript=Pp3c16_4640V3.1
locus=Pp3c16_4640 annot-version=v3.3   Physcomitrella_patens
OG0000008
Pp3c17_10190V3.1.p pacid=32908001 transcript=Pp3c17_10190V3.1
locus=Pp3c17_10190 annot-version=v3.3   Physcomitrella_patens
OG0000008
Pp3c16_2830V3.1.p pacid=32985699 transcript=Pp3c16_2830V3.1
locus=Pp3c16_2830 annot-version=v3.3   Physcomitrella_patens
OG0000008
Pp3c24_13720V3.1.p pacid=32908574 transcript=Pp3c24_13720V3.1
locus=Pp3c24_13720 annot-version=v3.3   Physcomitrella_patens
OG0000008
Pp3c24_19380V3.1.p pacid=32909513 transcript=Pp3c24_19380V3.1

locus=Pp3c24_19380 annot-version=v3.3 Physcomitrella_patens
OG0000008
Pp3c9_4530V3.1.p pacid=32911682 transcript=Pp3c9_4530V3.1
locus=Pp3c9_4530 annot-version=v3.3 Physcomitrella_patens
OG0000008
Pp3c9_1920V3.1.p pacid=32911865 transcript=Pp3c9_1920V3.1
locus=Pp3c9_1920 annot-version=v3.3 Physcomitrella_patens
OG0000008
Pp3c9_810V3.1.p pacid=32911882 transcript=Pp3c9_810V3.1
locus=Pp3c9_810 annot-version=v3.3 Physcomitrella_patens
OG0000008
Pp3c9_8640V3.1.p pacid=32912705 transcript=Pp3c9_8640V3.1
locus=Pp3c9_8640 annot-version=v3.3 Physcomitrella_patens
OG0000008
Pp3c10_8460V3.1.p pacid=32900862 transcript=Pp3c10_8460V3.1
locus=Pp3c10_8460 annot-version=v3.3 Physcomitrella_patens
OG0000008
Pp3c21_9290V3.1.p pacid=32916822 transcript=Pp3c21_9290V3.1
locus=Pp3c21_9290 annot-version=v3.3 Physcomitrella_patens
OG0000008
Pp3c26_10570V3.1.p pacid=32917700 transcript=Pp3c26_10570V3.1
locus=Pp3c26_10570 annot-version=v3.3 Physcomitrella_patens
OG0000008
Pp3c4_16190V3.1.p pacid=32919736 transcript=Pp3c4_16190V3.1
locus=Pp3c4_16190 annot-version=v3.3 Physcomitrella_patens
OG0000008
Pp3c4_2270V3.1.p pacid=32921416 transcript=Pp3c4_2270V3.1
locus=Pp3c4_2270 annot-version=v3.3 Physcomitrella_patens
OG0000008
Pp3c4_17450V3.1.p pacid=32921605 transcript=Pp3c4_17450V3.1
locus=Pp3c4_17450 annot-version=v3.3 Physcomitrella_patens
OG0000008
Pp3c4_18000V3.1.p pacid=32921650 transcript=Pp3c4_18000V3.1
locus=Pp3c4_18000 annot-version=v3.3 Physcomitrella_patens
OG0000008
Pp3c4_8720V3.1.p pacid=32921651 transcript=Pp3c4_8720V3.1
locus=Pp3c4_8720 annot-version=v3.3 Physcomitrella_patens
OG0000008
Pp3c4_24010V3.1.p pacid=32921679 transcript=Pp3c4_24010V3.1
locus=Pp3c4_24010 annot-version=v3.3 Physcomitrella_patens
OG0000008
Pp3c7_20810V3.1.p pacid=32922947 transcript=Pp3c7_20810V3.1
locus=Pp3c7_20810 annot-version=v3.3 Physcomitrella_patens
OG0000008
Pp3c7_19581V3.1.p pacid=32923432 transcript=Pp3c7_19581V3.1
locus=Pp3c7_19581 annot-version=v3.3 Physcomitrella_patens
OG0000008
Pp3c7_8690V3.1.p pacid=32923457 transcript=Pp3c7_8690V3.1
locus=Pp3c7_8690 annot-version=v3.3 Physcomitrella_patens
OG0000008
Pp3c7_19554V3.1.p pacid=32923914 transcript=Pp3c7_19554V3.1
locus=Pp3c7_19554 annot-version=v3.3 Physcomitrella_patens
OG0000008
Pp3c7_19570V3.1.p pacid=32923997 transcript=Pp3c7_19570V3.1

locus=Pp3c7_19570 annot-version=v3.3   Physcomitrella_patens
OG0000008
Pp3c7_19550V3.1.p pacid=32925371 transcript=Pp3c7_19550V3.1
locus=Pp3c7_19550 annot-version=v3.3   Physcomitrella_patens
OG0000008
AT2G36200.1 | P-loop containing nucleoside triphosphate hydrolases superfamily protein | Chr2:15180078-15185189 REVERSE LENGTH=1009 | 201606 Arabidopsis_thaliana   OG0000008
AT2G37420.1 | ATP binding microtubule motor family protein | Chr2:15700550-15705165 FORWARD LENGTH=1039 | 201606
Arabidopsis_thaliana   OG0000008
AT2G47500.1 | P-loop nucleoside triphosphate hydrolases superfamily protein with CH (Calponin Homology) domain-containing protein | Chr2:19493247-19497882 FORWARD LENGTH=983 | 201606
Arabidopsis_thaliana   OG0000008
AT1G12430.1 | armadillo repeat kinesin 3 | Chr1:4234122-4238552 REVERSE LENGTH=919 | 201606   Arabidopsis_thaliana
OG0000008
AT3G10180.1 | P-loop containing nucleoside triphosphate hydrolases superfamily protein | Chr3:3146393-3154644 REVERSE LENGTH=1273 | 201606 Arabidopsis_thaliana   OG0000008
AT3G10310.1 | P-loop nucleoside triphosphate hydrolases superfamily protein with CH (Calponin Homology) domain-containing protein | Chr3:3190208-3195005 FORWARD LENGTH=959 | 201606
Arabidopsis_thaliana   OG0000008
AT3G12020.1 | P-loop containing nucleoside triphosphate hydrolases superfamily protein | Chr3:3827016-3834146 FORWARD LENGTH=965 | 201606 Arabidopsis_thaliana   OG0000008
AT3G17360.1 | phragmoplast orienting kinesin 1 | Chr3:5936108-5946205 FORWARD LENGTH=2066 | 201606
Arabidopsis_thaliana   OG0000008
AT3G19050.1 | phragmoplast orienting kinesin 2 | Chr3:6578047-6590106 FORWARD LENGTH=2771 | 201606
Arabidopsis_thaliana   OG0000008
AT3G20150.1 | Kinesin motor family protein | Chr3:7031412-7036499 FORWARD LENGTH=1114 | 201606   Arabidopsis_thaliana
OG0000008
AT3G23670.1 | phragmoplast-associated kinesin-related protein | Chr3:8519290-8525055 FORWARD LENGTH=1313 | 201606
Arabidopsis_thaliana   OG0000008
AT3G43210.1 | ATP binding microtubule motor family protein | Chr3:15191429-15196021 FORWARD LENGTH=938 | 201606
Arabidopsis_thaliana   OG0000008
AT3G44050.1 | P-loop containing nucleoside triphosphate hydrolases superfamily protein | Chr3:15818738-15824792 FORWARD LENGTH=1263 | 201606 Arabidopsis_thaliana   OG0000008
AT3G44730.1 | kinesin-like protein 1 | Chr3:16285888-16290852 FORWARD LENGTH=1087 | 201606   Arabidopsis_thaliana
OG0000008
AT3G45850.1 | P-loop containing nucleoside triphosphate hydrolases superfamily protein | Chr3:16855814-16860971 REVERSE LENGTH=1065 | 201606 Arabidopsis_thaliana   OG0000008
AT3G49650.1 | P-loop containing nucleoside triphosphate hydrolases superfamily protein | Chr3:18405260-18409402 REVERSE LENGTH=813 |

201606	Arabidopsis_thaliana	OG0000008
AT3G50240.1 | ATP binding microtubule motor family protein | Chr3:18623380-18628784 REVERSE LENGTH=1051 | 201606	Arabidopsis_thaliana	OG0000008
AT3G51150.1 | ATP binding microtubule motor family protein | Chr3:19002006-19006509 FORWARD LENGTH=1052 | 201606	Arabidopsis_thaliana	OG0000008
AT3G54870.1 | Armadillo/beta-catenin repeat family protein / kinesin motor family protein | Chr3:20330806-20335823 FORWARD LENGTH=941 | 201606	Arabidopsis_thaliana	OG0000008
AT3G63480.1 | ATP binding microtubule motor family protein | Chr3:23441065-23443809 REVERSE LENGTH=469 | 201606	Arabidopsis_thaliana	OG0000008
AT4G05190.1 | kinesin 5 | Chr4:2675338-2679482 FORWARD LENGTH=790 | 201606	Arabidopsis_thaliana	OG0000008
AT4G14150.1 | phragmoplast-associated kinesin-related protein 1 | Chr4:8158645-8165008 REVERSE LENGTH=1292 | 201606	Arabidopsis_thaliana	OG0000008
AT1G18370.1 | ATP binding microtubule motor family protein | Chr1:6319732-6323820 REVERSE LENGTH=974 | 201606	Arabidopsis_thaliana	OG0000008
AT1G18410.1 | P-loop containing nucleoside triphosphate hydrolases superfamily protein | Chr1:6336528-6342460 REVERSE LENGTH=1145 | 201606	Arabidopsis_thaliana	OG0000008
AT1G18550.1 | ATP binding microtubule motor family protein | Chr1:6381656-6384340 REVERSE LENGTH=725 | 201606	Arabidopsis_thaliana	OG0000008
AT4G21270.1 | kinesin 1 | Chr4:11329579-11333884 REVERSE LENGTH=793 | 201606	Arabidopsis_thaliana	OG0000008
AT4G24170.1 | ATP binding microtubule motor family protein | Chr4:12543206-12546805 FORWARD LENGTH=1004 | 201606	Arabidopsis_thaliana	OG0000008
AT4G27180.1 | kinesin 2 | Chr4:13615057-13618689 REVERSE LENGTH=745 | 201606	Arabidopsis_thaliana	OG0000008
AT1G20060.1 | ATP binding microtubule motor family protein | Chr1:6950723-6956293 REVERSE LENGTH=970 | 201606	Arabidopsis_thaliana	OG0000008
AT4G38950.1 | ATP binding microtubule motor family protein | Chr4:18154606-18158461 REVERSE LENGTH=836 | 201606	Arabidopsis_thaliana	OG0000008
AT4G39050.1 | Kinesin motor family protein | Chr4:18193462-18200148 FORWARD LENGTH=1055 | 201606	Arabidopsis_thaliana	OG0000008
AT5G02370.1 | ATP binding microtubule motor family protein | Chr5:503444-505937 FORWARD LENGTH=564 | 201606	Arabidopsis_thaliana	OG0000008
AT5G06670.1 | P-loop containing nucleoside triphosphate hydrolases superfamily protein | Chr5:2048243-2055019 REVERSE LENGTH=986 | 201606	Arabidopsis_thaliana	OG0000008
AT5G10470.1 | kinesin like protein for actin based chloroplast movement 1 | Chr5:3290121-3297248 REVERSE LENGTH=1273 | 201606	Arabidopsis_thaliana	OG0000008
AT1G21730.1 | P-loop containing nucleoside triphosphate hydrolases superfamily protein | Chr1:7630365-7636247 FORWARD LENGTH=890 |

201606	Arabidopsis_thaliana	OG0000008
AT5G23910.1 | ATP binding microtubule motor family protein | Chr5:8068452-8072723 FORWARD LENGTH=701 | 201606	Arabidopsis_thaliana	OG0000008
AT5G27000.1 | kinesin 4 | Chr5:9498099-9502951 FORWARD LENGTH=987 | 201606	Arabidopsis_thaliana	OG0000008
AT5G27550.1 | P-loop containing nucleoside triphosphate hydrolases superfamily protein | Chr5:9727634-9731323 REVERSE LENGTH=765 | 201606	Arabidopsis_thaliana	OG0000008
AT5G27950.1 | P-loop containing nucleoside triphosphate hydrolases superfamily protein | Chr5:9984774-9987493 FORWARD LENGTH=625 | 201606	Arabidopsis_thaliana	OG0000008
AT5G41310.1 | P-loop nucleoside triphosphate hydrolases superfamily protein with CH (Calponin Homology) domain-containing protein | Chr5:16516634-16522392 REVERSE LENGTH=961 | 201606	Arabidopsis_thaliana	OG0000008
AT5G42490.1 | ATP binding microtubule motor family protein | Chr5:16988609-16992622 REVERSE LENGTH=1087 | 201606	Arabidopsis_thaliana	OG0000008
AT5G47820.1 | P-loop containing nucleoside triphosphate hydrolases superfamily protein | Chr5:19366505-19372229 FORWARD LENGTH=1035 | 201606	Arabidopsis_thaliana	OG0000008
AT5G54670.1 | kinesin 3 | Chr5:22209912-22213843 FORWARD LENGTH=754 | 201606	Arabidopsis_thaliana	OG0000008
AT5G60930.1 | P-loop containing nucleoside triphosphate hydrolases superfamily protein | Chr5:24515398-24522511 REVERSE LENGTH=1294 | 201606	Arabidopsis_thaliana	OG0000008
AT5G65460.1 | kinesin like protein for actin based chloroplast movement 2 | Chr5:26161831-26169001 REVERSE LENGTH=1264 | 201606	Arabidopsis_thaliana	OG0000008
AT5G65930.1 | kinesin-like calmodulin-binding protein (ZWICHEL) | Chr5:26370369-26376394 REVERSE LENGTH=1259 | 201606	Arabidopsis_thaliana	OG0000008
AT5G66310.1 | ATP binding microtubule motor family protein | Chr5:26485786-26490304 REVERSE LENGTH=1063 | 201606	Arabidopsis_thaliana	OG0000008
AT1G55550.1 | P-loop containing nucleoside triphosphate hydrolases superfamily protein | Chr1:20748915-20752862 FORWARD LENGTH=859 | 201606	Arabidopsis_thaliana	OG0000008
AT1G59540.1 | P-loop containing nucleoside triphosphate hydrolases superfamily protein | Chr1:21874083-21879382 FORWARD LENGTH=823 | 201606	Arabidopsis_thaliana	OG0000008
AT1G63640.1 | P-loop nucleoside triphosphate hydrolases superfamily protein with CH (Calponin Homology) domain-containing protein | Chr1:23589234-23595139 REVERSE LENGTH=1071 | 201606	Arabidopsis_thaliana	OG0000008
AT1G72250.1 | Di-glucose binding protein with Kinesin motor domain-containing protein | Chr1:27192902-27198118 FORWARD LENGTH=1195 | 201606	Arabidopsis_thaliana	OG0000008
AT1G73860.1 | P-loop containing nucleoside triphosphate hydrolases superfamily protein | Chr1:27771188-27775977 REVERSE LENGTH=1025 | 201606	Arabidopsis_thaliana	OG0000008
AT2G21300.1 | ATP binding microtubule motor family protein | Chr2:9114396-9118292 REVERSE LENGTH=862 | 201606

Arabidopsis_thaliana   OG0000008
AT2G21380.1 | Kinesin motor family protein | Chr2:9141833-9148883 FORWARD LENGTH=1058 | 201606   Arabidopsis_thaliana   OG0000008
AT1G09170.1 | P-loop nucleoside triphosphate hydrolases superfamily protein with CH (Calponin Homology) domain-containing protein | Chr1:2956589-2962207 REVERSE LENGTH=1010 | 201606   Arabidopsis_thaliana   OG0000008
AT2G22610.1 | Di-glucose binding protein with Kinesin motor domain-containing protein | Chr2:9599550-9604626 FORWARD LENGTH=1083 | 201606   Arabidopsis_thaliana   OG0000008
AT2G28620.1 | P-loop containing nucleoside triphosphate hydrolases superfamily protein | Chr2:12265167-12270020 REVERSE LENGTH=1042 | 201606   Arabidopsis_thaliana   OG0000008
AT1G01950.1 | armadillo repeat kinesin 2 | Chr1:325473-330403 FORWARD LENGTH=894 | 201606   Arabidopsis_thaliana   OG0000008
Os03t0301800-01 Similar to Kinesin-like polypeptides 9 (Fragment).   Oryza_sativa   OG0000008
Os01t0243100-01 Similar to Kinesin.   Oryza_sativa   OG0000008
Os03t0587200-01 Kinesin, motor region domain containing protein.   Oryza_sativa   OG0000008
Os03t0773600-01 Kinesin, motor region domain containing protein.   Oryza_sativa   OG0000008
Os04t0375900-01 Kinesin, motor region domain containing protein.   Oryza_sativa   OG0000008
Os04t0434600-00 Similar to OSIGBa0102D10.3 protein.   Oryza_sativa   OG0000008
Os04t0467901-00 Similar to OSIGBa0128P10.5 protein.   Oryza_sativa   OG0000008
Os04t0538800-01 Similar to Kinesin heavy chain (Fragment).   Oryza_sativa   OG0000008
Os04t0629700-01 Similar to Kinesin-1 (Kinesin-like protein A).   Oryza_sativa   OG0000008
Os04t0666900-01 Similar to H1005F08.22 protein.   Oryza_sativa   OG0000008
Os05t0117798-01 Kinesin, motor region domain containing protein.   Oryza_sativa   OG0000008
Os05t0397900-01 Kinesin, motor region domain containing protein.   Oryza_sativa   OG0000008
Os05t0459400-01 Kinesin, motor region domain containing protein.   Oryza_sativa   OG0000008
Os01t0513900-01 Similar to Kinesin heavy chain (Fragment).   Oryza_sativa   OG0000008
Os06t0137100-01 Similar to predicted protein.   Oryza_sativa   OG0000008
Os06t0206700-01 Similar to Kinesin-related protein.   Oryza_sativa   OG0000008
Os06t0217600-01 Similar to kinesin motor protein-related.   Oryza_sativa   OG0000008
Os06t0554700-01 Similar to kinesin motor protein-related.   Oryza_sativa   OG0000008
Os07t0105700-01 Similar to ATK5 (ARABIDOPSIS THALIANA KINESIN 5);

microtubule motor.	Oryza_sativa	OG0000008
Os01t0605500-00 Kinesin, motor region domain containing protein.
Oryza_sativa	OG0000008
Os08t0117000-01 Kinesin-1-like protein, Male meiosis and anther
dehiscence	Oryza_sativa	OG0000008
Os08t0547500-01 Kinesin, motor domain domain containing protein.
Oryza_sativa	OG0000008
Os08t0558400-01 Similar to Kinesin heavy chain (Fragment).
Oryza_sativa	OG0000008
Os09t0114500-01 Kinesin-4 protein with transcription regulation
activity, Cell cycle and wall modification, Cell elongation by
regulating GA biosynthesis pathway	Oryza_sativa
OG0000008
Os09t0528000-01 Kinesin, motor region domain containing protein.
Oryza_sativa	OG0000008
Os10t0512800-01 Similar to predicted protein.	Oryza_sativa
OG0000008
Os01t0744000-01 Similar to Kinesin heavy chain (Fragment).
Oryza_sativa	OG0000008
Os11t0648100-01 Kinesin, motor domain domain containing protein.
Oryza_sativa	OG0000008
Os11t0672400-00 Calponin homology domain domain containing protein.
Oryza_sativa	OG0000008
Os12t0547500-01 Kinesin, motor region domain containing protein.
Oryza_sativa	OG0000008
Os12t0590500-00 Similar to Kinesin motor domain containing protein,
expressed.	Oryza_sativa	OG0000008
Os12t0616000-01 Similar to Kinesin-like protein KIFC3.
Oryza_sativa	OG0000008
Os02t0101800-01 Kinesin, motor domain domain containing protein.
Oryza_sativa	OG0000008
Os02t0229600-00 Similar to ATP binding / microtubule motor.
Oryza_sativa	OG0000008
Os02t0644400-00 Similar to Kinesin heavy chain (Fragment).
Oryza_sativa	OG0000008
Os02t0645100-01 Kinesin, motor region domain containing protein.
Oryza_sativa	OG0000008
Os02t0742900-01 Similar to Kinesin-like protein.	Oryza_sativa
OG0000008
Os02t0775400-01 Similar to Kinesin heavy chain-like protein
(Fragment).	Oryza_sativa	OG0000008
Os03t0114000-01 Similar to Kinesin.	Oryza_sativa
OG0000008
Os03t0152900-01 Similar to predicted protein.	Oryza_sativa
OG0000008
CMC157CT pep chromosome:ASM9120v1:3:380216:382963:1 gene:CMC157C
transcript:CMC157CT gene_biotype:protein_coding
transcript_biotype:protein_coding Cyanidioschyzon_merolae
OG0000008
CMQ429CT pep chromosome:ASM9120v1:17:1119242:1122769:1 gene:CMQ429C
transcript:CMQ429CT gene_biotype:protein_coding
transcript_biotype:protein_coding Cyanidioschyzon_merolae
OG0000008
CMO070CT pep chromosome:ASM9120v1:15:182724:186596:1 gene:CMO070C

transcript:CMO070CT gene_biotype:protein_coding
transcript_biotype:protein_coding Cyanidioschyzon_merolae
OG0000008
CMT097CT pep chromosome:ASM9120v1:20:254502:257985:-1 gene:CMT097C
transcript:CMT097CT gene_biotype:protein_coding
transcript_biotype:protein_coding Cyanidioschyzon_merolae
OG0000008
CMR497CT pep chromosome:ASM9120v1:18:1226191:1228747:-1 gene:CMR497C
transcript:CMR497CT gene_biotype:protein_coding
transcript_biotype:protein_coding Cyanidioschyzon_merolae
OG0000008
evm.model.contig_2090.10 Porphyridium_purpureum OG0000008
evm.model.contig_2100.14 Porphyridium_purpureum OG0000008
evm.model.contig_3395.13 Porphyridium_purpureum OG0000008
evm.model.contig_4416.18 Porphyridium_purpureum OG0000008
evm.model.contig_4446.19 Porphyridium_purpureum OG0000008
evm.model.contig_2065.3 Porphyridium_purpureum OG0000008
CDF32880 pep supercontig:ASM35022v2:HG001593:159531:164700:1
gene:CHC_T00008304001 transcript:CDF32880
gene_biotype:protein_coding transcript_biotype:protein_coding
description:Kinesin Chondrus_crispus OG0000008
CDF34007 pep supercontig:ASM35022v2:HG001666:214570:216594:-1
gene:CHC_T00008641001 transcript:CDF34007
gene_biotype:protein_coding transcript_biotype:protein_coding
description:Kinesin-related Protein Chondrus_crispus
OG0000008
CDF32275 pep supercontig:ASM35022v2:HG001461:158154:160448:1
gene:CHC_T00009265001 transcript:CDF32275
gene_biotype:protein_coding transcript_biotype:protein_coding
description:Kinesin-like Protein Chondrus_crispus OG0000008
CDF32282 pep supercontig:ASM35022v2:HG001461:179194:183273:-1
gene:CHC_T00009138001 transcript:CDF32282
gene_biotype:protein_coding transcript_biotype:protein_coding
description:Kinesin-related protein Chondrus_crispus
OG0000008
CDF35474 pep supercontig:ASM35022v2:HG001731:178565:181418:1
gene:CHC_T00009381001 transcript:CDF35474
gene_biotype:protein_coding transcript_biotype:protein_coding
description:Kinesin Chondrus_crispus OG0000008
CDF38889 pep supercontig:ASM35022v2:HG001975:198099:201221:-1
gene:CHC_T00006378001 transcript:CDF38889
gene_biotype:protein_coding transcript_biotype:protein_coding
Chondrus_crispus OG0000008
CDF37548 pep supercontig:ASM35022v2:HG001850:51688:53592:-1
gene:CHC_T00008720001 transcript:CDF37548
gene_biotype:protein_coding transcript_biotype:protein_coding
description:Kinesin-like protein Chondrus_crispus OG0000008
g1747.t1 Caulerpa_lentillifera OG0000009
g2662.t1 Caulerpa_lentillifera OG0000009
g4684.t1 Caulerpa_lentillifera OG0000009
g6485.t1 Caulerpa_lentillifera OG0000009
g6542.t1 Caulerpa_lentillifera OG0000009
g713.t1 Caulerpa_lentillifera OG0000009
g8493.t1 Caulerpa_lentillifera OG0000009

Cre08.g364400.t1.1 pacid=30773488 transcript=Cre08.g364400.t1.1 locus=Cre08.g364400 ID=Cre08.g364400.t1.1.v5.5 annot-version=v5.5 Chlamydomonas_reinhardtii OG0000009
Cre07.g353500.t1.1 pacid=30775290 transcript=Cre07.g353500.t1.1 locus=Cre07.g353500 ID=Cre07.g353500.t1.1.v5.5 annot-version=v5.5 Chlamydomonas_reinhardtii OG0000009
Cre14.g620500.t1.2 pacid=30776478 transcript=Cre14.g620500.t1.2 locus=Cre14.g620500 ID=Cre14.g620500.t1.2.v5.5 annot-version=v5.5 Chlamydomonas_reinhardtii OG0000009
Cre16.g661650.t1.1 pacid=30777029 transcript=Cre16.g661650.t1.1 locus=Cre16.g661650 ID=Cre16.g661650.t1.1.v5.5 annot-version=v5.5 Chlamydomonas_reinhardtii OG0000009
Cre16.g649433.t1.1 pacid=30777250 transcript=Cre16.g649433.t1.1 locus=Cre16.g649433 ID=Cre16.g649433.t1.1.v5.5 annot-version=v5.5 Chlamydomonas_reinhardtii OG0000009
Cre16.g674890.t1.1 pacid=30777338 transcript=Cre16.g674890.t1.1 locus=Cre16.g674890 ID=Cre16.g674890.t1.1.v5.5 annot-version=v5.5 Chlamydomonas_reinhardtii OG0000009
Cre06.g275500.t1.2 pacid=30779983 transcript=Cre06.g275500.t1.2 locus=Cre06.g275500 ID=Cre06.g275500.t1.2.v5.5 annot-version=v5.5 Chlamydomonas_reinhardtii OG0000009
Vocar.0060s0003.1.p pacid=32895757 transcript=Vocar.0060s0003.1 locus=Vocar.0060s0003 ID=Vocar.0060s0003.1.v2.1 annot-version=v2.1 Volvox_carteri OG0000009
Vocar.0005s0116.1.p pacid=32883729 transcript=Vocar.0005s0116.1 locus=Vocar.0005s0116 ID=Vocar.0005s0116.1.v2.1 annot-version=v2.1 Volvox_carteri OG0000009
Vocar.0041s0059.1.p pacid=32888777 transcript=Vocar.0041s0059.1 locus=Vocar.0041s0059 ID=Vocar.0041s0059.1.v2.1 annot-version=v2.1 Volvox_carteri OG0000009
Vocar.0004s0259.1.p pacid=32889822 transcript=Vocar.0004s0259.1 locus=Vocar.0004s0259 ID=Vocar.0004s0259.1.v2.1 annot-version=v2.1 Volvox_carteri OG0000009
Vocar.0022s0152.1.p pacid=32891795 transcript=Vocar.0022s0152.1 locus=Vocar.0022s0152 ID=Vocar.0022s0152.1.v2.1 annot-version=v2.1 Volvox_carteri OG0000009
jgi|ChlNC64A_1|17998|gw1.7.372.1 Chlorella_variabilis OG0000009
jgi|ChlNC64A_1|57326|estExt_fgenesh3_pg.C_50293 Chlorella_variabilis OG0000009
66493 pacid=27385909 transcript=66493 locus=estExt_Genemark1.C_100079 ID=66493.2.0.227 annot-version=v2.0 Coccomyxa_subellipsoidea OG0000009
40506 pacid=27390076 transcript=40506 locus=fgenesh1_pg.4_#_155 ID=40506.2.0.227 annot-version=v2.0 Coccomyxa_subellipsoidea OG0000009
64766 pacid=27390647 transcript=64766 locus=estExt_Genemark1.C_30025 ID=64766.2.0.227 annot-version=v2.0 Coccomyxa_subellipsoidea OG0000009
48746 pacid=27394656 transcript=48746 locus=estExt_fgenesh1_pg.C_170126 ID=48746.2.0.227 annot-version=v2.0 Coccomyxa_subellipsoidea OG0000009
23659_estExt_fgenesh1_pg.C_Chr_04.00010339 Ostreococcus_tauri OG0000009

30470_0100010458 Ostreococcus_tauri OG0000009
9237 pacid=27415596 transcript=9237 locus=gwEuk.4.617.1
ID=9237.2.0.231 annot-version=v2.0 Ostreococcus_lucimarinus
OG0000009
23938 pacid=27417872 transcript=23938
locus=estExt_fgenesh1_pg.C_Chr_10369 ID=23938.2.0.231 annot-
version=v2.0 Ostreococcus_lucimarinus OG0000009
60155 pacid=27338990 transcript=60155 locus=MicpuC2.EuGene.
0000080349 ID=60155.3.0.228 annot-version=v3.0
Micromonas_pusilla OG0000009
50111 pacid=27345770 transcript=50111 locus=MicpuC2.EuGene.
0000010335 ID=50111.3.0.228 annot-version=v3.0
Micromonas_pusilla OG0000009
51075 pacid=27404408 transcript=51075
locus=est_orfs.Chr_07_946_3199283:1 ID=51075.3.0.229 annot-
version=v3.0 Micromonas_sp._RCC299 OG0000009
62515 pacid=27405286 transcript=62515 locus=EuGene.1100010301
ID=62515.3.0.229 annot-version=v3.0 Micromonas_sp._RCC299
OG0000009
kfl00677_0180_v1.1 Klebsormidium_flaccidum OG0000009
kfl00702_0050_v1.1 Klebsormidium_flaccidum OG0000009
kfl00809_0050_v1.1 Klebsormidium_flaccidum OG0000009
kfl01019_0040_v1.1 Klebsormidium_flaccidum OG0000009
kfl00326_g9_v1.1 Klebsormidium_flaccidum OG0000009
Pp3c7_20200V3.1.p pacid=32926144 transcript=Pp3c7_20200V3.1
locus=Pp3c7_20200 annot-version=v3.3 Physcomitrella_patens
OG0000009
Pp3c15_1990V3.1.p pacid=32926774 transcript=Pp3c15_1990V3.1
locus=Pp3c15_1990 annot-version=v3.3 Physcomitrella_patens
OG0000009
Pp3c15_3620V3.1.p pacid=32928180 transcript=Pp3c15_3620V3.1
locus=Pp3c15_3620 annot-version=v3.3 Physcomitrella_patens
OG0000009
Pp3c13_4270V3.1.p pacid=32930087 transcript=Pp3c13_4270V3.1
locus=Pp3c13_4270 annot-version=v3.3 Physcomitrella_patens
OG0000009
Pp3c10_9790V3.1.p pacid=32902554 transcript=Pp3c10_9790V3.1
locus=Pp3c10_9790 annot-version=v3.3 Physcomitrella_patens
OG0000009
Pp3c10_11910V3.1.p pacid=32902642 transcript=Pp3c10_11910V3.1
locus=Pp3c10_11910 annot-version=v3.3 Physcomitrella_patens
OG0000009
Pp3c13_24020V3.1.p pacid=32931047 transcript=Pp3c13_24020V3.1
locus=Pp3c13_24020 annot-version=v3.3 Physcomitrella_patens
OG0000009
Pp3c13_3830V3.1.p pacid=32931159 transcript=Pp3c13_3830V3.1
locus=Pp3c13_3830 annot-version=v3.3 Physcomitrella_patens
OG0000009
Pp3c13_3710V3.1.p pacid=32931573 transcript=Pp3c13_3710V3.1
locus=Pp3c13_3710 annot-version=v3.3 Physcomitrella_patens
OG0000009
Pp3c10_7030V3.1.p pacid=32899667 transcript=Pp3c10_7030V3.1
locus=Pp3c10_7030 annot-version=v3.3 Physcomitrella_patens
OG0000009

Pp3c13_9880V3.1.p pacid=32932100 transcript=Pp3c13_9880V3.1 locus=Pp3c13_9880 annot-version=v3.3 Physcomitrella_patens OG0000009
Pp3c2_11580V3.1.p pacid=32933478 transcript=Pp3c2_11580V3.1 locus=Pp3c2_11580 annot-version=v3.3 Physcomitrella_patens OG0000009
Pp3c2_5625V3.1.p pacid=32935107 transcript=Pp3c2_5625V3.1 locus=Pp3c2_5625 annot-version=v3.3 Physcomitrella_patens OG0000009
Pp3c2_25760V3.1.p pacid=32935893 transcript=Pp3c2_25760V3.1 locus=Pp3c2_25760 annot-version=v3.3 Physcomitrella_patens OG0000009
Pp3c2_15730V3.1.p pacid=32936037 transcript=Pp3c2_15730V3.1 locus=Pp3c2_15730 annot-version=v3.3 Physcomitrella_patens OG0000009
Pp3c2_33310V3.1.p pacid=32936534 transcript=Pp3c2_33310V3.1 locus=Pp3c2_33310 annot-version=v3.3 Physcomitrella_patens OG0000009
Pp3c2_36690V3.1.p pacid=32936657 transcript=Pp3c2_36690V3.1 locus=Pp3c2_36690 annot-version=v3.3 Physcomitrella_patens OG0000009
Pp3c2_5620V3.1.p pacid=32937410 transcript=Pp3c2_5620V3.1 locus=Pp3c2_5620 annot-version=v3.3 Physcomitrella_patens OG0000009
Pp3c2_1870V3.1.p pacid=32937465 transcript=Pp3c2_1870V3.1 locus=Pp3c2_1870 annot-version=v3.3 Physcomitrella_patens OG0000009
Pp3c19_6370V3.1.p pacid=32937955 transcript=Pp3c19_6370V3.1 locus=Pp3c19_6370 annot-version=v3.3 Physcomitrella_patens OG0000009
Pp3c19_10050V3.1.p pacid=32938202 transcript=Pp3c19_10050V3.1 locus=Pp3c19_10050 annot-version=v3.3 Physcomitrella_patens OG0000009
Pp3c19_14950V3.1.p pacid=32938262 transcript=Pp3c19_14950V3.1 locus=Pp3c19_14950 annot-version=v3.3 Physcomitrella_patens OG0000009
Pp3c19_20650V3.1.p pacid=32938280 transcript=Pp3c19_20650V3.1 locus=Pp3c19_20650 annot-version=v3.3 Physcomitrella_patens OG0000009
Pp3c22_10880V3.1.p pacid=32903504 transcript=Pp3c22_10880V3.1 locus=Pp3c22_10880 annot-version=v3.3 Physcomitrella_patens OG0000009
Pp3c3_7020V3.1.p pacid=32940607 transcript=Pp3c3_7020V3.1 locus=Pp3c3_7020 annot-version=v3.3 Physcomitrella_patens OG0000009
Pp3c3_19630V3.1.p pacid=32940751 transcript=Pp3c3_19630V3.1 locus=Pp3c3_19630 annot-version=v3.3 Physcomitrella_patens OG0000009
Pp3c3_17110V3.1.p pacid=32940828 transcript=Pp3c3_17110V3.1 locus=Pp3c3_17110 annot-version=v3.3 Physcomitrella_patens OG0000009
Pp3c3_9430V3.1.p pacid=32940887 transcript=Pp3c3_9430V3.1 locus=Pp3c3_9430 annot-version=v3.3 Physcomitrella_patens OG0000009

Pp3c3_6250V3.1.p pacid=32941186 transcript=Pp3c3_6250V3.1
locus=Pp3c3_6250 annot-version=v3.3 Physcomitrella_patens
OG0000009
Pp3c3_31490V3.1.p pacid=32941335 transcript=Pp3c3_31490V3.1
locus=Pp3c3_31490 annot-version=v3.3 Physcomitrella_patens
OG0000009
Pp3c3_33930V3.1.p pacid=32941347 transcript=Pp3c3_33930V3.1
locus=Pp3c3_33930 annot-version=v3.3 Physcomitrella_patens
OG0000009
Pp3c3_10430V3.1.p pacid=32942875 transcript=Pp3c3_10430V3.1
locus=Pp3c3_10430 annot-version=v3.3 Physcomitrella_patens
OG0000009
Pp3c3_31500V3.1.p pacid=32943080 transcript=Pp3c3_31500V3.1
locus=Pp3c3_31500 annot-version=v3.3 Physcomitrella_patens
OG0000009
Pp3c10_6570V3.1.p pacid=32899823 transcript=Pp3c10_6570V3.1
locus=Pp3c10_6570 annot-version=v3.3 Physcomitrella_patens
OG0000009
Pp3c3_13510V3.1.p pacid=32943605 transcript=Pp3c3_13510V3.1
locus=Pp3c3_13510 annot-version=v3.3 Physcomitrella_patens
OG0000009
Pp3c3_6370V3.1.p pacid=32943866 transcript=Pp3c3_6370V3.1
locus=Pp3c3_6370 annot-version=v3.3 Physcomitrella_patens
OG0000009
Pp3c3_6830V3.1.p pacid=32944438 transcript=Pp3c3_6830V3.1
locus=Pp3c3_6830 annot-version=v3.3 Physcomitrella_patens
OG0000009
Pp3c22_1800V3.1.p pacid=32904169 transcript=Pp3c22_1800V3.1
locus=Pp3c22_1800 annot-version=v3.3 Physcomitrella_patens
OG0000009
Pp3c20_3770V3.1.p pacid=32947644 transcript=Pp3c20_3770V3.1
locus=Pp3c20_3770 annot-version=v3.3 Physcomitrella_patens
OG0000009
Pp3c23_21450V3.1.p pacid=32949959 transcript=Pp3c23_21450V3.1
locus=Pp3c23_21450 annot-version=v3.3 Physcomitrella_patens
OG0000009
Pp3c27_5180V3.1.p pacid=32951392 transcript=Pp3c27_5180V3.1
locus=Pp3c27_5180 annot-version=v3.3 Physcomitrella_patens
OG0000009
Pp3c27_5490V3.1.p pacid=32951799 transcript=Pp3c27_5490V3.1
locus=Pp3c27_5490 annot-version=v3.3 Physcomitrella_patens
OG0000009
Pp3c27_1350V3.1.p pacid=32951867 transcript=Pp3c27_1350V3.1
locus=Pp3c27_1350 annot-version=v3.3 Physcomitrella_patens
OG0000009
Pp3c27_6030V3.1.p pacid=32951880 transcript=Pp3c27_6030V3.1
locus=Pp3c27_6030 annot-version=v3.3 Physcomitrella_patens
OG0000009
Pp3c5_11150V3.1.p pacid=32952864 transcript=Pp3c5_11150V3.1
locus=Pp3c5_11150 annot-version=v3.3 Physcomitrella_patens
OG0000009
Pp3c5_22030V3.1.p pacid=32953153 transcript=Pp3c5_22030V3.1
locus=Pp3c5_22030 annot-version=v3.3 Physcomitrella_patens
OG0000009

Pp3c5_22280V3.1.p pacid=32953749 transcript=Pp3c5_22280V3.1 locus=Pp3c5_22280 annot-version=v3.3 Physcomitrella_patens OG0000009
Pp3c5_820V3.1.p pacid=32954032 transcript=Pp3c5_820V3.1 locus=Pp3c5_820 annot-version=v3.3 Physcomitrella_patens OG0000009
Pp3c5_810V3.1.p pacid=32954034 transcript=Pp3c5_810V3.1 locus=Pp3c5_810 annot-version=v3.3 Physcomitrella_patens OG0000009
Pp3c22_20520V3.1.p pacid=32904980 transcript=Pp3c22_20520V3.1 locus=Pp3c22_20520 annot-version=v3.3 Physcomitrella_patens OG0000009
Pp3c5_11160V3.1.p pacid=32954329 transcript=Pp3c5_11160V3.1 locus=Pp3c5_11160 annot-version=v3.3 Physcomitrella_patens OG0000009
Pp3c5_10960V3.1.p pacid=32954514 transcript=Pp3c5_10960V3.1 locus=Pp3c5_10960 annot-version=v3.3 Physcomitrella_patens OG0000009
Pp3c5_2900V3.1.p pacid=32954642 transcript=Pp3c5_2900V3.1 locus=Pp3c5_2900 annot-version=v3.3 Physcomitrella_patens OG0000009
Pp3c5_4660V3.1.p pacid=32954776 transcript=Pp3c5_4660V3.1 locus=Pp3c5_4660 annot-version=v3.3 Physcomitrella_patens OG0000009
Pp3c5_8580V3.1.p pacid=32955230 transcript=Pp3c5_8580V3.1 locus=Pp3c5_8580 annot-version=v3.3 Physcomitrella_patens OG0000009
Pp3c5_880V3.1.p pacid=32956071 transcript=Pp3c5_880V3.1 locus=Pp3c5_880 annot-version=v3.3 Physcomitrella_patens OG0000009
Pp3c11_10660V3.1.p pacid=32956292 transcript=Pp3c11_10660V3.1 locus=Pp3c11_10660 annot-version=v3.3 Physcomitrella_patens OG0000009
Pp3c11_23290V3.1.p pacid=32956649 transcript=Pp3c11_23290V3.1 locus=Pp3c11_23290 annot-version=v3.3 Physcomitrella_patens OG0000009
Pp3c11_14690V3.1.p pacid=32956825 transcript=Pp3c11_14690V3.1 locus=Pp3c11_14690 annot-version=v3.3 Physcomitrella_patens OG0000009
Pp3c11_13860V3.1.p pacid=32958975 transcript=Pp3c11_13860V3.1 locus=Pp3c11_13860 annot-version=v3.3 Physcomitrella_patens OG0000009
Pp3c11_24520V3.1.p pacid=32959487 transcript=Pp3c11_24520V3.1 locus=Pp3c11_24520 annot-version=v3.3 Physcomitrella_patens OG0000009
Pp3c11_13880V3.1.p pacid=32959607 transcript=Pp3c11_13880V3.1 locus=Pp3c11_13880 annot-version=v3.3 Physcomitrella_patens OG0000009
Pp3c14_9940V3.1.p pacid=32960909 transcript=Pp3c14_9940V3.1 locus=Pp3c14_9940 annot-version=v3.3 Physcomitrella_patens OG0000009
Pp3c14_3310V3.1.p pacid=32961904 transcript=Pp3c14_3310V3.1 locus=Pp3c14_3310 annot-version=v3.3 Physcomitrella_patens OG0000009

Pp3c14_3280V3.1.p pacid=32961990 transcript=Pp3c14_3280V3.1 locus=Pp3c14_3280 annot-version=v3.3 Physcomitrella_patens OG0000009
Pp3c14_8460V3.1.p pacid=32962159 transcript=Pp3c14_8460V3.1 locus=Pp3c14_8460 annot-version=v3.3 Physcomitrella_patens OG0000009
Pp3s423_30V3.1.p pacid=32963346 transcript=Pp3s423_30V3.1 locus=Pp3s423_30 annot-version=v3.3 Physcomitrella_patens OG0000009
Pp3s423_10V3.1.p pacid=32963349 transcript=Pp3s423_10V3.1 locus=Pp3s423_10 annot-version=v3.3 Physcomitrella_patens OG0000009
Pp3c8_7340V3.1.p pacid=32964400 transcript=Pp3c8_7340V3.1 locus=Pp3c8_7340 annot-version=v3.3 Physcomitrella_patens OG0000009
Pp3c17_20740V3.1.p pacid=32906011 transcript=Pp3c17_20740V3.1 locus=Pp3c17_20740 annot-version=v3.3 Physcomitrella_patens OG0000009
Pp3c8_24200V3.1.p pacid=32965163 transcript=Pp3c8_24200V3.1 locus=Pp3c8_24200 annot-version=v3.3 Physcomitrella_patens OG0000009
Pp3c1_40330V3.1.p pacid=32967519 transcript=Pp3c1_40330V3.1 locus=Pp3c1_40330 annot-version=v3.3 Physcomitrella_patens OG0000009
Pp3c1_28430V3.1.p pacid=32967717 transcript=Pp3c1_28430V3.1 locus=Pp3c1_28430 annot-version=v3.3 Physcomitrella_patens OG0000009
Pp3c1_36120V3.1.p pacid=32968518 transcript=Pp3c1_36120V3.1 locus=Pp3c1_36120 annot-version=v3.3 Physcomitrella_patens OG0000009
Pp3c1_36129V3.1.p pacid=32968530 transcript=Pp3c1_36129V3.1 locus=Pp3c1_36129 annot-version=v3.3 Physcomitrella_patens OG0000009
Pp3c1_7740V3.1.p pacid=32969096 transcript=Pp3c1_7740V3.1 locus=Pp3c1_7740 annot-version=v3.3 Physcomitrella_patens OG0000009
Pp3c1_27710V3.1.p pacid=32969221 transcript=Pp3c1_27710V3.1 locus=Pp3c1_27710 annot-version=v3.3 Physcomitrella_patens OG0000009
Pp3c17_3450V3.1.p pacid=32906523 transcript=Pp3c17_3450V3.1 locus=Pp3c17_3450 annot-version=v3.3 Physcomitrella_patens OG0000009
Pp3c1_7800V3.1.p pacid=32970298 transcript=Pp3c1_7800V3.1 locus=Pp3c1_7800 annot-version=v3.3 Physcomitrella_patens OG0000009
Pp3c1_27440V3.1.p pacid=32970797 transcript=Pp3c1_27440V3.1 locus=Pp3c1_27440 annot-version=v3.3 Physcomitrella_patens OG0000009
Pp3c1_32440V3.1.p pacid=32971772 transcript=Pp3c1_32440V3.1 locus=Pp3c1_32440 annot-version=v3.3 Physcomitrella_patens OG0000009
Pp3c1_14230V3.1.p pacid=32971930 transcript=Pp3c1_14230V3.1 locus=Pp3c1_14230 annot-version=v3.3 Physcomitrella_patens OG0000009

Pp3c1_5010V3.1.p pacid=32972068 transcript=Pp3c1_5010V3.1 locus=Pp3c1_5010 annot-version=v3.3 Physcomitrella_patens OG0000009
Pp3c1_27630V3.1.p pacid=32972070 transcript=Pp3c1_27630V3.1 locus=Pp3c1_27630 annot-version=v3.3 Physcomitrella_patens OG0000009
Pp3c12_25330V3.1.p pacid=32972840 transcript=Pp3c12_25330V3.1 locus=Pp3c12_25330 annot-version=v3.3 Physcomitrella_patens OG0000009
Pp3c17_13620V3.1.p pacid=32906971 transcript=Pp3c17_13620V3.1 locus=Pp3c17_13620 annot-version=v3.3 Physcomitrella_patens OG0000009
Pp3c12_20550V3.1.p pacid=32974320 transcript=Pp3c12_20550V3.1 locus=Pp3c12_20550 annot-version=v3.3 Physcomitrella_patens OG0000009
Pp3c6_16660V3.1.p pacid=32975460 transcript=Pp3c6_16660V3.1 locus=Pp3c6_16660 annot-version=v3.3 Physcomitrella_patens OG0000009
Pp3c6_28260V3.1.p pacid=32975755 transcript=Pp3c6_28260V3.1 locus=Pp3c6_28260 annot-version=v3.3 Physcomitrella_patens OG0000009
Pp3c6_26080V3.1.p pacid=32976480 transcript=Pp3c6_26080V3.1 locus=Pp3c6_26080 annot-version=v3.3 Physcomitrella_patens OG0000009
Pp3c6_14880V3.1.p pacid=32976848 transcript=Pp3c6_14880V3.1 locus=Pp3c6_14880 annot-version=v3.3 Physcomitrella_patens OG0000009
Pp3c6_22030V3.1.p pacid=32976968 transcript=Pp3c6_22030V3.1 locus=Pp3c6_22030 annot-version=v3.3 Physcomitrella_patens OG0000009
Pp3c6_20480V3.1.p pacid=32978025 transcript=Pp3c6_20480V3.1 locus=Pp3c6_20480 annot-version=v3.3 Physcomitrella_patens OG0000009
Pp3c6_19350V3.1.p pacid=32978142 transcript=Pp3c6_19350V3.1 locus=Pp3c6_19350 annot-version=v3.3 Physcomitrella_patens OG0000009
Pp3c6_28290V3.1.p pacid=32978168 transcript=Pp3c6_28290V3.1 locus=Pp3c6_28290 annot-version=v3.3 Physcomitrella_patens OG0000009
Pp3c6_28370V3.1.p pacid=32978669 transcript=Pp3c6_28370V3.1 locus=Pp3c6_28370 annot-version=v3.3 Physcomitrella_patens OG0000009
Pp3c25_1760V3.1.p pacid=32979104 transcript=Pp3c25_1760V3.1 locus=Pp3c25_1760 annot-version=v3.3 Physcomitrella_patens OG0000009
Pp3c25_12880V3.1.p pacid=32979658 transcript=Pp3c25_12880V3.1 locus=Pp3c25_12880 annot-version=v3.3 Physcomitrella_patens OG0000009
Pp3c25_1190V3.1.p pacid=32979717 transcript=Pp3c25_1190V3.1 locus=Pp3c25_1190 annot-version=v3.3 Physcomitrella_patens OG0000009
Pp3c25_4250V3.1.p pacid=32980884 transcript=Pp3c25_4250V3.1 locus=Pp3c25_4250 annot-version=v3.3 Physcomitrella_patens OG0000009

Pp3c18_10680V3.1.p pacid=32982667 transcript=Pp3c18_10680V3.1 locus=Pp3c18_10680 annot-version=v3.3 Physcomitrella_patens OG0000009
Pp3c18_1800V3.1.p pacid=32982942 transcript=Pp3c18_1800V3.1 locus=Pp3c18_1800 annot-version=v3.3 Physcomitrella_patens OG0000009
Pp3c17_10170V3.1.p pacid=32907968 transcript=Pp3c17_10170V3.1 locus=Pp3c17_10170 annot-version=v3.3 Physcomitrella_patens OG0000009
Pp3c16_17550V3.1.p pacid=32984091 transcript=Pp3c16_17550V3.1 locus=Pp3c16_17550 annot-version=v3.3 Physcomitrella_patens OG0000009
Pp3c16_13260V3.1.p pacid=32984257 transcript=Pp3c16_13260V3.1 locus=Pp3c16_13260 annot-version=v3.3 Physcomitrella_patens OG0000009
Pp3c16_12850V3.1.p pacid=32984268 transcript=Pp3c16_12850V3.1 locus=Pp3c16_12850 annot-version=v3.3 Physcomitrella_patens OG0000009
Pp3c16_4710V3.1.p pacid=32984297 transcript=Pp3c16_4710V3.1 locus=Pp3c16_4710 annot-version=v3.3 Physcomitrella_patens OG0000009
Pp3c16_13280V3.1.p pacid=32984562 transcript=Pp3c16_13280V3.1 locus=Pp3c16_13280 annot-version=v3.3 Physcomitrella_patens OG0000009
Pp3c16_3770V3.1.p pacid=32985961 transcript=Pp3c16_3770V3.1 locus=Pp3c16_3770 annot-version=v3.3 Physcomitrella_patens OG0000009
Pp3c16_13060V3.1.p pacid=32986606 transcript=Pp3c16_13060V3.1 locus=Pp3c16_13060 annot-version=v3.3 Physcomitrella_patens OG0000009
Pp3c16_24430V3.1.p pacid=32986664 transcript=Pp3c16_24430V3.1 locus=Pp3c16_24430 annot-version=v3.3 Physcomitrella_patens OG0000009
Pp3c10_6600V3.1.p pacid=32900351 transcript=Pp3c10_6600V3.1 locus=Pp3c10_6600 annot-version=v3.3 Physcomitrella_patens OG0000009
Pp3c10_16720V3.1.p pacid=32899457 transcript=Pp3c10_16720V3.1 locus=Pp3c10_16720 annot-version=v3.3 Physcomitrella_patens OG0000009
Pp3c9_2020V3.1.p pacid=32913070 transcript=Pp3c9_2020V3.1 locus=Pp3c9_2020 annot-version=v3.3 Physcomitrella_patens OG0000009
Pp3c9_4590V3.1.p pacid=32913152 transcript=Pp3c9_4590V3.1 locus=Pp3c9_4590 annot-version=v3.3 Physcomitrella_patens OG0000009
Pp3c9_14080V3.1.p pacid=32913891 transcript=Pp3c9_14080V3.1 locus=Pp3c9_14080 annot-version=v3.3 Physcomitrella_patens OG0000009
Pp3c21_13130V3.1.p pacid=32914746 transcript=Pp3c21_13130V3.1 locus=Pp3c21_13130 annot-version=v3.3 Physcomitrella_patens OG0000009
Pp3c21_21070V3.1.p pacid=32915246 transcript=Pp3c21_21070V3.1 locus=Pp3c21_21070 annot-version=v3.3 Physcomitrella_patens OG0000009

Pp3c21_8710V3.1.p pacid=32915697 transcript=Pp3c21_8710V3.1
locus=Pp3c21_8710 annot-version=v3.3 Physcomitrella_patens
OG0000009
Pp3c21_16540V3.1.p pacid=32916762 transcript=Pp3c21_16540V3.1
locus=Pp3c21_16540 annot-version=v3.3 Physcomitrella_patens
OG0000009
Pp3c10_20000V3.1.p pacid=32901201 transcript=Pp3c10_20000V3.1
locus=Pp3c10_20000 annot-version=v3.3 Physcomitrella_patens
OG0000009
Pp3c26_13260V3.1.p pacid=32917238 transcript=Pp3c26_13260V3.1
locus=Pp3c26_13260 annot-version=v3.3 Physcomitrella_patens
OG0000009
Pp3c26_9880V3.1.p pacid=32917379 transcript=Pp3c26_9880V3.1
locus=Pp3c26_9880 annot-version=v3.3 Physcomitrella_patens
OG0000009
Pp3c26_1890V3.1.p pacid=32917878 transcript=Pp3c26_1890V3.1
locus=Pp3c26_1890 annot-version=v3.3 Physcomitrella_patens
OG0000009
Pp3c26_14710V3.1.p pacid=32918309 transcript=Pp3c26_14710V3.1
locus=Pp3c26_14710 annot-version=v3.3 Physcomitrella_patens
OG0000009
Pp3c26_10480V3.1.p pacid=32918741 transcript=Pp3c26_10480V3.1
locus=Pp3c26_10480 annot-version=v3.3 Physcomitrella_patens
OG0000009
Pp3c4_23930V3.1.p pacid=32919369 transcript=Pp3c4_23930V3.1
locus=Pp3c4_23930 annot-version=v3.3 Physcomitrella_patens
OG0000009
Pp3c4_14760V3.1.p pacid=32919873 transcript=Pp3c4_14760V3.1
locus=Pp3c4_14760 annot-version=v3.3 Physcomitrella_patens
OG0000009
Pp3c4_25130V3.1.p pacid=32920040 transcript=Pp3c4_25130V3.1
locus=Pp3c4_25130 annot-version=v3.3 Physcomitrella_patens
OG0000009
Pp3c10_17870V3.1.p pacid=32901546 transcript=Pp3c10_17870V3.1
locus=Pp3c10_17870 annot-version=v3.3 Physcomitrella_patens
OG0000009
Pp3c4_27870V3.1.p pacid=32920908 transcript=Pp3c4_27870V3.1
locus=Pp3c4_27870 annot-version=v3.3 Physcomitrella_patens
OG0000009
Pp3c4_2680V3.1.p pacid=32920919 transcript=Pp3c4_2680V3.1
locus=Pp3c4_2680 annot-version=v3.3 Physcomitrella_patens
OG0000009
Pp3c4_2530V3.1.p pacid=32921624 transcript=Pp3c4_2530V3.1
locus=Pp3c4_2530 annot-version=v3.3 Physcomitrella_patens
OG0000009
Pp3c4_31920V3.1.p pacid=32921737 transcript=Pp3c4_31920V3.1
locus=Pp3c4_31920 annot-version=v3.3 Physcomitrella_patens
OG0000009
Pp3c4_24710V3.1.p pacid=32922042 transcript=Pp3c4_24710V3.1
locus=Pp3c4_24710 annot-version=v3.3 Physcomitrella_patens
OG0000009
Pp3c4_2660V3.1.p pacid=32922292 transcript=Pp3c4_2660V3.1
locus=Pp3c4_2660 annot-version=v3.3 Physcomitrella_patens
OG0000009

Pp3c7_20170V3.1.p pacid=32922998 transcript=Pp3c7_20170V3.1 locus=Pp3c7_20170 annot-version=v3.3 Physcomitrella_patens OG0000009
Pp3c7_10780V3.1.p pacid=32924980 transcript=Pp3c7_10780V3.1 locus=Pp3c7_10780 annot-version=v3.3 Physcomitrella_patens OG0000009
Pp3c7_2300V3.1.p pacid=32925053 transcript=Pp3c7_2300V3.1 locus=Pp3c7_2300 annot-version=v3.3 Physcomitrella_patens OG0000009
Pp3c7_1240V3.1.p pacid=32925584 transcript=Pp3c7_1240V3.1 locus=Pp3c7_1240 annot-version=v3.3 Physcomitrella_patens OG0000009
Pp3c7_17700V3.1.p pacid=32925901 transcript=Pp3c7_17700V3.1 locus=Pp3c7_17700 annot-version=v3.3 Physcomitrella_patens OG0000009
Pp3c7_18530V3.1.p pacid=32925977 transcript=Pp3c7_18530V3.1 locus=Pp3c7_18530 annot-version=v3.3 Physcomitrella_patens OG0000009
AT2G35700.1 | ERF family protein 38 | Chr2:15005205-15005789 FORWARD LENGTH=194 | 201606 Arabidopsis_thaliana OG0000009
AT2G36450.1 | Integrase-type DNA-binding superfamily protein | Chr2:15294303-15294857 REVERSE LENGTH=184 | 201606 Arabidopsis_thaliana OG0000009
AT2G38340.1 | Integrase-type DNA-binding superfamily protein | Chr2:16067447-16068181 REVERSE LENGTH=244 | 201606 Arabidopsis_thaliana OG0000009
AT2G40220.1 | Integrase-type DNA-binding superfamily protein | Chr2:16796599-16797585 REVERSE LENGTH=328 | 201606 Arabidopsis_thaliana OG0000009
AT2G40340.1 | Integrase-type DNA-binding superfamily protein | Chr2:16848665-16850357 REVERSE LENGTH=341 | 201606 Arabidopsis_thaliana OG0000009
AT2G40350.1 | Integrase-type DNA-binding superfamily protein | Chr2:16851595-16852068 REVERSE LENGTH=157 | 201606 Arabidopsis_thaliana OG0000009
AT2G44840.1 | ethylene-responsive element binding factor 13 | Chr2:18495440-18496120 FORWARD LENGTH=226 | 201606 Arabidopsis_thaliana OG0000009
AT2G44940.1 | Integrase-type DNA-binding superfamily protein | Chr2:18537294-18538181 FORWARD LENGTH=295 | 201606 Arabidopsis_thaliana OG0000009
AT2G46310.1 | cytokinin response factor 5 | Chr2:19011614-19012498 FORWARD LENGTH=294 | 201606 Arabidopsis_thaliana OG0000009
AT2G47520.1 | Integrase-type DNA-binding superfamily protein | Chr2:19502891-19503490 REVERSE LENGTH=171 | 201606 Arabidopsis_thaliana OG0000009
AT3G11020.1 | DRE/CRT-binding protein 2B | Chr3:3456009-3457001 FORWARD LENGTH=330 | 201606 Arabidopsis_thaliana OG0000009
AT1G12610.1 | Integrase-type DNA-binding superfamily protein | Chr1:4290205-4290834 REVERSE LENGTH=209 | 201606 Arabidopsis_thaliana OG0000009
AT1G12630.1 | Integrase-type DNA-binding superfamily protein |

Chr1:4298897-4299475 FORWARD LENGTH=192 | 201606
Arabidopsis_thaliana   OG0000009
AT1G12890.1 | Integrase-type DNA-binding superfamily protein |
Chr1:4391734-4392393 FORWARD LENGTH=219 | 201606
Arabidopsis_thaliana   OG0000009
AT3G14230.1 | related to AP2 2 | Chr3:4737616-4739000 REVERSE
LENGTH=379 | 201606   Arabidopsis_thaliana   OG0000009
AT1G12980.1 | Integrase-type DNA-binding superfamily protein |
Chr1:4429791-4430777 FORWARD LENGTH=328 | 201606
Arabidopsis_thaliana   OG0000009
AT3G15210.1 | ethylene responsive element binding factor 4 |
Chr3:5121472-5122140 FORWARD LENGTH=222 | 201606
Arabidopsis_thaliana   OG0000009
AT3G16280.1 | Integrase-type DNA-binding superfamily protein |
Chr3:5518355-5519065 FORWARD LENGTH=236 | 201606
Arabidopsis_thaliana   OG0000009
AT3G16770.1 | ethylene-responsive element binding protein |
Chr3:5705784-5706768 FORWARD LENGTH=248 | 201606
Arabidopsis_thaliana   OG0000009
AT3G20310.1 | ethylene response factor 7 | Chr3:7085957-7086691
REVERSE LENGTH=244 | 201606   Arabidopsis_thaliana
OG0000009
AT3G23220.1 | Integrase-type DNA-binding superfamily protein |
Chr3:8287969-8288388 FORWARD LENGTH=139 | 201606
Arabidopsis_thaliana   OG0000009
AT3G23230.1 | Integrase-type DNA-binding superfamily protein |
Chr3:8289647-8290066 REVERSE LENGTH=139 | 201606
Arabidopsis_thaliana   OG0000009
AT3G23240.1 | ethylene response factor 1 | Chr3:8295705-8296361
FORWARD LENGTH=218 | 201606   Arabidopsis_thaliana
OG0000009
AT3G25890.1 | Integrase-type DNA-binding superfamily protein |
Chr3:9476052-9477050 FORWARD LENGTH=332 | 201606
Arabidopsis_thaliana   OG0000009
AT1G15360.1 | Integrase-type DNA-binding superfamily protein |
Chr1:5283609-5284506 FORWARD LENGTH=199 | 201606
Arabidopsis_thaliana   OG0000009
AT3G50260.1 | cooperatively regulated by ethylene and jasmonate 1 |
Chr3:18634687-18635148 FORWARD LENGTH=153 | 201606
Arabidopsis_thaliana   OG0000009
AT3G57600.1 | Integrase-type DNA-binding superfamily protein |
Chr3:21332841-21333674 FORWARD LENGTH=277 | 201606
Arabidopsis_thaliana   OG0000009
AT3G60490.1 | Integrase-type DNA-binding superfamily protein |
Chr3:22349527-22350297 FORWARD LENGTH=256 | 201606
Arabidopsis_thaliana   OG0000009
AT3G61630.1 | cytokinin response factor 6 | Chr3:22805274-22806221
FORWARD LENGTH=315 | 201606   Arabidopsis_thaliana
OG0000009
AT4G06746.1 | related to AP2 9 | Chr4:4074093-4074545 REVERSE
LENGTH=150 | 201606   Arabidopsis_thaliana   OG0000009
AT4G11140.1 | cytokinin response factor 1 | Chr4:6794930-6795793
REVERSE LENGTH=287 | 201606   Arabidopsis_thaliana
OG0000009

AT4G13620.1 | Integrase-type DNA-binding superfamily protein |
Chr4:7932138-7933304 FORWARD LENGTH=388 | 201606
Arabidopsis_thaliana OG0000009
AT4G16750.1 | Integrase-type DNA-binding superfamily protein |
Chr4:9421121-9421660 REVERSE LENGTH=179 | 201606
Arabidopsis_thaliana OG0000009
AT4G17490.1 | ethylene responsive element binding factor 6 |
Chr4:9752973-9753821 REVERSE LENGTH=282 | 201606
Arabidopsis_thaliana OG0000009
AT4G17500.1 | ethylene responsive element binding factor 1 |
Chr4:9759405-9760211 FORWARD LENGTH=268 | 201606
Arabidopsis_thaliana OG0000009
AT4G18450.1 | Integrase-type DNA-binding superfamily protein |
Chr4:10190250-10191161 REVERSE LENGTH=303 | 201606
Arabidopsis_thaliana OG0000009
AT4G23750.1 | cytokinin response factor 2 | Chr4:12376751-12377782
FORWARD LENGTH=343 | 201606 Arabidopsis_thaliana
OG0000009
AT1G19210.1 | Integrase-type DNA-binding superfamily protein |
Chr1:6626973-6627530 REVERSE LENGTH=185 | 201606
Arabidopsis_thaliana OG0000009
AT4G25470.1 | C-repeat/DRE binding factor 2 | Chr4:13015436-13016086
REVERSE LENGTH=216 | 201606 Arabidopsis_thaliana
OG0000009
AT4G25480.1 | dehydration response element B1A |
Chr4:13018353-13019003 REVERSE LENGTH=216 | 201606
Arabidopsis_thaliana OG0000009
AT4G25490.1 | C-repeat/DRE binding factor 1 | Chr4:13021921-13022562
REVERSE LENGTH=213 | 201606 Arabidopsis_thaliana
OG0000009
AT4G27950.1 | cytokinin response factor 4 | Chr4:13909732-13910739
REVERSE LENGTH=335 | 201606 Arabidopsis_thaliana
OG0000009
AT4G28140.1 | Integrase-type DNA-binding superfamily protein |
Chr4:13974905-13975783 REVERSE LENGTH=292 | 201606
Arabidopsis_thaliana OG0000009
AT4G31060.1 | Integrase-type DNA-binding superfamily protein |
Chr4:15116896-15117459 FORWARD LENGTH=187 | 201606
Arabidopsis_thaliana OG0000009
AT4G32800.1 | Integrase-type DNA-binding superfamily protein |
Chr4:15819812-15820477 FORWARD LENGTH=221 | 201606
Arabidopsis_thaliana OG0000009
AT4G34410.1 | redox responsive transcription factor 1 |
Chr4:16451992-16452798 FORWARD LENGTH=268 | 201606
Arabidopsis_thaliana OG0000009
AT4G36900.1 | related to AP2 10 | Chr4:17389015-17389605 FORWARD
LENGTH=196 | 201606 Arabidopsis_thaliana OG0000009
AT4G39780.1 | Integrase-type DNA-binding superfamily protein |
Chr4:18458216-18459034 REVERSE LENGTH=272 | 201606
Arabidopsis_thaliana OG0000009
AT5G05410.1 | DRE-binding protein 2A | Chr5:1602650-1603657 FORWARD
LENGTH=335 | 201606 Arabidopsis_thaliana OG0000009
AT5G07310.1 | Integrase-type DNA-binding superfamily protein |
Chr5:2305684-2306660 FORWARD LENGTH=263 | 201606

Arabidopsis_thaliana   OG0000009
AT5G07580.1 | ethylene-responsive transcription factor |
Chr5:2399726-2400349 FORWARD LENGTH=207 | 201606
Arabidopsis_thaliana   OG0000009
AT5G11190.1 | Integrase-type DNA-binding superfamily protein |
Chr5:3564976-3566051 FORWARD LENGTH=189 | 201606
Arabidopsis_thaliana   OG0000009
AT5G11590.1 | Integrase-type DNA-binding superfamily protein |
Chr5:3727789-3728499 REVERSE LENGTH=236 | 201606
Arabidopsis_thaliana   OG0000009
AT1G21910.1 | Integrase-type DNA-binding superfamily protein |
Chr1:7696655-7697347 FORWARD LENGTH=230 | 201606
Arabidopsis_thaliana   OG0000009
AT5G13330.1 | related to AP2 6l | Chr5:4272384-4274461 FORWARD
LENGTH=212 | 201606   Arabidopsis_thaliana   OG0000009
AT5G13910.1 | Integrase-type DNA-binding superfamily protein |
Chr5:4482450-4483085 REVERSE LENGTH=211 | 201606
Arabidopsis_thaliana   OG0000009
AT1G22190.1 | Integrase-type DNA-binding superfamily protein |
Chr1:7836245-7837030 FORWARD LENGTH=261 | 201606
Arabidopsis_thaliana   OG0000009
AT5G18450.1 | Integrase-type DNA-binding superfamily protein |
Chr5:6116097-6117020 REVERSE LENGTH=307 | 201606
Arabidopsis_thaliana   OG0000009
AT5G18560.1 | Integrase-type DNA-binding superfamily protein |
Chr5:6164859-6165905 REVERSE LENGTH=348 | 201606
Arabidopsis_thaliana   OG0000009
AT5G19790.1 | related to AP2 11 | Chr5:6689271-6690032 REVERSE
LENGTH=253 | 201606   Arabidopsis_thaliana   OG0000009
AT1G22810.1 | Integrase-type DNA-binding superfamily protein |
Chr1:8074535-8074969 REVERSE LENGTH=144 | 201606
Arabidopsis_thaliana   OG0000009
AT5G21960.1 | Integrase-type DNA-binding superfamily protein |
Chr5:7258560-7259210 REVERSE LENGTH=216 | 201606
Arabidopsis_thaliana   OG0000009
AT1G22985.1 | Integrase-type DNA-binding superfamily protein |
Chr1:8135346-8135825 REVERSE LENGTH=159 | 201606
Arabidopsis_thaliana   OG0000009
AT5G25190.1 | Integrase-type DNA-binding superfamily protein |
Chr5:8707007-8707655 REVERSE LENGTH=181 | 201606
Arabidopsis_thaliana   OG0000009
AT5G25390.1 | Integrase-type DNA-binding superfamily protein |
Chr5:8820637-8821741 FORWARD LENGTH=186 | 201606
Arabidopsis_thaliana   OG0000009
AT5G25810.1 | Integrase-type DNA-binding superfamily protein |
Chr5:8986976-8987632 REVERSE LENGTH=218 | 201606
Arabidopsis_thaliana   OG0000009
AT5G43410.1 | Integrase-type DNA-binding superfamily protein |
Chr5:17435010-17435405 REVERSE LENGTH=131 | 201606
Arabidopsis_thaliana   OG0000009
AT5G44210.1 | erf domain protein 9 | Chr5:17806742-17807344 FORWARD
LENGTH=200 | 201606   Arabidopsis_thaliana   OG0000009
AT1G01250.1 | Integrase-type DNA-binding superfamily protein |
Chr1:104731-105309 REVERSE LENGTH=192 | 201606

Arabidopsis_thaliana   OG0000009
AT1G24590.1 | DORNROSCHEN-like protein | Chr1:8714389-8715309
REVERSE LENGTH=306 | 201606   Arabidopsis_thaliana
OG0000009
AT5G47220.1 | ethylene responsive element binding factor 2 |
Chr5:19172023-19172754 REVERSE LENGTH=243 | 201606
Arabidopsis_thaliana   OG0000009
AT5G47230.1 | ethylene responsive element binding factor 5 |
Chr5:19180072-19180974 FORWARD LENGTH=300 | 201606
Arabidopsis_thaliana   OG0000009
AT5G50080.1 | ethylene response factor 110 | Chr5:20365948-20366835
FORWARD LENGTH=220 | 201606   Arabidopsis_thaliana
OG0000009
AT5G51190.1 | Integrase-type DNA-binding superfamily protein |
Chr5:20800708-20801373 REVERSE LENGTH=221 | 201606
Arabidopsis_thaliana   OG0000009
AT1G25470.1 | AP2 domain-containing transcription factor family
protein | Chr1:8944901-8945764 REVERSE LENGTH=287 | 201606
Arabidopsis_thaliana   OG0000009
AT5G51990.1 | C-repeat-binding factor 4 | Chr5:21117113-21117787
REVERSE LENGTH=224 | 201606   Arabidopsis_thaliana
OG0000009
AT5G52020.1 | Integrase-type DNA-binding superfamily protein |
Chr5:21124192-21124890 REVERSE LENGTH=232 | 201606
Arabidopsis_thaliana   OG0000009
AT5G53290.1 | cytokinin response factor 3 | Chr5:21617961-21619025
REVERSE LENGTH=354 | 201606   Arabidopsis_thaliana
OG0000009
AT5G61590.1 | Integrase-type DNA-binding superfamily protein |
Chr5:24764653-24765258 REVERSE LENGTH=201 | 201606
Arabidopsis_thaliana   OG0000009
AT5G61600.1 | ethylene response factor 104 | Chr5:24766643-24767368
REVERSE LENGTH=241 | 201606   Arabidopsis_thaliana
OG0000009
AT5G61890.1 | Integrase-type DNA-binding superfamily protein |
Chr5:24852639-24853744 REVERSE LENGTH=248 | 201606
Arabidopsis_thaliana   OG0000009
AT5G64750.1 | Integrase-type DNA-binding superfamily protein |
Chr5:25891679-25893656 FORWARD LENGTH=391 | 201606
Arabidopsis_thaliana   OG0000009
AT5G65130.1 | Integrase-type DNA-binding superfamily protein |
Chr5:26017403-26018236 FORWARD LENGTH=277 | 201606
Arabidopsis_thaliana   OG0000009
AT5G67000.1 | Integrase-type DNA-binding superfamily protein |
Chr5:26746677-26747231 REVERSE LENGTH=184 | 201606
Arabidopsis_thaliana   OG0000009
AT5G67010.1 | Integrase-type DNA-binding superfamily protein |
Chr5:26749058-26749616 REVERSE LENGTH=162 | 201606
Arabidopsis_thaliana   OG0000009
AT5G67190.1 | DREB and EAR motif protein 2 | Chr5:26809135-26809689
REVERSE LENGTH=184 | 201606   Arabidopsis_thaliana
OG0000009
AT1G28160.1 | Integrase-type DNA-binding superfamily protein |
Chr1:9839387-9840124 FORWARD LENGTH=245 | 201606

Arabidopsis_thaliana   OG0000009
AT1G28360.1 | ERF domain protein 12 | Chr1:9951934-9952503 FORWARD LENGTH=189 | 201606   Arabidopsis_thaliana   OG0000009
AT1G28370.1 | ERF domain protein 11 | Chr1:9956351-9956851 REVERSE LENGTH=166 | 201606   Arabidopsis_thaliana   OG0000009
AT1G03800.1 | ERF domain protein 10 | Chr1:957261-957998 REVERSE LENGTH=245 | 201606   Arabidopsis_thaliana   OG0000009
AT1G33760.1 | Integrase-type DNA-binding superfamily protein | Chr1:12237944-12238498 FORWARD LENGTH=184 | 201606
Arabidopsis_thaliana   OG0000009
AT1G04370.1 | Ethylene-responsive element binding factor 14 | Chr1:1175177-1175578 FORWARD LENGTH=133 | 201606
Arabidopsis_thaliana   OG0000009
AT1G36060.1 | Integrase-type DNA-binding superfamily protein | Chr1:13454718-13455662 REVERSE LENGTH=314 | 201606
Arabidopsis_thaliana   OG0000009
AT1G43160.1 | related to AP2 6 | Chr1:16263989-16264663 FORWARD LENGTH=192 | 201606   Arabidopsis_thaliana   OG0000009
AT1G44830.1 | Integrase-type DNA-binding superfamily protein | Chr1:16933792-16934427 FORWARD LENGTH=211 | 201606
Arabidopsis_thaliana   OG0000009
AT1G46768.1 | related to AP2 1 | Chr1:17266046-17266507 REVERSE LENGTH=153 | 201606   Arabidopsis_thaliana   OG0000009
AT1G49120.1 | Integrase-type DNA-binding superfamily protein | Chr1:18173427-18174416 FORWARD LENGTH=329 | 201606
Arabidopsis_thaliana   OG0000009
AT1G50640.1 | ethylene responsive element binding factor 3 | Chr1:18757602-18758279 REVERSE LENGTH=225 | 201606
Arabidopsis_thaliana   OG0000009
AT1G53170.1 | ethylene response factor 8 | Chr1:19821539-19822096 REVERSE LENGTH=185 | 201606   Arabidopsis_thaliana
OG0000009
AT1G53910.1 | related to AP2 12 | Chr1:20135242-20136581 FORWARD LENGTH=358 | 201606   Arabidopsis_thaliana   OG0000009
AT1G63030.1 | Integrase-type DNA-binding superfamily protein | Chr1:23367603-23368148 REVERSE LENGTH=181 | 201606
Arabidopsis_thaliana   OG0000009
AT1G64380.1 | Integrase-type DNA-binding superfamily protein | Chr1:23890981-23891988 REVERSE LENGTH=335 | 201606
Arabidopsis_thaliana   OG0000009
AT1G06160.1 | octadecanoid-responsive AP2/ERF 59 | Chr1:1883046-1883780 FORWARD LENGTH=244 | 201606
Arabidopsis_thaliana   OG0000009
AT1G68550.1 | Integrase-type DNA-binding superfamily protein | Chr1:25725810-25726784 REVERSE LENGTH=324 | 201606
Arabidopsis_thaliana   OG0000009
AT1G71130.1 | Integrase-type DNA-binding superfamily protein | Chr1:26822877-26823362 FORWARD LENGTH=161 | 201606
Arabidopsis_thaliana   OG0000009
AT1G71450.1 | Integrase-type DNA-binding superfamily protein | Chr1:26927088-26927639 FORWARD LENGTH=183 | 201606
Arabidopsis_thaliana   OG0000009
AT1G71520.1 | Integrase-type DNA-binding superfamily protein | Chr1:26938612-26939043 FORWARD LENGTH=143 | 201606

Arabidopsis_thaliana OG0000009
AT1G72360.1 | Integrase-type DNA-binding superfamily protein | Chr1:27242142-27242777 FORWARD LENGTH=211 | 201606 Arabidopsis_thaliana OG0000009
AT1G74930.1 | Integrase-type DNA-binding superfamily protein | Chr1:28144239-28144826 FORWARD LENGTH=195 | 201606 Arabidopsis_thaliana OG0000009
AT1G75490.1 | Integrase-type DNA-binding superfamily protein | Chr1:28336045-28336665 FORWARD LENGTH=206 | 201606 Arabidopsis_thaliana OG0000009
AT1G77200.1 | Integrase-type DNA-binding superfamily protein | Chr1:29004357-29005091 REVERSE LENGTH=244 | 201606 Arabidopsis_thaliana OG0000009
AT1G77640.1 | Integrase-type DNA-binding superfamily protein | Chr1:29178796-29179530 FORWARD LENGTH=244 | 201606 Arabidopsis_thaliana OG0000009
AT1G78080.1 | related to AP2 4 | Chr1:29364790-29365794 FORWARD LENGTH=334 | 201606 Arabidopsis_thaliana OG0000009
AT1G80580.1 | Integrase-type DNA-binding superfamily protein | Chr1:30293558-30294328 FORWARD LENGTH=256 | 201606 Arabidopsis_thaliana OG0000009
AT2G20350.1 | Integrase-type DNA-binding superfamily protein | Chr2:8784769-8785396 FORWARD LENGTH=158 | 201606 Arabidopsis_thaliana OG0000009
AT2G20880.1 | Integrase-type DNA-binding superfamily protein | Chr2:8986053-8987063 FORWARD LENGTH=336 | 201606 Arabidopsis_thaliana OG0000009
AT2G22200.1 | Integrase-type DNA-binding superfamily protein | Chr2:9443422-9444207 REVERSE LENGTH=261 | 201606 Arabidopsis_thaliana OG0000009
AT2G23340.1 | DREB and EAR motif protein 3 | Chr2:9938186-9938716 FORWARD LENGTH=176 | 201606 Arabidopsis_thaliana OG0000009
AT2G25820.1 | Integrase-type DNA-binding superfamily protein | Chr2:11014986-11015663 FORWARD LENGTH=225 | 201606 Arabidopsis_thaliana OG0000009
AT2G31230.1 | ethylene-responsive element binding factor 15 | Chr2:13306676-13307407 REVERSE LENGTH=243 | 201606 Arabidopsis_thaliana OG0000009
AT2G33710.1 | Integrase-type DNA-binding superfamily protein | Chr2:14259271-14260652 REVERSE LENGTH=218 | 201606 Arabidopsis_thaliana OG0000009
Os03t0263000-01 Similar to TINY-like protein. Oryza_sativa OG0000009
Os03t0341000-01 Similar to AP2 domain containing protein RAP2.2 (Fragment). Oryza_sativa OG0000009
Os03t0815800-01 Similar to Ethylene-responsive transcription factor 5 (Ethylene-responsive element binding factor 5) (EREBP-5) (AtERF5). Oryza_sativa OG0000009
Os03t0860100-01 Similar to Ethylene-responsive transcription factor 2 (Ethylene-responsive element binding factor 2) (EREBP-2) (AtERF2). Oryza_sativa OG0000009
Os04t0257500-01 Similar to Transcription factor TSRF1. Oryza_sativa OG0000009

Os04t0398000-01 Pathogenesis-related transcriptional factor and ERF domain containing protein.	Oryza_sativa	OG0000009
Os04t0399800-00 Similar to Ethylene-responsive transcription factor 12 (Ethylene-responsive element binding factor 12) (EREBP-12) (AtERF12).	Oryza_sativa	OG0000009
Os04t0429050-00 Similar to OSIGBa0093L02.1 protein.	Oryza_sativa	OG0000009
Os01t0313300-01 Similar to EREBP-3 protein (Fragment).	Oryza_sativa	OG0000009
Os04t0529100-01 Pathogenesis-related transcriptional factor and ERF domain containing protein.	Oryza_sativa	OG0000009
Os04t0546800-01 Pathogenesis-related transcriptional factor and ERF domain containing protein.	Oryza_sativa	OG0000009
Os04t0547600-01 Pathogenesis-related transcriptional factor and ERF domain containing protein.	Oryza_sativa	OG0000009
Os04t0549700-01 Similar to Dehydration responsive element binding protein 1F (DREB1F protein).	Oryza_sativa	OG0000009
Os04t0549800-00 Similar to OSIGBa0134H18.5 protein.	Oryza_sativa	OG0000009
Os04t0550200-01 Pathogenesis-related transcriptional factor and ERF domain containing protein.	Oryza_sativa	OG0000009
Os04t0572200-00 Similar to OSIGBa0127A14.7 protein.	Oryza_sativa	OG0000009
Os04t0572400-00 Similar to CRT/DRE binding factor 1.	Oryza_sativa	OG0000009
Os04t0610400-01 APETALA-2-like transcription Factor, Control of the abscisic acid (ABA)/gibberellin (GA) balance	Oryza_sativa	OG0000009
Os04t0648900-01 Similar to Dehydration responsive element binding protein 2F (DREB2F protein).	Oryza_sativa	OG0000009
Os04t0655700-00 Similar to OSIGBa0147J19.14 protein.	Oryza_sativa	OG0000009
Os04t0669200-01 Similar to Ethylene response factor 2 (Ethylene response factor 3).	Oryza_sativa	OG0000009
Os05t0316800-01 Similar to Ethylene-responsive transcription factor 9 (Ethylene-responsive element binding factor 9) (EREBP-9) (AtERF9).	Oryza_sativa	OG0000009
Os05t0351200-00 Similar to AP2 domain transcription factor.	Oryza_sativa	OG0000009
Os05t0361700-01 Similar to EREBP-2 protein (Fragment).	Oryza_sativa	OG0000009
Os05t0420300-01 Salt- and H2O2- responsive transcription factor, Salt tolerance	Oryza_sativa	OG0000009
Os05t0437100-00 Pathogenesis-related transcriptional factor and ERF domain containing protein.	Oryza_sativa	OG0000009
Os05t0473300-01 Hypothetical conserved gene.	Oryza_sativa	OG0000009
Os05t0497200-01 AP2/ERF (APETALA2/ethylene-responsive factor) protein, Regulation of spikelet meristem determinancy and floral organ identity	Oryza_sativa	OG0000009
Os05t0497300-01 Similar to Ethylene response factor 2.	Oryza_sativa	OG0000009
Os05t0572000-00 Pathogenesis-related transcriptional factor and ERF domain containing protein.	Oryza_sativa	OG0000009

Os06t0127100-01 Dehydration-responsive element-binding protein 1C. Oryza_sativa OG0000009
Os06t0165600-01 CRT/DRE binding factor (Transcription factor RCBF4). Oryza_sativa OG0000009
Os06t0166400-01 Similar to TINY-like protein (AP2 domain containing protein RAP2.10) (Fragment). Oryza_sativa OG0000009
Os06t0181700-01 Pathogenesis-related transcriptional factor and ERF domain containing protein. Oryza_sativa OG0000009
Os06t0194000-01 Similar to Ethylene-responsive transcription factor 3 (Ethylene-responsive element binding factor 3 homolog) (EREBP-5) (NtERF5). Oryza_sativa OG0000009
Os06t0604000-01 Similar to Ethylene response factor 1. Oryza_sativa OG0000009
Os06t0691100-00 Pathogenesis-related transcriptional factor and ERF domain containing protein. Oryza_sativa OG0000009
Os07t0204000-00 Pathogenesis-related transcriptional factor/ERF, DNA-binding domain containing protein. Oryza_sativa OG0000009
Os07t0227600-00 Pathogenesis-related transcriptional factor/ERF, DNA-binding domain containing protein. Oryza_sativa OG0000009
Os07t0410700-01 Similar to Ethylene-responsive element binding protein 1. Oryza_sativa OG0000009
Os07t0575000-00 Pathogenesis-related transcriptional factor/ERF, DNA-binding domain containing protein. Oryza_sativa OG0000009
Os07t0617000-01 Similar to Ethylene response factor 2. Oryza_sativa OG0000009
Os07t0669500-01 Similar to Branched silkless1. Oryza_sativa OG0000009
Os07t0674800-01 Similar to AP2 domain transcription factor EREBP. Oryza_sativa OG0000009
Os08t0173700-01 Similar to Ethylene-responsive transcription factor 4 (Ethylene-responsive element binding factor 4) (Related to APETALA-2 protein 5) (EREBP-4) (AtERF4). Oryza_sativa OG0000009
Os08t0408500-01 Pathogenesis-related transcriptional factor and ERF domain containing protein. Oryza_sativa OG0000009
Os08t0454000-01 Drought-responsive ethylene response factor, Transcriptional activator, Drought response through controlling the ethylene synthesis Oryza_sativa OG0000009
Os01t0657400-00 Similar to Ethylene-responsive transcription factor 5 (Ethylene-responsive element binding factor 5) (EREBP-5) (AtERF5). Oryza_sativa OG0000009
Os08t0474000-01 Similar to AP2 domain containing protein RAP2.6 (Fragment). Oryza_sativa OG0000009
Os08t0521600-01 Similar to Dehydration responsive element binding protein 2C (DREB2C protein). Oryza_sativa OG0000009
Os08t0565200-01 Pathogenesis-related transcriptional factor and ERF domain containing protein. Oryza_sativa OG0000009
Os09t0286600-01 Pathogenesis-related transcriptional factor and ERF domain containing protein. Oryza_sativa OG0000009
Os09t0287000-01 Similar to Ethylene-responsive transcription factor 5 (Ethylene-responsive element binding factor 5) (EREBP-5) (AtERF5).

Oryza_sativa OG0000009
Os09t0309700-01 Similar to Transcriptional factor TINY. Oryza_sativa OG0000009
Os09t0434500-01 Similar to Ethylene response factor 2. Oryza_sativa OG0000009
Os09t0457900-01 AP2/ERF transcription factor, Regulation of the internode elongation Oryza_sativa OG0000009
Os09t0522100-00 Similar to Dehydration-responsive element-binding protein 1H. Oryza_sativa OG0000009
Os09t0522200-01 DRE-binding protein 1A. Oryza_sativa OG0000009
Os09t0571700-00 Similar to Ethylene-responsive transcription factor ERF096. Oryza_sativa OG0000009
Os09t0572000-01 Pathogenesis-related transcriptional factor and ERF domain containing protein. Oryza_sativa OG0000009
Os10t0371100-00 Similar to AP2 domain containing protein, expressed. Oryza_sativa OG0000009
Os10t0390800-01 Similar to Ethylene-responsive transcription factor 3 (Ethylene-responsive element binding factor 3) (EREBP-3) (AtERF3). Oryza_sativa OG0000009
Os10t0523900-00 Similar to AP2 domain containing protein. Oryza_sativa OG0000009
Os11t0168500-00 Hypothetical conserved gene. Oryza_sativa OG0000009
Os11t0242300-00 Similar to AP2 domain containing protein, expressed. Oryza_sativa OG0000009
Os01t0752500-00 Hypothetical conserved gene. Oryza_sativa OG0000009
Os12t0168100-01 Similar to AP2 domain containing protein RAP2.6 (Fragment). Oryza_sativa OG0000009
Os12t0582900-01 Similar to AP2 domain containing protein, expressed. Oryza_sativa OG0000009
Os12t0603300-01 Similar to AP2 domain containing protein, expressed. Oryza_sativa OG0000009
Os01t0797600-01 AP2/ERF family protein, ERF-associated EAR-motif-containing repressor, Abiotic stress response, Stress signaling Oryza_sativa OG0000009
Os01t0868000-01 AP2/ERF transcription factor, Abiotic stress response, Salt stress response Oryza_sativa OG0000009
Os01t0885900-00 Similar to transcriptional factor TINY. Oryza_sativa OG0000009
Os01t0968800-00 Similar to Dehydration responsive element binding protein 1F (DREB1F protein). Oryza_sativa OG0000009
Os01t0165000-01 DRE binding protein 2. Oryza_sativa OG0000009
Os02t0202000-01 Ethylene response factor, Transcription factor, Regulation of wax synthesis, Drought tolerance Oryza_sativa OG0000009
Os02t0520000-00 Similar to OSIGBa0113L04.1 protein. Oryza_sativa OG0000009
Os02t0546600-01 Similar to AP2 domain containing protein RAP2.2 (Fragment). Oryza_sativa OG0000009
Os02t0558700-00 Similar to dehydration-responsive element-binding protein 1D. Oryza_sativa OG0000009

Os02t0594300-00 Similar to Enhancer of shoot regeneration ESR1. Oryza_sativa OG0000009
Os02t0638650-01 Pathogenesis-related transcriptional factor and ERF domain containing protein. Oryza_sativa OG0000009
Os02t0654700-01 AP2/ERF family protein, Abiotic stress response Oryza_sativa OG0000009
Os02t0656600-00 Similar to Dehydration responsive element binding protein 2B (DREB2B protein). Oryza_sativa OG0000009
Os02t0657000-01 AP2 domain-containing protein Rap211. Oryza_sativa OG0000009
Os01t0200600-01 Similar to Avr9/Cf-9 rapidly elicited protein 111A. Oryza_sativa OG0000009
Os02t0676800-01 Similar to Dehydration responsive element binding protein 1E (DREB1E protein). Oryza_sativa OG0000009
Os02t0677300-02 Similar to CRT/DRE binding factor 1. Oryza_sativa OG0000009
Os02t0712400-01 Similar to H0721B11.1 protein. Oryza_sativa OG0000009
Os02t0752800-01 Similar to Dehydration responsive element binding protein 2B (DREB2B protein). Oryza_sativa OG0000009
Os02t0764700-01 Similar to Ethylene-responsive transcription factor 4 (Ethylene-responsive element binding factor 4) (Related to APETALA-2 protein 5) (EREBP-4) (AtERF4). Oryza_sativa OG0000009
Os02t0767800-00 Similar to DNA binding protein. Oryza_sativa OG0000009
Os02t0781300-00 Similar to AP2/ERF domain-containing transcription factor. Oryza_sativa OG0000009
Os02t0782700-01 AP2/ERF transcription factor, Defense response, Abiotic stress response Oryza_sativa OG0000009
Os02t0797100-00 Similar to Dehydration responsive element binding protein 2B (DREB2B protein). Oryza_sativa OG0000009
Os03t0117900-01 Similar to Transcription factor. Oryza_sativa OG0000009
Os03t0150200-00 Similar to Ethylene-responsive transcription factor 5 (Ethylene-responsive element binding factor 5 homolog) (EREBP-4) (NtERF4). Oryza_sativa OG0000009
Os01t0224100-01 Similar to DNA binding protein-like protein. Oryza_sativa OG0000009
Os03t0174400-00 Hypothetical conserved gene. Oryza_sativa OG0000009
Os03t0182800-01 Similar to Ethylene responsive element binding factor3 (OsERF3). Oryza_sativa OG0000009
Os03t0183000-01 Similar to AP2 domain containing protein RAP2.6 (Fragment). Oryza_sativa OG0000009
Os03t0183300-01 Pathogenesis-related transcriptional factor and ERF domain containing protein. Oryza_sativa OG0000009
Os03t0191900-01 Pathogenesis-related transcriptional factor and ERF domain containing protein. Oryza_sativa OG0000009
Cre01.g003800.t1.1 pacid=30788915 transcript=Cre01.g003800.t1.1 locus=Cre01.g003800 ID=Cre01.g003800.t1.1.v5.5 annot-version=v5.5 Chlamydomonas_reinhardtii OG0000010
Cre12.g504250.t1.1 pacid=30792603 transcript=Cre12.g504250.t1.1 locus=Cre12.g504250 ID=Cre12.g504250.t1.1.v5.5 annot-version=v5.5

Chlamydomonas_reinhardtii OG0000010
Cre12.g490800.t1.1 pacid=30792819 transcript=Cre12.g490800.t1.1
locus=Cre12.g490800 ID=Cre12.g490800.t1.1.v5.5 annot-version=v5.5
Chlamydomonas_reinhardtii OG0000010
Cre13.g579650.t1.1 pacid=30784485 transcript=Cre13.g579650.t1.1
locus=Cre13.g579650 ID=Cre13.g579650.t1.1.v5.5 annot-version=v5.5
Chlamydomonas_reinhardtii OG0000010
jgi|ChlNC64A_1|132911|IGS.gm_1_00047 Chlorella_variabilis
OG0000010
66756 pacid=27391991 transcript=66756
locus=estExt_Genemark1.C_110214 ID=66756.2.0.227 annot-version=v2.0
Coccomyxa_subellipsoidea OG0000010
81229 pacid=27400036 transcript=81229 locus=e_gw2.04.468.1
ID=81229.3.0.229 annot-version=v3.0 Micromonas_sp._RCC299
OG0000010
kfl00019_g38_v1.1 Klebsormidium_flaccidum OG0000010
kfl00596_0070_v1.1 Klebsormidium_flaccidum OG0000010
kfl00620_0020_v1.1 Klebsormidium_flaccidum OG0000010
kfl00620_0060_v1.1 Klebsormidium_flaccidum OG0000010
kfl00780_0020_v1.1 Klebsormidium_flaccidum OG0000010
kfl01028_0010_v1.1 Klebsormidium_flaccidum OG0000010
kfl00102_g22_v1.1 Klebsormidium_flaccidum OG0000010
kfl00260_0050_v1.1 Klebsormidium_flaccidum OG0000010
kfl00013_0240_v1.1 Klebsormidium_flaccidum OG0000010
Pp3c7_20990V3.1.p pacid=32926017 transcript=Pp3c7_20990V3.1
locus=Pp3c7_20990 annot-version=v3.3 Physcomitrella_patens
OG0000010
Pp3c7_4130V3.1.p pacid=32926109 transcript=Pp3c7_4130V3.1
locus=Pp3c7_4130 annot-version=v3.3 Physcomitrella_patens
OG0000010
Pp3c7_21890V3.1.p pacid=32926134 transcript=Pp3c7_21890V3.1
locus=Pp3c7_21890 annot-version=v3.3 Physcomitrella_patens
OG0000010
Pp3c7_4420V3.1.p pacid=32926288 transcript=Pp3c7_4420V3.1
locus=Pp3c7_4420 annot-version=v3.3 Physcomitrella_patens
OG0000010
Pp3c7_21110V3.1.p pacid=32926339 transcript=Pp3c7_21110V3.1
locus=Pp3c7_21110 annot-version=v3.3 Physcomitrella_patens
OG0000010
Pp3c15_23820V3.1.p pacid=32927011 transcript=Pp3c15_23820V3.1
locus=Pp3c15_23820 annot-version=v3.3 Physcomitrella_patens
OG0000010
Pp3c15_16800V3.1.p pacid=32927215 transcript=Pp3c15_16800V3.1
locus=Pp3c15_16800 annot-version=v3.3 Physcomitrella_patens
OG0000010
Pp3c15_2320V3.1.p pacid=32927310 transcript=Pp3c15_2320V3.1
locus=Pp3c15_2320 annot-version=v3.3 Physcomitrella_patens
OG0000010
Pp3c15_18790V3.1.p pacid=32927476 transcript=Pp3c15_18790V3.1
locus=Pp3c15_18790 annot-version=v3.3 Physcomitrella_patens
OG0000010
Pp3c15_2310V3.1.p pacid=32927540 transcript=Pp3c15_2310V3.1
locus=Pp3c15_2310 annot-version=v3.3 Physcomitrella_patens
OG0000010

Pp3c15_23710V3.1.p pacid=32927567 transcript=Pp3c15_23710V3.1 locus=Pp3c15_23710 annot-version=v3.3 Physcomitrella_patens OG0000010
Pp3c15_4320V3.1.p pacid=32927695 transcript=Pp3c15_4320V3.1 locus=Pp3c15_4320 annot-version=v3.3 Physcomitrella_patens OG0000010
Pp3c15_2330V3.1.p pacid=32928232 transcript=Pp3c15_2330V3.1 locus=Pp3c15_2330 annot-version=v3.3 Physcomitrella_patens OG0000010
Pp3c15_4300V3.1.p pacid=32928319 transcript=Pp3c15_4300V3.1 locus=Pp3c15_4300 annot-version=v3.3 Physcomitrella_patens OG0000010
Pp3c15_16820V3.1.p pacid=32928471 transcript=Pp3c15_16820V3.1 locus=Pp3c15_16820 annot-version=v3.3 Physcomitrella_patens OG0000010
Pp3c15_16810V3.1.p pacid=32928608 transcript=Pp3c15_16810V3.1 locus=Pp3c15_16810 annot-version=v3.3 Physcomitrella_patens OG0000010
Pp3c10_20690V3.1.p pacid=32902377 transcript=Pp3c10_20690V3.1 locus=Pp3c10_20690 annot-version=v3.3 Physcomitrella_patens OG0000010
Pp3c15_2290V3.1.p pacid=32928649 transcript=Pp3c15_2290V3.1 locus=Pp3c15_2290 annot-version=v3.3 Physcomitrella_patens OG0000010
Pp3c10_16940V3.1.p pacid=32902385 transcript=Pp3c10_16940V3.1 locus=Pp3c10_16940 annot-version=v3.3 Physcomitrella_patens OG0000010
Pp3c15_14470V3.1.p pacid=32928701 transcript=Pp3c15_14470V3.1 locus=Pp3c15_14470 annot-version=v3.3 Physcomitrella_patens OG0000010
Pp3c10_7350V3.1.p pacid=32902394 transcript=Pp3c10_7350V3.1 locus=Pp3c10_7350 annot-version=v3.3 Physcomitrella_patens OG0000010
Pp3c15_950V3.1.p pacid=32929019 transcript=Pp3c15_950V3.1 locus=Pp3c15_950 annot-version=v3.3 Physcomitrella_patens OG0000010
Pp3c15_960V3.1.p pacid=32929167 transcript=Pp3c15_960V3.1 locus=Pp3c15_960 annot-version=v3.3 Physcomitrella_patens OG0000010
Pp3c15_21370V3.1.p pacid=32929745 transcript=Pp3c15_21370V3.1 locus=Pp3c15_21370 annot-version=v3.3 Physcomitrella_patens OG0000010
Pp3s876_30V3.1.p pacid=32929804 transcript=Pp3s876_30V3.1 locus=Pp3s876_30 annot-version=v3.3 Physcomitrella_patens OG0000010
Pp3c13_2920V3.1.p pacid=32929886 transcript=Pp3c13_2920V3.1 locus=Pp3c13_2920 annot-version=v3.3 Physcomitrella_patens OG0000010
Pp3c13_16410V3.1.p pacid=32929890 transcript=Pp3c13_16410V3.1 locus=Pp3c13_16410 annot-version=v3.3 Physcomitrella_patens OG0000010
Pp3c13_22120V3.1.p pacid=32930112 transcript=Pp3c13_22120V3.1 locus=Pp3c13_22120 annot-version=v3.3 Physcomitrella_patens OG0000010

Pp3c13_16400V3.1.p pacid=32930150 transcript=Pp3c13_16400V3.1 locus=Pp3c13_16400 annot-version=v3.3 Physcomitrella_patens OG0000010
Pp3c13_2950V3.1.p pacid=32930162 transcript=Pp3c13_2950V3.1 locus=Pp3c13_2950 annot-version=v3.3 Physcomitrella_patens OG0000010
Pp3c13_3360V3.1.p pacid=32930199 transcript=Pp3c13_3360V3.1 locus=Pp3c13_3360 annot-version=v3.3 Physcomitrella_patens OG0000010
Pp3c13_3190V3.1.p pacid=32930286 transcript=Pp3c13_3190V3.1 locus=Pp3c13_3190 annot-version=v3.3 Physcomitrella_patens OG0000010
Pp3c13_19900V3.1.p pacid=32930326 transcript=Pp3c13_19900V3.1 locus=Pp3c13_19900 annot-version=v3.3 Physcomitrella_patens OG0000010
Pp3c13_15270V3.1.p pacid=32930355 transcript=Pp3c13_15270V3.1 locus=Pp3c13_15270 annot-version=v3.3 Physcomitrella_patens OG0000010
Pp3c13_19910V3.1.p pacid=32930437 transcript=Pp3c13_19910V3.1 locus=Pp3c13_19910 annot-version=v3.3 Physcomitrella_patens OG0000010
Pp3c13_21800V3.1.p pacid=32930537 transcript=Pp3c13_21800V3.1 locus=Pp3c13_21800 annot-version=v3.3 Physcomitrella_patens OG0000010
Pp3c13_21690V3.1.p pacid=32930565 transcript=Pp3c13_21690V3.1 locus=Pp3c13_21690 annot-version=v3.3 Physcomitrella_patens OG0000010
Pp3c13_2470V3.1.p pacid=32930569 transcript=Pp3c13_2470V3.1 locus=Pp3c13_2470 annot-version=v3.3 Physcomitrella_patens OG0000010
Pp3c13_2820V3.1.p pacid=32930727 transcript=Pp3c13_2820V3.1 locus=Pp3c13_2820 annot-version=v3.3 Physcomitrella_patens OG0000010
Pp3c13_15190V3.1.p pacid=32930748 transcript=Pp3c13_15190V3.1 locus=Pp3c13_15190 annot-version=v3.3 Physcomitrella_patens OG0000010
Pp3c13_1180V3.1.p pacid=32930779 transcript=Pp3c13_1180V3.1 locus=Pp3c13_1180 annot-version=v3.3 Physcomitrella_patens OG0000010
Pp3c10_3630V3.1.p pacid=32902632 transcript=Pp3c10_3630V3.1 locus=Pp3c10_3630 annot-version=v3.3 Physcomitrella_patens OG0000010
Pp3c13_21080V3.1.p pacid=32930836 transcript=Pp3c13_21080V3.1 locus=Pp3c13_21080 annot-version=v3.3 Physcomitrella_patens OG0000010
Pp3c13_21810V3.1.p pacid=32931258 transcript=Pp3c13_21810V3.1 locus=Pp3c13_21810 annot-version=v3.3 Physcomitrella_patens OG0000010
Pp3c13_1250V3.1.p pacid=32931291 transcript=Pp3c13_1250V3.1 locus=Pp3c13_1250 annot-version=v3.3 Physcomitrella_patens OG0000010
Pp3c13_1691V3.1.p pacid=32931322 transcript=Pp3c13_1691V3.1 locus=Pp3c13_1691 annot-version=v3.3 Physcomitrella_patens OG0000010

Pp3c13_16690V3.1.p pacid=32931349 transcript=Pp3c13_16690V3.1 locus=Pp3c13_16690 annot-version=v3.3 Physcomitrella_patens OG0000010
Pp3c13_2610V3.1.p pacid=32931405 transcript=Pp3c13_2610V3.1 locus=Pp3c13_2610 annot-version=v3.3 Physcomitrella_patens OG0000010
Pp3c13_21710V3.1.p pacid=32931449 transcript=Pp3c13_21710V3.1 locus=Pp3c13_21710 annot-version=v3.3 Physcomitrella_patens OG0000010
Pp3c13_1240V3.1.p pacid=32931469 transcript=Pp3c13_1240V3.1 locus=Pp3c13_1240 annot-version=v3.3 Physcomitrella_patens OG0000010
Pp3c13_21820V3.1.p pacid=32931613 transcript=Pp3c13_21820V3.1 locus=Pp3c13_21820 annot-version=v3.3 Physcomitrella_patens OG0000010
Pp3c13_2910V3.1.p pacid=32931622 transcript=Pp3c13_2910V3.1 locus=Pp3c13_2910 annot-version=v3.3 Physcomitrella_patens OG0000010
Pp3c13_3110V3.1.p pacid=32931641 transcript=Pp3c13_3110V3.1 locus=Pp3c13_3110 annot-version=v3.3 Physcomitrella_patens OG0000010
Pp3c13_17850V3.1.p pacid=32931673 transcript=Pp3c13_17850V3.1 locus=Pp3c13_17850 annot-version=v3.3 Physcomitrella_patens OG0000010
Pp3c13_22110V3.1.p pacid=32931817 transcript=Pp3c13_22110V3.1 locus=Pp3c13_22110 annot-version=v3.3 Physcomitrella_patens OG0000010
Pp3c13_2490V3.1.p pacid=32931836 transcript=Pp3c13_2490V3.1 locus=Pp3c13_2490 annot-version=v3.3 Physcomitrella_patens OG0000010
Pp3c13_21290V3.1.p pacid=32931882 transcript=Pp3c13_21290V3.1 locus=Pp3c13_21290 annot-version=v3.3 Physcomitrella_patens OG0000010
Pp3c13_2510V3.1.p pacid=32931938 transcript=Pp3c13_2510V3.1 locus=Pp3c13_2510 annot-version=v3.3 Physcomitrella_patens OG0000010
Pp3c13_2810V3.1.p pacid=32932035 transcript=Pp3c13_2810V3.1 locus=Pp3c13_2810 annot-version=v3.3 Physcomitrella_patens OG0000010
Pp3c13_16480V3.1.p pacid=32932152 transcript=Pp3c13_16480V3.1 locus=Pp3c13_16480 annot-version=v3.3 Physcomitrella_patens OG0000010
Pp3c13_22130V3.1.p pacid=32932243 transcript=Pp3c13_22130V3.1 locus=Pp3c13_22130 annot-version=v3.3 Physcomitrella_patens OG0000010
Pp3c13_2730V3.1.p pacid=32932267 transcript=Pp3c13_2730V3.1 locus=Pp3c13_2730 annot-version=v3.3 Physcomitrella_patens OG0000010
Pp3c13_2900V3.1.p pacid=32932272 transcript=Pp3c13_2900V3.1 locus=Pp3c13_2900 annot-version=v3.3 Physcomitrella_patens OG0000010
Pp3c13_19890V3.1.p pacid=32932481 transcript=Pp3c13_19890V3.1 locus=Pp3c13_19890 annot-version=v3.3 Physcomitrella_patens OG0000010

Pp3c13_3150V3.1.p pacid=32932508 transcript=Pp3c13_3150V3.1
locus=Pp3c13_3150 annot-version=v3.3 Physcomitrella_patens
OG0000010
Pp3c13_15200V3.1.p pacid=32932518 transcript=Pp3c13_15200V3.1
locus=Pp3c13_15200 annot-version=v3.3 Physcomitrella_patens
OG0000010
Pp3c13_2500V3.1.p pacid=32932576 transcript=Pp3c13_2500V3.1
locus=Pp3c13_2500 annot-version=v3.3 Physcomitrella_patens
OG0000010
Pp3c2_13130V3.1.p pacid=32932924 transcript=Pp3c2_13130V3.1
locus=Pp3c2_13130 annot-version=v3.3 Physcomitrella_patens
OG0000010
Pp3c22_3640V3.1.p pacid=32902935 transcript=Pp3c22_3640V3.1
locus=Pp3c22_3640 annot-version=v3.3 Physcomitrella_patens
OG0000010
Pp3c2_17170V3.1.p pacid=32934080 transcript=Pp3c2_17170V3.1
locus=Pp3c2_17170 annot-version=v3.3 Physcomitrella_patens
OG0000010
Pp3c2_12950V3.1.p pacid=32934538 transcript=Pp3c2_12950V3.1
locus=Pp3c2_12950 annot-version=v3.3 Physcomitrella_patens
OG0000010
Pp3c2_30210V3.1.p pacid=32934660 transcript=Pp3c2_30210V3.1
locus=Pp3c2_30210 annot-version=v3.3 Physcomitrella_patens
OG0000010
Pp3c2_31070V3.1.p pacid=32934747 transcript=Pp3c2_31070V3.1
locus=Pp3c2_31070 annot-version=v3.3 Physcomitrella_patens
OG0000010
Pp3c2_10550V3.1.p pacid=32934798 transcript=Pp3c2_10550V3.1
locus=Pp3c2_10550 annot-version=v3.3 Physcomitrella_patens
OG0000010
Pp3c2_12960V3.1.p pacid=32934799 transcript=Pp3c2_12960V3.1
locus=Pp3c2_12960 annot-version=v3.3 Physcomitrella_patens
OG0000010
Pp3c2_13100V3.1.p pacid=32934907 transcript=Pp3c2_13100V3.1
locus=Pp3c2_13100 annot-version=v3.3 Physcomitrella_patens
OG0000010
Pp3c2_13120V3.1.p pacid=32935105 transcript=Pp3c2_13120V3.1
locus=Pp3c2_13120 annot-version=v3.3 Physcomitrella_patens
OG0000010
Pp3c2_31072V3.1.p pacid=32935223 transcript=Pp3c2_31072V3.1
locus=Pp3c2_31072 annot-version=v3.3 Physcomitrella_patens
OG0000010
Pp3c10_9503V3.1.p pacid=32899729 transcript=Pp3c10_9503V3.1
locus=Pp3c10_9503 annot-version=v3.3 Physcomitrella_patens
OG0000010
Pp3c2_12940V3.1.p pacid=32935673 transcript=Pp3c2_12940V3.1
locus=Pp3c2_12940 annot-version=v3.3 Physcomitrella_patens
OG0000010
Pp3c2_29660V3.1.p pacid=32935800 transcript=Pp3c2_29660V3.1
locus=Pp3c2_29660 annot-version=v3.3 Physcomitrella_patens
OG0000010
Pp3c2_26150V3.1.p pacid=32935856 transcript=Pp3c2_26150V3.1
locus=Pp3c2_26150 annot-version=v3.3 Physcomitrella_patens
OG0000010

Pp3c2_11990V3.1.p pacid=32936285 transcript=Pp3c2_11990V3.1
locus=Pp3c2_11990 annot-version=v3.3 Physcomitrella_patens
OG0000010
Pp3c2_26140V3.1.p pacid=32936485 transcript=Pp3c2_26140V3.1
locus=Pp3c2_26140 annot-version=v3.3 Physcomitrella_patens
OG0000010
Pp3c19_14790V3.1.p pacid=32938168 transcript=Pp3c19_14790V3.1
locus=Pp3c19_14790 annot-version=v3.3 Physcomitrella_patens
OG0000010
Pp3c19_10560V3.1.p pacid=32938720 transcript=Pp3c19_10560V3.1
locus=Pp3c19_10560 annot-version=v3.3 Physcomitrella_patens
OG0000010
Pp3c19_3620V3.1.p pacid=32939422 transcript=Pp3c19_3620V3.1
locus=Pp3c19_3620 annot-version=v3.3 Physcomitrella_patens
OG0000010
Pp3c3_15550V3.1.p pacid=32940421 transcript=Pp3c3_15550V3.1
locus=Pp3c3_15550 annot-version=v3.3 Physcomitrella_patens
OG0000010
Pp3c3_30870V3.1.p pacid=32940524 transcript=Pp3c3_30870V3.1
locus=Pp3c3_30870 annot-version=v3.3 Physcomitrella_patens
OG0000010
Pp3c3_11530V3.1.p pacid=32940760 transcript=Pp3c3_11530V3.1
locus=Pp3c3_11530 annot-version=v3.3 Physcomitrella_patens
OG0000010
Pp3c3_12150V3.1.p pacid=32941397 transcript=Pp3c3_12150V3.1
locus=Pp3c3_12150 annot-version=v3.3 Physcomitrella_patens
OG0000010
Pp3c3_17880V3.1.p pacid=32942134 transcript=Pp3c3_17880V3.1
locus=Pp3c3_17880 annot-version=v3.3 Physcomitrella_patens
OG0000010
Pp3c3_21800V3.1.p pacid=32942286 transcript=Pp3c3_21800V3.1
locus=Pp3c3_21800 annot-version=v3.3 Physcomitrella_patens
OG0000010
Pp3c3_12140V3.1.p pacid=32942731 transcript=Pp3c3_12140V3.1
locus=Pp3c3_12140 annot-version=v3.3 Physcomitrella_patens
OG0000010
Pp3c3_30860V3.1.p pacid=32943208 transcript=Pp3c3_30860V3.1
locus=Pp3c3_30860 annot-version=v3.3 Physcomitrella_patens
OG0000010
Pp3c3_22790V3.1.p pacid=32943837 transcript=Pp3c3_22790V3.1
locus=Pp3c3_22790 annot-version=v3.3 Physcomitrella_patens
OG0000010
Pp3c3_30880V3.1.p pacid=32944233 transcript=Pp3c3_30880V3.1
locus=Pp3c3_30880 annot-version=v3.3 Physcomitrella_patens
OG0000010
Pp3c3_1792V3.1.p pacid=32944452 transcript=Pp3c3_1792V3.1
locus=Pp3c3_1792 annot-version=v3.3 Physcomitrella_patens
OG0000010
Pp3c22_14890V3.1.p pacid=32904014 transcript=Pp3c22_14890V3.1
locus=Pp3c22_14890 annot-version=v3.3 Physcomitrella_patens
OG0000010
Pp3c20_22230V3.1.p pacid=32945209 transcript=Pp3c20_22230V3.1
locus=Pp3c20_22230 annot-version=v3.3 Physcomitrella_patens
OG0000010

Pp3c20_22240V3.1.p pacid=32945333 transcript=Pp3c20_22240V3.1 locus=Pp3c20_22240 annot-version=v3.3 Physcomitrella_patens OG0000010
Pp3c20_15847V3.1.p pacid=32945597 transcript=Pp3c20_15847V3.1 locus=Pp3c20_15847 annot-version=v3.3 Physcomitrella_patens OG0000010
Pp3c20_7960V3.1.p pacid=32945609 transcript=Pp3c20_7960V3.1 locus=Pp3c20_7960 annot-version=v3.3 Physcomitrella_patens OG0000010
Pp3c20_20760V3.1.p pacid=32945697 transcript=Pp3c20_20760V3.1 locus=Pp3c20_20760 annot-version=v3.3 Physcomitrella_patens OG0000010
Pp3c20_15920V3.1.p pacid=32945724 transcript=Pp3c20_15920V3.1 locus=Pp3c20_15920 annot-version=v3.3 Physcomitrella_patens OG0000010
Pp3c20_10290V3.1.p pacid=32945754 transcript=Pp3c20_10290V3.1 locus=Pp3c20_10290 annot-version=v3.3 Physcomitrella_patens OG0000010
Pp3c20_4110V3.1.p pacid=32945924 transcript=Pp3c20_4110V3.1 locus=Pp3c20_4110 annot-version=v3.3 Physcomitrella_patens OG0000010
Pp3c20_16100V3.1.p pacid=32946052 transcript=Pp3c20_16100V3.1 locus=Pp3c20_16100 annot-version=v3.3 Physcomitrella_patens OG0000010
Pp3c20_23190V3.1.p pacid=32946168 transcript=Pp3c20_23190V3.1 locus=Pp3c20_23190 annot-version=v3.3 Physcomitrella_patens OG0000010
Pp3c20_21220V3.1.p pacid=32946194 transcript=Pp3c20_21220V3.1 locus=Pp3c20_21220 annot-version=v3.3 Physcomitrella_patens OG0000010
Pp3c20_17670V3.1.p pacid=32946403 transcript=Pp3c20_17670V3.1 locus=Pp3c20_17670 annot-version=v3.3 Physcomitrella_patens OG0000010
Pp3c20_15960V3.1.p pacid=32946453 transcript=Pp3c20_15960V3.1 locus=Pp3c20_15960 annot-version=v3.3 Physcomitrella_patens OG0000010
Pp3c20_22850V3.1.p pacid=32946540 transcript=Pp3c20_22850V3.1 locus=Pp3c20_22850 annot-version=v3.3 Physcomitrella_patens OG0000010
Pp3c20_12310V3.1.p pacid=32946594 transcript=Pp3c20_12310V3.1 locus=Pp3c20_12310 annot-version=v3.3 Physcomitrella_patens OG0000010
Pp3c20_1300V3.1.p pacid=32946604 transcript=Pp3c20_1300V3.1 locus=Pp3c20_1300 annot-version=v3.3 Physcomitrella_patens OG0000010
Pp3c20_4130V3.1.p pacid=32946720 transcript=Pp3c20_4130V3.1 locus=Pp3c20_4130 annot-version=v3.3 Physcomitrella_patens OG0000010
Pp3c20_8580V3.1.p pacid=32946838 transcript=Pp3c20_8580V3.1 locus=Pp3c20_8580 annot-version=v3.3 Physcomitrella_patens OG0000010
Pp3c20_22019V3.1.p pacid=32946850 transcript=Pp3c20_22019V3.1 locus=Pp3c20_22019 annot-version=v3.3 Physcomitrella_patens OG0000010

Pp3c20_15954V3.1.p pacid=32946915 transcript=Pp3c20_15954V3.1 locus=Pp3c20_15954 annot-version=v3.3 Physcomitrella_patens OG0000010
Pp3c20_8700V3.1.p pacid=32946981 transcript=Pp3c20_8700V3.1 locus=Pp3c20_8700 annot-version=v3.3 Physcomitrella_patens OG0000010
Pp3c20_3010V3.1.p pacid=32947267 transcript=Pp3c20_3010V3.1 locus=Pp3c20_3010 annot-version=v3.3 Physcomitrella_patens OG0000010
Pp3c20_2990V3.1.p pacid=32947296 transcript=Pp3c20_2990V3.1 locus=Pp3c20_2990 annot-version=v3.3 Physcomitrella_patens OG0000010
Pp3c20_17680V3.1.p pacid=32947330 transcript=Pp3c20_17680V3.1 locus=Pp3c20_17680 annot-version=v3.3 Physcomitrella_patens OG0000010
Pp3c20_21210V3.1.p pacid=32947454 transcript=Pp3c20_21210V3.1 locus=Pp3c20_21210 annot-version=v3.3 Physcomitrella_patens OG0000010
Pp3c20_15970V3.1.p pacid=32947583 transcript=Pp3c20_15970V3.1 locus=Pp3c20_15970 annot-version=v3.3 Physcomitrella_patens OG0000010
Pp3c20_16090V3.1.p pacid=32947589 transcript=Pp3c20_16090V3.1 locus=Pp3c20_16090 annot-version=v3.3 Physcomitrella_patens OG0000010
Pp3c20_20770V3.1.p pacid=32947597 transcript=Pp3c20_20770V3.1 locus=Pp3c20_20770 annot-version=v3.3 Physcomitrella_patens OG0000010
Pp3c20_1301V3.1.p pacid=32947930 transcript=Pp3c20_1301V3.1 locus=Pp3c20_1301 annot-version=v3.3 Physcomitrella_patens OG0000010
Pp3s794_10V3.1.p pacid=32948338 transcript=Pp3s794_10V3.1 locus=Pp3s794_10 annot-version=v3.3 Physcomitrella_patens OG0000010
Pp3s794_20V3.1.p pacid=32948339 transcript=Pp3s794_20V3.1 locus=Pp3s794_20 annot-version=v3.3 Physcomitrella_patens OG0000010
Pp3c22_10670V3.1.p pacid=32904434 transcript=Pp3c22_10670V3.1 locus=Pp3c22_10670 annot-version=v3.3 Physcomitrella_patens OG0000010
Pp3c23_980V3.1.p pacid=32949214 transcript=Pp3c23_980V3.1 locus=Pp3c23_980 annot-version=v3.3 Physcomitrella_patens OG0000010
Pp3c23_20700V3.1.p pacid=32950000 transcript=Pp3c23_20700V3.1 locus=Pp3c23_20700 annot-version=v3.3 Physcomitrella_patens OG0000010
Pp3c22_14880V3.1.p pacid=32904603 transcript=Pp3c22_14880V3.1 locus=Pp3c22_14880 annot-version=v3.3 Physcomitrella_patens OG0000010
Pp3c23_19230V3.1.p pacid=32951010 transcript=Pp3c23_19230V3.1 locus=Pp3c23_19230 annot-version=v3.3 Physcomitrella_patens OG0000010
Pp3c27_6810V3.1.p pacid=32951402 transcript=Pp3c27_6810V3.1 locus=Pp3c27_6810 annot-version=v3.3 Physcomitrella_patens OG0000010

Pp3c27_6820V3.1.p pacid=32951433 transcript=Pp3c27_6820V3.1
locus=Pp3c27_6820 annot-version=v3.3 Physcomitrella_patens
OG0000010
Pp3c27_5770V3.1.p pacid=32951551 transcript=Pp3c27_5770V3.1
locus=Pp3c27_5770 annot-version=v3.3 Physcomitrella_patens
OG0000010
Pp3c27_5760V3.1.p pacid=32951710 transcript=Pp3c27_5760V3.1
locus=Pp3c27_5760 annot-version=v3.3 Physcomitrella_patens
OG0000010
Pp3c5_2300V3.1.p pacid=32952537 transcript=Pp3c5_2300V3.1
locus=Pp3c5_2300 annot-version=v3.3 Physcomitrella_patens
OG0000010
Pp3c5_2290V3.1.p pacid=32953113 transcript=Pp3c5_2290V3.1
locus=Pp3c5_2290 annot-version=v3.3 Physcomitrella_patens
OG0000010
Pp3c5_6020V3.1.p pacid=32953222 transcript=Pp3c5_6020V3.1
locus=Pp3c5_6020 annot-version=v3.3 Physcomitrella_patens
OG0000010
Pp3c5_6380V3.1.p pacid=32953613 transcript=Pp3c5_6380V3.1
locus=Pp3c5_6380 annot-version=v3.3 Physcomitrella_patens
OG0000010
Pp3c22_2140V3.1.p pacid=32904934 transcript=Pp3c22_2140V3.1
locus=Pp3c22_2140 annot-version=v3.3 Physcomitrella_patens
OG0000010
Pp3c5_12050V3.1.p pacid=32953843 transcript=Pp3c5_12050V3.1
locus=Pp3c5_12050 annot-version=v3.3 Physcomitrella_patens
OG0000010
Pp3c5_20420V3.1.p pacid=32954314 transcript=Pp3c5_20420V3.1
locus=Pp3c5_20420 annot-version=v3.3 Physcomitrella_patens
OG0000010
Pp3c5_15380V3.1.p pacid=32954406 transcript=Pp3c5_15380V3.1
locus=Pp3c5_15380 annot-version=v3.3 Physcomitrella_patens
OG0000010
Pp3c5_12060V3.1.p pacid=32955050 transcript=Pp3c5_12060V3.1
locus=Pp3c5_12060 annot-version=v3.3 Physcomitrella_patens
OG0000010
Pp3c5_12040V3.1.p pacid=32955449 transcript=Pp3c5_12040V3.1
locus=Pp3c5_12040 annot-version=v3.3 Physcomitrella_patens
OG0000010
Pp3c5_6370V3.1.p pacid=32955734 transcript=Pp3c5_6370V3.1
locus=Pp3c5_6370 annot-version=v3.3 Physcomitrella_patens
OG0000010
Pp3c5_20750V3.1.p pacid=32956004 transcript=Pp3c5_20750V3.1
locus=Pp3c5_20750 annot-version=v3.3 Physcomitrella_patens
OG0000010
Pp3c22_3610V3.1.p pacid=32905199 transcript=Pp3c22_3610V3.1
locus=Pp3c22_3610 annot-version=v3.3 Physcomitrella_patens
OG0000010
Pp3c11_8650V3.1.p pacid=32956773 transcript=Pp3c11_8650V3.1
locus=Pp3c11_8650 annot-version=v3.3 Physcomitrella_patens
OG0000010
Pp3c11_20200V3.1.p pacid=32956891 transcript=Pp3c11_20200V3.1
locus=Pp3c11_20200 annot-version=v3.3 Physcomitrella_patens
OG0000010

Pp3c11_21080V3.1.p pacid=32956997 transcript=Pp3c11_21080V3.1 locus=Pp3c11_21080 annot-version=v3.3 Physcomitrella_patens OG0000010
Pp3c11_9660V3.1.p pacid=32957009 transcript=Pp3c11_9660V3.1 locus=Pp3c11_9660 annot-version=v3.3 Physcomitrella_patens OG0000010
Pp3c11_21070V3.1.p pacid=32957448 transcript=Pp3c11_21070V3.1 locus=Pp3c11_21070 annot-version=v3.3 Physcomitrella_patens OG0000010
Pp3c11_2190V3.1.p pacid=32957476 transcript=Pp3c11_2190V3.1 locus=Pp3c11_2190 annot-version=v3.3 Physcomitrella_patens OG0000010
Pp3c11_25610V3.1.p pacid=32957609 transcript=Pp3c11_25610V3.1 locus=Pp3c11_25610 annot-version=v3.3 Physcomitrella_patens OG0000010
Pp3c11_23790V3.1.p pacid=32957664 transcript=Pp3c11_23790V3.1 locus=Pp3c11_23790 annot-version=v3.3 Physcomitrella_patens OG0000010
Pp3c11_21100V3.1.p pacid=32957780 transcript=Pp3c11_21100V3.1 locus=Pp3c11_21100 annot-version=v3.3 Physcomitrella_patens OG0000010
Pp3c11_22090V3.1.p pacid=32957824 transcript=Pp3c11_22090V3.1 locus=Pp3c11_22090 annot-version=v3.3 Physcomitrella_patens OG0000010
Pp3c11_22080V3.1.p pacid=32957868 transcript=Pp3c11_22080V3.1 locus=Pp3c11_22080 annot-version=v3.3 Physcomitrella_patens OG0000010
Pp3s920_10V3.1.p pacid=32905372 transcript=Pp3s920_10V3.1 locus=Pp3s920_10 annot-version=v3.3 Physcomitrella_patens OG0000010
Pp3c11_21104V3.1.p pacid=32958322 transcript=Pp3c11_21104V3.1 locus=Pp3c11_21104 annot-version=v3.3 Physcomitrella_patens OG0000010
Pp3c11_13940V3.1.p pacid=32958571 transcript=Pp3c11_13940V3.1 locus=Pp3c11_13940 annot-version=v3.3 Physcomitrella_patens OG0000010
Pp3c11_3280V3.1.p pacid=32958660 transcript=Pp3c11_3280V3.1 locus=Pp3c11_3280 annot-version=v3.3 Physcomitrella_patens OG0000010
Pp3c11_14020V3.1.p pacid=32958664 transcript=Pp3c11_14020V3.1 locus=Pp3c11_14020 annot-version=v3.3 Physcomitrella_patens OG0000010
Pp3c11_13930V3.1.p pacid=32958731 transcript=Pp3c11_13930V3.1 locus=Pp3c11_13930 annot-version=v3.3 Physcomitrella_patens OG0000010
Pp3c11_22170V3.1.p pacid=32959021 transcript=Pp3c11_22170V3.1 locus=Pp3c11_22170 annot-version=v3.3 Physcomitrella_patens OG0000010
Pp3c11_15520V3.1.p pacid=32959076 transcript=Pp3c11_15520V3.1 locus=Pp3c11_15520 annot-version=v3.3 Physcomitrella_patens OG0000010
Pp3c11_21090V3.1.p pacid=32959646 transcript=Pp3c11_21090V3.1 locus=Pp3c11_21090 annot-version=v3.3 Physcomitrella_patens OG0000010

Pp3c11_22180V3.1.p pacid=32959667 transcript=Pp3c11_22180V3.1 locus=Pp3c11_22180 annot-version=v3.3 Physcomitrella_patens OG0000010
Pp3c14_1440V3.1.p pacid=32960059 transcript=Pp3c14_1440V3.1 locus=Pp3c14_1440 annot-version=v3.3 Physcomitrella_patens OG0000010
Pp3c14_13610V3.1.p pacid=32960162 transcript=Pp3c14_13610V3.1 locus=Pp3c14_13610 annot-version=v3.3 Physcomitrella_patens OG0000010
Pp3c14_13600V3.1.p pacid=32960296 transcript=Pp3c14_13600V3.1 locus=Pp3c14_13600 annot-version=v3.3 Physcomitrella_patens OG0000010
Pp3c14_13230V3.1.p pacid=32960866 transcript=Pp3c14_13230V3.1 locus=Pp3c14_13230 annot-version=v3.3 Physcomitrella_patens OG0000010
Pp3c14_6740V3.1.p pacid=32961336 transcript=Pp3c14_6740V3.1 locus=Pp3c14_6740 annot-version=v3.3 Physcomitrella_patens OG0000010
Pp3c17_21720V3.1.p pacid=32905698 transcript=Pp3c17_21720V3.1 locus=Pp3c17_21720 annot-version=v3.3 Physcomitrella_patens OG0000010
Pp3c14_25450V3.1.p pacid=32961787 transcript=Pp3c14_25450V3.1 locus=Pp3c14_25450 annot-version=v3.3 Physcomitrella_patens OG0000010
Pp3c14_1111V3.1.p pacid=32961798 transcript=Pp3c14_1111V3.1 locus=Pp3c14_1111 annot-version=v3.3 Physcomitrella_patens OG0000010
Pp3c17_8770V3.1.p pacid=32905778 transcript=Pp3c17_8770V3.1 locus=Pp3c17_8770 annot-version=v3.3 Physcomitrella_patens OG0000010
Pp3c14_23480V3.1.p pacid=32962437 transcript=Pp3c14_23480V3.1 locus=Pp3c14_23480 annot-version=v3.3 Physcomitrella_patens OG0000010
Pp3c14_6750V3.1.p pacid=32962675 transcript=Pp3c14_6750V3.1 locus=Pp3c14_6750 annot-version=v3.3 Physcomitrella_patens OG0000010
Pp3c14_11380V3.1.p pacid=32962696 transcript=Pp3c14_11380V3.1 locus=Pp3c14_11380 annot-version=v3.3 Physcomitrella_patens OG0000010
Pp3c14_12500V3.1.p pacid=32963148 transcript=Pp3c14_12500V3.1 locus=Pp3c14_12500 annot-version=v3.3 Physcomitrella_patens OG0000010
Pp3c8_23980V3.1.p pacid=32963670 transcript=Pp3c8_23980V3.1 locus=Pp3c8_23980 annot-version=v3.3 Physcomitrella_patens OG0000010
Pp3c8_2160V3.1.p pacid=32963804 transcript=Pp3c8_2160V3.1 locus=Pp3c8_2160 annot-version=v3.3 Physcomitrella_patens OG0000010
Pp3c8_1850V3.1.p pacid=32963878 transcript=Pp3c8_1850V3.1 locus=Pp3c8_1850 annot-version=v3.3 Physcomitrella_patens OG0000010
Pp3c8_14413V3.1.p pacid=32963911 transcript=Pp3c8_14413V3.1 locus=Pp3c8_14413 annot-version=v3.3 Physcomitrella_patens OG0000010

Pp3c8_17980V3.1.p pacid=32964174 transcript=Pp3c8_17980V3.1 locus=Pp3c8_17980 annot-version=v3.3 Physcomitrella_patens OG0000010
Pp3c8_17880V3.1.p pacid=32964185 transcript=Pp3c8_17880V3.1 locus=Pp3c8_17880 annot-version=v3.3 Physcomitrella_patens OG0000010
Pp3c8_17870V3.1.p pacid=32964347 transcript=Pp3c8_17870V3.1 locus=Pp3c8_17870 annot-version=v3.3 Physcomitrella_patens OG0000010
Pp3c8_13800V3.1.p pacid=32964468 transcript=Pp3c8_13800V3.1 locus=Pp3c8_13800 annot-version=v3.3 Physcomitrella_patens OG0000010
Pp3c17_20961V3.1.p pacid=32906005 transcript=Pp3c17_20961V3.1 locus=Pp3c17_20961 annot-version=v3.3 Physcomitrella_patens OG0000010
Pp3c8_2020V3.1.p pacid=32965117 transcript=Pp3c8_2020V3.1 locus=Pp3c8_2020 annot-version=v3.3 Physcomitrella_patens OG0000010
Pp3c8_25420V3.1.p pacid=32965240 transcript=Pp3c8_25420V3.1 locus=Pp3c8_25420 annot-version=v3.3 Physcomitrella_patens OG0000010
Pp3c8_12210V3.1.p pacid=32965444 transcript=Pp3c8_12210V3.1 locus=Pp3c8_12210 annot-version=v3.3 Physcomitrella_patens OG0000010
Pp3c8_2380V3.1.p pacid=32965724 transcript=Pp3c8_2380V3.1 locus=Pp3c8_2380 annot-version=v3.3 Physcomitrella_patens OG0000010
Pp3c8_2140V3.1.p pacid=32965734 transcript=Pp3c8_2140V3.1 locus=Pp3c8_2140 annot-version=v3.3 Physcomitrella_patens OG0000010
Pp3c8_14400V3.1.p pacid=32966163 transcript=Pp3c8_14400V3.1 locus=Pp3c8_14400 annot-version=v3.3 Physcomitrella_patens OG0000010
Pp3c8_2360V3.1.p pacid=32966278 transcript=Pp3c8_2360V3.1 locus=Pp3c8_2360 annot-version=v3.3 Physcomitrella_patens OG0000010
Pp3c17_21750V3.1.p pacid=32906169 transcript=Pp3c17_21750V3.1 locus=Pp3c17_21750 annot-version=v3.3 Physcomitrella_patens OG0000010
Pp3c8_13790V3.1.p pacid=32966353 transcript=Pp3c8_13790V3.1 locus=Pp3c8_13790 annot-version=v3.3 Physcomitrella_patens OG0000010
Pp3c8_22280V3.1.p pacid=32966354 transcript=Pp3c8_22280V3.1 locus=Pp3c8_22280 annot-version=v3.3 Physcomitrella_patens OG0000010
Pp3c8_2630V3.1.p pacid=32966443 transcript=Pp3c8_2630V3.1 locus=Pp3c8_2630 annot-version=v3.3 Physcomitrella_patens OG0000010
Pp3c17_21570V3.1.p pacid=32906228 transcript=Pp3c17_21570V3.1 locus=Pp3c17_21570 annot-version=v3.3 Physcomitrella_patens OG0000010
Pp3c1_37810V3.1.p pacid=32966939 transcript=Pp3c1_37810V3.1 locus=Pp3c1_37810 annot-version=v3.3 Physcomitrella_patens OG0000010

Pp3c1_34500V3.1.p pacid=32967312 transcript=Pp3c1_34500V3.1 locus=Pp3c1_34500 annot-version=v3.3 Physcomitrella_patens OG0000010
Pp3c1_34490V3.1.p pacid=32967661 transcript=Pp3c1_34490V3.1 locus=Pp3c1_34490 annot-version=v3.3 Physcomitrella_patens OG0000010
Pp3c1_34480V3.1.p pacid=32968603 transcript=Pp3c1_34480V3.1 locus=Pp3c1_34480 annot-version=v3.3 Physcomitrella_patens OG0000010
Pp3c17_18970V3.1.p pacid=32906433 transcript=Pp3c17_18970V3.1 locus=Pp3c17_18970 annot-version=v3.3 Physcomitrella_patens OG0000010
Pp3c1_11020V3.1.p pacid=32968863 transcript=Pp3c1_11020V3.1 locus=Pp3c1_11020 annot-version=v3.3 Physcomitrella_patens OG0000010
Pp3c1_34504V3.1.p pacid=32969101 transcript=Pp3c1_34504V3.1 locus=Pp3c1_34504 annot-version=v3.3 Physcomitrella_patens OG0000010
Pp3c17_21563V3.1.p pacid=32906492 transcript=Pp3c17_21563V3.1 locus=Pp3c17_21563 annot-version=v3.3 Physcomitrella_patens OG0000010
Pp3c1_50V3.1.p pacid=32969223 transcript=Pp3c1_50V3.1 locus=Pp3c1_50 annot-version=v3.3 Physcomitrella_patens OG0000010
Pp3c10_2290V3.1.p pacid=32900079 transcript=Pp3c10_2290V3.1 locus=Pp3c10_2290 annot-version=v3.3 Physcomitrella_patens OG0000010
Pp3c1_30800V3.1.p pacid=32970453 transcript=Pp3c1_30800V3.1 locus=Pp3c1_30800 annot-version=v3.3 Physcomitrella_patens OG0000010
Pp3c1_14950V3.1.p pacid=32970571 transcript=Pp3c1_14950V3.1 locus=Pp3c1_14950 annot-version=v3.3 Physcomitrella_patens OG0000010
Pp3c1_8420V3.1.p pacid=32971222 transcript=Pp3c1_8420V3.1 locus=Pp3c1_8420 annot-version=v3.3 Physcomitrella_patens OG0000010
Pp3c17_18940V3.1.p pacid=32906719 transcript=Pp3c17_18940V3.1 locus=Pp3c17_18940 annot-version=v3.3 Physcomitrella_patens OG0000010
Pp3c1_11340V3.1.p pacid=32971643 transcript=Pp3c1_11340V3.1 locus=Pp3c1_11340 annot-version=v3.3 Physcomitrella_patens OG0000010
Pp3c17_230V3.1.p pacid=32906777 transcript=Pp3c17_230V3.1 locus=Pp3c17_230 annot-version=v3.3 Physcomitrella_patens OG0000010
Pp3c12_3030V3.1.p pacid=32972511 transcript=Pp3c12_3030V3.1 locus=Pp3c12_3030 annot-version=v3.3 Physcomitrella_patens OG0000010
Pp3c17_21740V3.1.p pacid=32906835 transcript=Pp3c17_21740V3.1 locus=Pp3c17_21740 annot-version=v3.3 Physcomitrella_patens OG0000010
Pp3c12_3080V3.1.p pacid=32972743 transcript=Pp3c12_3080V3.1 locus=Pp3c12_3080 annot-version=v3.3 Physcomitrella_patens OG0000010
Pp3c12_3820V3.1.p pacid=32972838 transcript=Pp3c12_3820V3.1

locus=Pp3c12_3820 annot-version=v3.3 Physcomitrella_patens
OG0000010
Pp3c12_3850V3.1.p pacid=32973942 transcript=Pp3c12_3850V3.1
locus=Pp3c12_3850 annot-version=v3.3 Physcomitrella_patens
OG0000010
Pp3c12_10510V3.1.p pacid=32974022 transcript=Pp3c12_10510V3.1
locus=Pp3c12_10510 annot-version=v3.3 Physcomitrella_patens
OG0000010
Pp3c17_20880V3.1.p pacid=32906975 transcript=Pp3c17_20880V3.1
locus=Pp3c17_20880 annot-version=v3.3 Physcomitrella_patens
OG0000010
Pp3c12_3810V3.1.p pacid=32974365 transcript=Pp3c12_3810V3.1
locus=Pp3c12_3810 annot-version=v3.3 Physcomitrella_patens
OG0000010
Pp3c12_24740V3.1.p pacid=32974471 transcript=Pp3c12_24740V3.1
locus=Pp3c12_24740 annot-version=v3.3 Physcomitrella_patens
OG0000010
Pp3c12_13640V3.1.p pacid=32974724 transcript=Pp3c12_13640V3.1
locus=Pp3c12_13640 annot-version=v3.3 Physcomitrella_patens
OG0000010
Pp3c12_24800V3.1.p pacid=32975132 transcript=Pp3c12_24800V3.1
locus=Pp3c12_24800 annot-version=v3.3 Physcomitrella_patens
OG0000010
Pp3c6_22490V3.1.p pacid=32975446 transcript=Pp3c6_22490V3.1
locus=Pp3c6_22490 annot-version=v3.3 Physcomitrella_patens
OG0000010
Pp3c6_20110V3.1.p pacid=32975946 transcript=Pp3c6_20110V3.1
locus=Pp3c6_20110 annot-version=v3.3 Physcomitrella_patens
OG0000010
Pp3c6_14630V3.1.p pacid=32975949 transcript=Pp3c6_14630V3.1
locus=Pp3c6_14630 annot-version=v3.3 Physcomitrella_patens
OG0000010
Pp3c6_12400V3.1.p pacid=32976141 transcript=Pp3c6_12400V3.1
locus=Pp3c6_12400 annot-version=v3.3 Physcomitrella_patens
OG0000010
Pp3c6_8150V3.1.p pacid=32976207 transcript=Pp3c6_8150V3.1
locus=Pp3c6_8150 annot-version=v3.3 Physcomitrella_patens
OG0000010
Pp3c6_790V3.1.p pacid=32976458 transcript=Pp3c6_790V3.1
locus=Pp3c6_790 annot-version=v3.3 Physcomitrella_patens
OG0000010
Pp3c6_14010V3.1.p pacid=32976592 transcript=Pp3c6_14010V3.1
locus=Pp3c6_14010 annot-version=v3.3 Physcomitrella_patens
OG0000010
Pp3c6_17130V3.1.p pacid=32976817 transcript=Pp3c6_17130V3.1
locus=Pp3c6_17130 annot-version=v3.3 Physcomitrella_patens
OG0000010
Pp3c6_25490V3.1.p pacid=32976836 transcript=Pp3c6_25490V3.1
locus=Pp3c6_25490 annot-version=v3.3 Physcomitrella_patens
OG0000010
Pp3c6_5620V3.1.p pacid=32976916 transcript=Pp3c6_5620V3.1
locus=Pp3c6_5620 annot-version=v3.3 Physcomitrella_patens
OG0000010
Pp3c6_14910V3.1.p pacid=32977015 transcript=Pp3c6_14910V3.1

locus=Pp3c6_14910 annot-version=v3.3 Physcomitrella_patens
OG0000010
Pp3c6_5930V3.1.p pacid=32977061 transcript=Pp3c6_5930V3.1
locus=Pp3c6_5930 annot-version=v3.3 Physcomitrella_patens
OG0000010
Pp3c6_5920V3.1.p pacid=32977466 transcript=Pp3c6_5920V3.1
locus=Pp3c6_5920 annot-version=v3.3 Physcomitrella_patens
OG0000010
Pp3c6_23570V3.1.p pacid=32977467 transcript=Pp3c6_23570V3.1
locus=Pp3c6_23570 annot-version=v3.3 Physcomitrella_patens
OG0000010
Pp3c6_14640V3.1.p pacid=32977528 transcript=Pp3c6_14640V3.1
locus=Pp3c6_14640 annot-version=v3.3 Physcomitrella_patens
OG0000010
Pp3c6_11870V3.1.p pacid=32977600 transcript=Pp3c6_11870V3.1
locus=Pp3c6_11870 annot-version=v3.3 Physcomitrella_patens
OG0000010
Pp3c17_21566V3.1.p pacid=32907308 transcript=Pp3c17_21566V3.1
locus=Pp3c17_21566 annot-version=v3.3 Physcomitrella_patens
OG0000010
Pp3c6_6000V3.1.p pacid=32977868 transcript=Pp3c6_6000V3.1
locus=Pp3c6_6000 annot-version=v3.3 Physcomitrella_patens
OG0000010
Pp3c6_5610V3.1.p pacid=32978035 transcript=Pp3c6_5610V3.1
locus=Pp3c6_5610 annot-version=v3.3 Physcomitrella_patens
OG0000010
Pp3c6_5670V3.1.p pacid=32978068 transcript=Pp3c6_5670V3.1
locus=Pp3c6_5670 annot-version=v3.3 Physcomitrella_patens
OG0000010
Pp3c17_22160V3.1.p pacid=32907336 transcript=Pp3c17_22160V3.1
locus=Pp3c17_22160 annot-version=v3.3 Physcomitrella_patens
OG0000010
Pp3c6_5910V3.1.p pacid=32978337 transcript=Pp3c6_5910V3.1
locus=Pp3c6_5910 annot-version=v3.3 Physcomitrella_patens
OG0000010
Pp3c6_12460V3.1.p pacid=32978364 transcript=Pp3c6_12460V3.1
locus=Pp3c6_12460 annot-version=v3.3 Physcomitrella_patens
OG0000010
Pp3c6_9050V3.1.p pacid=32978563 transcript=Pp3c6_9050V3.1
locus=Pp3c6_9050 annot-version=v3.3 Physcomitrella_patens
OG0000010
Pp3c6_9600V3.1.p pacid=32978921 transcript=Pp3c6_9600V3.1
locus=Pp3c6_9600 annot-version=v3.3 Physcomitrella_patens
OG0000010
Pp3c17_14980V3.1.p pacid=32907424 transcript=Pp3c17_14980V3.1
locus=Pp3c17_14980 annot-version=v3.3 Physcomitrella_patens
OG0000010
Pp3c25_5930V3.1.p pacid=32979409 transcript=Pp3c25_5930V3.1
locus=Pp3c25_5930 annot-version=v3.3 Physcomitrella_patens
OG0000010
Pp3c25_8790V3.1.p pacid=32979413 transcript=Pp3c25_8790V3.1
locus=Pp3c25_8790 annot-version=v3.3 Physcomitrella_patens
OG0000010
Pp3c25_10330V3.1.p pacid=32979495 transcript=Pp3c25_10330V3.1

locus=Pp3c25_10330 annot-version=v3.3   Physcomitrella_patens
OG0000010
Pp3c17_21730V3.1.p pacid=32907513 transcript=Pp3c17_21730V3.1
locus=Pp3c17_21730 annot-version=v3.3   Physcomitrella_patens
OG0000010
Pp3c25_10340V3.1.p pacid=32980238 transcript=Pp3c25_10340V3.1
locus=Pp3c25_10340 annot-version=v3.3   Physcomitrella_patens
OG0000010
Pp3c17_18950V3.1.p pacid=32907586 transcript=Pp3c17_18950V3.1
locus=Pp3c17_18950 annot-version=v3.3   Physcomitrella_patens
OG0000010
Pp3c25_510V3.1.p pacid=32980659 transcript=Pp3c25_510V3.1
locus=Pp3c25_510 annot-version=v3.3   Physcomitrella_patens
OG0000010
Pp3c25_5920V3.1.p pacid=32980733 transcript=Pp3c25_5920V3.1
locus=Pp3c25_5920 annot-version=v3.3   Physcomitrella_patens
OG0000010
Pp3c18_4530V3.1.p pacid=32980989 transcript=Pp3c18_4530V3.1
locus=Pp3c18_4530 annot-version=v3.3   Physcomitrella_patens
OG0000010
Pp3c18_4910V3.1.p pacid=32980993 transcript=Pp3c18_4910V3.1
locus=Pp3c18_4910 annot-version=v3.3   Physcomitrella_patens
OG0000010
Pp3c17_8750V3.1.p pacid=32907678 transcript=Pp3c17_8750V3.1
locus=Pp3c17_8750 annot-version=v3.3   Physcomitrella_patens
OG0000010
Pp3c18_4610V3.1.p pacid=32981362 transcript=Pp3c18_4610V3.1
locus=Pp3c18_4610 annot-version=v3.3   Physcomitrella_patens
OG0000010
Pp3c18_20820V3.1.p pacid=32981410 transcript=Pp3c18_20820V3.1
locus=Pp3c18_20820 annot-version=v3.3   Physcomitrella_patens
OG0000010
Pp3c17_20870V3.1.p pacid=32907726 transcript=Pp3c17_20870V3.1
locus=Pp3c17_20870 annot-version=v3.3   Physcomitrella_patens
OG0000010
Pp3c18_2400V3.1.p pacid=32981473 transcript=Pp3c18_2400V3.1
locus=Pp3c18_2400 annot-version=v3.3   Physcomitrella_patens
OG0000010
Pp3c18_2880V3.1.p pacid=32981487 transcript=Pp3c18_2880V3.1
locus=Pp3c18_2880 annot-version=v3.3   Physcomitrella_patens
OG0000010
Pp3c18_22320V3.1.p pacid=32981558 transcript=Pp3c18_22320V3.1
locus=Pp3c18_22320 annot-version=v3.3   Physcomitrella_patens
OG0000010
Pp3c18_2410V3.1.p pacid=32981570 transcript=Pp3c18_2410V3.1
locus=Pp3c18_2410 annot-version=v3.3   Physcomitrella_patens
OG0000010
Pp3c18_21560V3.1.p pacid=32982272 transcript=Pp3c18_21560V3.1
locus=Pp3c18_21560 annot-version=v3.3   Physcomitrella_patens
OG0000010
Pp3c18_6230V3.1.p pacid=32982482 transcript=Pp3c18_6230V3.1
locus=Pp3c18_6230 annot-version=v3.3   Physcomitrella_patens
OG0000010
Pp3c18_18616V3.1.p pacid=32982926 transcript=Pp3c18_18616V3.1

locus=Pp3c18_18616 annot-version=v3.3   Physcomitrella_patens
OG0000010
Pp3c18_2390V3.1.p pacid=32983073 transcript=Pp3c18_2390V3.1
locus=Pp3c18_2390 annot-version=v3.3   Physcomitrella_patens
OG0000010
Pp3c17_18930V3.1.p pacid=32907937 transcript=Pp3c17_18930V3.1
locus=Pp3c17_18930 annot-version=v3.3   Physcomitrella_patens
OG0000010
Pp3c18_4570V3.1.p pacid=32983484 transcript=Pp3c18_4570V3.1
locus=Pp3c18_4570 annot-version=v3.3   Physcomitrella_patens
OG0000010
Pp3c16_23160V3.1.p pacid=32983699 transcript=Pp3c16_23160V3.1
locus=Pp3c16_23160 annot-version=v3.3   Physcomitrella_patens
OG0000010
Pp3c17_20920V3.1.p pacid=32907977 transcript=Pp3c17_20920V3.1
locus=Pp3c17_20920 annot-version=v3.3   Physcomitrella_patens
OG0000010
Pp3c16_21720V3.1.p pacid=32984211 transcript=Pp3c16_21720V3.1
locus=Pp3c16_21720 annot-version=v3.3   Physcomitrella_patens
OG0000010
Pp3c16_21730V3.1.p pacid=32984214 transcript=Pp3c16_21730V3.1
locus=Pp3c16_21730 annot-version=v3.3   Physcomitrella_patens
OG0000010
Pp3c16_1650V3.1.p pacid=32984308 transcript=Pp3c16_1650V3.1
locus=Pp3c16_1650 annot-version=v3.3   Physcomitrella_patens
OG0000010
Pp3c16_21760V3.1.p pacid=32984752 transcript=Pp3c16_21760V3.1
locus=Pp3c16_21760 annot-version=v3.3   Physcomitrella_patens
OG0000010
Pp3c16_23150V3.1.p pacid=32985086 transcript=Pp3c16_23150V3.1
locus=Pp3c16_23150 annot-version=v3.3   Physcomitrella_patens
OG0000010
Pp3c16_16230V3.1.p pacid=32985649 transcript=Pp3c16_16230V3.1
locus=Pp3c16_16230 annot-version=v3.3   Physcomitrella_patens
OG0000010
Pp3c16_1660V3.1.p pacid=32985875 transcript=Pp3c16_1660V3.1
locus=Pp3c16_1660 annot-version=v3.3   Physcomitrella_patens
OG0000010
Pp3c16_8780V3.1.p pacid=32986537 transcript=Pp3c16_8780V3.1
locus=Pp3c16_8780 annot-version=v3.3   Physcomitrella_patens
OG0000010
Pp3c16_19550V3.1.p pacid=32986637 transcript=Pp3c16_19550V3.1
locus=Pp3c16_19550 annot-version=v3.3   Physcomitrella_patens
OG0000010
Pp3c24_12401V3.1.p pacid=32909033 transcript=Pp3c24_12401V3.1
locus=Pp3c24_12401 annot-version=v3.3   Physcomitrella_patens
OG0000010
Pp3c24_5360V3.1.p pacid=32909303 transcript=Pp3c24_5360V3.1
locus=Pp3c24_5360 annot-version=v3.3   Physcomitrella_patens
OG0000010
Pp3c24_4930V3.1.p pacid=32909926 transcript=Pp3c24_4930V3.1
locus=Pp3c24_4930 annot-version=v3.3   Physcomitrella_patens
OG0000010
Pp3c24_5770V3.1.p pacid=32910509 transcript=Pp3c24_5770V3.1

locus=Pp3c24_5770 annot-version=v3.3	Physcomitrella_patens
OG0000010
Pp3s203_40V3.1.p pacid=32911065 transcript=Pp3s203_40V3.1
locus=Pp3s203_40 annot-version=v3.3	Physcomitrella_patens
OG0000010
Pp3s203_50V3.1.p pacid=32911068 transcript=Pp3s203_50V3.1
locus=Pp3s203_50 annot-version=v3.3	Physcomitrella_patens
OG0000010
Pp3c9_9314V3.1.p pacid=32911096 transcript=Pp3c9_9314V3.1
locus=Pp3c9_9314 annot-version=v3.3	Physcomitrella_patens
OG0000010
Pp3c9_22180V3.1.p pacid=32911305 transcript=Pp3c9_22180V3.1
locus=Pp3c9_22180 annot-version=v3.3	Physcomitrella_patens
OG0000010
Pp3c9_16400V3.1.p pacid=32911521 transcript=Pp3c9_16400V3.1
locus=Pp3c9_16400 annot-version=v3.3	Physcomitrella_patens
OG0000010
Pp3c9_25040V3.1.p pacid=32911978 transcript=Pp3c9_25040V3.1
locus=Pp3c9_25040 annot-version=v3.3	Physcomitrella_patens
OG0000010
Pp3c9_24260V3.1.p pacid=32912213 transcript=Pp3c9_24260V3.1
locus=Pp3c9_24260 annot-version=v3.3	Physcomitrella_patens
OG0000010
Pp3c9_24930V3.1.p pacid=32912567 transcript=Pp3c9_24930V3.1
locus=Pp3c9_24930 annot-version=v3.3	Physcomitrella_patens
OG0000010
Pp3c9_1940V3.1.p pacid=32912569 transcript=Pp3c9_1940V3.1
locus=Pp3c9_1940 annot-version=v3.3	Physcomitrella_patens
OG0000010
Pp3c10_19140V3.1.p pacid=32900730 transcript=Pp3c10_19140V3.1
locus=Pp3c10_19140 annot-version=v3.3	Physcomitrella_patens
OG0000010
Pp3c9_16390V3.1.p pacid=32912775 transcript=Pp3c9_16390V3.1
locus=Pp3c9_16390 annot-version=v3.3	Physcomitrella_patens
OG0000010
Pp3c9_9320V3.1.p pacid=32912910 transcript=Pp3c9_9320V3.1
locus=Pp3c9_9320 annot-version=v3.3	Physcomitrella_patens
OG0000010
Pp3c10_3240V3.1.p pacid=32900762 transcript=Pp3c10_3240V3.1
locus=Pp3c10_3240 annot-version=v3.3	Physcomitrella_patens
OG0000010
Pp3c10_20780V3.1.p pacid=32900766 transcript=Pp3c10_20780V3.1
locus=Pp3c10_20780 annot-version=v3.3	Physcomitrella_patens
OG0000010
Pp3c9_24960V3.1.p pacid=32913198 transcript=Pp3c9_24960V3.1
locus=Pp3c9_24960 annot-version=v3.3	Physcomitrella_patens
OG0000010
Pp3c9_24950V3.1.p pacid=32913294 transcript=Pp3c9_24950V3.1
locus=Pp3c9_24950 annot-version=v3.3	Physcomitrella_patens
OG0000010
Pp3c9_10220V3.1.p pacid=32913516 transcript=Pp3c9_10220V3.1
locus=Pp3c9_10220 annot-version=v3.3	Physcomitrella_patens
OG0000010
Pp3c9_10230V3.1.p pacid=32913522 transcript=Pp3c9_10230V3.1

locus=Pp3c9_10230 annot-version=v3.3 Physcomitrella_patens
OG0000010
Pp3c9_16410V3.1.p pacid=32913650 transcript=Pp3c9_16410V3.1
locus=Pp3c9_16410 annot-version=v3.3 Physcomitrella_patens
OG0000010
Pp3c9_24920V3.1.p pacid=32913915 transcript=Pp3c9_24920V3.1
locus=Pp3c9_24920 annot-version=v3.3 Physcomitrella_patens
OG0000010
Pp3c9_9330V3.1.p pacid=32914307 transcript=Pp3c9_9330V3.1
locus=Pp3c9_9330 annot-version=v3.3 Physcomitrella_patens
OG0000010
Pp3c21_6250V3.1.p pacid=32914393 transcript=Pp3c21_6250V3.1
locus=Pp3c21_6250 annot-version=v3.3 Physcomitrella_patens
OG0000010
Pp3c21_17680V3.1.p pacid=32914405 transcript=Pp3c21_17680V3.1
locus=Pp3c21_17680 annot-version=v3.3 Physcomitrella_patens
OG0000010
Pp3c21_5860V3.1.p pacid=32914575 transcript=Pp3c21_5860V3.1
locus=Pp3c21_5860 annot-version=v3.3 Physcomitrella_patens
OG0000010
Pp3c21_6260V3.1.p pacid=32914622 transcript=Pp3c21_6260V3.1
locus=Pp3c21_6260 annot-version=v3.3 Physcomitrella_patens
OG0000010
Pp3c21_17690V3.1.p pacid=32914921 transcript=Pp3c21_17690V3.1
locus=Pp3c21_17690 annot-version=v3.3 Physcomitrella_patens
OG0000010
Pp3c21_14720V3.1.p pacid=32914943 transcript=Pp3c21_14720V3.1
locus=Pp3c21_14720 annot-version=v3.3 Physcomitrella_patens
OG0000010
Pp3c21_14690V3.1.p pacid=32915016 transcript=Pp3c21_14690V3.1
locus=Pp3c21_14690 annot-version=v3.3 Physcomitrella_patens
OG0000010
Pp3c21_15570V3.1.p pacid=32915073 transcript=Pp3c21_15570V3.1
locus=Pp3c21_15570 annot-version=v3.3 Physcomitrella_patens
OG0000010
Pp3c21_14710V3.1.p pacid=32915113 transcript=Pp3c21_14710V3.1
locus=Pp3c21_14710 annot-version=v3.3 Physcomitrella_patens
OG0000010
Pp3c21_16759V3.1.p pacid=32915205 transcript=Pp3c21_16759V3.1
locus=Pp3c21_16759 annot-version=v3.3 Physcomitrella_patens
OG0000010
Pp3c21_18290V3.1.p pacid=32915242 transcript=Pp3c21_18290V3.1
locus=Pp3c21_18290 annot-version=v3.3 Physcomitrella_patens
OG0000010
Pp3c21_7380V3.1.p pacid=32915261 transcript=Pp3c21_7380V3.1
locus=Pp3c21_7380 annot-version=v3.3 Physcomitrella_patens
OG0000010
Pp3c21_14263V3.1.p pacid=32915357 transcript=Pp3c21_14263V3.1
locus=Pp3c21_14263 annot-version=v3.3 Physcomitrella_patens
OG0000010
Pp3c21_7390V3.1.p pacid=32915724 transcript=Pp3c21_7390V3.1
locus=Pp3c21_7390 annot-version=v3.3 Physcomitrella_patens
OG0000010
Pp3c21_8760V3.1.p pacid=32915793 transcript=Pp3c21_8760V3.1

locus=Pp3c21_8760 annot-version=v3.3 Physcomitrella_patens
OG0000010
Pp3c10_3030V3.1.p pacid=32901077 transcript=Pp3c10_3030V3.1
locus=Pp3c10_3030 annot-version=v3.3 Physcomitrella_patens
OG0000010
Pp3c21_17700V3.1.p pacid=32916139 transcript=Pp3c21_17700V3.1
locus=Pp3c21_17700 annot-version=v3.3 Physcomitrella_patens
OG0000010
Pp3c21_6270V3.1.p pacid=32916482 transcript=Pp3c21_6270V3.1
locus=Pp3c21_6270 annot-version=v3.3 Physcomitrella_patens
OG0000010
Pp3c21_3920V3.1.p pacid=32916555 transcript=Pp3c21_3920V3.1
locus=Pp3c21_3920 annot-version=v3.3 Physcomitrella_patens
OG0000010
Pp3c21_14264V3.1.p pacid=32916586 transcript=Pp3c21_14264V3.1
locus=Pp3c21_14264 annot-version=v3.3 Physcomitrella_patens
OG0000010
Pp3c21_8750V3.1.p pacid=32916739 transcript=Pp3c21_8750V3.1
locus=Pp3c21_8750 annot-version=v3.3 Physcomitrella_patens
OG0000010
Pp3c21_11770V3.1.p pacid=32916761 transcript=Pp3c21_11770V3.1
locus=Pp3c21_11770 annot-version=v3.3 Physcomitrella_patens
OG0000010
Pp3c21_18280V3.1.p pacid=32916764 transcript=Pp3c21_18280V3.1
locus=Pp3c21_18280 annot-version=v3.3 Physcomitrella_patens
OG0000010
Pp3c26_2480V3.1.p pacid=32916997 transcript=Pp3c26_2480V3.1
locus=Pp3c26_2480 annot-version=v3.3 Physcomitrella_patens
OG0000010
Pp3c26_14210V3.1.p pacid=32917487 transcript=Pp3c26_14210V3.1
locus=Pp3c26_14210 annot-version=v3.3 Physcomitrella_patens
OG0000010
Pp3c26_2470V3.1.p pacid=32918074 transcript=Pp3c26_2470V3.1
locus=Pp3c26_2470 annot-version=v3.3 Physcomitrella_patens
OG0000010
Pp3c4_31670V3.1.p pacid=32918843 transcript=Pp3c4_31670V3.1
locus=Pp3c4_31670 annot-version=v3.3 Physcomitrella_patens
OG0000010
Pp3c4_15260V3.1.p pacid=32919003 transcript=Pp3c4_15260V3.1
locus=Pp3c4_15260 annot-version=v3.3 Physcomitrella_patens
OG0000010
Pp3c4_5340V3.1.p pacid=32920499 transcript=Pp3c4_5340V3.1
locus=Pp3c4_5340 annot-version=v3.3 Physcomitrella_patens
OG0000010
Pp3c10_16118V3.1.p pacid=32899570 transcript=Pp3c10_16118V3.1
locus=Pp3c10_16118 annot-version=v3.3 Physcomitrella_patens
OG0000010
Pp3c4_13350V3.1.p pacid=32921003 transcript=Pp3c4_13350V3.1
locus=Pp3c4_13350 annot-version=v3.3 Physcomitrella_patens
OG0000010
Pp3c4_21530V3.1.p pacid=32921093 transcript=Pp3c4_21530V3.1
locus=Pp3c4_21530 annot-version=v3.3 Physcomitrella_patens
OG0000010
Pp3c10_3620V3.1.p pacid=32901645 transcript=Pp3c10_3620V3.1

locus=Pp3c10_3620 annot-version=v3.3	Physcomitrella_patens
OG0000010
Pp3c4_13340V3.1.p pacid=32922376 transcript=Pp3c4_13340V3.1
locus=Pp3c4_13340 annot-version=v3.3	Physcomitrella_patens
OG0000010
Pp3c4_21050V3.1.p pacid=32922815 transcript=Pp3c4_21050V3.1
locus=Pp3c4_21050 annot-version=v3.3	Physcomitrella_patens
OG0000010
Pp3c7_17397V3.1.p pacid=32923150 transcript=Pp3c7_17397V3.1
locus=Pp3c7_17397 annot-version=v3.3	Physcomitrella_patens
OG0000010
Pp3c7_21880V3.1.p pacid=32923417 transcript=Pp3c7_21880V3.1
locus=Pp3c7_21880 annot-version=v3.3	Physcomitrella_patens
OG0000010
Pp3c7_17410V3.1.p pacid=32923495 transcript=Pp3c7_17410V3.1
locus=Pp3c7_17410 annot-version=v3.3	Physcomitrella_patens
OG0000010
Pp3c7_25700V3.1.p pacid=32923565 transcript=Pp3c7_25700V3.1
locus=Pp3c7_25700 annot-version=v3.3	Physcomitrella_patens
OG0000010
Pp3c7_4440V3.1.p pacid=32923573 transcript=Pp3c7_4440V3.1
locus=Pp3c7_4440 annot-version=v3.3	Physcomitrella_patens
OG0000010
Pp3c7_15660V3.1.p pacid=32923958 transcript=Pp3c7_15660V3.1
locus=Pp3c7_15660 annot-version=v3.3	Physcomitrella_patens
OG0000010
Pp3c7_21870V3.1.p pacid=32924164 transcript=Pp3c7_21870V3.1
locus=Pp3c7_21870 annot-version=v3.3	Physcomitrella_patens
OG0000010
Pp3c7_21090V3.1.p pacid=32924456 transcript=Pp3c7_21090V3.1
locus=Pp3c7_21090 annot-version=v3.3	Physcomitrella_patens
OG0000010
Pp3c7_26610V3.1.p pacid=32924549 transcript=Pp3c7_26610V3.1
locus=Pp3c7_26610 annot-version=v3.3	Physcomitrella_patens
OG0000010
Pp3c7_17420V3.1.p pacid=32924562 transcript=Pp3c7_17420V3.1
locus=Pp3c7_17420 annot-version=v3.3	Physcomitrella_patens
OG0000010
Pp3c7_4140V3.1.p pacid=32924577 transcript=Pp3c7_4140V3.1
locus=Pp3c7_4140 annot-version=v3.3	Physcomitrella_patens
OG0000010
Pp3c7_13170V3.1.p pacid=32924918 transcript=Pp3c7_13170V3.1
locus=Pp3c7_13170 annot-version=v3.3	Physcomitrella_patens
OG0000010
Pp3c10_16310V3.1.p pacid=32899598 transcript=Pp3c10_16310V3.1
locus=Pp3c10_16310 annot-version=v3.3	Physcomitrella_patens
OG0000010
Pp3c7_17400V3.1.p pacid=32925330 transcript=Pp3c7_17400V3.1
locus=Pp3c7_17400 annot-version=v3.3	Physcomitrella_patens
OG0000010
Pp3c7_21388V3.1.p pacid=32925352 transcript=Pp3c7_21388V3.1
locus=Pp3c7_21388 annot-version=v3.3	Physcomitrella_patens
OG0000010
Pp3c7_21000V3.1.p pacid=32925514 transcript=Pp3c7_21000V3.1

locus=Pp3c7_21000 annot-version=v3.3 Physcomitrella_patens OG0000010
Pp3c7_21100V3.1.p pacid=32925853 transcript=Pp3c7_21100V3.1 locus=Pp3c7_21100 annot-version=v3.3 Physcomitrella_patens OG0000010
Pp3c7_500V3.1.p pacid=32925987 transcript=Pp3c7_500V3.1 locus=Pp3c7_500 annot-version=v3.3 Physcomitrella_patens OG0000010
AT4G19050.1 | NB-ARC domain-containing disease resistance protein | Chr4:10440102-10443786 REVERSE LENGTH=1201 | 201606 Arabidopsis_thaliana OG0000010
Os01t0536466-00 Conserved hypothetical protein. Oryza_sativa OG0000010
Os09t0327575-00 Similar to Pi5-1. Oryza_sativa OG0000010
Os10t0507150-00 Similar to Leucine Rich Repeat family protein, expressed. Oryza_sativa OG0000010
Os11t0211600-00 Similar to Leucine Rich Repeat family protein. Oryza_sativa OG0000010
Os11t0213700-00 Leucine-rich repeat, typical subtype containing protein. Oryza_sativa OG0000010
Os02t0150600-01 Similar to Pyridine nucleotide-disulphide oxidoreductase (Fragment). Oryza_sativa OG0000010
Os02t0597300-01 Leucine-rich repeat domain containing protein. Oryza_sativa OG0000010
CDF33522 pep supercontig:ASM35022v2:HG001649:8133:9620:1 gene:CHC_T00009076001 transcript:CDF33522 gene_biotype:protein_coding transcript_biotype:protein_coding description:Serine/threonine protein kinase Chondrus_crispus OG0000010
CDF39122 pep supercontig:ASM35022v2:HG002002:413379:415624:1 gene:CHC_T00008767001 transcript:CDF39122 gene_biotype:protein_coding transcript_biotype:protein_coding description:Serine/threonine protein kinase, Rhodoplastic Chondrus_crispus OG0000010
CDF37245 pep supercontig:ASM35022v2:HG001822:36187:36698:1 gene:CHC_T00005367001 transcript:CDF37245 gene_biotype:protein_coding transcript_biotype:protein_coding Chondrus_crispus OG0000010
g2353.t1 Caulerpa_lentillifera OG0000011
g6391.t1 Caulerpa_lentillifera OG0000011
g8442.t1 Caulerpa_lentillifera OG0000011
g8489.t1 Caulerpa_lentillifera OG0000011
g9237.t1 Caulerpa_lentillifera OG0000011
g9261.t1 Caulerpa_lentillifera OG0000011
Cre02.g103450.t1.1 pacid=30784922 transcript=Cre02.g103450.t1.1 locus=Cre02.g103450 ID=Cre02.g103450.t1.1.v5.5 annot-version=v5.5 Chlamydomonas_reinhardtii OG0000011
Cre02.g110550.t1.1 pacid=30785972 transcript=Cre02.g110550.t1.1 locus=Cre02.g110550 ID=Cre02.g110550.t1.1.v5.5 annot-version=v5.5 Chlamydomonas_reinhardtii OG0000011
Cre02.g108350.t1.2 pacid=30786333 transcript=Cre02.g108350.t1.2 locus=Cre02.g108350 ID=Cre02.g108350.t1.2.v5.5 annot-version=v5.5 Chlamydomonas_reinhardtii OG0000011
Cre03.g149201.t1.1 pacid=30786565 transcript=Cre03.g149201.t1.1

locus=Cre03.g149201 ID=Cre03.g149201.t1.1.v5.5 annot-version=v5.5
Chlamydomonas_reinhardtii OG0000011
Cre03.g197100.t1.2 pacid=30787755 transcript=Cre03.g197100.t1.2
locus=Cre03.g197100 ID=Cre03.g197100.t1.2.v5.5 annot-version=v5.5
Chlamydomonas_reinhardtii OG0000011
Cre03.g144747.t1.1 pacid=30787894 transcript=Cre03.g144747.t1.1
locus=Cre03.g144747 ID=Cre03.g144747.t1.1.v5.5 annot-version=v5.5
Chlamydomonas_reinhardtii OG0000011
Cre01.g034350.t1.1 pacid=30789393 transcript=Cre01.g034350.t1.1
locus=Cre01.g034350 ID=Cre01.g034350.t1.1.v5.5 annot-version=v5.5
Chlamydomonas_reinhardtii OG0000011
Cre04.g217917.t1.1 pacid=30791099 transcript=Cre04.g217917.t1.1
locus=Cre04.g217917 ID=Cre04.g217917.t1.1.v5.5 annot-version=v5.5
Chlamydomonas_reinhardtii OG0000011
Cre07.g345350.t1.1 pacid=30775183 transcript=Cre07.g345350.t1.1
locus=Cre07.g345350 ID=Cre07.g345350.t1.1.v5.5 annot-version=v5.5
Chlamydomonas_reinhardtii OG0000011
Cre12.g522400.t1.1 pacid=30793439 transcript=Cre12.g522400.t1.1
locus=Cre12.g522400 ID=Cre12.g522400.t1.1.v5.5 annot-version=v5.5
Chlamydomonas_reinhardtii OG0000011
Cre14.g621050.t1.1 pacid=30776401 transcript=Cre14.g621050.t1.1
locus=Cre14.g621050 ID=Cre14.g621050.t1.1.v5.5 annot-version=v5.5
Chlamydomonas_reinhardtii OG0000011
Cre16.g677382.t1.1 pacid=30777694 transcript=Cre16.g677382.t1.1
locus=Cre16.g677382 ID=Cre16.g677382.t1.1.v5.5 annot-version=v5.5
Chlamydomonas_reinhardtii OG0000011
Cre06.g289900.t1.1 pacid=30779250 transcript=Cre06.g289900.t1.1
locus=Cre06.g289900 ID=Cre06.g289900.t1.1.v5.5 annot-version=v5.5
Chlamydomonas_reinhardtii OG0000011
Cre09.g399067.t1.1 pacid=30780866 transcript=Cre09.g399067.t1.1
locus=Cre09.g399067 ID=Cre09.g399067.t1.1.v5.5 annot-version=v5.5
Chlamydomonas_reinhardtii OG0000011
Cre09.g397475.t1.1 pacid=30781407 transcript=Cre09.g397475.t1.1
locus=Cre09.g397475 ID=Cre09.g397475.t1.1.v5.5 annot-version=v5.5
Chlamydomonas_reinhardtii OG0000011
Cre17.g714229.t1.2 pacid=30781658 transcript=Cre17.g714229.t1.2
locus=Cre17.g714229 ID=Cre17.g714229.t1.2.v5.5 annot-version=v5.5
Chlamydomonas_reinhardtii OG0000011
Vocar.0012s0162.1.p pacid=32894771 transcript=Vocar.0012s0162.1
locus=Vocar.0012s0162 ID=Vocar.0012s0162.1.v2.1 annot-version=v2.1
Volvox_carteri OG0000011
Vocar.0011s0012.1.p pacid=32895322 transcript=Vocar.0011s0012.1
locus=Vocar.0011s0012 ID=Vocar.0011s0012.1.v2.1 annot-version=v2.1
Volvox_carteri OG0000011
Vocar.0011s0308.1.p pacid=32895391 transcript=Vocar.0011s0308.1
locus=Vocar.0011s0308 ID=Vocar.0011s0308.1.v2.1 annot-version=v2.1
Volvox_carteri OG0000011
Vocar.0011s0039.1.p pacid=32895431 transcript=Vocar.0011s0039.1
locus=Vocar.0011s0039 ID=Vocar.0011s0039.1.v2.1 annot-version=v2.1
Volvox_carteri OG0000011
Vocar.0013s0008.1.p pacid=32896182 transcript=Vocar.0013s0008.1
locus=Vocar.0013s0008 ID=Vocar.0013s0008.1.v2.1 annot-version=v2.1
Volvox_carteri OG0000011
Vocar.0008s0006.1.p pacid=32897110 transcript=Vocar.0008s0006.1

locus=Vocar.0008s0006 ID=Vocar.0008s0006.1.v2.1 annot-version=v2.1
Volvox_carteri   OG0000011
Vocar.0001s1620.1.p pacid=32884611 transcript=Vocar.0001s1620.1
locus=Vocar.0001s1620 ID=Vocar.0001s1620.1.v2.1 annot-version=v2.1
Volvox_carteri   OG0000011
Vocar.0023s0018.1.p pacid=32883402 transcript=Vocar.0023s0018.1
locus=Vocar.0023s0018 ID=Vocar.0023s0018.1.v2.1 annot-version=v2.1
Volvox_carteri   OG0000011
Vocar.0001s0782.1.p pacid=32885751 transcript=Vocar.0001s0782.1
locus=Vocar.0001s0782 ID=Vocar.0001s0782.1.v2.1 annot-version=v2.1
Volvox_carteri   OG0000011
Vocar.0001s1594.1.p pacid=32886130 transcript=Vocar.0001s1594.1
locus=Vocar.0001s1594 ID=Vocar.0001s1594.1.v2.1 annot-version=v2.1
Volvox_carteri   OG0000011
Vocar.0014s0065.1.p pacid=32888566 transcript=Vocar.0014s0065.1
locus=Vocar.0014s0065 ID=Vocar.0014s0065.1.v2.1 annot-version=v2.1
Volvox_carteri   OG0000011
Vocar.0004s0316.1.p pacid=32889595 transcript=Vocar.0004s0316.1
locus=Vocar.0004s0316 ID=Vocar.0004s0316.1.v2.1 annot-version=v2.1
Volvox_carteri   OG0000011
Vocar.0024s0002.1.p pacid=32890239 transcript=Vocar.0024s0002.1
locus=Vocar.0024s0002 ID=Vocar.0024s0002.1.v2.1 annot-version=v2.1
Volvox_carteri   OG0000011
Vocar.0002s0488.1.p pacid=32891022 transcript=Vocar.0002s0488.1
locus=Vocar.0002s0488 ID=Vocar.0002s0488.1.v2.1 annot-version=v2.1
Volvox_carteri   OG0000011
Vocar.0017s0204.1.p pacid=32891362 transcript=Vocar.0017s0204.1
locus=Vocar.0017s0204 ID=Vocar.0017s0204.1.v2.1 annot-version=v2.1
Volvox_carteri   OG0000011
Vocar.0026s0128.1.p pacid=32892479 transcript=Vocar.0026s0128.1
locus=Vocar.0026s0128 ID=Vocar.0026s0128.1.v2.1 annot-version=v2.1
Volvox_carteri   OG0000011
Vocar.0009s0221.1.p pacid=32893145 transcript=Vocar.0009s0221.1
locus=Vocar.0009s0221 ID=Vocar.0009s0221.1.v2.1 annot-version=v2.1
Volvox_carteri   OG0000011
Vocar.0006s0441.1.p pacid=32894082 transcript=Vocar.0006s0441.1
locus=Vocar.0006s0441 ID=Vocar.0006s0441.1.v2.1 annot-version=v2.1
Volvox_carteri   OG0000011
jgi|ChlNC64A_1|20303|e_gw1.4.453.1   Chlorella_variabilis
OG0000011
jgi|ChlNC64A_1|15266|gw1.4.455.1 Chlorella_variabilis
OG0000011
jgi|ChlNC64A_1|143110|IGS.gm_5_00439   Chlorella_variabilis
OG0000011
jgi|ChlNC64A_1|11906|gw1.8.222.1 Chlorella_variabilis
OG0000011
jgi|ChlNC64A_1|13979|gw1.8.311.1 Chlorella_variabilis
OG0000011
jgi|ChlNC64A_1|13490|gw1.35.52.1 Chlorella_variabilis
OG0000011
jgi|ChlNC64A_1|14655|gw1.28.76.1 Chlorella_variabilis
OG0000011
46919 pacid=27387319 transcript=46919
locus=estExt_fgenesh1_pg.C_50239 ID=46919.2.0.227 annot-version=v2.0

Coccomyxa_subellipsoidea OG0000011
35694 pacid=27389870 transcript=35694 locus=fgenesh1_pm.4_#_96
ID=35694.2.0.227 annot-version=v2.0 Coccomyxa_subellipsoidea
OG0000011
9603 pacid=27390084 transcript=9603 locus=gw1.4.828.1
ID=9603.2.0.227 annot-version=v2.0 Coccomyxa_subellipsoidea
OG0000011
40767 pacid=27390104 transcript=40767 locus=fgenesh1_pg.4_#_416
ID=40767.2.0.227 annot-version=v2.0 Coccomyxa_subellipsoidea
OG0000011
12896 pacid=27390487 transcript=12896 locus=e_gw1.3.530.1
ID=12896.2.0.227 annot-version=v2.0 Coccomyxa_subellipsoidea
OG0000011
7682 pacid=27385729 transcript=7682 locus=gw1.10.406.1
ID=7682.2.0.227 annot-version=v2.0 Coccomyxa_subellipsoidea
OG0000011
13789_fgenesh1_pg.C_Chr_15.0001000108 Ostreococcus_tauri
OG0000011
19252_e_gw1.09.00.361.1 Ostreococcus_tauri OG0000011
20904_e_gw1.13.00.306.1 Ostreococcus_tauri OG0000011
21985_e_gw1.16.00.288.1 Ostreococcus_tauri OG0000011
32813_0500010178 Ostreococcus_tauri OG0000011
6990_gw1.10.00.387.1 Ostreococcus_tauri OG0000011
7799_gw1.05.00.538.1 Ostreococcus_tauri OG0000011
8749 pacid=27416071 transcript=8749 locus=gwEuk.10.443.1
ID=8749.2.0.231 annot-version=v2.0 Ostreococcus_lucimarinus
OG0000011
9540 pacid=27416739 transcript=9540 locus=gwEuk.5.576.1
ID=9540.2.0.231 annot-version=v2.0 Ostreococcus_lucimarinus
OG0000011
9737 pacid=27416800 transcript=9737 locus=gwEuk.5.591.1
ID=9737.2.0.231 annot-version=v2.0 Ostreococcus_lucimarinus
OG0000011
40769 pacid=27417388 transcript=40769 locus=e_gwEuk.15.293.1
ID=40769.2.0.231 annot-version=v2.0 Ostreococcus_lucimarinus
OG0000011
9404 pacid=27418839 transcript=9404 locus=gwEuk.13.411.1
ID=9404.2.0.231 annot-version=v2.0 Ostreococcus_lucimarinus
OG0000011
41981 pacid=27419034 transcript=41981 locus=e_gwEuk.17.213.1
ID=41981.2.0.231 annot-version=v2.0 Ostreococcus_lucimarinus
OG0000011
47583 pacid=27419640 transcript=47583
locus=estExt_GenewiseEukaryote.C_Chr_140435 ID=47583.2.0.231 annot-
version=v2.0 Ostreococcus_lucimarinus OG0000011
43387 pacid=27419958 transcript=43387 locus=e_gwEuk.14.429.1
ID=43387.2.0.231 annot-version=v2.0 Ostreococcus_lucimarinus
OG0000011
34891 pacid=27413604 transcript=34891 locus=e_gwEuk.9.410.1
ID=34891.2.0.231 annot-version=v2.0 Ostreococcus_lucimarinus
OG0000011
133654 pacid=27342233 transcript=133694 locus=gw1.14.192.1
ID=133694.3.0.228 annot-version=v3.0 Micromonas_pusilla
OG0000011

55785 pacid=27342588 transcript=55785 locus=MicpuC2.EuGene.
0000020814 ID=55785.3.0.228 annot-version=v3.0
Micromonas_pusilla OG0000011
122488 pacid=27342841 transcript=122528 locus=fgenesh2_kg.
2_#_192_#_4271075:1 ID=122528.3.0.228 annot-version=v3.0
Micromonas_pusilla OG0000011
193230 pacid=27346216 transcript=193270
locus=estExt_Genemark1.C_4_t10036 ID=193270.3.0.228 annot-
version=v3.0 Micromonas_pusilla OG0000011
130146 pacid=27339433 transcript=130186 locus=gw1.11.74.1
ID=130186.3.0.228 annot-version=v3.0 Micromonas_pusilla
OG0000011
192902 pacid=27347065 transcript=192942
locus=estExt_Genemark1.C_3_t10237 ID=192942.3.0.228 annot-
version=v3.0 Micromonas_pusilla OG0000011
9060 pacid=27347405 transcript=9060 locus=MicpuC2.gw1.3.920.1
ID=9060.3.0.228 annot-version=v3.0 Micromonas_pusilla
OG0000011
39026 pacid=27347507 transcript=39026
locus=MicpuC2.fgenesh1_pg.C_scaffold_3000533 ID=39026.3.0.228 annot-
version=v3.0 Micromonas_pusilla OG0000011
74650 pacid=27396311 transcript=74650 locus=gw2.02.861.1
ID=74650.3.0.229 annot-version=v3.0 Micromonas_sp._RCC299
OG0000011
74639 pacid=27396995 transcript=74639 locus=gw2.16.261.1
ID=74639.3.0.229 annot-version=v3.0 Micromonas_sp._RCC299
OG0000011
80246 pacid=27398523 transcript=80246 locus=e_gw2.03.717.1
ID=80246.3.0.229 annot-version=v3.0 Micromonas_sp._RCC299
OG0000011
113455 pacid=27398926 transcript=113455 locus=MSI_PierreAnnot2.00118
ID=113455.3.0.229 annot-version=v3.0 Micromonas_sp._RCC299
OG0000011
55061 pacid=27399118 transcript=55061 locus=EuGene.0100010288
ID=55061.3.0.229 annot-version=v3.0 Micromonas_sp._RCC299
OG0000011
73264 pacid=27395660 transcript=73264 locus=gw2.05.651.1
ID=73264.3.0.229 annot-version=v3.0 Micromonas_sp._RCC299
OG0000011
105509 pacid=27400523 transcript=105509
locus=estExt_fgenesh2_pg.C_Chr_040472 ID=105509.3.0.229 annot-
version=v3.0 Micromonas_sp._RCC299 OG0000011
73784 pacid=27400947 transcript=73784 locus=gw2.15.275.1
ID=73784.3.0.229 annot-version=v3.0 Micromonas_sp._RCC299
OG0000011
88104 pacid=27403928 transcript=88104 locus=e_gw2.14.322.1
ID=88104.3.0.229 annot-version=v3.0 Micromonas_sp._RCC299
OG0000011
83790 pacid=27404353 transcript=83790 locus=e_gw2.07.474.1
ID=83790.3.0.229 annot-version=v3.0 Micromonas_sp._RCC299
OG0000011
kfl00455_0010_v1.1 Klebsormidium_flaccidum OG0000011
kfl00455_0030_v1.1 Klebsormidium_flaccidum OG0000011
kfl00480_0040_v1.1 Klebsormidium_flaccidum OG0000011

kfl00493_0040_v1.1   Klebsormidium_flaccidum OG0000011
kfl00565_0070_v1.1   Klebsormidium_flaccidum OG0000011
kfl00698_0010_v1.1   Klebsormidium_flaccidum OG0000011
kfl00779_0010_v1.1   Klebsormidium_flaccidum OG0000011
kfl00793_0050_v1.1   Klebsormidium_flaccidum OG0000011
kfl00937_0030_v1.1   Klebsormidium_flaccidum OG0000011
kfl00026_0540_v1.1   Klebsormidium_flaccidum OG0000011
kfl00040_0130_v1.1   Klebsormidium_flaccidum OG0000011
kfl00048_0440_v1.1   Klebsormidium_flaccidum OG0000011
kfl00052_0040_v1.1   Klebsormidium_flaccidum OG0000011
kfl00064_0290_v1.1   Klebsormidium_flaccidum OG0000011
kfl00085_0320_v1.1   Klebsormidium_flaccidum OG0000011
kfl00097_0190_v1.1   Klebsormidium_flaccidum OG0000011
kfl00279_0100_v1.1   Klebsormidium_flaccidum OG0000011
Pp3c15_24080V3.1.p pacid=32928240 transcript=Pp3c15_24080V3.1 locus=Pp3c15_24080 annot-version=v3.3   Physcomitrella_patens OG0000011
Pp3c10_12410V3.1.p pacid=32902390 transcript=Pp3c10_12410V3.1 locus=Pp3c10_12410 annot-version=v3.3   Physcomitrella_patens OG0000011
Pp3c15_1270V3.1.p pacid=32928940 transcript=Pp3c15_1270V3.1 locus=Pp3c15_1270 annot-version=v3.3   Physcomitrella_patens OG0000011
Pp3c15_7790V3.1.p pacid=32929326 transcript=Pp3c15_7790V3.1 locus=Pp3c15_7790 annot-version=v3.3   Physcomitrella_patens OG0000011
Pp3c15_15960V3.1.p pacid=32929422 transcript=Pp3c15_15960V3.1 locus=Pp3c15_15960 annot-version=v3.3   Physcomitrella_patens OG0000011
Pp3c15_19246V3.1.p pacid=32929746 transcript=Pp3c15_19246V3.1 locus=Pp3c15_19246 annot-version=v3.3   Physcomitrella_patens OG0000011
Pp3c13_8880V3.1.p pacid=32931871 transcript=Pp3c13_8880V3.1 locus=Pp3c13_8880 annot-version=v3.3   Physcomitrella_patens OG0000011
Pp3c13_5070V3.1.p pacid=32932114 transcript=Pp3c13_5070V3.1 locus=Pp3c13_5070 annot-version=v3.3   Physcomitrella_patens OG0000011
Pp3c2_37040V3.1.p pacid=32934223 transcript=Pp3c2_37040V3.1 locus=Pp3c2_37040 annot-version=v3.3   Physcomitrella_patens OG0000011
Pp3c2_31770V3.1.p pacid=32935178 transcript=Pp3c2_31770V3.1 locus=Pp3c2_31770 annot-version=v3.3   Physcomitrella_patens OG0000011
Pp3c2_34670V3.1.p pacid=32936917 transcript=Pp3c2_34670V3.1 locus=Pp3c2_34670 annot-version=v3.3   Physcomitrella_patens OG0000011
Pp3c19_20750V3.1.p pacid=32938099 transcript=Pp3c19_20750V3.1 locus=Pp3c19_20750 annot-version=v3.3   Physcomitrella_patens OG0000011
Pp3c3_12080V3.1.p pacid=32941431 transcript=Pp3c3_12080V3.1 locus=Pp3c3_12080 annot-version=v3.3   Physcomitrella_patens OG0000011
Pp3c3_19360V3.1.p pacid=32944301 transcript=Pp3c3_19360V3.1

locus=Pp3c3_19360 annot-version=v3.3	Physcomitrella_patens
OG0000011
Pp3c20_8950V3.1.p pacid=32946693 transcript=Pp3c20_8950V3.1
locus=Pp3c20_8950 annot-version=v3.3	Physcomitrella_patens
OG0000011
Pp3c23_7530V3.1.p pacid=32950462 transcript=Pp3c23_7530V3.1
locus=Pp3c23_7530 annot-version=v3.3	Physcomitrella_patens
OG0000011
Pp3c5_2150V3.1.p pacid=32952664 transcript=Pp3c5_2150V3.1
locus=Pp3c5_2150 annot-version=v3.3	Physcomitrella_patens
OG0000011
Pp3c5_7650V3.1.p pacid=32953753 transcript=Pp3c5_7650V3.1
locus=Pp3c5_7650 annot-version=v3.3	Physcomitrella_patens
OG0000011
Pp3c11_10350V3.1.p pacid=32956501 transcript=Pp3c11_10350V3.1
locus=Pp3c11_10350 annot-version=v3.3	Physcomitrella_patens
OG0000011
Pp3c11_5420V3.1.p pacid=32957683 transcript=Pp3c11_5420V3.1
locus=Pp3c11_5420 annot-version=v3.3	Physcomitrella_patens
OG0000011
Pp3c11_5410V3.1.p pacid=32959241 transcript=Pp3c11_5410V3.1
locus=Pp3c11_5410 annot-version=v3.3	Physcomitrella_patens
OG0000011
Pp3c11_2650V3.1.p pacid=32959251 transcript=Pp3c11_2650V3.1
locus=Pp3c11_2650 annot-version=v3.3	Physcomitrella_patens
OG0000011
Pp3c14_14040V3.1.p pacid=32959862 transcript=Pp3c14_14040V3.1
locus=Pp3c14_14040 annot-version=v3.3	Physcomitrella_patens
OG0000011
Pp3c14_12390V3.1.p pacid=32960818 transcript=Pp3c14_12390V3.1
locus=Pp3c14_12390 annot-version=v3.3	Physcomitrella_patens
OG0000011
Pp3c14_14030V3.1.p pacid=32962956 transcript=Pp3c14_14030V3.1
locus=Pp3c14_14030 annot-version=v3.3	Physcomitrella_patens
OG0000011
Pp3c17_12730V3.1.p pacid=32906006 transcript=Pp3c17_12730V3.1
locus=Pp3c17_12730 annot-version=v3.3	Physcomitrella_patens
OG0000011
Pp3c17_7730V3.1.p pacid=32906138 transcript=Pp3c17_7730V3.1
locus=Pp3c17_7730 annot-version=v3.3	Physcomitrella_patens
OG0000011
Pp3c17_19780V3.1.p pacid=32906199 transcript=Pp3c17_19780V3.1
locus=Pp3c17_19780 annot-version=v3.3	Physcomitrella_patens
OG0000011
Pp3c1_1650V3.1.p pacid=32968033 transcript=Pp3c1_1650V3.1
locus=Pp3c1_1650 annot-version=v3.3	Physcomitrella_patens
OG0000011
Pp3c1_21610V3.1.p pacid=32969503 transcript=Pp3c1_21610V3.1
locus=Pp3c1_21610 annot-version=v3.3	Physcomitrella_patens
OG0000011
Pp3c1_42920V3.1.p pacid=32970379 transcript=Pp3c1_42920V3.1
locus=Pp3c1_42920 annot-version=v3.3	Physcomitrella_patens
OG0000011
Pp3c1_4970V3.1.p pacid=32971501 transcript=Pp3c1_4970V3.1

locus=Pp3c1_4970 annot-version=v3.3 Physcomitrella_patens
OG0000011
Pp3c12_10360V3.1.p pacid=32973451 transcript=Pp3c12_10360V3.1
locus=Pp3c12_10360 annot-version=v3.3 Physcomitrella_patens
OG0000011
Pp3c12_17180V3.1.p pacid=32974833 transcript=Pp3c12_17180V3.1
locus=Pp3c12_17180 annot-version=v3.3 Physcomitrella_patens
OG0000011
Pp3c6_9970V3.1.p pacid=32975382 transcript=Pp3c6_9970V3.1
locus=Pp3c6_9970 annot-version=v3.3 Physcomitrella_patens
OG0000011
Pp3c17_19947V3.1.p pacid=32907368 transcript=Pp3c17_19947V3.1
locus=Pp3c17_19947 annot-version=v3.3 Physcomitrella_patens
OG0000011
Pp3c6_23360V3.1.p pacid=32978497 transcript=Pp3c6_23360V3.1
locus=Pp3c6_23360 annot-version=v3.3 Physcomitrella_patens
OG0000011
Pp3c6_24650V3.1.p pacid=32978792 transcript=Pp3c6_24650V3.1
locus=Pp3c6_24650 annot-version=v3.3 Physcomitrella_patens
OG0000011
Pp3c25_3170V3.1.p pacid=32979228 transcript=Pp3c25_3170V3.1
locus=Pp3c25_3170 annot-version=v3.3 Physcomitrella_patens
OG0000011
Pp3c18_340V3.1.p pacid=32982416 transcript=Pp3c18_340V3.1
locus=Pp3c18_340 annot-version=v3.3 Physcomitrella_patens
OG0000011
Pp3c16_9970V3.1.p pacid=32983668 transcript=Pp3c16_9970V3.1
locus=Pp3c16_9970 annot-version=v3.3 Physcomitrella_patens
OG0000011
Pp3c16_10020V3.1.p pacid=32985286 transcript=Pp3c16_10020V3.1
locus=Pp3c16_10020 annot-version=v3.3 Physcomitrella_patens
OG0000011
Pp3c9_15970V3.1.p pacid=32911991 transcript=Pp3c9_15970V3.1
locus=Pp3c9_15970 annot-version=v3.3 Physcomitrella_patens
OG0000011
Pp3c9_15975V3.1.p pacid=32913824 transcript=Pp3c9_15975V3.1
locus=Pp3c9_15975 annot-version=v3.3 Physcomitrella_patens
OG0000011
Pp3c9_24170V3.1.p pacid=32913935 transcript=Pp3c9_24170V3.1
locus=Pp3c9_24170 annot-version=v3.3 Physcomitrella_patens
OG0000011
Pp3c9_10290V3.1.p pacid=32914107 transcript=Pp3c9_10290V3.1
locus=Pp3c9_10290 annot-version=v3.3 Physcomitrella_patens
OG0000011
Pp3c10_10240V3.1.p pacid=32901035 transcript=Pp3c10_10240V3.1
locus=Pp3c10_10240 annot-version=v3.3 Physcomitrella_patens
OG0000011
Pp3c26_13600V3.1.p pacid=32917015 transcript=Pp3c26_13600V3.1
locus=Pp3c26_13600 annot-version=v3.3 Physcomitrella_patens
OG0000011
Pp3c26_1390V3.1.p pacid=32918557 transcript=Pp3c26_1390V3.1
locus=Pp3c26_1390 annot-version=v3.3 Physcomitrella_patens
OG0000011
Pp3c4_13790V3.1.p pacid=32918821 transcript=Pp3c4_13790V3.1

locus=Pp3c4_13790 annot-version=v3.3 Physcomitrella_patens
OG0000011
Pp3c4_12510V3.1.p pacid=32922072 transcript=Pp3c4_12510V3.1
locus=Pp3c4_12510 annot-version=v3.3 Physcomitrella_patens
OG0000011
Pp3c7_23450V3.1.p pacid=32923058 transcript=Pp3c7_23450V3.1
locus=Pp3c7_23450 annot-version=v3.3 Physcomitrella_patens
OG0000011
Pp3c7_21220V3.1.p pacid=32924693 transcript=Pp3c7_21220V3.1
locus=Pp3c7_21220 annot-version=v3.3 Physcomitrella_patens
OG0000011
Pp3c7_23490V3.1.p pacid=32925199 transcript=Pp3c7_23490V3.1
locus=Pp3c7_23490 annot-version=v3.3 Physcomitrella_patens
OG0000011
AT2G36890.1 | Duplicated homeodomain-like superfamily protein |
Chr2:15485821-15487245 FORWARD LENGTH=298 | 201606
Arabidopsis_thaliana OG0000011
AT2G39880.1 | myb domain protein 25 | Chr2:16647945-16649479 REVERSE
LENGTH=367 | 201606 Arabidopsis_thaliana OG0000011
AT2G47190.1 | myb domain protein 2 | Chr2:19376284-19377297 FORWARD
LENGTH=273 | 201606 Arabidopsis_thaliana OG0000011
AT2G47460.1 | myb domain protein 12 | Chr2:19476438-19479242 FORWARD
LENGTH=371 | 201606 Arabidopsis_thaliana OG0000011
AT3G01140.1 | myb domain protein 106 | Chr3:46619-48143 REVERSE
LENGTH=388 | 201606 Arabidopsis_thaliana OG0000011
AT3G01530.1 | myb domain protein 57 | Chr3:210352-211151 REVERSE
LENGTH=206 | 201606 Arabidopsis_thaliana OG0000011
AT3G02940.1 | myb domain protein 107 | Chr3:662141-663830 FORWARD
LENGTH=321 | 201606 Arabidopsis_thaliana OG0000011
AT3G06490.1 | myb domain protein 108 | Chr3:2004298-2006358 FORWARD
LENGTH=323 | 201606 Arabidopsis_thaliana OG0000011
AT3G08500.1 | myb domain protein 83 | Chr3:2576958-2578072 REVERSE
LENGTH=343 | 201606 Arabidopsis_thaliana OG0000011
AT3G09230.1 | myb domain protein 1 | Chr3:2833460-2835134 FORWARD
LENGTH=393 | 201606 Arabidopsis_thaliana OG0000011
AT3G09370.1 | myb domain protein 3r-3 | Chr3:2879534-2882128 FORWARD
LENGTH=505 | 201606 Arabidopsis_thaliana OG0000011
AT3G11440.1 | myb domain protein 65 | Chr3:3603056-3604929 FORWARD
LENGTH=553 | 201606 Arabidopsis_thaliana OG0000011
AT3G12720.1 | myb domain protein 67 | Chr3:4043407-4044522 REVERSE
LENGTH=307 | 201606 Arabidopsis_thaliana OG0000011
AT3G12820.1 | myb domain protein 10 | Chr3:4074328-4075614 REVERSE
LENGTH=239 | 201606 Arabidopsis_thaliana OG0000011
AT3G13540.1 | myb domain protein 5 | Chr3:4420239-4421443 FORWARD
LENGTH=249 | 201606 Arabidopsis_thaliana OG0000011
AT3G13890.1 | myb domain protein 26 | Chr3:4576744-4578027 REVERSE
LENGTH=367 | 201606 Arabidopsis_thaliana OG0000011
AT3G18100.1 | myb domain protein 4r1 | Chr3:6200689-6204583 FORWARD
LENGTH=847 | 201606 Arabidopsis_thaliana OG0000011
AT3G23250.1 | myb domain protein 15 | Chr3:8309492-8310624 FORWARD
LENGTH=285 | 201606 Arabidopsis_thaliana OG0000011
AT3G24310.1 | myb domain protein 305 | Chr3:8811336-8812330 REVERSE
LENGTH=269 | 201606 Arabidopsis_thaliana OG0000011
AT1G14350.1 | Duplicated homeodomain-like superfamily protein |

Chr1:4908427-4910647 FORWARD LENGTH=436 | 201606
Arabidopsis_thaliana   OG0000011
AT3G27785.1 | myb domain protein 118 | Chr3:10288767-10290712
REVERSE LENGTH=437 | 201606   Arabidopsis_thaliana
OG0000011
AT3G27810.1 | myb domain protein 21 | Chr3:10307596-10310093 FORWARD
LENGTH=226 | 201606   Arabidopsis_thaliana   OG0000011
AT3G27920.1 | myb domain protein 0 | Chr3:10361945-10363506 REVERSE
LENGTH=228 | 201606   Arabidopsis_thaliana   OG0000011
AT3G28470.1 | Duplicated homeodomain-like superfamily protein |
Chr3:10674579-10675724 REVERSE LENGTH=317 | 201606
Arabidopsis_thaliana   OG0000011
AT3G28910.1 | myb domain protein 30 | Chr3:10911443-10912856 FORWARD
LENGTH=323 | 201606   Arabidopsis_thaliana   OG0000011
AT3G29020.1 | myb domain protein 110 | Chr3:11008614-11009413
REVERSE LENGTH=233 | 201606   Arabidopsis_thaliana
OG0000011
AT3G30210.1 | myb domain protein 121 | Chr3:11838567-11840313
FORWARD LENGTH=276 | 201606   Arabidopsis_thaliana
OG0000011
AT3G46130.1 | myb domain protein 48 | Chr3:16945427-16946376 FORWARD
LENGTH=258 | 201606   Arabidopsis_thaliana   OG0000011
AT3G47600.1 | myb domain protein 94 | Chr3:17539627-17541035 REVERSE
LENGTH=333 | 201606   Arabidopsis_thaliana   OG0000011
AT3G48920.1 | myb domain protein 45 | Chr3:18139392-18140561 FORWARD
LENGTH=261 | 201606   Arabidopsis_thaliana   OG0000011
AT3G49690.1 | myb domain protein 84 | Chr3:18427941-18429100 FORWARD
LENGTH=310 | 201606   Arabidopsis_thaliana   OG0000011
AT3G50060.1 | myb domain protein 77 | Chr3:18558146-18559051 REVERSE
LENGTH=301 | 201606   Arabidopsis_thaliana   OG0000011
AT3G53200.1 | myb domain protein 27 | Chr3:19718426-19719394 REVERSE
LENGTH=238 | 201606   Arabidopsis_thaliana   OG0000011
AT3G55730.1 | myb domain protein 109 | Chr3:20682114-20683963
REVERSE LENGTH=399 | 201606   Arabidopsis_thaliana
OG0000011
AT1G16490.1 | myb domain protein 58 | Chr1:5629859-5630844 REVERSE
LENGTH=274 | 201606   Arabidopsis_thaliana   OG0000011
AT3G61250.1 | myb domain protein 17 | Chr3:22671306-22672551 FORWARD
LENGTH=299 | 201606   Arabidopsis_thaliana   OG0000011
AT3G62610.1 | myb domain protein 11 | Chr3:23154758-23156371 FORWARD
LENGTH=343 | 201606   Arabidopsis_thaliana   OG0000011
AT4G00540.1 | myb domain protein 3r2 | Chr4:234597-237356 REVERSE
LENGTH=437 | 201606   Arabidopsis_thaliana   OG0000011
AT4G01680.1 | myb domain protein 55 | Chr4:716222-717415 REVERSE
LENGTH=336 | 201606   Arabidopsis_thaliana   OG0000011
AT4G05100.1 | myb domain protein 74 | Chr4:2618557-2619790 FORWARD
LENGTH=324 | 201606   Arabidopsis_thaliana   OG0000011
AT4G09460.1 | myb domain protein 6 | Chr4:5993019-5994038 FORWARD
LENGTH=236 | 201606   Arabidopsis_thaliana   OG0000011
AT4G12350.1 | myb domain protein 42 | Chr4:7324912-7326636 FORWARD
LENGTH=286 | 201606   Arabidopsis_thaliana   OG0000011
AT4G13480.1 | myb domain protein 79 | Chr4:7836675-7837653 FORWARD
LENGTH=261 | 201606   Arabidopsis_thaliana   OG0000011
AT1G17950.1 | myb domain protein 52 | Chr1:6177764-6179063 FORWARD

LENGTH=249 | 201606 Arabidopsis_thaliana OG0000011
AT4G17785.1 | myb domain protein 39 | Chr4:9881867-9883368 REVERSE
LENGTH=360 | 201606 Arabidopsis_thaliana OG0000011
AT4G18770.1 | myb domain protein 98 | Chr4:10311068-10312557 FORWARD
LENGTH=427 | 201606 Arabidopsis_thaliana OG0000011
AT1G18570.1 | myb domain protein 51 | Chr1:6389765-6391026 FORWARD
LENGTH=352 | 201606 Arabidopsis_thaliana OG0000011
AT1G18710.1 | myb domain protein 47 | Chr1:6450781-6452986 FORWARD
LENGTH=267 | 201606 Arabidopsis_thaliana OG0000011
AT4G21440.1 | MYB-like 102 | Chr4:11418425-11419652 REVERSE
LENGTH=350 | 201606 Arabidopsis_thaliana OG0000011
AT1G18960.1 | myb-like HTH transcriptional regulator family protein
| Chr1:6552854-6553940 FORWARD LENGTH=307 | 201606
Arabidopsis_thaliana OG0000011
AT4G22680.1 | myb domain protein 85 | Chr4:11922789-11924080 REVERSE
LENGTH=266 | 201606 Arabidopsis_thaliana OG0000011
AT4G25560.1 | myb domain protein 18 | Chr4:13052555-13053573 FORWARD
LENGTH=283 | 201606 Arabidopsis_thaliana OG0000011
AT4G26930.1 | myb domain protein 97 | Chr4:13527770-13529172 FORWARD
LENGTH=389 | 201606 Arabidopsis_thaliana OG0000011
AT4G28110.1 | myb domain protein 41 | Chr4:13968166-13969384 REVERSE
LENGTH=282 | 201606 Arabidopsis_thaliana OG0000011
AT4G32730.1 | Homeodomain-like protein | Chr4:15791039-15794252
FORWARD LENGTH=776 | 201606 Arabidopsis_thaliana
OG0000011
AT4G33450.1 | myb domain protein 69 | Chr4:16095746-16096603 REVERSE
LENGTH=250 | 201606 Arabidopsis_thaliana OG0000011
AT4G34990.1 | myb domain protein 32 | Chr4:16661370-16662289 REVERSE
LENGTH=274 | 201606 Arabidopsis_thaliana OG0000011
AT4G37260.1 | myb domain protein 73 | Chr4:17540602-17541564 FORWARD
LENGTH=320 | 201606 Arabidopsis_thaliana OG0000011
AT4G37780.1 | myb domain protein 87 | Chr4:17758404-17759526 REVERSE
LENGTH=305 | 201606 Arabidopsis_thaliana OG0000011
AT4G38620.1 | myb domain protein 4 | Chr4:18053866-18054876 FORWARD
LENGTH=282 | 201606 Arabidopsis_thaliana OG0000011
AT5G02320.1 | myb domain protein 3r-5 | Chr5:483445-486347 REVERSE
LENGTH=548 | 201606 Arabidopsis_thaliana OG0000011
AT5G06100.1 | myb domain protein 33 | Chr5:1838555-1839997 FORWARD
LENGTH=451 | 201606 Arabidopsis_thaliana OG0000011
AT5G07690.1 | myb domain protein 29 | Chr5:2447090-2448285 FORWARD
LENGTH=336 | 201606 Arabidopsis_thaliana OG0000011
AT5G07700.1 | myb domain protein 76 | Chr5:2450325-2451578 FORWARD
LENGTH=338 | 201606 Arabidopsis_thaliana OG0000011
AT5G10280.1 | myb domain protein 92 | Chr5:3232776-3233956 FORWARD
LENGTH=334 | 201606 Arabidopsis_thaliana OG0000011
AT5G11050.1 | myb domain protein 64 | Chr5:3502144-3503635 FORWARD
LENGTH=423 | 201606 Arabidopsis_thaliana OG0000011
AT5G11510.1 | myb domain protein 3r-4 | Chr5:3680452-3684684 FORWARD
LENGTH=961 | 201606 Arabidopsis_thaliana OG0000011
AT5G12870.1 | myb domain protein 46 | Chr5:4062939-4064939 REVERSE
LENGTH=280 | 201606 Arabidopsis_thaliana OG0000011
AT5G14340.1 | myb domain protein 40 | Chr5:4623331-4624779 FORWARD
LENGTH=275 | 201606 Arabidopsis_thaliana OG0000011
AT5G14750.1 | myb domain protein 66 | Chr5:4763656-4764738 REVERSE

LENGTH=203 | 201606   Arabidopsis_thaliana   OG0000011
AT5G15310.1 | myb domain protein 16 | Chr5:4974894-4976106 FORWARD
LENGTH=326 | 201606   Arabidopsis_thaliana   OG0000011
AT5G16600.1 | myb domain protein 43 | Chr5:5438291-5440214 FORWARD
LENGTH=327 | 201606   Arabidopsis_thaliana   OG0000011
AT5G16770.1 | myb domain protein 9 | Chr5:5515209-5516734 FORWARD
LENGTH=336 | 201606   Arabidopsis_thaliana   OG0000011
AT5G17800.1 | myb domain protein 56 | Chr5:5877247-5879071 FORWARD
LENGTH=323 | 201606   Arabidopsis_thaliana   OG0000011
AT1G22640.1 | myb domain protein 3 | Chr1:8006289-8007219 FORWARD
LENGTH=257 | 201606   Arabidopsis_thaliana   OG0000011
AT5G23000.1 | myb domain protein 37 | Chr5:7696234-7697712 FORWARD
LENGTH=329 | 201606   Arabidopsis_thaliana   OG0000011
AT5G26660.1 | myb domain protein 86 | Chr5:9331775-9333044 REVERSE
LENGTH=352 | 201606   Arabidopsis_thaliana   OG0000011
AT5G35550.1 | Duplicated homeodomain-like superfamily protein |
Chr5:13726807-13727729 FORWARD LENGTH=258 | 201606
Arabidopsis_thaliana   OG0000011
AT5G39700.1 | myb domain protein 89 | Chr5:15893761-15894424 REVERSE
LENGTH=190 | 201606   Arabidopsis_thaliana   OG0000011
AT5G40330.1 | myb domain protein 23 | Chr5:16127849-16129468 FORWARD
LENGTH=219 | 201606   Arabidopsis_thaliana   OG0000011
AT5G40350.1 | myb domain protein 24 | Chr5:16138703-16140946 REVERSE
LENGTH=214 | 201606   Arabidopsis_thaliana   OG0000011
AT5G40360.1 | myb domain protein 115 | Chr5:16145220-16146579
FORWARD LENGTH=359 | 201606   Arabidopsis_thaliana
OG0000011
AT5G40430.1 | myb domain protein 22 | Chr5:16176427-16177731 REVERSE
LENGTH=256 | 201606   Arabidopsis_thaliana   OG0000011
AT5G49330.1 | myb domain protein 111 | Chr5:19999147-20001293
REVERSE LENGTH=342 | 201606   Arabidopsis_thaliana
OG0000011
AT5G49620.1 | myb domain protein 78 | Chr5:20137491-20139384 REVERSE
LENGTH=307 | 201606   Arabidopsis_thaliana   OG0000011
AT1G25340.1 | myb domain protein 116 | Chr1:8885210-8886271 FORWARD
LENGTH=283 | 201606   Arabidopsis_thaliana   OG0000011
AT5G52260.1 | myb domain protein 19 | Chr5:21220165-21221223 FORWARD
LENGTH=268 | 201606   Arabidopsis_thaliana   OG0000011
AT5G52600.1 | myb domain protein 82 | Chr5:21343197-21343968 REVERSE
LENGTH=201 | 201606   Arabidopsis_thaliana   OG0000011
AT5G54230.1 | myb domain protein 49 | Chr5:22016357-22017845 REVERSE
LENGTH=319 | 201606   Arabidopsis_thaliana   OG0000011
AT5G55020.1 | myb domain protein 120 | Chr5:22324599-22326248
REVERSE LENGTH=523 | 201606   Arabidopsis_thaliana
OG0000011
AT5G56110.1 | myb domain protein 103 | Chr5:22719191-22720664
FORWARD LENGTH=320 | 201606   Arabidopsis_thaliana
OG0000011
AT5G57620.1 | myb domain protein 36 | Chr5:23334904-23336388 FORWARD
LENGTH=333 | 201606   Arabidopsis_thaliana   OG0000011
AT5G58850.1 | myb domain protein 119 | Chr5:23763945-23765441
FORWARD LENGTH=430 | 201606   Arabidopsis_thaliana
OG0000011
AT5G59780.1 | myb domain protein 59 | Chr5:24082425-24083044 REVERSE

LENGTH=170 | 201606	Arabidopsis_thaliana	OG0000011
AT1G26780.1 | myb domain protein 117 | Chr1:9271033-9272128 FORWARD LENGTH=280 | 201606	Arabidopsis_thaliana	OG0000011
AT5G60890.1 | myb domain protein 34 | Chr5:24495029-24496220 FORWARD LENGTH=295 | 201606	Arabidopsis_thaliana	OG0000011
AT5G62320.1 | myb domain protein 99 | Chr5:25028885-25029786 REVERSE LENGTH=245 | 201606	Arabidopsis_thaliana	OG0000011
AT5G62470.1 | myb domain protein 96 | Chr5:25079434-25080858 REVERSE LENGTH=351 | 201606	Arabidopsis_thaliana	OG0000011
AT5G65230.1 | myb domain protein 53 | Chr5:26068290-26069408 FORWARD LENGTH=310 | 201606	Arabidopsis_thaliana	OG0000011
AT5G65790.1 | myb domain protein 68 | Chr5:26323035-26324385 FORWARD LENGTH=374 | 201606	Arabidopsis_thaliana	OG0000011
AT5G67300.1 | myb domain protein r1 | Chr5:26854109-26855026 FORWARD LENGTH=305 | 201606	Arabidopsis_thaliana	OG0000011
AT1G34670.1 | myb domain protein 93 | Chr1:12709128-12710423 FORWARD LENGTH=365 | 201606	Arabidopsis_thaliana	OG0000011
AT1G35515.1 | high response to osmotic stress 10 | Chr1:13077926-13080265 FORWARD LENGTH=212 | 201606	Arabidopsis_thaliana	OG0000011
AT1G48000.1 | myb domain protein 112 | Chr1:17704269-17706377 REVERSE LENGTH=243 | 201606	Arabidopsis_thaliana	OG0000011
AT1G56160.1 | myb domain protein 72 | Chr1:21022384-21023444 FORWARD LENGTH=296 | 201606	Arabidopsis_thaliana	OG0000011
AT1G56650.1 | production of anthocyanin pigment 1 | Chr1:21233714-21235089 REVERSE LENGTH=248 | 201606	Arabidopsis_thaliana	OG0000011
AT1G57560.1 | myb domain protein 50 | Chr1:21316851-21317959 FORWARD LENGTH=314 | 201606	Arabidopsis_thaliana	OG0000011
AT1G63910.1 | myb domain protein 103 | Chr1:23719968-23721625 REVERSE LENGTH=370 | 201606	Arabidopsis_thaliana	OG0000011
AT1G06180.1 | myb domain protein 13 | Chr1:1889510-1891089 FORWARD LENGTH=246 | 201606	Arabidopsis_thaliana	OG0000011
AT1G66230.1 | myb domain protein 20 | Chr1:24677322-24678246 FORWARD LENGTH=282 | 201606	Arabidopsis_thaliana	OG0000011
AT1G66370.1 | myb domain protein 113 | Chr1:24753634-24754604 FORWARD LENGTH=246 | 201606	Arabidopsis_thaliana	OG0000011
AT1G66380.1 | myb domain protein 114 | Chr1:24757413-24758490 FORWARD LENGTH=139 | 201606	Arabidopsis_thaliana	OG0000011
AT1G66390.1 | myb domain protein 90 | Chr1:24764052-24765400 FORWARD LENGTH=249 | 201606	Arabidopsis_thaliana	OG0000011
AT1G68320.1 | myb domain protein 62 | Chr1:25603842-25604884 FORWARD LENGTH=286 | 201606	Arabidopsis_thaliana	OG0000011
AT1G69560.1 | myb domain protein 105 | Chr1:26157755-26158906 FORWARD LENGTH=330 | 201606	Arabidopsis_thaliana	OG0000011
AT1G73410.1 | myb domain protein 54 | Chr1:27601852-27603038 FORWARD LENGTH=243 | 201606	Arabidopsis_thaliana	OG0000011
AT1G74080.1 | myb domain protein 122 | Chr1:27855972-27857432 FORWARD LENGTH=333 | 201606	Arabidopsis_thaliana

OG0000011
AT1G74430.1 | myb domain protein 95 | Chr1:27975409-27977118 FORWARD LENGTH=271 | 201606	Arabidopsis_thaliana	OG0000011
AT1G74650.1 | myb domain protein 31 | Chr1:28041493-28042773 FORWARD LENGTH=330 | 201606	Arabidopsis_thaliana	OG0000011
AT1G79180.1 | myb domain protein 63 | Chr1:29786509-29787589 FORWARD LENGTH=294 | 201606	Arabidopsis_thaliana	OG0000011
AT2G02820.1 | myb domain protein 88 | Chr2:804890-807139 REVERSE LENGTH=455 | 201606	Arabidopsis_thaliana	OG0000011
AT2G13960.1 | Homeodomain-like superfamily protein | Chr2:5859683-5861105 FORWARD LENGTH=150 | 201606	Arabidopsis_thaliana	OG0000011
AT2G16720.1 | myb domain protein 7 | Chr2:7255669-7256550 REVERSE LENGTH=269 | 201606	Arabidopsis_thaliana	OG0000011
AT1G08810.1 | myb domain protein 60 | Chr1:2819279-2820329 REVERSE LENGTH=280 | 201606	Arabidopsis_thaliana	OG0000011
AT2G23290.1 | myb domain protein 70 | Chr2:9904895-9905824 REVERSE LENGTH=309 | 201606	Arabidopsis_thaliana	OG0000011
AT2G25230.1 | myb domain protein 100 | Chr2:10747211-10748467 REVERSE LENGTH=234 | 201606	Arabidopsis_thaliana	OG0000011
AT1G09540.1 | myb domain protein 61 | Chr1:3086333-3087689 FORWARD LENGTH=366 | 201606	Arabidopsis_thaliana	OG0000011
AT2G26950.1 | myb domain protein 104 | Chr2:11500721-11502073 REVERSE LENGTH=382 | 201606	Arabidopsis_thaliana	OG0000011
AT2G26960.1 | myb domain protein 81 | Chr2:11506065-11507425 REVERSE LENGTH=427 | 201606	Arabidopsis_thaliana	OG0000011
AT2G31180.1 | myb domain protein 14 | Chr2:13286806-13288175 REVERSE LENGTH=249 | 201606	Arabidopsis_thaliana	OG0000011
AT2G32460.1 | myb domain protein 101 | Chr2:13782419-13784363 REVERSE LENGTH=490 | 201606	Arabidopsis_thaliana	OG0000011
Os03t0236300-00 Similar to Myb-like DNA-binding domain containing protein, expressed.	Oryza_sativa	OG0000011
Os03t0315400-01 Similar to Typical P-type R2R3 Myb protein (Fragment).	Oryza_sativa	OG0000011
Os03t0371800-00 Similar to Myb-like DNA-binding domain containing protein.	Oryza_sativa	OG0000011
Os03t0388600-01 Hypothetical conserved gene.	Oryza_sativa	OG0000011
Os03t0424300-00 Homeodomain-like domain containing protein.	Oryza_sativa	OG0000011
Os03t0578900-00 Similar to MYB1.	Oryza_sativa	OG0000011
Os03t0720800-01 Similar to MYB transcription factor R2R3 type.	Oryza_sativa	OG0000011
Os03t0771100-01 Myb transcription factor domain containing protein.	Oryza_sativa	OG0000011
Os01t0274800-01 R2R3-type MYB transcription factor, Sugar partitioning into anther	Oryza_sativa	OG0000011
Os01t0285300-01 Myb transcription factor domain containing protein.	Oryza_sativa	OG0000011
Os01t0298400-01 Myb transcription factor domain containing protein.	Oryza_sativa	OG0000011

Os01t0305900-01 Similar to R2R3 Myb transcription factor MYB-IF35.
Oryza_sativa OG0000011
Os04t0461000-01 Similar to Myb-related transcription factor LBM1.
Oryza_sativa OG0000011
Os04t0508500-01 Similar to OSIGBa0101P20.9 protein.
Oryza_sativa OG0000011
Os04t0532800-01 Myb transcription factor domain containing protein.
Oryza_sativa OG0000011
Os04t0593200-01 Myb transcription factor domain containing protein.
Oryza_sativa OG0000011
Os04t0594100-01 Similar to P-type R2R3 Myb protein (Fragment).
Oryza_sativa OG0000011
Os05t0140100-01 R2R3 MYB transcription factor, Regulation of phosphate-starvation responses and root architecture
Oryza_sativa OG0000011
Os05t0429900-01 Similar to MybHv5 (Fragment). Oryza_sativa OG0000011
Os05t0459000-01 Similar to Gag/env/c-myb protein (Fragment).
Oryza_sativa OG0000011
Os05t0490600-00 OSMYB5. Oryza_sativa OG0000011
Os05t0543600-01 Similar to Myb-related protein. Oryza_sativa OG0000011
Os05t0553400-01 Similar to Myb-related transcription factor-like protein (MYB transcription factor). Oryza_sativa OG0000011
Os06t0112700-00 Similar to Typical P-type R2R3 Myb protein (Fragment). Oryza_sativa OG0000011
Os06t0141950-00 Similar to MYB transcription factor.
Oryza_sativa OG0000011
Os06t0162700-01 SANT domain, DNA binding domain containing protein.
Oryza_sativa OG0000011
Os06t0221000-00 Similar to P-type R2R3 Myb protein (Fragment).
Oryza_sativa OG0000011
Os06t0258000-01 Similar to Typical P-type R2R3 Myb protein (Fragment). Oryza_sativa OG0000011
Os06t0605750-00 Similar to GAMYB-like1. Oryza_sativa OG0000011
Os06t0637500-02 Similar to MYB transcription factor R2R3 type.
Oryza_sativa OG0000011
Os06t0679400-01 Similar to Myb-related protein Pp2.
Oryza_sativa OG0000011
Os07t0136800-01 Homeodomain-like containing protein.
Oryza_sativa OG0000011
Os07t0434300-00 Similar to Myb-like DNA-binding domain containing protein. Oryza_sativa OG0000011
Os01t0128000-01 Myb transcription factor domain containing protein.
Oryza_sativa OG0000011
Os07t0484700-01 Myb transcription factor domain containing protein.
Oryza_sativa OG0000011
Os07t0497500-00 Similar to MYB transcription factor.
Oryza_sativa OG0000011
Os07t0558100-00 Similar to Myb-related transcription factor LBM1.
Oryza_sativa OG0000011
Os07t0627300-01 Similar to Myb-related protein B (B-Myb).

Oryza_sativa	OG0000011
Os07t0629000-01 Similar to P-type R2R3 Myb protein (Fragment).
Oryza_sativa	OG0000011
Os07t0688200-01 Similar to Typical P-type R2R3 Myb protein
(Fragment).	Oryza_sativa	OG0000011
Os08t0151300-01 R2R3-MYB transcription factor, Regulation of leaf
shape, cellulose synthesis and mechanical strength Oryza_sativa
OG0000011
Os01t0637800-00 Similar to Transcription factor (Fragment).
Oryza_sativa	OG0000011
Os08t0248700-01 Similar to Blind. Oryza_sativa	OG0000011
Os08t0433400-01 Hypothetical conserved gene.	Oryza_sativa
OG0000011
Os08t0435700-00 Similar to MYB domain class transcription factor.
Oryza_sativa	OG0000011
Os08t0437300-00 Similar to Typical P-type R2R3 Myb protein
(Fragment).	Oryza_sativa	OG0000011
Os08t0450900-00 SANT domain, DNA binding domain containing protein.
Oryza_sativa	OG0000011
Os08t0486300-00 Similar to P-type R2R3 Myb protein (Fragment).
Oryza_sativa	OG0000011
Os09t0106700-01 Similar to Myb proto-oncogene protein (C-myb).
Oryza_sativa	OG0000011
Os09t0401000-01 Myb factor.	Oryza_sativa	OG0000011
Os09t0431300-00 Similar to MYB transcription factor MYB71
(Fragment).	Oryza_sativa	OG0000011
Os09t0532900-01 Similar to Myb13 protein. Oryza_sativa
OG0000011
Os09t0538400-01 Similar to P-type R2R3 Myb protein (Fragment).
Oryza_sativa	OG0000011
Os01t0695900-01 OSMYB4.	Oryza_sativa	OG0000011
Os01t0702700-01 Similar to Transcription factor MYB86 (Myb-related
protein 86) (AtMYB86) (Myb homolog 4) (AtMyb4).	Oryza_sativa
OG0000011
Os01t0709000-01 Similar to Transcription factor MYB1.
Oryza_sativa	OG0000011
Os10t0478300-01 Similar to Y19 protein.	Oryza_sativa
OG0000011
Os01t0722300-01 Myb transcription factor domain containing protein.
Oryza_sativa	OG0000011
Os11t0128500-01 Similar to Myb factor.	Oryza_sativa
OG0000011
Os11t0180900-00 Myb transcription factor domain containing protein.
Oryza_sativa	OG0000011
Os11t0207600-00 Myb-like protein. Oryza_sativa	OG0000011
Os11t0558200-01 R2R3-type MYB transcriptional activator, Tolerance
to Pi deficiency Oryza_sativa	OG0000011
Os11t0684000-01 Similar to Transcription factor MYB21 (Myb-related
protein 21) (AtMYB21) (Myb homolog 3) (AtMyb3).	Oryza_sativa
OG0000011
Os11t0700500-01 Similar to Snapdragon myb protein 305 homolog.
Oryza_sativa	OG0000011
Os12t0125000-01 Myb factor.	Oryza_sativa	OG0000011
Os12t0238000-00 SANT domain, DNA binding domain containing protein.

Oryza_sativa	OG0000011
Os12t0567300-00 Myb transcription factor domain containing protein.	Oryza_sativa	OG0000011
Os01t0812000-01 Transcriptional activator of gibberellin-dependent alpha-amylase expression, Regulation of nutrient mobilization in germination	Oryza_sativa	OG0000011
Os01t0841500-01 R1R2R3 MYB transcription factor, Stress tolerance	Oryza_sativa	OG0000011
Os01t0850400-01 Similar to MYBY1 protein (Fragment).	Oryza_sativa	OG0000011
Os01t0855400-00 SANT domain, DNA binding domain containing protein.	Oryza_sativa	OG0000011
Os01t0874300-01 Similar to MybHv5 (Fragment).	Oryza_sativa	OG0000011
Os01t0975300-01 MYB-related transcription factor, Drought and salinity tolerance	Oryza_sativa	OG0000011
Os01t0977300-01 Similar to MYB-related protein.	Oryza_sativa	OG0000011
Os02t0187700-00 Similar to Myb-related protein B (B-Myb) (Myb-related protein 1) (XMYB1).	Oryza_sativa	OG0000011
Os02t0271900-01 Myb transcription factor domain containing protein.	Oryza_sativa	OG0000011
Os01t0191900-01 Similar to Blind. Oryza_sativa	OG0000011
Os02t0618400-02 R2R3-type MYB transcription factor, Adaptive growth regulation	Oryza_sativa	OG0000011
Os02t0624300-01 Similar to Y19 protein.	Oryza_sativa	OG0000011
Os02t0641900-01 Similar to P-type R2R3 Myb protein.	Oryza_sativa	OG0000011
Os02t0695200-01 Similar to P-type R2R3 Myb protein (Fragment).	Oryza_sativa	OG0000011
Os02t0732600-01 Similar to Typical P-type R2R3 Myb protein (Fragment).	Oryza_sativa	OG0000011
Os03t0142600-00 Myb transcription factor domain containing protein.	Oryza_sativa	OG0000011
CMJ282CT pep chromosome:ASM9120v1:10:747119:748691:-1 gene:CMJ282C transcript:CMJ282CT gene_biotype:protein_coding transcript_biotype:protein_coding Cyanidioschyzon_merolae	OG0000011
CMT134CT pep chromosome:ASM9120v1:20:361576:364743:1 gene:CMT134C transcript:CMT134CT gene_biotype:protein_coding transcript_biotype:protein_coding Cyanidioschyzon_merolae	OG0000011
CMG052CT pep chromosome:ASM9120v1:7:150937:152088:1 gene:CMG052C transcript:CMG052CT gene_biotype:protein_coding transcript_biotype:protein_coding Cyanidioschyzon_merolae	OG0000011
CMK280CT pep chromosome:ASM9120v1:11:774474:776037:-1 gene:CMK280C transcript:CMK280CT gene_biotype:protein_coding transcript_biotype:protein_coding Cyanidioschyzon_merolae	OG0000011
CMQ253CT pep chromosome:ASM9120v1:17:645837:647139:-1 gene:CMQ253C transcript:CMQ253CT gene_biotype:protein_coding transcript_biotype:protein_coding Cyanidioschyzon_merolae

OG0000011
evm.model.contig_2090.6 Porphyridium_purpureum   OG0000011
evm.model.contig_2268.3 Porphyridium_purpureum   OG0000011
evm.model.contig_3397.12 Porphyridium_purpureum   OG0000011
evm.model.contig_3578.1 Porphyridium_purpureum   OG0000011
evm.model.contig_3821.1 Porphyridium_purpureum   OG0000011
evm.model.contig_4410.19 Porphyridium_purpureum   OG0000011
evm.model.contig_4428.5 Porphyridium_purpureum   OG0000011
evm.model.contig_444.1   Porphyridium_purpureum   OG0000011
evm.model.contig_481.6   Porphyridium_purpureum   OG0000011
evm.model.contig_518.6   Porphyridium_purpureum   OG0000011
evm.model.contig_540.1   Porphyridium_purpureum   OG0000011
evm.model.contig_651.4   Porphyridium_purpureum   OG0000011
CDF32864 pep supercontig:ASM35022v2:HG001593:124278:126245:-1
gene:CHC_T00001660001 transcript:CDF32864
gene_biotype:protein_coding transcript_biotype:protein_coding
Chondrus_crispus OG0000011
CDF32668 pep supercontig:ASM35022v2:HG001531:264658:265707:-1
gene:CHC_T00008608001 transcript:CDF32668
gene_biotype:protein_coding transcript_biotype:protein_coding
description:MYB-related protein   Chondrus_crispus OG0000011
CDF39437 pep supercontig:ASM35022v2:HG002031:424234:425082:-1
gene:CHC_T00006586001 transcript:CDF39437
gene_biotype:protein_coding transcript_biotype:protein_coding
Chondrus_crispus OG0000011
CDF38231 pep supercontig:ASM35022v2:HG001904:90664:91881:1
gene:CHC_T00000592001 transcript:CDF38231
gene_biotype:protein_coding transcript_biotype:protein_coding
Chondrus_crispus OG0000011
CDF77437 pep supercontig:ASM35022v2:HG001523:394880:396702:-1
gene:CHC_T00007941001 transcript:CDF77437
gene_biotype:protein_coding transcript_biotype:protein_coding
Chondrus_crispus OG0000011
CDF77474 pep supercontig:ASM35022v2:HG001523:573805:576813:-1
gene:CHC_T00007971001 transcript:CDF77474
gene_biotype:protein_coding transcript_biotype:protein_coding
Chondrus_crispus OG0000011
jgi|ChlNC64A_1|132995|IGS.gm_1_00131   Chlorella_variabilis
OG0000012
Pp3c19_2470V3.1.p pacid=32939607 transcript=Pp3c19_2470V3.1
locus=Pp3c19_2470 annot-version=v3.3   Physcomitrella_patens
OG0000012
Pp3c19_2480V3.1.p pacid=32940153 transcript=Pp3c19_2480V3.1
locus=Pp3c19_2480 annot-version=v3.3   Physcomitrella_patens
OG0000012
Pp3c3_570V3.1.p pacid=32940707 transcript=Pp3c3_570V3.1
locus=Pp3c3_570 annot-version=v3.3   Physcomitrella_patens
OG0000012
Pp3c3_6800V3.1.p pacid=32940794 transcript=Pp3c3_6800V3.1
locus=Pp3c3_6800 annot-version=v3.3   Physcomitrella_patens
OG0000012
Pp3c3_1350V3.1.p pacid=32941090 transcript=Pp3c3_1350V3.1
locus=Pp3c3_1350 annot-version=v3.3   Physcomitrella_patens
OG0000012

Pp3c3_2260V3.1.p pacid=32941348 transcript=Pp3c3_2260V3.1 locus=Pp3c3_2260 annot-version=v3.3 Physcomitrella_patens OG0000012
Pp3c3_1280V3.1.p pacid=32941484 transcript=Pp3c3_1280V3.1 locus=Pp3c3_1280 annot-version=v3.3 Physcomitrella_patens OG0000012
Pp3c3_1480V3.1.p pacid=32941604 transcript=Pp3c3_1480V3.1 locus=Pp3c3_1480 annot-version=v3.3 Physcomitrella_patens OG0000012
Pp3c3_1460V3.1.p pacid=32942239 transcript=Pp3c3_1460V3.1 locus=Pp3c3_1460 annot-version=v3.3 Physcomitrella_patens OG0000012
Pp3c3_1260V3.1.p pacid=32944757 transcript=Pp3c3_1260V3.1 locus=Pp3c3_1260 annot-version=v3.3 Physcomitrella_patens OG0000012
Pp3c23_12000V3.1.p pacid=32950110 transcript=Pp3c23_12000V3.1 locus=Pp3c23_12000 annot-version=v3.3 Physcomitrella_patens OG0000012
Pp3c14_23490V3.1.p pacid=32960175 transcript=Pp3c14_23490V3.1 locus=Pp3c14_23490 annot-version=v3.3 Physcomitrella_patens OG0000012
Pp3c16_20430V3.1.p pacid=32985723 transcript=Pp3c16_20430V3.1 locus=Pp3c16_20430 annot-version=v3.3 Physcomitrella_patens OG0000012
AT1G10920.1 | NB-ARC domain-containing disease resistance protein | Chr1:3644587-3647004 REVERSE LENGTH=727 | 201606 Arabidopsis_thaliana OG0000012
AT3G07040.1 | NB-ARC domain-containing disease resistance protein | Chr3:2226244-2229024 REVERSE LENGTH=926 | 201606 Arabidopsis_thaliana OG0000012
AT3G14460.1 | LRR and NB-ARC domains-containing disease resistance protein | Chr3:4851990-4856264 REVERSE LENGTH=1424 | 201606 Arabidopsis_thaliana OG0000012
AT3G14470.1 | NB-ARC domain-containing disease resistance protein | Chr3:4857940-4861104 FORWARD LENGTH=1054 | 201606 Arabidopsis_thaliana OG0000012
AT3G46530.1 | NB-ARC domain-containing disease resistance protein | Chr3:17130739-17133246 REVERSE LENGTH=835 | 201606 Arabidopsis_thaliana OG0000012
AT3G46710.1 | NB-ARC domain-containing disease resistance protein | Chr3:17206489-17209032 REVERSE LENGTH=847 | 201606 Arabidopsis_thaliana OG0000012
AT3G46730.1 | NB-ARC domain-containing disease resistance protein | Chr3:17213069-17215612 REVERSE LENGTH=847 | 201606 Arabidopsis_thaliana OG0000012
AT3G50950.1 | HOPZ-ACTIVATED RESISTANCE 1 | Chr3:18936127-18938685 FORWARD LENGTH=852 | 201606 Arabidopsis_thaliana OG0000012
AT5G35450.1 | Disease resistance protein (CC-NBS-LRR class) family | Chr5:13667809-13670685 FORWARD LENGTH=901 | 201606 Arabidopsis_thaliana OG0000012
AT5G43470.1 | Disease resistance protein (CC-NBS-LRR class) family | Chr5:17463130-17466658 REVERSE LENGTH=908 | 201606 Arabidopsis_thaliana OG0000012

AT5G48620.1 | Disease resistance protein (CC-NBS-LRR class) family | Chr5:19717406-19720932 FORWARD LENGTH=908 | 201606
Arabidopsis_thaliana OG0000012
AT1G50180.1 | NB-ARC domain-containing disease resistance protein | Chr1:18584235-18587136 FORWARD LENGTH=857 | 201606
Arabidopsis_thaliana OG0000012
AT1G53350.1 | Disease resistance protein (CC-NBS-LRR class) family | Chr1:19903899-19906912 FORWARD LENGTH=906 | 201606
Arabidopsis_thaliana OG0000012
AT1G58390.1 | Disease resistance protein (CC-NBS-LRR class) family | Chr1:21690962-21693891 REVERSE LENGTH=907 | 201606
Arabidopsis_thaliana OG0000012
AT1G58400.1 | Disease resistance protein (CC-NBS-LRR class) family | Chr1:21696165-21699118 REVERSE LENGTH=900 | 201606
Arabidopsis_thaliana OG0000012
AT1G58410.1 | Disease resistance protein (CC-NBS-LRR class) family | Chr1:21701286-21704255 REVERSE LENGTH=899 | 201606
Arabidopsis_thaliana OG0000012
AT1G58602.1 | LRR and NB-ARC domains-containing disease resistance protein | Chr1:21760167-21763765 FORWARD LENGTH=1138 | 201606
Arabidopsis_thaliana OG0000012
AT1G58807.1 | Disease resistance protein (CC-NBS-LRR class) family | Chr1:21780574-21783793 FORWARD LENGTH=1017 | 201606
Arabidopsis_thaliana OG0000012
AT1G58848.1 | Disease resistance protein (CC-NBS-LRR class) family | Chr1:21792140-21795455 FORWARD LENGTH=1049 | 201606
Arabidopsis_thaliana OG0000012
AT1G59124.1 | Disease resistance protein (CC-NBS-LRR class) family | Chr1:21816832-21819653 FORWARD LENGTH=855 | 201606
Arabidopsis_thaliana OG0000012
AT1G59218.1 | Disease resistance protein (CC-NBS-LRR class) family | Chr1:21828398-21831713 FORWARD LENGTH=1049 | 201606
Arabidopsis_thaliana OG0000012
AT1G59620.1 | Disease resistance protein (CC-NBS-LRR class) family | Chr1:21902627-21905527 FORWARD LENGTH=895 | 201606
Arabidopsis_thaliana OG0000012
AT1G59780.1 | NB-ARC domain-containing disease resistance protein | Chr1:21993581-21997691 REVERSE LENGTH=906 | 201606
Arabidopsis_thaliana OG0000012
Os01t0112800-00 Disease resistance protein domain containing protein. Oryza_sativa OG0000012
Os01t0113150-00 Disease resistance protein domain containing protein. Oryza_sativa OG0000012
Os03t0324600-01 NB-ARC domain containing protein. Oryza_sativa OG0000012
Os03t0379700-00 Hypothetical conserved gene. Oryza_sativa OG0000012
Os03t0422900-00 Similar to NB-ARC domain containing protein, expressed. Oryza_sativa OG0000012
Os03t0573500-00 Disease resistance protein domain containing protein. Oryza_sativa OG0000012
Os03t0579200-00 Similar to NB-ARC domain containing protein. Oryza_sativa OG0000012
Os03t0599000-01 NB-ARC domain containing protein. Oryza_sativa

OG0000012
Os01t0260500-01 Hypothetical conserved gene.	Oryza_sativa
OG0000012
Os03t0689400-01 Similar to NB-ARC domain containing protein, expressed.	Oryza_sativa	OG0000012
Os03t0689833-00 Similar to NB-ARC domain containing protein, expressed.	Oryza_sativa	OG0000012
Os03t0709200-01 NB-ARC domain containing protein.	Oryza_sativa
OG0000012
Os01t0269500-01 Conserved hypothetical protein.	Oryza_sativa
OG0000012
Os03t0848700-01 Similar to TDRGA-1 (Fragment).	Oryza_sativa
OG0000012
Os03t0849500-00 Similar to BPH14-1.	Oryza_sativa
OG0000012
Os04t0111100-00 Similar to H0215A08.1 protein.	Oryza_sativa
OG0000012
Os04t0111900-01 Similar to Resistance gene analog PIC22 (Fragment).
Oryza_sativa	OG0000012
Os04t0118800-00 NB-ARC domain containing protein.	Oryza_sativa
OG0000012
Os04t0193950-00 Disease resistance protein domain containing protein.	Oryza_sativa	OG0000012
Os04t0219600-01 Similar to NBS-LRR protein (Fragment).
Oryza_sativa	OG0000012
Os04t0325300-01 Similar to NB-ARC domain containing protein, expressed.	Oryza_sativa	OG0000012
Os04t0375300-00 Similar to NBS-LRR protein (Fragment).
Oryza_sativa	OG0000012
Os04t0378575-00 Similar to NBS-LRR protein (Fragment).
Oryza_sativa	OG0000012
Os01t0308300-00 NB-ARC domain containing protein.	Oryza_sativa
OG0000012
Os04t0512900-01 NB-ARC domain containing protein.	Oryza_sativa
OG0000012
Os01t0335700-01 NB-ARC domain containing protein.	Oryza_sativa
OG0000012
Os04t0620950-01 Hypothetical conserved gene.	Oryza_sativa
OG0000012
Os04t0621500-00 Disease resistance protein domain containing protein.	Oryza_sativa	OG0000012
Os04t0623066-01 Similar to XA1.	Oryza_sativa	OG0000012
Os04t0626450-01 NB-ARC domain containing protein.	Oryza_sativa
OG0000012
Os01t0350300-00 Disease resistance protein domain containing protein.	Oryza_sativa	OG0000012
Os01t0358700-01 Similar to Powdery mildew resistance protein PM3b.
Oryza_sativa	OG0000012
Os01t0359400-01 NB-ARC domain containing protein.	Oryza_sativa
OG0000012
Os01t0359600-00 Disease resistance protein domain containing protein.	Oryza_sativa	OG0000012
Os01t0359800-00 Similar to NB-ARC domain containing protein.
Oryza_sativa	OG0000012

Os01t0360100-01 Similar to Powdery mildew resistance protein pm3f (Fragment).	Oryza_sativa	OG0000012
Os05t0216950-00 NB-ARC domain containing protein.	Oryza_sativa	OG0000012
Os05t0240200-01 Similar to NB-ARC domain containing protein, expressed.	Oryza_sativa	OG0000012
Os05t0250600-00 Similar to NB-ARC domain containing protein.	Oryza_sativa	OG0000012
Os05t0250700-00 Similar to NB-ARC domain containing protein.	Oryza_sativa	OG0000012
Os05t0365300-01 Similar to NBS-LRR protein (Fragment).	Oryza_sativa	OG0000012
Os05t0380300-01 Similar to NBS-LRR protein (Fragment).	Oryza_sativa	OG0000012
Os05t0414200-01 NB-ARC domain containing protein.	Oryza_sativa	OG0000012
Os05t0414300-01 NB-ARC domain containing protein.	Oryza_sativa	OG0000012
Os05t0479800-01 Similar to NB-ARC domain containing protein, expressed.	Oryza_sativa	OG0000012
Os05t0492200-01 NB-ARC domain containing protein.	Oryza_sativa	OG0000012
Os05t0492600-00 Similar to NBS-LRR type resistance protein (Fragment).	Oryza_sativa	OG0000012
Os01t0520600-01 NB-ARC domain containing protein.	Oryza_sativa	OG0000012
Os06t0125000-00 Similar to NBS-LRR disease resistance protein homologue.	Oryza_sativa	OG0000012
Os06t0146100-01 NB-ARC domain containing protein.	Oryza_sativa	OG0000012
Os06t0158300-01 NB-ARC domain containing protein.	Oryza_sativa	OG0000012
Os06t0158400-01 Hypothetical conserved gene.	Oryza_sativa	OG0000012
Os06t0163900-01 Conserved hypothetical protein.	Oryza_sativa	OG0000012
Os01t0536501-00 Disease resistance protein domain containing protein.	Oryza_sativa	OG0000012
Os01t0547000-01 Similar to LRR19.	Oryza_sativa	OG0000012
Os06t0267900-01 NB-ARC domain containing protein.	Oryza_sativa	OG0000012
Os06t0268500-00 Similar to OSIGBa0139J17.1 protein.	Oryza_sativa	OG0000012
Os06t0279900-00 Similar to NB-ARC domain containing protein, expressed.	Oryza_sativa	OG0000012
Os06t0286500-01 Similar to NBS-LRR disease resistance protein homologue.	Oryza_sativa	OG0000012
Os06t0286700-00 Similar to Piz-t.	Oryza_sativa	OG0000012
Os06t0287000-00 Similar to NBS-LRR type R protein, Nbs4-Pi.	Oryza_sativa	OG0000012
Os06t0287200-00 Similar to NBS-LRR type R protein Nbs7-75.	Oryza_sativa	OG0000012
Os06t0287500-01 Similar to Nbs1-ON.	Oryza_sativa	OG0000012

Os06t0287700-01 Similar to NBS-LRR disease resistance protein homologue (Fragment).	Oryza_sativa	OG0000012
Os06t0330100-00 Similar to Blast resistance protein.	Oryza_sativa	OG0000012
Os06t0524900-01 NB-ARC domain containing protein.	Oryza_sativa	OG0000012
Os06t0594200-01 Disease resistance protein domain containing protein.	Oryza_sativa	OG0000012
Os06t0619000-01 NB-ARC domain containing protein.	Oryza_sativa	OG0000012
Os06t0667900-00 Disease resistance protein domain containing protein.	Oryza_sativa	OG0000012
Os06t0693100-01 NB-ARC domain containing protein.	Oryza_sativa	OG0000012
Os06t0698812-00 Disease resistance protein domain containing protein.	Oryza_sativa	OG0000012
Os06t0707700-01 NB-ARC domain containing protein.	Oryza_sativa	OG0000012
Os06t0707733-00 Disease resistance protein domain containing protein.	Oryza_sativa	OG0000012
Os07t0141900-00 Similar to OSIGBa0148A10.13 protein.	Oryza_sativa	OG0000012
Os07t0186500-01 NB-ARC domain containing protein.	Oryza_sativa	OG0000012
Os07t0197300-00 Disease resistance protein domain containing protein.	Oryza_sativa	OG0000012
Os07t0197500-00 Disease resistance protein domain containing protein.	Oryza_sativa	OG0000012
Os07t0273600-01 Disease resistance protein domain containing protein.	Oryza_sativa	OG0000012
Os07t0273700-00 Disease resistance protein domain containing protein.	Oryza_sativa	OG0000012
Os07t0273900-01 Disease resistance protein domain containing protein.	Oryza_sativa	OG0000012
Os01t0603100-01 NB-ARC domain containing protein.	Oryza_sativa	OG0000012
Os07t0294100-00 Similar to NB-ARC domain containing protein.	Oryza_sativa	OG0000012
Os07t0481300-01 Hypothetical conserved gene.	Oryza_sativa	OG0000012
Os07t0481400-01 NB-ARC domain containing protein.	Oryza_sativa	OG0000012
Os07t0501600-00 Conserved hypothetical protein.	Oryza_sativa	OG0000012
Os07t0521100-01 Leucine-rich repeat, typical subtype containing protein.	Oryza_sativa	OG0000012
Os07t0521500-01 NB-ARC domain containing protein.	Oryza_sativa	OG0000012
Os07t0521600-01 Similar to NBS-LRR protein (Fragment).	Oryza_sativa	OG0000012
Os07t0521800-01 NB-ARC domain containing protein.	Oryza_sativa	OG0000012
Os07t0599100-00 Hypothetical conserved gene.	Oryza_sativa	OG0000012

Os08t0170100-01 NB-ARC domain containing protein. Oryza_sativa
OG0000012
Os08t0170200-00 Disease resistance protein domain containing
protein. Oryza_sativa   OG0000012
Os08t0170500-00 Conserved hypothetical protein.   Oryza_sativa
OG0000012
Os08t0170700-00 Disease resistance protein domain containing
protein. Oryza_sativa   OG0000012
Os08t0174800-01 Similar to Benzothiadiazole-induced somatic
embryogenesis receptor kinase 1. Oryza_sativa   OG0000012
Os08t0190300-00 Disease resistance protein domain containing
protein. Oryza_sativa   OG0000012
Os08t0193700-00 Similar to NBS-LRR disease resistance protein
homologue (Fragment).   Oryza_sativa   OG0000012
Os08t0202400-01 Disease resistance protein domain containing
protein. Oryza_sativa   OG0000012
Os08t0205100-01 Disease resistance protein domain containing
protein. Oryza_sativa   OG0000012
Os08t0205150-01 NB-ARC domain containing protein. Oryza_sativa
OG0000012
Os08t0224000-01 NB-ARC domain containing protein. Oryza_sativa
OG0000012
Os08t0235600-00 Hypothetical conserved gene.   Oryza_sativa
OG0000012
Os08t0235651-00 NB-ARC domain containing protein. Oryza_sativa
OG0000012
Os08t0246100-01 Hypothetical conserved gene.   Oryza_sativa
OG0000012
Os08t0260800-00 Similar to NB-ARC domain containing protein.
Oryza_sativa   OG0000012
Os08t0261000-01 NB-ARC domain containing protein. Oryza_sativa
OG0000012
Os08t0265300-00 Disease resistance protein domain containing
protein. Oryza_sativa   OG0000012
Os08t0293300-01 NB-ARC domain containing protein. Oryza_sativa
OG0000012
Os08t0296600-01 NB-ARC domain containing protein. Oryza_sativa
OG0000012
Os08t0296700-01 NB-ARC domain containing protein. Oryza_sativa
OG0000012
Os08t0332600-00 Disease resistance protein domain containing
protein. Oryza_sativa   OG0000012
Os08t0372700-00 Hypothetical conserved gene.   Oryza_sativa
OG0000012
Os08t0373000-00 Hypothetical conserved gene.   Oryza_sativa
OG0000012
Os08t0387700-01 NB-ARC domain containing protein. Oryza_sativa
OG0000012
Os08t0388300-01 NB-ARC domain containing protein. Oryza_sativa
OG0000012
Os08t0412100-01 NB-ARC domain containing protein. Oryza_sativa
OG0000012
Os08t0424700-00 NB-ARC domain containing protein. Oryza_sativa
OG0000012

Os08t0539400-01 Similar to Resistance protein T10rga2-1A.
Oryza_sativa OG0000012
Os08t0539700-01 NB-ARC domain containing protein. Oryza_sativa
OG0000012
Os08t0542200-01 Similar to Resistance gene analog PIC25 (Fragment).
Oryza_sativa OG0000012
Os08t0543050-00 Similar to NBS2-RDG2A. Oryza_sativa
OG0000012
Os08t0543100-00 Similar to NBS-LRR type resistance protein
(Fragment). Oryza_sativa OG0000012
Os08t0543500-01 Similar to NBS-LRR type resistance protein
(Fragment). Oryza_sativa OG0000012
Os09t0267800-01 Hypothetical conserved gene. Oryza_sativa
OG0000012
Os09t0270700-00 Disease resistance protein domain containing
protein. Oryza_sativa OG0000012
Os09t0313500-01 NB-ARC domain containing protein. Oryza_sativa
OG0000012
Os09t0313600-00 Disease resistance protein domain containing
protein. Oryza_sativa OG0000012
Os09t0314200-00 Disease resistance protein domain containing
protein. Oryza_sativa OG0000012
Os09t0327600-01 Similar to Pi5-1. Oryza_sativa OG0000012
Os09t0327800-00 Similar to Pi5-2. Oryza_sativa OG0000012
Os09t0357400-00 Disease resistance protein domain containing
protein. Oryza_sativa OG0000012
Os09t0365000-00 Disease resistance protein domain containing
protein. Oryza_sativa OG0000012
Os09t0365300-01 NB-ARC domain containing protein. Oryza_sativa
OG0000012
Os09t0479500-01 NB-ARC domain containing protein. Oryza_sativa
OG0000012
Os09t0517100-01 NB-ARC domain containing protein. Oryza_sativa
OG0000012
Os09t0517200-00 Hypothetical conserved gene. Oryza_sativa
OG0000012
Os10t0124400-00 Hypothetical conserved gene. Oryza_sativa
OG0000012
Os10t0125700-00 NB-ARC domain containing protein. Oryza_sativa
OG0000012
Os10t0130600-00 Hypothetical conserved gene. Oryza_sativa
OG0000012
Os10t0130800-01 Conserved hypothetical protein. Oryza_sativa
OG0000012
Os10t0131000-00 Similar to NB-ARC domain containing protein,
expressed. Oryza_sativa OG0000012
Os10t0131100-00 Similar to NB-ARC domain containing protein.
Oryza_sativa OG0000012
Os10t0131800-01 Similar to NBS-LRR class disease resistance protein.
Oryza_sativa OG0000012
Os10t0132500-01 Hypothetical conserved gene. Oryza_sativa
OG0000012
Os10t0132700-01 Similar to NB-ARC domain containing protein,
expressed. Oryza_sativa OG0000012

Os10t0136100-01 Similar to Hv1LRR2.	Oryza_sativa	OG0000012
Os10t0161400-01 NB-ARC domain containing protein.	Oryza_sativa	OG0000012
Os10t0162832-01 Similar to LRR14.	Oryza_sativa	OG0000012
Os10t0162854-01 Similar to OSIGBa0106P14.3 protein.	Oryza_sativa	OG0000012
Os10t0163040-01 Similar to Blast resistance protein.	Oryza_sativa	OG0000012
Os10t0183000-01 NB-LRR protein, Immune response	Oryza_sativa	OG0000012
Os10t0358200-00 Similar to NB-ARC domain containing protein.	Oryza_sativa	OG0000012
Os10t0370400-01 Similar to NB-ARC domain containing protein.	Oryza_sativa	OG0000012
Os10t0394400-01 Disease resistance protein domain containing protein.	Oryza_sativa	OG0000012
Os10t0568600-00 Disease resistance protein domain containing protein.	Oryza_sativa	OG0000012
Os11t0130900-00 Similar to NB-ARC domain containing protein, expressed.	Oryza_sativa	OG0000012
Os11t0211300-01 Similar to NBS-LRR disease resistance protein homologue (Fragment).	Oryza_sativa	OG0000012
Os11t0212000-00 Similar to Leucine Rich Repeat family protein, expressed.	Oryza_sativa	OG0000012
Os11t0213800-01 Similar to NBS-LRR disease resistance protein homologue (Fragment).	Oryza_sativa	OG0000012
Os11t0222900-01 NB-ARC domain containing protein.	Oryza_sativa	OG0000012
Os11t0223201-01 Similar to NB-ARC domain containing protein, expressed.	Oryza_sativa	OG0000012
Os11t0224900-00 Similar to NBS-LRR protein (Fragment).	Oryza_sativa	OG0000012
Os11t0225300-01 NB-ARC domain containing protein.	Oryza_sativa	OG0000012
Os11t0226933-01 NB-ARC domain containing protein.	Oryza_sativa	OG0000012
Os11t0227100-00 Similar to NBS-LRR-like protein.	Oryza_sativa	OG0000012
Os11t0227200-01 Similar to NBS-LRR disease resistance protein homologue.	Oryza_sativa	OG0000012
Os11t0227700-01 Similar to Non-TIR-NBS-LRR type resistance protein.	Oryza_sativa	OG0000012
Os11t0227800-01 NB-ARC domain containing protein.	Oryza_sativa	OG0000012
Os11t0229300-00 Similar to NBS-LRR-like protein.	Oryza_sativa	OG0000012
Os11t0229400-01 Similar to NBS-LRR protein (Fragment).	Oryza_sativa	OG0000012
Os11t0229500-01 Similar to NBS-LRR protein (Fragment).	Oryza_sativa	OG0000012
Os11t0237600-01 Hypothetical conserved gene.	Oryza_sativa	OG0000012
Os11t0237900-00 Similar to NB-ARC domain containing protein.

Oryza_sativa   OG0000012
Os11t0238000-00 Similar to NB-ARC domain containing protein.
Oryza_sativa   OG0000012
Os11t0238700-00 Similar to NB-ARC domain containing protein.
Oryza_sativa   OG0000012
Os11t0249000-01 NB-ARC domain containing protein. Oryza_sativa
OG0000012
Os11t0258500-00 Disease resistance protein domain containing
protein. Oryza_sativa   OG0000012
Os11t0263000-00 Similar to Leucine Rich Repeat family protein,
expressed.   Oryza_sativa   OG0000012
Os11t0266500-01 Similar to NB-ARC domain containing protein,
expressed.   Oryza_sativa   OG0000012
Os11t0270500-01 NB-ARC domain containing protein. Oryza_sativa
OG0000012
Os11t0429100-00 Hypothetical conserved gene.   Oryza_sativa
OG0000012
Os11t0462500-00 Similar to NB-ARC domain containing protein.
Oryza_sativa   OG0000012
Os11t0479100-00 Similar to NB-ARC domain containing protein,
expressed.   Oryza_sativa   OG0000012
Os11t0481150-00 NB-ARC domain containing protein. Oryza_sativa
OG0000012
Os11t0485900-01 Similar to NB-ARC domain containing protein,
expressed.   Oryza_sativa   OG0000012
Os11t0492200-00 Similar to NB-ARC domain containing protein.
Oryza_sativa   OG0000012
Os11t0492300-00 Similar to NB-ARC domain containing protein.
Oryza_sativa   OG0000012
Os11t0492500-00 Similar to NB-ARC domain containing protein.
Oryza_sativa   OG0000012
Os11t0492900-00 Similar to NBS-LRR type resistance protein
(Fragment).   Oryza_sativa   OG0000012
Os11t0493100-00 Similar to NB-ARC domain containing protein.
Oryza_sativa   OG0000012
Os11t0493700-00 Similar to NBS-LRR type resistance protein
(Fragment).   Oryza_sativa   OG0000012
Os11t0494100-00 Similar to NBS-LRR type resistance protein
(Fragment).   Oryza_sativa   OG0000012
Os11t0550100-01 Similar to NB-ARC domain containing protein,
expressed.   Oryza_sativa   OG0000012
Os11t0550500-01 Similar to LZ-NBS-LRR class RGA.   Oryza_sativa
OG0000012
Os11t0555300-00 Similar to Rp1-like protein.   Oryza_sativa
OG0000012
Os11t0588400-01 Similar to NB-ARC domain containing protein,
expressed.   Oryza_sativa   OG0000012
Os11t0588600-01 NB-ARC domain containing protein. Oryza_sativa
OG0000012
Os11t0589800-00 Similar to NB-ARC domain containing protein.
Oryza_sativa   OG0000012
Os11t0590400-00 Similar to NB-ARC domain containing protein.
Oryza_sativa   OG0000012
Os11t0590700-01 NB-ARC domain containing protein. Oryza_sativa

OG0000012
Os11t0592500-01 Similar to NB-ARC domain containing protein, expressed.	Oryza_sativa	OG0000012
Os11t0604900-00 Similar to NB-ARC domain containing protein.	Oryza_sativa	OG0000012
Os11t0606100-01 NB-ARC domain containing protein.	Oryza_sativa	OG0000012
Os11t0606500-01 Similar to NB-ARC domain containing protein, expressed.	Oryza_sativa	OG0000012
Os11t0610000-00 Similar to NB-ARC domain containing protein.	Oryza_sativa	OG0000012
Os11t0623800-00 Similar to NB-ARC domain containing protein.	Oryza_sativa	OG0000012
Os11t0629550-00 Similar to Disease resistance protein RPM1 homolog.	Oryza_sativa	OG0000012
Os11t0633500-01 Similar to NBS-LRR disease resistance protein homologue (Fragment).	Oryza_sativa	OG0000012
Os11t0639600-01 NB-ARC domain containing protein.	Oryza_sativa	OG0000012
Os11t0640600-00 NB-ARC domain containing protein.	Oryza_sativa	OG0000012
Os11t0657900-00 Similar to NB-ARC domain containing protein.	Oryza_sativa	OG0000012
Os11t0668100-00 Similar to NB-ARC domain containing protein, expressed.	Oryza_sativa	OG0000012
Os11t0673600-00 Similar to NB-ARC domain containing protein, expressed.	Oryza_sativa	OG0000012
Os11t0673900-00 Similar to NB-ARC domain containing protein.	Oryza_sativa	OG0000012
Os11t0674400-00 Similar to NB-ARC domain containing protein, expressed.	Oryza_sativa	OG0000012
Os11t0675200-00 Similar to NB-ARC domain containing protein, expressed.	Oryza_sativa	OG0000012
Os11t0676100-00 Similar to NB-ARC domain containing protein, expressed.	Oryza_sativa	OG0000012
Os11t0676200-00 Similar to NBS-LRR-like protein NBA2 (Fragment).	Oryza_sativa	OG0000012
Os11t0676800-00 Similar to NB-ARC domain containing protein.	Oryza_sativa	OG0000012
Os11t0677000-00 Similar to NB-ARC domain containing protein, expressed.	Oryza_sativa	OG0000012
Os11t0677101-00 Similar to NB-ARC domain containing protein.	Oryza_sativa	OG0000012
Os11t0678400-00 Similar to NB-ARC domain containing protein, expressed.	Oryza_sativa	OG0000012
Os11t0682501-00 Disease resistance protein domain containing protein.	Oryza_sativa	OG0000012
Os11t0684100-01 NB-ARC domain containing protein.	Oryza_sativa	OG0000012
Os11t0684700-01 Similar to NB-ARC domain containing protein, expressed.	Oryza_sativa	OG0000012
Os11t0686400-00 Similar to WRKY transcription factor 41.	Oryza_sativa	OG0000012
Os11t0686500-01 NB-ARC domain containing protein.	Oryza_sativa

OG0000012
Os11t0686900-00 Similar to NB-ARC domain containing protein, expressed.	Oryza_sativa	OG0000012
Os11t0687800-01 NB-ARC domain containing protein.	Oryza_sativa	OG0000012
Os11t0700100-01 Similar to LZ-NBS-LRR class RGA.	Oryza_sativa	OG0000012
Os11t0704100-00 Similar to NB-ARC domain containing protein.	Oryza_sativa	OG0000012
Os12t0166600-00 Similar to NB-ARC domain containing protein.	Oryza_sativa	OG0000012
Os12t0198900-00 Similar to NB-ARC domain containing protein.	Oryza_sativa	OG0000012
Os12t0199100-01 NB-ARC domain containing protein.	Oryza_sativa	OG0000012
Os12t0204600-01 NB-ARC domain containing protein.	Oryza_sativa	OG0000012
Os12t0204800-01 Disease resistance protein domain containing protein.	Oryza_sativa	OG0000012
Os12t0209901-01 Similar to NB-ARC domain containing protein, expressed.	Oryza_sativa	OG0000012
Os12t0237900-01 NB-ARC domain containing protein.	Oryza_sativa	OG0000012
Os12t0246700-01 NB-ARC domain containing protein.	Oryza_sativa	OG0000012
Os12t0270300-01 NB-ARC domain containing protein.	Oryza_sativa	OG0000012
Os12t0272800-01 Hypothetical conserved gene.	Oryza_sativa	OG0000012
Os12t0273300-01 Similar to NB-ARC domain containing protein.	Oryza_sativa	OG0000012
Os12t0281300-01 Similar to NB-ARC domain containing protein, expressed.	Oryza_sativa	OG0000012
Os12t0438300-01 NB-ARC domain containing protein.	Oryza_sativa	OG0000012
Os12t0467300-01 NB-ARC domain containing protein.	Oryza_sativa	OG0000012
Os12t0481400-00 Similar to NBS-LRR disease resistance protein homologue.	Oryza_sativa	OG0000012
Os12t0481700-00 Disease resistance protein domain containing protein.	Oryza_sativa	OG0000012
Os12t0482100-00 Disease resistance protein domain containing protein.	Oryza_sativa	OG0000012
Os12t0489800-01 Similar to NBS-LRR protein (Fragment).	Oryza_sativa	OG0000012
Os12t0495600-01 NB-ARC domain containing protein.	Oryza_sativa	OG0000012
Os12t0500500-01 NB-ARC domain containing protein.	Oryza_sativa	OG0000012
Os12t0511400-01 Similar to NB-ARC domain containing protein.	Oryza_sativa	OG0000012
Os12t0511500-01 Disease resistance protein domain containing protein.	Oryza_sativa	OG0000012
Os12t0511600-00 Similar to NB-ARC domain containing protein.

Oryza_sativa OG0000012
Os12t0511900-00 Similar to NB-ARC domain containing protein, expressed. Oryza_sativa OG0000012
Os12t0512400-01 Similar to NB-ARC domain containing protein, expressed. Oryza_sativa OG0000012
Os12t0516300-01 NB-ARC domain containing protein. Oryza_sativa OG0000012
Os12t0552900-01 NB-ARC domain containing protein. Oryza_sativa OG0000012
Os12t0553200-01 NB-ARC domain containing protein. Oryza_sativa OG0000012
Os12t0553600-01 Similar to NB-ARC domain containing protein. Oryza_sativa OG0000012
Os12t0559600-00 Similar to Resistance protein RGA2. Oryza_sativa OG0000012
Os12t0564800-01 NB-ARC domain containing protein. Oryza_sativa OG0000012
Os12t0565100-01 NB-ARC domain containing protein. Oryza_sativa OG0000012
Os01t0781100-01 Similar to NBS-LRR protein (Fragment). Oryza_sativa OG0000012
Os01t0781200-01 Similar to Rust resistance protein. Oryza_sativa OG0000012
Os01t0781700-00 Similar to Blast resistance protein Pi37. Oryza_sativa OG0000012
Os01t0782100-01 Protein with NBS (nucleotide-binding site) and LRR(leucine-rich repeat), Race-specific resistance to blast Oryza_sativa OG0000012
Os01t0149350-00 NB-ARC domain containing protein. Oryza_sativa OG0000012
Os01t0149500-01 NBS-LRR domain-containing protein, Rice blast disease resistance Oryza_sativa OG0000012
Os01t0937300-01 NB-ARC domain containing protein. Oryza_sativa OG0000012
Os01t0937400-01 NB-ARC domain containing protein. Oryza_sativa OG0000012
Os02t0118800-00 Similar to NBS-LRR disease resistance protein homologue (Fragment). Oryza_sativa OG0000012
Os02t0118875-00 NB-ARC domain containing protein. Oryza_sativa OG0000012
Os02t0118900-00 Similar to NBS-LRR disease resistance protein homologue (Fragment). Oryza_sativa OG0000012
Os02t0118950-00 Similar to NBS-LRR disease resistance protein homologue (Fragment). Oryza_sativa OG0000012
Os02t0191000-00 NB-ARC domain containing protein. Oryza_sativa OG0000012
Os02t0262600-00 Disease resistance protein domain containing protein. Oryza_sativa OG0000012
Os02t0262800-01 Similar to NBS-LRR protein (Fragment). Oryza_sativa OG0000012
Os02t0263432-00 Conserved hypothetical protein. Oryza_sativa OG0000012
Os02t0272900-01 NB-ARC domain containing protein. Oryza_sativa OG0000012

Os02t0281200-01 Similar to NBS-LRR protein (Fragment).
Oryza_sativa OG0000012
Os02t0282000-01 NB-ARC domain containing protein. Oryza_sativa
OG0000012
Os02t0282500-00 Disease resistance protein domain containing
protein. Oryza_sativa OG0000012
Os02t0286850-00 Similar to Stripe rust resistance protein Yr10.
Oryza_sativa OG0000012
Os01t0178900-01 Similar to Symbiosis-related disease resistance
protein (Fragment). Oryza_sativa OG0000012
Os02t0300500-00 Disease resistance protein domain containing
protein. Oryza_sativa OG0000012
Os02t0301800-01 NB-ARC domain containing protein. Oryza_sativa
OG0000012
Os02t0307075-00 Conserved hypothetical protein. Oryza_sativa
OG0000012
Os02t0307400-00 Leucine-rich repeat domain containing protein.
Oryza_sativa OG0000012
Os02t0307700-00 Similar to RDG2A. Oryza_sativa OG0000012
Os02t0456800-01 Similar to Pollen signalling protein with adenylyl
cyclase activity (Fragment). Oryza_sativa OG0000012
Os02t0504200-00 NBS-LRR protein (Fragment). Oryza_sativa
OG0000012
Os02t0627900-00 Similar to cDNA clone:J013002N02, full insert
sequence. Oryza_sativa OG0000012
CDF36555 pep supercontig:ASM35022v2:HG001787:103019:107821:1
gene:CHC_T00008351001 transcript:CDF36555
gene_biotype:protein_coding transcript_biotype:protein_coding
description:NB-ARC protein Chondrus_crispus OG0000012
CDF40008 pep supercontig:ASM35022v2:HG002104:12974:17650:1
gene:CHC_T00009468001 transcript:CDF40008
gene_biotype:protein_coding transcript_biotype:protein_coding
description:WD40-repeat containing protein Chondrus_crispus
OG0000012
CDF40995 pep supercontig:ASM35022v2:HG002294:314998:316880:1
gene:CHC_T00008609001 transcript:CDF40995
gene_biotype:protein_coding transcript_biotype:protein_coding
description:NB-ARC protein Chondrus_crispus OG0000012
CDF37277 pep supercontig:ASM35022v2:HG001826:9227:14568:-1
gene:CHC_T00008788001 transcript:CDF37277
gene_biotype:protein_coding transcript_biotype:protein_coding
description:WD40-repeat containing protein Chondrus_crispus
OG0000012
Cre07.g322884.t1.2 pacid=30775063 transcript=Cre07.g322884.t1.2
locus=Cre07.g322884 ID=Cre07.g322884.t1.2.v5.5 annot-version=v5.5
Chlamydomonas_reinhardtii OG0000013
Cre09.g406750.t1.1 pacid=30781135 transcript=Cre09.g406750.t1.1
locus=Cre09.g406750 ID=Cre09.g406750.t1.1.v5.5 annot-version=v5.5
Chlamydomonas_reinhardtii OG0000013
jgi|ChlNC64A_1|140490|IGS.gm_3_00229 Chlorella_variabilis
OG0000013
jgi|ChlNC64A_1|134998|IGS.gm_12_00384 Chlorella_variabilis
OG0000013
jgi|ChlNC64A_1|134126|IGS.gm_10_00417 Chlorella_variabilis

OG0000013
jgi|ChlNC64A_1|57900|estExt_fgenesh3_pg.C_100197
Chlorella_variabilis   OG0000013
62318 pacid=27387066 transcript=62318 locus=Genemark1.3101_g
ID=62318.2.0.227 annot-version=v2.0   Coccomyxa_subellipsoidea
OG0000013
65542 pacid=27387078 transcript=65542 locus=estExt_Genemark1.C_50427
ID=65542.2.0.227 annot-version=v2.0   Coccomyxa_subellipsoidea
OG0000013
46961 pacid=27387256 transcript=46961
locus=estExt_fgenesh1_pg.C_50310 ID=46961.2.0.227 annot-version=v2.0
Coccomyxa_subellipsoidea OG0000013
47003 pacid=27387469 transcript=47003
locus=estExt_fgenesh1_pg.C_50369 ID=47003.2.0.227 annot-version=v2.0
Coccomyxa_subellipsoidea OG0000013
66757 pacid=27392052 transcript=66757
locus=estExt_Genemark1.C_110215 ID=66757.2.0.227 annot-version=v2.0
Coccomyxa_subellipsoidea OG0000013
46052 pacid=27393167 transcript=46052
locus=estExt_fgenesh1_pg.C_20075 ID=46052.2.0.227 annot-version=v2.0
Coccomyxa_subellipsoidea OG0000013
67635 pacid=27394582 transcript=67635
locus=estExt_Genemark1.C_170091 ID=67635.2.0.227 annot-version=v2.0
Coccomyxa_subellipsoidea OG0000013
kfl00491_0040_v1.1   Klebsormidium_flaccidum OG0000013
kfl00491_0050_v1.1   Klebsormidium_flaccidum OG0000013
kfl00491_0060_v1.1   Klebsormidium_flaccidum OG0000013
kfl00618_0040_v1.1   Klebsormidium_flaccidum OG0000013
kfl00006_0080_v1.1   Klebsormidium_flaccidum OG0000013
kfl00010_0050_v1.1   Klebsormidium_flaccidum OG0000013
kfl00010_0060_v1.1   Klebsormidium_flaccidum OG0000013
Pp3c2_1380V3.1.p pacid=32936361 transcript=Pp3c2_1380V3.1
locus=Pp3c2_1380 annot-version=v3.3   Physcomitrella_patens
OG0000013
Pp3c20_16410V3.1.p pacid=32947631 transcript=Pp3c20_16410V3.1
locus=Pp3c20_16410 annot-version=v3.3   Physcomitrella_patens
OG0000013
Pp3c23_12020V3.1.p pacid=32949386 transcript=Pp3c23_12020V3.1
locus=Pp3c23_12020 annot-version=v3.3   Physcomitrella_patens
OG0000013
Pp3c27_6100V3.1.p pacid=32951992 transcript=Pp3c27_6100V3.1
locus=Pp3c27_6100 annot-version=v3.3   Physcomitrella_patens
OG0000013
Pp3c5_19530V3.1.p pacid=32955869 transcript=Pp3c5_19530V3.1
locus=Pp3c5_19530 annot-version=v3.3   Physcomitrella_patens
OG0000013
Pp3c11_4850V3.1.p pacid=32958237 transcript=Pp3c11_4850V3.1
locus=Pp3c11_4850 annot-version=v3.3   Physcomitrella_patens
OG0000013
Pp3c14_15170V3.1.p pacid=32961128 transcript=Pp3c14_15170V3.1
locus=Pp3c14_15170 annot-version=v3.3   Physcomitrella_patens
OG0000013
Pp3c1_6260V3.1.p pacid=32966746 transcript=Pp3c1_6260V3.1
locus=Pp3c1_6260 annot-version=v3.3   Physcomitrella_patens

OG0000013
Pp3c12_18470V3.1.p pacid=32972744 transcript=Pp3c12_18470V3.1
locus=Pp3c12_18470 annot-version=v3.3   Physcomitrella_patens
OG0000013
Pp3c12_21260V3.1.p pacid=32973673 transcript=Pp3c12_21260V3.1
locus=Pp3c12_21260 annot-version=v3.3   Physcomitrella_patens
OG0000013
Pp3c6_10100V3.1.p pacid=32976252 transcript=Pp3c6_10100V3.1
locus=Pp3c6_10100 annot-version=v3.3   Physcomitrella_patens
OG0000013
Pp3c6_1850V3.1.p pacid=32978477 transcript=Pp3c6_1850V3.1
locus=Pp3c6_1850 annot-version=v3.3   Physcomitrella_patens
OG0000013
Pp3c16_20890V3.1.p pacid=32984892 transcript=Pp3c16_20890V3.1
locus=Pp3c16_20890 annot-version=v3.3   Physcomitrella_patens
OG0000013
Pp3c16_5590V3.1.p pacid=32986206 transcript=Pp3c16_5590V3.1
locus=Pp3c16_5590 annot-version=v3.3   Physcomitrella_patens
OG0000013
Pp3c24_8200V3.1.p pacid=32910201 transcript=Pp3c24_8200V3.1
locus=Pp3c24_8200 annot-version=v3.3   Physcomitrella_patens
OG0000013
Pp3c26_9010V3.1.p pacid=32918480 transcript=Pp3c26_9010V3.1
locus=Pp3c26_9010 annot-version=v3.3   Physcomitrella_patens
OG0000013
Pp3c4_13320V3.1.p pacid=32919144 transcript=Pp3c4_13320V3.1
locus=Pp3c4_13320 annot-version=v3.3   Physcomitrella_patens
OG0000013
Pp3c10_10730V3.1.p pacid=32901696 transcript=Pp3c10_10730V3.1
locus=Pp3c10_10730 annot-version=v3.3   Physcomitrella_patens
OG0000013
AT1G10400.1 | UDP-Glycosyltransferase superfamily protein |
Chr1:3414869-3416358 REVERSE LENGTH=467 | 201606
Arabidopsis_thaliana   OG0000013
AT2G36750.1 | UDP-glucosyl transferase 73C1 | Chr2:15410531-15412006
REVERSE LENGTH=491 | 201606   Arabidopsis_thaliana
OG0000013
AT2G36760.1 | UDP-glucosyl transferase 73C2 | Chr2:15413042-15414532
REVERSE LENGTH=496 | 201606   Arabidopsis_thaliana
OG0000013
AT2G36770.1 | UDP-Glycosyltransferase superfamily protein |
Chr2:15415227-15416717 REVERSE LENGTH=496 | 201606
Arabidopsis_thaliana   OG0000013
AT2G36780.1 | UDP-Glycosyltransferase superfamily protein |
Chr2:15417618-15419108 REVERSE LENGTH=496 | 201606
Arabidopsis_thaliana   OG0000013
AT2G36790.1 | UDP-glucosyl transferase 73C6 | Chr2:15420339-15421826
REVERSE LENGTH=495 | 201606   Arabidopsis_thaliana
OG0000013
AT2G36800.1 | don-glucosyltransferase 1 | Chr2:15423493-15424980
REVERSE LENGTH=495 | 201606   Arabidopsis_thaliana
OG0000013
AT2G36970.1 | UDP-Glycosyltransferase superfamily protein |
Chr2:15529050-15530712 FORWARD LENGTH=490 | 201606

Arabidopsis_thaliana   OG0000013
AT2G43820.1 | UDP-glucosyltransferase 74F2 | Chr2:18152279-18153715
FORWARD LENGTH=449 | 201606   Arabidopsis_thaliana
OG0000013
AT2G43840.1 | UDP-glycosyltransferase 74 F1 | Chr2:18157681-18159166
FORWARD LENGTH=449 | 201606   Arabidopsis_thaliana
OG0000013
AT3G02100.1 | UDP-Glycosyltransferase superfamily protein |
Chr3:368840-370484 REVERSE LENGTH=464 | 201606
Arabidopsis_thaliana   OG0000013
AT3G11340.1 | UDP-Glycosyltransferase superfamily protein |
Chr3:3556728-3558149 FORWARD LENGTH=447 | 201606
Arabidopsis_thaliana   OG0000013
AT3G16520.1 | UDP-glucosyl transferase 88A1 | Chr3:5618807-5620833
REVERSE LENGTH=451 | 201606   Arabidopsis_thaliana
OG0000013
AT3G21560.1 | UDP-Glycosyltransferase superfamily protein |
Chr3:7595884-7597374 FORWARD LENGTH=496 | 201606
Arabidopsis_thaliana   OG0000013
AT3G21750.1 | UDP-glucosyl transferase 71B1 | Chr3:7664565-7665986
FORWARD LENGTH=473 | 201606   Arabidopsis_thaliana
OG0000013
AT3G21760.1 | UDP-Glycosyltransferase superfamily protein |
Chr3:7667099-7668556 FORWARD LENGTH=485 | 201606
Arabidopsis_thaliana   OG0000013
AT3G21780.1 | UDP-glucosyl transferase 71B6 | Chr3:7675051-7676490
REVERSE LENGTH=479 | 201606   Arabidopsis_thaliana
OG0000013
AT3G21790.1 | UDP-Glycosyltransferase superfamily protein |
Chr3:7676927-7678414 REVERSE LENGTH=495 | 201606
Arabidopsis_thaliana   OG0000013
AT3G21800.1 | UDP-glucosyl transferase 71B8 | Chr3:7680243-7681685
REVERSE LENGTH=480 | 201606   Arabidopsis_thaliana
OG0000013
AT3G22250.1 | UDP-Glycosyltransferase superfamily protein |
Chr3:7867806-7870053 FORWARD LENGTH=461 | 201606
Arabidopsis_thaliana   OG0000013
AT3G29630.1 | UDP-Glycosyltransferase superfamily protein |
Chr3:11447178-11448524 REVERSE LENGTH=448 | 201606
Arabidopsis_thaliana   OG0000013
AT3G46650.1 | UDP-Glycosyltransferase superfamily protein |
Chr3:17185561-17187812 REVERSE LENGTH=435 | 201606
Arabidopsis_thaliana   OG0000013
AT3G46660.1 | UDP-glucosyl transferase 76E12 |
Chr3:17189406-17190862 REVERSE LENGTH=458 | 201606
Arabidopsis_thaliana   OG0000013
AT3G46670.1 | UDP-glucosyl transferase 76E11 |
Chr3:17192795-17194227 REVERSE LENGTH=451 | 201606
Arabidopsis_thaliana   OG0000013
AT3G46680.1 | UDP-Glycosyltransferase superfamily protein |
Chr3:17195318-17196743 REVERSE LENGTH=449 | 201606
Arabidopsis_thaliana   OG0000013
AT3G46690.1 | UDP-Glycosyltransferase superfamily protein |
Chr3:17197760-17199197 REVERSE LENGTH=452 | 201606

Arabidopsis_thaliana OG0000013
AT3G46700.1 | UDP-Glycosyltransferase superfamily protein | Chr3:17200430-17201848 REVERSE LENGTH=447 | 201606 Arabidopsis_thaliana OG0000013
AT3G46720.1 | UDP-Glycosyltransferase superfamily protein | Chr3:17210930-17212348 REVERSE LENGTH=447 | 201606 Arabidopsis_thaliana OG0000013
AT3G50740.1 | UDP-glucosyl transferase 72E1 | Chr3:18855348-18856811 REVERSE LENGTH=487 | 201606 Arabidopsis_thaliana OG0000013
AT3G53150.1 | UDP-glucosyl transferase 73D1 | Chr3:19697736-19699286 REVERSE LENGTH=516 | 201606 Arabidopsis_thaliana OG0000013
AT3G53160.1 | UDP-glucosyl transferase 73C7 | Chr3:19702485-19703957 REVERSE LENGTH=490 | 201606 Arabidopsis_thaliana OG0000013
AT3G55700.1 | UDP-Glycosyltransferase superfamily protein | Chr3:20671202-20673278 FORWARD LENGTH=460 | 201606 Arabidopsis_thaliana OG0000013
AT3G55710.1 | UDP-Glycosyltransferase superfamily protein | Chr3:20673847-20675811 FORWARD LENGTH=464 | 201606 Arabidopsis_thaliana OG0000013
AT4G01070.1 | UDP-Glycosyltransferase superfamily protein | Chr4:461858-463300 REVERSE LENGTH=480 | 201606 Arabidopsis_thaliana OG0000013
AT4G09500.1 | UDP-Glycosyltransferase superfamily protein | Chr4:6018250-6019578 FORWARD LENGTH=417 | 201606 Arabidopsis_thaliana OG0000013
AT4G14090.1 | UDP-Glycosyltransferase superfamily protein | Chr4:8122434-8123804 REVERSE LENGTH=456 | 201606 Arabidopsis_thaliana OG0000013
AT4G15260.1 | UDP-Glycosyltransferase superfamily protein | Chr4:8714065-8715144 FORWARD LENGTH=359 | 201606 Arabidopsis_thaliana OG0000013
AT4G15270.1 | glucosyltransferase-like protein | Chr4:8717008-8717796 REVERSE LENGTH=233 | 201606 Arabidopsis_thaliana OG0000013
AT4G15280.1 | UDP-glucosyl transferase 71B5 | Chr4:8719182-8720618 FORWARD LENGTH=478 | 201606 Arabidopsis_thaliana OG0000013
AT4G15480.1 | UDP-Glycosyltransferase superfamily protein | Chr4:8849000-8850472 REVERSE LENGTH=490 | 201606 Arabidopsis_thaliana OG0000013
AT4G15490.1 | UDP-Glycosyltransferase superfamily protein | Chr4:8852864-8854303 REVERSE LENGTH=479 | 201606 Arabidopsis_thaliana OG0000013
AT4G15500.1 | UDP-Glycosyltransferase superfamily protein | Chr4:8857095-8858522 REVERSE LENGTH=475 | 201606 Arabidopsis_thaliana OG0000013
AT4G15550.1 | indole-3-acetate beta-D-glucosyltransferase | Chr4:8877877-8879301 REVERSE LENGTH=474 | 201606 Arabidopsis_thaliana OG0000013
AT4G27560.1 | UDP-Glycosyltransferase superfamily protein | Chr4:13760114-13761481 REVERSE LENGTH=455 | 201606

Arabidopsis_thaliana   OG0000013
AT4G27570.1 | UDP-Glycosyltransferase superfamily protein | Chr4:13763657-13765018 REVERSE LENGTH=453 | 201606   Arabidopsis_thaliana   OG0000013
AT4G34131.1 | UDP-glucosyl transferase 73B3 | Chr4:16343268-16344713 REVERSE LENGTH=481 | 201606   Arabidopsis_thaliana   OG0000013
AT4G34135.1 | UDP-glucosyltransferase 73B2 | Chr4:16345476-16347016 REVERSE LENGTH=483 | 201606   Arabidopsis_thaliana   OG0000013
AT4G34138.1 | UDP-glucosyl transferase 73B1 | Chr4:16348267-16349858 REVERSE LENGTH=488 | 201606   Arabidopsis_thaliana   OG0000013
AT4G36770.1 | UDP-Glycosyltransferase superfamily protein | Chr4:17330217-17331590 REVERSE LENGTH=457 | 201606   Arabidopsis_thaliana   OG0000013
AT5G03490.1 | UDP-Glycosyltransferase superfamily protein | Chr5:871550-872947 FORWARD LENGTH=465 | 201606   Arabidopsis_thaliana   OG0000013
AT5G05860.1 | UDP-glucosyl transferase 76C2 | Chr5:1765545-1767348 FORWARD LENGTH=450 | 201606   Arabidopsis_thaliana   OG0000013
AT5G05870.1 | UDP-glucosyl transferase 76C1 | Chr5:1767683-1769177 FORWARD LENGTH=464 | 201606   Arabidopsis_thaliana   OG0000013
AT5G05880.1 | UDP-Glycosyltransferase superfamily protein | Chr5:1769648-1771515 FORWARD LENGTH=451 | 201606   Arabidopsis_thaliana   OG0000013
AT5G05890.1 | UDP-Glycosyltransferase superfamily protein | Chr5:1772567-1774012 FORWARD LENGTH=455 | 201606   Arabidopsis_thaliana   OG0000013
AT5G05900.1 | UDP-Glycosyltransferase superfamily protein | Chr5:1774513-1776381 FORWARD LENGTH=450 | 201606   Arabidopsis_thaliana   OG0000013
AT5G12890.1 | UDP-Glycosyltransferase superfamily protein | Chr5:4069658-4071124 REVERSE LENGTH=488 | 201606   Arabidopsis_thaliana   OG0000013
AT5G14860.1 | UDP-Glycosyltransferase superfamily protein | Chr5:4805887-4807759 FORWARD LENGTH=492 | 201606   Arabidopsis_thaliana   OG0000013
AT5G17030.1 | UDP-glucosyl transferase 78D3 | Chr5:5603198-5604723 REVERSE LENGTH=459 | 201606   Arabidopsis_thaliana   OG0000013
AT5G17040.1 | UDP-Glycosyltransferase superfamily protein | Chr5:5605358-5606963 REVERSE LENGTH=442 | 201606   Arabidopsis_thaliana   OG0000013
AT5G17050.1 | UDP-glucosyl transferase 78D2 | Chr5:5607828-5609392 REVERSE LENGTH=460 | 201606   Arabidopsis_thaliana   OG0000013
AT1G22340.1 | UDP-glucosyl transferase 85A7 | Chr1:7890464-7892090 REVERSE LENGTH=487 | 201606   Arabidopsis_thaliana   OG0000013
AT1G22360.1 | UDP-glucosyl transferase 85A2 | Chr1:7895068-7897527 REVERSE LENGTH=481 | 201606   Arabidopsis_thaliana

OG0000013
AT1G22370.1 | UDP-glucosyl transferase 85A5 | Chr1:7898116-7899045
REVERSE LENGTH=309 | 201606 Arabidopsis_thaliana
OG0000013
AT1G22380.1 | UDP-glucosyl transferase 85A3 | Chr1:7900522-7902332
REVERSE LENGTH=488 | 201606 Arabidopsis_thaliana
OG0000013
AT1G22400.1 | UDP-Glycosyltransferase superfamily protein |
Chr1:7903851-7906607 REVERSE LENGTH=489 | 201606
Arabidopsis_thaliana OG0000013
AT5G26310.1 | UDP-Glycosyltransferase superfamily protein |
Chr5:9234739-9236184 FORWARD LENGTH=481 | 201606
Arabidopsis_thaliana OG0000013
AT5G37950.1 | UDP-Glycosyltransferase superfamily protein |
Chr5:15116094-15117617 FORWARD LENGTH=351 | 201606
Arabidopsis_thaliana OG0000013
AT5G38010.1 | UDP-Glycosyltransferase superfamily protein |
Chr5:15158342-15160118 FORWARD LENGTH=453 | 201606
Arabidopsis_thaliana OG0000013
AT5G38040.1 | UDP-Glycosyltransferase superfamily protein |
Chr5:15185077-15186508 FORWARD LENGTH=449 | 201606
Arabidopsis_thaliana OG0000013
AT1G24100.1 | UDP-glucosyl transferase 74B1 | Chr1:8525547-8527010
REVERSE LENGTH=460 | 201606 Arabidopsis_thaliana
OG0000013
AT5G49690.1 | UDP-Glycosyltransferase superfamily protein |
Chr5:20189968-20191350 REVERSE LENGTH=460 | 201606
Arabidopsis_thaliana OG0000013
AT5G53990.1 | UDP-Glycosyltransferase superfamily protein |
Chr5:21915707-21917050 REVERSE LENGTH=447 | 201606
Arabidopsis_thaliana OG0000013
AT5G54010.1 | UDP-Glycosyltransferase superfamily protein |
Chr5:21919819-21921180 REVERSE LENGTH=453 | 201606
Arabidopsis_thaliana OG0000013
AT5G54060.1 | UDP-glucose:flavonoid 3-o-glucosyltransferase |
Chr5:21936902-21938308 REVERSE LENGTH=468 | 201606
Arabidopsis_thaliana OG0000013
AT5G59580.1 | UDP-glucosyl transferase 76E1 | Chr5:24006239-24007689
REVERSE LENGTH=453 | 201606 Arabidopsis_thaliana
OG0000013
AT5G59590.1 | UDP-glucosyl transferase 76E2 | Chr5:24009152-24010585
REVERSE LENGTH=449 | 201606 Arabidopsis_thaliana
OG0000013
AT5G65550.1 | UDP-Glycosyltransferase superfamily protein |
Chr5:26198410-26199810 REVERSE LENGTH=466 | 201606
Arabidopsis_thaliana OG0000013
AT5G66690.1 | UDP-Glycosyltransferase superfamily protein |
Chr5:26625155-26626600 FORWARD LENGTH=481 | 201606
Arabidopsis_thaliana OG0000013
AT1G30530.1 | UDP-glucosyl transferase 78D1 | Chr1:10814917-10816374
FORWARD LENGTH=453 | 201606 Arabidopsis_thaliana
OG0000013
AT1G01390.1 | UDP-Glycosyltransferase superfamily protein |
Chr1:148319-149761 REVERSE LENGTH=480 | 201606

Arabidopsis_thaliana	OG0000013
AT1G50580.1 | UDP-Glycosyltransferase superfamily protein | Chr1:18730831-18732177 FORWARD LENGTH=448 | 201606	Arabidopsis_thaliana	OG0000013
AT1G51210.1 | UDP-Glycosyltransferase superfamily protein | Chr1:18987809-18989110 FORWARD LENGTH=433 | 201606	Arabidopsis_thaliana	OG0000013
AT1G01420.1 | UDP-glucosyl transferase 72B3 | Chr1:154566-156011 REVERSE LENGTH=481 | 201606	Arabidopsis_thaliana	OG0000013
AT1G05530.1 | UDP-glucosyl transferase 75B2 | Chr1:1636496-1637863 REVERSE LENGTH=455 | 201606	Arabidopsis_thaliana	OG0000013
AT1G05560.1 | UDP-glucosyltransferase 75B1 | Chr1:1645674-1647083 REVERSE LENGTH=469 | 201606	Arabidopsis_thaliana	OG0000013
AT1G05675.1 | UDP-Glycosyltransferase superfamily protein | Chr1:1701213-1702715 REVERSE LENGTH=453 | 201606	Arabidopsis_thaliana	OG0000013
AT1G05680.1 | Uridine diphosphate glycosyltransferase 74E2 | Chr1:1703196-1704639 REVERSE LENGTH=453 | 201606	Arabidopsis_thaliana	OG0000013
AT1G06000.1 | UDP-Glycosyltransferase superfamily protein | Chr1:1820495-1821802 REVERSE LENGTH=435 | 201606	Arabidopsis_thaliana	OG0000013
AT1G64910.1 | UDP-Glycosyltransferase superfamily protein | Chr1:24115324-24116667 REVERSE LENGTH=447 | 201606	Arabidopsis_thaliana	OG0000013
AT1G64920.1 | UDP-Glycosyltransferase superfamily protein | Chr1:24117440-24118798 REVERSE LENGTH=452 | 201606	Arabidopsis_thaliana	OG0000013
AT1G73880.1 | UDP-glucosyl transferase 89B1 | Chr1:27785143-27786564 FORWARD LENGTH=473 | 201606	Arabidopsis_thaliana	OG0000013
AT1G07240.1 | UDP-glucosyl transferase 71C5 | Chr1:2223889-2225331 FORWARD LENGTH=480 | 201606	Arabidopsis_thaliana	OG0000013
AT1G07250.1 | UDP-glucosyl transferase 71C4 | Chr1:2225963-2227402 FORWARD LENGTH=479 | 201606	Arabidopsis_thaliana	OG0000013
AT1G07260.1 | UDP-glucosyl transferase 71C3 | Chr1:2227748-2229178 REVERSE LENGTH=476 | 201606	Arabidopsis_thaliana	OG0000013
AT1G78270.1 | UDP-glucosyl transferase 85A4 | Chr1:29450691-29452223 REVERSE LENGTH=489 | 201606	Arabidopsis_thaliana	OG0000013
AT2G15480.1 | UDP-glucosyl transferase 73B5 | Chr2:6758817-6760452 FORWARD LENGTH=484 | 201606	Arabidopsis_thaliana	OG0000013
AT2G15490.1 | UDP-glycosyltransferase 73B4 | Chr2:6761750-6763398 FORWARD LENGTH=484 | 201606	Arabidopsis_thaliana	OG0000013
AT2G16890.1 | UDP-Glycosyltransferase superfamily protein | Chr2:7316938-7317876 FORWARD LENGTH=312 | 201606

Arabidopsis_thaliana	OG0000013
AT2G18560.1 | UDP-Glycosyltransferase superfamily protein | Chr2:8059696-8060838 FORWARD LENGTH=380 | 201606	Arabidopsis_thaliana	OG0000013
AT2G18570.1 | UDP-Glycosyltransferase superfamily protein | Chr2:8063429-8064841 FORWARD LENGTH=470 | 201606	Arabidopsis_thaliana	OG0000013
AT2G22590.1 | UDP-Glycosyltransferase superfamily protein | Chr2:9593012-9594424 FORWARD LENGTH=470 | 201606	Arabidopsis_thaliana	OG0000013
AT2G22930.1 | UDP-Glycosyltransferase superfamily protein | Chr2:9759766-9761094 FORWARD LENGTH=442 | 201606	Arabidopsis_thaliana	OG0000013
AT2G23210.1 | UDP-Glycosyltransferase superfamily protein | Chr2:9882534-9883397 REVERSE LENGTH=287 | 201606	Arabidopsis_thaliana	OG0000013
AT2G23250.1 | UDP-glucosyl transferase 84B2 | Chr2:9897809-9899125 REVERSE LENGTH=438 | 201606	Arabidopsis_thaliana	OG0000013
AT2G23260.1 | UDP-glucosyl transferase 84B1 | Chr2:9900046-9901416 REVERSE LENGTH=456 | 201606	Arabidopsis_thaliana	OG0000013
AT2G26480.1 | UDP-glucosyl transferase 76D1 | Chr2:11263963-11265572 FORWARD LENGTH=452 | 201606	Arabidopsis_thaliana	OG0000013
AT2G28080.1 | UDP-Glycosyltransferase superfamily protein | Chr2:11960774-11963227 REVERSE LENGTH=482 | 201606	Arabidopsis_thaliana	OG0000013
AT2G29710.1 | UDP-Glycosyltransferase superfamily protein | Chr2:12698717-12700120 FORWARD LENGTH=467 | 201606	Arabidopsis_thaliana	OG0000013
AT2G29730.1 | UDP-glucosyl transferase 71D1 | Chr2:12703652-12705055 FORWARD LENGTH=467 | 201606	Arabidopsis_thaliana	OG0000013
AT2G29740.1 | UDP-glucosyl transferase 71C2 | Chr2:12706747-12708171 FORWARD LENGTH=474 | 201606	Arabidopsis_thaliana	OG0000013
AT2G29750.1 | UDP-glucosyl transferase 71C1 | Chr2:12709902-12711347 FORWARD LENGTH=481 | 201606	Arabidopsis_thaliana	OG0000013
AT2G30140.1 | UDP-Glycosyltransferase superfamily protein | Chr2:12872200-12873691 FORWARD LENGTH=455 | 201606	Arabidopsis_thaliana	OG0000013
AT2G30150.1 | UDP-Glycosyltransferase superfamily protein | Chr2:12874706-12876122 FORWARD LENGTH=440 | 201606	Arabidopsis_thaliana	OG0000013
AT2G31750.1 | UDP-glucosyl transferase 74D1 | Chr2:13497312-13499870 FORWARD LENGTH=456 | 201606	Arabidopsis_thaliana	OG0000013
AT2G31790.1 | UDP-Glycosyltransferase superfamily protein | Chr2:13518269-13520167 FORWARD LENGTH=457 | 201606	Arabidopsis_thaliana	OG0000013
Os03t0358800-00 Hypothetical conserved gene.	Oryza_sativa	OG0000013

Os03t0666600-01 UDP-glucuronosyl/UDP-glucosyltransferase family protein. Oryza_sativa OG0000013
Os03t0693600-00 Similar to indole-3-acetate beta-glucosyltransferase. Oryza_sativa OG0000013
Os03t0702000-01 UDP-glucuronosyl/UDP-glucosyltransferase family protein. Oryza_sativa OG0000013
Os03t0702500-01 UDP-glucuronosyl/UDP-glucosyltransferase family protein. Oryza_sativa OG0000013
Os03t0757100-00 UDP-glucuronosyl/UDP-glucosyltransferase family protein. Oryza_sativa OG0000013
Os03t0757200-01 UDP-glucuronosyl/UDP-glucosyltransferase family protein. Oryza_sativa OG0000013
Os03t0757500-01 UDP-glucuronosyl/UDP-glucosyltransferase family protein. Oryza_sativa OG0000013
Os03t0757600-01 UDP-glucuronosyl/UDP-glucosyltransferase family protein. Oryza_sativa OG0000013
Os03t0804900-00 UDP-glucuronosyl/UDP-glucosyltransferase family protein. Oryza_sativa OG0000013
Os03t0808200-00 UDP-glucuronosyl/UDP-glucosyltransferase family protein. Oryza_sativa OG0000013
Os03t0824600-00 Similar to cytokinin-N-glucosyltransferase 1. Oryza_sativa OG0000013
Os03t0841600-01 UDP-glucuronosyl/UDP-glucosyltransferase family protein. Oryza_sativa OG0000013
Os04t0197500-00 UDP-glucuronosyl/UDP-glucosyltransferase family protein. Oryza_sativa OG0000013
Os04t0203600-00 Similar to cDNA clone:002-100-D12, full insert sequence. Oryza_sativa OG0000013
Os04t0204000-00 Similar to H0825G02.2 protein. Oryza_sativa OG0000013
Os04t0204100-00 Similar to H0825G02.3 protein. Oryza_sativa OG0000013
Os04t0206000-00 Similar to H0825G02.4 protein. Oryza_sativa OG0000013
Os04t0206450-01 Similar to cDNA clone:002-100-D12, full insert sequence. Oryza_sativa OG0000013
Os04t0206500-01 UDP-glucuronosyl/UDP-glucosyltransferase family protein. Oryza_sativa OG0000013
Os04t0206600-01 UDP-glucuronosyl/UDP-glucosyltransferase family protein. Oryza_sativa OG0000013
Os04t0206700-01 UDP-glucuronosyl/UDP-glucosyltransferase family protein. Oryza_sativa OG0000013
Os04t0270900-00 Hypothetical conserved gene. Oryza_sativa OG0000013
Os04t0271700-01 UDP-glucuronosyl/UDP-glucosyltransferase family protein. Oryza_sativa OG0000013
Os04t0272200-01 UDP-glucuronosyl/UDP-glucosyltransferase family protein. Oryza_sativa OG0000013
Os04t0273600-00 Hypothetical conserved gene. Oryza_sativa OG0000013
Os04t0305700-01 Similar to OSIGBa0096F13.5 protein. Oryza_sativa OG0000013
Os04t0314100-00 Similar to H0107B07.2 protein. Oryza_sativa OG0000013

Os04t0319700-00 Similar to Glucosyltransferase (Fragment). Oryza_sativa OG0000013
Os04t0319800-01 Similar to Cytokinin-O-glucosyltransferase 2 (EC 2.4.1.-) (Zeatin O- glucosyltransferase 2). Oryza_sativa OG0000013
Os04t0320700-01 Similar to Glucosyltransferase (Fragment). Oryza_sativa OG0000013
Os04t0321100-00 Similar to H0107B07.6 protein. Oryza_sativa OG0000013
Os04t0324100-01 UDP-glucuronosyl/UDP-glucosyltransferase family protein. Oryza_sativa OG0000013
Os04t0326100-00 Similar to H0107B07.2 protein. Oryza_sativa OG0000013
Os04t0326201-00 Similar to H0107B07.1 protein. Oryza_sativa OG0000013
Os04t0451200-01 UDP-glucuronosyl/UDP-glucosyltransferase family protein. Oryza_sativa OG0000013
Os04t0506000-01 UDP-glucuronosyl/UDP-glucosyltransferase family protein. Oryza_sativa OG0000013
Os04t0523600-01 Similar to OSIGBa0153E02-OSIGBa0093I20.8 protein. Oryza_sativa OG0000013
Os04t0525100-01 UDP-glucuronosyl/UDP-glucosyltransferase family protein. Oryza_sativa OG0000013
Os04t0525200-01 UDP-glucuronosyl/UDP-glucosyltransferase family protein. Oryza_sativa OG0000013
Os04t0556400-01 Similar to UDP-glycosyltransferase UGT93B9. Oryza_sativa OG0000013
Os04t0556500-01 cis-Zeatin-O-glucosyltransferase, Catalyze O-glucosylation of cZ and cZ-riboside Oryza_sativa OG0000013
Os04t0556600-01 cis-Zeatin-O-glucosyltransferase, Catalyze O-glucosylation of cZ and cZ-riboside Oryza_sativa OG0000013
Os04t0565200-01 Similar to Cis-zeatin O-glucosyltransferase 1 (EC 2.4.1.215) (cisZOG1). Oryza_sativa OG0000013
Os04t0565400-01 cis-Zeatin-O-glucosyltransferase, Catalyze O-glucosylation of cZ and cZ-riboside Oryza_sativa OG0000013
Os05t0177500-01 UDP-glucuronosyl/UDP-glucosyltransferase family protein. Oryza_sativa OG0000013
Os05t0177800-00 Similar to UDP-glycosyltransferase UGT98B4. Oryza_sativa OG0000013
Os05t0179950-00 Similar to cDNA, clone Oryza_sativa OG0000013
Os05t0215300-01 UDP-glucuronosyl/UDP-glucosyltransferase family protein. Oryza_sativa OG0000013
Os05t0493600-00 Similar to indole-3-acetate beta-glucosyltransferase. Oryza_sativa OG0000013
Os05t0499600-01 UDP-glucuronosyl/UDP-glucosyltransferase family protein. Oryza_sativa OG0000013
Os05t0499800-01 UDP-glucuronosyl/UDP-glucosyltransferase family protein. Oryza_sativa OG0000013
Os05t0500000-00 UDP-glucuronosyl/UDP-glucosyltransferase family protein. Oryza_sativa OG0000013

Os05t0526800-01 UDP-glucuronosyl/UDP-glucosyltransferase family protein. Oryza_sativa OG0000013
Os05t0526900-01 UDP-glucuronosyl/UDP-glucosyltransferase family protein. Oryza_sativa OG0000013
Os05t0527000-01 UDP-glucuronosyl/UDP-glucosyltransferase family protein. Oryza_sativa OG0000013
Os05t0527100-00 UDP-glucuronosyl/UDP-glucosyltransferase family protein. Oryza_sativa OG0000013
Os05t0527600-00 Similar to anthocyanidin 5,3-O-glucosyltransferase. Oryza_sativa OG0000013
Os05t0527700-01 UDP-glucuronosyl/UDP-glucosyltransferase family protein. Oryza_sativa OG0000013
Os05t0527800-01 UDP-glucuronosyl/UDP-glucosyltransferase family protein. Oryza_sativa OG0000013
Os05t0527900-01 UDP-glucuronosyl/UDP-glucosyltransferase family protein. Oryza_sativa OG0000013
Os05t0552700-01 UDP-glucuronosyl/UDP-glucosyltransferase family protein. Oryza_sativa OG0000013
Os06t0137650-00 UDP-glucuronosyl/UDP-glucosyltransferase domain containing protein. Oryza_sativa OG0000013
Os06t0187500-00 Hypothetical conserved gene. Oryza_sativa OG0000013
Os06t0192100-00 Similar to UDP-glucose flavonoid-3-O-glucosyltransferase. Oryza_sativa OG0000013
Os06t0216100-00 Similar to UDP-glucoronosyl and UDP-glucosyl transferase family protein, expressed. Oryza_sativa OG0000013
Os06t0220500-01 UDP-glucuronosyl/UDP-glucosyltransferase family protein. Oryza_sativa OG0000013
Os06t0271000-00 UDP-glucuronosyl/UDP-glucosyltransferase domain containing protein. Oryza_sativa OG0000013
Os06t0282000-01 UDP-glucuronosyl/UDP-glucosyltransferase family protein. Oryza_sativa OG0000013
Os06t0282400-00 Similar to UDP-glycosyltransferase. Oryza_sativa OG0000013
Os06t0283100-00 Similar to UDP-glycosyltransferase. Oryza_sativa OG0000013
Os06t0288300-01 C-glucosyltransferase, Flavone-C-glycoside synthesis Oryza_sativa OG0000013
Os06t0289200-01 UDP-glucuronosyl/UDP-glucosyltransferase family protein. Oryza_sativa OG0000013
Os06t0289900-01 UDP-glucuronosyl/UDP-glucosyltransferase family protein. Oryza_sativa OG0000013
Os06t0343600-01 UDP-glucuronosyl/UDP-glucosyltransferase family protein. Oryza_sativa OG0000013
Os06t0590700-01 UDP-glucuronosyl/UDP-glucosyltransferase family protein. Oryza_sativa OG0000013
Os06t0590800-00 Hypothetical conserved gene. Oryza_sativa OG0000013
Os06t0593200-01 UDP-glucuronosyl/UDP-glucosyltransferase family protein. Oryza_sativa OG0000013
Os06t0593800-01 UDP-glucuronosyl/UDP-glucosyltransferase family protein. Oryza_sativa OG0000013
Os07t0148200-01 Similar to Flavonol 3-O-glucosyltransferase (EC

2.4.1.91) (UDP-glucose flavonoid 3-O-glucosyltransferase) (Bronze-1) (Bz-McC allele). Oryza_sativa OG0000013
Os07t0201300-00 UDP-glucuronosyl/UDP-glucosyltransferase domain containing protein. Oryza_sativa OG0000013
Os07t0201500-00 UDP-glucuronosyl/UDP-glucosyltransferase domain containing protein. Oryza_sativa OG0000013
Os07t0201800-00 UDP-glucuronosyl/UDP-glucosyltransferase domain containing protein. Oryza_sativa OG0000013
Os07t0202000-00 UDP-glucuronosyl/UDP-glucosyltransferase family protein. Oryza_sativa OG0000013
Os01t0597800-01 UDP-glucuronosyl/UDP-glucosyltransferase family protein. Oryza_sativa OG0000013
Os01t0598000-00 Similar to UDP-glycosyltransferase UGT703A5. Oryza_sativa OG0000013
Os07t0240600-01 UDP-glucuronosyl/UDP-glucosyltransferase family protein. Oryza_sativa OG0000013
Os07t0241500-01 UDP-glucuronosyl/UDP-glucosyltransferase family protein. Oryza_sativa OG0000013
Os07t0241600-00 UDP-glucuronosyl/UDP-glucosyltransferase domain containing protein. Oryza_sativa OG0000013
Os07t0241700-00 Similar to Cytokinin-N-glucosyltransferase 1. Oryza_sativa OG0000013
Os07t0241800-01 UDP-glucuronosyl/UDP-glucosyltransferase family protein. Oryza_sativa OG0000013
Os07t0250100-00 Similar to cytokinin-O-glucosyltransferase 2. Oryza_sativa OG0000013
Os07t0486700-00 Similar to cytokinin-O-glucosyltransferase 2. Oryza_sativa OG0000013
Os07t0489200-01 UDP-glucuronosyl/UDP-glucosyltransferase family protein. Oryza_sativa OG0000013
Os07t0489300-01 UDP-glucuronosyl/UDP-glucosyltransferase family protein. Oryza_sativa OG0000013
Os07t0489950-01 Similar to cytokinin-O-glucosyltransferase 2. Oryza_sativa OG0000013
Os07t0490100-01 UDP-glucuronosyl/UDP-glucosyltransferase family protein. Oryza_sativa OG0000013
Os07t0502900-00 UDP-glucuronosyl/UDP-glucosyltransferase family protein. Oryza_sativa OG0000013
Os07t0503300-01 UDP-glucuronosyl/UDP-glucosyltransferase family protein. Oryza_sativa OG0000013
Os07t0503500-01 UDP-glucuronosyl/UDP-glucosyltransferase family protein. Oryza_sativa OG0000013
Os07t0503900-01 UDP-glucuronosyl/UDP-glucosyltransferase family protein. Oryza_sativa OG0000013
Os07t0510400-01 UDP-glucuronosyl/UDP-glucosyltransferase family protein. Oryza_sativa OG0000013
Os07t0510500-01 UDP-glucuronosyl/UDP-glucosyltransferase family protein. Oryza_sativa OG0000013
Os07t0564100-01 UDP-glucuronosyl/UDP-glucosyltransferase family protein. Oryza_sativa OG0000013
Os01t0620800-01 UDP-glucuronosyl/UDP-glucosyltransferase family protein. Oryza_sativa OG0000013
Os07t0660500-00 Similar to Cis-zeatin O-glucosyltransferase 1 (EC 2.4.1.215) (cisZOG1). Oryza_sativa OG0000013

Os07t0672700-00 UDP-glucuronosyl/UDP-glucosyltransferase family protein. Oryza_sativa OG0000013
Os08t0168700-00 Similar to cytokinin-O-glucosyltransferase 2. Oryza_sativa OG0000013
Os01t0638000-01 UDP-glucuronosyl/UDP-glucosyltransferase family protein. Oryza_sativa OG0000013
Os08t0169400-00 Similar to cytokinin-O-glucosyltransferase 2. Oryza_sativa OG0000013
Os01t0638600-00 UDP-glucuronosyl/UDP-glucosyltransferase family protein. Oryza_sativa OG0000013
Os08t0404000-00 UDP-glucuronosyl/UDP-glucosyltransferase domain containing protein. Oryza_sativa OG0000013
Os08t0489100-00 Hypothetical conserved gene. Oryza_sativa OG0000013
Os09t0119600-01 UDP-glucuronosyl/UDP-glucosyltransferase family protein. Oryza_sativa OG0000013
Os09t0284800-00 Hypothetical conserved gene. Oryza_sativa OG0000013
Os09t0329200-00 UDP-glucuronosyl/UDP-glucosyltransferase family protein. Oryza_sativa OG0000013
Os09t0330000-00 UDP-glucuronosyl/UDP-glucosyltransferase family protein. Oryza_sativa OG0000013
Os09t0379400-00 Similar to OSIGBa0096F13.5 protein. Oryza_sativa OG0000013
Os01t0686200-01 UDP-glucuronosyl/UDP-glucosyltransferase family protein. Oryza_sativa OG0000013
Os01t0686300-01 UDP-glucuronosyl/UDP-glucosyltransferase family protein. Oryza_sativa OG0000013
Os09t0482860-01 Similar to cDNA clone:001-204-H11, full insert sequence. Oryza_sativa OG0000013
Os09t0482900-01 UDP-glucuronosyl/UDP-glucosyltransferase family protein. Oryza_sativa OG0000013
Os09t0517900-01 UDP-glucuronosyl/UDP-glucosyltransferase family protein. Oryza_sativa OG0000013
Os09t0518000-00 Similar to indole-3-acetate beta-glucosyltransferase. Oryza_sativa OG0000013
Os09t0518200-01 UDP-glucuronosyl/UDP-glucosyltransferase family protein. Oryza_sativa OG0000013
Os09t0518400-00 Similar to UDP-glycosyltransferase UGT74H5. Oryza_sativa OG0000013
Os01t0697100-01 UDP-glucuronosyl/UDP-glucosyltransferase family protein. Oryza_sativa OG0000013
Os10t0122000-00 UDP-glucuronosyl/UDP-glucosyltransferase family protein. Oryza_sativa OG0000013
Os10t0178500-01 UDP-glucuronosyl/UDP-glucosyltransferase family protein. Oryza_sativa OG0000013
Os10t0331600-00 UDP-glucuronosyl/UDP-glucosyltransferase domain containing protein. Oryza_sativa OG0000013
Os10t0332000-01 Arbuscular mycorrhizal specific marker 34. Oryza_sativa OG0000013
Os10t0332300-00 UDP-glucuronosyl/UDP-glucosyltransferase domain containing protein. Oryza_sativa OG0000013
Os10t0442400-01 Similar to UDP-glucoronosyl and UDP-glucosyl transferase family protein, expressed. Oryza_sativa

OG0000013
Os11t0145200-01 UDP-glucuronosyl/UDP-glucosyltransferase family protein. Oryza_sativa OG0000013
Os01t0734600-01 UDP-glucuronosyl/UDP-glucosyltransferase family protein. Oryza_sativa OG0000013
Os01t0734800-01 UDP-glucuronosyl/UDP-glucosyltransferase family protein. Oryza_sativa OG0000013
Os11t0202500-00 UDP-glucuronosyl/UDP-glucosyltransferase domain containing protein. Oryza_sativa OG0000013
Os01t0735300-01 UDP-glucuronosyl/UDP-glucosyltransferase family protein. Oryza_sativa OG0000013
Os01t0735900-01 UDP-glucuronosyl/UDP-glucosyltransferase family protein. Oryza_sativa OG0000013
Os01t0736100-01 UDP-glucuronosyl/UDP-glucosyltransferase family protein. Oryza_sativa OG0000013
Os01t0736300-01 Similar to anthocyanidin 5,3-O-glucosyltransferase. Oryza_sativa OG0000013
Os11t0441500-01 UDP-glucuronosyl/UDP-glucosyltransferase family protein. Oryza_sativa OG0000013
Os11t0444000-01 Similar to UDP-glucosyltransferase BX8. Oryza_sativa OG0000013
Os11t0457300-01 UDP-glucuronosyl/UDP-glucosyltransferase family protein. Oryza_sativa OG0000013
Os11t0461200-01 UDP-glucuronosyl/UDP-glucosyltransferase domain containing protein. Oryza_sativa OG0000013
Os11t0599200-01 UDP-glucuronosyl/UDP-glucosyltransferase family protein. Oryza_sativa OG0000013
Os12t0561900-01 Similar to UDP-glucoronosyl and UDP-glucosyl transferase family protein, expressed. Oryza_sativa OG0000013
Os01t0805400-01 UDP-glucuronosyl/UDP-glucosyltransferase family protein. Oryza_sativa OG0000013
Os01t0805500-00 Similar to indole-3-acetate beta-glucosyltransferase. Oryza_sativa OG0000013
Os01t0865400-01 UDP-glucuronosyl/UDP-glucosyltransferase family protein. Oryza_sativa OG0000013
Os01t0869400-01 Similar to Anthocyanidin 5,3-O-glucosyltransferase. Oryza_sativa OG0000013
Os02t0188000-01 UDP-glucuronosyl/UDP-glucosyltransferase family protein. Oryza_sativa OG0000013
Os02t0203300-00 Similar to UDP-glycosyltransferase UGT75E3. Oryza_sativa OG0000013
Os02t0206100-01 UDP-glucuronosyl/UDP-glucosyltransferase family protein. Oryza_sativa OG0000013
Os02t0206700-01 UDP-glucuronosyl/UDP-glucosyltransferase family protein. Oryza_sativa OG0000013
Os02t0207100-01 Similar to UDP-glycosyltransferase UGT99C4. Oryza_sativa OG0000013
Os02t0207400-00 Similar to UDP-glycosyltransferase UGT99C4. Oryza_sativa OG0000013
Os01t0176000-01 UDP-glucuronosyl/UDP-glucosyltransferase family protein. Oryza_sativa OG0000013
Os01t0176100-00 UDP-glucuronosyl/UDP-glucosyltransferase family protein. Oryza_sativa OG0000013

Os02t0242100-01 Similar to hydroquinone glucosyltransferase.
Oryza_sativa OG0000013
Os01t0176200-01 UDP-glucuronosyl/UDP-glucosyltransferase family protein. Oryza_sativa OG0000013
Os02t0242550-00 Similar to hydroquinone glucosyltransferase.
Oryza_sativa OG0000013
Os02t0242900-00 Similar to hydroquinone glucosyltransferase.
Oryza_sativa OG0000013
Os02t0243300-01 UDP-glucuronosyl/UDP-glucosyltransferase family protein. Oryza_sativa OG0000013
Os01t0179600-01 UDP-glucuronosyl/UDP-glucosyltransferase family protein. Oryza_sativa OG0000013
Os02t0329933-00 Similar to UDP-glycosyltransferase UGT99C4.
Oryza_sativa OG0000013
Os02t0490500-01 UDP-glucuronosyl/UDP-glucosyltransferase family protein. Oryza_sativa OG0000013
Os02t0577700-00 Similar to H0815C01.4 protein. Oryza_sativa OG0000013
Os02t0578300-01 Similar to H0815C01.4 protein. Oryza_sativa OG0000013
Os02t0589400-01 UDP-glucuronosyl/UDP-glucosyltransferase family protein. Oryza_sativa OG0000013
Os02t0634100-00 Similar to OSIGBa0153E02-OSIGBa0093I20.18 protein.
Oryza_sativa OG0000013
Os02t0755500-01 Similar to UDP-glycosyltransferase 85A8.
Oryza_sativa OG0000013
Os02t0755600-01 UDP-glucuronosyl/UDP-glucosyltransferase domain containing protein. Oryza_sativa OG0000013
Os02t0755900-01 UDP-glucuronosyl/UDP-glucosyltransferase domain containing protein. Oryza_sativa OG0000013
Os02t0803900-01 UDP-glucuronosyl/UDP-glucosyltransferase domain containing protein. Oryza_sativa OG0000013
Os03t0212000-00 Hypothetical conserved gene. Oryza_sativa OG0000013
evm.model.contig_2179.7 Porphyridium_purpureum OG0000013
g111.t1 Caulerpa_lentillifera OG0000014
g1394.t1 Caulerpa_lentillifera OG0000014
g1575.t1 Caulerpa_lentillifera OG0000014
g2591.t1 Caulerpa_lentillifera OG0000014
g3276.t1 Caulerpa_lentillifera OG0000014
g3811.t1 Caulerpa_lentillifera OG0000014
g3938.t1 Caulerpa_lentillifera OG0000014
g4257.t1 Caulerpa_lentillifera OG0000014
g4783.t1 Caulerpa_lentillifera OG0000014
g5160.t1 Caulerpa_lentillifera OG0000014
g6244.t1 Caulerpa_lentillifera OG0000014
g6842.t1 Caulerpa_lentillifera OG0000014
g6898.t1 Caulerpa_lentillifera OG0000014
g6899.t1 Caulerpa_lentillifera OG0000014
g6915.t1 Caulerpa_lentillifera OG0000014
g7276.t1 Caulerpa_lentillifera OG0000014
g7284.t1 Caulerpa_lentillifera OG0000014
g7964.t1 Caulerpa_lentillifera OG0000014
g7966.t1 Caulerpa_lentillifera OG0000014

g8332.t1 Caulerpa_lentillifera OG0000014
g8581.t1 Caulerpa_lentillifera OG0000014
g8723.t1 Caulerpa_lentillifera OG0000014
g8724.t1 Caulerpa_lentillifera OG0000014
g9018.t1 Caulerpa_lentillifera OG0000014
g9131.t1 Caulerpa_lentillifera OG0000014
g9160.t1 Caulerpa_lentillifera OG0000014
g9219.t1 Caulerpa_lentillifera OG0000014
g970.t1 Caulerpa_lentillifera OG0000014
Cre08.g358541.t1.1 pacid=30773487 transcript=Cre08.g358541.t1.1 locus=Cre08.g358541 ID=Cre08.g358541.t1.1.v5.5 annot-version=v5.5 Chlamydomonas_reinhardtii OG0000014
Cre03.g208300.t1.2 pacid=30787012 transcript=Cre03.g208300.t1.2 locus=Cre03.g208300 ID=Cre03.g208300.t1.2.v5.5 annot-version=v5.5 Chlamydomonas_reinhardtii OG0000014
Cre03.g208557.t1.1 pacid=30787410 transcript=Cre03.g208557.t1.1 locus=Cre03.g208557 ID=Cre03.g208557.t1.1.v5.5 annot-version=v5.5 Chlamydomonas_reinhardtii OG0000014
Cre01.g040750.t1.1 pacid=30788377 transcript=Cre01.g040750.t1.1 locus=Cre01.g040750 ID=Cre01.g040750.t1.1.v5.5 annot-version=v5.5 Chlamydomonas_reinhardtii OG0000014
Cre01.g006250.t1.2 pacid=30789206 transcript=Cre01.g006250.t1.2 locus=Cre01.g006250 ID=Cre01.g006250.t1.2.v5.5 annot-version=v5.5 Chlamydomonas_reinhardtii OG0000014
Cre10.g423050.t1.2 pacid=30790165 transcript=Cre10.g423050.t1.2 locus=Cre10.g423050 ID=Cre10.g423050.t1.2.v5.5 annot-version=v5.5 Chlamydomonas_reinhardtii OG0000014
Cre10.g446600.t1.2 pacid=30790904 transcript=Cre10.g446600.t1.2 locus=Cre10.g446600 ID=Cre10.g446600.t1.2.v5.5 annot-version=v5.5 Chlamydomonas_reinhardtii OG0000014
Cre12.g545650.t1.2 pacid=30793187 transcript=Cre12.g545650.t1.2 locus=Cre12.g545650 ID=Cre12.g545650.t1.2.v5.5 annot-version=v5.5 Chlamydomonas_reinhardtii OG0000014
Cre11.g467733.t1.1 pacid=30775863 transcript=Cre11.g467733.t1.1 locus=Cre11.g467733 ID=Cre11.g467733.t1.1.v5.5 annot-version=v5.5 Chlamydomonas_reinhardtii OG0000014
Cre08.g358542.t1.1 pacid=30773661 transcript=Cre08.g358542.t1.1 locus=Cre08.g358542 ID=Cre08.g358542.t1.1.v5.5 annot-version=v5.5 Chlamydomonas_reinhardtii OG0000014
Cre08.g358543.t1.1 pacid=30773751 transcript=Cre08.g358543.t1.1 locus=Cre08.g358543 ID=Cre08.g358543.t1.1.v5.5 annot-version=v5.5 Chlamydomonas_reinhardtii OG0000014
Cre34.g759147.t1.1 pacid=30778361 transcript=Cre34.g759147.t1.1 locus=Cre34.g759147 ID=Cre34.g759147.t1.1.v5.5 annot-version=v5.5 Chlamydomonas_reinhardtii OG0000014
Cre08.g380151.t1.1 pacid=30774050 transcript=Cre08.g380151.t1.1 locus=Cre08.g380151 ID=Cre08.g380151.t1.1.v5.5 annot-version=v5.5 Chlamydomonas_reinhardtii OG0000014
Cre09.g404503.t1.1 pacid=30780699 transcript=Cre09.g404503.t1.1 locus=Cre09.g404503 ID=Cre09.g404503.t1.1.v5.5 annot-version=v5.5 Chlamydomonas_reinhardtii OG0000014
Cre09.g398141.t1.1 pacid=30781038 transcript=Cre09.g398141.t1.1 locus=Cre09.g398141 ID=Cre09.g398141.t1.1.v5.5 annot-version=v5.5 Chlamydomonas_reinhardtii OG0000014

Cre30.g758097.t1.1 pacid=30781567 transcript=Cre30.g758097.t1.1
locus=Cre30.g758097 ID=Cre30.g758097.t1.1.v5.5 annot-version=v5.5
Chlamydomonas_reinhardtii OG0000014
Cre17.g733425.t1.1 pacid=30782157 transcript=Cre17.g733425.t1.1
locus=Cre17.g733425 ID=Cre17.g733425.t1.1.v5.5 annot-version=v5.5
Chlamydomonas_reinhardtii OG0000014
Cre17.g733450.t1.2 pacid=30782410 transcript=Cre17.g733450.t1.2
locus=Cre17.g733450 ID=Cre17.g733450.t1.2.v5.5 annot-version=v5.5
Chlamydomonas_reinhardtii OG0000014
Cre05.g235400.t1.2 pacid=30783481 transcript=Cre05.g235400.t1.2
locus=Cre05.g235400 ID=Cre05.g235400.t1.2.v5.5 annot-version=v5.5
Chlamydomonas_reinhardtii OG0000014
Cre13.g592100.t1.1 pacid=30783857 transcript=Cre13.g592100.t1.1
locus=Cre13.g592100 ID=Cre13.g592100.t1.1.v5.5 annot-version=v5.5
Chlamydomonas_reinhardtii OG0000014
Vocar.0012s0208.1.p pacid=32894868 transcript=Vocar.0012s0208.1
locus=Vocar.0012s0208 ID=Vocar.0012s0208.1.v2.1 annot-version=v2.1
Volvox_carteri OG0000014
Vocar.0064s0026.1.p pacid=32897722 transcript=Vocar.0064s0026.1
locus=Vocar.0064s0026 ID=Vocar.0064s0026.1.v2.1 annot-version=v2.1
Volvox_carteri OG0000014
Vocar.0029s0050.1.p pacid=32887364 transcript=Vocar.0029s0050.1
locus=Vocar.0029s0050 ID=Vocar.0029s0050.1.v2.1 annot-version=v2.1
Volvox_carteri OG0000014
Vocar.0042s0028.1.p pacid=32892021 transcript=Vocar.0042s0028.1
locus=Vocar.0042s0028 ID=Vocar.0042s0028.1.v2.1 annot-version=v2.1
Volvox_carteri OG0000014
Vocar.0026s0045.1.p pacid=32892414 transcript=Vocar.0026s0045.1
locus=Vocar.0026s0045 ID=Vocar.0026s0045.1.v2.1 annot-version=v2.1
Volvox_carteri OG0000014
jgi|ChlNC64A_1|133052|IGS.gm_1_00188 Chlorella_variabilis
OG0000014
jgi|ChlNC64A_1|133835|IGS.gm_10_00126 Chlorella_variabilis
OG0000014
jgi|ChlNC64A_1|20200|e_gw1.4.490.1 Chlorella_variabilis
OG0000014
55605 pacid=27389624 transcript=55605 locus=Genemark1.549_g
ID=55605.2.0.227 annot-version=v2.0 Coccomyxa_subellipsoidea
OG0000014
39509 pacid=27393330 transcript=39509 locus=fgenesh1_pg.2_#_121
ID=39509.2.0.227 annot-version=v2.0 Coccomyxa_subellipsoidea
OG0000014
31084_0200010415 Ostreococcus_tauri OG0000014
36895_1500010191 Ostreococcus_tauri OG0000014
18155 pacid=27419886 transcript=18155
locus=fgenesh1_pg.C_Chr_14000201 ID=18155.2.0.231 annot-version=v2.0
Ostreococcus_lucimarinus OG0000014
19463 pacid=27338656 transcript=19463 locus=MicpuC2.e_gw1.8.537.1
ID=19463.3.0.228 annot-version=v3.0 Micromonas_pusilla
OG0000014
55624 pacid=27342595 transcript=55624 locus=MicpuC2.EuGene.
0000020653 ID=55624.3.0.228 annot-version=v3.0
Micromonas_pusilla OG0000014
39051 pacid=27347047 transcript=39051

locus=MicpuC2.fgenesh1_pg.C_scaffold_3000558 ID=39051.3.0.228 annot-version=v3.0   Micromonas_pusilla   OG0000014
62154 pacid=27397500 transcript=62154 locus=EuGene.1000010491 ID=62154.3.0.229 annot-version=v3.0   Micromonas_sp._RCC299 OG0000014
113407 pacid=27395982 transcript=113407 locus=AZW_EuGene.0500010059 ID=113407.3.0.229 annot-version=v3.0   Micromonas_sp._RCC299 OG0000014
109680 pacid=27402979 transcript=109680 locus=est_cluster_kg.Chr_17__19__3200414:2 ID=109680.3.0.229 annot-version=v3.0   Micromonas_sp._RCC299   OG0000014
64962 pacid=27402985 transcript=64962 locus=EuGene.1700010023 ID=64962.3.0.229 annot-version=v3.0   Micromonas_sp._RCC299 OG0000014
kfl00335_g1_v1.1 Klebsormidium_flaccidum OG0000014
kfl00336_0010_v1.1   Klebsormidium_flaccidum OG0000014
kfl00015_0180_v1.1   Klebsormidium_flaccidum OG0000014
kfl00377_g1_v1.1 Klebsormidium_flaccidum OG0000014
kfl00391_0010_v1.1   Klebsormidium_flaccidum OG0000014
kfl00391_0050_v1.1   Klebsormidium_flaccidum OG0000014
kfl00391_0055_v1.1   Klebsormidium_flaccidum OG0000014
kfl00018_0010_v1.1   Klebsormidium_flaccidum OG0000014
kfl00018_0020_v1.1   Klebsormidium_flaccidum OG0000014
kfl00018_0030_v1.1   Klebsormidium_flaccidum OG0000014
kfl00500_0010_v1.1   Klebsormidium_flaccidum OG0000014
kfl00514_0110_v1.1   Klebsormidium_flaccidum OG0000014
kfl00525_0040_v1.1   Klebsormidium_flaccidum OG0000014
kfl00569_0010_v1.1   Klebsormidium_flaccidum OG0000014
kfl00569_0020_v1.1   Klebsormidium_flaccidum OG0000014
kfl00569_0100_v1.1   Klebsormidium_flaccidum OG0000014
kfl00569_g16_v1.1   Klebsormidium_flaccidum OG0000014
kfl00578_0060_v1.1   Klebsormidium_flaccidum OG0000014
kfl00578_0070_v1.1   Klebsormidium_flaccidum OG0000014
kfl00581_0010_v1.1   Klebsormidium_flaccidum OG0000014
kfl00590_0100_v1.1   Klebsormidium_flaccidum OG0000014
kfl00644_0060_v1.1   Klebsormidium_flaccidum OG0000014
kfl00732_0070_v1.1   Klebsormidium_flaccidum OG0000014
kfl00747_0020_v1.1   Klebsormidium_flaccidum OG0000014
kfl00747_0065_v1.1   Klebsormidium_flaccidum OG0000014
kfl00754_0020_v1.1   Klebsormidium_flaccidum OG0000014
kfl00754_0030_v1.1   Klebsormidium_flaccidum OG0000014
kfl00754_0035_v1.1   Klebsormidium_flaccidum OG0000014
kfl00021_0380_v1.1   Klebsormidium_flaccidum OG0000014
kfl00821_g9_v1.1 Klebsormidium_flaccidum OG0000014
kfl00841_0060_v1.1   Klebsormidium_flaccidum OG0000014
kfl00847_0010_v1.1   Klebsormidium_flaccidum OG0000014
kfl00853_0030_v1.1   Klebsormidium_flaccidum OG0000014
kfl00872_0010_v1.1   Klebsormidium_flaccidum OG0000014
kfl00872_0020_v1.1   Klebsormidium_flaccidum OG0000014
kfl00893_0010_v1.1   Klebsormidium_flaccidum OG0000014
kfl00900_g2_v1.1 Klebsormidium_flaccidum OG0000014
kfl00903_0040_v1.1   Klebsormidium_flaccidum OG0000014
kfl00918_0010_v1.1   Klebsormidium_flaccidum OG0000014
kfl00919_0030_v1.1   Klebsormidium_flaccidum OG0000014

kfl00931_0040_v1.1	Klebsormidium_flaccidum	OG0000014
kfl00938_0040_v1.1	Klebsormidium_flaccidum	OG0000014
kfl00980_0040_v1.1	Klebsormidium_flaccidum	OG0000014
kfl01010_0030_v1.1	Klebsormidium_flaccidum	OG0000014
kfl01011_0070_v1.1	Klebsormidium_flaccidum	OG0000014
kfl01025_0010_v1.1	Klebsormidium_flaccidum	OG0000014
kfl01025_0020_v1.1	Klebsormidium_flaccidum	OG0000014
kfl01047_0010_v1.1	Klebsormidium_flaccidum	OG0000014
kfl01057_0010_v1.1	Klebsormidium_flaccidum	OG0000014
kfl01058_0010_v1.1	Klebsormidium_flaccidum	OG0000014
kfl01062_0010_v1.1	Klebsormidium_flaccidum	OG0000014
kfl01067_g1_v1.1	Klebsormidium_flaccidum	OG0000014
kfl01113_0010_v1.1	Klebsormidium_flaccidum	OG0000014
kfl01118_0010_v1.1	Klebsormidium_flaccidum	OG0000014
kfl01118_0020_v1.1	Klebsormidium_flaccidum	OG0000014
kfl01145_0010_v1.1	Klebsormidium_flaccidum	OG0000014
kfl01147_0010_v1.1	Klebsormidium_flaccidum	OG0000014
kfl01242_0020_v1.1	Klebsormidium_flaccidum	OG0000014
kfl01267_0020_v1.1	Klebsormidium_flaccidum	OG0000014
kfl01267_0030_v1.1	Klebsormidium_flaccidum	OG0000014
kfl01267_0040_v1.1	Klebsormidium_flaccidum	OG0000014
kfl01273_0010_v1.1	Klebsormidium_flaccidum	OG0000014
kfl01297_0010_v1.1	Klebsormidium_flaccidum	OG0000014
kfl01297_0015_v1.1	Klebsormidium_flaccidum	OG0000014
kfl01332_0010_v1.1	Klebsormidium_flaccidum	OG0000014
kfl01378_0010_v1.1	Klebsormidium_flaccidum	OG0000014
kfl01384_0030_v1.1	Klebsormidium_flaccidum	OG0000014
kfl01422_0020_v1.1	Klebsormidium_flaccidum	OG0000014
kfl01424_0010_v1.1	Klebsormidium_flaccidum	OG0000014
kfl01445_0010_v1.1	Klebsormidium_flaccidum	OG0000014
kfl01478_g1_v1.1	Klebsormidium_flaccidum	OG0000014
kfl01531_0010_v1.1	Klebsormidium_flaccidum	OG0000014
kfl01537_0010_v1.1	Klebsormidium_flaccidum	OG0000014
kfl01537_0020_v1.1	Klebsormidium_flaccidum	OG0000014
kfl01545_0020_v1.1	Klebsormidium_flaccidum	OG0000014
kfl01557_0010_v1.1	Klebsormidium_flaccidum	OG0000014
kfl01557_0020_v1.1	Klebsormidium_flaccidum	OG0000014
kfl01577_0010_v1.1	Klebsormidium_flaccidum	OG0000014
kfl01596_0010_v1.1	Klebsormidium_flaccidum	OG0000014
kfl01638_0010_v1.1	Klebsormidium_flaccidum	OG0000014
kfl01707_0010_v1.1	Klebsormidium_flaccidum	OG0000014
kfl01707_0020_v1.1	Klebsormidium_flaccidum	OG0000014
kfl01799_0010_v1.1	Klebsormidium_flaccidum	OG0000014
kfl00028_0010_v1.1	Klebsormidium_flaccidum	OG0000014
kfl00054_0030_v1.1	Klebsormidium_flaccidum	OG0000014
kfl00004_0570_v1.1	Klebsormidium_flaccidum	OG0000014
kfl00064_0330_v1.1	Klebsormidium_flaccidum	OG0000014
kfl00064_0340_v1.1	Klebsormidium_flaccidum	OG0000014
kfl00067_0400_v1.1	Klebsormidium_flaccidum	OG0000014
kfl00111_0330_v1.1	Klebsormidium_flaccidum	OG0000014
kfl00142_0210_v1.1	Klebsormidium_flaccidum	OG0000014
kfl00142_0215_v1.1	Klebsormidium_flaccidum	OG0000014
kfl00147_0280_v1.1	Klebsormidium_flaccidum	OG0000014
kfl00169_0230_v1.1	Klebsormidium_flaccidum	OG0000014

kfl00193_0205_v1.1	Klebsormidium_flaccidum	OG0000014
kfl00271_0010_v1.1	Klebsormidium_flaccidum	OG0000014
Pp3c2_850V3.1.p pacid=32934234 transcript=Pp3c2_850V3.1 locus=Pp3c2_850 annot-version=v3.3	Physcomitrella_patens	OG0000014
Pp3c22_9190V3.1.p pacid=32902997 transcript=Pp3c22_9190V3.1 locus=Pp3c22_9190 annot-version=v3.3	Physcomitrella_patens	OG0000014
Pp3c2_840V3.1.p pacid=32935899 transcript=Pp3c2_840V3.1 locus=Pp3c2_840 annot-version=v3.3	Physcomitrella_patens	OG0000014
Pp3c2_2341V3.1.p pacid=32936732 transcript=Pp3c2_2341V3.1 locus=Pp3c2_2341 annot-version=v3.3	Physcomitrella_patens	OG0000014
Pp3c2_37790V3.1.p pacid=32936892 transcript=Pp3c2_37790V3.1 locus=Pp3c2_37790 annot-version=v3.3	Physcomitrella_patens	OG0000014
Pp3c19_23190V3.1.p pacid=32938213 transcript=Pp3c19_23190V3.1 locus=Pp3c19_23190 annot-version=v3.3	Physcomitrella_patens	OG0000014
Pp3c22_8770V3.1.p pacid=32903433 transcript=Pp3c22_8770V3.1 locus=Pp3c22_8770 annot-version=v3.3	Physcomitrella_patens	OG0000014
Pp3c19_220V3.1.p pacid=32939599 transcript=Pp3c19_220V3.1 locus=Pp3c19_220 annot-version=v3.3	Physcomitrella_patens	OG0000014
Pp3c3_35120V3.1.p pacid=32944366 transcript=Pp3c3_35120V3.1 locus=Pp3c3_35120 annot-version=v3.3	Physcomitrella_patens	OG0000014
Pp3c5_12670V3.1.p pacid=32952596 transcript=Pp3c5_12670V3.1 locus=Pp3c5_12670 annot-version=v3.3	Physcomitrella_patens	OG0000014
Pp3c5_17600V3.1.p pacid=32952644 transcript=Pp3c5_17600V3.1 locus=Pp3c5_17600 annot-version=v3.3	Physcomitrella_patens	OG0000014
Pp3c5_12720V3.1.p pacid=32953208 transcript=Pp3c5_12720V3.1 locus=Pp3c5_12720 annot-version=v3.3	Physcomitrella_patens	OG0000014
Pp3c11_8640V3.1.p pacid=32958973 transcript=Pp3c11_8640V3.1 locus=Pp3c11_8640 annot-version=v3.3	Physcomitrella_patens	OG0000014
Pp3c14_25520V3.1.p pacid=32960593 transcript=Pp3c14_25520V3.1 locus=Pp3c14_25520 annot-version=v3.3	Physcomitrella_patens	OG0000014
Pp3c14_25535V3.1.p pacid=32960983 transcript=Pp3c14_25535V3.1 locus=Pp3c14_25535 annot-version=v3.3	Physcomitrella_patens	OG0000014
Pp3c14_8060V3.1.p pacid=32961868 transcript=Pp3c14_8060V3.1 locus=Pp3c14_8060 annot-version=v3.3	Physcomitrella_patens	OG0000014
Pp3c14_25400V3.1.p pacid=32961876 transcript=Pp3c14_25400V3.1 locus=Pp3c14_25400 annot-version=v3.3	Physcomitrella_patens	OG0000014
Pp3c17_2370V3.1.p pacid=32905782 transcript=Pp3c17_2370V3.1

locus=Pp3c17_2370 annot-version=v3.3 Physcomitrella_patens OG0000014
Pp3c14_8050V3.1.p pacid=32963259 transcript=Pp3c14_8050V3.1 locus=Pp3c14_8050 annot-version=v3.3 Physcomitrella_patens OG0000014
Pp3c1_28163V3.1.p pacid=32966664 transcript=Pp3c1_28163V3.1 locus=Pp3c1_28163 annot-version=v3.3 Physcomitrella_patens OG0000014
Pp3c1_24295V3.1.p pacid=32970047 transcript=Pp3c1_24295V3.1 locus=Pp3c1_24295 annot-version=v3.3 Physcomitrella_patens OG0000014
Pp3c1_28191V3.1.p pacid=32970463 transcript=Pp3c1_28191V3.1 locus=Pp3c1_28191 annot-version=v3.3 Physcomitrella_patens OG0000014
Pp3c1_8100V3.1.p pacid=32971506 transcript=Pp3c1_8100V3.1 locus=Pp3c1_8100 annot-version=v3.3 Physcomitrella_patens OG0000014
Pp3c12_25480V3.1.p pacid=32972487 transcript=Pp3c12_25480V3.1 locus=Pp3c12_25480 annot-version=v3.3 Physcomitrella_patens OG0000014
Pp3c12_5040V3.1.p pacid=32974716 transcript=Pp3c12_5040V3.1 locus=Pp3c12_5040 annot-version=v3.3 Physcomitrella_patens OG0000014
Pp3c17_12480V3.1.p pacid=32907214 transcript=Pp3c17_12480V3.1 locus=Pp3c17_12480 annot-version=v3.3 Physcomitrella_patens OG0000014
Pp3c6_25990V3.1.p pacid=32976871 transcript=Pp3c6_25990V3.1 locus=Pp3c6_25990 annot-version=v3.3 Physcomitrella_patens OG0000014
Pp3c24_5670V3.1.p pacid=32909163 transcript=Pp3c24_5670V3.1 locus=Pp3c24_5670 annot-version=v3.3 Physcomitrella_patens OG0000014
Pp3c21_20460V3.1.p pacid=32915484 transcript=Pp3c21_20460V3.1 locus=Pp3c21_20460 annot-version=v3.3 Physcomitrella_patens OG0000014
Pp3c21_9680V3.1.p pacid=32916175 transcript=Pp3c21_9680V3.1 locus=Pp3c21_9680 annot-version=v3.3 Physcomitrella_patens OG0000014
Pp3c4_22130V3.1.p pacid=32919026 transcript=Pp3c4_22130V3.1 locus=Pp3c4_22130 annot-version=v3.3 Physcomitrella_patens OG0000014
Pp3c7_15210V3.1.p pacid=32923841 transcript=Pp3c7_15210V3.1 locus=Pp3c7_15210 annot-version=v3.3 Physcomitrella_patens OG0000014
AT3G05670.1 | RING/U-box protein | Chr3:1653888-1657022 FORWARD LENGTH=883 | 201606 Arabidopsis_thaliana OG0000014
AT1G43770.1 | RING/FYVE/PHD zinc finger superfamily protein | Chr1:16548509-16549907 FORWARD LENGTH=371 | 201606 Arabidopsis_thaliana OG0000014
Os03t0300700-01 Retrotransposon gag protein family protein. Oryza_sativa OG0000014
Os03t0302200-01 Zinc finger, RING/FYVE/PHD-type domain containing protein. Oryza_sativa OG0000014
Os03t0421000-01 Zinc finger, FYVE/PHD-type domain containing

protein. Oryza_sativa OG0000014
Os03t0627900-01 Similar to H0102C09.1 protein. Oryza_sativa OG0000014
Os04t0191000-01 Similar to OSIGBa0114M03.4 protein. Oryza_sativa OG0000014
Os04t0273100-01 Similar to Transposase. Oryza_sativa OG0000014
Os04t0603800-01 Chromo domain containing protein. Oryza_sativa OG0000014
Os04t0682600-01 Similar to H0502G05.7 protein. Oryza_sativa OG0000014
Os06t0170800-01 Similar to H0124B04.16 protein. Oryza_sativa OG0000014
Os06t0283200-01 Zinc finger, RING/FYVE/PHD-type domain containing protein. Oryza_sativa OG0000014
Os06t0497600-01 Similar to H0321H01.8 protein. Oryza_sativa OG0000014
Os07t0690300-01 Zinc finger, RING/FYVE/PHD-type domain containing protein. Oryza_sativa OG0000014
Os01t0640100-00 Conserved hypothetical protein. Oryza_sativa OG0000014
Os08t0210300-01 Similar to H0502G05.7 protein. Oryza_sativa OG0000014
Os08t0273000-01 Conserved hypothetical protein. Oryza_sativa OG0000014
Os09t0375700-01 Similar to H0616A11.2 protein. Oryza_sativa OG0000014
Os12t0121200-01 Conserved hypothetical protein. Oryza_sativa OG0000014
Os12t0142900-02 Similar to H0321H01.8 protein. Oryza_sativa OG0000014
Os02t0522100-02 Similar to cDNA clone:J013126H19, full insert sequence. Oryza_sativa OG0000014
Os02t0522300-01 Conserved hypothetical protein. Oryza_sativa OG0000014
evm.model.contig_2162.4 Porphyridium_purpureum OG0000014
evm.model.contig_2206.2 Porphyridium_purpureum OG0000014
evm.model.contig_2215.2 Porphyridium_purpureum OG0000014
evm.model.contig_2218.2 Porphyridium_purpureum OG0000014
evm.model.contig_2498.1 Porphyridium_purpureum OG0000014
evm.model.contig_2509.3 Porphyridium_purpureum OG0000014
evm.model.contig_3413.7 Porphyridium_purpureum OG0000014
evm.model.contig_3424.1 Porphyridium_purpureum OG0000014
evm.model.contig_3474.1 Porphyridium_purpureum OG0000014
evm.model.contig_3493.3 Porphyridium_purpureum OG0000014
evm.model.contig_3493.1 Porphyridium_purpureum OG0000014
evm.model.contig_3493.2 Porphyridium_purpureum OG0000014
evm.model.contig_3536.3 Porphyridium_purpureum OG0000014
evm.model.contig_3536.2 Porphyridium_purpureum OG0000014
evm.model.contig_3694.2 Porphyridium_purpureum OG0000014
evm.model.contig_3709.2 Porphyridium_purpureum OG0000014
evm.model.contig_3758.2 Porphyridium_purpureum OG0000014
evm.model.contig_3758.3 Porphyridium_purpureum OG0000014
evm.model.contig_3759.1 Porphyridium_purpureum OG0000014

evm.model.contig_2049.9 Porphyridium_purpureum   OG0000014
evm.model.contig_4518.3 Porphyridium_purpureum   OG0000014
evm.model.contig_4518.2 Porphyridium_purpureum   OG0000014
evm.model.contig_4577.2 Porphyridium_purpureum   OG0000014
CDF32213 pep supercontig:ASM35022v2:HG001460:95292:96608:1 gene:CHC_T00007593001 transcript:CDF32213 gene_biotype:protein_coding transcript_biotype:protein_coding Chondrus_crispus OG0000014
CDF33000 pep supercontig:ASM35022v2:HG001629:88451:90834:1 gene:CHC_T00001764001 transcript:CDF33000 gene_biotype:protein_coding transcript_biotype:protein_coding Chondrus_crispus OG0000014
CDF32406 pep supercontig:ASM35022v2:HG001489:128368:129060:-1 gene:CHC_T00008170001 transcript:CDF32406 gene_biotype:protein_coding transcript_biotype:protein_coding Chondrus_crispus OG0000014
CDF40678 pep supercontig:ASM35022v2:HG002227:484528:486427:1 gene:CHC_T00007049001 transcript:CDF40678 gene_biotype:protein_coding transcript_biotype:protein_coding Chondrus_crispus OG0000014
CDF38001 pep supercontig:ASM35022v2:HG001892:69184:70508:1 gene:CHC_T00006044001 transcript:CDF38001 gene_biotype:protein_coding transcript_biotype:protein_coding Chondrus_crispus OG0000014
CDF38002 pep supercontig:ASM35022v2:HG001892:71104:73908:-1 gene:CHC_T00006045001 transcript:CDF38002 gene_biotype:protein_coding transcript_biotype:protein_coding Chondrus_crispus OG0000014
CDF41213 pep supercontig:ASM35022v2:HG002328:155081:158655:-1 gene:CHC_T00007747001 transcript:CDF41213 gene_biotype:protein_coding transcript_biotype:protein_coding Chondrus_crispus OG0000014
CDF38852 pep supercontig:ASM35022v2:HG001973:61861:62037:1 gene:CHC_T00001282001 transcript:CDF38852 gene_biotype:protein_coding transcript_biotype:protein_coding Chondrus_crispus OG0000014
CDF39628 pep supercontig:ASM35022v2:HG002060:33011:33844:1 gene:CHC_T00009279001 transcript:CDF39628 gene_biotype:protein_coding transcript_biotype:protein_coding description:Ribonuclease H (fragment)   Chondrus_crispus OG0000014
CDF39069 pep supercontig:ASM35022v2:HG001993:1:1941:1 gene:CHC_T00000095001 transcript:CDF39069 gene_biotype:protein_coding transcript_biotype:protein_coding Chondrus_crispus OG0000014
CDF32635 pep supercontig:ASM35022v2:HG001531:59607:59792:-1 gene:CHC_T00001517001 transcript:CDF32635 gene_biotype:protein_coding transcript_biotype:protein_coding Chondrus_crispus OG0000014
CDF35792 pep supercontig:ASM35022v2:HG001747:98148:100946:-1 gene:CHC_T00004161001 transcript:CDF35792 gene_biotype:protein_coding transcript_biotype:protein_coding Chondrus_crispus OG0000014
CDF39153 pep supercontig:ASM35022v2:HG002007:96:2552:1

gene:CHC_T00000140001 transcript:CDF39153
gene_biotype:protein_coding transcript_biotype:protein_coding
Chondrus_crispus OG0000014
CDF40971 pep supercontig:ASM35022v2:HG002285:226298:227677:-1
gene:CHC_T00007558001 transcript:CDF40971
gene_biotype:protein_coding transcript_biotype:protein_coding
Chondrus_crispus OG0000014
CDF40411 pep supercontig:ASM35022v2:HG002168:167355:169028:-1
gene:CHC_T00007188001 transcript:CDF40411
gene_biotype:protein_coding transcript_biotype:protein_coding
Chondrus_crispus OG0000014
CDF38259 pep supercontig:ASM35022v2:HG001907:266886:267897:-1
gene:CHC_T00006098001 transcript:CDF38259
gene_biotype:protein_coding transcript_biotype:protein_coding
Chondrus_crispus OG0000014
CDF38161 pep supercontig:ASM35022v2:HG001898:808887:809144:-1
gene:CHC_T00006340001 transcript:CDF38161
gene_biotype:protein_coding transcript_biotype:protein_coding
Chondrus_crispus OG0000014
CDF38257 pep supercontig:ASM35022v2:HG001907:191139:192614:-1
gene:CHC_T00006096001 transcript:CDF38257
gene_biotype:protein_coding transcript_biotype:protein_coding
Chondrus_crispus OG0000014
CDF37935 pep supercontig:ASM35022v2:HG001885:11106:13652:1
gene:CHC_T00000057001 transcript:CDF37935
gene_biotype:protein_coding transcript_biotype:protein_coding
Chondrus_crispus OG0000014
CDF38075 pep supercontig:ASM35022v2:HG001898:223231:224967:1
gene:CHC_T00006264001 transcript:CDF38075
gene_biotype:protein_coding transcript_biotype:protein_coding
Chondrus_crispus OG0000014
CDF38384 pep supercontig:ASM35022v2:HG001929:13815:16464:1
gene:CHC_T00001026001 transcript:CDF38384
gene_biotype:protein_coding transcript_biotype:protein_coding
Chondrus_crispus OG0000014
CDF32470 pep supercontig:ASM35022v2:HG001501:213691:216078:1
gene:CHC_T00008257001 transcript:CDF32470
gene_biotype:protein_coding transcript_biotype:protein_coding
Chondrus_crispus OG0000014
CDF37990 pep supercontig:ASM35022v2:HG001891:24089:25691:1
gene:CHC_T00000501001 transcript:CDF37990
gene_biotype:protein_coding transcript_biotype:protein_coding
Chondrus_crispus OG0000014
CDF37989 pep supercontig:ASM35022v2:HG001891:6036:8080:1
gene:CHC_T00000500001 transcript:CDF37989
gene_biotype:protein_coding transcript_biotype:protein_coding
Chondrus_crispus OG0000014
CDF40036 pep supercontig:ASM35022v2:HG002111:15862:17561:-1
gene:CHC_T00008874001 transcript:CDF40036
gene_biotype:protein_coding transcript_biotype:protein_coding
description:retrotransposon protein Chondrus_crispus
OG0000014
CDF77564 pep supercontig:ASM35022v2:HG001843:477354:478970:1
gene:CHC_T00005419001 transcript:CDF77564

gene_biotype:protein_coding transcript_biotype:protein_coding
Chondrus_crispus OG0000014
CDF33987 pep supercontig:ASM35022v2:HG001664:533540:535916:1
gene:CHC_T00002487001 transcript:CDF33987
gene_biotype:protein_coding transcript_biotype:protein_coding
Chondrus_crispus OG0000014
CDF36773 pep supercontig:ASM35022v2:HG001800:450912:452713:-1
gene:CHC_T00004819001 transcript:CDF36773
gene_biotype:protein_coding transcript_biotype:protein_coding
Chondrus_crispus OG0000014
CDF33201 pep supercontig:ASM35022v2:HG001637:35836:37572:-1
gene:CHC_T00001893001 transcript:CDF33201
gene_biotype:protein_coding transcript_biotype:protein_coding
Chondrus_crispus OG0000014
CDF33203 pep supercontig:ASM35022v2:HG001637:58516:60594:1
gene:CHC_T00001895001 transcript:CDF33203
gene_biotype:protein_coding transcript_biotype:protein_coding
Chondrus_crispus OG0000014
CDF32726 pep supercontig:ASM35022v2:HG001553:156481:158838:-1
gene:CHC_T00001590001 transcript:CDF32726
gene_biotype:protein_coding transcript_biotype:protein_coding
Chondrus_crispus OG0000014
CDF33346 pep supercontig:ASM35022v2:HG001642:135634:138211:1
gene:CHC_T00001936001 transcript:CDF33346
gene_biotype:protein_coding transcript_biotype:protein_coding
Chondrus_crispus OG0000014
CDF39662 pep supercontig:ASM35022v2:HG002061:36113:39118:1
gene:CHC_T00000408001 transcript:CDF39662
gene_biotype:protein_coding transcript_biotype:protein_coding
Chondrus_crispus OG0000014
CDF38965 pep supercontig:ASM35022v2:HG001983:8283:8534:1
gene:CHC_T00006682001 transcript:CDF38965
gene_biotype:protein_coding transcript_biotype:protein_coding
Chondrus_crispus OG0000014
CDF32342 pep supercontig:ASM35022v2:HG001477:93305:93901:-1
gene:CHC_T00008074001 transcript:CDF32342
gene_biotype:protein_coding transcript_biotype:protein_coding
Chondrus_crispus OG0000014
CDF33409 pep supercontig:ASM35022v2:HG001644:89797:91445:1
gene:CHC_T00002183001 transcript:CDF33409
gene_biotype:protein_coding transcript_biotype:protein_coding
Chondrus_crispus OG0000014
CDF39394 pep supercontig:ASM35022v2:HG002027:24890:27636:-1
gene:CHC_T00000258001 transcript:CDF39394
gene_biotype:protein_coding transcript_biotype:protein_coding
Chondrus_crispus OG0000014
CDF40830 pep supercontig:ASM35022v2:HG002251:205044:206801:-1
gene:CHC_T00007461001 transcript:CDF40830
gene_biotype:protein_coding transcript_biotype:protein_coding
Chondrus_crispus OG0000014
CDF34164 pep supercontig:ASM35022v2:HG001675:324388:327017:-1
gene:CHC_T00002674001 transcript:CDF34164
gene_biotype:protein_coding transcript_biotype:protein_coding
Chondrus_crispus OG0000014

CDF38512 pep supercontig:ASM35022v2:HG001940:103590:106345:-1
gene:CHC_T00006233001 transcript:CDF38512
gene_biotype:protein_coding transcript_biotype:protein_coding
Chondrus_crispus OG0000014
CDF38508 pep supercontig:ASM35022v2:HG001940:11631:12464:1
gene:CHC_T00006230001 transcript:CDF38508
gene_biotype:protein_coding transcript_biotype:protein_coding
Chondrus_crispus OG0000014
CDF36681 pep supercontig:ASM35022v2:HG001796:95855:97140:1
gene:CHC_T00005063001 transcript:CDF36681
gene_biotype:protein_coding transcript_biotype:protein_coding
Chondrus_crispus OG0000014
CDF33588 pep supercontig:ASM35022v2:HG001650:123757:124026:1
gene:CHC_T00002311001 transcript:CDF33588
gene_biotype:protein_coding transcript_biotype:protein_coding
Chondrus_crispus OG0000014
CDF38390 pep supercontig:ASM35022v2:HG001931:19573:21404:-1
gene:CHC_T00001041001 transcript:CDF38390
gene_biotype:protein_coding transcript_biotype:protein_coding
Chondrus_crispus OG0000014
CDF38803 pep supercontig:ASM35022v2:HG001968:52236:54205:-1
gene:CHC_T00001223001 transcript:CDF38803
gene_biotype:protein_coding transcript_biotype:protein_coding
Chondrus_crispus OG0000014
CDF41288 pep supercontig:ASM35022v2:HG002339:270363:272576:-1
gene:CHC_T00007801001 transcript:CDF41288
gene_biotype:protein_coding transcript_biotype:protein_coding
Chondrus_crispus OG0000014
CDF36412 pep supercontig:ASM35022v2:HG001778:63187:64240:-1
gene:CHC_T00004824001 transcript:CDF36412
gene_biotype:protein_coding transcript_biotype:protein_coding
Chondrus_crispus OG0000014
CDF41202 pep supercontig:ASM35022v2:HG002320:261625:262197:-1
gene:CHC_T00007742001 transcript:CDF41202
gene_biotype:protein_coding transcript_biotype:protein_coding
Chondrus_crispus OG0000014
CDF33720 pep supercontig:ASM35022v2:HG001655:165371:166426:-1
gene:CHC_T00002507001 transcript:CDF33720
gene_biotype:protein_coding transcript_biotype:protein_coding
Chondrus_crispus OG0000014
CDF37514 pep supercontig:ASM35022v2:HG001846:74534:77138:-1
gene:CHC_T00005765001 transcript:CDF37514
gene_biotype:protein_coding transcript_biotype:protein_coding
Chondrus_crispus OG0000014
CDF39938 pep supercontig:ASM35022v2:HG002094:138649:139938:1
gene:CHC_T00006898001 transcript:CDF39938
gene_biotype:protein_coding transcript_biotype:protein_coding
Chondrus_crispus OG0000014
CDF39905 pep supercontig:ASM35022v2:HG002094:21:2258:1
gene:CHC_T00006867001 transcript:CDF39905
gene_biotype:protein_coding transcript_biotype:protein_coding
Chondrus_crispus OG0000014
CDF32570 pep supercontig:ASM35022v2:HG001519:74040:76766:1
gene:CHC_T00008242001 transcript:CDF32570

gene_biotype:protein_coding transcript_biotype:protein_coding
Chondrus_crispus OG0000014
CDF32578 pep supercontig:ASM35022v2:HG001519:118131:119964:1
gene:CHC_T00008250001 transcript:CDF32578
gene_biotype:protein_coding transcript_biotype:protein_coding
Chondrus_crispus OG0000014
CDF33132 pep supercontig:ASM35022v2:HG001635:6882:8180:-1
gene:CHC_T00001986001 transcript:CDF33132
gene_biotype:protein_coding transcript_biotype:protein_coding
Chondrus_crispus OG0000014
CDF39331 pep supercontig:ASM35022v2:HG002022:307947:310245:-1
gene:CHC_T00006619001 transcript:CDF39331
gene_biotype:protein_coding transcript_biotype:protein_coding
Chondrus_crispus OG0000014
CDF38334 pep supercontig:ASM35022v2:HG001918:9421:10080:1
gene:CHC_T00000934001 transcript:CDF38334
gene_biotype:protein_coding transcript_biotype:protein_coding
Chondrus_crispus OG0000014
CDF36784 pep supercontig:ASM35022v2:HG001802:113190:115828:-1
gene:CHC_T00005210001 transcript:CDF36784
gene_biotype:protein_coding transcript_biotype:protein_coding
Chondrus_crispus OG0000014
CDF38169 pep supercontig:ASM35022v2:HG001899:56:2125:1
gene:CHC_T00006052001 transcript:CDF38169
gene_biotype:protein_coding transcript_biotype:protein_coding
Chondrus_crispus OG0000014
CDF36417 pep supercontig:ASM35022v2:HG001779:168:2525:1
gene:CHC_T00004840001 transcript:CDF36417
gene_biotype:protein_coding transcript_biotype:protein_coding
Chondrus_crispus OG0000014
CDF36419 pep supercontig:ASM35022v2:HG001779:50695:52411:1
gene:CHC_T00004842001 transcript:CDF36419
gene_biotype:protein_coding transcript_biotype:protein_coding
Chondrus_crispus OG0000014
CDF32918 pep supercontig:ASM35022v2:HG001606:235088:236778:-1
gene:CHC_T00001741001 transcript:CDF32918
gene_biotype:protein_coding transcript_biotype:protein_coding
Chondrus_crispus OG0000014
CDF40870 pep supercontig:ASM35022v2:HG002260:211579:213414:1
gene:CHC_T00007507001 transcript:CDF40870
gene_biotype:protein_coding transcript_biotype:protein_coding
Chondrus_crispus OG0000014
CDF40838 pep supercontig:ASM35022v2:HG002260:35:1897:1
gene:CHC_T00007477001 transcript:CDF40838
gene_biotype:protein_coding transcript_biotype:protein_coding
Chondrus_crispus OG0000014
CDF40922 pep supercontig:ASM35022v2:HG002270:9024:11261:-1
gene:CHC_T00000900001 transcript:CDF40922
gene_biotype:protein_coding transcript_biotype:protein_coding
Chondrus_crispus OG0000014
CDF41006 pep supercontig:ASM35022v2:HG002299:5368:6483:-1
gene:CHC_T00000909001 transcript:CDF41006
gene_biotype:protein_coding transcript_biotype:protein_coding
Chondrus_crispus OG0000014

CDF35613 pep supercontig:ASM35022v2:HG001737:120046:121836:-1
gene:CHC_T00004066001 transcript:CDF35613
gene_biotype:protein_coding transcript_biotype:protein_coding
Chondrus_crispus OG0000014
CDF36421 pep supercontig:ASM35022v2:HG001780:100832:103588:-1
gene:CHC_T00004364001 transcript:CDF36421
gene_biotype:protein_coding transcript_biotype:protein_coding
Chondrus_crispus OG0000014
CDF34106 pep supercontig:ASM35022v2:HG001673:165717:166338:1
gene:CHC_T00002790001 transcript:CDF34106
gene_biotype:protein_coding transcript_biotype:protein_coding
Chondrus_crispus OG0000014
CDF35428 pep supercontig:ASM35022v2:HG001730:17316:21014:-1
gene:CHC_T00003944001 transcript:CDF35428
gene_biotype:protein_coding transcript_biotype:protein_coding
Chondrus_crispus OG0000014
CDF34348 pep supercontig:ASM35022v2:HG001686:198344:201135:-1
gene:CHC_T00003084001 transcript:CDF34348
gene_biotype:protein_coding transcript_biotype:protein_coding
Chondrus_crispus OG0000014
CDF37529 pep supercontig:ASM35022v2:HG001848:36371:37107:1
gene:CHC_T00005751001 transcript:CDF37529
gene_biotype:protein_coding transcript_biotype:protein_coding
Chondrus_crispus OG0000014
CDF37532 pep supercontig:ASM35022v2:HG001848:77272:78258:-1
gene:CHC_T00005754001 transcript:CDF37532
gene_biotype:protein_coding transcript_biotype:protein_coding
Chondrus_crispus OG0000014
CDF34964 pep supercontig:ASM35022v2:HG001712:59145:60977:-1
gene:CHC_T00003566001 transcript:CDF34964
gene_biotype:protein_coding transcript_biotype:protein_coding
Chondrus_crispus OG0000014
CDF40992 pep supercontig:ASM35022v2:HG002294:172767:175340:-1
gene:CHC_T00007572001 transcript:CDF40992
gene_biotype:protein_coding transcript_biotype:protein_coding
Chondrus_crispus OG0000014
CDF40047 pep supercontig:ASM35022v2:HG002116:121859:122697:-1
gene:CHC_T00006934001 transcript:CDF40047
gene_biotype:protein_coding transcript_biotype:protein_coding
Chondrus_crispus OG0000014
CDF40057 pep supercontig:ASM35022v2:HG002116:379728:382791:1
gene:CHC_T00006943001 transcript:CDF40057
gene_biotype:protein_coding transcript_biotype:protein_coding
Chondrus_crispus OG0000014
CDF38306 pep supercontig:ASM35022v2:HG001915:28238:30997:1
gene:CHC_T00000808001 transcript:CDF38306
gene_biotype:protein_coding transcript_biotype:protein_coding
Chondrus_crispus OG0000014
60297 pacid=27392698 transcript=60297 locus=Genemark1.8613_g
ID=60297.2.0.227 annot-version=v2.0 Coccomyxa_subellipsoidea
OG0000015
kfl00020_0350_v1.1 Klebsormidium_flaccidum OG0000015
kfl00834_0050_v1.1 Klebsormidium_flaccidum OG0000015
kfl00116_0180_v1.1 Klebsormidium_flaccidum OG0000015

kfl00008_0050_v1.1   Klebsormidium_flaccidum OG0000015
kfl00008_0060_v1.1   Klebsormidium_flaccidum OG0000015
kfl00190_0200_v1.1   Klebsormidium_flaccidum OG0000015
kfl00190_0210_v1.1   Klebsormidium_flaccidum OG0000015
kfl00011_0050_v1.1   Klebsormidium_flaccidum OG0000015
kfl00218_0170_v1.1   Klebsormidium_flaccidum OG0000015
kfl00266_0170_v1.1   Klebsormidium_flaccidum OG0000015
kfl00266_0175_v1.1   Klebsormidium_flaccidum OG0000015
kfl00300_0110_v1.1   Klebsormidium_flaccidum OG0000015
Pp3c7_22870V3.1.p pacid=32926241 transcript=Pp3c7_22870V3.1
locus=Pp3c7_22870 annot-version=v3.3   Physcomitrella_patens
OG0000015
Pp3c10_17370V3.1.p pacid=32902189 transcript=Pp3c10_17370V3.1
locus=Pp3c10_17370 annot-version=v3.3   Physcomitrella_patens
OG0000015
Pp3c13_9320V3.1.p pacid=32931257 transcript=Pp3c13_9320V3.1
locus=Pp3c13_9320 annot-version=v3.3   Physcomitrella_patens
OG0000015
Pp3c10_11150V3.1.p pacid=32902765 transcript=Pp3c10_11150V3.1
locus=Pp3c10_11150 annot-version=v3.3   Physcomitrella_patens
OG0000015
Pp3c2_2900V3.1.p pacid=32933081 transcript=Pp3c2_2900V3.1
locus=Pp3c2_2900 annot-version=v3.3   Physcomitrella_patens
OG0000015
Pp3c2_15960V3.1.p pacid=32933515 transcript=Pp3c2_15960V3.1
locus=Pp3c2_15960 annot-version=v3.3   Physcomitrella_patens
OG0000015
Pp3c2_17490V3.1.p pacid=32933637 transcript=Pp3c2_17490V3.1
locus=Pp3c2_17490 annot-version=v3.3   Physcomitrella_patens
OG0000015
Pp3c2_8640V3.1.p pacid=32933928 transcript=Pp3c2_8640V3.1
locus=Pp3c2_8640 annot-version=v3.3   Physcomitrella_patens
OG0000015
Pp3c2_23550V3.1.p pacid=32934529 transcript=Pp3c2_23550V3.1
locus=Pp3c2_23550 annot-version=v3.3   Physcomitrella_patens
OG0000015
Pp3c22_20830V3.1.p pacid=32903022 transcript=Pp3c22_20830V3.1
locus=Pp3c22_20830 annot-version=v3.3   Physcomitrella_patens
OG0000015
Pp3c2_30910V3.1.p pacid=32936371 transcript=Pp3c2_30910V3.1
locus=Pp3c2_30910 annot-version=v3.3   Physcomitrella_patens
OG0000015
Pp3c2_33980V3.1.p pacid=32936391 transcript=Pp3c2_33980V3.1
locus=Pp3c2_33980 annot-version=v3.3   Physcomitrella_patens
OG0000015
Pp3c2_2920V3.1.p pacid=32937620 transcript=Pp3c2_2920V3.1
locus=Pp3c2_2920 annot-version=v3.3   Physcomitrella_patens
OG0000015
Pp3c19_8950V3.1.p pacid=32939123 transcript=Pp3c19_8950V3.1
locus=Pp3c19_8950 annot-version=v3.3   Physcomitrella_patens
OG0000015
Pp3c19_9000V3.1.p pacid=32940116 transcript=Pp3c19_9000V3.1
locus=Pp3c19_9000 annot-version=v3.3   Physcomitrella_patens
OG0000015

Pp3c20_19320V3.1.p pacid=32947358 transcript=Pp3c20_19320V3.1 locus=Pp3c20_19320 annot-version=v3.3 Physcomitrella_patens OG0000015
Pp3c23_17300V3.1.p pacid=32950549 transcript=Pp3c23_17300V3.1 locus=Pp3c23_17300 annot-version=v3.3 Physcomitrella_patens OG0000015
Pp3c22_20780V3.1.p pacid=32904792 transcript=Pp3c22_20780V3.1 locus=Pp3c22_20780 annot-version=v3.3 Physcomitrella_patens OG0000015
Pp3c5_15550V3.1.p pacid=32954084 transcript=Pp3c5_15550V3.1 locus=Pp3c5_15550 annot-version=v3.3 Physcomitrella_patens OG0000015
Pp3c10_18270V3.1.p pacid=32899934 transcript=Pp3c10_18270V3.1 locus=Pp3c10_18270 annot-version=v3.3 Physcomitrella_patens OG0000015
Pp3c11_12330V3.1.p pacid=32958079 transcript=Pp3c11_12330V3.1 locus=Pp3c11_12330 annot-version=v3.3 Physcomitrella_patens OG0000015
Pp3c17_12600V3.1.p pacid=32905468 transcript=Pp3c17_12600V3.1 locus=Pp3c17_12600 annot-version=v3.3 Physcomitrella_patens OG0000015
Pp3c11_25640V3.1.p pacid=32959336 transcript=Pp3c11_25640V3.1 locus=Pp3c11_25640 annot-version=v3.3 Physcomitrella_patens OG0000015
Pp3c14_25780V3.1.p pacid=32960076 transcript=Pp3c14_25780V3.1 locus=Pp3c14_25780 annot-version=v3.3 Physcomitrella_patens OG0000015
Pp3c14_11270V3.1.p pacid=32962171 transcript=Pp3c14_11270V3.1 locus=Pp3c14_11270 annot-version=v3.3 Physcomitrella_patens OG0000015
Pp3c14_4080V3.1.p pacid=32962346 transcript=Pp3c14_4080V3.1 locus=Pp3c14_4080 annot-version=v3.3 Physcomitrella_patens OG0000015
Pp3c14_11277V3.1.p pacid=32962650 transcript=Pp3c14_11277V3.1 locus=Pp3c14_11277 annot-version=v3.3 Physcomitrella_patens OG0000015
Pp3c14_19530V3.1.p pacid=32963216 transcript=Pp3c14_19530V3.1 locus=Pp3c14_19530 annot-version=v3.3 Physcomitrella_patens OG0000015
Pp3c8_1460V3.1.p pacid=32964263 transcript=Pp3c8_1460V3.1 locus=Pp3c8_1460 annot-version=v3.3 Physcomitrella_patens OG0000015
Pp3c1_17860V3.1.p pacid=32968494 transcript=Pp3c1_17860V3.1 locus=Pp3c1_17860 annot-version=v3.3 Physcomitrella_patens OG0000015
Pp3c1_33660V3.1.p pacid=32968499 transcript=Pp3c1_33660V3.1 locus=Pp3c1_33660 annot-version=v3.3 Physcomitrella_patens OG0000015
Pp3c1_17870V3.1.p pacid=32968678 transcript=Pp3c1_17870V3.1 locus=Pp3c1_17870 annot-version=v3.3 Physcomitrella_patens OG0000015
Pp3c17_19335V3.1.p pacid=32906569 transcript=Pp3c17_19335V3.1 locus=Pp3c17_19335 annot-version=v3.3 Physcomitrella_patens OG0000015

Pp3c1_2040V3.1.p pacid=32970847 transcript=Pp3c1_2040V3.1
locus=Pp3c1_2040 annot-version=v3.3 Physcomitrella_patens
OG0000015
Pp3c1_16800V3.1.p pacid=32971155 transcript=Pp3c1_16800V3.1
locus=Pp3c1_16800 annot-version=v3.3 Physcomitrella_patens
OG0000015
Pp3c1_33670V3.1.p pacid=32971264 transcript=Pp3c1_33670V3.1
locus=Pp3c1_33670 annot-version=v3.3 Physcomitrella_patens
OG0000015
Pp3c1_35120V3.1.p pacid=32971756 transcript=Pp3c1_35120V3.1
locus=Pp3c1_35120 annot-version=v3.3 Physcomitrella_patens
OG0000015
Pp3c12_25610V3.1.p pacid=32973034 transcript=Pp3c12_25610V3.1
locus=Pp3c12_25610 annot-version=v3.3 Physcomitrella_patens
OG0000015
Pp3c6_1880V3.1.p pacid=32977923 transcript=Pp3c6_1880V3.1
locus=Pp3c6_1880 annot-version=v3.3 Physcomitrella_patens
OG0000015
Pp3c6_26470V3.1.p pacid=32978654 transcript=Pp3c6_26470V3.1
locus=Pp3c6_26470 annot-version=v3.3 Physcomitrella_patens
OG0000015
Pp3c25_620V3.1.p pacid=32979847 transcript=Pp3c25_620V3.1
locus=Pp3c25_620 annot-version=v3.3 Physcomitrella_patens
OG0000015
Pp3c17_21660V3.1.p pacid=32907698 transcript=Pp3c17_21660V3.1
locus=Pp3c17_21660 annot-version=v3.3 Physcomitrella_patens
OG0000015
Pp3c17_3620V3.1.p pacid=32907702 transcript=Pp3c17_3620V3.1
locus=Pp3c17_3620 annot-version=v3.3 Physcomitrella_patens
OG0000015
Pp3c10_18120V3.1.p pacid=32899431 transcript=Pp3c10_18120V3.1
locus=Pp3c10_18120 annot-version=v3.3 Physcomitrella_patens
OG0000015
Pp3c18_1530V3.1.p pacid=32981842 transcript=Pp3c18_1530V3.1
locus=Pp3c18_1530 annot-version=v3.3 Physcomitrella_patens
OG0000015
Pp3c18_1550V3.1.p pacid=32983220 transcript=Pp3c18_1550V3.1
locus=Pp3c18_1550 annot-version=v3.3 Physcomitrella_patens
OG0000015
Pp3c16_5600V3.1.p pacid=32985615 transcript=Pp3c16_5600V3.1
locus=Pp3c16_5600 annot-version=v3.3 Physcomitrella_patens
OG0000015
Pp3c16_6900V3.1.p pacid=32986101 transcript=Pp3c16_6900V3.1
locus=Pp3c16_6900 annot-version=v3.3 Physcomitrella_patens
OG0000015
Pp3c17_2780V3.1.p pacid=32908306 transcript=Pp3c17_2780V3.1
locus=Pp3c17_2780 annot-version=v3.3 Physcomitrella_patens
OG0000015
Pp3c24_15090V3.1.p pacid=32908625 transcript=Pp3c24_15090V3.1
locus=Pp3c24_15090 annot-version=v3.3 Physcomitrella_patens
OG0000015
Pp3c10_25890V3.1.p pacid=32900531 transcript=Pp3c10_25890V3.1
locus=Pp3c10_25890 annot-version=v3.3 Physcomitrella_patens
OG0000015

Pp3c9_4610V3.1.p pacid=32911650 transcript=Pp3c9_4610V3.1 locus=Pp3c9_4610 annot-version=v3.3 Physcomitrella_patens OG0000015
Pp3c9_4680V3.1.p pacid=32912226 transcript=Pp3c9_4680V3.1 locus=Pp3c9_4680 annot-version=v3.3 Physcomitrella_patens OG0000015
Pp3c10_5750V3.1.p pacid=32900689 transcript=Pp3c10_5750V3.1 locus=Pp3c10_5750 annot-version=v3.3 Physcomitrella_patens OG0000015
Pp3c9_2920V3.1.p pacid=32913911 transcript=Pp3c9_2920V3.1 locus=Pp3c9_2920 annot-version=v3.3 Physcomitrella_patens OG0000015
Pp3c21_18740V3.1.p pacid=32914773 transcript=Pp3c21_18740V3.1 locus=Pp3c21_18740 annot-version=v3.3 Physcomitrella_patens OG0000015
Pp3c21_7510V3.1.p pacid=32915114 transcript=Pp3c21_7510V3.1 locus=Pp3c21_7510 annot-version=v3.3 Physcomitrella_patens OG0000015
Pp3c21_21000V3.1.p pacid=32915448 transcript=Pp3c21_21000V3.1 locus=Pp3c21_21000 annot-version=v3.3 Physcomitrella_patens OG0000015
Pp3c21_20950V3.1.p pacid=32915574 transcript=Pp3c21_20950V3.1 locus=Pp3c21_20950 annot-version=v3.3 Physcomitrella_patens OG0000015
Pp3c21_7520V3.1.p pacid=32915600 transcript=Pp3c21_7520V3.1 locus=Pp3c21_7520 annot-version=v3.3 Physcomitrella_patens OG0000015
Pp3c21_20530V3.1.p pacid=32916474 transcript=Pp3c21_20530V3.1 locus=Pp3c21_20530 annot-version=v3.3 Physcomitrella_patens OG0000015
Pp3c26_3320V3.1.p pacid=32918211 transcript=Pp3c26_3320V3.1 locus=Pp3c26_3320 annot-version=v3.3 Physcomitrella_patens OG0000015
Pp3c4_16320V3.1.p pacid=32920193 transcript=Pp3c4_16320V3.1 locus=Pp3c4_16320 annot-version=v3.3 Physcomitrella_patens OG0000015
Pp3c10_19250V3.1.p pacid=32899380 transcript=Pp3c10_19250V3.1 locus=Pp3c10_19250 annot-version=v3.3 Physcomitrella_patens OG0000015
Pp3c4_3160V3.1.p pacid=32920804 transcript=Pp3c4_3160V3.1 locus=Pp3c4_3160 annot-version=v3.3 Physcomitrella_patens OG0000015
Pp3c10_18260V3.1.p pacid=32901710 transcript=Pp3c10_18260V3.1 locus=Pp3c10_18260 annot-version=v3.3 Physcomitrella_patens OG0000015
Pp3c7_14900V3.1.p pacid=32923843 transcript=Pp3c7_14900V3.1 locus=Pp3c7_14900 annot-version=v3.3 Physcomitrella_patens OG0000015
Pp3c7_12790V3.1.p pacid=32925609 transcript=Pp3c7_12790V3.1 locus=Pp3c7_12790 annot-version=v3.3 Physcomitrella_patens OG0000015
AT2G36325.1 | GDSL-like Lipase/Acylhydrolase superfamily protein | Chr2:15231409-15233224 FORWARD LENGTH=356 | 201606 Arabidopsis_thaliana OG0000015

AT2G40250.1 | SGNH hydrolase-type esterase superfamily protein | Chr2:16813330-16815251 FORWARD LENGTH=361 | 201606 Arabidopsis_thaliana OG0000015
AT2G42990.1 | GDSL-like Lipase/Acylhydrolase superfamily protein | Chr2:17879056-17880200 FORWARD LENGTH=350 | 201606 Arabidopsis_thaliana OG0000015
AT3G04290.1 | Li-tolerant lipase 1 | Chr3:1133620-1136223 REVERSE LENGTH=366 | 201606 Arabidopsis_thaliana OG0000015
AT3G05180.1 | GDSL-like Lipase/Acylhydrolase superfamily protein | Chr3:1468599-1470529 REVERSE LENGTH=379 | 201606 Arabidopsis_thaliana OG0000015
AT3G09930.1 | GDSL-like Lipase/Acylhydrolase superfamily protein | Chr3:3053356-3055203 FORWARD LENGTH=354 | 201606 Arabidopsis_thaliana OG0000015
AT3G14210.1 | GDSL-like lipase/acylhydrolase superfamily protein | Chr3:4729886-4731562 FORWARD LENGTH=392 | 201606 Arabidopsis_thaliana OG0000015
AT3G14220.1 | GDSL-like Lipase/Acylhydrolase superfamily protein | Chr3:4733039-4734483 FORWARD LENGTH=363 | 201606 Arabidopsis_thaliana OG0000015
AT3G14225.1 | GDSL-motif lipase 4 | Chr3:4734616-4735993 REVERSE LENGTH=377 | 201606 Arabidopsis_thaliana OG0000015
AT3G14820.1 | GDSL-like Lipase/Acylhydrolase superfamily protein | Chr3:4978726-4980241 FORWARD LENGTH=351 | 201606 Arabidopsis_thaliana OG0000015
AT3G16370.1 | GDSL-like Lipase/Acylhydrolase superfamily protein | Chr3:5556928-5558351 FORWARD LENGTH=353 | 201606 Arabidopsis_thaliana OG0000015
AT3G26430.1 | GDSL-like Lipase/Acylhydrolase superfamily protein | Chr3:9674419-9675889 FORWARD LENGTH=380 | 201606 Arabidopsis_thaliana OG0000015
AT3G27950.1 | GDSL-like Lipase/Acylhydrolase superfamily protein | Chr3:10378048-10379771 FORWARD LENGTH=375 | 201606 Arabidopsis_thaliana OG0000015
AT3G43550.1 | GDSL-like Lipase/Acylhydrolase superfamily protein | Chr3:15448635-15449870 FORWARD LENGTH=288 | 201606 Arabidopsis_thaliana OG0000015
AT3G43570.1 | GDSL-like Lipase/Acylhydrolase superfamily protein | Chr3:15473345-15474765 FORWARD LENGTH=320 | 201606 Arabidopsis_thaliana OG0000015
AT3G48460.1 | GDSL-like Lipase/Acylhydrolase superfamily protein | Chr3:17949496-17951082 FORWARD LENGTH=381 | 201606 Arabidopsis_thaliana OG0000015
AT3G50400.1 | GDSL-like Lipase/Acylhydrolase superfamily protein | Chr3:18704797-18706437 FORWARD LENGTH=374 | 201606 Arabidopsis_thaliana OG0000015
AT3G53100.1 | GDSL-like Lipase/Acylhydrolase superfamily protein | Chr3:19684989-19686542 REVERSE LENGTH=351 | 201606 Arabidopsis_thaliana OG0000015
AT3G62280.1 | GDSL-like Lipase/Acylhydrolase superfamily protein | Chr3:23049484-23051116 REVERSE LENGTH=365 | 201606 Arabidopsis_thaliana OG0000015
AT4G01130.1 | GDSL-like Lipase/Acylhydrolase superfamily protein | Chr4:485868-488007 FORWARD LENGTH=382 | 201606

Arabidopsis_thaliana   OG0000015
AT4G10950.1 | SGNH hydrolase-type esterase superfamily protein |
Chr4:6711502-6713512 REVERSE LENGTH=400 | 201606
Arabidopsis_thaliana   OG0000015
AT4G16220.1 | GDSL-like Lipase/Acylhydrolase superfamily protein |
Chr4:9182283-9183922 FORWARD LENGTH=245 | 201606
Arabidopsis_thaliana   OG0000015
AT4G16230.1 | GDSL-like Lipase/Acylhydrolase superfamily protein |
Chr4:9185298-9186447 FORWARD LENGTH=251 | 201606
Arabidopsis_thaliana   OG0000015
AT4G16233.1 | GDSL-like lipase/acylhydrolase superfamily protein |
Chr4:9188044-9188537 FORWARD LENGTH=134 | 201606
Arabidopsis_thaliana   OG0000015
AT4G18970.1 | GDSL-like Lipase/Acylhydrolase superfamily protein |
Chr4:10389308-10390808 REVERSE LENGTH=361 | 201606
Arabidopsis_thaliana   OG0000015
AT4G26790.1 | GDSL-like Lipase/Acylhydrolase superfamily protein |
Chr4:13487859-13489149 FORWARD LENGTH=351 | 201606
Arabidopsis_thaliana   OG0000015
AT4G28780.1 | GDSL-like Lipase/Acylhydrolase superfamily protein |
Chr4:14215603-14217159 FORWARD LENGTH=367 | 201606
Arabidopsis_thaliana   OG0000015
AT4G30140.1 | GDSL-like Lipase/Acylhydrolase superfamily protein |
Chr4:14738603-14740548 REVERSE LENGTH=348 | 201606
Arabidopsis_thaliana   OG0000015
AT1G20120.1 | GDSL-like Lipase/Acylhydrolase superfamily protein |
Chr1:6975504-6977123 FORWARD LENGTH=402 | 201606
Arabidopsis_thaliana   OG0000015
AT1G20130.1 | GDSL-like Lipase/Acylhydrolase superfamily protein |
Chr1:6977939-6980003 FORWARD LENGTH=534 | 201606
Arabidopsis_thaliana   OG0000015
AT1G20132.1 | GDSL-like Lipase/Acylhydrolase superfamily protein |
Chr1:6981358-6983495 FORWARD LENGTH=383 | 201606
Arabidopsis_thaliana   OG0000015
AT1G20135.1 | GDSL-like Lipase/Acylhydrolase family protein |
Chr1:6984124-6985498 FORWARD LENGTH=328 | 201606
Arabidopsis_thaliana   OG0000015
AT5G03590.1 | GDSL-like Lipase/Acylhydrolase superfamily protein |
Chr5:908563-910149 FORWARD LENGTH=231 | 201606
Arabidopsis_thaliana   OG0000015
AT5G03600.1 | SGNH hydrolase-type esterase superfamily protein |
Chr5:912807-914226 FORWARD LENGTH=322 | 201606
Arabidopsis_thaliana   OG0000015
AT5G03610.1 | GDSL-like Lipase/Acylhydrolase superfamily protein |
Chr5:915650-918326 FORWARD LENGTH=359 | 201606
Arabidopsis_thaliana   OG0000015
AT5G03810.1 | GDSL-like Lipase/Acylhydrolase family protein |
Chr5:1013939-1015345 REVERSE LENGTH=353 | 201606
Arabidopsis_thaliana   OG0000015
AT5G03820.1 | GDSL-like Lipase/Acylhydrolase family protein |
Chr5:1015803-1017344 REVERSE LENGTH=357 | 201606
Arabidopsis_thaliana   OG0000015
AT5G03980.1 | SGNH hydrolase-type esterase superfamily protein |
Chr5:1074205-1075289 REVERSE LENGTH=323 | 201606

Arabidopsis_thaliana   OG0000015
AT5G08460.1 | GDSL-like Lipase/Acylhydrolase superfamily protein | Chr5:2733220-2735351 FORWARD LENGTH=385 | 201606
Arabidopsis_thaliana   OG0000015
AT5G14450.1 | GDSL-like Lipase/Acylhydrolase superfamily protein | Chr5:4658488-4660034 FORWARD LENGTH=389 | 201606
Arabidopsis_thaliana   OG0000015
AT5G15720.1 | GDSL-motif lipase 7 | Chr5:5124684-5126155 REVERSE LENGTH=364 | 201606   Arabidopsis_thaliana   OG0000015
AT5G18430.1 | GDSL-like Lipase/Acylhydrolase superfamily protein | Chr5:6110363-6111934 REVERSE LENGTH=362 | 201606
Arabidopsis_thaliana   OG0000015
AT5G22810.1 | GDSL-like lipase/acylhydrolase superfamily protein | Chr5:7621502-7623367 FORWARD LENGTH=359 | 201606
Arabidopsis_thaliana   OG0000015
AT5G33370.1 | GDSL-like Lipase/Acylhydrolase superfamily protein | Chr5:12602773-12604670 REVERSE LENGTH=366 | 201606
Arabidopsis_thaliana   OG0000015
AT1G23500.1 | GDSL-like Lipase/Acylhydrolase superfamily protein | Chr1:8339668-8341072 FORWARD LENGTH=345 | 201606
Arabidopsis_thaliana   OG0000015
AT5G37690.1 | SGNH hydrolase-type esterase superfamily protein | Chr5:14973610-14976115 REVERSE LENGTH=356 | 201606
Arabidopsis_thaliana   OG0000015
AT5G40990.1 | GDSL lipase 1 | Chr5:16418920-16420400 FORWARD LENGTH=374 | 201606   Arabidopsis_thaliana   OG0000015
AT5G41890.1 | GDSL-like Lipase/Acylhydrolase superfamily protein | Chr5:16764292-16766680 REVERSE LENGTH=375 | 201606
Arabidopsis_thaliana   OG0000015
AT5G42170.1 | SGNH hydrolase-type esterase superfamily protein | Chr5:16848786-16851707 FORWARD LENGTH=369 | 201606
Arabidopsis_thaliana   OG0000015
AT5G45670.1 | GDSL-like Lipase/Acylhydrolase superfamily protein | Chr5:18528600-18530164 FORWARD LENGTH=362 | 201606
Arabidopsis_thaliana   OG0000015
AT5G45910.1 | GDSL-like Lipase/Acylhydrolase superfamily protein | Chr5:18620420-18622264 REVERSE LENGTH=372 | 201606
Arabidopsis_thaliana   OG0000015
AT5G45950.1 | GDSL-like Lipase/Acylhydrolase superfamily protein | Chr5:18634155-18636512 FORWARD LENGTH=357 | 201606
Arabidopsis_thaliana   OG0000015
AT5G45960.1 | GDSL-like Lipase/Acylhydrolase superfamily protein | Chr5:18637174-18640319 REVERSE LENGTH=375 | 201606
Arabidopsis_thaliana   OG0000015
AT5G55050.1 | GDSL-like Lipase/Acylhydrolase superfamily protein | Chr5:22337745-22339741 FORWARD LENGTH=376 | 201606
Arabidopsis_thaliana   OG0000015
AT5G63170.1 | GDSL-like Lipase/Acylhydrolase superfamily protein | Chr5:25338699-25340096 REVERSE LENGTH=338 | 201606
Arabidopsis_thaliana   OG0000015
AT1G28570.1 | SGNH hydrolase-type esterase superfamily protein | Chr1:10041838-10044112 REVERSE LENGTH=389 | 201606
Arabidopsis_thaliana   OG0000015
AT1G28580.1 | GDSL-like Lipase/Acylhydrolase superfamily protein |

Chr1:10044603-10046379 REVERSE LENGTH=390 | 201606
Arabidopsis_thaliana   OG0000015
AT1G28590.1 | GDSL-like Lipase/Acylhydrolase superfamily protein |
Chr1:10047509-10049300 REVERSE LENGTH=403 | 201606
Arabidopsis_thaliana   OG0000015
AT1G28600.1 | GDSL-like Lipase/Acylhydrolase superfamily protein |
Chr1:10051228-10053073 REVERSE LENGTH=393 | 201606
Arabidopsis_thaliana   OG0000015
AT1G28610.1 | GDSL-like Lipase/Acylhydrolase superfamily protein |
Chr1:10054477-10055665 REVERSE LENGTH=250 | 201606
Arabidopsis_thaliana   OG0000015
AT1G28640.1 | GDSL-like Lipase/Acylhydrolase superfamily protein |
Chr1:10067563-10069109 REVERSE LENGTH=389 | 201606
Arabidopsis_thaliana   OG0000015
AT1G28650.1 | GDSL-like Lipase/Acylhydrolase superfamily protein |
Chr1:10069547-10071082 REVERSE LENGTH=385 | 201606
Arabidopsis_thaliana   OG0000015
AT1G28660.1 | GDSL-like Lipase/Acylhydrolase superfamily protein |
Chr1:10071856-10073371 REVERSE LENGTH=383 | 201606
Arabidopsis_thaliana   OG0000015
AT1G28670.1 | GDSL-like Lipase/Acylhydrolase superfamily protein |
Chr1:10074669-10076250 REVERSE LENGTH=384 | 201606
Arabidopsis_thaliana   OG0000015
AT1G29660.1 | GDSL-like Lipase/Acylhydrolase superfamily protein |
Chr1:10371955-10373624 FORWARD LENGTH=364 | 201606
Arabidopsis_thaliana   OG0000015
AT1G29670.1 | GDSL-like Lipase/Acylhydrolase superfamily protein |
Chr1:10375843-10377717 FORWARD LENGTH=363 | 201606
Arabidopsis_thaliana   OG0000015
AT1G31550.1 | GDSL-like Lipase/Acylhydrolase superfamily protein |
Chr1:11295635-11297284 REVERSE LENGTH=391 | 201606
Arabidopsis_thaliana   OG0000015
AT1G33811.1 | GDSL-like Lipase/Acylhydrolase superfamily protein |
Chr1:12267918-12269690 FORWARD LENGTH=370 | 201606
Arabidopsis_thaliana   OG0000015
AT1G53920.1 | GDSL-motif lipase 5 | Chr1:20137725-20139637 FORWARD
LENGTH=385 | 201606   Arabidopsis_thaliana   OG0000015
AT1G53940.1 | GDSL-motif lipase 2 | Chr1:20143279-20144453 FORWARD
LENGTH=305 | 201606   Arabidopsis_thaliana   OG0000015
AT1G53970.1 | GDSL esterase/lipase-like protein |
Chr1:20148439-20148805 FORWARD LENGTH=97 | 201606
Arabidopsis_thaliana   OG0000015
AT1G53990.1 | GDSL-motif lipase 3 | Chr1:20151016-20152752 FORWARD
LENGTH=367 | 201606   Arabidopsis_thaliana   OG0000015
AT1G54000.1 | GDSL-like Lipase/Acylhydrolase superfamily protein |
Chr1:20154548-20156365 REVERSE LENGTH=391 | 201606
Arabidopsis_thaliana   OG0000015
AT1G54010.1 | GDSL-like Lipase/Acylhydrolase superfamily protein |
Chr1:20158854-20160747 REVERSE LENGTH=386 | 201606
Arabidopsis_thaliana   OG0000015
AT1G54020.1 | GDSL-like Lipase/Acylhydrolase superfamily protein |
Chr1:20161805-20162923 REVERSE LENGTH=286 | 201606
Arabidopsis_thaliana   OG0000015
AT1G54030.1 | GDSL-like Lipase/Acylhydrolase superfamily protein |

Chr1:20167685-20169476 FORWARD LENGTH=417 | 201606
Arabidopsis_thaliana   OG0000015
AT1G54790.1 | GDSL-like Lipase/Acylhydrolase superfamily protein |
Chr1:20441124-20443997 REVERSE LENGTH=382 | 201606
Arabidopsis_thaliana   OG0000015
AT1G56670.1 | GDSL-like Lipase/Acylhydrolase superfamily protein |
Chr1:21241688-21243965 FORWARD LENGTH=373 | 201606
Arabidopsis_thaliana   OG0000015
AT1G58430.1 | GDSL-like Lipase/Acylhydrolase family protein |
Chr1:21711684-21712992 REVERSE LENGTH=360 | 201606
Arabidopsis_thaliana   OG0000015
AT1G58525.1 | GDSL-like lipase/acylhydrolase superfamily protein |
Chr1:21729913-21731344 FORWARD LENGTH=342 | 201606
Arabidopsis_thaliana   OG0000015
AT1G58725.1 | GDSL-like Lipase/Acylhydrolase superfamily protein |
Chr1:21771935-21773365 REVERSE LENGTH=349 | 201606
Arabidopsis_thaliana   OG0000015
AT1G59030.1 | GDSL-like Lipase/Acylhydrolase superfamily protein |
Chr1:21808193-21809623 REVERSE LENGTH=349 | 201606
Arabidopsis_thaliana   OG0000015
AT1G59406.1 | GDSL-like Lipase/Acylhydrolase superfamily protein |
Chr1:21844451-21845881 REVERSE LENGTH=349 | 201606
Arabidopsis_thaliana   OG0000015
AT1G67830.1 | alpha-fucosidase 1 | Chr1:25431705-25432972 REVERSE
LENGTH=372 | 201606   Arabidopsis_thaliana   OG0000015
AT1G71120.1 | GDSL-motif lipase/hydrolase 6 | Chr1:26821072-26822420
REVERSE LENGTH=362 | 201606   Arabidopsis_thaliana
OG0000015
AT1G71250.1 | GDSL-like Lipase/Acylhydrolase superfamily protein |
Chr1:26860125-26861582 FORWARD LENGTH=374 | 201606
Arabidopsis_thaliana   OG0000015
AT1G71691.1 | GDSL-like Lipase/Acylhydrolase superfamily protein |
Chr1:26949395-26951173 REVERSE LENGTH=283 | 201606
Arabidopsis_thaliana   OG0000015
AT1G06990.1 | GDSL-like Lipase/Acylhydrolase superfamily protein |
Chr1:2148393-2150009 FORWARD LENGTH=360 | 201606
Arabidopsis_thaliana   OG0000015
AT1G73610.1 | GDSL-like Lipase/Acylhydrolase superfamily protein |
Chr1:27678377-27679807 FORWARD LENGTH=344 | 201606
Arabidopsis_thaliana   OG0000015
AT1G74460.1 | GDSL-like Lipase/Acylhydrolase superfamily protein |
Chr1:27988150-27989765 REVERSE LENGTH=366 | 201606
Arabidopsis_thaliana   OG0000015
AT1G75880.1 | SGNH hydrolase-type esterase superfamily protein |
Chr1:28490564-28492298 FORWARD LENGTH=375 | 201606
Arabidopsis_thaliana   OG0000015
AT1G75890.1 | GDSL-like Lipase/Acylhydrolase superfamily protein |
Chr1:28493051-28495047 FORWARD LENGTH=379 | 201606
Arabidopsis_thaliana   OG0000015
AT1G75900.1 | GDSL-like Lipase/Acylhydrolase superfamily protein |
Chr1:28499179-28500943 FORWARD LENGTH=364 | 201606
Arabidopsis_thaliana   OG0000015
AT1G75910.1 | extracellular lipase 4 | Chr1:28501511-28503096
FORWARD LENGTH=343 | 201606   Arabidopsis_thaliana

OG0000015
AT1G75920.1 | GDSL-like Lipase/Acylhydrolase superfamily protein | Chr1:28505591-28507023 FORWARD LENGTH=353 | 201606 Arabidopsis_thaliana OG0000015
AT1G75930.1 | extracellular lipase 6 | Chr1:28508109-28509708 FORWARD LENGTH=343 | 201606 Arabidopsis_thaliana OG0000015
AT2G03980.1 | GDSL-like Lipase/Acylhydrolase superfamily protein | Chr2:1260907-1262408 FORWARD LENGTH=367 | 201606 Arabidopsis_thaliana OG0000015
AT2G04020.1 | GDSL-like Lipase/Acylhydrolase superfamily protein | Chr2:1274427-1275524 FORWARD LENGTH=261 | 201606 Arabidopsis_thaliana OG0000015
AT2G04570.1 | GDSL-like Lipase/Acylhydrolase superfamily protein | Chr2:1594747-1596129 FORWARD LENGTH=350 | 201606 Arabidopsis_thaliana OG0000015
AT2G19010.1 | GDSL-like Lipase/Acylhydrolase superfamily protein | Chr2:8243077-8245378 FORWARD LENGTH=348 | 201606 Arabidopsis_thaliana OG0000015
AT2G19050.1 | GDSL-like Lipase/Acylhydrolase superfamily protein | Chr2:8253416-8255534 FORWARD LENGTH=349 | 201606 Arabidopsis_thaliana OG0000015
AT2G19060.1 | SGNH hydrolase-type esterase superfamily protein | Chr2:8257031-8259815 FORWARD LENGTH=349 | 201606 Arabidopsis_thaliana OG0000015
AT2G23540.1 | GDSL-like Lipase/Acylhydrolase superfamily protein | Chr2:10024366-10026058 FORWARD LENGTH=387 | 201606 Arabidopsis_thaliana OG0000015
AT2G24560.1 | GDSL-like Lipase/Acylhydrolase family protein | Chr2:10431537-10432933 FORWARD LENGTH=363 | 201606 Arabidopsis_thaliana OG0000015
AT1G09390.1 | GDSL-like Lipase/Acylhydrolase superfamily protein | Chr1:3031264-3033415 FORWARD LENGTH=370 | 201606 Arabidopsis_thaliana OG0000015
AT2G27360.1 | GDSL-like Lipase/Acylhydrolase superfamily protein | Chr2:11706233-11707905 FORWARD LENGTH=394 | 201606 Arabidopsis_thaliana OG0000015
AT2G30220.1 | GDSL-like Lipase/Acylhydrolase family protein | Chr2:12891266-12892537 REVERSE LENGTH=358 | 201606 Arabidopsis_thaliana OG0000015
AT2G30310.1 | GDSL-like Lipase/Acylhydrolase family protein | Chr2:12923055-12924371 FORWARD LENGTH=359 | 201606 Arabidopsis_thaliana OG0000015
AT2G31540.1 | GDSL-like Lipase/Acylhydrolase superfamily protein | Chr2:13430733-13432045 REVERSE LENGTH=360 | 201606 Arabidopsis_thaliana OG0000015
AT2G31550.1 | SGNH hydrolase-type esterase superfamily protein | Chr2:13433904-13434769 REVERSE LENGTH=219 | 201606 Arabidopsis_thaliana OG0000015
Os03t0310000-01 Lipase, GDSL domain containing protein. Oryza_sativa OG0000015
Os03t0365000-00 Similar to GDSL-like Lipase/Acylhydrolase family protein. Oryza_sativa OG0000015
Os03t0365800-01 Lipase, GDSL domain containing protein.

Oryza_sativa	OG0000015
Os03t0365850-00 Lipase, GDSL domain containing protein.
Oryza_sativa	OG0000015
Os03t0365900-01 Similar to GDSL-like Lipase/Acylhydrolase family protein. Oryza_sativa	OG0000015
Os03t0580200-01 Lipase, GDSL domain containing protein.
Oryza_sativa	OG0000015
Os03t0580800-01 Lipase, GDSL domain containing protein.
Oryza_sativa	OG0000015
Os03t0581400-00 Similar to anther-specific proline-rich protein APG.
Oryza_sativa	OG0000015
Os03t0683800-01 Similar to Proline-rich protein APG-like.
Oryza_sativa	OG0000015
Os03t0844600-01 Esterase, SGNH hydrolase-type domain containing protein. Oryza_sativa	OG0000015
Os03t0859100-01 Esterase, SGNH hydrolase-type domain containing protein. Oryza_sativa	OG0000015
Os04t0507700-01 Lipase, GDSL domain containing protein.
Oryza_sativa	OG0000015
Os04t0547800-00 Conserved hypothetical protein.	Oryza_sativa
OG0000015
Os04t0561800-00 Similar to H0211B05.3 protein.	Oryza_sativa
OG0000015
Os01t0329900-01 Similar to Lipase homolog (Fragment).
Oryza_sativa	OG0000015
Os01t0330100-01 Similar to Alpha-L-fucosidase 2.	Oryza_sativa
OG0000015
Os01t0331100-01 Similar to Alpha-L-fucosidase 2.	Oryza_sativa
OG0000015
Os04t0577300-01 Similar to GDSL-motif lipase/hydrolase-like protein.
Oryza_sativa	OG0000015
Os04t0650200-01 Lipase, GDSL domain containing protein.
Oryza_sativa	OG0000015
Os05t0133401-01 Esterase, SGNH hydrolase-type domain containing protein. Oryza_sativa	OG0000015
Os05t0159200-01 Lipase, GDSL domain containing protein.
Oryza_sativa	OG0000015
Os05t0159500-00 Similar to Alpha-L-fucosidase 2.	Oryza_sativa
OG0000015
Os05t0209600-01 Similar to Esterase.	Oryza_sativa
OG0000015
Os05t0210100-01 Lipase, GDSL domain containing protein.
Oryza_sativa	OG0000015
Os05t0210400-00 Lipase, GDSL domain containing protein.
Oryza_sativa	OG0000015
Os05t0401000-01 Lipase, GDSL domain containing protein.
Oryza_sativa	OG0000015
Os05t0419800-01 Lipase, GDSL domain containing protein.
Oryza_sativa	OG0000015
Os05t0468500-01 Esterase, SGNH hydrolase-type domain containing protein. Oryza_sativa	OG0000015
Os01t0509750-00 Similar to Anther-specific proline-rich protein APG.
Oryza_sativa	OG0000015
Os01t0509800-00 Similar to Anther-specific proline-rich protein APG.

Oryza_sativa	OG0000015
Os05t0506450-00 Similar to esterase.	Oryza_sativa	OG0000015
Os05t0506500-00 Conserved hypothetical protein.	Oryza_sativa	OG0000015
Os05t0506600-00 Lipase, GDSL domain containing protein.	Oryza_sativa	OG0000015
Os05t0506800-01 Hypothetical conserved gene.	Oryza_sativa	OG0000015
Os05t0518300-01 Lipase, GDSL domain containing protein.	Oryza_sativa	OG0000015
Os06t0129600-00 Similar to Alpha-L-fucosidase 2.	Oryza_sativa	OG0000015
Os06t0148200-01 Lipase, GDSL domain containing protein.	Oryza_sativa	OG0000015
Os06t0149100-01 Similar to mRNA, clone	Oryza_sativa	OG0000015
Os06t0156600-01 Lipase, GDSL domain containing protein.	Oryza_sativa	OG0000015
Os06t0156700-01 Lipase, GDSL domain containing protein.	Oryza_sativa	OG0000015
Os06t0157000-01 Esterase, SGNH hydrolase-type domain containing protein.	Oryza_sativa	OG0000015
Os06t0160200-01 Similar to Lanatoside 15'-O-acetylesterase precursor.	Oryza_sativa	OG0000015
Os06t0229400-00 Conserved hypothetical protein.	Oryza_sativa	OG0000015
Os06t0257600-01 Esterase, SGNH hydrolase-type domain containing protein.	Oryza_sativa	OG0000015
Os06t0351500-01 Lipase, GDSL domain containing protein.	Oryza_sativa	OG0000015
Os06t0531600-01 Lipase, GDSL domain containing protein.	Oryza_sativa	OG0000015
Os06t0531900-01 Lipase, GDSL domain containing protein.	Oryza_sativa	OG0000015
Os06t0560700-01 Lipase, GDSL domain containing protein.	Oryza_sativa	OG0000015
Os06t0636700-01 Esterase, SGNH hydrolase-type domain containing protein.	Oryza_sativa	OG0000015
Os06t0694200-01 Esterase, SGNH hydrolase-type domain containing protein.	Oryza_sativa	OG0000015
Os06t0725100-01 Lipase, GDSL domain containing protein.	Oryza_sativa	OG0000015
Os06t0725200-01 Lipase, GDSL domain containing protein.	Oryza_sativa	OG0000015
Os01t0612900-01 Lipase, GDSL domain containing protein.	Oryza_sativa	OG0000015
Os07t0586100-01 Similar to Esterase precursor (EC 3.1.1.-) (Early nodule-specific protein homolog) (Latex allergen Hev b 13).	Oryza_sativa	OG0000015
Os07t0586200-01 Similar to Esterase precursor (EC 3.1.1.-) (Early nodule-specific protein homolog) (Latex allergen Hev b 13).	Oryza_sativa	OG0000015
Os07t0642200-01 Lipase, GDSL domain containing protein.

Oryza_sativa	OG0000015
Os07t0668300-01 Lipase, GDSL domain containing protein.
Oryza_sativa	OG0000015
Os08t0112900-01 Lipase, GDSL domain containing protein.
Oryza_sativa	OG0000015
Os01t0649200-01 Similar to Esterase.	Oryza_sativa
OG0000015
Os01t0649400-00 Similar to Esterase.	Oryza_sativa
OG0000015
Os01t0649900-01 Lipase, GDSL domain containing protein.
Oryza_sativa	OG0000015
Os01t0650000-01 Similar to esterase.	Oryza_sativa
OG0000015
Os01t0650100-01 Similar to Esterase.	Oryza_sativa
OG0000015
Os01t0650200-01 Lipase, GDSL domain containing protein.
Oryza_sativa	OG0000015
Os01t0650900-00 Lipase, GDSL domain containing protein.
Oryza_sativa	OG0000015
Os01t0651000-01 Lipase, GDSL domain containing protein.
Oryza_sativa	OG0000015
Os01t0651300-01 Similar to GDSL-motif lipase/hydrolase-like protein.
	Oryza_sativa	OG0000015
Os01t0651400-01 Lipase, GDSL domain containing protein.
Oryza_sativa	OG0000015
Os08t0565900-01 Lipase, GDSL domain containing protein.
Oryza_sativa	OG0000015
Os09t0132200-01 Esterase, SGNH hydrolase-type domain containing
protein.	Oryza_sativa	OG0000015
Os09t0132900-01 Lipase, GDSL domain containing protein.
Oryza_sativa	OG0000015
Os09t0247600-01 Lipase, GDSL domain containing protein.
Oryza_sativa	OG0000015
Os09t0540400-01 Similar to Anther-specific proline-rich protein APG.
	Oryza_sativa	OG0000015
Os09t0567800-00 Hypothetical conserved gene.	Oryza_sativa
OG0000015
Os10t0140300-01 Esterase, SGNH hydrolase-type domain containing
protein.	Oryza_sativa	OG0000015
Os10t0392800-00 Similar to esterase.	Oryza_sativa
OG0000015
Os10t0392900-00 Similar to esterase.	Oryza_sativa
OG0000015
Os10t0393600-00 Similar to esterase.	Oryza_sativa
OG0000015
Os10t0393700-00 Similar to esterase.	Oryza_sativa
OG0000015
Os10t0393800-01 Lipase, GDSL domain containing protein.
Oryza_sativa	OG0000015
Os10t0394000-01 Similar to GDSL-like Lipase/Acylhydrolase family
protein, expressed.	Oryza_sativa	OG0000015
Os10t0438600-01 Similar to Family II lipase EXL3.	Oryza_sativa
OG0000015
Os10t0463200-01 Esterase, SGNH hydrolase-type domain containing

protein. Oryza_sativa OG0000015
Os01t0728100-01 Lipase, GDSL domain containing protein.
Oryza_sativa OG0000015
Os11t0521000-01 Lipase, GDSL domain containing protein.
Oryza_sativa OG0000015
Os01t0748500-01 Similar to anther-specific proline-rich protein APG.
Oryza_sativa OG0000015
Os12t0126100-00 Similar to GDSL-like Lipase/Acylhydrolase family
protein, expressed. Oryza_sativa OG0000015
Os12t0274200-01 Lipase, GDSL domain containing protein.
Oryza_sativa OG0000015
Os12t0563300-00 Hypothetical conserved gene. Oryza_sativa
OG0000015
Os12t0566700-01 Lipase, GDSL domain containing protein.
Oryza_sativa OG0000015
Os01t0827700-00 Lipase, GDSL domain containing protein.
Oryza_sativa OG0000015
Os01t0832100-01 Esterase, SGNH hydrolase-type domain containing
protein. Oryza_sativa OG0000015
Os02t0101400-01 Lipase, GDSL domain containing protein.
Oryza_sativa OG0000015
Os02t0110000-01 Lipase, GDSL domain containing protein.
Oryza_sativa OG0000015
Os02t0189200-01 Lipase, GDSL domain containing protein.
Oryza_sativa OG0000015
Os02t0189300-01 Esterase, SGNH hydrolase-type domain containing
protein. Oryza_sativa OG0000015
Os02t0250400-01 Similar to Esterase. Oryza_sativa
OG0000015
Os02t0290900-00 Similar to GDSL-motif lipase/hydrolase family
protein. Oryza_sativa OG0000015
Os02t0291600-01 Lipase, GDSL domain containing protein.
Oryza_sativa OG0000015
Os02t0292600-01 Lipase, GDSL domain containing protein.
Oryza_sativa OG0000015
Os02t0458900-00 Conserved hypothetical protein. Oryza_sativa
OG0000015
Os02t0604000-00 Similar to alpha-L-fucosidase 2. Oryza_sativa
OG0000015
Os02t0608801-00 Lipase, GDSL domain containing protein.
Oryza_sativa OG0000015
Os02t0617400-01 Lipase, GDSL domain containing protein.
Oryza_sativa OG0000015
Os02t0668900-00 Similar to H0211B05.3 protein. Oryza_sativa
OG0000015
Os02t0669000-01 Lipase, GDSL domain containing protein.
Oryza_sativa OG0000015
Os02t0732800-00 Similar to Anther-specific proline-rich protein APG.
Oryza_sativa OG0000015
Os02t0740400-01 Similar to GSDL-motif lipase. Oryza_sativa
OG0000015
Os01t0214600-01 Lipase, GDSL domain containing protein.
Oryza_sativa OG0000015
Os01t0214800-01 Lipase, GDSL domain containing protein.

Oryza_sativa	OG0000015
Os01t0215000-01 Lipase, GDSL domain containing protein.	Oryza_sativa	OG0000015
Os01t0215500-01 Similar to esterase.	Oryza_sativa	OG0000015
Os01t0215700-01 Esterase, SGNH hydrolase-type domain containing protein.	Oryza_sativa	OG0000015
Os01t0216000-01 Lipase, GDSL domain containing protein.	Oryza_sativa	OG0000015
Os02t0816200-02 Lipase, GDSL domain containing protein.	Oryza_sativa	OG0000015
Os01t0216300-01 Lipase, GDSL domain containing protein.	Oryza_sativa	OG0000015
Os01t0216400-00 Lipase, GDSL domain containing protein.	Oryza_sativa	OG0000015
Os01t0216500-00 Lipase, GDSL domain containing protein.	Oryza_sativa	OG0000015
Os01t0216900-00 Lipase, GDSL domain containing protein.	Oryza_sativa	OG0000015
Os01t0223000-01 Lipase, GDSL domain containing protein.	Oryza_sativa	OG0000015
Os01t0223200-01 Lipase, GDSL domain containing protein.	Oryza_sativa	OG0000015
Os01t0223500-00 Similar to Esterase.	Oryza_sativa	OG0000015
g2342.t1	Caulerpa_lentillifera	OG0000016
g2556.t1	Caulerpa_lentillifera	OG0000016
g2657.t1	Caulerpa_lentillifera	OG0000016
g3580.t1	Caulerpa_lentillifera	OG0000016
g4403.t1	Caulerpa_lentillifera	OG0000016
g4653.t1	Caulerpa_lentillifera	OG0000016
g4654.t1	Caulerpa_lentillifera	OG0000016
g4799.t1	Caulerpa_lentillifera	OG0000016
g5727.t1	Caulerpa_lentillifera	OG0000016
g5758.t1	Caulerpa_lentillifera	OG0000016
g5759.t1	Caulerpa_lentillifera	OG0000016
g7022.t1	Caulerpa_lentillifera	OG0000016
g7030.t1	Caulerpa_lentillifera	OG0000016
g7045.t1	Caulerpa_lentillifera	OG0000016
g9216.t1	Caulerpa_lentillifera	OG0000016
g9271.t1	Caulerpa_lentillifera	OG0000016
g9272.t1	Caulerpa_lentillifera	OG0000016
Cre02.g096000.t1.1 pacid=30785390 transcript=Cre02.g096000.t1.1 locus=Cre02.g096000 ID=Cre02.g096000.t1.1.v5.5 annot-version=v5.5	Chlamydomonas_reinhardtii	OG0000016
Cre02.g095151.t1.1 pacid=30785745 transcript=Cre02.g095151.t1.1 locus=Cre02.g095151 ID=Cre02.g095151.t1.1.v5.5 annot-version=v5.5	Chlamydomonas_reinhardtii	OG0000016
Cre03.g169300.t1.2 pacid=30787423 transcript=Cre03.g169300.t1.2 locus=Cre03.g169300 ID=Cre03.g169300.t1.2.v5.5 annot-version=v5.5	Chlamydomonas_reinhardtii	OG0000016
Cre01.g029900.t1.2 pacid=30788563 transcript=Cre01.g029900.t1.2 locus=Cre01.g029900 ID=Cre01.g029900.t1.2.v5.5 annot-version=v5.5	Chlamydomonas_reinhardtii	OG0000016

Cre01.g007000.t1.2 pacid=30789403 transcript=Cre01.g007000.t1.2
locus=Cre01.g007000 ID=Cre01.g007000.t1.2.v5.5 annot-version=v5.5
Chlamydomonas_reinhardtii OG0000016
Cre07.g313250.t1.2 pacid=30774970 transcript=Cre07.g313250.t1.2
locus=Cre07.g313250 ID=Cre07.g313250.t1.2.v5.5 annot-version=v5.5
Chlamydomonas_reinhardtii OG0000016
Cre07.g327800.t1.1 pacid=30775067 transcript=Cre07.g327800.t1.1
locus=Cre07.g327800 ID=Cre07.g327800.t1.1.v5.5 annot-version=v5.5
Chlamydomonas_reinhardtii OG0000016
Cre04.g224500.t1.1 pacid=30791046 transcript=Cre04.g224500.t1.1
locus=Cre04.g224500 ID=Cre04.g224500.t1.1.v5.5 annot-version=v5.5
Chlamydomonas_reinhardtii OG0000016
Cre04.g220850.t1.2 pacid=30791308 transcript=Cre04.g220850.t1.2
locus=Cre04.g220850 ID=Cre04.g220850.t1.2.v5.5 annot-version=v5.5
Chlamydomonas_reinhardtii OG0000016
Cre04.g217850.t1.1 pacid=30791368 transcript=Cre04.g217850.t1.1
locus=Cre04.g217850 ID=Cre04.g217850.t1.1.v5.5 annot-version=v5.5
Chlamydomonas_reinhardtii OG0000016
Cre04.g224400.t1.1 pacid=30791538 transcript=Cre04.g224400.t1.1
locus=Cre04.g224400 ID=Cre04.g224400.t1.1.v5.5 annot-version=v5.5
Chlamydomonas_reinhardtii OG0000016
Cre12.g530900.t1.2 pacid=30792803 transcript=Cre12.g530900.t1.2
locus=Cre12.g530900 ID=Cre12.g530900.t1.2.v5.5 annot-version=v5.5
Chlamydomonas_reinhardtii OG0000016
Cre12.g547300.t1.1 pacid=30793076 transcript=Cre12.g547300.t1.1
locus=Cre12.g547300 ID=Cre12.g547300.t1.1.v5.5 annot-version=v5.5
Chlamydomonas_reinhardtii OG0000016
Cre12.g540650.t1.2 pacid=30793452 transcript=Cre12.g540650.t1.2
locus=Cre12.g540650 ID=Cre12.g540650.t1.2.v5.5 annot-version=v5.5
Chlamydomonas_reinhardtii OG0000016
Cre11.g467597.t1.1 pacid=30775994 transcript=Cre11.g467597.t1.1
locus=Cre11.g467597 ID=Cre11.g467597.t1.1.v5.5 annot-version=v5.5
Chlamydomonas_reinhardtii OG0000016
Cre16.g648700.t1.1 pacid=30777650 transcript=Cre16.g648700.t1.1
locus=Cre16.g648700 ID=Cre16.g648700.t1.1.v5.5 annot-version=v5.5
Chlamydomonas_reinhardtii OG0000016
Cre16.g693203.t1.1 pacid=30777665 transcript=Cre16.g693203.t1.1
locus=Cre16.g693203 ID=Cre16.g693203.t1.1.v5.5 annot-version=v5.5
Chlamydomonas_reinhardtii OG0000016
Cre16.g655450.t1.1 pacid=30777961 transcript=Cre16.g655450.t1.1
locus=Cre16.g655450 ID=Cre16.g655450.t1.1.v5.5 annot-version=v5.5
Chlamydomonas_reinhardtii OG0000016
Cre09.g395750.t1.1 pacid=30780533 transcript=Cre09.g395750.t1.1
locus=Cre09.g395750 ID=Cre09.g395750.t1.1.v5.5 annot-version=v5.5
Chlamydomonas_reinhardtii OG0000016
Cre17.g728400.t1.1 pacid=30781806 transcript=Cre17.g728400.t1.1
locus=Cre17.g728400 ID=Cre17.g728400.t1.1.v5.5 annot-version=v5.5
Chlamydomonas_reinhardtii OG0000016
Cre17.g717050.t1.1 pacid=30782048 transcript=Cre17.g717050.t1.1
locus=Cre17.g717050 ID=Cre17.g717050.t1.1.v5.5 annot-version=v5.5
Chlamydomonas_reinhardtii OG0000016
Cre17.g723500.t1.1 pacid=30782322 transcript=Cre17.g723500.t1.1
locus=Cre17.g723500 ID=Cre17.g723500.t1.1.v5.5 annot-version=v5.5
Chlamydomonas_reinhardtii OG0000016

Cre17.g743547.t1.1 pacid=30782472 transcript=Cre17.g743547.t1.1 locus=Cre17.g743547 ID=Cre17.g743547.t1.1.v5.5 annot-version=v5.5
Chlamydomonas_reinhardtii OG0000016
Vocar.0012s0255.1.p pacid=32894986 transcript=Vocar.0012s0255.1 locus=Vocar.0012s0255 ID=Vocar.0012s0255.1.v2.1 annot-version=v2.1
Volvox_carteri OG0000016
Vocar.0028s0079.1.p pacid=32895526 transcript=Vocar.0028s0079.1 locus=Vocar.0028s0079 ID=Vocar.0028s0079.1.v2.1 annot-version=v2.1
Volvox_carteri OG0000016
Vocar.0112s0001.1.p pacid=32896546 transcript=Vocar.0112s0001.1 locus=Vocar.0112s0001 ID=Vocar.0112s0001.1.v2.1 annot-version=v2.1
Volvox_carteri OG0000016
Vocar.0039s0095.1.p pacid=32897789 transcript=Vocar.0039s0095.1 locus=Vocar.0039s0095 ID=Vocar.0039s0095.1.v2.1 annot-version=v2.1
Volvox_carteri OG0000016
Vocar.0025s0063.1.p pacid=32898286 transcript=Vocar.0025s0063.1 locus=Vocar.0025s0063 ID=Vocar.0025s0063.1.v2.1 annot-version=v2.1
Volvox_carteri OG0000016
Vocar.0025s0126.1.p pacid=32898298 transcript=Vocar.0025s0126.1 locus=Vocar.0025s0126 ID=Vocar.0025s0126.1.v2.1 annot-version=v2.1
Volvox_carteri OG0000016
Vocar.0001s0928.1.p pacid=32884830 transcript=Vocar.0001s0928.1 locus=Vocar.0001s0928 ID=Vocar.0001s0928.1.v2.1 annot-version=v2.1
Volvox_carteri OG0000016
Vocar.0049s0028.1.p pacid=32886547 transcript=Vocar.0049s0028.1 locus=Vocar.0049s0028 ID=Vocar.0049s0028.1.v2.1 annot-version=v2.1
Volvox_carteri OG0000016
Vocar.0027s0127.1.p pacid=32887224 transcript=Vocar.0027s0127.1 locus=Vocar.0027s0127 ID=Vocar.0027s0127.1.v2.1 annot-version=v2.1
Volvox_carteri OG0000016
Vocar.0007s0183.1.p pacid=32888238 transcript=Vocar.0007s0183.1 locus=Vocar.0007s0183 ID=Vocar.0007s0183.1.v2.1 annot-version=v2.1
Volvox_carteri OG0000016
Vocar.0003s0045.1.p pacid=32889278 transcript=Vocar.0003s0045.1 locus=Vocar.0003s0045 ID=Vocar.0003s0045.1.v2.1 annot-version=v2.1
Volvox_carteri OG0000016
Vocar.0004s0263.1.p pacid=32889546 transcript=Vocar.0004s0263.1 locus=Vocar.0004s0263 ID=Vocar.0004s0263.1.v2.1 annot-version=v2.1
Volvox_carteri OG0000016
Vocar.0024s0210.1.p pacid=32890325 transcript=Vocar.0024s0210.1 locus=Vocar.0024s0210 ID=Vocar.0024s0210.1.v2.1 annot-version=v2.1
Volvox_carteri OG0000016
Vocar.0036s0047.1.p pacid=32892220 transcript=Vocar.0036s0047.1 locus=Vocar.0036s0047 ID=Vocar.0036s0047.1.v2.1 annot-version=v2.1
Volvox_carteri OG0000016
Vocar.0032s0141.1.p pacid=32893724 transcript=Vocar.0032s0141.1 locus=Vocar.0032s0141 ID=Vocar.0032s0141.1.v2.1 annot-version=v2.1
Volvox_carteri OG0000016
Vocar.0006s0105.1.p pacid=32893899 transcript=Vocar.0006s0105.1 locus=Vocar.0006s0105 ID=Vocar.0006s0105.1.v2.1 annot-version=v2.1
Volvox_carteri OG0000016
jgi|ChlNC64A_1|56833|estExt_fgenesh3_pg.C_30036
Chlorella_variabilis OG0000016
jgi|ChlNC64A_1|34091|estExt_Genewise1Plus.C_30330

Chlorella_variabilis   OG0000016
jgi|ChlNC64A_1|134819|IGS.gm_12_00205   Chlorella_variabilis
OG0000016
jgi|ChlNC64A_1|134989|IGS.gm_12_00375   Chlorella_variabilis
OG0000016
jgi|ChlNC64A_1|136987|IGS.gm_18_00093   Chlorella_variabilis
OG0000016
jgi|ChlNC64A_1|36538|estExt_Genewise1Plus.C_180132
Chlorella_variabilis   OG0000016
jgi|ChlNC64A_1|58610|estExt_fgenesh3_pg.C_180076
Chlorella_variabilis   OG0000016
jgi|ChlNC64A_1|137099|IGS.gm_18_00205   Chlorella_variabilis
OG0000016
jgi|ChlNC64A_1|56680|estExt_fgenesh3_pg.C_20094
Chlorella_variabilis   OG0000016
jgi|ChlNC64A_1|19411|e_gw1.2.140.1   Chlorella_variabilis
OG0000016
jgi|ChlNC64A_1|137957|IGS.gm_2_00424   Chlorella_variabilis
OG0000016
jgi|ChlNC64A_1|133292|IGS.gm_1_00428   Chlorella_variabilis
OG0000016
jgi|ChlNC64A_1|19034|e_gw1.2.634.1   Chlorella_variabilis
OG0000016
jgi|ChlNC64A_1|20690|e_gw1.4.4.1 Chlorella_variabilis
OG0000016
jgi|ChlNC64A_1|10394|gw1.7.97.1   Chlorella_variabilis
OG0000016
jgi|ChlNC64A_1|58132|estExt_fgenesh3_pg.C_130023
Chlorella_variabilis   OG0000016
jgi|ChlNC64A_1|32365|estExt_Genewise1.C_190192
Chlorella_variabilis   OG0000016
jgi|ChlNC64A_1|140061|IGS.gm_28_00092   Chlorella_variabilis
OG0000016
jgi|ChlNC64A_1|142571|IGS.gm_43_00003   Chlorella_variabilis
OG0000016
36047 pacid=27387246 transcript=36047 locus=fgenesh1_pm.5_#_146
ID=36047.2.0.227 annot-version=v2.0   Coccomyxa_subellipsoidea
OG0000016
20365 pacid=27387613 transcript=20365 locus=e_gw1.22.45.1
ID=20365.2.0.227 annot-version=v2.0   Coccomyxa_subellipsoidea
OG0000016
45222 pacid=27387750 transcript=45222 locus=fgenesh1_pg.22_#_107
ID=45222.2.0.227 annot-version=v2.0   Coccomyxa_subellipsoidea
OG0000016
16269 pacid=27388551 transcript=16269 locus=e_gw1.9.59.1
ID=16269.2.0.227 annot-version=v2.0   Coccomyxa_subellipsoidea
OG0000016
11258 pacid=27389277 transcript=11258 locus=e_gw1.1.9.1
ID=11258.2.0.227 annot-version=v2.0   Coccomyxa_subellipsoidea
OG0000016
27760 pacid=27390088 transcript=27760
locus=estExt_Genewise1Plus.C_40059 ID=27760.2.0.227 annot-
version=v2.0   Coccomyxa_subellipsoidea OG0000016
35453 pacid=27390722 transcript=35453 locus=fgenesh1_pm.3_#_128

ID=35453.2.0.227 annot-version=v2.0 Coccomyxa_subellipsoidea OG0000016
52664 pacid=27390784 transcript=52664 locus=estExt_fgenesh1_pm.C_30191 ID=52664.2.0.227 annot-version=v2.0 Coccomyxa_subellipsoidea OG0000016
14971 pacid=27391318 transcript=14971 locus=e_gw1.6.62.1 ID=14971.2.0.227 annot-version=v2.0 Coccomyxa_subellipsoidea OG0000016
53156 pacid=27391398 transcript=53156 locus=estExt_fgenesh1_pm.C_60033 ID=53156.2.0.227 annot-version=v2.0 Coccomyxa_subellipsoidea OG0000016
66913 pacid=27391735 transcript=66913 locus=estExt_Genemark1.C_120183 ID=66913.2.0.227 annot-version=v2.0 Coccomyxa_subellipsoidea OG0000016
31416 pacid=27392960 transcript=31416 locus=estExt_Genewise1Plus.C_200261 ID=31416.2.0.227 annot-version=v2.0 Coccomyxa_subellipsoidea OG0000016
1342 pacid=27393006 transcript=1342 locus=gw1.20.39.1 ID=1342.2.0.227 annot-version=v2.0 Coccomyxa_subellipsoidea OG0000016
39467 pacid=27393308 transcript=39467 locus=fgenesh1_pg.2_#_79 ID=39467.2.0.227 annot-version=v2.0 Coccomyxa_subellipsoidea OG0000016
64664 pacid=27393316 transcript=64664 locus=estExt_Genemark1.C_20531 ID=64664.2.0.227 annot-version=v2.0 Coccomyxa_subellipsoidea OG0000016
38676 pacid=27394169 transcript=38676 locus=fgenesh1_pm.25_#_1 ID=38676.2.0.227 annot-version=v2.0 Coccomyxa_subellipsoidea OG0000016
55049 pacid=27394795 transcript=55049 locus=estExt_fgenesh1_pm.C_290001 ID=55049.2.0.227 annot-version=v2.0 Coccomyxa_subellipsoidea OG0000016
48534 pacid=27394920 transcript=48534 locus=estExt_fgenesh1_pg.C_150111 ID=48534.2.0.227 annot-version=v2.0 Coccomyxa_subellipsoidea OG0000016
15214 pacid=27386597 transcript=15214 locus=e_gw1.7.351.1 ID=15214.2.0.227 annot-version=v2.0 Coccomyxa_subellipsoidea OG0000016
125_gw1.07.00.7.1 Ostreococcus_tauri OG0000016
16690_e_gw1.04.00.113.1 Ostreococcus_tauri OG0000016
19132_e_gw1.09.00.239.1 Ostreococcus_tauri OG0000016
19708_e_gw1.10.00.120.1 Ostreococcus_tauri OG0000016
24079_estExt_fgenesh1_pg.C_Chr_09.00010123 Ostreococcus_tauri OG0000016
32428_0400010374 Ostreococcus_tauri OG0000016
35135_1000010155 Ostreococcus_tauri OG0000016
36549_1400010200 Ostreococcus_tauri OG0000016
1025_gw1.05.00.55.1 Ostreococcus_tauri OG0000016
1261_gw1.10.00.71.1 Ostreococcus_tauri OG0000016
35308 pacid=27414362 transcript=35308 locus=e_gwEuk.7.21.1 ID=35308.2.0.231 annot-version=v2.0 Ostreococcus_lucimarinus OG0000016
14894 pacid=27415457 transcript=14894 locus=fgenesh1_pg.C_Chr_4000046 ID=14894.2.0.231 annot-version=v2.0

Ostreococcus_lucimarinus OG0000016
36892 pacid=27415668 transcript=36892 locus=e_gwEuk.4.155.1
ID=36892.2.0.231 annot-version=v2.0 Ostreococcus_lucimarinus
OG0000016
88422 pacid=27416118 transcript=88422 locus=ost_10_007_070
ID=88422.2.0.231 annot-version=v2.0 Ostreococcus_lucimarinus
OG0000016
38326 pacid=27416135 transcript=38326 locus=e_gwEuk.10.55.1
ID=38326.2.0.231 annot-version=v2.0 Ostreococcus_lucimarinus
OG0000016
94849 pacid=27416258 transcript=94849 locus=C_Chr_10000071
ID=94849.2.0.231 annot-version=v2.0 Ostreococcus_lucimarinus
OG0000016
39403 pacid=27417101 transcript=39403 locus=e_gwEuk.12.455.1
ID=39403.2.0.231 annot-version=v2.0 Ostreococcus_lucimarinus
OG0000016
1450 pacid=27418393 transcript=1450 locus=gwEuk.6.113.1
ID=1450.2.0.231 annot-version=v2.0 Ostreococcus_lucimarinus
OG0000016
992 pacid=27420095 transcript=992 locus=gwEuk.20.39.1 ID=992.2.0.231
annot-version=v2.0 Ostreococcus_lucimarinus OG0000016
207274 pacid=27348752 transcript=207314 locus=fgenesh1_pm.15_#_138
ID=207314.3.0.228 annot-version=v3.0 Micromonas_pusilla
OG0000016
52802 pacid=27339827 transcript=52802 locus=MicpuC2.EuGene.
0000130359 ID=52802.3.0.228 annot-version=v3.0
Micromonas_pusilla OG0000016
17033 pacid=27341307 transcript=17033 locus=MicpuC2.e_gw1.5.117.1
ID=17033.3.0.228 annot-version=v3.0 Micromonas_pusilla
OG0000016
121613 pacid=27339032 transcript=121653 locus=fgenesh3_kg.
8_#_25_#_isotig00710 ID=121653.3.0.228 annot-version=v3.0
Micromonas_pusilla OG0000016
45095 pacid=27343857 transcript=45095
locus=MicpuC2.fgenesh1_pm.C_scaffold_18000009 ID=45095.3.0.228
annot-version=v3.0 Micromonas_pusilla OG0000016
50503 pacid=27345696 transcript=50503 locus=MicpuC2.EuGene.
0000010727 ID=50503.3.0.228 annot-version=v3.0
Micromonas_pusilla OG0000016
46306 pacid=27345717 transcript=46306
locus=MicpuC2.estExt_fgenesh1_pg.C_10383 ID=46306.3.0.228 annot-
version=v3.0 Micromonas_pusilla OG0000016
49543 pacid=27346407 transcript=49543
locus=MicpuC2.estExt_fgenesh1_kg.C_40013 ID=49543.3.0.228 annot-
version=v3.0 Micromonas_pusilla OG0000016
184700 pacid=27347759 transcript=184740 locus=gm1.1821_g
ID=184740.3.0.228 annot-version=v3.0 Micromonas_pusilla
OG0000016
36543 pacid=27348450 transcript=36543
locus=MicpuC2.estExt_Genewise1Plus.C_150110 ID=36543.3.0.228 annot-
version=v3.0 Micromonas_pusilla OG0000016
62193 pacid=27397557 transcript=62193 locus=EuGene.1000010530
ID=62193.3.0.229 annot-version=v3.0 Micromonas_sp._RCC299
OG0000016

85720 pacid=27397765 transcript=85720 locus=e_gw2.10.71.1 ID=85720.3.0.229 annot-version=v3.0 Micromonas_sp._RCC299 OG0000016
75255 pacid=27398223 transcript=75255 locus=gw2.03.838.1 ID=75255.3.0.229 annot-version=v3.0 Micromonas_sp._RCC299 OG0000016
95143 pacid=27401447 transcript=95143 locus=estExt_Genewise2Plus.C_Chr_090649 ID=95143.3.0.229 annot-version=v3.0 Micromonas_sp._RCC299 OG0000016
87771 pacid=27402692 transcript=87771 locus=e_gw2.13.10.1 ID=87771.3.0.229 annot-version=v3.0 Micromonas_sp._RCC299 OG0000016
103799 pacid=27402834 transcript=103799 locus=fgenesh2_pg.C_Chr_13000172 ID=103799.3.0.229 annot-version=v3.0 Micromonas_sp._RCC299 OG0000016
98752 pacid=27396169 transcript=98752 locus=fgenesh2_pg.C_Chr_02000128 ID=98752.3.0.229 annot-version=v3.0 Micromonas_sp._RCC299 OG0000016
83467 pacid=27404549 transcript=83467 locus=e_gw2.07.273.1 ID=83467.3.0.229 annot-version=v3.0 Micromonas_sp._RCC299 OG0000016
59906 pacid=27404556 transcript=59906 locus=EuGene.0700010191 ID=59906.3.0.229 annot-version=v3.0 Micromonas_sp._RCC299 OG0000016
67033 pacid=27404562 transcript=67033 locus=gw2.07.129.1 ID=67033.3.0.229 annot-version=v3.0 Micromonas_sp._RCC299 OG0000016
kfl00361_0030_v1.1 Klebsormidium_flaccidum OG0000016
kfl00425_0010_v1.1 Klebsormidium_flaccidum OG0000016
kfl00521_0030_v1.1 Klebsormidium_flaccidum OG0000016
kfl00018_0500_v1.1 Klebsormidium_flaccidum OG0000016
kfl00574_0100_v1.1 Klebsormidium_flaccidum OG0000016
kfl00616_0050_v1.1 Klebsormidium_flaccidum OG0000016
kfl00820_0010_v1.1 Klebsormidium_flaccidum OG0000016
kfl00855_0010_v1.1 Klebsormidium_flaccidum OG0000016
kfl00923_0010_v1.1 Klebsormidium_flaccidum OG0000016
kfl00003_0100_v1.1 Klebsormidium_flaccidum OG0000016
kfl00033_0400_v1.1 Klebsormidium_flaccidum OG0000016
kfl00034_0085_v1.1 Klebsormidium_flaccidum OG0000016
kfl00050_0290_v1.1 Klebsormidium_flaccidum OG0000016
kfl00056_0220_v1.1 Klebsormidium_flaccidum OG0000016
kfl00078_0360_v1.1 Klebsormidium_flaccidum OG0000016
kfl00126_0120_v1.1 Klebsormidium_flaccidum OG0000016
kfl00129_0270_v1.1 Klebsormidium_flaccidum OG0000016
kfl00008_0560_v1.1 Klebsormidium_flaccidum OG0000016
kfl00149_0085_v1.1 Klebsormidium_flaccidum OG0000016
kfl00163_0100_v1.1 Klebsormidium_flaccidum OG0000016
kfl00175_0190_v1.1 Klebsormidium_flaccidum OG0000016
kfl00010_0550_v1.1 Klebsormidium_flaccidum OG0000016
kfl00263_0070_v1.1 Klebsormidium_flaccidum OG0000016
Pp3c15_22860V3.1.p pacid=32927509 transcript=Pp3c15_22860V3.1 locus=Pp3c15_22860 annot-version=v3.3 Physcomitrella_patens OG0000016
Pp3c15_17010V3.1.p pacid=32928001 transcript=Pp3c15_17010V3.1

locus=Pp3c15_17010 annot-version=v3.3   Physcomitrella_patens
OG0000016
Pp3c15_14490V3.1.p pacid=32928677 transcript=Pp3c15_14490V3.1
locus=Pp3c15_14490 annot-version=v3.3   Physcomitrella_patens
OG0000016
Pp3c2_34070V3.1.p pacid=32933032 transcript=Pp3c2_34070V3.1
locus=Pp3c2_34070 annot-version=v3.3   Physcomitrella_patens
OG0000016
Pp3c22_9340V3.1.p pacid=32902940 transcript=Pp3c22_9340V3.1
locus=Pp3c22_9340 annot-version=v3.3   Physcomitrella_patens
OG0000016
Pp3c19_18700V3.1.p pacid=32937836 transcript=Pp3c19_18700V3.1
locus=Pp3c19_18700 annot-version=v3.3   Physcomitrella_patens
OG0000016
Pp3c19_17390V3.1.p pacid=32938139 transcript=Pp3c19_17390V3.1
locus=Pp3c19_17390 annot-version=v3.3   Physcomitrella_patens
OG0000016
Pp3c3_33380V3.1.p pacid=32942156 transcript=Pp3c3_33380V3.1
locus=Pp3c3_33380 annot-version=v3.3   Physcomitrella_patens
OG0000016
Pp3c3_34730V3.1.p pacid=32944272 transcript=Pp3c3_34730V3.1
locus=Pp3c3_34730 annot-version=v3.3   Physcomitrella_patens
OG0000016
Pp3c20_6990V3.1.p pacid=32947813 transcript=Pp3c20_6990V3.1
locus=Pp3c20_6990 annot-version=v3.3   Physcomitrella_patens
OG0000016
Pp3c22_15560V3.1.p pacid=32904488 transcript=Pp3c22_15560V3.1
locus=Pp3c22_15560 annot-version=v3.3   Physcomitrella_patens
OG0000016
Pp3c27_460V3.1.p pacid=32951784 transcript=Pp3c27_460V3.1
locus=Pp3c27_460 annot-version=v3.3   Physcomitrella_patens
OG0000016
Pp3c22_15570V3.1.p pacid=32904869 transcript=Pp3c22_15570V3.1
locus=Pp3c22_15570 annot-version=v3.3   Physcomitrella_patens
OG0000016
Pp3c5_3240V3.1.p pacid=32953715 transcript=Pp3c5_3240V3.1
locus=Pp3c5_3240 annot-version=v3.3   Physcomitrella_patens
OG0000016
Pp3c5_3340V3.1.p pacid=32953921 transcript=Pp3c5_3340V3.1
locus=Pp3c5_3340 annot-version=v3.3   Physcomitrella_patens
OG0000016
Pp3c5_23360V3.1.p pacid=32954685 transcript=Pp3c5_23360V3.1
locus=Pp3c5_23360 annot-version=v3.3   Physcomitrella_patens
OG0000016
Pp3c5_15980V3.1.p pacid=32955254 transcript=Pp3c5_15980V3.1
locus=Pp3c5_15980 annot-version=v3.3   Physcomitrella_patens
OG0000016
Pp3c5_16290V3.1.p pacid=32955339 transcript=Pp3c5_16290V3.1
locus=Pp3c5_16290 annot-version=v3.3   Physcomitrella_patens
OG0000016
Pp3c11_25800V3.1.p pacid=32956647 transcript=Pp3c11_25800V3.1
locus=Pp3c11_25800 annot-version=v3.3   Physcomitrella_patens
OG0000016
Pp3c14_1930V3.1.p pacid=32961721 transcript=Pp3c14_1930V3.1

locus=Pp3c14_1930 annot-version=v3.3	Physcomitrella_patens
OG0000016
Pp3c14_11830V3.1.p pacid=32962281 transcript=Pp3c14_11830V3.1
locus=Pp3c14_11830 annot-version=v3.3	Physcomitrella_patens
OG0000016
Pp3c8_1580V3.1.p pacid=32964956 transcript=Pp3c8_1580V3.1
locus=Pp3c8_1580 annot-version=v3.3	Physcomitrella_patens
OG0000016
Pp3c8_17020V3.1.p pacid=32964961 transcript=Pp3c8_17020V3.1
locus=Pp3c8_17020 annot-version=v3.3	Physcomitrella_patens
OG0000016
Pp3c1_25070V3.1.p pacid=32966763 transcript=Pp3c1_25070V3.1
locus=Pp3c1_25070 annot-version=v3.3	Physcomitrella_patens
OG0000016
Pp3c17_18730V3.1.p pacid=32906284 transcript=Pp3c17_18730V3.1
locus=Pp3c17_18730 annot-version=v3.3	Physcomitrella_patens
OG0000016
Pp3c1_17220V3.1.p pacid=32970251 transcript=Pp3c1_17220V3.1
locus=Pp3c1_17220 annot-version=v3.3	Physcomitrella_patens
OG0000016
Pp3c12_11190V3.1.p pacid=32973466 transcript=Pp3c12_11190V3.1
locus=Pp3c12_11190 annot-version=v3.3	Physcomitrella_patens
OG0000016
Pp3c12_17520V3.1.p pacid=32974581 transcript=Pp3c12_17520V3.1
locus=Pp3c12_17520 annot-version=v3.3	Physcomitrella_patens
OG0000016
Pp3c6_8850V3.1.p pacid=32975938 transcript=Pp3c6_8850V3.1
locus=Pp3c6_8850 annot-version=v3.3	Physcomitrella_patens
OG0000016
Pp3c6_10410V3.1.p pacid=32976632 transcript=Pp3c6_10410V3.1
locus=Pp3c6_10410 annot-version=v3.3	Physcomitrella_patens
OG0000016
Pp3c6_6330V3.1.p pacid=32978383 transcript=Pp3c6_6330V3.1
locus=Pp3c6_6330 annot-version=v3.3	Physcomitrella_patens
OG0000016
Pp3c18_7000V3.1.p pacid=32981101 transcript=Pp3c18_7000V3.1
locus=Pp3c18_7000 annot-version=v3.3	Physcomitrella_patens
OG0000016
Pp3c18_7010V3.1.p pacid=32981916 transcript=Pp3c18_7010V3.1
locus=Pp3c18_7010 annot-version=v3.3	Physcomitrella_patens
OG0000016
Pp3c18_21520V3.1.p pacid=32982251 transcript=Pp3c18_21520V3.1
locus=Pp3c18_21520 annot-version=v3.3	Physcomitrella_patens
OG0000016
Pp3c18_12400V3.1.p pacid=32982373 transcript=Pp3c18_12400V3.1
locus=Pp3c18_12400 annot-version=v3.3	Physcomitrella_patens
OG0000016
Pp3c18_21510V3.1.p pacid=32983096 transcript=Pp3c18_21510V3.1
locus=Pp3c18_21510 annot-version=v3.3	Physcomitrella_patens
OG0000016
Pp3c16_13851V3.1.p pacid=32984392 transcript=Pp3c16_13851V3.1
locus=Pp3c16_13851 annot-version=v3.3	Physcomitrella_patens
OG0000016
Pp3c16_9600V3.1.p pacid=32986331 transcript=Pp3c16_9600V3.1

locus=Pp3c16_9600 annot-version=v3.3   Physcomitrella_patens
OG0000016
Pp3c16_13830V3.1.p pacid=32986513 transcript=Pp3c16_13830V3.1
locus=Pp3c16_13830 annot-version=v3.3   Physcomitrella_patens
OG0000016
Pp3c9_19220V3.1.p pacid=32911319 transcript=Pp3c9_19220V3.1
locus=Pp3c9_19220 annot-version=v3.3   Physcomitrella_patens
OG0000016
Pp3c9_18880V3.1.p pacid=32913007 transcript=Pp3c9_18880V3.1
locus=Pp3c9_18880 annot-version=v3.3   Physcomitrella_patens
OG0000016
Pp3c10_22200V3.1.p pacid=32900982 transcript=Pp3c10_22200V3.1
locus=Pp3c10_22200 annot-version=v3.3   Physcomitrella_patens
OG0000016
Pp3c10_25760V3.1.p pacid=32901098 transcript=Pp3c10_25760V3.1
locus=Pp3c10_25760 annot-version=v3.3   Physcomitrella_patens
OG0000016
Pp3c26_3590V3.1.p pacid=32917297 transcript=Pp3c26_3590V3.1
locus=Pp3c26_3590 annot-version=v3.3   Physcomitrella_patens
OG0000016
Pp3c4_2380V3.1.p pacid=32919743 transcript=Pp3c4_2380V3.1
locus=Pp3c4_2380 annot-version=v3.3   Physcomitrella_patens
OG0000016
Pp3c4_6310V3.1.p pacid=32921132 transcript=Pp3c4_6310V3.1
locus=Pp3c4_6310 annot-version=v3.3   Physcomitrella_patens
OG0000016
AT2G36380.1 | pleiotropic drug resistance 6 | Chr2:15257583-15263627
FORWARD LENGTH=1453 | 201606   Arabidopsis_thaliana
OG0000016
AT2G37280.1 | pleiotropic drug resistance 5 | Chr2:15650400-15656417
FORWARD LENGTH=1413 | 201606   Arabidopsis_thaliana
OG0000016
AT2G37360.1 | ABC-2 type transporter family protein |
Chr2:15673555-15675822 REVERSE LENGTH=755 | 201606
Arabidopsis_thaliana   OG0000016
AT2G39350.1 | ABC-2 type transporter family protein |
Chr2:16430174-16432396 REVERSE LENGTH=740 | 201606
Arabidopsis_thaliana   OG0000016
AT3G13220.1 | ABC-2 type transporter family protein |
Chr3:4247968-4250703 REVERSE LENGTH=685 | 201606
Arabidopsis_thaliana   OG0000016
AT3G16340.1 | pleiotropic drug resistance 1 | Chr3:5539897-5546263
FORWARD LENGTH=1416 | 201606   Arabidopsis_thaliana
OG0000016
AT3G21090.1 | ABC-2 type transporter family protein |
Chr3:7391497-7394933 REVERSE LENGTH=691 | 201606
Arabidopsis_thaliana   OG0000016
AT3G25620.1 | ABC-2 type transporter family protein |
Chr3:9317513-9319505 REVERSE LENGTH=467 | 201606
Arabidopsis_thaliana   OG0000016
AT3G30842.1 | pleiotropic drug resistance 10 |
Chr3:12593959-12600432 REVERSE LENGTH=1418 | 201606
Arabidopsis_thaliana   OG0000016
AT1G15210.1 | pleiotropic drug resistance 7 | Chr1:5231552-5236573

REVERSE LENGTH=1442 | 201606	Arabidopsis_thaliana
OG0000016
AT1G15520.1 | pleiotropic drug resistance 12 | Chr1:5331993-5338175
REVERSE LENGTH=1423 | 201606	Arabidopsis_thaliana
OG0000016
AT3G52310.1 | ABC transporter G family member 27 |
Chr3:19399372-19402861 FORWARD LENGTH=743 | 201606
Arabidopsis_thaliana	OG0000016
AT3G53480.1 | pleiotropic drug resistance 9 | Chr3:19825366-19831644
FORWARD LENGTH=1450 | 201606	Arabidopsis_thaliana
OG0000016
AT3G53510.1 | ABC-2 type transporter family protein |
Chr3:19837302-19839521 REVERSE LENGTH=739 | 201606
Arabidopsis_thaliana	OG0000016
AT3G55090.1 | ABC-2 type transporter family protein |
Chr3:20416342-20418552 REVERSE LENGTH=736 | 201606
Arabidopsis_thaliana	OG0000016
AT3G55100.1 | ABC-2 type transporter family protein |
Chr3:20420352-20422340 REVERSE LENGTH=662 | 201606
Arabidopsis_thaliana	OG0000016
AT3G55110.1 | ABC-2 type transporter family protein |
Chr3:20424766-20426892 REVERSE LENGTH=708 | 201606
Arabidopsis_thaliana	OG0000016
AT3G55130.1 | white-brown complex homolog 19 |
Chr3:20434111-20436288 REVERSE LENGTH=725 | 201606
Arabidopsis_thaliana	OG0000016
AT1G17840.1 | white-brown complex-like protein |
Chr1:6142870-6145894 FORWARD LENGTH=703 | 201606
Arabidopsis_thaliana	OG0000016
AT4G15215.1 | pleiotropic drug resistance 13 | Chr4:8672070-8678874
FORWARD LENGTH=1390 | 201606	Arabidopsis_thaliana
OG0000016
AT4G15230.1 | pleiotropic drug resistance 2 | Chr4:8680295-8686880
FORWARD LENGTH=1400 | 201606	Arabidopsis_thaliana
OG0000016
AT4G15233.1 | ABC-2 and Plant PDR ABC-type transporter family
protein | Chr4:8688322-8694432 FORWARD LENGTH=1392 | 201606
Arabidopsis_thaliana	OG0000016
AT4G15236.1 | ABC-2 and Plant PDR ABC-type transporter family
protein | Chr4:8696677-8702727 FORWARD LENGTH=1390 | 201606
Arabidopsis_thaliana	OG0000016
AT4G25750.1 | ABC-2 type transporter family protein |
Chr4:13110627-13112360 REVERSE LENGTH=577 | 201606
Arabidopsis_thaliana	OG0000016
AT4G27420.1 | ABC-2 type transporter family protein |
Chr4:13712434-13714797 REVERSE LENGTH=638 | 201606
Arabidopsis_thaliana	OG0000016
AT5G06530.1 | ABC-2 type transporter family protein |
Chr5:1990060-1994605 REVERSE LENGTH=751 | 201606
Arabidopsis_thaliana	OG0000016
AT5G13580.1 | ABC-2 type transporter family protein |
Chr5:4370879-4373062 FORWARD LENGTH=727 | 201606
Arabidopsis_thaliana	OG0000016
AT5G19410.1 | ABC-2 type transporter family protein |

Chr5:6545237-6547111 REVERSE LENGTH=624 | 201606
Arabidopsis_thaliana OG0000016
AT5G52860.1 | ABC-2 type transporter family protein |
Chr5:21419776-21421545 REVERSE LENGTH=589 | 201606
Arabidopsis_thaliana OG0000016
AT1G31770.1 | ATP-binding cassette 14 | Chr1:11375252-11377644
REVERSE LENGTH=648 | 201606 Arabidopsis_thaliana
OG0000016
AT1G51460.1 | ABC-2 type transporter family protein |
Chr1:19077132-19081335 REVERSE LENGTH=678 | 201606
Arabidopsis_thaliana OG0000016
AT1G51500.1 | ABC-2 type transporter family protein |
Chr1:19097967-19100972 REVERSE LENGTH=687 | 201606
Arabidopsis_thaliana OG0000016
AT1G53270.1 | ABC-2 type transporter family protein |
Chr1:19862878-19864650 FORWARD LENGTH=590 | 201606
Arabidopsis_thaliana OG0000016
AT1G59870.1 | ABC-2 and Plant PDR ABC-type transporter family
protein | Chr1:22034661-22039844 FORWARD LENGTH=1469 | 201606
Arabidopsis_thaliana OG0000016
AT1G66950.1 | pleiotropic drug resistance 11 |
Chr1:24978239-24984461 FORWARD LENGTH=1454 | 201606
Arabidopsis_thaliana OG0000016
AT1G71960.1 | ATP-binding casette family G25 |
Chr1:27082587-27088163 REVERSE LENGTH=662 | 201606
Arabidopsis_thaliana OG0000016
AT2G01320.1 | ABC-2 type transporter family protein |
Chr2:154669-158063 REVERSE LENGTH=725 | 201606
Arabidopsis_thaliana OG0000016
AT2G13610.1 | ABC-2 type transporter family protein |
Chr2:5673827-5675776 REVERSE LENGTH=649 | 201606
Arabidopsis_thaliana OG0000016
AT2G26910.1 | pleiotropic drug resistance 4 | Chr2:11481623-11487874
FORWARD LENGTH=1420 | 201606 Arabidopsis_thaliana
OG0000016
AT2G28070.1 | ABC-2 type transporter family protein |
Chr2:11956432-11959782 FORWARD LENGTH=730 | 201606
Arabidopsis_thaliana OG0000016
AT2G29940.1 | pleiotropic drug resistance 3 | Chr2:12760139-12766455
FORWARD LENGTH=1426 | 201606 Arabidopsis_thaliana
OG0000016
Os03t0281900-01 ATP-binding cassette (ABC) transporter, Hypodermal
suberization of roots, Salt stress tolerance Oryza_sativa
OG0000016
Os03t0282100-00 Similar to ATPase, coupled to transmembrane movement
of substances. Oryza_sativa OG0000016
Os03t0859500-01 ABC transporter-like domain containing protein.
Oryza_sativa OG0000016
Os04t0528300-01 Similar to ABC transporter. Oryza_sativa
OG0000016
Os01t0342750-01 Hypothetical conserved gene. Oryza_sativa
OG0000016
Os05t0120000-00 Similar to Stigma/style ABC transporter.
Oryza_sativa OG0000016

Os05t0120200-01 Similar to ATPase, coupled to transmembrane movement of substances.	Oryza_sativa	OG0000016
Os05t0222200-01 ABC transporter-like domain containing protein.	Oryza_sativa	OG0000016
Os05t0384600-01 ABC transporter-like domain containing protein.	Oryza_sativa	OG0000016
Os01t0121700-01 ABC transporter-like domain containing protein.	Oryza_sativa	OG0000016
Os01t0516900-00 ABC transporter-like domain containing protein.	Oryza_sativa	OG0000016
Os06t0503100-01 ABC-2 type transporter domain containing protein.	Oryza_sativa	OG0000016
Os06t0554800-01 Similar to ABC transporter (PDR5-like) isolog (PDR1 ABC transporter).	Oryza_sativa	OG0000016
Os06t0607700-01 ATP-binding cassette (ABC) transporter, Post-meiotic anther and pollen development	Oryza_sativa	OG0000016
Os07t0288700-01 Similar to white-brown-complex ABC transporter family.	Oryza_sativa	OG0000016
Os01t0609000-00 Similar to Pleiotropic drug resistance protein 3.	Oryza_sativa	OG0000016
Os01t0609200-00 Similar to Pleiotropic drug resistance protein 3.	Oryza_sativa	OG0000016
Os01t0609300-01 Similar to Pleiotropic drug resistance protein 3.	Oryza_sativa	OG0000016
Os01t0609900-01 Similar to Pleiotropic drug resistance protein 4.	Oryza_sativa	OG0000016
Os07t0522500-01 Similar to PDR6 ABC transporter.	Oryza_sativa	OG0000016
Os01t0615500-00 ABC transporter-like domain containing protein.	Oryza_sativa	OG0000016
Os08t0167000-01 ABC transporter-like domain containing protein.	Oryza_sativa	OG0000016
Os08t0384500-01 ABC transporter-like domain containing protein.	Oryza_sativa	OG0000016
Os08t0544400-01 ABC-2 type transporter domain containing protein.	Oryza_sativa	OG0000016
Os09t0125900-01 Similar to PDR15 ABC transporter.	Oryza_sativa	OG0000016
Os09t0250800-00 Similar to ABC-2 type transporter family protein.	Oryza_sativa	OG0000016
Os09t0332300-00 Similar to PDR20.	Oryza_sativa	OG0000016
Os09t0332700-01 Similar to PDR-type ABC transporter 2 (Fragment).	Oryza_sativa	OG0000016
Os09t0333000-00 ABC transporter-like domain containing protein.	Oryza_sativa	OG0000016
Os09t0333500-01 ABC transporter-like domain containing protein.	Oryza_sativa	OG0000016
Os09t0333600-01 Similar to PDR-like ABC transporter (PDR4 ABC transporter).	Oryza_sativa	OG0000016
Os09t0401100-01 Half-size ABC transporter, Mycorrhizal arbuscule formation	Oryza_sativa	OG0000016
Os09t0472100-01 Similar to ABC transporter.	Oryza_sativa	OG0000016
Os09t0472200-00 Similar to white-brown-complex ABC transporter

family.	Oryza_sativa	OG0000016
Os10t0163290-00 Similar to ABC-2 type transporter family protein.	Oryza_sativa	OG0000016
Os10t0205500-01 Similar to PDR3 ABC transporter.	Oryza_sativa	OG0000016
Os10t0494300-01 ATP binding cassette G transporter, Regulation of male reproduction, Anther cuticle development	Oryza_sativa	OG0000016
Os11t0177400-01 Similar to ATPase, coupled to transmembrane movement of substances.	Oryza_sativa	OG0000016
Os11t0587600-01 Similar to PDR-like ABC transporter.	Oryza_sativa	OG0000016
Os12t0409700-00 Hypothetical conserved gene.	Oryza_sativa	OG0000016
Os12t0411700-01 ABC transporter-like domain containing protein.	Oryza_sativa	OG0000016
Os12t0512700-01 ABC transporter-like domain containing protein.	Oryza_sativa	OG0000016
Os01t0836600-01 ABC transporter-like domain containing protein.	Oryza_sativa	OG0000016
Os01t0177900-01 ATP-binding cassette transporter, Cuticle formation, Defense response	Oryza_sativa	OG0000016
Os02t0318450-00 ABC transporter-like domain containing protein.	Oryza_sativa	OG0000016
Os02t0318500-00 ABC transporter-like domain containing protein.	Oryza_sativa	OG0000016
Os03t0157400-01 ABC transporter-like domain containing protein.	Oryza_sativa	OG0000016
CMN295CT pep chromosome:ASM9120v1:14:724792:727317:1 gene:CMN295C transcript:CMN295CT gene_biotype:protein_coding transcript_biotype:protein_coding	Cyanidioschyzon_merolae	OG0000016
CMS467CT pep chromosome:ASM9120v1:19:1172402:1174453:1 gene:CMS467C transcript:CMS467CT gene_biotype:protein_coding transcript_biotype:protein_coding	Cyanidioschyzon_merolae	OG0000016
CMI088CT pep chromosome:ASM9120v1:9:252622:254599:-1 gene:CMI088C transcript:CMI088CT gene_biotype:protein_coding transcript_biotype:protein_coding	Cyanidioschyzon_merolae	OG0000016
CMD134CT pep chromosome:ASM9120v1:4:348843:351132:-1 gene:CMD134C transcript:CMD134CT gene_biotype:protein_coding transcript_biotype:protein_coding	Cyanidioschyzon_merolae	OG0000016
CMO130CT pep chromosome:ASM9120v1:15:324898:326922:1 gene:CMO130C transcript:CMO130CT gene_biotype:protein_coding transcript_biotype:protein_coding description:ABC-2 type transporter family protein [Source:Projected from Arabidopsis thaliana (AT2G01320) TAIR;Acc:AT2G01320]	Cyanidioschyzon_merolae	OG0000016
CMD135CT pep chromosome:ASM9120v1:4:351707:354184:1 gene:CMD135C transcript:CMD135CT gene_biotype:protein_coding transcript_biotype:protein_coding	Cyanidioschyzon_merolae	OG0000016

CMR291CT pep chromosome:ASM9120v1:18:701238:703302:-1 gene:CMR291C transcript:CMR291CT gene_biotype:protein_coding transcript_biotype:protein_coding Cyanidioschyzon_merolae OG0000016
evm.model.contig_2112.2 Porphyridium_purpureum   OG0000016
evm.model.contig_2187.6 Porphyridium_purpureum   OG0000016
evm.model.contig_2196.1 Porphyridium_purpureum   OG0000016
evm.model.contig_2197.1 Porphyridium_purpureum   OG0000016
evm.model.contig_2361.1 Porphyridium_purpureum   OG0000016
evm.model.contig_2405.1 Porphyridium_purpureum   OG0000016
evm.model.contig_3596.1 Porphyridium_purpureum   OG0000016
evm.model.contig_440.1   Porphyridium_purpureum   OG0000016
evm.model.contig_4430.1 Porphyridium_purpureum   OG0000016
CDF41434 pep supercontig:ASM35022v2:HG002379:300025:301952:-1 gene:CHC_T00008014001 transcript:CDF41434 gene_biotype:protein_coding transcript_biotype:protein_coding Chondrus_crispus OG0000016
CDF34030 pep supercontig:ASM35022v2:HG001669:110386:112658:-1 gene:CHC_T00002695001 transcript:CDF34030 gene_biotype:protein_coding transcript_biotype:protein_coding Chondrus_crispus OG0000016
CDF32154 pep supercontig:ASM35022v2:HG001459:365538:367856:-1 gene:CHC_T00001383001 transcript:CDF32154 gene_biotype:protein_coding transcript_biotype:protein_coding Chondrus_crispus OG0000016
CDF34514 pep supercontig:ASM35022v2:HG001695:123061:127339:-1 gene:CHC_T00002879001 transcript:CDF34514 gene_biotype:protein_coding transcript_biotype:protein_coding Chondrus_crispus OG0000016
CDF40597 pep supercontig:ASM35022v2:HG002211:1907:4823:-1 gene:CHC_T00000820001 transcript:CDF40597 gene_biotype:protein_coding transcript_biotype:protein_coding Chondrus_crispus OG0000016
g773.t1 Caulerpa_lentillifera   OG0000017
Cre10.g466526.t1.1 pacid=30789994 transcript=Cre10.g466526.t1.1 locus=Cre10.g466526 ID=Cre10.g466526.t1.1.v5.5 annot-version=v5.5 Chlamydomonas_reinhardtii OG0000017
Cre10.g466700.t1.1 pacid=30790846 transcript=Cre10.g466700.t1.1 locus=Cre10.g466700 ID=Cre10.g466700.t1.1.v5.5 annot-version=v5.5 Chlamydomonas_reinhardtii OG0000017
Cre09.g403550.t1.2 pacid=30781069 transcript=Cre09.g403550.t1.2 locus=Cre09.g403550 ID=Cre09.g403550.t1.2.v5.5 annot-version=v5.5 Chlamydomonas_reinhardtii OG0000017
Vocar.0029s0083.1.p pacid=32887285 transcript=Vocar.0029s0083.1 locus=Vocar.0029s0083 ID=Vocar.0029s0083.1.v2.1 annot-version=v2.1 Volvox_carteri   OG0000017
jgi|ChlNC64A_1|36779|estExt_Genewise1Plus.C_210112 Chlorella_variabilis   OG0000017
jgi|ChlNC64A_1|22157|e_gw1.7.96.1 Chlorella_variabilis OG0000017
jgi|ChlNC64A_1|21158|e_gw1.5.247.1   Chlorella_variabilis OG0000017
jgi|ChlNC64A_1|9789|gw1.28.19.1   Chlorella_variabilis OG0000017

jgi|ChlNC64A_1|10090|gw1.43.12.1 Chlorella_variabilis
OG0000017
jgi|ChlNC64A_1|143376|IGS.gm_530_00002   Chlorella_variabilis
OG0000017
52967 pacid=27387073 transcript=52967
locus=estExt_fgenesh1_pm.C_50029 ID=52967.2.0.227 annot-version=v2.0
        Coccomyxa_subellipsoidea OG0000017
54671 pacid=27388270 transcript=54671
locus=estExt_fgenesh1_pm.C_190064 ID=54671.2.0.227 annot-
version=v2.0   Coccomyxa_subellipsoidea OG0000017
44697 pacid=27388377 transcript=44697 locus=fgenesh1_pg.19_#_79
ID=44697.2.0.227 annot-version=v2.0   Coccomyxa_subellipsoidea
OG0000017
42466 pacid=27388645 transcript=42466 locus=fgenesh1_pg.9_#_252
ID=42466.2.0.227 annot-version=v2.0   Coccomyxa_subellipsoidea
OG0000017
35371 pacid=27390323 transcript=35371 locus=fgenesh1_pm.3_#_46
ID=35371.2.0.227 annot-version=v2.0   Coccomyxa_subellipsoidea
OG0000017
67043 pacid=27386134 transcript=67043
locus=estExt_Genemark1.C_130050 ID=67043.2.0.227 annot-version=v2.0
Coccomyxa_subellipsoidea OG0000017
56501 pacid=27391930 transcript=56501 locus=Genemark1.5530_g
ID=56501.2.0.227 annot-version=v2.0   Coccomyxa_subellipsoidea
OG0000017
5329 pacid=27386394 transcript=5329 locus=gw1.23.99.1
ID=5329.2.0.227 annot-version=v2.0   Coccomyxa_subellipsoidea
OG0000017
20337_e_gw1.12.00.235.1 Ostreococcus_tauri   OG0000017
20837_e_gw1.13.00.313.1 Ostreococcus_tauri   OG0000017
36774 pacid=27415192 transcript=36774 locus=e_gwEuk.21.144.1
ID=36774.2.0.231 annot-version=v2.0   Ostreococcus_lucimarinus
OG0000017
31624 pacid=27416682 transcript=31624 locus=eugene.0500010161
ID=31624.2.0.231 annot-version=v2.0   Ostreococcus_lucimarinus
OG0000017
17574 pacid=27417168 transcript=17574
locus=fgenesh1_pg.C_Chr_12000106 ID=17574.2.0.231 annot-version=v2.0
        Ostreococcus_lucimarinus OG0000017
39543 pacid=27417981 transcript=39543 locus=e_gwEuk.1.406.1
ID=39543.2.0.231 annot-version=v2.0   Ostreococcus_lucimarinus
OG0000017
51291 pacid=27418762 transcript=51291
locus=estExt_Genewise_ext.C_Chr_130364 ID=51291.2.0.231 annot-
version=v2.0   Ostreococcus_lucimarinus OG0000017
53678 pacid=27348686 transcript=53678 locus=MicpuC2.EuGene.
0000150353 ID=53678.3.0.228 annot-version=v3.0
Micromonas_pusilla   OG0000017
44274 pacid=27341480 transcript=44274
locus=MicpuC2.fgenesh1_pm.C_scaffold_5000033 ID=44274.3.0.228 annot-
version=v3.0   Micromonas_pusilla   OG0000017
188515 pacid=27344101 transcript=188555 locus=gm1.5636_g
ID=188555.3.0.228 annot-version=v3.0   Micromonas_pusilla
OG0000017

188623 pacid=27344457 transcript=188663 locus=gm1.5744_g
ID=188663.3.0.228 annot-version=v3.0	Micromonas_pusilla
OG0000017
56736 pacid=27398126 transcript=56736 locus=EuGene.0300010164
ID=56736.3.0.229 annot-version=v3.0	Micromonas_sp._RCC299
OG0000017
56773 pacid=27398455 transcript=56773 locus=EuGene.0300010201
ID=56773.3.0.229 annot-version=v3.0	Micromonas_sp._RCC299
OG0000017
79568 pacid=27398681 transcript=79568 locus=e_gw2.03.473.1
ID=79568.3.0.229 annot-version=v3.0	Micromonas_sp._RCC299
OG0000017
61394 pacid=27401418 transcript=61394 locus=EuGene.0900010377
ID=61394.3.0.229 annot-version=v3.0	Micromonas_sp._RCC299
OG0000017
59237 pacid=27402320 transcript=59237 locus=EuGene.0600010239
ID=59237.3.0.229 annot-version=v3.0	Micromonas_sp._RCC299
OG0000017
59138 pacid=27402475 transcript=59138 locus=EuGene.0600010140
ID=59138.3.0.229 annot-version=v3.0	Micromonas_sp._RCC299
OG0000017
101662 pacid=27404335 transcript=101662
locus=fgenesh2_pg.C_Chr_07000406 ID=101662.3.0.229 annot-
version=v3.0	Micromonas_sp._RCC299	OG0000017
kfl00347_0030_v1.1	Klebsormidium_flaccidum	OG0000017
kfl00517_0060_v1.1	Klebsormidium_flaccidum	OG0000017
kfl00531_0080_v1.1	Klebsormidium_flaccidum	OG0000017
kfl00090_0310_v1.1	Klebsormidium_flaccidum	OG0000017
kfl00114_0020_v1.1	Klebsormidium_flaccidum	OG0000017
Pp3c15_23510V3.1.p pacid=32927784 transcript=Pp3c15_23510V3.1
locus=Pp3c15_23510 annot-version=v3.3	Physcomitrella_patens
OG0000017
Pp3c13_10330V3.1.p pacid=32931157 transcript=Pp3c13_10330V3.1
locus=Pp3c13_10330 annot-version=v3.3	Physcomitrella_patens
OG0000017
Pp3c13_6170V3.1.p pacid=32931677 transcript=Pp3c13_6170V3.1
locus=Pp3c13_6170 annot-version=v3.3	Physcomitrella_patens
OG0000017
Pp3c2_25730V3.1.p pacid=32933611 transcript=Pp3c2_25730V3.1
locus=Pp3c2_25730 annot-version=v3.3	Physcomitrella_patens
OG0000017
Pp3c2_890V3.1.p pacid=32935268 transcript=Pp3c2_890V3.1
locus=Pp3c2_890 annot-version=v3.3	Physcomitrella_patens
OG0000017
Pp3c19_20510V3.1.p pacid=32939459 transcript=Pp3c19_20510V3.1
locus=Pp3c19_20510 annot-version=v3.3	Physcomitrella_patens
OG0000017
Pp3c3_32410V3.1.p pacid=32941392 transcript=Pp3c3_32410V3.1
locus=Pp3c3_32410 annot-version=v3.3	Physcomitrella_patens
OG0000017
Pp3c20_21820V3.1.p pacid=32945791 transcript=Pp3c20_21820V3.1
locus=Pp3c20_21820 annot-version=v3.3	Physcomitrella_patens
OG0000017
Pp3c20_21833V3.1.p pacid=32946931 transcript=Pp3c20_21833V3.1

locus=Pp3c20_21833 annot-version=v3.3   Physcomitrella_patens
OG0000017
Pp3c27_4820V3.1.p pacid=32951352 transcript=Pp3c27_4820V3.1
locus=Pp3c27_4820 annot-version=v3.3   Physcomitrella_patens
OG0000017
Pp3c27_4790V3.1.p pacid=32951548 transcript=Pp3c27_4790V3.1
locus=Pp3c27_4790 annot-version=v3.3   Physcomitrella_patens
OG0000017
Pp3c5_4920V3.1.p pacid=32953109 transcript=Pp3c5_4920V3.1
locus=Pp3c5_4920 annot-version=v3.3   Physcomitrella_patens
OG0000017
Pp3c5_2410V3.1.p pacid=32953213 transcript=Pp3c5_2410V3.1
locus=Pp3c5_2410 annot-version=v3.3   Physcomitrella_patens
OG0000017
Pp3c5_23450V3.1.p pacid=32955126 transcript=Pp3c5_23450V3.1
locus=Pp3c5_23450 annot-version=v3.3   Physcomitrella_patens
OG0000017
Pp3c8_3050V3.1.p pacid=32965689 transcript=Pp3c8_3050V3.1
locus=Pp3c8_3050 annot-version=v3.3   Physcomitrella_patens
OG0000017
Pp3c1_31910V3.1.p pacid=32967683 transcript=Pp3c1_31910V3.1
locus=Pp3c1_31910 annot-version=v3.3   Physcomitrella_patens
OG0000017
Pp3c1_41820V3.1.p pacid=32969183 transcript=Pp3c1_41820V3.1
locus=Pp3c1_41820 annot-version=v3.3   Physcomitrella_patens
OG0000017
Pp3c1_540V3.1.p pacid=32971803 transcript=Pp3c1_540V3.1
locus=Pp3c1_540 annot-version=v3.3   Physcomitrella_patens
OG0000017
Pp3c17_23940V3.1.p pacid=32906868 transcript=Pp3c17_23940V3.1
locus=Pp3c17_23940 annot-version=v3.3   Physcomitrella_patens
OG0000017
Pp3c17_21350V3.1.p pacid=32906950 transcript=Pp3c17_21350V3.1
locus=Pp3c17_21350 annot-version=v3.3   Physcomitrella_patens
OG0000017
Pp3c12_18540V3.1.p pacid=32974576 transcript=Pp3c12_18540V3.1
locus=Pp3c12_18540 annot-version=v3.3   Physcomitrella_patens
OG0000017
Pp3c6_5720V3.1.p pacid=32975584 transcript=Pp3c6_5720V3.1
locus=Pp3c6_5720 annot-version=v3.3   Physcomitrella_patens
OG0000017
Pp3c6_6220V3.1.p pacid=32976604 transcript=Pp3c6_6220V3.1
locus=Pp3c6_6220 annot-version=v3.3   Physcomitrella_patens
OG0000017
Pp3c6_22580V3.1.p pacid=32977031 transcript=Pp3c6_22580V3.1
locus=Pp3c6_22580 annot-version=v3.3   Physcomitrella_patens
OG0000017
Pp3c6_5830V3.1.p pacid=32977406 transcript=Pp3c6_5830V3.1
locus=Pp3c6_5830 annot-version=v3.3   Physcomitrella_patens
OG0000017
Pp3c25_8200V3.1.p pacid=32979602 transcript=Pp3c25_8200V3.1
locus=Pp3c25_8200 annot-version=v3.3   Physcomitrella_patens
OG0000017
Pp3c25_4690V3.1.p pacid=32980017 transcript=Pp3c25_4690V3.1

locus=Pp3c25_4690 annot-version=v3.3 Physcomitrella_patens
OG0000017
Pp3c25_13110V3.1.p pacid=32980251 transcript=Pp3c25_13110V3.1
locus=Pp3c25_13110 annot-version=v3.3 Physcomitrella_patens
OG0000017
Pp3c25_9570V3.1.p pacid=32980264 transcript=Pp3c25_9570V3.1
locus=Pp3c25_9570 annot-version=v3.3 Physcomitrella_patens
OG0000017
Pp3c18_7600V3.1.p pacid=32982184 transcript=Pp3c18_7600V3.1
locus=Pp3c18_7600 annot-version=v3.3 Physcomitrella_patens
OG0000017
Pp3c18_7580V3.1.p pacid=32982332 transcript=Pp3c18_7580V3.1
locus=Pp3c18_7580 annot-version=v3.3 Physcomitrella_patens
OG0000017
Pp3c18_11200V3.1.p pacid=32982594 transcript=Pp3c18_11200V3.1
locus=Pp3c18_11200 annot-version=v3.3 Physcomitrella_patens
OG0000017
Pp3c18_7530V3.1.p pacid=32983418 transcript=Pp3c18_7530V3.1
locus=Pp3c18_7530 annot-version=v3.3 Physcomitrella_patens
OG0000017
Pp3c16_4060V3.1.p pacid=32984426 transcript=Pp3c16_4060V3.1
locus=Pp3c16_4060 annot-version=v3.3 Physcomitrella_patens
OG0000017
Pp3c16_11720V3.1.p pacid=32984706 transcript=Pp3c16_11720V3.1
locus=Pp3c16_11720 annot-version=v3.3 Physcomitrella_patens
OG0000017
Pp3c16_4070V3.1.p pacid=32984835 transcript=Pp3c16_4070V3.1
locus=Pp3c16_4070 annot-version=v3.3 Physcomitrella_patens
OG0000017
Pp3c16_9800V3.1.p pacid=32985080 transcript=Pp3c16_9800V3.1
locus=Pp3c16_9800 annot-version=v3.3 Physcomitrella_patens
OG0000017
Pp3c16_8120V3.1.p pacid=32985272 transcript=Pp3c16_8120V3.1
locus=Pp3c16_8120 annot-version=v3.3 Physcomitrella_patens
OG0000017
Pp3c16_9590V3.1.p pacid=32985717 transcript=Pp3c16_9590V3.1
locus=Pp3c16_9590 annot-version=v3.3 Physcomitrella_patens
OG0000017
Pp3c16_20080V3.1.p pacid=32986096 transcript=Pp3c16_20080V3.1
locus=Pp3c16_20080 annot-version=v3.3 Physcomitrella_patens
OG0000017
Pp3c16_12680V3.1.p pacid=32986825 transcript=Pp3c16_12680V3.1
locus=Pp3c16_12680 annot-version=v3.3 Physcomitrella_patens
OG0000017
Pp3c17_7090V3.1.p pacid=32908480 transcript=Pp3c17_7090V3.1
locus=Pp3c17_7090 annot-version=v3.3 Physcomitrella_patens
OG0000017
Pp3c9_24710V3.1.p pacid=32912429 transcript=Pp3c9_24710V3.1
locus=Pp3c9_24710 annot-version=v3.3 Physcomitrella_patens
OG0000017
Pp3c21_1290V3.1.p pacid=32916168 transcript=Pp3c21_1290V3.1
locus=Pp3c21_1290 annot-version=v3.3 Physcomitrella_patens
OG0000017
Pp3c26_14320V3.1.p pacid=32917752 transcript=Pp3c26_14320V3.1

locus=Pp3c26_14320 annot-version=v3.3   Physcomitrella_patens
OG0000017
Pp3c4_2230V3.1.p pacid=32920973 transcript=Pp3c4_2230V3.1
locus=Pp3c4_2230 annot-version=v3.3   Physcomitrella_patens
OG0000017
Pp3c4_15600V3.1.p pacid=32921091 transcript=Pp3c4_15600V3.1
locus=Pp3c4_15600 annot-version=v3.3   Physcomitrella_patens
OG0000017
Pp3c4_3409V3.1.p pacid=32921527 transcript=Pp3c4_3409V3.1
locus=Pp3c4_3409 annot-version=v3.3   Physcomitrella_patens
OG0000017
Pp3c4_3410V3.1.p pacid=32922577 transcript=Pp3c4_3410V3.1
locus=Pp3c4_3410 annot-version=v3.3   Physcomitrella_patens
OG0000017
Pp3c7_13350V3.1.p pacid=32925510 transcript=Pp3c7_13350V3.1
locus=Pp3c7_13350 annot-version=v3.3   Physcomitrella_patens
OG0000017
AT2G34555.1 | gibberellin 2-oxidase 3 | Chr2:14557102-14558682
FORWARD LENGTH=335 | 201606   Arabidopsis_thaliana
OG0000017
AT2G36690.1 | 2-oxoglutarate (2OG) and Fe(II)-dependent oxygenase
superfamily protein | Chr2:15379930-15381987 FORWARD LENGTH=366 |
201606 Arabidopsis_thaliana   OG0000017
AT2G38240.1 | 2-oxoglutarate (2OG) and Fe(II)-dependent oxygenase
superfamily protein | Chr2:16012723-16014666 REVERSE LENGTH=353 |
201606 Arabidopsis_thaliana   OG0000017
AT2G44800.1 | 2-oxoglutarate (2OG) and Fe(II)-dependent oxygenase
superfamily protein | Chr2:18467004-18468551 FORWARD LENGTH=357 |
201606 Arabidopsis_thaliana   OG0000017
AT1G12010.1 | 2-oxoglutarate (2OG) and Fe(II)-dependent oxygenase
superfamily protein | Chr1:4056274-4057670 FORWARD LENGTH=320 |
201606 Arabidopsis_thaliana   OG0000017
AT3G11180.1 | 2-oxoglutarate (2OG) and Fe(II)-dependent oxygenase
superfamily protein | Chr3:3504249-3506688 FORWARD LENGTH=400 |
201606 Arabidopsis_thaliana   OG0000017
AT3G12900.1 | 2-oxoglutarate (2OG) and Fe(II)-dependent oxygenase
superfamily protein | Chr3:4104576-4106112 FORWARD LENGTH=357 |
201606 Arabidopsis_thaliana   OG0000017
AT3G13610.1 | 2-oxoglutarate (2OG) and Fe(II)-dependent oxygenase
superfamily protein | Chr3:4449448-4450616 FORWARD LENGTH=361 |
201606 Arabidopsis_thaliana   OG0000017
AT3G19000.1 | 2-oxoglutarate (2OG) and Fe(II)-dependent oxygenase
superfamily protein | Chr3:6553668-6554987 REVERSE LENGTH=352 |
201606 Arabidopsis_thaliana   OG0000017
AT3G19010.1 | 2-oxoglutarate (2OG) and Fe(II)-dependent oxygenase
superfamily protein | Chr3:6556306-6557862 REVERSE LENGTH=349 |
201606 Arabidopsis_thaliana   OG0000017
AT3G21420.1 | 2-oxoglutarate (2OG) and Fe(II)-dependent oxygenase
superfamily protein | Chr3:7541579-7543221 FORWARD LENGTH=364 |
201606 Arabidopsis_thaliana   OG0000017
AT1G14120.1 | 2-oxoglutarate (2OG) and Fe(II)-dependent oxygenase
superfamily protein | Chr1:4833648-4834833 REVERSE LENGTH=312 |
201606 Arabidopsis_thaliana   OG0000017
AT1G14130.1 | 2-oxoglutarate (2OG) and Fe(II)-dependent oxygenase

superfamily protein | Chr1:4836041-4837040 REVERSE LENGTH=308 | 201606 Arabidopsis_thaliana OG0000017
AT1G02400.1 | gibberellin 2-oxidase 6 | Chr1:486964-489391 FORWARD LENGTH=329 | 201606 Arabidopsis_thaliana OG0000017
AT3G46480.1 | 2-oxoglutarate (2OG) and Fe(II)-dependent oxygenase superfamily protein | Chr3:17103173-17105594 FORWARD LENGTH=286 | 201606 Arabidopsis_thaliana OG0000017
AT3G46490.1 | 2-oxoglutarate (2OG) and Fe(II)-dependent oxygenase superfamily protein | Chr3:17115629-17119451 FORWARD LENGTH=330 | 201606 Arabidopsis_thaliana OG0000017
AT3G46500.1 | 2-oxoglutarate (2OG) and Fe(II)-dependent oxygenase superfamily protein | Chr3:17120793-17122602 FORWARD LENGTH=251 | 201606 Arabidopsis_thaliana OG0000017
AT3G47190.1 | 2-oxoglutarate (2OG) and Fe(II)-dependent oxygenase superfamily protein | Chr3:17374513-17376032 REVERSE LENGTH=331 | 201606 Arabidopsis_thaliana OG0000017
AT3G49620.1 | 2-oxoglutarate (2OG) and Fe(II)-dependent oxygenase superfamily protein | Chr3:18393823-18396662 FORWARD LENGTH=357 | 201606 Arabidopsis_thaliana OG0000017
AT3G49630.1 | 2-oxoglutarate (2OG) and Fe(II)-dependent oxygenase superfamily protein | Chr3:18397759-18400255 FORWARD LENGTH=332 | 201606 Arabidopsis_thaliana OG0000017
AT3G50210.1 | 2-oxoglutarate (2OG) and Fe(II)-dependent oxygenase superfamily protein | Chr3:18614338-18616229 REVERSE LENGTH=332 | 201606 Arabidopsis_thaliana OG0000017
AT3G51240.1 | flavanone 3-hydroxylase | Chr3:19025409-19026658 FORWARD LENGTH=358 | 201606 Arabidopsis_thaliana OG0000017
AT1G15540.1 | 2-oxoglutarate-dependent dioxygenase-like protein | Chr1:5342587-5343705 FORWARD LENGTH=320 | 201606 Arabidopsis_thaliana OG0000017
AT1G15550.1 | gibberellin 3-oxidase 1 | Chr1:5344569-5346078 REVERSE LENGTH=358 | 201606 Arabidopsis_thaliana OG0000017
AT3G55970.1 | jasmonate-regulated gene 21 | Chr3:20766970-20769264 REVERSE LENGTH=363 | 201606 Arabidopsis_thaliana OG0000017
AT3G60290.1 | 2-oxoglutarate (2OG) and Fe(II)-dependent oxygenase superfamily protein | Chr3:22282662-22284414 FORWARD LENGTH=357 | 201606 Arabidopsis_thaliana OG0000017
AT3G61400.1 | 2-oxoglutarate (2OG) and Fe(II)-dependent oxygenase superfamily protein | Chr3:22718956-22720397 FORWARD LENGTH=370 | 201606 Arabidopsis_thaliana OG0000017
AT1G17010.1 | 2-oxoglutarate (2OG) and Fe(II)-dependent oxygenase superfamily protein | Chr1:5817667-5819183 FORWARD LENGTH=361 | 201606 Arabidopsis_thaliana OG0000017
AT1G17020.1 | senescence-related gene 1 | Chr1:5820258-5821741 FORWARD LENGTH=358 | 201606 Arabidopsis_thaliana OG0000017
AT4G03050.1 | 2-oxoglutarate-dependent dioxygenase | Chr4:1344182-1346127 FORWARD LENGTH=411 | 201606 Arabidopsis_thaliana OG0000017
AT4G03070.1 | 2-oxoglutarate (2OG) and Fe(II)-dependent oxygenase superfamily protein | Chr4:1358442-1359620 FORWARD LENGTH=322 | 201606 Arabidopsis_thaliana OG0000017

AT4G10490.1 | 2-oxoglutarate (2OG) and Fe(II)-dependent oxygenase superfamily protein | Chr4:6483900-6485179 FORWARD LENGTH=348 | 201606 Arabidopsis_thaliana OG0000017
AT4G10500.1 | 2-oxoglutarate (2OG) and Fe(II)-dependent oxygenase superfamily protein | Chr4:6491089-6492342 FORWARD LENGTH=349 | 201606 Arabidopsis_thaliana OG0000017
AT4G16330.1 | 2-oxoglutarate (2OG) and Fe(II)-dependent oxygenase superfamily protein | Chr4:9226298-9227235 REVERSE LENGTH=258 | 201606 Arabidopsis_thaliana OG0000017
AT4G16765.1 | 2-oxoglutarate (2OG) and Fe(II)-dependent oxygenase superfamily protein | Chr4:9429974-9431791 REVERSE LENGTH=247 | 201606 Arabidopsis_thaliana OG0000017
AT4G16770.1 | 2-oxoglutarate (2OG) and Fe(II)-dependent oxygenase superfamily protein | Chr4:9435116-9437183 REVERSE LENGTH=258 | 201606 Arabidopsis_thaliana OG0000017
AT4G21200.1 | gibberellin 2-oxidase 8 | Chr4:11302751-11306601 FORWARD LENGTH=338 | 201606 Arabidopsis_thaliana OG0000017
AT4G21690.1 | gibberellin 3-oxidase 3 | Chr4:11527229-11529060 FORWARD LENGTH=349 | 201606 Arabidopsis_thaliana OG0000017
AT4G22870.1 | 2-oxoglutarate (2OG) and Fe(II)-dependent oxygenase superfamily protein | Chr4:12001884-12002222 REVERSE LENGTH=112 | 201606 Arabidopsis_thaliana OG0000017
AT4G22880.1 | leucoanthocyanidin dioxygenase | Chr4:12004905-12006059 REVERSE LENGTH=356 | 201606 Arabidopsis_thaliana OG0000017
AT4G23340.1 | 2-oxoglutarate (2OG) and Fe(II)-dependent oxygenase superfamily protein | Chr4:12195719-12196793 REVERSE LENGTH=324 | 201606 Arabidopsis_thaliana OG0000017
AT4G25300.1 | 2-oxoglutarate (2OG) and Fe(II)-dependent oxygenase superfamily protein | Chr4:12945263-12946642 FORWARD LENGTH=356 | 201606 Arabidopsis_thaliana OG0000017
AT4G25310.1 | 2-oxoglutarate (2OG) and Fe(II)-dependent oxygenase superfamily protein | Chr4:12949753-12951138 FORWARD LENGTH=353 | 201606 Arabidopsis_thaliana OG0000017
AT4G25420.1 | 2-oxoglutarate (2OG) and Fe(II)-dependent oxygenase superfamily protein | Chr4:12990982-12992409 REVERSE LENGTH=377 | 201606 Arabidopsis_thaliana OG0000017
AT5G05600.1 | 2-oxoglutarate (2OG) and Fe(II)-dependent oxygenase superfamily protein | Chr5:1672266-1674602 FORWARD LENGTH=371 | 201606 Arabidopsis_thaliana OG0000017
AT5G07200.1 | gibberellin 20-oxidase 3 | Chr5:2243835-2245157 REVERSE LENGTH=380 | 201606 Arabidopsis_thaliana OG0000017
AT5G07480.1 | KAR-UP oxidoreductase 1 | Chr5:2367167-2369554 FORWARD LENGTH=356 | 201606 Arabidopsis_thaliana OG0000017
AT5G08640.1 | flavonol synthase 1 | Chr5:2804009-2805175 FORWARD LENGTH=336 | 201606 Arabidopsis_thaliana OG0000017
AT5G12270.1 | 2-oxoglutarate (2OG) and Fe(II)-dependent oxygenase superfamily protein | Chr5:3970131-3971301 REVERSE LENGTH=360 | 201606 Arabidopsis_thaliana OG0000017
AT5G20400.1 | 2-oxoglutarate (2OG) and Fe(II)-dependent oxygenase superfamily protein | Chr5:6894871-6896185 FORWARD LENGTH=348 |

201606	Arabidopsis_thaliana	OG0000017
AT5G20550.1 | 2-oxoglutarate (2OG) and Fe(II)-dependent oxygenase superfamily protein | Chr5:6952569-6953856 REVERSE LENGTH=349 | 201606	Arabidopsis_thaliana	OG0000017
AT5G24530.1 | 2-oxoglutarate (2OG) and Fe(II)-dependent oxygenase superfamily protein | Chr5:8378964-8383154 FORWARD LENGTH=341 | 201606	Arabidopsis_thaliana	OG0000017
AT5G43440.1 | 2-oxoglutarate (2OG) and Fe(II)-dependent oxygenase superfamily protein | Chr5:17455356-17456608 REVERSE LENGTH=365 | 201606	Arabidopsis_thaliana	OG0000017
AT5G43450.1 | 2-oxoglutarate (2OG) and Fe(II)-dependent oxygenase superfamily protein | Chr5:17457321-17458714 REVERSE LENGTH=362 | 201606	Arabidopsis_thaliana	OG0000017
AT5G43935.1 | flavonol synthase 6 | Chr5:17682179-17683446 FORWARD LENGTH=293 | 201606	Arabidopsis_thaliana	OG0000017
AT5G51310.1 | 2-oxoglutarate (2OG) and Fe(II)-dependent oxygenase superfamily protein | Chr5:20852854-20854718 REVERSE LENGTH=325 | 201606	Arabidopsis_thaliana	OG0000017
AT1G03400.1 | 2-oxoglutarate (2OG) and Fe(II)-dependent oxygenase superfamily protein | Chr1:842895-844158 REVERSE LENGTH=351 | 201606	Arabidopsis_thaliana	OG0000017
AT5G51810.1 | gibberellin 20 oxidase 2 | Chr5:21055389-21056746 REVERSE LENGTH=378 | 201606	Arabidopsis_thaliana	OG0000017
AT1G03410.1 | 2-oxoglutarate (2OG) and Fe(II)-dependent oxygenase superfamily protein | Chr1:844782-846574 REVERSE LENGTH=398 | 201606	Arabidopsis_thaliana	OG0000017
AT5G54000.1 | 2-oxoglutarate (2OG) and Fe(II)-dependent oxygenase superfamily protein | Chr5:21917776-21918750 REVERSE LENGTH=269 | 201606	Arabidopsis_thaliana	OG0000017
AT5G58660.1 | 2-oxoglutarate (2OG) and Fe(II)-dependent oxygenase superfamily protein | Chr5:23701509-23703802 FORWARD LENGTH=352 | 201606	Arabidopsis_thaliana	OG0000017
AT5G59530.1 | 2-oxoglutarate (2OG) and Fe(II)-dependent oxygenase superfamily protein | Chr5:23994434-23995715 REVERSE LENGTH=364 | 201606	Arabidopsis_thaliana	OG0000017
AT5G59540.1 | 2-oxoglutarate (2OG) and Fe(II)-dependent oxygenase superfamily protein | Chr5:23996293-23997576 REVERSE LENGTH=366 | 201606	Arabidopsis_thaliana	OG0000017
AT5G63580.1 | flavonol synthase 2 | Chr5:25454730-25456092 FORWARD LENGTH=250 | 201606	Arabidopsis_thaliana	OG0000017
AT5G63590.1 | flavonol synthase 3 | Chr5:25457172-25458427 REVERSE LENGTH=308 | 201606	Arabidopsis_thaliana	OG0000017
AT5G63595.1 | flavonol synthase 4 | Chr5:25459256-25460436 REVERSE LENGTH=279 | 201606	Arabidopsis_thaliana	OG0000017
AT5G63600.1 | flavonol synthase 5 | Chr5:25461082-25462270 REVERSE LENGTH=325 | 201606	Arabidopsis_thaliana	OG0000017
AT1G28030.1 | 2-oxoglutarate (2OG) and Fe(II)-dependent oxygenase superfamily protein | Chr1:9771793-9773345 FORWARD LENGTH=322 | 201606	Arabidopsis_thaliana	OG0000017
AT1G30040.1 | gibberellin 2-oxidase | Chr1:10537769-10539570 FORWARD LENGTH=341 | 201606	Arabidopsis_thaliana	OG0000017
AT1G35190.1 | 2-oxoglutarate (2OG) and Fe(II)-dependent oxygenase superfamily protein | Chr1:12890544-12892632 FORWARD LENGTH=329 |

201606	Arabidopsis_thaliana	OG0000017
AT1G04350.1 | 2-oxoglutarate (2OG) and Fe(II)-dependent oxygenase superfamily protein | Chr1:1165296-1166538 FORWARD LENGTH=360 | 201606	Arabidopsis_thaliana	OG0000017
AT1G04380.1 | 2-oxoglutarate (2OG) and Fe(II)-dependent oxygenase superfamily protein | Chr1:1177142-1178384 REVERSE LENGTH=345 | 201606	Arabidopsis_thaliana	OG0000017
AT1G44090.1 | gibberellin 20-oxidase 5 | Chr1:16760677-16762486 REVERSE LENGTH=385 | 201606	Arabidopsis_thaliana	OG0000017
AT1G47990.1 | gibberellin 2-oxidase 4 | Chr1:17698655-17700834 FORWARD LENGTH=321 | 201606	Arabidopsis_thaliana	OG0000017
AT1G49390.1 | 2-oxoglutarate (2OG) and Fe(II)-dependent oxygenase superfamily protein | Chr1:18279600-18280873 FORWARD LENGTH=348 | 201606	Arabidopsis_thaliana	OG0000017
AT1G50960.1 | gibberellin 2-oxidase 7 | Chr1:18889549-18891719 FORWARD LENGTH=336 | 201606	Arabidopsis_thaliana	OG0000017
AT1G05010.1 | ethylene-forming enzyme | Chr1:1431419-1432695 REVERSE LENGTH=323 | 201606	Arabidopsis_thaliana	OG0000017
AT1G52790.1 | 2-oxoglutarate (2OG) and Fe(II)-dependent oxygenase superfamily protein | Chr1:19662194-19663301 REVERSE LENGTH=310 | 201606	Arabidopsis_thaliana	OG0000017
AT1G52800.1 | 2-oxoglutarate (2OG) and Fe(II)-dependent oxygenase superfamily protein | Chr1:19664044-19665362 FORWARD LENGTH=314 | 201606	Arabidopsis_thaliana	OG0000017
AT1G52810.1 | 2-oxoglutarate (2OG) and Fe(II)-dependent oxygenase superfamily protein | Chr1:19667127-19668237 FORWARD LENGTH=289 | 201606	Arabidopsis_thaliana	OG0000017
AT1G52820.1 | 2-oxoglutarate (2OG) and Fe(II)-dependent oxygenase superfamily protein | Chr1:19669216-19670321 FORWARD LENGTH=317 | 201606	Arabidopsis_thaliana	OG0000017
AT1G55290.1 | 2-oxoglutarate (2OG) and Fe(II)-dependent oxygenase superfamily protein | Chr1:20626208-20627397 REVERSE LENGTH=361 | 201606	Arabidopsis_thaliana	OG0000017
AT1G60980.1 | gibberellin 20-oxidase 4 | Chr1:22452573-22454140 FORWARD LENGTH=376 | 201606	Arabidopsis_thaliana	OG0000017
AT1G62380.1 | ACC oxidase 2 | Chr1:23082340-23084068 FORWARD LENGTH=320 | 201606	Arabidopsis_thaliana	OG0000017
AT1G06620.1 | 2-oxoglutarate (2OG) and Fe(II)-dependent oxygenase superfamily protein | Chr1:2025618-2027094 FORWARD LENGTH=365 | 201606	Arabidopsis_thaliana	OG0000017
AT1G06640.1 | 2-oxoglutarate (2OG) and Fe(II)-dependent oxygenase superfamily protein | Chr1:2032366-2033775 FORWARD LENGTH=369 | 201606	Arabidopsis_thaliana	OG0000017
AT1G06645.1 | 2-oxoglutarate (2OG) and Fe(II)-dependent oxygenase superfamily protein | Chr1:2034190-2035499 FORWARD LENGTH=379 | 201606	Arabidopsis_thaliana	OG0000017
AT1G06650.1 | 2-oxoglutarate (2OG) and Fe(II)-dependent oxygenase superfamily protein | Chr1:2035909-2037047 FORWARD LENGTH=314 | 201606	Arabidopsis_thaliana	OG0000017
AT1G77330.1 | 2-oxoglutarate (2OG) and Fe(II)-dependent oxygenase

superfamily protein | Chr1:29063215-29064447 REVERSE LENGTH=307 | 201606 Arabidopsis_thaliana OG0000017
AT1G78440.1 | gibberellin 2-beta-dioxygenase | Chr1:29511772-29512990 REVERSE LENGTH=329 | 201606 Arabidopsis_thaliana OG0000017
AT1G78550.1 | 2-oxoglutarate (2OG) and Fe(II)-dependent oxygenase superfamily protein | Chr1:29545031-29546360 REVERSE LENGTH=356 | 201606 Arabidopsis_thaliana OG0000017
AT1G80320.1 | 2-oxoglutarate (2OG) and Fe(II)-dependent oxygenase superfamily protein | Chr1:30196782-30197896 FORWARD LENGTH=320 | 201606 Arabidopsis_thaliana OG0000017
AT1G80330.1 | gibberellin 3-oxidase 4 | Chr1:30198061-30199537 REVERSE LENGTH=355 | 201606 Arabidopsis_thaliana OG0000017
AT1G80340.1 | gibberellin 3-oxidase 2 | Chr1:30200695-30202163 REVERSE LENGTH=347 | 201606 Arabidopsis_thaliana OG0000017
AT2G06960.1 | 2-oxoglutarate (2OG) and Fe(II)-dependent oxygenase superfamily protein | Chr2:2867672-2870352 REVERSE LENGTH=148 | 201606 Arabidopsis_thaliana OG0000017
AT2G19590.1 | ACC oxidase 1 | Chr2:8476239-8477354 REVERSE LENGTH=310 | 201606 Arabidopsis_thaliana OG0000017
AT2G25450.1 | 2-oxoglutarate (2OG) and Fe(II)-dependent oxygenase superfamily protein | Chr2:10830286-10831563 REVERSE LENGTH=359 | 201606 Arabidopsis_thaliana OG0000017
AT2G30830.1 | 2-oxoglutarate (2OG) and Fe(II)-dependent oxygenase superfamily protein | Chr2:13132707-13134284 REVERSE LENGTH=358 | 201606 Arabidopsis_thaliana OG0000017
AT2G30840.1 | 2-oxoglutarate (2OG) and Fe(II)-dependent oxygenase superfamily protein | Chr2:13135581-13136833 REVERSE LENGTH=362 | 201606 Arabidopsis_thaliana OG0000017
Os03t0289800-01 Similar to Leucoanthocyanidin dioxygenase-like protein. Oryza_sativa OG0000017
Os03t0439500-01 2OG-Fe(II) oxygenase domain containing protein. Oryza_sativa OG0000017
Os03t0618300-01 Isopenicillin N synthase family protein. Oryza_sativa OG0000017
Os03t0690500-00 Similar to Oxidoreductase, 2OG-Fe oxygenase family protein, expressed. Oryza_sativa OG0000017
Os03t0856000-01 2OG-Fe(II) oxygenase domain containing protein. Oryza_sativa OG0000017
Os03t0856700-01 Gibberellin 20 oxidase 1 (EC 1.14.11.-) (Gibberellin C-20 oxidase 1) (GA 20-oxidase 1) (Os20ox). Oryza_sativa OG0000017
Os03t0860600-01 Similar to 2-oxoglutarate-dependent oxygenase. Oryza_sativa OG0000017
Os04t0182200-01 2OG-Fe(II) oxygenase domain containing protein. Oryza_sativa OG0000017
Os04t0407800-01 Similar to H0321H01.3 protein. Oryza_sativa OG0000017
Os04t0475600-01 2OG-Fe(II) oxygenase domain containing protein. Oryza_sativa OG0000017
Os04t0522500-01 2OG-Fe(II) oxygenase domain containing protein. Oryza_sativa OG0000017

Os01t0332200-01 GA 2-oxidase2, GA metabolism	Oryza_sativa	OG0000017
Os04t0581000-01 Similar to Flavanone 3-hydroxylase (Fragment).	Oryza_sativa	OG0000017
Os04t0581100-01 2OG-Fe(II) oxygenase domain containing protein.	Oryza_sativa	OG0000017
Os04t0643500-00 Similar to H0306F03.8 protein.	Oryza_sativa	OG0000017
Os04t0662600-00 Similar to Naringenin,2-oxoglutarate 3-dioxygenase.	Oryza_sativa	OG0000017
Os04t0667200-01 Similar to H1005F08.24 protein.	Oryza_sativa	OG0000017
Os04t0667400-01 2OG-Fe(II) oxygenase domain containing protein.	Oryza_sativa	OG0000017
Os01t0351800-01 2OG-Fe(II) oxygenase domain containing protein.	Oryza_sativa	OG0000017
Os01t0352100-01 2OG-Fe(II) oxygenase domain containing protein.	Oryza_sativa	OG0000017
Os05t0127500-01 Similar to Leucoanthocyanidin dioxygenase-like protein.	Oryza_sativa	OG0000017
Os05t0149300-00 Similar to 1-aminocyclopropane-1-carboxylate oxidase.	Oryza_sativa	OG0000017
Os05t0149400-01 ACC oxidase, Ethylene biosynthesis	Oryza_sativa	OG0000017
Os05t0178100-01 GA 3beta-hydroxylase1, GA metabolism	Oryza_sativa	OG0000017
Os05t0208550-00 Similar to gibberellin 2-beta-dioxygenase.	Oryza_sativa	OG0000017
Os01t0372500-00 Similar to Leucoanthocyanidin dioxygenase (EC 1.14.11.19) (LDOX) (Leucocyanidin oxygenase) (Leucoanthocyanidin hydroxylase).	Oryza_sativa	OG0000017
Os05t0421900-00 GA 20-oxidase4, GA metabolism	Oryza_sativa	OG0000017
Os05t0514600-01 GA 2-oxidase4, GA metabolism	Oryza_sativa	OG0000017
Os05t0560900-01 Similar to gibberellin 2-beta-dioxygenase.	Oryza_sativa	OG0000017
Os06t0162500-01 Similar to Naringenin 3-dioxygenase like protein.	Oryza_sativa	OG0000017
Os01t0536400-01 Isopenicillin N synthase family protein.	Oryza_sativa	OG0000017
Os06t0176300-00 2OG-Fe(II) oxygenase domain containing protein.	Oryza_sativa	OG0000017
Os06t0176500-00 Similar to leucoanthocyanidin dioxygenase.	Oryza_sativa	OG0000017
Os06t0176650-00 Similar to Leucoanthocyanidin dioxygenase.	Oryza_sativa	OG0000017
Os06t0176700-01 Isopenicillin N synthase family protein.	Oryza_sativa	OG0000017
Os06t0177600-00 2OG-Fe(II) oxygenase domain containing protein.	Oryza_sativa	OG0000017
Os06t0177700-00 Similar to leucoanthocyanidin dioxygenase.	Oryza_sativa	OG0000017
Os06t0178450-00 Similar to Leucoanthocyanidin dioxygenase.

Oryza_sativa	OG0000017
Os06t0178500-01 Isopenicillin N synthase family protein.
Oryza_sativa	OG0000017
Os06t0178700-01 Similar to Iron/ascorbate-dependent oxidoreductase.
Oryza_sativa	OG0000017
Os06t0255100-01 2OG-Fe(II) oxygenase domain containing protein.
Oryza_sativa	OG0000017
Os06t0573900-00 Similar to 1-aminocyclopropane-1-carboxylic acid oxidase. Oryza_sativa	OG0000017
Os06t0626700-00 Isopenicillin N synthase family protein.
Oryza_sativa	OG0000017
Os01t0580500-01 ACC oxidase, Ethylene biosynthesis Oryza_sativa
OG0000017
Os07t0103500-01 2OG-Fe(II) oxygenase domain containing protein.
Oryza_sativa	OG0000017
Os07t0169600-01 2OG-Fe(II) oxygenase domain containing protein.
Oryza_sativa	OG0000017
Os07t0169700-01 GA 20-oxidase3, GA metabolism	Oryza_sativa
OG0000017
Os08t0249900-00 Similar to Gibberellin 20 oxidase 2.
Oryza_sativa	OG0000017
Os08t0390200-01 Similar to Oxidoreductase, 2OG-Fe oxygenase family protein, expressed.	Oryza_sativa	OG0000017
Os08t0391700-00 Similar to Oxidoreductase, 2OG-Fe oxygenase family protein, expressed.	Oryza_sativa	OG0000017
Os08t0392100-00 2OG-Fe(II) oxygenase domain containing protein.
Oryza_sativa	OG0000017
Os08t0417000-01 2OG-Fe(II) oxygenase domain containing protein.
Oryza_sativa	OG0000017
Os08t0417100-01 Similar to cDNA, clone	Oryza_sativa
OG0000017
Os08t0480200-01 2OG-Fe(II) oxygenase domain containing protein.
Oryza_sativa	OG0000017
Os08t0560000-01 Similar to gibberellin 20 oxidase 2.
Oryza_sativa	OG0000017
Os09t0245500-01 Isopenicillin N synthase domain containing protein.
Oryza_sativa	OG0000017
Os09t0353400-00 Similar to Leucoanthocyanidin dioxygenase.
Oryza_sativa	OG0000017
Os09t0353700-00 Similar to Leucoanthocyanidin dioxygenase.
Oryza_sativa	OG0000017
Os09t0354100-00 Similar to Leucoanthocyanidin dioxygenase.
Oryza_sativa	OG0000017
Os09t0354300-00 Similar to leucoanthocyanidin dioxygenase.
Oryza_sativa	OG0000017
Os09t0451000-01 ACC oxidase, Ethylene biosynthesis Oryza_sativa
OG0000017
Os09t0451400-01 ACC oxidase, Ethylene biosynthesis Oryza_sativa
OG0000017
Os09t0570800-01 Isopenicillin N synthase family protein.
Oryza_sativa	OG0000017
Os10t0522900-01 Isopenicillin N synthase family protein.
Oryza_sativa	OG0000017
Os10t0523100-01 Isopenicillin N synthase family protein.

Oryza_sativa	OG0000017
Os10t0536400-01 Similar to Oxidoreductase, 2OG-Fe oxygenase family protein, expressed.	Oryza_sativa	OG0000017
Os10t0558200-00 2OG-Fe(II) oxygenase domain containing protein.	Oryza_sativa	OG0000017
Os10t0558400-00 Similar to Oxidoreductase, 2OG-Fe oxygenase family protein.	Oryza_sativa	OG0000017
Os10t0558700-01 Similar to Oxidoreductase, 2OG-Fe oxygenase family protein, expressed.	Oryza_sativa	OG0000017
Os10t0558750-01 Similar to Oxidoreductase, 2OG-Fe oxygenase family protein, expressed.	Oryza_sativa	OG0000017
Os10t0558900-01 Similar to Oxidoreductase, 2OG-Fe oxygenase family protein, expressed.	Oryza_sativa	OG0000017
Os10t0559200-01 Similar to Oxidoreductase, 2OG-Fe oxygenase family protein, expressed.	Oryza_sativa	OG0000017
Os10t0559500-01 2OG-Fe(II) oxygenase domain containing protein.	Oryza_sativa	OG0000017
Os11t0186900-01 ACC oxidase, Ethylene biosynthesis	Oryza_sativa	OG0000017
Os11t0265850-00 Conserved hypothetical protein.	Oryza_sativa	OG0000017
Os11t0437800-01 2OG-Fe(II) oxygenase domain containing protein.	Oryza_sativa	OG0000017
Os01t0757200-01 GA 2-oxidase3, GA metabolism	Oryza_sativa	OG0000017
Os01t0832600-01 Similar to Leucoanthocyanidin dioxygenase-like protein.	Oryza_sativa	OG0000017
Os01t0883800-01 Similar to GA C20oxidase2.	Oryza_sativa	OG0000017
Os01t0935400-00 2OG-Fe(II) oxygenase domain containing protein.	Oryza_sativa	OG0000017
Os01t0177400-01 GA 3 beta-hydroxylase2, GA metabolism	Oryza_sativa	OG0000017
Os02t0280700-01 Similar to Iron/ascorbate-dependent oxidoreductase.	Oryza_sativa	OG0000017
Os02t0320800-01 Similar to Iron/ascorbate-dependent oxidoreductase.	Oryza_sativa	OG0000017
Os01t0188100-01 Isopenicillin N synthase family protein.	Oryza_sativa	OG0000017
Os02t0592000-00 Similar to OSIGBa0106G07.8 protein.	Oryza_sativa	OG0000017
Os02t0630300-01 2OG-Fe(II) oxygenase domain containing protein.	Oryza_sativa	OG0000017
Os01t0209700-01 Similar to GA 2-oxidase 5.	Oryza_sativa	OG0000017
Os02t0767300-01 Similar to Flavonol synthase/flavanone 3-hydroxylase (EC 1.14.11.23) (EC 1.14.11.9) (FLS) (CitFLS).	Oryza_sativa	OG0000017
Os02t0771600-01 Similar to 1-aminocyclopropane-1-carboxylate oxidase (Fragment).	Oryza_sativa	OG0000017
Os03t0122300-01 Similar to Flavanone 3-hydroxylase-like protein.	Oryza_sativa	OG0000017
CMR064CT pep chromosome:ASM9120v1:18:172181:173609:-1 gene:CMR064C transcript:CMR064CT gene_biotype:protein_coding

transcript_biotype:protein_coding Cyanidioschyzon_merolae
OG0000017
evm.model.contig_2022.1 Porphyridium_purpureum OG0000017
evm.model.contig_3423.14 Porphyridium_purpureum OG0000017
evm.model.contig_4437.10 Porphyridium_purpureum OG0000017
g1080.t1 Caulerpa_lentillifera OG0000018
g1253.t1 Caulerpa_lentillifera OG0000018
g1286.t1 Caulerpa_lentillifera OG0000018
g1846.t1 Caulerpa_lentillifera OG0000018
g1916.t1 Caulerpa_lentillifera OG0000018
g2014.t1 Caulerpa_lentillifera OG0000018
g2337.t1 Caulerpa_lentillifera OG0000018
g2776.t1 Caulerpa_lentillifera OG0000018
g3875.t1 Caulerpa_lentillifera OG0000018
g4868.t1 Caulerpa_lentillifera OG0000018
g5266.t1 Caulerpa_lentillifera OG0000018
g5505.t1 Caulerpa_lentillifera OG0000018
g5990.t1 Caulerpa_lentillifera OG0000018
g7120.t1 Caulerpa_lentillifera OG0000018
g752.t1 Caulerpa_lentillifera OG0000018
g7909.t1 Caulerpa_lentillifera OG0000018
g8216.t1 Caulerpa_lentillifera OG0000018
g898.t1 Caulerpa_lentillifera OG0000018
g9015.t1 Caulerpa_lentillifera OG0000018
Cre02.g110750.t1.2 pacid=30786308 transcript=Cre02.g110750.t1.2
locus=Cre02.g110750 ID=Cre02.g110750.t1.2.v5.5 annot-version=v5.5
Chlamydomonas_reinhardtii OG0000018
Cre07.g344950.t1.2 pacid=30774737 transcript=Cre07.g344950.t1.2
locus=Cre07.g344950 ID=Cre07.g344950.t1.2.v5.5 annot-version=v5.5
Chlamydomonas_reinhardtii OG0000018
Cre03.g156900.t1.2 pacid=30787808 transcript=Cre03.g156900.t1.2
locus=Cre03.g156900 ID=Cre03.g156900.t1.2.v5.5 annot-version=v5.5
Chlamydomonas_reinhardtii OG0000018
Cre01.g066917.t1.1 pacid=30788321 transcript=Cre01.g066917.t1.1
locus=Cre01.g066917 ID=Cre01.g066917.t1.1.v5.5 annot-version=v5.5
Chlamydomonas_reinhardtii OG0000018
Cre10.g425900.t1.2 pacid=30790196 transcript=Cre10.g425900.t1.2
locus=Cre10.g425900 ID=Cre10.g425900.t1.2.v5.5 annot-version=v5.5
Chlamydomonas_reinhardtii OG0000018
Cre10.g452050.t1.2 pacid=30790835 transcript=Cre10.g452050.t1.2
locus=Cre10.g452050 ID=Cre10.g452050.t1.2.v5.5 annot-version=v5.5
Chlamydomonas_reinhardtii OG0000018
Cre04.g232104.t1.1 pacid=30791553 transcript=Cre04.g232104.t1.1
locus=Cre04.g232104 ID=Cre04.g232104.t1.1.v5.5 annot-version=v5.5
Chlamydomonas_reinhardtii OG0000018
Cre12.g508750.t1.2 pacid=30791684 transcript=Cre12.g508750.t1.2
locus=Cre12.g508750 ID=Cre12.g508750.t1.2.v5.5 annot-version=v5.5
Chlamydomonas_reinhardtii OG0000018
Cre12.g548400.t1.2 pacid=30793460 transcript=Cre12.g548400.t1.2
locus=Cre12.g548400 ID=Cre12.g548400.t1.2.v5.5 annot-version=v5.5
Chlamydomonas_reinhardtii OG0000018
Cre12.g548950.t1.2 pacid=30793494 transcript=Cre12.g548950.t1.2
locus=Cre12.g548950 ID=Cre12.g548950.t1.2.v5.5 annot-version=v5.5
Chlamydomonas_reinhardtii OG0000018

Cre11.g467573.t1.1 pacid=30776039 transcript=Cre11.g467573.t1.1 locus=Cre11.g467573 ID=Cre11.g467573.t1.1.v5.5 annot-version=v5.5
Chlamydomonas_reinhardtii OG0000018
Cre16.g673650.t1.1 pacid=30777655 transcript=Cre16.g673650.t1.1 locus=Cre16.g673650 ID=Cre16.g673650.t1.1.v5.5 annot-version=v5.5
Chlamydomonas_reinhardtii OG0000018
Cre16.g687900.t1.2 pacid=30778224 transcript=Cre16.g687900.t1.2 locus=Cre16.g687900 ID=Cre16.g687900.t1.2.v5.5 annot-version=v5.5
Chlamydomonas_reinhardtii OG0000018
Cre06.g284200.t1.2 pacid=30778931 transcript=Cre06.g284200.t1.2 locus=Cre06.g284200 ID=Cre06.g284200.t1.2.v5.5 annot-version=v5.5
Chlamydomonas_reinhardtii OG0000018
Cre06.g283050.t1.2 pacid=30779127 transcript=Cre06.g283050.t1.2 locus=Cre06.g283050 ID=Cre06.g283050.t1.2.v5.5 annot-version=v5.5
Chlamydomonas_reinhardtii OG0000018
Cre06.g278213.t1.1 pacid=30779171 transcript=Cre06.g278213.t1.1 locus=Cre06.g278213 ID=Cre06.g278213.t1.1.v5.5 annot-version=v5.5
Chlamydomonas_reinhardtii OG0000018
Cre06.g285250.t1.2 pacid=30779463 transcript=Cre06.g285250.t1.2 locus=Cre06.g285250 ID=Cre06.g285250.t1.2.v5.5 annot-version=v5.5
Chlamydomonas_reinhardtii OG0000018
Cre06.g283950.t1.2 pacid=30779716 transcript=Cre06.g283950.t1.2 locus=Cre06.g283950 ID=Cre06.g283950.t1.2.v5.5 annot-version=v5.5
Chlamydomonas_reinhardtii OG0000018
Cre06.g272650.t1.2 pacid=30779881 transcript=Cre06.g272650.t1.2 locus=Cre06.g272650 ID=Cre06.g272650.t1.2.v5.5 annot-version=v5.5
Chlamydomonas_reinhardtii OG0000018
Cre06.g284250.t1.2 pacid=30779962 transcript=Cre06.g284250.t1.2 locus=Cre06.g284250 ID=Cre06.g284250.t1.2.v5.5 annot-version=v5.5
Chlamydomonas_reinhardtii OG0000018
Vocar.0012s0145.1.p pacid=32894805 transcript=Vocar.0012s0145.1 locus=Vocar.0012s0145 ID=Vocar.0012s0145.1.v2.1 annot-version=v2.1
Volvox_carteri OG0000018
Vocar.0012s0221.1.p pacid=32894985 transcript=Vocar.0012s0221.1 locus=Vocar.0012s0221 ID=Vocar.0012s0221.1.v2.1 annot-version=v2.1
Volvox_carteri OG0000018
Vocar.0012s0172.1.p pacid=32895002 transcript=Vocar.0012s0172.1 locus=Vocar.0012s0172 ID=Vocar.0012s0172.1.v2.1 annot-version=v2.1
Volvox_carteri OG0000018
Vocar.0012s0144.1.p pacid=32895035 transcript=Vocar.0012s0144.1 locus=Vocar.0012s0144 ID=Vocar.0012s0144.1.v2.1 annot-version=v2.1
Volvox_carteri OG0000018
Vocar.0011s0017.1.p pacid=32895358 transcript=Vocar.0011s0017.1 locus=Vocar.0011s0017 ID=Vocar.0011s0017.1.v2.1 annot-version=v2.1
Volvox_carteri OG0000018
Vocar.0028s0157.1.p pacid=32895586 transcript=Vocar.0028s0157.1 locus=Vocar.0028s0157 ID=Vocar.0028s0157.1.v2.1 annot-version=v2.1
Volvox_carteri OG0000018
Vocar.0016s0243.1.p pacid=32897935 transcript=Vocar.0016s0243.1 locus=Vocar.0016s0243 ID=Vocar.0016s0243.1.v2.1 annot-version=v2.1
Volvox_carteri OG0000018
Vocar.0025s0135.1.p pacid=32898246 transcript=Vocar.0025s0135.1 locus=Vocar.0025s0135 ID=Vocar.0025s0135.1.v2.1 annot-version=v2.1
Volvox_carteri OG0000018

Vocar.0709s0001.1.p pacid=32898457 transcript=Vocar.0709s0001.1 locus=Vocar.0709s0001 ID=Vocar.0709s0001.1.v2.1 annot-version=v2.1
Volvox_carteri OG0000018
Vocar.0018s0037.1.p pacid=32898532 transcript=Vocar.0018s0037.1 locus=Vocar.0018s0037 ID=Vocar.0018s0037.1.v2.1 annot-version=v2.1
Volvox_carteri OG0000018
Vocar.0015s0072.1.p pacid=32899153 transcript=Vocar.0015s0072.1 locus=Vocar.0015s0072 ID=Vocar.0015s0072.1.v2.1 annot-version=v2.1
Volvox_carteri OG0000018
Vocar.0023s0180.1.p pacid=32883354 transcript=Vocar.0023s0180.1 locus=Vocar.0023s0180 ID=Vocar.0023s0180.1.v2.1 annot-version=v2.1
Volvox_carteri OG0000018
Vocar.0001s0479.1.p pacid=32885541 transcript=Vocar.0001s0479.1 locus=Vocar.0001s0479 ID=Vocar.0001s0479.1.v2.1 annot-version=v2.1
Volvox_carteri OG0000018
Vocar.0001s1616.1.p pacid=32885577 transcript=Vocar.0001s1616.1 locus=Vocar.0001s1616 ID=Vocar.0001s1616.1.v2.1 annot-version=v2.1
Volvox_carteri OG0000018
Vocar.0001s1775.1.p pacid=32885648 transcript=Vocar.0001s1775.1 locus=Vocar.0001s1775 ID=Vocar.0001s1775.1.v2.1 annot-version=v2.1
Volvox_carteri OG0000018
Vocar.0007s0215.1.p pacid=32888099 transcript=Vocar.0007s0215.1 locus=Vocar.0007s0215 ID=Vocar.0007s0215.1.v2.1 annot-version=v2.1
Volvox_carteri OG0000018
Vocar.0007s0216.1.p pacid=32888272 transcript=Vocar.0007s0216.1 locus=Vocar.0007s0216 ID=Vocar.0007s0216.1.v2.1 annot-version=v2.1
Volvox_carteri OG0000018
Vocar.0014s0016.1.p pacid=32888452 transcript=Vocar.0014s0016.1 locus=Vocar.0014s0016 ID=Vocar.0014s0016.1.v2.1 annot-version=v2.1
Volvox_carteri OG0000018
Vocar.0004s0477.1.p pacid=32889560 transcript=Vocar.0004s0477.1 locus=Vocar.0004s0477 ID=Vocar.0004s0477.1.v2.1 annot-version=v2.1
Volvox_carteri OG0000018
Vocar.0005s0361.1.p pacid=32883819 transcript=Vocar.0005s0361.1 locus=Vocar.0005s0361 ID=Vocar.0005s0361.1.v2.1 annot-version=v2.1
Volvox_carteri OG0000018
Vocar.0002s0044.1.p pacid=32890554 transcript=Vocar.0002s0044.1 locus=Vocar.0002s0044 ID=Vocar.0002s0044.1.v2.1 annot-version=v2.1
Volvox_carteri OG0000018
Vocar.0002s0281.1.p pacid=32890560 transcript=Vocar.0002s0281.1 locus=Vocar.0002s0281 ID=Vocar.0002s0281.1.v2.1 annot-version=v2.1
Volvox_carteri OG0000018
Vocar.0002s0071.1.p pacid=32890569 transcript=Vocar.0002s0071.1 locus=Vocar.0002s0071 ID=Vocar.0002s0071.1.v2.1 annot-version=v2.1
Volvox_carteri OG0000018
Vocar.0002s0041.1.p pacid=32890610 transcript=Vocar.0002s0041.1 locus=Vocar.0002s0041 ID=Vocar.0002s0041.1.v2.1 annot-version=v2.1
Volvox_carteri OG0000018
Vocar.0002s0052.1.p pacid=32890630 transcript=Vocar.0002s0052.1 locus=Vocar.0002s0052 ID=Vocar.0002s0052.1.v2.1 annot-version=v2.1
Volvox_carteri OG0000018
Vocar.0002s0030.1.p pacid=32890636 transcript=Vocar.0002s0030.1 locus=Vocar.0002s0030 ID=Vocar.0002s0030.1.v2.1 annot-version=v2.1
Volvox_carteri OG0000018

Vocar.0002s0051.1.p pacid=32890692 transcript=Vocar.0002s0051.1 locus=Vocar.0002s0051 ID=Vocar.0002s0051.1.v2.1 annot-version=v2.1
Volvox_carteri OG0000018
Vocar.0002s0333.1.p pacid=32890723 transcript=Vocar.0002s0333.1 locus=Vocar.0002s0333 ID=Vocar.0002s0333.1.v2.1 annot-version=v2.1
Volvox_carteri OG0000018
Vocar.0002s0046.1.p pacid=32890744 transcript=Vocar.0002s0046.1 locus=Vocar.0002s0046 ID=Vocar.0002s0046.1.v2.1 annot-version=v2.1
Volvox_carteri OG0000018
Vocar.0002s0042.1.p pacid=32890769 transcript=Vocar.0002s0042.1 locus=Vocar.0002s0042 ID=Vocar.0002s0042.1.v2.1 annot-version=v2.1
Volvox_carteri OG0000018
Vocar.0002s0050.1.p pacid=32890818 transcript=Vocar.0002s0050.1 locus=Vocar.0002s0050 ID=Vocar.0002s0050.1.v2.1 annot-version=v2.1
Volvox_carteri OG0000018
Vocar.0002s0054.1.p pacid=32891060 transcript=Vocar.0002s0054.1 locus=Vocar.0002s0054 ID=Vocar.0002s0054.1.v2.1 annot-version=v2.1
Volvox_carteri OG0000018
Vocar.0002s0047.1.p pacid=32891068 transcript=Vocar.0002s0047.1 locus=Vocar.0002s0047 ID=Vocar.0002s0047.1.v2.1 annot-version=v2.1
Volvox_carteri OG0000018
Vocar.0002s0048.1.p pacid=32891141 transcript=Vocar.0002s0048.1 locus=Vocar.0002s0048 ID=Vocar.0002s0048.1.v2.1 annot-version=v2.1
Volvox_carteri OG0000018
Vocar.0002s0055.1.p pacid=32891143 transcript=Vocar.0002s0055.1 locus=Vocar.0002s0055 ID=Vocar.0002s0055.1.v2.1 annot-version=v2.1
Volvox_carteri OG0000018
Vocar.0002s0043.1.p pacid=32891182 transcript=Vocar.0002s0043.1 locus=Vocar.0002s0043 ID=Vocar.0002s0043.1.v2.1 annot-version=v2.1
Volvox_carteri OG0000018
Vocar.0002s0049.1.p pacid=32891243 transcript=Vocar.0002s0049.1 locus=Vocar.0002s0049 ID=Vocar.0002s0049.1.v2.1 annot-version=v2.1
Volvox_carteri OG0000018
Vocar.0050s0033.1.p pacid=32892380 transcript=Vocar.0050s0033.1 locus=Vocar.0050s0033 ID=Vocar.0050s0033.1.v2.1 annot-version=v2.1
Volvox_carteri OG0000018
Vocar.0009s0370.1.p pacid=32893259 transcript=Vocar.0009s0370.1 locus=Vocar.0009s0370 ID=Vocar.0009s0370.1.v2.1 annot-version=v2.1
Volvox_carteri OG0000018
Vocar.0006s0236.1.p pacid=32894038 transcript=Vocar.0006s0236.1 locus=Vocar.0006s0236 ID=Vocar.0006s0236.1.v2.1 annot-version=v2.1
Volvox_carteri OG0000018
Vocar.0030s0156.1.p pacid=32894566 transcript=Vocar.0030s0156.1 locus=Vocar.0030s0156 ID=Vocar.0030s0156.1.v2.1 annot-version=v2.1
Volvox_carteri OG0000018
jgi|ChlNC64A_1|59790|estExt_fgenesh3_kg.C_260011
Chlorella_variabilis OG0000018
jgi|ChlNC64A_1|59584|estExt_fgenesh3_kg.C_30020
Chlorella_variabilis OG0000018
jgi|ChlNC64A_1|29899|estExt_Genewise1.C_30591
Chlorella_variabilis OG0000018
jgi|ChlNC64A_1|50418|fgenesh3_pg.C_scaffold_3000291
Chlorella_variabilis OG0000018
jgi|ChlNC64A_1|35818|estExt_Genewise1Plus.C_120416

Chlorella_variabilis	OG0000018
jgi|ChlNC64A_1|35848|estExt_Genewise1Plus.C_120473
Chlorella_variabilis	OG0000018
jgi|ChlNC64A_1|134943|IGS.gm_12_00329	Chlorella_variabilis
OG0000018
jgi|ChlNC64A_1|32710|estExt_Genewise1.C_240191
Chlorella_variabilis	OG0000018
jgi|ChlNC64A_1|32733|estExt_Genewise1.C_240234
Chlorella_variabilis	OG0000018
jgi|ChlNC64A_1|137219|IGS.gm_18_00325	Chlorella_variabilis
OG0000018
jgi|ChlNC64A_1|34387|estExt_Genewise1Plus.C_40251
Chlorella_variabilis	OG0000018
jgi|ChlNC64A_1|59709|estExt_fgenesh3_kg.C_140005
Chlorella_variabilis	OG0000018
jgi|ChlNC64A_1|56227|estExt_fgenesh3_pm.C_140028
Chlorella_variabilis	OG0000018
jgi|ChlNC64A_1|59626|estExt_fgenesh3_kg.C_50012
Chlorella_variabilis	OG0000018
jgi|ChlNC64A_1|24450|e_gw1.13.141.1	Chlorella_variabilis
OG0000018
jgi|ChlNC64A_1|31627|estExt_Genewise1.C_130161
Chlorella_variabilis	OG0000018
jgi|ChlNC64A_1|36644|estExt_Genewise1Plus.C_190074
Chlorella_variabilis	OG0000018
64185 pacid=27389211 transcript=64185 locus=estExt_Genemark1.C_10307
ID=64185.2.0.227 annot-version=v2.0	Coccomyxa_subellipsoidea
OG0000018
61250 pacid=27389730 transcript=61250 locus=Genemark1.2040_g
ID=61250.2.0.227 annot-version=v2.0	Coccomyxa_subellipsoidea
OG0000018
52854 pacid=27389914 transcript=52854
locus=estExt_fgenesh1_pm.C_40177 ID=52854.2.0.227 annot-version=v2.0
Coccomyxa_subellipsoidea	OG0000018
28488 pacid=27391114 transcript=28488
locus=estExt_Genewise1Plus.C_60020 ID=28488.2.0.227 annot-
version=v2.0	Coccomyxa_subellipsoidea	OG0000018
66689 pacid=27391966 transcript=66689
locus=estExt_Genemark1.C_110072 ID=66689.2.0.227 annot-version=v2.0
Coccomyxa_subellipsoidea	OG0000018
15915 pacid=27392412 transcript=15915 locus=e_gw1.8.299.1
ID=15915.2.0.227 annot-version=v2.0	Coccomyxa_subellipsoidea
OG0000018
44831 pacid=27392969 transcript=44831 locus=fgenesh1_pg.20_#_30
ID=44831.2.0.227 annot-version=v2.0	Coccomyxa_subellipsoidea
OG0000018
58975 pacid=27393083 transcript=58975 locus=Genemark1.937_g
ID=58975.2.0.227 annot-version=v2.0	Coccomyxa_subellipsoidea
OG0000018
46127 pacid=27393097 transcript=46127
locus=estExt_fgenesh1_pg.C_20205 ID=46127.2.0.227 annot-version=v2.0
Coccomyxa_subellipsoidea	OG0000018
27246 pacid=27393223 transcript=27246
locus=estExt_Genewise1Plus.C_20570 ID=27246.2.0.227 annot-

version=v2.0	Coccomyxa_subellipsoidea	OG0000018
52367 pacid=27393246 transcript=52367
locus=estExt_fgenesh1_pm.C_20118 ID=52367.2.0.227 annot-version=v2.0
	Coccomyxa_subellipsoidea	OG0000018
21950 pacid=27393356 transcript=21950 locus=estExt_Genewise1.C_20640
ID=21950.2.0.227 annot-version=v2.0	Coccomyxa_subellipsoidea
OG0000018
35121 pacid=27393490 transcript=35121 locus=fgenesh1_pm.2_#_131
ID=35121.2.0.227 annot-version=v2.0	Coccomyxa_subellipsoidea
OG0000018
44136 pacid=27393958 transcript=44136 locus=fgenesh1_pg.16_#_115
ID=44136.2.0.227 annot-version=v2.0	Coccomyxa_subellipsoidea
OG0000018
7387 pacid=27393967 transcript=7387 locus=gw1.16.279.1
ID=7387.2.0.227 annot-version=v2.0	Coccomyxa_subellipsoidea
OG0000018
37969 pacid=27393977 transcript=37969 locus=fgenesh1_pm.16_#_134
ID=37969.2.0.227 annot-version=v2.0	Coccomyxa_subellipsoidea
OG0000018
38026 pacid=27394620 transcript=38026 locus=fgenesh1_pm.17_#_55
ID=38026.2.0.227 annot-version=v2.0	Coccomyxa_subellipsoidea
OG0000018
25286 pacid=27394815 transcript=25286
locus=estExt_Genewise1.C_150142 ID=25286.2.0.227 annot-version=v2.0
Coccomyxa_subellipsoidea	OG0000018
48543 pacid=27394836 transcript=48543
locus=estExt_fgenesh1_pg.C_150125 ID=48543.2.0.227 annot-
version=v2.0	Coccomyxa_subellipsoidea	OG0000018
25284 pacid=27394962 transcript=25284
locus=estExt_Genewise1.C_150140 ID=25284.2.0.227 annot-version=v2.0
Coccomyxa_subellipsoidea	OG0000018
15720_e_gw1.02.00.512.1	Ostreococcus_tauri	OG0000018
16013_e_gw1.03.00.424.1	Ostreococcus_tauri	OG0000018
19353_e_gw1.09.00.338.1	Ostreococcus_tauri	OG0000018
21088_e_gw1.14.00.229.1	Ostreococcus_tauri	OG0000018
23114_estExt_fgenesh1_pm.C_Chr_18.00010014	Ostreococcus_tauri
OG0000018
26547_estExt_genewise1.C_Chr_11.00010240	Ostreococcus_tauri
OG0000018
27558_estExt_gwp_GeneWisePlus.C_Chr_02.00010168
Ostreococcus_tauri	OG0000018
33699 pacid=27413952 transcript=33699 locus=eugene.0900010403
ID=33699.2.0.231 annot-version=v2.0	Ostreococcus_lucimarinus
OG0000018
24045 pacid=27414841 transcript=24045
locus=estExt_fgenesh1_pg.C_Chr_20122 ID=24045.2.0.231 annot-
version=v2.0	Ostreococcus_lucimarinus	OG0000018
31850 pacid=27416869 transcript=31850 locus=eugene.0500010387
ID=31850.2.0.231 annot-version=v2.0	Ostreococcus_lucimarinus
OG0000018
34554 pacid=27413381 transcript=34554 locus=e_gwEuk.3.580.1
ID=34554.2.0.231 annot-version=v2.0	Ostreococcus_lucimarinus
OG0000018
47897 pacid=27419036 transcript=47897

locus=estExt_GenewiseEukaryote.C_Chr_170164 ID=47897.2.0.231 annot-version=v2.0   Ostreococcus_lucimarinus OG0000018
29411 pacid=27420116 transcript=29411 locus=eugene.2000010117 ID=29411.2.0.231 annot-version=v2.0   Ostreococcus_lucimarinus OG0000018
88576 pacid=27420648 transcript=88576 locus=ost_11_006_033 ID=88576.2.0.231 annot-version=v2.0   Ostreococcus_lucimarinus OG0000018
36592 pacid=27348662 transcript=36592 locus=MicpuC2.estExt_Genewise1Plus.C_150204 ID=36592.3.0.228 annot-version=v3.0   Micromonas_pusilla   OG0000018
70906 pacid=27340949 transcript=70906 locus=MicpuC2.POU_gw1.7.605.1 ID=70906.3.0.228 annot-version=v3.0   Micromonas_pusilla OG0000018
70884 pacid=27342730 transcript=70884 locus=MicpuC2.POU_AZW_EuGene.0000020452 ID=70884.3.0.228 annot-version=v3.0 Micromonas_pusilla   OG0000018
120996 pacid=27342852 transcript=121036 locus=fgenesh3_kg.2_#_184_#_isotig00963 ID=121036.3.0.228 annot-version=v3.0 Micromonas_pusilla   OG0000018
46049 pacid=27344823 transcript=46049 locus=MicpuC2.estExt_fgenesh1_pm.C_170029 ID=46049.3.0.228 annot-version=v3.0   Micromonas_pusilla   OG0000018
50023 pacid=27345612 transcript=50023 locus=MicpuC2.EuGene.0000010247 ID=50023.3.0.228 annot-version=v3.0 Micromonas_pusilla   OG0000018
49623 pacid=27348403 transcript=49623 locus=MicpuC2.estExt_fgenesh1_kg.C_60016 ID=49623.3.0.228 annot-version=v3.0   Micromonas_pusilla   OG0000018
104821 pacid=27395509 transcript=104821 locus=estExt_fgenesh2_kg.C_Chr_050019 ID=104821.3.0.229 annot-version=v3.0   Micromonas_sp._RCC299   OG0000018
90394 pacid=27398612 transcript=90394 locus=estExt_Genewise2.C_Chr_030908 ID=90394.3.0.229 annot-version=v3.0   Micromonas_sp._RCC299   OG0000018
54950 pacid=27398730 transcript=54950 locus=EuGene.0100010177 ID=54950.3.0.229 annot-version=v3.0   Micromonas_sp._RCC299 OG0000018
55391 pacid=27399228 transcript=55391 locus=EuGene.0100010618 ID=55391.3.0.229 annot-version=v3.0   Micromonas_sp._RCC299 OG0000018
104693 pacid=27399415 transcript=104693 locus=estExt_fgenesh2_kg.C_Chr_010010 ID=104693.3.0.229 annot-version=v3.0   Micromonas_sp._RCC299   OG0000018
82194 pacid=27395881 transcript=82194 locus=e_gw2.05.952.1 ID=82194.3.0.229 annot-version=v3.0   Micromonas_sp._RCC299 OG0000018
95759 pacid=27402890 transcript=95759 locus=estExt_Genewise2Plus.C_Chr_130337 ID=95759.3.0.229 annot-version=v3.0   Micromonas_sp._RCC299   OG0000018
104964 pacid=27405095 transcript=104964 locus=estExt_fgenesh2_kg.C_Chr_110001 ID=104964.3.0.229 annot-version=v3.0   Micromonas_sp._RCC299   OG0000018
kfl00422_0020_v1.1   Klebsormidium_flaccidum OG0000018

kfl00517_0030_v1.1   Klebsormidium_flaccidum OG0000018
kfl00517_0040_v1.1   Klebsormidium_flaccidum OG0000018
kfl00560_0090_v1.1   Klebsormidium_flaccidum OG0000018
kfl00564_0030_v1.1   Klebsormidium_flaccidum OG0000018
kfl00809_0010_v1.1   Klebsormidium_flaccidum OG0000018
kfl00023_0070_v1.1   Klebsormidium_flaccidum OG0000018
kfl01434_0010_v1.1   Klebsormidium_flaccidum OG0000018
kfl00098_0080_v1.1   Klebsormidium_flaccidum OG0000018
kfl00098_0090_v1.1   Klebsormidium_flaccidum OG0000018
kfl00100_0170_v1.1   Klebsormidium_flaccidum OG0000018
kfl00104_0350_v1.1   Klebsormidium_flaccidum OG0000018
kfl00104_0370_v1.1   Klebsormidium_flaccidum OG0000018
kfl00120_0160_v1.1   Klebsormidium_flaccidum OG0000018
kfl00009_0500_v1.1   Klebsormidium_flaccidum OG0000018
kfl00009_0510_v1.1   Klebsormidium_flaccidum OG0000018
kfl00214_0160_v1.1   Klebsormidium_flaccidum OG0000018
kfl00276_0010_v1.1   Klebsormidium_flaccidum OG0000018
Pp3c10_25450V3.1.p pacid=32902176 transcript=Pp3c10_25450V3.1 locus=Pp3c10_25450 annot-version=v3.3   Physcomitrella_patens OG0000018
Pp3c10_3020V3.1.p pacid=32899612 transcript=Pp3c10_3020V3.1 locus=Pp3c10_3020 annot-version=v3.3   Physcomitrella_patens OG0000018
Pp3c13_5930V3.1.p pacid=32929852 transcript=Pp3c13_5930V3.1 locus=Pp3c13_5930 annot-version=v3.3   Physcomitrella_patens OG0000018
Pp3c10_25410V3.1.p pacid=32902634 transcript=Pp3c10_25410V3.1 locus=Pp3c10_25410 annot-version=v3.3   Physcomitrella_patens OG0000018
Pp3c13_14980V3.1.p pacid=32931427 transcript=Pp3c13_14980V3.1 locus=Pp3c13_14980 annot-version=v3.3   Physcomitrella_patens OG0000018
Pp3c13_7900V3.1.p pacid=32931619 transcript=Pp3c13_7900V3.1 locus=Pp3c13_7900 annot-version=v3.3   Physcomitrella_patens OG0000018
Pp3c2_36380V3.1.p pacid=32933002 transcript=Pp3c2_36380V3.1 locus=Pp3c2_36380 annot-version=v3.3   Physcomitrella_patens OG0000018
Pp3c2_36480V3.1.p pacid=32934047 transcript=Pp3c2_36480V3.1 locus=Pp3c2_36480 annot-version=v3.3   Physcomitrella_patens OG0000018
Pp3c2_35930V3.1.p pacid=32934063 transcript=Pp3c2_35930V3.1 locus=Pp3c2_35930 annot-version=v3.3   Physcomitrella_patens OG0000018
Pp3c2_36570V3.1.p pacid=32934231 transcript=Pp3c2_36570V3.1 locus=Pp3c2_36570 annot-version=v3.3   Physcomitrella_patens OG0000018
Pp3c22_5610V3.1.p pacid=32903024 transcript=Pp3c22_5610V3.1 locus=Pp3c22_5610 annot-version=v3.3   Physcomitrella_patens OG0000018
Pp3c2_36220V3.1.p pacid=32936525 transcript=Pp3c2_36220V3.1 locus=Pp3c2_36220 annot-version=v3.3   Physcomitrella_patens OG0000018
Pp3c2_36500V3.1.p pacid=32936924 transcript=Pp3c2_36500V3.1

locus=Pp3c2_36500 annot-version=v3.3	Physcomitrella_patens
OG0000018
Pp3c2_36190V3.1.p pacid=32937453 transcript=Pp3c2_36190V3.1
locus=Pp3c2_36190 annot-version=v3.3	Physcomitrella_patens
OG0000018
Pp3c19_20900V3.1.p pacid=32939326 transcript=Pp3c19_20900V3.1
locus=Pp3c19_20900 annot-version=v3.3	Physcomitrella_patens
OG0000018
Pp3c3_4140V3.1.p pacid=32941007 transcript=Pp3c3_4140V3.1
locus=Pp3c3_4140 annot-version=v3.3	Physcomitrella_patens
OG0000018
Pp3c3_4100V3.1.p pacid=32941267 transcript=Pp3c3_4100V3.1
locus=Pp3c3_4100 annot-version=v3.3	Physcomitrella_patens
OG0000018
Pp3c3_12510V3.1.p pacid=32943909 transcript=Pp3c3_12510V3.1
locus=Pp3c3_12510 annot-version=v3.3	Physcomitrella_patens
OG0000018
Pp3c20_23270V3.1.p pacid=32945375 transcript=Pp3c20_23270V3.1
locus=Pp3c20_23270 annot-version=v3.3	Physcomitrella_patens
OG0000018
Pp3c20_23240V3.1.p pacid=32946064 transcript=Pp3c20_23240V3.1
locus=Pp3c20_23240 annot-version=v3.3	Physcomitrella_patens
OG0000018
Pp3c27_7020V3.1.p pacid=32951545 transcript=Pp3c27_7020V3.1
locus=Pp3c27_7020 annot-version=v3.3	Physcomitrella_patens
OG0000018
Pp3c27_7010V3.1.p pacid=32951782 transcript=Pp3c27_7010V3.1
locus=Pp3c27_7010 annot-version=v3.3	Physcomitrella_patens
OG0000018
Pp3c5_22920V3.1.p pacid=32952925 transcript=Pp3c5_22920V3.1
locus=Pp3c5_22920 annot-version=v3.3	Physcomitrella_patens
OG0000018
Pp3c5_7180V3.1.p pacid=32953194 transcript=Pp3c5_7180V3.1
locus=Pp3c5_7180 annot-version=v3.3	Physcomitrella_patens
OG0000018
Pp3c5_14930V3.1.p pacid=32953240 transcript=Pp3c5_14930V3.1
locus=Pp3c5_14930 annot-version=v3.3	Physcomitrella_patens
OG0000018
Pp3c5_7150V3.1.p pacid=32953818 transcript=Pp3c5_7150V3.1
locus=Pp3c5_7150 annot-version=v3.3	Physcomitrella_patens
OG0000018
Pp3c5_7110V3.1.p pacid=32955588 transcript=Pp3c5_7110V3.1
locus=Pp3c5_7110 annot-version=v3.3	Physcomitrella_patens
OG0000018
Pp3c1_34240V3.1.p pacid=32967222 transcript=Pp3c1_34240V3.1
locus=Pp3c1_34240 annot-version=v3.3	Physcomitrella_patens
OG0000018
Pp3c1_34210V3.1.p pacid=32967353 transcript=Pp3c1_34210V3.1
locus=Pp3c1_34210 annot-version=v3.3	Physcomitrella_patens
OG0000018
Pp3c12_19200V3.1.p pacid=32973257 transcript=Pp3c12_19200V3.1
locus=Pp3c12_19200 annot-version=v3.3	Physcomitrella_patens
OG0000018
Pp3c12_19190V3.1.p pacid=32974232 transcript=Pp3c12_19190V3.1

locus=Pp3c12_19190 annot-version=v3.3	Physcomitrella_patens
OG0000018
Pp3c6_6830V3.1.p pacid=32976041 transcript=Pp3c6_6830V3.1
locus=Pp3c6_6830 annot-version=v3.3	Physcomitrella_patens
OG0000018
Pp3c6_22090V3.1.p pacid=32977398 transcript=Pp3c6_22090V3.1
locus=Pp3c6_22090 annot-version=v3.3	Physcomitrella_patens
OG0000018
Pp3c6_12510V3.1.p pacid=32977471 transcript=Pp3c6_12510V3.1
locus=Pp3c6_12510 annot-version=v3.3	Physcomitrella_patens
OG0000018
Pp3c25_1690V3.1.p pacid=32980224 transcript=Pp3c25_1690V3.1
locus=Pp3c25_1690 annot-version=v3.3	Physcomitrella_patens
OG0000018
Pp3c18_8100V3.1.p pacid=32982902 transcript=Pp3c18_8100V3.1
locus=Pp3c18_8100 annot-version=v3.3	Physcomitrella_patens
OG0000018
Pp3c16_16040V3.1.p pacid=32984015 transcript=Pp3c16_16040V3.1
locus=Pp3c16_16040 annot-version=v3.3	Physcomitrella_patens
OG0000018
Pp3c10_25415V3.1.p pacid=32900747 transcript=Pp3c10_25415V3.1
locus=Pp3c10_25415 annot-version=v3.3	Physcomitrella_patens
OG0000018
Pp3c21_3950V3.1.p pacid=32916651 transcript=Pp3c21_3950V3.1
locus=Pp3c21_3950 annot-version=v3.3	Physcomitrella_patens
OG0000018
Pp3c26_12240V3.1.p pacid=32916969 transcript=Pp3c26_12240V3.1
locus=Pp3c26_12240 annot-version=v3.3	Physcomitrella_patens
OG0000018
Pp3c26_12230V3.1.p pacid=32916998 transcript=Pp3c26_12230V3.1
locus=Pp3c26_12230 annot-version=v3.3	Physcomitrella_patens
OG0000018
Pp3c26_6740V3.1.p pacid=32918457 transcript=Pp3c26_6740V3.1
locus=Pp3c26_6740 annot-version=v3.3	Physcomitrella_patens
OG0000018
Pp3c4_28410V3.1.p pacid=32919282 transcript=Pp3c4_28410V3.1
locus=Pp3c4_28410 annot-version=v3.3	Physcomitrella_patens
OG0000018
Pp3c4_11854V3.1.p pacid=32919378 transcript=Pp3c4_11854V3.1
locus=Pp3c4_11854 annot-version=v3.3	Physcomitrella_patens
OG0000018
Pp3c4_17240V3.1.p pacid=32919580 transcript=Pp3c4_17240V3.1
locus=Pp3c4_17240 annot-version=v3.3	Physcomitrella_patens
OG0000018
Pp3c10_25371V3.1.p pacid=32901668 transcript=Pp3c10_25371V3.1
locus=Pp3c10_25371 annot-version=v3.3	Physcomitrella_patens
OG0000018
Pp3c4_29150V3.1.p pacid=32922047 transcript=Pp3c4_29150V3.1
locus=Pp3c4_29150 annot-version=v3.3	Physcomitrella_patens
OG0000018
Pp3c4_29270V3.1.p pacid=32922180 transcript=Pp3c4_29270V3.1
locus=Pp3c4_29270 annot-version=v3.3	Physcomitrella_patens
OG0000018
Pp3c4_29130V3.1.p pacid=32922229 transcript=Pp3c4_29130V3.1

locus=Pp3c4_29130 annot-version=v3.3 Physcomitrella_patens
OG0000018
Pp3c4_28370V3.1.p pacid=32922857 transcript=Pp3c4_28370V3.1
locus=Pp3c4_28370 annot-version=v3.3 Physcomitrella_patens
OG0000018
Pp3c10_25360V3.1.p pacid=32899591 transcript=Pp3c10_25360V3.1
locus=Pp3c10_25360 annot-version=v3.3 Physcomitrella_patens
OG0000018
AT2G34420.1 | photosystem II light harvesting complex protein B1B2 |
Chr2:14522716-14523513 REVERSE LENGTH=265 | 201606
Arabidopsis_thaliana OG0000018
AT2G34430.1 | light-harvesting chlorophyll-protein complex II
subunit B1 | Chr2:14524818-14525618 FORWARD LENGTH=266 | 201606
Arabidopsis_thaliana OG0000018
AT3G27690.1 | photosystem II light harvesting complex protein 2.3 |
Chr3:10256002-10256921 FORWARD LENGTH=266 | 201606
Arabidopsis_thaliana OG0000018
AT3G47470.1 | light-harvesting chlorophyll-protein complex I subunit
A4 | Chr3:17493622-17494773 REVERSE LENGTH=251 | 201606
Arabidopsis_thaliana OG0000018
AT1G15820.1 | light harvesting complex photosystem II subunit 6 |
Chr1:5446685-5447676 REVERSE LENGTH=258 | 201606
Arabidopsis_thaliana OG0000018
AT3G54890.1 | chlorophyll a-b binding protein 6 |
Chr3:20339706-20340922 REVERSE LENGTH=241 | 201606
Arabidopsis_thaliana OG0000018
AT3G61470.1 | photosystem I light harvesting complex protein |
Chr3:22745736-22747032 FORWARD LENGTH=257 | 201606
Arabidopsis_thaliana OG0000018
AT4G10340.1 | light harvesting complex of photosystem II 5 |
Chr4:6408200-6409496 FORWARD LENGTH=280 | 201606
Arabidopsis_thaliana OG0000018
AT1G19150.1 | PSI type II chlorophyll a/b-binding protein (Lhca2-1)
| Chr1:6612806-6613799 FORWARD LENGTH=270 | 201606
Arabidopsis_thaliana OG0000018
AT5G28450.1 | Chlorophyll A-B binding family protein |
Chr5:10372978-10374190 REVERSE LENGTH=173 | 201606
Arabidopsis_thaliana OG0000018
AT5G54270.1 | light-harvesting chlorophyll B-binding protein 3 |
Chr5:22038424-22039383 FORWARD LENGTH=265 | 201606
Arabidopsis_thaliana OG0000018
AT1G29910.1 | chlorophyll A/B binding protein 3 |
Chr1:10472443-10473246 REVERSE LENGTH=267 | 201606
Arabidopsis_thaliana OG0000018
AT1G29920.1 | chlorophyll A/B-binding protein 2 |
Chr1:10475089-10475892 REVERSE LENGTH=267 | 201606
Arabidopsis_thaliana OG0000018
AT1G29930.1 | chlorophyll A/B binding protein 1 |
Chr1:10478071-10478874 FORWARD LENGTH=267 | 201606
Arabidopsis_thaliana OG0000018
AT1G45474.1 | photosystem I light harvesting complex protein 5 |
Chr1:17179353-17180439 FORWARD LENGTH=256 | 201606
Arabidopsis_thaliana OG0000018
AT1G61520.1 | PSI type III chlorophyll a/b-binding protein |

Chr1:22700152-22701149 FORWARD LENGTH=273 | 201606
Arabidopsis_thaliana   OG0000018
AT1G76570.1 | Chlorophyll A-B binding family protein |
Chr1:28729132-28730754 FORWARD LENGTH=327 | 201606
Arabidopsis_thaliana   OG0000018
AT2G05070.1 | photosystem II light harvesting complex protein 2.2 |
Chr2:1799436-1800329 REVERSE LENGTH=265 | 201606
Arabidopsis_thaliana   OG0000018
AT2G05100.1 | photosystem II light harvesting complex protein 2.1 |
Chr2:1823449-1824331 REVERSE LENGTH=265 | 201606
Arabidopsis_thaliana   OG0000018
Os03t0592500-01 Similar to Photosystem II type II chlorophyll a/b binding protein (Fragment).   Oryza_sativa   OG0000018
Os04t0457000-01 Similar to Chlorophyll a/b-binding protein CP24, photosystem II (Fragment).   Oryza_sativa   OG0000018
Os06t0320500-01 Similar to Light-harvesting complex I (Fragment).
Oryza_sativa   OG0000018
Os01t0600900-02 Chlorophyll a-b binding protein 2, chloroplast precursor (LHCII type I CAB-2) (LHCP).   Oryza_sativa
OG0000018
Os07t0562700-01 Similar to Type III chlorophyll a/b-binding protein (Fragment).   Oryza_sativa   OG0000018
Os07t0577600-01 Similar to Type II chlorophyll a/b binding protein from photosystem I precursor.   Oryza_sativa   OG0000018
Os08t0435900-01 Similar to LHC I type IV chlorophyll binding protein (Fragment).   Oryza_sativa   OG0000018
Os09t0296800-01 Chlorophyll A-B binding protein family protein.
Oryza_sativa   OG0000018
Os09t0346500-04 Similar to Chlorophyll a-b binding protein, chloroplast precursor (LHCII type I CAB) (LHCP).   Oryza_sativa
OG0000018
Os09t0439500-01 Similar to Type II chlorophyll a/b binding protein from photosystem I precursor.   Oryza_sativa   OG0000018
Os01t0720500-01 Similar to Type I chlorophyll a/b-binding protein b (Fragment).   Oryza_sativa   OG0000018
Os11t0242800-01 Similar to ASCAB9-A (ASCAB9-B) (Fragment).
Oryza_sativa   OG0000018
Os02t0197600-01 Chlorophyll a/b-binding protein type III (Fragment).
Oryza_sativa   OG0000018
Os02t0764500-01 Similar to Lhca5 protein. Oryza_sativa
OG0000018
CMN234CT pep chromosome:ASM9120v1:14:578489:579158:-1 gene:CMN234C transcript:CMN234CT gene_biotype:protein_coding transcript_biotype:protein_coding Cyanidioschyzon_merolae
OG0000018
CMQ142CT pep chromosome:ASM9120v1:17:337674:338319:-1 gene:CMQ142C transcript:CMQ142CT gene_biotype:protein_coding transcript_biotype:protein_coding Cyanidioschyzon_merolae
OG0000018
evm.model.contig_2142.1 Porphyridium_purpureum   OG0000018
evm.model.contig_2493.3 Porphyridium_purpureum   OG0000018
evm.model.contig_3421.1 Porphyridium_purpureum   OG0000018
evm.model.contig_435.19 Porphyridium_purpureum   OG0000018
evm.model.contig_4406.7 Porphyridium_purpureum   OG0000018

evm.model.contig_491.7	Porphyridium_purpureum	OG0000018
CDF41051 pep supercontig:ASM35022v2:HG002301:166255:167303:-1
gene:CHC_T00007661001 transcript:CDF41051
gene_biotype:protein_coding transcript_biotype:protein_coding
Chondrus_crispus OG0000018
CDF39753 pep supercontig:ASM35022v2:HG002070:368031:369084:1
gene:CHC_T00006793001 transcript:CDF39753
gene_biotype:protein_coding transcript_biotype:protein_coding
Chondrus_crispus OG0000018
CDF39364 pep supercontig:ASM35022v2:HG002023:35361:36737:-1
gene:CHC_T00008505001 transcript:CDF39364
gene_biotype:protein_coding transcript_biotype:protein_coding
description:Light-harvest protein Chondrus_crispus OG0000018
CDF39790 pep supercontig:ASM35022v2:HG002073:22537:23343:-1
gene:CHC_T00009105001 transcript:CDF39790
gene_biotype:protein_coding transcript_biotype:protein_coding
description:Light-harvesting complex I polypeptide Chondrus_crispus
OG0000018
g4228.t1 Caulerpa_lentillifera	OG0000019
g4446.t1 Caulerpa_lentillifera	OG0000019
g7348.t1 Caulerpa_lentillifera	OG0000019
Cre02.g093051.t1.1 pacid=30785068 transcript=Cre02.g093051.t1.1
locus=Cre02.g093051 ID=Cre02.g093051.t1.1.v5.5 annot-version=v5.5
Chlamydomonas_reinhardtii OG0000019
Cre02.g095034.t1.1 pacid=30785146 transcript=Cre02.g095034.t1.1
locus=Cre02.g095034 ID=Cre02.g095034.t1.1.v5.5 annot-version=v5.5
Chlamydomonas_reinhardtii OG0000019
Cre02.g095035.t1.1 pacid=30785710 transcript=Cre02.g095035.t1.1
locus=Cre02.g095035 ID=Cre02.g095035.t1.1.v5.5 annot-version=v5.5
Chlamydomonas_reinhardtii OG0000019
Cre02.g093084.t1.1 pacid=30785970 transcript=Cre02.g093084.t1.1
locus=Cre02.g093084 ID=Cre02.g093084.t1.1.v5.5 annot-version=v5.5
Chlamydomonas_reinhardtii OG0000019
Cre02.g095032.t1.1 pacid=30786061 transcript=Cre02.g095032.t1.1
locus=Cre02.g095032 ID=Cre02.g095032.t1.1.v5.5 annot-version=v5.5
Chlamydomonas_reinhardtii OG0000019
Cre02.g093252.t1.1 pacid=30786219 transcript=Cre02.g093252.t1.1
locus=Cre02.g093252 ID=Cre02.g093252.t1.1.v5.5 annot-version=v5.5
Chlamydomonas_reinhardtii OG0000019
Cre02.g095036.t1.1 pacid=30786226 transcript=Cre02.g095036.t1.1
locus=Cre02.g095036 ID=Cre02.g095036.t1.1.v5.5 annot-version=v5.5
Chlamydomonas_reinhardtii OG0000019
Cre02.g095033.t1.1 pacid=30786311 transcript=Cre02.g095033.t1.1
locus=Cre02.g095033 ID=Cre02.g095033.t1.1.v5.5 annot-version=v5.5
Chlamydomonas_reinhardtii OG0000019
Cre02.g093017.t1.1 pacid=30786390 transcript=Cre02.g093017.t1.1
locus=Cre02.g093017 ID=Cre02.g093017.t1.1.v5.5 annot-version=v5.5
Chlamydomonas_reinhardtii OG0000019
Cre02.g092983.t1.1 pacid=30786430 transcript=Cre02.g092983.t1.1
locus=Cre02.g092983 ID=Cre02.g092983.t1.1.v5.5 annot-version=v5.5
Chlamydomonas_reinhardtii OG0000019
Cre27.g757197.t1.1 pacid=30786510 transcript=Cre27.g757197.t1.1
locus=Cre27.g757197 ID=Cre27.g757197.t1.1.v5.5 annot-version=v5.5
Chlamydomonas_reinhardtii OG0000019

Cre27.g757347.t1.1 pacid=30786511 transcript=Cre27.g757347.t1.1 locus=Cre27.g757347 ID=Cre27.g757347.t1.1.v5.5 annot-version=v5.5 Chlamydomonas_reinhardtii OG0000019
Cre27.g757147.t1.1 pacid=30786513 transcript=Cre27.g757147.t1.1 locus=Cre27.g757147 ID=Cre27.g757147.t1.1.v5.5 annot-version=v5.5 Chlamydomonas_reinhardtii OG0000019
Cre27.g757447.t1.1 pacid=30786514 transcript=Cre27.g757447.t1.1 locus=Cre27.g757447 ID=Cre27.g757447.t1.1.v5.5 annot-version=v5.5 Chlamydomonas_reinhardtii OG0000019
Cre27.g757297.t1.1 pacid=30786517 transcript=Cre27.g757297.t1.1 locus=Cre27.g757297 ID=Cre27.g757297.t1.1.v5.5 annot-version=v5.5 Chlamydomonas_reinhardtii OG0000019
Cre27.g757397.t1.1 pacid=30786518 transcript=Cre27.g757397.t1.1 locus=Cre27.g757397 ID=Cre27.g757397.t1.1.v5.5 annot-version=v5.5 Chlamydomonas_reinhardtii OG0000019
Cre27.g757247.t1.1 pacid=30786519 transcript=Cre27.g757247.t1.1 locus=Cre27.g757247 ID=Cre27.g757247.t1.1.v5.5 annot-version=v5.5 Chlamydomonas_reinhardtii OG0000019
Cre03.g198467.t1.1 pacid=30786577 transcript=Cre03.g198467.t1.1 locus=Cre03.g198467 ID=Cre03.g198467.t1.1.v5.5 annot-version=v5.5 Chlamydomonas_reinhardtii OG0000019
Cre03.g208551.t1.1 pacid=30786628 transcript=Cre03.g208551.t1.1 locus=Cre03.g208551 ID=Cre03.g208551.t1.1.v5.5 annot-version=v5.5 Chlamydomonas_reinhardtii OG0000019
Cre03.g208555.t1.1 pacid=30786634 transcript=Cre03.g208555.t1.1 locus=Cre03.g208555 ID=Cre03.g208555.t1.1.v5.5 annot-version=v5.5 Chlamydomonas_reinhardtii OG0000019
Cre03.g208554.t1.1 pacid=30786778 transcript=Cre03.g208554.t1.1 locus=Cre03.g208554 ID=Cre03.g208554.t1.1.v5.5 annot-version=v5.5 Chlamydomonas_reinhardtii OG0000019
Cre03.g208305.t1.1 pacid=30786813 transcript=Cre03.g208305.t1.1 locus=Cre03.g208305 ID=Cre03.g208305.t1.1.v5.5 annot-version=v5.5 Chlamydomonas_reinhardtii OG0000019
Cre03.g208303.t1.1 pacid=30787048 transcript=Cre03.g208303.t1.1 locus=Cre03.g208303 ID=Cre03.g208303.t1.1.v5.5 annot-version=v5.5 Chlamydomonas_reinhardtii OG0000019
Cre03.g206087.t1.1 pacid=30787071 transcript=Cre03.g206087.t1.1 locus=Cre03.g206087 ID=Cre03.g206087.t1.1.v5.5 annot-version=v5.5 Chlamydomonas_reinhardtii OG0000019
Cre03.g208556.t1.1 pacid=30787542 transcript=Cre03.g208556.t1.1 locus=Cre03.g208556 ID=Cre03.g208556.t1.1.v5.5 annot-version=v5.5 Chlamydomonas_reinhardtii OG0000019
Cre03.g208553.t1.1 pacid=30787633 transcript=Cre03.g208553.t1.1 locus=Cre03.g208553 ID=Cre03.g208553.t1.1.v5.5 annot-version=v5.5 Chlamydomonas_reinhardtii OG0000019
Cre03.g208306.t1.1 pacid=30787794 transcript=Cre03.g208306.t1.1 locus=Cre03.g208306 ID=Cre03.g208306.t1.1.v5.5 annot-version=v5.5 Chlamydomonas_reinhardtii OG0000019
Cre03.g208304.t1.1 pacid=30788023 transcript=Cre03.g208304.t1.1 locus=Cre03.g208304 ID=Cre03.g208304.t1.1.v5.5 annot-version=v5.5 Chlamydomonas_reinhardtii OG0000019
Cre03.g198288.t2.1 pacid=30788254 transcript=Cre03.g198288.t2.1 locus=Cre03.g198288 ID=Cre03.g198288.t2.1.v5.5 annot-version=v5.5 Chlamydomonas_reinhardtii OG0000019

Cre01.g006700.t1.2 pacid=30788354 transcript=Cre01.g006700.t1.2 locus=Cre01.g006700 ID=Cre01.g006700.t1.2.v5.5 annot-version=v5.5
Chlamydomonas_reinhardtii OG0000019
Cre01.g006670.t1.1 pacid=30788720 transcript=Cre01.g006670.t1.1 locus=Cre01.g006670 ID=Cre01.g006670.t1.1.v5.5 annot-version=v5.5
Chlamydomonas_reinhardtii OG0000019
Cre01.g006600.t1.2 pacid=30788808 transcript=Cre01.g006600.t1.2 locus=Cre01.g006600 ID=Cre01.g006600.t1.2.v5.5 annot-version=v5.5
Chlamydomonas_reinhardtii OG0000019
Cre01.g006628.t1.1 pacid=30788937 transcript=Cre01.g006628.t1.1 locus=Cre01.g006628 ID=Cre01.g006628.t1.1.v5.5 annot-version=v5.5
Chlamydomonas_reinhardtii OG0000019
Cre01.g006576.t1.1 pacid=30789322 transcript=Cre01.g006576.t1.1 locus=Cre01.g006576 ID=Cre01.g006576.t1.1.v5.5 annot-version=v5.5
Chlamydomonas_reinhardtii OG0000019
Cre01.g006656.t1.1 pacid=30789463 transcript=Cre01.g006656.t1.1 locus=Cre01.g006656 ID=Cre01.g006656.t1.1.v5.5 annot-version=v5.5
Chlamydomonas_reinhardtii OG0000019
Cre07.g333816.t1.1 pacid=30774963 transcript=Cre07.g333816.t1.1 locus=Cre07.g333816 ID=Cre07.g333816.t1.1.v5.5 annot-version=v5.5
Chlamydomonas_reinhardtii OG0000019
Cre07.g333570.t1.1 pacid=30774964 transcript=Cre07.g333570.t1.1 locus=Cre07.g333570 ID=Cre07.g333570.t1.1.v5.5 annot-version=v5.5
Chlamydomonas_reinhardtii OG0000019
Cre01.g006614.t1.1 pacid=30789666 transcript=Cre01.g006614.t1.1 locus=Cre01.g006614 ID=Cre01.g006614.t1.1.v5.5 annot-version=v5.5
Chlamydomonas_reinhardtii OG0000019
Cre10.g445251.t1.1 pacid=30789802 transcript=Cre10.g445251.t1.1 locus=Cre10.g445251 ID=Cre10.g445251.t1.1.v5.5 annot-version=v5.5
Chlamydomonas_reinhardtii OG0000019
Cre10.g445226.t1.1 pacid=30789927 transcript=Cre10.g445226.t1.1 locus=Cre10.g445226 ID=Cre10.g445226.t1.1.v5.5 annot-version=v5.5
Chlamydomonas_reinhardtii OG0000019
Cre10.g445347.t1.1 pacid=30790126 transcript=Cre10.g445347.t1.1 locus=Cre10.g445347 ID=Cre10.g445347.t1.1.v5.5 annot-version=v5.5
Chlamydomonas_reinhardtii OG0000019
Cre10.g445299.t1.1 pacid=30790274 transcript=Cre10.g445299.t1.1 locus=Cre10.g445299 ID=Cre10.g445299.t1.1.v5.5 annot-version=v5.5
Chlamydomonas_reinhardtii OG0000019
Cre10.g445443.t1.1 pacid=30790324 transcript=Cre10.g445443.t1.1 locus=Cre10.g445443 ID=Cre10.g445443.t1.1.v5.5 annot-version=v5.5
Chlamydomonas_reinhardtii OG0000019
Cre10.g445275.t1.1 pacid=30790501 transcript=Cre10.g445275.t1.1 locus=Cre10.g445275 ID=Cre10.g445275.t1.1.v5.5 annot-version=v5.5
Chlamydomonas_reinhardtii OG0000019
Cre07.g333675.t1.1 pacid=30775114 transcript=Cre07.g333675.t1.1 locus=Cre07.g333675 ID=Cre07.g333675.t1.1.v5.5 annot-version=v5.5
Chlamydomonas_reinhardtii OG0000019
Cre04.g217986.t1.1 pacid=30790991 transcript=Cre04.g217986.t1.1 locus=Cre04.g217986 ID=Cre04.g217986.t1.1.v5.5 annot-version=v5.5
Chlamydomonas_reinhardtii OG0000019
Cre04.g217965.t1.1 pacid=30791128 transcript=Cre04.g217965.t1.1 locus=Cre04.g217965 ID=Cre04.g217965.t1.1.v5.5 annot-version=v5.5
Chlamydomonas_reinhardtii OG0000019

Cre04.g217984.t1.1 pacid=30791203 transcript=Cre04.g217984.t1.1 locus=Cre04.g217984 ID=Cre04.g217984.t1.1.v5.5 annot-version=v5.5 Chlamydomonas_reinhardtii OG0000019
Cre04.g217990.t1.1 pacid=30791393 transcript=Cre04.g217990.t1.1 locus=Cre04.g217990 ID=Cre04.g217990.t1.1.v5.5 annot-version=v5.5 Chlamydomonas_reinhardtii OG0000019
Cre04.g217978.t1.1 pacid=30791482 transcript=Cre04.g217978.t1.1 locus=Cre04.g217978 ID=Cre04.g217978.t1.1.v5.5 annot-version=v5.5 Chlamydomonas_reinhardtii OG0000019
Cre04.g217972.t1.1 pacid=30791592 transcript=Cre04.g217972.t1.1 locus=Cre04.g217972 ID=Cre04.g217972.t1.1.v5.5 annot-version=v5.5 Chlamydomonas_reinhardtii OG0000019
Cre21.g753097.t1.1 pacid=30791616 transcript=Cre21.g753097.t1.1 locus=Cre21.g753097 ID=Cre21.g753097.t1.1.v5.5 annot-version=v5.5 Chlamydomonas_reinhardtii OG0000019
Cre21.g753147.t1.1 pacid=30791633 transcript=Cre21.g753147.t1.1 locus=Cre21.g753147 ID=Cre21.g753147.t1.1.v5.5 annot-version=v5.5 Chlamydomonas_reinhardtii OG0000019
Cre21.g753197.t1.1 pacid=30791640 transcript=Cre21.g753197.t1.1 locus=Cre21.g753197 ID=Cre21.g753197.t1.1.v5.5 annot-version=v5.5 Chlamydomonas_reinhardtii OG0000019
Cre12.g539202.t1.1 pacid=30791784 transcript=Cre12.g539202.t1.1 locus=Cre12.g539202 ID=Cre12.g539202.t1.1.v5.5 annot-version=v5.5 Chlamydomonas_reinhardtii OG0000019
Cre12.g519401.t1.1 pacid=30791916 transcript=Cre12.g519401.t1.1 locus=Cre12.g519401 ID=Cre12.g519401.t1.1.v5.5 annot-version=v5.5 Chlamydomonas_reinhardtii OG0000019
Cre12.g539203.t1.1 pacid=30791929 transcript=Cre12.g539203.t1.1 locus=Cre12.g539203 ID=Cre12.g539203.t1.1.v5.5 annot-version=v5.5 Chlamydomonas_reinhardtii OG0000019
Cre12.g539150.t1.2 pacid=30792074 transcript=Cre12.g539150.t1.2 locus=Cre12.g539150 ID=Cre12.g539150.t1.2.v5.5 annot-version=v5.5 Chlamydomonas_reinhardtii OG0000019
Cre12.g539204.t1.1 pacid=30792220 transcript=Cre12.g539204.t1.1 locus=Cre12.g539204 ID=Cre12.g539204.t1.1.v5.5 annot-version=v5.5 Chlamydomonas_reinhardtii OG0000019
Cre12.g539207.t1.1 pacid=30792293 transcript=Cre12.g539207.t1.1 locus=Cre12.g539207 ID=Cre12.g539207.t1.1.v5.5 annot-version=v5.5 Chlamydomonas_reinhardtii OG0000019
Cre12.g539208.t1.1 pacid=30792345 transcript=Cre12.g539208.t1.1 locus=Cre12.g539208 ID=Cre12.g539208.t1.1.v5.5 annot-version=v5.5 Chlamydomonas_reinhardtii OG0000019
Cre12.g517925.t1.1 pacid=30792678 transcript=Cre12.g517925.t1.1 locus=Cre12.g517925 ID=Cre12.g517925.t1.1.v5.5 annot-version=v5.5 Chlamydomonas_reinhardtii OG0000019
Cre12.g546633.t1.1 pacid=30792696 transcript=Cre12.g546633.t1.1 locus=Cre12.g546633 ID=Cre12.g546633.t1.1.v5.5 annot-version=v5.5 Chlamydomonas_reinhardtii OG0000019
Cre12.g539209.t1.1 pacid=30792928 transcript=Cre12.g539209.t1.1 locus=Cre12.g539209 ID=Cre12.g539209.t1.1.v5.5 annot-version=v5.5 Chlamydomonas_reinhardtii OG0000019
Cre12.g539206.t1.1 pacid=30793167 transcript=Cre12.g539206.t1.1 locus=Cre12.g539206 ID=Cre12.g539206.t1.1.v5.5 annot-version=v5.5 Chlamydomonas_reinhardtii OG0000019

Cre12.g539161.t1.1 pacid=30793247 transcript=Cre12.g539161.t1.1 locus=Cre12.g539161 ID=Cre12.g539161.t1.1.v5.5 annot-version=v5.5
Chlamydomonas_reinhardtii OG0000019
Cre12.g554116.t1.1 pacid=30793325 transcript=Cre12.g554116.t1.1 locus=Cre12.g554116 ID=Cre12.g554116.t1.1.v5.5 annot-version=v5.5
Chlamydomonas_reinhardtii OG0000019
Cre07.g333640.t1.1 pacid=30775433 transcript=Cre07.g333640.t1.1 locus=Cre07.g333640 ID=Cre07.g333640.t1.1.v5.5 annot-version=v5.5
Chlamydomonas_reinhardtii OG0000019
Cre08.g373436.t1.1 pacid=30773373 transcript=Cre08.g373436.t1.1 locus=Cre08.g373436 ID=Cre08.g373436.t1.1.v5.5 annot-version=v5.5
Chlamydomonas_reinhardtii OG0000019
Cre11.g467522.t1.1 pacid=30775957 transcript=Cre11.g467522.t1.1 locus=Cre11.g467522 ID=Cre11.g467522.t1.1.v5.5 annot-version=v5.5
Chlamydomonas_reinhardtii OG0000019
Cre08.g373428.t1.1 pacid=30773618 transcript=Cre08.g373428.t1.1 locus=Cre08.g373428 ID=Cre08.g373428.t1.1.v5.5 annot-version=v5.5
Chlamydomonas_reinhardtii OG0000019
Cre11.g467523.t1.1 pacid=30776042 transcript=Cre11.g467523.t1.1 locus=Cre11.g467523 ID=Cre11.g467523.t1.1.v5.5 annot-version=v5.5
Chlamydomonas_reinhardtii OG0000019
Cre14.g622270.t1.1 pacid=30776187 transcript=Cre14.g622270.t1.1 locus=Cre14.g622270 ID=Cre14.g622270.t1.1.v5.5 annot-version=v5.5
Chlamydomonas_reinhardtii OG0000019
Cre14.g622340.t1.1 pacid=30776554 transcript=Cre14.g622340.t1.1 locus=Cre14.g622340 ID=Cre14.g622340.t1.1.v5.5 annot-version=v5.5
Chlamydomonas_reinhardtii OG0000019
Cre14.g622410.t1.1 pacid=30776655 transcript=Cre14.g622410.t1.1 locus=Cre14.g622410 ID=Cre14.g622410.t1.1.v5.5 annot-version=v5.5
Chlamydomonas_reinhardtii OG0000019
Cre16.g669905.t1.1 pacid=30777010 transcript=Cre16.g669905.t1.1 locus=Cre16.g669905 ID=Cre16.g669905.t1.1.v5.5 annot-version=v5.5
Chlamydomonas_reinhardtii OG0000019
Cre16.g660550.t1.2 pacid=30777194 transcript=Cre16.g660550.t1.2 locus=Cre16.g660550 ID=Cre16.g660550.t1.2.v5.5 annot-version=v5.5
Chlamydomonas_reinhardtii OG0000019
Cre16.g668837.t1.1 pacid=30777239 transcript=Cre16.g668837.t1.1 locus=Cre16.g668837 ID=Cre16.g668837.t1.1.v5.5 annot-version=v5.5
Chlamydomonas_reinhardtii OG0000019
Cre16.g667769.t1.1 pacid=30777337 transcript=Cre16.g667769.t1.1 locus=Cre16.g667769 ID=Cre16.g667769.t1.1.v5.5 annot-version=v5.5
Chlamydomonas_reinhardtii OG0000019
Cre16.g666677.t1.1 pacid=30777346 transcript=Cre16.g666677.t1.1 locus=Cre16.g666677 ID=Cre16.g666677.t1.1.v5.5 annot-version=v5.5
Chlamydomonas_reinhardtii OG0000019
Cre16.g666602.t1.1 pacid=30777360 transcript=Cre16.g666602.t1.1 locus=Cre16.g666602 ID=Cre16.g666602.t1.1.v5.5 annot-version=v5.5
Chlamydomonas_reinhardtii OG0000019
Cre16.g667413.t1.1 pacid=30777368 transcript=Cre16.g667413.t1.1 locus=Cre16.g667413 ID=Cre16.g667413.t1.1.v5.5 annot-version=v5.5
Chlamydomonas_reinhardtii OG0000019
Cre16.g667057.t1.1 pacid=30777654 transcript=Cre16.g667057.t1.1 locus=Cre16.g667057 ID=Cre16.g667057.t1.1.v5.5 annot-version=v5.5
Chlamydomonas_reinhardtii OG0000019

Cre16.g666627.t1.1 pacid=30777698 transcript=Cre16.g666627.t1.1 locus=Cre16.g666627 ID=Cre16.g666627.t1.1.v5.5 annot-version=v5.5 Chlamydomonas_reinhardtii OG0000019
Cre16.g668481.t1.1 pacid=30777736 transcript=Cre16.g668481.t1.1 locus=Cre16.g668481 ID=Cre16.g668481.t1.1.v5.5 annot-version=v5.5 Chlamydomonas_reinhardtii OG0000019
Cre16.g668125.t1.1 pacid=30777747 transcript=Cre16.g668125.t1.1 locus=Cre16.g668125 ID=Cre16.g668125.t1.1.v5.5 annot-version=v5.5 Chlamydomonas_reinhardtii OG0000019
Cre16.g666700.t1.2 pacid=30777758 transcript=Cre16.g666700.t1.2 locus=Cre16.g666700 ID=Cre16.g666700.t1.2.v5.5 annot-version=v5.5 Chlamydomonas_reinhardtii OG0000019
Cre16.g669549.t1.1 pacid=30777811 transcript=Cre16.g669549.t1.1 locus=Cre16.g669549 ID=Cre16.g669549.t1.1.v5.5 annot-version=v5.5 Chlamydomonas_reinhardtii OG0000019
Cre08.g373421.t1.1 pacid=30773836 transcript=Cre08.g373421.t1.1 locus=Cre08.g373421 ID=Cre08.g373421.t1.1.v5.5 annot-version=v5.5 Chlamydomonas_reinhardtii OG0000019
Cre16.g685451.t1.1 pacid=30778160 transcript=Cre16.g685451.t1.1 locus=Cre16.g685451 ID=Cre16.g685451.t1.1.v5.5 annot-version=v5.5 Chlamydomonas_reinhardtii OG0000019
Cre16.g666653.t1.2 pacid=30778242 transcript=Cre16.g666653.t1.2 locus=Cre16.g666653 ID=Cre16.g666653.t1.2.v5.5 annot-version=v5.5 Chlamydomonas_reinhardtii OG0000019
Cre16.g669193.t1.1 pacid=30778248 transcript=Cre16.g669193.t1.1 locus=Cre16.g669193 ID=Cre16.g669193.t1.1.v5.5 annot-version=v5.5 Chlamydomonas_reinhardtii OG0000019
Cre34.g759097.t1.1 pacid=30778366 transcript=Cre34.g759097.t1.1 locus=Cre34.g759097 ID=Cre34.g759097.t1.1.v5.5 annot-version=v5.5 Chlamydomonas_reinhardtii OG0000019
Cre06.g278299.t1.1 pacid=30778475 transcript=Cre06.g278299.t1.1 locus=Cre06.g278299 ID=Cre06.g278299.t1.1.v5.5 annot-version=v5.5 Chlamydomonas_reinhardtii OG0000019
Cre06.g278290.t1.1 pacid=30778632 transcript=Cre06.g278290.t1.1 locus=Cre06.g278290 ID=Cre06.g278290.t1.1.v5.5 annot-version=v5.5 Chlamydomonas_reinhardtii OG0000019
Cre06.g278285.t1.1 pacid=30778642 transcript=Cre06.g278285.t1.1 locus=Cre06.g278285 ID=Cre06.g278285.t1.1.v5.5 annot-version=v5.5 Chlamydomonas_reinhardtii OG0000019
Cre06.g278302.t1.1 pacid=30778766 transcript=Cre06.g278302.t1.1 locus=Cre06.g278302 ID=Cre06.g278302.t1.1.v5.5 annot-version=v5.5 Chlamydomonas_reinhardtii OG0000019
Cre06.g278286.t1.1 pacid=30778824 transcript=Cre06.g278286.t1.1 locus=Cre06.g278286 ID=Cre06.g278286.t1.1.v5.5 annot-version=v5.5 Chlamydomonas_reinhardtii OG0000019
Cre06.g278275.t1.1 pacid=30778870 transcript=Cre06.g278275.t1.1 locus=Cre06.g278275 ID=Cre06.g278275.t1.1.v5.5 annot-version=v5.5 Chlamydomonas_reinhardtii OG0000019
Cre06.g278273.t1.1 pacid=30778902 transcript=Cre06.g278273.t1.1 locus=Cre06.g278273 ID=Cre06.g278273.t1.1.v5.5 annot-version=v5.5 Chlamydomonas_reinhardtii OG0000019
Cre06.g278283.t1.1 pacid=30778990 transcript=Cre06.g278283.t1.1 locus=Cre06.g278283 ID=Cre06.g278283.t1.1.v5.5 annot-version=v5.5 Chlamydomonas_reinhardtii OG0000019

Cre06.g278274.t1.1 pacid=30779107 transcript=Cre06.g278274.t1.1 locus=Cre06.g278274 ID=Cre06.g278274.t1.1.v5.5 annot-version=v5.5
Chlamydomonas_reinhardtii OG0000019
Cre06.g278272.t1.1 pacid=30779139 transcript=Cre06.g278272.t1.1 locus=Cre06.g278272 ID=Cre06.g278272.t1.1.v5.5 annot-version=v5.5
Chlamydomonas_reinhardtii OG0000019
Cre08.g373414.t1.1 pacid=30773948 transcript=Cre08.g373414.t1.1 locus=Cre08.g373414 ID=Cre08.g373414.t1.1.v5.5 annot-version=v5.5
Chlamydomonas_reinhardtii OG0000019
Cre06.g246958.t1.1 pacid=30779511 transcript=Cre06.g246958.t1.1 locus=Cre06.g246958 ID=Cre06.g246958.t1.1.v5.5 annot-version=v5.5
Chlamydomonas_reinhardtii OG0000019
Cre06.g278276.t1.1 pacid=30779593 transcript=Cre06.g278276.t1.1 locus=Cre06.g278276 ID=Cre06.g278276.t1.1.v5.5 annot-version=v5.5
Chlamydomonas_reinhardtii OG0000019
Cre06.g278289.t1.1 pacid=30779618 transcript=Cre06.g278289.t1.1 locus=Cre06.g278289 ID=Cre06.g278289.t1.1.v5.5 annot-version=v5.5
Chlamydomonas_reinhardtii OG0000019
Cre06.g278287.t1.1 pacid=30779770 transcript=Cre06.g278287.t1.1 locus=Cre06.g278287 ID=Cre06.g278287.t1.1.v5.5 annot-version=v5.5
Chlamydomonas_reinhardtii OG0000019
Cre06.g278284.t1.1 pacid=30779849 transcript=Cre06.g278284.t1.1 locus=Cre06.g278284 ID=Cre06.g278284.t1.1.v5.5 annot-version=v5.5
Chlamydomonas_reinhardtii OG0000019
Cre06.g278282.t1.1 pacid=30779861 transcript=Cre06.g278282.t1.1 locus=Cre06.g278282 ID=Cre06.g278282.t1.1.v5.5 annot-version=v5.5
Chlamydomonas_reinhardtii OG0000019
Cre06.g278296.t1.1 pacid=30779938 transcript=Cre06.g278296.t1.1 locus=Cre06.g278296 ID=Cre06.g278296.t1.1.v5.5 annot-version=v5.5
Chlamydomonas_reinhardtii OG0000019
Cre06.g278291.t1.1 pacid=30779974 transcript=Cre06.g278291.t1.1 locus=Cre06.g278291 ID=Cre06.g278291.t1.1.v5.5 annot-version=v5.5
Chlamydomonas_reinhardtii OG0000019
Cre06.g278280.t1.1 pacid=30780008 transcript=Cre06.g278280.t1.1 locus=Cre06.g278280 ID=Cre06.g278280.t1.1.v5.5 annot-version=v5.5
Chlamydomonas_reinhardtii OG0000019
Cre09.g400108.t1.1 pacid=30780523 transcript=Cre09.g400108.t1.1 locus=Cre09.g400108 ID=Cre09.g400108.t1.1.v5.5 annot-version=v5.5
Chlamydomonas_reinhardtii OG0000019
Cre09.g400145.t1.1 pacid=30780759 transcript=Cre09.g400145.t1.1 locus=Cre09.g400145 ID=Cre09.g400145.t1.1.v5.5 annot-version=v5.5
Chlamydomonas_reinhardtii OG0000019
Cre09.g399886.t1.1 pacid=30780828 transcript=Cre09.g399886.t1.1 locus=Cre09.g399886 ID=Cre09.g399886.t1.1.v5.5 annot-version=v5.5
Chlamydomonas_reinhardtii OG0000019
Cre09.g399923.t1.1 pacid=30781011 transcript=Cre09.g399923.t1.1 locus=Cre09.g399923 ID=Cre09.g399923.t1.1.v5.5 annot-version=v5.5
Chlamydomonas_reinhardtii OG0000019
Cre09.g399960.t1.1 pacid=30781162 transcript=Cre09.g399960.t1.1 locus=Cre09.g399960 ID=Cre09.g399960.t1.1.v5.5 annot-version=v5.5
Chlamydomonas_reinhardtii OG0000019
Cre09.g399997.t1.1 pacid=30781258 transcript=Cre09.g399997.t1.1 locus=Cre09.g399997 ID=Cre09.g399997.t1.1.v5.5 annot-version=v5.5
Chlamydomonas_reinhardtii OG0000019

Cre09.g400071.t1.1 pacid=30781287 transcript=Cre09.g400071.t1.1 locus=Cre09.g400071 ID=Cre09.g400071.t1.1.v5.5 annot-version=v5.5 Chlamydomonas_reinhardtii OG0000019
Cre09.g413566.t1.1 pacid=30781299 transcript=Cre09.g413566.t1.1 locus=Cre09.g413566 ID=Cre09.g413566.t1.1.v5.5 annot-version=v5.5 Chlamydomonas_reinhardtii OG0000019
Cre09.g400182.t1.1 pacid=30781316 transcript=Cre09.g400182.t1.1 locus=Cre09.g400182 ID=Cre09.g400182.t1.1.v5.5 annot-version=v5.5 Chlamydomonas_reinhardtii OG0000019
Cre09.g399775.t1.1 pacid=30781343 transcript=Cre09.g399775.t1.1 locus=Cre09.g399775 ID=Cre09.g399775.t1.1.v5.5 annot-version=v5.5 Chlamydomonas_reinhardtii OG0000019
Cre09.g400034.t1.1 pacid=30781393 transcript=Cre09.g400034.t1.1 locus=Cre09.g400034 ID=Cre09.g400034.t1.1.v5.5 annot-version=v5.5 Chlamydomonas_reinhardtii OG0000019
Cre30.g758197.t1.1 pacid=30781559 transcript=Cre30.g758197.t1.1 locus=Cre30.g758197 ID=Cre30.g758197.t1.1.v5.5 annot-version=v5.5 Chlamydomonas_reinhardtii OG0000019
Cre30.g757997.t1.1 pacid=30781561 transcript=Cre30.g757997.t1.1 locus=Cre30.g757997 ID=Cre30.g757997.t1.1.v5.5 annot-version=v5.5 Chlamydomonas_reinhardtii OG0000019
Cre30.g758147.t1.1 pacid=30781563 transcript=Cre30.g758147.t1.1 locus=Cre30.g758147 ID=Cre30.g758147.t1.1.v5.5 annot-version=v5.5 Chlamydomonas_reinhardtii OG0000019
Cre40.g760247.t1.1 pacid=30781575 transcript=Cre40.g760247.t1.1 locus=Cre40.g760247 ID=Cre40.g760247.t1.1.v5.5 annot-version=v5.5 Chlamydomonas_reinhardtii OG0000019
Cre17.g743269.t1.1 pacid=30781713 transcript=Cre17.g743269.t1.1 locus=Cre17.g743269 ID=Cre17.g743269.t1.1.v5.5 annot-version=v5.5 Chlamydomonas_reinhardtii OG0000019
Cre17.g748047.t1.1 pacid=30781801 transcript=Cre17.g748047.t1.1 locus=Cre17.g748047 ID=Cre17.g748047.t1.1.v5.5 annot-version=v5.5 Chlamydomonas_reinhardtii OG0000019
Cre17.g743497.t1.1 pacid=30781942 transcript=Cre17.g743497.t1.1 locus=Cre17.g743497 ID=Cre17.g743497.t1.1.v5.5 annot-version=v5.5 Chlamydomonas_reinhardtii OG0000019
Cre17.g742866.t1.1 pacid=30782526 transcript=Cre17.g742866.t1.1 locus=Cre17.g742866 ID=Cre17.g742866.t1.1.v5.5 annot-version=v5.5 Chlamydomonas_reinhardtii OG0000019
Cre17.g747997.t1.1 pacid=30782529 transcript=Cre17.g747997.t1.1 locus=Cre17.g747997 ID=Cre17.g747997.t1.1.v5.5 annot-version=v5.5 Chlamydomonas_reinhardtii OG0000019
Cre17.g743447.t1.1 pacid=30782694 transcript=Cre17.g743447.t1.1 locus=Cre17.g743447 ID=Cre17.g743447.t1.1.v5.5 annot-version=v5.5 Chlamydomonas_reinhardtii OG0000019
Cre17.g743130.t1.1 pacid=30782808 transcript=Cre17.g743130.t1.1 locus=Cre17.g743130 ID=Cre17.g743130.t1.1.v5.5 annot-version=v5.5 Chlamydomonas_reinhardtii OG0000019
Cre28.g757747.t1.1 pacid=30782924 transcript=Cre28.g757747.t1.1 locus=Cre28.g757747 ID=Cre28.g757747.t1.1.v5.5 annot-version=v5.5 Chlamydomonas_reinhardtii OG0000019
Cre28.g757647.t1.1 pacid=30782927 transcript=Cre28.g757647.t1.1 locus=Cre28.g757647 ID=Cre28.g757647.t1.1.v5.5 annot-version=v5.5 Chlamydomonas_reinhardtii OG0000019

Cre28.g757697.t1.1 pacid=30782928 transcript=Cre28.g757697.t1.1 locus=Cre28.g757697 ID=Cre28.g757697.t1.1.v5.5 annot-version=v5.5 Chlamydomonas_reinhardtii OG0000019
Cre28.g757497.t1.1 pacid=30782929 transcript=Cre28.g757497.t1.1 locus=Cre28.g757497 ID=Cre28.g757497.t1.1.v5.5 annot-version=v5.5 Chlamydomonas_reinhardtii OG0000019
Cre28.g757597.t1.1 pacid=30782930 transcript=Cre28.g757597.t1.1 locus=Cre28.g757597 ID=Cre28.g757597.t1.1.v5.5 annot-version=v5.5 Chlamydomonas_reinhardtii OG0000019
Cre28.g757547.t1.1 pacid=30782931 transcript=Cre28.g757547.t1.1 locus=Cre28.g757547 ID=Cre28.g757547.t1.1.v5.5 annot-version=v5.5 Chlamydomonas_reinhardtii OG0000019
Cre20.g751597.t1.1 pacid=30782939 transcript=Cre20.g751597.t1.1 locus=Cre20.g751597 ID=Cre20.g751597.t1.1.v5.5 annot-version=v5.5 Chlamydomonas_reinhardtii OG0000019
Cre20.g751497.t1.1 pacid=30782940 transcript=Cre20.g751497.t1.1 locus=Cre20.g751497 ID=Cre20.g751497.t1.1.v5.5 annot-version=v5.5 Chlamydomonas_reinhardtii OG0000019
Cre20.g751397.t1.1 pacid=30782943 transcript=Cre20.g751397.t1.1 locus=Cre20.g751397 ID=Cre20.g751397.t1.1.v5.5 annot-version=v5.5 Chlamydomonas_reinhardtii OG0000019
Cre20.g751447.t1.1 pacid=30782944 transcript=Cre20.g751447.t1.1 locus=Cre20.g751447 ID=Cre20.g751447.t1.1.v5.5 annot-version=v5.5 Chlamydomonas_reinhardtii OG0000019
Cre20.g751547.t1.1 pacid=30782945 transcript=Cre20.g751547.t1.1 locus=Cre20.g751547 ID=Cre20.g751547.t1.1.v5.5 annot-version=v5.5 Chlamydomonas_reinhardtii OG0000019
Cre20.g751647.t1.1 pacid=30782947 transcript=Cre20.g751647.t1.1 locus=Cre20.g751647 ID=Cre20.g751647.t1.1.v5.5 annot-version=v5.5 Chlamydomonas_reinhardtii OG0000019
Cre05.g234892.t1.1 pacid=30782955 transcript=Cre05.g234892.t1.1 locus=Cre05.g234892 ID=Cre05.g234892.t1.1.v5.5 annot-version=v5.5 Chlamydomonas_reinhardtii OG0000019
Cre05.g234934.t1.1 pacid=30783018 transcript=Cre05.g234934.t1.1 locus=Cre05.g234934 ID=Cre05.g234934.t1.1.v5.5 annot-version=v5.5 Chlamydomonas_reinhardtii OG0000019
Cre05.g241630.t1.1 pacid=30783107 transcript=Cre05.g241630.t1.1 locus=Cre05.g241630 ID=Cre05.g241630.t1.1.v5.5 annot-version=v5.5 Chlamydomonas_reinhardtii OG0000019
Cre05.g235060.t1.1 pacid=30783124 transcript=Cre05.g235060.t1.1 locus=Cre05.g235060 ID=Cre05.g235060.t1.1.v5.5 annot-version=v5.5 Chlamydomonas_reinhardtii OG0000019
Cre05.g233402.t1.1 pacid=30783153 transcript=Cre05.g233402.t1.1 locus=Cre05.g233402 ID=Cre05.g233402.t1.1.v5.5 annot-version=v5.5 Chlamydomonas_reinhardtii OG0000019
Cre05.g235102.t1.1 pacid=30783328 transcript=Cre05.g235102.t1.1 locus=Cre05.g235102 ID=Cre05.g235102.t1.1.v5.5 annot-version=v5.5 Chlamydomonas_reinhardtii OG0000019
Cre05.g235018.t1.1 pacid=30783424 transcript=Cre05.g235018.t1.1 locus=Cre05.g235018 ID=Cre05.g235018.t1.1.v5.5 annot-version=v5.5 Chlamydomonas_reinhardtii OG0000019
Cre05.g235144.t1.1 pacid=30783480 transcript=Cre05.g235144.t1.1 locus=Cre05.g235144 ID=Cre05.g235144.t1.1.v5.5 annot-version=v5.5 Chlamydomonas_reinhardtii OG0000019

Cre25.g756247.t1.1 pacid=30783791 transcript=Cre25.g756247.t1.1 locus=Cre25.g756247 ID=Cre25.g756247.t1.1.v5.5 annot-version=v5.5 Chlamydomonas_reinhardtii OG0000019
Cre25.g756447.t1.1 pacid=30783794 transcript=Cre25.g756447.t1.1 locus=Cre25.g756447 ID=Cre25.g756447.t1.1.v5.5 annot-version=v5.5 Chlamydomonas_reinhardtii OG0000019
Cre25.g756397.t1.1 pacid=30783795 transcript=Cre25.g756397.t1.1 locus=Cre25.g756397 ID=Cre25.g756397.t1.1.v5.5 annot-version=v5.5 Chlamydomonas_reinhardtii OG0000019
Cre25.g756297.t1.1 pacid=30783799 transcript=Cre25.g756297.t1.1 locus=Cre25.g756297 ID=Cre25.g756297.t1.1.v5.5 annot-version=v5.5 Chlamydomonas_reinhardtii OG0000019
Cre25.g756197.t1.1 pacid=30783803 transcript=Cre25.g756197.t1.1 locus=Cre25.g756197 ID=Cre25.g756197.t1.1.v5.5 annot-version=v5.5 Chlamydomonas_reinhardtii OG0000019
Cre25.g756097.t1.1 pacid=30783804 transcript=Cre25.g756097.t1.1 locus=Cre25.g756097 ID=Cre25.g756097.t1.1.v5.5 annot-version=v5.5 Chlamydomonas_reinhardtii OG0000019
Cre25.g756147.t1.1 pacid=30783808 transcript=Cre25.g756147.t1.1 locus=Cre25.g756147 ID=Cre25.g756147.t1.1.v5.5 annot-version=v5.5 Chlamydomonas_reinhardtii OG0000019
Cre13.g600238.t1.1 pacid=30783905 transcript=Cre13.g600238.t1.1 locus=Cre13.g600238 ID=Cre13.g600238.t1.1.v5.5 annot-version=v5.5 Chlamydomonas_reinhardtii OG0000019
Cre13.g601519.t1.1 pacid=30784261 transcript=Cre13.g601519.t1.1 locus=Cre13.g601519 ID=Cre13.g601519.t1.1.v5.5 annot-version=v5.5 Chlamydomonas_reinhardtii OG0000019
Cre13.g605300.t1.1 pacid=30784505 transcript=Cre13.g605300.t1.1 locus=Cre13.g605300 ID=Cre13.g605300.t1.1.v5.5 annot-version=v5.5 Chlamydomonas_reinhardtii OG0000019
Cre08.g373350.t1.2 pacid=30773462 transcript=Cre08.g373350.t1.2 locus=Cre08.g373350 ID=Cre08.g373350.t1.2.v5.5 annot-version=v5.5 Chlamydomonas_reinhardtii OG0000019
Cre02.g093202.t1.1 pacid=30784804 transcript=Cre02.g093202.t1.1 locus=Cre02.g093202 ID=Cre02.g093202.t1.1.v5.5 annot-version=v5.5 Chlamydomonas_reinhardtii OG0000019
jgi|ChlNC64A_1|17890|gw1.30.43.1 Chlorella_variabilis OG0000019
jgi|ChlNC64A_1|138418|IGS.gm_20_00167 Chlorella_variabilis OG0000019
jgi|ChlNC64A_1|17888|gw1.6.410.1 Chlorella_variabilis OG0000019
jgi|ChlNC64A_1|26569|e_gw1.21.179.1 Chlorella_variabilis OG0000019
jgi|ChlNC64A_1|14789|gw1.29.147.1 Chlorella_variabilis OG0000019
jgi|ChlNC64A_1|26263|e_gw1.19.219.1 Chlorella_variabilis OG0000019
9938 pacid=27388193 transcript=9938 locus=gw1.26.73.1 ID=9938.2.0.227 annot-version=v2.0 Coccomyxa_subellipsoidea OG0000019
9992 pacid=27394635 transcript=9992 locus=gw1.17.216.1 ID=9992.2.0.227 annot-version=v2.0 Coccomyxa_subellipsoidea OG0000019

kfl00026_0210_v1.1   Klebsormidium_flaccidum OG0000019
kfl00006_0530_v1.1   Klebsormidium_flaccidum OG0000019
kfl00308_0090_v1.1   Klebsormidium_flaccidum OG0000019
Pp3c15_15580V3.1.p pacid=32926611 transcript=Pp3c15_15580V3.1 locus=Pp3c15_15580 annot-version=v3.3   Physcomitrella_patens OG0000019
Pp3c10_7790V3.1.p pacid=32902283 transcript=Pp3c10_7790V3.1 locus=Pp3c10_7790 annot-version=v3.3   Physcomitrella_patens OG0000019
Pp3c15_2730V3.1.p pacid=32928129 transcript=Pp3c15_2730V3.1 locus=Pp3c15_2730 annot-version=v3.3   Physcomitrella_patens OG0000019
Pp3c13_20290V3.1.p pacid=32932551 transcript=Pp3c13_20290V3.1 locus=Pp3c13_20290 annot-version=v3.3   Physcomitrella_patens OG0000019
Pp3c2_15650V3.1.p pacid=32934537 transcript=Pp3c2_15650V3.1 locus=Pp3c2_15650 annot-version=v3.3   Physcomitrella_patens OG0000019
Pp3c2_2462V3.1.p pacid=32936951 transcript=Pp3c2_2462V3.1 locus=Pp3c2_2462 annot-version=v3.3   Physcomitrella_patens OG0000019
Pp3c2_34600V3.1.p pacid=32937223 transcript=Pp3c2_34600V3.1 locus=Pp3c2_34600 annot-version=v3.3   Physcomitrella_patens OG0000019
Pp3c19_21420V3.1.p pacid=32937778 transcript=Pp3c19_21420V3.1 locus=Pp3c19_21420 annot-version=v3.3   Physcomitrella_patens OG0000019
Pp3c19_21430V3.1.p pacid=32938623 transcript=Pp3c19_21430V3.1 locus=Pp3c19_21430 annot-version=v3.3   Physcomitrella_patens OG0000019
Pp3c19_8170V3.1.p pacid=32939117 transcript=Pp3c19_8170V3.1 locus=Pp3c19_8170 annot-version=v3.3   Physcomitrella_patens OG0000019
Pp3c19_9339V3.1.p pacid=32939543 transcript=Pp3c19_9339V3.1 locus=Pp3c19_9339 annot-version=v3.3   Physcomitrella_patens OG0000019
Pp3c3_28850V3.1.p pacid=32941129 transcript=Pp3c3_28850V3.1 locus=Pp3c3_28850 annot-version=v3.3   Physcomitrella_patens OG0000019
Pp3c20_5460V3.1.p pacid=32947544 transcript=Pp3c20_5460V3.1 locus=Pp3c20_5460 annot-version=v3.3   Physcomitrella_patens OG0000019
Pp3c20_9580V3.1.p pacid=32948018 transcript=Pp3c20_9580V3.1 locus=Pp3c20_9580 annot-version=v3.3   Physcomitrella_patens OG0000019
Pp3c23_13000V3.1.p pacid=32949716 transcript=Pp3c23_13000V3.1 locus=Pp3c23_13000 annot-version=v3.3   Physcomitrella_patens OG0000019
Pp3c23_20490V3.1.p pacid=32950108 transcript=Pp3c23_20490V3.1 locus=Pp3c23_20490 annot-version=v3.3   Physcomitrella_patens OG0000019
Pp3c27_5700V3.1.p pacid=32952026 transcript=Pp3c27_5700V3.1 locus=Pp3c27_5700 annot-version=v3.3   Physcomitrella_patens OG0000019

Pp3c27_1601V3.1.p pacid=32952277 transcript=Pp3c27_1601V3.1 locus=Pp3c27_1601 annot-version=v3.3 Physcomitrella_patens OG0000019
Pp3c5_14170V3.1.p pacid=32953134 transcript=Pp3c5_14170V3.1 locus=Pp3c5_14170 annot-version=v3.3 Physcomitrella_patens OG0000019
Pp3c11_14193V3.1.p pacid=32957420 transcript=Pp3c11_14193V3.1 locus=Pp3c11_14193 annot-version=v3.3 Physcomitrella_patens OG0000019
Pp3c11_9190V3.1.p pacid=32958049 transcript=Pp3c11_9190V3.1 locus=Pp3c11_9190 annot-version=v3.3 Physcomitrella_patens OG0000019
Pp3c11_10220V3.1.p pacid=32958678 transcript=Pp3c11_10220V3.1 locus=Pp3c11_10220 annot-version=v3.3 Physcomitrella_patens OG0000019
Pp3c11_1280V3.1.p pacid=32959044 transcript=Pp3c11_1280V3.1 locus=Pp3c11_1280 annot-version=v3.3 Physcomitrella_patens OG0000019
Pp3c14_21330V3.1.p pacid=32960170 transcript=Pp3c14_21330V3.1 locus=Pp3c14_21330 annot-version=v3.3 Physcomitrella_patens OG0000019
Pp3c14_2700V3.1.p pacid=32961509 transcript=Pp3c14_2700V3.1 locus=Pp3c14_2700 annot-version=v3.3 Physcomitrella_patens OG0000019
Pp3c14_14620V3.1.p pacid=32961543 transcript=Pp3c14_14620V3.1 locus=Pp3c14_14620 annot-version=v3.3 Physcomitrella_patens OG0000019
Pp3c8_1400V3.1.p pacid=32964367 transcript=Pp3c8_1400V3.1 locus=Pp3c8_1400 annot-version=v3.3 Physcomitrella_patens OG0000019
Pp3c17_3030V3.1.p pacid=32906072 transcript=Pp3c17_3030V3.1 locus=Pp3c17_3030 annot-version=v3.3 Physcomitrella_patens OG0000019
Pp3c8_9850V3.1.p pacid=32966202 transcript=Pp3c8_9850V3.1 locus=Pp3c8_9850 annot-version=v3.3 Physcomitrella_patens OG0000019
Pp3c1_10710V3.1.p pacid=32967018 transcript=Pp3c1_10710V3.1 locus=Pp3c1_10710 annot-version=v3.3 Physcomitrella_patens OG0000019
Pp3c1_38370V3.1.p pacid=32967695 transcript=Pp3c1_38370V3.1 locus=Pp3c1_38370 annot-version=v3.3 Physcomitrella_patens OG0000019
Pp3c1_40810V3.1.p pacid=32971345 transcript=Pp3c1_40810V3.1 locus=Pp3c1_40810 annot-version=v3.3 Physcomitrella_patens OG0000019
Pp3c6_17607V3.1.p pacid=32976896 transcript=Pp3c6_17607V3.1 locus=Pp3c6_17607 annot-version=v3.3 Physcomitrella_patens OG0000019
Pp3c6_21650V3.1.p pacid=32978307 transcript=Pp3c6_21650V3.1 locus=Pp3c6_21650 annot-version=v3.3 Physcomitrella_patens OG0000019
Pp3c18_21460V3.1.p pacid=32982945 transcript=Pp3c18_21460V3.1 locus=Pp3c18_21460 annot-version=v3.3 Physcomitrella_patens OG0000019

Pp3c16_25280V3.1.p pacid=32983619 transcript=Pp3c16_25280V3.1 locus=Pp3c16_25280 annot-version=v3.3 Physcomitrella_patens OG0000019
Pp3c16_2611V3.1.p pacid=32985073 transcript=Pp3c16_2611V3.1 locus=Pp3c16_2611 annot-version=v3.3 Physcomitrella_patens OG0000019
Pp3c16_13980V3.1.p pacid=32985824 transcript=Pp3c16_13980V3.1 locus=Pp3c16_13980 annot-version=v3.3 Physcomitrella_patens OG0000019
Pp3c16_3200V3.1.p pacid=32985966 transcript=Pp3c16_3200V3.1 locus=Pp3c16_3200 annot-version=v3.3 Physcomitrella_patens OG0000019
Pp3c16_2600V3.1.p pacid=32986152 transcript=Pp3c16_2600V3.1 locus=Pp3c16_2600 annot-version=v3.3 Physcomitrella_patens OG0000019
Pp3c24_19560V3.1.p pacid=32910679 transcript=Pp3c24_19560V3.1 locus=Pp3c24_19560 annot-version=v3.3 Physcomitrella_patens OG0000019
Pp3c24_16620V3.1.p pacid=32910755 transcript=Pp3c24_16620V3.1 locus=Pp3c24_16620 annot-version=v3.3 Physcomitrella_patens OG0000019
Pp3c24_490V3.1.p pacid=32910845 transcript=Pp3c24_490V3.1 locus=Pp3c24_490 annot-version=v3.3 Physcomitrella_patens OG0000019
Pp3c24_11220V3.1.p pacid=32911058 transcript=Pp3c24_11220V3.1 locus=Pp3c24_11220 annot-version=v3.3 Physcomitrella_patens OG0000019
Pp3c4_31850V3.1.p pacid=32919551 transcript=Pp3c4_31850V3.1 locus=Pp3c4_31850 annot-version=v3.3 Physcomitrella_patens OG0000019
Pp3c4_24040V3.1.p pacid=32921009 transcript=Pp3c4_24040V3.1 locus=Pp3c4_24040 annot-version=v3.3 Physcomitrella_patens OG0000019
Pp3c4_20050V3.1.p pacid=32921116 transcript=Pp3c4_20050V3.1 locus=Pp3c4_20050 annot-version=v3.3 Physcomitrella_patens OG0000019
Pp3c7_17370V3.1.p pacid=32923592 transcript=Pp3c7_17370V3.1 locus=Pp3c7_17370 annot-version=v3.3 Physcomitrella_patens OG0000019
Pp3c7_3520V3.1.p pacid=32923720 transcript=Pp3c7_3520V3.1 locus=Pp3c7_3520 annot-version=v3.3 Physcomitrella_patens OG0000019
Pp3c7_16880V3.1.p pacid=32925581 transcript=Pp3c7_16880V3.1 locus=Pp3c7_16880 annot-version=v3.3 Physcomitrella_patens OG0000019
AT1G40390.1 | DNAse I-like superfamily protein | Chr1:15432576-15434674 REVERSE LENGTH=426 | 201606 Arabidopsis_thaliana OG0000019
AT1G43760.1 | DNAse I-like superfamily protein | Chr1:16528880-16531065 REVERSE LENGTH=626 | 201606 Arabidopsis_thaliana OG0000019
Os03t0643433-00 Similar to J013022N21, full insert sequence. Oryza_sativa OG0000019
Os04t0371500-00 Conserved hypothetical protein. Oryza_sativa

OG0000019
Os04t0382550-00 Conserved hypothetical protein.   Oryza_sativa
OG0000019
Os06t0205337-00 Endonuclease/exonuclease/phosphatase domain
containing protein.   Oryza_sativa   OG0000019
Os06t0484800-00 Reverse transcriptase domain containing protein.
Oryza_sativa   OG0000019
Os08t0561250-00 Hypothetical protein.   Oryza_sativa
OG0000019
Os10t0131701-00 Conserved hypothetical protein.   Oryza_sativa
OG0000019
Os10t0351414-00 Endonuclease/exonuclease/phosphatase domain
containing protein.   Oryza_sativa   OG0000019
Os01t0714225-00 Hypothetical protein.   Oryza_sativa
OG0000019
Os11t0194050-00 Conserved hypothetical protein.   Oryza_sativa
OG0000019
Os11t0298866-00 Conserved hypothetical protein.   Oryza_sativa
OG0000019
Os11t0672600-01 Reverse transcriptase domain containing protein.
Oryza_sativa   OG0000019
Os02t0818601-00 Similar to H0215A08.3 protein.   Oryza_sativa
OG0000019
g1801.t1 Caulerpa_lentillifera   OG0000020
g2115.t1 Caulerpa_lentillifera   OG0000020
g4249.t1 Caulerpa_lentillifera   OG0000020
g5480.t1 Caulerpa_lentillifera   OG0000020
g7931.t1 Caulerpa_lentillifera   OG0000020
g8311.t1 Caulerpa_lentillifera   OG0000020
g8973.t1 Caulerpa_lentillifera   OG0000020
Cre02.g083750.t1.1 pacid=30785302 transcript=Cre02.g083750.t1.1
locus=Cre02.g083750 ID=Cre02.g083750.t1.1.v5.5 annot-version=v5.5
Chlamydomonas_reinhardtii OG0000020
Cre02.g094150.t1.2 pacid=30785337 transcript=Cre02.g094150.t1.2
locus=Cre02.g094150 ID=Cre02.g094150.t1.2.v5.5 annot-version=v5.5
Chlamydomonas_reinhardtii OG0000020
Cre12.g495100.t1.2 pacid=30792897 transcript=Cre12.g495100.t1.2
locus=Cre12.g495100 ID=Cre12.g495100.t1.2.v5.5 annot-version=v5.5
Chlamydomonas_reinhardtii OG0000020
Cre11.g467604.t1.1 pacid=30775894 transcript=Cre11.g467604.t1.1
locus=Cre11.g467604 ID=Cre11.g467604.t1.1.v5.5 annot-version=v5.5
Chlamydomonas_reinhardtii OG0000020
Cre16.g676421.t1.1 pacid=30777897 transcript=Cre16.g676421.t1.1
locus=Cre16.g676421 ID=Cre16.g676421.t1.1.v5.5 annot-version=v5.5
Chlamydomonas_reinhardtii OG0000020
Cre09.g410450.t1.2 pacid=30780486 transcript=Cre09.g410450.t1.2
locus=Cre09.g410450 ID=Cre09.g410450.t1.2.v5.5 annot-version=v5.5
Chlamydomonas_reinhardtii OG0000020
Cre17.g696750.t1.1 pacid=30782331 transcript=Cre17.g696750.t1.1
locus=Cre17.g696750 ID=Cre17.g696750.t1.1.v5.5 annot-version=v5.5
Chlamydomonas_reinhardtii OG0000020
Cre05.g243000.t1.1 pacid=30783266 transcript=Cre05.g243000.t1.1
locus=Cre05.g243000 ID=Cre05.g243000.t1.1.v5.5 annot-version=v5.5
Chlamydomonas_reinhardtii OG0000020

Cre13.g572450.t1.2 pacid=30784011 transcript=Cre13.g572450.t1.2
locus=Cre13.g572450 ID=Cre13.g572450.t1.2.v5.5 annot-version=v5.5
Chlamydomonas_reinhardtii OG0000020
Vocar.0001s0186.1.p pacid=32884351 transcript=Vocar.0001s0186.1
locus=Vocar.0001s0186 ID=Vocar.0001s0186.1.v2.1 annot-version=v2.1
Volvox_carteri OG0000020
Vocar.0008s0156.1.p pacid=32897033 transcript=Vocar.0008s0156.1
locus=Vocar.0008s0156 ID=Vocar.0008s0156.1.v2.1 annot-version=v2.1
Volvox_carteri OG0000020
Vocar.0074s0018.1.p pacid=32899261 transcript=Vocar.0074s0018.1
locus=Vocar.0074s0018 ID=Vocar.0074s0018.1.v2.1 annot-version=v2.1
Volvox_carteri OG0000020
Vocar.0004s0521.1.p pacid=32889638 transcript=Vocar.0004s0521.1
locus=Vocar.0004s0521 ID=Vocar.0004s0521.1.v2.1 annot-version=v2.1
Volvox_carteri OG0000020
Vocar.0031s0066.1.p pacid=32890168 transcript=Vocar.0031s0066.1
locus=Vocar.0031s0066 ID=Vocar.0031s0066.1.v2.1 annot-version=v2.1
Volvox_carteri OG0000020
Vocar.0005s0508.1.p pacid=32883895 transcript=Vocar.0005s0508.1
locus=Vocar.0005s0508 ID=Vocar.0005s0508.1.v2.1 annot-version=v2.1
Volvox_carteri OG0000020
Vocar.0078s0011.1.p pacid=32891716 transcript=Vocar.0078s0011.1
locus=Vocar.0078s0011 ID=Vocar.0078s0011.1.v2.1 annot-version=v2.1
Volvox_carteri OG0000020
Vocar.0036s0068.1.p pacid=32892274 transcript=Vocar.0036s0068.1
locus=Vocar.0036s0068 ID=Vocar.0036s0068.1.v2.1 annot-version=v2.1
Volvox_carteri OG0000020
jgi|ChlNC64A_1|140816|IGS.gm_3_00555 Chlorella_variabilis
OG0000020
jgi|ChlNC64A_1|137569|IGS.gm_2_00036 Chlorella_variabilis
OG0000020
jgi|ChlNC64A_1|141917|IGS.gm_4_00058 Chlorella_variabilis
OG0000020
jgi|ChlNC64A_1|142361|IGS.gm_4_00502 Chlorella_variabilis
OG0000020
jgi|ChlNC64A_1|30989|estExt_Genewise1.C_90203
Chlorella_variabilis OG0000020
jgi|ChlNC64A_1|145560|IGS.gm_9_00367 Chlorella_variabilis
OG0000020
jgi|ChlNC64A_1|142770|IGS.gm_5_00099 Chlorella_variabilis
OG0000020
jgi|ChlNC64A_1|143134|IGS.gm_5_00463 Chlorella_variabilis
OG0000020
jgi|ChlNC64A_1|141785|IGS.gm_38_00048 Chlorella_variabilis
OG0000020
jgi|ChlNC64A_1|136336|IGS.gm_16_00059 Chlorella_variabilis
OG0000020
jgi|ChlNC64A_1|136058|IGS.gm_15_00154 Chlorella_variabilis
OG0000020
11147 pacid=27389509 transcript=11147 locus=e_gw1.1.548.1
ID=11147.2.0.227 annot-version=v2.0 Coccomyxa_subellipsoidea
OG0000020
46620 pacid=27389873 transcript=46620
locus=estExt_fgenesh1_pg.C_40143 ID=46620.2.0.227 annot-version=v2.0

Coccomyxa_subellipsoidea OG0000020
64410 pacid=27393387 transcript=64410 locus=estExt_Genemark1.C_20019 ID=64410.2.0.227 annot-version=v2.0 Coccomyxa_subellipsoidea OG0000020
57853 pacid=27394029 transcript=57853 locus=Genemark1.6882_g ID=57853.2.0.227 annot-version=v2.0 Coccomyxa_subellipsoidea OG0000020
9065 pacid=27395032 transcript=9065 locus=gw1.15.222.1 ID=9065.2.0.227 annot-version=v2.0 Coccomyxa_subellipsoidea OG0000020
9894 pacid=27395044 transcript=9894 locus=gw1.15.242.1 ID=9894.2.0.227 annot-version=v2.0 Coccomyxa_subellipsoidea OG0000020
24566_estExt_fgenesh1_pg.C_Chr_16.00010090 Ostreococcus_tauri OG0000020
35849_1200010147 Ostreococcus_tauri OG0000020
35984_1200010282 Ostreococcus_tauri OG0000020
38278 pacid=27416303 transcript=38278 locus=e_gwEuk.10.377.1 ID=38278.2.0.231 annot-version=v2.0 Ostreococcus_lucimarinus OG0000020
26932 pacid=27416971 transcript=26932 locus=eugene.1200010277 ID=26932.2.0.231 annot-version=v2.0 Ostreococcus_lucimarinus OG0000020
26838 pacid=27417059 transcript=26838 locus=eugene.1200010183 ID=26838.2.0.231 annot-version=v2.0 Ostreococcus_lucimarinus OG0000020
5500 pacid=27417955 transcript=5500 locus=gwEuk.1.479.1 ID=5500.2.0.231 annot-version=v2.0 Ostreococcus_lucimarinus OG0000020
52653 pacid=27339707 transcript=52653 locus=MicpuC2.EuGene.0000130210 ID=52653.3.0.228 annot-version=v3.0 Micromonas_pusilla OG0000020
62878 pacid=27340276 transcript=62878 locus=MicpuC2.est_orfs.10_1861_4270447:1 ID=62878.3.0.228 annot-version=v3.0 Micromonas_pusilla OG0000020
60557 pacid=27344441 transcript=60557 locus=MicpuC2.EuGene.0000090108 ID=60557.3.0.228 annot-version=v3.0 Micromonas_pusilla OG0000020
48139 pacid=27344468 transcript=48139 locus=MicpuC2.estExt_fgenesh1_pg.C_90268 ID=48139.3.0.228 annot-version=v3.0 Micromonas_pusilla OG0000020
61323 pacid=27345009 transcript=61323 locus=MicpuC2.est_orfs.1_306_4269596:1 ID=61323.3.0.228 annot-version=v3.0 Micromonas_pusilla OG0000020
106146 pacid=27397423 transcript=106146 locus=estExt_fgenesh2_pg.C_Chr_100175 ID=106146.3.0.229 annot-version=v3.0 Micromonas_sp._RCC299 OG0000020
106326 pacid=27399493 transcript=106326 locus=estExt_fgenesh2_pg.C_Chr_120081 ID=106326.3.0.229 annot-version=v3.0 Micromonas_sp._RCC299 OG0000020
59098 pacid=27402158 transcript=59098 locus=EuGene.0600010100 ID=59098.3.0.229 annot-version=v3.0 Micromonas_sp._RCC299 OG0000020
60184 pacid=27404158 transcript=60184 locus=EuGene.0700010469

ID=60184.3.0.229 annot-version=v3.0	Micromonas_sp._RCC299 OG0000020
kfl00537_0090_v1.1	Klebsormidium_flaccidum	OG0000020
kfl00030_0250_v1.1	Klebsormidium_flaccidum	OG0000020
kfl00059_0230_v1.1	Klebsormidium_flaccidum	OG0000020
kfl00118_0040_v1.1	Klebsormidium_flaccidum	OG0000020
kfl00133_0040_v1.1	Klebsormidium_flaccidum	OG0000020
kfl00165_0030_v1.1	Klebsormidium_flaccidum	OG0000020
kfl00262_0120_v1.1	Klebsormidium_flaccidum	OG0000020
kfl00262_0130_v1.1	Klebsormidium_flaccidum	OG0000020
Pp3c7_7640V3.1.p pacid=32926341 transcript=Pp3c7_7640V3.1 locus=Pp3c7_7640 annot-version=v3.3	Physcomitrella_patens OG0000020
Pp3c15_13960V3.1.p pacid=32929105 transcript=Pp3c15_13960V3.1 locus=Pp3c15_13960 annot-version=v3.3	Physcomitrella_patens OG0000020
Pp3c13_9170V3.1.p pacid=32930411 transcript=Pp3c13_9170V3.1 locus=Pp3c13_9170 annot-version=v3.3	Physcomitrella_patens OG0000020
Pp3c13_23440V3.1.p pacid=32930695 transcript=Pp3c13_23440V3.1 locus=Pp3c13_23440 annot-version=v3.3	Physcomitrella_patens OG0000020
Pp3c10_13030V3.1.p pacid=32902768 transcript=Pp3c10_13030V3.1 locus=Pp3c10_13030 annot-version=v3.3	Physcomitrella_patens OG0000020
Pp3c2_2200V3.1.p pacid=32934023 transcript=Pp3c2_2200V3.1 locus=Pp3c2_2200 annot-version=v3.3	Physcomitrella_patens OG0000020
Pp3c2_310V3.1.p pacid=32935275 transcript=Pp3c2_310V3.1 locus=Pp3c2_310 annot-version=v3.3	Physcomitrella_patens OG0000020
Pp3c19_2940V3.1.p pacid=32939272 transcript=Pp3c19_2940V3.1 locus=Pp3c19_2940 annot-version=v3.3	Physcomitrella_patens OG0000020
Pp3c3_23200V3.1.p pacid=32940452 transcript=Pp3c3_23200V3.1 locus=Pp3c3_23200 annot-version=v3.3	Physcomitrella_patens OG0000020
Pp3c3_34700V3.1.p pacid=32942074 transcript=Pp3c3_34700V3.1 locus=Pp3c3_34700 annot-version=v3.3	Physcomitrella_patens OG0000020
Pp3c3_34900V3.1.p pacid=32942322 transcript=Pp3c3_34900V3.1 locus=Pp3c3_34900 annot-version=v3.3	Physcomitrella_patens OG0000020
Pp3c3_23120V3.1.p pacid=32942913 transcript=Pp3c3_23120V3.1 locus=Pp3c3_23120 annot-version=v3.3	Physcomitrella_patens OG0000020
Pp3c3_14670V3.1.p pacid=32943571 transcript=Pp3c3_14670V3.1 locus=Pp3c3_14670 annot-version=v3.3	Physcomitrella_patens OG0000020
Pp3c3_10940V3.1.p pacid=32944181 transcript=Pp3c3_10940V3.1 locus=Pp3c3_10940 annot-version=v3.3	Physcomitrella_patens OG0000020
Pp3c20_7050V3.1.p pacid=32945885 transcript=Pp3c20_7050V3.1 locus=Pp3c20_7050 annot-version=v3.3	Physcomitrella_patens

OG0000020
Pp3c20_11890V3.1.p pacid=32946817 transcript=Pp3c20_11890V3.1
locus=Pp3c20_11890 annot-version=v3.3   Physcomitrella_patens
OG0000020
Pp3c23_7400V3.1.p pacid=32948689 transcript=Pp3c23_7400V3.1
locus=Pp3c23_7400 annot-version=v3.3   Physcomitrella_patens
OG0000020
Pp3c23_11030V3.1.p pacid=32949359 transcript=Pp3c23_11030V3.1
locus=Pp3c23_11030 annot-version=v3.3   Physcomitrella_patens
OG0000020
Pp3c23_11010V3.1.p pacid=32949517 transcript=Pp3c23_11010V3.1
locus=Pp3c23_11010 annot-version=v3.3   Physcomitrella_patens
OG0000020
Pp3c23_19680V3.1.p pacid=32949557 transcript=Pp3c23_19680V3.1
locus=Pp3c23_19680 annot-version=v3.3   Physcomitrella_patens
OG0000020
Pp3c23_3520V3.1.p pacid=32949931 transcript=Pp3c23_3520V3.1
locus=Pp3c23_3520 annot-version=v3.3   Physcomitrella_patens
OG0000020
Pp3c23_5390V3.1.p pacid=32950264 transcript=Pp3c23_5390V3.1
locus=Pp3c23_5390 annot-version=v3.3   Physcomitrella_patens
OG0000020
Pp3c22_8217V3.1.p pacid=32904762 transcript=Pp3c22_8217V3.1
locus=Pp3c22_8217 annot-version=v3.3   Physcomitrella_patens
OG0000020
Pp3c22_8210V3.1.p pacid=32904768 transcript=Pp3c22_8210V3.1
locus=Pp3c22_8210 annot-version=v3.3   Physcomitrella_patens
OG0000020
Pp3c11_21510V3.1.p pacid=32957387 transcript=Pp3c11_21510V3.1
locus=Pp3c11_21510 annot-version=v3.3   Physcomitrella_patens
OG0000020
Pp3c14_26270V3.1.p pacid=32960031 transcript=Pp3c14_26270V3.1
locus=Pp3c14_26270 annot-version=v3.3   Physcomitrella_patens
OG0000020
Pp3c8_16910V3.1.p pacid=32964806 transcript=Pp3c8_16910V3.1
locus=Pp3c8_16910 annot-version=v3.3   Physcomitrella_patens
OG0000020
Pp3c8_21000V3.1.p pacid=32965136 transcript=Pp3c8_21000V3.1
locus=Pp3c8_21000 annot-version=v3.3   Physcomitrella_patens
OG0000020
Pp3c17_23560V3.1.p pacid=32906073 transcript=Pp3c17_23560V3.1
locus=Pp3c17_23560 annot-version=v3.3   Physcomitrella_patens
OG0000020
Pp3c1_41530V3.1.p pacid=32968557 transcript=Pp3c1_41530V3.1
locus=Pp3c1_41530 annot-version=v3.3   Physcomitrella_patens
OG0000020
Pp3c17_23620V3.1.p pacid=32906530 transcript=Pp3c17_23620V3.1
locus=Pp3c17_23620 annot-version=v3.3   Physcomitrella_patens
OG0000020
Pp3c12_10900V3.1.p pacid=32972555 transcript=Pp3c12_10900V3.1
locus=Pp3c12_10900 annot-version=v3.3   Physcomitrella_patens
OG0000020
Pp3c12_11150V3.1.p pacid=32973741 transcript=Pp3c12_11150V3.1
locus=Pp3c12_11150 annot-version=v3.3   Physcomitrella_patens

OG0000020
Pp3c25_5040V3.1.p pacid=32979339 transcript=Pp3c25_5040V3.1
locus=Pp3c25_5040 annot-version=v3.3 Physcomitrella_patens
OG0000020
Pp3c25_4920V3.1.p pacid=32979436 transcript=Pp3c25_4920V3.1
locus=Pp3c25_4920 annot-version=v3.3 Physcomitrella_patens
OG0000020
Pp3c18_20920V3.1.p pacid=32982951 transcript=Pp3c18_20920V3.1
locus=Pp3c18_20920 annot-version=v3.3 Physcomitrella_patens
OG0000020
Pp3c16_18460V3.1.p pacid=32983729 transcript=Pp3c16_18460V3.1
locus=Pp3c16_18460 annot-version=v3.3 Physcomitrella_patens
OG0000020
Pp3c16_18610V3.1.p pacid=32984253 transcript=Pp3c16_18610V3.1
locus=Pp3c16_18610 annot-version=v3.3 Physcomitrella_patens
OG0000020
Pp3c24_7690V3.1.p pacid=32909330 transcript=Pp3c24_7690V3.1
locus=Pp3c24_7690 annot-version=v3.3 Physcomitrella_patens
OG0000020
Pp3c24_12830V3.1.p pacid=32910307 transcript=Pp3c24_12830V3.1
locus=Pp3c24_12830 annot-version=v3.3 Physcomitrella_patens
OG0000020
Pp3c24_7590V3.1.p pacid=32910620 transcript=Pp3c24_7590V3.1
locus=Pp3c24_7590 annot-version=v3.3 Physcomitrella_patens
OG0000020
Pp3c9_14240V3.1.p pacid=32911447 transcript=Pp3c9_14240V3.1
locus=Pp3c9_14240 annot-version=v3.3 Physcomitrella_patens
OG0000020
Pp3c9_14170V3.1.p pacid=32912671 transcript=Pp3c9_14170V3.1
locus=Pp3c9_14170 annot-version=v3.3 Physcomitrella_patens
OG0000020
Pp3c21_2850V3.1.p pacid=32914355 transcript=Pp3c21_2850V3.1
locus=Pp3c21_2850 annot-version=v3.3 Physcomitrella_patens
OG0000020
Pp3c21_6770V3.1.p pacid=32916351 transcript=Pp3c21_6770V3.1
locus=Pp3c21_6770 annot-version=v3.3 Physcomitrella_patens
OG0000020
Pp3c26_3820V3.1.p pacid=32917596 transcript=Pp3c26_3820V3.1
locus=Pp3c26_3820 annot-version=v3.3 Physcomitrella_patens
OG0000020
Pp3c26_6050V3.1.p pacid=32917623 transcript=Pp3c26_6050V3.1
locus=Pp3c26_6050 annot-version=v3.3 Physcomitrella_patens
OG0000020
Pp3c26_3290V3.1.p pacid=32917689 transcript=Pp3c26_3290V3.1
locus=Pp3c26_3290 annot-version=v3.3 Physcomitrella_patens
OG0000020
Pp3c4_6870V3.1.p pacid=32919650 transcript=Pp3c4_6870V3.1
locus=Pp3c4_6870 annot-version=v3.3 Physcomitrella_patens
OG0000020
Pp3c4_16540V3.1.p pacid=32919921 transcript=Pp3c4_16540V3.1
locus=Pp3c4_16540 annot-version=v3.3 Physcomitrella_patens
OG0000020
Pp3c4_23130V3.1.p pacid=32922055 transcript=Pp3c4_23130V3.1
locus=Pp3c4_23130 annot-version=v3.3 Physcomitrella_patens

OG0000020
Pp3c4_20290V3.1.p pacid=32922772 transcript=Pp3c4_20290V3.1
locus=Pp3c4_20290 annot-version=v3.3   Physcomitrella_patens
OG0000020
Pp3c10_24540V3.1.p pacid=32901863 transcript=Pp3c10_24540V3.1
locus=Pp3c10_24540 annot-version=v3.3   Physcomitrella_patens
OG0000020
Pp3c7_20870V3.1.p pacid=32924287 transcript=Pp3c7_20870V3.1
locus=Pp3c7_20870 annot-version=v3.3   Physcomitrella_patens
OG0000020
AT2G38300.1 | myb-like HTH transcriptional regulator family protein
| Chr2:16044175-16045679 REVERSE LENGTH=340 | 201606
Arabidopsis_thaliana   OG0000020
AT2G40260.1 | Homeodomain-like superfamily protein |
Chr2:16816818-16818473 REVERSE LENGTH=410 | 201606
Arabidopsis_thaliana   OG0000020
AT2G40970.1 | Homeodomain-like superfamily protein |
Chr2:17097772-17098518 REVERSE LENGTH=248 | 201606
Arabidopsis_thaliana   OG0000020
AT2G42660.1 | Homeodomain-like superfamily protein |
Chr2:17767065-17768258 REVERSE LENGTH=255 | 201606
Arabidopsis_thaliana   OG0000020
AT2G46790.1 | pseudo-response regulator 9 | Chr2:19232874-19234901
FORWARD LENGTH=468 | 201606   Arabidopsis_thaliana
OG0000020
AT3G04030.1 | Homeodomain-like superfamily protein |
Chr3:1042920-1044574 REVERSE LENGTH=388 | 201606
Arabidopsis_thaliana   OG0000020
AT3G04450.1 | Homeodomain-like superfamily protein |
Chr3:1184302-1186264 FORWARD LENGTH=442 | 201606
Arabidopsis_thaliana   OG0000020
AT3G10760.1 | Homeodomain-like superfamily protein |
Chr3:3369814-3370821 FORWARD LENGTH=335 | 201606
Arabidopsis_thaliana   OG0000020
AT3G12730.1 | Homeodomain-like superfamily protein |
Chr3:4047234-4048356 REVERSE LENGTH=235 | 201606
Arabidopsis_thaliana   OG0000020
AT3G13040.1 | myb-like HTH transcriptional regulator family protein
| Chr3:4172415-4174456 REVERSE LENGTH=449 | 201606
Arabidopsis_thaliana   OG0000020
AT3G16857.1 | response regulator 1 | Chr3:5756113-5758853 FORWARD
LENGTH=669 | 201606   Arabidopsis_thaliana   OG0000020
AT1G13300.1 | myb-like transcription factor family protein |
Chr1:4556977-4558591 FORWARD LENGTH=344 | 201606
Arabidopsis_thaliana   OG0000020
AT3G24120.1 | Homeodomain-like superfamily protein |
Chr3:8705925-8708148 REVERSE LENGTH=295 | 201606
Arabidopsis_thaliana   OG0000020
AT3G25790.1 | myb-like transcription factor family protein |
Chr3:9413196-9414951 FORWARD LENGTH=357 | 201606
Arabidopsis_thaliana   OG0000020
AT1G14600.1 | Homeodomain-like superfamily protein |
Chr1:5001185-5003370 REVERSE LENGTH=255 | 201606
Arabidopsis_thaliana   OG0000020

AT3G46640.1 | Homeodomain-like superfamily protein |
Chr3:17183248-17184219 FORWARD LENGTH=323 | 201606
Arabidopsis_thaliana OG0000020
AT3G62670.1 | two-component response regulator |
Chr3:23176556-23178294 REVERSE LENGTH=426 | 201606
Arabidopsis_thaliana OG0000020
AT4G04555.1 | Myb family transcription factor | Chr4:2265822-2266790
REVERSE LENGTH=106 | 201606 Arabidopsis_thaliana
OG0000020
AT4G04580.1 | Homeodomain-like superfamily protein |
Chr4:2293761-2294906 REVERSE LENGTH=166 | 201606
Arabidopsis_thaliana OG0000020
AT4G04605.1 | Myb-like DNA-binding domain protein |
Chr4:2316596-2318003 REVERSE LENGTH=220 | 201606
Arabidopsis_thaliana OG0000020
AT4G13640.1 | Homeodomain-like superfamily protein |
Chr4:7936864-7938497 REVERSE LENGTH=292 | 201606
Arabidopsis_thaliana OG0000020
AT4G16110.1 | response regulator 2 | Chr4:9112979-9115785 FORWARD
LENGTH=664 | 201606 Arabidopsis_thaliana OG0000020
AT4G17695.1 | Homeodomain-like superfamily protein |
Chr4:9848134-9850698 REVERSE LENGTH=322 | 201606
Arabidopsis_thaliana OG0000020
AT4G18020.1 | CheY-like two-component responsive regulator family
protein | Chr4:10003738-10006682 REVERSE LENGTH=535 | 201606
Arabidopsis_thaliana OG0000020
AT4G28610.1 | phosphate starvation response 1 |
Chr4:14133164-14134951 REVERSE LENGTH=409 | 201606
Arabidopsis_thaliana OG0000020
AT4G31920.1 | response regulator 10 | Chr4:15444290-15446766 REVERSE
LENGTH=552 | 201606 Arabidopsis_thaliana OG0000020
AT4G37180.1 | Homeodomain-like superfamily protein |
Chr4:17504648-17506107 FORWARD LENGTH=356 | 201606
Arabidopsis_thaliana OG0000020
AT5G02810.1 | pseudo-response regulator 7 | Chr5:638283-641461
REVERSE LENGTH=727 | 201606 Arabidopsis_thaliana
OG0000020
AT5G05090.1 | Homeodomain-like superfamily protein |
Chr5:1503393-1504193 FORWARD LENGTH=266 | 201606
Arabidopsis_thaliana OG0000020
AT5G06800.1 | myb-like HTH transcriptional regulator family protein
| Chr5:2103374-2105680 FORWARD LENGTH=375 | 201606
Arabidopsis_thaliana OG0000020
AT5G07210.1 | response regulator 21 | Chr5:2252237-2256018 FORWARD
LENGTH=621 | 201606 Arabidopsis_thaliana OG0000020
AT5G16560.1 | Homeodomain-like superfamily protein |
Chr5:5407365-5411092 REVERSE LENGTH=403 | 201606
Arabidopsis_thaliana OG0000020
AT5G18240.1 | myb-related protein 1 | Chr5:6028617-6030573 REVERSE
LENGTH=402 | 201606 Arabidopsis_thaliana OG0000020
AT5G24470.1 | two-component response regulator-like protein |
Chr5:8356204-8358546 REVERSE LENGTH=558 | 201606
Arabidopsis_thaliana OG0000020
AT5G29000.1 | Homeodomain-like superfamily protein |

Chr5:11022336-11024100 REVERSE LENGTH=370 | 201606
Arabidopsis_thaliana   OG0000020
AT5G42630.1 | Homeodomain-like superfamily protein |
Chr5:17073997-17075747 REVERSE LENGTH=276 | 201606
Arabidopsis_thaliana   OG0000020
AT5G45580.1 | Homeodomain-like superfamily protein |
Chr5:18481092-18482598 REVERSE LENGTH=264 | 201606
Arabidopsis_thaliana   OG0000020
AT5G49240.1 | pseudo-response regulator 4 | Chr5:19962934-19964351
FORWARD LENGTH=292 | 201606   Arabidopsis_thaliana
OG0000020
AT1G25550.1 | myb-like transcription factor family protein |
Chr1:8976644-8977942 FORWARD LENGTH=344 | 201606
Arabidopsis_thaliana   OG0000020
AT5G58080.1 | response regulator 18 | Chr5:23501785-23504150 REVERSE
LENGTH=635 | 201606   Arabidopsis_thaliana   OG0000020
AT5G59570.1 | Homeodomain-like superfamily protein |
Chr5:24004047-24004943 FORWARD LENGTH=298 | 201606
Arabidopsis_thaliana   OG0000020
AT5G60100.1 | pseudo-response regulator 3 | Chr5:24198215-24200502
REVERSE LENGTH=495 | 201606   Arabidopsis_thaliana
OG0000020
AT5G61380.1 | CCT motif -containing response regulator protein |
Chr5:24675540-24678176 FORWARD LENGTH=618 | 201606
Arabidopsis_thaliana   OG0000020
AT1G32240.1 | Homeodomain-like superfamily protein |
Chr1:11625882-11630355 REVERSE LENGTH=388 | 201606
Arabidopsis_thaliana   OG0000020
AT1G49190.1 | response regulator 19 | Chr1:18191342-18193598 FORWARD
LENGTH=407 | 201606   Arabidopsis_thaliana   OG0000020
AT1G49560.1 | Homeodomain-like superfamily protein |
Chr1:18342725-18344101 FORWARD LENGTH=333 | 201606
Arabidopsis_thaliana   OG0000020
AT1G67710.1 | response regulator 11 | Chr1:25376994-25378905 REVERSE
LENGTH=521 | 201606   Arabidopsis_thaliana   OG0000020
AT1G68210.1 | pseudo-response regulator 6 | Chr1:25565983-25569302
FORWARD LENGTH=755 | 201606   Arabidopsis_thaliana
OG0000020
AT1G68670.1 | myb-like transcription factor family protein |
Chr1:25782344-25783873 FORWARD LENGTH=354 | 201606
Arabidopsis_thaliana   OG0000020
AT1G69580.1 | Homeodomain-like superfamily protein |
Chr1:26172127-26173612 FORWARD LENGTH=336 | 201606
Arabidopsis_thaliana   OG0000020
AT1G79430.1 | Homeodomain-like superfamily protein |
Chr1:29877521-29878850 REVERSE LENGTH=293 | 201606
Arabidopsis_thaliana   OG0000020
AT2G01060.1 | myb-like HTH transcriptional regulator family protein
| Chr2:73456-74902 REVERSE LENGTH=286 | 201606
Arabidopsis_thaliana   OG0000020
AT2G01760.1 | response regulator 14 | Chr2:333041-334514 FORWARD
LENGTH=382 | 201606   Arabidopsis_thaliana   OG0000020
AT2G02060.1 | Homeodomain-like superfamily protein |
Chr2:495691-497609 FORWARD LENGTH=256 | 201606

Arabidopsis_thaliana	OG0000020
AT2G06020.1 | Homeodomain-like superfamily protein | Chr2:2342535-2346207 FORWARD LENGTH=301 | 201606	Arabidopsis_thaliana	OG0000020
AT2G20400.1 | myb-like HTH transcriptional regulator family protein | Chr2:8799624-8801621 FORWARD LENGTH=397 | 201606	Arabidopsis_thaliana	OG0000020
AT2G25180.1 | response regulator 12 | Chr2:10724490-10726961 REVERSE LENGTH=596 | 201606	Arabidopsis_thaliana	OG0000020
AT2G27070.1 | response regulator 13 | Chr2:11556448-11560215 REVERSE LENGTH=572 | 201606	Arabidopsis_thaliana	OG0000020
Os03t0241001-00 Similar to DNA binding protein.	Oryza_sativa	OG0000020
Os03t0284100-01 Similar to Two-component response regulator-like PRR73.	Oryza_sativa	OG0000020
Os03t0325500-01 Similar to Phosphate starvation regulator protein (Regulatory protein of P- starvation acclimation response Psr1).	Oryza_sativa	OG0000020
Os03t0329900-01 MYB-CC family protein, Pi-starvation signaling	Oryza_sativa	OG0000020
Os03t0624000-00 Similar to KANADI 3.	Oryza_sativa	OG0000020
Os03t0764600-01 Homeodomain-like containing protein.	Oryza_sativa	OG0000020
Os03t0766500-01 Similar to Two-component response regulator ARR1. Splice isoform 2.	Oryza_sativa	OG0000020
Os04t0348800-00 Signal transduction response regulator, receiver domain domain containing protein.	Oryza_sativa	OG0000020
Os04t0566600-01 Homeodomain-like containing protein.	Oryza_sativa	OG0000020
Os04t0600000-01 Similar to Transfactor-like protein.	Oryza_sativa	OG0000020
Os04t0665600-01 Similar to Phosphate starvation response regulator-like protein.	Oryza_sativa	OG0000020
Os05t0395600-01 Hypothetical conserved gene.	Oryza_sativa	OG0000020
Os05t0412000-00 Similar to Homeodomain-related.	Oryza_sativa	OG0000020
Os05t0488600-01 Similar to Phosphate starvation regulator protein (Regulatory protein of P- starvation acclimation response Psr1).	Oryza_sativa	OG0000020
Os05t0491500-01 Similar to Transfactor-like protein.	Oryza_sativa	OG0000020
Os06t0183100-01 B-type response regulator, Cytokinin signaling	Oryza_sativa	OG0000020
Os06t0543200-01 Similar to CDPK substrate protein 1.	Oryza_sativa	OG0000020
Os06t0609500-01 Homeodomain-like containing protein.	Oryza_sativa	OG0000020
Os06t0647200-01 Similar to Response regulator.	Oryza_sativa	OG0000020
Os06t0664800-01 Similar to Phosphate starvation regulator protein (Regulatory protein of P- starvation acclimation response Psr1).	Oryza_sativa	OG0000020

Os06t0664900-00 Conserved hypothetical protein.	Oryza_sativa	OG0000020
Os06t0670300-01 Homeodomain-like containing protein.	Oryza_sativa	OG0000020
Os06t0703900-01 Homeodomain-like containing protein.	Oryza_sativa	OG0000020
Os07t0119300-01 Homeodomain-related domain containing protein.	Oryza_sativa	OG0000020
Os07t0438800-01 MYB-CC family protein, Pi-starvation signaling	Oryza_sativa	OG0000020
Os07t0685300-01 Similar to Phosphate starvation regulator protein (Regulatory protein of P- starvation acclimation response Psr1).	Oryza_sativa	OG0000020
Os07t0695100-01 Pseudo response regulator, Heading date, long-day repression	Oryza_sativa	OG0000020
Os08t0160300-00 Similar to KANADI-like transcription factor FEATHERED.	Oryza_sativa	OG0000020
Os08t0346400-01 Similar to Phosphate starvation regulator protein (Regulatory protein of P- starvation acclimation response Psr1).	Oryza_sativa	OG0000020
Os08t0346500-00 Transfactor-like protein.	Oryza_sativa	OG0000020
Os08t0426866-00 Similar to Kanadi1.	Oryza_sativa	OG0000020
Os08t0434700-01 Homeodomain-like containing protein.	Oryza_sativa	OG0000020
Os09t0299000-01 Similar to CDPK substrate protein 1.	Oryza_sativa	OG0000020
Os09t0299200-01 MYB-type transcription factor, Reguration of high-affinity potassium transporter, Salt tolerance	Oryza_sativa	OG0000020
Os09t0395300-01 GARP DNA-binding protien, Leaf polarity modeling	Oryza_sativa	OG0000020
Os09t0532400-01 Signal transduction response regulator, receiver region domain containing protein.	Oryza_sativa	OG0000020
Os10t0463400-01 B-type response regulator, Floral inducer to promote short-day (SD) flowering pathway	Oryza_sativa	OG0000020
Os11t0106100-00 Similar to Myb-like DNA-binding domain, SHAQKYF class family protein, expressed.	Oryza_sativa	OG0000020
Os11t0157600-01 Similar to CCT motif family protein, expressed.	Oryza_sativa	OG0000020
Os12t0105600-01 Similar to Phosphate starvation regulator protein (Regulatory protein of P- starvation acclimation response Psr1).	Oryza_sativa	OG0000020
Os12t0586300-00 Myb-like DNA-binding domain, SHAQKYF class domain containing protein.	Oryza_sativa	OG0000020
Os01t0844900-01 Homeodomain-like containing protein.	Oryza_sativa	OG0000020
Os01t0904700-01 B-type response regulator, Cytokinin signaling	Oryza_sativa	OG0000020
Os01t0971800-00 Similar to Two-component response regulator ARR11 (Receiver-like protein 3).	Oryza_sativa	OG0000020
Os02t0139000-01 Transcription factor, Regulation of Pi signaling and homeostasis, Tolerance to low-Pi stress	Oryza_sativa

OG0000020
Os02t0168200-01 Similar to Transfactor-like protein.
Oryza_sativa OG0000020
Os02t0174000-00 Myb-like DNA-binding domain, SHAQKYF class domain containing protein. Oryza_sativa OG0000020
Os02t0182100-01 B-type response regulator, Cytokinin signaling
Oryza_sativa OG0000020
Os02t0241200-00 Myb-like DNA-binding domain, SHAQKYF class domain containing protein. Oryza_sativa OG0000020
Os02t0325600-01 Nitrate-inducible and autorepressible transcriptional repressor, Nitrogen response Oryza_sativa OG0000020
Os02t0618200-01 Signal transduction response regulator, receiver region domain containing protein. Oryza_sativa OG0000020
Os02t0696900-00 Myb-like DNA-binding domain, SHAQKYF class domain containing protein. Oryza_sativa OG0000020
Os02t0700300-01 Similar to Phosphate starvation response regulator-like protein. Oryza_sativa OG0000020
Os02t0796500-01 B-type response regulator, Cytokinin signaling
Oryza_sativa OG0000020
Os03t0224200-01 B-type response regulator, Cytokinin signaling
Oryza_sativa OG0000020
CMB079CT pep chromosome:ASM9120v1:2:217586:219982:1 gene:CMB079C transcript:CMB079CT gene_biotype:protein_coding transcript_biotype:protein_coding Cyanidioschyzon_merolae OG0000020
evm.model.contig_4486.1 Porphyridium_purpureum OG0000020
evm.model.contig_2051.13 Porphyridium_purpureum OG0000020
CDF39554 pep supercontig:ASM35022v2:HG002054:25556:29066:1 gene:CHC_T00006643001 transcript:CDF39554 gene_biotype:protein_coding transcript_biotype:protein_coding Chondrus_crispus OG0000020
Cre03.g206593.t1.1 pacid=30786756 transcript=Cre03.g206593.t1.1 locus=Cre03.g206593 ID=Cre03.g206593.t1.1.v5.5 annot-version=v5.5 Chlamydomonas_reinhardtii OG0000021
Cre03.g145927.t1.1 pacid=30787164 transcript=Cre03.g145927.t1.1 locus=Cre03.g145927 ID=Cre03.g145927.t1.1.v5.5 annot-version=v5.5 Chlamydomonas_reinhardtii OG0000021
Cre01.g021550.t1.1 pacid=30789004 transcript=Cre01.g021550.t1.1 locus=Cre01.g021550 ID=Cre01.g021550.t1.1.v5.5 annot-version=v5.5 Chlamydomonas_reinhardtii OG0000021
Cre08.g368550.t1.1 pacid=30773366 transcript=Cre08.g368550.t1.1 locus=Cre08.g368550 ID=Cre08.g368550.t1.1.v5.5 annot-version=v5.5 Chlamydomonas_reinhardtii OG0000021
Cre10.g421400.t1.2 pacid=30790137 transcript=Cre10.g421400.t1.2 locus=Cre10.g421400 ID=Cre10.g421400.t1.2.v5.5 annot-version=v5.5 Chlamydomonas_reinhardtii OG0000021
Cre04.g220100.t1.1 pacid=30791163 transcript=Cre04.g220100.t1.1 locus=Cre04.g220100 ID=Cre04.g220100.t1.1.v5.5 annot-version=v5.5 Chlamydomonas_reinhardtii OG0000021
Cre07.g343750.t1.2 pacid=30775414 transcript=Cre07.g343750.t1.2 locus=Cre07.g343750 ID=Cre07.g343750.t1.2.v5.5 annot-version=v5.5 Chlamydomonas_reinhardtii OG0000021
Cre11.g469375.t1.1 pacid=30775661 transcript=Cre11.g469375.t1.1

locus=Cre11.g469375 ID=Cre11.g469375.t1.1.v5.5 annot-version=v5.5
Chlamydomonas_reinhardtii OG0000021
Cre11.g468150.t1.1 pacid=30776074 transcript=Cre11.g468150.t1.1
locus=Cre11.g468150 ID=Cre11.g468150.t1.1.v5.5 annot-version=v5.5
Chlamydomonas_reinhardtii OG0000021
Cre14.g627750.t1.1 pacid=30776151 transcript=Cre14.g627750.t1.1
locus=Cre14.g627750 ID=Cre14.g627750.t1.1.v5.5 annot-version=v5.5
Chlamydomonas_reinhardtii OG0000021
Cre14.g626550.t1.1 pacid=30776184 transcript=Cre14.g626550.t1.1
locus=Cre14.g626550 ID=Cre14.g626550.t1.1.v5.5 annot-version=v5.5
Chlamydomonas_reinhardtii OG0000021
Cre14.g627788.t1.1 pacid=30776428 transcript=Cre14.g627788.t1.1
locus=Cre14.g627788 ID=Cre14.g627788.t1.1.v5.5 annot-version=v5.5
Chlamydomonas_reinhardtii OG0000021
Cre16.g686002.t1.1 pacid=30777004 transcript=Cre16.g686002.t1.1
locus=Cre16.g686002 ID=Cre16.g686002.t1.1.v5.5 annot-version=v5.5
Chlamydomonas_reinhardtii OG0000021
Cre16.g648600.t1.1 pacid=30777355 transcript=Cre16.g648600.t1.1
locus=Cre16.g648600 ID=Cre16.g648600.t1.1.v5.5 annot-version=v5.5
Chlamydomonas_reinhardtii OG0000021
Cre06.g298600.t1.2 pacid=30778494 transcript=Cre06.g298600.t1.2
locus=Cre06.g298600 ID=Cre06.g298600.t1.2.v5.5 annot-version=v5.5
Chlamydomonas_reinhardtii OG0000021
Cre06.g266350.t1.1 pacid=30779361 transcript=Cre06.g266350.t1.1
locus=Cre06.g266350 ID=Cre06.g266350.t1.1.v5.5 annot-version=v5.5
Chlamydomonas_reinhardtii OG0000021
Cre06.g266052.t1.1 pacid=30779407 transcript=Cre06.g266052.t1.1
locus=Cre06.g266052 ID=Cre06.g266052.t1.1.v5.5 annot-version=v5.5
Chlamydomonas_reinhardtii OG0000021
Cre06.g282251.t1.2 pacid=30779865 transcript=Cre06.g282251.t1.2
locus=Cre06.g282251 ID=Cre06.g282251.t1.2.v5.5 annot-version=v5.5
Chlamydomonas_reinhardtii OG0000021
Cre06.g266200.t1.2 pacid=30780140 transcript=Cre06.g266200.t1.2
locus=Cre06.g266200 ID=Cre06.g266200.t1.2.v5.5 annot-version=v5.5
Chlamydomonas_reinhardtii OG0000021
Cre08.g368000.t1.1 pacid=30774063 transcript=Cre08.g368000.t1.1
locus=Cre08.g368000 ID=Cre08.g368000.t1.1.v5.5 annot-version=v5.5
Chlamydomonas_reinhardtii OG0000021
Cre09.g408400.t1.1 pacid=30780541 transcript=Cre09.g408400.t1.1
locus=Cre09.g408400 ID=Cre09.g408400.t1.1.v5.5 annot-version=v5.5
Chlamydomonas_reinhardtii OG0000021
Cre09.g401589.t1.1 pacid=30780810 transcript=Cre09.g401589.t1.1
locus=Cre09.g401589 ID=Cre09.g401589.t1.1.v5.5 annot-version=v5.5
Chlamydomonas_reinhardtii OG0000021
Cre09.g401849.t1.1 pacid=30780880 transcript=Cre09.g401849.t1.1
locus=Cre09.g401849 ID=Cre09.g401849.t1.1.v5.5 annot-version=v5.5
Chlamydomonas_reinhardtii OG0000021
Cre09.g401404.t1.1 pacid=30781248 transcript=Cre09.g401404.t1.1
locus=Cre09.g401404 ID=Cre09.g401404.t1.1.v5.5 annot-version=v5.5
Chlamydomonas_reinhardtii OG0000021
Cre09.g401626.t1.1 pacid=30781254 transcript=Cre09.g401626.t1.1
locus=Cre09.g401626 ID=Cre09.g401626.t1.1.v5.5 annot-version=v5.5
Chlamydomonas_reinhardtii OG0000021
Cre17.g730400.t1.1 pacid=30781651 transcript=Cre17.g730400.t1.1

locus=Cre17.g730400 ID=Cre17.g730400.t1.1.v5.5 annot-version=v5.5
Chlamydomonas_reinhardtii OG0000021
Cre17.g704750.t1.1 pacid=30781821 transcript=Cre17.g704750.t1.1
locus=Cre17.g704750 ID=Cre17.g704750.t1.1.v5.5 annot-version=v5.5
Chlamydomonas_reinhardtii OG0000021
Cre17.g739500.t1.1 pacid=30782148 transcript=Cre17.g739500.t1.1
locus=Cre17.g739500 ID=Cre17.g739500.t1.1.v5.5 annot-version=v5.5
Chlamydomonas_reinhardtii OG0000021
Cre17.g742500.t1.1 pacid=30782212 transcript=Cre17.g742500.t1.1
locus=Cre17.g742500 ID=Cre17.g742500.t1.1.v5.5 annot-version=v5.5
Chlamydomonas_reinhardtii OG0000021
Cre17.g704400.t1.1 pacid=30782471 transcript=Cre17.g704400.t1.1
locus=Cre17.g704400 ID=Cre17.g704400.t1.1.v5.5 annot-version=v5.5
Chlamydomonas_reinhardtii OG0000021
Cre17.g704700.t1.1 pacid=30782552 transcript=Cre17.g704700.t1.1
locus=Cre17.g704700 ID=Cre17.g704700.t1.1.v5.5 annot-version=v5.5
Chlamydomonas_reinhardtii OG0000021
Cre17.g746297.t1.1 pacid=30782623 transcript=Cre17.g746297.t1.1
locus=Cre17.g746297 ID=Cre17.g746297.t1.1.v5.5 annot-version=v5.5
Chlamydomonas_reinhardtii OG0000021
Cre05.g242857.t1.1 pacid=30783258 transcript=Cre05.g242857.t1.1
locus=Cre05.g242857 ID=Cre05.g242857.t1.1.v5.5 annot-version=v5.5
Chlamydomonas_reinhardtii OG0000021
Cre15.g639050.t1.1 pacid=30783754 transcript=Cre15.g639050.t1.1
locus=Cre15.g639050 ID=Cre15.g639050.t1.1.v5.5 annot-version=v5.5
Chlamydomonas_reinhardtii OG0000021
Cre07.g354050.t1.1 pacid=30774492 transcript=Cre07.g354050.t1.1
locus=Cre07.g354050 ID=Cre07.g354050.t1.1.v5.5 annot-version=v5.5
Chlamydomonas_reinhardtii OG0000021
Vocar.0012s0240.1.p pacid=32894914 transcript=Vocar.0012s0240.1
locus=Vocar.0012s0240 ID=Vocar.0012s0240.1.v2.1 annot-version=v2.1
Volvox_carteri OG0000021
Vocar.0021s0182.1.p pacid=32895955 transcript=Vocar.0021s0182.1
locus=Vocar.0021s0182 ID=Vocar.0021s0182.1.v2.1 annot-version=v2.1
Volvox_carteri OG0000021
Vocar.0013s0014.1.p pacid=32896362 transcript=Vocar.0013s0014.1
locus=Vocar.0013s0014 ID=Vocar.0013s0014.1.v2.1 annot-version=v2.1
Volvox_carteri OG0000021
Vocar.0002s0211.1.p pacid=32891214 transcript=Vocar.0002s0211.1
locus=Vocar.0002s0211 ID=Vocar.0002s0211.1.v2.1 annot-version=v2.1
Volvox_carteri OG0000021
jgi|ChlNC64A_1|133800|IGS.gm_10_00091 Chlorella_variabilis
OG0000021
jgi|ChlNC64A_1|133802|IGS.gm_10_00093 Chlorella_variabilis
OG0000021
jgi|ChlNC64A_1|133812|IGS.gm_10_00103 Chlorella_variabilis
OG0000021
jgi|ChlNC64A_1|133818|IGS.gm_10_00109 Chlorella_variabilis
OG0000021
jgi|ChlNC64A_1|141006|IGS.gm_30_00044 Chlorella_variabilis
OG0000021
jgi|ChlNC64A_1|137646|IGS.gm_2_00113 Chlorella_variabilis
OG0000021
jgi|ChlNC64A_1|142043|IGS.gm_4_00184 Chlorella_variabilis

OG0000021
jgi|ChlNC64A_1|133389|IGS.gm_1_00525	Chlorella_variabilis
OG0000021
jgi|ChlNC64A_1|133393|IGS.gm_1_00529	Chlorella_variabilis
OG0000021
jgi|ChlNC64A_1|143686|IGS.gm_6_00260	Chlorella_variabilis
OG0000021
jgi|ChlNC64A_1|144433|IGS.gm_7_00375	Chlorella_variabilis
OG0000021
jgi|ChlNC64A_1|144547|IGS.gm_7_00489	Chlorella_variabilis
OG0000021
jgi|ChlNC64A_1|143314|IGS.gm_5_00643	Chlorella_variabilis
OG0000021
jgi|ChlNC64A_1|59342|estExt_fgenesh3_pg.C_350006
Chlorella_variabilis	OG0000021
jgi|ChlNC64A_1|141498|IGS.gm_35_00010	Chlorella_variabilis
OG0000021
jgi|ChlNC64A_1|141502|IGS.gm_35_00014	Chlorella_variabilis
OG0000021
jgi|ChlNC64A_1|141504|IGS.gm_35_00016	Chlorella_variabilis
OG0000021
jgi|ChlNC64A_1|141507|IGS.gm_35_00019	Chlorella_variabilis
OG0000021
jgi|ChlNC64A_1|59345|estExt_fgenesh3_pg.C_350010
Chlorella_variabilis	OG0000021
jgi|ChlNC64A_1|58422|estExt_fgenesh3_pg.C_160016
Chlorella_variabilis	OG0000021
jgi|ChlNC64A_1|136393|IGS.gm_16_00116	Chlorella_variabilis
OG0000021
jgi|ChlNC64A_1|136848|IGS.gm_17_00252	Chlorella_variabilis
OG0000021
jgi|ChlNC64A_1|136093|IGS.gm_15_00189	Chlorella_variabilis
OG0000021
jgi|ChlNC64A_1|136095|IGS.gm_15_00191	Chlorella_variabilis
OG0000021
jgi|ChlNC64A_1|136131|IGS.gm_15_00227	Chlorella_variabilis
OG0000021
jgi|ChlNC64A_1|136176|IGS.gm_15_00272	Chlorella_variabilis
OG0000021
jgi|ChlNC64A_1|139983|IGS.gm_28_00014	Chlorella_variabilis
OG0000021
jgi|ChlNC64A_1|54991|fgenesh3_pg.C_scaffold_28000021
Chlorella_variabilis	OG0000021
43570 pacid=27386102 transcript=43570 locus=fgenesh1_pg.13_#_213
ID=43570.2.0.227 annot-version=v2.0	Coccomyxa_subellipsoidea
OG0000021
43568 pacid=27386113 transcript=43568 locus=fgenesh1_pg.13_#_211
ID=43568.2.0.227 annot-version=v2.0	Coccomyxa_subellipsoidea
OG0000021
53293 pacid=27391075 transcript=53293
locus=estExt_fgenesh1_pm.C_60214 ID=53293.2.0.227 annot-version=v2.0
	Coccomyxa_subellipsoidea	OG0000021
63456 pacid=27392431 transcript=63456 locus=Genemark1.4239_g
ID=63456.2.0.227 annot-version=v2.0	Coccomyxa_subellipsoidea

OG0000021
57173 pacid=27386325 transcript=57173 locus=Genemark1.6202_g
ID=57173.2.0.227 annot-version=v2.0	Coccomyxa_subellipsoidea
OG0000021
36071_1300010043 Ostreococcus_tauri	OG0000021
27017 pacid=27418731 transcript=27017 locus=eugene.1300010039
ID=27017.2.0.231 annot-version=v2.0	Ostreococcus_lucimarinus
OG0000021
53561 pacid=27348745 transcript=53561 locus=MicpuC2.EuGene.
0000150236 ID=53561.3.0.228 annot-version=v3.0
Micromonas_pusilla	OG0000021
68598 pacid=27348884 transcript=68598 locus=MicpuC2.est_orfs.
12_7581_4277806:2 ID=68598.3.0.228 annot-version=v3.0
Micromonas_pusilla	OG0000021
59358 pacid=27340691 transcript=59358 locus=MicpuC2.EuGene.
0000070207 ID=59358.3.0.228 annot-version=v3.0
Micromonas_pusilla	OG0000021
59406 pacid=27340769 transcript=59406 locus=MicpuC2.EuGene.
0000070255 ID=59406.3.0.228 annot-version=v3.0
Micromonas_pusilla	OG0000021
210140 pacid=27341714 transcript=210180
locus=estExt_fgenesh1_pg.C_50251 ID=210180.3.0.228 annot-
version=v3.0	Micromonas_pusilla	OG0000021
60242 pacid=27339099 transcript=60242 locus=MicpuC2.EuGene.
0000080436 ID=60242.3.0.228 annot-version=v3.0
Micromonas_pusilla	OG0000021
54751 pacid=27343896 transcript=54751 locus=MicpuC2.EuGene.
0000180221 ID=54751.3.0.228 annot-version=v3.0
Micromonas_pusilla	OG0000021
63421 pacid=27344198 transcript=63421 locus=MicpuC2.est_orfs.
9_2404_4276070:2 ID=63421.3.0.228 annot-version=v3.0
Micromonas_pusilla	OG0000021
60863 pacid=27344314 transcript=60863 locus=MicpuC2.EuGene.
0000090414 ID=60863.3.0.228 annot-version=v3.0
Micromonas_pusilla	OG0000021
154532 pacid=27346339 transcript=154572 locus=e_gw1.4.1004.1
ID=154572.3.0.228 annot-version=v3.0	Micromonas_pusilla
OG0000021
57117 pacid=27346464 transcript=57117 locus=MicpuC2.EuGene.
0000040243 ID=57117.3.0.228 annot-version=v3.0
Micromonas_pusilla	OG0000021
56872 pacid=27346859 transcript=56872 locus=MicpuC2.EuGene.
0000031026 ID=56872.3.0.228 annot-version=v3.0
Micromonas_pusilla	OG0000021
69359 pacid=27348205 transcript=69359 locus=MicpuC2.est_orfs.
6_8342_4273520:1 ID=69359.3.0.228 annot-version=v3.0
Micromonas_pusilla	OG0000021
53556 pacid=27348519 transcript=53556 locus=MicpuC2.EuGene.
0000150231 ID=53556.3.0.228 annot-version=v3.0
Micromonas_pusilla	OG0000021
53557 pacid=27348551 transcript=53557 locus=MicpuC2.EuGene.
0000150232 ID=53557.3.0.228 annot-version=v3.0
Micromonas_pusilla	OG0000021
56093 pacid=27396557 transcript=56093 locus=EuGene.0200010470

ID=56093.3.0.229 annot-version=v3.0	Micromonas_sp._RCC299	OG0000021
61898 pacid=27397763 transcript=61898 locus=EuGene.1000010235 ID=61898.3.0.229 annot-version=v3.0	Micromonas_sp._RCC299	OG0000021
108917 pacid=27401232 transcript=108917 locus=est_cluster_kg.Chr_09__81__3201857:1 ID=108917.3.0.229 annot-version=v3.0	Micromonas_sp._RCC299	OG0000021
101975 pacid=27403255 transcript=101975 locus=fgenesh2_pg.C_Chr_08000266 ID=101975.3.0.229 annot-version=v3.0	Micromonas_sp._RCC299	OG0000021
60997 pacid=27403583 transcript=60997 locus=EuGene.0800010565 ID=60997.3.0.229 annot-version=v3.0	Micromonas_sp._RCC299	OG0000021
52068 pacid=27403996 transcript=52068 locus=est_orfs.Chr_14_1939_3200612:1 ID=52068.3.0.229 annot-version=v3.0	Micromonas_sp._RCC299	OG0000021
98698 pacid=27396218 transcript=98698 locus=fgenesh2_pg.C_Chr_02000074 ID=98698.3.0.229 annot-version=v3.0	Micromonas_sp._RCC299	OG0000021
62798 pacid=27404921 transcript=62798 locus=EuGene.1100010584 ID=62798.3.0.229 annot-version=v3.0	Micromonas_sp._RCC299	OG0000021
kfl00331_0080_v1.1	Klebsormidium_flaccidum	OG0000021
kfl00360_0050_v1.1	Klebsormidium_flaccidum	OG0000021
kfl00380_0060_v1.1	Klebsormidium_flaccidum	OG0000021
kfl00380_0080_v1.1	Klebsormidium_flaccidum	OG0000021
kfl00385_0040_v1.1	Klebsormidium_flaccidum	OG0000021
kfl00393_g13_v1.1	Klebsormidium_flaccidum	OG0000021
kfl00404_0140_v1.1	Klebsormidium_flaccidum	OG0000021
kfl00016_0535_v1.1	Klebsormidium_flaccidum	OG0000021
kfl00430_0110_v1.1	Klebsormidium_flaccidum	OG0000021
kfl00462_0130_v1.1	Klebsormidium_flaccidum	OG0000021
kfl00511_0030_v1.1	Klebsormidium_flaccidum	OG0000021
kfl00643_0080_v1.1	Klebsormidium_flaccidum	OG0000021
kfl00652_g2_v1.1 Klebsormidium_flaccidum	OG0000021
kfl00020_0180_v1.1	Klebsormidium_flaccidum	OG0000021
kfl00662_0070_v1.1	Klebsormidium_flaccidum	OG0000021
kfl00662_0080_v1.1	Klebsormidium_flaccidum	OG0000021
kfl00662_g9_v1.1 Klebsormidium_flaccidum	OG0000021
kfl00665_0040_v1.1	Klebsormidium_flaccidum	OG0000021
kfl00668_0040_v1.1	Klebsormidium_flaccidum	OG0000021
kfl00668_0050_v1.1	Klebsormidium_flaccidum	OG0000021
kfl00668_0045_v1.1	Klebsormidium_flaccidum	OG0000021
kfl00668_0055_v1.1	Klebsormidium_flaccidum	OG0000021
kfl00668_0060_v1.1	Klebsormidium_flaccidum	OG0000021
kfl00668_0065_v1.1	Klebsormidium_flaccidum	OG0000021
kfl00670_0080_v1.1	Klebsormidium_flaccidum	OG0000021
kfl00692_0030_v1.1	Klebsormidium_flaccidum	OG0000021
kfl00020_0615_v1.1	Klebsormidium_flaccidum	OG0000021
kfl00020_0620_v1.1	Klebsormidium_flaccidum	OG0000021
kfl00736_0020_v1.1	Klebsormidium_flaccidum	OG0000021
kfl00736_g3_v1.1 Klebsormidium_flaccidum	OG0000021
kfl00736_0040_v1.1	Klebsormidium_flaccidum	OG0000021

kfl00736_0060_v1.1	Klebsormidium_flaccidum	OG0000021
kfl00736_0030_v1.1	Klebsormidium_flaccidum	OG0000021
kfl00743_0060_v1.1	Klebsormidium_flaccidum	OG0000021
kfl00743_0065_v1.1	Klebsormidium_flaccidum	OG0000021
kfl00784_g5_v1.1	Klebsormidium_flaccidum	OG0000021
kfl00784_0050_v1.1	Klebsormidium_flaccidum	OG0000021
kfl00804_0010_v1.1	Klebsormidium_flaccidum	OG0000021
kfl00817_0020_v1.1	Klebsormidium_flaccidum	OG0000021
kfl00022_0330_v1.1	Klebsormidium_flaccidum	OG0000021
kfl00893_0040_v1.1	Klebsormidium_flaccidum	OG0000021
kfl00926_0010_v1.1	Klebsormidium_flaccidum	OG0000021
kfl00950_0040_v1.1	Klebsormidium_flaccidum	OG0000021
kfl00950_0050_v1.1	Klebsormidium_flaccidum	OG0000021
kfl01176_0020_v1.1	Klebsormidium_flaccidum	OG0000021
kfl01243_0010_v1.1	Klebsormidium_flaccidum	OG0000021
kfl01291_0010_v1.1	Klebsormidium_flaccidum	OG0000021
kfl00026_0310_v1.1	Klebsormidium_flaccidum	OG0000021
kfl00027_0110_v1.1	Klebsormidium_flaccidum	OG0000021
kfl00028_0120_v1.1	Klebsormidium_flaccidum	OG0000021
kfl00031_0220_v1.1	Klebsormidium_flaccidum	OG0000021
kfl00034_0290_v1.1	Klebsormidium_flaccidum	OG0000021
kfl00040_0040_v1.1	Klebsormidium_flaccidum	OG0000021
kfl00042_0220_v1.1	Klebsormidium_flaccidum	OG0000021
kfl00046_0040_v1.1	Klebsormidium_flaccidum	OG0000021
kfl00046_0350_v1.1	Klebsormidium_flaccidum	OG0000021
kfl00046_0355_v1.1	Klebsormidium_flaccidum	OG0000021
kfl00052_0100_v1.1	Klebsormidium_flaccidum	OG0000021
kfl00052_0290_v1.1	Klebsormidium_flaccidum	OG0000021
kfl00053_0470_v1.1	Klebsormidium_flaccidum	OG0000021
kfl00055_0040_v1.1	Klebsormidium_flaccidum	OG0000021
kfl00055_0070_v1.1	Klebsormidium_flaccidum	OG0000021
kfl00058_0140_v1.1	Klebsormidium_flaccidum	OG0000021
kfl00069_0100_v1.1	Klebsormidium_flaccidum	OG0000021
kfl00069_0155_v1.1	Klebsormidium_flaccidum	OG0000021
kfl00069_0160_v1.1	Klebsormidium_flaccidum	OG0000021
kfl00069_0170_v1.1	Klebsormidium_flaccidum	OG0000021
kfl00069_0175_v1.1	Klebsormidium_flaccidum	OG0000021
kfl00069_0230_v1.1	Klebsormidium_flaccidum	OG0000021
kfl00069_0235_v1.1	Klebsormidium_flaccidum	OG0000021
kfl00069_0320_v1.1	Klebsormidium_flaccidum	OG0000021
kfl00081_0230_v1.1	Klebsormidium_flaccidum	OG0000021
kfl00092_0170_v1.1	Klebsormidium_flaccidum	OG0000021
kfl00109_0150_v1.1	Klebsormidium_flaccidum	OG0000021
kfl00109_0180_v1.1	Klebsormidium_flaccidum	OG0000021
kfl00109_0185_v1.1	Klebsormidium_flaccidum	OG0000021
kfl00110_0300_v1.1	Klebsormidium_flaccidum	OG0000021
kfl00110_0320_v1.1	Klebsormidium_flaccidum	OG0000021
kfl00111_0080_v1.1	Klebsormidium_flaccidum	OG0000021
kfl00113_0040_v1.1	Klebsormidium_flaccidum	OG0000021
kfl00113_0050_v1.1	Klebsormidium_flaccidum	OG0000021
kfl00113_0080_v1.1	Klebsormidium_flaccidum	OG0000021
kfl00115_0070_v1.1	Klebsormidium_flaccidum	OG0000021
kfl00124_0080_v1.1	Klebsormidium_flaccidum	OG0000021
kfl00124_0190_v1.1	Klebsormidium_flaccidum	OG0000021

kfl00008_0020_v1.1	Klebsormidium_flaccidum	OG0000021
kfl00128_0130_v1.1	Klebsormidium_flaccidum	OG0000021
kfl00131_0040_v1.1	Klebsormidium_flaccidum	OG0000021
kfl00133_0150_v1.1	Klebsormidium_flaccidum	OG0000021
kfl00133_0160_v1.1	Klebsormidium_flaccidum	OG0000021
kfl00136_0070_v1.1	Klebsormidium_flaccidum	OG0000021
kfl00137_0190_v1.1	Klebsormidium_flaccidum	OG0000021
kfl00137_0200_v1.1	Klebsormidium_flaccidum	OG0000021
kfl00144_0320_v1.1	Klebsormidium_flaccidum	OG0000021
kfl00154_0270_v1.1	Klebsormidium_flaccidum	OG0000021
kfl00154_0280_v1.1	Klebsormidium_flaccidum	OG0000021
kfl00163_0170_v1.1	Klebsormidium_flaccidum	OG0000021
kfl00173_g18_v1.1	Klebsormidium_flaccidum	OG0000021
kfl00173_0330_v1.1	Klebsormidium_flaccidum	OG0000021
kfl00174_0080_v1.1	Klebsormidium_flaccidum	OG0000021
kfl00185_0050_v1.1	Klebsormidium_flaccidum	OG0000021
kfl00188_0170_v1.1	Klebsormidium_flaccidum	OG0000021
kfl00201_0070_v1.1	Klebsormidium_flaccidum	OG0000021
kfl00201_0080_v1.1	Klebsormidium_flaccidum	OG0000021
kfl00203_g8_v1.1 Klebsormidium_flaccidum	OG0000021
kfl00203_0140_v1.1	Klebsormidium_flaccidum	OG0000021
kfl00210_0070_v1.1	Klebsormidium_flaccidum	OG0000021
kfl00213_0210_v1.1	Klebsormidium_flaccidum	OG0000021
kfl00220_0170_v1.1	Klebsormidium_flaccidum	OG0000021
kfl00224_0210_v1.1	Klebsormidium_flaccidum	OG0000021
kfl00230_0130_v1.1	Klebsormidium_flaccidum	OG0000021
kfl00230_0135_v1.1	Klebsormidium_flaccidum	OG0000021
kfl00261_0020_v1.1	Klebsormidium_flaccidum	OG0000021
kfl00272_0150_v1.1	Klebsormidium_flaccidum	OG0000021
kfl00291_0050_v1.1	Klebsormidium_flaccidum	OG0000021
kfl00291_0170_v1.1	Klebsormidium_flaccidum	OG0000021
kfl00295_g17_v1.1	Klebsormidium_flaccidum	OG0000021
kfl00014_0030_v1.1	Klebsormidium_flaccidum	OG0000021
kfl00309_0030_v1.1	Klebsormidium_flaccidum	OG0000021
kfl00322_0080_v1.1	Klebsormidium_flaccidum	OG0000021
kfl00322_0140_v1.1	Klebsormidium_flaccidum	OG0000021
kfl00322_0150_v1.1	Klebsormidium_flaccidum	OG0000021
kfl00323_0070_v1.1	Klebsormidium_flaccidum	OG0000021
Pp3c15_22710V3.1.p pacid=32926583 transcript=Pp3c15_22710V3.1
locus=Pp3c15_22710 annot-version=v3.3	Physcomitrella_patens
OG0000021
Pp3c22_580V3.1.p pacid=32903147 transcript=Pp3c22_580V3.1
locus=Pp3c22_580 annot-version=v3.3	Physcomitrella_patens
OG0000021
Pp3c2_9630V3.1.p pacid=32937195 transcript=Pp3c2_9630V3.1
locus=Pp3c2_9630 annot-version=v3.3	Physcomitrella_patens
OG0000021
Pp3c1_32520V3.1.p pacid=32970706 transcript=Pp3c1_32520V3.1
locus=Pp3c1_32520 annot-version=v3.3	Physcomitrella_patens
OG0000021
Pp3c12_24870V3.1.p pacid=32972902 transcript=Pp3c12_24870V3.1
locus=Pp3c12_24870 annot-version=v3.3	Physcomitrella_patens
OG0000021
Pp3c17_13570V3.1.p pacid=32907322 transcript=Pp3c17_13570V3.1

locus=Pp3c17_13570 annot-version=v3.3   Physcomitrella_patens
OG0000021
CMR314CT pep chromosome:ASM9120v1:18:756262:757977:1 gene:CMR314C
transcript:CMR314CT gene_biotype:protein_coding
transcript_biotype:protein_coding Cyanidioschyzon_merolae
OG0000021
evm.model.contig_4404.14 Porphyridium_purpureum   OG0000021
CDF37965 pep supercontig:ASM35022v2:HG001890:166154:167989:-1
gene:CHC_T00005964001 transcript:CDF37965
gene_biotype:protein_coding transcript_biotype:protein_coding
Chondrus_crispus OG0000021
kfl00333_0190_v1.1   Klebsormidium_flaccidum OG0000022
kfl00817_0040_v1.1   Klebsormidium_flaccidum OG0000022
kfl00130_0120_v1.1   Klebsormidium_flaccidum OG0000022
Pp3c7_6590V3.1.p pacid=32926426 transcript=Pp3c7_6590V3.1
locus=Pp3c7_6590 annot-version=v3.3   Physcomitrella_patens
OG0000022
Pp3c13_6470V3.1.p pacid=32931118 transcript=Pp3c13_6470V3.1
locus=Pp3c13_6470 annot-version=v3.3   Physcomitrella_patens
OG0000022
Pp3c13_10800V3.1.p pacid=32931597 transcript=Pp3c13_10800V3.1
locus=Pp3c13_10800 annot-version=v3.3   Physcomitrella_patens
OG0000022
Pp3c13_20650V3.1.p pacid=32931806 transcript=Pp3c13_20650V3.1
locus=Pp3c13_20650 annot-version=v3.3   Physcomitrella_patens
OG0000022
Pp3c2_32530V3.1.p pacid=32937002 transcript=Pp3c2_32530V3.1
locus=Pp3c2_32530 annot-version=v3.3   Physcomitrella_patens
OG0000022
Pp3c3_12890V3.1.p pacid=32940836 transcript=Pp3c3_12890V3.1
locus=Pp3c3_12890 annot-version=v3.3   Physcomitrella_patens
OG0000022
Pp3c3_8880V3.1.p pacid=32943536 transcript=Pp3c3_8880V3.1
locus=Pp3c3_8880 annot-version=v3.3   Physcomitrella_patens
OG0000022
Pp3c3_25610V3.1.p pacid=32943681 transcript=Pp3c3_25610V3.1
locus=Pp3c3_25610 annot-version=v3.3   Physcomitrella_patens
OG0000022
Pp3c3_2470V3.1.p pacid=32944374 transcript=Pp3c3_2470V3.1
locus=Pp3c3_2470 annot-version=v3.3   Physcomitrella_patens
OG0000022
Pp3c20_18130V3.1.p pacid=32947041 transcript=Pp3c20_18130V3.1
locus=Pp3c20_18130 annot-version=v3.3   Physcomitrella_patens
OG0000022
Pp3c23_9520V3.1.p pacid=32950169 transcript=Pp3c23_9520V3.1
locus=Pp3c23_9520 annot-version=v3.3   Physcomitrella_patens
OG0000022
Pp3c27_7430V3.1.p pacid=32951840 transcript=Pp3c27_7430V3.1
locus=Pp3c27_7430 annot-version=v3.3   Physcomitrella_patens
OG0000022
Pp3c27_7560V3.1.p pacid=32952075 transcript=Pp3c27_7560V3.1
locus=Pp3c27_7560 annot-version=v3.3   Physcomitrella_patens
OG0000022
Pp3c27_7510V3.1.p pacid=32952191 transcript=Pp3c27_7510V3.1

locus=Pp3c27_7510 annot-version=v3.3 Physcomitrella_patens
OG0000022
Pp3c5_740V3.1.p pacid=32952554 transcript=Pp3c5_740V3.1
locus=Pp3c5_740 annot-version=v3.3 Physcomitrella_patens
OG0000022
Pp3c5_630V3.1.p pacid=32952780 transcript=Pp3c5_630V3.1
locus=Pp3c5_630 annot-version=v3.3 Physcomitrella_patens
OG0000022
Pp3c5_570V3.1.p pacid=32954580 transcript=Pp3c5_570V3.1
locus=Pp3c5_570 annot-version=v3.3 Physcomitrella_patens
OG0000022
Pp3c8_6080V3.1.p pacid=32963572 transcript=Pp3c8_6080V3.1
locus=Pp3c8_6080 annot-version=v3.3 Physcomitrella_patens
OG0000022
Pp3c12_18020V3.1.p pacid=32972937 transcript=Pp3c12_18020V3.1
locus=Pp3c12_18020 annot-version=v3.3 Physcomitrella_patens
OG0000022
Pp3c12_15190V3.1.p pacid=32975100 transcript=Pp3c12_15190V3.1
locus=Pp3c12_15190 annot-version=v3.3 Physcomitrella_patens
OG0000022
Pp3c6_28230V3.1.p pacid=32975780 transcript=Pp3c6_28230V3.1
locus=Pp3c6_28230 annot-version=v3.3 Physcomitrella_patens
OG0000022
Pp3c6_1310V3.1.p pacid=32978796 transcript=Pp3c6_1310V3.1
locus=Pp3c6_1310 annot-version=v3.3 Physcomitrella_patens
OG0000022
Pp3c6_27690V3.1.p pacid=32978848 transcript=Pp3c6_27690V3.1
locus=Pp3c6_27690 annot-version=v3.3 Physcomitrella_patens
OG0000022
Pp3c16_13150V3.1.p pacid=32984123 transcript=Pp3c16_13150V3.1
locus=Pp3c16_13150 annot-version=v3.3 Physcomitrella_patens
OG0000022
Pp3c16_19830V3.1.p pacid=32984681 transcript=Pp3c16_19830V3.1
locus=Pp3c16_19830 annot-version=v3.3 Physcomitrella_patens
OG0000022
Pp3c16_23260V3.1.p pacid=32985129 transcript=Pp3c16_23260V3.1
locus=Pp3c16_23260 annot-version=v3.3 Physcomitrella_patens
OG0000022
Pp3c24_8270V3.1.p pacid=32909414 transcript=Pp3c24_8270V3.1
locus=Pp3c24_8270 annot-version=v3.3 Physcomitrella_patens
OG0000022
Pp3c26_10130V3.1.p pacid=32917521 transcript=Pp3c26_10130V3.1
locus=Pp3c26_10130 annot-version=v3.3 Physcomitrella_patens
OG0000022
Pp3c26_4660V3.1.p pacid=32918630 transcript=Pp3c26_4660V3.1
locus=Pp3c26_4660 annot-version=v3.3 Physcomitrella_patens
OG0000022
Pp3c26_13950V3.1.p pacid=32918652 transcript=Pp3c26_13950V3.1
locus=Pp3c26_13950 annot-version=v3.3 Physcomitrella_patens
OG0000022
Pp3c4_20430V3.1.p pacid=32920934 transcript=Pp3c4_20430V3.1
locus=Pp3c4_20430 annot-version=v3.3 Physcomitrella_patens
OG0000022
Pp3c4_29470V3.1.p pacid=32921886 transcript=Pp3c4_29470V3.1

locus=Pp3c4_29470 annot-version=v3.3   Physcomitrella_patens
OG0000022
Pp3c4_11490V3.1.p pacid=32922762 transcript=Pp3c4_11490V3.1
locus=Pp3c4_11490 annot-version=v3.3   Physcomitrella_patens
OG0000022
AT1G01010.1 | NAC domain containing protein 1 | Chr1:3760-5630
FORWARD LENGTH=429 | 201606   Arabidopsis_thaliana
OG0000022
AT2G43000.1 | NAC domain containing protein 42 |
Chr2:17880617-17882584 REVERSE LENGTH=275 | 201606
Arabidopsis_thaliana   OG0000022
AT2G46770.1 | NAC (No Apical Meristem) domain transcriptional
regulator superfamily protein | Chr2:19220917-19222822 REVERSE
LENGTH=365 | 201606   Arabidopsis_thaliana   OG0000022
AT3G03200.1 | NAC domain containing protein 45 | Chr3:736141-738527
REVERSE LENGTH=479 | 201606   Arabidopsis_thaliana
OG0000022
AT3G04060.1 | NAC domain containing protein 46 |
Chr3:1053625-1054952 REVERSE LENGTH=338 | 201606
Arabidopsis_thaliana   OG0000022
AT3G04070.1 | NAC domain containing protein 47 |
Chr3:1061573-1062976 REVERSE LENGTH=359 | 201606
Arabidopsis_thaliana   OG0000022
AT3G04420.1 | NAC domain containing protein 48 |
Chr3:1172840-1174295 FORWARD LENGTH=342 | 201606
Arabidopsis_thaliana   OG0000022
AT3G04430.1 | NAC domain containing protein 49 |
Chr3:1175506-1176986 REVERSE LENGTH=198 | 201606
Arabidopsis_thaliana   OG0000022
AT1G12260.1 | NAC 007 | Chr1:4163058-4164486 REVERSE LENGTH=395 |
201606 Arabidopsis_thaliana   OG0000022
AT3G10480.1 | NAC domain containing protein 50 |
Chr3:3264410-3266781 FORWARD LENGTH=447 | 201606
Arabidopsis_thaliana   OG0000022
AT3G10490.1 | NAC domain containing protein 52 |
Chr3:3268075-3269926 FORWARD LENGTH=238 | 201606
Arabidopsis_thaliana   OG0000022
AT3G10500.1 | NAC domain containing protein 53 |
Chr3:3271718-3273887 FORWARD LENGTH=549 | 201606
Arabidopsis_thaliana   OG0000022
AT1G02210.1 | NAC (No Apical Meristem) domain transcriptional
regulator superfamily protein | Chr1:427548-427811 REVERSE LENGTH=87
| 201606 Arabidopsis_thaliana   OG0000022
AT1G02220.1 | NAC domain containing protein 3 | Chr1:428902-430567
REVERSE LENGTH=394 | 201606   Arabidopsis_thaliana
OG0000022
AT3G12910.1 | NAC (No Apical Meristem) domain transcriptional
regulator superfamily protein | Chr3:4109417-4110648 FORWARD
LENGTH=303 | 201606   Arabidopsis_thaliana   OG0000022
AT3G12977.1 | NAC (No Apical Meristem) domain transcriptional
regulator superfamily protein | Chr3:4143832-4145860 FORWARD
LENGTH=279 | 201606   Arabidopsis_thaliana   OG0000022
AT1G02230.1 | NAC domain containing protein 4 | Chr1:435174-436775
REVERSE LENGTH=359 | 201606   Arabidopsis_thaliana

OG0000022
AT1G02250.1 | NAC domain containing protein 5 | Chr1:437951-439559 REVERSE LENGTH=362 | 201606 Arabidopsis_thaliana OG0000022
AT3G15170.1 | NAC (No Apical Meristem) domain transcriptional regulator superfamily protein | Chr3:5109912-5111435 FORWARD LENGTH=310 | 201606 Arabidopsis_thaliana OG0000022
AT3G15500.1 | NAC domain containing protein 3 | Chr3:5234731-5235882 FORWARD LENGTH=317 | 201606 Arabidopsis_thaliana OG0000022
AT3G15510.1 | NAC domain containing protein 2 | Chr3:5243696-5245037 FORWARD LENGTH=364 | 201606 Arabidopsis_thaliana OG0000022
AT3G17730.1 | NAC domain containing protein 57 | Chr3:6064379-6065813 FORWARD LENGTH=246 | 201606 Arabidopsis_thaliana OG0000022
AT3G18400.1 | NAC domain containing protein 58 | Chr3:6318745-6320593 REVERSE LENGTH=314 | 201606 Arabidopsis_thaliana OG0000022
AT3G29035.1 | NAC domain containing protein 3 | Chr3:11033839-11035006 FORWARD LENGTH=318 | 201606 Arabidopsis_thaliana OG0000022
AT3G44290.1 | NAC domain containing protein 60 | Chr3:15972909-15975019 REVERSE LENGTH=335 | 201606 Arabidopsis_thaliana OG0000022
AT3G44350.1 | NAC domain containing protein 61 | Chr3:16023651-16024487 REVERSE LENGTH=160 | 201606 Arabidopsis_thaliana OG0000022
AT3G49530.1 | NAC domain containing protein 62 | Chr3:18362639-18364717 REVERSE LENGTH=469 | 201606 Arabidopsis_thaliana OG0000022
AT3G55210.1 | NAC domain containing protein 63 | Chr3:20465215-20466240 FORWARD LENGTH=280 | 201606 Arabidopsis_thaliana OG0000022
AT3G56520.1 | NAC (No Apical Meristem) domain transcriptional regulator superfamily protein | Chr3:20947107-20947731 REVERSE LENGTH=175 | 201606 Arabidopsis_thaliana OG0000022
AT3G56530.1 | NAC domain containing protein 64 | Chr3:20948911-20950045 REVERSE LENGTH=319 | 201606 Arabidopsis_thaliana OG0000022
AT3G56560.1 | NAC domain containing protein 65 | Chr3:20955404-20956867 FORWARD LENGTH=228 | 201606 Arabidopsis_thaliana OG0000022
AT3G61910.1 | NAC domain protein 66 | Chr3:22929193-22930407 REVERSE LENGTH=334 | 201606 Arabidopsis_thaliana OG0000022
AT4G01520.1 | NAC domain containing protein 67 | Chr4:656407-659178 REVERSE LENGTH=302 | 201606 Arabidopsis_thaliana OG0000022
AT4G01540.1 | NAC with transmembrane motif1 | Chr4:670483-672629 REVERSE LENGTH=473 | 201606 Arabidopsis_thaliana OG0000022
AT4G01550.1 | NAC domain containing protein 69 | Chr4:674025-676225 REVERSE LENGTH=457 | 201606 Arabidopsis_thaliana OG0000022

AT4G10350.1 | NAC domain containing protein 70 |
Chr4:6415287-6416829 REVERSE LENGTH=341 | 201606
Arabidopsis_thaliana OG0000022
AT4G17980.1 | NAC domain containing protein 71 |
Chr4:9978850-9980038 REVERSE LENGTH=262 | 201606
Arabidopsis_thaliana OG0000022
AT1G19040.1 | NAC (No Apical Meristem) domain transcriptional
regulator superfamily protein | Chr1:6576895-6577413 FORWARD
LENGTH=172 | 201606 Arabidopsis_thaliana OG0000022
AT4G27410.1 | NAC (No Apical Meristem) domain transcriptional
regulator superfamily protein | Chr4:13707928-13708505 REVERSE
LENGTH=164 | 201606 Arabidopsis_thaliana OG0000022
AT4G28530.1 | NAC domain containing protein 74 |
Chr4:14090725-14094603 REVERSE LENGTH=352 | 201606
Arabidopsis_thaliana OG0000022
AT4G35580.1 | NAC transcription factor-like 9 |
Chr4:16888581-16890744 REVERSE LENGTH=512 | 201606
Arabidopsis_thaliana OG0000022
AT4G36160.1 | NAC domain containing protein 76 |
Chr4:17110877-17112891 REVERSE LENGTH=377 | 201606
Arabidopsis_thaliana OG0000022
AT5G04395.1 | NAC transcription factor-like protein |
Chr5:1241555-1242373 FORWARD LENGTH=187 | 201606
Arabidopsis_thaliana OG0000022
AT5G04410.1 | NAC domain containing protein 2 | Chr5:1243980-1246416
FORWARD LENGTH=567 | 201606 Arabidopsis_thaliana
OG0000022
AT5G07680.1 | NAC domain containing protein 80 |
Chr5:2436092-2437322 FORWARD LENGTH=329 | 201606
Arabidopsis_thaliana OG0000022
AT5G08790.1 | NAC (No Apical Meristem) domain transcriptional
regulator superfamily protein | Chr5:2859113-2860144 REVERSE
LENGTH=283 | 201606 Arabidopsis_thaliana OG0000022
AT5G09330.1 | NAC domain containing protein 82 |
Chr5:2892623-2894708 REVERSE LENGTH=489 | 201606
Arabidopsis_thaliana OG0000022
AT5G13180.1 | NAC domain containing protein 83 |
Chr5:4196643-4197577 FORWARD LENGTH=252 | 201606
Arabidopsis_thaliana OG0000022
AT5G14000.1 | NAC domain containing protein 84 |
Chr5:4518177-4519034 FORWARD LENGTH=206 | 201606
Arabidopsis_thaliana OG0000022
AT5G17260.1 | NAC domain containing protein 86 |
Chr5:5675323-5677912 REVERSE LENGTH=476 | 201606
Arabidopsis_thaliana OG0000022
AT5G18270.1 | NAC domain containing protein 87 |
Chr5:6041241-6042743 REVERSE LENGTH=335 | 201606
Arabidopsis_thaliana OG0000022
AT5G18300.1 | NAC domain containing protein 88 |
Chr5:6057773-6058216 FORWARD LENGTH=147 | 201606
Arabidopsis_thaliana OG0000022
AT5G22290.1 | NAC domain containing protein 89 |
Chr5:7376044-7377498 REVERSE LENGTH=340 | 201606
Arabidopsis_thaliana OG0000022

AT5G22380.1 | NAC domain containing protein 90 |
Chr5:7408924-7410038 REVERSE LENGTH=235 | 201606
Arabidopsis_thaliana   OG0000022
AT5G24590.2 | TCV-interacting protein | Chr5:8416920-8418723 REVERSE
LENGTH=451 | 201606   Arabidopsis_thaliana   OG0000022
AT5G39610.1 | NAC domain containing protein 6 |
Chr5:15858591-15859650 REVERSE LENGTH=285 | 201606
Arabidopsis_thaliana   OG0000022
AT5G39690.1 | NAC domain containing protein 93 |
Chr5:15888171-15890187 FORWARD LENGTH=294 | 201606
Arabidopsis_thaliana   OG0000022
AT5G39820.1 | NAC domain containing protein 94 |
Chr5:15939300-15940491 REVERSE LENGTH=337 | 201606
Arabidopsis_thaliana   OG0000022
AT5G41090.1 | NAC domain containing protein 95 |
Chr5:16445764-16446537 FORWARD LENGTH=212 | 201606
Arabidopsis_thaliana   OG0000022
AT5G46590.1 | NAC domain containing protein 96 |
Chr5:18905679-18906811 FORWARD LENGTH=292 | 201606
Arabidopsis_thaliana   OG0000022
AT5G50820.1 | NAC domain containing protein 97 |
Chr5:20679225-20680006 FORWARD LENGTH=193 | 201606
Arabidopsis_thaliana   OG0000022
AT1G25580.1 | NAC (No Apical Meristem) domain transcriptional
regulator superfamily protein | Chr1:8997064-8999600 REVERSE
LENGTH=449 | 201606   Arabidopsis_thaliana   OG0000022
AT5G53950.1 | NAC (No Apical Meristem) domain transcriptional
regulator superfamily protein | Chr5:21901966-21903795 REVERSE
LENGTH=375 | 201606   Arabidopsis_thaliana   OG0000022
AT1G03490.1 | NAC domain containing protein 6 | Chr1:871874-872906
FORWARD LENGTH=281 | 201606   Arabidopsis_thaliana
OG0000022
AT1G26870.1 | NAC (No Apical Meristem) domain transcriptional
regulator superfamily protein | Chr1:9313029-9314983 FORWARD
LENGTH=418 | 201606   Arabidopsis_thaliana   OG0000022
AT5G61430.1 | NAC domain containing protein 100 |
Chr5:24701328-24702553 REVERSE LENGTH=336 | 201606
Arabidopsis_thaliana   OG0000022
AT5G62380.1 | NAC-domain protein 101 | Chr5:25050684-25051858
FORWARD LENGTH=348 | 201606   Arabidopsis_thaliana
OG0000022
AT5G63790.1 | NAC domain containing protein 102 |
Chr5:25526887-25528013 REVERSE LENGTH=312 | 201606
Arabidopsis_thaliana   OG0000022
AT5G64060.1 | NAC domain containing protein 103 |
Chr5:25633818-25635152 REVERSE LENGTH=356 | 201606
Arabidopsis_thaliana   OG0000022
AT5G64530.1 | xylem NAC domain 1 | Chr5:25795360-25796699 FORWARD
LENGTH=187 | 201606   Arabidopsis_thaliana   OG0000022
AT5G66300.1 | NAC domain containing protein 105 |
Chr5:26480005-26481247 REVERSE LENGTH=292 | 201606
Arabidopsis_thaliana   OG0000022
AT1G32510.1 | NAC domain containing protein 11 |
Chr1:11757000-11758118 FORWARD LENGTH=283 | 201606

Arabidopsis_thaliana   OG0000022
AT1G32770.1 | NAC domain containing protein 12 |
Chr1:11865343-11866950 REVERSE LENGTH=358 | 201606
Arabidopsis_thaliana   OG0000022
AT1G32870.1 | NAC domain protein 13 | Chr1:11911745-11913692 FORWARD
LENGTH=528 | 201606   Arabidopsis_thaliana   OG0000022
AT1G33060.1 | NAC 014 | Chr1:11975518-11978493 REVERSE LENGTH=648 |
201606 Arabidopsis_thaliana   OG0000022
AT1G33280.1 | NAC domain containing protein 15 |
Chr1:12072741-12073833 FORWARD LENGTH=305 | 201606
Arabidopsis_thaliana   OG0000022
AT1G34180.1 | NAC domain containing protein 16 |
Chr1:12448726-12451120 FORWARD LENGTH=564 | 201606
Arabidopsis_thaliana   OG0000022
AT1G34190.1 | NAC domain containing protein 17 |
Chr1:12451729-12453914 FORWARD LENGTH=557 | 201606
Arabidopsis_thaliana   OG0000022
AT1G52880.1 | NAC (No Apical Meristem) domain transcriptional
regulator superfamily protein | Chr1:19689285-19690430 REVERSE
LENGTH=320 | 201606   Arabidopsis_thaliana   OG0000022
AT1G52890.1 | NAC domain containing protein 19 |
Chr1:19697292-19698444 REVERSE LENGTH=317 | 201606
Arabidopsis_thaliana   OG0000022
AT1G54330.1 | NAC domain containing protein 20 |
Chr1:20279715-20280889 REVERSE LENGTH=301 | 201606
Arabidopsis_thaliana   OG0000022
AT1G56010.1 | NAC domain containing protein 1 |
Chr1:20946852-20947727 REVERSE LENGTH=257 | 201606
Arabidopsis_thaliana   OG0000022
AT1G60280.1 | NAC domain containing protein 23 |
Chr1:22226885-22227928 REVERSE LENGTH=347 | 201606
Arabidopsis_thaliana   OG0000022
AT1G60350.1 | NAC domain containing protein 24 |
Chr1:22241273-22242235 REVERSE LENGTH=320 | 201606
Arabidopsis_thaliana   OG0000022
AT1G61110.1 | NAC domain containing protein 25 |
Chr1:22516730-22518055 FORWARD LENGTH=323 | 201606
Arabidopsis_thaliana   OG0000022
AT1G62700.1 | NAC (No apical meristem) domain transcriptional
regulator superfamily protein | Chr1:23216218-23217916 REVERSE
LENGTH=394 | 201606   Arabidopsis_thaliana   OG0000022
AT1G64105.1 | NAC domain containing protein 27 |
Chr1:23795078-23795563 FORWARD LENGTH=161 | 201606
Arabidopsis_thaliana   OG0000022
AT1G65910.1 | NAC domain containing protein 28 |
Chr1:24520933-24524108 REVERSE LENGTH=631 | 201606
Arabidopsis_thaliana   OG0000022
AT1G69490.1 | NAC-like%2C activated by AP3/PI |
Chr1:26122233-26123222 FORWARD LENGTH=268 | 201606
Arabidopsis_thaliana   OG0000022
AT1G71930.1 | vascular related NAC-domain protein 7 |
Chr1:27076205-27077829 FORWARD LENGTH=324 | 201606
Arabidopsis_thaliana   OG0000022
AT1G76420.1 | NAC (No Apical Meristem) domain transcriptional

regulator superfamily protein | Chr1:28672029-28673835 REVERSE LENGTH=334 | 201606	Arabidopsis_thaliana	OG0000022
AT1G77450.1 | NAC domain containing protein 32 | Chr1:29100029-29100981 FORWARD LENGTH=253 | 201606	Arabidopsis_thaliana	OG0000022
AT1G79580.1 | NAC (No Apical Meristem) domain transcriptional regulator superfamily protein | Chr1:29941020-29942925 REVERSE LENGTH=371 | 201606	Arabidopsis_thaliana	OG0000022
AT2G02450.1 | NAC domain containing protein 35 | Chr2:648044-650649 FORWARD LENGTH=379 | 201606	Arabidopsis_thaliana	OG0000022
AT1G01720.1 | NAC (No Apical Meristem) domain transcriptional regulator superfamily protein | Chr1:268471-269514 FORWARD LENGTH=289 | 201606	Arabidopsis_thaliana	OG0000022
AT2G17040.1 | NAC domain containing protein 36 | Chr2:7407123-7408120 FORWARD LENGTH=276 | 201606	Arabidopsis_thaliana	OG0000022
AT2G18060.1 | vascular related NAC-domain protein 1 | Chr2:7848399-7850303 REVERSE LENGTH=365 | 201606	Arabidopsis_thaliana	OG0000022
AT2G24430.1 | NAC domain containing protein 38 | Chr2:10383517-10386386 REVERSE LENGTH=316 | 201606	Arabidopsis_thaliana	OG0000022
AT2G27300.1 | NTM1-like 8 | Chr2:11680417-11681955 REVERSE LENGTH=335 | 201606	Arabidopsis_thaliana	OG0000022
AT2G33480.1 | NAC domain containing protein 41 | Chr2:14181275-14182247 FORWARD LENGTH=268 | 201606	Arabidopsis_thaliana	OG0000022
Os03t0327100-01 Similar to CUC1.	Oryza_sativa	OG0000022
Os03t0327800-01 NAC Family transcriptional activator, Abiotic stress response, Positive regulator of leaf senescence	Oryza_sativa	OG0000022
Os03t0624600-01 No apical meristem (NAM) protein domain containing protein.	Oryza_sativa	OG0000022
Os01t0261200-01 No apical meristem (NAM) protein domain containing protein.	Oryza_sativa	OG0000022
Os03t0777000-01 Similar to NAC-domain containing protein 19 (ANAC019) (ANAC) (Abscicic-acid- responsive NAC).	Oryza_sativa	OG0000022
Os03t0815100-01 Similar to OsNAC6 protein.	Oryza_sativa	OG0000022
Os04t0437000-01 No apical meristem (NAM) protein domain containing protein.	Oryza_sativa	OG0000022
Os04t0460600-01 Similar to H0219H12.10 protein.	Oryza_sativa	OG0000022
Os04t0515900-01 Similar to NAM / CUC2-like protein.	Oryza_sativa	OG0000022
Os04t0536500-01 Similar to H0502G05.5 protein.	Oryza_sativa	OG0000022
Os04t0619000-01 Similar to L1332.9 protein.	Oryza_sativa	OG0000022
Os04t0691300-00 Similar to ANAC030.	Oryza_sativa	OG0000022
Os05t0415400-01 Similar to OsNAC6 protein.	Oryza_sativa

OG0000022
Os05t0418800-00 No apical meristem (NAM) protein domain containing protein. Oryza_sativa OG0000022
Os01t0393100-01 Similar to CUC2. Oryza_sativa OG0000022
Os05t0421600-01 No apical meristem (NAM) protein domain containing protein. Oryza_sativa OG0000022
Os05t0426200-02 No apical meristem (NAM) protein domain containing protein. Oryza_sativa OG0000022
Os05t0442700-01 No apical meristem (NAM) protein domain containing protein. Oryza_sativa OG0000022
Os06t0101800-01 Similar to NAC-domain protein 1-1. Oryza_sativa OG0000022
Os06t0104200-01 Similar to OsNAC7 protein. Oryza_sativa OG0000022
Os06t0131700-01 Similar to NAM protein. Oryza_sativa OG0000022
Os06t0344900-00 Similar to NAM / CUC2-like protein. Oryza_sativa OG0000022
Os06t0530400-01 Similar to NAC domain-containing protein 76. Oryza_sativa OG0000022
Os06t0675600-01 Similar to GRAB2 protein. Oryza_sativa OG0000022
Os06t0726300-00 Similar to NAM-like protein. Oryza_sativa OG0000022
Os07t0225300-01 OsNAC3 protein. Oryza_sativa OG0000022
Os07t0272700-00 No apical meristem (NAM) protein domain containing protein. Oryza_sativa OG0000022
Os07t0566500-01 Similar to NAC domain protein. Oryza_sativa OG0000022
Os07t0683200-01 Similar to NAC domain transcription factor. Oryza_sativa OG0000022
Os07t0684800-01 Similar to NAM / CUC2-like protein. Oryza_sativa OG0000022
Os08t0103900-01 Similar to NAM-like protein. Oryza_sativa OG0000022
Os08t0115800-01 No apical meristem (NAM) protein domain containing protein. Oryza_sativa OG0000022
Os08t0157900-01 Similar to NAM protein. Oryza_sativa OG0000022
Os08t0200600-01 Similar to NAC-domain containing protein 21/22 (ANAC021) (ANAC022). Splice isoform 2. Oryza_sativa OG0000022
Os08t0433500-01 Senescence-associated NAC transcription factor, Negative regulation of leaf senescence, Salt stress tolerance, Plant architecture Oryza_sativa OG0000022
Os08t0436700-01 Similar to NAC transcription factor. Oryza_sativa OG0000022
Os08t0511200-00 Similar to Nam-like protein 16. Oryza_sativa OG0000022
Os08t0535800-01 No apical meristem (NAM) protein domain containing protein. Oryza_sativa OG0000022
Os08t0562200-02 Similar to CUC2. Oryza_sativa OG0000022
Os01t0675800-01 No apical meristem (NAM) protein domain containing protein. Oryza_sativa OG0000022

Os09t0411900-01 Similar to NAC-domain containing protein 18 (ANAC018) (NO APICAL MERISTEM protein) (AtNAM). Oryza_sativa OG0000022
Os09t0493700-01 Similar to CUC2. Oryza_sativa OG0000022
Os09t0497900-01 No apical meristem (NAM) protein domain containing protein. Oryza_sativa OG0000022
Os09t0509100-01 No apical meristem (NAM) protein domain containing protein. Oryza_sativa OG0000022
Os09t0552800-00 No apical meristem (NAM) protein domain containing protein. Oryza_sativa OG0000022
Os09t0552900-00 Similar to NAC domain protein, IPR003441. Oryza_sativa OG0000022
Os10t0359500-01 No apical meristem (NAM) protein domain containing protein. Oryza_sativa OG0000022
Os10t0413700-01 No apical meristem (NAM) protein domain containing protein. Oryza_sativa OG0000022
Os10t0414000-01 No apical meristem (NAM) protein domain containing protein. Oryza_sativa OG0000022
Os10t0477600-01 Similar to NAM / CUC2-like protein. Oryza_sativa OG0000022
Os10t0532000-01 Similar to GRAB2 protein. Oryza_sativa OG0000022
Os10t0571600-01 No apical meristem (NAM) protein domain containing protein. Oryza_sativa OG0000022
Os11t0126900-01 NAC-domain protein, Drought tolerance Oryza_sativa OG0000022
Os11t0127000-01 Similar to NAC domain-containing protein 77. Oryza_sativa OG0000022
Os11t0127600-01 No apical meristem (NAM) protein domain containing protein. Oryza_sativa OG0000022
Os11t0154500-01 No apical meristem (NAM) protein domain containing protein. Oryza_sativa OG0000022
Os11t0184900-01 Similar to OsNAC5 protein [imported]-rice. Oryza_sativa OG0000022
Os11t0512000-01 No apical meristem (NAM) protein domain containing protein. Oryza_sativa OG0000022
Os11t0512100-01 Similar to No apical meristem protein, expressed. Oryza_sativa OG0000022
Os11t0512200-00 Similar to No apical meristem protein, expressed. Oryza_sativa OG0000022
Os11t0512600-00 Similar to No apical meristem protein. Oryza_sativa OG0000022
Os12t0123700-01 NAC domain containing transcription factor, Abiotic and biotic stress response Oryza_sativa OG0000022
Os12t0123800-01 No apical meristem (NAM) protein domain containing protein. Oryza_sativa OG0000022
Os12t0135850-00 Similar to No apical meristem protein. Oryza_sativa OG0000022
Os12t0156100-01 Similar to NAC-domain containing protein 90 (ANAC090). Oryza_sativa OG0000022
Os12t0477400-01 No apical meristem (NAM) protein domain containing protein. Oryza_sativa OG0000022
Os12t0610600-01 Similar to NAM / CUC2-like protein. Oryza_sativa OG0000022

Os12t0630800-00 Hypothetical conserved gene. Oryza_sativa OG0000022
Os01t0816100-01 Similar to NAC domain protein. Oryza_sativa OG0000022
Os01t0104200-00 No apical meristem (NAM) protein domain containing protein. Oryza_sativa OG0000022
Os01t0862800-01 No apical meristem (NAM) protein domain containing protein. Oryza_sativa OG0000022
Os01t0104500-01 No apical meristem (NAM) protein domain containing protein. Oryza_sativa OG0000022
Os01t0884300-01 No apical meristem (NAM) protein domain containing protein. Oryza_sativa OG0000022
Os01t0888300-01 Similar to NAC-domain containing protein 18 (ANAC018) (NO APICAL MERISTEM protein) (AtNAM). Oryza_sativa OG0000022
Os01t0925400-01 No apical meristem (NAM) protein domain containing protein. Oryza_sativa OG0000022
Os01t0946200-01 No apical meristem (NAM) protein domain containing protein. Oryza_sativa OG0000022
Os02t0214500-01 No apical meristem (NAM) protein domain containing protein. Oryza_sativa OG0000022
Os02t0252200-01 Similar to GRAB2 protein. Oryza_sativa OG0000022
Os02t0555300-01 No apical meristem (NAM) protein domain containing protein. Oryza_sativa OG0000022
Os02t0579000-01 No apical meristem (NAM) protein domain containing protein. Oryza_sativa OG0000022
Os02t0643600-00 Similar to H0502G05.5 protein. Oryza_sativa OG0000022
Os02t0745250-00 Similar to NAC domain class transcription factor. Oryza_sativa OG0000022
Os02t0810900-01 Similar to NAC-domain containing protein 21/22 (ANAC021) (ANAC022). Splice isoform 2. Oryza_sativa OG0000022
Os02t0822400-01 No apical meristem (NAM) protein domain containing protein. Oryza_sativa OG0000022
Os03t0109000-01 Similar to NAC domain protein. Oryza_sativa OG0000022
Os03t0119966-01 NAC-domain protein, Resistance to rice dwarf virus, Regulator of jasmonate (JA) signaling Oryza_sativa OG0000022
Os03t0133000-01 Similar to NAC-domain protein 14. Oryza_sativa OG0000022
g164.t1 Caulerpa_lentillifera OG0000023
g199.t1 Caulerpa_lentillifera OG0000023
g1998.t1 Caulerpa_lentillifera OG0000023
g2099.t1 Caulerpa_lentillifera OG0000023
g2327.t1 Caulerpa_lentillifera OG0000023
g2830.t1 Caulerpa_lentillifera OG0000023
g2831.t1 Caulerpa_lentillifera OG0000023
g2879.t1 Caulerpa_lentillifera OG0000023
g2945.t1 Caulerpa_lentillifera OG0000023
g2946.t1 Caulerpa_lentillifera OG0000023
g2983.t1 Caulerpa_lentillifera OG0000023

g3031.t1	Caulerpa_lentillifera	OG0000023
g3032.t1	Caulerpa_lentillifera	OG0000023
g349.t1	Caulerpa_lentillifera	OG0000023
g3488.t1	Caulerpa_lentillifera	OG0000023
g3489.t1	Caulerpa_lentillifera	OG0000023
g3490.t1	Caulerpa_lentillifera	OG0000023
g350.t1	Caulerpa_lentillifera	OG0000023
g3491.t1	Caulerpa_lentillifera	OG0000023
g3492.t1	Caulerpa_lentillifera	OG0000023
g351.t1	Caulerpa_lentillifera	OG0000023
g352.t1	Caulerpa_lentillifera	OG0000023
g3550.t1	Caulerpa_lentillifera	OG0000023
g3555.t1	Caulerpa_lentillifera	OG0000023
g3556.t1	Caulerpa_lentillifera	OG0000023
g3557.t1	Caulerpa_lentillifera	OG0000023
g3558.t1	Caulerpa_lentillifera	OG0000023
g3601.t1	Caulerpa_lentillifera	OG0000023
g3602.t1	Caulerpa_lentillifera	OG0000023
g3603.t1	Caulerpa_lentillifera	OG0000023
g3687.t1	Caulerpa_lentillifera	OG0000023
g3760.t1	Caulerpa_lentillifera	OG0000023
g3761.t1	Caulerpa_lentillifera	OG0000023
g3762.t1	Caulerpa_lentillifera	OG0000023
g3772.t1	Caulerpa_lentillifera	OG0000023
g3773.t1	Caulerpa_lentillifera	OG0000023
g3774.t1	Caulerpa_lentillifera	OG0000023
g3775.t1	Caulerpa_lentillifera	OG0000023
g3776.t1	Caulerpa_lentillifera	OG0000023
g3777.t1	Caulerpa_lentillifera	OG0000023
g3778.t1	Caulerpa_lentillifera	OG0000023
g3779.t1	Caulerpa_lentillifera	OG0000023
g3780.t1	Caulerpa_lentillifera	OG0000023
g3781.t1	Caulerpa_lentillifera	OG0000023
g3782.t1	Caulerpa_lentillifera	OG0000023
g3785.t1	Caulerpa_lentillifera	OG0000023
g3786.t1	Caulerpa_lentillifera	OG0000023
g3787.t1	Caulerpa_lentillifera	OG0000023
g3789.t1	Caulerpa_lentillifera	OG0000023
g3790.t1	Caulerpa_lentillifera	OG0000023
g3791.t1	Caulerpa_lentillifera	OG0000023
g3792.t1	Caulerpa_lentillifera	OG0000023
g3793.t1	Caulerpa_lentillifera	OG0000023
g3794.t1	Caulerpa_lentillifera	OG0000023
g3796.t1	Caulerpa_lentillifera	OG0000023
g3805.t1	Caulerpa_lentillifera	OG0000023
g3806.t1	Caulerpa_lentillifera	OG0000023
g3808.t1	Caulerpa_lentillifera	OG0000023
g3813.t1	Caulerpa_lentillifera	OG0000023
g3814.t1	Caulerpa_lentillifera	OG0000023
g3819.t1	Caulerpa_lentillifera	OG0000023
g3824.t1	Caulerpa_lentillifera	OG0000023
g3826.t1	Caulerpa_lentillifera	OG0000023
g3832.t1	Caulerpa_lentillifera	OG0000023
g3834.t1	Caulerpa_lentillifera	OG0000023

g3837.t1	Caulerpa_lentillifera	OG0000023
g3838.t1	Caulerpa_lentillifera	OG0000023
g385.t1	Caulerpa_lentillifera	OG0000023
g3848.t1	Caulerpa_lentillifera	OG0000023
g394.t1	Caulerpa_lentillifera	OG0000023
g397.t1	Caulerpa_lentillifera	OG0000023
g3968.t1	Caulerpa_lentillifera	OG0000023
g3976.t1	Caulerpa_lentillifera	OG0000023
g3986.t1	Caulerpa_lentillifera	OG0000023
g4075.t1	Caulerpa_lentillifera	OG0000023
g4085.t1	Caulerpa_lentillifera	OG0000023
g411.t1	Caulerpa_lentillifera	OG0000023
g4106.t1	Caulerpa_lentillifera	OG0000023
g412.t1	Caulerpa_lentillifera	OG0000023
g4111.t1	Caulerpa_lentillifera	OG0000023
g4112.t1	Caulerpa_lentillifera	OG0000023
g420.t1	Caulerpa_lentillifera	OG0000023
g438.t1	Caulerpa_lentillifera	OG0000023
g4597.t1	Caulerpa_lentillifera	OG0000023
g4769.t1	Caulerpa_lentillifera	OG0000023
g4771.t1	Caulerpa_lentillifera	OG0000023
g4772.t1	Caulerpa_lentillifera	OG0000023
g4773.t1	Caulerpa_lentillifera	OG0000023
g4790.t1	Caulerpa_lentillifera	OG0000023
g5226.t1	Caulerpa_lentillifera	OG0000023
g5235.t1	Caulerpa_lentillifera	OG0000023
g5378.t1	Caulerpa_lentillifera	OG0000023
g5657.t1	Caulerpa_lentillifera	OG0000023
g5695.t1	Caulerpa_lentillifera	OG0000023
g5701.t1	Caulerpa_lentillifera	OG0000023
g5726.t1	Caulerpa_lentillifera	OG0000023
g5780.t1	Caulerpa_lentillifera	OG0000023
g5781.t1	Caulerpa_lentillifera	OG0000023
g5782.t1	Caulerpa_lentillifera	OG0000023
g5790.t1	Caulerpa_lentillifera	OG0000023
g5921.t1	Caulerpa_lentillifera	OG0000023
g5922.t1	Caulerpa_lentillifera	OG0000023
g6002.t1	Caulerpa_lentillifera	OG0000023
g6122.t1	Caulerpa_lentillifera	OG0000023
g6318.t1	Caulerpa_lentillifera	OG0000023
g6336.t1	Caulerpa_lentillifera	OG0000023
g6360.t1	Caulerpa_lentillifera	OG0000023
g6361.t1	Caulerpa_lentillifera	OG0000023
g6362.t1	Caulerpa_lentillifera	OG0000023
g6399.t1	Caulerpa_lentillifera	OG0000023
g6409.t1	Caulerpa_lentillifera	OG0000023
g6410.t1	Caulerpa_lentillifera	OG0000023
g6429.t1	Caulerpa_lentillifera	OG0000023
g7017.t1	Caulerpa_lentillifera	OG0000023
g7018.t1	Caulerpa_lentillifera	OG0000023
g7019.t1	Caulerpa_lentillifera	OG0000023
g7062.t1	Caulerpa_lentillifera	OG0000023
g7075.t1	Caulerpa_lentillifera	OG0000023
g7104.t1	Caulerpa_lentillifera	OG0000023

g7127.t1 Caulerpa_lentillifera OG0000023
g7128.t1 Caulerpa_lentillifera OG0000023
g7129.t1 Caulerpa_lentillifera OG0000023
g7130.t1 Caulerpa_lentillifera OG0000023
g7131.t1 Caulerpa_lentillifera OG0000023
g7132.t1 Caulerpa_lentillifera OG0000023
g7133.t1 Caulerpa_lentillifera OG0000023
g7134.t1 Caulerpa_lentillifera OG0000023
g7135.t1 Caulerpa_lentillifera OG0000023
g7136.t1 Caulerpa_lentillifera OG0000023
g7137.t1 Caulerpa_lentillifera OG0000023
g7435.t1 Caulerpa_lentillifera OG0000023
g7583.t1 Caulerpa_lentillifera OG0000023
g7732.t1 Caulerpa_lentillifera OG0000023
g8019.t1 Caulerpa_lentillifera OG0000023
g8421.t1 Caulerpa_lentillifera OG0000023
g8743.t1 Caulerpa_lentillifera OG0000023
g8812.t1 Caulerpa_lentillifera OG0000023
g9074.t1 Caulerpa_lentillifera OG0000023
g9089.t1 Caulerpa_lentillifera OG0000023
g93.t1 Caulerpa_lentillifera OG0000023
g9231.t1 Caulerpa_lentillifera OG0000023
Cre02.g118700.t1.1 pacid=30785112 transcript=Cre02.g118700.t1.1 locus=Cre02.g118700 ID=Cre02.g118700.t1.1.v5.5 annot-version=v5.5 Chlamydomonas_reinhardtii OG0000023
Cre02.g109501.t1.1 pacid=30785247 transcript=Cre02.g109501.t1.1 locus=Cre02.g109501 ID=Cre02.g109501.t1.1.v5.5 annot-version=v5.5 Chlamydomonas_reinhardtii OG0000023
Cre02.g094801.t1.1 pacid=30785731 transcript=Cre02.g094801.t1.1 locus=Cre02.g094801 ID=Cre02.g094801.t1.1.v5.5 annot-version=v5.5 Chlamydomonas_reinhardtii OG0000023
Cre02.g099055.t1.2 pacid=30785949 transcript=Cre02.g099055.t1.2 locus=Cre02.g099055 ID=Cre02.g099055.t1.2.v5.5 annot-version=v5.5 Chlamydomonas_reinhardtii OG0000023
Cre03.g155700.t1.1 pacid=30788133 transcript=Cre03.g155700.t1.1 locus=Cre03.g155700 ID=Cre03.g155700.t1.1.v5.5 annot-version=v5.5 Chlamydomonas_reinhardtii OG0000023
Cre01.g042450.t1.1 pacid=30789117 transcript=Cre01.g042450.t1.1 locus=Cre01.g042450 ID=Cre01.g042450.t1.1.v5.5 annot-version=v5.5 Chlamydomonas_reinhardtii OG0000023
Cre01.g052750.t1.1 pacid=30789153 transcript=Cre01.g052750.t1.1 locus=Cre01.g052750 ID=Cre01.g052750.t1.1.v5.5 annot-version=v5.5 Chlamydomonas_reinhardtii OG0000023
Cre01.g040850.t1.2 pacid=30789298 transcript=Cre01.g040850.t1.2 locus=Cre01.g040850 ID=Cre01.g040850.t1.2.v5.5 annot-version=v5.5 Chlamydomonas_reinhardtii OG0000023
Cre01.g001300.t1.1 pacid=30789566 transcript=Cre01.g001300.t1.1 locus=Cre01.g001300 ID=Cre01.g001300.t1.1.v5.5 annot-version=v5.5 Chlamydomonas_reinhardtii OG0000023
Cre01.g040650.t1.1 pacid=30789598 transcript=Cre01.g040650.t1.1 locus=Cre01.g040650 ID=Cre01.g040650.t1.1.v5.5 annot-version=v5.5 Chlamydomonas_reinhardtii OG0000023
Cre10.g420600.t1.1 pacid=30790008 transcript=Cre10.g420600.t1.1 locus=Cre10.g420600 ID=Cre10.g420600.t1.1.v5.5 annot-version=v5.5

Chlamydomonas_reinhardtii OG0000023
Cre10.g434850.t1.1 pacid=30790069 transcript=Cre10.g434850.t1.1
locus=Cre10.g434850 ID=Cre10.g434850.t1.1.v5.5 annot-version=v5.5
Chlamydomonas_reinhardtii OG0000023
Cre10.g421900.t1.1 pacid=30790193 transcript=Cre10.g421900.t1.1
locus=Cre10.g421900 ID=Cre10.g421900.t1.1.v5.5 annot-version=v5.5
Chlamydomonas_reinhardtii OG0000023
Cre10.g419200.t1.1 pacid=30790424 transcript=Cre10.g419200.t1.1
locus=Cre10.g419200 ID=Cre10.g419200.t1.1.v5.5 annot-version=v5.5
Chlamydomonas_reinhardtii OG0000023
Cre10.g420561.t1.2 pacid=30790692 transcript=Cre10.g420561.t1.2
locus=Cre10.g420561 ID=Cre10.g420561.t1.2.v5.5 annot-version=v5.5
Chlamydomonas_reinhardtii OG0000023
Cre04.g227500.t1.1 pacid=30790983 transcript=Cre04.g227500.t1.1
locus=Cre04.g227500 ID=Cre04.g227500.t1.1.v5.5 annot-version=v5.5
Chlamydomonas_reinhardtii OG0000023
Cre04.g222650.t1.1 pacid=30791141 transcript=Cre04.g222650.t1.1
locus=Cre04.g222650 ID=Cre04.g222650.t1.1.v5.5 annot-version=v5.5
Chlamydomonas_reinhardtii OG0000023
Cre04.g219150.t1.1 pacid=30791179 transcript=Cre04.g219150.t1.1
locus=Cre04.g219150 ID=Cre04.g219150.t1.1.v5.5 annot-version=v5.5
Chlamydomonas_reinhardtii OG0000023
Cre04.g227251.t1.1 pacid=30791346 transcript=Cre04.g227251.t1.1
locus=Cre04.g227251 ID=Cre04.g227251.t1.1.v5.5 annot-version=v5.5
Chlamydomonas_reinhardtii OG0000023
Cre04.g229550.t1.1 pacid=30791407 transcript=Cre04.g229550.t1.1
locus=Cre04.g229550 ID=Cre04.g229550.t1.1.v5.5 annot-version=v5.5
Chlamydomonas_reinhardtii OG0000023
Cre12.g552700.t1.1 pacid=30792194 transcript=Cre12.g552700.t1.1
locus=Cre12.g552700 ID=Cre12.g552700.t1.1.v5.5 annot-version=v5.5
Chlamydomonas_reinhardtii OG0000023
Cre14.g619400.t1.2 pacid=30776297 transcript=Cre14.g619400.t1.2
locus=Cre14.g619400 ID=Cre14.g619400.t1.2.v5.5 annot-version=v5.5
Chlamydomonas_reinhardtii OG0000023
Cre14.g633300.t1.1 pacid=30776748 transcript=Cre14.g633300.t1.1
locus=Cre14.g633300 ID=Cre14.g633300.t1.1.v5.5 annot-version=v5.5
Chlamydomonas_reinhardtii OG0000023
Cre16.g654950.t1.1 pacid=30778203 transcript=Cre16.g654950.t1.1
locus=Cre16.g654950 ID=Cre16.g654950.t1.1.v5.5 annot-version=v5.5
Chlamydomonas_reinhardtii OG0000023
Cre06.g305350.t1.1 pacid=30778390 transcript=Cre06.g305350.t1.1
locus=Cre06.g305350 ID=Cre06.g305350.t1.1.v5.5 annot-version=v5.5
Chlamydomonas_reinhardtii OG0000023
Cre06.g309800.t1.1 pacid=30778396 transcript=Cre06.g309800.t1.1
locus=Cre06.g309800 ID=Cre06.g309800.t1.1.v5.5 annot-version=v5.5
Chlamydomonas_reinhardtii OG0000023
Cre06.g309650.t1.1 pacid=30778898 transcript=Cre06.g309650.t1.1
locus=Cre06.g309650 ID=Cre06.g309650.t1.1.v5.5 annot-version=v5.5
Chlamydomonas_reinhardtii OG0000023
Cre06.g265652.t1.1 pacid=30779118 transcript=Cre06.g265652.t1.1
locus=Cre06.g265652 ID=Cre06.g265652.t1.1.v5.5 annot-version=v5.5
Chlamydomonas_reinhardtii OG0000023
Cre17.g709000.t1.2 pacid=30782047 transcript=Cre17.g709000.t1.2
locus=Cre17.g709000 ID=Cre17.g709000.t1.2.v5.5 annot-version=v5.5

Chlamydomonas_reinhardtii OG0000023
Cre17.g706700.t1.1 pacid=30782220 transcript=Cre17.g706700.t1.1
locus=Cre17.g706700 ID=Cre17.g706700.t1.1.v5.5 annot-version=v5.5
Chlamydomonas_reinhardtii OG0000023
Cre17.g728450.t1.2 pacid=30782438 transcript=Cre17.g728450.t1.2
locus=Cre17.g728450 ID=Cre17.g728450.t1.2.v5.5 annot-version=v5.5
Chlamydomonas_reinhardtii OG0000023
Cre07.g338550.t1.1 pacid=30774344 transcript=Cre07.g338550.t1.1
locus=Cre07.g338550 ID=Cre07.g338550.t1.1.v5.5 annot-version=v5.5
Chlamydomonas_reinhardtii OG0000023
Cre15.g641150.t1.1 pacid=30783538 transcript=Cre15.g641150.t1.1
locus=Cre15.g641150 ID=Cre15.g641150.t1.1.v5.5 annot-version=v5.5
Chlamydomonas_reinhardtii OG0000023
Cre13.g591300.t1.1 pacid=30784305 transcript=Cre13.g591300.t1.1
locus=Cre13.g591300 ID=Cre13.g591300.t1.1.v5.5 annot-version=v5.5
Chlamydomonas_reinhardtii OG0000023
Cre13.g564151.t1.1 pacid=30784350 transcript=Cre13.g564151.t1.1
locus=Cre13.g564151 ID=Cre13.g564151.t1.1.v5.5 annot-version=v5.5
Chlamydomonas_reinhardtii OG0000023
Cre13.g604050.t1.2 pacid=30784417 transcript=Cre13.g604050.t1.2
locus=Cre13.g604050 ID=Cre13.g604050.t1.2.v5.5 annot-version=v5.5
Chlamydomonas_reinhardtii OG0000023
Vocar.0012s0119.1.p pacid=32894821 transcript=Vocar.0012s0119.1
locus=Vocar.0012s0119 ID=Vocar.0012s0119.1.v2.1 annot-version=v2.1
Volvox_carteri OG0000023
Vocar.0021s0095.1.p pacid=32895969 transcript=Vocar.0021s0095.1
locus=Vocar.0021s0095 ID=Vocar.0021s0095.1.v2.1 annot-version=v2.1
Volvox_carteri OG0000023
Vocar.0001s1777.1.p pacid=32884599 transcript=Vocar.0001s1777.1
locus=Vocar.0001s1777 ID=Vocar.0001s1777.1.v2.1 annot-version=v2.1
Volvox_carteri OG0000023
Vocar.0001s0667.1.p pacid=32885140 transcript=Vocar.0001s0667.1
locus=Vocar.0001s0667 ID=Vocar.0001s0667.1.v2.1 annot-version=v2.1
Volvox_carteri OG0000023
Vocar.0033s0048.1.p pacid=32886412 transcript=Vocar.0033s0048.1
locus=Vocar.0033s0048 ID=Vocar.0033s0048.1.v2.1 annot-version=v2.1
Volvox_carteri OG0000023
Vocar.0033s0050.1.p pacid=32886417 transcript=Vocar.0033s0050.1
locus=Vocar.0033s0050 ID=Vocar.0033s0050.1.v2.1 annot-version=v2.1
Volvox_carteri OG0000023
Vocar.0033s0049.1.p pacid=32886484 transcript=Vocar.0033s0049.1
locus=Vocar.0033s0049 ID=Vocar.0033s0049.1.v2.1 annot-version=v2.1
Volvox_carteri OG0000023
Vocar.0005s0421.1.p pacid=32883533 transcript=Vocar.0005s0421.1
locus=Vocar.0005s0421 ID=Vocar.0005s0421.1.v2.1 annot-version=v2.1
Volvox_carteri OG0000023
Vocar.0029s0094.1.p pacid=32887345 transcript=Vocar.0029s0094.1
locus=Vocar.0029s0094 ID=Vocar.0029s0094.1.v2.1 annot-version=v2.1
Volvox_carteri OG0000023
Vocar.0029s0093.1.p pacid=32887582 transcript=Vocar.0029s0093.1
locus=Vocar.0029s0093 ID=Vocar.0029s0093.1.v2.1 annot-version=v2.1
Volvox_carteri OG0000023
Vocar.0005s0418.1.p pacid=32883924 transcript=Vocar.0005s0418.1
locus=Vocar.0005s0418 ID=Vocar.0005s0418.1.v2.1 annot-version=v2.1

Volvox_carteri	OG0000023
Vocar.0005s0419.1.p pacid=32884070 transcript=Vocar.0005s0419.1 locus=Vocar.0005s0419 ID=Vocar.0005s0419.1.v2.1 annot-version=v2.1	Volvox_carteri	OG0000023
Vocar.0001s1007.1.p pacid=32884169 transcript=Vocar.0001s1007.1 locus=Vocar.0001s1007 ID=Vocar.0001s1007.1.v2.1 annot-version=v2.1	Volvox_carteri	OG0000023
jgi|ChlNC64A_1|133816|IGS.gm_10_00107	Chlorella_variabilis	OG0000023
jgi|ChlNC64A_1|133817|IGS.gm_10_00108	Chlorella_variabilis	OG0000023
jgi|ChlNC64A_1|136006|IGS.gm_15_00102	Chlorella_variabilis	OG0000023
41952 pacid=27386923 transcript=41952 locus=fgenesh1_pg.7_#_356 ID=41952.2.0.227 annot-version=v2.0	Coccomyxa_subellipsoidea	OG0000023
64923 pacid=27390861 transcript=64923 locus=estExt_Genemark1.C_30329 ID=64923.2.0.227 annot-version=v2.0	Coccomyxa_subellipsoidea	OG0000023
48850 pacid=27394496 transcript=48850 locus=estExt_fgenesh1_pg.C_180166 ID=48850.2.0.227 annot-version=v2.0	Coccomyxa_subellipsoidea	OG0000023
58210 pacid=27394598 transcript=58210 locus=Genemark1.7239_g ID=58210.2.0.227 annot-version=v2.0	Coccomyxa_subellipsoidea	OG0000023
32718_0500010083	Ostreococcus_tauri	OG0000023
15312 pacid=27416514 transcript=15312 locus=fgenesh1_pg.C_Chr_5000064 ID=15312.2.0.231 annot-version=v2.0	Ostreococcus_lucimarinus	OG0000023
17510 pacid=27417159 transcript=17510 locus=fgenesh1_pg.C_Chr_12000042 ID=17510.2.0.231 annot-version=v2.0	Ostreococcus_lucimarinus	OG0000023
190686 pacid=27342058 transcript=190726 locus=gm1.7807_g ID=190726.3.0.228 annot-version=v3.0	Micromonas_pusilla	OG0000023
64134 pacid=27342489 transcript=64134 locus=MicpuC2.est_orfs.2_3117_4275317:3 ID=64134.3.0.228 annot-version=v3.0	Micromonas_pusilla	OG0000023
64566 pacid=27400803 transcript=64566 locus=EuGene.1500010287 ID=64566.3.0.229 annot-version=v3.0	Micromonas_sp._RCC299	OG0000023
kfl00333_0170_v1.1	Klebsormidium_flaccidum	OG0000023
kfl00388_0110_v1.1	Klebsormidium_flaccidum	OG0000023
kfl00016_0250_v1.1	Klebsormidium_flaccidum	OG0000023
kfl00480_0100_v1.1	Klebsormidium_flaccidum	OG0000023
kfl00575_0020_v1.1	Klebsormidium_flaccidum	OG0000023
kfl00721_0030_v1.1	Klebsormidium_flaccidum	OG0000023
kfl00854_0060_v1.1	Klebsormidium_flaccidum	OG0000023
kfl00026_0570_v1.1	Klebsormidium_flaccidum	OG0000023
kfl00031_0235_v1.1	Klebsormidium_flaccidum	OG0000023
kfl00047_0130_v1.1	Klebsormidium_flaccidum	OG0000023
kfl00054_0130_v1.1	Klebsormidium_flaccidum	OG0000023
kfl00054_0170_v1.1	Klebsormidium_flaccidum	OG0000023
kfl00132_0020_v1.1	Klebsormidium_flaccidum	OG0000023

kfl00207_0080_v1.1   Klebsormidium_flaccidum OG0000023
Pp3c11_5670V3.1.p pacid=32957528 transcript=Pp3c11_5670V3.1
locus=Pp3c11_5670 annot-version=v3.3   Physcomitrella_patens
OG0000023
Pp3c1_3090V3.1.p pacid=32968910 transcript=Pp3c1_3090V3.1
locus=Pp3c1_3090 annot-version=v3.3   Physcomitrella_patens
OG0000023
61329 pacid=27401426 transcript=61329 locus=EuGene.0900010312
ID=61329.3.0.229 annot-version=v3.0   Micromonas_sp._RCC299
OG0000024
kfl00389_0070_v1.1   Klebsormidium_flaccidum OG0000024
kfl00456_0180_v1.1   Klebsormidium_flaccidum OG0000024
kfl00707_0040_v1.1   Klebsormidium_flaccidum OG0000024
kfl00709_0080_v1.1   Klebsormidium_flaccidum OG0000024
kfl00908_0050_v1.1   Klebsormidium_flaccidum OG0000024
kfl00935_0080_v1.1   Klebsormidium_flaccidum OG0000024
kfl01385_0010_v1.1   Klebsormidium_flaccidum OG0000024
kfl00053_0230_v1.1   Klebsormidium_flaccidum OG0000024
kfl00103_0040_v1.1   Klebsormidium_flaccidum OG0000024
kfl00189_0150_v1.1   Klebsormidium_flaccidum OG0000024
kfl00219_g18_v1.1   Klebsormidium_flaccidum OG0000024
Pp3c2_36990V3.1.p pacid=32933689 transcript=Pp3c2_36990V3.1
locus=Pp3c2_36990 annot-version=v3.3   Physcomitrella_patens
OG0000024
Pp3c22_2470V3.1.p pacid=32903183 transcript=Pp3c22_2470V3.1
locus=Pp3c22_2470 annot-version=v3.3   Physcomitrella_patens
OG0000024
Pp3c2_2790V3.1.p pacid=32936377 transcript=Pp3c2_2790V3.1
locus=Pp3c2_2790 annot-version=v3.3   Physcomitrella_patens
OG0000024
Pp3c19_3460V3.1.p pacid=32938400 transcript=Pp3c19_3460V3.1
locus=Pp3c19_3460 annot-version=v3.3   Physcomitrella_patens
OG0000024
Pp3c19_12230V3.1.p pacid=32939339 transcript=Pp3c19_12230V3.1
locus=Pp3c19_12230 annot-version=v3.3   Physcomitrella_patens
OG0000024
Pp3c3_18870V3.1.p pacid=32941553 transcript=Pp3c3_18870V3.1
locus=Pp3c3_18870 annot-version=v3.3   Physcomitrella_patens
OG0000024
Pp3c3_27720V3.1.p pacid=32943650 transcript=Pp3c3_27720V3.1
locus=Pp3c3_27720 annot-version=v3.3   Physcomitrella_patens
OG0000024
Pp3c22_6790V3.1.p pacid=32903907 transcript=Pp3c22_6790V3.1
locus=Pp3c22_6790 annot-version=v3.3   Physcomitrella_patens
OG0000024
Pp3c20_20350V3.1.p pacid=32947858 transcript=Pp3c20_20350V3.1
locus=Pp3c20_20350 annot-version=v3.3   Physcomitrella_patens
OG0000024
Pp3c20_890V3.1.p pacid=32947899 transcript=Pp3c20_890V3.1
locus=Pp3c20_890 annot-version=v3.3   Physcomitrella_patens
OG0000024
Pp3c20_1220V3.1.p pacid=32948165 transcript=Pp3c20_1220V3.1
locus=Pp3c20_1220 annot-version=v3.3   Physcomitrella_patens
OG0000024

Pp3c27_1960V3.1.p pacid=32951622 transcript=Pp3c27_1960V3.1 locus=Pp3c27_1960 annot-version=v3.3 Physcomitrella_patens OG0000024
Pp3c27_550V3.1.p pacid=32951832 transcript=Pp3c27_550V3.1 locus=Pp3c27_550 annot-version=v3.3 Physcomitrella_patens OG0000024
Pp3c5_20650V3.1.p pacid=32953245 transcript=Pp3c5_20650V3.1 locus=Pp3c5_20650 annot-version=v3.3 Physcomitrella_patens OG0000024
Pp3c11_5330V3.1.p pacid=32957521 transcript=Pp3c11_5330V3.1 locus=Pp3c11_5330 annot-version=v3.3 Physcomitrella_patens OG0000024
Pp3c11_4120V3.1.p pacid=32958323 transcript=Pp3c11_4120V3.1 locus=Pp3c11_4120 annot-version=v3.3 Physcomitrella_patens OG0000024
Pp3c17_13760V3.1.p pacid=32905476 transcript=Pp3c17_13760V3.1 locus=Pp3c17_13760 annot-version=v3.3 Physcomitrella_patens OG0000024
Pp3c11_25840V3.1.p pacid=32959347 transcript=Pp3c11_25840V3.1 locus=Pp3c11_25840 annot-version=v3.3 Physcomitrella_patens OG0000024
Pp3c14_5200V3.1.p pacid=32960993 transcript=Pp3c14_5200V3.1 locus=Pp3c14_5200 annot-version=v3.3 Physcomitrella_patens OG0000024
Pp3c17_5150V3.1.p pacid=32905843 transcript=Pp3c17_5150V3.1 locus=Pp3c17_5150 annot-version=v3.3 Physcomitrella_patens OG0000024
Pp3c8_940V3.1.p pacid=32964398 transcript=Pp3c8_940V3.1 locus=Pp3c8_940 annot-version=v3.3 Physcomitrella_patens OG0000024
Pp3c17_5130V3.1.p pacid=32906845 transcript=Pp3c17_5130V3.1 locus=Pp3c17_5130 annot-version=v3.3 Physcomitrella_patens OG0000024
Pp3c12_13470V3.1.p pacid=32973453 transcript=Pp3c12_13470V3.1 locus=Pp3c12_13470 annot-version=v3.3 Physcomitrella_patens OG0000024
Pp3c6_17670V3.1.p pacid=32976105 transcript=Pp3c6_17670V3.1 locus=Pp3c6_17670 annot-version=v3.3 Physcomitrella_patens OG0000024
Pp3c6_26340V3.1.p pacid=32976426 transcript=Pp3c6_26340V3.1 locus=Pp3c6_26340 annot-version=v3.3 Physcomitrella_patens OG0000024
Pp3c6_8800V3.1.p pacid=32977957 transcript=Pp3c6_8800V3.1 locus=Pp3c6_8800 annot-version=v3.3 Physcomitrella_patens OG0000024
Pp3c16_15860V3.1.p pacid=32983808 transcript=Pp3c16_15860V3.1 locus=Pp3c16_15860 annot-version=v3.3 Physcomitrella_patens OG0000024
Pp3c16_16680V3.1.p pacid=32984374 transcript=Pp3c16_16680V3.1 locus=Pp3c16_16680 annot-version=v3.3 Physcomitrella_patens OG0000024
Pp3c24_590V3.1.p pacid=32910803 transcript=Pp3c24_590V3.1 locus=Pp3c24_590 annot-version=v3.3 Physcomitrella_patens OG0000024

Pp3c10_5480V3.1.p pacid=32900804 transcript=Pp3c10_5480V3.1 locus=Pp3c10_5480 annot-version=v3.3 Physcomitrella_patens OG0000024
Pp3c21_17800V3.1.p pacid=32915408 transcript=Pp3c21_17800V3.1 locus=Pp3c21_17800 annot-version=v3.3 Physcomitrella_patens OG0000024
Pp3c21_14040V3.1.p pacid=32915437 transcript=Pp3c21_14040V3.1 locus=Pp3c21_14040 annot-version=v3.3 Physcomitrella_patens OG0000024
Pp3c21_6460V3.1.p pacid=32916137 transcript=Pp3c21_6460V3.1 locus=Pp3c21_6460 annot-version=v3.3 Physcomitrella_patens OG0000024
Pp3c26_7190V3.1.p pacid=32918121 transcript=Pp3c26_7190V3.1 locus=Pp3c26_7190 annot-version=v3.3 Physcomitrella_patens OG0000024
Pp3c26_9710V3.1.p pacid=32918549 transcript=Pp3c26_9710V3.1 locus=Pp3c26_9710 annot-version=v3.3 Physcomitrella_patens OG0000024
Pp3c4_9820V3.1.p pacid=32920761 transcript=Pp3c4_9820V3.1 locus=Pp3c4_9820 annot-version=v3.3 Physcomitrella_patens OG0000024
Pp3c10_15740V3.1.p pacid=32901567 transcript=Pp3c10_15740V3.1 locus=Pp3c10_15740 annot-version=v3.3 Physcomitrella_patens OG0000024
Pp3c4_11830V3.1.p pacid=32922417 transcript=Pp3c4_11830V3.1 locus=Pp3c4_11830 annot-version=v3.3 Physcomitrella_patens OG0000024
Pp3c4_26220V3.1.p pacid=32922500 transcript=Pp3c4_26220V3.1 locus=Pp3c4_26220 annot-version=v3.3 Physcomitrella_patens OG0000024
AT2G39640.1 | glycosyl hydrolase family 17 protein | Chr2:16525171-16527012 REVERSE LENGTH=549 | 201606 Arabidopsis_thaliana OG0000024
AT2G42930.1 | Carbohydrate-binding X8 domain superfamily protein | Chr2:17860868-17861272 REVERSE LENGTH=134 | 201606 Arabidopsis_thaliana OG0000024
AT2G43660.1 | Carbohydrate-binding X8 domain superfamily protein | Chr2:18103818-18104545 REVERSE LENGTH=122 | 201606 Arabidopsis_thaliana OG0000024
AT2G43670.1 | Carbohydrate-binding X8 domain superfamily protein | Chr2:18105145-18105874 FORWARD LENGTH=121 | 201606 Arabidopsis_thaliana OG0000024
AT1G11820.1 | O-Glycosyl hydrolases family 17 protein | Chr1:3991259-3993327 REVERSE LENGTH=384 | 201606 Arabidopsis_thaliana OG0000024
AT3G04010.1 | O-Glycosyl hydrolases family 17 protein | Chr3:1036879-1039263 REVERSE LENGTH=491 | 201606 Arabidopsis_thaliana OG0000024
AT3G07320.1 | O-Glycosyl hydrolases family 17 protein | Chr3:2332324-2333925 REVERSE LENGTH=460 | 201606 Arabidopsis_thaliana OG0000024
AT3G13560.1 | O-Glycosyl hydrolases family 17 protein | Chr3:4425484-4427284 REVERSE LENGTH=505 | 201606 Arabidopsis_thaliana OG0000024

AT3G15800.1 | Glycosyl hydrolase superfamily protein |
Chr3:5345051-5346988 FORWARD LENGTH=399 | 201606
Arabidopsis_thaliana   OG0000024
AT1G13830.1 | Carbohydrate-binding X8 domain superfamily protein |
Chr1:4739999-4740926 REVERSE LENGTH=197 | 201606
Arabidopsis_thaliana   OG0000024
AT3G23770.1 | O-Glycosyl hydrolases family 17 protein |
Chr3:8565556-8567196 FORWARD LENGTH=476 | 201606
Arabidopsis_thaliana   OG0000024
AT3G24330.1 | O-Glycosyl hydrolases family 17 protein |
Chr3:8830336-8831838 FORWARD LENGTH=500 | 201606
Arabidopsis_thaliana   OG0000024
AT3G28250.1 | Carbohydrate-binding X8 domain superfamily protein |
Chr3:10533324-10533689 FORWARD LENGTH=121 | 201606
Arabidopsis_thaliana   OG0000024
AT3G46570.1 | Glycosyl hydrolase superfamily protein |
Chr3:17145797-17146867 REVERSE LENGTH=356 | 201606
Arabidopsis_thaliana   OG0000024
AT3G55430.1 | O-Glycosyl hydrolases family 17 protein |
Chr3:20549806-20552004 REVERSE LENGTH=449 | 201606
Arabidopsis_thaliana   OG0000024
AT3G55780.1 | Glycosyl hydrolase superfamily protein |
Chr3:20705627-20706769 FORWARD LENGTH=380 | 201606
Arabidopsis_thaliana   OG0000024
AT3G57240.1 | beta-1%2C3-glucanase 3 | Chr3:21181916-21183045
REVERSE LENGTH=341 | 201606   Arabidopsis_thaliana
OG0000024
AT3G57260.1 | beta-1%2C3-glucanase 2 | Chr3:21188709-21189822
REVERSE LENGTH=339 | 201606   Arabidopsis_thaliana
OG0000024
AT3G57270.1 | beta-1%2C3-glucanase 1 | Chr3:21191336-21193118
REVERSE LENGTH=340 | 201606   Arabidopsis_thaliana
OG0000024
AT3G58100.1 | plasmodesmata callose-binding protein 5 |
Chr3:21515397-21516329 REVERSE LENGTH=180 | 201606
Arabidopsis_thaliana   OG0000024
AT3G61810.1 | Glycosyl hydrolase family 17 protein |
Chr3:22877168-22878469 FORWARD LENGTH=375 | 201606
Arabidopsis_thaliana   OG0000024
AT4G05430.1 | Carbohydrate-binding X8 domain superfamily protein |
Chr4:2753582-2754324 FORWARD LENGTH=143 | 201606
Arabidopsis_thaliana   OG0000024
AT4G09090.1 | Carbohydrate-binding X8 domain superfamily protein |
Chr4:5807320-5807670 REVERSE LENGTH=116 | 201606
Arabidopsis_thaliana   OG0000024
AT4G09462.1 | Carbohydrate-binding X8 domain superfamily protein |
Chr4:5996860-5997204 FORWARD LENGTH=114 | 201606
Arabidopsis_thaliana   OG0000024
AT4G09464.1 | Carbohydrate-binding X8 domain superfamily protein |
Chr4:5999131-5999481 FORWARD LENGTH=116 | 201606
Arabidopsis_thaliana   OG0000024
AT4G09465.1 | Carbohydrate-binding X8 domain superfamily protein |
Chr4:6001411-6001761 FORWARD LENGTH=116 | 201606
Arabidopsis_thaliana   OG0000024

AT4G09466.1 | Carbohydrate-binding X8 domain superfamily protein | Chr4:6003690-6004040 FORWARD LENGTH=116 | 201606 Arabidopsis_thaliana OG0000024
AT4G09467.1 | Carbohydrate-binding X8 domain superfamily protein | Chr4:6005965-6006315 FORWARD LENGTH=116 | 201606 Arabidopsis_thaliana OG0000024
AT4G13600.1 | Carbohydrate-binding X8 domain superfamily protein | Chr4:7911179-7912892 REVERSE LENGTH=231 | 201606 Arabidopsis_thaliana OG0000024
AT4G14080.1 | O-Glycosyl hydrolases family 17 protein | Chr4:8118697-8120292 REVERSE LENGTH=478 | 201606 Arabidopsis_thaliana OG0000024
AT4G16165.1 | Carbohydrate-binding X8 domain superfamily protein | Chr4:9164167-9164502 FORWARD LENGTH=111 | 201606 Arabidopsis_thaliana OG0000024
AT4G16260.1 | Glycosyl hydrolase superfamily protein | Chr4:9200180-9201441 REVERSE LENGTH=344 | 201606 Arabidopsis_thaliana OG0000024
AT4G17180.1 | O-Glycosyl hydrolases family 17 protein | Chr4:9646541-9648045 FORWARD LENGTH=475 | 201606 Arabidopsis_thaliana OG0000024
AT4G18340.1 | Glycosyl hydrolase superfamily protein | Chr4:10130245-10132019 REVERSE LENGTH=397 | 201606 Arabidopsis_thaliana OG0000024
AT1G18650.1 | plasmodesmata callose-binding protein 3 | Chr1:6419036-6420413 REVERSE LENGTH=184 | 201606 Arabidopsis_thaliana OG0000024
AT4G26830.1 | O-Glycosyl hydrolases family 17 protein | Chr4:13494726-13496487 REVERSE LENGTH=455 | 201606 Arabidopsis_thaliana OG0000024
AT4G29360.1 | O-Glycosyl hydrolases family 17 protein | Chr4:14451562-14453687 REVERSE LENGTH=534 | 201606 Arabidopsis_thaliana OG0000024
AT4G31140.1 | O-Glycosyl hydrolases family 17 protein | Chr4:15141581-15143188 FORWARD LENGTH=484 | 201606 Arabidopsis_thaliana OG0000024
AT4G34480.1 | O-Glycosyl hydrolases family 17 protein | Chr4:16481147-16483988 REVERSE LENGTH=504 | 201606 Arabidopsis_thaliana OG0000024
AT5G08000.1 | glucan endo-1%2C3-beta-glucosidase-like protein 3 | Chr5:2563684-2565282 FORWARD LENGTH=194 | 201606 Arabidopsis_thaliana OG0000024
AT5G18220.1 | O-Glycosyl hydrolases family 17 protein | Chr5:6018914-6020453 REVERSE LENGTH=488 | 201606 Arabidopsis_thaliana OG0000024
AT5G20330.1 | beta-1%2C3-glucanase 4 | Chr5:6871620-6872657 FORWARD LENGTH=345 | 201606 Arabidopsis_thaliana OG0000024
AT5G20340.1 | beta-1%2C3-glucanase 5 | Chr5:6874787-6875851 FORWARD LENGTH=354 | 201606 Arabidopsis_thaliana OG0000024
AT5G20390.1 | Glycosyl hydrolase superfamily protein | Chr5:6892982-6894016 REVERSE LENGTH=344 | 201606 Arabidopsis_thaliana OG0000024
AT5G20560.1 | Glycosyl hydrolase superfamily protein | Chr5:6955368-6956381 FORWARD LENGTH=337 | 201606

Arabidopsis_thaliana OG0000024
AT5G20870.1 | O-Glycosyl hydrolases family 17 protein | Chr5:7080037-7081739 REVERSE LENGTH=501 | 201606
Arabidopsis_thaliana OG0000024
AT5G24318.1 | O-Glycosyl hydrolases family 17 protein | Chr5:8282285-8283956 REVERSE LENGTH=458 | 201606
Arabidopsis_thaliana OG0000024
AT5G35740.1 | Carbohydrate-binding X8 domain superfamily protein | Chr5:13910572-13911095 REVERSE LENGTH=119 | 201606
Arabidopsis_thaliana OG0000024
AT5G42100.1 | beta-1%2C3-glucanase | Chr5:16829460-16831168 REVERSE LENGTH=425 | 201606 Arabidopsis_thaliana OG0000024
AT5G42720.1 | Glycosyl hydrolase family 17 protein | Chr5:17130535-17132763 FORWARD LENGTH=438 | 201606
Arabidopsis_thaliana OG0000024
AT5G53600.1 | Carbohydrate-binding X8 domain superfamily protein | Chr5:21777107-21777442 FORWARD LENGTH=111 | 201606
Arabidopsis_thaliana OG0000024
AT5G53610.1 | Carbohydrate-binding X8 domain superfamily protein | Chr5:21779061-21779393 FORWARD LENGTH=110 | 201606
Arabidopsis_thaliana OG0000024
AT5G55180.1 | O-Glycosyl hydrolases family 17 protein | Chr5:22388834-22390550 FORWARD LENGTH=460 | 201606
Arabidopsis_thaliana OG0000024
AT1G26450.1 | Carbohydrate-binding X8 domain superfamily protein | Chr1:9149003-9150063 REVERSE LENGTH=197 | 201606
Arabidopsis_thaliana OG0000024
AT5G56590.1 | O-Glycosyl hydrolases family 17 protein | Chr5:22907521-22909436 FORWARD LENGTH=506 | 201606
Arabidopsis_thaliana OG0000024
AT5G58090.1 | O-Glycosyl hydrolases family 17 protein | Chr5:23505556-23507193 REVERSE LENGTH=477 | 201606
Arabidopsis_thaliana OG0000024
AT5G58480.1 | O-Glycosyl hydrolases family 17 protein | Chr5:23641227-23642826 REVERSE LENGTH=476 | 201606
Arabidopsis_thaliana OG0000024
AT5G61130.1 | plasmodesmata callose-binding protein 1 | Chr5:24587438-24589369 REVERSE LENGTH=201 | 201606
Arabidopsis_thaliana OG0000024
AT5G63225.1 | Carbohydrate-binding X8 domain superfamily protein | Chr5:25356351-25356683 REVERSE LENGTH=110 | 201606
Arabidopsis_thaliana OG0000024
AT5G63230.1 | Carbohydrate-binding X8 domain superfamily protein | Chr5:25357473-25357808 REVERSE LENGTH=111 | 201606
Arabidopsis_thaliana OG0000024
AT5G63240.1 | Carbohydrate-binding X8 domain superfamily protein | Chr5:25359050-25359439 REVERSE LENGTH=129 | 201606
Arabidopsis_thaliana OG0000024
AT5G63250.1 | Carbohydrate-binding X8 domain superfamily protein | Chr5:25360263-25360661 REVERSE LENGTH=132 | 201606
Arabidopsis_thaliana OG0000024
AT5G64790.1 | O-Glycosyl hydrolases family 17 protein | Chr5:25902713-25904559 FORWARD LENGTH=485 | 201606
Arabidopsis_thaliana OG0000024

AT5G67460.1 | O-Glycosyl hydrolases family 17 protein |
Chr5:26921984-26923274 REVERSE LENGTH=380 | 201606
Arabidopsis_thaliana   OG0000024
AT1G29380.1 | Carbohydrate-binding X8 domain superfamily protein |
Chr1:10284209-10285671 FORWARD LENGTH=315 | 201606
Arabidopsis_thaliana   OG0000024
AT1G30080.1 | Glycosyl hydrolase superfamily protein |
Chr1:10551231-10553167 REVERSE LENGTH=408 | 201606
Arabidopsis_thaliana   OG0000024
AT1G32860.1 | Glycosyl hydrolase superfamily protein |
Chr1:11907308-11908803 REVERSE LENGTH=426 | 201606
Arabidopsis_thaliana   OG0000024
AT1G33220.1 | Glycosyl hydrolase superfamily protein |
Chr1:12044872-12045879 FORWARD LENGTH=335 | 201606
Arabidopsis_thaliana   OG0000024
AT1G64760.1 | O-Glycosyl hydrolases family 17 protein |
Chr1:24054220-24056194 REVERSE LENGTH=481 | 201606
Arabidopsis_thaliana   OG0000024
AT1G66250.1 | O-Glycosyl hydrolases family 17 protein |
Chr1:24693063-24695416 FORWARD LENGTH=505 | 201606
Arabidopsis_thaliana   OG0000024
AT1G66852.1 | Carbohydrate-binding X8 domain superfamily protein |
Chr1:24938500-24938832 REVERSE LENGTH=110 | 201606
Arabidopsis_thaliana   OG0000024
AT1G66855.1 | Carbohydrate-binding X8 domain superfamily protein |
Chr1:24939676-24940011 REVERSE LENGTH=111 | 201606
Arabidopsis_thaliana   OG0000024
AT1G66870.1 | Carbohydrate-binding X8 domain superfamily protein |
Chr1:24945542-24945877 REVERSE LENGTH=111 | 201606
Arabidopsis_thaliana   OG0000024
AT1G69295.1 | plasmodesmata callose-binding protein 4 |
Chr1:26050492-26051843 REVERSE LENGTH=222 | 201606
Arabidopsis_thaliana   OG0000024
AT1G77780.1 | Glycosyl hydrolase superfamily protein |
Chr1:29248437-29249528 REVERSE LENGTH=363 | 201606
Arabidopsis_thaliana   OG0000024
AT1G77790.1 | Glycosyl hydrolase superfamily protein |
Chr1:29250692-29251976 REVERSE LENGTH=346 | 201606
Arabidopsis_thaliana   OG0000024
AT1G78520.1 | Carbohydrate-binding X8 domain superfamily protein |
Chr1:29537976-29538656 REVERSE LENGTH=115 | 201606
Arabidopsis_thaliana   OG0000024
AT1G79480.1 | Carbohydrate-binding X8 domain superfamily protein |
Chr1:29897905-29899267 REVERSE LENGTH=397 | 201606
Arabidopsis_thaliana   OG0000024
AT2G01630.1 | O-Glycosyl hydrolases family 17 protein |
Chr2:279541-281955 REVERSE LENGTH=501 | 201606
Arabidopsis_thaliana   OG0000024
AT2G03505.1 | Carbohydrate-binding X8 domain superfamily protein |
Chr2:1064317-1065094 FORWARD LENGTH=168 | 201606
Arabidopsis_thaliana   OG0000024
AT2G04910.1 | Carbohydrate-binding X8 domain superfamily protein |
Chr2:1723980-1724354 REVERSE LENGTH=124 | 201606
Arabidopsis_thaliana   OG0000024

AT2G05790.1 | O-Glycosyl hydrolases family 17 protein |
Chr2:2199450-2201293 FORWARD LENGTH=472 | 201606
Arabidopsis_thaliana   OG0000024
AT2G16230.1 | O-Glycosyl hydrolases family 17 protein |
Chr2:7035463-7038326 REVERSE LENGTH=503 | 201606
Arabidopsis_thaliana   OG0000024
AT2G19440.1 | O-Glycosyl hydrolases family 17 protein |
Chr2:8418164-8419806 REVERSE LENGTH=478 | 201606
Arabidopsis_thaliana   OG0000024
AT1G09460.1 | Carbohydrate-binding X8 domain superfamily protein |
Chr1:3053901-3055090 FORWARD LENGTH=330 | 201606
Arabidopsis_thaliana   OG0000024
AT2G26600.1 | Glycosyl hydrolase superfamily protein |
Chr2:11316727-11318215 FORWARD LENGTH=388 | 201606
Arabidopsis_thaliana   OG0000024
AT2G27500.1 | Glycosyl hydrolase superfamily protein |
Chr2:11752364-11753844 REVERSE LENGTH=392 | 201606
Arabidopsis_thaliana   OG0000024
AT2G29735.1 | major pollen allergen Ole e-like protein |
Chr2:12705346-12705699 REVERSE LENGTH=117 | 201606
Arabidopsis_thaliana   OG0000024
AT2G30933.1 | Carbohydrate-binding X8 domain superfamily protein |
Chr2:13164179-13166517 REVERSE LENGTH=227 | 201606
Arabidopsis_thaliana   OG0000024
Os03t0246100-01 Glycoside hydrolase, family 17 protein.
Oryza_sativa   OG0000024
Os03t0296500-00 Similar to Glycosyl hydrolases family 17 protein,
expressed.   Oryza_sativa   OG0000024
Os01t0243700-01 Similar to Beta-1,3-glucanase-like protein.
Oryza_sativa   OG0000024
Os03t0374600-01 Glycoside hydrolase, family 17 protein.
Oryza_sativa   OG0000024
Os03t0397600-00 Glycoside hydrolase, family 17 protein.
Oryza_sativa   OG0000024
Os03t0421800-01 X8 domain containing protein.   Oryza_sativa
OG0000024
Os03t0600600-00 Similar to Beta-1,3-glucanase.   Oryza_sativa
OG0000024
Os03t0656800-01 Similar to 3-glucanase.   Oryza_sativa
OG0000024
Os03t0669300-01 Beta-1,3-glucanase-like protein.   Oryza_sativa
OG0000024
Os03t0722500-01 Glycoside hydrolase, family 17 protein.
Oryza_sativa   OG0000024
Os03t0756300-01 X8 domain containing protein.   Oryza_sativa
OG0000024
Os03t0792800-01 Beta-1,3-glucanase-like protein.   Oryza_sativa
OG0000024
Os03t0833600-01 X8 domain containing protein.   Oryza_sativa
OG0000024
Os03t0845600-01 Glycoside hydrolase, family 17 protein.
Oryza_sativa   OG0000024
Os04t0412300-01 Similar to H0717B12.10 protein.   Oryza_sativa
OG0000024

Os04t0681950-00 Similar to H0124B04.9 protein.	Oryza_sativa	OG0000024
Os05t0375400-01 Beta-glucanase precursor.	Oryza_sativa	OG0000024
Os05t0495900-01 Similar to Beta-1,3-glucanase precursor (Fragment).	Oryza_sativa	OG0000024
Os05t0512600-01 X8 domain containing protein.	Oryza_sativa	OG0000024
Os05t0535100-01 Similar to Beta-1,3-glucanase-like protein.	Oryza_sativa	OG0000024
Os05t0581900-01 X8 domain containing protein.	Oryza_sativa	OG0000024
Os06t0131500-01 Glycoside hydrolase, family 17 protein.	Oryza_sativa	OG0000024
Os06t0531000-00 Similar to predicted protein.	Oryza_sativa	OG0000024
Os06t0537700-01 X8 domain containing protein.	Oryza_sativa	OG0000024
Os06t0590600-01 Similar to Beta-1,3-glucanase-like protein.	Oryza_sativa	OG0000024
Os06t0607000-01 Similar to Beta-1,3-glucanase.	Oryza_sativa	OG0000024
Os06t0665200-00 Similar to glycosyl hydrolase family protein 17.	Oryza_sativa	OG0000024
Os07t0149900-01 X8 domain containing protein.	Oryza_sativa	OG0000024
Os07t0168600-01 Similar to 3-glucanase.	Oryza_sativa	OG0000024
Os07t0240200-01 Similar to Beta-1,3 glucanase precursor (EC 3.2.1.39).	Oryza_sativa	OG0000024
Os07t0510200-01 Glycoside hydrolase, family 17 protein.	Oryza_sativa	OG0000024
Os07t0538000-01 Similar to hydrolase, hydrolyzing O-glycosyl compounds.	Oryza_sativa	OG0000024
Os07t0539100-01 Glycoside hydrolase, family 17 protein.	Oryza_sativa	OG0000024
Os07t0539300-01 Glycoside hydrolase, family 17 protein.	Oryza_sativa	OG0000024
Os07t0539400-00 Similar to hydrolase, hydrolyzing O-glycosyl compounds.	Oryza_sativa	OG0000024
Os07t0539900-01 Similar to Beta-1,3-glucanase-like protein.	Oryza_sativa	OG0000024
Os07t0577300-01 Glycoside hydrolase, family 17 protein.	Oryza_sativa	OG0000024
Os07t0600700-01 X8 domain containing protein.	Oryza_sativa	OG0000024
Os07t0633100-01 Similar to Glucan endo-1,3-beta-glucosidase 4.	Oryza_sativa	OG0000024
Os01t0631500-01 Similar to Beta-1,3-glucanase-like protein.	Oryza_sativa	OG0000024
Os08t0135500-01 X8 domain containing protein.	Oryza_sativa	OG0000024
Os08t0224500-01 Similar to 3-glucanase.	Oryza_sativa	OG0000024

Os08t0244500-00 Similar to hydrolase, hydrolyzing O-glycosyl compounds.	Oryza_sativa	OG0000024
Os08t0326500-01 Similar to glycosyl hydrolase family 17 protein.	Oryza_sativa	OG0000024
Os08t0525800-00 Similar to glucan endo-1,3-beta-glucosidase A6.	Oryza_sativa	OG0000024
Os09t0272300-01 Similar to 3-glucanase.	Oryza_sativa	OG0000024
Os09t0502200-01 Similar to Beta-1,3-glucanase (Fragment).	Oryza_sativa	OG0000024
Os09t0533200-00 Glycoside hydrolase, family 17 domain containing protein.	Oryza_sativa	OG0000024
Os09t0560850-00 Similar to T8K14.10.	Oryza_sativa	OG0000024
Os10t0160100-01 Glycoside hydrolase, family 17 protein.	Oryza_sativa	OG0000024
Os10t0347000-01 X8 domain containing protein.	Oryza_sativa	OG0000024
Os01t0713200-01 Similar to Beta-glucanase.	Oryza_sativa	OG0000024
Os01t0739700-01 Glycoside hydrolase, family 17 protein.	Oryza_sativa	OG0000024
Os11t0577800-00 Similar to Glucan endo-1,3-beta-glucosidase 5.	Oryza_sativa	OG0000024
Os11t0704600-01 Similar to Beta-1,3 glucanase precursor (EC 3.2.1.39).	Oryza_sativa	OG0000024
Os01t0763900-01 X8 domain containing protein.	Oryza_sativa	OG0000024
Os01t0801500-01 Beta-1,3-glucanase precursor.	Oryza_sativa	OG0000024
Os01t0860800-01 Glycoside hydrolase, family 17 protein.	Oryza_sativa	OG0000024
Os01t0940700-01 Similar to Glucan endo-1,3-beta-glucosidase GII precursor (EC 3.2.1.39) ((1->3)- beta-glucan endohydrolase GII) ((1->3)-beta-glucanase isoenzyme GII) (Beta-1,3-endoglucanase GII).	Oryza_sativa	OG0000024
Os01t0940800-01 Similar to Beta-1,3-glucanase precursor.	Oryza_sativa	OG0000024
Os01t0941200-01 Similar to Glucan endo-1,3-beta-glucosidase GII precursor (EC 3.2.1.39) ((1->3)- beta-glucan endohydrolase GII) ((1->3)-beta-glucanase isoenzyme GII) (Beta-1,3-endoglucanase GII).	Oryza_sativa	OG0000024
Os01t0941500-00 Similar to Beta-1,3-glucanase.	Oryza_sativa	OG0000024
Os01t0942300-01 Similar to Beta glucanase precursor (EC 3.2.1.73) (Fragment).	Oryza_sativa	OG0000024
Os01t0944500-00 Glycoside hydrolase, family 17 domain containing protein.	Oryza_sativa	OG0000024
Os01t0944700-01 Similar to Beta-1,3-glucanase precursor.	Oryza_sativa	OG0000024
Os01t0944800-00 Similar to Beta-1,3-glucanase.	Oryza_sativa	OG0000024
Os01t0946500-01 Similar to Glucan endo-1,3-beta-glucosidase GV (EC 3.2.1.39) ((1->3)-beta-glucan endohydrolase GV) ((1->3)-beta-

glucanase isoenzyme GV) (Beta-1,3- endoglucanase GV).
Oryza_sativa OG0000024
Os01t0946700-01 Similar to Glucan endo-1,3-beta-glucosidase GV (EC 3.2.1.39) ((1->3)-beta-glucan endohydrolase GV) ((1->3)-beta-glucanase isoenzyme GV) (Beta-1,3- endoglucanase GV).
Oryza_sativa OG0000024
Os01t0947000-01 Similar to Beta-1,3-glucanase precursor.
Oryza_sativa OG0000024
Os02t0139300-01 Glycoside hydrolase, family 17 protein.
Oryza_sativa OG0000024
Os02t0200300-01 Similar to Beta-1,3-glucanase-like protein.
Oryza_sativa OG0000024
Os02t0532900-01 Glycoside hydrolase, family 17 protein.
Oryza_sativa OG0000024
Os02t0771700-01 Glycoside hydrolase, family 17 protein.
Oryza_sativa OG0000024
Os03t0221500-01 Glycoside hydrolase, family 17 protein.
Oryza_sativa OG0000024
Os03t0227400-01 Similar to glucan endo-1,3-beta-glucosidase 7.
Oryza_sativa OG0000024
g1570.t1 Caulerpa_lentillifera OG0000025
g6331.t1 Caulerpa_lentillifera OG0000025
g6347.t1 Caulerpa_lentillifera OG0000025
g6363.t1 Caulerpa_lentillifera OG0000025
g6393.t1 Caulerpa_lentillifera OG0000025
g8495.t1 Caulerpa_lentillifera OG0000025
Cre03.g206800.t1.1 pacid=30788187 transcript=Cre03.g206800.t1.1 locus=Cre03.g206800 ID=Cre03.g206800.t1.1.v5.5 annot-version=v5.5
Chlamydomonas_reinhardtii OG0000025
Cre17.g740470.t1.1 pacid=30781692 transcript=Cre17.g740470.t1.1 locus=Cre17.g740470 ID=Cre17.g740470.t1.1.v5.5 annot-version=v5.5
Chlamydomonas_reinhardtii OG0000025
Cre17.g740430.t1.1 pacid=30782575 transcript=Cre17.g740430.t1.1 locus=Cre17.g740430 ID=Cre17.g740430.t1.1.v5.5 annot-version=v5.5
Chlamydomonas_reinhardtii OG0000025
Vocar.0011s0175.1.p pacid=32895290 transcript=Vocar.0011s0175.1 locus=Vocar.0011s0175 ID=Vocar.0011s0175.1.v2.1 annot-version=v2.1
Volvox_carteri OG0000025
Vocar.0040s0016.1.p pacid=32886228 transcript=Vocar.0040s0016.1 locus=Vocar.0040s0016 ID=Vocar.0040s0016.1.v2.1 annot-version=v2.1
Volvox_carteri OG0000025
jgi|ChlNC64A_1|23239|e_gw1.10.28.1 Chlorella_variabilis OG0000025
jgi|ChlNC64A_1|33524|estExt_Genewise1Plus.C_10281
Chlorella_variabilis OG0000025
jgi|ChlNC64A_1|55965|estExt_fgenesh3_pm.C_20010
Chlorella_variabilis OG0000025
jgi|ChlNC64A_1|137750|IGS.gm_2_00217 Chlorella_variabilis OG0000025
jgi|ChlNC64A_1|33950|estExt_Genewise1Plus.C_20586
Chlorella_variabilis OG0000025
jgi|ChlNC64A_1|136418|IGS.gm_16_00141 Chlorella_variabilis OG0000025
jgi|ChlNC64A_1|59732|estExt_fgenesh3_kg.C_160005

Chlorella_variabilis	OG0000025
jgi|ChlNC64A_1|8066|gw1.13.28.1	Chlorella_variabilis
OG0000025
jgi|ChlNC64A_1|139619|IGS.gm_25_00170	Chlorella_variabilis
OG0000025
jgi|ChlNC64A_1|25635|e_gw1.17.22.1	Chlorella_variabilis
OG0000025
jgi|ChlNC64A_1|28837|e_gw1.50.10.1	Chlorella_variabilis
OG0000025
jgi|ChlNC64A_1|59832|estExt_fgenesh3_kg.C_500001
Chlorella_variabilis	OG0000025
jgi|ChlNC64A_1|28988|e_gw1.178.1.1	Chlorella_variabilis
OG0000025
29643 pacid=27385650 transcript=29643
locus=estExt_Genewise1Plus.C_100250 ID=29643.2.0.227 annot-
version=v2.0	Coccomyxa_subellipsoidea	OG0000025
67173 pacid=27387804 transcript=67173
locus=estExt_Genemark1.C_140034 ID=67173.2.0.227 annot-version=v2.0
Coccomyxa_subellipsoidea	OG0000025
53762 pacid=27385853 transcript=53762
locus=estExt_fgenesh1_pm.C_100087 ID=53762.2.0.227 annot-
version=v2.0	Coccomyxa_subellipsoidea	OG0000025
43798 pacid=27388020 transcript=43798 locus=fgenesh1_pg.14_#_213
ID=43798.2.0.227 annot-version=v2.0	Coccomyxa_subellipsoidea
OG0000025
37001 pacid=27385947 transcript=37001 locus=fgenesh1_pm.10_#_89
ID=37001.2.0.227 annot-version=v2.0	Coccomyxa_subellipsoidea
OG0000025
29606 pacid=27385974 transcript=29606
locus=estExt_Genewise1Plus.C_100201 ID=29606.2.0.227 annot-
version=v2.0	Coccomyxa_subellipsoidea	OG0000025
34726 pacid=27389027 transcript=34726 locus=fgenesh1_pm.1_#_67
ID=34726.2.0.227 annot-version=v2.0	Coccomyxa_subellipsoidea
OG0000025
52747 pacid=27389673 transcript=52747
locus=estExt_fgenesh1_pm.C_40025 ID=52747.2.0.227 annot-version=v2.0
	Coccomyxa_subellipsoidea	OG0000025
13590 pacid=27390167 transcript=13590 locus=e_gw1.4.253.1
ID=13590.2.0.227 annot-version=v2.0	Coccomyxa_subellipsoidea
OG0000025
32675 pacid=27390245 transcript=32675 locus=fgenesh1_kg.
4_#_150_#_581_2_CBOZ_CBPA ID=32675.2.0.227 annot-version=v2.0
Coccomyxa_subellipsoidea	OG0000025
13172 pacid=27390422 transcript=13172 locus=e_gw1.3.106.1
ID=13172.2.0.227 annot-version=v2.0	Coccomyxa_subellipsoidea
OG0000025
27364 pacid=27390896 transcript=27364
locus=estExt_Genewise1Plus.C_30010 ID=27364.2.0.227 annot-
version=v2.0	Coccomyxa_subellipsoidea	OG0000025
10278 pacid=27392032 transcript=10278 locus=gw1.11.350.1
ID=10278.2.0.227 annot-version=v2.0	Coccomyxa_subellipsoidea
OG0000025
17100 pacid=27392103 transcript=17100 locus=e_gw1.11.87.1
ID=17100.2.0.227 annot-version=v2.0	Coccomyxa_subellipsoidea

OG0000025
66691 pacid=27392127 transcript=66691
locus=estExt_Genemark1.C_110079 ID=66691.2.0.227 annot-version=v2.0
Coccomyxa_subellipsoidea OG0000025
38319 pacid=27392880 transcript=38319 locus=fgenesh1_pm.20_#_11
ID=38319.2.0.227 annot-version=v2.0 Coccomyxa_subellipsoidea
OG0000025
12177 pacid=27393733 transcript=12177 locus=e_gw1.2.225.1
ID=12177.2.0.227 annot-version=v2.0 Coccomyxa_subellipsoidea
OG0000025
30742 pacid=27393963 transcript=30742
locus=estExt_Genewise1Plus.C_160060 ID=30742.2.0.227 annot-
version=v2.0 Coccomyxa_subellipsoidea OG0000025
54368 pacid=27394840 transcript=54368
locus=estExt_fgenesh1_pm.C_150101 ID=54368.2.0.227 annot-
version=v2.0 Coccomyxa_subellipsoidea OG0000025
16077_e_gw1.03.00.159.1 Ostreococcus_tauri OG0000025
29494_estExt_gwp_GeneWisePlus.C_Chr_14.00010351
Ostreococcus_tauri OG0000025
29895_0000060001 Ostreococcus_tauri OG0000025
31482_0300010036 Ostreococcus_tauri OG0000025
31673_0300010227 Ostreococcus_tauri OG0000025
33642_0600010489 Ostreococcus_tauri OG0000025
30357 pacid=27412990 transcript=30357 locus=eugene.0300010033
ID=30357.2.0.231 annot-version=v2.0 Ostreococcus_lucimarinus
OG0000025
34203 pacid=27413096 transcript=34203 locus=e_gwEuk.3.140.1
ID=34203.2.0.231 annot-version=v2.0 Ostreococcus_lucimarinus
OG0000025
3240 pacid=27413107 transcript=3240 locus=gwEuk.3.235.1
ID=3240.2.0.231 annot-version=v2.0 Ostreococcus_lucimarinus
OG0000025
41151 pacid=27418225 transcript=41151 locus=e_gwEuk.6.165.1
ID=41151.2.0.231 annot-version=v2.0 Ostreococcus_lucimarinus
OG0000025
52000 pacid=27420092 transcript=52000
locus=estExt_Genewise_ext.C_Chr_200384 ID=52000.2.0.231 annot-
version=v2.0 Ostreococcus_lucimarinus OG0000025
35010 pacid=27413614 transcript=35010 locus=e_gwEuk.9.140.1
ID=35010.2.0.231 annot-version=v2.0 Ostreococcus_lucimarinus
OG0000025
21673 pacid=27348986 transcript=21673 locus=MicpuC2.e_gw1.12.90.1
ID=21673.3.0.228 annot-version=v3.0 Micromonas_pusilla
OG0000025
34108 pacid=27340772 transcript=34108
locus=MicpuC2.estExt_Genewise1Plus.C_70057 ID=34108.3.0.228 annot-
version=v3.0 Micromonas_pusilla OG0000025
70846 pacid=27343180 transcript=70846 locus=MicpuC2.DRO_EuGene.
0000020121 ID=70846.3.0.228 annot-version=v3.0
Micromonas_pusilla OG0000025
208966 pacid=27343751 transcript=209006
locus=estExt_fgenesh1_kg.C_190004 ID=209006.3.0.228 annot-
version=v3.0 Micromonas_pusilla OG0000025
44777 pacid=27339298 transcript=44777

locus=MicpuC2.fgenesh1_pm.C_scaffold_11000033 ID=44777.3.0.228
annot-version=v3.0   Micromonas_pusilla   OG0000025
30532 pacid=27345795 transcript=30532
locus=MicpuC2.estExt_Genewise1Plus.C_10003 ID=30532.3.0.228 annot-version=v3.0   Micromonas_pusilla   OG0000025
178918 pacid=27347959 transcript=178958
locus=estExt_Genewise1Plus.C_6_t20077 ID=178958.3.0.228 annot-version=v3.0   Micromonas_pusilla   OG0000025
45985 pacid=27348478 transcript=45985
locus=MicpuC2.estExt_fgenesh1_pm.C_150053 ID=45985.3.0.228 annot-version=v3.0   Micromonas_pusilla   OG0000025
56847 pacid=27398289 transcript=56847 locus=EuGene.0300010275
ID=56847.3.0.229 annot-version=v3.0   Micromonas_sp._RCC299
OG0000025
54776 pacid=27399425 transcript=54776 locus=EuGene.0100010003
ID=54776.3.0.229 annot-version=v3.0   Micromonas_sp._RCC299
OG0000025
113379 pacid=27400065 transcript=113379 locus=AZW_EuGene.0400010352
ID=113379.3.0.229 annot-version=v3.0   Micromonas_sp._RCC299
OG0000025
87431 pacid=27402886 transcript=87431 locus=e_gw2.13.82.1
ID=87431.3.0.229 annot-version=v3.0   Micromonas_sp._RCC299
OG0000025
109423 pacid=27402926 transcript=109423
locus=est_cluster_kg.Chr_13__106__3202092:1 ID=109423.3.0.229 annot-version=v3.0   Micromonas_sp._RCC299   OG0000025
108749 pacid=27403069 transcript=108749
locus=est_cluster_kg.Chr_08__32__3199321:1 ID=108749.3.0.229 annot-version=v3.0   Micromonas_sp._RCC299   OG0000025
109447 pacid=27403923 transcript=109447
locus=est_cluster_kg.Chr_14__23__3200873:1 ID=109447.3.0.229 annot-version=v3.0   Micromonas_sp._RCC299   OG0000025
kfl00745_0020_v1.1   Klebsormidium_flaccidum OG0000025
kfl00844_0010_v1.1   Klebsormidium_flaccidum OG0000025
kfl00923_0030_v1.1   Klebsormidium_flaccidum OG0000025
kfl00036_0160_v1.1   Klebsormidium_flaccidum OG0000025
kfl00036_0180_v1.1   Klebsormidium_flaccidum OG0000025
kfl00036_0190_v1.1   Klebsormidium_flaccidum OG0000025
kfl00046_0020_v1.1   Klebsormidium_flaccidum OG0000025
kfl00053_0460_v1.1   Klebsormidium_flaccidum OG0000025
kfl00080_0060_v1.1   Klebsormidium_flaccidum OG0000025
kfl00090_0290_v1.1   Klebsormidium_flaccidum OG0000025
kfl00175_0180_v1.1   Klebsormidium_flaccidum OG0000025
Pp3c15_10670V3.1.p pacid=32926882 transcript=Pp3c15_10670V3.1
locus=Pp3c15_10670 annot-version=v3.3   Physcomitrella_patens
OG0000025
Pp3c15_3470V3.1.p pacid=32929594 transcript=Pp3c15_3470V3.1
locus=Pp3c15_3470 annot-version=v3.3   Physcomitrella_patens
OG0000025
Pp3c20_880V3.1.p pacid=32946151 transcript=Pp3c20_880V3.1
locus=Pp3c20_880 annot-version=v3.3   Physcomitrella_patens
OG0000025
Pp3c20_16420V3.1.p pacid=32948146 transcript=Pp3c20_16420V3.1
locus=Pp3c20_16420 annot-version=v3.3   Physcomitrella_patens

OG0000025
Pp3c22_23090V3.1.p pacid=32904548 transcript=Pp3c22_23090V3.1
locus=Pp3c22_23090 annot-version=v3.3   Physcomitrella_patens
OG0000025
Pp3c5_28630V3.1.p pacid=32955573 transcript=Pp3c5_28630V3.1
locus=Pp3c5_28630 annot-version=v3.3   Physcomitrella_patens
OG0000025
Pp3c8_25010V3.1.p pacid=32963552 transcript=Pp3c8_25010V3.1
locus=Pp3c8_25010 annot-version=v3.3   Physcomitrella_patens
OG0000025
Pp3c8_7000V3.1.p pacid=32964667 transcript=Pp3c8_7000V3.1
locus=Pp3c8_7000 annot-version=v3.3   Physcomitrella_patens
OG0000025
Pp3c8_6990V3.1.p pacid=32965586 transcript=Pp3c8_6990V3.1
locus=Pp3c8_6990 annot-version=v3.3   Physcomitrella_patens
OG0000025
Pp3c8_25020V3.1.p pacid=32965846 transcript=Pp3c8_25020V3.1
locus=Pp3c8_25020 annot-version=v3.3   Physcomitrella_patens
OG0000025
Pp3c25_14870V3.1.p pacid=32979312 transcript=Pp3c25_14870V3.1
locus=Pp3c25_14870 annot-version=v3.3   Physcomitrella_patens
OG0000025
Pp3c18_1410V3.1.p pacid=32982459 transcript=Pp3c18_1410V3.1
locus=Pp3c18_1410 annot-version=v3.3   Physcomitrella_patens
OG0000025
Pp3c18_17510V3.1.p pacid=32983125 transcript=Pp3c18_17510V3.1
locus=Pp3c18_17510 annot-version=v3.3   Physcomitrella_patens
OG0000025
Pp3c16_24860V3.1.p pacid=32986841 transcript=Pp3c16_24860V3.1
locus=Pp3c16_24860 annot-version=v3.3   Physcomitrella_patens
OG0000025
Pp3c24_20800V3.1.p pacid=32908807 transcript=Pp3c24_20800V3.1
locus=Pp3c24_20800 annot-version=v3.3   Physcomitrella_patens
OG0000025
Pp3c24_20780V3.1.p pacid=32910477 transcript=Pp3c24_20780V3.1
locus=Pp3c24_20780 annot-version=v3.3   Physcomitrella_patens
OG0000025
Pp3c9_17060V3.1.p pacid=32912442 transcript=Pp3c9_17060V3.1
locus=Pp3c9_17060 annot-version=v3.3   Physcomitrella_patens
OG0000025
Pp3c9_4110V3.1.p pacid=32913088 transcript=Pp3c9_4110V3.1
locus=Pp3c9_4110 annot-version=v3.3   Physcomitrella_patens
OG0000025
Pp3c26_3990V3.1.p pacid=32918213 transcript=Pp3c26_3990V3.1
locus=Pp3c26_3990 annot-version=v3.3   Physcomitrella_patens
OG0000025
Pp3c7_6450V3.1.p pacid=32923459 transcript=Pp3c7_6450V3.1
locus=Pp3c7_6450 annot-version=v3.3   Physcomitrella_patens
OG0000025
AT2G35740.1 | nositol transporter 3 | Chr2:15024489-15026414 REVERSE
LENGTH=580 | 201606   Arabidopsis_thaliana   OG0000025
AT2G43330.1 | inositol transporter 1 | Chr2:18001135-18003854
FORWARD LENGTH=509 | 201606   Arabidopsis_thaliana
OG0000025

AT1G11260.1 | sugar transporter 1 | Chr1:3777460-3780133 FORWARD LENGTH=522 | 201606 Arabidopsis_thaliana OG0000025
AT2G48020.1 | Major facilitator superfamily protein | Chr2:19644441-19647007 FORWARD LENGTH=463 | 201606 Arabidopsis_thaliana OG0000025
AT3G03090.1 | vacuolar glucose transporter 1 | Chr3:700749-704579 REVERSE LENGTH=503 | 201606 Arabidopsis_thaliana OG0000025
AT3G05150.1 | Major facilitator superfamily protein | Chr3:1440216-1443361 FORWARD LENGTH=470 | 201606 Arabidopsis_thaliana OG0000025
AT3G05155.1 | Major facilitator superfamily protein | Chr3:1448647-1452023 FORWARD LENGTH=459 | 201606 Arabidopsis_thaliana OG0000025
AT3G05160.1 | Major facilitator superfamily protein | Chr3:1453267-1456997 REVERSE LENGTH=458 | 201606 Arabidopsis_thaliana OG0000025
AT3G05165.1 | Major facilitator superfamily protein | Chr3:1458287-1462737 REVERSE LENGTH=467 | 201606 Arabidopsis_thaliana OG0000025
AT3G05400.1 | Major facilitator superfamily protein | Chr3:1549702-1553942 FORWARD LENGTH=462 | 201606 Arabidopsis_thaliana OG0000025
AT3G05960.1 | sugar transporter 6 | Chr3:1783587-1785334 REVERSE LENGTH=507 | 201606 Arabidopsis_thaliana OG0000025
AT3G18830.1 | polyol/monosaccharide transporter 5 | Chr3:6489000-6491209 REVERSE LENGTH=539 | 201606 Arabidopsis_thaliana OG0000025
AT3G19930.1 | sugar transporter 4 | Chr3:6935048-6936841 FORWARD LENGTH=514 | 201606 Arabidopsis_thaliana OG0000025
AT3G19940.1 | Major facilitator superfamily protein | Chr3:6938211-6939975 FORWARD LENGTH=514 | 201606 Arabidopsis_thaliana OG0000025
AT3G20460.1 | Major facilitator superfamily protein | Chr3:7135050-7139469 FORWARD LENGTH=488 | 201606 Arabidopsis_thaliana OG0000025
AT4G02050.1 | sugar transporter protein 7 | Chr4:898387-900095 REVERSE LENGTH=513 | 201606 Arabidopsis_thaliana OG0000025
AT4G04750.1 | Major facilitator superfamily protein | Chr4:2418110-2422624 FORWARD LENGTH=482 | 201606 Arabidopsis_thaliana OG0000025
AT4G04760.1 | Major facilitator superfamily protein | Chr4:2424164-2427769 FORWARD LENGTH=478 | 201606 Arabidopsis_thaliana OG0000025
AT4G16480.1 | inositol transporter 4 | Chr4:9291246-9293083 FORWARD LENGTH=582 | 201606 Arabidopsis_thaliana OG0000025
AT4G21480.1 | sugar transporter protein 12 | Chr4:11433320-11435284 REVERSE LENGTH=502 | 201606 Arabidopsis_thaliana OG0000025
AT1G19450.1 | Major facilitator superfamily protein | Chr1:6731671-6734633 REVERSE LENGTH=488 | 201606 Arabidopsis_thaliana OG0000025
AT4G36670.1 | Major facilitator superfamily protein |

Chr4:17287680-17289483 REVERSE LENGTH=493 | 201606
Arabidopsis_thaliana   OG0000025
AT5G16150.1 | plastidic GLC translocator | Chr5:5272904-5275678
FORWARD LENGTH=546 | 201606   Arabidopsis_thaliana
OG0000025
AT5G17010.1 | Major facilitator superfamily protein |
Chr5:5587851-5592332 REVERSE LENGTH=503 | 201606
Arabidopsis_thaliana   OG0000025
AT5G18840.1 | Major facilitator superfamily protein |
Chr5:6282954-6286399 FORWARD LENGTH=482 | 201606
Arabidopsis_thaliana   OG0000025
AT5G23270.1 | sugar transporter 11 | Chr5:7839132-7840874 FORWARD
LENGTH=514 | 201606   Arabidopsis_thaliana   OG0000025
AT5G26250.1 | Major facilitator superfamily protein |
Chr5:9196758-9198681 FORWARD LENGTH=507 | 201606
Arabidopsis_thaliana   OG0000025
AT5G26340.1 | Major facilitator superfamily protein |
Chr5:9243851-9246994 REVERSE LENGTH=526 | 201606
Arabidopsis_thaliana   OG0000025
AT5G27350.1 | Major facilitator superfamily protein |
Chr5:9648958-9654176 FORWARD LENGTH=474 | 201606
Arabidopsis_thaliana   OG0000025
AT5G27360.1 | Major facilitator superfamily protein |
Chr5:9657119-9662425 FORWARD LENGTH=478 | 201606
Arabidopsis_thaliana   OG0000025
AT5G59250.1 | Major facilitator superfamily protein |
Chr5:23903958-23906853 FORWARD LENGTH=558 | 201606
Arabidopsis_thaliana   OG0000025
AT5G61520.1 | Major facilitator superfamily protein |
Chr5:24739358-24741175 REVERSE LENGTH=514 | 201606
Arabidopsis_thaliana   OG0000025
AT1G30220.1 | inositol transporter 2 | Chr1:10632957-10635439
REVERSE LENGTH=580 | 201606   Arabidopsis_thaliana
OG0000025
AT1G34580.1 | Major facilitator superfamily protein |
Chr1:12660631-12663553 FORWARD LENGTH=506 | 201606
Arabidopsis_thaliana   OG0000025
AT1G50310.1 | sugar transporter 9 | Chr1:18635984-18638110 FORWARD
LENGTH=517 | 201606   Arabidopsis_thaliana   OG0000025
AT1G05030.1 | Major facilitator superfamily protein |
Chr1:1438324-1441385 REVERSE LENGTH=524 | 201606
Arabidopsis_thaliana   OG0000025
AT1G67300.1 | Major facilitator superfamily protein |
Chr1:25193832-25196751 REVERSE LENGTH=493 | 201606
Arabidopsis_thaliana   OG0000025
AT1G75220.1 | Major facilitator superfamily protein |
Chr1:28229412-28232606 REVERSE LENGTH=487 | 201606
Arabidopsis_thaliana   OG0000025
AT1G07340.1 | sugar transporter 2 | Chr1:2254873-2256712 FORWARD
LENGTH=498 | 201606   Arabidopsis_thaliana   OG0000025
AT1G77210.1 | sugar transporter 14 | Chr1:29009036-29010980 REVERSE
LENGTH=504 | 201606   Arabidopsis_thaliana   OG0000025
AT1G79820.1 | Major facilitator superfamily protein |
Chr1:30022581-30026771 REVERSE LENGTH=495 | 201606

Arabidopsis_thaliana   OG0000025
AT2G16120.1 | polyol/monosaccharide transporter 1 | Chr2:6996727-6998441 REVERSE LENGTH=511 | 201606   Arabidopsis_thaliana   OG0000025
AT2G16130.1 | polyol/monosaccharide transporter 2 | Chr2:7002322-7004043 FORWARD LENGTH=511 | 201606   Arabidopsis_thaliana   OG0000025
AT2G18480.1 | Major facilitator superfamily protein | Chr2:8009582-8011243 REVERSE LENGTH=508 | 201606   Arabidopsis_thaliana   OG0000025
AT1G08890.1 | Major facilitator superfamily protein | Chr1:2848374-2852016 FORWARD LENGTH=464 | 201606   Arabidopsis_thaliana   OG0000025
AT1G08900.1 | Major facilitator superfamily protein | Chr1:2852478-2855610 FORWARD LENGTH=462 | 201606   Arabidopsis_thaliana   OG0000025
AT1G08920.1 | ERD (early response to dehydration) six-like 1 | Chr1:2867446-2870360 FORWARD LENGTH=470 | 201606   Arabidopsis_thaliana   OG0000025
AT1G08930.1 | Major facilitator superfamily protein | Chr1:2873604-2876979 FORWARD LENGTH=496 | 201606   Arabidopsis_thaliana   OG0000025
AT2G20780.1 | Major facilitator superfamily protein | Chr2:8947496-8949170 REVERSE LENGTH=526 | 201606   Arabidopsis_thaliana   OG0000025
Os03t0363600-01 Similar to Sugar transporter-like protein.   Oryza_sativa   OG0000025
Os03t0594400-01 Monosaccharide transporter 2.   Oryza_sativa   OG0000025
Os03t0823100-01 Major facilitator superfamily protein.   Oryza_sativa   OG0000025
Os03t0823200-01 Similar to predicted protein.   Oryza_sativa   OG0000025
Os04t0452600-00 Similar to H0505F09.2 protein.   Oryza_sativa   OG0000025
Os04t0452700-01 Similar to Monosaccharide transporter 1.   Oryza_sativa   OG0000025
Os04t0453200-01 Similar to Monosaccharide transporter 1.   Oryza_sativa   OG0000025
Os04t0453266-00 Similar to H0505F09.5 protein.   Oryza_sativa   OG0000025
Os04t0453300-00 Similar to H0505F09.5 protein.   Oryza_sativa   OG0000025
Os04t0454200-01 Similar to Monosaccharide transporter 1.   Oryza_sativa   OG0000025
Os04t0491700-01 Sugar/inositol transporter domain containing protein. Oryza_sativa   OG0000025
Os04t0511400-01 Sugar/inositol transporter domain containing protein. Oryza_sativa   OG0000025
Os04t0529800-01 Sugar/inositol transporter domain containing protein. Oryza_sativa   OG0000025
Os04t0679000-00 Similar to H0801D08.9 protein.   Oryza_sativa   OG0000025
Os04t0679050-00 Similar to H0801D08.10 protein.   Oryza_sativa

OG0000025
Os05t0169700-00 Similar to Sugar transporter family protein, expressed.	Oryza_sativa	OG0000025
Os05t0396000-00 Similar to Hexose carrier.	Oryza_sativa	OG0000025
Os05t0579000-01 Similar to Integral membrane protein.	Oryza_sativa	OG0000025
Os06t0141100-00 Monosaccharide transporter.	Oryza_sativa	OG0000025
Os01t0567500-01 Similar to Monosaccharide transporter 3.	Oryza_sativa	OG0000025
Os01t0567600-00 Similar to Monosaccharide transporter 3.	Oryza_sativa	OG0000025
Os07t0106200-02 Similar to Hexose transporter.	Oryza_sativa	OG0000025
Os07t0131250-00 Similar to Hexose transporter HT2.	Oryza_sativa	OG0000025
Os07t0131600-01 Similar to Monosaccharide transporter.	Oryza_sativa	OG0000025
Os07t0151200-00 Sugar/inositol transporter domain containing protein.	Oryza_sativa	OG0000025
Os07t0206600-01 Similar to Hexose transporter.	Oryza_sativa	OG0000025
Os07t0559700-00 Similar to Monosaccharide transporter 3.	Oryza_sativa	OG0000025
Os07t0582400-01 Similar to Proton myo-inositol cotransporter.	Oryza_sativa	OG0000025
Os07t0582500-01 Similar to Proton myo-inositol cotransporter.	Oryza_sativa	OG0000025
Os07t0582850-00 General substrate transporter family protein.	Oryza_sativa	OG0000025
Os08t0178200-02 Similar to Monosaccharide transporter 3.	Oryza_sativa	OG0000025
Os01t0133400-01 Similar to Hexose transporter (Fragment).	Oryza_sativa	OG0000025
Os09t0268300-01 Similar to Hexose carrier.	Oryza_sativa	OG0000025
Os09t0297300-00 Similar to sugar carrier protein C.	Oryza_sativa	OG0000025
Os09t0322000-01 Similar to PaMst-1.	Oryza_sativa	OG0000025
Os09t0394500-01 Similar to predicted protein.	Oryza_sativa	OG0000025
Os09t0416200-01 Similar to Glucose transporter (Fragment).	Oryza_sativa	OG0000025
Os09t0452300-01 Similar to cDNA, clone	Oryza_sativa	OG0000025
Os10t0360100-01 Similar to Sugar transporter protein.	Oryza_sativa	OG0000025
Os10t0561300-01 Similar to Monosaccharid transporter.	Oryza_sativa	OG0000025
Os10t0579200-01 Sugar/inositol transporter domain containing protein.	Oryza_sativa	OG0000025
Os11t0594000-01 Sugar/inositol transporter domain containing

protein. Oryza_sativa OG0000025
Os11t0637000-01 Similar to Sugar transporter family protein.
Oryza_sativa OG0000025
Os11t0637100-00 Similar to Sugar transporter family protein.
Oryza_sativa OG0000025
Os11t0637200-01 Similar to Sorbitol transporter. Oryza_sativa
OG0000025
Os11t0643800-01 Similar to Sugar transporter family protein,
expressed. Oryza_sativa OG0000025
Os12t0512100-01 Sugar/inositol transporter domain containing
protein. Oryza_sativa OG0000025
Os12t0514000-01 Similar to Sorbitol transporter. Oryza_sativa
OG0000025
Os01t0966900-00 Similar to Sorbitol transporter. Oryza_sativa
OG0000025
Os02t0160400-01 Similar to Monosaccharide transporter 3.
Oryza_sativa OG0000025
Os02t0274900-01 Major facilitator superfamily protein.
Oryza_sativa OG0000025
Os02t0573500-01 Similar to Monosaccharide transporter 1.
Oryza_sativa OG0000025
Os02t0574000-01 Similar to Monosaccharide transporter 1.
Oryza_sativa OG0000025
Os02t0574100-01 Sugar/inositol transporter domain containing
protein. Oryza_sativa OG0000025
Os03t0101300-01 Similar to Hexose transporter. Oryza_sativa
OG0000025
Os03t0197100-01 Similar to Sugar transporter protein.
Oryza_sativa OG0000025
Os03t0197200-01 Similar to Sorbitol transporter. Oryza_sativa
OG0000025
Os03t0218400-01 Similar to Hexose transporter. Oryza_sativa
OG0000025
CMK066CT pep chromosome:ASM9120v1:11:195286:196912:-1 gene:CMK066C
transcript:CMK066CT gene_biotype:protein_coding
transcript_biotype:protein_coding Cyanidioschyzon_merolae
OG0000025
evm.model.contig_2279.4 Porphyridium_purpureum OG0000025
evm.model.contig_3385.17 Porphyridium_purpureum OG0000025
evm.model.contig_493.15 Porphyridium_purpureum OG0000025
CDF41107 pep supercontig:ASM35022v2:HG002309:254597:256246:-1
gene:CHC_T00007330001 transcript:CDF41107
gene_biotype:protein_coding transcript_biotype:protein_coding
Chondrus_crispus OG0000025
g8342.t1 Caulerpa_lentillifera OG0000026
Cre02.g079750.t1.1 pacid=30784947 transcript=Cre02.g079750.t1.1
locus=Cre02.g079750 ID=Cre02.g079750.t1.1.v5.5 annot-version=v5.5
Chlamydomonas_reinhardtii OG0000026
Cre02.g074150.t1.1 pacid=30785488 transcript=Cre02.g074150.t1.1
locus=Cre02.g074150 ID=Cre02.g074150.t1.1.v5.5 annot-version=v5.5
Chlamydomonas_reinhardtii OG0000026
Cre07.g329900.t1.1 pacid=30775034 transcript=Cre07.g329900.t1.1
locus=Cre07.g329900 ID=Cre07.g329900.t1.1.v5.5 annot-version=v5.5
Chlamydomonas_reinhardtii OG0000026

Cre11.g467678.t1.1 pacid=30775629 transcript=Cre11.g467678.t1.1
locus=Cre11.g467678 ID=Cre11.g467678.t1.1.v5.5 annot-version=v5.5
Chlamydomonas_reinhardtii OG0000026
Cre16.g678900.t1.2 pacid=30778058 transcript=Cre16.g678900.t1.2
locus=Cre16.g678900 ID=Cre16.g678900.t1.2.v5.5 annot-version=v5.5
Chlamydomonas_reinhardtii OG0000026
Cre06.g251250.t1.1 pacid=30780027 transcript=Cre06.g251250.t1.1
locus=Cre06.g251250 ID=Cre06.g251250.t1.1.v5.5 annot-version=v5.5
Chlamydomonas_reinhardtii OG0000026
Cre09.g412600.t1.1 pacid=30780949 transcript=Cre09.g412600.t1.1
locus=Cre09.g412600 ID=Cre09.g412600.t1.1.v5.5 annot-version=v5.5
Chlamydomonas_reinhardtii OG0000026
Cre17.g733150.t1.1 pacid=30781867 transcript=Cre17.g733150.t1.1
locus=Cre17.g733150 ID=Cre17.g733150.t1.1.v5.5 annot-version=v5.5
Chlamydomonas_reinhardtii OG0000026
Cre13.g571200.t1.1 pacid=30784146 transcript=Cre13.g571200.t1.1
locus=Cre13.g571200 ID=Cre13.g571200.t1.1.v5.5 annot-version=v5.5
Chlamydomonas_reinhardtii OG0000026
Vocar.0044s0018.1.p pacid=32896640 transcript=Vocar.0044s0018.1
locus=Vocar.0044s0018 ID=Vocar.0044s0018.1.v2.1 annot-version=v2.1
Volvox_carteri OG0000026
Vocar.0069s0008.1.p pacid=32896762 transcript=Vocar.0069s0008.1
locus=Vocar.0069s0008 ID=Vocar.0069s0008.1.v2.1 annot-version=v2.1
Volvox_carteri OG0000026
Vocar.0018s0081.1.p pacid=32898547 transcript=Vocar.0018s0081.1
locus=Vocar.0018s0081 ID=Vocar.0018s0081.1.v2.1 annot-version=v2.1
Volvox_carteri OG0000026
Vocar.0003s0423.1.p pacid=32889392 transcript=Vocar.0003s0423.1
locus=Vocar.0003s0423 ID=Vocar.0003s0423.1.v2.1 annot-version=v2.1
Volvox_carteri OG0000026
Vocar.0003s0424.1.p pacid=32889399 transcript=Vocar.0003s0424.1
locus=Vocar.0003s0424 ID=Vocar.0003s0424.1.v2.1 annot-version=v2.1
Volvox_carteri OG0000026
Vocar.0046s0013.1.p pacid=32892989 transcript=Vocar.0046s0013.1
locus=Vocar.0046s0013 ID=Vocar.0046s0013.1.v2.1 annot-version=v2.1
Volvox_carteri OG0000026
Vocar.0009s0166.1.p pacid=32893134 transcript=Vocar.0009s0166.1
locus=Vocar.0009s0166 ID=Vocar.0009s0166.1.v2.1 annot-version=v2.1
Volvox_carteri OG0000026
Vocar.0001s0318.1.p pacid=32884175 transcript=Vocar.0001s0318.1
locus=Vocar.0001s0318 ID=Vocar.0001s0318.1.v2.1 annot-version=v2.1
Volvox_carteri OG0000026
Vocar.0009s0380.1.p pacid=32893358 transcript=Vocar.0009s0380.1
locus=Vocar.0009s0380 ID=Vocar.0009s0380.1.v2.1 annot-version=v2.1
Volvox_carteri OG0000026
jgi|ChlNC64A_1|132895|IGS.gm_1_00031 Chlorella_variabilis
OG0000026
jgi|ChlNC64A_1|56764|estExt_fgenesh3_pg.C_20255
Chlorella_variabilis OG0000026
jgi|ChlNC64A_1|145218|IGS.gm_9_00025 Chlorella_variabilis
OG0000026
jgi|ChlNC64A_1|144976|IGS.gm_8_00304 Chlorella_variabilis
OG0000026
jgi|ChlNC64A_1|138871|IGS.gm_22_00112 Chlorella_variabilis

OG0000026
jgi|ChlNC64A_1|144050|IGS.gm_69_00001 Chlorella_variabilis
OG0000026
46955 pacid=27387100 transcript=46955
locus=estExt_fgenesh1_pg.C_50301 ID=46955.2.0.227 annot-version=v2.0
Coccomyxa_subellipsoidea OG0000026
11065 pacid=27388957 transcript=11065 locus=e_gw1.1.783.1
ID=11065.2.0.227 annot-version=v2.0 Coccomyxa_subellipsoidea
OG0000026
64878 pacid=27390474 transcript=64878 locus=estExt_Genemark1.C_30234
ID=64878.2.0.227 annot-version=v2.0 Coccomyxa_subellipsoidea
OG0000026
7070 pacid=27391898 transcript=7070 locus=gw1.11.227.1
ID=7070.2.0.227 annot-version=v2.0 Coccomyxa_subellipsoidea
OG0000026
3937_gw1.09.00.217.1 Ostreococcus_tauri OG0000026
19170_e_gw1.09.00.470.1 Ostreococcus_tauri OG0000026
9190 pacid=27415789 transcript=9190 locus=gwEuk.16.303.1
ID=9190.2.0.231 annot-version=v2.0 Ostreococcus_lucimarinus
OG0000026
9098 pacid=27416928 transcript=9098 locus=gwEuk.12.387.1
ID=9098.2.0.231 annot-version=v2.0 Ostreococcus_lucimarinus
OG0000026
1394 pacid=27413593 transcript=1394 locus=gwEuk.9.88.1
ID=1394.2.0.231 annot-version=v2.0 Ostreococcus_lucimarinus
OG0000026
181486 pacid=27339791 transcript=181526
locus=estExt_Genewise1Plus.C_13_t10132 ID=181526.3.0.228 annot-
version=v3.0 Micromonas_pusilla OG0000026
9052 pacid=27340087 transcript=9052 locus=MicpuC2.gw1.13.339.1
ID=9052.3.0.228 annot-version=v3.0 Micromonas_pusilla
OG0000026
60651 pacid=27344194 transcript=60651 locus=MicpuC2.EuGene.
0000090202 ID=60651.3.0.228 annot-version=v3.0
Micromonas_pusilla OG0000026
127373 pacid=27344277 transcript=127413
locus=estExt_fgenesh2_kg.C_90035 ID=127413.3.0.228 annot-
version=v3.0 Micromonas_pusilla OG0000026
10373 pacid=27344733 transcript=10373 locus=MicpuC2.gw1.17.214.1
ID=10373.3.0.228 annot-version=v3.0 Micromonas_pusilla
OG0000026
122977 pacid=27347044 transcript=123017 locus=fgenesh2_kg.
3_#_108_#_4277793:1 ID=123017.3.0.228 annot-version=v3.0
Micromonas_pusilla OG0000026
11312 pacid=27347277 transcript=11312 locus=MicpuC2.gw1.3.1123.1
ID=11312.3.0.228 annot-version=v3.0 Micromonas_pusilla
OG0000026
151502 pacid=27347278 transcript=151542 locus=e_gw1.3.1632.1
ID=151542.3.0.228 annot-version=v3.0 Micromonas_pusilla
OG0000026
179026 pacid=27348250 transcript=179066
locus=estExt_Genewise1Plus.C_6_t20336 ID=179066.3.0.228 annot-
version=v3.0 Micromonas_pusilla OG0000026
85490 pacid=27397287 transcript=85490 locus=e_gw2.10.716.1

ID=85490.3.0.229 annot-version=v3.0	Micromonas_sp._RCC299	OG0000026
71636 pacid=27397338 transcript=71636 locus=gw2.10.380.1 ID=71636.3.0.229 annot-version=v3.0	Micromonas_sp._RCC299	OG0000026
102756 pacid=27397790 transcript=102756 locus=fgenesh2_pg.C_Chr_10000242 ID=102756.3.0.229 annot-version=v3.0	Micromonas_sp._RCC299	OG0000026
74145 pacid=27401832 transcript=74145 locus=gw2.06.663.1 ID=74145.3.0.229 annot-version=v3.0	Micromonas_sp._RCC299	OG0000026
84309 pacid=27403441 transcript=84309 locus=e_gw2.08.134.1 ID=84309.3.0.229 annot-version=v3.0	Micromonas_sp._RCC299	OG0000026
77031 pacid=27404894 transcript=77031 locus=gw2.11.687.1 ID=77031.3.0.229 annot-version=v3.0	Micromonas_sp._RCC299	OG0000026
kfl00349_0070_v1.1	Klebsormidium_flaccidum	OG0000026
kfl00015_0240_v1.1	Klebsormidium_flaccidum	OG0000026
kfl00385_0110_v1.1	Klebsormidium_flaccidum	OG0000026
kfl00389_0010_v1.1	Klebsormidium_flaccidum	OG0000026
kfl00408_0060_v1.1	Klebsormidium_flaccidum	OG0000026
kfl00421_0010_v1.1	Klebsormidium_flaccidum	OG0000026
kfl00421_0020_v1.1	Klebsormidium_flaccidum	OG0000026
kfl00427_0060_v1.1	Klebsormidium_flaccidum	OG0000026
kfl00442_0030_v1.1	Klebsormidium_flaccidum	OG0000026
kfl00494_0060_v1.1	Klebsormidium_flaccidum	OG0000026
kfl00514_0020_v1.1	Klebsormidium_flaccidum	OG0000026
kfl00524_0010_v1.1	Klebsormidium_flaccidum	OG0000026
kfl00524_0070_v1.1	Klebsormidium_flaccidum	OG0000026
kfl00542_0050_v1.1	Klebsormidium_flaccidum	OG0000026
kfl00552_0060_v1.1	Klebsormidium_flaccidum	OG0000026
kfl00553_0010_v1.1	Klebsormidium_flaccidum	OG0000026
kfl00564_0090_v1.1	Klebsormidium_flaccidum	OG0000026
kfl00585_0020_v1.1	Klebsormidium_flaccidum	OG0000026
kfl00587_0010_v1.1	Klebsormidium_flaccidum	OG0000026
kfl00620_0030_v1.1	Klebsormidium_flaccidum	OG0000026
kfl00624_0010_v1.1	Klebsormidium_flaccidum	OG0000026
kfl00628_0050_v1.1	Klebsormidium_flaccidum	OG0000026
kfl00652_0100_v1.1	Klebsormidium_flaccidum	OG0000026
kfl00665_0050_v1.1	Klebsormidium_flaccidum	OG0000026
kfl00700_0010_v1.1	Klebsormidium_flaccidum	OG0000026
kfl00708_0130_v1.1	Klebsormidium_flaccidum	OG0000026
kfl00748_0010_v1.1	Klebsormidium_flaccidum	OG0000026
kfl00748_0020_v1.1	Klebsormidium_flaccidum	OG0000026
kfl00781_0020_v1.1	Klebsormidium_flaccidum	OG0000026
kfl00021_0480_v1.1	Klebsormidium_flaccidum	OG0000026
kfl00815_0010_v1.1	Klebsormidium_flaccidum	OG0000026
kfl00867_0010_v1.1	Klebsormidium_flaccidum	OG0000026
kfl00867_0020_v1.1	Klebsormidium_flaccidum	OG0000026
kfl00867_0030_v1.1	Klebsormidium_flaccidum	OG0000026
kfl00875_0010_v1.1	Klebsormidium_flaccidum	OG0000026
kfl00880_0020_v1.1	Klebsormidium_flaccidum	OG0000026
kfl00920_0060_v1.1	Klebsormidium_flaccidum	OG0000026

kfl00933_0010_v1.1	Klebsormidium_flaccidum	OG0000026
kfl00954_0010_v1.1	Klebsormidium_flaccidum	OG0000026
kfl01073_0010_v1.1	Klebsormidium_flaccidum	OG0000026
kfl01115_0030_v1.1	Klebsormidium_flaccidum	OG0000026
kfl01121_0020_v1.1	Klebsormidium_flaccidum	OG0000026
kfl00002_0770_v1.1	Klebsormidium_flaccidum	OG0000026
kfl00003_0010_v1.1	Klebsormidium_flaccidum	OG0000026
kfl00026_0290_v1.1	Klebsormidium_flaccidum	OG0000026
kfl00029_0130_v1.1	Klebsormidium_flaccidum	OG0000026
kfl00035_0070_v1.1	Klebsormidium_flaccidum	OG0000026
kfl00038_0190_v1.1	Klebsormidium_flaccidum	OG0000026
kfl00040_0110_v1.1	Klebsormidium_flaccidum	OG0000026
kfl00041_0230_v1.1	Klebsormidium_flaccidum	OG0000026
kfl00058_0090_v1.1	Klebsormidium_flaccidum	OG0000026
kfl00081_0030_v1.1	Klebsormidium_flaccidum	OG0000026
kfl00086_0010_v1.1	Klebsormidium_flaccidum	OG0000026
kfl00088_0140_v1.1	Klebsormidium_flaccidum	OG0000026
kfl00095_0250_v1.1	Klebsormidium_flaccidum	OG0000026
kfl00096_0260_v1.1	Klebsormidium_flaccidum	OG0000026
kfl00103_0020_v1.1	Klebsormidium_flaccidum	OG0000026
kfl00152_0160_v1.1	Klebsormidium_flaccidum	OG0000026
kfl00165_0130_v1.1	Klebsormidium_flaccidum	OG0000026
kfl00167_0100_v1.1	Klebsormidium_flaccidum	OG0000026
kfl00190_0110_v1.1	Klebsormidium_flaccidum	OG0000026
kfl00193_0190_v1.1	Klebsormidium_flaccidum	OG0000026
kfl00196_0020_v1.1	Klebsormidium_flaccidum	OG0000026
kfl00203_0210_v1.1	Klebsormidium_flaccidum	OG0000026
kfl00205_0150_v1.1	Klebsormidium_flaccidum	OG0000026
kfl00210_0160_v1.1	Klebsormidium_flaccidum	OG0000026
kfl00210_0165_v1.1	Klebsormidium_flaccidum	OG0000026
kfl00011_0090_v1.1	Klebsormidium_flaccidum	OG0000026
kfl00219_0010_v1.1	Klebsormidium_flaccidum	OG0000026
kfl00219_0200_v1.1	Klebsormidium_flaccidum	OG0000026
kfl00219_0290_v1.1	Klebsormidium_flaccidum	OG0000026
kfl00234_0040_v1.1	Klebsormidium_flaccidum	OG0000026
kfl00234_0100_v1.1	Klebsormidium_flaccidum	OG0000026
kfl00256_0080_v1.1	Klebsormidium_flaccidum	OG0000026
kfl00271_0210_v1.1	Klebsormidium_flaccidum	OG0000026
kfl00278_0080_v1.1	Klebsormidium_flaccidum	OG0000026
kfl00286_0050_v1.1	Klebsormidium_flaccidum	OG0000026
kfl00303_0040_v1.1	Klebsormidium_flaccidum	OG0000026
kfl00303_0060_v1.1	Klebsormidium_flaccidum	OG0000026
kfl00306_0030_v1.1	Klebsormidium_flaccidum	OG0000026
kfl00331_0060_v1.1	Klebsormidium_flaccidum	OG0000026
Pp3c15_21590V3.1.p pacid=32929159 transcript=Pp3c15_21590V3.1 locus=Pp3c15_21590 annot-version=v3.3	Physcomitrella_patens OG0000026
Pp3c15_24460V3.1.p pacid=32929747 transcript=Pp3c15_24460V3.1 locus=Pp3c15_24460 annot-version=v3.3	Physcomitrella_patens OG0000026
Pp3c13_1440V3.1.p pacid=32931818 transcript=Pp3c13_1440V3.1 locus=Pp3c13_1440 annot-version=v3.3	Physcomitrella_patens OG0000026
Pp3c2_36790V3.1.p pacid=32933056 transcript=Pp3c2_36790V3.1

locus=Pp3c2_36790 annot-version=v3.3 Physcomitrella_patens
OG0000026
Pp3c2_6520V3.1.p pacid=32933125 transcript=Pp3c2_6520V3.1
locus=Pp3c2_6520 annot-version=v3.3 Physcomitrella_patens
OG0000026
Pp3c2_20110V3.1.p pacid=32933819 transcript=Pp3c2_20110V3.1
locus=Pp3c2_20110 annot-version=v3.3 Physcomitrella_patens
OG0000026
Pp3c2_16310V3.1.p pacid=32934089 transcript=Pp3c2_16310V3.1
locus=Pp3c2_16310 annot-version=v3.3 Physcomitrella_patens
OG0000026
Pp3c3_1510V3.1.p pacid=32940632 transcript=Pp3c3_1510V3.1
locus=Pp3c3_1510 annot-version=v3.3 Physcomitrella_patens
OG0000026
Pp3c20_17580V3.1.p pacid=32948068 transcript=Pp3c20_17580V3.1
locus=Pp3c20_17580 annot-version=v3.3 Physcomitrella_patens
OG0000026
Pp3s32_693V3.1.p pacid=32948381 transcript=Pp3s32_693V3.1
locus=Pp3s32_693 annot-version=v3.3 Physcomitrella_patens
OG0000026
Pp3s32_260V3.1.p pacid=32948413 transcript=Pp3s32_260V3.1
locus=Pp3s32_260 annot-version=v3.3 Physcomitrella_patens
OG0000026
Pp3s30_340V3.1.p pacid=32948538 transcript=Pp3s30_340V3.1
locus=Pp3s30_340 annot-version=v3.3 Physcomitrella_patens
OG0000026
Pp3c27_2730V3.1.p pacid=32952403 transcript=Pp3c27_2730V3.1
locus=Pp3c27_2730 annot-version=v3.3 Physcomitrella_patens
OG0000026
Pp3c27_3830V3.1.p pacid=32952460 transcript=Pp3c27_3830V3.1
locus=Pp3c27_3830 annot-version=v3.3 Physcomitrella_patens
OG0000026
Pp3c5_9120V3.1.p pacid=32952704 transcript=Pp3c5_9120V3.1
locus=Pp3c5_9120 annot-version=v3.3 Physcomitrella_patens
OG0000026
Pp3c22_15240V3.1.p pacid=32904946 transcript=Pp3c22_15240V3.1
locus=Pp3c22_15240 annot-version=v3.3 Physcomitrella_patens
OG0000026
Pp3c5_3920V3.1.p pacid=32955457 transcript=Pp3c5_3920V3.1
locus=Pp3c5_3920 annot-version=v3.3 Physcomitrella_patens
OG0000026
Pp3c5_21420V3.1.p pacid=32955470 transcript=Pp3c5_21420V3.1
locus=Pp3c5_21420 annot-version=v3.3 Physcomitrella_patens
OG0000026
Pp3c5_3880V3.1.p pacid=32956131 transcript=Pp3c5_3880V3.1
locus=Pp3c5_3880 annot-version=v3.3 Physcomitrella_patens
OG0000026
Pp3c14_18760V3.1.p pacid=32962478 transcript=Pp3c14_18760V3.1
locus=Pp3c14_18760 annot-version=v3.3 Physcomitrella_patens
OG0000026
Pp3c1_29920V3.1.p pacid=32966887 transcript=Pp3c1_29920V3.1
locus=Pp3c1_29920 annot-version=v3.3 Physcomitrella_patens
OG0000026
Pp3c1_20050V3.1.p pacid=32968660 transcript=Pp3c1_20050V3.1

locus=Pp3c1_20050 annot-version=v3.3   Physcomitrella_patens
OG0000026
Pp3c1_9540V3.1.p pacid=32969891 transcript=Pp3c1_9540V3.1
locus=Pp3c1_9540 annot-version=v3.3   Physcomitrella_patens
OG0000026
Pp3c1_29330V3.1.p pacid=32971746 transcript=Pp3c1_29330V3.1
locus=Pp3c1_29330 annot-version=v3.3   Physcomitrella_patens
OG0000026
Pp3c6_7030V3.1.p pacid=32976734 transcript=Pp3c6_7030V3.1
locus=Pp3c6_7030 annot-version=v3.3   Physcomitrella_patens
OG0000026
Pp3c6_11730V3.1.p pacid=32977073 transcript=Pp3c6_11730V3.1
locus=Pp3c6_11730 annot-version=v3.3   Physcomitrella_patens
OG0000026
Pp3c6_20120V3.1.p pacid=32977643 transcript=Pp3c6_20120V3.1
locus=Pp3c6_20120 annot-version=v3.3   Physcomitrella_patens
OG0000026
Pp3c6_17340V3.1.p pacid=32978971 transcript=Pp3c6_17340V3.1
locus=Pp3c6_17340 annot-version=v3.3   Physcomitrella_patens
OG0000026
Pp3c25_8540V3.1.p pacid=32980351 transcript=Pp3c25_8540V3.1
locus=Pp3c25_8540 annot-version=v3.3   Physcomitrella_patens
OG0000026
Pp3c18_2110V3.1.p pacid=32982467 transcript=Pp3c18_2110V3.1
locus=Pp3c18_2110 annot-version=v3.3   Physcomitrella_patens
OG0000026
Pp3c18_4620V3.1.p pacid=32983205 transcript=Pp3c18_4620V3.1
locus=Pp3c18_4620 annot-version=v3.3   Physcomitrella_patens
OG0000026
Pp3c18_4650V3.1.p pacid=32983388 transcript=Pp3c18_4650V3.1
locus=Pp3c18_4650 annot-version=v3.3   Physcomitrella_patens
OG0000026
Pp3c16_300V3.1.p pacid=32984366 transcript=Pp3c16_300V3.1
locus=Pp3c16_300 annot-version=v3.3   Physcomitrella_patens
OG0000026
Pp3c16_13660V3.1.p pacid=32985793 transcript=Pp3c16_13660V3.1
locus=Pp3c16_13660 annot-version=v3.3   Physcomitrella_patens
OG0000026
Pp3c16_14810V3.1.p pacid=32986239 transcript=Pp3c16_14810V3.1
locus=Pp3c16_14810 annot-version=v3.3   Physcomitrella_patens
OG0000026
Pp3c16_7610V3.1.p pacid=32986441 transcript=Pp3c16_7610V3.1
locus=Pp3c16_7610 annot-version=v3.3   Physcomitrella_patens
OG0000026
Pp3c17_4020V3.1.p pacid=32908380 transcript=Pp3c17_4020V3.1
locus=Pp3c17_4020 annot-version=v3.3   Physcomitrella_patens
OG0000026
Pp3c24_10780V3.1.p pacid=32908954 transcript=Pp3c24_10780V3.1
locus=Pp3c24_10780 annot-version=v3.3   Physcomitrella_patens
OG0000026
Pp3c10_3860V3.1.p pacid=32900401 transcript=Pp3c10_3860V3.1
locus=Pp3c10_3860 annot-version=v3.3   Physcomitrella_patens
OG0000026
Pp3c9_21840V3.1.p pacid=32911555 transcript=Pp3c9_21840V3.1

locus=Pp3c9_21840 annot-version=v3.3 Physcomitrella_patens
OG0000026
Pp3c9_4870V3.1.p pacid=32911748 transcript=Pp3c9_4870V3.1
locus=Pp3c9_4870 annot-version=v3.3 Physcomitrella_patens
OG0000026
Pp3c9_18950V3.1.p pacid=32913710 transcript=Pp3c9_18950V3.1
locus=Pp3c9_18950 annot-version=v3.3 Physcomitrella_patens
OG0000026
Pp3c21_21550V3.1.p pacid=32915319 transcript=Pp3c21_21550V3.1
locus=Pp3c21_21550 annot-version=v3.3 Physcomitrella_patens
OG0000026
Pp3c21_18040V3.1.p pacid=32915679 transcript=Pp3c21_18040V3.1
locus=Pp3c21_18040 annot-version=v3.3 Physcomitrella_patens
OG0000026
Pp3c4_27560V3.1.p pacid=32921299 transcript=Pp3c4_27560V3.1
locus=Pp3c4_27560 annot-version=v3.3 Physcomitrella_patens
OG0000026
Pp3c4_27580V3.1.p pacid=32921697 transcript=Pp3c4_27580V3.1
locus=Pp3c4_27580 annot-version=v3.3 Physcomitrella_patens
OG0000026
Pp3c4_1360V3.1.p pacid=32922279 transcript=Pp3c4_1360V3.1
locus=Pp3c4_1360 annot-version=v3.3 Physcomitrella_patens
OG0000026
Pp3c7_17730V3.1.p pacid=32923875 transcript=Pp3c7_17730V3.1
locus=Pp3c7_17730 annot-version=v3.3 Physcomitrella_patens
OG0000026
AT2G40940.1 | ethylene response sensor 1 | Chr2:17084635-17086819
REVERSE LENGTH=613 | 201606 Arabidopsis_thaliana
OG0000026
AT2G47430.1 | Signal transduction histidine kinase |
Chr2:19459167-19463122 REVERSE LENGTH=1122 | 201606
Arabidopsis_thaliana OG0000026
AT3G04280.1 | response regulator 22 | Chr3:1130138-1130689 REVERSE
LENGTH=142 | 201606 Arabidopsis_thaliana OG0000026
AT3G04580.1 | Signal transduction histidine kinase%2C hybrid-type%2C
ethylene sensor | Chr3:1235576-1237965 REVERSE LENGTH=766 | 201606
Arabidopsis_thaliana OG0000026
AT3G23150.1 | Signal transduction histidine kinase%2C hybrid-type%2C
ethylene sensor | Chr3:8255156-8257560 FORWARD LENGTH=773 | 201606
Arabidopsis_thaliana OG0000026
AT5G10680.1 | calmodulin-binding protein-like protein |
Chr5:3373584-3374309 FORWARD LENGTH=241 | 201606
Arabidopsis_thaliana OG0000026
AT5G10720.1 | histidine kinase 5 | Chr5:3386835-3390541 FORWARD
LENGTH=922 | 201606 Arabidopsis_thaliana OG0000026
AT5G26594.1 | response regulator 24 | Chr5:9269282-9270254 FORWARD
LENGTH=139 | 201606 Arabidopsis_thaliana OG0000026
AT5G35750.1 | histidine kinase 2 | Chr5:13911743-13916337 REVERSE
LENGTH=1176 | 201606 Arabidopsis_thaliana OG0000026
AT1G27320.1 | histidine kinase 3 | Chr1:9487780-9492027 FORWARD
LENGTH=1036 | 201606 Arabidopsis_thaliana OG0000026
AT1G04310.1 | ethylene response sensor 2 | Chr1:1155116-1157125
REVERSE LENGTH=645 | 201606 Arabidopsis_thaliana
OG0000026

AT1G66340.1 | Signal transduction histidine kinase%2C hybrid-type%2C ethylene sensor | Chr1:24734698-24737366 FORWARD LENGTH=738 | 201606
        Arabidopsis_thaliana   OG0000026
AT2G01830.1 | CHASE domain containing histidine kinase protein | Chr2:363332-367429 REVERSE LENGTH=1057 | 201606
Arabidopsis_thaliana   OG0000026
AT2G17820.1 | histidine kinase 1 | Chr2:7743133-7748013 REVERSE LENGTH=1207 | 201606   Arabidopsis_thaliana   OG0000026
Os03t0701700-01 Similar to Ethylene receptor.   Oryza_sativa
OG0000026
Os03t0717700-01 Histidine kinase, Cytokinin signaling
Oryza_sativa   OG0000026
Os03t0752100-01 Similar to Phytochrome C. Oryza_sativa
OG0000026
Os04t0169100-01 Similar to Ethylene receptor-like protein 1.
Oryza_sativa   OG0000026
Os04t0212450-00 Hypothetical conserved gene.   Oryza_sativa
OG0000026
Os05t0155200-01 Similar to Ethylene receptor.   Oryza_sativa
OG0000026
Os06t0183200-01 Histidine kinase, Cytokinin signaling
Oryza_sativa   OG0000026
Os06t0654300-00 Similar to Histidine kinase (Fragment).
Oryza_sativa   OG0000026
Os07t0259100-01 Ethylene receptor, Ethylene response
Oryza_sativa   OG0000026
Os10t0362300-01 Similar to Histidine kinase 3.   Oryza_sativa
OG0000026
Os12t0454800-01 Receptor-like serine/threonine kinase, Cytokinin signaling   Oryza_sativa   OG0000026
Os02t0738400-01 Similar to Histidine kinase.   Oryza_sativa
OG0000026
Os02t0820900-01 Similar to Ethylene receptor-like protein 2.
Oryza_sativa   OG0000026
jgi|ChlNC64A_1|135693|IGS.gm_14_00226   Chlorella_variabilis
OG0000027
37592_1700010268 Ostreococcus_tauri   OG0000027
28573 pacid=27419117 transcript=28573 locus=eugene.1900010059 ID=28573.2.0.231 annot-version=v2.0   Ostreococcus_lucimarinus
OG0000027
43033 pacid=27348696 transcript=43033 locus=MicpuC2.fgenesh1_pg.C_scaffold_15000159 ID=43033.3.0.228 annot-version=v3.0   Micromonas_pusilla   OG0000027
53643 pacid=27348697 transcript=53643 locus=MicpuC2.EuGene.0000150318 ID=53643.3.0.228 annot-version=v3.0
Micromonas_pusilla   OG0000027
53357 pacid=27348715 transcript=53357 locus=MicpuC2.EuGene.0000150032 ID=53357.3.0.228 annot-version=v3.0
Micromonas_pusilla   OG0000027
43009 pacid=27348729 transcript=43009 locus=MicpuC2.fgenesh1_pg.C_scaffold_15000135 ID=43009.3.0.228 annot-version=v3.0   Micromonas_pusilla   OG0000027
53342 pacid=27348749 transcript=53342 locus=MicpuC2.EuGene.0000150017 ID=53342.3.0.228 annot-version=v3.0

Micromonas_pusilla	OG0000027
43093 pacid=27348760 transcript=43093
locus=MicpuC2.fgenesh1_pg.C_scaffold_15000219 ID=43093.3.0.228
annot-version=v3.0	Micromonas_pusilla	OG0000027
52378 pacid=27348893 transcript=52378 locus=MicpuC2.EuGene.
0000120443 ID=52378.3.0.228 annot-version=v3.0
Micromonas_pusilla	OG0000027
42308 pacid=27348911 transcript=42308
locus=MicpuC2.fgenesh1_pg.C_scaffold_12000257 ID=42308.3.0.228
annot-version=v3.0	Micromonas_pusilla	OG0000027
52398 pacid=27348920 transcript=52398 locus=MicpuC2.EuGene.
0000120463 ID=52398.3.0.228 annot-version=v3.0
Micromonas_pusilla	OG0000027
52226 pacid=27348923 transcript=52226 locus=MicpuC2.EuGene.
0000120291 ID=52226.3.0.228 annot-version=v3.0
Micromonas_pusilla	OG0000027
52194 pacid=27348932 transcript=52194 locus=MicpuC2.EuGene.
0000120259 ID=52194.3.0.228 annot-version=v3.0
Micromonas_pusilla	OG0000027
42307 pacid=27348945 transcript=42307
locus=MicpuC2.fgenesh1_pg.C_scaffold_12000256 ID=42307.3.0.228
annot-version=v3.0	Micromonas_pusilla	OG0000027
52358 pacid=27348995 transcript=52358 locus=MicpuC2.EuGene.
0000120423 ID=52358.3.0.228 annot-version=v3.0
Micromonas_pusilla	OG0000027
52370 pacid=27349005 transcript=52370 locus=MicpuC2.EuGene.
0000120435 ID=52370.3.0.228 annot-version=v3.0
Micromonas_pusilla	OG0000027
42328 pacid=27349117 transcript=42328
locus=MicpuC2.fgenesh1_pg.C_scaffold_12000277 ID=42328.3.0.228
annot-version=v3.0	Micromonas_pusilla	OG0000027
68604 pacid=27349137 transcript=68604 locus=MicpuC2.est_orfs.
12_7587_4271813:2 ID=68604.3.0.228 annot-version=v3.0
Micromonas_pusilla	OG0000027
52127 pacid=27349194 transcript=52127 locus=MicpuC2.EuGene.
0000120192 ID=52127.3.0.228 annot-version=v3.0
Micromonas_pusilla	OG0000027
52109 pacid=27349196 transcript=52109 locus=MicpuC2.EuGene.
0000120174 ID=52109.3.0.228 annot-version=v3.0
Micromonas_pusilla	OG0000027
68452 pacid=27349249 transcript=68452 locus=MicpuC2.est_orfs.
12_7435_4277382:1 ID=68452.3.0.228 annot-version=v3.0
Micromonas_pusilla	OG0000027
52612 pacid=27339855 transcript=52612 locus=MicpuC2.EuGene.
0000130169 ID=52612.3.0.228 annot-version=v3.0
Micromonas_pusilla	OG0000027
52765 pacid=27339890 transcript=52765 locus=MicpuC2.EuGene.
0000130322 ID=52765.3.0.228 annot-version=v3.0
Micromonas_pusilla	OG0000027
48724 pacid=27339935 transcript=48724
locus=MicpuC2.estExt_fgenesh1_pg.C_130188 ID=48724.3.0.228 annot-
version=v3.0	Micromonas_pusilla	OG0000027
52651 pacid=27339962 transcript=52651 locus=MicpuC2.EuGene.
0000130208 ID=52651.3.0.228 annot-version=v3.0

Micromonas_pusilla   OG0000027
52541 pacid=27340012 transcript=52541 locus=MicpuC2.EuGene.
0000130098 ID=52541.3.0.228 annot-version=v3.0
Micromonas_pusilla   OG0000027
52465 pacid=27340086 transcript=52465 locus=MicpuC2.EuGene.
0000130022 ID=52465.3.0.228 annot-version=v3.0
Micromonas_pusilla   OG0000027
52542 pacid=27340091 transcript=52542 locus=MicpuC2.EuGene.
0000130099 ID=52542.3.0.228 annot-version=v3.0
Micromonas_pusilla   OG0000027
52859 pacid=27340118 transcript=52859 locus=MicpuC2.EuGene.
0000130416 ID=52859.3.0.228 annot-version=v3.0
Micromonas_pusilla   OG0000027
51082 pacid=27340135 transcript=51082 locus=MicpuC2.EuGene.
0000100153 ID=51082.3.0.228 annot-version=v3.0
Micromonas_pusilla   OG0000027
60267 pacid=27338793 transcript=60267 locus=MicpuC2.EuGene.
0000080461 ID=60267.3.0.228 annot-version=v3.0
Micromonas_pusilla   OG0000027
51397 pacid=27340173 transcript=51397 locus=MicpuC2.EuGene.
0000100468 ID=51397.3.0.228 annot-version=v3.0
Micromonas_pusilla   OG0000027
51373 pacid=27340221 transcript=51373 locus=MicpuC2.EuGene.
0000100444 ID=51373.3.0.228 annot-version=v3.0
Micromonas_pusilla   OG0000027
51361 pacid=27340251 transcript=51361 locus=MicpuC2.EuGene.
0000100432 ID=51361.3.0.228 annot-version=v3.0
Micromonas_pusilla   OG0000027
51392 pacid=27340294 transcript=51392 locus=MicpuC2.EuGene.
0000100463 ID=51392.3.0.228 annot-version=v3.0
Micromonas_pusilla   OG0000027
60136 pacid=27338811 transcript=60136 locus=MicpuC2.EuGene.
0000080330 ID=60136.3.0.228 annot-version=v3.0
Micromonas_pusilla   OG0000027
62989 pacid=27340424 transcript=62989 locus=MicpuC2.est_orfs.
10_1972_4272836:1 ID=62989.3.0.228 annot-version=v3.0
Micromonas_pusilla   OG0000027
41515 pacid=27340536 transcript=41515
locus=MicpuC2.fgenesh1_pg.C_scaffold_10000028 ID=41515.3.0.228
annot-version=v3.0   Micromonas_pusilla   OG0000027
51476 pacid=27340603 transcript=51476 locus=MicpuC2.EuGene.
0000100547 ID=51476.3.0.228 annot-version=v3.0
Micromonas_pusilla   OG0000027
59417 pacid=27340671 transcript=59417 locus=MicpuC2.EuGene.
0000070266 ID=59417.3.0.228 annot-version=v3.0
Micromonas_pusilla   OG0000027
125344 pacid=27340704 transcript=125384
locus=estExt_fgenesh3_kg.C_70017 ID=125384.3.0.228 annot-
version=v3.0   Micromonas_pusilla   OG0000027
59499 pacid=27340739 transcript=59499 locus=MicpuC2.EuGene.
0000070348 ID=59499.3.0.228 annot-version=v3.0
Micromonas_pusilla   OG0000027
59494 pacid=27340795 transcript=59494 locus=MicpuC2.EuGene.
0000070343 ID=59494.3.0.228 annot-version=v3.0

Micromonas_pusilla   OG0000027
59379 pacid=27340836 transcript=59379 locus=MicpuC2.EuGene.
0000070228 ID=59379.3.0.228 annot-version=v3.0
Micromonas_pusilla   OG0000027
59773 pacid=27340906 transcript=59773 locus=MicpuC2.EuGene.
0000070622 ID=59773.3.0.228 annot-version=v3.0
Micromonas_pusilla   OG0000027
59416 pacid=27340910 transcript=59416 locus=MicpuC2.EuGene.
0000070265 ID=59416.3.0.228 annot-version=v3.0
Micromonas_pusilla   OG0000027
59205 pacid=27340933 transcript=59205 locus=MicpuC2.EuGene.
0000070054 ID=59205.3.0.228 annot-version=v3.0
Micromonas_pusilla   OG0000027
59637 pacid=27341054 transcript=59637 locus=MicpuC2.EuGene.
0000070486 ID=59637.3.0.228 annot-version=v3.0
Micromonas_pusilla   OG0000027
40634 pacid=27341150 transcript=40634
locus=MicpuC2.fgenesh1_pg.C_scaffold_7000228 ID=40634.3.0.228 annot-
version=v3.0   Micromonas_pusilla   OG0000027
68414 pacid=27341299 transcript=68414 locus=MicpuC2.est_orfs.
5_7397_4279916:1 ID=68414.3.0.228 annot-version=v3.0
Micromonas_pusilla   OG0000027
60395 pacid=27338910 transcript=60395 locus=MicpuC2.EuGene.
0000080589 ID=60395.3.0.228 annot-version=v3.0
Micromonas_pusilla   OG0000027
58304 pacid=27341359 transcript=58304 locus=MicpuC2.EuGene.
0000050609 ID=58304.3.0.228 annot-version=v3.0
Micromonas_pusilla   OG0000027
39941 pacid=27341389 transcript=39941
locus=MicpuC2.fgenesh1_pg.C_scaffold_5000370 ID=39941.3.0.228 annot-
version=v3.0   Micromonas_pusilla   OG0000027
60103 pacid=27338924 transcript=60103 locus=MicpuC2.EuGene.
0000080297 ID=60103.3.0.228 annot-version=v3.0
Micromonas_pusilla   OG0000027
57745 pacid=27341516 transcript=57745 locus=MicpuC2.EuGene.
0000050050 ID=57745.3.0.228 annot-version=v3.0
Micromonas_pusilla   OG0000027
57951 pacid=27341693 transcript=57951 locus=MicpuC2.EuGene.
0000050256 ID=57951.3.0.228 annot-version=v3.0
Micromonas_pusilla   OG0000027
60284 pacid=27338956 transcript=60284 locus=MicpuC2.EuGene.
0000080478 ID=60284.3.0.228 annot-version=v3.0
Micromonas_pusilla   OG0000027
58319 pacid=27341798 transcript=58319 locus=MicpuC2.EuGene.
0000050624 ID=58319.3.0.228 annot-version=v3.0
Micromonas_pusilla   OG0000027
58278 pacid=27341858 transcript=58278 locus=MicpuC2.EuGene.
0000050583 ID=58278.3.0.228 annot-version=v3.0
Micromonas_pusilla   OG0000027
60305 pacid=27338975 transcript=60305 locus=MicpuC2.EuGene.
0000080499 ID=60305.3.0.228 annot-version=v3.0
Micromonas_pusilla   OG0000027
57885 pacid=27341995 transcript=57885 locus=MicpuC2.EuGene.
0000050190 ID=57885.3.0.228 annot-version=v3.0

Micromonas_pusilla   OG0000027
53114 pacid=27342047 transcript=53114 locus=MicpuC2.EuGene.
0000140209 ID=53114.3.0.228 annot-version=v3.0
Micromonas_pusilla   OG0000027
190954 pacid=27342105 transcript=190994 locus=gm1.8075_g
ID=190994.3.0.228 annot-version=v3.0   Micromonas_pusilla
OG0000027
53031 pacid=27342124 transcript=53031 locus=MicpuC2.EuGene.
0000140126 ID=53031.3.0.228 annot-version=v3.0
Micromonas_pusilla   OG0000027
53305 pacid=27342176 transcript=53305 locus=MicpuC2.EuGene.
0000140400 ID=53305.3.0.228 annot-version=v3.0
Micromonas_pusilla   OG0000027
53045 pacid=27342193 transcript=53045 locus=MicpuC2.EuGene.
0000140140 ID=53045.3.0.228 annot-version=v3.0
Micromonas_pusilla   OG0000027
53213 pacid=27342196 transcript=53213 locus=MicpuC2.EuGene.
0000140308 ID=53213.3.0.228 annot-version=v3.0
Micromonas_pusilla   OG0000027
53002 pacid=27342236 transcript=53002 locus=MicpuC2.EuGene.
0000140097 ID=53002.3.0.228 annot-version=v3.0
Micromonas_pusilla   OG0000027
53272 pacid=27342243 transcript=53272 locus=MicpuC2.EuGene.
0000140367 ID=53272.3.0.228 annot-version=v3.0
Micromonas_pusilla   OG0000027
53221 pacid=27342258 transcript=53221 locus=MicpuC2.EuGene.
0000140316 ID=53221.3.0.228 annot-version=v3.0
Micromonas_pusilla   OG0000027
53091 pacid=27342267 transcript=53091 locus=MicpuC2.EuGene.
0000140186 ID=53091.3.0.228 annot-version=v3.0
Micromonas_pusilla   OG0000027
53003 pacid=27342325 transcript=53003 locus=MicpuC2.EuGene.
0000140098 ID=53003.3.0.228 annot-version=v3.0
Micromonas_pusilla   OG0000027
63696 pacid=27342369 transcript=63696 locus=MicpuC2.est_orfs.
14_2679_4273326:1 ID=63696.3.0.228 annot-version=v3.0
Micromonas_pusilla   OG0000027
63908 pacid=27342391 transcript=63908 locus=MicpuC2.est_orfs.
14_2891_4278002:2 ID=63908.3.0.228 annot-version=v3.0
Micromonas_pusilla   OG0000027
60425 pacid=27339018 transcript=60425 locus=MicpuC2.EuGene.
0000080619 ID=60425.3.0.228 annot-version=v3.0
Micromonas_pusilla   OG0000027
55140 pacid=27342527 transcript=55140 locus=MicpuC2.EuGene.
0000020169 ID=55140.3.0.228 annot-version=v3.0
Micromonas_pusilla   OG0000027
55049 pacid=27342543 transcript=55049 locus=MicpuC2.EuGene.
0000020078 ID=55049.3.0.228 annot-version=v3.0
Micromonas_pusilla   OG0000027
55058 pacid=27342714 transcript=55058 locus=MicpuC2.EuGene.
0000020087 ID=55058.3.0.228 annot-version=v3.0
Micromonas_pusilla   OG0000027
41060 pacid=27339074 transcript=41060
locus=MicpuC2.fgenesh1_pg.C_scaffold_8000302 ID=41060.3.0.228 annot-

version=v3.0   Micromonas_pusilla   OG0000027
55075 pacid=27342994 transcript=55075 locus=MicpuC2.EuGene.
0000020104 ID=55075.3.0.228 annot-version=v3.0
Micromonas_pusilla   OG0000027
55004 pacid=27343082 transcript=55004 locus=MicpuC2.EuGene.
0000020033 ID=55004.3.0.228 annot-version=v3.0
Micromonas_pusilla   OG0000027
55001 pacid=27343146 transcript=55001 locus=MicpuC2.EuGene.
0000020030 ID=55001.3.0.228 annot-version=v3.0
Micromonas_pusilla   OG0000027
55066 pacid=27343159 transcript=55066 locus=MicpuC2.EuGene.
0000020095 ID=55066.3.0.228 annot-version=v3.0
Micromonas_pusilla   OG0000027
59977 pacid=27339097 transcript=59977 locus=MicpuC2.EuGene.
0000080171 ID=59977.3.0.228 annot-version=v3.0
Micromonas_pusilla   OG0000027
60394 pacid=27339102 transcript=60394 locus=MicpuC2.EuGene.
0000080588 ID=60394.3.0.228 annot-version=v3.0
Micromonas_pusilla   OG0000027
53779 pacid=27343285 transcript=53779 locus=MicpuC2.EuGene.
0000160055 ID=53779.3.0.228 annot-version=v3.0
Micromonas_pusilla   OG0000027
60384 pacid=27339117 transcript=60384 locus=MicpuC2.EuGene.
0000080578 ID=60384.3.0.228 annot-version=v3.0
Micromonas_pusilla   OG0000027
43159 pacid=27343425 transcript=43159
locus=MicpuC2.fgenesh1_pg.C_scaffold_16000040 ID=43159.3.0.228
annot-version=v3.0   Micromonas_pusilla   OG0000027
54131 pacid=27343470 transcript=54131 locus=MicpuC2.EuGene.
0000160407 ID=54131.3.0.228 annot-version=v3.0
Micromonas_pusilla   OG0000027
54148 pacid=27343541 transcript=54148 locus=MicpuC2.EuGene.
0000160424 ID=54148.3.0.228 annot-version=v3.0
Micromonas_pusilla   OG0000027
54087 pacid=27343547 transcript=54087 locus=MicpuC2.EuGene.
0000160363 ID=54087.3.0.228 annot-version=v3.0
Micromonas_pusilla   OG0000027
53764 pacid=27343563 transcript=53764 locus=MicpuC2.EuGene.
0000160040 ID=53764.3.0.228 annot-version=v3.0
Micromonas_pusilla   OG0000027
53760 pacid=27343671 transcript=53760 locus=MicpuC2.EuGene.
0000160036 ID=53760.3.0.228 annot-version=v3.0
Micromonas_pusilla   OG0000027
54700 pacid=27343801 transcript=54700 locus=MicpuC2.EuGene.
0000180170 ID=54700.3.0.228 annot-version=v3.0
Micromonas_pusilla   OG0000027
67797 pacid=27343804 transcript=67797 locus=MicpuC2.est_orfs.
18_6780_4277293:2 ID=67797.3.0.228 annot-version=v3.0
Micromonas_pusilla   OG0000027
54642 pacid=27343808 transcript=54642 locus=MicpuC2.EuGene.
0000180112 ID=54642.3.0.228 annot-version=v3.0
Micromonas_pusilla   OG0000027
54638 pacid=27343868 transcript=54638 locus=MicpuC2.EuGene.
0000180108 ID=54638.3.0.228 annot-version=v3.0

Micromonas_pusilla	OG0000027
54664 pacid=27343921 transcript=54664 locus=MicpuC2.EuGene.
0000180134 ID=54664.3.0.228 annot-version=v3.0
Micromonas_pusilla	OG0000027
54802 pacid=27343923 transcript=54802 locus=MicpuC2.EuGene.
0000180272 ID=54802.3.0.228 annot-version=v3.0
Micromonas_pusilla	OG0000027
54796 pacid=27344038 transcript=54796 locus=MicpuC2.EuGene.
0000180266 ID=54796.3.0.228 annot-version=v3.0
Micromonas_pusilla	OG0000027
54672 pacid=27344043 transcript=54672 locus=MicpuC2.EuGene.
0000180142 ID=54672.3.0.228 annot-version=v3.0
Micromonas_pusilla	OG0000027
60069 pacid=27338696 transcript=60069 locus=MicpuC2.EuGene.
0000080263 ID=60069.3.0.228 annot-version=v3.0
Micromonas_pusilla	OG0000027
48156 pacid=27344092 transcript=48156
locus=MicpuC2.estExt_fgenesh1_pg.C_90293 ID=48156.3.0.228 annot-
version=v3.0	Micromonas_pusilla	OG0000027
60699 pacid=27344183 transcript=60699 locus=MicpuC2.EuGene.
0000090250 ID=60699.3.0.228 annot-version=v3.0
Micromonas_pusilla	OG0000027
63465 pacid=27344240 transcript=63465 locus=MicpuC2.est_orfs.
9_2448_4273744:1 ID=63465.3.0.228 annot-version=v3.0
Micromonas_pusilla	OG0000027
59832 pacid=27338699 transcript=59832 locus=MicpuC2.EuGene.
0000080026 ID=59832.3.0.228 annot-version=v3.0
Micromonas_pusilla	OG0000027
41429 pacid=27344413 transcript=41429
locus=MicpuC2.fgenesh1_pg.C_scaffold_9000300 ID=41429.3.0.228 annot-
version=v3.0	Micromonas_pusilla	OG0000027
40794 pacid=27339225 transcript=40794
locus=MicpuC2.fgenesh1_pg.C_scaffold_8000036 ID=40794.3.0.228 annot-
version=v3.0	Micromonas_pusilla	OG0000027
54361 pacid=27344615 transcript=54361 locus=MicpuC2.EuGene.
0000170207 ID=54361.3.0.228 annot-version=v3.0
Micromonas_pusilla	OG0000027
54512 pacid=27344618 transcript=54512 locus=MicpuC2.EuGene.
0000170358 ID=54512.3.0.228 annot-version=v3.0
Micromonas_pusilla	OG0000027
54263 pacid=27344623 transcript=54263 locus=MicpuC2.EuGene.
0000170109 ID=54263.3.0.228 annot-version=v3.0
Micromonas_pusilla	OG0000027
49254 pacid=27344629 transcript=49254
locus=MicpuC2.estExt_fgenesh1_pg.C_170164 ID=49254.3.0.228 annot-
version=v3.0	Micromonas_pusilla	OG0000027
54329 pacid=27344647 transcript=54329 locus=MicpuC2.EuGene.
0000170175 ID=54329.3.0.228 annot-version=v3.0
Micromonas_pusilla	OG0000027
54315 pacid=27344659 transcript=54315 locus=MicpuC2.EuGene.
0000170161 ID=54315.3.0.228 annot-version=v3.0
Micromonas_pusilla	OG0000027
54348 pacid=27344667 transcript=54348 locus=MicpuC2.EuGene.
0000170194 ID=54348.3.0.228 annot-version=v3.0

Micromonas_pusilla	OG0000027
54176 pacid=27344689 transcript=54176 locus=MicpuC2.EuGene.
0000170022 ID=54176.3.0.228 annot-version=v3.0
Micromonas_pusilla	OG0000027
43479 pacid=27344708 transcript=43479
locus=MicpuC2.fgenesh1_pg.C_scaffold_17000111 ID=43479.3.0.228
annot-version=v3.0	Micromonas_pusilla	OG0000027
43557 pacid=27344716 transcript=43557
locus=MicpuC2.fgenesh1_pg.C_scaffold_17000189 ID=43557.3.0.228
annot-version=v3.0	Micromonas_pusilla	OG0000027
54328 pacid=27344749 transcript=54328 locus=MicpuC2.EuGene.
0000170174 ID=54328.3.0.228 annot-version=v3.0
Micromonas_pusilla	OG0000027
54320 pacid=27344772 transcript=54320 locus=MicpuC2.EuGene.
0000170166 ID=54320.3.0.228 annot-version=v3.0
Micromonas_pusilla	OG0000027
54327 pacid=27344775 transcript=54327 locus=MicpuC2.EuGene.
0000170173 ID=54327.3.0.228 annot-version=v3.0
Micromonas_pusilla	OG0000027
54365 pacid=27344785 transcript=54365 locus=MicpuC2.EuGene.
0000170211 ID=54365.3.0.228 annot-version=v3.0
Micromonas_pusilla	OG0000027
54474 pacid=27344835 transcript=54474 locus=MicpuC2.EuGene.
0000170320 ID=54474.3.0.228 annot-version=v3.0
Micromonas_pusilla	OG0000027
54400 pacid=27344875 transcript=54400 locus=MicpuC2.EuGene.
0000170246 ID=54400.3.0.228 annot-version=v3.0
Micromonas_pusilla	OG0000027
54366 pacid=27344887 transcript=54366 locus=MicpuC2.EuGene.
0000170212 ID=54366.3.0.228 annot-version=v3.0
Micromonas_pusilla	OG0000027
54338 pacid=27344890 transcript=54338 locus=MicpuC2.EuGene.
0000170184 ID=54338.3.0.228 annot-version=v3.0
Micromonas_pusilla	OG0000027
66507 pacid=27344895 transcript=66507 locus=MicpuC2.est_orfs.
17_5490_4277881:2 ID=66507.3.0.228 annot-version=v3.0
Micromonas_pusilla	OG0000027
51912 pacid=27339268 transcript=51912 locus=MicpuC2.EuGene.
0000110434 ID=51912.3.0.228 annot-version=v3.0
Micromonas_pusilla	OG0000027
54401 pacid=27344918 transcript=54401 locus=MicpuC2.EuGene.
0000170247 ID=54401.3.0.228 annot-version=v3.0
Micromonas_pusilla	OG0000027
54240 pacid=27344930 transcript=54240 locus=MicpuC2.EuGene.
0000170086 ID=54240.3.0.228 annot-version=v3.0
Micromonas_pusilla	OG0000027
50778 pacid=27344948 transcript=50778 locus=MicpuC2.EuGene.
0000011002 ID=50778.3.0.228 annot-version=v3.0
Micromonas_pusilla	OG0000027
51610 pacid=27339272 transcript=51610 locus=MicpuC2.EuGene.
0000110132 ID=51610.3.0.228 annot-version=v3.0
Micromonas_pusilla	OG0000027
49984 pacid=27344960 transcript=49984 locus=MicpuC2.EuGene.
0000010208 ID=49984.3.0.228 annot-version=v3.0

Micromonas_pusilla   OG0000027
50433 pacid=27345091 transcript=50433 locus=MicpuC2.EuGene.
0000010657 ID=50433.3.0.228 annot-version=v3.0
Micromonas_pusilla   OG0000027
49886 pacid=27345201 transcript=49886 locus=MicpuC2.EuGene.
0000010110 ID=49886.3.0.228 annot-version=v3.0
Micromonas_pusilla   OG0000027
49981 pacid=27345419 transcript=49981 locus=MicpuC2.EuGene.
0000010205 ID=49981.3.0.228 annot-version=v3.0
Micromonas_pusilla   OG0000027
148485 pacid=27345620 transcript=148525 locus=e_gw1.1.1456.1
ID=148525.3.0.228 annot-version=v3.0   Micromonas_pusilla
OG0000027
51894 pacid=27339358 transcript=51894 locus=MicpuC2.EuGene.
0000110416 ID=51894.3.0.228 annot-version=v3.0
Micromonas_pusilla   OG0000027
50505 pacid=27345892 transcript=50505 locus=MicpuC2.EuGene.
0000010729 ID=50505.3.0.228 annot-version=v3.0
Micromonas_pusilla   OG0000027
123903 pacid=27339372 transcript=123943 locus=fgenesh2_kg.
11_#_14_#_4273039:2 ID=123943.3.0.228 annot-version=v3.0
Micromonas_pusilla   OG0000027
61041 pacid=27345997 transcript=61041 locus=MicpuC2.est_orfs.
1_24_4278202:1 ID=61041.3.0.228 annot-version=v3.0
Micromonas_pusilla   OG0000027
57673 pacid=27346047 transcript=57673 locus=MicpuC2.EuGene.
0000040799 ID=57673.3.0.228 annot-version=v3.0
Micromonas_pusilla   OG0000027
56974 pacid=27346056 transcript=56974 locus=MicpuC2.EuGene.
0000040100 ID=56974.3.0.228 annot-version=v3.0
Micromonas_pusilla   OG0000027
56977 pacid=27346172 transcript=56977 locus=MicpuC2.EuGene.
0000040103 ID=56977.3.0.228 annot-version=v3.0
Micromonas_pusilla   OG0000027
41855 pacid=27339405 transcript=41855
locus=MicpuC2.fgenesh1_pg.C_scaffold_11000068 ID=41855.3.0.228
annot-version=v3.0   Micromonas_pusilla   OG0000027
57012 pacid=27346445 transcript=57012 locus=MicpuC2.EuGene.
0000040138 ID=57012.3.0.228 annot-version=v3.0
Micromonas_pusilla   OG0000027
56900 pacid=27346458 transcript=56900 locus=MicpuC2.EuGene.
0000040026 ID=56900.3.0.228 annot-version=v3.0
Micromonas_pusilla   OG0000027
51567 pacid=27339430 transcript=51567 locus=MicpuC2.EuGene.
0000110089 ID=51567.3.0.228 annot-version=v3.0
Micromonas_pusilla   OG0000027
56958 pacid=27346688 transcript=56958 locus=MicpuC2.EuGene.
0000040084 ID=56958.3.0.228 annot-version=v3.0
Micromonas_pusilla   OG0000027
56931 pacid=27346817 transcript=56931 locus=MicpuC2.EuGene.
0000040057 ID=56931.3.0.228 annot-version=v3.0
Micromonas_pusilla   OG0000027
51529 pacid=27339460 transcript=51529 locus=MicpuC2.EuGene.
0000110051 ID=51529.3.0.228 annot-version=v3.0

Micromonas_pusilla	OG0000027
56685 pacid=27346848 transcript=56685 locus=MicpuC2.EuGene.
0000030839 ID=56685.3.0.228 annot-version=v3.0
Micromonas_pusilla	OG0000027
56101 pacid=27346932 transcript=56101 locus=MicpuC2.EuGene.
0000030255 ID=56101.3.0.228 annot-version=v3.0
Micromonas_pusilla	OG0000027
39033 pacid=27346951 transcript=39033
locus=MicpuC2.fgenesh1_pg.C_scaffold_3000540 ID=39033.3.0.228 annot-
version=v3.0	Micromonas_pusilla	OG0000027
62697 pacid=27346958 transcript=62697 locus=MicpuC2.est_orfs.
3_1680_4278541:1 ID=62697.3.0.228 annot-version=v3.0
Micromonas_pusilla	OG0000027
51524 pacid=27339479 transcript=51524 locus=MicpuC2.EuGene.
0000110046 ID=51524.3.0.228 annot-version=v3.0
Micromonas_pusilla	OG0000027
55984 pacid=27347072 transcript=55984 locus=MicpuC2.EuGene.
0000030138 ID=55984.3.0.228 annot-version=v3.0
Micromonas_pusilla	OG0000027
38834 pacid=27347109 transcript=38834
locus=MicpuC2.fgenesh1_pg.C_scaffold_3000341 ID=38834.3.0.228 annot-
version=v3.0	Micromonas_pusilla	OG0000027
56384 pacid=27347149 transcript=56384 locus=MicpuC2.EuGene.
0000030538 ID=56384.3.0.228 annot-version=v3.0
Micromonas_pusilla	OG0000027
56684 pacid=27347232 transcript=56684 locus=MicpuC2.EuGene.
0000030838 ID=56684.3.0.228 annot-version=v3.0
Micromonas_pusilla	OG0000027
51910 pacid=27339511 transcript=51910 locus=MicpuC2.EuGene.
0000110432 ID=51910.3.0.228 annot-version=v3.0
Micromonas_pusilla	OG0000027
56665 pacid=27347392 transcript=56665 locus=MicpuC2.EuGene.
0000030819 ID=56665.3.0.228 annot-version=v3.0
Micromonas_pusilla	OG0000027
56392 pacid=27347466 transcript=56392 locus=MicpuC2.EuGene.
0000030546 ID=56392.3.0.228 annot-version=v3.0
Micromonas_pusilla	OG0000027
56744 pacid=27347467 transcript=56744 locus=MicpuC2.EuGene.
0000030898 ID=56744.3.0.228 annot-version=v3.0
Micromonas_pusilla	OG0000027
56272 pacid=27347557 transcript=56272 locus=MicpuC2.EuGene.
0000030426 ID=56272.3.0.228 annot-version=v3.0
Micromonas_pusilla	OG0000027
51802 pacid=27339535 transcript=51802 locus=MicpuC2.EuGene.
0000110324 ID=51802.3.0.228 annot-version=v3.0
Micromonas_pusilla	OG0000027
51523 pacid=27339546 transcript=51523 locus=MicpuC2.EuGene.
0000110045 ID=51523.3.0.228 annot-version=v3.0
Micromonas_pusilla	OG0000027
56100 pacid=27347730 transcript=56100 locus=MicpuC2.EuGene.
0000030254 ID=56100.3.0.228 annot-version=v3.0
Micromonas_pusilla	OG0000027
51492 pacid=27339555 transcript=51492 locus=MicpuC2.EuGene.
0000110014 ID=51492.3.0.228 annot-version=v3.0

Micromonas_pusilla   OG0000027
51601 pacid=27339559 transcript=51601 locus=MicpuC2.EuGene.
0000110123 ID=51601.3.0.228 annot-version=v3.0
Micromonas_pusilla   OG0000027
58544 pacid=27347876 transcript=58544 locus=MicpuC2.EuGene.
0000060053 ID=58544.3.0.228 annot-version=v3.0
Micromonas_pusilla   OG0000027
59107 pacid=27347877 transcript=59107 locus=MicpuC2.EuGene.
0000060616 ID=59107.3.0.228 annot-version=v3.0
Micromonas_pusilla   OG0000027
51874 pacid=27339570 transcript=51874 locus=MicpuC2.EuGene.
0000110396 ID=51874.3.0.228 annot-version=v3.0
Micromonas_pusilla   OG0000027
51721 pacid=27339572 transcript=51721 locus=MicpuC2.EuGene.
0000110243 ID=51721.3.0.228 annot-version=v3.0
Micromonas_pusilla   OG0000027
58577 pacid=27347980 transcript=58577 locus=MicpuC2.EuGene.
0000060086 ID=58577.3.0.228 annot-version=v3.0
Micromonas_pusilla   OG0000027
59043 pacid=27348046 transcript=59043 locus=MicpuC2.EuGene.
0000060552 ID=59043.3.0.228 annot-version=v3.0
Micromonas_pusilla   OG0000027
69556 pacid=27348092 transcript=69556 locus=MicpuC2.est_orfs.
6_8539_4271228:1 ID=69556.3.0.228 annot-version=v3.0
Micromonas_pusilla   OG0000027
58571 pacid=27348132 transcript=58571 locus=MicpuC2.EuGene.
0000060080 ID=58571.3.0.228 annot-version=v3.0
Micromonas_pusilla   OG0000027
47591 pacid=27348164 transcript=47591
locus=MicpuC2.estExt_fgenesh1_pg.C_60314 ID=47591.3.0.228 annot-
version=v3.0   Micromonas_pusilla   OG0000027
59053 pacid=27348193 transcript=59053 locus=MicpuC2.EuGene.
0000060562 ID=59053.3.0.228 annot-version=v3.0
Micromonas_pusilla   OG0000027
58507 pacid=27348239 transcript=58507 locus=MicpuC2.EuGene.
0000060016 ID=58507.3.0.228 annot-version=v3.0
Micromonas_pusilla   OG0000027
58556 pacid=27348276 transcript=58556 locus=MicpuC2.EuGene.
0000060065 ID=58556.3.0.228 annot-version=v3.0
Micromonas_pusilla   OG0000027
58835 pacid=27348317 transcript=58835 locus=MicpuC2.EuGene.
0000060344 ID=58835.3.0.228 annot-version=v3.0
Micromonas_pusilla   OG0000027
51911 pacid=27339614 transcript=51911 locus=MicpuC2.EuGene.
0000110433 ID=51911.3.0.228 annot-version=v3.0
Micromonas_pusilla   OG0000027
58688 pacid=27348377 transcript=58688 locus=MicpuC2.EuGene.
0000060197 ID=58688.3.0.228 annot-version=v3.0
Micromonas_pusilla   OG0000027
40353 pacid=27348407 transcript=40353
locus=MicpuC2.fgenesh1_pg.C_scaffold_6000330 ID=40353.3.0.228 annot-
version=v3.0   Micromonas_pusilla   OG0000027
51512 pacid=27339621 transcript=51512 locus=MicpuC2.EuGene.
0000110034 ID=51512.3.0.228 annot-version=v3.0

Micromonas_pusilla	OG0000027
43030 pacid=27348568 transcript=43030
locus=MicpuC2.fgenesh1_pg.C_scaffold_15000156 ID=43030.3.0.228
annot-version=v3.0	Micromonas_pusilla	OG0000027
53386 pacid=27348600 transcript=53386 locus=MicpuC2.EuGene.
0000150061 ID=53386.3.0.228 annot-version=v3.0
Micromonas_pusilla	OG0000027
53360 pacid=27348651 transcript=53360 locus=MicpuC2.EuGene.
0000150035 ID=53360.3.0.228 annot-version=v3.0
Micromonas_pusilla	OG0000027
64896 pacid=27397106 transcript=64896 locus=EuGene.1600010257
ID=64896.3.0.229 annot-version=v3.0	Micromonas_sp._RCC299
OG0000027
61806 pacid=27397738 transcript=61806 locus=EuGene.1000010143
ID=61806.3.0.229 annot-version=v3.0	Micromonas_sp._RCC299
OG0000027
102665 pacid=27397754 transcript=102665
locus=fgenesh2_pg.C_Chr_10000151 ID=102665.3.0.229 annot-
version=v3.0	Micromonas_sp._RCC299	OG0000027
57455 pacid=27397883 transcript=57455 locus=EuGene.0300010883
ID=57455.3.0.229 annot-version=v3.0	Micromonas_sp._RCC299
OG0000027
57351 pacid=27397929 transcript=57351 locus=EuGene.0300010779
ID=57351.3.0.229 annot-version=v3.0	Micromonas_sp._RCC299
OG0000027
58120 pacid=27400291 transcript=58120 locus=EuGene.0400010649
ID=58120.3.0.229 annot-version=v3.0	Micromonas_sp._RCC299
OG0000027
64322 pacid=27401023 transcript=64322 locus=EuGene.1500010043
ID=64322.3.0.229 annot-version=v3.0	Micromonas_sp._RCC299
OG0000027
61396 pacid=27401307 transcript=61396 locus=EuGene.0900010379
ID=61396.3.0.229 annot-version=v3.0	Micromonas_sp._RCC299
OG0000027
63590 pacid=27402752 transcript=63590 locus=EuGene.1300010240
ID=63590.3.0.229 annot-version=v3.0	Micromonas_sp._RCC299
OG0000027
60318 pacid=27404354 transcript=60318 locus=EuGene.0700010603
ID=60318.3.0.229 annot-version=v3.0	Micromonas_sp._RCC299
OG0000027
kfl00530_0060_v1.1	Klebsormidium_flaccidum	OG0000027
kfl00007_0540_v1.1	Klebsormidium_flaccidum	OG0000027
kfl00175_0240_v1.1	Klebsormidium_flaccidum	OG0000027
CDF35551 pep supercontig:ASM35022v2:HG001735:104399:105571:1
gene:CHC_T00004081001 transcript:CDF35551
gene_biotype:protein_coding transcript_biotype:protein_coding
Chondrus_crispus OG0000027
g6012.t1 Caulerpa_lentillifera	OG0000028
Cre02.g076987.t1.1 pacid=30786328 transcript=Cre02.g076987.t1.1
locus=Cre02.g076987 ID=Cre02.g076987.t1.1.v5.5 annot-version=v5.5
Chlamydomonas_reinhardtii OG0000028
Cre03.g153850.t1.2 pacid=30787539 transcript=Cre03.g153850.t1.2
locus=Cre03.g153850 ID=Cre03.g153850.t1.2.v5.5 annot-version=v5.5
Chlamydomonas_reinhardtii OG0000028

Cre03.g177800.t1.1 pacid=30787616 transcript=Cre03.g177800.t1.1 locus=Cre03.g177800 ID=Cre03.g177800.t1.1.v5.5 annot-version=v5.5 Chlamydomonas_reinhardtii OG0000028
Cre10.g462816.t1.1 pacid=30790012 transcript=Cre10.g462816.t1.1 locus=Cre10.g462816 ID=Cre10.g462816.t1.1.v5.5 annot-version=v5.5 Chlamydomonas_reinhardtii OG0000028
Cre04.g226750.t1.2 pacid=30791524 transcript=Cre04.g226750.t1.2 locus=Cre04.g226750 ID=Cre04.g226750.t1.2.v5.5 annot-version=v5.5 Chlamydomonas_reinhardtii OG0000028
Cre14.g631950.t1.2 pacid=30776455 transcript=Cre14.g631950.t1.2 locus=Cre14.g631950 ID=Cre14.g631950.t1.2.v5.5 annot-version=v5.5 Chlamydomonas_reinhardtii OG0000028
Cre16.g686700.t1.1 pacid=30777001 transcript=Cre16.g686700.t1.1 locus=Cre16.g686700 ID=Cre16.g686700.t1.1.v5.5 annot-version=v5.5 Chlamydomonas_reinhardtii OG0000028
Cre08.g381600.t1.2 pacid=30773906 transcript=Cre08.g381600.t1.2 locus=Cre08.g381600 ID=Cre08.g381600.t1.2.v5.5 annot-version=v5.5 Chlamydomonas_reinhardtii OG0000028
Cre08.g381550.t1.2 pacid=30773959 transcript=Cre08.g381550.t1.2 locus=Cre08.g381550 ID=Cre08.g381550.t1.2.v5.5 annot-version=v5.5 Chlamydomonas_reinhardtii OG0000028
Cre17.g723100.t1.2 pacid=30782892 transcript=Cre17.g723100.t1.2 locus=Cre17.g723100 ID=Cre17.g723100.t1.2.v5.5 annot-version=v5.5 Chlamydomonas_reinhardtii OG0000028
Vocar.0001s1760.1.p pacid=32884840 transcript=Vocar.0001s1760.1 locus=Vocar.0001s1760 ID=Vocar.0001s1760.1.v2.1 annot-version=v2.1 Volvox_carteri OG0000028
Vocar.0029s0018.1.p pacid=32887563 transcript=Vocar.0029s0018.1 locus=Vocar.0029s0018 ID=Vocar.0029s0018.1.v2.1 annot-version=v2.1 Volvox_carteri OG0000028
Vocar.0004s0005.1.p pacid=32889744 transcript=Vocar.0004s0005.1 locus=Vocar.0004s0005 ID=Vocar.0004s0005.1.v2.1 annot-version=v2.1 Volvox_carteri OG0000028
Vocar.0024s0080.1.p pacid=32890389 transcript=Vocar.0024s0080.1 locus=Vocar.0024s0080 ID=Vocar.0024s0080.1.v2.1 annot-version=v2.1 Volvox_carteri OG0000028
Vocar.0073s0002.1.p pacid=32891685 transcript=Vocar.0073s0002.1 locus=Vocar.0073s0002 ID=Vocar.0073s0002.1.v2.1 annot-version=v2.1 Volvox_carteri OG0000028
Vocar.0022s0205.1.p pacid=32891786 transcript=Vocar.0022s0205.1 locus=Vocar.0022s0205 ID=Vocar.0022s0205.1.v2.1 annot-version=v2.1 Volvox_carteri OG0000028
Vocar.0042s0065.1.p pacid=32892099 transcript=Vocar.0042s0065.1 locus=Vocar.0042s0065 ID=Vocar.0042s0065.1.v2.1 annot-version=v2.1 Volvox_carteri OG0000028
Vocar.0019s0161.1.p pacid=32892691 transcript=Vocar.0019s0161.1 locus=Vocar.0019s0161 ID=Vocar.0019s0161.1.v2.1 annot-version=v2.1 Volvox_carteri OG0000028
jgi|ChlNC64A_1|140772|IGS.gm_3_00511 Chlorella_variabilis OG0000028
jgi|ChlNC64A_1|49465|fgenesh3_pg.C_scaffold_1000163 Chlorella_variabilis OG0000028
jgi|ChlNC64A_1|143802|IGS.gm_6_00376 Chlorella_variabilis OG0000028

jgi|ChlNC64A_1|143026|IGS.gm_5_00355	Chlorella_variabilis
OG0000028
jgi|ChlNC64A_1|136255|IGS.gm_15_00351	Chlorella_variabilis
OG0000028
jgi|ChlNC64A_1|136257|IGS.gm_15_00353	Chlorella_variabilis
OG0000028
41892 pacid=27386926 transcript=41892 locus=fgenesh1_pg.7_#_296
ID=41892.2.0.227 annot-version=v2.0	Coccomyxa_subellipsoidea
OG0000028
46828 pacid=27387071 transcript=46828
locus=estExt_fgenesh1_pg.C_50078 ID=46828.2.0.227 annot-version=v2.0
	Coccomyxa_subellipsoidea	OG0000028
60082 pacid=27387758 transcript=60082 locus=Genemark1.8398_g
ID=60082.2.0.227 annot-version=v2.0	Coccomyxa_subellipsoidea
OG0000028
46000 pacid=27389228 transcript=46000
locus=estExt_fgenesh1_pg.C_10555 ID=46000.2.0.227 annot-version=v2.0
	Coccomyxa_subellipsoidea	OG0000028
62378 pacid=27390926 transcript=62378 locus=Genemark1.3161_g
ID=62378.2.0.227 annot-version=v2.0	Coccomyxa_subellipsoidea
OG0000028
41217 pacid=27390942 transcript=41217 locus=fgenesh1_pg.6_#_20
ID=41217.2.0.227 annot-version=v2.0	Coccomyxa_subellipsoidea
OG0000028
62379 pacid=27391367 transcript=62379 locus=Genemark1.3162_g
ID=62379.2.0.227 annot-version=v2.0	Coccomyxa_subellipsoidea
OG0000028
36246_1300010218 Ostreococcus_tauri	OG0000028
60822 pacid=27344315 transcript=60822 locus=MicpuC2.EuGene.
0000090373 ID=60822.3.0.228 annot-version=v3.0
Micromonas_pusilla	OG0000028
101026 pacid=27402025 transcript=101026
locus=fgenesh2_pg.C_Chr_06000227 ID=101026.3.0.229 annot-
version=v3.0	Micromonas_sp._RCC299	OG0000028
kfl00557_g9_v1.1 Klebsormidium_flaccidum	OG0000028
kfl00557_0080_v1.1	Klebsormidium_flaccidum	OG0000028
kfl00568_0060_v1.1	Klebsormidium_flaccidum	OG0000028
kfl00586_g9_v1.1 Klebsormidium_flaccidum	OG0000028
kfl00728_0080_v1.1	Klebsormidium_flaccidum	OG0000028
kfl00857_0040_v1.1	Klebsormidium_flaccidum	OG0000028
kfl00920_0050_v1.1	Klebsormidium_flaccidum	OG0000028
kfl01015_0040_v1.1	Klebsormidium_flaccidum	OG0000028
kfl01234_0010_v1.1	Klebsormidium_flaccidum	OG0000028
kfl00036_0270_v1.1	Klebsormidium_flaccidum	OG0000028
kfl00080_0150_v1.1	Klebsormidium_flaccidum	OG0000028
kfl00093_0150_v1.1	Klebsormidium_flaccidum	OG0000028
kfl00218_0060_v1.1	Klebsormidium_flaccidum	OG0000028
kfl00219_0300_v1.1	Klebsormidium_flaccidum	OG0000028
kfl00239_0180_v1.1	Klebsormidium_flaccidum	OG0000028
kfl00012_0440_v1.1	Klebsormidium_flaccidum	OG0000028
Pp3c7_12870V3.1.p pacid=32926141 transcript=Pp3c7_12870V3.1
locus=Pp3c7_12870 annot-version=v3.3	Physcomitrella_patens
OG0000028
Pp3c13_20360V3.1.p pacid=32929985 transcript=Pp3c13_20360V3.1

locus=Pp3c13_20360 annot-version=v3.3	Physcomitrella_patens
OG0000028
Pp3c13_8440V3.1.p pacid=32932044 transcript=Pp3c13_8440V3.1
locus=Pp3c13_8440 annot-version=v3.3	Physcomitrella_patens
OG0000028
Pp3c22_17770V3.1.p pacid=32902836 transcript=Pp3c22_17770V3.1
locus=Pp3c22_17770 annot-version=v3.3	Physcomitrella_patens
OG0000028
Pp3c2_3390V3.1.p pacid=32935742 transcript=Pp3c2_3390V3.1
locus=Pp3c2_3390 annot-version=v3.3	Physcomitrella_patens
OG0000028
Pp3c3_37280V3.1.p pacid=32940891 transcript=Pp3c3_37280V3.1
locus=Pp3c3_37280 annot-version=v3.3	Physcomitrella_patens
OG0000028
Pp3c3_16280V3.1.p pacid=32944862 transcript=Pp3c3_16280V3.1
locus=Pp3c3_16280 annot-version=v3.3	Physcomitrella_patens
OG0000028
Pp3c22_760V3.1.p pacid=32904131 transcript=Pp3c22_760V3.1
locus=Pp3c22_760 annot-version=v3.3	Physcomitrella_patens
OG0000028
Pp3c22_3550V3.1.p pacid=32904194 transcript=Pp3c22_3550V3.1
locus=Pp3c22_3550 annot-version=v3.3	Physcomitrella_patens
OG0000028
Pp3c20_5780V3.1.p pacid=32947751 transcript=Pp3c20_5780V3.1
locus=Pp3c20_5780 annot-version=v3.3	Physcomitrella_patens
OG0000028
Pp3c22_630V3.1.p pacid=32904727 transcript=Pp3c22_630V3.1
locus=Pp3c22_630 annot-version=v3.3	Physcomitrella_patens
OG0000028
Pp3c11_12000V3.1.p pacid=32956516 transcript=Pp3c11_12000V3.1
locus=Pp3c11_12000 annot-version=v3.3	Physcomitrella_patens
OG0000028
Pp3c11_12090V3.1.p pacid=32958760 transcript=Pp3c11_12090V3.1
locus=Pp3c11_12090 annot-version=v3.3	Physcomitrella_patens
OG0000028
Pp3c14_18510V3.1.p pacid=32961092 transcript=Pp3c14_18510V3.1
locus=Pp3c14_18510 annot-version=v3.3	Physcomitrella_patens
OG0000028
Pp3c14_18030V3.1.p pacid=32961302 transcript=Pp3c14_18030V3.1
locus=Pp3c14_18030 annot-version=v3.3	Physcomitrella_patens
OG0000028
Pp3c14_18590V3.1.p pacid=32962242 transcript=Pp3c14_18590V3.1
locus=Pp3c14_18590 annot-version=v3.3	Physcomitrella_patens
OG0000028
Pp3c14_18450V3.1.p pacid=32962581 transcript=Pp3c14_18450V3.1
locus=Pp3c14_18450 annot-version=v3.3	Physcomitrella_patens
OG0000028
Pp3c14_18040V3.1.p pacid=32962796 transcript=Pp3c14_18040V3.1
locus=Pp3c14_18040 annot-version=v3.3	Physcomitrella_patens
OG0000028
Pp3c14_18440V3.1.p pacid=32963137 transcript=Pp3c14_18440V3.1
locus=Pp3c14_18440 annot-version=v3.3	Physcomitrella_patens
OG0000028
Pp3c8_870V3.1.p pacid=32963898 transcript=Pp3c8_870V3.1

locus=Pp3c8_870 annot-version=v3.3 Physcomitrella_patens
OG0000028
Pp3c8_1320V3.1.p pacid=32964128 transcript=Pp3c8_1320V3.1
locus=Pp3c8_1320 annot-version=v3.3 Physcomitrella_patens
OG0000028
Pp3c8_13450V3.1.p pacid=32965005 transcript=Pp3c8_13450V3.1
locus=Pp3c8_13450 annot-version=v3.3 Physcomitrella_patens
OG0000028
Pp3c8_1330V3.1.p pacid=32965501 transcript=Pp3c8_1330V3.1
locus=Pp3c8_1330 annot-version=v3.3 Physcomitrella_patens
OG0000028
Pp3c8_15200V3.1.p pacid=32965529 transcript=Pp3c8_15200V3.1
locus=Pp3c8_15200 annot-version=v3.3 Physcomitrella_patens
OG0000028
Pp3c8_13470V3.1.p pacid=32965821 transcript=Pp3c8_13470V3.1
locus=Pp3c8_13470 annot-version=v3.3 Physcomitrella_patens
OG0000028
Pp3c8_14900V3.1.p pacid=32965909 transcript=Pp3c8_14900V3.1
locus=Pp3c8_14900 annot-version=v3.3 Physcomitrella_patens
OG0000028
Pp3c1_37980V3.1.p pacid=32968791 transcript=Pp3c1_37980V3.1
locus=Pp3c1_37980 annot-version=v3.3 Physcomitrella_patens
OG0000028
Pp3c17_12970V3.1.p pacid=32906760 transcript=Pp3c17_12970V3.1
locus=Pp3c17_12970 annot-version=v3.3 Physcomitrella_patens
OG0000028
Pp3c12_4560V3.1.p pacid=32974609 transcript=Pp3c12_4560V3.1
locus=Pp3c12_4560 annot-version=v3.3 Physcomitrella_patens
OG0000028
Pp3c17_12980V3.1.p pacid=32907390 transcript=Pp3c17_12980V3.1
locus=Pp3c17_12980 annot-version=v3.3 Physcomitrella_patens
OG0000028
Pp3c18_19660V3.1.p pacid=32982460 transcript=Pp3c18_19660V3.1
locus=Pp3c18_19660 annot-version=v3.3 Physcomitrella_patens
OG0000028
Pp3c18_19720V3.1.p pacid=32982753 transcript=Pp3c18_19720V3.1
locus=Pp3c18_19720 annot-version=v3.3 Physcomitrella_patens
OG0000028
Pp3c18_19690V3.1.p pacid=32983093 transcript=Pp3c18_19690V3.1
locus=Pp3c18_19690 annot-version=v3.3 Physcomitrella_patens
OG0000028
Pp3c24_9260V3.1.p pacid=32909044 transcript=Pp3c24_9260V3.1
locus=Pp3c24_9260 annot-version=v3.3 Physcomitrella_patens
OG0000028
Pp3c24_15400V3.1.p pacid=32909056 transcript=Pp3c24_15400V3.1
locus=Pp3c24_15400 annot-version=v3.3 Physcomitrella_patens
OG0000028
Pp3c24_9250V3.1.p pacid=32910500 transcript=Pp3c24_9250V3.1
locus=Pp3c24_9250 annot-version=v3.3 Physcomitrella_patens
OG0000028
Pp3c21_1890V3.1.p pacid=32914788 transcript=Pp3c21_1890V3.1
locus=Pp3c21_1890 annot-version=v3.3 Physcomitrella_patens
OG0000028
Pp3c21_1850V3.1.p pacid=32915544 transcript=Pp3c21_1850V3.1

locus=Pp3c21_1850 annot-version=v3.3 Physcomitrella_patens OG0000028
Pp3c21_1940V3.1.p pacid=32916295 transcript=Pp3c21_1940V3.1 locus=Pp3c21_1940 annot-version=v3.3 Physcomitrella_patens OG0000028
Pp3c7_26000V3.1.p pacid=32922983 transcript=Pp3c7_26000V3.1 locus=Pp3c7_26000 annot-version=v3.3 Physcomitrella_patens OG0000028
Pp3c7_25830V3.1.p pacid=32923761 transcript=Pp3c7_25830V3.1 locus=Pp3c7_25830 annot-version=v3.3 Physcomitrella_patens OG0000028
Pp3c7_25820V3.1.p pacid=32924107 transcript=Pp3c7_25820V3.1 locus=Pp3c7_25820 annot-version=v3.3 Physcomitrella_patens OG0000028
Pp3c7_12940V3.1.p pacid=32925030 transcript=Pp3c7_12940V3.1 locus=Pp3c7_12940 annot-version=v3.3 Physcomitrella_patens OG0000028
Pp3c7_12810V3.1.p pacid=32925106 transcript=Pp3c7_12810V3.1 locus=Pp3c7_12810 annot-version=v3.3 Physcomitrella_patens OG0000028
Pp3c7_25260V3.1.p pacid=32925341 transcript=Pp3c7_25260V3.1 locus=Pp3c7_25260 annot-version=v3.3 Physcomitrella_patens OG0000028
AT2G37640.1 | Barwin-like endoglucanases superfamily protein | Chr2:15788077-15789812 REVERSE LENGTH=262 | 201606 Arabidopsis_thaliana OG0000028
AT2G39700.1 | expansin A4 | Chr2:16544246-16545434 REVERSE LENGTH=257 | 201606 Arabidopsis_thaliana OG0000028
AT2G40610.1 | expansin A8 | Chr2:16949121-16950472 REVERSE LENGTH=253 | 201606 Arabidopsis_thaliana OG0000028
AT2G45110.1 | expansin B4 | Chr2:18599575-18601237 FORWARD LENGTH=259 | 201606 Arabidopsis_thaliana OG0000028
AT3G03220.1 | expansin A13 | Chr3:742655-743975 REVERSE LENGTH=266 | 201606 Arabidopsis_thaliana OG0000028
AT1G12560.1 | expansin A7 | Chr1:4276557-4277693 FORWARD LENGTH=262 | 201606 Arabidopsis_thaliana OG0000028
AT3G15370.1 | expansin 12 | Chr3:5190649-5191982 FORWARD LENGTH=252 | 201606 Arabidopsis_thaliana OG0000028
AT3G29030.1 | expansin A5 | Chr3:11011538-11013068 REVERSE LENGTH=255 | 201606 Arabidopsis_thaliana OG0000028
AT3G45960.1 | expansin-like A3 | Chr3:16893058-16893789 FORWARD LENGTH=215 | 201606 Arabidopsis_thaliana OG0000028
AT3G45970.1 | expansin-like A1 | Chr3:16896238-16897189 FORWARD LENGTH=265 | 201606 Arabidopsis_thaliana OG0000028
AT3G55500.1 | expansin A16 | Chr3:20575073-20576102 REVERSE LENGTH=260 | 201606 Arabidopsis_thaliana OG0000028
AT3G60570.1 | expansin B5 | Chr3:22391247-22392463 FORWARD LENGTH=264 | 201606 Arabidopsis_thaliana OG0000028
AT4G01630.1 | expansin A17 | Chr4:700653-701527 FORWARD LENGTH=255 | 201606 Arabidopsis_thaliana OG0000028
AT4G17030.1 | expansin-like B1 | Chr4:9581817-9583181 REVERSE LENGTH=250 | 201606 Arabidopsis_thaliana OG0000028
AT4G28250.1 | expansin B3 | Chr4:14000446-14001945 REVERSE LENGTH=264 | 201606 Arabidopsis_thaliana OG0000028

AT1G20190.1 | expansin 11 | Chr1:6998762-6999710 REVERSE LENGTH=252 | 201606 Arabidopsis_thaliana OG0000028
AT4G38210.1 | expansin A20 | Chr4:17922975-17923821 REVERSE LENGTH=256 | 201606 Arabidopsis_thaliana OG0000028
AT4G38400.1 | expansin-like A2 | Chr4:17978675-17979665 REVERSE LENGTH=265 | 201606 Arabidopsis_thaliana OG0000028
AT5G02260.1 | expansin A9 | Chr5:463224-464840 FORWARD LENGTH=258 | 201606 Arabidopsis_thaliana OG0000028
AT5G05290.1 | expansin A2 | Chr5:1568752-1569712 FORWARD LENGTH=255 | 201606 Arabidopsis_thaliana OG0000028
AT5G39260.1 | expansin A21 | Chr5:15726479-15727416 REVERSE LENGTH=262 | 201606 Arabidopsis_thaliana OG0000028
AT5G39270.1 | expansin A22 | Chr5:15729118-15730198 REVERSE LENGTH=279 | 201606 Arabidopsis_thaliana OG0000028
AT5G39280.1 | expansin A23 | Chr5:15730713-15731754 REVERSE LENGTH=275 | 201606 Arabidopsis_thaliana OG0000028
AT5G39290.1 | expansin-A25 protein | Chr5:15735871-15736956 REVERSE LENGTH=279 | 201606 Arabidopsis_thaliana OG0000028
AT5G39300.1 | expansin A25 | Chr5:15737427-15738435 REVERSE LENGTH=276 | 201606 Arabidopsis_thaliana OG0000028
AT5G39310.1 | expansin A24 | Chr5:15739280-15740562 REVERSE LENGTH=312 | 201606 Arabidopsis_thaliana OG0000028
AT5G56320.1 | expansin A14 | Chr5:22808854-22809906 FORWARD LENGTH=255 | 201606 Arabidopsis_thaliana OG0000028
AT1G26770.1 | expansin A10 | Chr1:9259805-9260792 FORWARD LENGTH=249 | 201606 Arabidopsis_thaliana OG0000028
AT1G62980.1 | expansin A18 | Chr1:23331728-23332934 FORWARD LENGTH=257 | 201606 Arabidopsis_thaliana OG0000028
AT1G65680.1 | expansin B2 | Chr1:24427266-24428399 FORWARD LENGTH=273 | 201606 Arabidopsis_thaliana OG0000028
AT1G65681.1 | beta expansin 6 | Chr1:24428875-24430670 FORWARD LENGTH=223 | 201606 Arabidopsis_thaliana OG0000028
AT1G69530.1 | expansin A1 | Chr1:26142034-26143051 FORWARD LENGTH=250 | 201606 Arabidopsis_thaliana OG0000028
AT2G03090.1 | expansin A15 | Chr2:917361-918554 REVERSE LENGTH=253 | 201606 Arabidopsis_thaliana OG0000028
AT2G20750.1 | expansin B1 | Chr2:8941185-8942430 FORWARD LENGTH=271 | 201606 Arabidopsis_thaliana OG0000028
AT2G28950.1 | expansin A6 | Chr2:12431840-12433482 REVERSE LENGTH=257 | 201606 Arabidopsis_thaliana OG0000028
Os03t0336400-01 Similar to Alpha-expansin OsEXPA4. Oryza_sativa OG0000028
Os03t0377100-01 Similar to Expansin (Expansin2). Oryza_sativa OG0000028
Os01t0248900-01 Similar to Expansin Os-EXPA3. Oryza_sativa OG0000028
Os01t0249100-00 Similar to Expansin-A9. Oryza_sativa OG0000028
Os03t0428700-01 Similar to Expansin-A31. Oryza_sativa OG0000028
Os03t0645000-00 Similar to Expansin-B12. Oryza_sativa OG0000028
Os01t0274500-00 Similar to Expansin-A11. Oryza_sativa OG0000028

Os03t0822000-01 Alpha-expansin OsEXPA7.	Oryza_sativa	OG0000028
Os04t0228400-01 Expansin precursor (Alpha-expansin OsEXPA1).	Oryza_sativa	OG0000028
Os04t0317400-00 Similar to OSIGBa0113K06.3 protein.	Oryza_sativa	OG0000028
Os04t0317500-01 Pollen allergen Lol p2 family protein.	Oryza_sativa	OG0000028
Os04t0317800-01 Pollen allergen Lol p2 family protein.	Oryza_sativa	OG0000028
Os04t0328900-00 Similar to OSIGBa0113K06.3 protein.	Oryza_sativa	OG0000028
Os04t0329200-00 Pollen allergen Lol p2 family protein.	Oryza_sativa	OG0000028
Os04t0329300-00 Pollen allergen/expansin, C-terminal domain containing protein.	Oryza_sativa	OG0000028
Os04t0530100-01 Similar to Beta-expansin 1 precursor (AtEXPB1) (At-EXPB1) (Ath-ExpBeta-1.5).	Oryza_sativa	OG0000028
Os04t0552000-00 Barwin-related endoglucanase domain containing protein.	Oryza_sativa	OG0000028
Os04t0552200-00 Beta-expansin 5.	Oryza_sativa	OG0000028
Os04t0583500-01 Similar to Expansin 4 (Fragment).	Oryza_sativa	OG0000028
Os05t0246300-01 Barwin-related endoglucanase domain containing protein.	Oryza_sativa	OG0000028
Os05t0276500-01 Expansin Os-EXPA3.	Oryza_sativa	OG0000028
Os05t0277000-00 Similar to Expansin Os-EXPA3.	Oryza_sativa	OG0000028
Os05t0477600-01 Alpha-expansin OsEXPA4.	Oryza_sativa	OG0000028
Os06t0108600-00 Similar to Expansin-A17.	Oryza_sativa	OG0000028
Os06t0621900-01 Similar to Alpha-expansin OsEXPA16 (Fragment).	Oryza_sativa	OG0000028
Os06t0655200-01 Pollen allergen Lol p2 family protein.	Oryza_sativa	OG0000028
Os06t0662225-00 Similar to OSIGBa0113K06.3 protein.	Oryza_sativa	OG0000028
Os06t0662300-01 Pollen allergen Lol p2 family protein.	Oryza_sativa	OG0000028
Os06t0662500-00 Similar to OSIGBa0113K06.3 protein.	Oryza_sativa	OG0000028
Os06t0662600-00 Pollen allergen Lol p2 family protein.	Oryza_sativa	OG0000028
Os06t0662700-01 Pollen allergen Lol p2 family protein.	Oryza_sativa	OG0000028
Os06t0662850-00 Similar to OSIGBa0113K06.3 protein.	Oryza_sativa	OG0000028
Os06t0662900-00 Similar to OSIGBa0113K06.3 protein.	Oryza_sativa	OG0000028
Os06t0663300-00 Pollen allergen Lol p2 family protein.	Oryza_sativa	OG0000028
Os06t0718100-01 Similar to Alpha-expansin precursor.

Oryza_sativa	OG0000028
Os06t0725300-01 Barwin-related endoglucanase domain containing protein.	Oryza_sativa	OG0000028
Os07t0475400-01 Similar to Expansin-like protein A (Fragment).	Oryza_sativa	OG0000028
Os07t0496250-01 Similar to Expansin-like B1.	Oryza_sativa	OG0000028
Os08t0561900-01 Similar to Alpha expansin 26.	Oryza_sativa	OG0000028
Os10t0439100-00 Similar to cDNA clone:002-180-G03, full insert sequence.	Oryza_sativa	OG0000028
Os10t0439200-00 Similar to Expansin-A28.	Oryza_sativa	OG0000028
Os10t0535900-00 Similar to ATEXPA7 (ARABIDOPSIS THALIANA EXPANSIN A7).	Oryza_sativa	OG0000028
Os10t0542400-01 Expansin/Lol pI family protein.	Oryza_sativa	OG0000028
Os10t0548600-01 Beta-expansin precursor.	Oryza_sativa	OG0000028
Os10t0555600-01 Beta-expansin precursor.	Oryza_sativa	OG0000028
Os10t0555700-02 Beta-expansin.	Oryza_sativa	OG0000028
Os10t0555900-01 Similar to Beta-expansin.	Oryza_sativa	OG0000028
Os10t0556100-01 Similar to beta-expansin EXPB4.	Oryza_sativa	OG0000028
Os12t0546800-01 Alpha expansin 26.	Oryza_sativa	OG0000028
Os01t0823100-01 Alpha-expansin OsEXPA2.	Oryza_sativa	OG0000028
Os02t0267200-00 Alpha-expansin OsEXPA13.	Oryza_sativa	OG0000028
Os02t0267700-00 Similar to Expansin-A14.	Oryza_sativa	OG0000028
Os02t0267900-00 Similar to Expansin-A24.	Oryza_sativa	OG0000028
Os02t0268050-00 Similar to Expansin-A23.	Oryza_sativa	OG0000028
Os02t0268400-00 Similar to Expansin-A23.	Oryza_sativa	OG0000028
Os02t0268600-00 Similar to Expansin-A22.	Oryza_sativa	OG0000028
Os02t0639500-01 Similar to Expansin-B16.	Oryza_sativa	OG0000028
Os02t0658600-00 Similar to Beta-expansin (Fragment).	Oryza_sativa	OG0000028
Os02t0658800-01 Beta-expansin.	Oryza_sativa	OG0000028
Os02t0744200-01 Alpha-expansin OsEXPA5.	Oryza_sativa	OG0000028
Os03t0102500-01 Similar to Beta-expansin precursor (Fragment).	Oryza_sativa	OG0000028
Os03t0102700-01 Similar to Expansin-B7.	Oryza_sativa	OG0000028
Os03t0106500-01 Beta-expansin precursor (Beta-expansin 1).

Oryza_sativa   OG0000028
Os03t0106700-00 Expansin-B13.   Oryza_sativa   OG0000028
Os03t0106800-01 Beta-expansin precursor (Beta-expansin OsEXPB10).
Oryza_sativa   OG0000028
Os03t0106900-00 Beta-expansin precursor (Beta-expansin 1).
Oryza_sativa   OG0000028
Os03t0132200-01 Expansin-like protein A. Oryza_sativa
OG0000028
Os03t0155300-00 Similar to Alpha-expansin precursor.
Oryza_sativa   OG0000028
Os03t0155500-00 Similar to Expansin-A25. Oryza_sativa
OG0000028
Os03t0155700-00 Similar to Expansin-A15. Oryza_sativa
OG0000028
Os03t0155900-01 Alpha-expansin OsEXPA18. Oryza_sativa
OG0000028
Os03t0156000-00 Alpha-expansin OsEXPA19. Oryza_sativa
OG0000028
Os03t0156300-00 Alpha-expansin OsEXPA20. Oryza_sativa
OG0000028
AT2G39415.1 | F-box family protein | Chr2:16458607-16458966 REVERSE
LENGTH=119 | 201606   Arabidopsis_thaliana   OG0000029
AT2G42720.1 | FBD%2C F-box%2C Skp2-like and Leucine Rich Repeat
domains containing protein | Chr2:17785653-17787211 FORWARD
LENGTH=443 | 201606   Arabidopsis_thaliana   OG0000029
AT2G42730.1 | F-box/FBD/LRR protein | Chr2:17789606-17791218 REVERSE
LENGTH=457 | 201606   Arabidopsis_thaliana   OG0000029
AT3G03030.1 | F-box/RNI-like superfamily protein |
Chr3:682252-683850 FORWARD LENGTH=474 | 201606
Arabidopsis_thaliana   OG0000029
AT3G03040.1 | F-box/RNI-like superfamily protein |
Chr3:684685-686277 FORWARD LENGTH=472 | 201606
Arabidopsis_thaliana   OG0000029
AT3G10750.1 | FBD domain family | Chr3:3366927-3368269 FORWARD
LENGTH=299 | 201606   Arabidopsis_thaliana   OG0000029
AT3G14710.1 | RNI-like superfamily protein | Chr3:4941545-4943055
FORWARD LENGTH=442 | 201606   Arabidopsis_thaliana
OG0000029
AT3G19085.1 | F-box/RNI/FBD-like domain protein |
Chr3:6599589-6600211 FORWARD LENGTH=176 | 201606
Arabidopsis_thaliana   OG0000029
AT1G13780.1 | F-box/RNI-like/FBD-like domains-containing protein |
Chr1:4724681-4726314 FORWARD LENGTH=456 | 201606
Arabidopsis_thaliana   OG0000029
AT3G23955.1 | F-box family protein | Chr3:8655266-8657399 REVERSE
LENGTH=401 | 201606   Arabidopsis_thaliana   OG0000029
AT3G26920.1 | FBD / Leucine Rich Repeat domains containing protein |
Chr3:9921241-9922429 FORWARD LENGTH=340 | 201606
Arabidopsis_thaliana   OG0000029
AT3G26922.1 | F-box/RNI-like superfamily protein |
Chr3:9922858-9923891 FORWARD LENGTH=306 | 201606
Arabidopsis_thaliana   OG0000029
AT3G26930.1 | Protein with RNI-like/FBD-like domain |
Chr3:9925931-9927351 FORWARD LENGTH=409 | 201606

Arabidopsis_thaliana   OG0000029
AT3G42770.1 | F-box/RNI-like/FBD-like domains-containing protein | Chr3:14867122-14870083 FORWARD LENGTH=532 | 201606 Arabidopsis_thaliana   OG0000029
AT3G43415.1 | F-box/LRR protein | Chr3:15342812-15343309 REVERSE LENGTH=122 | 201606   Arabidopsis_thaliana   OG0000029
AT3G44060.1 | F-box/RNI-like superfamily protein | Chr3:15824938-15826391 REVERSE LENGTH=427 | 201606 Arabidopsis_thaliana   OG0000029
AT3G44080.1 | F-box family protein | Chr3:15835632-15837126 REVERSE LENGTH=387 | 201606   Arabidopsis_thaliana   OG0000029
AT3G44090.1 | F-box family protein | Chr3:15839123-15840759 FORWARD LENGTH=449 | 201606   Arabidopsis_thaliana   OG0000029
AT3G44180.1 | syntaxin-related family protein | Chr3:15900795-15901716 FORWARD LENGTH=202 | 201606 Arabidopsis_thaliana   OG0000029
AT3G44810.1 | F-box family protein | Chr3:16358496-16359983 REVERSE LENGTH=448 | 201606   Arabidopsis_thaliana   OG0000029
AT3G49020.1 | FBD%2C F-box and Leucine Rich Repeat domains containing protein | Chr3:18169079-18170660 FORWARD LENGTH=447 | 201606 Arabidopsis_thaliana   OG0000029
AT3G49030.1 | FBD%2C F-box and Leucine Rich Repeat domains containing protein | Chr3:18173138-18174769 FORWARD LENGTH=443 | 201606 Arabidopsis_thaliana   OG0000029
AT3G49040.1 | F-box/FBD/LRR protein | Chr3:18177676-18178839 FORWARD LENGTH=311 | 201606   Arabidopsis_thaliana   OG0000029
AT3G49045.1 | F-box/FBD/LRR protein | Chr3:18178882-18179339 FORWARD LENGTH=118 | 201606   Arabidopsis_thaliana   OG0000029
AT3G49150.1 | F-box/FBD/LRR protein | Chr3:18218858-18220430 FORWARD LENGTH=467 | 201606   Arabidopsis_thaliana   OG0000029
AT3G49480.1 | F-box/FBD/LRR protein | Chr3:18342544-18343473 REVERSE LENGTH=309 | 201606   Arabidopsis_thaliana   OG0000029
AT3G50710.1 | F-box/RNI-like/FBD-like domains-containing protein | Chr3:18845199-18846673 REVERSE LENGTH=427 | 201606 Arabidopsis_thaliana   OG0000029
AT3G51530.1 | F-box/RNI-like/FBD-like domains-containing protein | Chr3:19112873-19114497 REVERSE LENGTH=455 | 201606 Arabidopsis_thaliana   OG0000029
AT3G52680.1 | F-box/RNI-like/FBD-like domains-containing protein | Chr3:19527075-19528838 FORWARD LENGTH=456 | 201606 Arabidopsis_thaliana   OG0000029
AT3G52690.1 | RNI-like superfamily protein | Chr3:19530736-19531811 FORWARD LENGTH=299 | 201606   Arabidopsis_thaliana OG0000029
AT3G53550.1 | FBD-like domain family protein | Chr3:19856870-19858308 FORWARD LENGTH=256 | 201606 Arabidopsis_thaliana   OG0000029
AT3G54160.1 | RNI-like superfamily protein | Chr3:20053468-20055027 FORWARD LENGTH=434 | 201606   Arabidopsis_thaliana OG0000029
AT3G54910.1 | RNI-like superfamily protein | Chr3:20342967-20344277 REVERSE LENGTH=373 | 201606   Arabidopsis_thaliana OG0000029
AT3G55670.1 | F-box/FBD/LRR protein | Chr3:20658013-20659503 REVERSE

LENGTH=218 | 201606   Arabidopsis_thaliana   OG0000029
AT3G56780.1 | FBD%2C F-box and Leucine Rich Repeat domains containing protein | Chr3:21031613-21033202 FORWARD LENGTH=431 | 201606 Arabidopsis_thaliana   OG0000029
AT3G58820.1 | F-box/RNI-like superfamily protein | Chr3:21752960-21754615 FORWARD LENGTH=413 | 201606 Arabidopsis_thaliana   OG0000029
AT3G58860.1 | F-box/RNI-like superfamily protein | Chr3:21763742-21765306 REVERSE LENGTH=457 | 201606 Arabidopsis_thaliana   OG0000029
AT3G58880.1 | F-box/RNI-like superfamily protein | Chr3:21768485-21770167 REVERSE LENGTH=454 | 201606 Arabidopsis_thaliana   OG0000029
AT3G58890.1 | RNI-like superfamily protein | Chr3:21771046-21772577 FORWARD LENGTH=293 | 201606   Arabidopsis_thaliana OG0000029
AT3G58900.1 | F-box/RNI-like superfamily protein | Chr3:21773030-21774013 FORWARD LENGTH=327 | 201606 Arabidopsis_thaliana   OG0000029
AT3G58910.1 | F-box family protein | Chr3:21775306-21776079 REVERSE LENGTH=214 | 201606   Arabidopsis_thaliana   OG0000029
AT3G58920.1 | F-box/RNI-like superfamily protein | Chr3:21776293-21777988 FORWARD LENGTH=470 | 201606 Arabidopsis_thaliana   OG0000029
AT3G58930.1 | F-box/RNI-like superfamily protein | Chr3:21778381-21780014 REVERSE LENGTH=482 | 201606 Arabidopsis_thaliana   OG0000029
AT3G58940.1 | F-box/RNI-like superfamily protein | Chr3:21781002-21783213 REVERSE LENGTH=618 | 201606 Arabidopsis_thaliana   OG0000029
AT3G58950.1 | F-box/RNI-like superfamily protein | Chr3:21784116-21785840 REVERSE LENGTH=417 | 201606 Arabidopsis_thaliana   OG0000029
AT3G58960.1 | F-box/RNI-like/FBD-like domains-containing protein | Chr3:21787146-21788807 REVERSE LENGTH=475 | 201606 Arabidopsis_thaliana   OG0000029
AT3G58980.1 | F-box family protein | Chr3:21794445-21797006 REVERSE LENGTH=594 | 201606   Arabidopsis_thaliana   OG0000029
AT3G59000.1 | F-box/RNI-like superfamily protein | Chr3:21799757-21801536 FORWARD LENGTH=491 | 201606 Arabidopsis_thaliana   OG0000029
AT3G59150.1 | F-box/RNI superfamily protein | Chr3:21872831-21874422 FORWARD LENGTH=475 | 201606   Arabidopsis_thaliana OG0000029
AT3G59160.1 | F-box/RNI-like superfamily protein | Chr3:21875023-21876641 FORWARD LENGTH=464 | 201606 Arabidopsis_thaliana   OG0000029
AT3G59170.1 | F-box/RNI-like superfamily protein | Chr3:21878167-21879799 FORWARD LENGTH=473 | 201606 Arabidopsis_thaliana   OG0000029
AT3G59180.1 | Protein with RNI-like/FBD-like domain | Chr3:21880719-21882459 FORWARD LENGTH=475 | 201606 Arabidopsis_thaliana   OG0000029
AT3G59190.1 | F-box/RNI-like superfamily protein |

Chr3:21884740-21886101 FORWARD LENGTH=388 | 201606
Arabidopsis_thaliana OG0000029
AT3G59200.1 | F-box/RNI-like superfamily protein |
Chr3:21887484-21889214 FORWARD LENGTH=520 | 201606
Arabidopsis_thaliana OG0000029
AT3G59210.1 | F-box/RNI-like superfamily protein |
Chr3:21889942-21891553 FORWARD LENGTH=484 | 201606
Arabidopsis_thaliana OG0000029
AT3G59230.1 | RNI-like superfamily protein | Chr3:21896052-21897787
REVERSE LENGTH=491 | 201606 Arabidopsis_thaliana
OG0000029
AT3G59240.1 | RNI-like superfamily protein | Chr3:21898682-21900364
REVERSE LENGTH=504 | 201606 Arabidopsis_thaliana
OG0000029
AT3G59250.1 | F-box/RNI-like superfamily protein |
Chr3:21901726-21903284 REVERSE LENGTH=425 | 201606
Arabidopsis_thaliana OG0000029
AT3G59270.1 | FBD-like domain family protein |
Chr3:21906593-21907872 REVERSE LENGTH=335 | 201606
Arabidopsis_thaliana OG0000029
AT3G59460.1 | F-box/LRR protein | Chr3:21977513-21978183 FORWARD
LENGTH=63 | 201606 Arabidopsis_thaliana OG0000029
AT3G60040.1 | F-box family protein | Chr3:22175937-22179728 REVERSE
LENGTH=838 | 201606 Arabidopsis_thaliana OG0000029
AT3G60710.1 | F-box family protein | Chr3:22440198-22441472 FORWARD
LENGTH=320 | 201606 Arabidopsis_thaliana OG0000029
AT3G62430.1 | Protein with RNI-like/FBD-like domain |
Chr3:23093795-23095304 REVERSE LENGTH=437 | 201606
Arabidopsis_thaliana OG0000029
AT3G62440.1 | F-box/RNI-like superfamily protein |
Chr3:23096104-23097726 REVERSE LENGTH=457 | 201606
Arabidopsis_thaliana OG0000029
AT4G00160.1 | F-box/RNI-like/FBD-like domains-containing protein |
Chr4:63465-65365 FORWARD LENGTH=453 | 201606
Arabidopsis_thaliana OG0000029
AT4G00315.1 | F-box/RNI-like/FBD-like domains-containing protein |
Chr4:137404-138888 FORWARD LENGTH=441 | 201606
Arabidopsis_thaliana OG0000029
AT4G00320.1 | F-box/RNI-like superfamily protein |
Chr4:139939-141643 FORWARD LENGTH=507 | 201606
Arabidopsis_thaliana OG0000029
AT1G16930.1 | F-box/RNI-like/FBD-like domains-containing protein |
Chr1:5789987-5791534 FORWARD LENGTH=449 | 201606
Arabidopsis_thaliana OG0000029
AT1G16940.1 | F-box/RNI-like/FBD-like domains-containing protein |
Chr1:5791983-5793491 FORWARD LENGTH=434 | 201606
Arabidopsis_thaliana OG0000029
AT4G09920.1 | FBD%2C F-box and Leucine Rich Repeat domains
containing protein | Chr4:6224691-6225905 FORWARD LENGTH=316 |
201606 Arabidopsis_thaliana OG0000029
AT4G10400.1 | F-box/RNI-like/FBD-like domains-containing protein |
Chr4:6446335-6447715 REVERSE LENGTH=409 | 201606
Arabidopsis_thaliana OG0000029
AT4G10410.1 | Leucine-rich repeat (LRR) family protein |

Chr4:6448983-6449998 REVERSE LENGTH=263 | 201606
Arabidopsis_thaliana OG0000029
AT4G10420.1 | FBD / Leucine Rich Repeat domains containing protein |
Chr4:6453085-6454209 REVERSE LENGTH=322 | 201606
Arabidopsis_thaliana OG0000029
AT4G13960.1 | F-box/RNI-like superfamily protein |
Chr4:8060681-8062231 REVERSE LENGTH=464 | 201606
Arabidopsis_thaliana OG0000029
AT4G13965.1 | F-box/FBD/LRR protein | Chr4:8066138-8067576 REVERSE
LENGTH=423 | 201606 Arabidopsis_thaliana OG0000029
AT4G13985.1 | FBD-associated F-box protein | Chr4:8080118-8081669
REVERSE LENGTH=459 | 201606 Arabidopsis_thaliana
OG0000029
AT4G14096.1 | F-box/RNI-like superfamily protein |
Chr4:8124606-8126202 FORWARD LENGTH=468 | 201606
Arabidopsis_thaliana OG0000029
AT4G14103.1 | F-box/RNI-like superfamily protein |
Chr4:8126948-8128305 FORWARD LENGTH=381 | 201606
Arabidopsis_thaliana OG0000029
AT4G15060.1 | FBD%2C F-box/LRR protein | Chr4:8599035-8601761
FORWARD LENGTH=539 | 201606 Arabidopsis_thaliana
OG0000029
AT4G15075.1 | FBD-like domain family protein | Chr4:8607813-8608519
FORWARD LENGTH=168 | 201606 Arabidopsis_thaliana
OG0000029
AT4G22280.1 | F-box/RNI-like superfamily protein |
Chr4:11776054-11777517 REVERSE LENGTH=415 | 201606
Arabidopsis_thaliana OG0000029
AT1G19070.1 | F-box family protein | Chr1:6584454-6584705 FORWARD
LENGTH=83 | 201606 Arabidopsis_thaliana OG0000029
AT4G26340.1 | F-box/RNI-like/FBD-like domains-containing protein |
Chr4:13324130-13325559 FORWARD LENGTH=419 | 201606
Arabidopsis_thaliana OG0000029
AT4G26350.1 | F-box/RNI-like/FBD-like domains-containing protein |
Chr4:13326853-13328315 FORWARD LENGTH=431 | 201606
Arabidopsis_thaliana OG0000029
AT4G27050.1 | F-box/RNI-like superfamily protein |
Chr4:13575884-13577429 REVERSE LENGTH=453 | 201606
Arabidopsis_thaliana OG0000029
AT1G19410.1 | FBD / Leucine Rich Repeat domains containing protein |
Chr1:6714492-6716439 REVERSE LENGTH=435 | 201606
Arabidopsis_thaliana OG0000029
AT5G11370.1 | FBD / Leucine Rich Repeat domains containing protein |
Chr5:3627849-3629717 FORWARD LENGTH=311 | 201606
Arabidopsis_thaliana OG0000029
AT5G12450.1 | FBD-like domain family protein | Chr5:4040937-4041128
FORWARD LENGTH=63 | 201606 Arabidopsis_thaliana
OG0000029
AT1G21990.1 | F-box/RNI-like/FBD-like domains-containing protein |
Chr1:7740530-7742106 REVERSE LENGTH=465 | 201606
Arabidopsis_thaliana OG0000029
AT1G22000.1 | LOW protein Arabidopsis_thaliana OG0000029
AT5G15620.1 | F-box/RNI-like superfamily protein |
Chr5:5082465-5083991 FORWARD LENGTH=440 | 201606

Arabidopsis_thaliana	OG0000029
AT5G18770.1 | F-box/FBD-like domains containing protein | Chr5:6261426-6263172 FORWARD LENGTH=481 | 201606
Arabidopsis_thaliana	OG0000029
AT5G18780.1 | F-box/RNI-like superfamily protein | Chr5:6264610-6266097 FORWARD LENGTH=441 | 201606
Arabidopsis_thaliana	OG0000029
AT5G22590.1 | Leucine Rich Repeat protein family | Chr5:7504536-7505838 FORWARD LENGTH=304 | 201606
Arabidopsis_thaliana	OG0000029
AT5G22600.1 | FBD / Leucine Rich Repeat domains containing protein | Chr5:7510438-7511632 FORWARD LENGTH=333 | 201606
Arabidopsis_thaliana	OG0000029
AT5G22610.1 | F-box/RNI-like/FBD-like domains-containing protein | Chr5:7515217-7516916 FORWARD LENGTH=472 | 201606
Arabidopsis_thaliana	OG0000029
AT5G22660.1 | FBD%2C F-box%2C Skp2-like and Leucine Rich Repeat domains containing protein | Chr5:7536906-7537853 REVERSE LENGTH=315 | 201606 Arabidopsis_thaliana	OG0000029
AT5G22670.1 | F-box/RNI-like/FBD-like domains-containing protein | Chr5:7538192-7539685 REVERSE LENGTH=443 | 201606
Arabidopsis_thaliana	OG0000029
AT5G22700.1 | LOW protein Arabidopsis_thaliana	OG0000029
AT5G22720.1 | F-box/FBD/LRR protein | Chr5:7549565-7551174 REVERSE LENGTH=468 | 201606	Arabidopsis_thaliana	OG0000029
AT5G22730.1 | F-box/RNI-like/FBD-like domains-containing protein | Chr5:7551632-7553219 REVERSE LENGTH=466 | 201606
Arabidopsis_thaliana	OG0000029
AT5G25850.1 | F-box/RNI-like/FBD-like domains-containing protein | Chr5:9014082-9015917 FORWARD LENGTH=481 | 201606
Arabidopsis_thaliana	OG0000029
AT5G25860.1 | F-box/RNI-like superfamily protein | Chr5:9016908-9018424 REVERSE LENGTH=448 | 201606
Arabidopsis_thaliana	OG0000029
AT5G27750.1 | F-box/FBD-like domains containing protein | Chr5:9828430-9829981 FORWARD LENGTH=459 | 201606
Arabidopsis_thaliana	OG0000029
AT5G35926.1 | Protein with RNI-like/FBD-like domain | Chr5:14062676-14065597 FORWARD LENGTH=405 | 201606
Arabidopsis_thaliana	OG0000029
AT5G35995.1 | F-box/RNI-like superfamily protein | Chr5:14136707-14138230 REVERSE LENGTH=455 | 201606
Arabidopsis_thaliana	OG0000029
AT5G38386.1 | F-box/RNI-like superfamily protein | Chr5:15363178-15364815 FORWARD LENGTH=403 | 201606
Arabidopsis_thaliana	OG0000029
AT5G38390.1 | F-box/RNI-like superfamily protein | Chr5:15366432-15368053 FORWARD LENGTH=456 | 201606
Arabidopsis_thaliana	OG0000029
AT5G38391.1 | F-box/RNI superfamily protein | Chr5:15368254-15368851 FORWARD LENGTH=178 | 201606	Arabidopsis_thaliana
OG0000029
AT5G38392.1 | F-box/RNI superfamily protein | Chr5:15369685-15370338 FORWARD LENGTH=217 | 201606	Arabidopsis_thaliana

OG0000029
AT5G38396.1 | F-box/RNI-like superfamily protein |
Chr5:15373094-15374726 FORWARD LENGTH=462 | 201606
Arabidopsis_thaliana   OG0000029
AT5G38565.1 | F-box/FBD-like domains containing protein |
Chr5:15444571-15446009 REVERSE LENGTH=349 | 201606
Arabidopsis_thaliana   OG0000029
AT5G38570.1 | F-box/RNI-like superfamily protein |
Chr5:15449279-15450601 REVERSE LENGTH=379 | 201606
Arabidopsis_thaliana   OG0000029
AT5G38580.1 | FBD-like domain family protein |
Chr5:15451164-15451842 REVERSE LENGTH=168 | 201606
Arabidopsis_thaliana   OG0000029
AT5G38590.1 | F-box/RNI-like/FBD-like domains-containing protein |
Chr5:15452586-15453455 REVERSE LENGTH=289 | 201606
Arabidopsis_thaliana   OG0000029
AT5G40050.1 | F-box/FBD-like domains containing protein |
Chr5:16032083-16033551 REVERSE LENGTH=415 | 201606
Arabidopsis_thaliana   OG0000029
AT5G40750.1 | FBD / Leucine Rich Repeat domains containing protein |
Chr5:16308356-16309819 FORWARD LENGTH=297 | 201606
Arabidopsis_thaliana   OG0000029
AT5G41630.1 | F-box/RNI-like superfamily protein |
Chr5:16650282-16651835 FORWARD LENGTH=455 | 201606
Arabidopsis_thaliana   OG0000029
AT5G41830.1 | RNI-like superfamily protein | Chr5:16743762-16745334
REVERSE LENGTH=463 | 201606   Arabidopsis_thaliana
OG0000029
AT5G41840.1 | F-box/RNI-like superfamily protein |
Chr5:16754436-16756263 REVERSE LENGTH=540 | 201606
Arabidopsis_thaliana   OG0000029
AT5G44490.1 | FBD%2C F-box%2C Skp2-like and Leucine Rich Repeat
domains containing protein | Chr5:17924035-17925843 FORWARD
LENGTH=481 | 201606   Arabidopsis_thaliana   OG0000029
AT5G44850.1 | Protein with RNI-like/FBD-like domain |
Chr5:18108304-18109593 FORWARD LENGTH=331 | 201606
Arabidopsis_thaliana   OG0000029
AT5G44940.1 | F-box/RNI-like superfamily protein |
Chr5:18149369-18150892 FORWARD LENGTH=377 | 201606
Arabidopsis_thaliana   OG0000029
AT5G44950.1 | F-box/RNI-like/FBD-like domains-containing protein |
Chr5:18151665-18153164 FORWARD LENGTH=438 | 201606
Arabidopsis_thaliana   OG0000029
AT5G44960.1 | F-box/RNI-like/FBD-like domains-containing protein |
Chr5:18153852-18155391 FORWARD LENGTH=441 | 201606
Arabidopsis_thaliana   OG0000029
AT5G44970.1 | Protein with RNI-like/FBD-like domain |
Chr5:18155837-18157340 FORWARD LENGTH=389 | 201606
Arabidopsis_thaliana   OG0000029
AT5G44980.1 | F-box/RNI-like/FBD-like domains-containing protein |
Chr5:18160600-18162058 FORWARD LENGTH=435 | 201606
Arabidopsis_thaliana   OG0000029
AT5G50270.1 | F-box/RNI-like/FBD-like domains-containing protein |
Chr5:20457487-20458873 FORWARD LENGTH=408 | 201606

Arabidopsis_thaliana	OG0000029
AT5G52460.1 | FBD%2C F-box and Leucine Rich Repeat domains containing protein | Chr5:21292375-21293758 REVERSE LENGTH=369 | 201606	Arabidopsis_thaliana	OG0000029
AT5G53635.1 | F-box/RNI-like/FBD-like domains-containing protein | Chr5:21785999-21787444 REVERSE LENGTH=426 | 201606
Arabidopsis_thaliana	OG0000029
AT5G53637.1 | LOW protein	Arabidopsis_thaliana	OG0000029
AT5G53640.1 | F-box/FBD/LRR protein | Chr5:21789919-21790690 REVERSE LENGTH=225 | 201606	Arabidopsis_thaliana	OG0000029
AT5G53840.1 | F-box/RNI-like/FBD-like domains-containing protein | Chr5:21859148-21860672 REVERSE LENGTH=444 | 201606
Arabidopsis_thaliana	OG0000029
AT5G54820.1 | F-box/RNI-like superfamily protein | Chr5:22269859-22271777 FORWARD LENGTH=472 | 201606
Arabidopsis_thaliana	OG0000029
AT5G56325.1 | FBD-like domain family protein | Chr5:22812213-22813193 FORWARD LENGTH=264 | 201606
Arabidopsis_thaliana	OG0000029
AT5G56370.1 | F-box/RNI-like/FBD-like domains-containing protein | Chr5:22836200-22837619 REVERSE LENGTH=421 | 201606
Arabidopsis_thaliana	OG0000029
AT5G56380.1 | F-box/RNI-like/FBD-like domains-containing protein | Chr5:22838836-22840353 REVERSE LENGTH=439 | 201606
Arabidopsis_thaliana	OG0000029
AT5G56390.1 | F-box/RNI-like/FBD-like domains-containing protein | Chr5:22842790-22844273 REVERSE LENGTH=428 | 201606
Arabidopsis_thaliana	OG0000029
AT5G56400.1 | FBD%2C F-box%2C Skp2-like and Leucine Rich Repeat domains containing protein | Chr5:22846069-22847615 REVERSE LENGTH=455 | 201606	Arabidopsis_thaliana	OG0000029
AT5G56410.1 | Protein with RNI-like/FBD-like domain | Chr5:22848508-22850331 REVERSE LENGTH=503 | 201606
Arabidopsis_thaliana	OG0000029
AT5G56420.1 | F-box/RNI-like/FBD-like domains-containing protein | Chr5:22850863-22852334 REVERSE LENGTH=422 | 201606
Arabidopsis_thaliana	OG0000029
AT5G56430.1 | F-box/FBD-like domains containing protein | Chr5:22853323-22854575 REVERSE LENGTH=360 | 201606
Arabidopsis_thaliana	OG0000029
AT5G56440.1 | F-box/RNI-like/FBD-like domains-containing protein | Chr5:22855742-22857117 REVERSE LENGTH=430 | 201606
Arabidopsis_thaliana	OG0000029
AT5G56452.1 | FBD-like domain family protein | Chr5:22860483-22861336 REVERSE LENGTH=227 | 201606
Arabidopsis_thaliana	OG0000029
AT5G56560.1 | FBD%2C F-box and Leucine Rich Repeat domains containing protein | Chr5:22899724-22901985 FORWARD LENGTH=607 | 201606	Arabidopsis_thaliana	OG0000029
AT5G56570.1 | Leucine-rich repeat (LRR) family protein | Chr5:22902736-22904404 FORWARD LENGTH=439 | 201606
Arabidopsis_thaliana	OG0000029
AT5G56690.1 | FBD%2C F-box and Leucine Rich Repeat domains containing protein | Chr5:22939723-22941099 FORWARD LENGTH=402 |

201606	Arabidopsis_thaliana	OG0000029
AT5G56700.1 | FBD / Leucine Rich Repeat domains containing protein | Chr5:22942104-22942958 FORWARD LENGTH=284 | 201606	Arabidopsis_thaliana	OG0000029
AT5G56800.1 | Protein with RNI-like/FBD-like domain | Chr5:22972989-22974208 FORWARD LENGTH=344 | 201606	Arabidopsis_thaliana	OG0000029
AT5G56810.1 | F-box/RNI-like/FBD-like domains-containing protein | Chr5:22974520-22975992 FORWARD LENGTH=435 | 201606	Arabidopsis_thaliana	OG0000029
AT5G56820.1 | F-box/RNI-like/FBD-like domains-containing protein | Chr5:22976334-22977838 FORWARD LENGTH=435 | 201606	Arabidopsis_thaliana	OG0000029
AT5G60610.1 | F-box/RNI-like superfamily protein | Chr5:24364049-24365386 FORWARD LENGTH=388 | 201606	Arabidopsis_thaliana	OG0000029
AT1G26815.1 | FBD domain family | Chr1:9290874-9291194 REVERSE LENGTH=106 | 201606	Arabidopsis_thaliana	OG0000029
AT1G26890.1 | FBD%2C F-box and Leucine Rich Repeat domains containing protein | Chr1:9317589-9319078 FORWARD LENGTH=419 | 201606	Arabidopsis_thaliana	OG0000029
AT5G62970.1 | Protein with RNI-like/FBD-like domain | Chr5:25272033-25273537 FORWARD LENGTH=449 | 201606	Arabidopsis_thaliana	OG0000029
AT1G32020.1 | F-box family protein | Chr1:11512552-11513067 REVERSE LENGTH=171 | 201606	Arabidopsis_thaliana	OG0000029
AT1G32375.1 | F-box/RNI-like/FBD-like domains-containing protein | Chr1:11679230-11680679 FORWARD LENGTH=422 | 201606	Arabidopsis_thaliana	OG0000029
AT1G47695.1 | F-box/RNI/FBD-like domain protein | Chr1:17546354-17546545 REVERSE LENGTH=63 | 201606	Arabidopsis_thaliana	OG0000029
AT1G47915.1 | F-box family protein | Chr1:17660752-17660973 FORWARD LENGTH=73 | 201606	Arabidopsis_thaliana	OG0000029
AT1G47920.1 | RNI-like superfamily protein | Chr1:17661095-17662394 FORWARD LENGTH=379 | 201606	Arabidopsis_thaliana	OG0000029
AT1G48390.1 | RNI-like superfamily protein | Chr1:17879448-17880903 REVERSE LENGTH=413 | 201606	Arabidopsis_thaliana	OG0000029
AT1G48400.1 | F-box/RNI-like/FBD-like domains-containing protein | Chr1:17882136-17883855 REVERSE LENGTH=513 | 201606	Arabidopsis_thaliana	OG0000029
AT1G50980.1 | F-box/RNI-like/FBD-like domains-containing protein | Chr1:18900541-18902057 REVERSE LENGTH=370 | 201606	Arabidopsis_thaliana	OG0000029
AT1G51055.1 | FBD-like domain family protein | Chr1:18925286-18926315 REVERSE LENGTH=161 | 201606	Arabidopsis_thaliana	OG0000029
AT1G51370.1 | F-box/RNI-like/FBD-like domains-containing protein | Chr1:19045615-19047141 FORWARD LENGTH=435 | 201606	Arabidopsis_thaliana	OG0000029
AT1G05080.1 | F-box/RNI-like/FBD-like domains-containing protein | Chr1:1459091-1460579 FORWARD LENGTH=439 | 201606

Arabidopsis_thaliana	OG0000029
AT1G52650.1 | F-box/RNI superfamily protein | Chr1:19610856-19612417 FORWARD LENGTH=465 | 201606	Arabidopsis_thaliana	OG0000029
AT1G55030.1 | RNI-like superfamily protein | Chr1:20530417-20531768 REVERSE LENGTH=422 | 201606	Arabidopsis_thaliana	OG0000029
AT1G55660.1 | FBD%2C F-box and Leucine Rich Repeat domains containing protein | Chr1:20800694-20802340 FORWARD LENGTH=492 | 201606	Arabidopsis_thaliana	OG0000029
AT1G56610.1 | Protein with RNI-like/FBD-like domain | Chr1:21212189-21213960 FORWARD LENGTH=535 | 201606	Arabidopsis_thaliana	OG0000029
AT1G58310.1 | F-box/RNI-like superfamily protein | Chr1:21631947-21633642 FORWARD LENGTH=505 | 201606	Arabidopsis_thaliana	OG0000029
AT1G60400.1 | F-box/RNI-like superfamily protein | Chr1:22254405-22255798 FORWARD LENGTH=403 | 201606	Arabidopsis_thaliana	OG0000029
AT1G60410.1 | F-box family protein | Chr1:22259129-22260526 FORWARD LENGTH=406 | 201606	Arabidopsis_thaliana	OG0000029
AT1G64540.1 | F-box/FBD-like domains containing protein | Chr1:23966909-23968425 FORWARD LENGTH=444 | 201606	Arabidopsis_thaliana	OG0000029
AT1G66290.1 | F-box/RNI-like superfamily protein | Chr1:24713306-24714843 REVERSE LENGTH=453 | 201606	Arabidopsis_thaliana	OG0000029
AT1G66300.1 | F-box/RNI-like/FBD-like domains-containing protein | Chr1:24716497-24718050 REVERSE LENGTH=456 | 201606	Arabidopsis_thaliana	OG0000029
AT1G66310.1 | F-box/RNI-like/FBD-like domains-containing protein | Chr1:24722971-24724475 REVERSE LENGTH=442 | 201606	Arabidopsis_thaliana	OG0000029
AT1G66320.1 | F-box/RNI-like superfamily protein | Chr1:24727041-24728614 REVERSE LENGTH=457 | 201606	Arabidopsis_thaliana	OG0000029
AT1G66640.1 | RNI-like superfamily protein | Chr1:24857438-24859011 FORWARD LENGTH=416 | 201606	Arabidopsis_thaliana	OG0000029
AT1G69630.1 | F-box/RNI-like superfamily protein | Chr1:26191640-26193174 REVERSE LENGTH=451 | 201606	Arabidopsis_thaliana	OG0000029
AT1G06630.1 | F-box/RNI-like superfamily protein | Chr1:2028071-2031173 FORWARD LENGTH=477 | 201606	Arabidopsis_thaliana	OG0000029
AT1G78730.1 | FBD%2C F-box%2C Skp2-like and Leucine Rich Repeat domains containing protein | Chr1:29607840-29609441 REVERSE LENGTH=420 | 201606	Arabidopsis_thaliana	OG0000029
AT1G78740.1 | RNI-like superfamily protein | Chr1:29610494-29611438 REVERSE LENGTH=287 | 201606	Arabidopsis_thaliana	OG0000029
AT1G78750.1 | F-box/RNI-like superfamily protein | Chr1:29613122-29614695 REVERSE LENGTH=458 | 201606	Arabidopsis_thaliana	OG0000029

AT1G78760.1 | F-box/RNI-like superfamily protein |
Chr1:29615174-29616738 REVERSE LENGTH=452 | 201606
Arabidopsis_thaliana OG0000029
AT1G78840.1 | F-box/RNI-like/FBD-like domains-containing protein |
Chr1:29639290-29640817 REVERSE LENGTH=439 | 201606
Arabidopsis_thaliana OG0000029
AT1G80470.1 | F-box/RNI-like/FBD-like domains-containing protein |
Chr1:30256360-30257916 FORWARD LENGTH=464 | 201606
Arabidopsis_thaliana OG0000029
AT2G04230.1 | FBD%2C F-box and Leucine Rich Repeat domains
containing protein | Chr2:1447025-1448678 REVERSE LENGTH=448 |
201606 Arabidopsis_thaliana OG0000029
AT2G26030.1 | F-box/RNI-like/FBD-like domains-containing protein |
Chr2:11091505-11093000 REVERSE LENGTH=442 | 201606
Arabidopsis_thaliana OG0000029
AT2G26860.1 | FBD%2C F-box and Leucine Rich Repeat domains
containing protein | Chr2:11452196-11453584 REVERSE LENGTH=405 |
201606 Arabidopsis_thaliana OG0000029
AT2G29910.1 | F-box/RNI-like superfamily protein |
Chr2:12751686-12752830 REVERSE LENGTH=352 | 201606
Arabidopsis_thaliana OG0000029
AT2G29930.1 | F-box/RNI-like superfamily protein |
Chr2:12756457-12758087 REVERSE LENGTH=459 | 201606
Arabidopsis_thaliana OG0000029
Os04t0267600-01 Cyclin-like F-box domain containing protein.
Oryza_sativa OG0000029
Os04t0651600-00 Similar to OSIGBa0113E10.10 protein.
Oryza_sativa OG0000029
Os07t0285900-00 F-box domain, cyclin-like domain containing protein.
Oryza_sativa OG0000029
Os07t0547100-00 F-box domain, cyclin-like domain containing protein.
Oryza_sativa OG0000029
Os08t0197000-01 Cyclin-like F-box domain containing protein.
Oryza_sativa OG0000029
Os08t0197050-00 Hypothetical conserved gene. Oryza_sativa
OG0000029
Os08t0197300-01 F-box domain, cyclin-like domain containing protein.
Oryza_sativa OG0000029
Os08t0461300-01 Cyclin-like F-box domain containing protein.
Oryza_sativa OG0000029
Os09t0443000-00 F-box domain, Skp2-like domain containing protein.
Oryza_sativa OG0000029
Os09t0498700-01 F-box domain, cyclin-like domain containing protein.
Oryza_sativa OG0000029
Os12t0558400-01 Similar to F-box domain containing protein.
Oryza_sativa OG0000029
Cre02.g099400.t1.1 pacid=30784873 transcript=Cre02.g099400.t1.1
locus=Cre02.g099400 ID=Cre02.g099400.t1.1.v5.5 annot-version=v5.5
Chlamydomonas_reinhardtii OG0000030
Cre02.g144100.t1.2 pacid=30784893 transcript=Cre02.g144100.t1.2
locus=Cre02.g144100 ID=Cre02.g144100.t1.2.v5.5 annot-version=v5.5
Chlamydomonas_reinhardtii OG0000030
Cre02.g111150.t1.1 pacid=30785056 transcript=Cre02.g111150.t1.1
locus=Cre02.g111150 ID=Cre02.g111150.t1.1.v5.5 annot-version=v5.5

Chlamydomonas_reinhardtii OG0000030
Cre02.g116600.t1.2 pacid=30785587 transcript=Cre02.g116600.t1.2
locus=Cre02.g116600 ID=Cre02.g116600.t1.2.v5.5 annot-version=v5.5
Chlamydomonas_reinhardtii OG0000030
Cre02.g144050.t1.2 pacid=30785706 transcript=Cre02.g144050.t1.2
locus=Cre02.g144050 ID=Cre02.g144050.t1.2.v5.5 annot-version=v5.5
Chlamydomonas_reinhardtii OG0000030
Cre03.g205250.t1.1 pacid=30786856 transcript=Cre03.g205250.t1.1
locus=Cre03.g205250 ID=Cre03.g205250.t1.1.v5.5 annot-version=v5.5
Chlamydomonas_reinhardtii OG0000030
Cre03.g202550.t1.1 pacid=30786870 transcript=Cre03.g202550.t1.1
locus=Cre03.g202550 ID=Cre03.g202550.t1.1.v5.5 annot-version=v5.5
Chlamydomonas_reinhardtii OG0000030
Cre03.g157000.t1.1 pacid=30787575 transcript=Cre03.g157000.t1.1
locus=Cre03.g157000 ID=Cre03.g157000.t1.1.v5.5 annot-version=v5.5
Chlamydomonas_reinhardtii OG0000030
Cre03.g204100.t1.1 pacid=30787821 transcript=Cre03.g204100.t1.1
locus=Cre03.g204100 ID=Cre03.g204100.t1.1.v5.5 annot-version=v5.5
Chlamydomonas_reinhardtii OG0000030
Cre03.g204050.t1.1 pacid=30787956 transcript=Cre03.g204050.t1.1
locus=Cre03.g204050 ID=Cre03.g204050.t1.1.v5.5 annot-version=v5.5
Chlamydomonas_reinhardtii OG0000030
Cre10.g430250.t1.2 pacid=30789949 transcript=Cre10.g430250.t1.2
locus=Cre10.g430250 ID=Cre10.g430250.t1.2.v5.5 annot-version=v5.5
Chlamydomonas_reinhardtii OG0000030
Cre10.g430100.t1.1 pacid=30790691 transcript=Cre10.g430100.t1.1
locus=Cre10.g430100 ID=Cre10.g430100.t1.1.v5.5 annot-version=v5.5
Chlamydomonas_reinhardtii OG0000030
Cre04.g211900.t1.1 pacid=30791009 transcript=Cre04.g211900.t1.1
locus=Cre04.g211900 ID=Cre04.g211900.t1.1.v5.5 annot-version=v5.5
Chlamydomonas_reinhardtii OG0000030
Cre04.g211950.t1.1 pacid=30791287 transcript=Cre04.g211950.t1.1
locus=Cre04.g211950 ID=Cre04.g211950.t1.1.v5.5 annot-version=v5.5
Chlamydomonas_reinhardtii OG0000030
Cre12.g502450.t1.1 pacid=30792029 transcript=Cre12.g502450.t1.1
locus=Cre12.g502450 ID=Cre12.g502450.t1.1.v5.5 annot-version=v5.5
Chlamydomonas_reinhardtii OG0000030
Cre12.g561101.t1.1 pacid=30792212 transcript=Cre12.g561101.t1.1
locus=Cre12.g561101 ID=Cre12.g561101.t1.1.v5.5 annot-version=v5.5
Chlamydomonas_reinhardtii OG0000030
Cre12.g502500.t1.2 pacid=30793102 transcript=Cre12.g502500.t1.2
locus=Cre12.g502500 ID=Cre12.g502500.t1.2.v5.5 annot-version=v5.5
Chlamydomonas_reinhardtii OG0000030
Cre12.g561050.t1.1 pacid=30793276 transcript=Cre12.g561050.t1.1
locus=Cre12.g561050 ID=Cre12.g561050.t1.1.v5.5 annot-version=v5.5
Chlamydomonas_reinhardtii OG0000030
Cre11.g483400.t1.1 pacid=30775498 transcript=Cre11.g483400.t1.1
locus=Cre11.g483400 ID=Cre11.g483400.t1.1.v5.5 annot-version=v5.5
Chlamydomonas_reinhardtii OG0000030
Cre11.g467660.t1.1 pacid=30775899 transcript=Cre11.g467660.t1.1
locus=Cre11.g467660 ID=Cre11.g467660.t1.1.v5.5 annot-version=v5.5
Chlamydomonas_reinhardtii OG0000030
Cre14.g632847.t1.1 pacid=30776263 transcript=Cre14.g632847.t1.1
locus=Cre14.g632847 ID=Cre14.g632847.t1.1.v5.5 annot-version=v5.5

Chlamydomonas_reinhardtii OG0000030
Cre14.g619854.t1.2 pacid=30776683 transcript=Cre14.g619854.t1.2
locus=Cre14.g619854 ID=Cre14.g619854.t1.2.v5.5 annot-version=v5.5
Chlamydomonas_reinhardtii OG0000030
Cre14.g622050.t1.1 pacid=30776707 transcript=Cre14.g622050.t1.1
locus=Cre14.g622050 ID=Cre14.g622050.t1.1.v5.5 annot-version=v5.5
Chlamydomonas_reinhardtii OG0000030
Cre08.g382560.t1.1 pacid=30773736 transcript=Cre08.g382560.t1.1
locus=Cre08.g382560 ID=Cre08.g382560.t1.1.v5.5 annot-version=v5.5
Chlamydomonas_reinhardtii OG0000030
Cre06.g278220.t1.1 pacid=30778388 transcript=Cre06.g278220.t1.1
locus=Cre06.g278220 ID=Cre06.g278220.t1.1.v5.5 annot-version=v5.5
Chlamydomonas_reinhardtii OG0000030
Cre06.g278093.t1.1 pacid=30778470 transcript=Cre06.g278093.t1.1
locus=Cre06.g278093 ID=Cre06.g278093.t1.1.v5.5 annot-version=v5.5
Chlamydomonas_reinhardtii OG0000030
Cre06.g311950.t1.1 pacid=30778541 transcript=Cre06.g311950.t1.1
locus=Cre06.g311950 ID=Cre06.g311950.t1.1.v5.5 annot-version=v5.5
Chlamydomonas_reinhardtii OG0000030
Cre06.g278094.t1.1 pacid=30780171 transcript=Cre06.g278094.t1.1
locus=Cre06.g278094 ID=Cre06.g278094.t1.1.v5.5 annot-version=v5.5
Chlamydomonas_reinhardtii OG0000030
Cre09.g414750.t1.1 pacid=30780368 transcript=Cre09.g414750.t1.1
locus=Cre09.g414750 ID=Cre09.g414750.t1.1.v5.5 annot-version=v5.5
Chlamydomonas_reinhardtii OG0000030
Cre09.g397956.t1.1 pacid=30780850 transcript=Cre09.g397956.t1.1
locus=Cre09.g397956 ID=Cre09.g397956.t1.1.v5.5 annot-version=v5.5
Chlamydomonas_reinhardtii OG0000030
Cre09.g415100.t1.1 pacid=30781122 transcript=Cre09.g415100.t1.1
locus=Cre09.g415100 ID=Cre09.g415100.t1.1.v5.5 annot-version=v5.5
Chlamydomonas_reinhardtii OG0000030
Cre09.g417250.t1.1 pacid=30781134 transcript=Cre09.g417250.t1.1
locus=Cre09.g417250 ID=Cre09.g417250.t1.1.v5.5 annot-version=v5.5
Chlamydomonas_reinhardtii OG0000030
Cre09.g414900.t1.1 pacid=30781214 transcript=Cre09.g414900.t1.1
locus=Cre09.g414900 ID=Cre09.g414900.t1.1.v5.5 annot-version=v5.5
Chlamydomonas_reinhardtii OG0000030
Cre09.g388393.t1.1 pacid=30781276 transcript=Cre09.g388393.t1.1
locus=Cre09.g388393 ID=Cre09.g388393.t1.1.v5.5 annot-version=v5.5
Chlamydomonas_reinhardtii OG0000030
Cre09.g390750.t1.1 pacid=30781541 transcript=Cre09.g390750.t1.1
locus=Cre09.g390750 ID=Cre09.g390750.t1.1.v5.5 annot-version=v5.5
Chlamydomonas_reinhardtii OG0000030
Cre17.g721750.t1.1 pacid=30781961 transcript=Cre17.g721750.t1.1
locus=Cre17.g721750 ID=Cre17.g721750.t1.1.v5.5 annot-version=v5.5
Chlamydomonas_reinhardtii OG0000030
Cre07.g325050.t1.1 pacid=30774230 transcript=Cre07.g325050.t1.1
locus=Cre07.g325050 ID=Cre07.g325050.t1.1.v5.5 annot-version=v5.5
Chlamydomonas_reinhardtii OG0000030
Cre24.g755397.t1.1 pacid=30782911 transcript=Cre24.g755397.t1.1
locus=Cre24.g755397 ID=Cre24.g755397.t1.1.v5.5 annot-version=v5.5
Chlamydomonas_reinhardtii OG0000030
Cre05.g233450.t1.2 pacid=30782950 transcript=Cre05.g233450.t1.2
locus=Cre05.g233450 ID=Cre05.g233450.t1.2.v5.5 annot-version=v5.5

Chlamydomonas_reinhardtii OG0000030
Cre13.g577400.t1.1 pacid=30784467 transcript=Cre13.g577400.t1.1
locus=Cre13.g577400 ID=Cre13.g577400.t1.1.v5.5 annot-version=v5.5
Chlamydomonas_reinhardtii OG0000030
Cre13.g577050.t1.1 pacid=30784540 transcript=Cre13.g577050.t1.1
locus=Cre13.g577050 ID=Cre13.g577050.t1.1.v5.5 annot-version=v5.5
Chlamydomonas_reinhardtii OG0000030
Vocar.0054s0024.1.p pacid=32894660 transcript=Vocar.0054s0024.1
locus=Vocar.0054s0024 ID=Vocar.0054s0024.1.v2.1 annot-version=v2.1
Volvox_carteri OG0000030
Vocar.0001s0947.1.p pacid=32884344 transcript=Vocar.0001s0947.1
locus=Vocar.0001s0947 ID=Vocar.0001s0947.1.v2.1 annot-version=v2.1
Volvox_carteri OG0000030
Vocar.0011s0147.1.p pacid=32895272 transcript=Vocar.0011s0147.1
locus=Vocar.0011s0147 ID=Vocar.0011s0147.1.v2.1 annot-version=v2.1
Volvox_carteri OG0000030
Vocar.0011s0196.1.p pacid=32895410 transcript=Vocar.0011s0196.1
locus=Vocar.0011s0196 ID=Vocar.0011s0196.1.v2.1 annot-version=v2.1
Volvox_carteri OG0000030
Vocar.0096s0004.1.p pacid=32896555 transcript=Vocar.0096s0004.1
locus=Vocar.0096s0004 ID=Vocar.0096s0004.1.v2.1 annot-version=v2.1
Volvox_carteri OG0000030
Vocar.0123s0001.1.p pacid=32896937 transcript=Vocar.0123s0001.1
locus=Vocar.0123s0001 ID=Vocar.0123s0001.1.v2.1 annot-version=v2.1
Volvox_carteri OG0000030
Vocar.0025s0110.1.p pacid=32898404 transcript=Vocar.0025s0110.1
locus=Vocar.0025s0110 ID=Vocar.0025s0110.1.v2.1 annot-version=v2.1
Volvox_carteri OG0000030
Vocar.0001s0945.1.p pacid=32884737 transcript=Vocar.0001s0945.1
locus=Vocar.0001s0945 ID=Vocar.0001s0945.1.v2.1 annot-version=v2.1
Volvox_carteri OG0000030
Vocar.0015s0025.1.p pacid=32899186 transcript=Vocar.0015s0025.1
locus=Vocar.0015s0025 ID=Vocar.0015s0025.1.v2.1 annot-version=v2.1
Volvox_carteri OG0000030
Vocar.0047s0030.1.p pacid=32899298 transcript=Vocar.0047s0030.1
locus=Vocar.0047s0030 ID=Vocar.0047s0030.1.v2.1 annot-version=v2.1
Volvox_carteri OG0000030
Vocar.0047s0031.1.p pacid=32899347 transcript=Vocar.0047s0031.1
locus=Vocar.0047s0031 ID=Vocar.0047s0031.1.v2.1 annot-version=v2.1
Volvox_carteri OG0000030
Vocar.0047s0033.1.p pacid=32899354 transcript=Vocar.0047s0033.1
locus=Vocar.0047s0033 ID=Vocar.0047s0033.1.v2.1 annot-version=v2.1
Volvox_carteri OG0000030
Vocar.0001s0946.1.p pacid=32885315 transcript=Vocar.0001s0946.1
locus=Vocar.0001s0946 ID=Vocar.0001s0946.1.v2.1 annot-version=v2.1
Volvox_carteri OG0000030
Vocar.0001s1131.1.p pacid=32885979 transcript=Vocar.0001s1131.1
locus=Vocar.0001s1131 ID=Vocar.0001s1131.1.v2.1 annot-version=v2.1
Volvox_carteri OG0000030
Vocar.0033s0034.1.p pacid=32886504 transcript=Vocar.0033s0034.1
locus=Vocar.0033s0034 ID=Vocar.0033s0034.1.v2.1 annot-version=v2.1
Volvox_carteri OG0000030
Vocar.0005s0264.1.p pacid=32883545 transcript=Vocar.0005s0264.1
locus=Vocar.0005s0264 ID=Vocar.0005s0264.1.v2.1 annot-version=v2.1

Volvox_carteri   OG0000030
Vocar.0007s0362.1.p pacid=32888318 transcript=Vocar.0007s0362.1
locus=Vocar.0007s0362 ID=Vocar.0007s0362.1.v2.1 annot-version=v2.1
Volvox_carteri   OG0000030
Vocar.0005s0263.1.p pacid=32883774 transcript=Vocar.0005s0263.1
locus=Vocar.0005s0263 ID=Vocar.0005s0263.1.v2.1 annot-version=v2.1
Volvox_carteri   OG0000030
Vocar.0003s0406.1.p pacid=32889204 transcript=Vocar.0003s0406.1
locus=Vocar.0003s0406 ID=Vocar.0003s0406.1.v2.1 annot-version=v2.1
Volvox_carteri   OG0000030
Vocar.0005s0261.1.p pacid=32883840 transcript=Vocar.0005s0261.1
locus=Vocar.0005s0261 ID=Vocar.0005s0261.1.v2.1 annot-version=v2.1
Volvox_carteri   OG0000030
Vocar.0002s0279.1.p pacid=32890981 transcript=Vocar.0002s0279.1
locus=Vocar.0002s0279 ID=Vocar.0002s0279.1.v2.1 annot-version=v2.1
Volvox_carteri   OG0000030
Vocar.0017s0185.1.p pacid=32891465 transcript=Vocar.0017s0185.1
locus=Vocar.0017s0185 ID=Vocar.0017s0185.1.v2.1 annot-version=v2.1
Volvox_carteri   OG0000030
Vocar.0017s0181.1.p pacid=32891533 transcript=Vocar.0017s0181.1
locus=Vocar.0017s0181 ID=Vocar.0017s0181.1.v2.1 annot-version=v2.1
Volvox_carteri   OG0000030
Vocar.0022s0066.1.p pacid=32891899 transcript=Vocar.0022s0066.1
locus=Vocar.0022s0066 ID=Vocar.0022s0066.1.v2.1 annot-version=v2.1
Volvox_carteri   OG0000030
Vocar.0022s0173.1.p pacid=32891965 transcript=Vocar.0022s0173.1
locus=Vocar.0022s0173 ID=Vocar.0022s0173.1.v2.1 annot-version=v2.1
Volvox_carteri   OG0000030
Vocar.0042s0044.1.p pacid=32892114 transcript=Vocar.0042s0044.1
locus=Vocar.0042s0044 ID=Vocar.0042s0044.1.v2.1 annot-version=v2.1
Volvox_carteri   OG0000030
Vocar.0019s0088.1.p pacid=32892830 transcript=Vocar.0019s0088.1
locus=Vocar.0019s0088 ID=Vocar.0019s0088.1.v2.1 annot-version=v2.1
Volvox_carteri   OG0000030
jgi|ChlNC64A_1|140476|IGS.gm_3_00215   Chlorella_variabilis
OG0000030
jgi|ChlNC64A_1|56485|estExt_fgenesh3_pg.C_10128
Chlorella_variabilis   OG0000030
jgi|ChlNC64A_1|133110|IGS.gm_1_00246   Chlorella_variabilis
OG0000030
jgi|ChlNC64A_1|134888|IGS.gm_12_00274   Chlorella_variabilis
OG0000030
jgi|ChlNC64A_1|135016|IGS.gm_12_00402   Chlorella_variabilis
OG0000030
jgi|ChlNC64A_1|59023|estExt_fgenesh3_pg.C_240078
Chlorella_variabilis   OG0000030
jgi|ChlNC64A_1|133230|IGS.gm_1_00366   Chlorella_variabilis
OG0000030
jgi|ChlNC64A_1|138314|IGS.gm_20_00063   Chlorella_variabilis
OG0000030
jgi|ChlNC64A_1|52572|fgenesh3_pg.C_scaffold_11000148
Chlorella_variabilis   OG0000030
jgi|ChlNC64A_1|51514|fgenesh3_pg.C_scaffold_7000061
Chlorella_variabilis   OG0000030

jgi|ChlNC64A_1|133704|IGS.gm_1_00840	Chlorella_variabilis
OG0000030
jgi|ChlNC64A_1|143109|IGS.gm_5_00438	Chlorella_variabilis
OG0000030
jgi|ChlNC64A_1|141180|IGS.gm_32_00018	Chlorella_variabilis
OG0000030
13404 pacid=27390062 transcript=13404 locus=e_gw1.4.127.1
ID=13404.2.0.227 annot-version=v2.0	Coccomyxa_subellipsoidea
OG0000030
40557 pacid=27390087 transcript=40557 locus=fgenesh1_pg.4_#_206
ID=40557.2.0.227 annot-version=v2.0	Coccomyxa_subellipsoidea
OG0000030
12762 pacid=27390636 transcript=12762 locus=e_gw1.3.36.1
ID=12762.2.0.227 annot-version=v2.0	Coccomyxa_subellipsoidea
OG0000030
48268 pacid=27386155 transcript=48268
locus=estExt_fgenesh1_pg.C_130075 ID=48268.2.0.227 annot-
version=v2.0	Coccomyxa_subellipsoidea	OG0000030
9432 pacid=27392627 transcript=9432 locus=gw1.24.80.1
ID=9432.2.0.227 annot-version=v2.0	Coccomyxa_subellipsoidea
OG0000030
9438 pacid=27386481 transcript=9438 locus=gw1.23.188.1
ID=9438.2.0.227 annot-version=v2.0	Coccomyxa_subellipsoidea
OG0000030
9502 pacid=27394548 transcript=9502 locus=gw1.27.40.1
ID=9502.2.0.227 annot-version=v2.0	Coccomyxa_subellipsoidea
OG0000030
31865 pacid=27394560 transcript=31865
locus=estExt_Genewise1Plus.C_270024 ID=31865.2.0.227 annot-
version=v2.0	Coccomyxa_subellipsoidea	OG0000030
45043 pacid=27395227 transcript=45043 locus=fgenesh1_pg.21_#_68
ID=45043.2.0.227 annot-version=v2.0	Coccomyxa_subellipsoidea
OG0000030
30479_0100010467 Ostreococcus_tauri	OG0000030
9991_fgenesh1_pg.C_Chr_02.0001000286	Ostreococcus_tauri
OG0000030
18436 pacid=27415859 transcript=18436
locus=fgenesh1_pg.C_Chr_16000001 ID=18436.2.0.231 annot-version=v2.0
Ostreococcus_lucimarinus	OG0000030
27359 pacid=27419732 transcript=27359 locus=eugene.1400010074
ID=27359.2.0.231 annot-version=v2.0	Ostreococcus_lucimarinus
OG0000030
26328 pacid=27420609 transcript=26328 locus=eugene.1100010001
ID=26328.2.0.231 annot-version=v2.0	Ostreococcus_lucimarinus
OG0000030
70776 pacid=27345777 transcript=70776 locus=MicpuC2.AZW_EuGene.
0000010046 ID=70776.3.0.228 annot-version=v3.0
Micromonas_pusilla	OG0000030
54821 pacid=27398764 transcript=54821 locus=EuGene.0100010048
ID=54821.3.0.229 annot-version=v3.0	Micromonas_sp._RCC299
OG0000030
99882 pacid=27400536 transcript=99882
locus=fgenesh2_pg.C_Chr_04000077 ID=99882.3.0.229 annot-version=v3.0
Micromonas_sp._RCC299	OG0000030

50255 pacid=27395322 transcript=50255
locus=est_orfs.Chr_05_126_3199555:1 ID=50255.3.0.229 annot-version=v3.0 Micromonas_sp._RCC299 OG0000030
kfl00369_0050_v1.1 Klebsormidium_flaccidum OG0000030
kfl00398_0130_v1.1 Klebsormidium_flaccidum OG0000030
kfl00454_0010_v1.1 Klebsormidium_flaccidum OG0000030
kfl00479_0055_v1.1 Klebsormidium_flaccidum OG0000030
kfl00503_0040_v1.1 Klebsormidium_flaccidum OG0000030
kfl00547_0020_v1.1 Klebsormidium_flaccidum OG0000030
kfl01003_0010_v1.1 Klebsormidium_flaccidum OG0000030
kfl00004_0440_v1.1 Klebsormidium_flaccidum OG0000030
kfl00053_0180_v1.1 Klebsormidium_flaccidum OG0000030
kfl00113_0290_v1.1 Klebsormidium_flaccidum OG0000030
kfl00123_0130_v1.1 Klebsormidium_flaccidum OG0000030
kfl00144_0240_v1.1 Klebsormidium_flaccidum OG0000030
kfl00009_0310_v1.1 Klebsormidium_flaccidum OG0000030
kfl00190_0020_v1.1 Klebsormidium_flaccidum OG0000030
kfl00216_0150_v1.1 Klebsormidium_flaccidum OG0000030
kfl00254_0250_v1.1 Klebsormidium_flaccidum OG0000030
Pp3c22_20140V3.1.p pacid=32902937 transcript=Pp3c22_20140V3.1 locus=Pp3c22_20140 annot-version=v3.3 Physcomitrella_patens OG0000030
Pp3c2_8340V3.1.p pacid=32935843 transcript=Pp3c2_8340V3.1 locus=Pp3c2_8340 annot-version=v3.3 Physcomitrella_patens OG0000030
Pp3c2_14440V3.1.p pacid=32936347 transcript=Pp3c2_14440V3.1 locus=Pp3c2_14440 annot-version=v3.3 Physcomitrella_patens OG0000030
Pp3c2_6740V3.1.p pacid=32936514 transcript=Pp3c2_6740V3.1 locus=Pp3c2_6740 annot-version=v3.3 Physcomitrella_patens OG0000030
Pp3c2_6500V3.1.p pacid=32937334 transcript=Pp3c2_6500V3.1 locus=Pp3c2_6500 annot-version=v3.3 Physcomitrella_patens OG0000030
Pp3c3_7840V3.1.p pacid=32941256 transcript=Pp3c3_7840V3.1 locus=Pp3c3_7840 annot-version=v3.3 Physcomitrella_patens OG0000030
Pp3c3_35380V3.1.p pacid=32942168 transcript=Pp3c3_35380V3.1 locus=Pp3c3_35380 annot-version=v3.3 Physcomitrella_patens OG0000030
Pp3c3_17750V3.1.p pacid=32944234 transcript=Pp3c3_17750V3.1 locus=Pp3c3_17750 annot-version=v3.3 Physcomitrella_patens OG0000030
Pp3c22_20240V3.1.p pacid=32904657 transcript=Pp3c22_20240V3.1 locus=Pp3c22_20240 annot-version=v3.3 Physcomitrella_patens OG0000030
Pp3c5_15410V3.1.p pacid=32954357 transcript=Pp3c5_15410V3.1 locus=Pp3c5_15410 annot-version=v3.3 Physcomitrella_patens OG0000030
Pp3c11_1480V3.1.p pacid=32957813 transcript=Pp3c11_1480V3.1 locus=Pp3c11_1480 annot-version=v3.3 Physcomitrella_patens OG0000030
Pp3c14_19620V3.1.p pacid=32959925 transcript=Pp3c14_19620V3.1 locus=Pp3c14_19620 annot-version=v3.3 Physcomitrella_patens

OG0000030
Pp3c14_16130V3.1.p pacid=32961192 transcript=Pp3c14_16130V3.1
locus=Pp3c14_16130 annot-version=v3.3   Physcomitrella_patens
OG0000030
Pp3c14_17240V3.1.p pacid=32962710 transcript=Pp3c14_17240V3.1
locus=Pp3c14_17240 annot-version=v3.3   Physcomitrella_patens
OG0000030
Pp3c1_5560V3.1.p pacid=32969557 transcript=Pp3c1_5560V3.1
locus=Pp3c1_5560 annot-version=v3.3   Physcomitrella_patens
OG0000030
Pp3c1_29970V3.1.p pacid=32970152 transcript=Pp3c1_29970V3.1
locus=Pp3c1_29970 annot-version=v3.3   Physcomitrella_patens
OG0000030
Pp3c1_30010V3.1.p pacid=32971169 transcript=Pp3c1_30010V3.1
locus=Pp3c1_30010 annot-version=v3.3   Physcomitrella_patens
OG0000030
Pp3c12_16390V3.1.p pacid=32974908 transcript=Pp3c12_16390V3.1
locus=Pp3c12_16390 annot-version=v3.3   Physcomitrella_patens
OG0000030
Pp3c6_140V3.1.p pacid=32975541 transcript=Pp3c6_140V3.1
locus=Pp3c6_140 annot-version=v3.3   Physcomitrella_patens
OG0000030
Pp3c6_15330V3.1.p pacid=32976428 transcript=Pp3c6_15330V3.1
locus=Pp3c6_15330 annot-version=v3.3   Physcomitrella_patens
OG0000030
Pp3c6_24480V3.1.p pacid=32977987 transcript=Pp3c6_24480V3.1
locus=Pp3c6_24480 annot-version=v3.3   Physcomitrella_patens
OG0000030
Pp3c25_2840V3.1.p pacid=32979618 transcript=Pp3c25_2840V3.1
locus=Pp3c25_2840 annot-version=v3.3   Physcomitrella_patens
OG0000030
Pp3c25_4460V3.1.p pacid=32980858 transcript=Pp3c25_4460V3.1
locus=Pp3c25_4460 annot-version=v3.3   Physcomitrella_patens
OG0000030
Pp3c16_11220V3.1.p pacid=32985057 transcript=Pp3c16_11220V3.1
locus=Pp3c16_11220 annot-version=v3.3   Physcomitrella_patens
OG0000030
Pp3c16_12540V3.1.p pacid=32985568 transcript=Pp3c16_12540V3.1
locus=Pp3c16_12540 annot-version=v3.3   Physcomitrella_patens
OG0000030
Pp3c26_8080V3.1.p pacid=32918137 transcript=Pp3c26_8080V3.1
locus=Pp3c26_8080 annot-version=v3.3   Physcomitrella_patens
OG0000030
Pp3c26_3030V3.1.p pacid=32918571 transcript=Pp3c26_3030V3.1
locus=Pp3c26_3030 annot-version=v3.3   Physcomitrella_patens
OG0000030
Pp3c4_26380V3.1.p pacid=32918865 transcript=Pp3c4_26380V3.1
locus=Pp3c4_26380 annot-version=v3.3   Physcomitrella_patens
OG0000030
Pp3c4_17130V3.1.p pacid=32922836 transcript=Pp3c4_17130V3.1
locus=Pp3c4_17130 annot-version=v3.3   Physcomitrella_patens
OG0000030
AT2G35100.1 | Exostosin family protein | Chr2:14793993-14795564
REVERSE LENGTH=447 | 201606   Arabidopsis_thaliana

OG0000030
AT3G03650.1 | Exostosin family protein | Chr3:884298-886166 REVERSE LENGTH=499 | 201606   Arabidopsis_thaliana   OG0000030
AT3G07620.1 | glycosyltransferase | Chr3:2433267-2434988 REVERSE LENGTH=459 | 201606   Arabidopsis_thaliana   OG0000030
AT3G42180.1 | Exostosin family protein | Chr3:14324300-14328644 REVERSE LENGTH=470 | 201606   Arabidopsis_thaliana
OG0000030
AT3G45400.1 | exostosin family protein | Chr3:16651963-16653479 FORWARD LENGTH=475 | 201606   Arabidopsis_thaliana
OG0000030
AT3G57630.1 | exostosin family protein | Chr3:21339543-21343080 REVERSE LENGTH=793 | 201606   Arabidopsis_thaliana
OG0000030
AT4G16745.1 | Exostosin family protein | Chr4:9412185-9414053 FORWARD LENGTH=542 | 201606   Arabidopsis_thaliana
OG0000030
AT4G32790.1 | Exostosin family protein | Chr4:15812566-15814908 FORWARD LENGTH=593 | 201606   Arabidopsis_thaliana
OG0000030
AT4G38040.1 | Exostosin family protein | Chr4:17867501-17869131 FORWARD LENGTH=425 | 201606   Arabidopsis_thaliana
OG0000030
AT5G03795.1 | Exostosin family protein | Chr5:1007554-1010373 REVERSE LENGTH=518 | 201606   Arabidopsis_thaliana
OG0000030
AT1G21480.1 | Exostosin family protein | Chr1:7519116-7521426 REVERSE LENGTH=462 | 201606   Arabidopsis_thaliana
OG0000030
AT5G11130.1 | Exostosin family protein | Chr5:3542320-3544724 FORWARD LENGTH=480 | 201606   Arabidopsis_thaliana
OG0000030
AT5G11610.1 | Exostosin family protein | Chr5:3735569-3737952 REVERSE LENGTH=546 | 201606   Arabidopsis_thaliana
OG0000030
AT5G16890.1 | Exostosin family protein | Chr5:5551664-5554741 FORWARD LENGTH=511 | 201606   Arabidopsis_thaliana
OG0000030
AT5G19670.1 | Exostosin family protein | Chr5:6647025-6649358 FORWARD LENGTH=610 | 201606   Arabidopsis_thaliana
OG0000030
AT5G20260.1 | Exostosin family protein | Chr5:6836806-6837887 REVERSE LENGTH=334 | 201606   Arabidopsis_thaliana
OG0000030
AT5G22940.1 | glucuronoxylan glucuronosyltransferase%2C putative | Chr5:7677197-7678892 FORWARD LENGTH=469 | 201606
Arabidopsis_thaliana   OG0000030
AT5G25310.1 | Exostosin family protein | Chr5:8784820-8787235 FORWARD LENGTH=480 | 201606   Arabidopsis_thaliana
OG0000030
AT5G25820.1 | Exostosin family protein | Chr5:8997248-8999574 REVERSE LENGTH=654 | 201606   Arabidopsis_thaliana
OG0000030
AT5G33290.1 | xylogalacturonan deficient 1 | Chr5:12558439-12561840

FORWARD LENGTH=500 | 201606 Arabidopsis_thaliana
OG0000030
AT5G37000.1 | glycosyltransferase family exostosin protein |
Chr5:14618107-14620298 FORWARD LENGTH=554 | 201606
Arabidopsis_thaliana OG0000030
AT5G44930.1 | Exostosin family protein | Chr5:18140626-18142144
REVERSE LENGTH=443 | 201606 Arabidopsis_thaliana
OG0000030
AT5G61840.1 | Exostosin family protein | Chr5:24839365-24841703
REVERSE LENGTH=415 | 201606 Arabidopsis_thaliana
OG0000030
AT1G27440.1 | Exostosin family protein | Chr1:9529265-9531213
REVERSE LENGTH=412 | 201606 Arabidopsis_thaliana
OG0000030
AT1G34270.1 | Exostosin family protein | Chr1:12492571-12494514
REVERSE LENGTH=477 | 201606 Arabidopsis_thaliana
OG0000030
AT1G67410.1 | Exostosin family protein | Chr1:25251606-25253568
REVERSE LENGTH=430 | 201606 Arabidopsis_thaliana
OG0000030
AT1G74680.1 | Exostosin family protein | Chr1:28059528-28060984
FORWARD LENGTH=461 | 201606 Arabidopsis_thaliana
OG0000030
AT2G28110.1 | Exostosin family protein | Chr2:11978024-11979641
REVERSE LENGTH=448 | 201606 Arabidopsis_thaliana
OG0000030
Os03t0324700-01 Exostosin-like family protein. Oryza_sativa
OG0000030
Os04t0109900-00 Similar to H0201G08.5 protein. Oryza_sativa
OG0000030
Os04t0398600-01 Similar to Pectin-glucuronyltransferase.
Oryza_sativa OG0000030
Os04t0670600-01 Similar to H0624F09.5 protein. Oryza_sativa
OG0000030
Os06t0176100-00 Similar to H0201G08.5 protein. Oryza_sativa
OG0000030
Os06t0177200-00 Similar to H0201G08.5 protein. Oryza_sativa
OG0000030
Os06t0638300-01 Similar to Exostosin-like. Oryza_sativa
OG0000030
Os07t0188700-01 Similar to EXO. Oryza_sativa OG0000030
Os07t0567000-00 Similar to Exostosin-like. Oryza_sativa
OG0000030
Os01t0640600-01 Similar to LIMONENE cyclase like protein.
Oryza_sativa OG0000030
Os08t0438600-01 Exostosin-like family protein. Oryza_sativa
OG0000030
Os10t0180000-01 NpGUT1 homolog. Oryza_sativa OG0000030
Os11t0128000-01 Similar to secondary cell wall-related
glycosyltransferase family 47. Oryza_sativa OG0000030
Os12t0124400-01 Exostosin-like family protein. Oryza_sativa
OG0000030
Os01t0811400-01 Similar to predicted protein. Oryza_sativa
OG0000030

Os01t0921300-01 Exostosin-like family protein.	Oryza_sativa
OG0000030
Os01t0926400-01 Similar to Pectin-glucuronyltransferase.
Oryza_sativa	OG0000030
Os01t0926600-01 Similar to Pectin-glucuronyltransferase.
Oryza_sativa	OG0000030
Os01t0926700-00 Similar to secondary cell wall-related
glycosyltransferase family 47.	Oryza_sativa	OG0000030
Os02t0187200-01 Exostosin-like family protein.	Oryza_sativa
OG0000030
Os01t0107700-01 Similar to LIMONENE cyclase like protein.
Oryza_sativa	OG0000030
Os02t0520750-01 Similar to H0209H04.3 protein.	Oryza_sativa
OG0000030
Os02t0613100-00 Hypothetical conserved gene.	Oryza_sativa
OG0000030
Os03t0107900-01 Exostosin-like family protein.	Oryza_sativa
OG0000030
Os03t0174300-01 Exostosin-like family protein.	Oryza_sativa
OG0000030
CDF35110 pep supercontig:ASM35022v2:HG001718:197907:198911:1
gene:CHC_T00009549001 transcript:CDF35110
gene_biotype:protein_coding transcript_biotype:protein_coding
description:Glycosyltransferase, family GT47	Chondrus_crispus
OG0000030
CDF36359 pep supercontig:ASM35022v2:HG001774:40319:41632:-1
gene:CHC_T00009415001 transcript:CDF36359
gene_biotype:protein_coding transcript_biotype:protein_coding
description:Glycosyltransferase, family GT47	Chondrus_crispus
OG0000030
CDF40746 pep supercontig:ASM35022v2:HG002236:311297:312915:1
gene:CHC_T00008920001 transcript:CDF40746
gene_biotype:protein_coding transcript_biotype:protein_coding
description:Glycosyltransferase, family GT47	Chondrus_crispus
OG0000030
g3302.t1 Caulerpa_lentillifera	OG0000031
g802.t1 Caulerpa_lentillifera	OG0000031
Cre04.g228400.t1.1 pacid=30791391 transcript=Cre04.g228400.t1.1
locus=Cre04.g228400 ID=Cre04.g228400.t1.1.v5.5 annot-version=v5.5
Chlamydomonas_reinhardtii OG0000031
Vocar.0001s1791.1.p pacid=32884943 transcript=Vocar.0001s1791.1
locus=Vocar.0001s1791 ID=Vocar.0001s1791.1.v2.1 annot-version=v2.1
Volvox_carteri	OG0000031
jgi|ChlNC64A_1|23935|e_gw1.12.292.1	Chlorella_variabilis
OG0000031
8449 pacid=27389648 transcript=8449 locus=gw1.1.725.1
ID=8449.2.0.227 annot-version=v2.0	Coccomyxa_subellipsoidea
OG0000031
12928_fgenesh1_pg.C_Chr_11.0001000193	Ostreococcus_tauri
OG0000031
34068_0700010421 Ostreococcus_tauri	OG0000031
26580 pacid=27420570 transcript=26580 locus=eugene.1100010253
ID=26580.2.0.231 annot-version=v2.0	Ostreococcus_lucimarinus
OG0000031

62997 pacid=27340604 transcript=62997 locus=MicpuC2.est_orfs.
10_1980_4270394:1 ID=62997.3.0.228 annot-version=v3.0
Micromonas_pusilla	OG0000031
50253 pacid=27345333 transcript=50253 locus=MicpuC2.EuGene.
0000010477 ID=50253.3.0.228 annot-version=v3.0
Micromonas_pusilla	OG0000031
63124 pacid=27399912 transcript=63124 locus=EuGene.1200010296
ID=63124.3.0.229 annot-version=v3.0	Micromonas_sp._RCC299
OG0000031
kfl00096_0150_v1.1	Klebsormidium_flaccidum	OG0000031
kfl00189_0180_v1.1	Klebsormidium_flaccidum	OG0000031
Pp3c7_24490V3.1.p pacid=32926335 transcript=Pp3c7_24490V3.1
locus=Pp3c7_24490 annot-version=v3.3	Physcomitrella_patens
OG0000031
Pp3c13_15520V3.1.p pacid=32929967 transcript=Pp3c13_15520V3.1
locus=Pp3c13_15520 annot-version=v3.3	Physcomitrella_patens
OG0000031
Pp3c13_10830V3.1.p pacid=32932429 transcript=Pp3c13_10830V3.1
locus=Pp3c13_10830 annot-version=v3.3	Physcomitrella_patens
OG0000031
Pp3c2_32150V3.1.p pacid=32934982 transcript=Pp3c2_32150V3.1
locus=Pp3c2_32150 annot-version=v3.3	Physcomitrella_patens
OG0000031
Pp3c2_32160V3.1.p pacid=32935437 transcript=Pp3c2_32160V3.1
locus=Pp3c2_32160 annot-version=v3.3	Physcomitrella_patens
OG0000031
Pp3c2_23590V3.1.p pacid=32936005 transcript=Pp3c2_23590V3.1
locus=Pp3c2_23590 annot-version=v3.3	Physcomitrella_patens
OG0000031
Pp3c2_2510V3.1.p pacid=32936964 transcript=Pp3c2_2510V3.1
locus=Pp3c2_2510 annot-version=v3.3	Physcomitrella_patens
OG0000031
Pp3c2_9700V3.1.p pacid=32937304 transcript=Pp3c2_9700V3.1
locus=Pp3c2_9700 annot-version=v3.3	Physcomitrella_patens
OG0000031
Pp3c19_3000V3.1.p pacid=32938118 transcript=Pp3c19_3000V3.1
locus=Pp3c19_3000 annot-version=v3.3	Physcomitrella_patens
OG0000031
Pp3c22_12880V3.1.p pacid=32903467 transcript=Pp3c22_12880V3.1
locus=Pp3c22_12880 annot-version=v3.3	Physcomitrella_patens
OG0000031
Pp3c3_15040V3.1.p pacid=32940875 transcript=Pp3c3_15040V3.1
locus=Pp3c3_15040 annot-version=v3.3	Physcomitrella_patens
OG0000031
Pp3c3_21440V3.1.p pacid=32940958 transcript=Pp3c3_21440V3.1
locus=Pp3c3_21440 annot-version=v3.3	Physcomitrella_patens
OG0000031
Pp3c3_8830V3.1.p pacid=32941596 transcript=Pp3c3_8830V3.1
locus=Pp3c3_8830 annot-version=v3.3	Physcomitrella_patens
OG0000031
Pp3c3_27940V3.1.p pacid=32941999 transcript=Pp3c3_27940V3.1
locus=Pp3c3_27940 annot-version=v3.3	Physcomitrella_patens
OG0000031
Pp3c3_32860V3.1.p pacid=32944844 transcript=Pp3c3_32860V3.1

locus=Pp3c3_32860 annot-version=v3.3 Physcomitrella_patens
OG0000031
Pp3c20_12200V3.1.p pacid=32945393 transcript=Pp3c20_12200V3.1
locus=Pp3c20_12200 annot-version=v3.3 Physcomitrella_patens
OG0000031
Pp3c23_10680V3.1.p pacid=32950926 transcript=Pp3c23_10680V3.1
locus=Pp3c23_10680 annot-version=v3.3 Physcomitrella_patens
OG0000031
Pp3c11_1840V3.1.p pacid=32956236 transcript=Pp3c11_1840V3.1
locus=Pp3c11_1840 annot-version=v3.3 Physcomitrella_patens
OG0000031
Pp3c11_1790V3.1.p pacid=32956640 transcript=Pp3c11_1790V3.1
locus=Pp3c11_1790 annot-version=v3.3 Physcomitrella_patens
OG0000031
Pp3c11_14240V3.1.p pacid=32958720 transcript=Pp3c11_14240V3.1
locus=Pp3c11_14240 annot-version=v3.3 Physcomitrella_patens
OG0000031
Pp3c17_19970V3.1.p pacid=32905689 transcript=Pp3c17_19970V3.1
locus=Pp3c17_19970 annot-version=v3.3 Physcomitrella_patens
OG0000031
Pp3c14_17020V3.1.p pacid=32962005 transcript=Pp3c14_17020V3.1
locus=Pp3c14_17020 annot-version=v3.3 Physcomitrella_patens
OG0000031
Pp3c8_5110V3.1.p pacid=32964054 transcript=Pp3c8_5110V3.1
locus=Pp3c8_5110 annot-version=v3.3 Physcomitrella_patens
OG0000031
Pp3c8_4400V3.1.p pacid=32966312 transcript=Pp3c8_4400V3.1
locus=Pp3c8_4400 annot-version=v3.3 Physcomitrella_patens
OG0000031
Pp3c1_40230V3.1.p pacid=32966738 transcript=Pp3c1_40230V3.1
locus=Pp3c1_40230 annot-version=v3.3 Physcomitrella_patens
OG0000031
Pp3c12_4260V3.1.p pacid=32973836 transcript=Pp3c12_4260V3.1
locus=Pp3c12_4260 annot-version=v3.3 Physcomitrella_patens
OG0000031
Pp3c24_7120V3.1.p pacid=32910096 transcript=Pp3c24_7120V3.1
locus=Pp3c24_7120 annot-version=v3.3 Physcomitrella_patens
OG0000031
Pp3c21_11270V3.1.p pacid=32914573 transcript=Pp3c21_11270V3.1
locus=Pp3c21_11270 annot-version=v3.3 Physcomitrella_patens
OG0000031
Pp3c26_5090V3.1.p pacid=32917712 transcript=Pp3c26_5090V3.1
locus=Pp3c26_5090 annot-version=v3.3 Physcomitrella_patens
OG0000031
Pp3c26_5095V3.1.p pacid=32918418 transcript=Pp3c26_5095V3.1
locus=Pp3c26_5095 annot-version=v3.3 Physcomitrella_patens
OG0000031
Pp3c4_15559V3.1.p pacid=32918934 transcript=Pp3c4_15559V3.1
locus=Pp3c4_15559 annot-version=v3.3 Physcomitrella_patens
OG0000031
Pp3c4_26880V3.1.p pacid=32922126 transcript=Pp3c4_26880V3.1
locus=Pp3c4_26880 annot-version=v3.3 Physcomitrella_patens
OG0000031
Pp3c7_7550V3.1.p pacid=32924338 transcript=Pp3c7_7550V3.1

locus=Pp3c7_7550 annot-version=v3.3	Physcomitrella_patens
OG0000031
AT2G34830.1 | WRKY DNA-binding protein 35 | Chr2:14693839-14696378
REVERSE LENGTH=427 | 201606	Arabidopsis_thaliana
OG0000031
AT2G37260.1 | WRKY family transcription factor family protein |
Chr2:15645277-15646792 FORWARD LENGTH=429 | 201606
Arabidopsis_thaliana	OG0000031
AT2G38470.1 | WRKY DNA-binding protein 33 | Chr2:16108476-16110539
FORWARD LENGTH=519 | 201606	Arabidopsis_thaliana
OG0000031
AT2G40740.1 | WRKY DNA-binding protein 55 | Chr2:16997177-16999276
FORWARD LENGTH=292 | 201606	Arabidopsis_thaliana
OG0000031
AT2G40750.1 | WRKY DNA-binding protein 54 | Chr2:17000636-17002354
REVERSE LENGTH=346 | 201606	Arabidopsis_thaliana
OG0000031
AT2G44745.1 | WRKY family transcription factor |
Chr2:18447482-18449004 REVERSE LENGTH=218 | 201606
Arabidopsis_thaliana	OG0000031
AT2G46130.1 | WRKY DNA-binding protein 43 | Chr2:18957254-18957669
FORWARD LENGTH=109 | 201606	Arabidopsis_thaliana
OG0000031
AT2G46400.1 | WRKY DNA-binding protein 46 | Chr2:19043676-19044754
REVERSE LENGTH=295 | 201606	Arabidopsis_thaliana
OG0000031
AT2G47260.1 | WRKY DNA-binding protein 23 | Chr2:19405045-19406446
REVERSE LENGTH=337 | 201606	Arabidopsis_thaliana
OG0000031
AT3G01080.1 | WRKY DNA-binding protein 58 | Chr3:25507-27449 FORWARD
LENGTH=423 | 201606	Arabidopsis_thaliana	OG0000031
AT3G01970.1 | WRKY DNA-binding protein 45 | Chr3:326614-327159
REVERSE LENGTH=147 | 201606	Arabidopsis_thaliana
OG0000031
AT3G04670.1 | WRKY DNA-binding protein 39 | Chr3:1266530-1267691
REVERSE LENGTH=330 | 201606	Arabidopsis_thaliana
OG0000031
AT1G13960.1 | WRKY DNA-binding protein 4 | Chr1:4776622-4779043
FORWARD LENGTH=514 | 201606	Arabidopsis_thaliana
OG0000031
AT3G56400.1 | WRKY DNA-binding protein 70 | Chr3:20909082-20910409
REVERSE LENGTH=294 | 201606	Arabidopsis_thaliana
OG0000031
AT3G58710.1 | WRKY DNA-binding protein 69 | Chr3:21715192-21716766
FORWARD LENGTH=272 | 201606	Arabidopsis_thaliana
OG0000031
AT3G62340.1 | WRKY family transcription factor |
Chr3:23069628-23070634 REVERSE LENGTH=277 | 201606
Arabidopsis_thaliana	OG0000031
AT4G01250.1 | WRKY family transcription factor | Chr4:522839-524129
REVERSE LENGTH=298 | 201606	Arabidopsis_thaliana
OG0000031
AT4G01720.1 | WRKY family transcription factor | Chr4:744949-748219
FORWARD LENGTH=489 | 201606	Arabidopsis_thaliana

OG0000031
AT4G04450.1 | WRKY family transcription factor |
Chr4:2218379-2221113 FORWARD LENGTH=528 | 201606
Arabidopsis_thaliana   OG0000031
AT4G11070.1 | WRKY family transcription factor |
Chr4:6759303-6760763 FORWARD LENGTH=313 | 201606
Arabidopsis_thaliana   OG0000031
AT4G18170.1 | WRKY DNA-binding protein 28 | Chr4:10061508-10062691
FORWARD LENGTH=318 | 201606   Arabidopsis_thaliana
OG0000031
AT1G18860.1 | WRKY DNA-binding protein 61 | Chr1:6508907-6511209
FORWARD LENGTH=528 | 201606   Arabidopsis_thaliana
OG0000031
AT4G22070.1 | WRKY DNA-binding protein 31 | Chr4:11691381-11694234
REVERSE LENGTH=538 | 201606   Arabidopsis_thaliana
OG0000031
AT4G23550.1 | WRKY family transcription factor |
Chr4:12291813-12293088 FORWARD LENGTH=310 | 201606
Arabidopsis_thaliana   OG0000031
AT4G23810.1 | WRKY family transcription factor |
Chr4:12392666-12393739 REVERSE LENGTH=324 | 201606
Arabidopsis_thaliana   OG0000031
AT4G24240.1 | WRKY DNA-binding protein 7 | Chr4:12571930-12573446
FORWARD LENGTH=353 | 201606   Arabidopsis_thaliana
OG0000031
AT4G26440.1 | WRKY DNA-binding protein 34 | Chr4:13357596-13359550
REVERSE LENGTH=568 | 201606   Arabidopsis_thaliana
OG0000031
AT4G26640.1 | WRKY family transcription factor family protein |
Chr4:13437298-13439774 REVERSE LENGTH=485 | 201606
Arabidopsis_thaliana   OG0000031
AT4G30935.1 | WRKY DNA-binding protein 32 | Chr4:15051916-15053908
REVERSE LENGTH=466 | 201606   Arabidopsis_thaliana
OG0000031
AT4G31550.1 | WRKY DNA-binding protein 11 | Chr4:15290065-15291458
REVERSE LENGTH=325 | 201606   Arabidopsis_thaliana
OG0000031
AT4G31800.1 | WRKY DNA-binding protein 18 | Chr4:15383296-15384812
FORWARD LENGTH=310 | 201606   Arabidopsis_thaliana
OG0000031
AT4G39410.1 | WRKY DNA-binding protein 13 | Chr4:18332937-18334789
REVERSE LENGTH=304 | 201606   Arabidopsis_thaliana
OG0000031
AT5G01900.1 | WRKY DNA-binding protein 62 | Chr5:351136-352013
REVERSE LENGTH=263 | 201606   Arabidopsis_thaliana
OG0000031
AT5G07100.1 | WRKY DNA-binding protein 26 | Chr5:2204313-2205647
FORWARD LENGTH=309 | 201606   Arabidopsis_thaliana
OG0000031
AT5G13080.1 | WRKY DNA-binding protein 75 | Chr5:4149928-4151019
REVERSE LENGTH=145 | 201606   Arabidopsis_thaliana
OG0000031
AT5G15130.1 | WRKY DNA-binding protein 72 | Chr5:4904426-4906879
FORWARD LENGTH=548 | 201606   Arabidopsis_thaliana

OG0000031
AT5G22570.1 | WRKY DNA-binding protein 38 | Chr5:7495608-7496707
REVERSE LENGTH=289 | 201606	Arabidopsis_thaliana
OG0000031
AT5G24110.1 | WRKY DNA-binding protein 30 | Chr5:8153416-8154633
REVERSE LENGTH=303 | 201606	Arabidopsis_thaliana
OG0000031
AT5G26170.1 | WRKY DNA-binding protein 50 | Chr5:9147176-9148128
REVERSE LENGTH=173 | 201606	Arabidopsis_thaliana
OG0000031
AT5G28650.1 | WRKY DNA-binding protein 74 | Chr5:10677790-10678945
REVERSE LENGTH=330 | 201606	Arabidopsis_thaliana
OG0000031
AT5G41570.1 | WRKY DNA-binding protein 24 | Chr5:16624220-16625689
FORWARD LENGTH=179 | 201606	Arabidopsis_thaliana
OG0000031
AT5G43290.1 | WRKY DNA-binding protein 49 | Chr5:17371987-17373083
REVERSE LENGTH=274 | 201606	Arabidopsis_thaliana
OG0000031
AT5G46350.1 | WRKY DNA-binding protein 8 | Chr5:18801403-18803901
REVERSE LENGTH=326 | 201606	Arabidopsis_thaliana
OG0000031
AT5G49520.1 | WRKY DNA-binding protein 48 | Chr5:20090890-20092867
FORWARD LENGTH=399 | 201606	Arabidopsis_thaliana
OG0000031
AT5G52830.1 | WRKY DNA-binding protein 27 | Chr5:21410996-21412218
FORWARD LENGTH=348 | 201606	Arabidopsis_thaliana
OG0000031
AT5G56270.1 | WRKY DNA-binding protein 2 | Chr5:22780816-22783137
FORWARD LENGTH=687 | 201606	Arabidopsis_thaliana
OG0000031
AT5G64810.1 | WRKY DNA-binding protein 51 | Chr5:25908415-25909687
FORWARD LENGTH=194 | 201606	Arabidopsis_thaliana
OG0000031
AT1G29280.1 | WRKY DNA-binding protein 65 | Chr1:10236589-10237467
FORWARD LENGTH=259 | 201606	Arabidopsis_thaliana
OG0000031
AT1G29860.1 | WRKY DNA-binding protein 71 | Chr1:10454482-10455652
FORWARD LENGTH=282 | 201606	Arabidopsis_thaliana
OG0000031
AT1G30650.1 | WRKY DNA-binding protein 14 | Chr1:10868612-10870973
FORWARD LENGTH=430 | 201606	Arabidopsis_thaliana
OG0000031
AT1G55600.1 | WRKY DNA-binding protein 10 | Chr1:20774049-20776293
REVERSE LENGTH=485 | 201606	Arabidopsis_thaliana
OG0000031
AT1G62300.1 | WRKY family transcription factor |
Chr1:23016887-23019155 REVERSE LENGTH=553 | 201606
Arabidopsis_thaliana	OG0000031
AT1G64000.1 | WRKY DNA-binding protein 56 | Chr1:23747434-23748883
FORWARD LENGTH=195 | 201606	Arabidopsis_thaliana
OG0000031
AT1G66550.1 | WRKY DNA-binding protein 67 | Chr1:24828537-24829589
FORWARD LENGTH=254 | 201606	Arabidopsis_thaliana

OG0000031
AT1G66560.1 | WRKY DNA-binding protein 64 | Chr1:24833579-24834631
FORWARD LENGTH=249 | 201606   Arabidopsis_thaliana
OG0000031
AT1G66600.1 | ABA overly sensitive mutant 3 | Chr1:24848406-24849335
FORWARD LENGTH=241 | 201606   Arabidopsis_thaliana
OG0000031
AT1G68150.1 | WRKY DNA-binding protein 9 | Chr1:25543970-25545615
FORWARD LENGTH=374 | 201606   Arabidopsis_thaliana
OG0000031
AT1G69310.1 | WRKY DNA-binding protein 57 | Chr1:26056118-26057909
REVERSE LENGTH=287 | 201606   Arabidopsis_thaliana
OG0000031
AT1G69810.1 | WRKY DNA-binding protein 36 | Chr1:26277238-26279116
REVERSE LENGTH=387 | 201606   Arabidopsis_thaliana
OG0000031
AT1G80590.1 | WRKY DNA-binding protein 66 | Chr1:30296210-30297156
REVERSE LENGTH=235 | 201606   Arabidopsis_thaliana
OG0000031
AT1G80840.1 | WRKY DNA-binding protein 40 | Chr1:30383834-30385356
FORWARD LENGTH=302 | 201606   Arabidopsis_thaliana
OG0000031
AT2G03340.1 | WRKY DNA-binding protein 3 | Chr2:1014724-1016936
REVERSE LENGTH=513 | 201606   Arabidopsis_thaliana
OG0000031
AT2G04880.1 | zinc-dependent activator protein-1 |
Chr2:1718557-1720219 FORWARD LENGTH=487 | 201606
Arabidopsis_thaliana   OG0000031
AT2G21900.1 | WRKY DNA-binding protein 59 | Chr2:9334149-9336222
REVERSE LENGTH=202 | 201606   Arabidopsis_thaliana
OG0000031
AT2G23320.1 | WRKY DNA-binding protein 15 | Chr2:9924998-9926154
FORWARD LENGTH=317 | 201606   Arabidopsis_thaliana
OG0000031
AT2G24570.1 | WRKY DNA-binding protein 17 | Chr2:10437676-10439222
REVERSE LENGTH=321 | 201606   Arabidopsis_thaliana
OG0000031
AT2G25000.1 | WRKY DNA-binding protein 60 | Chr2:10629812-10631095
FORWARD LENGTH=271 | 201606   Arabidopsis_thaliana
OG0000031
AT2G30250.1 | WRKY DNA-binding protein 25 | Chr2:12903553-12905089
REVERSE LENGTH=393 | 201606   Arabidopsis_thaliana
OG0000031
AT2G30590.1 | WRKY DNA-binding protein 21 | Chr2:13033891-13035303
FORWARD LENGTH=380 | 201606   Arabidopsis_thaliana
OG0000031
Os03t0321700-01 Similar to WRKY transcription factor 55.
Oryza_sativa   OG0000031
Os03t0335200-01 Similar to WRKY1 (WRKY transcription factor 17).
Oryza_sativa   OG0000031
Os03t0444900-00 Similar to WRKY transcription factor 30.
Oryza_sativa   OG0000031
Os03t0657400-00 Hypothetical conserved gene.   Oryza_sativa
OG0000031

Os03t0741400-01 Similar to SUSIBA2.	Oryza_sativa	OG0000031
Os03t0758000-01 Similar to WRKY23 transcription factor (Fragment).	Oryza_sativa	OG0000031
Os03t0758950-01 Transcriptional activator, Defense responses to rice sheath blight	Oryza_sativa	OG0000031
Os03t0798500-01 WRKY transcription factor, Positive regulation of defense responses	Oryza_sativa	OG0000031
Os03t0855100-01 Similar to WRKY transcription factor 12.	Oryza_sativa	OG0000031
Os01t0289600-00 Similar to WRKY transcription factor 28.	Oryza_sativa	OG0000031
Os04t0287400-01 Similar to WRKY transcription factor 51.	Oryza_sativa	OG0000031
Os04t0471700-00 Similar to WRKY10 (WRKY transcription factor 35).	Oryza_sativa	OG0000031
Os04t0545000-01 Similar to WRKY transcription factor 34.	Oryza_sativa	OG0000031
Os04t0597300-01 Similar to WERKY protein (Fragment).	Oryza_sativa	OG0000031
Os04t0605100-01 WRKY transcription factor 68.	Oryza_sativa	OG0000031
Os05t0129800-01 DNA-binding WRKY domain containing protein.	Oryza_sativa	OG0000031
Os05t0137500-00 Similar to WRKY transcription factor 5.	Oryza_sativa	OG0000031
Os05t0183100-01 Similar to WRKY transcription factor 16 (Fragment).	Oryza_sativa	OG0000031
Os05t0321900-01 DNA-binding WRKY domain containing protein.	Oryza_sativa	OG0000031
Os05t0322900-01 WRKY transcription factor, Benzothiadiazole (BTH)-inducible blast resistance	Oryza_sativa	OG0000031
Os05t0343400-01 WRKY transcription factor, Disease resistance, Defense response	Oryza_sativa	OG0000031
Os05t0474800-01 WRKY transcription factor 70.	Oryza_sativa	OG0000031
Os05t0478400-00 Similar to WRKY transcription factor 48.	Oryza_sativa	OG0000031
Os05t0478700-00 Hypothetical conserved gene.	Oryza_sativa	OG0000031
Os05t0478800-00 Similar to WRKY transcription factor 48.	Oryza_sativa	OG0000031
Os05t0537100-00 WRKY transcription factor 10.	Oryza_sativa	OG0000031
Os05t0565900-00 DNA-binding WRKY domain containing protein.	Oryza_sativa	OG0000031
Os05t0567200-00 Similar to WRKY transcription factor 43.	Oryza_sativa	OG0000031
Os05t0571200-01 Similar to WRKY transcription factor 19.	Oryza_sativa	OG0000031
Os05t0583000-01 Similar to WRKY8.	Oryza_sativa	OG0000031
Os06t0146250-00 Similar to WRKY transcription factor 73.	Oryza_sativa	OG0000031
Os06t0158100-00 Similar to WRKY transcription factor 63.

Oryza_sativa	OG0000031
Os06t0504900-00 Similar to WRKY transcription factor 31.
Oryza_sativa	OG0000031
Os06t0649000-01 PAMP (pathogen-associated molecular pattern)-responsive transrepressor, Defense response	Oryza_sativa	OG0000031
Os01t0584900-01 WRKY transcription factor 28-like (WRKY5) (WRKY transcription factor 77).	Oryza_sativa	OG0000031
Os01t0586800-00 Similar to WRKY transcription factor 5 (Fragment).
Oryza_sativa	OG0000031
Os07t0111400-00 WRKY transcription factor 29.	Oryza_sativa	OG0000031
Os07t0583700-01 WRKY transcription factor 78.	Oryza_sativa	OG0000031
Os07t0596900-00 Similar to SUSIBA2-like (WRKY transcription factor 80).	Oryza_sativa	OG0000031
Os01t0624700-01 Similar to WRKY transcription factor 12.
Oryza_sativa	OG0000031
Os01t0626400-01 DNA-binding protein WRKY2-like (WRKY transcription factor 11) (WRKY16).	Oryza_sativa	OG0000031
Os07t0680400-01 WRKY transcription factor, Drought tolerance
Oryza_sativa	OG0000031
Os08t0235800-00 Similar to WRKY transcription factor 25.
Oryza_sativa	OG0000031
Os08t0276200-01 Similar to WRKY transcription factor 44 (WRKY DNA-binding protein 44) (TRANSPARENT TESTA GLABRA 2).	Oryza_sativa	OG0000031
Os08t0386200-01 WRKY transcription factor 69.	Oryza_sativa	OG0000031
Os01t0656400-00 Similar to WRKY transcription factor 15.
Oryza_sativa	OG0000031
Os08t0499300-01 WRKY transcription factor 30.	Oryza_sativa	OG0000031
Os09t0334500-01 WRKY transcription factor, Tolerance to phosphate starvation, Regulator of Fe starvation and cold stress
Oryza_sativa	OG0000031
Os09t0417600-01 WRKY transcription factor, Transcriptional repressor, Pathogen defense	Oryza_sativa	OG0000031
Os09t0417800-01 WRKY transcription factor, Transcriptional repressor, Pathogen defense	Oryza_sativa	OG0000031
Os09t0481700-01 SUSIBA2-like (WRKY transcription factor 80).
Oryza_sativa	OG0000031
Os01t0714800-01 WRKY transcription factor 26.	Oryza_sativa	OG0000031
Os11t0116900-01 Similar to WRKY DNA binding domain containing protein, expressed.	Oryza_sativa	OG0000031
Os11t0117400-00 Similar to WRKY DNA binding domain containing protein, expressed.	Oryza_sativa	OG0000031
Os11t0117500-01 DNA-binding WRKY domain containing protein.
Oryza_sativa	OG0000031
Os11t0117600-00 Similar to WRKY transcription factor 50 (Fragment).
Oryza_sativa	OG0000031
Os01t0730700-01 WRKY transcription factor 14 (WRKY14).
Oryza_sativa	OG0000031

Os01t0734000-01 Similar to WRKY DNA binding protein.
Oryza_sativa OG0000031
Os11t0490900-01 Similar to WRKY transcription factor 72.
Oryza_sativa OG0000031
Os01t0750100-01 Similar to WRKY transcription factor-like (WRKY transcription factor 13) (WRKY13). Oryza_sativa OG0000031
Os11t0685700-00 Similar to WRKY transcription factor 61.
Oryza_sativa OG0000031
Os12t0116400-00 Similar to WRKY DNA binding domain containing protein, expressed. Oryza_sativa OG0000031
Os12t0116600-01 Similar to Transcription factor (Fragment).
Oryza_sativa OG0000031
Os12t0116700-01 Similar to WRKY transcription factor 64.
Oryza_sativa OG0000031
Os12t0507300-00 Similar to WRKY DNA binding domain containing protein, expressed. Oryza_sativa OG0000031
Os12t0597700-01 Similar to WRKY2 (Fragment). Oryza_sativa OG0000031
Os01t0820400-00 Similar to WRKY22 - superfamily of TFs having WRKY and zinc finger domains. Oryza_sativa OG0000031
Os01t0820700-00 DNA-binding WRKY domain containing protein.
Oryza_sativa OG0000031
Os01t0821600-01 WRKY transcription factor 48-like protein (WRKY transcription factor 21). Oryza_sativa OG0000031
Os01t0826400-01 WRKY transcription factor 24 (WRKY24).
Oryza_sativa OG0000031
Os01t0842801-00 Similar to WRKY transcription factor 56.
Oryza_sativa OG0000031
Os01t0972800-01 WRKY1 (WRKY transcription factor 17).
Oryza_sativa OG0000031
Os02t0181300-01 WRKY transcription factor, Defense response
Oryza_sativa OG0000031
Os02t0265200-01 WRKY transcription factor 39. Oryza_sativa OG0000031
Os02t0462800-01 WRKY transcription factor 42, Transcriptional repressor, Promotion of leaf senescence Oryza_sativa OG0000031
Os01t0182700-01 Similar to WRKY12. Oryza_sativa OG0000031
Os01t0185900-01 Similar to WRKY 1 (Fragment). Oryza_sativa OG0000031
Os01t0186000-01 Similar to WRKY transcription factor 10.
Oryza_sativa OG0000031
Os02t0652100-01 Similar to WRKY-like DNA-binding protein.
Oryza_sativa OG0000031
Os02t0698800-01 WRKY transcription factor 66. Oryza_sativa OG0000031
Os02t0770500-00 Similar to WRKY transcription factor 32.
Oryza_sativa OG0000031
g1474.t1 Caulerpa_lentillifera OG0000032
g5956.t1 Caulerpa_lentillifera OG0000032
g6001.t1 Caulerpa_lentillifera OG0000032
g6739.t1 Caulerpa_lentillifera OG0000032

g6769.t1 Caulerpa_lentillifera OG0000032
g6889.t1 Caulerpa_lentillifera OG0000032
Cre02.g142800.t1.2 pacid=30785747 transcript=Cre02.g142800.t1.2 locus=Cre02.g142800 ID=Cre02.g142800.t1.2.v5.5 annot-version=v5.5 Chlamydomonas_reinhardtii OG0000032
Cre01.g027450.t1.2 pacid=30788389 transcript=Cre01.g027450.t1.2 locus=Cre01.g027450 ID=Cre01.g027450.t1.2.v5.5 annot-version=v5.5 Chlamydomonas_reinhardtii OG0000032
Cre01.g066552.t1.1 pacid=30789368 transcript=Cre01.g066552.t1.1 locus=Cre01.g066552 ID=Cre01.g066552.t1.1.v5.5 annot-version=v5.5 Chlamydomonas_reinhardtii OG0000032
Cre01.g052250.t1.2 pacid=30789580 transcript=Cre01.g052250.t1.2 locus=Cre01.g052250 ID=Cre01.g052250.t1.2.v5.5 annot-version=v5.5 Chlamydomonas_reinhardtii OG0000032
Cre10.g446100.t1.2 pacid=30789951 transcript=Cre10.g446100.t1.2 locus=Cre10.g446100 ID=Cre10.g446100.t1.2.v5.5 annot-version=v5.5 Chlamydomonas_reinhardtii OG0000032
Cre12.g554850.t1.2 pacid=30792428 transcript=Cre12.g554850.t1.2 locus=Cre12.g554850 ID=Cre12.g554850.t1.2.v5.5 annot-version=v5.5 Chlamydomonas_reinhardtii OG0000032
Cre12.g496700.t1.2 pacid=30792698 transcript=Cre12.g496700.t1.2 locus=Cre12.g496700 ID=Cre12.g496700.t1.2.v5.5 annot-version=v5.5 Chlamydomonas_reinhardtii OG0000032
Cre09.g391900.t1.1 pacid=30780233 transcript=Cre09.g391900.t1.1 locus=Cre09.g391900 ID=Cre09.g391900.t1.1.v5.5 annot-version=v5.5 Chlamydomonas_reinhardtii OG0000032
Cre05.g243050.t1.2 pacid=30783290 transcript=Cre05.g243050.t1.2 locus=Cre05.g243050 ID=Cre05.g243050.t1.2.v5.5 annot-version=v5.5 Chlamydomonas_reinhardtii OG0000032
Cre05.g248500.t1.2 pacid=30783293 transcript=Cre05.g248500.t1.2 locus=Cre05.g248500 ID=Cre05.g248500.t1.2.v5.5 annot-version=v5.5 Chlamydomonas_reinhardtii OG0000032
Vocar.0054s0027.1.p pacid=32894666 transcript=Vocar.0054s0027.1 locus=Vocar.0054s0027 ID=Vocar.0054s0027.1.v2.1 annot-version=v2.1 Volvox_carteri OG0000032
Vocar.0008s0449.1.p pacid=32897200 transcript=Vocar.0008s0449.1 locus=Vocar.0008s0449 ID=Vocar.0008s0449.1.v2.1 annot-version=v2.1 Volvox_carteri OG0000032
Vocar.0001s0395.1.p pacid=32884921 transcript=Vocar.0001s0395.1 locus=Vocar.0001s0395 ID=Vocar.0001s0395.1.v2.1 annot-version=v2.1 Volvox_carteri OG0000032
Vocar.0001s0884.1.p pacid=32885685 transcript=Vocar.0001s0884.1 locus=Vocar.0001s0884 ID=Vocar.0001s0884.1.v2.1 annot-version=v2.1 Volvox_carteri OG0000032
Vocar.0001s0480.1.p pacid=32885809 transcript=Vocar.0001s0480.1 locus=Vocar.0001s0480 ID=Vocar.0001s0480.1.v2.1 annot-version=v2.1 Volvox_carteri OG0000032
Vocar.0007s0363.1.p pacid=32887878 transcript=Vocar.0007s0363.1 locus=Vocar.0007s0363 ID=Vocar.0007s0363.1.v2.1 annot-version=v2.1 Volvox_carteri OG0000032
Vocar.0004s0109.1.p pacid=32889838 transcript=Vocar.0004s0109.1 locus=Vocar.0004s0109 ID=Vocar.0004s0109.1.v2.1 annot-version=v2.1 Volvox_carteri OG0000032
Vocar.0073s0025.1.p pacid=32891657 transcript=Vocar.0073s0025.1

locus=Vocar.0073s0025 ID=Vocar.0073s0025.1.v2.1 annot-version=v2.1
Volvox_carteri   OG0000032
Vocar.0026s0003.1.p pacid=32892430 transcript=Vocar.0026s0003.1
locus=Vocar.0026s0003 ID=Vocar.0026s0003.1.v2.1 annot-version=v2.1
Volvox_carteri   OG0000032
Vocar.0009s0283.1.p pacid=32893335 transcript=Vocar.0009s0283.1
locus=Vocar.0009s0283 ID=Vocar.0009s0283.1.v2.1 annot-version=v2.1
Volvox_carteri   OG0000032
Vocar.0006s0391.1.p pacid=32894152 transcript=Vocar.0006s0391.1
locus=Vocar.0006s0391 ID=Vocar.0006s0391.1.v2.1 annot-version=v2.1
Volvox_carteri   OG0000032
jgi|ChlNC64A_1|133836|IGS.gm_10_00127   Chlorella_variabilis
OG0000032
jgi|ChlNC64A_1|52344|fgenesh3_pg.C_scaffold_10000129
Chlorella_variabilis   OG0000032
jgi|ChlNC64A_1|33111|estExt_Genewise1.C_300034
Chlorella_variabilis   OG0000032
jgi|ChlNC64A_1|15244|gw1.6.319.1 Chlorella_variabilis
OG0000032
jgi|ChlNC64A_1|20868|e_gw1.5.457.1   Chlorella_variabilis
OG0000032
jgi|ChlNC64A_1|26868|e_gw1.22.159.1   Chlorella_variabilis
OG0000032
jgi|ChlNC64A_1|15163|gw1.25.162.1 Chlorella_variabilis
OG0000032
jgi|ChlNC64A_1|36198|estExt_Genewise1Plus.C_150012
Chlorella_variabilis   OG0000032
jgi|ChlNC64A_1|14388|gw1.15.208.1 Chlorella_variabilis
OG0000032
63276 pacid=27386797 transcript=63276 locus=Genemark1.4059_g
ID=63276.2.0.227 annot-version=v2.0   Coccomyxa_subellipsoidea
OG0000032
53444 pacid=27386854 transcript=53444
locus=estExt_fgenesh1_pm.C_70178 ID=53444.2.0.227 annot-version=v2.0
        Coccomyxa_subellipsoidea OG0000032
33087 pacid=27386858 transcript=33087 locus=fgenesh1_kg.
7_#_42_#_2107_1_CBOZ_CBPA ID=33087.2.0.227 annot-version=v2.0
Coccomyxa_subellipsoidea OG0000032
67188 pacid=27387941 transcript=67188
locus=estExt_Genemark1.C_140069 ID=67188.2.0.227 annot-version=v2.0
Coccomyxa_subellipsoidea OG0000032
19702 pacid=27388449 transcript=19702 locus=e_gw1.19.302.1
ID=19702.2.0.227 annot-version=v2.0   Coccomyxa_subellipsoidea
OG0000032
9567 pacid=27390406 transcript=9567 locus=gw1.3.540.1
ID=9567.2.0.227 annot-version=v2.0   Coccomyxa_subellipsoidea
OG0000032
46495 pacid=27390865 transcript=46495
locus=estExt_fgenesh1_pg.C_30317 ID=46495.2.0.227 annot-version=v2.0
        Coccomyxa_subellipsoidea OG0000032
54744 pacid=27392828 transcript=54744
locus=estExt_fgenesh1_pm.C_200042 ID=54744.2.0.227 annot-
version=v2.0   Coccomyxa_subellipsoidea OG0000032
11832 pacid=27393570 transcript=11832 locus=e_gw1.2.720.1

ID=11832.2.0.227 annot-version=v2.0	Coccomyxa_subellipsoidea
OG0000032
47343 pacid=27386555 transcript=47343
locus=estExt_fgenesh1_pg.C_70165 ID=47343.2.0.227 annot-version=v2.0
	Coccomyxa_subellipsoidea OG0000032
15098_e_gw1.01.00.680.1	Ostreococcus_tauri	OG0000032
16234_e_gw1.03.00.567.1	Ostreococcus_tauri	OG0000032
17215_e_gw1.05.00.532.1	Ostreococcus_tauri	OG0000032
19652_e_gw1.10.00.417.1	Ostreococcus_tauri	OG0000032
24215_estExt_fgenesh1_pg.C_Chr_11.00010006 Ostreococcus_tauri
OG0000032
33125_0500010490 Ostreococcus_tauri	OG0000032
7139_gw1.05.00.489.1	Ostreococcus_tauri	OG0000032
7641_gw1.03.00.557.1	Ostreococcus_tauri	OG0000032
7669_gw1.05.00.529.1	Ostreococcus_tauri	OG0000032
7672_gw1.10.00.420.1	Ostreococcus_tauri	OG0000032
7739_gw1.17.00.275.1	Ostreococcus_tauri	OG0000032
7775_gw1.03.00.565.1	Ostreococcus_tauri	OG0000032
7831_gw1.04.00.532.1	Ostreococcus_tauri	OG0000032
37354 pacid=27415514 transcript=37354 locus=e_gwEuk.4.545.1
ID=37354.2.0.231 annot-version=v2.0	Ostreococcus_lucimarinus
OG0000032
37753 pacid=27415871 transcript=37753 locus=e_gwEuk.16.359.1
ID=37753.2.0.231 annot-version=v2.0	Ostreococcus_lucimarinus
OG0000032
26095 pacid=27416032 transcript=26095 locus=eugene.1000010129
ID=26095.2.0.231 annot-version=v2.0	Ostreococcus_lucimarinus
OG0000032
10652 pacid=27413204 transcript=10652 locus=gwEuk.3.816.1
ID=10652.2.0.231 annot-version=v2.0	Ostreococcus_lucimarinus
OG0000032
9654 pacid=27416356 transcript=9654 locus=gwEuk.10.483.1
ID=9654.2.0.231 annot-version=v2.0	Ostreococcus_lucimarinus
OG0000032
9279 pacid=27416504 transcript=9279 locus=gwEuk.5.552.1
ID=9279.2.0.231 annot-version=v2.0	Ostreococcus_lucimarinus
OG0000032
9748 pacid=27416543 transcript=9748 locus=gwEuk.5.592.1
ID=9748.2.0.231 annot-version=v2.0	Ostreococcus_lucimarinus
OG0000032
31565 pacid=27416592 transcript=31565 locus=eugene.0500010102
ID=31565.2.0.231 annot-version=v2.0	Ostreococcus_lucimarinus
OG0000032
9576 pacid=27417950 transcript=9576 locus=gwEuk.1.825.1
ID=9576.2.0.231 annot-version=v2.0	Ostreococcus_lucimarinus
OG0000032
9423 pacid=27420093 transcript=9423 locus=gwEuk.20.470.1
ID=9423.2.0.231 annot-version=v2.0	Ostreococcus_lucimarinus
OG0000032
26335 pacid=27420440 transcript=26335 locus=eugene.1100010008
ID=26335.2.0.231 annot-version=v2.0	Ostreococcus_lucimarinus
OG0000032
52353 pacid=27349024 transcript=52353 locus=MicpuC2.EuGene.
0000120418 ID=52353.3.0.228 annot-version=v3.0

Micromonas_pusilla   OG0000032
34621 pacid=27338790 transcript=34621
locus=MicpuC2.estExt_Genewise1Plus.C_80316 ID=34621.3.0.228 annot-
version=v3.0   Micromonas_pusilla   OG0000032
127462 pacid=27340213 transcript=127502
locus=estExt_fgenesh2_kg.C_100014 ID=127502.3.0.228 annot-
version=v3.0   Micromonas_pusilla   OG0000032
157920 pacid=27340711 transcript=157960 locus=e_gw1.7.490.1
ID=157960.3.0.228 annot-version=v3.0   Micromonas_pusilla
OG0000032
9085 pacid=27341104 transcript=9085 locus=MicpuC2.gw1.7.482.1
ID=9085.3.0.228 annot-version=v3.0   Micromonas_pusilla
OG0000032
64553 pacid=27342473 transcript=64553 locus=MicpuC2.est_orfs.
2_3536_4275462:19 ID=64553.3.0.228 annot-version=v3.0
Micromonas_pusilla   OG0000032
60314 pacid=27339084 transcript=60314 locus=MicpuC2.EuGene.
0000080508 ID=60314.3.0.228 annot-version=v3.0
Micromonas_pusilla   OG0000032
142158 pacid=27343850 transcript=142198 locus=gw1.18.218.1
ID=142198.3.0.228 annot-version=v3.0   Micromonas_pusilla
OG0000032
19725 pacid=27344437 transcript=19725 locus=MicpuC2.e_gw1.9.675.1
ID=19725.3.0.228 annot-version=v3.0   Micromonas_pusilla
OG0000032
50406 pacid=27345136 transcript=50406 locus=MicpuC2.EuGene.
0000010630 ID=50406.3.0.228 annot-version=v3.0
Micromonas_pusilla   OG0000032
50029 pacid=27345259 transcript=50029 locus=MicpuC2.EuGene.
0000010253 ID=50029.3.0.228 annot-version=v3.0
Micromonas_pusilla   OG0000032
32838 pacid=27346192 transcript=32838
locus=MicpuC2.estExt_Genewise1Plus.C_40215 ID=32838.3.0.228 annot-
version=v3.0   Micromonas_pusilla   OG0000032
146153 pacid=27346323 transcript=146193 locus=gw1.4.1450.1
ID=146193.3.0.228 annot-version=v3.0   Micromonas_pusilla
OG0000032
152885 pacid=27347201 transcript=152925 locus=e_gw1.3.109.1
ID=152925.3.0.228 annot-version=v3.0   Micromonas_pusilla
OG0000032
77096 pacid=27397185 transcript=77096 locus=gw2.16.363.1
ID=77096.3.0.229 annot-version=v3.0   Micromonas_sp._RCC299
OG0000032
80239 pacid=27398246 transcript=80239 locus=e_gw2.03.738.1
ID=80239.3.0.229 annot-version=v3.0   Micromonas_sp._RCC299
OG0000032
55177 pacid=27398865 transcript=55177 locus=EuGene.0100010404
ID=55177.3.0.229 annot-version=v3.0   Micromonas_sp._RCC299
OG0000032
62922 pacid=27399994 transcript=62922 locus=EuGene.1200010094
ID=62922.3.0.229 annot-version=v3.0   Micromonas_sp._RCC299
OG0000032
58107 pacid=27400274 transcript=58107 locus=EuGene.0400010636
ID=58107.3.0.229 annot-version=v3.0   Micromonas_sp._RCC299

OG0000032
105474 pacid=27400754 transcript=105474
locus=estExt_fgenesh2_pg.C_Chr_040370 ID=105474.3.0.229 annot-version=v3.0 Micromonas_sp._RCC299 OG0000032
61493 pacid=27401400 transcript=61493 locus=EuGene.0900010476
ID=61493.3.0.229 annot-version=v3.0 Micromonas_sp._RCC299
OG0000032
95021 pacid=27401612 transcript=95021
locus=estExt_Genewise2Plus.C_Chr_090179 ID=95021.3.0.229 annot-version=v3.0 Micromonas_sp._RCC299 OG0000032
82546 pacid=27402417 transcript=82546 locus=e_gw2.06.910.1
ID=82546.3.0.229 annot-version=v3.0 Micromonas_sp._RCC299
OG0000032
74289 pacid=27402509 transcript=74289 locus=gw2.13.447.1
ID=74289.3.0.229 annot-version=v3.0 Micromonas_sp._RCC299
OG0000032
63937 pacid=27403825 transcript=63937 locus=EuGene.1400010072
ID=63937.3.0.229 annot-version=v3.0 Micromonas_sp._RCC299
OG0000032
59955 pacid=27404292 transcript=59955 locus=EuGene.0700010240
ID=59955.3.0.229 annot-version=v3.0 Micromonas_sp._RCC299
OG0000032
92004 pacid=27405071 transcript=92004
locus=estExt_Genewise2.C_Chr_110338 ID=92004.3.0.229 annot-version=v3.0 Micromonas_sp._RCC299 OG0000032
kfl00369_0040_v1.1 Klebsormidium_flaccidum OG0000032
kfl00573_0030_v1.1 Klebsormidium_flaccidum OG0000032
kfl00654_0030_v1.1 Klebsormidium_flaccidum OG0000032
kfl00839_0050_v1.1 Klebsormidium_flaccidum OG0000032
kfl00859_0010_v1.1 Klebsormidium_flaccidum OG0000032
kfl00003_0040_v1.1 Klebsormidium_flaccidum OG0000032
kfl00046_0060_v1.1 Klebsormidium_flaccidum OG0000032
kfl00085_0260_v1.1 Klebsormidium_flaccidum OG0000032
kfl00160_0130_v1.1 Klebsormidium_flaccidum OG0000032
kfl00192_0150_v1.1 Klebsormidium_flaccidum OG0000032
kfl00237_0110_v1.1 Klebsormidium_flaccidum OG0000032
kfl00263_0170_v1.1 Klebsormidium_flaccidum OG0000032
Pp3c7_2920V3.1.p pacid=32926147 transcript=Pp3c7_2920V3.1
locus=Pp3c7_2920 annot-version=v3.3 Physcomitrella_patens
OG0000032
Pp3c15_21760V3.1.p pacid=32929336 transcript=Pp3c15_21760V3.1
locus=Pp3c15_21760 annot-version=v3.3 Physcomitrella_patens
OG0000032
Pp3c22_9100V3.1.p pacid=32903164 transcript=Pp3c22_9100V3.1
locus=Pp3c22_9100 annot-version=v3.3 Physcomitrella_patens
OG0000032
Pp3c2_11410V3.1.p pacid=32936745 transcript=Pp3c2_11410V3.1
locus=Pp3c2_11410 annot-version=v3.3 Physcomitrella_patens
OG0000032
Pp3c19_1800V3.1.p pacid=32938974 transcript=Pp3c19_1800V3.1
locus=Pp3c19_1800 annot-version=v3.3 Physcomitrella_patens
OG0000032
Pp3c5_23070V3.1.p pacid=32954110 transcript=Pp3c5_23070V3.1
locus=Pp3c5_23070 annot-version=v3.3 Physcomitrella_patens

OG0000032
Pp3c8_8140V3.1.p pacid=32966043 transcript=Pp3c8_8140V3.1
locus=Pp3c8_8140 annot-version=v3.3 Physcomitrella_patens
OG0000032
Pp3c1_18440V3.1.p pacid=32969590 transcript=Pp3c1_18440V3.1
locus=Pp3c1_18440 annot-version=v3.3 Physcomitrella_patens
OG0000032
Pp3c1_28390V3.1.p pacid=32971597 transcript=Pp3c1_28390V3.1
locus=Pp3c1_28390 annot-version=v3.3 Physcomitrella_patens
OG0000032
Pp3c6_10090V3.1.p pacid=32975378 transcript=Pp3c6_10090V3.1
locus=Pp3c6_10090 annot-version=v3.3 Physcomitrella_patens
OG0000032
Pp3c6_10040V3.1.p pacid=32977930 transcript=Pp3c6_10040V3.1
locus=Pp3c6_10040 annot-version=v3.3 Physcomitrella_patens
OG0000032
Pp3c25_2880V3.1.p pacid=32979223 transcript=Pp3c25_2880V3.1
locus=Pp3c25_2880 annot-version=v3.3 Physcomitrella_patens
OG0000032
Pp3c17_13510V3.1.p pacid=32907906 transcript=Pp3c17_13510V3.1
locus=Pp3c17_13510 annot-version=v3.3 Physcomitrella_patens
OG0000032
Pp3c16_11171V3.1.p pacid=32984424 transcript=Pp3c16_11171V3.1
locus=Pp3c16_11171 annot-version=v3.3 Physcomitrella_patens
OG0000032
Pp3c16_11180V3.1.p pacid=32985410 transcript=Pp3c16_11180V3.1
locus=Pp3c16_11180 annot-version=v3.3 Physcomitrella_patens
OG0000032
Pp3c16_11170V3.1.p pacid=32986700 transcript=Pp3c16_11170V3.1
locus=Pp3c16_11170 annot-version=v3.3 Physcomitrella_patens
OG0000032
Pp3c24_4980V3.1.p pacid=32910453 transcript=Pp3c24_4980V3.1
locus=Pp3c24_4980 annot-version=v3.3 Physcomitrella_patens
OG0000032
Pp3c9_9220V3.1.p pacid=32912794 transcript=Pp3c9_9220V3.1
locus=Pp3c9_9220 annot-version=v3.3 Physcomitrella_patens
OG0000032
Pp3c9_20570V3.1.p pacid=32913870 transcript=Pp3c9_20570V3.1
locus=Pp3c9_20570 annot-version=v3.3 Physcomitrella_patens
OG0000032
Pp3c26_10440V3.1.p pacid=32917848 transcript=Pp3c26_10440V3.1
locus=Pp3c26_10440 annot-version=v3.3 Physcomitrella_patens
OG0000032
Pp3c4_3890V3.1.p pacid=32920830 transcript=Pp3c4_3890V3.1
locus=Pp3c4_3890 annot-version=v3.3 Physcomitrella_patens
OG0000032
Pp3c4_27660V3.1.p pacid=32920924 transcript=Pp3c4_27660V3.1
locus=Pp3c4_27660 annot-version=v3.3 Physcomitrella_patens
OG0000032
AT2G35010.1 | thioredoxin O1 | Chr2:14754398-14755888 FORWARD
LENGTH=194 | 201606 Arabidopsis_thaliana OG0000032
AT2G40790.1 | C-terminal cysteine residue is changed to a serine 2 |
Chr2:17023109-17023915 REVERSE LENGTH=154 | 201606
Arabidopsis_thaliana OG0000032

AT1G11530.1 | C-terminal cysteine residue is changed to a serine 1 |
Chr1:3874518-3875311 FORWARD LENGTH=118 | 201606
Arabidopsis_thaliana OG0000032
AT3G02730.1 | thioredoxin F-type 1 | Chr3:588570-589591 REVERSE
LENGTH=178 | 201606 Arabidopsis_thaliana OG0000032
AT3G06730.1 | Thioredoxin z | Chr3:2124276-2125845 FORWARD
LENGTH=183 | 201606 Arabidopsis_thaliana OG0000032
AT3G08710.1 | thioredoxin H-type 9 | Chr3:2645590-2646304 FORWARD
LENGTH=140 | 201606 Arabidopsis_thaliana OG0000032
AT3G15360.1 | thioredoxin M-type 4 | Chr3:5188448-5189457 FORWARD
LENGTH=193 | 201606 Arabidopsis_thaliana OG0000032
AT3G51030.1 | thioredoxin H-type 1 | Chr3:18951123-18951955 REVERSE
LENGTH=114 | 201606 Arabidopsis_thaliana OG0000032
AT3G53220.1 | Thioredoxin superfamily protein |
Chr3:19722032-19722615 FORWARD LENGTH=126 | 201606
Arabidopsis_thaliana OG0000032
AT3G56420.1 | Thioredoxin superfamily protein |
Chr3:20922140-20922890 REVERSE LENGTH=154 | 201606
Arabidopsis_thaliana OG0000032
AT4G03520.1 | Thioredoxin superfamily protein | Chr4:1562585-1564055
REVERSE LENGTH=186 | 201606 Arabidopsis_thaliana
OG0000032
AT1G19730.1 | Thioredoxin superfamily protein | Chr1:6823163-6824020
REVERSE LENGTH=119 | 201606 Arabidopsis_thaliana
OG0000032
AT5G16400.1 | thioredoxin F2 | Chr5:5363905-5365249 REVERSE
LENGTH=185 | 201606 Arabidopsis_thaliana OG0000032
AT5G39950.1 | thioredoxin 2 | Chr5:15990885-15991881 REVERSE
LENGTH=133 | 201606 Arabidopsis_thaliana OG0000032
AT5G42980.1 | thioredoxin 3 | Chr5:17242772-17243718 FORWARD
LENGTH=118 | 201606 Arabidopsis_thaliana OG0000032
AT1G03680.1 | thioredoxin M-type 1 | Chr1:916990-917865 REVERSE
LENGTH=179 | 201606 Arabidopsis_thaliana OG0000032
AT1G31020.1 | thioredoxin O2 | Chr1:11057252-11058703 FORWARD
LENGTH=159 | 201606 Arabidopsis_thaliana OG0000032
AT1G43560.1 | thioredoxin Y2 | Chr1:16398359-16399828 REVERSE
LENGTH=167 | 201606 Arabidopsis_thaliana OG0000032
AT1G45145.1 | thioredoxin H-type 5 | Chr1:17075264-17076256 REVERSE
LENGTH=118 | 201606 Arabidopsis_thaliana OG0000032
AT1G50320.1 | thioredoxin X | Chr1:18638606-18639464 REVERSE
LENGTH=182 | 201606 Arabidopsis_thaliana OG0000032
AT1G59730.1 | thioredoxin H-type 7 | Chr1:21952759-21953392 REVERSE
LENGTH=129 | 201606 Arabidopsis_thaliana OG0000032
AT1G69880.1 | thioredoxin H-type 8 | Chr1:26321540-26322794 FORWARD
LENGTH=148 | 201606 Arabidopsis_thaliana OG0000032
AT1G76760.1 | thioredoxin Y1 | Chr1:28811873-28812948 REVERSE
LENGTH=172 | 201606 Arabidopsis_thaliana OG0000032
AT2G15570.1 | Thioredoxin superfamily protein | Chr2:6791556-6792902
REVERSE LENGTH=173 | 201606 Arabidopsis_thaliana
OG0000032
Os03t0800700-01 Similar to Thioredoxin H-type 5 (TRX-H-5).
Oryza_sativa OG0000032
Os04t0430800-01 Similar to OSIGBa0160I14.3 protein.
Oryza_sativa OG0000032

Os04t0530600-01 Similar to Thioredoxin 1 (TRX-1) (Thioredoxin M).
Oryza_sativa OG0000032
Os04t0560200-01 Thioredoxin domain 2 containing protein.
Oryza_sativa OG0000032
Os04t0629500-01 Similar to Thioredoxin h. Oryza_sativa
OG0000032
Os04t0676100-01 Similar to Thioredoxin X, chloroplast precursor.
Oryza_sativa OG0000032
Os05t0169000-01 Similar to Thioredoxin-like protein.
Oryza_sativa OG0000032
Os05t0480200-01 Thioredoxin domain 2 containing protein.
Oryza_sativa OG0000032
Os06t0665900-00 Similar to thioredoxin domain-containing protein 2.
Oryza_sativa OG0000032
Os07t0186000-02 H-type thioredoxin, Regulation of the apoplastic reactive oxygen species, Stress response Oryza_sativa
OG0000032
Os07t0190800-01 Similar to Thioredoxin h. Oryza_sativa
OG0000032
Os01t0616300-01 Thioredoxin, core domain containing protein.
Oryza_sativa OG0000032
Os08t0378900-01 Similar to Thioredoxin protein. Oryza_sativa
OG0000032
Os09t0559600-01 Thioredoxin domain 2 containing protein.
Oryza_sativa OG0000032
Os12t0188700-01 Similar to Thioredoxin M5, chloroplastic.
Oryza_sativa OG0000032
Os12t0280050-00 Similar to Thioredoxin family protein.
Oryza_sativa OG0000032
Os01t0913000-01 Similar to Thioredoxin F-type 2, chloroplast precursor (TRX-F2). Oryza_sativa OG0000032
Os01t0963400-01 Thioredoxin family protein. Oryza_sativa
OG0000032
Os01t0168200-01 Similar to Thioredoxin-like protein.
Oryza_sativa OG0000032
Os02t0639900-01 Similar to Thioredoxin M-type, chloroplast precursor (TRX-M). Oryza_sativa OG0000032
CMQ093CT pep chromosome:ASM9120v1:17:214438:214963:-1 gene:CMQ093C transcript:CMQ093CT gene_biotype:protein_coding transcript_biotype:protein_coding Cyanidioschyzon_merolae
OG0000032
CMM285CT pep chromosome:ASM9120v1:13:717304:718660:-1 gene:CMM285C transcript:CMM285CT gene_biotype:protein_coding transcript_biotype:protein_coding Cyanidioschyzon_merolae
OG0000032
CMS271CT pep chromosome:ASM9120v1:19:682433:683104:1 gene:CMS271C transcript:CMS271CT gene_biotype:protein_coding transcript_biotype:protein_coding Cyanidioschyzon_merolae
OG0000032
CMN279CT pep chromosome:ASM9120v1:14:693664:694240:-1 gene:CMN279C transcript:CMN279CT gene_biotype:protein_coding transcript_biotype:protein_coding Cyanidioschyzon_merolae
OG0000032
CMJ096CT pep chromosome:ASM9120v1:10:287729:288047:-1 gene:CMJ096C

transcript:CMJ096CT gene_biotype:protein_coding
transcript_biotype:protein_coding Cyanidioschyzon_merolae
OG0000032
evm.model.contig_2494.6 Porphyridium_purpureum OG0000032
evm.model.contig_3401.6 Porphyridium_purpureum OG0000032
evm.model.contig_2033.19 Porphyridium_purpureum OG0000032
evm.model.contig_3583.4 Porphyridium_purpureum OG0000032
evm.model.contig_3835.2 Porphyridium_purpureum OG0000032
evm.model.contig_4408.17 Porphyridium_purpureum OG0000032
evm.model.contig_4415.8 Porphyridium_purpureum OG0000032
evm.model.contig_4415.7 Porphyridium_purpureum OG0000032
evm.model.contig_596.2 Porphyridium_purpureum OG0000032
evm.model.contig_2075.4 Porphyridium_purpureum OG0000032
CDF38295 pep supercontig:ASM35022v2:HG001914:365125:365784:1
gene:CHC_T00006120001 transcript:CDF38295
gene_biotype:protein_coding transcript_biotype:protein_coding
Chondrus_crispus OG0000032
CDF33615 pep supercontig:ASM35022v2:HG001651:73046:75269:-1
gene:CHC_T00002151001 transcript:CDF33615
gene_biotype:protein_coding transcript_biotype:protein_coding
Chondrus_crispus OG0000032
CDF38652 pep supercontig:ASM35022v2:HG001949:342480:343302:-1
gene:CHC_T00008576001 transcript:CDF38652
gene_biotype:protein_coding transcript_biotype:protein_coding
description:Thioredoxin TRX Chondrus_crispus OG0000032
CDF37297 pep supercontig:ASM35022v2:HG001830:5609:7690:-1
gene:CHC_T00010277001 transcript:CDF37297
gene_biotype:protein_coding transcript_biotype:protein_coding
description:thioredoxin domain containing protein Chondrus_crispus
OG0000032
CDF37727 pep supercontig:ASM35022v2:HG001864:70178:71298:1
gene:CHC_T00005936001 transcript:CDF37727
gene_biotype:protein_coding transcript_biotype:protein_coding
Chondrus_crispus OG0000032
Cre01.g026500.t1.1 pacid=30788666 transcript=Cre01.g026500.t1.1
locus=Cre01.g026500 ID=Cre01.g026500.t1.1.v5.5 annot-version=v5.5
Chlamydomonas_reinhardtii OG0000033
Cre01.g051950.t1.2 pacid=30789426 transcript=Cre01.g051950.t1.2
locus=Cre01.g051950 ID=Cre01.g051950.t1.2.v5.5 annot-version=v5.5
Chlamydomonas_reinhardtii OG0000033
Vocar.0001s1012.1.p pacid=32884890 transcript=Vocar.0001s1012.1
locus=Vocar.0001s1012 ID=Vocar.0001s1012.1.v2.1 annot-version=v2.1
Volvox_carteri OG0000033
Vocar.0001s0618.1.p pacid=32885864 transcript=Vocar.0001s0618.1
locus=Vocar.0001s0618 ID=Vocar.0001s0618.1.v2.1 annot-version=v2.1
Volvox_carteri OG0000033
jgi|ChlNC64A_1|59135|estExt_fgenesh3_pg.C_260065
Chlorella_variabilis OG0000033
46347 pacid=27390488 transcript=46347
locus=estExt_fgenesh1_pg.C_30061 ID=46347.2.0.227 annot-version=v2.0
Coccomyxa_subellipsoidea OG0000033
35965_1200010263 Ostreococcus_tauri OG0000033
37724_1800010128 Ostreococcus_tauri OG0000033
16121 pacid=27414106 transcript=16121

locus=fgenesh1_pg.C_Chr_7000165 ID=16121.2.0.231 annot-version=v2.0
Ostreococcus_lucimarinus OG0000033
26236 pacid=27416318 transcript=26236 locus=eugene.1000010270
ID=26236.2.0.231 annot-version=v2.0 Ostreococcus_lucimarinus
OG0000033
28323 pacid=27419102 transcript=28323 locus=eugene.1700010116
ID=28323.2.0.231 annot-version=v2.0 Ostreococcus_lucimarinus
OG0000033
17358 pacid=27341842 transcript=17358 locus=MicpuC2.e_gw1.5.840.1
ID=17358.3.0.228 annot-version=v3.0 Micromonas_pusilla
OG0000033
59495 pacid=27401863 transcript=59495 locus=EuGene.0600010497
ID=59495.3.0.229 annot-version=v3.0 Micromonas_sp._RCC299
OG0000033
kfl00337_0080_v1.1 Klebsormidium_flaccidum OG0000033
kfl00479_0070_v1.1 Klebsormidium_flaccidum OG0000033
kfl01542_0010_v1.1 Klebsormidium_flaccidum OG0000033
kfl00042_0240_v1.1 Klebsormidium_flaccidum OG0000033
kfl00054_0260_v1.1 Klebsormidium_flaccidum OG0000033
kfl00009_0120_v1.1 Klebsormidium_flaccidum OG0000033
kfl00155_0120_v1.1 Klebsormidium_flaccidum OG0000033
kfl00162_0010_v1.1 Klebsormidium_flaccidum OG0000033
kfl00270_0090_v1.1 Klebsormidium_flaccidum OG0000033
Pp3c15_4820V3.1.p pacid=32927789 transcript=Pp3c15_4820V3.1
locus=Pp3c15_4820 annot-version=v3.3 Physcomitrella_patens
OG0000033
Pp3c15_8690V3.1.p pacid=32929509 transcript=Pp3c15_8690V3.1
locus=Pp3c15_8690 annot-version=v3.3 Physcomitrella_patens
OG0000033
Pp3c13_14230V3.1.p pacid=32931457 transcript=Pp3c13_14230V3.1
locus=Pp3c13_14230 annot-version=v3.3 Physcomitrella_patens
OG0000033
Pp3c2_21330V3.1.p pacid=32933361 transcript=Pp3c2_21330V3.1
locus=Pp3c2_21330 annot-version=v3.3 Physcomitrella_patens
OG0000033
Pp3c2_30100V3.1.p pacid=32934778 transcript=Pp3c2_30100V3.1
locus=Pp3c2_30100 annot-version=v3.3 Physcomitrella_patens
OG0000033
Pp3c19_10740V3.1.p pacid=32939413 transcript=Pp3c19_10740V3.1
locus=Pp3c19_10740 annot-version=v3.3 Physcomitrella_patens
OG0000033
Pp3c3_11110V3.1.p pacid=32942183 transcript=Pp3c3_11110V3.1
locus=Pp3c3_11110 annot-version=v3.3 Physcomitrella_patens
OG0000033
Pp3c3_28440V3.1.p pacid=32945031 transcript=Pp3c3_28440V3.1
locus=Pp3c3_28440 annot-version=v3.3 Physcomitrella_patens
OG0000033
Pp3c3_17600V3.1.p pacid=32945062 transcript=Pp3c3_17600V3.1
locus=Pp3c3_17600 annot-version=v3.3 Physcomitrella_patens
OG0000033
Pp3c22_10540V3.1.p pacid=32904884 transcript=Pp3c22_10540V3.1
locus=Pp3c22_10540 annot-version=v3.3 Physcomitrella_patens
OG0000033
Pp3c22_22040V3.1.p pacid=32905042 transcript=Pp3c22_22040V3.1

locus=Pp3c22_22040 annot-version=v3.3 Physcomitrella_patens
OG0000033
Pp3c5_12070V3.1.p pacid=32954941 transcript=Pp3c5_12070V3.1
locus=Pp3c5_12070 annot-version=v3.3 Physcomitrella_patens
OG0000033
Pp3c11_6660V3.1.p pacid=32956548 transcript=Pp3c11_6660V3.1
locus=Pp3c11_6660 annot-version=v3.3 Physcomitrella_patens
OG0000033
Pp3c14_14440V3.1.p pacid=32962879 transcript=Pp3c14_14440V3.1
locus=Pp3c14_14440 annot-version=v3.3 Physcomitrella_patens
OG0000033
Pp3c17_6290V3.1.p pacid=32905846 transcript=Pp3c17_6290V3.1
locus=Pp3c17_6290 annot-version=v3.3 Physcomitrella_patens
OG0000033
Pp3c8_11860V3.1.p pacid=32966500 transcript=Pp3c8_11860V3.1
locus=Pp3c8_11860 annot-version=v3.3 Physcomitrella_patens
OG0000033
Pp3c1_6950V3.1.p pacid=32971001 transcript=Pp3c1_6950V3.1
locus=Pp3c1_6950 annot-version=v3.3 Physcomitrella_patens
OG0000033
Pp3c1_6450V3.1.p pacid=32971741 transcript=Pp3c1_6450V3.1
locus=Pp3c1_6450 annot-version=v3.3 Physcomitrella_patens
OG0000033
Pp3c25_200V3.1.p pacid=32980756 transcript=Pp3c25_200V3.1
locus=Pp3c25_200 annot-version=v3.3 Physcomitrella_patens
OG0000033
Pp3c18_3870V3.1.p pacid=32981223 transcript=Pp3c18_3870V3.1
locus=Pp3c18_3870 annot-version=v3.3 Physcomitrella_patens
OG0000033
Pp3c24_5970V3.1.p pacid=32909209 transcript=Pp3c24_5970V3.1
locus=Pp3c24_5970 annot-version=v3.3 Physcomitrella_patens
OG0000033
Pp3c9_9590V3.1.p pacid=32911477 transcript=Pp3c9_9590V3.1
locus=Pp3c9_9590 annot-version=v3.3 Physcomitrella_patens
OG0000033
Pp3c9_490V3.1.p pacid=32913160 transcript=Pp3c9_490V3.1
locus=Pp3c9_490 annot-version=v3.3 Physcomitrella_patens
OG0000033
Pp3c21_12290V3.1.p pacid=32915506 transcript=Pp3c21_12290V3.1
locus=Pp3c21_12290 annot-version=v3.3 Physcomitrella_patens
OG0000033
Pp3c26_9050V3.1.p pacid=32917556 transcript=Pp3c26_9050V3.1
locus=Pp3c26_9050 annot-version=v3.3 Physcomitrella_patens
OG0000033
Pp3c26_9350V3.1.p pacid=32917747 transcript=Pp3c26_9350V3.1
locus=Pp3c26_9350 annot-version=v3.3 Physcomitrella_patens
OG0000033
Pp3c26_9030V3.1.p pacid=32918349 transcript=Pp3c26_9030V3.1
locus=Pp3c26_9030 annot-version=v3.3 Physcomitrella_patens
OG0000033
Pp3c7_23300V3.1.p pacid=32924518 transcript=Pp3c7_23300V3.1
locus=Pp3c7_23300 annot-version=v3.3 Physcomitrella_patens
OG0000033
Pp3c7_25370V3.1.p pacid=32924829 transcript=Pp3c7_25370V3.1

locus=Pp3c7_25370 annot-version=v3.3	Physcomitrella_patens
OG0000033
AT2G35615.1 | Eukaryotic aspartyl protease family protein |
Chr2:14959391-14960734 FORWARD LENGTH=447 | 201606
Arabidopsis_thaliana	OG0000033
AT2G36670.1 | Eukaryotic aspartyl protease family protein |
Chr2:15364949-15368016 REVERSE LENGTH=512 | 201606
Arabidopsis_thaliana	OG0000033
AT2G39710.1 | Eukaryotic aspartyl protease family protein |
Chr2:16562051-16563379 REVERSE LENGTH=442 | 201606
Arabidopsis_thaliana	OG0000033
AT2G42980.1 | Eukaryotic aspartyl protease family protein |
Chr2:17875005-17876588 REVERSE LENGTH=527 | 201606
Arabidopsis_thaliana	OG0000033
AT3G02740.1 | Eukaryotic aspartyl protease family protein |
Chr3:590561-593089 FORWARD LENGTH=488 | 201606
Arabidopsis_thaliana	OG0000033
AT3G12700.1 | Eukaryotic aspartyl protease family protein |
Chr3:4037136-4039043 FORWARD LENGTH=461 | 201606
Arabidopsis_thaliana	OG0000033
AT3G18490.1 | Eukaryotic aspartyl protease family protein |
Chr3:6349090-6350592 REVERSE LENGTH=500 | 201606
Arabidopsis_thaliana	OG0000033
AT3G20015.1 | Eukaryotic aspartyl protease family protein |
Chr3:6978746-6980158 REVERSE LENGTH=470 | 201606
Arabidopsis_thaliana	OG0000033
AT3G25700.1 | Eukaryotic aspartyl protease family protein |
Chr3:9358937-9360295 FORWARD LENGTH=452 | 201606
Arabidopsis_thaliana	OG0000033
AT3G42550.1 | Eukaryotic aspartyl protease family protein |
Chr3:14665728-14669135 REVERSE LENGTH=430 | 201606
Arabidopsis_thaliana	OG0000033
AT3G50050.1 | Eukaryotic aspartyl protease family protein |
Chr3:18554138-18557115 REVERSE LENGTH=632 | 201606
Arabidopsis_thaliana	OG0000033
AT3G51330.1 | Eukaryotic aspartyl protease family protein |
Chr3:19053480-19056152 REVERSE LENGTH=529 | 201606
Arabidopsis_thaliana	OG0000033
AT3G51340.1 | Eukaryotic aspartyl protease family protein |
Chr3:19057013-19059788 REVERSE LENGTH=530 | 201606
Arabidopsis_thaliana	OG0000033
AT3G51350.1 | Eukaryotic aspartyl protease family protein |
Chr3:19060485-19063248 REVERSE LENGTH=528 | 201606
Arabidopsis_thaliana	OG0000033
AT3G51360.1 | Eukaryotic aspartyl protease family protein |
Chr3:19064294-19066560 REVERSE LENGTH=488 | 201606
Arabidopsis_thaliana	OG0000033
AT3G54400.1 | Eukaryotic aspartyl protease family protein |
Chr3:20140291-20142599 REVERSE LENGTH=425 | 201606
Arabidopsis_thaliana	OG0000033
AT3G59080.1 | Eukaryotic aspartyl protease family protein |
Chr3:21836812-21838419 FORWARD LENGTH=535 | 201606
Arabidopsis_thaliana	OG0000033
AT3G61820.1 | Eukaryotic aspartyl protease family protein |

Chr3:22880074-22881525 REVERSE LENGTH=483 | 201606
Arabidopsis_thaliana   OG0000033
AT4G12920.1 | Eukaryotic aspartyl protease family protein |
Chr4:7568286-7569455 FORWARD LENGTH=389 | 201606
Arabidopsis_thaliana   OG0000033
AT4G30030.1 | Eukaryotic aspartyl protease family protein |
Chr4:14682210-14683484 REVERSE LENGTH=424 | 201606
Arabidopsis_thaliana   OG0000033
AT4G30040.1 | Eukaryotic aspartyl protease family protein |
Chr4:14685602-14686885 FORWARD LENGTH=427 | 201606
Arabidopsis_thaliana   OG0000033
AT4G33490.1 | Eukaryotic aspartyl protease family protein |
Chr4:16108928-16110670 REVERSE LENGTH=401 | 201606
Arabidopsis_thaliana   OG0000033
AT4G35880.1 | Eukaryotic aspartyl protease family protein |
Chr4:16993339-16995721 FORWARD LENGTH=524 | 201606
Arabidopsis_thaliana   OG0000033
AT5G02190.1 | Eukaryotic aspartyl protease family protein |
Chr5:435322-436683 FORWARD LENGTH=453 | 201606
Arabidopsis_thaliana   OG0000033
AT5G07030.1 | Eukaryotic aspartyl protease family protein |
Chr5:2183600-2185717 REVERSE LENGTH=455 | 201606
Arabidopsis_thaliana   OG0000033
AT5G10080.1 | Eukaryotic aspartyl protease family protein |
Chr5:3150843-3153380 FORWARD LENGTH=528 | 201606
Arabidopsis_thaliana   OG0000033
AT5G10760.1 | Eukaryotic aspartyl protease family protein |
Chr5:3400671-3402165 REVERSE LENGTH=464 | 201606
Arabidopsis_thaliana   OG0000033
AT5G10770.1 | Eukaryotic aspartyl protease family protein |
Chr5:3403331-3405331 REVERSE LENGTH=474 | 201606
Arabidopsis_thaliana   OG0000033
AT5G19100.1 | Eukaryotic aspartyl protease family protein |
Chr5:6408242-6409417 REVERSE LENGTH=391 | 201606
Arabidopsis_thaliana   OG0000033
AT5G22850.1 | Eukaryotic aspartyl protease family protein |
Chr5:7633717-7636298 REVERSE LENGTH=493 | 201606
Arabidopsis_thaliana   OG0000033
AT5G33340.1 | Eukaryotic aspartyl protease family protein |
Chr5:12594474-12595787 FORWARD LENGTH=437 | 201606
Arabidopsis_thaliana   OG0000033
AT5G36260.1 | Eukaryotic aspartyl protease family protein |
Chr5:14285068-14288179 REVERSE LENGTH=482 | 201606
Arabidopsis_thaliana   OG0000033
AT5G37540.1 | Eukaryotic aspartyl protease family protein |
Chr5:14912862-14914190 FORWARD LENGTH=442 | 201606
Arabidopsis_thaliana   OG0000033
AT5G43100.1 | Eukaryotic aspartyl protease family protein |
Chr5:17299264-17302718 FORWARD LENGTH=631 | 201606
Arabidopsis_thaliana   OG0000033
AT1G25510.1 | Eukaryotic aspartyl protease family protein |
Chr1:8959372-8960823 REVERSE LENGTH=483 | 201606
Arabidopsis_thaliana   OG0000033
AT1G01300.1 | Eukaryotic aspartyl protease family protein |

Chr1:117065-118522 FORWARD LENGTH=485 | 201606
Arabidopsis_thaliana OG0000033
AT1G31450.1 | Eukaryotic aspartyl protease family protein |
Chr1:11259872-11261209 REVERSE LENGTH=445 | 201606
Arabidopsis_thaliana OG0000033
AT1G44130.1 | Eukaryotic aspartyl protease family protein |
Chr1:16787508-16789318 REVERSE LENGTH=405 | 201606
Arabidopsis_thaliana OG0000033
AT1G49050.1 | Eukaryotic aspartyl protease family protein |
Chr1:18150638-18153186 FORWARD LENGTH=583 | 201606
Arabidopsis_thaliana OG0000033
AT1G05840.1 | Eukaryotic aspartyl protease family protein |
Chr1:1762843-1766150 REVERSE LENGTH=485 | 201606
Arabidopsis_thaliana OG0000033
AT1G64830.1 | Eukaryotic aspartyl protease family protein |
Chr1:24091271-24092566 REVERSE LENGTH=431 | 201606
Arabidopsis_thaliana OG0000033
AT1G65240.1 | Eukaryotic aspartyl protease family protein |
Chr1:24230963-24233349 REVERSE LENGTH=475 | 201606
Arabidopsis_thaliana OG0000033
AT1G66180.1 | Eukaryotic aspartyl protease family protein |
Chr1:24647221-24648513 FORWARD LENGTH=430 | 201606
Arabidopsis_thaliana OG0000033
AT1G77480.1 | Eukaryotic aspartyl protease family protein |
Chr1:29114705-29117150 REVERSE LENGTH=466 | 201606
Arabidopsis_thaliana OG0000033
AT1G79720.1 | Eukaryotic aspartyl protease family protein |
Chr1:29997259-29998951 REVERSE LENGTH=484 | 201606
Arabidopsis_thaliana OG0000033
AT2G03200.1 | Eukaryotic aspartyl protease family protein |
Chr2:966506-967891 REVERSE LENGTH=461 | 201606
Arabidopsis_thaliana OG0000033
AT1G08210.1 | Eukaryotic aspartyl protease family protein |
Chr1:2577119-2580581 REVERSE LENGTH=492 | 201606
Arabidopsis_thaliana OG0000033
AT2G17760.1 | Eukaryotic aspartyl protease family protein |
Chr2:7713488-7716269 FORWARD LENGTH=513 | 201606
Arabidopsis_thaliana OG0000033
AT2G23945.1 | Eukaryotic aspartyl protease family protein |
Chr2:10185229-10186605 REVERSE LENGTH=458 | 201606
Arabidopsis_thaliana OG0000033
AT2G28010.1 | Eukaryotic aspartyl protease family protein |
Chr2:11930579-11931769 REVERSE LENGTH=396 | 201606
Arabidopsis_thaliana OG0000033
AT2G28030.1 | Eukaryotic aspartyl protease family protein |
Chr2:11934208-11935386 REVERSE LENGTH=392 | 201606
Arabidopsis_thaliana OG0000033
AT2G28040.1 | Eukaryotic aspartyl protease family protein |
Chr2:11936203-11937390 REVERSE LENGTH=395 | 201606
Arabidopsis_thaliana OG0000033
AT2G28220.1 | aspartic proteinase CDR1-like protein |
Chr2:12033998-12035206 FORWARD LENGTH=402 | 201606
Arabidopsis_thaliana OG0000033
AT2G28225.1 | aspartic proteinase CDR1-like protein |

Chr2:12036319-12037527 FORWARD LENGTH=402 | 201606
Arabidopsis_thaliana OG0000033
AT1G09750.1 | Eukaryotic aspartyl protease family protein |
Chr1:3157541-3158960 FORWARD LENGTH=449 | 201606
Arabidopsis_thaliana OG0000033
Os03t0271900-01 Peptidase aspartic, catalytic domain containing
protein. Oryza_sativa OG0000033
Os03t0317300-01 Peptidase A1 domain containing protein.
Oryza_sativa OG0000033
Os03t0317900-00 Similar to Eukaryotic aspartyl protease family
protein. Oryza_sativa OG0000033
Os03t0318400-01 Peptidase A1 domain containing protein.
Oryza_sativa OG0000033
Os04t0228000-01 Peptidase A1 domain containing protein.
Oryza_sativa OG0000033
Os04t0334700-01 Peptidase aspartic, catalytic domain containing
protein. Oryza_sativa OG0000033
Os04t0334951-01 Similar to OSIGBa0137O04.7 protein.
Oryza_sativa OG0000033
Os04t0336600-00 Peptidase aspartic, catalytic domain containing
protein. Oryza_sativa OG0000033
Os04t0337000-01 Peptidase aspartic, catalytic domain containing
protein. Oryza_sativa OG0000033
Os04t0448300-01 Peptidase A1 domain containing protein.
Oryza_sativa OG0000033
Os04t0448500-00 Peptidase A1 domain containing protein.
Oryza_sativa OG0000033
Os04t0596200-01 Peptidase A1 domain containing protein.
Oryza_sativa OG0000033
Os04t0677100-00 Similar to H0402C08.5 protein. Oryza_sativa
OG0000033
Os05t0375700-00 Hypothetical conserved gene. Oryza_sativa
OG0000033
Os05t0384300-01 Peptidase aspartic, catalytic domain containing
protein. Oryza_sativa OG0000033
Os05t0557100-01 Peptidase A1 domain containing protein.
Oryza_sativa OG0000033
Os05t0590000-01 Peptidase A1 domain containing protein.
Oryza_sativa OG0000033
Os05t0596000-01 Peptidase A1 domain containing protein.
Oryza_sativa OG0000033
Os06t0118000-01 Peptidase A1 domain containing protein.
Oryza_sativa OG0000033
Os06t0118700-01 Peptidase A1 domain containing protein.
Oryza_sativa OG0000033
Os06t0119600-01 Peptidase aspartic, catalytic domain containing
protein. Oryza_sativa OG0000033
Os06t0121500-01 Peptidase A1 domain containing protein.
Oryza_sativa OG0000033
Os06t0121800-00 Hypothetical conserved gene. Oryza_sativa
OG0000033
Os06t0209100-00 Peptidase A1 domain containing protein.
Oryza_sativa OG0000033
Os06t0268700-01 Peptidase A1 domain containing protein.

Oryza_sativa	OG0000033
Os06t0305400-01 Peptidase A1 domain containing protein.	Oryza_sativa	OG0000033
Os06t0306500-00 Similar to aspartic proteinase nepenthesin-2.	Oryza_sativa	OG0000033
Os06t0610800-01 Peptidase A1 domain containing protein.	Oryza_sativa	OG0000033
Os06t0717900-01 Peptidase A1 domain containing protein.	Oryza_sativa	OG0000033
Os01t0598600-01 Peptidase A1 domain containing protein.	Oryza_sativa	OG0000033
Os01t0608300-00 Similar to aspartic proteinase nepenthesin-2.	Oryza_sativa	OG0000033
Os07t0532800-00 Peptidase A1 domain containing protein.	Oryza_sativa	OG0000033
Os07t0533000-00 Peptidase A1 domain containing protein.	Oryza_sativa	OG0000033
Os07t0533300-00 Peptidase A1 domain containing protein.	Oryza_sativa	OG0000033
Os07t0533600-01 Peptidase aspartic, catalytic domain containing protein.	Oryza_sativa	OG0000033
Os07t0533800-01 Peptidase A1 domain containing protein.	Oryza_sativa	OG0000033
Os07t0592200-01 Similar to predicted protein.	Oryza_sativa	OG0000033
Os07t0658600-00 Similar to Nucleoid DNA-binding-like protein.	Oryza_sativa	OG0000033
Os08t0207800-01 Peptidase aspartic, catalytic domain containing protein.	Oryza_sativa	OG0000033
Os08t0267300-00 Peptidase A1 domain containing protein.	Oryza_sativa	OG0000033
Os08t0469000-00 Peptidase A1 domain containing protein.	Oryza_sativa	OG0000033
Os08t0469100-01 Peptidase aspartic, catalytic domain containing protein.	Oryza_sativa	OG0000033
Os01t0679550-00 Similar to Aspartic proteinase nepenthesin-2.	Oryza_sativa	OG0000033
Os09t0423500-01 Peptidase A1 domain containing protein.	Oryza_sativa	OG0000033
Os09t0452400-01 Peptidase A1 domain containing protein.	Oryza_sativa	OG0000033
Os09t0452800-01 Peptidase A1 domain containing protein.	Oryza_sativa	OG0000033
Os09t0482200-01 Peptidase A1 domain containing protein.	Oryza_sativa	OG0000033
Os09t0482500-01 Peptidase A1 domain containing protein.	Oryza_sativa	OG0000033
Os09t0542100-01 Peptidase A1 domain containing protein.	Oryza_sativa	OG0000033
Os01t0696800-01 Peptidase A1 domain containing protein.	Oryza_sativa	OG0000033
Os01t0720000-00 Peptidase A1 domain containing protein.	Oryza_sativa	OG0000033
Os10t0537800-01 Peptidase aspartic, catalytic domain containing

protein. Oryza_sativa   OG0000033
Os10t0538200-01 Peptidase aspartic, catalytic domain containing protein. Oryza_sativa   OG0000033
Os10t0539200-00 Hypothetical conserved gene.   Oryza_sativa   OG0000033
Os11t0183900-01 Similar to Aspartic proteinase Asp1 precursor (EC 3.4.23.-) (OsAsp1) (Nucellin- like protein).   Oryza_sativa   OG0000033
Os11t0184600-00 Similar to Aspartic proteinase Asp1.   Oryza_sativa   OG0000033
Os11t0184800-01 Similar to Aspartic proteinase Asp1 precursor (EC 3.4.23.-) (OsAsp1) (Nucellin- like protein).   Oryza_sativa   OG0000033
Os11t0215400-01 Peptidase aspartic, catalytic domain containing protein. Oryza_sativa   OG0000033
Os01t0140100-01 Peptidase A1 domain containing protein.   Oryza_sativa   OG0000033
Os12t0153700-00 Peptidase aspartic, catalytic domain containing protein. Oryza_sativa   OG0000033
Os12t0177500-01 Peptidase A1 domain containing protein.   Oryza_sativa   OG0000033
Os12t0450300-00 Similar to Eukaryotic aspartyl protease family protein, expressed.   Oryza_sativa   OG0000033
Os01t0777200-01 Peptidase aspartic, catalytic domain containing protein. Oryza_sativa   OG0000033
Os12t0583300-01 Peptidase aspartic, catalytic domain containing protein. Oryza_sativa   OG0000033
Os01t0844500-00 Peptidase aspartic, catalytic domain containing protein. Oryza_sativa   OG0000033
Os01t0868600-01 Hypothetical conserved gene.   Oryza_sativa   OG0000033
Os01t0937100-01 Similar to Xylanase inhibitor precursor (Xylanase inhibitor TAXI-I).   Oryza_sativa   OG0000033
Os01t0954900-01 Similar to Nucleoid DNA-binding-like protein.   Oryza_sativa   OG0000033
Os02t0314600-01 Peptidase A1 domain containing protein.   Oryza_sativa   OG0000033
Os02t0473200-01 Peptidase aspartic, catalytic domain containing protein. Oryza_sativa   OG0000033
Os02t0720500-01 Peptidase A1 domain containing protein.   Oryza_sativa   OG0000033
Os02t0720600-01 Peptidase A1 domain containing protein.   Oryza_sativa   OG0000033
Os02t0720900-01 Peptidase A1 domain containing protein.   Oryza_sativa   OG0000033
Os02t0730700-02 Peptidase A1 domain containing protein.   Oryza_sativa   OG0000033
Os02t0751100-01 Peptidase A1 domain containing protein.   Oryza_sativa   OG0000033
Os03t0186900-01 Radc1.   Oryza_sativa   OG0000033
CMT203CT pep chromosome:ASM9120v1:20:515885:517799:-1 gene:CMT203C transcript:CMT203CT gene_biotype:protein_coding transcript_biotype:protein_coding Cyanidioschyzon_merolae OG0000033

CMN193CT pep chromosome:ASM9120v1:14:477888:479583:-1 gene:CMN193C transcript:CMN193CT gene_biotype:protein_coding transcript_biotype:protein_coding Cyanidioschyzon_merolae OG0000033
CMN194CT pep chromosome:ASM9120v1:14:482859:484287:-1 gene:CMN194C transcript:CMN194CT gene_biotype:protein_coding transcript_biotype:protein_coding Cyanidioschyzon_merolae OG0000033
evm.model.contig_2122.3 Porphyridium_purpureum OG0000033
evm.model.contig_3423.13 Porphyridium_purpureum OG0000033
evm.model.contig_2015.5 Porphyridium_purpureum OG0000033
evm.model.contig_4585.1 Porphyridium_purpureum OG0000033
CDF36747 pep supercontig:ASM35022v2:HG001800:55196:57001:1 gene:CHC_T00004793001 transcript:CDF36747 gene_biotype:protein_coding transcript_biotype:protein_coding Chondrus_crispus OG0000033
CDF39929 pep supercontig:ASM35022v2:HG002094:113512:115594:1 gene:CHC_T00006889001 transcript:CDF39929 gene_biotype:protein_coding transcript_biotype:protein_coding Chondrus_crispus OG0000033
CDF40549 pep supercontig:ASM35022v2:HG002199:3331:5363:-1 gene:CHC_T00000777001 transcript:CDF40549 gene_biotype:protein_coding transcript_biotype:protein_coding Chondrus_crispus OG0000033
CDF40925 pep supercontig:ASM35022v2:HG002272:776:1582:1 gene:CHC_T00000891001 transcript:CDF40925 gene_biotype:protein_coding transcript_biotype:protein_coding Chondrus_crispus OG0000033
g5730.t1 Caulerpa_lentillifera OG0000034
g5731.t1 Caulerpa_lentillifera OG0000034
Cre04.g224700.t1.2 pacid=30791047 transcript=Cre04.g224700.t1.2 locus=Cre04.g224700 ID=Cre04.g224700.t1.2.v5.5 annot-version=v5.5 Chlamydomonas_reinhardtii OG0000034
Vocar.0049s0020.1.p pacid=32886558 transcript=Vocar.0049s0020.1 locus=Vocar.0049s0020 ID=Vocar.0049s0020.1.v2.1 annot-version=v2.1 Volvox_carteri OG0000034
jgi|ChlNC64A_1|23105|e_gw1.10.316.1 Chlorella_variabilis OG0000034
jgi|ChlNC64A_1|144528|IGS.gm_7_00470 Chlorella_variabilis OG0000034
48785 pacid=27394442 transcript=48785 locus=estExt_fgenesh1_pg.C_180039 ID=48785.2.0.227 annot-version=v2.0 Coccomyxa_subellipsoidea OG0000034
19259 pacid=27394463 transcript=19259 locus=e_gw1.18.39.1 ID=19259.2.0.227 annot-version=v2.0 Coccomyxa_subellipsoidea OG0000034
2706_gw1.11.00.162.1 Ostreococcus_tauri OG0000034
50811 pacid=27420441 transcript=50811 locus=estExt_Genewise_ext.C_Chr_110077 ID=50811.2.0.231 annot-version=v2.0 Ostreococcus_lucimarinus OG0000034
62932 pacid=27399911 transcript=62932 locus=EuGene.1200010104 ID=62932.3.0.229 annot-version=v3.0 Micromonas_sp._RCC299 OG0000034
kfl00057_0100_v1.1 Klebsormidium_flaccidum OG0000034

kfl00235_0060_v1.1	Klebsormidium_flaccidum	OG0000034
Pp3c15_19120V3.1.p pacid=32929615 transcript=Pp3c15_19120V3.1 locus=Pp3c15_19120 annot-version=v3.3	Physcomitrella_patens	OG0000034
Pp3c13_4730V3.1.p pacid=32931941 transcript=Pp3c13_4730V3.1 locus=Pp3c13_4730 annot-version=v3.3	Physcomitrella_patens	OG0000034
Pp3c22_14360V3.1.p pacid=32903011 transcript=Pp3c22_14360V3.1 locus=Pp3c22_14360 annot-version=v3.3	Physcomitrella_patens	OG0000034
Pp3c20_18670V3.1.p pacid=32946217 transcript=Pp3c20_18670V3.1 locus=Pp3c20_18670 annot-version=v3.3	Physcomitrella_patens	OG0000034
Pp3c20_6850V3.1.p pacid=32946839 transcript=Pp3c20_6850V3.1 locus=Pp3c20_6850 annot-version=v3.3	Physcomitrella_patens	OG0000034
Pp3c5_1870V3.1.p pacid=32952729 transcript=Pp3c5_1870V3.1 locus=Pp3c5_1870 annot-version=v3.3	Physcomitrella_patens	OG0000034
Pp3c5_16340V3.1.p pacid=32952942 transcript=Pp3c5_16340V3.1 locus=Pp3c5_16340 annot-version=v3.3	Physcomitrella_patens	OG0000034
Pp3c11_5710V3.1.p pacid=32957399 transcript=Pp3c11_5710V3.1 locus=Pp3c11_5710 annot-version=v3.3	Physcomitrella_patens	OG0000034
Pp3c14_23410V3.1.p pacid=32961190 transcript=Pp3c14_23410V3.1 locus=Pp3c14_23410 annot-version=v3.3	Physcomitrella_patens	OG0000034
Pp3c8_15410V3.1.p pacid=32963504 transcript=Pp3c8_15410V3.1 locus=Pp3c8_15410 annot-version=v3.3	Physcomitrella_patens	OG0000034
Pp3c17_16320V3.1.p pacid=32906211 transcript=Pp3c17_16320V3.1 locus=Pp3c17_16320 annot-version=v3.3	Physcomitrella_patens	OG0000034
Pp3c1_38540V3.1.p pacid=32967232 transcript=Pp3c1_38540V3.1 locus=Pp3c1_38540 annot-version=v3.3	Physcomitrella_patens	OG0000034
Pp3c1_31230V3.1.p pacid=32968134 transcript=Pp3c1_31230V3.1 locus=Pp3c1_31230 annot-version=v3.3	Physcomitrella_patens	OG0000034
Pp3c12_4100V3.1.p pacid=32974425 transcript=Pp3c12_4100V3.1 locus=Pp3c12_4100 annot-version=v3.3	Physcomitrella_patens	OG0000034
Pp3c6_10660V3.1.p pacid=32975885 transcript=Pp3c6_10660V3.1 locus=Pp3c6_10660 annot-version=v3.3	Physcomitrella_patens	OG0000034
Pp3c6_18680V3.1.p pacid=32978368 transcript=Pp3c6_18680V3.1 locus=Pp3c6_18680 annot-version=v3.3	Physcomitrella_patens	OG0000034
Pp3c17_20530V3.1.p pacid=32908025 transcript=Pp3c17_20530V3.1 locus=Pp3c17_20530 annot-version=v3.3	Physcomitrella_patens	OG0000034
Pp3c16_20730V3.1.p pacid=32984865 transcript=Pp3c16_20730V3.1 locus=Pp3c16_20730 annot-version=v3.3	Physcomitrella_patens

OG0000034
Pp3c16_20729V3.1.p pacid=32986609 transcript=Pp3c16_20729V3.1
locus=Pp3c16_20729 annot-version=v3.3   Physcomitrella_patens
OG0000034
Pp3c17_17090V3.1.p pacid=32908277 transcript=Pp3c17_17090V3.1
locus=Pp3c17_17090 annot-version=v3.3   Physcomitrella_patens
OG0000034
Pp3c9_14660V3.1.p pacid=32914084 transcript=Pp3c9_14660V3.1
locus=Pp3c9_14660 annot-version=v3.3   Physcomitrella_patens
OG0000034
Pp3c4_21460V3.1.p pacid=32920365 transcript=Pp3c4_21460V3.1
locus=Pp3c4_21460 annot-version=v3.3   Physcomitrella_patens
OG0000034
Pp3c4_21340V3.1.p pacid=32920813 transcript=Pp3c4_21340V3.1
locus=Pp3c4_21340 annot-version=v3.3   Physcomitrella_patens
OG0000034
Pp3c4_7710V3.1.p pacid=32922714 transcript=Pp3c4_7710V3.1
locus=Pp3c4_7710 annot-version=v3.3   Physcomitrella_patens
OG0000034
Pp3c7_13920V3.1.p pacid=32923283 transcript=Pp3c7_13920V3.1
locus=Pp3c7_13920 annot-version=v3.3   Physcomitrella_patens
OG0000034
AT2G37900.1 | Major facilitator superfamily protein |
Chr2:15864396-15866408 REVERSE LENGTH=575 | 201606
Arabidopsis_thaliana   OG0000034
AT2G38100.1 | proton-dependent oligopeptide transport (POT) family
protein | Chr2:15948484-15950228 REVERSE LENGTH=521 | 201606
Arabidopsis_thaliana   OG0000034
AT2G40460.1 | Major facilitator superfamily protein |
Chr2:16897123-16901171 FORWARD LENGTH=583 | 201606
Arabidopsis_thaliana   OG0000034
AT3G01350.1 | Major facilitator superfamily protein |
Chr3:135024-137460 FORWARD LENGTH=563 | 201606
Arabidopsis_thaliana   OG0000034
AT1G12110.1 | nitrate transporter 1.1 | Chr1:4105341-4109290 FORWARD
LENGTH=590 | 201606   Arabidopsis_thaliana   OG0000034
AT3G16180.1 | Major facilitator superfamily protein |
Chr3:5481477-5484943 REVERSE LENGTH=591 | 201606
Arabidopsis_thaliana   OG0000034
AT3G21670.1 | Major facilitator superfamily protein |
Chr3:7626942-7628954 REVERSE LENGTH=590 | 201606
Arabidopsis_thaliana   OG0000034
AT3G25260.1 | Major facilitator superfamily protein |
Chr3:9199594-9201764 FORWARD LENGTH=515 | 201606
Arabidopsis_thaliana   OG0000034
AT3G25280.1 | Major facilitator superfamily protein |
Chr3:9206183-9208036 FORWARD LENGTH=521 | 201606
Arabidopsis_thaliana   OG0000034
AT3G45650.1 | nitrate excretion transporter1 |
Chr3:16759253-16761266 FORWARD LENGTH=558 | 201606
Arabidopsis_thaliana   OG0000034
AT3G45660.1 | Major facilitator superfamily protein |
Chr3:16762205-16764241 FORWARD LENGTH=557 | 201606
Arabidopsis_thaliana   OG0000034

AT3G45680.1 | Major facilitator superfamily protein |
Chr3:16770995-16772908 FORWARD LENGTH=558 | 201606
Arabidopsis_thaliana OG0000034
AT3G45690.1 | Major facilitator superfamily protein |
Chr3:16776268-16778150 FORWARD LENGTH=516 | 201606
Arabidopsis_thaliana OG0000034
AT3G45700.1 | Major facilitator superfamily protein |
Chr3:16778765-16781068 FORWARD LENGTH=548 | 201606
Arabidopsis_thaliana OG0000034
AT3G45710.1 | Major facilitator superfamily protein |
Chr3:16782719-16784617 FORWARD LENGTH=560 | 201606
Arabidopsis_thaliana OG0000034
AT3G45720.1 | Major facilitator superfamily protein |
Chr3:16785046-16786945 FORWARD LENGTH=555 | 201606
Arabidopsis_thaliana OG0000034
AT3G47960.1 | Major facilitator superfamily protein |
Chr3:17698126-17700771 REVERSE LENGTH=636 | 201606
Arabidopsis_thaliana OG0000034
AT3G53960.1 | Major facilitator superfamily protein |
Chr3:19978306-19980886 REVERSE LENGTH=602 | 201606
Arabidopsis_thaliana OG0000034
AT3G54140.1 | peptide transporter 1 | Chr3:20045885-20048154 REVERSE
LENGTH=570 | 201606 Arabidopsis_thaliana OG0000034
AT3G54450.1 | Major facilitator superfamily protein |
Chr3:20158534-20161937 FORWARD LENGTH=555 | 201606
Arabidopsis_thaliana OG0000034
AT4G21680.1 | NITRATE TRANSPORTER 1.8 | Chr4:11517540-11519576
REVERSE LENGTH=589 | 201606 Arabidopsis_thaliana
OG0000034
AT1G18880.1 | Major facilitator superfamily protein |
Chr1:6520800-6523241 FORWARD LENGTH=587 | 201606
Arabidopsis_thaliana OG0000034
AT5G01180.1 | peptide transporter 5 | Chr5:61257-63240 REVERSE
LENGTH=570 | 201606 Arabidopsis_thaliana OG0000034
AT5G11570.1 | Major facilitator superfamily protein |
Chr5:3715943-3718276 REVERSE LENGTH=481 | 201606
Arabidopsis_thaliana OG0000034
AT5G13400.1 | Major facilitator superfamily protein |
Chr5:4296854-4299079 REVERSE LENGTH=624 | 201606
Arabidopsis_thaliana OG0000034
AT5G14940.1 | Major facilitator superfamily protein |
Chr5:4831748-4834312 REVERSE LENGTH=552 | 201606
Arabidopsis_thaliana OG0000034
AT1G22540.1 | Major facilitator superfamily protein |
Chr1:7964202-7966222 FORWARD LENGTH=557 | 201606
Arabidopsis_thaliana OG0000034
AT1G22550.1 | Major facilitator superfamily protein |
Chr1:7966608-7968552 REVERSE LENGTH=564 | 201606
Arabidopsis_thaliana OG0000034
AT1G22570.1 | Major facilitator superfamily protein |
Chr1:7976620-7978573 REVERSE LENGTH=565 | 201606
Arabidopsis_thaliana OG0000034
AT5G19640.1 | Major facilitator superfamily protein |
Chr5:6636460-6638590 FORWARD LENGTH=609 | 201606

Arabidopsis_thaliana   OG0000034
AT5G28470.1 | Major facilitator superfamily protein |
Chr5:10429813-10432357 FORWARD LENGTH=559 | 201606
Arabidopsis_thaliana   OG0000034
AT5G46040.1 | Major facilitator superfamily protein |
Chr5:18671397-18673551 REVERSE LENGTH=586 | 201606
Arabidopsis_thaliana   OG0000034
AT5G46050.1 | peptide transporter 3 | Chr5:18675062-18679071 REVERSE
LENGTH=582 | 201606   Arabidopsis_thaliana   OG0000034
AT5G62680.1 | Major facilitator superfamily protein |
Chr5:25165430-25167822 REVERSE LENGTH=616 | 201606
Arabidopsis_thaliana   OG0000034
AT5G62730.1 | Major facilitator superfamily protein |
Chr5:25197494-25200033 FORWARD LENGTH=589 | 201606
Arabidopsis_thaliana   OG0000034
AT1G27040.1 | Major facilitator superfamily protein |
Chr1:9386893-9390018 REVERSE LENGTH=567 | 201606
Arabidopsis_thaliana   OG0000034
AT1G27080.1 | nitrate transporter 1.6 | Chr1:9400664-9403789 FORWARD
LENGTH=576 | 201606   Arabidopsis_thaliana   OG0000034
AT1G32450.1 | nitrate transporter 1.5 | Chr1:11715337-11719807
REVERSE LENGTH=614 | 201606   Arabidopsis_thaliana
OG0000034
AT1G33440.1 | Major facilitator superfamily protein |
Chr1:12127712-12130327 REVERSE LENGTH=601 | 201606
Arabidopsis_thaliana   OG0000034
AT1G52190.1 | Major facilitator superfamily protein |
Chr1:19434671-19438673 FORWARD LENGTH=607 | 201606
Arabidopsis_thaliana   OG0000034
AT1G59740.1 | Major facilitator superfamily protein |
Chr1:21968227-21972312 FORWARD LENGTH=591 | 201606
Arabidopsis_thaliana   OG0000034
AT1G62200.1 | Major facilitator superfamily protein |
Chr1:22982147-22984334 REVERSE LENGTH=590 | 201606
Arabidopsis_thaliana   OG0000034
AT1G68570.1 | Major facilitator superfamily protein |
Chr1:25746811-25750110 FORWARD LENGTH=596 | 201606
Arabidopsis_thaliana   OG0000034
AT1G69850.1 | nitrate transporter 1:2 | Chr1:26296945-26300407
REVERSE LENGTH=585 | 201606   Arabidopsis_thaliana
OG0000034
AT1G69860.1 | Major facilitator superfamily protein |
Chr1:26309628-26312174 FORWARD LENGTH=555 | 201606
Arabidopsis_thaliana   OG0000034
AT1G69870.1 | nitrate transporter 1.7 | Chr1:26316208-26320097
FORWARD LENGTH=620 | 201606   Arabidopsis_thaliana
OG0000034
AT1G72120.1 | Major facilitator superfamily protein |
Chr1:27132133-27133975 FORWARD LENGTH=557 | 201606
Arabidopsis_thaliana   OG0000034
AT1G72125.1 | Major facilitator superfamily protein |
Chr1:27134168-27136257 FORWARD LENGTH=561 | 201606
Arabidopsis_thaliana   OG0000034
AT1G72130.1 | Major facilitator superfamily protein |

Chr1:27137201-27139223 FORWARD LENGTH=538 | 201606
Arabidopsis_thaliana   OG0000034
AT1G72140.1 | Major facilitator superfamily protein |
Chr1:27141877-27144346 FORWARD LENGTH=555 | 201606
Arabidopsis_thaliana   OG0000034
AT2G02020.1 | Major facilitator superfamily protein |
Chr2:479117-481131 FORWARD LENGTH=545 | 201606
Arabidopsis_thaliana   OG0000034
AT2G02040.1 | peptide transporter 2 | Chr2:487542-489707 FORWARD
LENGTH=585 | 201606   Arabidopsis_thaliana   OG0000034
AT2G26690.1 | Major facilitator superfamily protein |
Chr2:11347347-11350916 REVERSE LENGTH=577 | 201606
Arabidopsis_thaliana   OG0000034
Os03t0235300-01 Similar to Peptide transporter PTR2.
Oryza_sativa   OG0000034
Os03t0235700-01 Similar to Peptide transporter 1. Oryza_sativa
OG0000034
Os03t0235900-01 Similar to Low affinity nitrate transporter NRT1.2.
Oryza_sativa   OG0000034
Os03t0687000-01 Similar to predicted protein.   Oryza_sativa
OG0000034
Os03t0719900-01 Similar to Peptide transporter 1. Oryza_sativa
OG0000034
Os03t0823500-02 TGF-beta receptor, type I/II extracellular region
family protein. Oryza_sativa   OG0000034
Os04t0441800-01 TGF-beta receptor, type I/II extracellular region
family protein. Oryza_sativa   OG0000034
Os04t0464400-00 Similar to peptide transporter PTR2.
Oryza_sativa   OG0000034
Os04t0491200-00 Oligopeptide transporter domain containing protein.
Oryza_sativa   OG0000034
Os04t0491350-00 Similar to OSIGBa0130B08.8 protein.
Oryza_sativa   OG0000034
Os04t0491500-01 Similar to OSIGBa0130B08.10 protein.
Oryza_sativa   OG0000034
Os04t0597400-00 Similar to OSJNba0093F12.10 protein.
Oryza_sativa   OG0000034
Os04t0597501-00 Similar to OSJNba0093F12.10 protein.
Oryza_sativa   OG0000034
Os04t0597600-01 TGF-beta receptor, type I/II extracellular region
family protein. Oryza_sativa   OG0000034
Os04t0597800-01 TGF-beta receptor, type I/II extracellular region
family protein. Oryza_sativa   OG0000034
Os04t0660900-01 TGF-beta receptor, type I/II extracellular region
family protein. Oryza_sativa   OG0000034
Os04t0691366-00 Similar to POT family protein.   Oryza_sativa
OG0000034
Os04t0691400-00 Similar to POT family protein.   Oryza_sativa
OG0000034
Os05t0335800-01 TGF-beta receptor, type I/II extracellular region
family protein. Oryza_sativa   OG0000034
Os05t0336200-00 Similar to predicted protein.   Oryza_sativa
OG0000034
Os05t0338900-00 Oligopeptide transporter domain containing protein.

Oryza_sativa OG0000034
Os01t0386600-00 TGF-beta receptor, type I/II extracellular region family protein. Oryza_sativa OG0000034
Os05t0410500-01 TGF-beta receptor, type I/II extracellular region family protein. Oryza_sativa OG0000034
Os05t0410800-01 TGF-beta receptor, type I/II extracellular region family protein. Oryza_sativa OG0000034
Os05t0410900-02 Similar to peptide transporter PTR2. Oryza_sativa OG0000034
Os05t0411100-00 Similar to peptide transporter PTR2. Oryza_sativa OG0000034
Os05t0430900-01 TGF-beta receptor, type I/II extracellular region family protein. Oryza_sativa OG0000034
Os05t0431700-01 Similar to peptide transporter PTR2-B. Oryza_sativa OG0000034
Os06t0239300-00 Oligopeptide transporter domain containing protein. Oryza_sativa OG0000034
Os06t0239500-01 TGF-beta receptor, type I/II extracellular region family protein. Oryza_sativa OG0000034
Os06t0264500-01 TGF-beta receptor, type I/II extracellular region family protein. Oryza_sativa OG0000034
Os06t0299500-00 Similar to J023036E09, full insert sequence (Fragment). Oryza_sativa OG0000034
Os06t0324300-00 Similar to predicted protein. Oryza_sativa OG0000034
Os01t0556700-00 Hypothetical conserved gene. Oryza_sativa OG0000034
Os06t0581000-01 Similar to Nitrate transporter NTL1. Oryza_sativa OG0000034
Os06t0705600-00 Oligopeptide transporter domain containing protein. Oryza_sativa OG0000034
Os06t0705700-01 TGF-beta receptor, type I/II extracellular region family protein. Oryza_sativa OG0000034
Os06t0706100-00 Similar to Peptide transporter PTR2. Oryza_sativa OG0000034
Os06t0706400-01 PTR/NRT1 (peptide transporter/nitrate transporter 1) family protein, Nitrogen utilization efficiency, growth and grain yield Oryza_sativa OG0000034
Os07t0100600-01 Similar to Peptide transporter. Oryza_sativa OG0000034
Os07t0190650-00 Similar to predicted protein. Oryza_sativa OG0000034
Os07t0403800-00 Similar to ligA. Oryza_sativa OG0000034
Os07t0603800-01 TGF-beta receptor, type I/II extracellular region family protein. Oryza_sativa OG0000034
Os08t0155400-01 Similar to Nitrate/chlorate transporter. Oryza_sativa OG0000034
Os08t0527700-00 Similar to POT family protein. Oryza_sativa OG0000034
Os08t0527733-00 Similar to proton-dependent oligopeptide transporter. Oryza_sativa OG0000034
Os10t0109700-00 Similar to POT family protein. Oryza_sativa OG0000034
Os10t0109900-00 Major facilitator superfamily, general substrate

transporter domain containing protein.	Oryza_sativa	OG0000034
Os10t0110250-00 Oligopeptide transporter domain containing protein.	Oryza_sativa	OG0000034
Os10t0110600-01 Similar to cDNA clone:J023063N08, full insert sequence.	Oryza_sativa	OG0000034
Os10t0110800-01 Similar to Nitrate transporter (Fragment).	Oryza_sativa	OG0000034
Os10t0111300-01 Similar to Nitrate transporter (Fragment).	Oryza_sativa	OG0000034
Os10t0111700-01 Similar to POT family protein, expressed.	Oryza_sativa	OG0000034
Os10t0112500-00 Similar to POT family protein, expressed.	Oryza_sativa	OG0000034
Os10t0148400-00 Similar to POT family protein.	Oryza_sativa	OG0000034
Os01t0103100-01 TGF-beta receptor, type I/II extracellular region family protein.	Oryza_sativa	OG0000034
Os10t0370700-01 Similar to Nitrate transporter (Fragment).	Oryza_sativa	OG0000034
Os10t0469900-01 TGF-beta receptor, type I/II extracellular region family protein.	Oryza_sativa	OG0000034
Os10t0470700-01 Similar to Peptide transporter.	Oryza_sativa	OG0000034
Os10t0554200-01 TGF-beta receptor, type I/II extracellular region family protein.	Oryza_sativa	OG0000034
Os10t0579600-01 TGF-beta receptor, type I/II extracellular region family protein.	Oryza_sativa	OG0000034
Os10t0579800-01 TGF-beta receptor, type I/II extracellular region family protein.	Oryza_sativa	OG0000034
Os11t0235200-01 Peptide transporter (PTR) family transporter, Proton oligopeptide tansporter (POT), Panicle elongation	Oryza_sativa	OG0000034
Os11t0282800-00 Similar to POT family protein, expressed.	Oryza_sativa	OG0000034
Os11t0283500-01 TGF-beta receptor, type I/II extracellular region family protein.	Oryza_sativa	OG0000034
Os11t0284032-00 Similar to POT family protein.	Oryza_sativa	OG0000034
Os11t0284300-00 Similar to POT family protein.	Oryza_sativa	OG0000034
Os01t0748950-01 Similar to predicted protein.	Oryza_sativa	OG0000034
Os01t0761400-01 TGF-beta receptor, type I/II extracellular region family protein.	Oryza_sativa	OG0000034
Os01t0761500-01 TGF-beta receptor, type I/II extracellular region family protein.	Oryza_sativa	OG0000034
Os12t0231000-01 TGF-beta receptor, type I/II extracellular region family protein.	Oryza_sativa	OG0000034
Os12t0240875-00 TGF-beta receptor, type I/II extracellular region domain containing protein.	Oryza_sativa	OG0000034
Os01t0142800-01 Similar to Peptide transporter.	Oryza_sativa	OG0000034
Os12t0638200-01 Similar to Peptide transporter.	Oryza_sativa

OG0000034
Os12t0638300-01 Similar to Peptide transporter.	Oryza_sativa	OG0000034
Os01t0871500-01 TGF-beta receptor, type I/II extracellular region family protein.	Oryza_sativa	OG0000034
Os01t0871600-01 Similar to Peptide transporter PTR2-B.	Oryza_sativa	OG0000034
Os01t0871700-00 Oligopeptide transporter domain containing protein.	Oryza_sativa	OG0000034
Os01t0871800-01 TGF-beta receptor, type I/II extracellular region family protein.	Oryza_sativa	OG0000034
Os01t0871900-00 Similar to POT family protein.	Oryza_sativa	OG0000034
Os01t0872000-01 TGF-beta receptor, type I/II extracellular region family protein.	Oryza_sativa	OG0000034
Os01t0872100-01 TGF-beta receptor, type I/II extracellular region family protein.	Oryza_sativa	OG0000034
Os01t0872300-00 Similar to POT family protein.	Oryza_sativa	OG0000034
Os01t0872400-01 Similar to POT family protein.	Oryza_sativa	OG0000034
Os01t0872450-00 Similar to POT family protein.	Oryza_sativa	OG0000034
Os01t0872500-01 Oligopeptide transporter domain containing protein.	Oryza_sativa	OG0000034
Os01t0872600-01 TGF-beta receptor, type I/II extracellular region family protein.	Oryza_sativa	OG0000034
Os01t0902700-01 Similar to Peptide transporter PTR2.	Oryza_sativa	OG0000034
Os01t0902800-00 Similar to cDNA, clone	Oryza_sativa	OG0000034
Os01t0913300-01 TGF-beta receptor, type I/II extracellular region family protein.	Oryza_sativa	OG0000034
Os01t0960900-00 Oligopeptide transporter domain containing protein.	Oryza_sativa	OG0000034
Os02t0519401-00 Similar to POT family protein.	Oryza_sativa	OG0000034
Os02t0580900-01 TGF-beta receptor, type I/II extracellular region family protein.	Oryza_sativa	OG0000034
Os02t0689900-01 TGF-beta receptor, type I/II extracellular region family protein.	Oryza_sativa	OG0000034
Os02t0699000-01 TGF-beta receptor, type I/II extracellular region family protein.	Oryza_sativa	OG0000034
Os02t0716800-01 TGF-beta receptor, type I/II extracellular region family protein.	Oryza_sativa	OG0000034
Os02t0757400-00 Oligopeptide transporter domain containing protein.	Oryza_sativa	OG0000034
Os03t0103000-01 Similar to Nitrate transporter.	Oryza_sativa	OG0000034
Os03t0138700-01 TGF-beta receptor, type I/II extracellular region family protein.	Oryza_sativa	OG0000034
g5566.t1 Caulerpa_lentillifera	OG0000035
g7844.t1 Caulerpa_lentillifera	OG0000035
g8696.t1 Caulerpa_lentillifera	OG0000035

Cre02.g110300.t1.2 pacid=30784981 transcript=Cre02.g110300.t1.2 locus=Cre02.g110300 ID=Cre02.g110300.t1.2.v5.5 annot-version=v5.5 Chlamydomonas_reinhardtii OG0000035
Cre07.g327450.t1.2 pacid=30774814 transcript=Cre07.g327450.t1.2 locus=Cre07.g327450 ID=Cre07.g327450.t1.2.v5.5 annot-version=v5.5 Chlamydomonas_reinhardtii OG0000035
Cre07.g316050.t1.2 pacid=30775379 transcript=Cre07.g316050.t1.2 locus=Cre07.g316050 ID=Cre07.g316050.t1.2.v5.5 annot-version=v5.5 Chlamydomonas_reinhardtii OG0000035
Cre12.g555700.t1.1 pacid=30793501 transcript=Cre12.g555700.t1.1 locus=Cre12.g555700 ID=Cre12.g555700.t1.1.v5.5 annot-version=v5.5 Chlamydomonas_reinhardtii OG0000035
Cre16.g694500.t1.1 pacid=30777658 transcript=Cre16.g694500.t1.1 locus=Cre16.g694500 ID=Cre16.g694500.t1.1.v5.5 annot-version=v5.5 Chlamydomonas_reinhardtii OG0000035
Cre09.g416100.t1.2 pacid=30780886 transcript=Cre09.g416100.t1.2 locus=Cre09.g416100 ID=Cre09.g416100.t1.2.v5.5 annot-version=v5.5 Chlamydomonas_reinhardtii OG0000035
Vocar.0011s0269.1.p pacid=32895176 transcript=Vocar.0011s0269.1 locus=Vocar.0011s0269 ID=Vocar.0011s0269.1.v2.1 annot-version=v2.1 Volvox_carteri OG0000035
Vocar.0001s1626.1.p pacid=32885145 transcript=Vocar.0001s1626.1 locus=Vocar.0001s1626 ID=Vocar.0001s1626.1.v2.1 annot-version=v2.1 Volvox_carteri OG0000035
Vocar.0033s0043.1.p pacid=32886426 transcript=Vocar.0033s0043.1 locus=Vocar.0033s0043 ID=Vocar.0033s0043.1.v2.1 annot-version=v2.1 Volvox_carteri OG0000035
Vocar.0003s0122.1.p pacid=32889189 transcript=Vocar.0003s0122.1 locus=Vocar.0003s0122 ID=Vocar.0003s0122.1.v2.1 annot-version=v2.1 Volvox_carteri OG0000035
Vocar.0004s0066.1.p pacid=32889629 transcript=Vocar.0004s0066.1 locus=Vocar.0004s0066 ID=Vocar.0004s0066.1.v2.1 annot-version=v2.1 Volvox_carteri OG0000035
Vocar.0002s0090.1.p pacid=32891146 transcript=Vocar.0002s0090.1 locus=Vocar.0002s0090 ID=Vocar.0002s0090.1.v2.1 annot-version=v2.1 Volvox_carteri OG0000035
Vocar.0017s0095.1.p pacid=32891555 transcript=Vocar.0017s0095.1 locus=Vocar.0017s0095 ID=Vocar.0017s0095.1.v2.1 annot-version=v2.1 Volvox_carteri OG0000035
Vocar.0009s0264.1.p pacid=32893148 transcript=Vocar.0009s0264.1 locus=Vocar.0009s0264 ID=Vocar.0009s0264.1.v2.1 annot-version=v2.1 Volvox_carteri OG0000035
Vocar.0006s0217.1.p pacid=32894341 transcript=Vocar.0006s0217.1 locus=Vocar.0006s0217 ID=Vocar.0006s0217.1.v2.1 annot-version=v2.1 Volvox_carteri OG0000035
jgi|ChlNC64A_1|19791|e_gw1.3.602.1 Chlorella_variabilis OG0000035
jgi|ChlNC64A_1|133862|IGS.gm_10_00153 Chlorella_variabilis OG0000035
jgi|ChlNC64A_1|23262|e_gw1.10.278.1 Chlorella_variabilis OG0000035
jgi|ChlNC64A_1|133376|IGS.gm_1_00512 Chlorella_variabilis OG0000035
jgi|ChlNC64A_1|133380|IGS.gm_1_00516 Chlorella_variabilis

OG0000035
jgi|ChlNC64A_1|53215|fgenesh3_pg.C_scaffold_14000125
Chlorella_variabilis OG0000035
jgi|ChlNC64A_1|135747|IGS.gm_14_00280 Chlorella_variabilis
OG0000035
jgi|ChlNC64A_1|132924|IGS.gm_1_00060 Chlorella_variabilis
OG0000035
jgi|ChlNC64A_1|145222|IGS.gm_9_00029 Chlorella_variabilis
OG0000035
jgi|ChlNC64A_1|17952|gw1.5.687.1 Chlorella_variabilis
OG0000035
jgi|ChlNC64A_1|15756|gw1.5.519.1 Chlorella_variabilis
OG0000035
jgi|ChlNC64A_1|15844|gw1.16.225.1 Chlorella_variabilis
OG0000035
jgi|ChlNC64A_1|15594|gw1.13.234.1 Chlorella_variabilis
OG0000035
jgi|ChlNC64A_1|140221|IGS.gm_29_00101 Chlorella_variabilis
OG0000035
jgi|ChlNC64A_1|15591|gw1.29.159.1 Chlorella_variabilis
OG0000035
jgi|ChlNC64A_1|137366|IGS.gm_19_00133 Chlorella_variabilis
OG0000035
52983 pacid=27387194 transcript=52983
locus=estExt_fgenesh1_pm.C_50055 ID=52983.2.0.227 annot-version=v2.0
Coccomyxa_subellipsoidea OG0000035
54257 pacid=27388094 transcript=54257
locus=estExt_fgenesh1_pm.C_140096 ID=54257.2.0.227 annot-
version=v2.0 Coccomyxa_subellipsoidea OG0000035
19772 pacid=27388331 transcript=19772 locus=e_gw1.19.338.1
ID=19772.2.0.227 annot-version=v2.0 Coccomyxa_subellipsoidea
OG0000035
45976 pacid=27389008 transcript=45976
locus=estExt_fgenesh1_pg.C_10518 ID=45976.2.0.227 annot-version=v2.0
Coccomyxa_subellipsoidea OG0000035
65248 pacid=27390031 transcript=65248 locus=estExt_Genemark1.C_40430
ID=65248.2.0.227 annot-version=v2.0 Coccomyxa_subellipsoidea
OG0000035
66749 pacid=27391894 transcript=66749
locus=estExt_Genemark1.C_110195 ID=66749.2.0.227 annot-version=v2.0
Coccomyxa_subellipsoidea OG0000035
9815 pacid=27393555 transcript=9815 locus=gw1.2.733.1
ID=9815.2.0.227 annot-version=v2.0 Coccomyxa_subellipsoidea
OG0000035
16313_e_gw1.03.00.526.1 Ostreococcus_tauri OG0000035
17372_e_gw1.05.00.551.1 Ostreococcus_tauri OG0000035
19904_e_gw1.10.00.444.1 Ostreococcus_tauri OG0000035
33328_0600010175 Ostreococcus_tauri OG0000035
34581_0900010047 Ostreococcus_tauri OG0000035
7873_gw1.06.00.466.1 Ostreococcus_tauri OG0000035
7945_gw1.01.00.701.1 Ostreococcus_tauri OG0000035
36713_1500010009 Ostreococcus_tauri OG0000035
37546_1700010222 Ostreococcus_tauri OG0000035
9681 pacid=27413059 transcript=9681 locus=gwEuk.3.740.1

ID=9681.2.0.231 annot-version=v2.0   Ostreococcus_lucimarinus
OG0000035
92328 pacid=27414957 transcript=92328 locus=ost_02_020_123
ID=92328.2.0.231 annot-version=v2.0   Ostreococcus_lucimarinus
OG0000035
9736 pacid=27415797 transcript=9736 locus=gwEuk.16.326.1
ID=9736.2.0.231 annot-version=v2.0   Ostreococcus_lucimarinus
OG0000035
9940 pacid=27415994 transcript=9940 locus=gwEuk.16.334.1
ID=9940.2.0.231 annot-version=v2.0   Ostreococcus_lucimarinus
OG0000035
5385 pacid=27416082 transcript=5385 locus=gwEuk.10.294.1
ID=5385.2.0.231 annot-version=v2.0   Ostreococcus_lucimarinus
OG0000035
9611 pacid=27416770 transcript=9611 locus=gwEuk.5.582.1
ID=9611.2.0.231 annot-version=v2.0   Ostreococcus_lucimarinus
OG0000035
92168 pacid=27417512 transcript=92168 locus=ost_01_018_087
ID=92168.2.0.231 annot-version=v2.0   Ostreococcus_lucimarinus
OG0000035
9549 pacid=27418118 transcript=9549 locus=gwEuk.1.821.1
ID=9549.2.0.231 annot-version=v2.0   Ostreococcus_lucimarinus
OG0000035
9323 pacid=27418577 transcript=9323 locus=gwEuk.6.609.1
ID=9323.2.0.231 annot-version=v2.0   Ostreococcus_lucimarinus
OG0000035
42592 pacid=27419615 transcript=42592 locus=e_gwEuk.8.544.1
ID=42592.2.0.231 annot-version=v2.0   Ostreococcus_lucimarinus
OG0000035
9218 pacid=27413734 transcript=9218 locus=gwEuk.9.546.1
ID=9218.2.0.231 annot-version=v2.0   Ostreococcus_lucimarinus
OG0000035
11950 pacid=27349028 transcript=11950 locus=MicpuC2.gw1.12.524.1
ID=11950.3.0.228 annot-version=v3.0   Micromonas_pusilla
OG0000035
162388 pacid=27349256 transcript=162428 locus=e_gw1.12.239.1
ID=162428.3.0.228 annot-version=v3.0   Micromonas_pusilla
OG0000035
162152 pacid=27349276 transcript=162192 locus=e_gw1.12.673.1
ID=162192.3.0.228 annot-version=v3.0   Micromonas_pusilla
OG0000035
7992 pacid=27339737 transcript=7992 locus=MicpuC2.gw1.13.298.1
ID=7992.3.0.228 annot-version=v3.0   Micromonas_pusilla
OG0000035
9418 pacid=27340875 transcript=9418 locus=MicpuC2.gw1.7.507.1
ID=9418.3.0.228 annot-version=v3.0   Micromonas_pusilla
OG0000035
18620 pacid=27341086 transcript=18620 locus=MicpuC2.e_gw1.7.419.1
ID=18620.3.0.228 annot-version=v3.0   Micromonas_pusilla
OG0000035
158064 pacid=27341255 transcript=158104 locus=e_gw1.7.840.1
ID=158104.3.0.228 annot-version=v3.0   Micromonas_pusilla
OG0000035
8511 pacid=27338915 transcript=8511 locus=MicpuC2.gw1.8.447.1

ID=8511.3.0.228 annot-version=v3.0   Micromonas_pusilla
OG0000035
186221 pacid=27341483 transcript=186261 locus=gm1.3342_g
ID=186261.3.0.228 annot-version=v3.0   Micromonas_pusilla
OG0000035
23328 pacid=27343623 transcript=23328 locus=MicpuC2.e_gw1.16.358.1
ID=23328.3.0.228 annot-version=v3.0   Micromonas_pusilla
OG0000035
9952 pacid=27344853 transcript=9952 locus=MicpuC2.gw1.17.200.1
ID=9952.3.0.228 annot-version=v3.0   Micromonas_pusilla
OG0000035
161788 pacid=27339283 transcript=161828 locus=e_gw1.11.245.1
ID=161828.3.0.228 annot-version=v3.0   Micromonas_pusilla
OG0000035
49920 pacid=27345081 transcript=49920 locus=MicpuC2.EuGene.
0000010144 ID=49920.3.0.228 annot-version=v3.0
Micromonas_pusilla   OG0000035
51499 pacid=27339469 transcript=51499 locus=MicpuC2.EuGene.
0000110021 ID=51499.3.0.228 annot-version=v3.0
Micromonas_pusilla   OG0000035
11393 pacid=27347076 transcript=11393 locus=MicpuC2.gw1.3.1133.1
ID=11393.3.0.228 annot-version=v3.0   Micromonas_pusilla
OG0000035
32190 pacid=27347192 transcript=32190
locus=MicpuC2.estExt_Genewise1Plus.C_30298 ID=32190.3.0.228 annot-
version=v3.0   Micromonas_pusilla   OG0000035
46816 pacid=27347450 transcript=46816
locus=MicpuC2.estExt_fgenesh1_pg.C_30437 ID=46816.3.0.228 annot-
version=v3.0   Micromonas_pusilla   OG0000035
152611 pacid=27347735 transcript=152651 locus=e_gw1.3.372.1
ID=152651.3.0.228 annot-version=v3.0   Micromonas_pusilla
OG0000035
58613 pacid=27347912 transcript=58613 locus=MicpuC2.EuGene.
0000060122 ID=58613.3.0.228 annot-version=v3.0
Micromonas_pusilla   OG0000035
55641 pacid=27396889 transcript=55641 locus=EuGene.0200010018
ID=55641.3.0.229 annot-version=v3.0   Micromonas_sp._RCC299
OG0000035
89153 pacid=27396987 transcript=89153 locus=e_gw2.16.358.1
ID=89153.3.0.229 annot-version=v3.0   Micromonas_sp._RCC299
OG0000035
85945 pacid=27397446 transcript=85945 locus=e_gw2.10.715.1
ID=85945.3.0.229 annot-version=v3.0   Micromonas_sp._RCC299
OG0000035
81460 pacid=27395508 transcript=81460 locus=e_gw2.05.716.1
ID=81460.3.0.229 annot-version=v3.0   Micromonas_sp._RCC299
OG0000035
56682 pacid=27397841 transcript=56682 locus=EuGene.0300010110
ID=56682.3.0.229 annot-version=v3.0   Micromonas_sp._RCC299
OG0000035
77048 pacid=27397940 transcript=77048 locus=gw2.03.991.1
ID=77048.3.0.229 annot-version=v3.0   Micromonas_sp._RCC299
OG0000035
107520 pacid=27398848 transcript=107520

locus=est_cluster_kg.Chr_01__84__3200701:1 ID=107520.3.0.229 annot-version=v3.0 Micromonas_sp._RCC299 OG0000035
55086 pacid=27398874 transcript=55086 locus=EuGene.0100010313 ID=55086.3.0.229 annot-version=v3.0 Micromonas_sp._RCC299 OG0000035
74892 pacid=27399820 transcript=74892 locus=gw2.12.492.1 ID=74892.3.0.229 annot-version=v3.0 Micromonas_sp._RCC299 OG0000035
57480 pacid=27400641 transcript=57480 locus=EuGene.0400010009 ID=57480.3.0.229 annot-version=v3.0 Micromonas_sp._RCC299 OG0000035
74177 pacid=27401431 transcript=74177 locus=gw2.09.585.1 ID=74177.3.0.229 annot-version=v3.0 Micromonas_sp._RCC299 OG0000035
107385 pacid=27401484 transcript=107385 locus=PierreAnnot_DnaJ ID=107385.3.0.229 annot-version=v3.0 Micromonas_sp._RCC299 OG0000035
61253 pacid=27401729 transcript=61253 locus=EuGene.0900010236 ID=61253.3.0.229 annot-version=v3.0 Micromonas_sp._RCC299 OG0000035
59428 pacid=27401830 transcript=59428 locus=EuGene.0600010430 ID=59428.3.0.229 annot-version=v3.0 Micromonas_sp._RCC299 OG0000035
100480 pacid=27396007 transcript=100480 locus=fgenesh2_pg.C_Chr_05000173 ID=100480.3.0.229 annot-version=v3.0 Micromonas_sp._RCC299 OG0000035
60534 pacid=27403628 transcript=60534 locus=EuGene.0800010102 ID=60534.3.0.229 annot-version=v3.0 Micromonas_sp._RCC299 OG0000035
109466 pacid=27403691 transcript=109466 locus=est_cluster_kg.Chr_14__42__3200423:1 ID=109466.3.0.229 annot-version=v3.0 Micromonas_sp._RCC299 OG0000035
74160 pacid=27403705 transcript=74160 locus=gw2.14.401.1 ID=74160.3.0.229 annot-version=v3.0 Micromonas_sp._RCC299 OG0000035
kfl00018_0370_v1.1 Klebsormidium_flaccidum OG0000035
kfl00042_0120_v1.1 Klebsormidium_flaccidum OG0000035
kfl00173_0320_v1.1 Klebsormidium_flaccidum OG0000035
kfl00175_0160_v1.1 Klebsormidium_flaccidum OG0000035
kfl00011_0120_v1.1 Klebsormidium_flaccidum OG0000035
kfl00304_0110_v1.1 Klebsormidium_flaccidum OG0000035
Pp3c7_20090V3.1.p pacid=32925992 transcript=Pp3c7_20090V3.1 locus=Pp3c7_20090 annot-version=v3.3 Physcomitrella_patens OG0000035
Pp3c19_10460V3.1.p pacid=32939029 transcript=Pp3c19_10460V3.1 locus=Pp3c19_10460 annot-version=v3.3 Physcomitrella_patens OG0000035
Pp3c22_22180V3.1.p pacid=32903497 transcript=Pp3c22_22180V3.1 locus=Pp3c22_22180 annot-version=v3.3 Physcomitrella_patens OG0000035
Pp3c3_19970V3.1.p pacid=32941799 transcript=Pp3c3_19970V3.1 locus=Pp3c3_19970 annot-version=v3.3 Physcomitrella_patens OG0000035
Pp3c3_35390V3.1.p pacid=32944029 transcript=Pp3c3_35390V3.1

locus=Pp3c3_35390 annot-version=v3.3	Physcomitrella_patens
OG0000035
Pp3c22_22070V3.1.p pacid=32904035 transcript=Pp3c22_22070V3.1
locus=Pp3c22_22070 annot-version=v3.3	Physcomitrella_patens
OG0000035
Pp3c23_1890V3.1.p pacid=32949709 transcript=Pp3c23_1890V3.1
locus=Pp3c23_1890 annot-version=v3.3	Physcomitrella_patens
OG0000035
Pp3c23_10390V3.1.p pacid=32951011 transcript=Pp3c23_10390V3.1
locus=Pp3c23_10390 annot-version=v3.3	Physcomitrella_patens
OG0000035
Pp3c22_22330V3.1.p pacid=32904906 transcript=Pp3c22_22330V3.1
locus=Pp3c22_22330 annot-version=v3.3	Physcomitrella_patens
OG0000035
Pp3c14_21010V3.1.p pacid=32960230 transcript=Pp3c14_21010V3.1
locus=Pp3c14_21010 annot-version=v3.3	Physcomitrella_patens
OG0000035
Pp3c14_16570V3.1.p pacid=32963245 transcript=Pp3c14_16570V3.1
locus=Pp3c14_16570 annot-version=v3.3	Physcomitrella_patens
OG0000035
Pp3c8_2480V3.1.p pacid=32965934 transcript=Pp3c8_2480V3.1
locus=Pp3c8_2480 annot-version=v3.3	Physcomitrella_patens
OG0000035
Pp3c12_8390V3.1.p pacid=32972666 transcript=Pp3c12_8390V3.1
locus=Pp3c12_8390 annot-version=v3.3	Physcomitrella_patens
OG0000035
Pp3c12_16420V3.1.p pacid=32972675 transcript=Pp3c12_16420V3.1
locus=Pp3c12_16420 annot-version=v3.3	Physcomitrella_patens
OG0000035
Pp3c12_15530V3.1.p pacid=32974884 transcript=Pp3c12_15530V3.1
locus=Pp3c12_15530 annot-version=v3.3	Physcomitrella_patens
OG0000035
Pp3c17_19540V3.1.p pacid=32907107 transcript=Pp3c17_19540V3.1
locus=Pp3c17_19540 annot-version=v3.3	Physcomitrella_patens
OG0000035
Pp3c18_2160V3.1.p pacid=32981209 transcript=Pp3c18_2160V3.1
locus=Pp3c18_2160 annot-version=v3.3	Physcomitrella_patens
OG0000035
Pp3c18_3800V3.1.p pacid=32981801 transcript=Pp3c18_3800V3.1
locus=Pp3c18_3800 annot-version=v3.3	Physcomitrella_patens
OG0000035
Pp3c18_17490V3.1.p pacid=32982232 transcript=Pp3c18_17490V3.1
locus=Pp3c18_17490 annot-version=v3.3	Physcomitrella_patens
OG0000035
Pp3c18_3780V3.1.p pacid=32982335 transcript=Pp3c18_3780V3.1
locus=Pp3c18_3780 annot-version=v3.3	Physcomitrella_patens
OG0000035
Pp3c18_3720V3.1.p pacid=32983429 transcript=Pp3c18_3720V3.1
locus=Pp3c18_3720 annot-version=v3.3	Physcomitrella_patens
OG0000035
Pp3c10_15440V3.1.p pacid=32899472 transcript=Pp3c10_15440V3.1
locus=Pp3c10_15440 annot-version=v3.3	Physcomitrella_patens
OG0000035
Pp3c9_17410V3.1.p pacid=32913145 transcript=Pp3c9_17410V3.1

locus=Pp3c9_17410 annot-version=v3.3	Physcomitrella_patens	OG0000035
Pp3c26_5100V3.1.p pacid=32918521 transcript=Pp3c26_5100V3.1 locus=Pp3c26_5100 annot-version=v3.3	Physcomitrella_patens	OG0000035
Pp3c4_11750V3.1.p pacid=32919163 transcript=Pp3c4_11750V3.1 locus=Pp3c4_11750 annot-version=v3.3	Physcomitrella_patens	OG0000035
Pp3c4_11330V3.1.p pacid=32919877 transcript=Pp3c4_11330V3.1 locus=Pp3c4_11330 annot-version=v3.3	Physcomitrella_patens	OG0000035
Pp3c4_1450V3.1.p pacid=32921614 transcript=Pp3c4_1450V3.1 locus=Pp3c4_1450 annot-version=v3.3	Physcomitrella_patens	OG0000035
Pp3c4_26000V3.1.p pacid=32922681 transcript=Pp3c4_26000V3.1 locus=Pp3c4_26000 annot-version=v3.3	Physcomitrella_patens	OG0000035
Pp3c7_20040V3.1.p pacid=32924685 transcript=Pp3c7_20040V3.1 locus=Pp3c7_20040 annot-version=v3.3	Physcomitrella_patens	OG0000035
AT2G41000.1 | Chaperone DnaJ-domain superfamily protein | Chr2:17111673-17113247 FORWARD LENGTH=184 | 201606	Arabidopsis_thaliana	OG0000035
AT3G06778.1 | Chaperone DnaJ-domain superfamily protein | Chr3:2140249-2141437 REVERSE LENGTH=229 | 201606	Arabidopsis_thaliana	OG0000035
AT3G13310.1 | Chaperone DnaJ-domain superfamily protein | Chr3:4310827-4311300 REVERSE LENGTH=157 | 201606	Arabidopsis_thaliana	OG0000035
AT3G14200.1 | Chaperone DnaJ-domain superfamily protein | Chr3:4712881-4714361 REVERSE LENGTH=230 | 201606	Arabidopsis_thaliana	OG0000035
AT3G57340.1 | DnaJ heat shock amino-terminal domain protein (DUF1977) | Chr3:21219175-21220278 FORWARD LENGTH=367 | 201606	Arabidopsis_thaliana	OG0000035
AT4G10130.1 | DNAJ heat shock N-terminal domain-containing protein | Chr4:6320959-6321483 REVERSE LENGTH=174 | 201606	Arabidopsis_thaliana	OG0000035
AT4G13830.1 | DNAJ-like 20 | Chr4:8011518-8011985 FORWARD LENGTH=155 | 201606 Arabidopsis_thaliana	OG0000035
AT4G36040.1 | Chaperone DnaJ-domain superfamily protein | Chr4:17049708-17050193 REVERSE LENGTH=161 | 201606	Arabidopsis_thaliana	OG0000035
AT5G05750.1 | DNAJ heat shock N-terminal domain-containing protein | Chr5:1727595-1728479 FORWARD LENGTH=294 | 201606	Arabidopsis_thaliana	OG0000035
AT5G16650.1 | Chaperone DnaJ-domain superfamily protein | Chr5:5463446-5465075 REVERSE LENGTH=128 | 201606	Arabidopsis_thaliana	OG0000035
AT5G37760.1 | Chaperone DnaJ-domain superfamily protein | Chr5:14997168-14998219 FORWARD LENGTH=207 | 201606	Arabidopsis_thaliana	OG0000035
AT5G49060.1 | DnaJ heat shock amino-terminal domain protein (DUF1977) | Chr5:19886764-19888136 FORWARD LENGTH=354 | 201606

Arabidopsis_thaliana   OG0000035
AT5G59610.1 | Chaperone DnaJ-domain superfamily protein | Chr5:24013165-24014954 FORWARD LENGTH=268 | 201606
Arabidopsis_thaliana   OG0000035
AT1G56300.1 | Chaperone DnaJ-domain superfamily protein | Chr1:21079022-21080168 REVERSE LENGTH=156 | 201606
Arabidopsis_thaliana   OG0000035
AT1G71000.1 | Chaperone DnaJ-domain superfamily protein | Chr1:26769336-26770111 REVERSE LENGTH=165 | 201606
Arabidopsis_thaliana   OG0000035
AT1G72070.1 | Chaperone DnaJ-domain superfamily protein | Chr1:27118851-27119608 REVERSE LENGTH=126 | 201606
Arabidopsis_thaliana   OG0000035
AT1G72416.1 | Chaperone DnaJ-domain superfamily protein | Chr1:27258995-27260109 REVERSE LENGTH=187 | 201606
Arabidopsis_thaliana   OG0000035
AT1G80920.1 | Chaperone DnaJ-domain superfamily protein | Chr1:30403863-30404549 REVERSE LENGTH=163 | 201606
Arabidopsis_thaliana   OG0000035
AT2G17880.1 | Chaperone DnaJ-domain superfamily protein | Chr2:7767176-7767658 REVERSE LENGTH=160 | 201606
Arabidopsis_thaliana   OG0000035
AT2G33735.1 | Chaperone DnaJ-domain superfamily protein | Chr2:14268532-14269394 REVERSE LENGTH=119 | 201606
Arabidopsis_thaliana   OG0000035
Os03t0244950-00 Heat shock protein DnaJ, N-terminal domain containing protein.   Oryza_sativa   OG0000035
Os03t0300600-01 Similar to Chaperone protein dnaJ. Oryza_sativa OG0000035
Os03t0822800-01 Similar to Chaperone protein dnaJ 1. Oryza_sativa   OG0000035
Os03t0831000-00 Hypothetical conserved gene.   Oryza_sativa OG0000035
Os01t0101700-00 Similar to chaperone protein dnaJ 20. Oryza_sativa   OG0000035
Os05t0364500-01 Heat shock protein DnaJ, N-terminal domain containing protein.   Oryza_sativa   OG0000035
Os06t0195800-01 Similar to DNAJ heat shock N-terminal domain-containing protein.   Oryza_sativa   OG0000035
Os01t0556400-01 Heat shock protein DnaJ family protein. Oryza_sativa   OG0000035
Os06t0716100-01 Similar to Chaperone protein dnaJ. Oryza_sativa OG0000035
Os01t0606900-01 Heat shock protein DnaJ, N-terminal domain containing protein.   Oryza_sativa   OG0000035
Os07t0637150-01 Similar to DnaJ domain containing protein. Oryza_sativa   OG0000035
Os08t0474600-01 Heat shock protein DnaJ, N-terminal domain containing protein.   Oryza_sativa   OG0000035
Os08t0548400-00 Similar to chaperone protein dnaJ 11. Oryza_sativa   OG0000035
Os09t0460000-01 Heat shock protein DnaJ, N-terminal domain containing protein.   Oryza_sativa   OG0000035
Os02t0693200-01 Similar to Chaperone protein dnaJ. Oryza_sativa

OG0000035
Os02t0760000-00 Similar to chaperone protein dnaJ. Oryza_sativa
OG0000035
CMD162CT pep chromosome:ASM9120v1:4:430298:430634:-1 gene:CMD162C
transcript:CMD162CT gene_biotype:protein_coding
transcript_biotype:protein_coding Cyanidioschyzon_merolae
OG0000035
CMT623CT pep chromosome:ASM9120v1:20:1585952:1587457:1 gene:CMT623C
transcript:CMT623CT gene_biotype:protein_coding
transcript_biotype:protein_coding Cyanidioschyzon_merolae
OG0000035
CMI136CT pep chromosome:ASM9120v1:9:377346:379325:1 gene:CMI136C
transcript:CMI136CT gene_biotype:protein_coding
transcript_biotype:protein_coding Cyanidioschyzon_merolae
OG0000035
CMB036CT pep chromosome:ASM9120v1:2:91240:92055:1 gene:CMB036C
transcript:CMB036CT gene_biotype:protein_coding
transcript_biotype:protein_coding Cyanidioschyzon_merolae
OG0000035
CMF076CT pep chromosome:ASM9120v1:6:198261:199043:1 gene:CMF076C
transcript:CMF076CT gene_biotype:protein_coding
transcript_biotype:protein_coding Cyanidioschyzon_merolae
OG0000035
CMD125CT pep chromosome:ASM9120v1:4:326284:327354:1 gene:CMD125C
transcript:CMD125CT gene_biotype:protein_coding
transcript_biotype:protein_coding Cyanidioschyzon_merolae
OG0000035
evm.model.contig_2090.41 Porphyridium_purpureum OG0000035
evm.model.contig_2174.5 Porphyridium_purpureum OG0000035
evm.model.contig_3442.2 Porphyridium_purpureum OG0000035
evm.model.contig_3654.1 Porphyridium_purpureum OG0000035
evm.model.contig_4410.12 Porphyridium_purpureum OG0000035
evm.model.contig_4511.2 Porphyridium_purpureum OG0000035
evm.model.contig_4519.1 Porphyridium_purpureum OG0000035
evm.model.contig_554.5 Porphyridium_purpureum OG0000035
evm.model.contig_602.2 Porphyridium_purpureum OG0000035
evm.model.contig_700.2 Porphyridium_purpureum OG0000035
CDF37337 pep supercontig:ASM35022v2:HG001832:36187:38047:-1
gene:CHC_T00005482001 transcript:CDF37337
gene_biotype:protein_coding transcript_biotype:protein_coding
Chondrus_crispus OG0000035
CDF35288 pep supercontig:ASM35022v2:HG001726:107343:108778:-1
gene:CHC_T00003939001 transcript:CDF35288
gene_biotype:protein_coding transcript_biotype:protein_coding
Chondrus_crispus OG0000035
CDF39803 pep supercontig:ASM35022v2:HG002074:39592:42412:-1
gene:CHC_T00000375001 transcript:CDF39803
gene_biotype:protein_coding transcript_biotype:protein_coding
Chondrus_crispus OG0000035
CDF39941 pep supercontig:ASM35022v2:HG002094:157742:158344:1
gene:CHC_T00006901001 transcript:CDF39941
gene_biotype:protein_coding transcript_biotype:protein_coding
Chondrus_crispus OG0000035
g2939.t1 Caulerpa_lentillifera OG0000036

9290_fgenesh1_pg.C_Chr_01.0001000056	Ostreococcus_tauri
OG0000036
28702 pacid=27417784 transcript=28702 locus=eugene.0100010096
ID=28702.2.0.231 annot-version=v2.0	Ostreococcus_lucimarinus
OG0000036
123940 pacid=27339312 transcript=123980 locus=fgenesh2_kg.
11_#_51_#_4271625:1 ID=123980.3.0.228 annot-version=v3.0
Micromonas_pusilla	OG0000036
60191 pacid=27404243 transcript=60191 locus=EuGene.0700010476
ID=60191.3.0.229 annot-version=v3.0	Micromonas_sp._RCC299
OG0000036
kfl00513_0110_v1.1	Klebsormidium_flaccidum	OG0000036
kfl00047_0300_v1.1	Klebsormidium_flaccidum	OG0000036
kfl00070_0280_v1.1	Klebsormidium_flaccidum	OG0000036
kfl00010_0070_v1.1	Klebsormidium_flaccidum	OG0000036
kfl00302_0150_v1.1	Klebsormidium_flaccidum	OG0000036
Pp3c7_26870V3.1.p pacid=32926111 transcript=Pp3c7_26870V3.1
locus=Pp3c7_26870 annot-version=v3.3	Physcomitrella_patens
OG0000036
Pp3c2_29140V3.1.p pacid=32934892 transcript=Pp3c2_29140V3.1
locus=Pp3c2_29140 annot-version=v3.3	Physcomitrella_patens
OG0000036
Pp3c22_4140V3.1.p pacid=32903579 transcript=Pp3c22_4140V3.1
locus=Pp3c22_4140 annot-version=v3.3	Physcomitrella_patens
OG0000036
Pp3c3_250V3.1.p pacid=32943997 transcript=Pp3c3_250V3.1
locus=Pp3c3_250 annot-version=v3.3	Physcomitrella_patens
OG0000036
Pp3c22_3160V3.1.p pacid=32904148 transcript=Pp3c22_3160V3.1
locus=Pp3c22_3160 annot-version=v3.3	Physcomitrella_patens
OG0000036
Pp3c27_3140V3.1.p pacid=32951593 transcript=Pp3c27_3140V3.1
locus=Pp3c27_3140 annot-version=v3.3	Physcomitrella_patens
OG0000036
Pp3c27_8400V3.1.p pacid=32952010 transcript=Pp3c27_8400V3.1
locus=Pp3c27_8400 annot-version=v3.3	Physcomitrella_patens
OG0000036
Pp3c27_3130V3.1.p pacid=32952045 transcript=Pp3c27_3130V3.1
locus=Pp3c27_3130 annot-version=v3.3	Physcomitrella_patens
OG0000036
Pp3c27_3230V3.1.p pacid=32952458 transcript=Pp3c27_3230V3.1
locus=Pp3c27_3230 annot-version=v3.3	Physcomitrella_patens
OG0000036
Pp3c5_8420V3.1.p pacid=32955752 transcript=Pp3c5_8420V3.1
locus=Pp3c5_8420 annot-version=v3.3	Physcomitrella_patens
OG0000036
Pp3c14_6190V3.1.p pacid=32960531 transcript=Pp3c14_6190V3.1
locus=Pp3c14_6190 annot-version=v3.3	Physcomitrella_patens
OG0000036
Pp3c16_22890V3.1.p pacid=32985319 transcript=Pp3c16_22890V3.1
locus=Pp3c16_22890 annot-version=v3.3	Physcomitrella_patens
OG0000036
Pp3c16_11740V3.1.p pacid=32985720 transcript=Pp3c16_11740V3.1
locus=Pp3c16_11740 annot-version=v3.3	Physcomitrella_patens

OG0000036
Pp3c24_11730V3.1.p pacid=32910113 transcript=Pp3c24_11730V3.1 locus=Pp3c24_11730 annot-version=v3.3 Physcomitrella_patens OG0000036
Pp3c21_8270V3.1.p pacid=32916799 transcript=Pp3c21_8270V3.1 locus=Pp3c21_8270 annot-version=v3.3 Physcomitrella_patens OG0000036
AT2G39980.1 | HXXXD-type acyl-transferase family protein | Chr2:16688437-16689885 REVERSE LENGTH=482 | 201606 Arabidopsis_thaliana OG0000036
AT2G40230.1 | HXXXD-type acyl-transferase family protein | Chr2:16803285-16804586 REVERSE LENGTH=433 | 201606 Arabidopsis_thaliana OG0000036
AT3G03480.1 | acetyl CoA:(Z)-3-hexen-1-ol acetyltransferase | Chr3:828400-829863 REVERSE LENGTH=454 | 201606 Arabidopsis_thaliana OG0000036
AT3G26040.1 | HXXXD-type acyl-transferase family protein | Chr3:9519741-9521069 FORWARD LENGTH=442 | 201606 Arabidopsis_thaliana OG0000036
AT3G29590.1 | HXXXD-type acyl-transferase family protein | Chr3:11398917-11400266 REVERSE LENGTH=449 | 201606 Arabidopsis_thaliana OG0000036
AT3G29635.1 | HXXXD-type acyl-transferase family protein | Chr3:11465851-11467227 REVERSE LENGTH=458 | 201606 Arabidopsis_thaliana OG0000036
AT3G29636.1 | transferase-like protein | Chr3:11467964-11468857 REVERSE LENGTH=199 | 201606 Arabidopsis_thaliana OG0000036
AT3G29670.1 | HXXXD-type acyl-transferase family protein | Chr3:11527952-11529307 FORWARD LENGTH=451 | 201606 Arabidopsis_thaliana OG0000036
AT3G29680.1 | HXXXD-type acyl-transferase family protein | Chr3:11531449-11532804 REVERSE LENGTH=451 | 201606 Arabidopsis_thaliana OG0000036
AT3G29690.1 | HXXXD-type acyl-transferase family protein | Chr3:11540770-11541549 REVERSE LENGTH=180 | 201606 Arabidopsis_thaliana OG0000036
AT3G29720.1 | HXXXD-type acyl-transferase family protein | Chr3:11549418-11551369 REVERSE LENGTH=220 | 201606 Arabidopsis_thaliana OG0000036
AT3G30280.1 | HXXXD-type acyl-transferase family protein | Chr3:11914366-11915697 FORWARD LENGTH=443 | 201606 Arabidopsis_thaliana OG0000036
AT3G47170.1 | HXXXD-type acyl-transferase family protein | Chr3:17368672-17370494 REVERSE LENGTH=468 | 201606 Arabidopsis_thaliana OG0000036
AT3G48720.1 | HXXXD-type acyl-transferase family protein | Chr3:18046527-18049295 FORWARD LENGTH=430 | 201606 Arabidopsis_thaliana OG0000036
AT3G50270.1 | HXXXD-type acyl-transferase family protein | Chr3:18635926-18637278 FORWARD LENGTH=450 | 201606 Arabidopsis_thaliana OG0000036
AT3G50280.1 | HXXXD-type acyl-transferase family protein | Chr3:18637854-18639185 FORWARD LENGTH=443 | 201606

Arabidopsis_thaliana   OG0000036
AT3G50290.1 | HXXXD-type acyl-transferase family protein |
Chr3:18640172-18640993 FORWARD LENGTH=273 | 201606
Arabidopsis_thaliana   OG0000036
AT3G50300.1 | HXXXD-type acyl-transferase family protein |
Chr3:18644043-18645389 FORWARD LENGTH=448 | 201606
Arabidopsis_thaliana   OG0000036
AT3G62160.1 | HXXXD-type acyl-transferase family protein |
Chr3:23014333-23015808 REVERSE LENGTH=428 | 201606
Arabidopsis_thaliana   OG0000036
AT4G15390.1 | HXXXD-type acyl-transferase family protein |
Chr4:8792941-8794281 REVERSE LENGTH=446 | 201606
Arabidopsis_thaliana   OG0000036
AT4G15400.1 | HXXXD-type acyl-transferase family protein |
Chr4:8812121-8813428 REVERSE LENGTH=435 | 201606
Arabidopsis_thaliana   OG0000036
AT4G31910.1 | HXXXD-type acyl-transferase family protein |
Chr4:15441125-15443696 FORWARD LENGTH=458 | 201606
Arabidopsis_thaliana   OG0000036
AT5G01210.1 | HXXXD-type acyl-transferase family protein |
Chr5:84554-85981 FORWARD LENGTH=475 | 201606
Arabidopsis_thaliana   OG0000036
AT5G07080.1 | HXXXD-type acyl-transferase family protein |
Chr5:2200353-2201975 FORWARD LENGTH=450 | 201606
Arabidopsis_thaliana   OG0000036
AT5G07850.1 | HXXXD-type acyl-transferase family protein |
Chr5:2508917-2510503 FORWARD LENGTH=456 | 201606
Arabidopsis_thaliana   OG0000036
AT5G07860.1 | HXXXD-type acyl-transferase family protein |
Chr5:2511449-2512976 FORWARD LENGTH=454 | 201606
Arabidopsis_thaliana   OG0000036
AT5G07870.1 | HXXXD-type acyl-transferase family protein |
Chr5:2513864-2515351 FORWARD LENGTH=464 | 201606
Arabidopsis_thaliana   OG0000036
AT5G16410.1 | HXXXD-type acyl-transferase family protein |
Chr5:5365685-5367386 REVERSE LENGTH=481 | 201606
Arabidopsis_thaliana   OG0000036
AT5G17540.1 | HXXXD-type acyl-transferase family protein |
Chr5:5782061-5783682 REVERSE LENGTH=461 | 201606
Arabidopsis_thaliana   OG0000036
AT5G23940.1 | HXXXD-type acyl-transferase family protein |
Chr5:8076616-8079677 REVERSE LENGTH=484 | 201606
Arabidopsis_thaliana   OG0000036
AT5G23970.1 | HXXXD-type acyl-transferase family protein |
Chr5:8096326-8097612 FORWARD LENGTH=428 | 201606
Arabidopsis_thaliana   OG0000036
AT5G38130.1 | HXXXD-type acyl-transferase family protein |
Chr5:15217956-15219344 REVERSE LENGTH=462 | 201606
Arabidopsis_thaliana   OG0000036
AT5G39050.1 | HXXXD-type acyl-transferase family protein |
Chr5:15634596-15636005 FORWARD LENGTH=469 | 201606
Arabidopsis_thaliana   OG0000036
AT5G39080.1 | HXXXD-type acyl-transferase family protein |
Chr5:15641681-15643072 FORWARD LENGTH=463 | 201606

Arabidopsis_thaliana   OG0000036
AT5G39090.1 | HXXXD-type acyl-transferase family protein | Chr5:15643612-15644958 FORWARD LENGTH=448 | 201606   Arabidopsis_thaliana   OG0000036
AT5G41040.1 | HXXXD-type acyl-transferase family protein | Chr5:16431448-16433086 FORWARD LENGTH=457 | 201606   Arabidopsis_thaliana   OG0000036
AT5G42830.1 | HXXXD-type acyl-transferase family protein | Chr5:17176384-17177906 FORWARD LENGTH=450 | 201606   Arabidopsis_thaliana   OG0000036
AT1G24420.1 | HXXXD-type acyl-transferase family protein | Chr1:8656687-8657997 FORWARD LENGTH=436 | 201606   Arabidopsis_thaliana   OG0000036
AT1G24430.1 | HXXXD-type acyl-transferase family protein | Chr1:8658190-8659497 REVERSE LENGTH=435 | 201606   Arabidopsis_thaliana   OG0000036
AT5G47950.1 | HXXXD-type acyl-transferase family protein | Chr5:19417218-19418498 REVERSE LENGTH=426 | 201606   Arabidopsis_thaliana   OG0000036
AT5G47980.1 | HXXXD-type acyl-transferase family protein | Chr5:19428970-19430301 FORWARD LENGTH=443 | 201606   Arabidopsis_thaliana   OG0000036
AT5G48930.1 | hydroxycinnamoyl-CoA shikimate/quinate hydroxycinnamoyl transferase | Chr5:19836654-19838092 REVERSE LENGTH=433 | 201606   Arabidopsis_thaliana   OG0000036
AT1G03390.1 | HXXXD-type acyl-transferase family protein | Chr1:841033-842418 REVERSE LENGTH=461 | 201606   Arabidopsis_thaliana   OG0000036
AT5G57840.1 | HXXXD-type acyl-transferase family protein | Chr5:23432945-23435161 REVERSE LENGTH=443 | 201606   Arabidopsis_thaliana   OG0000036
AT5G61160.1 | anthocyanin 5-aromatic acyltransferase 1 | Chr5:24608781-24610139 FORWARD LENGTH=452 | 201606   Arabidopsis_thaliana   OG0000036
AT5G63560.1 | HXXXD-type acyl-transferase family protein | Chr5:25449517-25451234 FORWARD LENGTH=426 | 201606   Arabidopsis_thaliana   OG0000036
AT5G67150.1 | HXXXD-type acyl-transferase family protein | Chr5:26795880-26797226 REVERSE LENGTH=448 | 201606   Arabidopsis_thaliana   OG0000036
AT5G67160.1 | HXXXD-type acyl-transferase family protein | Chr5:26797700-26799004 REVERSE LENGTH=434 | 201606   Arabidopsis_thaliana   OG0000036
AT1G27620.1 | HXXXD-type acyl-transferase family protein | Chr1:9608444-9610432 FORWARD LENGTH=442 | 201606   Arabidopsis_thaliana   OG0000036
AT1G28680.1 | HXXXD-type acyl-transferase family protein | Chr1:10078222-10079763 FORWARD LENGTH=451 | 201606   Arabidopsis_thaliana   OG0000036
AT1G31490.1 | HXXXD-type acyl-transferase family protein | Chr1:11271744-11273078 REVERSE LENGTH=444 | 201606   Arabidopsis_thaliana   OG0000036
AT1G32910.1 | HXXXD-type acyl-transferase family protein | Chr1:11925426-11926908 FORWARD LENGTH=464 | 201606

Arabidopsis_thaliana   OG0000036
AT1G65450.1 | HXXXD-type acyl-transferase family protein | Chr1:24315875-24318895 FORWARD LENGTH=450 | 201606
Arabidopsis_thaliana   OG0000036
AT1G78990.1 | HXXXD-type acyl-transferase family protein | Chr1:29713531-29714969 REVERSE LENGTH=455 | 201606
Arabidopsis_thaliana   OG0000036
AT2G19070.1 | spermidine hydroxycinnamoyl transferase | Chr2:8260185-8261958 REVERSE LENGTH=451 | 201606
Arabidopsis_thaliana   OG0000036
AT2G23510.1 | spermidine disinapoyl acyltransferase | Chr2:10011517-10013533 REVERSE LENGTH=451 | 201606
Arabidopsis_thaliana   OG0000036
AT2G25150.1 | HXXXD-type acyl-transferase family protein | Chr2:10702582-10704266 REVERSE LENGTH=461 | 201606
Arabidopsis_thaliana   OG0000036
Os03t0446200-00 Hypothetical conserved gene.   Oryza_sativa OG0000036
Os01t0261600-00 Similar to agmatine coumaroyltransferase. Oryza_sativa   OG0000036
Os01t0261700-01 Similar to Agmatine coumaroyltransferase. Oryza_sativa   OG0000036
Os01t0262000-00 Similar to agmatine coumaroyltransferase. Oryza_sativa   OG0000036
Os04t0172400-01 Transferase family protein.   Oryza_sativa OG0000036
Os04t0175500-00 Transferase domain containing protein. Oryza_sativa   OG0000036
Os04t0194466-00 Transferase domain containing protein. Oryza_sativa   OG0000036
Os01t0289900-00 Transferase domain containing protein. Oryza_sativa   OG0000036
Os01t0291500-01 Transferase family protein.   Oryza_sativa OG0000036
Os04t0374400-00 Hypothetical conserved gene.   Oryza_sativa OG0000036
Os04t0374601-00 Transferase domain containing protein. Oryza_sativa   OG0000036
Os04t0500700-01 Similar to Hydroxyanthranilate hydroxycinnamoyltransferase 3.   Oryza_sativa   OG0000036
Os04t0507400-00 Similar to OSIGBa0157A06.5 protein. Oryza_sativa   OG0000036
Os04t0606000-01 Transferase family protein.   Oryza_sativa OG0000036
Os04t0609300-01 Transferase family protein.   Oryza_sativa OG0000036
Os04t0609500-00 Transferase family protein.   Oryza_sativa OG0000036
Os04t0638100-01 Transferase family protein.   Oryza_sativa OG0000036
Os04t0638401-00 Similar to transferase.   Oryza_sativa OG0000036
Os01t0350000-01 Transferase family protein.   Oryza_sativa OG0000036

Os04t0664500-01 Similar to H1005F08.2 protein.	Oryza_sativa
OG0000036
Os04t0664600-00 Similar to H1005F08.3 protein.	Oryza_sativa
OG0000036
Os05t0116800-00 Transferase domain containing protein.
Oryza_sativa	OG0000036
Os05t0136900-01 Transferase family protein.	Oryza_sativa
OG0000036
Os05t0141300-00 Transferase family protein.	Oryza_sativa
OG0000036
Os05t0155800-00 Similar to OSIGBa0157A06.5 protein.
Oryza_sativa	OG0000036
Os05t0179300-01 Transferase family protein.	Oryza_sativa
OG0000036
Os05t0267850-00 Similar to Agmatine coumaroyltransferase-1.
Oryza_sativa	OG0000036
Os05t0278500-01 Transferase family protein.	Oryza_sativa
OG0000036
Os05t0289700-01 Arbuscular mycorrhizal specific marker 10.,Benzyl alcohol benzoyl transferase.	Oryza_sativa	OG0000036
Os05t0315700-00 Transferase domain containing protein.
Oryza_sativa	OG0000036
Os01t0382200-01 Transferase family protein.	Oryza_sativa
OG0000036
Os05t0449200-01 Transferase family protein.	Oryza_sativa
OG0000036
Os06t0103200-01 Transferase family protein.	Oryza_sativa
OG0000036
Os06t0145200-01 Transferase family protein.	Oryza_sativa
OG0000036
Os06t0145400-00 Transferase domain containing protein.
Oryza_sativa	OG0000036
Os06t0145600-01 Transferase family protein.	Oryza_sativa
OG0000036
Os06t0150600-01 Transferase family protein.	Oryza_sativa
OG0000036
Os06t0151100-00 Similar to Transferase.	Oryza_sativa
OG0000036
Os06t0155500-00 Transferase family protein.	Oryza_sativa
OG0000036
Os06t0184900-01 Transferase family protein.	Oryza_sativa
OG0000036
Os06t0185300-01 Transferase family protein.	Oryza_sativa
OG0000036
Os06t0185500-00 Transferase family protein.	Oryza_sativa
OG0000036
Os06t0594600-01 BAHD acyltransferase, Glucuronoarabinoxylan modification in grass cell wall	Oryza_sativa	OG0000036
Os06t0595800-01 Transferase family protein.	Oryza_sativa
OG0000036
Os06t0699100-00 Transferase domain containing protein.
Oryza_sativa	OG0000036
Os06t0710700-01 Similar to Benzoyl coenzyme A	Oryza_sativa
OG0000036

Os07t0142700-00 Transferase family protein.	Oryza_sativa	OG0000036
Os07t0244200-01 Transferase family protein.	Oryza_sativa	OG0000036
Os07t0244600-00 Similar to Agmatine coumaroyltransferase.	Oryza_sativa	OG0000036
Os07t0414000-01 Transferase domain containing protein.	Oryza_sativa	OG0000036
Os07t0421600-00 Similar to transferase family protein.	Oryza_sativa	OG0000036
Os07t0550600-01 Transferase family protein.	Oryza_sativa	OG0000036
Os01t0615200-01 Transferase family protein.	Oryza_sativa	OG0000036
Os01t0615300-01 Transferase family protein.	Oryza_sativa	OG0000036
Os08t0111300-01 Transferase family protein.	Oryza_sativa	OG0000036
Os08t0111500-00 Hypothetical conserved gene.	Oryza_sativa	OG0000036
Os08t0111800-00 Transferase family protein.	Oryza_sativa	OG0000036
Os08t0112300-01 Transferase domain containing protein.	Oryza_sativa	OG0000036
Os08t0174100-00 Similar to transferase family protein.	Oryza_sativa	OG0000036
Os08t0174300-00 Transferase domain containing protein.	Oryza_sativa	OG0000036
Os08t0205000-00 Transferase domain containing protein.	Oryza_sativa	OG0000036
Os08t0331100-00 Chloramphenicol acetyltransferase-like domain domain containing protein.	Oryza_sativa	OG0000036
Os08t0543400-01 Transferase family protein.	Oryza_sativa	OG0000036
Os08t0562500-01 Transferase family protein.	Oryza_sativa	OG0000036
Os09t0422000-01 Transferase family protein.	Oryza_sativa	OG0000036
Os09t0543900-01 Transferase family protein.	Oryza_sativa	OG0000036
Os09t0544000-01 Transferase family protein.	Oryza_sativa	OG0000036
Os10t0105850-00 Similar to Transferase family protein.	Oryza_sativa	OG0000036
Os10t0105900-00 Hypothetical conserved gene.	Oryza_sativa	OG0000036
Os10t0105933-00 Similar to transferase family protein.	Oryza_sativa	OG0000036
Os10t0106600-00 Similar to Transferase family protein.	Oryza_sativa	OG0000036
Os10t0108700-01 Transferase family protein.	Oryza_sativa	OG0000036
Os10t0108800-00 Hypothetical conserved gene.	Oryza_sativa	OG0000036

Os10t0122300-00 Transferase domain containing protein.
Oryza_sativa OG0000036
Os10t0122500-00 Hypothetical conserved gene. Oryza_sativa
OG0000036
Os10t0133500-01 Transferase family protein. Oryza_sativa
OG0000036
Os10t0195600-01 Transferase family protein. Oryza_sativa
OG0000036
Os10t0379100-01 Transferase family protein. Oryza_sativa
OG0000036
Os10t0380100-01 Transferase family protein. Oryza_sativa
OG0000036
Os10t0402400-00 Transferase family protein. Oryza_sativa
OG0000036
Os11t0182200-01 Transferase family protein. Oryza_sativa
OG0000036
Os11t0244100-00 Transferase family protein. Oryza_sativa
OG0000036
Os11t0268300-00 Similar to transferase family protein.
Oryza_sativa OG0000036
Os11t0507200-00 Similar to transferase. Oryza_sativa
OG0000036
Os11t0642400-01 Transferase family protein. Oryza_sativa
OG0000036
Os11t0643100-01 Transferase family protein. Oryza_sativa
OG0000036
Os11t0644500-01 Similar to Transferase family protein, expressed.
Oryza_sativa OG0000036
Os12t0134700-01 Transferase family protein. Oryza_sativa
OG0000036
Os12t0458100-01 Transferase family protein. Oryza_sativa
OG0000036
Os12t0459100-00 Similar to Transferase family protein.
Oryza_sativa OG0000036
Os01t0854000-01 Similar to AER. Oryza_sativa OG0000036
Os01t0104900-01 Transferase family protein. Oryza_sativa
OG0000036
Os01t0924933-01 Transferase family protein. Oryza_sativa
OG0000036
Os01t0179000-01 Transferase family protein. Oryza_sativa
OG0000036
Os02t0483500-01 Transferase family protein. Oryza_sativa
OG0000036
Os02t0483800-00 Transferase domain containing protein.
Oryza_sativa OG0000036
Os02t0484200-01 Transferase family protein. Oryza_sativa
OG0000036
Os02t0485000-01 Transferase domain containing protein.
Oryza_sativa OG0000036
Os02t0485800-00 Transferase domain containing protein.
Oryza_sativa OG0000036
Os02t0486500-00 Hypothetical conserved gene. Oryza_sativa
OG0000036
Os01t0185300-01 Transferase family protein. Oryza_sativa

OG0000036
Os02t0611800-01 Similar to Hydroxyanthranilate hydroxycinnamoyltransferase 3.	Oryza_sativa	OG0000036
Os03t0185700-01 Transferase family protein.	Oryza_sativa	OG0000036
g1139.t1	Caulerpa_lentillifera	OG0000037
g2845.t1	Caulerpa_lentillifera	OG0000037
g33.t1	Caulerpa_lentillifera	OG0000037
g3357.t1	Caulerpa_lentillifera	OG0000037
g3438.t1	Caulerpa_lentillifera	OG0000037
g4582.t1	Caulerpa_lentillifera	OG0000037
g5201.t1	Caulerpa_lentillifera	OG0000037
g8628.t1	Caulerpa_lentillifera	OG0000037
Cre02.g087600.t1.1 pacid=30785983 transcript=Cre02.g087600.t1.1 locus=Cre02.g087600 ID=Cre02.g087600.t1.1.v5.5 annot-version=v5.5	Chlamydomonas_reinhardtii	OG0000037
Cre03.g179300.t1.1 pacid=30786668 transcript=Cre03.g179300.t1.1 locus=Cre03.g179300 ID=Cre03.g179300.t1.1.v5.5 annot-version=v5.5	Chlamydomonas_reinhardtii	OG0000037
Cre03.g158550.t1.2 pacid=30787160 transcript=Cre03.g158550.t1.2 locus=Cre03.g158550 ID=Cre03.g158550.t1.2.v5.5 annot-version=v5.5	Chlamydomonas_reinhardtii	OG0000037
Cre03.g162701.t1.1 pacid=30788203 transcript=Cre03.g162701.t1.1 locus=Cre03.g162701 ID=Cre03.g162701.t1.1.v5.5 annot-version=v5.5	Chlamydomonas_reinhardtii	OG0000037
Cre08.g377200.t1.1 pacid=30773522 transcript=Cre08.g377200.t1.1 locus=Cre08.g377200 ID=Cre08.g377200.t1.1.v5.5 annot-version=v5.5	Chlamydomonas_reinhardtii	OG0000037
Cre12.g537671.t1.1 pacid=30792915 transcript=Cre12.g537671.t1.1 locus=Cre12.g537671 ID=Cre12.g537671.t1.1.v5.5 annot-version=v5.5	Chlamydomonas_reinhardtii	OG0000037
Cre12.g508150.t1.2 pacid=30793288 transcript=Cre12.g508150.t1.2 locus=Cre12.g508150 ID=Cre12.g508150.t1.2.v5.5 annot-version=v5.5	Chlamydomonas_reinhardtii	OG0000037
Cre14.g618860.t1.1 pacid=30776654 transcript=Cre14.g618860.t1.1 locus=Cre14.g618860 ID=Cre14.g618860.t1.1.v5.5 annot-version=v5.5	Chlamydomonas_reinhardtii	OG0000037
Cre16.g647602.t1.1 pacid=30777036 transcript=Cre16.g647602.t1.1 locus=Cre16.g647602 ID=Cre16.g647602.t1.1.v5.5 annot-version=v5.5	Chlamydomonas_reinhardtii	OG0000037
Cre09.g390000.t1.2 pacid=30781417 transcript=Cre09.g390000.t1.2 locus=Cre09.g390000 ID=Cre09.g390000.t1.2.v5.5 annot-version=v5.5	Chlamydomonas_reinhardtii	OG0000037
Cre07.g325700.t1.2 pacid=30774388 transcript=Cre07.g325700.t1.2 locus=Cre07.g325700 ID=Cre07.g325700.t1.2.v5.5 annot-version=v5.5	Chlamydomonas_reinhardtii	OG0000037
Vocar.0021s0108.1.p pacid=32895927 transcript=Vocar.0021s0108.1 locus=Vocar.0021s0108 ID=Vocar.0021s0108.1.v2.1 annot-version=v2.1	Volvox_carteri	OG0000037
Vocar.0021s0130.1.p pacid=32895939 transcript=Vocar.0021s0130.1 locus=Vocar.0021s0130 ID=Vocar.0021s0130.1.v2.1 annot-version=v2.1	Volvox_carteri	OG0000037
Vocar.0016s0256.1.p pacid=32897896 transcript=Vocar.0016s0256.1 locus=Vocar.0016s0256 ID=Vocar.0016s0256.1.v2.1 annot-version=v2.1

Volvox_carteri	OG0000037
Vocar.0015s0331.1.p pacid=32898932 transcript=Vocar.0015s0331.1 locus=Vocar.0015s0331 ID=Vocar.0015s0331.1.v2.1 annot-version=v2.1	Volvox_carteri	OG0000037
Vocar.0063s0045.1.p pacid=32883497 transcript=Vocar.0063s0045.1 locus=Vocar.0063s0045 ID=Vocar.0063s0045.1.v2.1 annot-version=v2.1	Volvox_carteri	OG0000037
Vocar.0053s0011.1.p pacid=32887634 transcript=Vocar.0053s0011.1 locus=Vocar.0053s0011 ID=Vocar.0053s0011.1.v2.1 annot-version=v2.1	Volvox_carteri	OG0000037
Vocar.0004s0442.1.p pacid=32889776 transcript=Vocar.0004s0442.1 locus=Vocar.0004s0442 ID=Vocar.0004s0442.1.v2.1 annot-version=v2.1	Volvox_carteri	OG0000037
Vocar.0019s0100.1.p pacid=32892722 transcript=Vocar.0019s0100.1 locus=Vocar.0019s0100 ID=Vocar.0019s0100.1.v2.1 annot-version=v2.1	Volvox_carteri	OG0000037
Vocar.0034s0009.1.p pacid=32893121 transcript=Vocar.0034s0009.1 locus=Vocar.0034s0009 ID=Vocar.0034s0009.1.v2.1 annot-version=v2.1	Volvox_carteri	OG0000037
Vocar.0032s0115.1.p pacid=32893725 transcript=Vocar.0032s0115.1 locus=Vocar.0032s0115 ID=Vocar.0032s0115.1.v2.1 annot-version=v2.1	Volvox_carteri	OG0000037
jgi|ChlNC64A_1|56509|estExt_fgenesh3_pg.C_10175	Chlorella_variabilis	OG0000037
jgi|ChlNC64A_1|9310|gw1.2.166.1	Chlorella_variabilis	OG0000037
jgi|ChlNC64A_1|138032|IGS.gm_2_00499	Chlorella_variabilis	OG0000037
jgi|ChlNC64A_1|34395|estExt_Genewise1Plus.C_40267	Chlorella_variabilis	OG0000037
jgi|ChlNC64A_1|17545|gw1.4.623.1	Chlorella_variabilis	OG0000037
jgi|ChlNC64A_1|13557|gw1.20.135.1	Chlorella_variabilis	OG0000037
jgi|ChlNC64A_1|17845|gw1.6.407.1	Chlorella_variabilis	OG0000037
jgi|ChlNC64A_1|22847|e_gw1.9.26.1	Chlorella_variabilis	OG0000037
jgi|ChlNC64A_1|144734|IGS.gm_8_00062	Chlorella_variabilis	OG0000037
jgi|ChlNC64A_1|9749|gw1.13.65.1	Chlorella_variabilis	OG0000037
47361 pacid=27386801 transcript=47361 locus=estExt_fgenesh1_pg.C_70189 ID=47361.2.0.227 annot-version=v2.0	Coccomyxa_subellipsoidea	OG0000037
14080 pacid=27387075 transcript=14080 locus=e_gw1.5.18.1 ID=14080.2.0.227 annot-version=v2.0	Coccomyxa_subellipsoidea	OG0000037
221 pacid=27387592 transcript=221 locus=gw1.22.4.1 ID=221.2.0.227 annot-version=v2.0	Coccomyxa_subellipsoidea	OG0000037
45866 pacid=27388903 transcript=45866 locus=estExt_fgenesh1_pg.C_10300 ID=45866.2.0.227 annot-version=v2.0	Coccomyxa_subellipsoidea	OG0000037
13454 pacid=27390091 transcript=13454 locus=e_gw1.4.200.1

ID=13454.2.0.227 annot-version=v2.0	Coccomyxa_subellipsoidea
OG0000037
46585 pacid=27390228 transcript=46585
locus=estExt_fgenesh1_pg.C_40083 ID=46585.2.0.227 annot-version=v2.0
	Coccomyxa_subellipsoidea	OG0000037
46779 pacid=27390293 transcript=46779
locus=estExt_fgenesh1_pg.C_40423 ID=46779.2.0.227 annot-version=v2.0
	Coccomyxa_subellipsoidea	OG0000037
12787 pacid=27390792 transcript=12787 locus=e_gw1.3.13.1
ID=12787.2.0.227 annot-version=v2.0	Coccomyxa_subellipsoidea
OG0000037
46223 pacid=27393593 transcript=46223
locus=estExt_fgenesh1_pg.C_20368 ID=46223.2.0.227 annot-version=v2.0
	Coccomyxa_subellipsoidea	OG0000037
31120 pacid=27394409 transcript=31120
locus=estExt_Genewise1Plus.C_180258 ID=31120.2.0.227 annot-
version=v2.0	Coccomyxa_subellipsoidea	OG0000037
15222_e_gw1.01.00.103.1	Ostreococcus_tauri	OG0000037
17347_e_gw1.05.00.94.1	Ostreococcus_tauri	OG0000037
20661_e_gw1.12.00.140.1	Ostreococcus_tauri	OG0000037
34063_0700010416 Ostreococcus_tauri	OG0000037
34652_0900010118 Ostreococcus_tauri	OG0000037
847_gw1.02.00.68.1	Ostreococcus_tauri	OG0000037
913_gw1.17.00.24.1	Ostreococcus_tauri	OG0000037
1225_gw1.07.00.81.1	Ostreococcus_tauri	OG0000037
9771_fgenesh1_pg.C_Chr_02.0001000066	Ostreococcus_tauri
OG0000037
16250 pacid=27413975 transcript=16250
locus=fgenesh1_pg.C_Chr_7000294 ID=16250.2.0.231 annot-version=v2.0
Ostreococcus_lucimarinus	OG0000037
32497 pacid=27414003 transcript=32497 locus=eugene.0700010094
ID=32497.2.0.231 annot-version=v2.0	Ostreococcus_lucimarinus
OG0000037
12759 pacid=27414016 transcript=12759
locus=fgenesh1_pm.C_Chr_7000086 ID=12759.2.0.231 annot-version=v2.0
Ostreococcus_lucimarinus	OG0000037
119553 pacid=27414475 transcript=119553 locus=eugene1.1.0200012810
ID=119553.2.0.231 annot-version=v2.0	Ostreococcus_lucimarinus
OG0000037
30094 pacid=27414522 transcript=30094 locus=eugene.0200010281
ID=30094.2.0.231 annot-version=v2.0	Ostreococcus_lucimarinus
OG0000037
37662 pacid=27415931 transcript=37662 locus=e_gwEuk.16.22.1
ID=37662.2.0.231 annot-version=v2.0	Ostreococcus_lucimarinus
OG0000037
38436 pacid=27416660 transcript=38436 locus=e_gwEuk.5.67.1
ID=38436.2.0.231 annot-version=v2.0	Ostreococcus_lucimarinus
OG0000037
39168 pacid=27416920 transcript=39168 locus=e_gwEuk.12.12.1
ID=39168.2.0.231 annot-version=v2.0	Ostreococcus_lucimarinus
OG0000037
40372 pacid=27417483 transcript=40372 locus=e_gwEuk.1.102.1
ID=40372.2.0.231 annot-version=v2.0	Ostreococcus_lucimarinus
OG0000037

15881 pacid=27418189 transcript=15881
locus=fgenesh1_pg.C_Chr_6000268 ID=15881.2.0.231 annot-version=v2.0
Ostreococcus_lucimarinus OG0000037
199294 pacid=27348852 transcript=199334 locus=fgenesh1_pg.12_#_26
ID=199334.3.0.228 annot-version=v3.0 Micromonas_pusilla
OG0000037
52710 pacid=27339809 transcript=52710 locus=MicpuC2.EuGene.
0000130267 ID=52710.3.0.228 annot-version=v3.0
Micromonas_pusilla OG0000037
59573 pacid=27340757 transcript=59573 locus=MicpuC2.EuGene.
0000070422 ID=59573.3.0.228 annot-version=v3.0
Micromonas_pusilla OG0000037
58412 pacid=27342004 transcript=58412 locus=MicpuC2.EuGene.
0000050717 ID=58412.3.0.228 annot-version=v3.0
Micromonas_pusilla OG0000037
54122 pacid=27343629 transcript=54122 locus=MicpuC2.EuGene.
0000160398 ID=54122.3.0.228 annot-version=v3.0
Micromonas_pusilla OG0000037
60694 pacid=27344230 transcript=60694 locus=MicpuC2.EuGene.
0000090245 ID=60694.3.0.228 annot-version=v3.0
Micromonas_pusilla OG0000037
180083 pacid=27344435 transcript=180123
locus=estExt_Genewise1Plus.C_9_t10402 ID=180123.3.0.228 annot-
version=v3.0 Micromonas_pusilla OG0000037
196529 pacid=27345286 transcript=196569 locus=fgenesh1_pg.1_#_416
ID=196569.3.0.228 annot-version=v3.0 Micromonas_pusilla
OG0000037
21039 pacid=27339467 transcript=21039 locus=MicpuC2.e_gw1.11.43.1
ID=21039.3.0.228 annot-version=v3.0 Micromonas_pusilla
OG0000037
51787 pacid=27339580 transcript=51787 locus=MicpuC2.EuGene.
0000110309 ID=51787.3.0.228 annot-version=v3.0
Micromonas_pusilla OG0000037
56143 pacid=27396300 transcript=56143 locus=EuGene.0200010520
ID=56143.3.0.229 annot-version=v3.0 Micromonas_sp._RCC299
OG0000037
56506 pacid=27396348 transcript=56506 locus=EuGene.0200010883
ID=56506.3.0.229 annot-version=v3.0 Micromonas_sp._RCC299
OG0000037
64845 pacid=27397033 transcript=64845 locus=EuGene.1600010206
ID=64845.3.0.229 annot-version=v3.0 Micromonas_sp._RCC299
OG0000037
99853 pacid=27400069 transcript=99853
locus=fgenesh2_pg.C_Chr_04000048 ID=99853.3.0.229 annot-version=v3.0
Micromonas_sp._RCC299 OG0000037
104935 pacid=27401143 transcript=104935
locus=estExt_fgenesh2_kg.C_Chr_090043 ID=104935.3.0.229 annot-
version=v3.0 Micromonas_sp._RCC299 OG0000037
104934 pacid=27401170 transcript=104934
locus=estExt_fgenesh2_kg.C_Chr_090042 ID=104934.3.0.229 annot-
version=v3.0 Micromonas_sp._RCC299 OG0000037
58978 pacid=27395895 transcript=58978 locus=EuGene.0500010711
ID=58978.3.0.229 annot-version=v3.0 Micromonas_sp._RCC299
OG0000037

59206 pacid=27402067 transcript=59206 locus=EuGene.0600010208
ID=59206.3.0.229 annot-version=v3.0 Micromonas_sp._RCC299
OG0000037
59225 pacid=27402373 transcript=59225 locus=EuGene.0600010227
ID=59225.3.0.229 annot-version=v3.0 Micromonas_sp._RCC299
OG0000037
91017 pacid=27402458 transcript=91017
locus=estExt_Genewise2.C_Chr_060298 ID=91017.3.0.229 annot-
version=v3.0 Micromonas_sp._RCC299 OG0000037
60550 pacid=27403086 transcript=60550 locus=EuGene.0800010118
ID=60550.3.0.229 annot-version=v3.0 Micromonas_sp._RCC299
OG0000037
60953 pacid=27403160 transcript=60953 locus=EuGene.0800010521
ID=60953.3.0.229 annot-version=v3.0 Micromonas_sp._RCC299
OG0000037
60518 pacid=27403554 transcript=60518 locus=EuGene.0800010086
ID=60518.3.0.229 annot-version=v3.0 Micromonas_sp._RCC299
OG0000037
kfl00590_0040_v1.1 Klebsormidium_flaccidum OG0000037
kfl00030_0270_v1.1 Klebsormidium_flaccidum OG0000037
kfl00045_0020_v1.1 Klebsormidium_flaccidum OG0000037
kfl00055_0090_v1.1 Klebsormidium_flaccidum OG0000037
kfl00004_0580_v1.1 Klebsormidium_flaccidum OG0000037
kfl00005_0350_v1.1 Klebsormidium_flaccidum OG0000037
kfl00125_0020_v1.1 Klebsormidium_flaccidum OG0000037
kfl00130_0050_v1.1 Klebsormidium_flaccidum OG0000037
kfl00142_0080_v1.1 Klebsormidium_flaccidum OG0000037
kfl00148_0270_v1.1 Klebsormidium_flaccidum OG0000037
kfl00010_0280_v1.1 Klebsormidium_flaccidum OG0000037
kfl00210_0140_v1.1 Klebsormidium_flaccidum OG0000037
kfl00223_0110_v1.1 Klebsormidium_flaccidum OG0000037
kfl00291_0150_v1.1 Klebsormidium_flaccidum OG0000037
Pp3c15_6140V3.1.p pacid=32929381 transcript=Pp3c15_6140V3.1
locus=Pp3c15_6140 annot-version=v3.3 Physcomitrella_patens
OG0000037
Pp3c13_19680V3.1.p pacid=32930998 transcript=Pp3c13_19680V3.1
locus=Pp3c13_19680 annot-version=v3.3 Physcomitrella_patens
OG0000037
Pp3c13_14440V3.1.p pacid=32931260 transcript=Pp3c13_14440V3.1
locus=Pp3c13_14440 annot-version=v3.3 Physcomitrella_patens
OG0000037
Pp3c2_32500V3.1.p pacid=32933281 transcript=Pp3c2_32500V3.1
locus=Pp3c2_32500 annot-version=v3.3 Physcomitrella_patens
OG0000037
Pp3c2_32501V3.1.p pacid=32935906 transcript=Pp3c2_32501V3.1
locus=Pp3c2_32501 annot-version=v3.3 Physcomitrella_patens
OG0000037
Pp3c3_23680V3.1.p pacid=32940743 transcript=Pp3c3_23680V3.1
locus=Pp3c3_23680 annot-version=v3.3 Physcomitrella_patens
OG0000037
Pp3c3_30220V3.1.p pacid=32942405 transcript=Pp3c3_30220V3.1
locus=Pp3c3_30220 annot-version=v3.3 Physcomitrella_patens
OG0000037
Pp3c3_15820V3.1.p pacid=32942992 transcript=Pp3c3_15820V3.1

locus=Pp3c3_15820 annot-version=v3.3 Physcomitrella_patens
OG0000037
Pp3c20_11500V3.1.p pacid=32946341 transcript=Pp3c20_11500V3.1
locus=Pp3c20_11500 annot-version=v3.3 Physcomitrella_patens
OG0000037
Pp3c27_3890V3.1.p pacid=32951541 transcript=Pp3c27_3890V3.1
locus=Pp3c27_3890 annot-version=v3.3 Physcomitrella_patens
OG0000037
Pp3c5_19940V3.1.p pacid=32955172 transcript=Pp3c5_19940V3.1
locus=Pp3c5_19940 annot-version=v3.3 Physcomitrella_patens
OG0000037
Pp3c5_280V3.1.p pacid=32955481 transcript=Pp3c5_280V3.1
locus=Pp3c5_280 annot-version=v3.3 Physcomitrella_patens
OG0000037
Pp3c11_18940V3.1.p pacid=32958629 transcript=Pp3c11_18940V3.1
locus=Pp3c11_18940 annot-version=v3.3 Physcomitrella_patens
OG0000037
Pp3c14_2650V3.1.p pacid=32960137 transcript=Pp3c14_2650V3.1
locus=Pp3c14_2650 annot-version=v3.3 Physcomitrella_patens
OG0000037
Pp3c14_90V3.1.p pacid=32961357 transcript=Pp3c14_90V3.1
locus=Pp3c14_90 annot-version=v3.3 Physcomitrella_patens
OG0000037
Pp3c1_3340V3.1.p pacid=32969388 transcript=Pp3c1_3340V3.1
locus=Pp3c1_3340 annot-version=v3.3 Physcomitrella_patens
OG0000037
Pp3c6_27590V3.1.p pacid=32978397 transcript=Pp3c6_27590V3.1
locus=Pp3c6_27590 annot-version=v3.3 Physcomitrella_patens
OG0000037
Pp3c6_10390V3.1.p pacid=32978459 transcript=Pp3c6_10390V3.1
locus=Pp3c6_10390 annot-version=v3.3 Physcomitrella_patens
OG0000037
Pp3c17_19520V3.1.p pacid=32907743 transcript=Pp3c17_19520V3.1
locus=Pp3c17_19520 annot-version=v3.3 Physcomitrella_patens
OG0000037
Pp3c18_19045V3.1.p pacid=32982306 transcript=Pp3c18_19045V3.1
locus=Pp3c18_19045 annot-version=v3.3 Physcomitrella_patens
OG0000037
Pp3c18_19040V3.1.p pacid=32983268 transcript=Pp3c18_19040V3.1
locus=Pp3c18_19040 annot-version=v3.3 Physcomitrella_patens
OG0000037
Pp3c10_6710V3.1.p pacid=32900285 transcript=Pp3c10_6710V3.1
locus=Pp3c10_6710 annot-version=v3.3 Physcomitrella_patens
OG0000037
Pp3c21_10800V3.1.p pacid=32915813 transcript=Pp3c21_10800V3.1
locus=Pp3c21_10800 annot-version=v3.3 Physcomitrella_patens
OG0000037
Pp3c26_5950V3.1.p pacid=32917290 transcript=Pp3c26_5950V3.1
locus=Pp3c26_5950 annot-version=v3.3 Physcomitrella_patens
OG0000037
Pp3c10_2280V3.1.p pacid=32901642 transcript=Pp3c10_2280V3.1
locus=Pp3c10_2280 annot-version=v3.3 Physcomitrella_patens
OG0000037
Pp3c10_13800V3.1.p pacid=32901651 transcript=Pp3c10_13800V3.1

locus=Pp3c10_13800 annot-version=v3.3	Physcomitrella_patens	OG0000037
Pp3c7_3340V3.1.p pacid=32923123 transcript=Pp3c7_3340V3.1 locus=Pp3c7_3340 annot-version=v3.3	Physcomitrella_patens	OG0000037
AT2G44980.1 | SNF2 domain-containing protein / helicase domain-containing protein | Chr2:18552440-18556669 REVERSE LENGTH=851 | 201606	Arabidopsis_thaliana	OG0000037
AT2G46020.1 | transcription regulatory protein SNF2 | Chr2:18923304-18931769 FORWARD LENGTH=2192 | 201606	Arabidopsis_thaliana	OG0000037
AT3G06010.1 | SNF2/Brahma-type chromatin-remodeling protein CHR12 | Chr3:1802435-1807284 REVERSE LENGTH=1102 | 201606	Arabidopsis_thaliana	OG0000037
AT3G06400.1 | chromatin-remodeling protein 11 | Chr3:1941066-1946700 FORWARD LENGTH=1055 | 201606	Arabidopsis_thaliana	OG0000037
AT3G12810.1 | SNF2 domain-containing protein / helicase domain-containing protein | Chr3:4065636-4073992 FORWARD LENGTH=2055 | 201606	Arabidopsis_thaliana	OG0000037
AT3G57300.1 | DNA helicase INO80-like protein | Chr3:21199612-21207635 FORWARD LENGTH=1507 | 201606	Arabidopsis_thaliana	OG0000037
AT4G31900.1 | chromatin remodeling factor | Chr4:15431528-15438443 FORWARD LENGTH=1202 | 201606	Arabidopsis_thaliana	OG0000037
AT5G18620.1 | chromatin remodeling factor17 | Chr5:6196190-6202058 REVERSE LENGTH=1069 | 201606	Arabidopsis_thaliana	OG0000037
AT5G19310.1 | homeotic protein regulator | Chr5:6498906-6503432 FORWARD LENGTH=1064 | 201606	Arabidopsis_thaliana	OG0000037
AT5G44800.1 | chromatin remodeling 4 | Chr5:18083659-18092162 REVERSE LENGTH=2223 | 201606	Arabidopsis_thaliana	OG0000037
AT5G66750.1 | chromatin remodeling 1 | Chr5:26649050-26652869 FORWARD LENGTH=764 | 201606	Arabidopsis_thaliana	OG0000037
AT2G02090.1 | SNF2 domain-containing protein / helicase domain-containing protein | Chr2:523481-526884 FORWARD LENGTH=763 | 201606	Arabidopsis_thaliana	OG0000037
AT1G08060.1 | ATP-dependent helicase family protein | Chr1:2501981-2510488 REVERSE LENGTH=2001 | 201606	Arabidopsis_thaliana	OG0000037
AT2G13370.1 | chromatin remodeling 5 | Chr2:5544601-5555543 REVERSE LENGTH=1724 | 201606	Arabidopsis_thaliana	OG0000037
AT2G25170.1 | chromatin remodeling factor CHD3 (PICKLE) | Chr2:10714411-10723763 FORWARD LENGTH=1384 | 201606	Arabidopsis_thaliana	OG0000037
AT2G28290.1 | P-loop containing nucleoside triphosphate hydrolases superfamily protein | Chr2:12056771-12072950 FORWARD LENGTH=3574 | 201606	Arabidopsis_thaliana	OG0000037
Os03t0352450-00 Hypothetical conserved gene.	Oryza_sativa	OG0000037

Os04t0566100-00 Similar to OSIGBa0158F05.11 protein.
Oryza_sativa OG0000037
Os05t0144300-01 DEAD-like helicase, N-terminal domain containing protein. Oryza_sativa OG0000037
Os05t0150300-01 Similar to Possible global transcription activator SNF2L1 (SWI/SNF related matrix associated actin dependent regulator of chromatin subfamily A member 1). Oryza_sativa OG0000037
Os01t0367900-01 Similar to Possible global transcription activator SNF2L1 (SWI/SNF related matrix associated actin dependent regulator of chromatin subfamily A member 1). Oryza_sativa OG0000037
Os06t0102800-01 SNF2-related domain containing protein.
Oryza_sativa OG0000037
Os07t0497100-01 Chromodomain, helicase/ATPase, and DNA-binding domain (CHD) protein, Chromatin-remodeling factor, Mi-2-like protein, Crown root development, Chloroplast development in adaxial mesophyll, Maintenance of H3K4me3 Oryza_sativa OG0000037
Os07t0660200-01 Similar to chromatin remodeling complex subunit.
Oryza_sativa OG0000037
Os09t0442700-00 Similar to chromatin complex subunit A101.
Oryza_sativa OG0000037
Os01t0881000-00 SNF2-related domain containing protein.
Oryza_sativa OG0000037
Os02t0114033-00 Glutamine-Leucine-Glutamine, QLQ domain containing protein. Oryza_sativa OG0000037
Os02t0689800-01 Similar to DEAD/DEAH box helicase domain-containing protein PIE1. Oryza_sativa OG0000037
CMM316CT pep chromosome:ASM9120v1:13:798359:802733:-1 gene:CMM316C transcript:CMM316CT gene_biotype:protein_coding transcript_biotype:protein_coding Cyanidioschyzon_merolae OG0000037
CMH195CT pep chromosome:ASM9120v1:8:511866:515865:-1 gene:CMH195C transcript:CMH195CT gene_biotype:protein_coding transcript_biotype:protein_coding Cyanidioschyzon_merolae OG0000037
CMT353CT pep chromosome:ASM9120v1:20:884500:888914:1 gene:CMT353C transcript:CMT353CT gene_biotype:protein_coding transcript_biotype:protein_coding Cyanidioschyzon_merolae OG0000037
CMJ200CT pep chromosome:ASM9120v1:10:539475:542024:1 gene:CMJ200C transcript:CMJ200CT gene_biotype:protein_coding transcript_biotype:protein_coding description:chromatin remodeling 1 [Source:Projected from Arabidopsis thaliana (AT5G66750) TAIR;Acc:AT5G66750] Cyanidioschyzon_merolae OG0000037
CMQ363CT pep chromosome:ASM9120v1:17:938723:942046:1 gene:CMQ363C transcript:CMQ363CT gene_biotype:protein_coding transcript_biotype:protein_coding Cyanidioschyzon_merolae OG0000037
CMQ215CT pep chromosome:ASM9120v1:17:542811:548855:1 gene:CMQ215C transcript:CMQ215CT gene_biotype:protein_coding transcript_biotype:protein_coding Cyanidioschyzon_merolae OG0000037
evm.model.contig_2132.9 Porphyridium_purpureum OG0000037

evm.model.contig_2672.1 Porphyridium_purpureum OG0000037
evm.model.contig_2035b.13 Porphyridium_purpureum OG0000037
evm.model.contig_4438.19 Porphyridium_purpureum OG0000037
evm.model.contig_4474.1 Porphyridium_purpureum OG0000037
evm.model.contig_4527.1 Porphyridium_purpureum OG0000037
evm.model.contig_483.3 Porphyridium_purpureum OG0000037
evm.model.contig_664.3 Porphyridium_purpureum OG0000037
evm.model.contig_2077.13 Porphyridium_purpureum OG0000037
CDF39883 pep supercontig:ASM35022v2:HG002086:246987:252078:-1 gene:CHC_T00006862001 transcript:CDF39883 gene_biotype:protein_coding transcript_biotype:protein_coding Chondrus_crispus OG0000037
CDF36036 pep supercontig:ASM35022v2:HG001756:107358:110459:-1 gene:CHC_T00010228001 transcript:CDF36036 gene_biotype:protein_coding transcript_biotype:protein_coding description:chromatin-remodelling complex ATPase ISWI2 Chondrus_crispus OG0000037
CDF39174 pep supercontig:ASM35022v2:HG002009:24563:28622:-1 gene:CHC_T00000150001 transcript:CDF39174 gene_biotype:protein_coding transcript_biotype:protein_coding Chondrus_crispus OG0000037
CDF35476 pep supercontig:ASM35022v2:HG001731:182588:186511:1 gene:CHC_T00003981001 transcript:CDF35476 gene_biotype:protein_coding transcript_biotype:protein_coding Chondrus_crispus OG0000037
CDF41309 pep supercontig:ASM35022v2:HG002355:60419:65810:1 gene:CHC_T00007825001 transcript:CDF41309 gene_biotype:protein_coding transcript_biotype:protein_coding Chondrus_crispus OG0000037
CDF32159 pep supercontig:ASM35022v2:HG001459:440305:444966:1 gene:CHC_T00001386001 transcript:CDF32159 gene_biotype:protein_coding transcript_biotype:protein_coding Chondrus_crispus OG0000037
CDF39284 pep supercontig:ASM35022v2:HG002017:39806:40901:1 gene:CHC_T00000229001 transcript:CDF39284 gene_biotype:protein_coding transcript_biotype:protein_coding Chondrus_crispus OG0000037
CDF33786 pep supercontig:ASM35022v2:HG001658:11985:18866:-1 gene:CHC_T00002398001 transcript:CDF33786 gene_biotype:protein_coding transcript_biotype:protein_coding Chondrus_crispus OG0000037
CDF39449 pep supercontig:ASM35022v2:HG002033:11101:12274:1 gene:CHC_T00000273001 transcript:CDF39449 gene_biotype:protein_coding transcript_biotype:protein_coding Chondrus_crispus OG0000037
g104.t1 Caulerpa_lentillifera OG0000038
g1096.t1 Caulerpa_lentillifera OG0000038
g1191.t1 Caulerpa_lentillifera OG0000038
g2064.t1 Caulerpa_lentillifera OG0000038
g2131.t1 Caulerpa_lentillifera OG0000038
g2191.t1 Caulerpa_lentillifera OG0000038
g2367.t1 Caulerpa_lentillifera OG0000038
g240.t1 Caulerpa_lentillifera OG0000038
g241.t1 Caulerpa_lentillifera OG0000038

g3242.t1 Caulerpa_lentillifera OG0000038
g3275.t1 Caulerpa_lentillifera OG0000038
g3593.t1 Caulerpa_lentillifera OG0000038
g3642.t1 Caulerpa_lentillifera OG0000038
g3923.t1 Caulerpa_lentillifera OG0000038
g3925.t1 Caulerpa_lentillifera OG0000038
g3992.t1 Caulerpa_lentillifera OG0000038
g4203.t1 Caulerpa_lentillifera OG0000038
g4237.t1 Caulerpa_lentillifera OG0000038
g4274.t1 Caulerpa_lentillifera OG0000038
g4470.t1 Caulerpa_lentillifera OG0000038
g4725.t1 Caulerpa_lentillifera OG0000038
g4824.t1 Caulerpa_lentillifera OG0000038
g4895.t1 Caulerpa_lentillifera OG0000038
g5236.t1 Caulerpa_lentillifera OG0000038
g604.t1 Caulerpa_lentillifera OG0000038
g6125.t1 Caulerpa_lentillifera OG0000038
g647.t1 Caulerpa_lentillifera OG0000038
g6516.t1 Caulerpa_lentillifera OG0000038
g6666.t1 Caulerpa_lentillifera OG0000038
g7057.t1 Caulerpa_lentillifera OG0000038
g7123.t1 Caulerpa_lentillifera OG0000038
g7236.t1 Caulerpa_lentillifera OG0000038
g7524.t1 Caulerpa_lentillifera OG0000038
g7684.t1 Caulerpa_lentillifera OG0000038
g8888.t1 Caulerpa_lentillifera OG0000038
Cre03.g188100.t1.2 pacid=30787982 transcript=Cre03.g188100.t1.2 locus=Cre03.g188100 ID=Cre03.g188100.t1.2.v5.5 annot-version=v5.5 Chlamydomonas_reinhardtii OG0000038
Cre01.g047650.t1.2 pacid=30789191 transcript=Cre01.g047650.t1.2 locus=Cre01.g047650 ID=Cre01.g047650.t1.2.v5.5 annot-version=v5.5 Chlamydomonas_reinhardtii OG0000038
Cre12.g546900.t1.1 pacid=30792068 transcript=Cre12.g546900.t1.1 locus=Cre12.g546900 ID=Cre12.g546900.t1.1.v5.5 annot-version=v5.5 Chlamydomonas_reinhardtii OG0000038
Cre12.g559050.t1.2 pacid=30792451 transcript=Cre12.g559050.t1.2 locus=Cre12.g559050 ID=Cre12.g559050.t1.2.v5.5 annot-version=v5.5 Chlamydomonas_reinhardtii OG0000038
Cre12.g535400.t1.1 pacid=30792512 transcript=Cre12.g535400.t1.1 locus=Cre12.g535400 ID=Cre12.g535400.t1.1.v5.5 annot-version=v5.5 Chlamydomonas_reinhardtii OG0000038
Cre08.g358553.t1.1 pacid=30773571 transcript=Cre08.g358553.t1.1 locus=Cre08.g358553 ID=Cre08.g358553.t1.1.v5.5 annot-version=v5.5 Chlamydomonas_reinhardtii OG0000038
Cre11.g467559.t1.1 pacid=30775824 transcript=Cre11.g467559.t1.1 locus=Cre11.g467559 ID=Cre11.g467559.t1.1.v5.5 annot-version=v5.5 Chlamydomonas_reinhardtii OG0000038
Cre16.g688863.t1.1 pacid=30777867 transcript=Cre16.g688863.t1.1 locus=Cre16.g688863 ID=Cre16.g688863.t1.1.v5.5 annot-version=v5.5 Chlamydomonas_reinhardtii OG0000038
Cre06.g303000.t1.1 pacid=30778749 transcript=Cre06.g303000.t1.1 locus=Cre06.g303000 ID=Cre06.g303000.t1.1.v5.5 annot-version=v5.5 Chlamydomonas_reinhardtii OG0000038
Cre06.g306057.t1.2 pacid=30778796 transcript=Cre06.g306057.t1.2

locus=Cre06.g306057 ID=Cre06.g306057.t1.2.v5.5 annot-version=v5.5
Chlamydomonas_reinhardtii OG0000038
Cre15.g640101.t1.1 pacid=30783678 transcript=Cre15.g640101.t1.1
locus=Cre15.g640101 ID=Cre15.g640101.t1.1.v5.5 annot-version=v5.5
Chlamydomonas_reinhardtii OG0000038
Vocar.0011s0228.1.p pacid=32895422 transcript=Vocar.0011s0228.1
locus=Vocar.0011s0228 ID=Vocar.0011s0228.1.v2.1 annot-version=v2.1
Volvox_carteri OG0000038
Vocar.0071s0027.1.p pacid=32896163 transcript=Vocar.0071s0027.1
locus=Vocar.0071s0027 ID=Vocar.0071s0027.1.v2.1 annot-version=v2.1
Volvox_carteri OG0000038
Vocar.0001s0541.1.p pacid=32884671 transcript=Vocar.0001s0541.1
locus=Vocar.0001s0541 ID=Vocar.0001s0541.1.v2.1 annot-version=v2.1
Volvox_carteri OG0000038
Vocar.0001s1170.1.p pacid=32885512 transcript=Vocar.0001s1170.1
locus=Vocar.0001s1170 ID=Vocar.0001s1170.1.v2.1 annot-version=v2.1
Volvox_carteri OG0000038
Vocar.0043s0051.1.p pacid=32886953 transcript=Vocar.0043s0051.1
locus=Vocar.0043s0051 ID=Vocar.0043s0051.1.v2.1 annot-version=v2.1
Volvox_carteri OG0000038
Vocar.0029s0115.1.p pacid=32887329 transcript=Vocar.0029s0115.1
locus=Vocar.0029s0115 ID=Vocar.0029s0115.1.v2.1 annot-version=v2.1
Volvox_carteri OG0000038
Vocar.0029s0174.1.p pacid=32887337 transcript=Vocar.0029s0174.1
locus=Vocar.0029s0174 ID=Vocar.0029s0174.1.v2.1 annot-version=v2.1
Volvox_carteri OG0000038
Vocar.0007s0191.1.p pacid=32888232 transcript=Vocar.0007s0191.1
locus=Vocar.0007s0191 ID=Vocar.0007s0191.1.v2.1 annot-version=v2.1
Volvox_carteri OG0000038
Vocar.0014s0238.1.p pacid=32888552 transcript=Vocar.0014s0238.1
locus=Vocar.0014s0238 ID=Vocar.0014s0238.1.v2.1 annot-version=v2.1
Volvox_carteri OG0000038
jgi|ChlNC64A_1|133919|IGS.gm_10_00210 Chlorella_variabilis
OG0000038
jgi|ChlNC64A_1|133934|IGS.gm_10_00225 Chlorella_variabilis
OG0000038
jgi|ChlNC64A_1|52541|fgenesh3_pg.C_scaffold_11000117
Chlorella_variabilis OG0000038
jgi|ChlNC64A_1|145384|IGS.gm_9_00191 Chlorella_variabilis
OG0000038
jgi|ChlNC64A_1|57520|estExt_fgenesh3_pg.C_70147
Chlorella_variabilis OG0000038
jgi|ChlNC64A_1|138842|IGS.gm_22_00083 Chlorella_variabilis
OG0000038
jgi|ChlNC64A_1|145011|IGS.gm_8_00339 Chlorella_variabilis
OG0000038
jgi|ChlNC64A_1|141508|IGS.gm_35_00020 Chlorella_variabilis
OG0000038
42660 pacid=27385748 transcript=42660 locus=fgenesh1_pg.10_#_163
ID=42660.2.0.227 annot-version=v2.0 Coccomyxa_subellipsoidea
OG0000038
62146 pacid=27387362 transcript=62146 locus=Genemark1.2929_g
ID=62146.2.0.227 annot-version=v2.0 Coccomyxa_subellipsoidea
OG0000038

60079 pacid=27387636 transcript=60079 locus=Genemark1.8395_g ID=60079.2.0.227 annot-version=v2.0	Coccomyxa_subellipsoidea OG0000038
36782 pacid=27388781 transcript=36782 locus=fgenesh1_pm.9_#_39 ID=36782.2.0.227 annot-version=v2.0	Coccomyxa_subellipsoidea OG0000038
55082 pacid=27389181 transcript=55082 locus=Genemark1.26_g ID=55082.2.0.227 annot-version=v2.0	Coccomyxa_subellipsoidea OG0000038
42632 pacid=27386009 transcript=42632 locus=fgenesh1_pg.10_#_135 ID=42632.2.0.227 annot-version=v2.0	Coccomyxa_subellipsoidea OG0000038
11052 pacid=27389461 transcript=11052 locus=e_gw1.1.175.1 ID=11052.2.0.227 annot-version=v2.0	Coccomyxa_subellipsoidea OG0000038
55654 pacid=27389493 transcript=55654 locus=Genemark1.598_g ID=55654.2.0.227 annot-version=v2.0	Coccomyxa_subellipsoidea OG0000038
61161 pacid=27390389 transcript=61161 locus=Genemark1.1972_g ID=61161.2.0.227 annot-version=v2.0	Coccomyxa_subellipsoidea OG0000038
48161 pacid=27391663 transcript=48161 locus=estExt_fgenesh1_pg.C_120187 ID=48161.2.0.227 annot-version=v2.0	Coccomyxa_subellipsoidea OG0000038
43138 pacid=27391806 transcript=43138 locus=fgenesh1_pg.12_#_77 ID=43138.2.0.227 annot-version=v2.0	Coccomyxa_subellipsoidea OG0000038
56335 pacid=27392221 transcript=56335 locus=Genemark1.5364_g ID=56335.2.0.227 annot-version=v2.0	Coccomyxa_subellipsoidea OG0000038
42048 pacid=27392317 transcript=42048 locus=fgenesh1_pg.8_#_96 ID=42048.2.0.227 annot-version=v2.0	Coccomyxa_subellipsoidea OG0000038
59386 pacid=27393504 transcript=59386 locus=Genemark1.1348_g ID=59386.2.0.227 annot-version=v2.0	Coccomyxa_subellipsoidea OG0000038
45337 pacid=27386525 transcript=45337 locus=fgenesh1_pg.23_#_91 ID=45337.2.0.227 annot-version=v2.0	Coccomyxa_subellipsoidea OG0000038
190150 pacid=27348976 transcript=190190 locus=gm1.7271_g ID=190190.3.0.228 annot-version=v3.0	Micromonas_pusilla OG0000038
68991 pacid=27338658 transcript=68991 locus=MicpuC2.est_orfs.8_7974_4277657:2 ID=68991.3.0.228 annot-version=v3.0	Micromonas_pusilla	OG0000038
187094 pacid=27348187 transcript=187134 locus=gm1.4215_g ID=187134.3.0.228 annot-version=v3.0	Micromonas_pusilla OG0000038
63943 pacid=27403949 transcript=63943 locus=EuGene.1400010078 ID=63943.3.0.229 annot-version=v3.0	Micromonas_sp._RCC299 OG0000038
kfl00434_0100_v1.1	Klebsormidium_flaccidum	OG0000038
kfl00508_0080_v1.1	Klebsormidium_flaccidum	OG0000038
kfl00513_0020_v1.1	Klebsormidium_flaccidum	OG0000038

kfl00513_0040_v1.1	Klebsormidium_flaccidum	OG0000038
kfl00581_0070_v1.1	Klebsormidium_flaccidum	OG0000038
kfl00581_0090_v1.1	Klebsormidium_flaccidum	OG0000038
kfl00595_0010_v1.1	Klebsormidium_flaccidum	OG0000038
kfl00595_0040_v1.1	Klebsormidium_flaccidum	OG0000038
kfl00595_0060_v1.1	Klebsormidium_flaccidum	OG0000038
kfl00020_0410_v1.1	Klebsormidium_flaccidum	OG0000038
kfl00020_0460_v1.1	Klebsormidium_flaccidum	OG0000038
kfl00730_0080_v1.1	Klebsormidium_flaccidum	OG0000038
kfl00834_0040_v1.1	Klebsormidium_flaccidum	OG0000038
kfl00028_0560_v1.1	Klebsormidium_flaccidum	OG0000038
kfl00036_0340_v1.1	Klebsormidium_flaccidum	OG0000038
kfl00088_0180_v1.1	Klebsormidium_flaccidum	OG0000038
kfl00099_0260_v1.1	Klebsormidium_flaccidum	OG0000038
kfl00101_0280_v1.1	Klebsormidium_flaccidum	OG0000038
kfl00102_0050_v1.1	Klebsormidium_flaccidum	OG0000038
kfl00009_0200_v1.1	Klebsormidium_flaccidum	OG0000038
kfl00169_0150_v1.1	Klebsormidium_flaccidum	OG0000038
kfl00175_0070_v1.1	Klebsormidium_flaccidum	OG0000038
kfl00175_0270_v1.1	Klebsormidium_flaccidum	OG0000038
kfl00181_0090_v1.1	Klebsormidium_flaccidum	OG0000038
kfl00010_0620_v1.1	Klebsormidium_flaccidum	OG0000038
kfl00213_0050_v1.1	Klebsormidium_flaccidum	OG0000038
kfl00011_0110_v1.1	Klebsormidium_flaccidum	OG0000038
kfl00011_0490_v1.1	Klebsormidium_flaccidum	OG0000038
kfl00247_0220_v1.1	Klebsormidium_flaccidum	OG0000038
kfl00285_0160_v1.1	Klebsormidium_flaccidum	OG0000038
kfl00285_0190_v1.1	Klebsormidium_flaccidum	OG0000038
kfl00290_0040_v1.1	Klebsormidium_flaccidum	OG0000038
kfl00325_0170_v1.1	Klebsormidium_flaccidum	OG0000038
Pp3c2_31270V3.1.p pacid=32934409 transcript=Pp3c2_31270V3.1 locus=Pp3c2_31270 annot-version=v3.3	Physcomitrella_patens	OG0000038
Pp3c2_31590V3.1.p pacid=32936227 transcript=Pp3c2_31590V3.1 locus=Pp3c2_31590 annot-version=v3.3	Physcomitrella_patens	OG0000038
Pp3c23_2930V3.1.p pacid=32949431 transcript=Pp3c23_2930V3.1 locus=Pp3c23_2930 annot-version=v3.3	Physcomitrella_patens	OG0000038
Pp3c11_6360V3.1.p pacid=32956527 transcript=Pp3c11_6360V3.1 locus=Pp3c11_6360 annot-version=v3.3	Physcomitrella_patens	OG0000038
Pp3c22_12260V3.1.p pacid=32905292 transcript=Pp3c22_12260V3.1 locus=Pp3c22_12260 annot-version=v3.3	Physcomitrella_patens	OG0000038
Pp3c11_7220V3.1.p pacid=32959172 transcript=Pp3c11_7220V3.1 locus=Pp3c11_7220 annot-version=v3.3	Physcomitrella_patens	OG0000038
Pp3c8_1510V3.1.p pacid=32964135 transcript=Pp3c8_1510V3.1 locus=Pp3c8_1510 annot-version=v3.3	Physcomitrella_patens	OG0000038
Pp3c1_8810V3.1.p pacid=32967875 transcript=Pp3c1_8810V3.1 locus=Pp3c1_8810 annot-version=v3.3	Physcomitrella_patens	OG0000038

Pp3c12_1700V3.1.p pacid=32973653 transcript=Pp3c12_1700V3.1
locus=Pp3c12_1700 annot-version=v3.3 Physcomitrella_patens
OG0000038
Pp3c12_11790V3.1.p pacid=32973655 transcript=Pp3c12_11790V3.1
locus=Pp3c12_11790 annot-version=v3.3 Physcomitrella_patens
OG0000038
Pp3c6_26950V3.1.p pacid=32978998 transcript=Pp3c6_26950V3.1
locus=Pp3c6_26950 annot-version=v3.3 Physcomitrella_patens
OG0000038
Pp3c10_8230V3.1.p pacid=32901539 transcript=Pp3c10_8230V3.1
locus=Pp3c10_8230 annot-version=v3.3 Physcomitrella_patens
OG0000038
Pp3c4_3150V3.1.p pacid=32921305 transcript=Pp3c4_3150V3.1
locus=Pp3c4_3150 annot-version=v3.3 Physcomitrella_patens
OG0000038
Pp3c4_4760V3.1.p pacid=32921774 transcript=Pp3c4_4760V3.1
locus=Pp3c4_4760 annot-version=v3.3 Physcomitrella_patens
OG0000038
Pp3c7_26040V3.1.p pacid=32922993 transcript=Pp3c7_26040V3.1
locus=Pp3c7_26040 annot-version=v3.3 Physcomitrella_patens
OG0000038
AT3G07550.1 | RNI-like superfamily protein | Chr3:2409946-2411133
FORWARD LENGTH=395 | 201606 Arabidopsis_thaliana
OG0000038
AT1G15740.1 | Leucine-rich repeat family protein |
Chr1:5411509-5414544 FORWARD LENGTH=585 | 201606
Arabidopsis_thaliana OG0000038
AT3G58530.1 | RNI-like superfamily protein | Chr3:21645759-21648219
FORWARD LENGTH=353 | 201606 Arabidopsis_thaliana
OG0000038
AT4G15475.1 | F-box/RNI-like superfamily protein |
Chr4:8845927-8848701 FORWARD LENGTH=610 | 201606
Arabidopsis_thaliana OG0000038
AT4G23840.1 | Leucine-rich repeat (LRR) family protein |
Chr4:12400379-12403190 FORWARD LENGTH=597 | 201606
Arabidopsis_thaliana OG0000038
AT5G01720.1 | RNI-like superfamily protein | Chr5:267118-270391
REVERSE LENGTH=665 | 201606 Arabidopsis_thaliana
OG0000038
AT1G21410.1 | F-box/RNI-like superfamily protein |
Chr1:7497479-7499386 FORWARD LENGTH=360 | 201606
Arabidopsis_thaliana OG0000038
AT5G21900.1 | RNI-like superfamily protein | Chr5:7238239-7240338
FORWARD LENGTH=544 | 201606 Arabidopsis_thaliana
OG0000038
AT5G23340.1 | RNI-like superfamily protein | Chr5:7856314-7857983
FORWARD LENGTH=405 | 201606 Arabidopsis_thaliana
OG0000038
AT5G25350.1 | EIN3-binding F box protein 2 | Chr5:8794842-8796882
REVERSE LENGTH=623 | 201606 Arabidopsis_thaliana
OG0000038
AT5G27920.1 | F-box family protein | Chr5:9942063-9944507 REVERSE
LENGTH=642 | 201606 Arabidopsis_thaliana OG0000038
AT5G40470.1 | RNI-like superfamily protein | Chr5:16208255-16209745

REVERSE LENGTH=496 | 201606 Arabidopsis_thaliana
OG0000038
AT1G55590.1 | RNI-like superfamily protein | Chr1:20769476-20771756
REVERSE LENGTH=607 | 201606 Arabidopsis_thaliana
OG0000038
AT1G77000.1 | RNI-like superfamily protein | Chr1:28940888-28942401
FORWARD LENGTH=360 | 201606 Arabidopsis_thaliana
OG0000038
AT1G80630.1 | RNI-like superfamily protein | Chr1:30308879-30310615
REVERSE LENGTH=578 | 201606 Arabidopsis_thaliana
OG0000038
AT2G06040.1 | F-box/LRR protein | Chr2:2352333-2355419 REVERSE
LENGTH=762 | 201606 Arabidopsis_thaliana OG0000038
AT2G25490.1 | EIN3-binding F box protein 1 | Chr2:10848018-10850275
REVERSE LENGTH=628 | 201606 Arabidopsis_thaliana
OG0000038
Os04t0503500-01 Leucine-rich repeat, cysteine-containing subtype
containing protein. Oryza_sativa OG0000038
Os05t0212200-01 Hypothetical conserved gene. Oryza_sativa
OG0000038
Os05t0425700-01 Similar to F-box protein AtFBL5. Oryza_sativa
OG0000038
Os06t0304700-01 Conserved hypothetical protein. Oryza_sativa
OG0000038
Os06t0605900-01 Leucine-rich repeat, cysteine-containing subtype
containing protein. Oryza_sativa OG0000038
Os08t0400800-00 Similar to F-box domain containing protein,
expressed. Oryza_sativa OG0000038
Os08t0459100-01 Leucine-rich repeat, cysteine-containing containing
protein. Oryza_sativa OG0000038
Os09t0328066-00 Hypothetical conserved gene. Oryza_sativa
OG0000038
Os10t0148800-01 Similar to F-box domain containing protein,
expressed. Oryza_sativa OG0000038
Os11t0108932-01 Similar to Leucine Rich Repeat family protein,
expressed. Oryza_sativa OG0000038
Os11t0264200-01 Cyclin-like F-box domain containing protein.
Oryza_sativa OG0000038
Os11t0486000-01 Similar to H0714H04.10 protein. Oryza_sativa
OG0000038
Os12t0108500-01 Similar to Leucine Rich Repeat family protein,
expressed. Oryza_sativa OG0000038
Os12t0552700-01 Similar to Leucine Rich Repeat family protein,
expressed. Oryza_sativa OG0000038
Os01t0881900-01 Leucine-rich repeat, cysteine-containing containing
protein. Oryza_sativa OG0000038
Os02t0200900-01 Hypothetical conserved gene. Oryza_sativa
OG0000038
Os02t0281150-01 Similar to Leucine Rich Repeat family protein.
Oryza_sativa OG0000038
Os02t0613200-01 Hypothetical conserved gene. Oryza_sativa
OG0000038
Os02t0636400-01 Leucine-rich repeat, cysteine-containing subtype
containing protein. Oryza_sativa OG0000038

CMM061CT pep chromosome:ASM9120v1:13:130721:131710:1 gene:CMM061C transcript:CMM061CT gene_biotype:protein_coding transcript_biotype:protein_coding Cyanidioschyzon_merolae OG0000038
CMI121CT pep chromosome:ASM9120v1:9:342323:344146:1 gene:CMI121C transcript:CMI121CT gene_biotype:protein_coding transcript_biotype:protein_coding Cyanidioschyzon_merolae OG0000038
CMR435CT pep chromosome:ASM9120v1:18:1072113:1079091:-1 gene:CMR435C transcript:CMR435CT gene_biotype:protein_coding transcript_biotype:protein_coding Cyanidioschyzon_merolae OG0000038
evm.model.contig_2273.4 Porphyridium_purpureum OG0000038
evm.model.contig_2031.12 Porphyridium_purpureum OG0000038
evm.model.contig_3551.1 Porphyridium_purpureum OG0000038
evm.model.contig_3643.2 Porphyridium_purpureum OG0000038
evm.model.contig_4451.4 Porphyridium_purpureum OG0000038
evm.model.contig_4522.2 Porphyridium_purpureum OG0000038
evm.model.contig_506.7 Porphyridium_purpureum OG0000038
evm.model.contig_786.1 Porphyridium_purpureum OG0000038
CDF40898 pep supercontig:ASM35022v2:HG002263:4353:7874:-1 gene:CHC_T00000887001 transcript:CDF40898 gene_biotype:protein_coding transcript_biotype:protein_coding Chondrus_crispus OG0000038
CDF40622 pep supercontig:ASM35022v2:HG002219:223:2216:-1 gene:CHC_T00008706001 transcript:CDF40622 gene_biotype:protein_coding transcript_biotype:protein_coding description:F-box and leucine rich repeat containing protein Chondrus_crispus OG0000038
CDF40631 pep supercontig:ASM35022v2:HG002219:71895:73575:1 gene:CHC_T00008983001 transcript:CDF40631 gene_biotype:protein_coding transcript_biotype:protein_coding description:Leucine rich repeat containing protein Chondrus_crispus OG0000038
CDF40633 pep supercontig:ASM35022v2:HG002219:80848:82263:-1 gene:CHC_T00009124001 transcript:CDF40633 gene_biotype:protein_coding transcript_biotype:protein_coding description:F-box and leucine rich repeat containing protein Chondrus_crispus OG0000038
CDF39340 pep supercontig:ASM35022v2:HG002022:368986:370871:-1 gene:CHC_T00009575001 transcript:CDF39340 gene_biotype:protein_coding transcript_biotype:protein_coding description:F-box and leucine rich repeat containing protein Chondrus_crispus OG0000038
CDF38317 pep supercontig:ASM35022v2:HG001916:60181:61695:1 gene:CHC_T00000926001 transcript:CDF38317 gene_biotype:protein_coding transcript_biotype:protein_coding Chondrus_crispus OG0000038
g2133.t1 Caulerpa_lentillifera OG0000039
g5563.t1 Caulerpa_lentillifera OG0000039
g7004.t1 Caulerpa_lentillifera OG0000039
g7156.t1 Caulerpa_lentillifera OG0000039
g7473.t1 Caulerpa_lentillifera OG0000039
g947.t1 Caulerpa_lentillifera OG0000039

Cre02.g092500.t1.1 pacid=30785207 transcript=Cre02.g092500.t1.1 locus=Cre02.g092500 ID=Cre02.g092500.t1.1.v5.5 annot-version=v5.5 Chlamydomonas_reinhardtii OG0000039
Cre03.g187950.t1.1 pacid=30786536 transcript=Cre03.g187950.t1.1 locus=Cre03.g187950 ID=Cre03.g187950.t1.1.v5.5 annot-version=v5.5 Chlamydomonas_reinhardtii OG0000039
Cre03.g150350.t1.1 pacid=30786728 transcript=Cre03.g150350.t1.1 locus=Cre03.g150350 ID=Cre03.g150350.t1.1.v5.5 annot-version=v5.5 Chlamydomonas_reinhardtii OG0000039
Cre01.g048200.t1.1 pacid=30788746 transcript=Cre01.g048200.t1.1 locus=Cre01.g048200 ID=Cre01.g048200.t1.1.v5.5 annot-version=v5.5 Chlamydomonas_reinhardtii OG0000039
Cre01.g040379.t1.2 pacid=30788967 transcript=Cre01.g040379.t1.2 locus=Cre01.g040379 ID=Cre01.g040379.t1.2.v5.5 annot-version=v5.5 Chlamydomonas_reinhardtii OG0000039
Cre10.g442650.t1.2 pacid=30790906 transcript=Cre10.g442650.t1.2 locus=Cre10.g442650 ID=Cre10.g442650.t1.2.v5.5 annot-version=v5.5 Chlamydomonas_reinhardtii OG0000039
Cre12.g528200.t1.1 pacid=30791672 transcript=Cre12.g528200.t1.1 locus=Cre12.g528200 ID=Cre12.g528200.t1.1.v5.5 annot-version=v5.5 Chlamydomonas_reinhardtii OG0000039
Cre12.g542450.t1.2 pacid=30791674 transcript=Cre12.g542450.t1.2 locus=Cre12.g542450 ID=Cre12.g542450.t1.2.v5.5 annot-version=v5.5 Chlamydomonas_reinhardtii OG0000039
Cre06.g263800.t1.1 pacid=30779441 transcript=Cre06.g263800.t1.1 locus=Cre06.g263800 ID=Cre06.g263800.t1.1.v5.5 annot-version=v5.5 Chlamydomonas_reinhardtii OG0000039
Cre09.g396139.t1.1 pacid=30780743 transcript=Cre09.g396139.t1.1 locus=Cre09.g396139 ID=Cre09.g396139.t1.1.v5.5 annot-version=v5.5 Chlamydomonas_reinhardtii OG0000039
Cre15.g641650.t1.1 pacid=30783668 transcript=Cre15.g641650.t1.1 locus=Cre15.g641650 ID=Cre15.g641650.t1.1.v5.5 annot-version=v5.5 Chlamydomonas_reinhardtii OG0000039
Cre07.g342450.t1.1 pacid=30774463 transcript=Cre07.g342450.t1.1 locus=Cre07.g342450 ID=Cre07.g342450.t1.1.v5.5 annot-version=v5.5 Chlamydomonas_reinhardtii OG0000039
Vocar.0012s0057.1.p pacid=32895034 transcript=Vocar.0012s0057.1 locus=Vocar.0012s0057 ID=Vocar.0012s0057.1.v2.1 annot-version=v2.1 Volvox_carteri OG0000039
Vocar.0012s0021.1.p pacid=32895052 transcript=Vocar.0012s0021.1 locus=Vocar.0012s0021 ID=Vocar.0012s0021.1.v2.1 annot-version=v2.1 Volvox_carteri OG0000039
Vocar.0011s0324.1.p pacid=32895436 transcript=Vocar.0011s0324.1 locus=Vocar.0011s0324 ID=Vocar.0011s0324.1.v2.1 annot-version=v2.1 Volvox_carteri OG0000039
Vocar.0081s0003.1.p pacid=32897471 transcript=Vocar.0081s0003.1 locus=Vocar.0081s0003 ID=Vocar.0081s0003.1.v2.1 annot-version=v2.1 Volvox_carteri OG0000039
Vocar.0037s0045.1.p pacid=32897619 transcript=Vocar.0037s0045.1 locus=Vocar.0037s0045 ID=Vocar.0037s0045.1.v2.1 annot-version=v2.1 Volvox_carteri OG0000039
Vocar.0001s1190.1.p pacid=32884881 transcript=Vocar.0001s1190.1 locus=Vocar.0001s1190 ID=Vocar.0001s1190.1.v2.1 annot-version=v2.1 Volvox_carteri OG0000039

Vocar.0001s0794.1.p pacid=32885484 transcript=Vocar.0001s0794.1 locus=Vocar.0001s0794 ID=Vocar.0001s0794.1.v2.1 annot-version=v2.1 Volvox_carteri OG0000039
Vocar.0001s0564.1.p pacid=32885754 transcript=Vocar.0001s0564.1 locus=Vocar.0001s0564 ID=Vocar.0001s0564.1.v2.1 annot-version=v2.1 Volvox_carteri OG0000039
Vocar.0051s0001.1.p pacid=32887015 transcript=Vocar.0051s0001.1 locus=Vocar.0051s0001 ID=Vocar.0051s0001.1.v2.1 annot-version=v2.1 Volvox_carteri OG0000039
Vocar.0002s0527.1.p pacid=32891287 transcript=Vocar.0002s0527.1 locus=Vocar.0002s0527 ID=Vocar.0002s0527.1.v2.1 annot-version=v2.1 Volvox_carteri OG0000039
Vocar.0036s0037.1.p pacid=32892267 transcript=Vocar.0036s0037.1 locus=Vocar.0036s0037 ID=Vocar.0036s0037.1.v2.1 annot-version=v2.1 Volvox_carteri OG0000039
Vocar.0019s0207.1.p pacid=32892639 transcript=Vocar.0019s0207.1 locus=Vocar.0019s0207 ID=Vocar.0019s0207.1.v2.1 annot-version=v2.1 Volvox_carteri OG0000039
Vocar.0067s0031.1.p pacid=32884149 transcript=Vocar.0067s0031.1 locus=Vocar.0067s0031 ID=Vocar.0067s0031.1.v2.1 annot-version=v2.1 Volvox_carteri OG0000039
Vocar.0001s1220.1.p pacid=32884304 transcript=Vocar.0001s1220.1 locus=Vocar.0001s1220 ID=Vocar.0001s1220.1.v2.1 annot-version=v2.1 Volvox_carteri OG0000039
jgi|ChlNC64A_1|140782|IGS.gm_3_00521 Chlorella_variabilis OG0000039
jgi|ChlNC64A_1|133757|IGS.gm_10_00048 Chlorella_variabilis OG0000039
jgi|ChlNC64A_1|133825|IGS.gm_10_00116 Chlorella_variabilis OG0000039
jgi|ChlNC64A_1|142077|IGS.gm_4_00218 Chlorella_variabilis OG0000039
jgi|ChlNC64A_1|59413|estExt_fgenesh3_pg.C_380038 Chlorella_variabilis OG0000039
jgi|ChlNC64A_1|57676|estExt_fgenesh3_pg.C_80231 Chlorella_variabilis OG0000039
jgi|ChlNC64A_1|58418|estExt_fgenesh3_pg.C_160009 Chlorella_variabilis OG0000039
jgi|ChlNC64A_1|137486|IGS.gm_19_00253 Chlorella_variabilis OG0000039
48445 pacid=27388118 transcript=48445 locus=estExt_fgenesh1_pg.C_140171 ID=48445.2.0.227 annot-version=v2.0 Coccomyxa_subellipsoidea OG0000039
16037 pacid=27388861 transcript=16037 locus=e_gw1.9.237.1 ID=16037.2.0.227 annot-version=v2.0 Coccomyxa_subellipsoidea OG0000039
46515 pacid=27390674 transcript=46515 locus=estExt_fgenesh1_pg.C_30344 ID=46515.2.0.227 annot-version=v2.0 Coccomyxa_subellipsoidea OG0000039
2407 pacid=27391877 transcript=2407 locus=gw1.11.80.1 ID=2407.2.0.227 annot-version=v2.0 Coccomyxa_subellipsoidea OG0000039
17170 pacid=27391922 transcript=17170 locus=e_gw1.11.123.1 ID=17170.2.0.227 annot-version=v2.0 Coccomyxa_subellipsoidea

OG0000039
47507 pacid=27392290 transcript=47507
locus=estExt_fgenesh1_pg.C_80121 ID=47507.2.0.227 annot-version=v2.0
Coccomyxa_subellipsoidea OG0000039
59056 pacid=27393074 transcript=59056 locus=Genemark1.1018_g
ID=59056.2.0.227 annot-version=v2.0 Coccomyxa_subellipsoidea
OG0000039
10620_fgenesh1_pg.C_Chr_04.0001000131 Ostreococcus_tauri
OG0000039
13231_fgenesh1_pg.C_Chr_13.0001000012 Ostreococcus_tauri
OG0000039
17507_e_gw1.05.00.261.1 Ostreococcus_tauri OG0000039
19728_e_gw1.10.00.189.1 Ostreococcus_tauri OG0000039
19765_e_gw1.10.00.139.1 Ostreococcus_tauri OG0000039
20012_e_gw1.11.00.269.1 Ostreococcus_tauri OG0000039
23338_estExt_fgenesh1_pg.C_Chr_02.00010134 Ostreococcus_tauri
OG0000039
24111_estExt_fgenesh1_pg.C_Chr_09.00010203 Ostreococcus_tauri
OG0000039
28654_estExt_gwp_GeneWisePlus.C_Chr_08.00010033
Ostreococcus_tauri OG0000039
34413_0800010300 Ostreococcus_tauri OG0000039
36172_1300010144 Ostreococcus_tauri OG0000039
36274_1300010246 Ostreococcus_tauri OG0000039
1242_gw1.07.00.84.1 Ostreococcus_tauri OG0000039
4992 pacid=27413880 transcript=4992 locus=gwEuk.9.267.1
ID=4992.2.0.231 annot-version=v2.0 Ostreococcus_lucimarinus
OG0000039
1178 pacid=27414035 transcript=1178 locus=gwEuk.7.72.1
ID=1178.2.0.231 annot-version=v2.0 Ostreococcus_lucimarinus
OG0000039
44917 pacid=27414513 transcript=44917
locus=estExt_GenewiseEukaryote.C_Chr_20373 ID=44917.2.0.231 annot-version=v2.0 Ostreococcus_lucimarinus OG0000039
30189 pacid=27414848 transcript=30189 locus=eugene.0200010376
ID=30189.2.0.231 annot-version=v2.0 Ostreococcus_lucimarinus
OG0000039
119646 pacid=27414952 transcript=119646 locus=eugene1.1.0200013760
ID=119646.2.0.231 annot-version=v2.0 Ostreococcus_lucimarinus
OG0000039
29705 pacid=27415228 transcript=29705 locus=eugene.2100010079
ID=29705.2.0.231 annot-version=v2.0 Ostreococcus_lucimarinus
OG0000039
49458 pacid=27415601 transcript=49458
locus=estExt_Genewise_ext.C_Chr_40547 ID=49458.2.0.231 annot-version=v2.0 Ostreococcus_lucimarinus OG0000039
37994 pacid=27416052 transcript=37994 locus=e_gwEuk.10.121.1
ID=37994.2.0.231 annot-version=v2.0 Ostreococcus_lucimarinus
OG0000039
37931 pacid=27416077 transcript=37931 locus=e_gwEuk.10.199.1
ID=37931.2.0.231 annot-version=v2.0 Ostreococcus_lucimarinus
OG0000039
27204 pacid=27418695 transcript=27204 locus=eugene.1300010226
ID=27204.2.0.231 annot-version=v2.0 Ostreococcus_lucimarinus

OG0000039
51262 pacid=27418833 transcript=51262
locus=estExt_Genewise_ext.C_Chr_130288 ID=51262.2.0.231 annot-version=v2.0 Ostreococcus_lucimarinus OG0000039
41525 pacid=27418845 transcript=41525 locus=e_gwEuk.13.89.1
ID=41525.2.0.231 annot-version=v2.0 Ostreococcus_lucimarinus
OG0000039
42380 pacid=27419313 transcript=42380 locus=e_gwEuk.8.274.1
ID=42380.2.0.231 annot-version=v2.0 Ostreococcus_lucimarinus
OG0000039
33296 pacid=27419593 transcript=33296 locus=eugene.0800010422
ID=33296.2.0.231 annot-version=v2.0 Ostreococcus_lucimarinus
OG0000039
443 pacid=27413615 transcript=443 locus=gwEuk.9.31.1 ID=443.2.0.231
annot-version=v2.0 Ostreococcus_lucimarinus OG0000039
2292 pacid=27420421 transcript=2292 locus=gwEuk.11.115.1
ID=2292.2.0.231 annot-version=v2.0 Ostreococcus_lucimarinus
OG0000039
16728 pacid=27413703 transcript=16728
locus=fgenesh1_pg.C_Chr_9000113 ID=16728.2.0.231 annot-version=v2.0
Ostreococcus_lucimarinus OG0000039
29100 pacid=27349186 transcript=29100
locus=MicpuC2.estExt_Genewise1.C_120049 ID=29100.3.0.228 annot-version=v3.0 Micromonas_pusilla OG0000039
142252 pacid=27338789 transcript=142292 locus=gw1.8.836.1
ID=142292.3.0.228 annot-version=v3.0 Micromonas_pusilla
OG0000039
133002 pacid=27340538 transcript=133042 locus=gw1.10.218.1
ID=133042.3.0.228 annot-version=v3.0 Micromonas_pusilla
OG0000039
187777 pacid=27340789 transcript=187817 locus=gm1.4898_g
ID=187817.3.0.228 annot-version=v3.0 Micromonas_pusilla
OG0000039
59572 pacid=27341228 transcript=59572 locus=MicpuC2.EuGene.
0000070421 ID=59572.3.0.228 annot-version=v3.0
Micromonas_pusilla OG0000039
197898 pacid=27341272 transcript=197938 locus=fgenesh1_pg.5_#_185
ID=197938.3.0.228 annot-version=v3.0 Micromonas_pusilla
OG0000039
58296 pacid=27341805 transcript=58296 locus=MicpuC2.EuGene.
0000050601 ID=58296.3.0.228 annot-version=v3.0
Micromonas_pusilla OG0000039
60082 pacid=27339028 transcript=60082 locus=MicpuC2.EuGene.
0000080276 ID=60082.3.0.228 annot-version=v3.0
Micromonas_pusilla OG0000039
133200 pacid=27343537 transcript=133240 locus=gw1.16.163.1
ID=133240.3.0.228 annot-version=v3.0 Micromonas_pusilla
OG0000039
11977 pacid=27339153 transcript=11977 locus=MicpuC2.gw1.8.628.1
ID=11977.3.0.228 annot-version=v3.0 Micromonas_pusilla
OG0000039
63385 pacid=27344094 transcript=63385 locus=MicpuC2.est_orfs.
9_2368_4277823:2 ID=63385.3.0.228 annot-version=v3.0
Micromonas_pusilla OG0000039

60839 pacid=27344260 transcript=60839 locus=MicpuC2.EuGene.
0000090390 ID=60839.3.0.228 annot-version=v3.0
Micromonas_pusilla	OG0000039
203622 pacid=27347347 transcript=203662 locus=fgenesh1_pm.3_#_73
ID=203662.3.0.228 annot-version=v3.0	Micromonas_pusilla
OG0000039
143282 pacid=27347851 transcript=143322 locus=gw1.6.899.1
ID=143322.3.0.228 annot-version=v3.0	Micromonas_pusilla
OG0000039
77160 pacid=27398225 transcript=77160 locus=gw2.03.1003.1
ID=77160.3.0.229 annot-version=v3.0	Micromonas_sp._RCC299
OG0000039
87113 pacid=27399818 transcript=87113 locus=e_gw2.12.69.1
ID=87113.3.0.229 annot-version=v3.0	Micromonas_sp._RCC299
OG0000039
77151 pacid=27395776 transcript=77151 locus=gw2.05.1004.1
ID=77151.3.0.229 annot-version=v3.0	Micromonas_sp._RCC299
OG0000039
99831 pacid=27400472 transcript=99831
locus=fgenesh2_pg.C_Chr_04000026 ID=99831.3.0.229 annot-version=v3.0
Micromonas_sp._RCC299	OG0000039
61481 pacid=27401158 transcript=61481 locus=EuGene.0900010464
ID=61481.3.0.229 annot-version=v3.0	Micromonas_sp._RCC299
OG0000039
84731 pacid=27401583 transcript=84731 locus=e_gw2.09.198.1
ID=84731.3.0.229 annot-version=v3.0	Micromonas_sp._RCC299
OG0000039
61222 pacid=27401692 transcript=61222 locus=EuGene.0900010205
ID=61222.3.0.229 annot-version=v3.0	Micromonas_sp._RCC299
OG0000039
60774 pacid=27403358 transcript=60774 locus=EuGene.0800010342
ID=60774.3.0.229 annot-version=v3.0	Micromonas_sp._RCC299
OG0000039
60859 pacid=27403552 transcript=60859 locus=EuGene.0800010427
ID=60859.3.0.229 annot-version=v3.0	Micromonas_sp._RCC299
OG0000039
65270 pacid=27403846 transcript=65270 locus=gw2.14.8.1
ID=65270.3.0.229 annot-version=v3.0	Micromonas_sp._RCC299
OG0000039
86278 pacid=27405009 transcript=86278 locus=e_gw2.11.94.1
ID=86278.3.0.229 annot-version=v3.0	Micromonas_sp._RCC299
OG0000039
76700 pacid=27405181 transcript=76700 locus=gw2.11.673.1
ID=76700.3.0.229 annot-version=v3.0	Micromonas_sp._RCC299
OG0000039
kfl00400_0090_v1.1	Klebsormidium_flaccidum	OG0000039
kfl00425_0060_v1.1	Klebsormidium_flaccidum	OG0000039
kfl00432_0070_v1.1	Klebsormidium_flaccidum	OG0000039
kfl00649_0050_v1.1	Klebsormidium_flaccidum	OG0000039
kfl00044_0020_v1.1	Klebsormidium_flaccidum	OG0000039
kfl00057_0030_v1.1	Klebsormidium_flaccidum	OG0000039
kfl00057_0520_v1.1	Klebsormidium_flaccidum	OG0000039
kfl00077_0020_v1.1	Klebsormidium_flaccidum	OG0000039
kfl00006_0480_v1.1	Klebsormidium_flaccidum	OG0000039

kfl00120_0100_v1.1   Klebsormidium_flaccidum OG0000039
kfl00152_0040_v1.1   Klebsormidium_flaccidum OG0000039
kfl00186_0060_v1.1   Klebsormidium_flaccidum OG0000039
kfl00250_0040_v1.1   Klebsormidium_flaccidum OG0000039
kfl00323_0120_v1.1   Klebsormidium_flaccidum OG0000039
Pp3c19_7860V3.1.p pacid=32937865 transcript=Pp3c19_7860V3.1
locus=Pp3c19_7860 annot-version=v3.3   Physcomitrella_patens
OG0000039
Pp3c22_1720V3.1.p pacid=32903508 transcript=Pp3c22_1720V3.1
locus=Pp3c22_1720 annot-version=v3.3   Physcomitrella_patens
OG0000039
Pp3c20_16510V3.1.p pacid=32945283 transcript=Pp3c20_16510V3.1
locus=Pp3c20_16510 annot-version=v3.3   Physcomitrella_patens
OG0000039
Pp3c23_9370V3.1.p pacid=32950230 transcript=Pp3c23_9370V3.1
locus=Pp3c23_9370 annot-version=v3.3   Physcomitrella_patens
OG0000039
Pp3c5_2640V3.1.p pacid=32953761 transcript=Pp3c5_2640V3.1
locus=Pp3c5_2640 annot-version=v3.3   Physcomitrella_patens
OG0000039
Pp3c5_28660V3.1.p pacid=32955498 transcript=Pp3c5_28660V3.1
locus=Pp3c5_28660 annot-version=v3.3   Physcomitrella_patens
OG0000039
Pp3c12_8920V3.1.p pacid=32972790 transcript=Pp3c12_8920V3.1
locus=Pp3c12_8920 annot-version=v3.3   Physcomitrella_patens
OG0000039
Pp3c6_7590V3.1.p pacid=32975999 transcript=Pp3c6_7590V3.1
locus=Pp3c6_7590 annot-version=v3.3   Physcomitrella_patens
OG0000039
Pp3c6_5360V3.1.p pacid=32977111 transcript=Pp3c6_5360V3.1
locus=Pp3c6_5360 annot-version=v3.3   Physcomitrella_patens
OG0000039
Pp3c25_3370V3.1.p pacid=32980010 transcript=Pp3c25_3370V3.1
locus=Pp3c25_3370 annot-version=v3.3   Physcomitrella_patens
OG0000039
Pp3c10_11160V3.1.p pacid=32901146 transcript=Pp3c10_11160V3.1
locus=Pp3c10_11160 annot-version=v3.3   Physcomitrella_patens
OG0000039
Pp3c4_21380V3.1.p pacid=32921804 transcript=Pp3c4_21380V3.1
locus=Pp3c4_21380 annot-version=v3.3   Physcomitrella_patens
OG0000039
Pp3c10_25870V3.1.p pacid=32901681 transcript=Pp3c10_25870V3.1
locus=Pp3c10_25870 annot-version=v3.3   Physcomitrella_patens
OG0000039
AT1G16800.1 | P-loop containing nucleoside triphosphate hydrolases
superfamily protein | Chr1:5745523-5755263 REVERSE LENGTH=2114 |
201606   Arabidopsis_thaliana   OG0000039
AT4G05540.1 | P-loop containing nucleoside triphosphate hydrolases
superfamily protein | Chr4:2818776-2821065 REVERSE LENGTH=689 |
201606   Arabidopsis_thaliana   OG0000039
AT4G15570.1 | P-loop containing nucleoside triphosphate hydrolases
superfamily protein | Chr4:8893043-8898858 FORWARD LENGTH=818 |
201606   Arabidopsis_thaliana   OG0000039
AT4G30100.1 | P-loop containing nucleoside triphosphate hydrolases

superfamily protein | Chr4:14714191-14719335 FORWARD LENGTH=1311 | 201606 Arabidopsis_thaliana OG0000039
AT5G35970.1 | P-loop containing nucleoside triphosphate hydrolases superfamily protein | Chr5:14119060-14123078 REVERSE LENGTH=961 | 201606 Arabidopsis_thaliana OG0000039
AT5G37030.1 | P-loop containing nucleoside triphosphate hydrolases superfamily protein | Chr5:14634614-14636939 REVERSE LENGTH=638 | 201606 Arabidopsis_thaliana OG0000039
AT5G37140.1 | P-loop containing nucleoside triphosphate hydrolases superfamily protein | Chr5:14690436-14692851 FORWARD LENGTH=692 | 201606 Arabidopsis_thaliana OG0000039
AT5G37150.1 | P-loop containing nucleoside triphosphate hydrolases superfamily protein | Chr5:14701330-14704562 FORWARD LENGTH=839 | 201606 Arabidopsis_thaliana OG0000039
AT5G37165.1 | P-loop nucleoside triphosphate hydrolase superfamily protein | Chr5:14707022-14708376 FORWARD LENGTH=424 | 201606 Arabidopsis_thaliana OG0000039
AT5G47010.1 | RNA helicase | Chr5:19072009-19078856 FORWARD LENGTH=1254 | 201606 Arabidopsis_thaliana OG0000039
AT5G52090.1 | P-loop containing nucleoside triphosphate hydrolases superfamily protein | Chr5:21167432-21169462 REVERSE LENGTH=676 | 201606 Arabidopsis_thaliana OG0000039
AT1G05460.1 | P-loop containing nucleoside triphosphate hydrolases superfamily protein | Chr1:1601357-1604658 REVERSE LENGTH=1002 | 201606 Arabidopsis_thaliana OG0000039
AT1G65780.1 | P-loop containing nucleoside triphosphate hydrolases superfamily protein | Chr1:24462958-24466888 REVERSE LENGTH=1065 | 201606 Arabidopsis_thaliana OG0000039
AT1G65810.1 | P-loop containing nucleoside triphosphate hydrolases superfamily protein | Chr1:24477043-24480728 REVERSE LENGTH=1050 | 201606 Arabidopsis_thaliana OG0000039
AT2G03270.1 | DNA-binding protein | Chr2:994071-995990 FORWARD LENGTH=639 | 201606 Arabidopsis_thaliana OG0000039
AT2G19120.1 | P-loop containing nucleoside triphosphate hydrolases superfamily protein | Chr2:8287036-8291802 REVERSE LENGTH=1090 | 201606 Arabidopsis_thaliana OG0000039
AT1G08840.1 | DNA replication helicase | Chr1:2829579-2838369 REVERSE LENGTH=1296 | 201606 Arabidopsis_thaliana OG0000039
Os03t0440200-00 Similar to OSIGBa0076I14.10 protein. Oryza_sativa OG0000039
Os03t0586900-01 DNA helicase, putative domain containing protein. Oryza_sativa OG0000039
Os04t0424200-00 Similar to OSIGBa0076I14.10 protein. Oryza_sativa OG0000039
Os04t0582000-00 Conserved hypothetical protein. Oryza_sativa OG0000039
Os04t0582600-01 Conserved hypothetical protein. Oryza_sativa OG0000039
Os04t0582700-00 Conserved hypothetical protein. Oryza_sativa OG0000039
Os04t0582900-01 Peptidase, cysteine peptidase active site domain containing protein. Oryza_sativa OG0000039
Os04t0588200-00 Similar to emb2411 (embryo defective 2411); ATP

binding / ATP-dependent DNA helicase/ DNA binding. Oryza_sativa
OG0000039
Os06t0310200-01 Similar to OSIGBa0076I14.10 protein.
Oryza_sativa OG0000039
Os07t0495900-01 Similar to low-level beta-amylase 1.
Oryza_sativa OG0000039
Os09t0130800-01 Similar to predicted protein. Oryza_sativa
OG0000039
Os10t0118900-01 Similar to tRNA-splicing endonuclease positive
effector-related. Oryza_sativa OG0000039
Os10t0537600-01 Conserved hypothetical protein. Oryza_sativa
OG0000039
Os02t0684150-00 Conserved hypothetical protein. Oryza_sativa
OG0000039
Os02t0704300-01 Conserved hypothetical protein. Oryza_sativa
OG0000039
Os03t0160400-01 Similar to ACL098Cp. Oryza_sativa
OG0000039
CMN106CT pep chromosome:ASM9120v1:14:276944:280886:-1 gene:CMN106C
transcript:CMN106CT gene_biotype:protein_coding
transcript_biotype:protein_coding Cyanidioschyzon_merolae
OG0000039
CMS334CT pep chromosome:ASM9120v1:19:856617:859943:1 gene:CMS334C
transcript:CMS334CT gene_biotype:protein_coding
transcript_biotype:protein_coding Cyanidioschyzon_merolae
OG0000039
CMK133CT pep chromosome:ASM9120v1:11:354646:359329:-1 gene:CMK133C
transcript:CMK133CT gene_biotype:protein_coding
transcript_biotype:protein_coding Cyanidioschyzon_merolae
OG0000039
CMP054CT pep chromosome:ASM9120v1:16:143759:147512:-1 gene:CMP054C
transcript:CMP054CT gene_biotype:protein_coding
transcript_biotype:protein_coding Cyanidioschyzon_merolae
OG0000039
evm.model.contig_2032.20 Porphyridium_purpureum OG0000039
evm.model.contig_2032.32 Porphyridium_purpureum OG0000039
evm.model.contig_3581.2 Porphyridium_purpureum OG0000039
evm.model.contig_4399.1 Porphyridium_purpureum OG0000039
evm.model.contig_2044.12 Porphyridium_purpureum OG0000039
evm.model.contig_4432.7 Porphyridium_purpureum OG0000039
evm.model.contig_460.2 Porphyridium_purpureum OG0000039
evm.model.contig_707.2 Porphyridium_purpureum OG0000039
CDF41218 pep supercontig:ASM35022v2:HG002328:170709:174095:1
gene:CHC_T00007752001 transcript:CDF41218
gene_biotype:protein_coding transcript_biotype:protein_coding
Chondrus_crispus OG0000039
CDF41103 pep supercontig:ASM35022v2:HG002309:242812:246550:1
gene:CHC_T00007327001 transcript:CDF41103
gene_biotype:protein_coding transcript_biotype:protein_coding
Chondrus_crispus OG0000039
CDF41405 pep supercontig:ASM35022v2:HG002370:188867:190522:1
gene:CHC_T00007991001 transcript:CDF41405
gene_biotype:protein_coding transcript_biotype:protein_coding
Chondrus_crispus OG0000039

CDF32944 pep supercontig:ASM35022v2:HG001623:73343:81094:-1
gene:CHC_T00001778001 transcript:CDF32944
gene_biotype:protein_coding transcript_biotype:protein_coding
Chondrus_crispus OG0000039
CDF40866 pep supercontig:ASM35022v2:HG002260:99486:103506:1
gene:CHC_T00007503001 transcript:CDF40866
gene_biotype:protein_coding transcript_biotype:protein_coding
Chondrus_crispus OG0000039
CDF35430 pep supercontig:ASM35022v2:HG001730:47197:52057:-1
gene:CHC_T00003946001 transcript:CDF35430
gene_biotype:protein_coding transcript_biotype:protein_coding
Chondrus_crispus OG0000039
g3643.t1 Caulerpa_lentillifera OG0000040
Cre03.g205850.t1.2 pacid=30788191 transcript=Cre03.g205850.t1.2
locus=Cre03.g205850 ID=Cre03.g205850.t1.2.v5.5 annot-version=v5.5
Chlamydomonas_reinhardtii OG0000040
Cre01.g014150.t1.2 pacid=30788361 transcript=Cre01.g014150.t1.2
locus=Cre01.g014150 ID=Cre01.g014150.t1.2.v5.5 annot-version=v5.5
Chlamydomonas_reinhardtii OG0000040
Cre01.g027764.t1.2 pacid=30789265 transcript=Cre01.g027764.t1.2
locus=Cre01.g027764 ID=Cre01.g027764.t1.2.v5.5 annot-version=v5.5
Chlamydomonas_reinhardtii OG0000040
Cre01.g007901.t1.1 pacid=30789348 transcript=Cre01.g007901.t1.1
locus=Cre01.g007901 ID=Cre01.g007901.t1.1.v5.5 annot-version=v5.5
Chlamydomonas_reinhardtii OG0000040
Cre01.g051211.t1.1 pacid=30789421 transcript=Cre01.g051211.t1.1
locus=Cre01.g051211 ID=Cre01.g051211.t1.1.v5.5 annot-version=v5.5
Chlamydomonas_reinhardtii OG0000040
Cre10.g445850.t1.1 pacid=30790079 transcript=Cre10.g445850.t1.1
locus=Cre10.g445850 ID=Cre10.g445850.t1.1.v5.5 annot-version=v5.5
Chlamydomonas_reinhardtii OG0000040
Cre10.g444900.t1.1 pacid=30790721 transcript=Cre10.g444900.t1.1
locus=Cre10.g444900 ID=Cre10.g444900.t1.1.v5.5 annot-version=v5.5
Chlamydomonas_reinhardtii OG0000040
Cre12.g523250.t1.1 pacid=30792890 transcript=Cre12.g523250.t1.1
locus=Cre12.g523250 ID=Cre12.g523250.t1.1.v5.5 annot-version=v5.5
Chlamydomonas_reinhardtii OG0000040
Cre12.g499700.t1.1 pacid=30793636 transcript=Cre12.g499700.t1.1
locus=Cre12.g499700 ID=Cre12.g499700.t1.1.v5.5 annot-version=v5.5
Chlamydomonas_reinhardtii OG0000040
Cre14.g610750.t1.1 pacid=30776145 transcript=Cre14.g610750.t1.1
locus=Cre14.g610750 ID=Cre14.g610750.t1.1.v5.5 annot-version=v5.5
Chlamydomonas_reinhardtii OG0000040
Cre14.g625350.t1.1 pacid=30776774 transcript=Cre14.g625350.t1.1
locus=Cre14.g625350 ID=Cre14.g625350.t1.1.v5.5 annot-version=v5.5
Chlamydomonas_reinhardtii OG0000040
Vocar.0001s0891.1.p pacid=32884363 transcript=Vocar.0001s0891.1
locus=Vocar.0001s0891 ID=Vocar.0001s0891.1.v2.1 annot-version=v2.1
Volvox_carteri OG0000040
Vocar.0007s0278.1.p pacid=32888315 transcript=Vocar.0007s0278.1
locus=Vocar.0007s0278 ID=Vocar.0007s0278.1.v2.1 annot-version=v2.1
Volvox_carteri OG0000040
Vocar.0004s0320.1.p pacid=32889453 transcript=Vocar.0004s0320.1
locus=Vocar.0004s0320 ID=Vocar.0004s0320.1.v2.1 annot-version=v2.1

Volvox_carteri	OG0000040
Vocar.0024s0041.1.p pacid=32890321 transcript=Vocar.0024s0041.1 locus=Vocar.0024s0041 ID=Vocar.0024s0041.1.v2.1 annot-version=v2.1	Volvox_carteri	OG0000040
Vocar.0024s0193.1.p pacid=32890492 transcript=Vocar.0024s0193.1 locus=Vocar.0024s0193 ID=Vocar.0024s0193.1.v2.1 annot-version=v2.1	Volvox_carteri	OG0000040
Vocar.0050s0043.1.p pacid=32892363 transcript=Vocar.0050s0043.1 locus=Vocar.0050s0043 ID=Vocar.0050s0043.1.v2.1 annot-version=v2.1	Volvox_carteri	OG0000040
Vocar.0006s0460.1.p pacid=32894033 transcript=Vocar.0006s0460.1 locus=Vocar.0006s0460 ID=Vocar.0006s0460.1.v2.1 annot-version=v2.1	Volvox_carteri	OG0000040
jgi|ChlNC64A_1|35872|estExt_Genewise1Plus.C_120585	Chlorella_variabilis	OG0000040
jgi|ChlNC64A_1|19008|e_gw1.2.125.1	Chlorella_variabilis	OG0000040
jgi|ChlNC64A_1|133260|IGS.gm_1_00396	Chlorella_variabilis	OG0000040
jgi|ChlNC64A_1|142267|IGS.gm_4_00408	Chlorella_variabilis	OG0000040
jgi|ChlNC64A_1|26336|e_gw1.20.47.1	Chlorella_variabilis	OG0000040
jgi|ChlNC64A_1|54230|fgenesh3_pg.C_scaffold_20000103	Chlorella_variabilis	OG0000040
jgi|ChlNC64A_1|144825|IGS.gm_8_00153	Chlorella_variabilis	OG0000040
jgi|ChlNC64A_1|144826|IGS.gm_8_00154	Chlorella_variabilis	OG0000040
jgi|ChlNC64A_1|58521|estExt_fgenesh3_pg.C_170048	Chlorella_variabilis	OG0000040
20364 pacid=27387756 transcript=20364 locus=e_gw1.22.61.1 ID=20364.2.0.227 annot-version=v2.0	Coccomyxa_subellipsoidea	OG0000040
55602 pacid=27389597 transcript=55602 locus=Genemark1.546_g ID=55602.2.0.227 annot-version=v2.0	Coccomyxa_subellipsoidea	OG0000040
40375 pacid=27390073 transcript=40375 locus=fgenesh1_pg.4_#_24 ID=40375.2.0.227 annot-version=v2.0	Coccomyxa_subellipsoidea	OG0000040
61075 pacid=27390701 transcript=61075 locus=Genemark1.1886_g ID=61075.2.0.227 annot-version=v2.0	Coccomyxa_subellipsoidea	OG0000040
10548_fgenesh1_pg.C_Chr_04.0001000059	Ostreococcus_tauri	OG0000040
30071_0100010059 Ostreococcus_tauri	OG0000040
35721_1200010019 Ostreococcus_tauri	OG0000040
37346_1700010022 Ostreococcus_tauri	OG0000040
2943 pacid=27415532 transcript=2943 locus=gwEuk.4.216.1 ID=2943.2.0.231 annot-version=v2.0	Ostreococcus_lucimarinus	OG0000040
27967 pacid=27415998 transcript=27967 locus=eugene.1600010023 ID=27967.2.0.231 annot-version=v2.0	Ostreococcus_lucimarinus	OG0000040

47105 pacid=27417073 transcript=47105
locus=estExt_GenewiseEukaryote.C_Chr_120109 ID=47105.2.0.231 annot-version=v2.0	Ostreococcus_lucimarinus	OG0000040
3441 pacid=27417476 transcript=3441 locus=gwEuk.1.299.1
ID=3441.2.0.231 annot-version=v2.0	Ostreococcus_lucimarinus
OG0000040
2767 pacid=27348941 transcript=2767 locus=MicpuC2.gw1.12.100.1
ID=2767.3.0.228 annot-version=v3.0	Micromonas_pusilla
OG0000040
34110 pacid=27340920 transcript=34110
locus=MicpuC2.estExt_Genewise1Plus.C_70059 ID=34110.3.0.228 annot-version=v3.0	Micromonas_pusilla	OG0000040
81649 pacid=27395875 transcript=81649 locus=e_gw2.05.148.1
ID=81649.3.0.229 annot-version=v3.0	Micromonas_sp._RCC299
OG0000040
50445 pacid=27401683 transcript=50445
locus=est_orfs.Chr_09_316_3199936:1 ID=50445.3.0.229 annot-version=v3.0	Micromonas_sp._RCC299	OG0000040
87767 pacid=27402619 transcript=87767 locus=e_gw2.13.152.1
ID=87767.3.0.229 annot-version=v3.0	Micromonas_sp._RCC299
OG0000040
75308 pacid=27402928 transcript=75308 locus=gw2.13.490.1
ID=75308.3.0.229 annot-version=v3.0	Micromonas_sp._RCC299
OG0000040
kfl00346_0010_v1.1	Klebsormidium_flaccidum	OG0000040
kfl00471_0030_v1.1	Klebsormidium_flaccidum	OG0000040
kfl00481_0080_v1.1	Klebsormidium_flaccidum	OG0000040
kfl00481_0100_v1.1	Klebsormidium_flaccidum	OG0000040
kfl00545_0030_v1.1	Klebsormidium_flaccidum	OG0000040
kfl00018_0590_v1.1	Klebsormidium_flaccidum	OG0000040
kfl00615_0080_v1.1	Klebsormidium_flaccidum	OG0000040
kfl00684_0060_v1.1	Klebsormidium_flaccidum	OG0000040
kfl00021_0050_v1.1	Klebsormidium_flaccidum	OG0000040
kfl00884_0020_v1.1	Klebsormidium_flaccidum	OG0000040
kfl00030_0420_v1.1	Klebsormidium_flaccidum	OG0000040
kfl00044_0200_v1.1	Klebsormidium_flaccidum	OG0000040
kfl00047_0290_v1.1	Klebsormidium_flaccidum	OG0000040
kfl00084_0100_v1.1	Klebsormidium_flaccidum	OG0000040
kfl00097_0030_v1.1	Klebsormidium_flaccidum	OG0000040
kfl00174_0160_v1.1	Klebsormidium_flaccidum	OG0000040
kfl00174_0220_v1.1	Klebsormidium_flaccidum	OG0000040
kfl00011_0410_v1.1	Klebsormidium_flaccidum	OG0000040
kfl00225_0230_v1.1	Klebsormidium_flaccidum	OG0000040
kfl00232_0040_v1.1	Klebsormidium_flaccidum	OG0000040
kfl00258_0100_v1.1	Klebsormidium_flaccidum	OG0000040
Pp3c10_10570V3.1.p pacid=32899636 transcript=Pp3c10_10570V3.1
locus=Pp3c10_10570 annot-version=v3.3	Physcomitrella_patens
OG0000040
Pp3c2_12080V3.1.p pacid=32936831 transcript=Pp3c2_12080V3.1
locus=Pp3c2_12080 annot-version=v3.3	Physcomitrella_patens
OG0000040
Pp3c10_15660V3.1.p pacid=32899393 transcript=Pp3c10_15660V3.1
locus=Pp3c10_15660 annot-version=v3.3	Physcomitrella_patens
OG0000040

Pp3c5_21630V3.1.p pacid=32954774 transcript=Pp3c5_21630V3.1 locus=Pp3c5_21630 annot-version=v3.3	Physcomitrella_patens	OG0000040
Pp3c14_17840V3.1.p pacid=32960845 transcript=Pp3c14_17840V3.1 locus=Pp3c14_17840 annot-version=v3.3	Physcomitrella_patens	OG0000040
Pp3c14_23000V3.1.p pacid=32961134 transcript=Pp3c14_23000V3.1 locus=Pp3c14_23000 annot-version=v3.3	Physcomitrella_patens	OG0000040
Pp3c1_37630V3.1.p pacid=32968127 transcript=Pp3c1_37630V3.1 locus=Pp3c1_37630 annot-version=v3.3	Physcomitrella_patens	OG0000040
Pp3c1_37590V3.1.p pacid=32968440 transcript=Pp3c1_37590V3.1 locus=Pp3c1_37590 annot-version=v3.3	Physcomitrella_patens	OG0000040
Pp3c12_15740V3.1.p pacid=32974371 transcript=Pp3c12_15740V3.1 locus=Pp3c12_15740 annot-version=v3.3	Physcomitrella_patens	OG0000040
Pp3c6_11630V3.1.p pacid=32977123 transcript=Pp3c6_11630V3.1 locus=Pp3c6_11630 annot-version=v3.3	Physcomitrella_patens	OG0000040
Pp3c17_22040V3.1.p pacid=32908097 transcript=Pp3c17_22040V3.1 locus=Pp3c17_22040 annot-version=v3.3	Physcomitrella_patens	OG0000040
Pp3c4_16610V3.1.p pacid=32922206 transcript=Pp3c4_16610V3.1 locus=Pp3c4_16610 annot-version=v3.3	Physcomitrella_patens	OG0000040
Pp3c10_17490V3.1.p pacid=32901699 transcript=Pp3c10_17490V3.1 locus=Pp3c10_17490 annot-version=v3.3	Physcomitrella_patens	OG0000040
AT2G38510.1 | MATE efflux family protein | Chr2:16123985-16125445 FORWARD LENGTH=486 | 201606	Arabidopsis_thaliana	OG0000040
AT1G11670.1 | MATE efflux family protein | Chr1:3928520-3931482 REVERSE LENGTH=503 | 201606	Arabidopsis_thaliana	OG0000040
AT3G03620.1 | MATE efflux family protein | Chr3:873904-876252 REVERSE LENGTH=500 | 201606	Arabidopsis_thaliana	OG0000040
AT1G12950.1 | root hair specific 2 | Chr1:4419849-4422462 FORWARD LENGTH=522 | 201606	Arabidopsis_thaliana	OG0000040
AT3G21690.1 | MATE efflux family protein | Chr3:7638750-7641861 FORWARD LENGTH=506 | 201606	Arabidopsis_thaliana	OG0000040
AT3G23550.1 | MATE efflux family protein | Chr3:8448435-8450649 REVERSE LENGTH=469 | 201606	Arabidopsis_thaliana	OG0000040
AT3G23560.1 | MATE efflux family protein | Chr3:8454361-8456588 REVERSE LENGTH=477 | 201606	Arabidopsis_thaliana	OG0000040
AT3G26590.1 | MATE efflux family protein | Chr3:9761927-9765259 REVERSE LENGTH=500 | 201606	Arabidopsis_thaliana	OG0000040
AT1G15150.1 | MATE efflux family protein | Chr1:5212674-5214723

FORWARD LENGTH=487 | 201606	Arabidopsis_thaliana
OG0000040
AT1G15160.1 | MATE efflux family protein | Chr1:5215475-5217545
FORWARD LENGTH=487 | 201606	Arabidopsis_thaliana
OG0000040
AT1G15170.1 | MATE efflux family protein | Chr1:5220690-5222756
FORWARD LENGTH=481 | 201606	Arabidopsis_thaliana
OG0000040
AT1G15180.1 | MATE efflux family protein | Chr1:5224452-5226531
FORWARD LENGTH=482 | 201606	Arabidopsis_thaliana
OG0000040
AT3G59030.1 | MATE efflux family protein | Chr3:21819124-21821288
FORWARD LENGTH=507 | 201606	Arabidopsis_thaliana
OG0000040
AT4G00350.1 | MATE efflux family protein | Chr4:151978-153988
FORWARD LENGTH=542 | 201606	Arabidopsis_thaliana
OG0000040
AT4G21903.1 | MATE efflux family protein | Chr4:11621169-11623738
REVERSE LENGTH=504 | 201606	Arabidopsis_thaliana
OG0000040
AT4G21910.1 | MATE efflux family protein | Chr4:11625742-11630964
REVERSE LENGTH=507 | 201606	Arabidopsis_thaliana
OG0000040
AT4G22790.1 | MATE efflux family protein | Chr4:11975153-11976628
REVERSE LENGTH=491 | 201606	Arabidopsis_thaliana
OG0000040
AT4G23030.1 | MATE efflux family protein | Chr4:12072857-12074365
FORWARD LENGTH=502 | 201606	Arabidopsis_thaliana
OG0000040
AT4G25640.1 | detoxifying efflux carrier 35 | Chr4:13076953-13078965
REVERSE LENGTH=488 | 201606	Arabidopsis_thaliana
OG0000040
AT4G29140.1 | MATE efflux family protein | Chr4:14369148-14370746
FORWARD LENGTH=532 | 201606	Arabidopsis_thaliana
OG0000040
AT5G10420.1 | MATE efflux family protein | Chr5:3273578-3276490
REVERSE LENGTH=489 | 201606	Arabidopsis_thaliana
OG0000040
AT5G17700.1 | MATE efflux family protein | Chr5:5831025-5833415
REVERSE LENGTH=497 | 201606	Arabidopsis_thaliana
OG0000040
AT5G19700.1 | MATE efflux family protein | Chr5:6660821-6662347
REVERSE LENGTH=508 | 201606	Arabidopsis_thaliana
OG0000040
AT1G23300.1 | MATE efflux family protein | Chr1:8263827-8266048
REVERSE LENGTH=515 | 201606	Arabidopsis_thaliana
OG0000040
AT5G38030.1 | MATE efflux family protein | Chr5:15171486-15175302
REVERSE LENGTH=498 | 201606	Arabidopsis_thaliana
OG0000040
AT5G44050.1 | MATE efflux family protein | Chr5:17722484-17726209
FORWARD LENGTH=491 | 201606	Arabidopsis_thaliana
OG0000040
AT5G49130.1 | MATE efflux family protein | Chr5:19915904-19917525

FORWARD LENGTH=502 | 201606   Arabidopsis_thaliana
OG0000040
AT5G52050.1 | MATE efflux family protein | Chr5:21138933-21140450
FORWARD LENGTH=505 | 201606   Arabidopsis_thaliana
OG0000040
AT5G52450.1 | MATE efflux family protein | Chr5:21289042-21291749
REVERSE LENGTH=486 | 201606   Arabidopsis_thaliana
OG0000040
AT5G65380.1 | MATE efflux family protein | Chr5:26123241-26126352
REVERSE LENGTH=486 | 201606   Arabidopsis_thaliana
OG0000040
AT1G33080.1 | MATE efflux family protein | Chr1:11985752-11990327
FORWARD LENGTH=494 | 201606   Arabidopsis_thaliana
OG0000040
AT1G33090.1 | MATE efflux family protein | Chr1:11993458-11996542
FORWARD LENGTH=494 | 201606   Arabidopsis_thaliana
OG0000040
AT1G33100.1 | MATE efflux family protein | Chr1:11997683-12001308
FORWARD LENGTH=491 | 201606   Arabidopsis_thaliana
OG0000040
AT1G33110.1 | MATE efflux family protein | Chr1:12005084-12008618
FORWARD LENGTH=494 | 201606   Arabidopsis_thaliana
OG0000040
AT1G47530.1 | MATE efflux family protein | Chr1:17451724-17454110
FORWARD LENGTH=484 | 201606   Arabidopsis_thaliana
OG0000040
AT1G58340.1 | MATE efflux family protein | Chr1:21653162-21655117
FORWARD LENGTH=532 | 201606   Arabidopsis_thaliana
OG0000040
AT1G61890.1 | MATE efflux family protein | Chr1:22868103-22871163
REVERSE LENGTH=501 | 201606   Arabidopsis_thaliana
OG0000040
AT1G64820.1 | MATE efflux family protein | Chr1:24088605-24090558
FORWARD LENGTH=502 | 201606   Arabidopsis_thaliana
OG0000040
AT1G66760.1 | MATE efflux family protein | Chr1:24902110-24904054
FORWARD LENGTH=466 | 201606   Arabidopsis_thaliana
OG0000040
AT1G66780.1 | MATE efflux family protein | Chr1:24909213-24911485
FORWARD LENGTH=485 | 201606   Arabidopsis_thaliana
OG0000040
AT1G71140.1 | MATE efflux family protein | Chr1:26824762-26826748
FORWARD LENGTH=485 | 201606   Arabidopsis_thaliana
OG0000040
AT1G71870.1 | MATE efflux family protein | Chr1:27032456-27034895
REVERSE LENGTH=510 | 201606   Arabidopsis_thaliana
OG0000040
AT1G73700.1 | MATE efflux family protein | Chr1:27717554-27719630
REVERSE LENGTH=476 | 201606   Arabidopsis_thaliana
OG0000040
AT2G04040.1 | MATE efflux family protein | Chr2:1334614-1336480
REVERSE LENGTH=476 | 201606   Arabidopsis_thaliana
OG0000040
AT2G04050.1 | MATE efflux family protein | Chr2:1337386-1339270

REVERSE LENGTH=476 | 201606 Arabidopsis_thaliana
OG0000040
AT2G04066.1 | MATE efflux family protein | Chr2:1352887-1353517
REVERSE LENGTH=171 | 201606 Arabidopsis_thaliana
OG0000040
AT2G04070.1 | MATE efflux family protein | Chr2:1353947-1355790
REVERSE LENGTH=476 | 201606 Arabidopsis_thaliana
OG0000040
AT2G04080.1 | MATE efflux family protein | Chr2:1357327-1359159
REVERSE LENGTH=476 | 201606 Arabidopsis_thaliana
OG0000040
AT2G04090.1 | MATE efflux family protein | Chr2:1362653-1364690
REVERSE LENGTH=477 | 201606 Arabidopsis_thaliana
OG0000040
AT2G04100.1 | MATE efflux family protein | Chr2:1377020-1379051
REVERSE LENGTH=483 | 201606 Arabidopsis_thaliana
OG0000040
AT2G34360.1 | MATE efflux family protein | Chr2:14507294-14510231
FORWARD LENGTH=480 | 201606 Arabidopsis_thaliana
OG0000040
Os03t0570800-01 Multi antimicrobial extrusion protein MatE family
protein. Oryza_sativa OG0000040
Os03t0571700-01 Similar to TRANSPARENT TESTA 12 protein.
Oryza_sativa OG0000040
Os03t0571900-01 Similar to transparent testa 12 protein.
Oryza_sativa OG0000040
Os03t0572900-01 Multi antimicrobial extrusion protein MatE family
protein. Oryza_sativa OG0000040
Os03t0626700-01 Multi antimicrobial extrusion protein MatE family
protein. Oryza_sativa OG0000040
Os03t0839200-01 Multi antimicrobial extrusion protein MatE family
protein. Oryza_sativa OG0000040
Os03t0858800-01 Multi antimicrobial extrusion protein MatE family
protein. Oryza_sativa OG0000040
Os04t0373400-01 Similar to MATE efflux family protein, expressed.
Oryza_sativa OG0000040
Os04t0571600-01 Multi antimicrobial extrusion protein MatE family
protein. Oryza_sativa OG0000040
Os05t0554000-01 Similar to cDNA clone:001-123-D07, full insert
sequence. Oryza_sativa OG0000040
Os06t0494400-01 Multi antimicrobial extrusion protein MatE family
protein. Oryza_sativa OG0000040
Os06t0495100-00 Similar to cDNA, clone Oryza_sativa
OG0000040
Os06t0495500-01 Multi antimicrobial extrusion protein MatE family
protein. Oryza_sativa OG0000040
Os06t0558300-00 Similar to MATE efflux protein-related.
Oryza_sativa OG0000040
Os06t0707100-01 Multi antimicrobial extrusion protein MatE family
protein. Oryza_sativa OG0000040
Os07t0108200-00 Similar to transparent testa 12 protein.
Oryza_sativa OG0000040
Os07t0502200-01 Multi antimicrobial extrusion protein MatE family
protein. Oryza_sativa OG0000040

Os07t0516600-01 Multi antimicrobial extrusion protein MatE family protein. Oryza_sativa OG0000040
Os08t0480000-01 Multi antimicrobial extrusion protein MatE family protein. Oryza_sativa OG0000040
Os08t0545900-00 Multi antimicrobial extrusion protein MatE family protein. Oryza_sativa OG0000040
Os08t0550200-01 Multi antimicrobial extrusion protein MatE family protein. Oryza_sativa OG0000040
Os08t0562800-01 Similar to Transparent testa 12 protein. Oryza_sativa OG0000040
Os01t0684900-01 Multi antimicrobial extrusion protein MatE family protein. Oryza_sativa OG0000040
Os09t0468000-01 Multi antimicrobial extrusion protein MatE family protein. Oryza_sativa OG0000040
Os09t0524300-00 Multi antimicrobial extrusion protein MatE family protein. Oryza_sativa OG0000040
Os10t0190900-01 Multi antimicrobial extrusion protein MatE family protein. Oryza_sativa OG0000040
Os10t0195000-01 Multi antimicrobial extrusion protein MatE family protein. Oryza_sativa OG0000040
Os10t0344500-00 Similar to cDNA clone:001-128-E01, full insert sequence. Oryza_sativa OG0000040
Os10t0344900-01 Multi antimicrobial extrusion protein MatE family protein. Oryza_sativa OG0000040
Os10t0345100-01 Multi antimicrobial extrusion protein MatE family protein. Oryza_sativa OG0000040
Os11t0126100-01 Multi antimicrobial extrusion protein MatE family protein. Oryza_sativa OG0000040
Os11t0129000-00 Similar to Transparent testa 12 protein. Oryza_sativa OG0000040
Os11t0129200-00 Multi antimicrobial extrusion protein MatE family protein. Oryza_sativa OG0000040
Os12t0125800-00 Similar to Transparent testa 12 protein. Oryza_sativa OG0000040
Os12t0126000-01 Multi antimicrobial extrusion protein MatE family protein. Oryza_sativa OG0000040
Os01t0766000-00 Multi antimicrobial extrusion protein MatE family protein. Oryza_sativa OG0000040
Os12t0552600-00 Similar to MATE efflux family protein. Oryza_sativa OG0000040
Os12t0615700-01 Similar to TRANSPARENT TESTA 12 protein. Oryza_sativa OG0000040
Os02t0676400-00 Multi antimicrobial extrusion protein MatE family protein. Oryza_sativa OG0000040
Os02t0821600-00 Similar to mate efflux family protein. Oryza_sativa OG0000040
Os03t0188100-01 Similar to transparent testa 12 protein. Oryza_sativa OG0000040
Os03t0229500-00 Similar to MATE efflux family protein, expressed. Oryza_sativa OG0000040
CMI258CT pep chromosome:ASM9120v1:9:686280:687954:-1 gene:CMI258C transcript:CMI258CT gene_biotype:protein_coding transcript_biotype:protein_coding Cyanidioschyzon_merolae OG0000040

evm.model.contig_481.15	Porphyridium_purpureum	OG0000040
g8376.t1 Caulerpa_lentillifera	OG0000041
kfl00170_0010_v1.1	Klebsormidium_flaccidum	OG0000041
kfl00224_0170_v1.1	Klebsormidium_flaccidum	OG0000041
kfl00295_0020_v1.1	Klebsormidium_flaccidum	OG0000041
kfl00295_0030_v1.1	Klebsormidium_flaccidum	OG0000041
Pp3c10_21693V3.1.p pacid=32899641 transcript=Pp3c10_21693V3.1 locus=Pp3c10_21693 annot-version=v3.3	Physcomitrella_patens OG0000041
Pp3c22_5030V3.1.p pacid=32902954 transcript=Pp3c22_5030V3.1 locus=Pp3c22_5030 annot-version=v3.3	Physcomitrella_patens OG0000041
Pp3c22_1030V3.1.p pacid=32904295 transcript=Pp3c22_1030V3.1 locus=Pp3c22_1030 annot-version=v3.3	Physcomitrella_patens OG0000041
Pp3c14_8070V3.1.p pacid=32962972 transcript=Pp3c14_8070V3.1 locus=Pp3c14_8070 annot-version=v3.3	Physcomitrella_patens OG0000041
Pp3c14_8120V3.1.p pacid=32963202 transcript=Pp3c14_8120V3.1 locus=Pp3c14_8120 annot-version=v3.3	Physcomitrella_patens OG0000041
Pp3c1_17909V3.1.p pacid=32968259 transcript=Pp3c1_17909V3.1 locus=Pp3c1_17909 annot-version=v3.3	Physcomitrella_patens OG0000041
Pp3c1_17930V3.1.p pacid=32970763 transcript=Pp3c1_17930V3.1 locus=Pp3c1_17930 annot-version=v3.3	Physcomitrella_patens OG0000041
Pp3c6_19080V3.1.p pacid=32976390 transcript=Pp3c6_19080V3.1 locus=Pp3c6_19080 annot-version=v3.3	Physcomitrella_patens OG0000041
Pp3c6_19100V3.1.p pacid=32977602 transcript=Pp3c6_19100V3.1 locus=Pp3c6_19100 annot-version=v3.3	Physcomitrella_patens OG0000041
Pp3c6_19090V3.1.p pacid=32978619 transcript=Pp3c6_19090V3.1 locus=Pp3c6_19090 annot-version=v3.3	Physcomitrella_patens OG0000041
Pp3c6_19077V3.1.p pacid=32978736 transcript=Pp3c6_19077V3.1 locus=Pp3c6_19077 annot-version=v3.3	Physcomitrella_patens OG0000041
Pp3c26_15380V3.1.p pacid=32917945 transcript=Pp3c26_15380V3.1 locus=Pp3c26_15380 annot-version=v3.3	Physcomitrella_patens OG0000041
Pp3c10_15970V3.1.p pacid=32901500 transcript=Pp3c10_15970V3.1 locus=Pp3c10_15970 annot-version=v3.3	Physcomitrella_patens OG0000041
Pp3c10_21680V3.1.p pacid=32901923 transcript=Pp3c10_21680V3.1 locus=Pp3c10_21680 annot-version=v3.3	Physcomitrella_patens OG0000041
AT3G04210.1 | Disease resistance protein (TIR-NBS class) | Chr3:1106243-1108005 REVERSE LENGTH=531 | 201606 Arabidopsis_thaliana	OG0000041
AT3G04220.1 | Disease resistance protein (TIR-NBS-LRR class) family | Chr3:1109118-1112188 REVERSE LENGTH=867 | 201606 Arabidopsis_thaliana	OG0000041

AT3G25505.1 | TIR-NBS-LRR class disease resistance protein |
Chr3:9260838-9263171 REVERSE LENGTH=702 | 201606
Arabidopsis_thaliana   OG0000041
AT3G25510.1 | disease resistance protein (TIR-NBS-LRR class) family
protein | Chr3:9264388-9267932 REVERSE LENGTH=1325 | 201606
Arabidopsis_thaliana   OG0000041
AT3G44400.1 | Disease resistance protein (TIR-NBS-LRR class) family
| Chr3:16046331-16049668 REVERSE LENGTH=1007 | 201606
Arabidopsis_thaliana   OG0000041
AT3G44480.1 | Disease resistance protein (TIR-NBS-LRR class) family
| Chr3:16090878-16096041 REVERSE LENGTH=1194 | 201606
Arabidopsis_thaliana   OG0000041
AT3G44630.1 | Disease resistance protein (TIR-NBS-LRR class) family
| Chr3:16196292-16200423 FORWARD LENGTH=1214 | 201606
Arabidopsis_thaliana   OG0000041
AT3G44670.1 | Disease resistance protein (TIR-NBS-LRR class) family
| Chr3:16217242-16221425 FORWARD LENGTH=1219 | 201606
Arabidopsis_thaliana   OG0000041
AT3G51560.1 | Disease resistance protein (TIR-NBS-LRR class) family
| Chr3:19121808-19125913 REVERSE LENGTH=1253 | 201606
Arabidopsis_thaliana   OG0000041
AT3G51570.1 | Disease resistance protein (TIR-NBS-LRR class) family
| Chr3:19126358-19130456 FORWARD LENGTH=1226 | 201606
Arabidopsis_thaliana   OG0000041
AT4G04110.1 | Toll-Interleukin-Resistance (TIR) domain family
protein | Chr4:1972591-1974014 FORWARD LENGTH=239 | 201606
Arabidopsis_thaliana   OG0000041
AT4G08450.1 | Disease resistance protein (TIR-NBS-LRR class) family
| Chr4:5365610-5371101 FORWARD LENGTH=1234 | 201606
Arabidopsis_thaliana   OG0000041
AT4G09360.1 | NB-ARC domain-containing disease resistance protein |
Chr4:5940186-5943280 FORWARD LENGTH=853 | 201606
Arabidopsis_thaliana   OG0000041
AT4G09420.1 | Disease resistance protein (TIR-NBS class) |
Chr4:5962319-5963776 REVERSE LENGTH=457 | 201606
Arabidopsis_thaliana   OG0000041
AT4G09430.1 | disease resistance protein (TIR-NBS-LRR class) |
Chr4:5970932-5973113 FORWARD LENGTH=555 | 201606
Arabidopsis_thaliana   OG0000041
AT1G17600.1 | Disease resistance protein (TIR-NBS-LRR class) family
| Chr1:6053026-6056572 REVERSE LENGTH=1049 | 201606
Arabidopsis_thaliana   OG0000041
AT1G17610.1 | Disease resistance protein (TIR-NBS class) |
Chr1:6056895-6058157 FORWARD LENGTH=420 | 201606
Arabidopsis_thaliana   OG0000041
AT1G17615.1 | Disease resistance protein (TIR-NBS class) |
Chr1:6059441-6060667 FORWARD LENGTH=380 | 201606
Arabidopsis_thaliana   OG0000041
AT4G11170.1 | Disease resistance protein (TIR-NBS-LRR class) family
| Chr4:6811127-6817130 FORWARD LENGTH=1095 | 201606
Arabidopsis_thaliana   OG0000041
AT4G11340.1 | Disease resistance protein (TIR-NBS-LRR class) family
| Chr4:6894208-6899130 REVERSE LENGTH=495 | 201606
Arabidopsis_thaliana   OG0000041

AT4G12010.1 | Disease resistance protein (TIR-NBS-LRR class) family | Chr4:7197325-7201393 REVERSE LENGTH=1219 | 201606 Arabidopsis_thaliana OG0000041
AT4G14370.1 | Disease resistance protein (TIR-NBS-LRR class) family | Chr4:8279946-8283263 REVERSE LENGTH=1008 | 201606 Arabidopsis_thaliana OG0000041
AT4G16860.1 | Disease resistance protein (TIR-NBS-LRR class) family | Chr4:9488584-9495700 REVERSE LENGTH=1147 | 201606 Arabidopsis_thaliana OG0000041
AT4G16880.1 | Leucine-rich repeat (LRR) family protein | Chr4:9496550-9497149 REVERSE LENGTH=153 | 201606 Arabidopsis_thaliana OG0000041
AT4G16890.1 | TIR-NBS-LRR class disease resistance protein | Chr4:9500506-9505482 REVERSE LENGTH=1437 | 201606 Arabidopsis_thaliana OG0000041
AT4G16900.1 | Disease resistance protein (TIR-NBS-LRR class) family | Chr4:9512329-9516541 REVERSE LENGTH=1040 | 201606 Arabidopsis_thaliana OG0000041
AT4G16920.1 | Disease resistance protein (TIR-NBS-LRR class) family | Chr4:9519173-9525691 REVERSE LENGTH=1304 | 201606 Arabidopsis_thaliana OG0000041
AT4G16930.1 | Toll-Interleukin-Resistance (TIR) domain-containing protein | Chr4:9529564-9530115 REVERSE LENGTH=154 | 201606 Arabidopsis_thaliana OG0000041
AT4G16940.1 | Disease resistance protein (TIR-NBS-LRR class) family | Chr4:9533149-9537510 REVERSE LENGTH=1147 | 201606 Arabidopsis_thaliana OG0000041
AT4G16950.1 | Disease resistance protein (TIR-NBS-LRR class) family | Chr4:9539010-9544340 REVERSE LENGTH=1449 | 201606 Arabidopsis_thaliana OG0000041
AT4G16960.1 | disease resistance protein (TIR-NBS-LRR class) | Chr4:9547429-9551007 REVERSE LENGTH=1001 | 201606 Arabidopsis_thaliana OG0000041
AT4G16990.1 | disease resistance protein (TIR-NBS class) | Chr4:9560155-9564616 FORWARD LENGTH=670 | 201606 Arabidopsis_thaliana OG0000041
AT4G19500.1 | nucleoside-triphosphatase/transmembrane receptor/nucleotide binding/ATP binding protein | Chr4:10625787-10630140 FORWARD LENGTH=1309 | 201606 Arabidopsis_thaliana OG0000041
AT4G19510.1 | Disease resistance protein (TIR-NBS-LRR class) | Chr4:10633685-10637841 FORWARD LENGTH=1210 | 201606 Arabidopsis_thaliana OG0000041
AT4G19520.1 | disease resistance protein (TIR-NBS-LRR class) family | Chr4:10639488-10647070 REVERSE LENGTH=1744 | 201606 Arabidopsis_thaliana OG0000041
AT4G19530.1 | disease resistance protein (TIR-NBS-LRR class) family | Chr4:10651962-10657090 FORWARD LENGTH=1167 | 201606 Arabidopsis_thaliana OG0000041
AT4G19910.1 | Toll-Interleukin-Resistance (TIR) domain family protein | Chr4:10795869-10796687 REVERSE LENGTH=241 | 201606 Arabidopsis_thaliana OG0000041
AT4G19920.1 | Toll-Interleukin-Resistance (TIR) domain family protein | Chr4:10797392-10798360 REVERSE LENGTH=274 | 201606

Arabidopsis_thaliana   OG0000041
AT4G19925.1 | Toll-Interleukin-Resistance (TIR) domain family protein | Chr4:10799875-10800726 REVERSE LENGTH=249 | 201606
Arabidopsis_thaliana   OG0000041
AT4G23510.1 | Disease resistance protein (TIR-NBS-LRR class) family | Chr4:12267516-12270526 REVERSE LENGTH=635 | 201606
Arabidopsis_thaliana   OG0000041
AT4G23515.1 | Toll-Interleukin-Resistance (TIR) domain family protein | Chr4:12271521-12272842 REVERSE LENGTH=317 | 201606
Arabidopsis_thaliana   OG0000041
AT4G36140.1 | disease resistance protein (TIR-NBS-LRR class) | Chr4:17098956-17104479 REVERSE LENGTH=1607 | 201606
Arabidopsis_thaliana   OG0000041
AT4G36150.1 | Disease resistance protein (TIR-NBS-LRR class) family | Chr4:17104776-17108711 FORWARD LENGTH=1179 | 201606
Arabidopsis_thaliana   OG0000041
AT5G11250.1 | Disease resistance protein (TIR-NBS-LRR class) | Chr5:3587978-3591714 REVERSE LENGTH=1107 | 201606
Arabidopsis_thaliana   OG0000041
AT5G17680.1 | disease resistance protein (TIR-NBS-LRR class) | Chr5:5822999-5827153 FORWARD LENGTH=1294 | 201606
Arabidopsis_thaliana   OG0000041
AT5G17880.1 | disease resistance protein (TIR-NBS-LRR class) | Chr5:5908874-5913096 REVERSE LENGTH=1197 | 201606
Arabidopsis_thaliana   OG0000041
AT5G17970.1 | Disease resistance protein (TIR-NBS-LRR class) family | Chr5:5948999-5951619 REVERSE LENGTH=780 | 201606
Arabidopsis_thaliana   OG0000041
AT5G18350.1 | Disease resistance protein (TIR-NBS-LRR class) family | Chr5:6074069-6078569 REVERSE LENGTH=1245 | 201606
Arabidopsis_thaliana   OG0000041
AT5G18360.1 | Disease resistance protein (TIR-NBS-LRR class) family | Chr5:6080049-6083027 REVERSE LENGTH=900 | 201606
Arabidopsis_thaliana   OG0000041
AT5G18370.1 | disease resistance protein (TIR-NBS-LRR class) family protein | Chr5:6085036-6088926 REVERSE LENGTH=1210 | 201606
Arabidopsis_thaliana   OG0000041
AT5G22690.1 | Disease resistance protein (TIR-NBS-LRR class) family | Chr5:7541369-7544888 FORWARD LENGTH=1008 | 201606
Arabidopsis_thaliana   OG0000041
AT5G36930.1 | Disease resistance protein (TIR-NBS-LRR class) family | Chr5:14567771-14571907 REVERSE LENGTH=1188 | 201606
Arabidopsis_thaliana   OG0000041
AT5G38340.1 | Disease resistance protein (TIR-NBS-LRR class) family | Chr5:15320507-15324061 FORWARD LENGTH=1059 | 201606
Arabidopsis_thaliana   OG0000041
AT5G38344.1 | Toll-Interleukin-Resistance (TIR) domain family protein | Chr5:15325950-15326591 FORWARD LENGTH=213 | 201606
Arabidopsis_thaliana   OG0000041
AT5G38350.1 | Disease resistance protein (NBS-LRR class) family | Chr5:15328659-15331528 FORWARD LENGTH=833 | 201606
Arabidopsis_thaliana   OG0000041
AT5G38850.1 | Disease resistance protein (TIR-NBS-LRR class) | Chr5:15555187-15558430 FORWARD LENGTH=986 | 201606

Arabidopsis_thaliana   OG0000041
AT5G40060.1 | Disease resistance protein (NBS-LRR class) family | Chr5:16035246-16038730 FORWARD LENGTH=968 | 201606
Arabidopsis_thaliana   OG0000041
AT5G40090.1 | Disease resistance protein (TIR-NBS class) | Chr5:16042115-16043494 REVERSE LENGTH=459 | 201606
Arabidopsis_thaliana   OG0000041
AT5G40100.1 | Disease resistance protein (TIR-NBS-LRR class) family | Chr5:16043976-16047355 FORWARD LENGTH=1017 | 201606
Arabidopsis_thaliana   OG0000041
AT5G40910.1 | Disease resistance protein (TIR-NBS-LRR class) family | Chr5:16395507-16399129 FORWARD LENGTH=1104 | 201606
Arabidopsis_thaliana   OG0000041
AT5G41540.1 | Disease resistance protein (TIR-NBS-LRR class) family | Chr5:16612659-16616063 REVERSE LENGTH=1038 | 201606
Arabidopsis_thaliana   OG0000041
AT5G41550.1 | Disease resistance protein (TIR-NBS-LRR class) family | Chr5:16617232-16620785 REVERSE LENGTH=1085 | 201606
Arabidopsis_thaliana   OG0000041
AT5G41740.1 | Disease resistance protein (TIR-NBS-LRR class) family | Chr5:16688687-16692235 FORWARD LENGTH=1046 | 201606
Arabidopsis_thaliana   OG0000041
AT5G41750.1 | Disease resistance protein (TIR-NBS-LRR class) family | Chr5:16694047-16697527 FORWARD LENGTH=1068 | 201606
Arabidopsis_thaliana   OG0000041
AT5G44510.1 | target of AVRB operation1 | Chr5:17929673-17934188 REVERSE LENGTH=1187 | 201606   Arabidopsis_thaliana
OG0000041
AT5G44870.1 | Disease resistance protein (TIR-NBS-LRR class) family | Chr5:18114666-18118608 FORWARD LENGTH=1170 | 201606
Arabidopsis_thaliana   OG0000041
AT5G44900.1 | Toll-Interleukin-Resistance (TIR) domain family protein | Chr5:18135495-18136383 FORWARD LENGTH=252 | 201606
Arabidopsis_thaliana   OG0000041
AT5G44910.1 | Toll-Interleukin-Resistance (TIR) domain family protein | Chr5:18137354-18138235 FORWARD LENGTH=241 | 201606
Arabidopsis_thaliana   OG0000041
AT5G44920.1 | Toll-Interleukin-Resistance (TIR) domain family protein | Chr5:18138965-18139994 FORWARD LENGTH=255 | 201606
Arabidopsis_thaliana   OG0000041
AT5G45000.1 | Disease resistance protein (TIR-NBS-LRR class) family | Chr5:18165427-18167005 FORWARD LENGTH=419 | 201606
Arabidopsis_thaliana   OG0000041
AT5G45050.1 | Disease resistance protein (TIR-NBS-LRR class) | Chr5:18177016-18181805 REVERSE LENGTH=1372 | 201606
Arabidopsis_thaliana   OG0000041
AT5G45060.1 | Disease resistance protein (TIR-NBS-LRR class) family | Chr5:18182038-18186067 FORWARD LENGTH=1165 | 201606
Arabidopsis_thaliana   OG0000041
AT5G45070.1 | phloem protein 2-A8 | Chr5:18187899-18189362 REVERSE LENGTH=354 | 201606   Arabidopsis_thaliana   OG0000041
AT5G45080.1 | phloem protein 2-A6 | Chr5:18191575-18193207 REVERSE LENGTH=392 | 201606   Arabidopsis_thaliana   OG0000041
AT5G45090.1 | phloem protein 2-A7 | Chr5:18202753-18205000 REVERSE

LENGTH=332 | 201606 Arabidopsis_thaliana OG0000041
AT5G45200.1 | Disease resistance protein (TIR-NBS-LRR class) family | Chr5:18283967-18290332 REVERSE LENGTH=1261 | 201606 Arabidopsis_thaliana OG0000041
AT5G45210.1 | Disease resistance protein (TIR-NBS-LRR class) family | Chr5:18295521-18298434 FORWARD LENGTH=697 | 201606 Arabidopsis_thaliana OG0000041
AT5G45220.1 | Disease resistance protein (TIR-NBS-LRR class) family | Chr5:18298926-18301069 REVERSE LENGTH=546 | 201606 Arabidopsis_thaliana OG0000041
AT5G45230.1 | Disease resistance protein (TIR-NBS-LRR class) family | Chr5:18302147-18308303 REVERSE LENGTH=1231 | 201606 Arabidopsis_thaliana OG0000041
AT5G45240.1 | Disease resistance protein (TIR-NBS-LRR class) | Chr5:18313706-18319089 FORWARD LENGTH=812 | 201606 Arabidopsis_thaliana OG0000041
AT5G45250.1 | Disease resistance protein (TIR-NBS-LRR class) family | Chr5:18321914-18326022 REVERSE LENGTH=1217 | 201606 Arabidopsis_thaliana OG0000041
AT5G45260.1 | Disease resistance protein (TIR-NBS-LRR class) | Chr5:18326277-18332229 FORWARD LENGTH=1288 | 201606 Arabidopsis_thaliana OG0000041
AT5G46260.1 | disease resistance protein (TIR-NBS-LRR class) family | Chr5:18759102-18763358 REVERSE LENGTH=1205 | 201606 Arabidopsis_thaliana OG0000041
AT5G46270.1 | Disease resistance protein (TIR-NBS-LRR class) family | Chr5:18764833-18769090 REVERSE LENGTH=1139 | 201606 Arabidopsis_thaliana OG0000041
AT5G46450.1 | Disease resistance protein (TIR-NBS-LRR class) family | Chr5:18835618-18839546 FORWARD LENGTH=1123 | 201606 Arabidopsis_thaliana OG0000041
AT5G46470.1 | disease resistance protein (TIR-NBS-LRR class) family | Chr5:18842701-18846809 FORWARD LENGTH=1127 | 201606 Arabidopsis_thaliana OG0000041
AT5G46490.1 | Disease resistance protein (TIR-NBS-LRR class) family | Chr5:18850848-18852418 FORWARD LENGTH=357 | 201606 Arabidopsis_thaliana OG0000041
AT5G46500.1 | protein VARIATION IN COMPOUND TRIGGERED ROOT growth protein | Chr5:18856454-18857787 FORWARD LENGTH=417 | 201606 Arabidopsis_thaliana OG0000041
AT5G46510.1 | Disease resistance protein (TIR-NBS-LRR class) family | Chr5:18860451-18865210 FORWARD LENGTH=1353 | 201606 Arabidopsis_thaliana OG0000041
AT5G46520.1 | Disease resistance protein (TIR-NBS-LRR class) family | Chr5:18867840-18871976 FORWARD LENGTH=1168 | 201606 Arabidopsis_thaliana OG0000041
AT5G48770.1 | Disease resistance protein (TIR-NBS-LRR class) family | Chr5:19773277-19777242 REVERSE LENGTH=1190 | 201606 Arabidopsis_thaliana OG0000041
AT5G48780.1 | disease resistance protein (TIR-NBS class) | Chr5:19777511-19779632 FORWARD LENGTH=639 | 201606 Arabidopsis_thaliana OG0000041
AT5G49140.1 | Disease resistance protein (TIR-NBS-LRR class) family | Chr5:19919085-19923415 REVERSE LENGTH=980 | 201606

Arabidopsis_thaliana   OG0000041
AT5G51630.1 | Disease resistance protein (TIR-NBS-LRR class) family | Chr5:20970069-20974666 FORWARD LENGTH=1229 | 201606
Arabidopsis_thaliana   OG0000041
AT5G58120.1 | Disease resistance protein (TIR-NBS-LRR class) family | Chr5:23517492-23520927 FORWARD LENGTH=1046 | 201606
Arabidopsis_thaliana   OG0000041
AT1G27170.1 | transmembrane receptors / ATP binding protein | Chr1:9434718-9439219 FORWARD LENGTH=1384 | 201606
Arabidopsis_thaliana   OG0000041
AT1G27180.1 | disease resistance protein (TIR-NBS-LRR class) | Chr1:9439859-9445818 FORWARD LENGTH=1556 | 201606
Arabidopsis_thaliana   OG0000041
AT1G31540.1 | Disease resistance protein (TIR-NBS-LRR class) family | Chr1:11291051-11293697 REVERSE LENGTH=776 | 201606
Arabidopsis_thaliana   OG0000041
AT1G47370.1 | Toll-Interleukin-Resistance (TIR) domain family protein | Chr1:17365898-17368703 REVERSE LENGTH=363 | 201606
Arabidopsis_thaliana   OG0000041
AT1G52900.1 | Toll-Interleukin-Resistance (TIR) domain family protein | Chr1:19702211-19702916 REVERSE LENGTH=199 | 201606
Arabidopsis_thaliana   OG0000041
AT1G56510.1 | Disease resistance protein (TIR-NBS-LRR class) | Chr1:21167704-21172260 FORWARD LENGTH=1007 | 201606
Arabidopsis_thaliana   OG0000041
AT1G56520.1 | Disease resistance protein (TIR-NBS-LRR class) family | Chr1:21175614-21178920 REVERSE LENGTH=897 | 201606
Arabidopsis_thaliana   OG0000041
AT1G56540.1 | Disease resistance protein (TIR-NBS-LRR class) family | Chr1:21181664-21185306 FORWARD LENGTH=1096 | 201606
Arabidopsis_thaliana   OG0000041
AT1G57630.1 | Toll-Interleukin-Resistance (TIR) domain family protein | Chr1:21345639-21346157 FORWARD LENGTH=172 | 201606
Arabidopsis_thaliana   OG0000041
AT1G57650.1 | ATP binding protein | Chr1:21351291-21354311 FORWARD LENGTH=709 | 201606   Arabidopsis_thaliana   OG0000041
AT1G57670.1 | Toll-Interleukin-Resistance (TIR) domain family protein | Chr1:21357169-21359872 REVERSE LENGTH=387 | 201606
Arabidopsis_thaliana   OG0000041
AT1G57830.1 | Toll-Interleukin-Resistance (TIR) domain family protein | Chr1:21420297-21420803 FORWARD LENGTH=168 | 201606
Arabidopsis_thaliana   OG0000041
AT1G57850.1 | Toll-Interleukin-Resistance (TIR) domain family protein | Chr1:21426690-21427993 FORWARD LENGTH=192 | 201606
Arabidopsis_thaliana   OG0000041
AT1G60320.1 | Toll-Interleukin-Resistance (TIR) domain family protein | Chr1:22235091-22235606 FORWARD LENGTH=171 | 201606
Arabidopsis_thaliana   OG0000041
AT1G61105.1 | Toll-Interleukin-Resistance (TIR) domain family protein | Chr1:22513616-22514492 REVERSE LENGTH=188 | 201606
Arabidopsis_thaliana   OG0000041
AT1G63730.1 | Disease resistance protein (TIR-NBS-LRR class) family | Chr1:23641770-23645132 FORWARD LENGTH=966 | 201606
Arabidopsis_thaliana   OG0000041

AT1G63740.1 | Disease resistance protein (TIR-NBS-LRR class) family | Chr1:23645525-23648807 FORWARD LENGTH=992 | 201606 Arabidopsis_thaliana OG0000041
AT1G63750.1 | Disease resistance protein (TIR-NBS-LRR class) family | Chr1:23652263-23655333 FORWARD LENGTH=964 | 201606 Arabidopsis_thaliana OG0000041
AT1G63860.1 | Disease resistance protein (TIR-NBS-LRR class) family | Chr1:23701920-23706005 REVERSE LENGTH=1004 | 201606 Arabidopsis_thaliana OG0000041
AT1G63870.1 | Disease resistance protein (TIR-NBS-LRR class) family | Chr1:23707131-23711901 REVERSE LENGTH=1031 | 201606 Arabidopsis_thaliana OG0000041
AT1G63880.1 | Disease resistance protein (TIR-NBS-LRR class) family | Chr1:23712514-23716047 REVERSE LENGTH=1017 | 201606 Arabidopsis_thaliana OG0000041
AT1G64070.1 | Disease resistance protein (TIR-NBS-LRR class) family | Chr1:23779949-23783449 FORWARD LENGTH=997 | 201606 Arabidopsis_thaliana OG0000041
AT1G65390.1 | phloem protein 2 A5 | Chr1:24292566-24294566 FORWARD LENGTH=411 | 201606 Arabidopsis_thaliana OG0000041
AT1G65850.1 | Disease resistance protein (TIR-NBS-LRR class) family | Chr1:24494734-24498485 FORWARD LENGTH=1036 | 201606 Arabidopsis_thaliana OG0000041
AT1G66090.1 | Disease resistance protein (TIR-NBS class) | Chr1:24602221-24604573 FORWARD LENGTH=429 | 201606 Arabidopsis_thaliana OG0000041
AT1G69550.1 | disease resistance protein (TIR-NBS-LRR class) | Chr1:26148836-26153374 REVERSE LENGTH=1400 | 201606 Arabidopsis_thaliana OG0000041
AT1G72840.1 | Disease resistance protein (TIR-NBS-LRR class) | Chr1:27410020-27413485 REVERSE LENGTH=1042 | 201606 Arabidopsis_thaliana OG0000041
AT1G72850.1 | Disease resistance protein (TIR-NBS class) | Chr1:27413971-27415335 FORWARD LENGTH=422 | 201606 Arabidopsis_thaliana OG0000041
AT1G72860.1 | Disease resistance protein (TIR-NBS-LRR class) family | Chr1:27417096-27420778 REVERSE LENGTH=1163 | 201606 Arabidopsis_thaliana OG0000041
AT1G72870.1 | Disease resistance protein (TIR-NBS class) | Chr1:27421086-27422999 FORWARD LENGTH=512 | 201606 Arabidopsis_thaliana OG0000041
AT1G72890.1 | Disease resistance protein (TIR-NBS class) | Chr1:27429947-27431717 FORWARD LENGTH=438 | 201606 Arabidopsis_thaliana OG0000041
AT1G72900.1 | Toll-Interleukin-Resistance (TIR) domain-containing protein | Chr1:27432216-27433532 FORWARD LENGTH=363 | 201606 Arabidopsis_thaliana OG0000041
AT1G72910.1 | Toll-Interleukin-Resistance (TIR) domain-containing protein | Chr1:27435634-27436887 FORWARD LENGTH=380 | 201606 Arabidopsis_thaliana OG0000041
AT1G72920.1 | Toll-Interleukin-Resistance (TIR) domain family protein | Chr1:27437947-27438868 FORWARD LENGTH=275 | 201606 Arabidopsis_thaliana OG0000041
AT1G72930.1 | toll/interleukin-1 receptor-like protein |

Chr1:27439476-27440147 FORWARD LENGTH=176 | 201606
Arabidopsis_thaliana OG0000041
AT1G72940.1 | Toll-Interleukin-Resistance (TIR) domain-containing protein | Chr1:27442278-27443487 FORWARD LENGTH=371 | 201606
Arabidopsis_thaliana OG0000041
AT1G72950.1 | Disease resistance protein (TIR-NBS class) | Chr1:27444585-27445814 FORWARD LENGTH=379 | 201606
Arabidopsis_thaliana OG0000041
AT2G03030.1 | Toll-Interleukin-Resistance (TIR) domain family protein | Chr2:889764-890520 FORWARD LENGTH=204 | 201606
Arabidopsis_thaliana OG0000041
AT2G03300.1 | Toll-Interleukin-Resistance (TIR) domain family protein | Chr2:1003468-1004222 REVERSE LENGTH=203 | 201606
Arabidopsis_thaliana OG0000041
AT2G14080.1 | Disease resistance protein (TIR-NBS-LRR class) family | Chr2:5925225-5929600 FORWARD LENGTH=1215 | 201606
Arabidopsis_thaliana OG0000041
AT2G16870.1 | Disease resistance protein (TIR-NBS-LRR class) family | Chr2:7308077-7311686 REVERSE LENGTH=1109 | 201606
Arabidopsis_thaliana OG0000041
AT2G17050.1 | disease resistance protein (TIR-NBS-LRR class) | Chr2:7410835-7415610 REVERSE LENGTH=1355 | 201606
Arabidopsis_thaliana OG0000041
AT2G17060.1 | Disease resistance protein (TIR-NBS-LRR class) family | Chr2:7422411-7426877 FORWARD LENGTH=1195 | 201606
Arabidopsis_thaliana OG0000041
AT2G20142.1 | Toll-Interleukin-Resistance (TIR) domain family protein | Chr2:8695387-8696566 FORWARD LENGTH=313 | 201606
Arabidopsis_thaliana OG0000041
AT1G09665.1 | Toll-Interleukin-Resistance (TIR) domain family protein | Chr1:3133095-3133674 FORWARD LENGTH=165 | 201606
Arabidopsis_thaliana OG0000041
AT2G32140.1 | transmembrane receptor | Chr2:13655714-13657033 REVERSE LENGTH=353 | 201606 Arabidopsis_thaliana
OG0000041
Os07t0566800-01 Toll-Interleukin receptor domain containing protein. Oryza_sativa OG0000041
CML224CT pep chromosome:ASM9120v1:12:562823:563678:-1 gene:CML224C transcript:CML224CT gene_biotype:protein_coding transcript_biotype:protein_coding Cyanidioschyzon_merolae
OG0000041
CDF36337 pep supercontig:ASM35022v2:HG001773:60768:62715:-1 gene:CHC_T00008334001 transcript:CDF36337 gene_biotype:protein_coding transcript_biotype:protein_coding description:TIR domain-containing protein Chondrus_crispus
OG0000041
CDF36338 pep supercontig:ASM35022v2:HG001773:64176:65864:1 gene:CHC_T00009256001 transcript:CDF36338 gene_biotype:protein_coding transcript_biotype:protein_coding description:TIR domain-containing protein Chondrus_crispus
OG0000041
CDF38850 pep supercontig:ASM35022v2:HG001973:54922:58481:-1 gene:CHC_T00009302001 transcript:CDF38850 gene_biotype:protein_coding transcript_biotype:protein_coding

description:WD40-repeat containing protein Chondrus_crispus
OG0000041
CDF39472 pep supercontig:ASM35022v2:HG002042:38097:43401:1
gene:CHC_T00009561001 transcript:CDF39472
gene_biotype:protein_coding transcript_biotype:protein_coding
description:WD40-repeat containing protein Chondrus_crispus
OG0000041
CDF37690 pep supercontig:ASM35022v2:HG001861:17792:21868:-1
gene:CHC_T00009405001 transcript:CDF37690
gene_biotype:protein_coding transcript_biotype:protein_coding
description:TIR domain-containing protein GTPase Chondrus_crispus
OG0000041
CDF40092 pep supercontig:ASM35022v2:HG002126:26406:30898:1
gene:CHC_T00008269001 transcript:CDF40092
gene_biotype:protein_coding transcript_biotype:protein_coding
description:TIR domain-containing protein Chondrus_crispus
OG0000041
CDF40096 pep supercontig:ASM35022v2:HG002126:91494:95561:1
gene:CHC_T00009572001 transcript:CDF40096
gene_biotype:protein_coding transcript_biotype:protein_coding
description:NB-ARC protein Chondrus_crispus OG0000041
CDF39398 pep supercontig:ASM35022v2:HG002030:408:3058:-1
gene:CHC_T00009069001 transcript:CDF39398
gene_biotype:protein_coding transcript_biotype:protein_coding
description:TIR domain-containing protein GTPase Chondrus_crispus
OG0000041
CDF32725 pep supercontig:ASM35022v2:HG001553:148272:150755:-1
gene:CHC_T00008965001 transcript:CDF32725
gene_biotype:protein_coding transcript_biotype:protein_coding
description:NB-ARC and TPR repeat-containing protein
Chondrus_crispus OG0000041
CDF37505 pep supercontig:ASM35022v2:HG001845:32034:37359:-1
gene:CHC_T00009310001 transcript:CDF37505
gene_biotype:protein_coding transcript_biotype:protein_coding
description:WD40-repeat containing protein Chondrus_crispus
OG0000041
CDF40019 pep supercontig:ASM35022v2:HG002106:28848:32921:1
gene:CHC_T00008786001 transcript:CDF40019
gene_biotype:protein_coding transcript_biotype:protein_coding
description:WD40-repeat containing protein Chondrus_crispus
OG0000041
CDF33637 pep supercontig:ASM35022v2:HG001652:109328:113211:1
gene:CHC_T00009272001 transcript:CDF33637
gene_biotype:protein_coding transcript_biotype:protein_coding
description:Sel1-repeat containing protein Chondrus_crispus
OG0000041
CDF40243 pep supercontig:ASM35022v2:HG002147:239825:241947:1
gene:CHC_T00008460001 transcript:CDF40243
gene_biotype:protein_coding transcript_biotype:protein_coding
description:TIR domain-containing protein Chondrus_crispus
OG0000041
g2140.t1 Caulerpa_lentillifera OG0000042
g5451.t1 Caulerpa_lentillifera OG0000042
g6309.t1 Caulerpa_lentillifera OG0000042

g7954.t1 Caulerpa_lentillifera OG0000042
g8770.t1 Caulerpa_lentillifera OG0000042
Cre03.g182700.t1.1 pacid=30787016 transcript=Cre03.g182700.t1.1 locus=Cre03.g182700 ID=Cre03.g182700.t1.1.v5.5 annot-version=v5.5 Chlamydomonas_reinhardtii OG0000042
Cre12.g534100.t1.1 pacid=30792413 transcript=Cre12.g534100.t1.1 locus=Cre12.g534100 ID=Cre12.g534100.t1.1.v5.5 annot-version=v5.5 Chlamydomonas_reinhardtii OG0000042
Cre16.g675100.t1.1 pacid=30777301 transcript=Cre16.g675100.t1.1 locus=Cre16.g675100 ID=Cre16.g675100.t1.1.v5.5 annot-version=v5.5 Chlamydomonas_reinhardtii OG0000042
Cre06.g278200.t1.1 pacid=30779660 transcript=Cre06.g278200.t1.1 locus=Cre06.g278200 ID=Cre06.g278200.t1.1.v5.5 annot-version=v5.5 Chlamydomonas_reinhardtii OG0000042
Cre06.g278159.t1.1 pacid=30779842 transcript=Cre06.g278159.t1.1 locus=Cre06.g278159 ID=Cre06.g278159.t1.1.v5.5 annot-version=v5.5 Chlamydomonas_reinhardtii OG0000042
Cre15.g635100.t1.2 pacid=30783724 transcript=Cre15.g635100.t1.2 locus=Cre15.g635100 ID=Cre15.g635100.t1.2.v5.5 annot-version=v5.5 Chlamydomonas_reinhardtii OG0000042
Vocar.0071s0005.1.p pacid=32896166 transcript=Vocar.0071s0005.1 locus=Vocar.0071s0005 ID=Vocar.0071s0005.1.v2.1 annot-version=v2.1 Volvox_carteri OG0000042
Vocar.0015s0269.1.p pacid=32899222 transcript=Vocar.0015s0269.1 locus=Vocar.0015s0269 ID=Vocar.0015s0269.1.v2.1 annot-version=v2.1 Volvox_carteri OG0000042
Vocar.0002s0569.1.p pacid=32891019 transcript=Vocar.0002s0569.1 locus=Vocar.0002s0569 ID=Vocar.0002s0569.1.v2.1 annot-version=v2.1 Volvox_carteri OG0000042
Vocar.0002s0120.1.p pacid=32891264 transcript=Vocar.0002s0120.1 locus=Vocar.0002s0120 ID=Vocar.0002s0120.1.v2.1 annot-version=v2.1 Volvox_carteri OG0000042
jgi|ChlNC64A_1|133921|IGS.gm_10_00212 Chlorella_variabilis OG0000042
jgi|ChlNC64A_1|133653|IGS.gm_1_00789 Chlorella_variabilis OG0000042
jgi|ChlNC64A_1|136706|IGS.gm_17_00110 Chlorella_variabilis OG0000042
55541 pacid=27388991 transcript=55541 locus=Genemark1.485_g ID=55541.2.0.227 annot-version=v2.0 Coccomyxa_subellipsoidea OG0000042
64395 pacid=27389448 transcript=64395 locus=estExt_Genemark1.C_10729 ID=64395.2.0.227 annot-version=v2.0 Coccomyxa_subellipsoidea OG0000042
64367 pacid=27389488 transcript=64367 locus=estExt_Genemark1.C_10682 ID=64367.2.0.227 annot-version=v2.0 Coccomyxa_subellipsoidea OG0000042
58803 pacid=27393336 transcript=58803 locus=Genemark1.765_g ID=58803.2.0.227 annot-version=v2.0 Coccomyxa_subellipsoidea OG0000042
23893_estExt_fgenesh1_pg.C_Chr_07.00010056 Ostreococcus_tauri OG0000042
24014_estExt_fgenesh1_pg.C_Chr_08.00010175 Ostreococcus_tauri OG0000042

28099_estExt_gwp_GeneWisePlus.C_Chr_04.00010305
Ostreococcus_tauri OG0000042
34685_0900010151 Ostreococcus_tauri OG0000042
24762 pacid=27414124 transcript=24762
locus=estExt_fgenesh1_pg.C_Chr_70071 ID=24762.2.0.231 annot-version=v2.0 Ostreococcus_lucimarinus OG0000042
37312 pacid=27415658 transcript=37312 locus=e_gwEuk.4.304.1
ID=37312.2.0.231 annot-version=v2.0 Ostreococcus_lucimarinus
OG0000042
33113 pacid=27419282 transcript=33113 locus=eugene.0800010239
ID=33113.2.0.231 annot-version=v2.0 Ostreococcus_lucimarinus
OG0000042
33434 pacid=27413796 transcript=33434 locus=eugene.0900010138
ID=33434.2.0.231 annot-version=v2.0 Ostreococcus_lucimarinus
OG0000042
195113 pacid=27349116 transcript=195153
locus=estExt_Genemark1.C_120015 ID=195153.3.0.228 annot-version=v3.0
Micromonas_pusilla OG0000042
58157 pacid=27341907 transcript=58157 locus=MicpuC2.EuGene.
0000050462 ID=58157.3.0.228 annot-version=v3.0
Micromonas_pusilla OG0000042
56149 pacid=27347595 transcript=56149 locus=MicpuC2.EuGene.
0000030303 ID=56149.3.0.228 annot-version=v3.0
Micromonas_pusilla OG0000042
59091 pacid=27348332 transcript=59091 locus=MicpuC2.EuGene.
0000060600 ID=59091.3.0.228 annot-version=v3.0
Micromonas_pusilla OG0000042
108103 pacid=27400223 transcript=108103
locus=est_cluster_kg.Chr_04__81__3200304:1 ID=108103.3.0.229 annot-version=v3.0 Micromonas_sp._RCC299 OG0000042
57487 pacid=27400445 transcript=57487 locus=EuGene.0400010016
ID=57487.3.0.229 annot-version=v3.0 Micromonas_sp._RCC299
OG0000042
63995 pacid=27403937 transcript=63995 locus=EuGene.1400010130
ID=63995.3.0.229 annot-version=v3.0 Micromonas_sp._RCC299
OG0000042
101672 pacid=27404678 transcript=101672
locus=fgenesh2_pg.C_Chr_07000416 ID=101672.3.0.229 annot-version=v3.0 Micromonas_sp._RCC299 OG0000042
kfl00451_0030_v1.1 Klebsormidium_flaccidum OG0000042
kfl00103_0190_v1.1 Klebsormidium_flaccidum OG0000042
kfl00212_0040_v1.1 Klebsormidium_flaccidum OG0000042
kfl00001_0850_v1.1 Klebsormidium_flaccidum OG0000042
kfl00279_0070_v1.1 Klebsormidium_flaccidum OG0000042
Pp3c7_5280V3.1.p pacid=32926291 transcript=Pp3c7_5280V3.1
locus=Pp3c7_5280 annot-version=v3.3 Physcomitrella_patens
OG0000042
Pp3c2_35540V3.1.p pacid=32935566 transcript=Pp3c2_35540V3.1
locus=Pp3c2_35540 annot-version=v3.3 Physcomitrella_patens
OG0000042
Pp3c2_31580V3.1.p pacid=32937192 transcript=Pp3c2_31580V3.1
locus=Pp3c2_31580 annot-version=v3.3 Physcomitrella_patens
OG0000042
Pp3c19_1880V3.1.p pacid=32938326 transcript=Pp3c19_1880V3.1

locus=Pp3c19_1880 annot-version=v3.3 Physcomitrella_patens
OG0000042
Pp3c19_2450V3.1.p pacid=32938579 transcript=Pp3c19_2450V3.1
locus=Pp3c19_2450 annot-version=v3.3 Physcomitrella_patens
OG0000042
Pp3c22_5440V3.1.p pacid=32903575 transcript=Pp3c22_5440V3.1
locus=Pp3c22_5440 annot-version=v3.3 Physcomitrella_patens
OG0000042
Pp3c3_35020V3.1.p pacid=32942681 transcript=Pp3c3_35020V3.1
locus=Pp3c3_35020 annot-version=v3.3 Physcomitrella_patens
OG0000042
Pp3c3_34580V3.1.p pacid=32942969 transcript=Pp3c3_34580V3.1
locus=Pp3c3_34580 annot-version=v3.3 Physcomitrella_patens
OG0000042
Pp3c22_550V3.1.p pacid=32904378 transcript=Pp3c22_550V3.1
locus=Pp3c22_550 annot-version=v3.3 Physcomitrella_patens
OG0000042
Pp3c5_12180V3.1.p pacid=32955626 transcript=Pp3c5_12180V3.1
locus=Pp3c5_12180 annot-version=v3.3 Physcomitrella_patens
OG0000042
Pp3c11_20560V3.1.p pacid=32957410 transcript=Pp3c11_20560V3.1
locus=Pp3c11_20560 annot-version=v3.3 Physcomitrella_patens
OG0000042
Pp3c11_6370V3.1.p pacid=32958658 transcript=Pp3c11_6370V3.1
locus=Pp3c11_6370 annot-version=v3.3 Physcomitrella_patens
OG0000042
Pp3c14_11960V3.1.p pacid=32961605 transcript=Pp3c14_11960V3.1
locus=Pp3c14_11960 annot-version=v3.3 Physcomitrella_patens
OG0000042
Pp3c14_10320V3.1.p pacid=32962642 transcript=Pp3c14_10320V3.1
locus=Pp3c14_10320 annot-version=v3.3 Physcomitrella_patens
OG0000042
Pp3c14_8140V3.1.p pacid=32962667 transcript=Pp3c14_8140V3.1
locus=Pp3c14_8140 annot-version=v3.3 Physcomitrella_patens
OG0000042
Pp3c8_7520V3.1.p pacid=32964327 transcript=Pp3c8_7520V3.1
locus=Pp3c8_7520 annot-version=v3.3 Physcomitrella_patens
OG0000042
Pp3c8_1870V3.1.p pacid=32964433 transcript=Pp3c8_1870V3.1
locus=Pp3c8_1870 annot-version=v3.3 Physcomitrella_patens
OG0000042
Pp3c17_14230V3.1.p pacid=32906263 transcript=Pp3c17_14230V3.1
locus=Pp3c17_14230 annot-version=v3.3 Physcomitrella_patens
OG0000042
Pp3c17_6790V3.1.p pacid=32906367 transcript=Pp3c17_6790V3.1
locus=Pp3c17_6790 annot-version=v3.3 Physcomitrella_patens
OG0000042
Pp3c1_950V3.1.p pacid=32970242 transcript=Pp3c1_950V3.1
locus=Pp3c1_950 annot-version=v3.3 Physcomitrella_patens
OG0000042
Pp3c12_21300V3.1.p pacid=32973311 transcript=Pp3c12_21300V3.1
locus=Pp3c12_21300 annot-version=v3.3 Physcomitrella_patens
OG0000042
Pp3c12_13600V3.1.p pacid=32974922 transcript=Pp3c12_13600V3.1

locus=Pp3c12_13600 annot-version=v3.3   Physcomitrella_patens
OG0000042
Pp3c6_14410V3.1.p pacid=32978532 transcript=Pp3c6_14410V3.1
locus=Pp3c6_14410 annot-version=v3.3   Physcomitrella_patens
OG0000042
Pp3c18_13350V3.1.p pacid=32981601 transcript=Pp3c18_13350V3.1
locus=Pp3c18_13350 annot-version=v3.3   Physcomitrella_patens
OG0000042
Pp3c10_22950V3.1.p pacid=32900469 transcript=Pp3c10_22950V3.1
locus=Pp3c10_22950 annot-version=v3.3   Physcomitrella_patens
OG0000042
Pp3c21_10290V3.1.p pacid=32916073 transcript=Pp3c21_10290V3.1
locus=Pp3c21_10290 annot-version=v3.3   Physcomitrella_patens
OG0000042
Pp3c4_7080V3.1.p pacid=32921184 transcript=Pp3c4_7080V3.1
locus=Pp3c4_7080 annot-version=v3.3   Physcomitrella_patens
OG0000042
Pp3c4_6770V3.1.p pacid=32921791 transcript=Pp3c4_6770V3.1
locus=Pp3c4_6770 annot-version=v3.3   Physcomitrella_patens
OG0000042
Pp3c7_25600V3.1.p pacid=32924614 transcript=Pp3c7_25600V3.1
locus=Pp3c7_25600 annot-version=v3.3   Physcomitrella_patens
OG0000042
Pp3c7_23900V3.1.p pacid=32924704 transcript=Pp3c7_23900V3.1
locus=Pp3c7_23900 annot-version=v3.3   Physcomitrella_patens
OG0000042
AT2G47890.1 | B-box type zinc finger protein with CCT domain-containing protein | Chr2:19608245-19609476 FORWARD LENGTH=332 |
201606   Arabidopsis_thaliana   OG0000042
AT3G02380.1 | CONSTANS-like 2 | Chr3:487438-488624 REVERSE
LENGTH=347 | 201606   Arabidopsis_thaliana   OG0000042
AT3G07650.1 | CONSTANS-like 9 | Chr3:2442494-2443901 FORWARD
LENGTH=372 | 201606   Arabidopsis_thaliana   OG0000042
AT3G12890.1 | activator of spomin::LUC2 | Chr3:4099223-4100277
FORWARD LENGTH=251 | 201606   Arabidopsis_thaliana
OG0000042
AT3G21150.1 | B-box 32 | Chr3:7412713-7413390 REVERSE LENGTH=225 |
201606   Arabidopsis_thaliana   OG0000042
AT3G21880.1 | B-box type zinc finger protein with CCT domain-containing protein | Chr3:7706748-7708093 FORWARD LENGTH=364 |
201606   Arabidopsis_thaliana   OG0000042
AT4G10240.1 | B-box zinc finger family protein |
Chr4:6368967-6369526 REVERSE LENGTH=162 | 201606
Arabidopsis_thaliana   OG0000042
AT4G15250.1 | B-box type zinc finger protein with CCT domain-containing protein | Chr4:8709942-8711106 REVERSE LENGTH=330 |
201606   Arabidopsis_thaliana   OG0000042
AT4G25990.1 | CCT motif family protein | Chr4:13191937-13193543
REVERSE LENGTH=394 | 201606   Arabidopsis_thaliana
OG0000042
AT4G27900.1 | CCT motif family protein | Chr4:13890858-13892777
FORWARD LENGTH=261 | 201606   Arabidopsis_thaliana
OG0000042
AT4G38960.1 | B-box type zinc finger family protein |

Chr4:18161576-18163045 FORWARD LENGTH=183 | 201606
Arabidopsis_thaliana   OG0000042
AT4G39070.1 | B-box zinc finger family protein |
Chr4:18205061-18206421 REVERSE LENGTH=242 | 201606
Arabidopsis_thaliana   OG0000042
AT5G14370.1 | CCT motif family protein | Chr5:4632147-4633651
REVERSE LENGTH=339 | 201606   Arabidopsis_thaliana
OG0000042
AT5G15840.1 | B-box type zinc finger protein with CCT domain-
containing protein | Chr5:5171343-5172697 REVERSE LENGTH=373 |
201606   Arabidopsis_thaliana   OG0000042
AT5G15850.1 | CONSTANS-like 1 | Chr5:5176297-5177473 REVERSE
LENGTH=355 | 201606   Arabidopsis_thaliana   OG0000042
AT5G24930.1 | zinc finger CONSTANS-like protein |
Chr5:8589457-8590949 FORWARD LENGTH=362 | 201606
Arabidopsis_thaliana   OG0000042
AT5G41380.1 | CCT motif family protein | Chr5:16562129-16563553
REVERSE LENGTH=307 | 201606   Arabidopsis_thaliana
OG0000042
AT5G48250.1 | B-box type zinc finger protein with CCT domain-
containing protein | Chr5:19561719-19563113 REVERSE LENGTH=373 |
201606   Arabidopsis_thaliana   OG0000042
AT1G25440.1 | B-box type zinc finger protein with CCT domain-
containing protein | Chr1:8933939-8935284 REVERSE LENGTH=417 |
201606   Arabidopsis_thaliana   OG0000042
AT5G53420.1 | CCT motif family protein | Chr5:21673683-21675469
FORWARD LENGTH=264 | 201606   Arabidopsis_thaliana
OG0000042
AT5G57180.1 | chloroplast import apparatus 2 |
Chr5:23168393-23170294 FORWARD LENGTH=424 | 201606
Arabidopsis_thaliana   OG0000042
AT5G57660.1 | CONSTANS-like 5 | Chr5:23355573-23356729 FORWARD
LENGTH=355 | 201606   Arabidopsis_thaliana   OG0000042
AT5G59990.1 | CCT motif family protein | Chr5:24151206-24153084
REVERSE LENGTH=241 | 201606   Arabidopsis_thaliana
OG0000042
AT1G28050.1 | B-box type zinc finger protein with CCT domain-
containing protein | Chr1:9775768-9777657 REVERSE LENGTH=433 |
201606   Arabidopsis_thaliana   OG0000042
AT1G04500.1 | CCT motif family protein | Chr1:1221757-1224235
REVERSE LENGTH=386 | 201606   Arabidopsis_thaliana
OG0000042
AT1G49130.1 | B-box type zinc finger protein with CCT domain-
containing protein | Chr1:18174741-18175936 REVERSE LENGTH=326 |
201606   Arabidopsis_thaliana   OG0000042
AT1G60250.1 | B-box zinc finger family protein |
Chr1:22217077-22217832 REVERSE LENGTH=251 | 201606
Arabidopsis_thaliana   OG0000042
AT1G63820.1 | CCT motif family protein | Chr1:23682529-23684050
REVERSE LENGTH=293 | 201606   Arabidopsis_thaliana
OG0000042
AT1G06040.1 | B-box zinc finger family protein |
Chr1:1828662-1829659 REVERSE LENGTH=248 | 201606
Arabidopsis_thaliana   OG0000042

AT1G68190.1 | B-box zinc finger family protein |
Chr1:25559384-25561004 FORWARD LENGTH=356 | 201606
Arabidopsis_thaliana   OG0000042
AT1G68520.1 | B-box type zinc finger protein with CCT domain-containing protein | Chr1:25709331-25710749 REVERSE LENGTH=406 |
201606 Arabidopsis_thaliana   OG0000042
AT1G73870.1 | B-box type zinc finger protein with CCT domain-containing protein | Chr1:27779214-27780522 FORWARD LENGTH=392 |
201606 Arabidopsis_thaliana   OG0000042
AT1G07050.1 | CCT motif family protein | Chr1:2164327-2165133
REVERSE LENGTH=195 | 201606   Arabidopsis_thaliana
OG0000042
AT1G75540.1 | salt tolerance homolog2 | Chr1:28366059-28367398
FORWARD LENGTH=331 | 201606   Arabidopsis_thaliana
OG0000042
AT1G78600.1 | light-regulated zinc finger protein 1 |
Chr1:29567370-29568662 FORWARD LENGTH=299 | 201606
Arabidopsis_thaliana   OG0000042
AT2G21320.1 | B-box zinc finger family protein |
Chr2:9126502-9127652 FORWARD LENGTH=172 | 201606
Arabidopsis_thaliana   OG0000042
AT2G24790.1 | CONSTANS-like 3 | Chr2:10566959-10567946 FORWARD
LENGTH=294 | 201606   Arabidopsis_thaliana   OG0000042
AT2G31380.1 | salt tolerance homologue | Chr2:13382150-13383302
FORWARD LENGTH=238 | 201606   Arabidopsis_thaliana
OG0000042
AT2G32310.1 | CCT motif family protein | Chr2:13724710-13726207
FORWARD LENGTH=325 | 201606   Arabidopsis_thaliana
OG0000042
AT2G33350.1 | CCT motif family protein | Chr2:14134116-14136836
FORWARD LENGTH=410 | 201606   Arabidopsis_thaliana
OG0000042
AT2G33500.1 | B-box type zinc finger protein with CCT domain-containing protein | Chr2:14188159-14190046 REVERSE LENGTH=402 |
201606 Arabidopsis_thaliana   OG0000042
Os03t0351100-01 Similar to CONSTANS-like protein CO9 (Fragment).
Oryza_sativa   OG0000042
Os03t0711100-01 Member of the CONSTANS-like (COL) family, Flowering
time repressor   Oryza_sativa   OG0000042
Os04t0493000-01 Similar to H0425E08.14 protein.   Oryza_sativa
OG0000042
Os04t0497700-01 Similar to CONSTANS-like protein. Oryza_sativa
OG0000042
Os04t0540200-01 Zinc finger, B-box domain containing protein.
Oryza_sativa   OG0000042
Os05t0204600-01 B-box zinc finger protein, Repressor of flowering
under long and short-day conditions   Oryza_sativa
OG0000042
Os05t0466100-01 CCT domain containing protein.   Oryza_sativa
OG0000042
Os05t0595300-01 Similar to CCT motif family protein.
Oryza_sativa   OG0000042
Os06t0152200-01 Zinc-finger protein R2931. Oryza_sativa
OG0000042

Os06t0264200-00 Zinc finger, B-box domain containing protein.
Oryza_sativa   OG0000042
Os06t0275000-01 Zinc finger protein, Heading date Oryza_sativa
OG0000042
Os06t0298200-01 Similar to CCT motif family protein.
Oryza_sativa   OG0000042
Os06t0654900-01 Similar to CONSTANS-like protein CO6.
Oryza_sativa   OG0000042
Os06t0661200-00 Similar to B-box zinc finger family protein.
Oryza_sativa   OG0000042
Os06t0699600-01 CCT domain containing protein.   Oryza_sativa
OG0000042
Os06t0713000-01 Zinc finger, B-box domain containing protein.
Oryza_sativa   OG0000042
Os07t0261200-01 CCT(CONSTANS, CONSTANS-LIKE, and TIMING OF CHLOROPHYLL A/B BINDING1) domain protein, Heading date, Long-day repression, Regulator of growth, development, and stress-response
Oryza_sativa   OG0000042
Os07t0667300-01 Zinc finger, B-box domain containing protein.
Oryza_sativa   OG0000042
Os08t0119700-00 CCT domain domain containing protein.
Oryza_sativa   OG0000042
Os08t0249000-01 Zinc finger, B-box domain containing protein.
Oryza_sativa   OG0000042
Os08t0536300-01 Similar to Hd1.   Oryza_sativa   OG0000042
Os09t0240200-01 Zinc finger, B-box domain containing protein.
Oryza_sativa   OG0000042
Os09t0509700-01 Zinc finger, B-box domain containing protein.
Oryza_sativa   OG0000042
Os09t0527900-01 Similar to Hd1-like protein.   Oryza_sativa
OG0000042
Os10t0466500-01 CCT domain containing protein.   Oryza_sativa
OG0000042
Os10t0560400-01 Similar to CONSTANS-like protein CO9 (Fragment).
Oryza_sativa   OG0000042
Os12t0209200-01 Similar to CONSTANS-LIKE a.   Oryza_sativa
OG0000042
Os01t0835700-01 CCT domain containing protein.   Oryza_sativa
OG0000042
Os02t0148000-01 CCT domain containing protein.   Oryza_sativa
OG0000042
Os02t0176000-00 Zinc finger, B-box domain containing protein.
Oryza_sativa   OG0000042
Os02t0178100-01 Similar to CONSTANS-like protein CO6.
Oryza_sativa   OG0000042
Os02t0606200-01 Zinc finger, B-box domain containing protein.
Oryza_sativa   OG0000042
Os02t0610500-01 CO-like protein containing two B-box zinc finger domains and one CCT domain, Constitutive flowering repressor
Oryza_sativa   OG0000042
Os02t0646200-01 Zinc finger, B-box domain containing protein.
Oryza_sativa   OG0000042
Os01t0202500-01 Similar to B-box zinc finger family protein.
Oryza_sativa   OG0000042

Os02t0724000-01 CONSTANS-like protein, Heading promotion under long-day condition	Oryza_sativa	OG0000042
Os02t0731700-01 CCT domain domain containing protein.	Oryza_sativa	OG0000042
Os03t0139500-01 Similar to CONSTANS-like protein CO9 (Fragment).	Oryza_sativa	OG0000042
CMH127CT pep chromosome:ASM9120v1:8:308322:312092:1 gene:CMH127C transcript:CMH127CT gene_biotype:protein_coding transcript_biotype:protein_coding Cyanidioschyzon_merolae	OG0000042
CMS163CT pep chromosome:ASM9120v1:19:432585:433458:-1 gene:CMS163C transcript:CMS163CT gene_biotype:protein_coding transcript_biotype:protein_coding Cyanidioschyzon_merolae	OG0000042
CME043CT pep chromosome:ASM9120v1:5:117091:118783:-1 gene:CME043C transcript:CME043CT gene_biotype:protein_coding transcript_biotype:protein_coding Cyanidioschyzon_merolae	OG0000042
evm.model.contig_3458.11	Porphyridium_purpureum	OG0000042
evm.model.contig_482.9	Porphyridium_purpureum	OG0000042
evm.model.contig_494.11	Porphyridium_purpureum	OG0000042
evm.model.contig_569.7	Porphyridium_purpureum	OG0000042
evm.model.contig_614.7	Porphyridium_purpureum	OG0000042
CDF32814 pep supercontig:ASM35022v2:HG001592:66912:67653:1 gene:CHC_T00001428001 transcript:CDF32814 gene_biotype:protein_coding transcript_biotype:protein_coding Chondrus_crispus OG0000042
CDF36220 pep supercontig:ASM35022v2:HG001766:119891:121223:-1 gene:CHC_T00004610001 transcript:CDF36220 gene_biotype:protein_coding transcript_biotype:protein_coding Chondrus_crispus OG0000042
CDF41347 pep supercontig:ASM35022v2:HG002355:247105:248211:-1 gene:CHC_T00007862001 transcript:CDF41347 gene_biotype:protein_coding transcript_biotype:protein_coding Chondrus_crispus OG0000042
CDF36563 pep supercontig:ASM35022v2:HG001789:23451:24281:-1 gene:CHC_T00004878001 transcript:CDF36563 gene_biotype:protein_coding transcript_biotype:protein_coding Chondrus_crispus OG0000042
CDF32361 pep supercontig:ASM35022v2:HG001477:263567:264918:1 gene:CHC_T00008092001 transcript:CDF32361 gene_biotype:protein_coding transcript_biotype:protein_coding Chondrus_crispus OG0000042
CDF34199 pep supercontig:ASM35022v2:HG001676:213765:214703:-1 gene:CHC_T00002859001 transcript:CDF34199 gene_biotype:protein_coding transcript_biotype:protein_coding Chondrus_crispus OG0000042
CDF40713 pep supercontig:ASM35022v2:HG002233:5842:7149:-1 gene:CHC_T00000858001 transcript:CDF40713 gene_biotype:protein_coding transcript_biotype:protein_coding Chondrus_crispus OG0000042
CDF39610 pep supercontig:ASM35022v2:HG002055:40604:42760:1 gene:CHC_T00000361001 transcript:CDF39610 gene_biotype:protein_coding transcript_biotype:protein_coding

Chondrus_crispus OG0000042
CDF32176 pep supercontig:ASM35022v2:HG001459:608320:608868:-1
gene:CHC_T00001403001 transcript:CDF32176
gene_biotype:protein_coding transcript_biotype:protein_coding
Chondrus_crispus OG0000042
CDF38371 pep supercontig:ASM35022v2:HG001923:39100:39758:1
gene:CHC_T00000958001 transcript:CDF38371
gene_biotype:protein_coding transcript_biotype:protein_coding
Chondrus_crispus OG0000042
CDF39138 pep supercontig:ASM35022v2:HG002003:33352:33775:1
gene:CHC_T00000117001 transcript:CDF39138
gene_biotype:protein_coding transcript_biotype:protein_coding
Chondrus_crispus OG0000042
CDF37798 pep supercontig:ASM35022v2:HG001871:121631:122017:1
gene:CHC_T00005726001 transcript:CDF37798
gene_biotype:protein_coding transcript_biotype:protein_coding
Chondrus_crispus OG0000042
g1781.t1 Caulerpa_lentillifera OG0000043
g2419.t1 Caulerpa_lentillifera OG0000043
g2504.t1 Caulerpa_lentillifera OG0000043
g3028.t1 Caulerpa_lentillifera OG0000043
g3952.t1 Caulerpa_lentillifera OG0000043
g4511.t1 Caulerpa_lentillifera OG0000043
g4552.t1 Caulerpa_lentillifera OG0000043
g4733.t1 Caulerpa_lentillifera OG0000043
g5371.t1 Caulerpa_lentillifera OG0000043
g5850.t1 Caulerpa_lentillifera OG0000043
g5907.t1 Caulerpa_lentillifera OG0000043
g6642.t1 Caulerpa_lentillifera OG0000043
g6668.t1 Caulerpa_lentillifera OG0000043
g6672.t1 Caulerpa_lentillifera OG0000043
g6935.t1 Caulerpa_lentillifera OG0000043
g709.t1 Caulerpa_lentillifera OG0000043
g8950.t1 Caulerpa_lentillifera OG0000043
g9087.t1 Caulerpa_lentillifera OG0000043
Cre03.g144344.t1.1 pacid=30786967 transcript=Cre03.g144344.t1.1
locus=Cre03.g144344 ID=Cre03.g144344.t1.1.v5.5 annot-version=v5.5
Chlamydomonas_reinhardtii OG0000043
Cre01.g016800.t1.1 pacid=30789219 transcript=Cre01.g016800.t1.1
locus=Cre01.g016800 ID=Cre01.g016800.t1.1.v5.5 annot-version=v5.5
Chlamydomonas_reinhardtii OG0000043
Cre10.g417850.t1.1 pacid=30790386 transcript=Cre10.g417850.t1.1
locus=Cre10.g417850 ID=Cre10.g417850.t1.1.v5.5 annot-version=v5.5
Chlamydomonas_reinhardtii OG0000043
Cre10.g444100.t1.1 pacid=30790477 transcript=Cre10.g444100.t1.1
locus=Cre10.g444100 ID=Cre10.g444100.t1.1.v5.5 annot-version=v5.5
Chlamydomonas_reinhardtii OG0000043
Cre10.g443950.t1.1 pacid=30790488 transcript=Cre10.g443950.t1.1
locus=Cre10.g443950 ID=Cre10.g443950.t1.1.v5.5 annot-version=v5.5
Chlamydomonas_reinhardtii OG0000043
Cre14.g612850.t1.1 pacid=30776377 transcript=Cre14.g612850.t1.1
locus=Cre14.g612850 ID=Cre14.g612850.t1.1.v5.5 annot-version=v5.5
Chlamydomonas_reinhardtii OG0000043
Cre16.g689250.t1.1 pacid=30778174 transcript=Cre16.g689250.t1.1

locus=Cre16.g689250 ID=Cre16.g689250.t1.1.v5.5 annot-version=v5.5
Chlamydomonas_reinhardtii OG0000043
Cre09.g390356.t1.1 pacid=30780572 transcript=Cre09.g390356.t1.1
locus=Cre09.g390356 ID=Cre09.g390356.t1.1.v5.5 annot-version=v5.5
Chlamydomonas_reinhardtii OG0000043
Vocar.0025s0203.1.p pacid=32898235 transcript=Vocar.0025s0203.1
locus=Vocar.0025s0203 ID=Vocar.0025s0203.1.v2.1 annot-version=v2.1
Volvox_carteri OG0000043
Vocar.0001s1347.1.p pacid=32885231 transcript=Vocar.0001s1347.1
locus=Vocar.0001s1347 ID=Vocar.0001s1347.1.v2.1 annot-version=v2.1
Volvox_carteri OG0000043
Vocar.0007s0430.1.p pacid=32888119 transcript=Vocar.0007s0430.1
locus=Vocar.0007s0430 ID=Vocar.0007s0430.1.v2.1 annot-version=v2.1
Volvox_carteri OG0000043
Vocar.0005s0420.1.p pacid=32884043 transcript=Vocar.0005s0420.1
locus=Vocar.0005s0420 ID=Vocar.0005s0420.1.v2.1 annot-version=v2.1
Volvox_carteri OG0000043
Vocar.0059s0023.1.p pacid=32893612 transcript=Vocar.0059s0023.1
locus=Vocar.0059s0023 ID=Vocar.0059s0023.1.v2.1 annot-version=v2.1
Volvox_carteri OG0000043
jgi|ChlNC64A_1|133889|IGS.gm_10_00180 Chlorella_variabilis
OG0000043
jgi|ChlNC64A_1|144450|IGS.gm_7_00392 Chlorella_variabilis
OG0000043
jgi|ChlNC64A_1|15720|gw1.22.186.1 Chlorella_variabilis
OG0000043
jgi|ChlNC64A_1|136362|IGS.gm_16_00085 Chlorella_variabilis
OG0000043
66038 pacid=27387022 transcript=66038 locus=estExt_Genemark1.C_70331
ID=66038.2.0.227 annot-version=v2.0 Coccomyxa_subellipsoidea
OG0000043
65478 pacid=27387312 transcript=65478 locus=estExt_Genemark1.C_50299
ID=65478.2.0.227 annot-version=v2.0 Coccomyxa_subellipsoidea
OG0000043
55295 pacid=27389574 transcript=55295 locus=Genemark1.239_g
ID=55295.2.0.227 annot-version=v2.0 Coccomyxa_subellipsoidea
OG0000043
63467 pacid=27392375 transcript=63467 locus=Genemark1.4250_g
ID=63467.2.0.227 annot-version=v2.0 Coccomyxa_subellipsoidea
OG0000043
10872_fgenesh1_pg.C_Chr_05.0001000007 Ostreococcus_tauri
OG0000043
31475 pacid=27416643 transcript=31475 locus=eugene.0500010012
ID=31475.2.0.231 annot-version=v2.0 Ostreococcus_lucimarinus
OG0000043
9923 pacid=27413863 transcript=9923 locus=gwEuk.9.573.1
ID=9923.2.0.231 annot-version=v2.0 Ostreococcus_lucimarinus
OG0000043
179454 pacid=27340715 transcript=179494
locus=estExt_Genewise1Plus.C_7_t20427 ID=179494.3.0.228 annot-
version=v3.0 Micromonas_pusilla OG0000043
9130 pacid=27344637 transcript=9130 locus=MicpuC2.gw1.17.180.1
ID=9130.3.0.228 annot-version=v3.0 Micromonas_pusilla
OG0000043

107978 pacid=27398416 transcript=107978
locus=est_cluster_kg.Chr_03__135__3202130:2 ID=107978.3.0.229 annot-version=v3.0   Micromonas_sp._RCC299   OG0000043
58878 pacid=27395595 transcript=58878 locus=EuGene.0500010611
ID=58878.3.0.229 annot-version=v3.0   Micromonas_sp._RCC299
OG0000043
55612 pacid=27399186 transcript=55612 locus=EuGene.0100010839
ID=55612.3.0.229 annot-version=v3.0   Micromonas_sp._RCC299
OG0000043
61042 pacid=27401152 transcript=61042 locus=EuGene.0900010025
ID=61042.3.0.229 annot-version=v3.0   Micromonas_sp._RCC299
OG0000043
64941 pacid=27403002 transcript=64941 locus=EuGene.1700010002
ID=64941.3.0.229 annot-version=v3.0   Micromonas_sp._RCC299
OG0000043
kfl00335_0110_v1.1   Klebsormidium_flaccidum OG0000043
kfl00372_0100_v1.1   Klebsormidium_flaccidum OG0000043
kfl00376_0010_v1.1   Klebsormidium_flaccidum OG0000043
kfl00020_0500_v1.1   Klebsormidium_flaccidum OG0000043
kfl00021_0150_v1.1   Klebsormidium_flaccidum OG0000043
kfl00003_0330_v1.1   Klebsormidium_flaccidum OG0000043
kfl00038_0360_v1.1   Klebsormidium_flaccidum OG0000043
kfl00121_0220_v1.1   Klebsormidium_flaccidum OG0000043
kfl00200_0180_v1.1   Klebsormidium_flaccidum OG0000043
kfl00229_0150_v1.1   Klebsormidium_flaccidum OG0000043
kfl00013_0630_v1.1   Klebsormidium_flaccidum OG0000043
Pp3c10_4480V3.1.p pacid=32902552 transcript=Pp3c10_4480V3.1
locus=Pp3c10_4480 annot-version=v3.3   Physcomitrella_patens
OG0000043
Pp3c13_7510V3.1.p pacid=32930755 transcript=Pp3c13_7510V3.1
locus=Pp3c13_7510 annot-version=v3.3   Physcomitrella_patens
OG0000043
Pp3c13_16440V3.1.p pacid=32931376 transcript=Pp3c13_16440V3.1
locus=Pp3c13_16440 annot-version=v3.3   Physcomitrella_patens
OG0000043
Pp3c3_32490V3.1.p pacid=32940270 transcript=Pp3c3_32490V3.1
locus=Pp3c3_32490 annot-version=v3.3   Physcomitrella_patens
OG0000043
Pp3c3_4891V3.1.p pacid=32944363 transcript=Pp3c3_4891V3.1
locus=Pp3c3_4891 annot-version=v3.3   Physcomitrella_patens
OG0000043
Pp3c3_24250V3.1.p pacid=32944533 transcript=Pp3c3_24250V3.1
locus=Pp3c3_24250 annot-version=v3.3   Physcomitrella_patens
OG0000043
Pp3c20_8020V3.1.p pacid=32947711 transcript=Pp3c20_8020V3.1
locus=Pp3c20_8020 annot-version=v3.3   Physcomitrella_patens
OG0000043
Pp3c20_16680V3.1.p pacid=32947950 transcript=Pp3c20_16680V3.1
locus=Pp3c20_16680 annot-version=v3.3   Physcomitrella_patens
OG0000043
Pp3c23_15760V3.1.p pacid=32950480 transcript=Pp3c23_15760V3.1
locus=Pp3c23_15760 annot-version=v3.3   Physcomitrella_patens
OG0000043
Pp3c23_8770V3.1.p pacid=32950497 transcript=Pp3c23_8770V3.1

locus=Pp3c23_8770 annot-version=v3.3   Physcomitrella_patens
OG0000043
Pp3c5_19350V3.1.p pacid=32952991 transcript=Pp3c5_19350V3.1
locus=Pp3c5_19350 annot-version=v3.3   Physcomitrella_patens
OG0000043
Pp3c5_12340V3.1.p pacid=32953523 transcript=Pp3c5_12340V3.1
locus=Pp3c5_12340 annot-version=v3.3   Physcomitrella_patens
OG0000043
Pp3c17_5550V3.1.p pacid=32905737 transcript=Pp3c17_5550V3.1
locus=Pp3c17_5550 annot-version=v3.3   Physcomitrella_patens
OG0000043
Pp3c8_3110V3.1.p pacid=32965003 transcript=Pp3c8_3110V3.1
locus=Pp3c8_3110 annot-version=v3.3   Physcomitrella_patens
OG0000043
Pp3c1_32770V3.1.p pacid=32967168 transcript=Pp3c1_32770V3.1
locus=Pp3c1_32770 annot-version=v3.3   Physcomitrella_patens
OG0000043
Pp3c12_4020V3.1.p pacid=32973893 transcript=Pp3c12_4020V3.1
locus=Pp3c12_4020 annot-version=v3.3   Physcomitrella_patens
OG0000043
Pp3c6_15250V3.1.p pacid=32975646 transcript=Pp3c6_15250V3.1
locus=Pp3c6_15250 annot-version=v3.3   Physcomitrella_patens
OG0000043
Pp3c17_580V3.1.p pacid=32908544 transcript=Pp3c17_580V3.1
locus=Pp3c17_580 annot-version=v3.3   Physcomitrella_patens
OG0000043
Pp3c24_16470V3.1.p pacid=32909499 transcript=Pp3c24_16470V3.1
locus=Pp3c24_16470 annot-version=v3.3   Physcomitrella_patens
OG0000043
Pp3c24_15640V3.1.p pacid=32910165 transcript=Pp3c24_15640V3.1
locus=Pp3c24_15640 annot-version=v3.3   Physcomitrella_patens
OG0000043
Pp3c10_20630V3.1.p pacid=32900508 transcript=Pp3c10_20630V3.1
locus=Pp3c10_20630 annot-version=v3.3   Physcomitrella_patens
OG0000043
Pp3c24_4740V3.1.p pacid=32910701 transcript=Pp3c24_4740V3.1
locus=Pp3c24_4740 annot-version=v3.3   Physcomitrella_patens
OG0000043
Pp3c9_17047V3.1.p pacid=32912204 transcript=Pp3c9_17047V3.1
locus=Pp3c9_17047 annot-version=v3.3   Physcomitrella_patens
OG0000043
Pp3c9_17040V3.1.p pacid=32913183 transcript=Pp3c9_17040V3.1
locus=Pp3c9_17040 annot-version=v3.3   Physcomitrella_patens
OG0000043
Pp3c26_12730V3.1.p pacid=32917316 transcript=Pp3c26_12730V3.1
locus=Pp3c26_12730 annot-version=v3.3   Physcomitrella_patens
OG0000043
Pp3c26_8290V3.1.p pacid=32917621 transcript=Pp3c26_8290V3.1
locus=Pp3c26_8290 annot-version=v3.3   Physcomitrella_patens
OG0000043
Pp3c4_28800V3.1.p pacid=32920015 transcript=Pp3c4_28800V3.1
locus=Pp3c4_28800 annot-version=v3.3   Physcomitrella_patens
OG0000043
Pp3c4_20710V3.1.p pacid=32920233 transcript=Pp3c4_20710V3.1

locus=Pp3c4_20710 annot-version=v3.3   Physcomitrella_patens
OG0000043
Pp3c4_6500V3.1.p pacid=32921121 transcript=Pp3c4_6500V3.1
locus=Pp3c4_6500 annot-version=v3.3   Physcomitrella_patens
OG0000043
AT2G35930.1 | plant U-box 23 | Chr2:15083101-15084336 REVERSE
LENGTH=411 | 201606   Arabidopsis_thaliana   OG0000043
AT1G10560.1 | plant U-box 18 | Chr1:3484613-3486706 FORWARD
LENGTH=697 | 201606   Arabidopsis_thaliana   OG0000043
AT3G01400.1 | ARM repeat superfamily protein | Chr3:151920-152987
FORWARD LENGTH=355 | 201606   Arabidopsis_thaliana
OG0000043
AT3G02840.1 | ARM repeat superfamily protein | Chr3:618480-619619
FORWARD LENGTH=379 | 201606   Arabidopsis_thaliana
OG0000043
AT3G07360.1 | plant U-box 9 | Chr3:2354884-2356613 FORWARD
LENGTH=460 | 201606   Arabidopsis_thaliana   OG0000043
AT3G11840.1 | E3 ubiquitin-protein ligase PUB24-like protein |
Chr3:3736578-3738208 REVERSE LENGTH=456 | 201606
Arabidopsis_thaliana   OG0000043
AT3G18710.1 | plant U-box 29 | Chr3:6434234-6435481 REVERSE
LENGTH=415 | 201606   Arabidopsis_thaliana   OG0000043
AT3G19380.1 | plant U-box 25 | Chr3:6714602-6715867 REVERSE
LENGTH=421 | 201606   Arabidopsis_thaliana   OG0000043
AT3G46510.1 | plant U-box 13 | Chr3:17124106-17126539 REVERSE
LENGTH=660 | 201606   Arabidopsis_thaliana   OG0000043
AT3G47820.1 | PLANT U-BOX 39 | Chr3:17644368-17645963 FORWARD
LENGTH=531 | 201606   Arabidopsis_thaliana   OG0000043
AT3G52450.1 | plant U-box 22 | Chr3:19440943-19442250 REVERSE
LENGTH=435 | 201606   Arabidopsis_thaliana   OG0000043
AT3G54790.1 | ARM repeat superfamily protein |
Chr3:20281830-20284363 REVERSE LENGTH=760 | 201606
Arabidopsis_thaliana   OG0000043
AT3G54850.1 | plant U-box 14 | Chr3:20321524-20323848 FORWARD
LENGTH=632 | 201606   Arabidopsis_thaliana   OG0000043
AT4G12710.1 | ARM repeat superfamily protein | Chr4:7485040-7486733
REVERSE LENGTH=402 | 201606   Arabidopsis_thaliana
OG0000043
AT4G16490.1 | ARM repeat superfamily protein | Chr4:9293891-9295530
REVERSE LENGTH=472 | 201606   Arabidopsis_thaliana
OG0000043
AT4G21350.1 | plant U-box 8 | Chr4:11356143-11357267 REVERSE
LENGTH=374 | 201606   Arabidopsis_thaliana   OG0000043
AT4G36550.1 | ARM repeat superfamily protein |
Chr4:17245400-17247926 REVERSE LENGTH=718 | 201606
Arabidopsis_thaliana   OG0000043
AT5G01830.1 | ARM repeat superfamily protein | Chr5:320983-323007
FORWARD LENGTH=674 | 201606   Arabidopsis_thaliana
OG0000043
AT5G09800.1 | ARM repeat superfamily protein | Chr5:3043123-3044352
REVERSE LENGTH=409 | 201606   Arabidopsis_thaliana
OG0000043
AT5G14510.1 | ARM repeat superfamily protein | Chr5:4678125-4679194
REVERSE LENGTH=327 | 201606   Arabidopsis_thaliana

OG0000043
AT5G18320.1 | ARM repeat superfamily protein | Chr5:6064431-6066186
REVERSE LENGTH=458 | 201606 Arabidopsis_thaliana
OG0000043
AT5G18330.1 | ARM repeat superfamily protein | Chr5:6068474-6070042
REVERSE LENGTH=445 | 201606 Arabidopsis_thaliana
OG0000043
AT5G18340.1 | ARM repeat superfamily protein | Chr5:6070640-6072198
REVERSE LENGTH=456 | 201606 Arabidopsis_thaliana
OG0000043
AT1G23030.1 | ARM repeat superfamily protein | Chr1:8156745-8158842
FORWARD LENGTH=612 | 201606 Arabidopsis_thaliana
OG0000043
AT5G37490.1 | ARM repeat superfamily protein |
Chr5:14887744-14889051 FORWARD LENGTH=435 | 201606
Arabidopsis_thaliana OG0000043
AT5G40140.1 | RING/U-box superfamily protein with ARM repeat domain-
containing protein | Chr5:16057347-16058999 FORWARD LENGTH=550 |
201606 Arabidopsis_thaliana OG0000043
AT5G42340.1 | Plant U-Box 15 | Chr5:16928086-16930367 REVERSE
LENGTH=660 | 201606 Arabidopsis_thaliana OG0000043
AT5G58680.1 | ARM repeat superfamily protein |
Chr5:23708247-23709320 REVERSE LENGTH=357 | 201606
Arabidopsis_thaliana OG0000043
AT5G62560.1 | RING/U-box superfamily protein with ARM repeat domain-
containing protein | Chr5:25110073-25111752 FORWARD LENGTH=559 |
201606 Arabidopsis_thaliana OG0000043
AT5G64660.1 | CYS%2C MET%2C PRO%2C and GLY protein 2 |
Chr5:25842119-25843381 REVERSE LENGTH=420 | 201606
Arabidopsis_thaliana OG0000043
AT5G65200.1 | plant U-box 38 | Chr5:26048173-26049843 REVERSE
LENGTH=556 | 201606 Arabidopsis_thaliana OG0000043
AT5G67340.1 | ARM repeat superfamily protein |
Chr5:26864996-26867450 FORWARD LENGTH=707 | 201606
Arabidopsis_thaliana OG0000043
AT1G29340.1 | plant U-box 17 | Chr1:10264412-10266601 FORWARD
LENGTH=729 | 201606 Arabidopsis_thaliana OG0000043
AT1G49780.1 | plant U-box 26 | Chr1:18429024-18430289 REVERSE
LENGTH=421 | 201606 Arabidopsis_thaliana OG0000043
AT1G60190.1 | ARM repeat superfamily protein |
Chr1:22198403-22200463 FORWARD LENGTH=686 | 201606
Arabidopsis_thaliana OG0000043
AT1G66160.1 | CYS%2C MET%2C PRO%2C and GLY protein 1 |
Chr1:24637218-24638513 FORWARD LENGTH=431 | 201606
Arabidopsis_thaliana OG0000043
AT1G71020.1 | ARM repeat superfamily protein |
Chr1:26790825-26793105 REVERSE LENGTH=628 | 201606
Arabidopsis_thaliana OG0000043
AT1G01660.1 | RING/U-box superfamily protein | Chr1:240057-242608
REVERSE LENGTH=568 | 201606 Arabidopsis_thaliana
OG0000043
AT2G23140.1 | RING/U-box superfamily protein with ARM repeat domain-
containing protein | Chr2:9845696-9849105 REVERSE LENGTH=829 |
201606 Arabidopsis_thaliana OG0000043

AT2G28830.1 | armadillo/beta-catenin repeat protein |
Chr2:12368388-12370388 REVERSE LENGTH=582 | 201606
Arabidopsis_thaliana OG0000043
Os03t0240600-01 Zinc finger, RING/FYVE/PHD-type domain containing protein. Oryza_sativa OG0000043
Os03t0275900-01 Similar to Spotted leaf protein 11 (Spotted leaf11) (Cell death-related protein SPL11). Oryza_sativa
OG0000043
Os03t0435300-00 Similar to exodeoxyribonuclease V. Oryza_sativa
OG0000043
Os04t0348400-01 Similar to OSIGBa0130K07.9 protein.
Oryza_sativa OG0000043
Os04t0418500-01 Similar to photoperiod responsive protein.
Oryza_sativa OG0000043
Os04t0437300-01 Zinc finger, RING/FYVE/PHD-type domain containing protein. Oryza_sativa OG0000043
Os04t0589700-01 Zinc finger, RING/FYVE/PHD-type domain containing protein. Oryza_sativa OG0000043
Os04t0686000-01 Zinc finger, RING/FYVE/PHD-type domain containing protein. Oryza_sativa OG0000043
Os05t0439400-01 Similar to Arm repeat containing protein.
Oryza_sativa OG0000043
Os05t0476700-01 Similar to Arm repeat protein. Oryza_sativa
OG0000043
Os06t0102700-01 Similar to predicted protein. Oryza_sativa
OG0000043
Os06t0159900-01 Similar to Arm repeat containing protein.
Oryza_sativa OG0000043
Os06t0238000-00 Armadillo-like helical domain containing protein.
Oryza_sativa OG0000043
Os06t0238200-00 Similar to ubiquitin-protein ligase.
Oryza_sativa OG0000043
Os06t0248500-00 U box domain containing protein. Oryza_sativa
OG0000043
Os06t0480000-01 Zinc finger, RING/FYVE/PHD-type domain containing protein. Oryza_sativa OG0000043
Os06t0726900-00 Armadillo domain containing protein.
Oryza_sativa OG0000043
Os07t0584900-01 Armadillo-like helical domain containing protein.
Oryza_sativa OG0000043
Os07t0587500-01 Armadillo-like helical domain containing protein.
Oryza_sativa OG0000043
Os08t0110500-01 U-box/ARM repeat E3 ligase, Stress response, Regulation of cell death, Blast disease resistance Oryza_sativa
OG0000043
Os08t0415600-00 Similar to ubiquitin-protein ligase.
Oryza_sativa OG0000043
Os08t0421850-00 Similar to ubiquitin-protein ligase.
Oryza_sativa OG0000043
Os08t0481200-01 Similar to Arm repeat containing protein.
Oryza_sativa OG0000043
Os09t0378700-00 Similar to ubiquitin-protein ligase.
Oryza_sativa OG0000043
Os09t0386200-01 Armadillo-like helical domain containing protein.

Oryza_sativa	OG0000043
Os12t0161100-00 Zinc finger, RING/FYVE/PHD-type domain containing protein.	Oryza_sativa	OG0000043
Os12t0570000-01 Armadillo-like helical domain containing protein.	Oryza_sativa	OG0000043
Os01t0823900-01 Arm repeat protein.	Oryza_sativa	OG0000043
Os01t0865700-00 Zinc finger, RING/FYVE/PHD-type domain containing protein.	Oryza_sativa	OG0000043
Os01t0884400-01 Armadillo domain containing protein.	Oryza_sativa	OG0000043
Os02t0215650-00 Similar to OSIGBa0092M08.11 protein.	Oryza_sativa	OG0000043
Os02t0234300-01 Armadillo-like helical domain containing protein.	Oryza_sativa	OG0000043
Os02t0488600-01 Armadillo-like helical domain containing protein.	Oryza_sativa	OG0000043
Os02t0539200-01 Zinc finger, RING/FYVE/PHD-type domain containing protein.	Oryza_sativa	OG0000043
Os02t0540700-00 Similar to Arm repeat-containing protein.	Oryza_sativa	OG0000043
Os02t0548700-00 Similar to ubiquitin-protein ligase.	Oryza_sativa	OG0000043
Os02t0690600-01 Zinc finger, RING/FYVE/PHD-type domain containing protein.	Oryza_sativa	OG0000043
Os02t0705600-01 Armadillo-like helical domain containing protein.	Oryza_sativa	OG0000043
Os02t0732200-01 Armadillo-like helical domain containing protein.	Oryza_sativa	OG0000043
Os02t0738200-01 Zinc finger, RING/FYVE/PHD-type domain containing protein.	Oryza_sativa	OG0000043
Os01t0229700-00 WD40 repeat domain containing protein.	Oryza_sativa	OG0000043
g2387.t1	Caulerpa_lentillifera	OG0000044
Cre03.g169000.t1.2 pacid=30787148 transcript=Cre03.g169000.t1.2 locus=Cre03.g169000 ID=Cre03.g169000.t1.2.v5.5 annot-version=v5.5	Chlamydomonas_reinhardtii	OG0000044
Cre06.g272200.t1.2 pacid=30779418 transcript=Cre06.g272200.t1.2 locus=Cre06.g272200 ID=Cre06.g272200.t1.2.v5.5 annot-version=v5.5	Chlamydomonas_reinhardtii	OG0000044
Vocar.0040s0073.1.p pacid=32886272 transcript=Vocar.0040s0073.1 locus=Vocar.0040s0073 ID=Vocar.0040s0073.1.v2.1 annot-version=v2.1	Volvox_carteri	OG0000044
jgi|ChlNC64A_1|133431|IGS.gm_1_00567	Chlorella_variabilis	OG0000044
31600_0300010154	Ostreococcus_tauri	OG0000044
34318_0800010205	Ostreococcus_tauri	OG0000044
33071 pacid=27419586 transcript=33071 locus=eugene.0800010197 ID=33071.2.0.231 annot-version=v2.0	Ostreococcus_lucimarinus	OG0000044
53713 pacid=27348804 transcript=53713 locus=MicpuC2.EuGene.0000150388 ID=53713.3.0.228 annot-version=v3.0	Micromonas_pusilla	OG0000044
52171 pacid=27349149 transcript=52171 locus=MicpuC2.EuGene.

0000120236 ID=52171.3.0.228 annot-version=v3.0
Micromonas_pusilla	OG0000044
190221 pacid=27349253 transcript=190261 locus=gm1.7342_g
ID=190261.3.0.228 annot-version=v3.0	Micromonas_pusilla
OG0000044
52364 pacid=27349281 transcript=52364 locus=MicpuC2.EuGene.
0000120429 ID=52364.3.0.228 annot-version=v3.0
Micromonas_pusilla	OG0000044
6517 pacid=27339726 transcript=6517 locus=MicpuC2.gw1.13.236.1
ID=6517.3.0.228 annot-version=v3.0	Micromonas_pusilla
OG0000044
190290 pacid=27339767 transcript=190330 locus=gm1.7411_g
ID=190330.3.0.228 annot-version=v3.0	Micromonas_pusilla
OG0000044
190536 pacid=27339831 transcript=190576 locus=gm1.7657_g
ID=190576.3.0.228 annot-version=v3.0	Micromonas_pusilla
OG0000044
190544 pacid=27339834 transcript=190584 locus=gm1.7665_g
ID=190584.3.0.228 annot-version=v3.0	Micromonas_pusilla
OG0000044
190483 pacid=27339886 transcript=190523 locus=gm1.7604_g
ID=190523.3.0.228 annot-version=v3.0	Micromonas_pusilla
OG0000044
190541 pacid=27339942 transcript=190581 locus=gm1.7662_g
ID=190581.3.0.228 annot-version=v3.0	Micromonas_pusilla
OG0000044
52819 pacid=27339958 transcript=52819 locus=MicpuC2.EuGene.
0000130376 ID=52819.3.0.228 annot-version=v3.0
Micromonas_pusilla	OG0000044
190293 pacid=27340093 transcript=190333 locus=gm1.7414_g
ID=190333.3.0.228 annot-version=v3.0	Micromonas_pusilla
OG0000044
42528 pacid=27340107 transcript=42528
locus=MicpuC2.fgenesh1_pg.C_scaffold_13000178 ID=42528.3.0.228
annot-version=v3.0	Micromonas_pusilla	OG0000044
21992 pacid=27340125 transcript=21992 locus=MicpuC2.e_gw1.13.417.1
ID=21992.3.0.228 annot-version=v3.0	Micromonas_pusilla
OG0000044
187812 pacid=27338809 transcript=187852 locus=gm1.4933_g
ID=187852.3.0.228 annot-version=v3.0	Micromonas_pusilla
OG0000044
51128 pacid=27340395 transcript=51128 locus=MicpuC2.EuGene.
0000100199 ID=51128.3.0.228 annot-version=v3.0
Micromonas_pusilla	OG0000044
65230 pacid=27340683 transcript=65230 locus=MicpuC2.est_orfs.
7_4213_4270175:2 ID=65230.3.0.228 annot-version=v3.0
Micromonas_pusilla	OG0000044
7705 pacid=27340751 transcript=7705 locus=MicpuC2.gw1.7.405.1
ID=7705.3.0.228 annot-version=v3.0	Micromonas_pusilla
OG0000044
198336 pacid=27340817 transcript=198376 locus=fgenesh1_pg.7_#_133
ID=198376.3.0.228 annot-version=v3.0	Micromonas_pusilla
OG0000044
127230 pacid=27340843 transcript=127270

locus=estExt_fgenesh2_kg.C_70086 ID=127270.3.0.228 annot-version=v3.0 Micromonas_pusilla OG0000044
123483 pacid=27340976 transcript=123523 locus=fgenesh2_kg.7_#_2_#_4270175:1 ID=123523.3.0.228 annot-version=v3.0 Micromonas_pusilla OG0000044
10629 pacid=27340989 transcript=10629 locus=MicpuC2.gw1.7.567.1 ID=10629.3.0.228 annot-version=v3.0 Micromonas_pusilla OG0000044
187763 pacid=27341089 transcript=187803 locus=gm1.4884_g ID=187803.3.0.228 annot-version=v3.0 Micromonas_pusilla OG0000044
157318 pacid=27341221 transcript=157358 locus=e_gw1.7.986.1 ID=157358.3.0.228 annot-version=v3.0 Micromonas_pusilla OG0000044
58433 pacid=27341327 transcript=58433 locus=MicpuC2.EuGene.0000050738 ID=58433.3.0.228 annot-version=v3.0 Micromonas_pusilla OG0000044
58385 pacid=27341641 transcript=58385 locus=MicpuC2.EuGene.0000050690 ID=58385.3.0.228 annot-version=v3.0 Micromonas_pusilla OG0000044
57814 pacid=27341712 transcript=57814 locus=MicpuC2.EuGene.0000050119 ID=57814.3.0.228 annot-version=v3.0 Micromonas_pusilla OG0000044
187810 pacid=27338987 transcript=187850 locus=gm1.4931_g ID=187850.3.0.228 annot-version=v3.0 Micromonas_pusilla OG0000044
22202 pacid=27342166 transcript=22202 locus=MicpuC2.e_gw1.14.228.1 ID=22202.3.0.228 annot-version=v3.0 Micromonas_pusilla OG0000044
187891 pacid=27339004 transcript=187931 locus=gm1.5012_g ID=187931.3.0.228 annot-version=v3.0 Micromonas_pusilla OG0000044
53296 pacid=27342271 transcript=53296 locus=MicpuC2.EuGene.0000140391 ID=53296.3.0.228 annot-version=v3.0 Micromonas_pusilla OG0000044
190864 pacid=27342294 transcript=190904 locus=gm1.7985_g ID=190904.3.0.228 annot-version=v3.0 Micromonas_pusilla OG0000044
52918 pacid=27342315 transcript=52918 locus=MicpuC2.EuGene.0000140013 ID=52918.3.0.228 annot-version=v3.0 Micromonas_pusilla OG0000044
59818 pacid=27339017 transcript=59818 locus=MicpuC2.EuGene.0000080012 ID=59818.3.0.228 annot-version=v3.0 Micromonas_pusilla OG0000044
184029 pacid=27342676 transcript=184069 locus=gm1.1150_g ID=184069.3.0.228 annot-version=v3.0 Micromonas_pusilla OG0000044
188254 pacid=27339049 transcript=188294 locus=gm1.5375_g ID=188294.3.0.228 annot-version=v3.0 Micromonas_pusilla OG0000044
158966 pacid=27339083 transcript=159006 locus=e_gw1.8.155.1 ID=159006.3.0.228 annot-version=v3.0 Micromonas_pusilla OG0000044
191473 pacid=27343300 transcript=191513 locus=gm1.8594_g

ID=191513.3.0.228 annot-version=v3.0	Micromonas_pusilla
OG0000044
53884 pacid=27343321 transcript=53884 locus=MicpuC2.EuGene.
0000160160 ID=53884.3.0.228 annot-version=v3.0
Micromonas_pusilla	OG0000044
53959 pacid=27343335 transcript=53959 locus=MicpuC2.EuGene.
0000160235 ID=53959.3.0.228 annot-version=v3.0
Micromonas_pusilla	OG0000044
173885 pacid=27343385 transcript=173925
locus=estExt_Genewise1.C_16_t10090 ID=173925.3.0.228 annot-
version=v3.0	Micromonas_pusilla	OG0000044
60360 pacid=27339128 transcript=60360 locus=MicpuC2.EuGene.
0000080554 ID=60360.3.0.228 annot-version=v3.0
Micromonas_pusilla	OG0000044
191522 pacid=27343512 transcript=191562 locus=gm1.8643_g
ID=191562.3.0.228 annot-version=v3.0	Micromonas_pusilla
OG0000044
191486 pacid=27343519 transcript=191526 locus=gm1.8607_g
ID=191526.3.0.228 annot-version=v3.0	Micromonas_pusilla
OG0000044
188347 pacid=27339131 transcript=188387 locus=gm1.5468_g
ID=188387.3.0.228 annot-version=v3.0	Micromonas_pusilla
OG0000044
11504 pacid=27343550 transcript=11504 locus=MicpuC2.gw1.16.418.1
ID=11504.3.0.228 annot-version=v3.0	Micromonas_pusilla
OG0000044
49052 pacid=27343577 transcript=49052
locus=MicpuC2.estExt_fgenesh1_pg.C_160025 ID=49052.3.0.228 annot-
version=v3.0	Micromonas_pusilla	OG0000044
23273 pacid=27343664 transcript=23273 locus=MicpuC2.e_gw1.16.267.1
ID=23273.3.0.228 annot-version=v3.0	Micromonas_pusilla
OG0000044
53932 pacid=27343673 transcript=53932 locus=MicpuC2.EuGene.
0000160208 ID=53932.3.0.228 annot-version=v3.0
Micromonas_pusilla	OG0000044
19323 pacid=27339170 transcript=19323 locus=MicpuC2.e_gw1.8.209.1
ID=19323.3.0.228 annot-version=v3.0	Micromonas_pusilla
OG0000044
60762 pacid=27344108 transcript=60762 locus=MicpuC2.EuGene.
0000090313 ID=60762.3.0.228 annot-version=v3.0
Micromonas_pusilla	OG0000044
159028 pacid=27339224 transcript=159068 locus=e_gw1.8.298.1
ID=159068.3.0.228 annot-version=v3.0	Micromonas_pusilla
OG0000044
10326 pacid=27344506 transcript=10326 locus=MicpuC2.gw1.9.587.1
ID=10326.3.0.228 annot-version=v3.0	Micromonas_pusilla
OG0000044
54194 pacid=27344734 transcript=54194 locus=MicpuC2.EuGene.
0000170040 ID=54194.3.0.228 annot-version=v3.0
Micromonas_pusilla	OG0000044
165393 pacid=27344784 transcript=165433 locus=e_gw1.17.103.1
ID=165433.3.0.228 annot-version=v3.0	Micromonas_pusilla
OG0000044
182886 pacid=27345124 transcript=182926 locus=gm1.7_g

ID=182926.3.0.228 annot-version=v3.0   Micromonas_pusilla
OG0000044
50439 pacid=27345156 transcript=50439 locus=MicpuC2.EuGene.
0000010663 ID=50439.3.0.228 annot-version=v3.0
Micromonas_pusilla   OG0000044
148730 pacid=27345203 transcript=148770 locus=e_gw1.1.590.1
ID=148770.3.0.228 annot-version=v3.0   Micromonas_pusilla
OG0000044
50339 pacid=27345327 transcript=50339 locus=MicpuC2.EuGene.
0000010563 ID=50339.3.0.228 annot-version=v3.0
Micromonas_pusilla   OG0000044
50447 pacid=27345430 transcript=50447 locus=MicpuC2.EuGene.
0000010671 ID=50447.3.0.228 annot-version=v3.0
Micromonas_pusilla   OG0000044
50593 pacid=27345488 transcript=50593 locus=MicpuC2.EuGene.
0000010817 ID=50593.3.0.228 annot-version=v3.0
Micromonas_pusilla   OG0000044
50422 pacid=27345560 transcript=50422 locus=MicpuC2.EuGene.
0000010646 ID=50422.3.0.228 annot-version=v3.0
Micromonas_pusilla   OG0000044
13333 pacid=27345838 transcript=13333 locus=MicpuC2.e_gw1.1.855.1
ID=13333.3.0.228 annot-version=v3.0   Micromonas_pusilla
OG0000044
183517 pacid=27345856 transcript=183557 locus=gm1.638_g
ID=183557.3.0.228 annot-version=v3.0   Micromonas_pusilla
OG0000044
7651 pacid=27339364 transcript=7651 locus=MicpuC2.gw1.11.326.1
ID=7651.3.0.228 annot-version=v3.0   Micromonas_pusilla
OG0000044
183472 pacid=27346015 transcript=183512 locus=gm1.593_g
ID=183512.3.0.228 annot-version=v3.0   Micromonas_pusilla
OG0000044
127555 pacid=27339393 transcript=127595
locus=estExt_fgenesh2_kg.C_110028 ID=127595.3.0.228 annot-
version=v3.0   Micromonas_pusilla   OG0000044
57097 pacid=27346189 transcript=57097 locus=MicpuC2.EuGene.
0000040223 ID=57097.3.0.228 annot-version=v3.0
Micromonas_pusilla   OG0000044
39219 pacid=27346229 transcript=39219
locus=MicpuC2.fgenesh1_pg.C_scaffold_4000126 ID=39219.3.0.228 annot-
version=v3.0   Micromonas_pusilla   OG0000044
51924 pacid=27339423 transcript=51924 locus=MicpuC2.EuGene.
0000110446 ID=51924.3.0.228 annot-version=v3.0
Micromonas_pusilla   OG0000044
57085 pacid=27346515 transcript=57085 locus=MicpuC2.EuGene.
0000040211 ID=57085.3.0.228 annot-version=v3.0
Micromonas_pusilla   OG0000044
57668 pacid=27346536 transcript=57668 locus=MicpuC2.EuGene.
0000040794 ID=57668.3.0.228 annot-version=v3.0
Micromonas_pusilla   OG0000044
7727 pacid=27346589 transcript=7727 locus=MicpuC2.gw1.4.661.1
ID=7727.3.0.228 annot-version=v3.0   Micromonas_pusilla
OG0000044
39537 pacid=27346732 transcript=39537

locus=MicpuC2.fgenesh1_pg.C_scaffold_4000444 ID=39537.3.0.228 annot-version=v3.0   Micromonas_pusilla   OG0000044
51856 pacid=27339484 transcript=51856 locus=MicpuC2.EuGene.0000110378 ID=51856.3.0.228 annot-version=v3.0
Micromonas_pusilla   OG0000044
56845 pacid=27347206 transcript=56845 locus=MicpuC2.EuGene.0000030999 ID=56845.3.0.228 annot-version=v3.0
Micromonas_pusilla   OG0000044
184470 pacid=27347239 transcript=184510 locus=gm1.1591_g
ID=184510.3.0.228 annot-version=v3.0   Micromonas_pusilla
OG0000044
56576 pacid=27347282 transcript=56576 locus=MicpuC2.EuGene.0000030730 ID=56576.3.0.228 annot-version=v3.0
Micromonas_pusilla   OG0000044
56386 pacid=27347358 transcript=56386 locus=MicpuC2.EuGene.0000030540 ID=56386.3.0.228 annot-version=v3.0
Micromonas_pusilla   OG0000044
15076 pacid=27347655 transcript=15076 locus=MicpuC2.e_gw1.3.805.1
ID=15076.3.0.228 annot-version=v3.0   Micromonas_pusilla
OG0000044
56551 pacid=27347751 transcript=56551 locus=MicpuC2.EuGene.0000030705 ID=56551.3.0.228 annot-version=v3.0
Micromonas_pusilla   OG0000044
59836 pacid=27338734 transcript=59836 locus=MicpuC2.EuGene.0000080030 ID=59836.3.0.228 annot-version=v3.0
Micromonas_pusilla   OG0000044
194303 pacid=27338736 transcript=194343
locus=estExt_Genemark1.C_8_t10029 ID=194343.3.0.228 annot-version=v3.0   Micromonas_pusilla   OG0000044
40143 pacid=27348321 transcript=40143
locus=MicpuC2.fgenesh1_pg.C_scaffold_6000120 ID=40143.3.0.228 annot-version=v3.0   Micromonas_pusilla   OG0000044
80272 pacid=27398411 transcript=80272 locus=e_gw2.03.662.1
ID=80272.3.0.229 annot-version=v3.0   Micromonas_sp._RCC299
OG0000044
109352 pacid=27402964 transcript=109352
locus=est_cluster_kg.Chr_13__35__3202471:1 ID=109352.3.0.229 annot-version=v3.0   Micromonas_sp._RCC299   OG0000044
103635 pacid=27402973 transcript=103635
locus=fgenesh2_pg.C_Chr_13000008 ID=103635.3.0.229 annot-version=v3.0   Micromonas_sp._RCC299   OG0000044
kfl00331_0070_v1.1   Klebsormidium_flaccidum OG0000044
kfl00333_0010_v1.1   Klebsormidium_flaccidum OG0000044
kfl00446_0070_v1.1   Klebsormidium_flaccidum OG0000044
kfl00557_0010_v1.1   Klebsormidium_flaccidum OG0000044
kfl00587_0040_v1.1   Klebsormidium_flaccidum OG0000044
kfl00620_0040_v1.1   Klebsormidium_flaccidum OG0000044
kfl00719_0040_v1.1   Klebsormidium_flaccidum OG0000044
kfl00784_0040_v1.1   Klebsormidium_flaccidum OG0000044
kfl00885_0040_v1.1   Klebsormidium_flaccidum OG0000044
kfl00908_0010_v1.1   Klebsormidium_flaccidum OG0000044
kfl00942_0010_v1.1   Klebsormidium_flaccidum OG0000044
kfl00942_0020_v1.1   Klebsormidium_flaccidum OG0000044
kfl01060_0020_v1.1   Klebsormidium_flaccidum OG0000044

kfl01163_0020_v1.1	Klebsormidium_flaccidum	OG0000044
kfl01207_0020_v1.1	Klebsormidium_flaccidum	OG0000044
kfl01275_0030_v1.1	Klebsormidium_flaccidum	OG0000044
kfl01446_0020_v1.1	Klebsormidium_flaccidum	OG0000044
kfl00026_0020_v1.1	Klebsormidium_flaccidum	OG0000044
kfl00002_0800_v1.1	Klebsormidium_flaccidum	OG0000044
kfl00054_0120_v1.1	Klebsormidium_flaccidum	OG0000044
kfl00087_0210_v1.1	Klebsormidium_flaccidum	OG0000044
kfl00106_g25_v1.1	Klebsormidium_flaccidum	OG0000044
kfl00125_0110_v1.1	Klebsormidium_flaccidum	OG0000044
kfl00008_0280_v1.1	Klebsormidium_flaccidum	OG0000044
kfl00008_0550_v1.1	Klebsormidium_flaccidum	OG0000044
kfl00162_0150_v1.1	Klebsormidium_flaccidum	OG0000044
kfl00176_0050_v1.1	Klebsormidium_flaccidum	OG0000044
kfl00248_0090_v1.1	Klebsormidium_flaccidum	OG0000044
kfl00276_0070_v1.1	Klebsormidium_flaccidum	OG0000044
kfl00325_0210_v1.1	Klebsormidium_flaccidum	OG0000044
Pp3c16_22820V3.1.p pacid=32985261 transcript=Pp3c16_22820V3.1 locus=Pp3c16_22820 annot-version=v3.3	Physcomitrella_patens	OG0000044
AT1G70590.1 | F-box family protein | Chr1:26618403-26620159 FORWARD LENGTH=351 | 201606	Arabidopsis_thaliana	OG0000044
Os04t0476000-01 Tetratricopeptide-like helical domain containing protein.	Oryza_sativa	OG0000044
CMB078CT pep chromosome:ASM9120v1:2:214283:215867:-1 gene:CMB078C transcript:CMB078CT gene_biotype:protein_coding transcript_biotype:protein_coding Cyanidioschyzon_merolae	OG0000044
evm.model.contig_2432.1	Porphyridium_purpureum	OG0000044
evm.model.contig_3411.1	Porphyridium_purpureum	OG0000044
evm.model.contig_4400.16	Porphyridium_purpureum	OG0000044
evm.model.contig_4401.8	Porphyridium_purpureum	OG0000044
evm.model.contig_4401.6	Porphyridium_purpureum	OG0000044
evm.model.contig_4401.3	Porphyridium_purpureum	OG0000044
evm.model.contig_4417.3	Porphyridium_purpureum	OG0000044
evm.model.contig_4417.4	Porphyridium_purpureum	OG0000044
evm.model.contig_4437.3	Porphyridium_purpureum	OG0000044
evm.model.contig_2046.1	Porphyridium_purpureum	OG0000044
evm.model.contig_2046.2	Porphyridium_purpureum	OG0000044
evm.model.contig_4450.8	Porphyridium_purpureum	OG0000044
evm.model.contig_513.1	Porphyridium_purpureum	OG0000044
evm.model.contig_544.6	Porphyridium_purpureum	OG0000044
evm.model.contig_544.5	Porphyridium_purpureum	OG0000044
CDF40677 pep supercontig:ASM35022v2:HG002227:438138:440509:1 gene:CHC_T00008659001 transcript:CDF40677 gene_biotype:protein_coding transcript_biotype:protein_coding description:Sel1-repeat containing protein	Chondrus_crispus	OG0000044
CDF39714 pep supercontig:ASM35022v2:HG002070:141071:143105:-1 gene:CHC_T00008468001 transcript:CDF39714 gene_biotype:protein_coding transcript_biotype:protein_coding description:Sel1-repeat containing protein	Chondrus_crispus	OG0000044
CDF40958 pep supercontig:ASM35022v2:HG002285:75949:79273:1

gene:CHC_T00008398001 transcript:CDF40958
gene_biotype:protein_coding transcript_biotype:protein_coding
description:Sel1-repeat containing protein Chondrus_crispus
OG0000044
CDF40950 pep supercontig:ASM35022v2:HG002285:23961:27276:-1
gene:CHC_T00008999001 transcript:CDF40950
gene_biotype:protein_coding transcript_biotype:protein_coding
description:Sel1-repeat containing protein Chondrus_crispus
OG0000044
CDF40948 pep supercontig:ASM35022v2:HG002285:16885:20487:-1
gene:CHC_T00008282001 transcript:CDF40948
gene_biotype:protein_coding transcript_biotype:protein_coding
description:Sel1-repeat containing protein Chondrus_crispus
OG0000044
CDF37937 pep supercontig:ASM35022v2:HG001885:95462:96991:-1
gene:CHC_T00009156001 transcript:CDF37937
gene_biotype:protein_coding transcript_biotype:protein_coding
description:Sel1-repeat containing protein Chondrus_crispus
OG0000044
CDF37936 pep supercontig:ASM35022v2:HG001885:20777:22395:-1
gene:CHC_T00008957001 transcript:CDF37936
gene_biotype:protein_coding transcript_biotype:protein_coding
description:Sel1-repeat containing protein Chondrus_crispus
OG0000044
CDF34233 pep supercontig:ASM35022v2:HG001679:30226:35207:1
gene:CHC_T00008688001 transcript:CDF34233
gene_biotype:protein_coding transcript_biotype:protein_coding
description:Sel1-repeat containing protein Chondrus_crispus
OG0000044
CDF35963 pep supercontig:ASM35022v2:HG001753:132158:136120:1
gene:CHC_T00008679001 transcript:CDF35963
gene_biotype:protein_coding transcript_biotype:protein_coding
description:Sel1-repeat containing protein Chondrus_crispus
OG0000044
CDF35959 pep supercontig:ASM35022v2:HG001753:33996:36473:1
gene:CHC_T00009232001 transcript:CDF35959
gene_biotype:protein_coding transcript_biotype:protein_coding
description:Sel1-repeat containing protein Chondrus_crispus
OG0000044
CDF37955 pep supercontig:ASM35022v2:HG001890:6658:9747:1
gene:CHC_T00009085001 transcript:CDF37955
gene_biotype:protein_coding transcript_biotype:protein_coding
description:Sel1-repeat containing protein Chondrus_crispus
OG0000044
CDF33623 pep supercontig:ASM35022v2:HG001651:162890:166992:1
gene:CHC_T00008860001 transcript:CDF33623
gene_biotype:protein_coding transcript_biotype:protein_coding
description:Sel1-repeat containing protein Chondrus_crispus
OG0000044
CDF36669 pep supercontig:ASM35022v2:HG001795:111759:113616:1
gene:CHC_T00008382001 transcript:CDF36669
gene_biotype:protein_coding transcript_biotype:protein_coding
description:Sel1-repeat containing protein Chondrus_crispus
OG0000044

CDF37825 pep supercontig:ASM35022v2:HG001873:80064:81023:-1 gene:CHC_T00006014001 transcript:CDF37825 gene_biotype:protein_coding transcript_biotype:protein_coding Chondrus_crispus OG0000044
CDF37826 pep supercontig:ASM35022v2:HG001873:99289:100590:-1 gene:CHC_T00008960001 transcript:CDF37826 gene_biotype:protein_coding transcript_biotype:protein_coding description:Sel1-repeat containing protein Chondrus_crispus OG0000044
CDF40066 pep supercontig:ASM35022v2:HG002119:27285:28244:-1 gene:CHC_T00000645001 transcript:CDF40066 gene_biotype:protein_coding transcript_biotype:protein_coding Chondrus_crispus OG0000044
CDF33057 pep supercontig:ASM35022v2:HG001632:100200:104018:-1 gene:CHC_T00008933001 transcript:CDF33057 gene_biotype:protein_coding transcript_biotype:protein_coding description:Sel1-repeat containing protein Chondrus_crispus OG0000044
CDF32355 pep supercontig:ASM35022v2:HG001477:207063:211453:-1 gene:CHC_T00008087001 transcript:CDF32355 gene_biotype:protein_coding transcript_biotype:protein_coding Chondrus_crispus OG0000044
CDF39980 pep supercontig:ASM35022v2:HG002095:27603:31647:-1 gene:CHC_T00009371001 transcript:CDF39980 gene_biotype:protein_coding transcript_biotype:protein_coding description:Sel1-repeat containing protein Chondrus_crispus OG0000044
CDF33647 pep supercontig:ASM35022v2:HG001652:184731:186459:1 gene:CHC_T00008352001 transcript:CDF33647 gene_biotype:protein_coding transcript_biotype:protein_coding description:Sel1-repeat containing protein Chondrus_crispus OG0000044
CDF39968 pep supercontig:ASM35022v2:HG002094:339288:340833:1 gene:CHC_T00009291001 transcript:CDF39968 gene_biotype:protein_coding transcript_biotype:protein_coding description:Sel1-repeat containing protein Chondrus_crispus OG0000044
CDF34918 pep supercontig:ASM35022v2:HG001710:55536:58292:-1 gene:CHC_T00008859001 transcript:CDF34918 gene_biotype:protein_coding transcript_biotype:protein_coding description:Sel1-repeat containing protein Chondrus_crispus OG0000044
CDF38170 pep supercontig:ASM35022v2:HG001899:8926:11394:1 gene:CHC_T00009271001 transcript:CDF38170 gene_biotype:protein_coding transcript_biotype:protein_coding description:Sel1-repeat containing protein Chondrus_crispus OG0000044
CDF40252 pep supercontig:ASM35022v2:HG002147:330641:332773:-1 gene:CHC_T00009334001 transcript:CDF40252 gene_biotype:protein_coding transcript_biotype:protein_coding description:Sel1-repeat containing protein Chondrus_crispus OG0000044
CDF32314 pep supercontig:ASM35022v2:HG001472:2286:3584:-1 gene:CHC_T00009151001 transcript:CDF32314

gene_biotype:protein_coding transcript_biotype:protein_coding description:Sel1-repeat containing protein Chondrus_crispus OG0000044
CDF38491 pep supercontig:ASM35022v2:HG001939:6969:12969:1 gene:CHC_T00008953001 transcript:CDF38491 gene_biotype:protein_coding transcript_biotype:protein_coding description:Sel1-repeat containing protein Chondrus_crispus OG0000044
g1350.t1 Caulerpa_lentillifera OG0000045
g1704.t1 Caulerpa_lentillifera OG0000045
g2888.t1 Caulerpa_lentillifera OG0000045
g4138.t1 Caulerpa_lentillifera OG0000045
g4856.t1 Caulerpa_lentillifera OG0000045
g6500.t1 Caulerpa_lentillifera OG0000045
g7588.t1 Caulerpa_lentillifera OG0000045
g7956.t1 Caulerpa_lentillifera OG0000045
g8097.t1 Caulerpa_lentillifera OG0000045
g8207.t1 Caulerpa_lentillifera OG0000045
g8941.t1 Caulerpa_lentillifera OG0000045
Cre02.g078966.t1.1 pacid=30784942 transcript=Cre02.g078966.t1.1 locus=Cre02.g078966 ID=Cre02.g078966.t1.1.v5.5 annot-version=v5.5 Chlamydomonas_reinhardtii OG0000045
Cre07.g352350.t1.2 pacid=30774541 transcript=Cre07.g352350.t1.2 locus=Cre07.g352350 ID=Cre07.g352350.t1.2.v5.5 annot-version=v5.5 Chlamydomonas_reinhardtii OG0000045
Cre03.g201100.t1.2 pacid=30787050 transcript=Cre03.g201100.t1.2 locus=Cre03.g201100 ID=Cre03.g201100.t1.2.v5.5 annot-version=v5.5 Chlamydomonas_reinhardtii OG0000045
Cre01.g019850.t1.1 pacid=30788646 transcript=Cre01.g019850.t1.1 locus=Cre01.g019850 ID=Cre01.g019850.t1.1.v5.5 annot-version=v5.5 Chlamydomonas_reinhardtii OG0000045
Cre12.g485800.t1.2 pacid=30791981 transcript=Cre12.g485800.t1.2 locus=Cre12.g485800 ID=Cre12.g485800.t1.2.v5.5 annot-version=v5.5 Chlamydomonas_reinhardtii OG0000045
Cre14.g625625.t1.1 pacid=30776209 transcript=Cre14.g625625.t1.1 locus=Cre14.g625625 ID=Cre14.g625625.t1.1.v5.5 annot-version=v5.5 Chlamydomonas_reinhardtii OG0000045
Cre09.g393950.t1.2 pacid=30780473 transcript=Cre09.g393950.t1.2 locus=Cre09.g393950 ID=Cre09.g393950.t1.2.v5.5 annot-version=v5.5 Chlamydomonas_reinhardtii OG0000045
Cre17.g720050.t1.2 pacid=30782442 transcript=Cre17.g720050.t1.2 locus=Cre17.g720050 ID=Cre17.g720050.t1.2.v5.5 annot-version=v5.5 Chlamydomonas_reinhardtii OG0000045
Cre17.g739752.t1.1 pacid=30782539 transcript=Cre17.g739752.t1.1 locus=Cre17.g739752 ID=Cre17.g739752.t1.1.v5.5 annot-version=v5.5 Chlamydomonas_reinhardtii OG0000045
Cre13.g568400.t1.2 pacid=30784443 transcript=Cre13.g568400.t1.2 locus=Cre13.g568400 ID=Cre13.g568400.t1.2.v5.5 annot-version=v5.5 Chlamydomonas_reinhardtii OG0000045
Vocar.0028s0163.1.p pacid=32895585 transcript=Vocar.0028s0163.1 locus=Vocar.0028s0163 ID=Vocar.0028s0163.1.v2.1 annot-version=v2.1 Volvox_carteri OG0000045
Vocar.0013s0271.1.p pacid=32896473 transcript=Vocar.0013s0271.1 locus=Vocar.0013s0271 ID=Vocar.0013s0271.1.v2.1 annot-version=v2.1

Volvox_carteri   OG0000045
Vocar.0035s0016.1.p pacid=32896860 transcript=Vocar.0035s0016.1
locus=Vocar.0035s0016 ID=Vocar.0035s0016.1.v2.1 annot-version=v2.1
Volvox_carteri   OG0000045
Vocar.0016s0146.1.p pacid=32898067 transcript=Vocar.0016s0146.1
locus=Vocar.0016s0146 ID=Vocar.0016s0146.1.v2.1 annot-version=v2.1
Volvox_carteri   OG0000045
Vocar.0007s0464.1.p pacid=32888313 transcript=Vocar.0007s0464.1
locus=Vocar.0007s0464 ID=Vocar.0007s0464.1.v2.1 annot-version=v2.1
Volvox_carteri   OG0000045
Vocar.0004s0344.1.p pacid=32889652 transcript=Vocar.0004s0344.1
locus=Vocar.0004s0344 ID=Vocar.0004s0344.1.v2.1 annot-version=v2.1
Volvox_carteri   OG0000045
Vocar.0004s0172.1.p pacid=32889955 transcript=Vocar.0004s0172.1
locus=Vocar.0004s0172 ID=Vocar.0004s0172.1.v2.1 annot-version=v2.1
Volvox_carteri   OG0000045
Vocar.0022s0198.1.p pacid=32891764 transcript=Vocar.0022s0198.1
locus=Vocar.0022s0198 ID=Vocar.0022s0198.1.v2.1 annot-version=v2.1
Volvox_carteri   OG0000045
Vocar.0005s0095.1.p pacid=32884072 transcript=Vocar.0005s0095.1
locus=Vocar.0005s0095 ID=Vocar.0005s0095.1.v2.1 annot-version=v2.1
Volvox_carteri   OG0000045
Vocar.0001s1639.1.p pacid=32884324 transcript=Vocar.0001s1639.1
locus=Vocar.0001s1639 ID=Vocar.0001s1639.1.v2.1 annot-version=v2.1
Volvox_carteri   OG0000045
jgi|ChlNC64A_1|140544|IGS.gm_3_00283   Chlorella_variabilis
OG0000045
jgi|ChlNC64A_1|37431|estExt_Genewise1Plus.C_300004
Chlorella_variabilis   OG0000045
jgi|ChlNC64A_1|142278|IGS.gm_4_00419   Chlorella_variabilis
OG0000045
jgi|ChlNC64A_1|132927|IGS.gm_1_00063   Chlorella_variabilis
OG0000045
jgi|ChlNC64A_1|33680|estExt_Genewise1Plus.C_10647
Chlorella_variabilis   OG0000045
jgi|ChlNC64A_1|51635|fgenesh3_pg.C_scaffold_7000182
Chlorella_variabilis   OG0000045
jgi|ChlNC64A_1|34770|estExt_Genewise1Plus.C_50498
Chlorella_variabilis   OG0000045
jgi|ChlNC64A_1|141222|IGS.gm_32_00060   Chlorella_variabilis
OG0000045
jgi|ChlNC64A_1|136599|IGS.gm_17_00003   Chlorella_variabilis
OG0000045
61951 pacid=27387182 transcript=61951 locus=Genemark1.2734_g
ID=61951.2.0.227 annot-version=v2.0   Coccomyxa_subellipsoidea
OG0000045
67248 pacid=27387963 transcript=67248
locus=estExt_Genemark1.C_140178 ID=67248.2.0.227 annot-version=v2.0
Coccomyxa_subellipsoidea OG0000045
16265 pacid=27388659 transcript=16265 locus=e_gw1.9.44.1
ID=16265.2.0.227 annot-version=v2.0   Coccomyxa_subellipsoidea
OG0000045
52790 pacid=27389780 transcript=52790
locus=estExt_fgenesh1_pm.C_40085 ID=52790.2.0.227 annot-version=v2.0

Coccomyxa_subellipsoidea OG0000045
22213 pacid=27390421 transcript=22213 locus=estExt_Genewise1.C_30362
ID=22213.2.0.227 annot-version=v2.0 Coccomyxa_subellipsoidea
OG0000045
22106 pacid=27390504 transcript=22106 locus=estExt_Genewise1.C_30186
ID=22106.2.0.227 annot-version=v2.0 Coccomyxa_subellipsoidea
OG0000045
24964 pacid=27386129 transcript=24964
locus=estExt_Genewise1.C_130266 ID=24964.2.0.227 annot-version=v2.0
Coccomyxa_subellipsoidea OG0000045
27699 pacid=27390608 transcript=27699
locus=estExt_Genewise1Plus.C_30615 ID=27699.2.0.227 annot-
version=v2.0 Coccomyxa_subellipsoidea OG0000045
15008 pacid=27391142 transcript=15008 locus=e_gw1.6.68.1
ID=15008.2.0.227 annot-version=v2.0 Coccomyxa_subellipsoidea
OG0000045
12248 pacid=27393166 transcript=12248 locus=e_gw1.2.769.1
ID=12248.2.0.227 annot-version=v2.0 Coccomyxa_subellipsoidea
OG0000045
1880 pacid=27393722 transcript=1880 locus=gw1.2.164.1
ID=1880.2.0.227 annot-version=v2.0 Coccomyxa_subellipsoidea
OG0000045
1818_gw1.10.00.101.1 Ostreococcus_tauri OG0000045
14645_e_gw1.01.00.244.1 Ostreococcus_tauri OG0000045
18762_e_gw1.08.00.68.1 Ostreococcus_tauri OG0000045
20212_e_gw1.11.00.60.1 Ostreococcus_tauri OG0000045
21043_e_gw1.14.00.33.1 Ostreococcus_tauri OG0000045
21801_e_gw1.16.00.45.1 Ostreococcus_tauri OG0000045
22050_e_gw1.17.00.77.1 Ostreococcus_tauri OG0000045
28922_estExt_gwp_GeneWisePlus.C_Chr_09.00010447
Ostreococcus_tauri OG0000045
29060_estExt_gwp_GeneWisePlus.C_Chr_10.00010428
Ostreococcus_tauri OG0000045
785_gw1.10.00.39.1 Ostreococcus_tauri OG0000045
8100_fgenesh1_pm.C_Chr_01.0001000058 Ostreococcus_tauri
OG0000045
37796 pacid=27415768 transcript=37796 locus=e_gwEuk.16.26.1
ID=37796.2.0.231 annot-version=v2.0 Ostreococcus_lucimarinus
OG0000045
26133 pacid=27416090 transcript=26133 locus=eugene.1000010167
ID=26133.2.0.231 annot-version=v2.0 Ostreococcus_lucimarinus
OG0000045
38163 pacid=27416159 transcript=38163 locus=e_gwEuk.10.39.1
ID=38163.2.0.231 annot-version=v2.0 Ostreococcus_lucimarinus
OG0000045
38040 pacid=27416233 transcript=38040 locus=e_gwEuk.10.71.1
ID=38040.2.0.231 annot-version=v2.0 Ostreococcus_lucimarinus
OG0000045
40626 pacid=27417434 transcript=40626 locus=e_gwEuk.15.323.1
ID=40626.2.0.231 annot-version=v2.0 Ostreococcus_lucimarinus
OG0000045
3025 pacid=27417737 transcript=3025 locus=gwEuk.1.254.1
ID=3025.2.0.231 annot-version=v2.0 Ostreococcus_lucimarinus
OG0000045

29118 pacid=27417791 transcript=29118 locus=eugene.0100010512
ID=29118.2.0.231 annot-version=v2.0 Ostreococcus_lucimarinus
OG0000045
42764 pacid=27419246 transcript=42764 locus=e_gwEuk.8.95.1
ID=42764.2.0.231 annot-version=v2.0 Ostreococcus_lucimarinus
OG0000045
13471 pacid=27420293 transcript=13471
locus=fgenesh1_pm.C_Chr_20000034 ID=13471.2.0.231 annot-version=v2.0
Ostreococcus_lucimarinus OG0000045
44098 pacid=27420538 transcript=44098 locus=e_gwEuk.11.470.1
ID=44098.2.0.231 annot-version=v2.0 Ostreococcus_lucimarinus
OG0000045
29396 pacid=27339985 transcript=29396
locus=MicpuC2.estExt_Genewise1.C_130117 ID=29396.3.0.228 annot-
version=v3.0 Micromonas_pusilla OG0000045
28671 pacid=27340386 transcript=28671
locus=MicpuC2.estExt_Genewise1.C_100320 ID=28671.3.0.228 annot-
version=v3.0 Micromonas_pusilla OG0000045
179229 pacid=27340720 transcript=179269
locus=estExt_Genewise1Plus.C_7_t10313 ID=179269.3.0.228 annot-
version=v3.0 Micromonas_pusilla OG0000045
58438 pacid=27341742 transcript=58438 locus=MicpuC2.EuGene.
0000050743 ID=58438.3.0.228 annot-version=v3.0
Micromonas_pusilla OG0000045
203299 pacid=27342775 transcript=203339 locus=fgenesh1_pm.2_#_385
ID=203339.3.0.228 annot-version=v3.0 Micromonas_pusilla
OG0000045
140321 pacid=27343018 transcript=140361 locus=gw1.2.1056.1
ID=140361.3.0.228 annot-version=v3.0 Micromonas_pusilla
OG0000045
23056 pacid=27343580 transcript=23056 locus=MicpuC2.e_gw1.16.23.1
ID=23056.3.0.228 annot-version=v3.0 Micromonas_pusilla
OG0000045
211579 pacid=27345138 transcript=211619
locus=estExt_fgenesh1_pm.C_1_t10428 ID=211619.3.0.228 annot-
version=v3.0 Micromonas_pusilla OG0000045
1627 pacid=27345276 transcript=1627 locus=MicpuC2.gw1.1.165.1
ID=1627.3.0.228 annot-version=v3.0 Micromonas_pusilla
OG0000045
174963 pacid=27345316 transcript=175003
locus=estExt_Genewise1Plus.C_1_t30110 ID=175003.3.0.228 annot-
version=v3.0 Micromonas_pusilla OG0000045
148873 pacid=27345579 transcript=148913 locus=e_gw1.1.964.1
ID=148913.3.0.228 annot-version=v3.0 Micromonas_pusilla
OG0000045
55999 pacid=27346915 transcript=55999 locus=MicpuC2.EuGene.
0000030153 ID=55999.3.0.228 annot-version=v3.0
Micromonas_pusilla OG0000045
95367 pacid=27397348 transcript=95367
locus=estExt_Genewise2Plus.C_Chr_100692 ID=95367.3.0.229 annot-
version=v3.0 Micromonas_sp._RCC299 OG0000045
95257 pacid=27397717 transcript=95257
locus=estExt_Genewise2Plus.C_Chr_100226 ID=95257.3.0.229 annot-
version=v3.0 Micromonas_sp._RCC299 OG0000045

104714 pacid=27399107 transcript=104714
locus=estExt_fgenesh2_kg.C_Chr_010034 ID=104714.3.0.229 annot-version=v3.0 Micromonas_sp._RCC299 OG0000045
87320 pacid=27399562 transcript=87320 locus=e_gw2.12.112.1
ID=87320.3.0.229 annot-version=v3.0 Micromonas_sp._RCC299
OG0000045
92259 pacid=27402789 transcript=92259
locus=estExt_Genewise2.C_Chr_130199 ID=92259.3.0.229 annot-version=v3.0 Micromonas_sp._RCC299 OG0000045
94954 pacid=27403447 transcript=94954
locus=estExt_Genewise2Plus.C_Chr_080671 ID=94954.3.0.229 annot-version=v3.0 Micromonas_sp._RCC299 OG0000045
106506 pacid=27403793 transcript=106506
locus=estExt_fgenesh2_pg.C_Chr_140164 ID=106506.3.0.229 annot-version=v3.0 Micromonas_sp._RCC299 OG0000045
59768 pacid=27404124 transcript=59768 locus=EuGene.0700010053
ID=59768.3.0.229 annot-version=v3.0 Micromonas_sp._RCC299
OG0000045
59979 pacid=27404567 transcript=59979 locus=EuGene.0700010264
ID=59979.3.0.229 annot-version=v3.0 Micromonas_sp._RCC299
OG0000045
83563 pacid=27404601 transcript=83563 locus=e_gw2.07.543.1
ID=83563.3.0.229 annot-version=v3.0 Micromonas_sp._RCC299
OG0000045
59866 pacid=27404729 transcript=59866 locus=EuGene.0700010151
ID=59866.3.0.229 annot-version=v3.0 Micromonas_sp._RCC299
OG0000045
kfl00015_0350_v1.1 Klebsormidium_flaccidum OG0000045
kfl00466_0050_v1.1 Klebsormidium_flaccidum OG0000045
kfl00964_0010_v1.1 Klebsormidium_flaccidum OG0000045
kfl00095_0050_v1.1 Klebsormidium_flaccidum OG0000045
kfl00100_0210_v1.1 Klebsormidium_flaccidum OG0000045
kfl00132_0250_v1.1 Klebsormidium_flaccidum OG0000045
kfl00136_0270_v1.1 Klebsormidium_flaccidum OG0000045
kfl00001_0670_v1.1 Klebsormidium_flaccidum OG0000045
kfl00201_0150_v1.1 Klebsormidium_flaccidum OG0000045
kfl00212_0060_v1.1 Klebsormidium_flaccidum OG0000045
Pp3c22_8480V3.1.p pacid=32903080 transcript=Pp3c22_8480V3.1
locus=Pp3c22_8480 annot-version=v3.3 Physcomitrella_patens
OG0000045
Pp3c2_30820V3.1.p pacid=32936811 transcript=Pp3c2_30820V3.1
locus=Pp3c2_30820 annot-version=v3.3 Physcomitrella_patens
OG0000045
Pp3c19_2770V3.1.p pacid=32938852 transcript=Pp3c19_2770V3.1
locus=Pp3c19_2770 annot-version=v3.3 Physcomitrella_patens
OG0000045
Pp3c20_5760V3.1.p pacid=32945277 transcript=Pp3c20_5760V3.1
locus=Pp3c20_5760 annot-version=v3.3 Physcomitrella_patens
OG0000045
Pp3c20_7080V3.1.p pacid=32947446 transcript=Pp3c20_7080V3.1
locus=Pp3c20_7080 annot-version=v3.3 Physcomitrella_patens
OG0000045
Pp3c20_15780V3.1.p pacid=32947551 transcript=Pp3c20_15780V3.1
locus=Pp3c20_15780 annot-version=v3.3 Physcomitrella_patens

OG0000045
Pp3c23_14660V3.1.p pacid=32948588 transcript=Pp3c23_14660V3.1
locus=Pp3c23_14660 annot-version=v3.3   Physcomitrella_patens
OG0000045
Pp3c23_7140V3.1.p pacid=32949091 transcript=Pp3c23_7140V3.1
locus=Pp3c23_7140 annot-version=v3.3   Physcomitrella_patens
OG0000045
Pp3c23_7144V3.1.p pacid=32950064 transcript=Pp3c23_7144V3.1
locus=Pp3c23_7144 annot-version=v3.3   Physcomitrella_patens
OG0000045
Pp3c23_14665V3.1.p pacid=32950218 transcript=Pp3c23_14665V3.1
locus=Pp3c23_14665 annot-version=v3.3   Physcomitrella_patens
OG0000045
Pp3c23_8400V3.1.p pacid=32950338 transcript=Pp3c23_8400V3.1
locus=Pp3c23_8400 annot-version=v3.3   Physcomitrella_patens
OG0000045
Pp3c5_23480V3.1.p pacid=32954022 transcript=Pp3c5_23480V3.1
locus=Pp3c5_23480 annot-version=v3.3   Physcomitrella_patens
OG0000045
Pp3c11_1240V3.1.p pacid=32956239 transcript=Pp3c11_1240V3.1
locus=Pp3c11_1240 annot-version=v3.3   Physcomitrella_patens
OG0000045
Pp3c1_2390V3.1.p pacid=32967621 transcript=Pp3c1_2390V3.1
locus=Pp3c1_2390 annot-version=v3.3   Physcomitrella_patens
OG0000045
Pp3c12_22830V3.1.p pacid=32974696 transcript=Pp3c12_22830V3.1
locus=Pp3c12_22830 annot-version=v3.3   Physcomitrella_patens
OG0000045
Pp3c6_6210V3.1.p pacid=32977841 transcript=Pp3c6_6210V3.1
locus=Pp3c6_6210 annot-version=v3.3   Physcomitrella_patens
OG0000045
Pp3c17_20370V3.1.p pacid=32908267 transcript=Pp3c17_20370V3.1
locus=Pp3c17_20370 annot-version=v3.3   Physcomitrella_patens
OG0000045
Pp3c24_15420V3.1.p pacid=32910847 transcript=Pp3c24_15420V3.1
locus=Pp3c24_15420 annot-version=v3.3   Physcomitrella_patens
OG0000045
Pp3c4_8640V3.1.p pacid=32919087 transcript=Pp3c4_8640V3.1
locus=Pp3c4_8640 annot-version=v3.3   Physcomitrella_patens
OG0000045
Pp3c4_20860V3.1.p pacid=32919804 transcript=Pp3c4_20860V3.1
locus=Pp3c4_20860 annot-version=v3.3   Physcomitrella_patens
OG0000045
AT3G02450.1 | cell division protein ftsH | Chr3:502876-505030
REVERSE LENGTH=622 | 201606   Arabidopsis_thaliana
OG0000045
AT3G16290.1 | AAA-type ATPase family protein | Chr3:5521187-5524995
REVERSE LENGTH=876 | 201606   Arabidopsis_thaliana
OG0000045
AT3G47060.1 | FTSH protease 7 | Chr3:17332999-17336613 FORWARD
LENGTH=802 | 201606   Arabidopsis_thaliana   OG0000045
AT4G23940.1 | FtsH extracellular protease family |
Chr4:12437108-12441841 FORWARD LENGTH=946 | 201606
Arabidopsis_thaliana   OG0000045

AT5G15250.1 | FTSH protease 6 | Chr5:4950411-4952777 REVERSE LENGTH=688 | 201606	Arabidopsis_thaliana	OG0000045
AT5G42270.1 | FtsH extracellular protease family | Chr5:16902659-16905102 FORWARD LENGTH=704 | 201606	Arabidopsis_thaliana	OG0000045
AT5G53170.1 | FTSH protease 11 | Chr5:21563023-21567922 REVERSE LENGTH=806 | 201606	Arabidopsis_thaliana	OG0000045
AT5G58870.1 | FTSH protease 9 | Chr5:23770080-23773719 REVERSE LENGTH=806 | 201606	Arabidopsis_thaliana	OG0000045
AT5G64580.1 | AAA-type ATPase family protein | Chr5:25817391-25821465 REVERSE LENGTH=855 | 201606	Arabidopsis_thaliana	OG0000045
AT1G50250.1 | FTSH protease 1 | Chr1:18614398-18616930 REVERSE LENGTH=716 | 201606	Arabidopsis_thaliana	OG0000045
AT1G06430.1 | FTSH protease 8 | Chr1:1960214-1962525 REVERSE LENGTH=685 | 201606	Arabidopsis_thaliana	OG0000045
AT1G07510.1 | FTSH protease 10 | Chr1:2305689-2309380 FORWARD LENGTH=813 | 201606	Arabidopsis_thaliana	OG0000045
AT2G26140.1 | FTSH protease 4 | Chr2:11131939-11135126 REVERSE LENGTH=717 | 201606	Arabidopsis_thaliana	OG0000045
AT2G29080.1 | FTSH protease 3 | Chr2:12489911-12492999 REVERSE LENGTH=809 | 201606	Arabidopsis_thaliana	OG0000045
AT2G30950.1 | FtsH extracellular protease family | Chr2:13174692-13177064 FORWARD LENGTH=695 | 201606	Arabidopsis_thaliana	OG0000045
Os04t0466100-01 Similar to Cell division protein FtsH-like protein.	Oryza_sativa	OG0000045
Os05t0458400-01 Similar to AAA-metalloprotease FtsH.	Oryza_sativa	OG0000045
Os06t0229066-00 Similar to ATP-dependent zinc metalloprotease FTSH 6, chloroplastic.	Oryza_sativa	OG0000045
Os01t0574400-01 Similar to Cell division protein ftsH (EC 3.4.24.-).	Oryza_sativa	OG0000045
Os01t0574500-01 ATPase, AAA-type, core domain containing protein.	Oryza_sativa	OG0000045
Os06t0669400-01 Similar to Cell division protease ftsH homolog 2, chloroplastic.	Oryza_sativa	OG0000045
Os06t0725900-01 Similar to Cell division protein ftsH homolog, chloroplast precursor (EC 3.4.24.-) (DS9).	Oryza_sativa	OG0000045
Os01t0618800-01 Similar to ATP-dependent zinc metalloprotease FTSH 9, chloroplastic/mitochondrial.	Oryza_sativa	OG0000045
Os01t0842600-01 Similar to AAA-metalloprotease FtsH.	Oryza_sativa	OG0000045
Os02t0649700-01 ATPase, AAA-type, core domain containing protein.	Oryza_sativa	OG0000045
CMQ295CT pep chromosome:ASM9120v1:17:763794:766125:-1 gene:CMQ295C transcript:CMQ295CT gene_biotype:protein_coding transcript_biotype:protein_coding Cyanidioschyzon_merolae	OG0000045
CMI157CT pep chromosome:ASM9120v1:9:428710:431016:1 gene:CMI157C transcript:CMI157CT gene_biotype:protein_coding transcript_biotype:protein_coding description:FTSH protease 4 [Source:Projected from Arabidopsis thaliana (AT2G26140)

TAIR;Acc:AT2G26140]	Cyanidioschyzon_merolae	OG0000045
CMF159CT pep chromosome:ASM9120v1:6:440795:443332:-1 gene:CMF159C transcript:CMF159CT gene_biotype:protein_coding transcript_biotype:protein_coding Cyanidioschyzon_merolae OG0000045
CME055CT pep chromosome:ASM9120v1:5:157117:159879:1 gene:CME055C transcript:CME055CT gene_biotype:protein_coding transcript_biotype:protein_coding description:FtsH2 [Source:UniProtKB/TrEMBL;Acc:Q9SLX5]	Cyanidioschyzon_merolae OG0000045
evm.model.contig_4437.1	Porphyridium_purpureum	OG0000045
evm.model.contig_503.12	Porphyridium_purpureum	OG0000045
evm.model.contig_522.1	Porphyridium_purpureum	OG0000045
evm.model.contig_547.1	Porphyridium_purpureum	OG0000045
evm.model.contig_2059.27	Porphyridium_purpureum	OG0000045
CDF34632 pep supercontig:ASM35022v2:HG001703:132677:134644:-1 gene:CHC_T00009342001 transcript:CDF34632 gene_biotype:protein_coding transcript_biotype:protein_coding description:Partial ftsH2	Chondrus_crispus	OG0000045
CDF33542 pep supercontig:ASM35022v2:HG001649:44805:46940:1 gene:CHC_T00009248001 transcript:CDF33542 gene_biotype:protein_coding transcript_biotype:protein_coding description:cell division protein FtsH	Chondrus_crispus OG0000045
CDF33172 pep supercontig:ASM35022v2:HG001635:186624:190250:1 gene:CHC_T00009243001 transcript:CDF33172 gene_biotype:protein_coding transcript_biotype:protein_coding description:ATP-dependent Zn protease FtsH2	Chondrus_crispus OG0000045
CDF40438 pep supercontig:ASM35022v2:HG002179:8443:11333:-1 gene:CHC_T00008637001 transcript:CDF40438 gene_biotype:protein_coding transcript_biotype:protein_coding description:ATP-dependent zinc protease	Chondrus_crispus OG0000045
g3958.t1	Caulerpa_lentillifera	OG0000046
g5525.t1	Caulerpa_lentillifera	OG0000046
g6254.t1	Caulerpa_lentillifera	OG0000046
g7529.t1	Caulerpa_lentillifera	OG0000046
g990.t1	Caulerpa_lentillifera	OG0000046
Cre02.g095125.t1.1 pacid=30785843 transcript=Cre02.g095125.t1.1 locus=Cre02.g095125 ID=Cre02.g095125.t1.1.v5.5 annot-version=v5.5 Chlamydomonas_reinhardtii	OG0000046
Cre01.g025900.t1.2 pacid=30789384 transcript=Cre01.g025900.t1.2 locus=Cre01.g025900 ID=Cre01.g025900.t1.2.v5.5 annot-version=v5.5 Chlamydomonas_reinhardtii	OG0000046
Cre12.g528350.t1.2 pacid=30791846 transcript=Cre12.g528350.t1.2 locus=Cre12.g528350 ID=Cre12.g528350.t1.2.v5.5 annot-version=v5.5 Chlamydomonas_reinhardtii	OG0000046
Cre16.g663876.t1.1 pacid=30777394 transcript=Cre16.g663876.t1.1 locus=Cre16.g663876 ID=Cre16.g663876.t1.1.v5.5 annot-version=v5.5 Chlamydomonas_reinhardtii	OG0000046
Cre16.g691050.t1.2 pacid=30778183 transcript=Cre16.g691050.t1.2 locus=Cre16.g691050 ID=Cre16.g691050.t1.2.v5.5 annot-version=v5.5 Chlamydomonas_reinhardtii	OG0000046

Cre08.g359200.t1.1 pacid=30773926 transcript=Cre08.g359200.t1.1 locus=Cre08.g359200 ID=Cre08.g359200.t1.1.v5.5 annot-version=v5.5 Chlamydomonas_reinhardtii OG0000046
Cre17.g709651.t1.1 pacid=30782615 transcript=Cre17.g709651.t1.1 locus=Cre17.g709651 ID=Cre17.g709651.t1.1.v5.5 annot-version=v5.5 Chlamydomonas_reinhardtii OG0000046
Cre05.g243353.t1.2 pacid=30782974 transcript=Cre05.g243353.t1.2 locus=Cre05.g243353 ID=Cre05.g243353.t1.2.v5.5 annot-version=v5.5 Chlamydomonas_reinhardtii OG0000046
Cre05.g230600.t1.1 pacid=30783444 transcript=Cre05.g230600.t1.1 locus=Cre05.g230600 ID=Cre05.g230600.t1.1.v5.5 annot-version=v5.5 Chlamydomonas_reinhardtii OG0000046
Vocar.0012s0305.1.p pacid=32894862 transcript=Vocar.0012s0305.1 locus=Vocar.0012s0305 ID=Vocar.0012s0305.1.v2.1 annot-version=v2.1 Volvox_carteri OG0000046
Vocar.0012s0307.1.p pacid=32894905 transcript=Vocar.0012s0307.1 locus=Vocar.0012s0307 ID=Vocar.0012s0307.1.v2.1 annot-version=v2.1 Volvox_carteri OG0000046
Vocar.0075s0004.1.p pacid=32895804 transcript=Vocar.0075s0004.1 locus=Vocar.0075s0004 ID=Vocar.0075s0004.1.v2.1 annot-version=v2.1 Volvox_carteri OG0000046
Vocar.0021s0048.1.p pacid=32895914 transcript=Vocar.0021s0048.1 locus=Vocar.0021s0048 ID=Vocar.0021s0048.1.v2.1 annot-version=v2.1 Volvox_carteri OG0000046
Vocar.0037s0041.1.p pacid=32897545 transcript=Vocar.0037s0041.1 locus=Vocar.0037s0041 ID=Vocar.0037s0041.1.v2.1 annot-version=v2.1 Volvox_carteri OG0000046
Vocar.0142s0003.1.p pacid=32899356 transcript=Vocar.0142s0003.1 locus=Vocar.0142s0003 ID=Vocar.0142s0003.1.v2.1 annot-version=v2.1 Volvox_carteri OG0000046
Vocar.0142s0002.1.p pacid=32899357 transcript=Vocar.0142s0002.1 locus=Vocar.0142s0002 ID=Vocar.0142s0002.1.v2.1 annot-version=v2.1 Volvox_carteri OG0000046
Vocar.0001s0860.1.p pacid=32886041 transcript=Vocar.0001s0860.1 locus=Vocar.0001s0860 ID=Vocar.0001s0860.1.v2.1 annot-version=v2.1 Volvox_carteri OG0000046
Vocar.0017s0213.1.p pacid=32891365 transcript=Vocar.0017s0213.1 locus=Vocar.0017s0213 ID=Vocar.0017s0213.1.v2.1 annot-version=v2.1 Volvox_carteri OG0000046
Vocar.0078s0015.1.p pacid=32891714 transcript=Vocar.0078s0015.1 locus=Vocar.0078s0015 ID=Vocar.0078s0015.1.v2.1 annot-version=v2.1 Volvox_carteri OG0000046
Vocar.0032s0155.1.p pacid=32893767 transcript=Vocar.0032s0155.1 locus=Vocar.0032s0155 ID=Vocar.0032s0155.1.v2.1 annot-version=v2.1 Volvox_carteri OG0000046
Vocar.0032s0154.1.p pacid=32893780 transcript=Vocar.0032s0154.1 locus=Vocar.0032s0154 ID=Vocar.0032s0154.1.v2.1 annot-version=v2.1 Volvox_carteri OG0000046
Vocar.0032s0159.1.p pacid=32893806 transcript=Vocar.0032s0159.1 locus=Vocar.0032s0159 ID=Vocar.0032s0159.1.v2.1 annot-version=v2.1 Volvox_carteri OG0000046
jgi|ChlNC64A_1|134694|IGS.gm_12_00080 Chlorella_variabilis OG0000046
jgi|ChlNC64A_1|20586|e_gw1.4.292.1 Chlorella_variabilis

OG0000046
jgi|ChlNC64A_1|20698|e_gw1.4.569.1	Chlorella_variabilis
OG0000046
jgi|ChlNC64A_1|24715|e_gw1.14.425.1	Chlorella_variabilis
OG0000046
jgi|ChlNC64A_1|13561|gw1.14.296.1 Chlorella_variabilis
OG0000046
jgi|ChlNC64A_1|134397|IGS.gm_11_00243	Chlorella_variabilis
OG0000046
jgi|ChlNC64A_1|57991|estExt_fgenesh3_pg.C_110180
Chlorella_variabilis	OG0000046
jgi|ChlNC64A_1|10671|gw1.5.248.1	Chlorella_variabilis
OG0000046
jgi|ChlNC64A_1|57670|estExt_fgenesh3_pg.C_80222
Chlorella_variabilis	OG0000046
jgi|ChlNC64A_1|136724|IGS.gm_17_00128	Chlorella_variabilis
OG0000046
jgi|ChlNC64A_1|141087|IGS.gm_31_00030	Chlorella_variabilis
OG0000046
58692 pacid=27388295 transcript=58692 locus=Genemark1.7721_g
ID=58692.2.0.227 annot-version=v2.0	Coccomyxa_subellipsoidea
OG0000046
48937 pacid=27388365 transcript=48937
locus=estExt_fgenesh1_pg.C_190115 ID=48937.2.0.227 annot-
version=v2.0	Coccomyxa_subellipsoidea OG0000046
46772 pacid=27389869 transcript=46772
locus=estExt_fgenesh1_pg.C_40412 ID=46772.2.0.227 annot-version=v2.0
	Coccomyxa_subellipsoidea OG0000046
46591 pacid=27390019 transcript=46591
locus=estExt_fgenesh1_pg.C_40097 ID=46591.2.0.227 annot-version=v2.0
	Coccomyxa_subellipsoidea OG0000046
67111 pacid=27386269 transcript=67111
locus=estExt_Genemark1.C_130186 ID=67111.2.0.227 annot-version=v2.0
Coccomyxa_subellipsoidea OG0000046
15606 pacid=27392379 transcript=15606 locus=e_gw1.8.383.1
ID=15606.2.0.227 annot-version=v2.0	Coccomyxa_subellipsoidea
OG0000046
29203 pacid=27392523 transcript=29203
locus=estExt_Genewise1Plus.C_80344 ID=29203.2.0.227 annot-
version=v2.0	Coccomyxa_subellipsoidea OG0000046
59609 pacid=27392798 transcript=59609 locus=Genemark1.7925_g
ID=59609.2.0.227 annot-version=v2.0	Coccomyxa_subellipsoidea
OG0000046
14538_fgenesh1_pg.C_Chr_20.0001000053	Ostreococcus_tauri
OG0000046
14732_e_gw1.01.00.334.1	Ostreococcus_tauri	OG0000046
14843_e_gw1.01.00.505.1	Ostreococcus_tauri	OG0000046
32220_0400010166 Ostreococcus_tauri	OG0000046
35087_1000010107 Ostreococcus_tauri	OG0000046
37219_1600010207 Ostreococcus_tauri	OG0000046
36955 pacid=27415400 transcript=36955 locus=e_gwEuk.4.260.1
ID=36955.2.0.231 annot-version=v2.0	Ostreococcus_lucimarinus
OG0000046
26074 pacid=27416264 transcript=26074 locus=eugene.1000010108

ID=26074.2.0.231 annot-version=v2.0	Ostreococcus_lucimarinus
OG0000046
40439 pacid=27417232 transcript=40439 locus=e_gwEuk.15.243.1
ID=40439.2.0.231 annot-version=v2.0	Ostreococcus_lucimarinus
OG0000046
4127 pacid=27417396 transcript=4127 locus=gwEuk.15.129.1
ID=4127.2.0.231 annot-version=v2.0	Ostreococcus_lucimarinus
OG0000046
4661 pacid=27417997 transcript=4661 locus=gwEuk.1.407.1
ID=4661.2.0.231 annot-version=v2.0	Ostreococcus_lucimarinus
OG0000046
32200 pacid=27418539 transcript=32200 locus=eugene.0600010259
ID=32200.2.0.231 annot-version=v2.0	Ostreococcus_lucimarinus
OG0000046
89498 pacid=27419107 transcript=89498 locus=ost_17_003_061
ID=89498.2.0.231 annot-version=v2.0	Ostreococcus_lucimarinus
OG0000046
28588 pacid=27419160 transcript=28588 locus=eugene.1900010074
ID=28588.2.0.231 annot-version=v2.0	Ostreococcus_lucimarinus
OG0000046
28587 pacid=27419188 transcript=28587 locus=eugene.1900010073
ID=28587.2.0.231 annot-version=v2.0	Ostreococcus_lucimarinus
OG0000046
6750 pacid=27419484 transcript=6750 locus=gwEuk.8.383.1
ID=6750.2.0.231 annot-version=v2.0	Ostreococcus_lucimarinus
OG0000046
36528 pacid=27348810 transcript=36528
locus=MicpuC2.estExt_Genewise1Plus.C_150092 ID=36528.3.0.228 annot-version=v3.0	Micromonas_pusilla	OG0000046
42406 pacid=27339781 transcript=42406
locus=MicpuC2.fgenesh1_pg.C_scaffold_13000056 ID=42406.3.0.228
annot-version=v3.0	Micromonas_pusilla	OG0000046
53243 pacid=27342272 transcript=53243 locus=MicpuC2.EuGene.
0000140338 ID=53243.3.0.228 annot-version=v3.0
Micromonas_pusilla	OG0000046
9232 pacid=27339064 transcript=9232 locus=MicpuC2.gw1.8.496.1
ID=9232.3.0.228 annot-version=v3.0	Micromonas_pusilla
OG0000046
196336 pacid=27345623 transcript=196376 locus=fgenesh1_pg.1_#_223
ID=196376.3.0.228 annot-version=v3.0	Micromonas_pusilla
OG0000046
183502 pacid=27345710 transcript=183542 locus=gm1.623_g
ID=183542.3.0.228 annot-version=v3.0	Micromonas_pusilla
OG0000046
69057 pacid=27338741 transcript=69057 locus=MicpuC2.est_orfs.
8_8040_4275315:1 ID=69057.3.0.228 annot-version=v3.0
Micromonas_pusilla	OG0000046
69279 pacid=27397279 transcript=69279 locus=gw2.10.213.1
ID=69279.3.0.229 annot-version=v3.0	Micromonas_sp._RCC299
OG0000046
86032 pacid=27397415 transcript=86032 locus=e_gw2.10.177.1
ID=86032.3.0.229 annot-version=v3.0	Micromonas_sp._RCC299
OG0000046
79669 pacid=27397920 transcript=79669 locus=e_gw2.03.579.1

ID=79669.3.0.229 annot-version=v3.0   Micromonas_sp._RCC299
OG0000046
75639 pacid=27398995 transcript=75639 locus=gw2.01.1035.1
ID=75639.3.0.229 annot-version=v3.0   Micromonas_sp._RCC299
OG0000046
55564 pacid=27399129 transcript=55564 locus=EuGene.0100010791
ID=55564.3.0.229 annot-version=v3.0   Micromonas_sp._RCC299
OG0000046
88752 pacid=27400997 transcript=88752 locus=e_gw2.15.139.1
ID=88752.3.0.229 annot-version=v3.0   Micromonas_sp._RCC299
OG0000046
50521 pacid=27401580 transcript=50521
locus=est_orfs.Chr_09_392_3200744:1 ID=50521.3.0.229 annot-
version=v3.0   Micromonas_sp._RCC299   OG0000046
59594 pacid=27402363 transcript=59594 locus=EuGene.0600010596
ID=59594.3.0.229 annot-version=v3.0   Micromonas_sp._RCC299
OG0000046
89262 pacid=27403011 transcript=89262 locus=e_gw2.17.24.1
ID=89262.3.0.229 annot-version=v3.0   Micromonas_sp._RCC299
OG0000046
68774 pacid=27403885 transcript=68774 locus=gw2.14.167.1
ID=68774.3.0.229 annot-version=v3.0   Micromonas_sp._RCC299
OG0000046
101382 pacid=27404687 transcript=101382
locus=fgenesh2_pg.C_Chr_07000126 ID=101382.3.0.229 annot-
version=v3.0   Micromonas_sp._RCC299   OG0000046
62560 pacid=27405343 transcript=62560 locus=EuGene.1100010346
ID=62560.3.0.229 annot-version=v3.0   Micromonas_sp._RCC299
OG0000046
kfl00419_0020_v1.1   Klebsormidium_flaccidum OG0000046
kfl00419_0050_v1.1   Klebsormidium_flaccidum OG0000046
kfl00029_0260_v1.1   Klebsormidium_flaccidum OG0000046
kfl00048_0110_v1.1   Klebsormidium_flaccidum OG0000046
kfl00093_0130_v1.1   Klebsormidium_flaccidum OG0000046
kfl00103_0110_v1.1   Klebsormidium_flaccidum OG0000046
kfl00115_0080_v1.1   Klebsormidium_flaccidum OG0000046
kfl00140_0130_v1.1   Klebsormidium_flaccidum OG0000046
kfl00157_0040_v1.1   Klebsormidium_flaccidum OG0000046
kfl00164_0110_v1.1   Klebsormidium_flaccidum OG0000046
kfl00269_0070_v1.1   Klebsormidium_flaccidum OG0000046
Pp3c10_2230V3.1.p pacid=32902443 transcript=Pp3c10_2230V3.1
locus=Pp3c10_2230 annot-version=v3.3   Physcomitrella_patens
OG0000046
Pp3c2_32690V3.1.p pacid=32934177 transcript=Pp3c2_32690V3.1
locus=Pp3c2_32690 annot-version=v3.3   Physcomitrella_patens
OG0000046
Pp3c3_6440V3.1.p pacid=32940486 transcript=Pp3c3_6440V3.1
locus=Pp3c3_6440 annot-version=v3.3   Physcomitrella_patens
OG0000046
Pp3c3_15300V3.1.p pacid=32941721 transcript=Pp3c3_15300V3.1
locus=Pp3c3_15300 annot-version=v3.3   Physcomitrella_patens
OG0000046
Pp3c3_17660V3.1.p pacid=32944054 transcript=Pp3c3_17660V3.1
locus=Pp3c3_17660 annot-version=v3.3   Physcomitrella_patens

OG0000046
Pp3c20_17190V3.1.p pacid=32945836 transcript=Pp3c20_17190V3.1 locus=Pp3c20_17190 annot-version=v3.3 Physcomitrella_patens OG0000046
Pp3c23_5890V3.1.p pacid=32948631 transcript=Pp3c23_5890V3.1 locus=Pp3c23_5890 annot-version=v3.3 Physcomitrella_patens OG0000046
Pp3c23_11270V3.1.p pacid=32950015 transcript=Pp3c23_11270V3.1 locus=Pp3c23_11270 annot-version=v3.3 Physcomitrella_patens OG0000046
Pp3c14_4640V3.1.p pacid=32960522 transcript=Pp3c14_4640V3.1 locus=Pp3c14_4640 annot-version=v3.3 Physcomitrella_patens OG0000046
Pp3c14_4760V3.1.p pacid=32961125 transcript=Pp3c14_4760V3.1 locus=Pp3c14_4760 annot-version=v3.3 Physcomitrella_patens OG0000046
Pp3c14_25730V3.1.p pacid=32962978 transcript=Pp3c14_25730V3.1 locus=Pp3c14_25730 annot-version=v3.3 Physcomitrella_patens OG0000046
Pp3c24_9770V3.1.p pacid=32910530 transcript=Pp3c24_9770V3.1 locus=Pp3c24_9770 annot-version=v3.3 Physcomitrella_patens OG0000046
Pp3c10_3890V3.1.p pacid=32900684 transcript=Pp3c10_3890V3.1 locus=Pp3c10_3890 annot-version=v3.3 Physcomitrella_patens OG0000046
Pp3c10_3893V3.1.p pacid=32900817 transcript=Pp3c10_3893V3.1 locus=Pp3c10_3893 annot-version=v3.3 Physcomitrella_patens OG0000046
Pp3c10_6070V3.1.p pacid=32901115 transcript=Pp3c10_6070V3.1 locus=Pp3c10_6070 annot-version=v3.3 Physcomitrella_patens OG0000046
AT3G02510.1 | Regulator of chromosome condensation (RCC1) family protein | Chr3:522086-524536 REVERSE LENGTH=393 | 201606 Arabidopsis_thaliana OG0000046
AT3G15430.1 | Regulator of chromosome condensation (RCC1) family protein | Chr3:5209408-5211562 FORWARD LENGTH=488 | 201606 Arabidopsis_thaliana OG0000046
AT3G23270.1 | Regulator of chromosome condensation (RCC1) family with FYVE zinc finger domain-containing protein | Chr3:8316436-8320337 FORWARD LENGTH=1045 | 201606 Arabidopsis_thaliana OG0000046
AT3G26100.1 | Regulator of chromosome condensation (RCC1) family protein | Chr3:9538458-9540149 FORWARD LENGTH=432 | 201606 Arabidopsis_thaliana OG0000046
AT3G47660.1 | Regulator of chromosome condensation (RCC1) family protein | Chr3:17571192-17574805 FORWARD LENGTH=954 | 201606 Arabidopsis_thaliana OG0000046
AT3G53830.1 | Regulator of chromosome condensation (RCC1) family protein | Chr3:19940814-19943039 FORWARD LENGTH=424 | 201606 Arabidopsis_thaliana OG0000046
AT3G55580.1 | Regulator of chromosome condensation (RCC1) family protein | Chr3:20612766-20615730 FORWARD LENGTH=488 | 201606 Arabidopsis_thaliana OG0000046
AT4G14368.1 | Regulator of chromosome condensation (RCC1) family

protein | Chr4:8274279-8278810 REVERSE LENGTH=1106 | 201606
Arabidopsis_thaliana OG0000046
AT5G08710.1 | Regulator of chromosome condensation (RCC1) family protein | Chr5:2834858-2837866 REVERSE LENGTH=434 | 201606
Arabidopsis_thaliana OG0000046
AT5G11580.1 | Regulator of chromosome condensation (RCC1) family protein | Chr5:3718913-3721125 FORWARD LENGTH=553 | 201606
Arabidopsis_thaliana OG0000046
AT5G12350.1 | Regulator of chromosome condensation (RCC1) family with FYVE zinc finger domain-containing protein | Chr5:3995789-4000545 FORWARD LENGTH=1075 | 201606
Arabidopsis_thaliana OG0000046
AT5G16040.1 | Regulator of chromosome condensation (RCC1) family protein | Chr5:5240860-5243308 REVERSE LENGTH=396 | 201606
Arabidopsis_thaliana OG0000046
AT5G19420.1 | Regulator of chromosome condensation (RCC1) family with FYVE zinc finger domain-containing protein | Chr5:6547945-6552866 REVERSE LENGTH=1105 | 201606
Arabidopsis_thaliana OG0000046
AT5G42140.1 | Regulator of chromosome condensation (RCC1) family with FYVE zinc finger domain-containing protein | Chr5:16837547-16841985 REVERSE LENGTH=1078 | 201606
Arabidopsis_thaliana OG0000046
AT5G48330.1 | Regulator of chromosome condensation (RCC1) family protein | Chr5:19585989-19587729 FORWARD LENGTH=455 | 201606
Arabidopsis_thaliana OG0000046
AT5G60870.1 | Regulator of chromosome condensation (RCC1) family protein | Chr5:24485788-24487771 REVERSE LENGTH=445 | 201606
Arabidopsis_thaliana OG0000046
AT1G27060.1 | Regulator of chromosome condensation (RCC1) family protein | Chr1:9395023-9396673 FORWARD LENGTH=386 | 201606
Arabidopsis_thaliana OG0000046
AT5G63860.1 | Regulator of chromosome condensation (RCC1) family protein | Chr5:25554821-25558587 REVERSE LENGTH=440 | 201606
Arabidopsis_thaliana OG0000046
AT1G65920.1 | Regulator of chromosome condensation (RCC1) family with FYVE zinc finger domain-containing protein | Chr1:24525267-24529204 REVERSE LENGTH=1006 | 201606
Arabidopsis_thaliana OG0000046
AT1G69710.1 | Regulator of chromosome condensation (RCC1) family with FYVE zinc finger domain-containing protein | Chr1:26222325-26226530 FORWARD LENGTH=1041 | 201606
Arabidopsis_thaliana OG0000046
AT1G76950.1 | Regulator of chromosome condensation (RCC1) family with FYVE zinc finger domain-containing protein | Chr1:28906952-28911325 FORWARD LENGTH=1103 | 201606
Arabidopsis_thaliana OG0000046
Os04t0226400-01 Regulator of chromosome condensation/beta-lactamase-inhibitor protein II domain containing protein. Oryza_sativa OG0000046
Os04t0435700-01 Similar to UVB-resistance protein UVR8. Oryza_sativa OG0000046
Os04t0583700-00 Zinc finger, FYVE-type domain containing protein. Oryza_sativa OG0000046

Os04t0686200-01 Similar to Chromosome condensation regulator protein (Fragment).   Oryza_sativa   OG0000046
Os05t0106700-00 Similar to PRAF1; Ran GTPase binding / chromatin binding / zinc ion binding.   Oryza_sativa   OG0000046
Os05t0384800-01 Similar to Chromosome condensation regulator protein (Fragment).   Oryza_sativa   OG0000046
Os05t0456925-00 Similar to regulator of chromosome condensation (RCC1) family protein.   Oryza_sativa   OG0000046
Os05t0545200-00 Similar to zinc ion binding.   Oryza_sativa   OG0000046
Os07t0459400-01 Regulator of chromosome condensation, RCC1 domain containing protein.   Oryza_sativa   OG0000046
Os08t0430700-01 Similar to UVB-resistance protein-like.   Oryza_sativa   OG0000046
Os09t0560450-01 Similar to Regulator of chromosome condensation/ beta-lactamase-inhibitor protein II.   Oryza_sativa   OG0000046
Os01t0700200-01 Similar to Chromosome condensation regulator protein (Fragment).   Oryza_sativa   OG0000046
Os11t0545800-01 Similar to regulator of chromosome condensation (RCC1) family protein / UVB-resistance protein-related.   Oryza_sativa   OG0000046
Os12t0284000-01 Regulator of chromosome condensation, RCC1 domain containing protein.   Oryza_sativa   OG0000046
Os01t0777800-01 Hypothetical conserved gene.   Oryza_sativa   OG0000046
Os01t0846800-01 Regulator of chromosome condensation, RCC1 domain containing protein.   Oryza_sativa   OG0000046
Os01t0952300-00 Similar to Ran GTPase binding / chromatin binding / zinc ion binding.   Oryza_sativa   OG0000046
Os01t0964800-01 Similar to PRAF1; Ran GTPase binding / chromatin binding / zinc ion binding.   Oryza_sativa   OG0000046
Os02t0197500-01 Regulator of chromosome condensation, RCC1 domain containing protein.   Oryza_sativa   OG0000046
Os02t0554100-01 Similar to UVB-resistance protein UVR8.   Oryza_sativa   OG0000046
Os02t0597000-01 Regulator of chromosome condensation, RCC1 domain containing protein.   Oryza_sativa   OG0000046
Os02t0684900-01 Similar to Chromosome condensation regulator protein (Fragment).   Oryza_sativa   OG0000046
CMH019CT pep chromosome:ASM9120v1:8:55186:56560:-1 gene:CMH019C transcript:CMH019CT gene_biotype:protein_coding transcript_biotype:protein_coding Cyanidioschyzon_merolae OG0000046
CMC176CT pep chromosome:ASM9120v1:3:434904:436533:-1 gene:CMC176C transcript:CMC176CT gene_biotype:protein_coding transcript_biotype:protein_coding Cyanidioschyzon_merolae OG0000046
evm.model.contig_2108.7 Porphyridium_purpureum   OG0000046
evm.model.contig_2301.3 Porphyridium_purpureum   OG0000046
evm.model.contig_3715.4 Porphyridium_purpureum   OG0000046
evm.model.contig_4409.1 Porphyridium_purpureum   OG0000046
CDF41037 pep supercontig:ASM35022v2:HG002301:142626:145024:1 gene:CHC_T00007651001 transcript:CDF41037

gene_biotype:protein_coding transcript_biotype:protein_coding
Chondrus_crispus OG0000046
CDF41147 pep supercontig:ASM35022v2:HG002310:205916:207820:1
gene:CHC_T00007700001 transcript:CDF41147
gene_biotype:protein_coding transcript_biotype:protein_coding
Chondrus_crispus OG0000046
CDF33819 pep supercontig:ASM35022v2:HG001658:207956:208711:1
gene:CHC_T00002425001 transcript:CDF33819
gene_biotype:protein_coding transcript_biotype:protein_coding
Chondrus_crispus OG0000046
g1093.t1 Caulerpa_lentillifera OG0000047
g1188.t1 Caulerpa_lentillifera OG0000047
g2460.t1 Caulerpa_lentillifera OG0000047
g3845.t1 Caulerpa_lentillifera OG0000047
g4317.t1 Caulerpa_lentillifera OG0000047
g4527.t1 Caulerpa_lentillifera OG0000047
g4936.t1 Caulerpa_lentillifera OG0000047
g5017.t1 Caulerpa_lentillifera OG0000047
g508.t1 Caulerpa_lentillifera OG0000047
g5549.t1 Caulerpa_lentillifera OG0000047
g6397.t1 Caulerpa_lentillifera OG0000047
g7851.t1 Caulerpa_lentillifera OG0000047
g824.t1 Caulerpa_lentillifera OG0000047
g8298.t1 Caulerpa_lentillifera OG0000047
g8444.t1 Caulerpa_lentillifera OG0000047
g8620.t1 Caulerpa_lentillifera OG0000047
g8649.t1 Caulerpa_lentillifera OG0000047
Cre02.g141250.t1.2 pacid=30785628 transcript=Cre02.g141250.t1.2
locus=Cre02.g141250 ID=Cre02.g141250.t1.2.v5.5 annot-version=v5.5
Chlamydomonas_reinhardtii OG0000047
Cre03.g211073.t1.1 pacid=30787032 transcript=Cre03.g211073.t1.1
locus=Cre03.g211073 ID=Cre03.g211073.t1.1.v5.5 annot-version=v5.5
Chlamydomonas_reinhardtii OG0000047
Cre04.g218150.t1.2 pacid=30791276 transcript=Cre04.g218150.t1.2
locus=Cre04.g218150 ID=Cre04.g218150.t1.2.v5.5 annot-version=v5.5
Chlamydomonas_reinhardtii OG0000047
Cre12.g529150.t1.2 pacid=30792340 transcript=Cre12.g529150.t1.2
locus=Cre12.g529150 ID=Cre12.g529150.t1.2.v5.5 annot-version=v5.5
Chlamydomonas_reinhardtii OG0000047
Cre17.g715700.t1.2 pacid=30782880 transcript=Cre17.g715700.t1.2
locus=Cre17.g715700 ID=Cre17.g715700.t1.2.v5.5 annot-version=v5.5
Chlamydomonas_reinhardtii OG0000047
Vocar.0012s0066.1.p pacid=32894930 transcript=Vocar.0012s0066.1
locus=Vocar.0012s0066 ID=Vocar.0012s0066.1.v2.1 annot-version=v2.1
Volvox_carteri OG0000047
Vocar.0013s0168.1.p pacid=32896256 transcript=Vocar.0013s0168.1
locus=Vocar.0013s0168 ID=Vocar.0013s0168.1.v2.1 annot-version=v2.1
Volvox_carteri OG0000047
Vocar.0008s0010.1.p pacid=32897406 transcript=Vocar.0008s0010.1
locus=Vocar.0008s0010 ID=Vocar.0008s0010.1.v2.1 annot-version=v2.1
Volvox_carteri OG0000047
Vocar.0037s0027.1.p pacid=32897578 transcript=Vocar.0037s0027.1
locus=Vocar.0037s0027 ID=Vocar.0037s0027.1.v2.1 annot-version=v2.1
Volvox_carteri OG0000047

Vocar.0025s0118.1.p pacid=32898340 transcript=Vocar.0025s0118.1 locus=Vocar.0025s0118 ID=Vocar.0025s0118.1.v2.1 annot-version=v2.1 Volvox_carteri OG0000047
Vocar.0005s0464.1.p pacid=32883768 transcript=Vocar.0005s0464.1 locus=Vocar.0005s0464 ID=Vocar.0005s0464.1.v2.1 annot-version=v2.1 Volvox_carteri OG0000047
jgi|ChlNC64A_1|36133|estExt_Genewise1Plus.C_140353 Chlorella_variabilis OG0000047
jgi|ChlNC64A_1|58836|estExt_fgenesh3_pg.C_210114 Chlorella_variabilis OG0000047
jgi|ChlNC64A_1|35180|estExt_Genewise1Plus.C_80317 Chlorella_variabilis OG0000047
jgi|ChlNC64A_1|58367|estExt_fgenesh3_pg.C_150065 Chlorella_variabilis OG0000047
jgi|ChlNC64A_1|141147|IGS.gm_31_00090 Chlorella_variabilis OG0000047
48919 pacid=27388455 transcript=48919 locus=estExt_fgenesh1_pg.C_190078 ID=48919.2.0.227 annot-version=v2.0 Coccomyxa_subellipsoidea OG0000047
52794 pacid=27389943 transcript=52794 locus=estExt_fgenesh1_pm.C_40089 ID=52794.2.0.227 annot-version=v2.0 Coccomyxa_subellipsoidea OG0000047
6402 pacid=27391914 transcript=6402 locus=gw1.11.201.1 ID=6402.2.0.227 annot-version=v2.0 Coccomyxa_subellipsoidea OG0000047
59328 pacid=27393076 transcript=59328 locus=Genemark1.1290_g ID=59328.2.0.227 annot-version=v2.0 Coccomyxa_subellipsoidea OG0000047
36967 pacid=27385711 transcript=36967 locus=fgenesh1_pm.10_#_55 ID=36967.2.0.227 annot-version=v2.0 Coccomyxa_subellipsoidea OG0000047
4137_gw1.07.00.256.1 Ostreococcus_tauri OG0000047
20474_e_gw1.12.00.62.1 Ostreococcus_tauri OG0000047
9829_fgenesh1_pg.C_Chr_02.0001000124 Ostreococcus_tauri OG0000047
16113 pacid=27414072 transcript=16113 locus=fgenesh1_pg.C_Chr_7000157 ID=16113.2.0.231 annot-version=v2.0 Ostreococcus_lucimarinus OG0000047
119508 pacid=27414837 transcript=119508 locus=eugene1.1.0200012310 ID=119508.2.0.231 annot-version=v2.0 Ostreococcus_lucimarinus OG0000047
30044 pacid=27414996 transcript=30044 locus=eugene.0200010231 ID=30044.2.0.231 annot-version=v2.0 Ostreococcus_lucimarinus OG0000047
51117 pacid=27417002 transcript=51117 locus=estExt_Genewise_ext.C_Chr_120387 ID=51117.2.0.231 annot-version=v2.0 Ostreococcus_lucimarinus OG0000047
125626 pacid=27339838 transcript=125666 locus=estExt_fgenesh3_kg.C_130033 ID=125666.3.0.228 annot-version=v3.0 Micromonas_pusilla OG0000047
22355 pacid=27342362 transcript=22355 locus=MicpuC2.e_gw1.14.31.1 ID=22355.3.0.228 annot-version=v3.0 Micromonas_pusilla OG0000047
177954 pacid=27346117 transcript=177994

locus=estExt_Genewise1Plus.C_4_t30230 ID=177994.3.0.228 annot-version=v3.0	Micromonas_pusilla	OG0000047
56172 pacid=27396914 transcript=56172 locus=EuGene.0200010549 ID=56172.3.0.229 annot-version=v3.0	Micromonas_sp._RCC299 OG0000047
109652 pacid=27397220 transcript=109652 locus=est_cluster_kg.Chr_16__49__3202264:1 ID=109652.3.0.229 annot-version=v3.0	Micromonas_sp._RCC299	OG0000047
88758 pacid=27401113 transcript=88758 locus=e_gw2.15.40.1 ID=88758.3.0.229 annot-version=v3.0	Micromonas_sp._RCC299 OG0000047
51003 pacid=27404412 transcript=51003 locus=est_orfs.Chr_07_874_3201116:1 ID=51003.3.0.229 annot-version=v3.0	Micromonas_sp._RCC299	OG0000047
kfl00343_0110_v1.1	Klebsormidium_flaccidum	OG0000047
kfl00395_0090_v1.1	Klebsormidium_flaccidum	OG0000047
kfl00680_0020_v1.1	Klebsormidium_flaccidum	OG0000047
kfl00724_0050_v1.1	Klebsormidium_flaccidum	OG0000047
kfl00753_0080_v1.1	Klebsormidium_flaccidum	OG0000047
kfl00126_0200_v1.1	Klebsormidium_flaccidum	OG0000047
kfl00170_0040_v1.1	Klebsormidium_flaccidum	OG0000047
Pp3c2_5310V3.1.p pacid=32935971 transcript=Pp3c2_5310V3.1 locus=Pp3c2_5310 annot-version=v3.3	Physcomitrella_patens OG0000047
Pp3c2_10660V3.1.p pacid=32936277 transcript=Pp3c2_10660V3.1 locus=Pp3c2_10660 annot-version=v3.3	Physcomitrella_patens OG0000047
Pp3c19_9620V3.1.p pacid=32938711 transcript=Pp3c19_9620V3.1 locus=Pp3c19_9620 annot-version=v3.3	Physcomitrella_patens OG0000047
Pp3c3_22160V3.1.p pacid=32941192 transcript=Pp3c3_22160V3.1 locus=Pp3c3_22160 annot-version=v3.3	Physcomitrella_patens OG0000047
Pp3c27_290V3.1.p pacid=32951468 transcript=Pp3c27_290V3.1 locus=Pp3c27_290 annot-version=v3.3	Physcomitrella_patens OG0000047
Pp3c5_20390V3.1.p pacid=32954009 transcript=Pp3c5_20390V3.1 locus=Pp3c5_20390 annot-version=v3.3	Physcomitrella_patens OG0000047
Pp3c11_18330V3.1.p pacid=32956990 transcript=Pp3c11_18330V3.1 locus=Pp3c11_18330 annot-version=v3.3	Physcomitrella_patens OG0000047
Pp3c22_21360V3.1.p pacid=32905328 transcript=Pp3c22_21360V3.1 locus=Pp3c22_21360 annot-version=v3.3	Physcomitrella_patens OG0000047
Pp3c14_14460V3.1.p pacid=32960688 transcript=Pp3c14_14460V3.1 locus=Pp3c14_14460 annot-version=v3.3	Physcomitrella_patens OG0000047
Pp3c1_24090V3.1.p pacid=32968716 transcript=Pp3c1_24090V3.1 locus=Pp3c1_24090 annot-version=v3.3	Physcomitrella_patens OG0000047
Pp3c6_3580V3.1.p pacid=32976571 transcript=Pp3c6_3580V3.1 locus=Pp3c6_3580 annot-version=v3.3	Physcomitrella_patens OG0000047

Pp3c25_12860V3.1.p pacid=32980666 transcript=Pp3c25_12860V3.1 locus=Pp3c25_12860 annot-version=v3.3 Physcomitrella_patens OG0000047
Pp3c18_2230V3.1.p pacid=32983493 transcript=Pp3c18_2230V3.1 locus=Pp3c18_2230 annot-version=v3.3 Physcomitrella_patens OG0000047
Pp3c17_11030V3.1.p pacid=32908147 transcript=Pp3c17_11030V3.1 locus=Pp3c17_11030 annot-version=v3.3 Physcomitrella_patens OG0000047
Pp3c16_21950V3.1.p pacid=32985695 transcript=Pp3c16_21950V3.1 locus=Pp3c16_21950 annot-version=v3.3 Physcomitrella_patens OG0000047
Pp3c4_23550V3.1.p pacid=32922455 transcript=Pp3c4_23550V3.1 locus=Pp3c4_23550 annot-version=v3.3 Physcomitrella_patens OG0000047
Pp3c7_5390V3.1.p pacid=32923453 transcript=Pp3c7_5390V3.1 locus=Pp3c7_5390 annot-version=v3.3 Physcomitrella_patens OG0000047
Pp3c7_5900V3.1.p pacid=32925096 transcript=Pp3c7_5900V3.1 locus=Pp3c7_5900 annot-version=v3.3 Physcomitrella_patens OG0000047
AT2G34740.1 | protein phosphatase 2C family protein | Chr2:14658730-14660305 FORWARD LENGTH=339 | 201606 Arabidopsis_thaliana OG0000047
AT2G40180.1 | phosphatase 2C5 | Chr2:16782522-16784014 FORWARD LENGTH=390 | 201606 Arabidopsis_thaliana OG0000047
AT2G40860.1 | protein kinase family protein / protein phosphatase 2C (PP2C) family protein | Chr2:17053747-17057108 REVERSE LENGTH=658 | 201606 Arabidopsis_thaliana OG0000047
AT3G11410.1 | protein phosphatase 2CA | Chr3:3584181-3585649 REVERSE LENGTH=399 | 201606 Arabidopsis_thaliana OG0000047
AT3G15260.1 | Protein phosphatase 2C family protein | Chr3:5138842-5140242 FORWARD LENGTH=289 | 201606 Arabidopsis_thaliana OG0000047
AT3G17250.1 | Protein phosphatase 2C family protein | Chr3:5892875-5894426 REVERSE LENGTH=422 | 201606 Arabidopsis_thaliana OG0000047
AT3G27140.1 | Protein phosphatase 2C family protein | Chr3:10006891-10008174 REVERSE LENGTH=245 | 201606 Arabidopsis_thaliana OG0000047
AT3G51470.1 | Protein phosphatase 2C family protein | Chr3:19097924-19099244 REVERSE LENGTH=361 | 201606 Arabidopsis_thaliana OG0000047
AT3G62260.1 | Protein phosphatase 2C family protein | Chr3:23038516-23040391 REVERSE LENGTH=383 | 201606 Arabidopsis_thaliana OG0000047
AT4G08260.1 | Protein phosphatase 2C family protein | Chr4:5200847-5201865 FORWARD LENGTH=212 | 201606 Arabidopsis_thaliana OG0000047
AT1G17550.1 | homology to ABI2 | Chr1:6034917-6036939 FORWARD LENGTH=511 | 201606 Arabidopsis_thaliana OG0000047
AT4G26080.1 | Protein phosphatase 2C family protein | Chr4:13220231-13221828 REVERSE LENGTH=434 | 201606 Arabidopsis_thaliana OG0000047

AT4G27800.1 | thylakoid-associated phosphatase 38 |
Chr4:13852013-13854091 REVERSE LENGTH=388 | 201606
Arabidopsis_thaliana OG0000047
AT4G28400.1 | Protein phosphatase 2C family protein |
Chr4:14048499-14050118 FORWARD LENGTH=283 | 201606
Arabidopsis_thaliana OG0000047
AT4G31750.1 | HOPW1-1-interacting 2 | Chr4:15364657-15367207 REVERSE
LENGTH=311 | 201606 Arabidopsis_thaliana OG0000047
AT5G10740.1 | Protein phosphatase 2C family protein |
Chr5:3393797-3395848 REVERSE LENGTH=354 | 201606
Arabidopsis_thaliana OG0000047
AT1G22280.1 | phytochrome-associated protein phosphatase type 2C |
Chr1:7874236-7875496 FORWARD LENGTH=281 | 201606
Arabidopsis_thaliana OG0000047
AT5G24940.1 | Protein phosphatase 2C family protein |
Chr5:8591407-8593601 REVERSE LENGTH=447 | 201606
Arabidopsis_thaliana OG0000047
AT5G51760.1 | Protein phosphatase 2C family protein |
Chr5:21026916-21028912 FORWARD LENGTH=416 | 201606
Arabidopsis_thaliana OG0000047
AT5G53140.1 | Protein phosphatase 2C family protein |
Chr5:21549228-21552132 FORWARD LENGTH=420 | 201606
Arabidopsis_thaliana OG0000047
AT5G57050.1 | Protein phosphatase 2C family protein |
Chr5:23087720-23089303 FORWARD LENGTH=423 | 201606
Arabidopsis_thaliana OG0000047
AT5G59220.1 | PP2C protein (Clade A protein phosphatases type 2C) |
Chr5:23894672-23896497 REVERSE LENGTH=413 | 201606
Arabidopsis_thaliana OG0000047
AT1G34750.1 | Protein phosphatase 2C family protein |
Chr1:12736386-12737727 REVERSE LENGTH=282 | 201606
Arabidopsis_thaliana OG0000047
AT1G43900.1 | Protein phosphatase 2C family protein |
Chr1:16654045-16655810 FORWARD LENGTH=371 | 201606
Arabidopsis_thaliana OG0000047
AT1G48040.1 | Protein phosphatase 2C family protein |
Chr1:17720064-17721698 REVERSE LENGTH=383 | 201606
Arabidopsis_thaliana OG0000047
AT1G67820.1 | Protein phosphatase 2C family protein |
Chr1:25429882-25431484 FORWARD LENGTH=445 | 201606
Arabidopsis_thaliana OG0000047
AT1G72770.1 | HYPERSENSITIVE TO ABA1 | Chr1:27390998-27392851
FORWARD LENGTH=511 | 201606 Arabidopsis_thaliana
OG0000047
AT1G07160.1 | Protein phosphatase 2C family protein |
Chr1:2198155-2199678 REVERSE LENGTH=380 | 201606
Arabidopsis_thaliana OG0000047
AT1G07430.1 | highly ABA-induced PP2C protein 2 |
Chr1:2281151-2282656 REVERSE LENGTH=442 | 201606
Arabidopsis_thaliana OG0000047
AT1G78200.1 | Protein phosphatase 2C family protein |
Chr1:29420483-29421650 FORWARD LENGTH=283 | 201606
Arabidopsis_thaliana OG0000047
AT2G20630.1 | PP2C induced by AVRRPM1 | Chr2:8897826-8899648 REVERSE

LENGTH=279 | 201606	Arabidopsis_thaliana	OG0000047
AT2G25620.1 | DNA-binding protein phosphatase 1 | Chr2:10903154-10904978 REVERSE LENGTH=392 | 201606	Arabidopsis_thaliana	OG0000047
AT2G29380.1 | highly ABA-induced PP2C protein 3 | Chr2:12608855-12610124 FORWARD LENGTH=362 | 201606	Arabidopsis_thaliana	OG0000047
AT2G30020.1 | Protein phosphatase 2C family protein | Chr2:12814437-12815904 FORWARD LENGTH=396 | 201606	Arabidopsis_thaliana	OG0000047
AT2G33700.1 | Protein phosphatase 2C family protein | Chr2:14254200-14255784 FORWARD LENGTH=380 | 201606	Arabidopsis_thaliana	OG0000047
Os03t0268600-01 Similar to Protein phosphatase type 2C.	Oryza_sativa	OG0000047
Os03t0292100-01 Hypothetical conserved gene.	Oryza_sativa	OG0000047
Os01t0295700-01 Similar to Protein phosphatase-2C.	Oryza_sativa	OG0000047
Os04t0452000-01 Similar to H0815C01.7 protein.	Oryza_sativa	OG0000047
Os04t0609600-01 Similar to Protein phosphatase type 2C.	Oryza_sativa	OG0000047
Os04t0659500-01 Protein phosphatase 2C domain containing protein.	Oryza_sativa	OG0000047
Os05t0134200-01 Similar to Protein phosphatase-2C.	Oryza_sativa	OG0000047
Os05t0537400-00 Similar to Protein phosphatase 2C.	Oryza_sativa	OG0000047
Os05t0587100-01 Protein phosphatase 2C family protein.	Oryza_sativa	OG0000047
Os05t0592800-01 Splicing variant of protein phosphatase 2C 53	Oryza_sativa	OG0000047
Os06t0179700-01 Similar to DNA-binding protein phosphatase 2C.	Oryza_sativa	OG0000047
Os01t0541900-01 Protein kinase, core domain containing protein.	Oryza_sativa	OG0000047
Os01t0552300-01 Similar to Protein phosphatase-2C.	Oryza_sativa	OG0000047
Os06t0526700-00 Probable protein phosphatase 2C 55.	Oryza_sativa	OG0000047
Os06t0526800-00 Probable protein phosphatase 2C 56.	Oryza_sativa	OG0000047
Os06t0597200-01 Similar to DNA-binding protein phosphatase 2C.	Oryza_sativa	OG0000047
Os01t0583100-01 Similar to Protein phosphatase 2C.	Oryza_sativa	OG0000047
Os06t0698300-01 Protein phosphatase 2C family protein.	Oryza_sativa	OG0000047
Os07t0507000-01 Protein phosphatase 2C.	Oryza_sativa	OG0000047
Os01t0656200-01 Protein phosphatase 2C family protein.	Oryza_sativa	OG0000047
Os09t0325700-01 Similar to Protein phpsphatase 2C (PP2C) (EC

3.1.3.16).	Oryza_sativa	OG0000047
Os10t0541200-01 Similar to Protein phosphatase 2C containing protein, expressed.	Oryza_sativa	OG0000047
Os11t0242200-00 Protein phosphatase 2C-like domain containing protein.	Oryza_sativa	OG0000047
Os12t0198200-00 Similar to Protein phosphatase 2C. Oryza_sativa OG0000047
Os01t0846300-01 Similar to Protein phosphatase 2C. Oryza_sativa OG0000047
Os02t0149800-01 Stress-responsive NAC1-regulated protein phosphatase, Drought and oxidative stress tolerance Oryza_sativa	OG0000047
Os02t0255100-01 Mitochondrial protein phosphatase 2C protein 13, Pollen germination	Oryza_sativa	OG0000047
Os02t0799000-01 Similar to DNA-binding protein phosphatase 2C. Oryza_sativa	OG0000047
CMK117CT pep chromosome:ASM9120v1:11:325328:326899:1 gene:CMK117C transcript:CMK117CT gene_biotype:protein_coding transcript_biotype:protein_coding Cyanidioschyzon_merolae OG0000047
CMG113CT pep chromosome:ASM9120v1:7:288333:290691:-1 gene:CMG113C transcript:CMG113CT gene_biotype:protein_coding transcript_biotype:protein_coding Cyanidioschyzon_merolae OG0000047
CMS179CT pep chromosome:ASM9120v1:19:472421:473480:-1 gene:CMS179C transcript:CMS179CT gene_biotype:protein_coding transcript_biotype:protein_coding Cyanidioschyzon_merolae OG0000047
evm.model.contig_2082.9	Porphyridium_purpureum	OG0000047
evm.model.contig_3441.20	Porphyridium_purpureum	OG0000047
evm.model.contig_3479.1	Porphyridium_purpureum	OG0000047
evm.model.contig_3807.1	Porphyridium_purpureum	OG0000047
evm.model.contig_4456.15	Porphyridium_purpureum	OG0000047
evm.model.contig_4590.3	Porphyridium_purpureum	OG0000047
evm.model.contig_510.16	Porphyridium_purpureum	OG0000047
CDF41111 pep supercontig:ASM35022v2:HG002309:265945:268088:-1 gene:CHC_T00008508001 transcript:CDF41111 gene_biotype:protein_coding transcript_biotype:protein_coding description:Serine/threonine protein kinase and serine/threonine protein phosphatase 2C Rhodoplastic	Chondrus_crispus OG0000047
CDF39494 pep supercontig:ASM35022v2:HG002045:2980:4398:-1 gene:CHC_T00000279001 transcript:CDF39494 gene_biotype:protein_coding transcript_biotype:protein_coding Chondrus_crispus OG0000047
g3403.t1 Caulerpa_lentillifera	OG0000048
g6131.t1 Caulerpa_lentillifera	OG0000048
g6137.t1 Caulerpa_lentillifera	OG0000048
g6161.t1 Caulerpa_lentillifera	OG0000048
g7254.t1 Caulerpa_lentillifera	OG0000048
g7257.t1 Caulerpa_lentillifera	OG0000048
g7258.t1 Caulerpa_lentillifera	OG0000048
g7319.t1 Caulerpa_lentillifera	OG0000048
g7322.t1 Caulerpa_lentillifera	OG0000048

Cre02.g104800.t1.1 pacid=30786414 transcript=Cre02.g104800.t1.1
locus=Cre02.g104800 ID=Cre02.g104800.t1.1.v5.5 annot-version=v5.5
Chlamydomonas_reinhardtii OG0000048
Cre03.g197050.t1.2 pacid=30786769 transcript=Cre03.g197050.t1.2
locus=Cre03.g197050 ID=Cre03.g197050.t1.2.v5.5 annot-version=v5.5
Chlamydomonas_reinhardtii OG0000048
Cre12.g504650.t1.2 pacid=30791932 transcript=Cre12.g504650.t1.2
locus=Cre12.g504650 ID=Cre12.g504650.t1.2.v5.5 annot-version=v5.5
Chlamydomonas_reinhardtii OG0000048
Cre12.g506300.t1.2 pacid=30792290 transcript=Cre12.g506300.t1.2
locus=Cre12.g506300 ID=Cre12.g506300.t1.2.v5.5 annot-version=v5.5
Chlamydomonas_reinhardtii OG0000048
Cre12.g506500.t1.2 pacid=30792666 transcript=Cre12.g506500.t1.2
locus=Cre12.g506500 ID=Cre12.g506500.t1.2.v5.5 annot-version=v5.5
Chlamydomonas_reinhardtii OG0000048
Cre12.g505500.t1.2 pacid=30793498 transcript=Cre12.g505500.t1.2
locus=Cre12.g505500 ID=Cre12.g505500.t1.2.v5.5 annot-version=v5.5
Chlamydomonas_reinhardtii OG0000048
Cre12.g504800.t1.2 pacid=30793536 transcript=Cre12.g504800.t1.2
locus=Cre12.g504800 ID=Cre12.g504800.t1.2.v5.5 annot-version=v5.5
Chlamydomonas_reinhardtii OG0000048
Cre16.g648500.t1.2 pacid=30777221 transcript=Cre16.g648500.t1.2
locus=Cre16.g648500 ID=Cre16.g648500.t1.2.v5.5 annot-version=v5.5
Chlamydomonas_reinhardtii OG0000048
Cre16.g650300.t1.2 pacid=30777639 transcript=Cre16.g650300.t1.2
locus=Cre16.g650300 ID=Cre16.g650300.t1.2.v5.5 annot-version=v5.5
Chlamydomonas_reinhardtii OG0000048
Cre16.g661450.t1.2 pacid=30777754 transcript=Cre16.g661450.t1.2
locus=Cre16.g661450 ID=Cre16.g661450.t1.2.v5.5 annot-version=v5.5
Chlamydomonas_reinhardtii OG0000048
Cre16.g649900.t1.1 pacid=30777869 transcript=Cre16.g649900.t1.1
locus=Cre16.g649900 ID=Cre16.g649900.t1.1.v5.5 annot-version=v5.5
Chlamydomonas_reinhardtii OG0000048
Cre06.g274850.t1.2 pacid=30778497 transcript=Cre06.g274850.t1.2
locus=Cre06.g274850 ID=Cre06.g274850.t1.2.v5.5 annot-version=v5.5
Chlamydomonas_reinhardtii OG0000048
Cre06.g274350.t1.2 pacid=30778588 transcript=Cre06.g274350.t1.2
locus=Cre06.g274350 ID=Cre06.g274350.t1.2.v5.5 annot-version=v5.5
Chlamydomonas_reinhardtii OG0000048
Cre06.g271250.t1.2 pacid=30778696 transcript=Cre06.g271250.t1.2
locus=Cre06.g271250 ID=Cre06.g271250.t1.2.v5.5 annot-version=v5.5
Chlamydomonas_reinhardtii OG0000048
Cre06.g268350.t1.2 pacid=30778762 transcript=Cre06.g268350.t1.2
locus=Cre06.g268350 ID=Cre06.g268350.t1.2.v5.5 annot-version=v5.5
Chlamydomonas_reinhardtii OG0000048
Cre06.g265000.t1.2 pacid=30778874 transcript=Cre06.g265000.t1.2
locus=Cre06.g265000 ID=Cre06.g265000.t1.2.v5.5 annot-version=v5.5
Chlamydomonas_reinhardtii OG0000048
Cre06.g265500.t1.2 pacid=30778907 transcript=Cre06.g265500.t1.2
locus=Cre06.g265500 ID=Cre06.g265500.t1.2.v5.5 annot-version=v5.5
Chlamydomonas_reinhardtii OG0000048
Cre06.g276850.t1.2 pacid=30778983 transcript=Cre06.g276850.t1.2
locus=Cre06.g276850 ID=Cre06.g276850.t1.2.v5.5 annot-version=v5.5
Chlamydomonas_reinhardtii OG0000048

Cre06.g275750.t1.2 pacid=30779137 transcript=Cre06.g275750.t1.2 locus=Cre06.g275750 ID=Cre06.g275750.t1.2.v5.5 annot-version=v5.5
Chlamydomonas_reinhardtii OG0000048
Cre06.g265250.t1.2 pacid=30779294 transcript=Cre06.g265250.t1.2 locus=Cre06.g265250 ID=Cre06.g265250.t1.2.v5.5 annot-version=v5.5
Chlamydomonas_reinhardtii OG0000048
Cre06.g267950.t1.2 pacid=30779543 transcript=Cre06.g267950.t1.2 locus=Cre06.g267950 ID=Cre06.g267950.t1.2.v5.5 annot-version=v5.5
Chlamydomonas_reinhardtii OG0000048
Cre06.g276600.t1.2 pacid=30779595 transcript=Cre06.g276600.t1.2 locus=Cre06.g276600 ID=Cre06.g276600.t1.2.v5.5 annot-version=v5.5
Chlamydomonas_reinhardtii OG0000048
Cre06.g274101.t1.1 pacid=30779608 transcript=Cre06.g274101.t1.1 locus=Cre06.g274101 ID=Cre06.g274101.t1.1.v5.5 annot-version=v5.5
Chlamydomonas_reinhardtii OG0000048
Cre06.g266650.t1.2 pacid=30779741 transcript=Cre06.g266650.t1.2 locus=Cre06.g266650 ID=Cre06.g266650.t1.2.v5.5 annot-version=v5.5
Chlamydomonas_reinhardtii OG0000048
Cre06.g274000.t1.2 pacid=30779747 transcript=Cre06.g274000.t1.2 locus=Cre06.g274000 ID=Cre06.g274000.t1.2.v5.5 annot-version=v5.5
Chlamydomonas_reinhardtii OG0000048
Cre06.g264650.t1.2 pacid=30779911 transcript=Cre06.g264650.t1.2 locus=Cre06.g264650 ID=Cre06.g264650.t1.2.v5.5 annot-version=v5.5
Chlamydomonas_reinhardtii OG0000048
Cre17.g710550.t1.2 pacid=30781687 transcript=Cre17.g710550.t1.2 locus=Cre17.g710550 ID=Cre17.g710550.t1.2.v5.5 annot-version=v5.5
Chlamydomonas_reinhardtii OG0000048
Cre17.g713950.t1.2 pacid=30781861 transcript=Cre17.g713950.t1.2 locus=Cre17.g713950 ID=Cre17.g713950.t1.2.v5.5 annot-version=v5.5
Chlamydomonas_reinhardtii OG0000048
Cre17.g711850.t1.2 pacid=30781891 transcript=Cre17.g711850.t1.2 locus=Cre17.g711850 ID=Cre17.g711850.t1.2.v5.5 annot-version=v5.5
Chlamydomonas_reinhardtii OG0000048
Cre17.g708700.t1.2 pacid=30782453 transcript=Cre17.g708700.t1.2 locus=Cre17.g708700 ID=Cre17.g708700.t1.2.v5.5 annot-version=v5.5
Chlamydomonas_reinhardtii OG0000048
Cre17.g713550.t1.2 pacid=30782548 transcript=Cre17.g713550.t1.2 locus=Cre17.g713550 ID=Cre17.g713550.t1.2.v5.5 annot-version=v5.5
Chlamydomonas_reinhardtii OG0000048
Cre17.g709050.t1.2 pacid=30782593 transcript=Cre17.g709050.t1.2 locus=Cre17.g709050 ID=Cre17.g709050.t1.2.v5.5 annot-version=v5.5
Chlamydomonas_reinhardtii OG0000048
Cre17.g708150.t1.2 pacid=30782647 transcript=Cre17.g708150.t1.2 locus=Cre17.g708150 ID=Cre17.g708150.t1.2.v5.5 annot-version=v5.5
Chlamydomonas_reinhardtii OG0000048
Cre17.g714650.t1.2 pacid=30782865 transcript=Cre17.g714650.t1.2 locus=Cre17.g714650 ID=Cre17.g714650.t1.2.v5.5 annot-version=v5.5
Chlamydomonas_reinhardtii OG0000048
Cre13.g569950.t1.2 pacid=30784075 transcript=Cre13.g569950.t1.2 locus=Cre13.g569950 ID=Cre13.g569950.t1.2.v5.5 annot-version=v5.5
Chlamydomonas_reinhardtii OG0000048
Vocar.0011s0307.1.p pacid=32895346 transcript=Vocar.0011s0307.1 locus=Vocar.0011s0307 ID=Vocar.0011s0307.1.v2.1 annot-version=v2.1
Volvox_carteri OG0000048

Vocar.0028s0101.1.p pacid=32895667 transcript=Vocar.0028s0101.1 locus=Vocar.0028s0101 ID=Vocar.0028s0101.1.v2.1 annot-version=v2.1 Volvox_carteri   OG0000048
Vocar.0028s0150.1.p pacid=32895676 transcript=Vocar.0028s0150.1 locus=Vocar.0028s0150 ID=Vocar.0028s0150.1.v2.1 annot-version=v2.1 Volvox_carteri   OG0000048
Vocar.0033s0145.1.p pacid=32886459 transcript=Vocar.0033s0145.1 locus=Vocar.0033s0145 ID=Vocar.0033s0145.1.v2.1 annot-version=v2.1 Volvox_carteri   OG0000048
Vocar.0027s0142.1.p pacid=32887220 transcript=Vocar.0027s0142.1 locus=Vocar.0027s0142 ID=Vocar.0027s0142.1.v2.1 annot-version=v2.1 Volvox_carteri   OG0000048
Vocar.0027s0149.1.p pacid=32887227 transcript=Vocar.0027s0149.1 locus=Vocar.0027s0149 ID=Vocar.0027s0149.1.v2.1 annot-version=v2.1 Volvox_carteri   OG0000048
Vocar.0122s0001.1.p pacid=32887588 transcript=Vocar.0122s0001.1 locus=Vocar.0122s0001 ID=Vocar.0122s0001.1.v2.1 annot-version=v2.1 Volvox_carteri   OG0000048
Vocar.0002s0598.1.p pacid=32891063 transcript=Vocar.0002s0598.1 locus=Vocar.0002s0598 ID=Vocar.0002s0598.1.v2.1 annot-version=v2.1 Volvox_carteri   OG0000048
Vocar.0002s0595.1.p pacid=32891186 transcript=Vocar.0002s0595.1 locus=Vocar.0002s0595 ID=Vocar.0002s0595.1.v2.1 annot-version=v2.1 Volvox_carteri   OG0000048
Vocar.0042s0089.1.p pacid=32892042 transcript=Vocar.0042s0089.1 locus=Vocar.0042s0089 ID=Vocar.0042s0089.1.v2.1 annot-version=v2.1 Volvox_carteri   OG0000048
Vocar.0042s0091.1.p pacid=32892098 transcript=Vocar.0042s0091.1 locus=Vocar.0042s0091 ID=Vocar.0042s0091.1.v2.1 annot-version=v2.1 Volvox_carteri   OG0000048
Vocar.0006s0042.1.p pacid=32894081 transcript=Vocar.0006s0042.1 locus=Vocar.0006s0042 ID=Vocar.0006s0042.1.v2.1 annot-version=v2.1 Volvox_carteri   OG0000048
Vocar.0006s0183.1.p pacid=32894260 transcript=Vocar.0006s0183.1 locus=Vocar.0006s0183 ID=Vocar.0006s0183.1.v2.1 annot-version=v2.1 Volvox_carteri   OG0000048
jgi|ChlNC64A_1|24354|e_gw1.12.442.1   Chlorella_variabilis OG0000048
jgi|ChlNC64A_1|48847|fgenesh3_pm.C_scaffold_11000024 Chlorella_variabilis   OG0000048
jgi|ChlNC64A_1|56611|estExt_fgenesh3_pg.C_10377 Chlorella_variabilis   OG0000048
jgi|ChlNC64A_1|20865|e_gw1.5.437.1   Chlorella_variabilis OG0000048
jgi|ChlNC64A_1|141496|IGS.gm_35_00008   Chlorella_variabilis OG0000048
jgi|ChlNC64A_1|141512|IGS.gm_35_00024   Chlorella_variabilis OG0000048
jgi|ChlNC64A_1|59160|estExt_fgenesh3_pg.C_270047 Chlorella_variabilis   OG0000048
jgi|ChlNC64A_1|27828|e_gw1.28.116.1   Chlorella_variabilis OG0000048
jgi|ChlNC64A_1|27880|e_gw1.28.134.1   Chlorella_variabilis OG0000048

jgi|ChlNC64A_1|139997|IGS.gm_28_00028	Chlorella_variabilis
OG0000048
jgi|ChlNC64A_1|28967|e_gw1.138.2.1	Chlorella_variabilis
OG0000048
41817 pacid=27386860 transcript=41817 locus=fgenesh1_pg.7_#_221
ID=41817.2.0.227 annot-version=v2.0	Coccomyxa_subellipsoidea
OG0000048
24064 pacid=27388832 transcript=24064 locus=estExt_Genewise1.C_90313
ID=24064.2.0.227 annot-version=v2.0	Coccomyxa_subellipsoidea
OG0000048
42902 pacid=27392024 transcript=42902 locus=fgenesh1_pg.11_#_110
ID=42902.2.0.227 annot-version=v2.0	Coccomyxa_subellipsoidea
OG0000048
17246 pacid=27392049 transcript=17246 locus=e_gw1.11.405.1
ID=17246.2.0.227 annot-version=v2.0	Coccomyxa_subellipsoidea
OG0000048
44881 pacid=27392790 transcript=44881 locus=fgenesh1_pg.20_#_80
ID=44881.2.0.227 annot-version=v2.0	Coccomyxa_subellipsoidea
OG0000048
26007 pacid=27392800 transcript=26007
locus=estExt_Genewise1.C_200192 ID=26007.2.0.227 annot-version=v2.0
Coccomyxa_subellipsoidea OG0000048
44903 pacid=27392886 transcript=44903 locus=fgenesh1_pg.20_#_102
ID=44903.2.0.227 annot-version=v2.0	Coccomyxa_subellipsoidea
OG0000048
38357 pacid=27392964 transcript=38357 locus=fgenesh1_pm.20_#_49
ID=38357.2.0.227 annot-version=v2.0	Coccomyxa_subellipsoidea
OG0000048
31070 pacid=27394487 transcript=31070
locus=estExt_Genewise1Plus.C_180128 ID=31070.2.0.227 annot-
version=v2.0	Coccomyxa_subellipsoidea OG0000048
15107_e_gw1.01.00.648.1	Ostreococcus_tauri	OG0000048
19980_e_gw1.11.00.328.1	Ostreococcus_tauri	OG0000048
22907_estExt_fgenesh1_pm.C_Chr_08.00010003	Ostreococcus_tauri
OG0000048
8460_fgenesh1_pm.C_Chr_05.0001000054	Ostreococcus_tauri
OG0000048
38934 pacid=27416735 transcript=38934 locus=e_gwEuk.5.521.1
ID=38934.2.0.231 annot-version=v2.0	Ostreococcus_lucimarinus
OG0000048
29021 pacid=27418050 transcript=29021 locus=eugene.0100010415
ID=29021.2.0.231 annot-version=v2.0	Ostreococcus_lucimarinus
OG0000048
32891 pacid=27419513 transcript=32891 locus=eugene.0800010017
ID=32891.2.0.231 annot-version=v2.0	Ostreococcus_lucimarinus
OG0000048
17380 pacid=27420656 transcript=17380
locus=fgenesh1_pg.C_Chr_11000182 ID=17380.2.0.231 annot-version=v2.0
	Ostreococcus_lucimarinus OG0000048
48289 pacid=27340169 transcript=48289
locus=MicpuC2.estExt_fgenesh1_pg.C_100195 ID=48289.3.0.228 annot-
version=v3.0	Micromonas_pusilla	OG0000048
194842 pacid=27340326 transcript=194882
locus=estExt_Genemark1.C_100227 ID=194882.3.0.228 annot-version=v3.0

Micromonas_pusilla OG0000048
64879 pacid=27342482 transcript=64879 locus=MicpuC2.est_orfs.
2_3862_4271895:1 ID=64879.3.0.228 annot-version=v3.0
Micromonas_pusilla OG0000048
37404 pacid=27346705 transcript=37404
locus=MicpuC2.fgenesh1_kg.C_scaffold_4000002 ID=37404.3.0.228 annot-
version=v3.0 Micromonas_pusilla OG0000048
123436 pacid=27348389 transcript=123476 locus=fgenesh2_kg.
6_#_83_#_4275702:1 ID=123476.3.0.228 annot-version=v3.0
Micromonas_pusilla OG0000048
93197 pacid=27398766 transcript=93197
locus=estExt_Genewise2Plus.C_Chr_010869 ID=93197.3.0.229 annot-
version=v3.0 Micromonas_sp._RCC299 OG0000048
58772 pacid=27395646 transcript=58772 locus=EuGene.0500010505
ID=58772.3.0.229 annot-version=v3.0 Micromonas_sp._RCC299
OG0000048
63067 pacid=27399595 transcript=63067 locus=EuGene.1200010239
ID=63067.3.0.229 annot-version=v3.0 Micromonas_sp._RCC299
OG0000048
86780 pacid=27399707 transcript=86780 locus=e_gw2.12.608.1
ID=86780.3.0.229 annot-version=v3.0 Micromonas_sp._RCC299
OG0000048
94116 pacid=27395972 transcript=94116
locus=estExt_Genewise2Plus.C_Chr_040928 ID=94116.3.0.229 annot-
version=v3.0 Micromonas_sp._RCC299 OG0000048
kfl00417_0090_v1.1 Klebsormidium_flaccidum OG0000048
kfl00509_0020_v1.1 Klebsormidium_flaccidum OG0000048
kfl00940_0030_v1.1 Klebsormidium_flaccidum OG0000048
kfl01513_g2_v1.1 Klebsormidium_flaccidum OG0000048
kfl00053_0050_v1.1 Klebsormidium_flaccidum OG0000048
kfl00240_g18_v1.1 Klebsormidium_flaccidum OG0000048
Pp3c22_8680V3.1.p pacid=32902897 transcript=Pp3c22_8680V3.1
locus=Pp3c22_8680 annot-version=v3.3 Physcomitrella_patens
OG0000048
Pp3c22_4790V3.1.p pacid=32903367 transcript=Pp3c22_4790V3.1
locus=Pp3c22_4790 annot-version=v3.3 Physcomitrella_patens
OG0000048
Pp3c3_37020V3.1.p pacid=32944693 transcript=Pp3c3_37020V3.1
locus=Pp3c3_37020 annot-version=v3.3 Physcomitrella_patens
OG0000048
Pp3c20_8550V3.1.p pacid=32947024 transcript=Pp3c20_8550V3.1
locus=Pp3c20_8550 annot-version=v3.3 Physcomitrella_patens
OG0000048
Pp3c23_15100V3.1.p pacid=32950872 transcript=Pp3c23_15100V3.1
locus=Pp3c23_15100 annot-version=v3.3 Physcomitrella_patens
OG0000048
Pp3c27_7370V3.1.p pacid=32952164 transcript=Pp3c27_7370V3.1
locus=Pp3c27_7370 annot-version=v3.3 Physcomitrella_patens
OG0000048
Pp3c8_2810V3.1.p pacid=32965445 transcript=Pp3c8_2810V3.1
locus=Pp3c8_2810 annot-version=v3.3 Physcomitrella_patens
OG0000048
Pp3c1_20640V3.1.p pacid=32967507 transcript=Pp3c1_20640V3.1
locus=Pp3c1_20640 annot-version=v3.3 Physcomitrella_patens

OG0000048
Pp3c1_20670V3.1.p pacid=32970628 transcript=Pp3c1_20670V3.1
locus=Pp3c1_20670 annot-version=v3.3 Physcomitrella_patens
OG0000048
Pp3c18_12300V3.1.p pacid=32981456 transcript=Pp3c18_12300V3.1
locus=Pp3c18_12300 annot-version=v3.3 Physcomitrella_patens
OG0000048
Pp3c18_14481V3.1.p pacid=32982567 transcript=Pp3c18_14481V3.1
locus=Pp3c18_14481 annot-version=v3.3 Physcomitrella_patens
OG0000048
Pp3c18_14470V3.1.p pacid=32983129 transcript=Pp3c18_14470V3.1
locus=Pp3c18_14470 annot-version=v3.3 Physcomitrella_patens
OG0000048
Pp3c18_12280V3.1.p pacid=32983256 transcript=Pp3c18_12280V3.1
locus=Pp3c18_12280 annot-version=v3.3 Physcomitrella_patens
OG0000048
Pp3c17_5850V3.1.p pacid=32908048 transcript=Pp3c17_5850V3.1
locus=Pp3c17_5850 annot-version=v3.3 Physcomitrella_patens
OG0000048
Pp3c24_13230V3.1.p pacid=32908994 transcript=Pp3c24_13230V3.1
locus=Pp3c24_13230 annot-version=v3.3 Physcomitrella_patens
OG0000048
Pp3c24_11100V3.1.p pacid=32909624 transcript=Pp3c24_11100V3.1
locus=Pp3c24_11100 annot-version=v3.3 Physcomitrella_patens
OG0000048
AT1G13370.1 | Histone superfamily protein | Chr1:4587926-4588522
REVERSE LENGTH=136 | 201606 Arabidopsis_thaliana
OG0000048
AT3G27360.1 | Histone superfamily protein | Chr3:10129459-10129869
REVERSE LENGTH=136 | 201606 Arabidopsis_thaliana
OG0000048
AT1G19890.1 | male-gamete-specific histone H3 | Chr1:6905034-6905792
FORWARD LENGTH=137 | 201606 Arabidopsis_thaliana
OG0000048
AT4G40030.1 | Histone superfamily protein | Chr4:18555840-18556417
REVERSE LENGTH=136 | 201606 Arabidopsis_thaliana
OG0000048
AT4G40040.1 | Histone superfamily protein | Chr4:18557539-18558236
REVERSE LENGTH=136 | 201606 Arabidopsis_thaliana
OG0000048
AT5G10390.1 | Histone superfamily protein | Chr5:3269065-3269475
REVERSE LENGTH=136 | 201606 Arabidopsis_thaliana
OG0000048
AT5G10400.1 | Histone superfamily protein | Chr5:3270478-3270888
REVERSE LENGTH=136 | 201606 Arabidopsis_thaliana
OG0000048
AT5G10980.1 | Histone superfamily protein | Chr5:3472591-3473349
REVERSE LENGTH=136 | 201606 Arabidopsis_thaliana
OG0000048
AT5G12910.1 | Histone superfamily protein | Chr5:4077162-4077557
FORWARD LENGTH=131 | 201606 Arabidopsis_thaliana
OG0000048
AT5G65350.1 | histone 3 11 | Chr5:26119406-26119825 REVERSE
LENGTH=139 | 201606 Arabidopsis_thaliana OG0000048

AT5G65360.1 | Histone superfamily protein | Chr5:26120099-26120509 REVERSE LENGTH=136 | 201606 Arabidopsis_thaliana OG0000048
AT1G01370.1 | Histone superfamily protein | Chr1:143773-145400 FORWARD LENGTH=178 | 201606 Arabidopsis_thaliana OG0000048
AT1G75600.1 | Histone superfamily protein | Chr1:28390753-28391461 FORWARD LENGTH=136 | 201606 Arabidopsis_thaliana OG0000048
AT1G09200.1 | Histone superfamily protein | Chr1:2971722-2972132 REVERSE LENGTH=136 | 201606 Arabidopsis_thaliana OG0000048
Os04t0419600-01 Histone H3. Oryza_sativa OG0000048
Os04t0450800-01 Similar to Histone H3. Oryza_sativa OG0000048
Os05t0489800-01 Centromeric histone 3 (Histone H3). Oryza_sativa OG0000048
Os06t0130900-00 Similar to Histone H3 (Fragment). Oryza_sativa OG0000048
Os06t0159725-00 Similar to histone H3. Oryza_sativa OG0000048
Os06t0160001-00 Histone H3. Oryza_sativa OG0000048
Os06t0160100-00 Similar to Histones H3 and H4 (ISS). Oryza_sativa OG0000048
Os11t0155900-00 Histone H3. Oryza_sativa OG0000048
Os12t0415800-00 Similar to Histone H3. Oryza_sativa OG0000048
Os02t0457100-00 Similar to Histone H3. Oryza_sativa OG0000048
Os02t0457300-00 Similar to Histone H3. Oryza_sativa OG0000048
CME099CT pep chromosome:ASM9120v1:5:276598:277425:1 gene:CME099C transcript:CME099CT gene_biotype:protein_coding transcript_biotype:protein_coding Cyanidioschyzon_merolae OG0000048
CMN165CT pep chromosome:ASM9120v1:14:444055:444465:1 gene:CMN165C transcript:CMN165CT gene_biotype:protein_coding transcript_biotype:protein_coding Cyanidioschyzon_merolae OG0000048
evm.model.contig_2255.2 Porphyridium_purpureum OG0000048
evm.model.contig_3435.2 Porphyridium_purpureum OG0000048
evm.model.contig_2059.6 Porphyridium_purpureum OG0000048
CDF32207 pep supercontig:ASM35022v2:HG001460:46574:46984:-1 gene:CHC_T00008319001 transcript:CDF32207 gene_biotype:protein_coding transcript_biotype:protein_coding description:Histone H3 Chondrus_crispus OG0000048
CDF35136 pep supercontig:ASM35022v2:HG001718:417793:418203:1 gene:CHC_T00008783001 transcript:CDF35136 gene_biotype:protein_coding transcript_biotype:protein_coding description:histone H3 Chondrus_crispus OG0000048
CDF35733 pep supercontig:ASM35022v2:HG001742:165135:165765:1 gene:CHC_T00009360001 transcript:CDF35733 gene_biotype:protein_coding transcript_biotype:protein_coding description:similar to Histone H3 Chondrus_crispus OG0000048

CDF39944 pep supercontig:ASM35022v2:HG002094:166666:167341:-1
gene:CHC_T00008476001 transcript:CDF39944
gene_biotype:protein_coding transcript_biotype:protein_coding
description:Histone H3 Chondrus_crispus OG0000048
g5459.t1 Caulerpa_lentillifera OG0000049
Cre02.g083354.t1.2 pacid=30785555 transcript=Cre02.g083354.t1.2
locus=Cre02.g083354 ID=Cre02.g083354.t1.2.v5.5 annot-version=v5.5
Chlamydomonas_reinhardtii OG0000049
Cre16.g669525.t1.1 pacid=30777112 transcript=Cre16.g669525.t1.1
locus=Cre16.g669525 ID=Cre16.g669525.t1.1.v5.5 annot-version=v5.5
Chlamydomonas_reinhardtii OG0000049
Cre16.g692300.t1.2 pacid=30777566 transcript=Cre16.g692300.t1.2
locus=Cre16.g692300 ID=Cre16.g692300.t1.2.v5.5 annot-version=v5.5
Chlamydomonas_reinhardtii OG0000049
Cre17.g725150.t1.2 pacid=30782210 transcript=Cre17.g725150.t1.2
locus=Cre17.g725150 ID=Cre17.g725150.t1.2.v5.5 annot-version=v5.5
Chlamydomonas_reinhardtii OG0000049
Cre17.g725200.t1.2 pacid=30782751 transcript=Cre17.g725200.t1.2
locus=Cre17.g725200 ID=Cre17.g725200.t1.2.v5.5 annot-version=v5.5
Chlamydomonas_reinhardtii OG0000049
Vocar.0028s0139.1.p pacid=32895550 transcript=Vocar.0028s0139.1
locus=Vocar.0028s0139 ID=Vocar.0028s0139.1.v2.1 annot-version=v2.1
Volvox_carteri OG0000049
Vocar.0028s0140.1.p pacid=32895649 transcript=Vocar.0028s0140.1
locus=Vocar.0028s0140 ID=Vocar.0028s0140.1.v2.1 annot-version=v2.1
Volvox_carteri OG0000049
Vocar.0023s0168.1.p pacid=32883330 transcript=Vocar.0023s0168.1
locus=Vocar.0023s0168 ID=Vocar.0023s0168.1.v2.1 annot-version=v2.1
Volvox_carteri OG0000049
Vocar.0001s1440.1.p pacid=32884741 transcript=Vocar.0001s1440.1
locus=Vocar.0001s1440 ID=Vocar.0001s1440.1.v2.1 annot-version=v2.1
Volvox_carteri OG0000049
Vocar.0032s0010.1.p pacid=32893720 transcript=Vocar.0032s0010.1
locus=Vocar.0032s0010 ID=Vocar.0032s0010.1.v2.1 annot-version=v2.1
Volvox_carteri OG0000049
jgi|ChlNC64A_1|34209|estExt_Genewise1Plus.C_30589
Chlorella_variabilis OG0000049
jgi|ChlNC64A_1|36019|estExt_Genewise1Plus.C_140087
Chlorella_variabilis OG0000049
jgi|ChlNC64A_1|36050|estExt_Genewise1Plus.C_140173
Chlorella_variabilis OG0000049
jgi|ChlNC64A_1|135696|IGS.gm_14_00229 Chlorella_variabilis
OG0000049
jgi|ChlNC64A_1|31851|estExt_Genewise1.C_140411
Chlorella_variabilis OG0000049
jgi|ChlNC64A_1|51094|fgenesh3_pg.C_scaffold_5000261
Chlorella_variabilis OG0000049
jgi|ChlNC64A_1|36831|estExt_Genewise1Plus.C_220081
Chlorella_variabilis OG0000049
jgi|ChlNC64A_1|135150|IGS.gm_13_00078 Chlorella_variabilis
OG0000049
36464 pacid=27386668 transcript=36464 locus=fgenesh1_pm.7_#_80
ID=36464.2.0.227 annot-version=v2.0 Coccomyxa_subellipsoidea
OG0000049

67208 pacid=27387983 transcript=67208
locus=estExt_Genemark1.C_140109 ID=67208.2.0.227 annot-version=v2.0
Coccomyxa_subellipsoidea OG0000049
38257 pacid=27388361 transcript=38257 locus=fgenesh1_pm.19_#_79
ID=38257.2.0.227 annot-version=v2.0 Coccomyxa_subellipsoidea
OG0000049
47758 pacid=27385970 transcript=47758
locus=estExt_fgenesh1_pg.C_100022 ID=47758.2.0.227 annot-
version=v2.0 Coccomyxa_subellipsoidea OG0000049
21191 pacid=27385640 transcript=21191 locus=e_gw1.45.5.1
ID=21191.2.0.227 annot-version=v2.0 Coccomyxa_subellipsoidea
OG0000049
21188 pacid=27385641 transcript=21188 locus=e_gw1.45.1.1
ID=21188.2.0.227 annot-version=v2.0 Coccomyxa_subellipsoidea
OG0000049
35186 pacid=27393110 transcript=35186 locus=fgenesh1_pm.2_#_196
ID=35186.2.0.227 annot-version=v2.0 Coccomyxa_subellipsoidea
OG0000049
18274_e_gw1.07.00.69.1 Ostreococcus_tauri OG0000049
27444_estExt_gwp_GeneWisePlus.C_Chr_01.00010580
Ostreococcus_tauri OG0000049
49993 pacid=27414401 transcript=49993
locus=estExt_Genewise_ext.C_Chr_70132 ID=49993.2.0.231 annot-
version=v2.0 Ostreococcus_lucimarinus OG0000049
48483 pacid=27418133 transcript=48483
locus=estExt_Genewise_ext.C_Chr_10621 ID=48483.2.0.231 annot-
version=v2.0 Ostreococcus_lucimarinus OG0000049
20532 pacid=27340199 transcript=20532 locus=MicpuC2.e_gw1.10.54.1
ID=20532.3.0.228 annot-version=v3.0 Micromonas_pusilla
OG0000049
27897 pacid=27338922 transcript=27897
locus=MicpuC2.estExt_Genewise1.C_80212 ID=27897.3.0.228 annot-
version=v3.0 Micromonas_pusilla OG0000049
19395 pacid=27338974 transcript=19395 locus=MicpuC2.e_gw1.8.70.1
ID=19395.3.0.228 annot-version=v3.0 Micromonas_pusilla
OG0000049
15544 pacid=27347094 transcript=15544 locus=MicpuC2.e_gw1.3.1002.1
ID=15544.3.0.228 annot-version=v3.0 Micromonas_pusilla
OG0000049
203551 pacid=27347483 transcript=203591 locus=fgenesh1_pm.3_#_2
ID=203591.3.0.228 annot-version=v3.0 Micromonas_pusilla
OG0000049
25591 pacid=27347576 transcript=25591
locus=MicpuC2.estExt_Genewise1.C_30332 ID=25591.3.0.228 annot-
version=v3.0 Micromonas_pusilla OG0000049
66493 pacid=27398661 transcript=66493 locus=gw2.03.106.1
ID=66493.3.0.229 annot-version=v3.0 Micromonas_sp._RCC299
OG0000049
98162 pacid=27399934 transcript=98162
locus=fgenesh2_pm.C_Chr_12000042 ID=98162.3.0.229 annot-version=v3.0
Micromonas_sp._RCC299 OG0000049
87744 pacid=27402632 transcript=87744 locus=e_gw2.13.70.1
ID=87744.3.0.229 annot-version=v3.0 Micromonas_sp._RCC299
OG0000049

87670 pacid=27402733 transcript=87670 locus=e_gw2.13.83.1 ID=87670.3.0.229 annot-version=v3.0	Micromonas_sp._RCC299 OG0000049
88240 pacid=27403911 transcript=88240 locus=e_gw2.14.65.1 ID=88240.3.0.229 annot-version=v3.0	Micromonas_sp._RCC299 OG0000049
75054 pacid=27405156 transcript=75054 locus=gw2.11.610.1 ID=75054.3.0.229 annot-version=v3.0	Micromonas_sp._RCC299 OG0000049
62621 pacid=27405164 transcript=62621 locus=EuGene.1100010407 ID=62621.3.0.229 annot-version=v3.0	Micromonas_sp._RCC299 OG0000049
kfl00016_0070_v1.1	Klebsormidium_flaccidum	OG0000049
kfl00461_0020_v1.1	Klebsormidium_flaccidum	OG0000049
kfl00544_0080_v1.1	Klebsormidium_flaccidum	OG0000049
kfl00023_0200_v1.1	Klebsormidium_flaccidum	OG0000049
kfl00979_0020_v1.1	Klebsormidium_flaccidum	OG0000049
kfl00047_0060_v1.1	Klebsormidium_flaccidum	OG0000049
kfl00008_0360_v1.1	Klebsormidium_flaccidum	OG0000049
kfl00168_0060_v1.1	Klebsormidium_flaccidum	OG0000049
kfl00277_0100_v1.1	Klebsormidium_flaccidum	OG0000049
Pp3c2_13930V3.1.p pacid=32934293 transcript=Pp3c2_13930V3.1 locus=Pp3c2_13930 annot-version=v3.3	Physcomitrella_patens OG0000049
Pp3c2_36840V3.1.p pacid=32934693 transcript=Pp3c2_36840V3.1 locus=Pp3c2_36840 annot-version=v3.3	Physcomitrella_patens OG0000049
Pp3c19_4510V3.1.p pacid=32939394 transcript=Pp3c19_4510V3.1 locus=Pp3c19_4510 annot-version=v3.3	Physcomitrella_patens OG0000049
Pp3c3_5680V3.1.p pacid=32944418 transcript=Pp3c3_5680V3.1 locus=Pp3c3_5680 annot-version=v3.3	Physcomitrella_patens OG0000049
Pp3c22_11240V3.1.p pacid=32904708 transcript=Pp3c22_11240V3.1 locus=Pp3c22_11240 annot-version=v3.3	Physcomitrella_patens OG0000049
Pp3c14_2470V3.1.p pacid=32960752 transcript=Pp3c14_2470V3.1 locus=Pp3c14_2470 annot-version=v3.3	Physcomitrella_patens OG0000049
Pp3c8_6290V3.1.p pacid=32964704 transcript=Pp3c8_6290V3.1 locus=Pp3c8_6290 annot-version=v3.3	Physcomitrella_patens OG0000049
Pp3c1_7210V3.1.p pacid=32967034 transcript=Pp3c1_7210V3.1 locus=Pp3c1_7210 annot-version=v3.3	Physcomitrella_patens OG0000049
Pp3c1_7215V3.1.p pacid=32968147 transcript=Pp3c1_7215V3.1 locus=Pp3c1_7215 annot-version=v3.3	Physcomitrella_patens OG0000049
Pp3c1_34680V3.1.p pacid=32969529 transcript=Pp3c1_34680V3.1 locus=Pp3c1_34680 annot-version=v3.3	Physcomitrella_patens OG0000049
Pp3c1_31680V3.1.p pacid=32969582 transcript=Pp3c1_31680V3.1 locus=Pp3c1_31680 annot-version=v3.3	Physcomitrella_patens OG0000049

Pp3c17_16450V3.1.p pacid=32906756 transcript=Pp3c17_16450V3.1 locus=Pp3c17_16450 annot-version=v3.3   Physcomitrella_patens OG0000049
Pp3c12_11230V3.1.p pacid=32973503 transcript=Pp3c12_11230V3.1 locus=Pp3c12_11230 annot-version=v3.3   Physcomitrella_patens OG0000049
Pp3c12_14200V3.1.p pacid=32973756 transcript=Pp3c12_14200V3.1 locus=Pp3c12_14200 annot-version=v3.3   Physcomitrella_patens OG0000049
Pp3c6_20190V3.1.p pacid=32975929 transcript=Pp3c6_20190V3.1 locus=Pp3c6_20190 annot-version=v3.3   Physcomitrella_patens OG0000049
Pp3c6_7260V3.1.p pacid=32978672 transcript=Pp3c6_7260V3.1 locus=Pp3c6_7260 annot-version=v3.3   Physcomitrella_patens OG0000049
Pp3c16_7170V3.1.p pacid=32984920 transcript=Pp3c16_7170V3.1 locus=Pp3c16_7170 annot-version=v3.3   Physcomitrella_patens OG0000049
Pp3c24_8380V3.1.p pacid=32910173 transcript=Pp3c24_8380V3.1 locus=Pp3c24_8380 annot-version=v3.3   Physcomitrella_patens OG0000049
Pp3c9_13390V3.1.p pacid=32911138 transcript=Pp3c9_13390V3.1 locus=Pp3c9_13390 annot-version=v3.3   Physcomitrella_patens OG0000049
Pp3c21_230V3.1.p pacid=32914668 transcript=Pp3c21_230V3.1 locus=Pp3c21_230 annot-version=v3.3   Physcomitrella_patens OG0000049
Pp3c21_18190V3.1.p pacid=32915783 transcript=Pp3c21_18190V3.1 locus=Pp3c21_18190 annot-version=v3.3   Physcomitrella_patens OG0000049
Pp3c21_243V3.1.p pacid=32916690 transcript=Pp3c21_243V3.1 locus=Pp3c21_243 annot-version=v3.3   Physcomitrella_patens OG0000049
Pp3c4_5170V3.1.p pacid=32920156 transcript=Pp3c4_5170V3.1 locus=Pp3c4_5170 annot-version=v3.3   Physcomitrella_patens OG0000049
AT2G36910.1 | ATP binding cassette subfamily B1 | Chr2:15502162-15507050 FORWARD LENGTH=1286 | 201606 Arabidopsis_thaliana   OG0000049
AT1G10680.1 | P-glycoprotein 10 | Chr1:3538470-3543782 REVERSE LENGTH=1227 | 201606   Arabidopsis_thaliana   OG0000049
AT2G39480.1 | P-glycoprotein 6 | Chr2:16478249-16484827 REVERSE LENGTH=1407 | 201606   Arabidopsis_thaliana   OG0000049
AT2G47000.1 | ATP binding cassette subfamily B4 | Chr2:19310008-19314750 REVERSE LENGTH=1286 | 201606 Arabidopsis_thaliana   OG0000049
AT3G28345.1 | ABC transporter family protein | Chr3:10593921-10598775 REVERSE LENGTH=1240 | 201606 Arabidopsis_thaliana   OG0000049
AT3G28360.1 | P-glycoprotein 16 | Chr3:10611071-10616301 REVERSE LENGTH=1228 | 201606   Arabidopsis_thaliana   OG0000049
AT3G28380.1 | P-glycoprotein 17 | Chr3:10623742-10628201 REVERSE LENGTH=1240 | 201606   Arabidopsis_thaliana   OG0000049
AT3G28390.1 | P-glycoprotein 18 | Chr3:10629425-10633967 REVERSE

LENGTH=1225 | 201606   Arabidopsis_thaliana   OG0000049
AT3G28415.1 | ABC transporter family protein |
Chr3:10647123-10651540 REVERSE LENGTH=1221 | 201606
Arabidopsis_thaliana   OG0000049
AT3G28860.1 | ATP binding cassette subfamily B19 |
Chr3:10870287-10877286 REVERSE LENGTH=1252 | 201606
Arabidopsis_thaliana   OG0000049
AT1G02520.1 | P-glycoprotein 11 | Chr1:524134-528745 FORWARD
LENGTH=1278 | 201606   Arabidopsis_thaliana   OG0000049
AT1G02530.1 | P-glycoprotein 12 | Chr1:529836-534542 FORWARD
LENGTH=1273 | 201606   Arabidopsis_thaliana   OG0000049
AT3G55320.1 | P-glycoprotein 20 | Chr3:20507391-20513393 REVERSE
LENGTH=1408 | 201606   Arabidopsis_thaliana   OG0000049
AT3G62150.1 | P-glycoprotein 21 | Chr3:23008755-23013579 REVERSE
LENGTH=1296 | 201606   Arabidopsis_thaliana   OG0000049
AT4G01820.1 | P-glycoprotein 3 | Chr4:780734-785329 REVERSE
LENGTH=1229 | 201606   Arabidopsis_thaliana   OG0000049
AT4G01830.1 | P-glycoprotein 5 | Chr4:785683-790447 REVERSE
LENGTH=1230 | 201606   Arabidopsis_thaliana   OG0000049
AT4G18050.1 | P-glycoprotein 9 | Chr4:10022205-10027280 FORWARD
LENGTH=1236 | 201606   Arabidopsis_thaliana   OG0000049
AT4G25450.1 | non-intrinsic ABC protein 8 | Chr4:13009845-13013912
REVERSE LENGTH=714 | 201606   Arabidopsis_thaliana
OG0000049
AT4G25960.1 | P-glycoprotein 2 | Chr4:13177438-13183425 FORWARD
LENGTH=1273 | 201606   Arabidopsis_thaliana   OG0000049
AT5G03910.1 | ABC2 homolog 12 | Chr5:1054313-1057105 REVERSE
LENGTH=634 | 201606   Arabidopsis_thaliana   OG0000049
AT5G39040.1 | transporter associated with antigen processing protein
2 | Chr5:15625660-15629621 FORWARD LENGTH=644 | 201606
Arabidopsis_thaliana   OG0000049
AT5G46540.1 | P-glycoprotein 7 | Chr5:18877192-18882347 REVERSE
LENGTH=1248 | 201606   Arabidopsis_thaliana   OG0000049
AT1G27940.1 | P-glycoprotein 13 | Chr1:9733597-9738129 REVERSE
LENGTH=1245 | 201606   Arabidopsis_thaliana   OG0000049
AT1G28010.1 | P-glycoprotein 14 | Chr1:9763436-9767917 FORWARD
LENGTH=1247 | 201606   Arabidopsis_thaliana   OG0000049
AT1G70610.1 | transporter associated with antigen processing protein
1 | Chr1:26622086-26626331 FORWARD LENGTH=700 | 201606
Arabidopsis_thaliana   OG0000049
Os03t0280000-01 Similar to MDR-like ABC transporter.
Oryza_sativa   OG0000049
Os03t0755100-01 Half-size ABC transporter, Internal detoxification
of aluminum   Oryza_sativa   OG0000049
Os01t0290700-01 Similar to CjMDR1.   Oryza_sativa
OG0000049
Os04t0413000-00 Similar to OSIGBa0134P10.6 protein.
Oryza_sativa   OG0000049
Os04t0459000-01 Multidrug-resistance(MDR)-like ATP Binding Cassette
B (ABCB) transporter, P-glyco-protein, Auxin transport, Iron
homeostasis   Oryza_sativa   OG0000049
Os04t0481700-01 Similar to MDR-like ABC transporter.
Oryza_sativa   OG0000049
Os04t0642000-01 Similar to MDR-like p-glycoprotein.

Oryza_sativa	OG0000049
Os05t0137100-01 Similar to MDR-like ABC transporter.
Oryza_sativa	OG0000049
Os05t0137200-01 Similar to P-glycoprotein ABCB5.	Oryza_sativa
OG0000049
Os05t0548300-01 ABC transporter, transmembrane domain domain
containing protein.	Oryza_sativa	OG0000049
Os05t0548500-00 Similar to MDR-like ABC transporter.
Oryza_sativa	OG0000049
Os01t0533900-01 Similar to Multidrug resistance protein 1 homolog.
Oryza_sativa	OG0000049
Os01t0534700-00 ABC transporter, transmembrane domain domain
containing protein.	Oryza_sativa	OG0000049
Os07t0464600-01 ABC transporter-like domain containing protein.
Oryza_sativa	OG0000049
Os08t0153000-00 Similar to MDR-like ABC transporter.
Oryza_sativa	OG0000049
Os08t0564300-01 ABC transporter-like domain containing protein.
Oryza_sativa	OG0000049
Os01t0695700-00 Similar to MDR-like ABC transporter.
Oryza_sativa	OG0000049
Os01t0695800-01 Similar to MDR-like ABC transporter.
Oryza_sativa	OG0000049
Os01t0723800-01 Similar to P-glycoprotein homologue.
Oryza_sativa	OG0000049
Os01t0911300-01 Similar to Transporter associated with antigen
processing-like protein.	Oryza_sativa	OG0000049
Os01t0976100-01 ABC transporter-like domain containing protein.
Oryza_sativa	OG0000049
Os02t0189800-00 Similar to Felis catus multi-drug resistance related
(Fragment).	Oryza_sativa	OG0000049
Os02t0190000-00 Putative uncharacterized protein.	Oryza_sativa
OG0000049
Os02t0190300-01 P-glycoprotein homologue.	Oryza_sativa
OG0000049
Os02t0323000-01 Similar to Felis catus multi-drug resistance related
(Fragment).	Oryza_sativa	OG0000049
Os02t0693700-01 Hypothetical conserved gene.	Oryza_sativa
OG0000049
Os03t0181675-00 ABC transporter, transmembrane domain domain
containing protein.	Oryza_sativa	OG0000049
Os03t0181750-00 ABC transporter, transmembrane domain domain
containing protein.	Oryza_sativa	OG0000049
CMN323CT pep chromosome:ASM9120v1:14:804458:805879:1 gene:CMN323C
transcript:CMN323CT gene_biotype:protein_coding
transcript_biotype:protein_coding Cyanidioschyzon_merolae
OG0000049
CMN325CT pep chromosome:ASM9120v1:14:803294:805885:1 gene:CMN325C
transcript:CMN325CT gene_biotype:protein_coding
transcript_biotype:protein_coding Cyanidioschyzon_merolae
OG0000049
CMS401CT pep chromosome:ASM9120v1:19:1002706:1003996:-1 gene:CMS401C
transcript:CMS401CT gene_biotype:protein_coding
transcript_biotype:protein_coding Cyanidioschyzon_merolae

OG0000049
CMO105CT pep chromosome:ASM9120v1:15:268161:270171:-1 gene:CMO105C transcript:CMO105CT gene_biotype:protein_coding transcript_biotype:protein_coding Cyanidioschyzon_merolae OG0000049
CMD148CT pep chromosome:ASM9120v1:4:386616:388707:-1 gene:CMD148C transcript:CMD148CT gene_biotype:protein_coding transcript_biotype:protein_coding description:transporter associated with antigen processing protein 2 [Source:Projected from Arabidopsis thaliana (AT5G39040) TAIR;Acc:AT5G39040] Cyanidioschyzon_merolae OG0000049
evm.model.contig_2303.6 Porphyridium_purpureum OG0000049
evm.model.contig_3487.3 Porphyridium_purpureum OG0000049
evm.model.contig_4436.1 Porphyridium_purpureum OG0000049
evm.model.contig_618.5 Porphyridium_purpureum OG0000049
CDF36620 pep supercontig:ASM35022v2:HG001793:99843:101088:-1 gene:CHC_T00005019001 transcript:CDF36620 gene_biotype:protein_coding transcript_biotype:protein_coding Chondrus_crispus OG0000049
CDF33040 pep supercontig:ASM35022v2:HG001631:241923:243116:1 gene:CHC_T00001929001 transcript:CDF33040 gene_biotype:protein_coding transcript_biotype:protein_coding Chondrus_crispus OG0000049
CDF36232 pep supercontig:ASM35022v2:HG001767:144747:146204:1 gene:CHC_T00004718001 transcript:CDF36232 gene_biotype:protein_coding transcript_biotype:protein_coding Chondrus_crispus OG0000049
CDF39648 pep supercontig:ASM35022v2:HG002060:374266:376413:1 gene:CHC_T00006824001 transcript:CDF39648 gene_biotype:protein_coding transcript_biotype:protein_coding Chondrus_crispus OG0000049
CDF40488 pep supercontig:ASM35022v2:HG002190:11361:12818:1 gene:CHC_T00000757001 transcript:CDF40488 gene_biotype:protein_coding transcript_biotype:protein_coding Chondrus_crispus OG0000049
CDF37870 pep supercontig:ASM35022v2:HG001882:57971:62854:-1 gene:CHC_T00006026001 transcript:CDF37870 gene_biotype:protein_coding transcript_biotype:protein_coding Chondrus_crispus OG0000049
CDF33364 pep supercontig:ASM35022v2:HG001642:332532:336939:1 gene:CHC_T00001951001 transcript:CDF33364 gene_biotype:protein_coding transcript_biotype:protein_coding Chondrus_crispus OG0000049
CDF40343 pep supercontig:ASM35022v2:HG002157:4799:6055:-1 gene:CHC_T00000701001 transcript:CDF40343 gene_biotype:protein_coding transcript_biotype:protein_coding Chondrus_crispus OG0000049
CDF39970 pep supercontig:ASM35022v2:HG002094:341676:346103:-1 gene:CHC_T00006926001 transcript:CDF39970 gene_biotype:protein_coding transcript_biotype:protein_coding Chondrus_crispus OG0000049
CDF38575 pep supercontig:ASM35022v2:HG001947:62967:66507:1 gene:CHC_T00001159001 transcript:CDF38575 gene_biotype:protein_coding transcript_biotype:protein_coding

Chondrus_crispus OG0000049
CDF77442 pep supercontig:ASM35022v2:HG001523:407551:410205:1
gene:CHC_T00007944001 transcript:CDF77442
gene_biotype:protein_coding transcript_biotype:protein_coding
Chondrus_crispus OG0000049
CDF34924 pep supercontig:ASM35022v2:HG001710:163112:164149:1
gene:CHC_T00003552001 transcript:CDF34924
gene_biotype:protein_coding transcript_biotype:protein_coding
Chondrus_crispus OG0000049
Cre11.g467577.t1.1 pacid=30775785 transcript=Cre11.g467577.t1.1
locus=Cre11.g467577 ID=Cre11.g467577.t1.1.v5.5 annot-version=v5.5
Chlamydomonas_reinhardtii OG0000050
Vocar.0014s0224.1.p pacid=32888487 transcript=Vocar.0014s0224.1
locus=Vocar.0014s0224 ID=Vocar.0014s0224.1.v2.1 annot-version=v2.1
Volvox_carteri OG0000050
16128 pacid=27388790 transcript=16128 locus=e_gw1.9.373.1
ID=16128.2.0.227 annot-version=v2.0 Coccomyxa_subellipsoidea
OG0000050
15144_e_gw1.01.00.699.1 Ostreococcus_tauri OG0000050
29140 pacid=27417846 transcript=29140 locus=eugene.0100010534
ID=29140.2.0.231 annot-version=v2.0 Ostreococcus_lucimarinus
OG0000050
21861 pacid=27339865 transcript=21861 locus=MicpuC2.e_gw1.13.374.1
ID=21861.3.0.228 annot-version=v3.0 Micromonas_pusilla
OG0000050
64870 pacid=27397041 transcript=64870 locus=EuGene.1600010231
ID=64870.3.0.229 annot-version=v3.0 Micromonas_sp._RCC299
OG0000050
kfl00560_0030_v1.1 Klebsormidium_flaccidum OG0000050
Pp3c3_33360V3.1.p pacid=32940851 transcript=Pp3c3_33360V3.1
locus=Pp3c3_33360 annot-version=v3.3 Physcomitrella_patens
OG0000050
Pp3c3_33370V3.1.p pacid=32941386 transcript=Pp3c3_33370V3.1
locus=Pp3c3_33370 annot-version=v3.3 Physcomitrella_patens
OG0000050
Pp3c3_31980V3.1.p pacid=32941681 transcript=Pp3c3_31980V3.1
locus=Pp3c3_31980 annot-version=v3.3 Physcomitrella_patens
OG0000050
Pp3c3_31770V3.1.p pacid=32942260 transcript=Pp3c3_31770V3.1
locus=Pp3c3_31770 annot-version=v3.3 Physcomitrella_patens
OG0000050
Pp3c3_31810V3.1.p pacid=32942372 transcript=Pp3c3_31810V3.1
locus=Pp3c3_31810 annot-version=v3.3 Physcomitrella_patens
OG0000050
Pp3c14_14900V3.1.p pacid=32960083 transcript=Pp3c14_14900V3.1
locus=Pp3c14_14900 annot-version=v3.3 Physcomitrella_patens
OG0000050
Pp3c14_22180V3.1.p pacid=32960620 transcript=Pp3c14_22180V3.1
locus=Pp3c14_22180 annot-version=v3.3 Physcomitrella_patens
OG0000050
Pp3c14_22210V3.1.p pacid=32962519 transcript=Pp3c14_22210V3.1
locus=Pp3c14_22210 annot-version=v3.3 Physcomitrella_patens
OG0000050
Pp3c8_6890V3.1.p pacid=32964827 transcript=Pp3c8_6890V3.1

locus=Pp3c8_6890 annot-version=v3.3 Physcomitrella_patens
OG0000050
Pp3c8_6920V3.1.p pacid=32966076 transcript=Pp3c8_6920V3.1
locus=Pp3c8_6920 annot-version=v3.3 Physcomitrella_patens
OG0000050
Pp3c1_39760V3.1.p pacid=32967315 transcript=Pp3c1_39760V3.1
locus=Pp3c1_39760 annot-version=v3.3 Physcomitrella_patens
OG0000050
Pp3c1_30500V3.1.p pacid=32968044 transcript=Pp3c1_30500V3.1
locus=Pp3c1_30500 annot-version=v3.3 Physcomitrella_patens
OG0000050
Pp3c17_24040V3.1.p pacid=32906515 transcript=Pp3c17_24040V3.1
locus=Pp3c17_24040 annot-version=v3.3 Physcomitrella_patens
OG0000050
Pp3c1_39780V3.1.p pacid=32969851 transcript=Pp3c1_39780V3.1
locus=Pp3c1_39780 annot-version=v3.3 Physcomitrella_patens
OG0000050
Pp3c12_22890V3.1.p pacid=32972793 transcript=Pp3c12_22890V3.1
locus=Pp3c12_22890 annot-version=v3.3 Physcomitrella_patens
OG0000050
Pp3c12_22500V3.1.p pacid=32973541 transcript=Pp3c12_22500V3.1
locus=Pp3c12_22500 annot-version=v3.3 Physcomitrella_patens
OG0000050
Pp3c25_6940V3.1.p pacid=32979621 transcript=Pp3c25_6940V3.1
locus=Pp3c25_6940 annot-version=v3.3 Physcomitrella_patens
OG0000050
Pp3c17_13070V3.1.p pacid=32907963 transcript=Pp3c17_13070V3.1
locus=Pp3c17_13070 annot-version=v3.3 Physcomitrella_patens
OG0000050
Pp3c17_5920V3.1.p pacid=32908033 transcript=Pp3c17_5920V3.1
locus=Pp3c17_5920 annot-version=v3.3 Physcomitrella_patens
OG0000050
Pp3c16_19170V3.1.p pacid=32984545 transcript=Pp3c16_19170V3.1
locus=Pp3c16_19170 annot-version=v3.3 Physcomitrella_patens
OG0000050
Pp3c9_1370V3.1.p pacid=32912179 transcript=Pp3c9_1370V3.1
locus=Pp3c9_1370 annot-version=v3.3 Physcomitrella_patens
OG0000050
Pp3c4_540V3.1.p pacid=32919831 transcript=Pp3c4_540V3.1
locus=Pp3c4_540 annot-version=v3.3 Physcomitrella_patens
OG0000050
Pp3c4_3820V3.1.p pacid=32920459 transcript=Pp3c4_3820V3.1
locus=Pp3c4_3820 annot-version=v3.3 Physcomitrella_patens
OG0000050
AT2G34440.1 | AGAMOUS-like 29 | Chr2:14526950-14527468 FORWARD
LENGTH=172 | 201606 Arabidopsis_thaliana OG0000050
AT2G42830.1 | K-box region and MADS-box transcription factor family
protein | Chr2:17820602-17823806 FORWARD LENGTH=246 | 201606
Arabidopsis_thaliana OG0000050
AT2G45650.1 | AGAMOUS-like 6 | Chr2:18804453-18806291 FORWARD
LENGTH=252 | 201606 Arabidopsis_thaliana OG0000050
AT2G45660.1 | AGAMOUS-like 20 | Chr2:18807799-18810193 REVERSE
LENGTH=214 | 201606 Arabidopsis_thaliana OG0000050
AT3G02310.1 | K-box region and MADS-box transcription factor family

protein | Chr3:464554-466687 REVERSE LENGTH=250 | 201606
Arabidopsis_thaliana OG0000050
AT3G04100.1 | AGAMOUS-like 57 | Chr3:1075299-1075922 FORWARD
LENGTH=207 | 201606 Arabidopsis_thaliana OG0000050
AT3G30260.1 | AGAMOUS-like 79 | Chr3:11909119-11912880 FORWARD
LENGTH=249 | 201606 Arabidopsis_thaliana OG0000050
AT3G54340.1 | K-box region and MADS-box transcription factor family
protein | Chr3:20119428-20121087 REVERSE LENGTH=232 | 201606
Arabidopsis_thaliana OG0000050
AT3G57230.1 | AGAMOUS-like 16 | Chr3:21177710-21180671 FORWARD
LENGTH=240 | 201606 Arabidopsis_thaliana OG0000050
AT3G57390.1 | AGAMOUS-like 18 | Chr3:21233910-21235735 FORWARD
LENGTH=256 | 201606 Arabidopsis_thaliana OG0000050
AT3G58780.1 | K-box region and MADS-box transcription factor family
protein | Chr3:21739150-21741766 FORWARD LENGTH=248 | 201606
Arabidopsis_thaliana OG0000050
AT3G61120.1 | AGAMOUS-like 13 | Chr3:22618414-22620466 REVERSE
LENGTH=244 | 201606 Arabidopsis_thaliana OG0000050
AT3G66656.1 | AGAMOUS-like 91 | Chr3:2091262-2091798 REVERSE
LENGTH=178 | 201606 Arabidopsis_thaliana OG0000050
AT1G17310.1 | MADS-box transcription factor family protein |
Chr1:5928014-5928667 REVERSE LENGTH=217 | 201606
Arabidopsis_thaliana OG0000050
AT4G09960.1 | K-box region and MADS-box transcription factor family
protein | Chr4:6236713-6239409 REVERSE LENGTH=230 | 201606
Arabidopsis_thaliana OG0000050
AT4G11880.1 | AGAMOUS-like 14 | Chr4:7143512-7147108 FORWARD
LENGTH=221 | 201606 Arabidopsis_thaliana OG0000050
AT4G18960.1 | K-box region and MADS-box transcription factor family
protein | Chr4:10383917-10388272 FORWARD LENGTH=252 | 201606
Arabidopsis_thaliana OG0000050
AT1G18750.1 | AGAMOUS-like 65 | Chr1:6467266-6469640 FORWARD
LENGTH=389 | 201606 Arabidopsis_thaliana OG0000050
AT4G22950.1 | AGAMOUS-like 19 | Chr4:12023946-12027421 REVERSE
LENGTH=219 | 201606 Arabidopsis_thaliana OG0000050
AT4G24540.1 | AGAMOUS-like 24 | Chr4:12671160-12673645 REVERSE
LENGTH=220 | 201606 Arabidopsis_thaliana OG0000050
AT4G36590.1 | MADS-box transcription factor family protein |
Chr4:17261146-17262189 REVERSE LENGTH=248 | 201606
Arabidopsis_thaliana OG0000050
AT4G37435.1 | MADS-box transcription factor-like protein |
Chr4:17599115-17600626 REVERSE LENGTH=186 | 201606
Arabidopsis_thaliana OG0000050
AT4G37940.1 | AGAMOUS-like 21 | Chr4:17835695-17838621 REVERSE
LENGTH=228 | 201606 Arabidopsis_thaliana OG0000050
AT5G06500.1 | AGAMOUS-like 96 | Chr5:1982444-1983172 FORWARD
LENGTH=242 | 201606 Arabidopsis_thaliana OG0000050
AT5G10140.1 | K-box region and MADS-box transcription factor family
protein | Chr5:3173724-3179339 REVERSE LENGTH=196 | 201606
Arabidopsis_thaliana OG0000050
AT5G13790.1 | AGAMOUS-like 15 | Chr5:4449128-4450802 REVERSE
LENGTH=268 | 201606 Arabidopsis_thaliana OG0000050
AT1G22130.1 | AGAMOUS-like 104 | Chr1:7812387-7814259 REVERSE
LENGTH=335 | 201606 Arabidopsis_thaliana OG0000050

AT5G15800.1 | K-box region and MADS-box transcription factor family protein | Chr5:5151594-5153767 REVERSE LENGTH=251 | 201606 Arabidopsis_thaliana OG0000050
AT5G20240.1 | K-box region and MADS-box transcription factor family protein | Chr5:6829203-6831208 FORWARD LENGTH=208 | 201606 Arabidopsis_thaliana OG0000050
AT5G23260.1 | K-box region and MADS-box transcription factor family protein | Chr5:7836294-7838340 FORWARD LENGTH=263 | 201606 Arabidopsis_thaliana OG0000050
AT1G24260.1 | K-box region and MADS-box transcription factor family protein | Chr1:8593790-8595862 REVERSE LENGTH=250 | 201606 Arabidopsis_thaliana OG0000050
AT5G51860.1 | K-box region and MADS-box transcription factor family protein | Chr5:21081844-21084126 REVERSE LENGTH=211 | 201606 Arabidopsis_thaliana OG0000050
AT5G51870.1 | AGAMOUS-like 71 | Chr5:21085635-21087923 REVERSE LENGTH=207 | 201606 Arabidopsis_thaliana OG0000050
AT1G26310.1 | K-box region and MADS-box transcription factor family protein | Chr1:9100330-9103510 REVERSE LENGTH=255 | 201606 Arabidopsis_thaliana OG0000050
AT5G55690.1 | MADS-box transcription factor family protein | Chr5:22548790-22549623 REVERSE LENGTH=277 | 201606 Arabidopsis_thaliana OG0000050
AT5G60440.1 | AGAMOUS-like 62 | Chr5:24306329-24307520 FORWARD LENGTH=299 | 201606 Arabidopsis_thaliana OG0000050
AT5G60910.1 | AGAMOUS-like 8 | Chr5:24502736-24506013 REVERSE LENGTH=242 | 201606 Arabidopsis_thaliana OG0000050
AT5G62165.1 | AGAMOUS-like 42 | Chr5:24965075-24968437 FORWARD LENGTH=210 | 201606 Arabidopsis_thaliana OG0000050
AT5G65050.1 | AGAMOUS-like 31 | Chr5:25982415-25986114 FORWARD LENGTH=182 | 201606 Arabidopsis_thaliana OG0000050
AT5G65060.1 | K-box region and MADS-box transcription factor family protein | Chr5:25987527-25991065 FORWARD LENGTH=196 | 201606 Arabidopsis_thaliana OG0000050
AT5G65070.1 | K-box region and MADS-box transcription factor family protein | Chr5:25992310-25995930 FORWARD LENGTH=200 | 201606 Arabidopsis_thaliana OG0000050
AT1G28450.1 | AGAMOUS-like 58 | Chr1:10003966-10004523 FORWARD LENGTH=185 | 201606 Arabidopsis_thaliana OG0000050
AT1G28460.1 | AGAMOUS-like 59 | Chr1:10006230-10006778 FORWARD LENGTH=182 | 201606 Arabidopsis_thaliana OG0000050
AT1G29962.1 | AGAMOUS-like 64 | Chr1:10496730-10497287 FORWARD LENGTH=185 | 201606 Arabidopsis_thaliana OG0000050
AT1G31140.1 | GORDITA | Chr1:11118031-11119673 FORWARD LENGTH=213 | 201606 Arabidopsis_thaliana OG0000050
AT1G46408.1 | AGAMOUS-like 97 | Chr1:17232135-17232935 REVERSE LENGTH=266 | 201606 Arabidopsis_thaliana OG0000050
AT1G47760.1 | AGAMOUS-like 102 | Chr1:17572451-17573159 FORWARD LENGTH=184 | 201606 Arabidopsis_thaliana OG0000050
AT1G60920.1 | AGAMOUS-like 55 | Chr1:22429692-22430267 REVERSE LENGTH=191 | 201606 Arabidopsis_thaliana OG0000050
AT1G01530.1 | AGAMOUS-like 28 | Chr1:192640-193662 REVERSE LENGTH=247 | 201606 Arabidopsis_thaliana OG0000050
AT1G65360.1 | AGAMOUS-like 23 | Chr1:24281337-24282151 FORWARD

LENGTH=226 | 201606 Arabidopsis_thaliana OG0000050
AT1G69120.1 | K-box region and MADS-box transcription factor family protein | Chr1:25982576-25986102 REVERSE LENGTH=256 | 201606 Arabidopsis_thaliana OG0000050
AT1G69540.1 | AGAMOUS-like 94 | Chr1:26145306-26147159 REVERSE LENGTH=344 | 201606 Arabidopsis_thaliana OG0000050
AT1G71692.1 | AGAMOUS-like 12 | Chr1:26952903-26954939 REVERSE LENGTH=211 | 201606 Arabidopsis_thaliana OG0000050
AT1G72350.1 | MADS-box transcription factor family protein | Chr1:27239273-27239947 REVERSE LENGTH=224 | 201606 Arabidopsis_thaliana OG0000050
AT1G77080.2 | K-box region and MADS-box transcription factor family protein | Chr1:28955679-28959845 FORWARD LENGTH=192 | 201606 Arabidopsis_thaliana OG0000050
AT1G77950.1 | AGAMOUS-like 67 | Chr1:29307029-29309667 FORWARD LENGTH=252 | 201606 Arabidopsis_thaliana OG0000050
AT1G77980.1 | AGAMOUS-like 66 | Chr1:29315212-29317067 REVERSE LENGTH=332 | 201606 Arabidopsis_thaliana OG0000050
AT2G03060.1 | AGAMOUS-like 30 | Chr2:901614-903639 FORWARD LENGTH=381 | 201606 Arabidopsis_thaliana OG0000050
AT2G03710.1 | K-box region and MADS-box transcription factor family protein | Chr2:1129622-1131628 FORWARD LENGTH=258 | 201606 Arabidopsis_thaliana OG0000050
AT2G14210.1 | AGAMOUS-like 44 | Chr2:6018841-6023585 FORWARD LENGTH=234 | 201606 Arabidopsis_thaliana OG0000050
AT2G22540.1 | K-box region and MADS-box transcription factor family protein | Chr2:9580417-9583603 FORWARD LENGTH=240 | 201606 Arabidopsis_thaliana OG0000050
AT2G22630.1 | AGAMOUS-like 17 | Chr2:9618372-9621641 FORWARD LENGTH=227 | 201606 Arabidopsis_thaliana OG0000050
AT2G24840.1 | AGAMOUS-like 61 | Chr2:10581082-10581876 FORWARD LENGTH=264 | 201606 Arabidopsis_thaliana OG0000050
AT2G26320.1 | AGAMOUS-like 33 | Chr2:11205389-11206287 REVERSE LENGTH=109 | 201606 Arabidopsis_thaliana OG0000050
Os03t0752800-01 Similar to Isoform 2 of MADS-box transcription factor 14. Oryza_sativa OG0000050
Os03t0753100-01 MADS-box transcription factor, Inflorescence and spikelet development Oryza_sativa OG0000050
Os03t0834466-00 Similar to MADS-box transcription factor 21. Oryza_sativa OG0000050
Os04t0304400-01 Similar to MADS-box protein AGL16-II. Oryza_sativa OG0000050
Os04t0387300-01 Similar to OSIGBa0075F02.1 protein. Oryza_sativa OG0000050
Os04t0461300-01 Similar to MIKC-type MADS-box transcription factor WM30. Oryza_sativa OG0000050
Os04t0580700-01 MADS-box transcription factor, Regulation of floral organ identity and meristem fate Oryza_sativa OG0000050
Os04t0614100-00 Transcription factor, MADS-box domain containing protein. Oryza_sativa OG0000050
Os05t0203800-01 Similar to MADS box protein. Oryza_sativa OG0000050
Os05t0423400-01 Similar to PISTILLATA-like MADS box protein. Oryza_sativa OG0000050

Os06t0108500-01 Similar to MIKC-type MADS-box transcription factor AGL1.	Oryza_sativa	OG0000050
Os06t0162800-01 MADS box protein.	Oryza_sativa	OG0000050
Os06t0217300-01 Similar to Transcription factor MADS55.	Oryza_sativa	OG0000050
Os06t0223300-01 MADS box transcription factor, Pollen maturation	Oryza_sativa	OG0000050
Os06t0347700-00 Transcription factor, MADS-box domain containing protein.	Oryza_sativa	OG0000050
Os06t0504100-00 Similar to SRF-type transcription factor family protein, expressed.	Oryza_sativa	OG0000050
Os06t0667200-01 MADS-box transcription factor, Vegetative development	Oryza_sativa	OG0000050
Os06t0712700-01 Transcription factor, Development of lodicules and stamens	Oryza_sativa	OG0000050
Os07t0108900-01 Similar to MADS-box transcription factor 15.	Oryza_sativa	OG0000050
Os07t0605200-01 APETALA1 (AP1)/ FRUITFULL (FUL)-like MADS box transcription factor, Specification of inflorescence meristem identity	Oryza_sativa	OG0000050
Os08t0112700-01 MADS-box transcription factor, Regulator of both biotic and abiotic stress responses	Oryza_sativa	OG0000050
Os08t0431900-00 Transcription factor MADS23.	Oryza_sativa	OG0000050
Os08t0494100-01 MADS box transcription factor, Pollen maturation	Oryza_sativa	OG0000050
Os08t0531700-01 Similar to MADS-box transcription factor 7.	Oryza_sativa	OG0000050
Os08t0531900-01 Similar to MADS box transcription factor-like protein (MADS-box protein AGL72).	Oryza_sativa	OG0000050
Os09t0116000-00 Transcription factor, MADS-box domain containing protein.	Oryza_sativa	OG0000050
Os09t0116800-00 Similar to DNA binding protein.	Oryza_sativa	OG0000050
Os09t0402533-00 Similar to DNA binding protein.	Oryza_sativa	OG0000050
Os09t0507200-01 MADS box protein.	Oryza_sativa	OG0000050
Os10t0536100-01 Similar to MADS-box transcription factor 56.	Oryza_sativa	OG0000050
Os01t0726400-01 MADS box transcription factor, Regulation of floral organ identity	Oryza_sativa	OG0000050
Os11t0658700-01 MADS box transcription factor, Pollen maturation	Oryza_sativa	OG0000050
Os12t0206800-00 Transcription factor, MADS-box domain containing protein.	Oryza_sativa	OG0000050
Os12t0207000-01 Transcription factor.	Oryza_sativa	OG0000050
Os12t0407000-00 Similar to SRF-type transcription factor family protein, expressed.	Oryza_sativa	OG0000050
Os12t0501700-00 Transcription factor, MADS-box domain containing protein.	Oryza_sativa	OG0000050
Os01t0883100-01 Similar to PISTILLATA-like MADS box protein.	Oryza_sativa	OG0000050

Os01t0886200-01 Similar to AGAMOUS homolog.	Oryza_sativa	OG0000050
Os01t0903800-00 Similar to MADS-box transcription factor 21.	Oryza_sativa	OG0000050
Os01t0922800-01 MADS-box transcription factor, Short-day flowering promoter	Oryza_sativa	OG0000050
Os01t0975800-00 Similar to DNA binding protein.	Oryza_sativa	OG0000050
Os02t0104100-00 Similar to MADS-box transcription factor TaAGL18.	Oryza_sativa	OG0000050
Os02t0170300-01 MADS box transcription factor, Regulator of early seed development	Oryza_sativa	OG0000050
Os02t0579600-00 Transcription factor MADS27.	Oryza_sativa	OG0000050
Os02t0682200-01 MADS-box transcription factor, Regulation of floral organ identity and meristem fate, Endosperm development	Oryza_sativa	OG0000050
Os01t0201700-01 Similar to M23 protein (Fragment).	Oryza_sativa	OG0000050
Os02t0731200-01 Transcription factor MADS57.	Oryza_sativa	OG0000050
Os02t0761000-01 Similar to Transcription factor MADS55.	Oryza_sativa	OG0000050
Os03t0122600-01 MIKC-type MADS-box protein, Flowering time, Short-day/long-day promotion	Oryza_sativa	OG0000050
Os03t0186600-01 Transcription factor, MADS-box domain containing protein.	Oryza_sativa	OG0000050
Os03t0215400-01 MADS-domain-containing protein, Sexual reproduction	Oryza_sativa	OG0000050
CMA095CT pep chromosome:ASM9120v1:1:296064:297602:1 gene:CMA095C transcript:CMA095CT gene_biotype:protein_coding transcript_biotype:protein_coding	Cyanidioschyzon_merolae	OG0000050
evm.model.contig_2031.18	Porphyridium_purpureum	OG0000050
evm.model.contig_454.6	Porphyridium_purpureum	OG0000050
kfl00053_0010_v1.1	Klebsormidium_flaccidum	OG0000051
Pp3c10_1100V3.1.p pacid=32899623 transcript=Pp3c10_1100V3.1 locus=Pp3c10_1100 annot-version=v3.3	Physcomitrella_patens	OG0000051
Pp3c15_8250V3.1.p pacid=32928225 transcript=Pp3c15_8250V3.1 locus=Pp3c15_8250 annot-version=v3.3	Physcomitrella_patens	OG0000051
Pp3c15_12280V3.1.p pacid=32928523 transcript=Pp3c15_12280V3.1 locus=Pp3c15_12280 annot-version=v3.3	Physcomitrella_patens	OG0000051
Pp3c10_19460V3.1.p pacid=32902627 transcript=Pp3c10_19460V3.1 locus=Pp3c10_19460 annot-version=v3.3	Physcomitrella_patens	OG0000051
Pp3c2_35260V3.1.p pacid=32934718 transcript=Pp3c2_35260V3.1 locus=Pp3c2_35260 annot-version=v3.3	Physcomitrella_patens	OG0000051
Pp3c10_20120V3.1.p pacid=32899716 transcript=Pp3c10_20120V3.1 locus=Pp3c10_20120 annot-version=v3.3	Physcomitrella_patens	OG0000051

Pp3c19_8330V3.1.p pacid=32938019 transcript=Pp3c19_8330V3.1
locus=Pp3c19_8330 annot-version=v3.3 Physcomitrella_patens
OG0000051
Pp3c3_35240V3.1.p pacid=32940913 transcript=Pp3c3_35240V3.1
locus=Pp3c3_35240 annot-version=v3.3 Physcomitrella_patens
OG0000051
Pp3c3_35217V3.1.p pacid=32941043 transcript=Pp3c3_35217V3.1
locus=Pp3c3_35217 annot-version=v3.3 Physcomitrella_patens
OG0000051
Pp3c3_30560V3.1.p pacid=32942021 transcript=Pp3c3_30560V3.1
locus=Pp3c3_30560 annot-version=v3.3 Physcomitrella_patens
OG0000051
Pp3c3_32350V3.1.p pacid=32943556 transcript=Pp3c3_32350V3.1
locus=Pp3c3_32350 annot-version=v3.3 Physcomitrella_patens
OG0000051
Pp3c3_24830V3.1.p pacid=32943963 transcript=Pp3c3_24830V3.1
locus=Pp3c3_24830 annot-version=v3.3 Physcomitrella_patens
OG0000051
Pp3c3_36450V3.1.p pacid=32943975 transcript=Pp3c3_36450V3.1
locus=Pp3c3_36450 annot-version=v3.3 Physcomitrella_patens
OG0000051
Pp3c3_35040V3.1.p pacid=32945112 transcript=Pp3c3_35040V3.1
locus=Pp3c3_35040 annot-version=v3.3 Physcomitrella_patens
OG0000051
Pp3c5_12660V3.1.p pacid=32952735 transcript=Pp3c5_12660V3.1
locus=Pp3c5_12660 annot-version=v3.3 Physcomitrella_patens
OG0000051
Pp3c5_23400V3.1.p pacid=32954138 transcript=Pp3c5_23400V3.1
locus=Pp3c5_23400 annot-version=v3.3 Physcomitrella_patens
OG0000051
Pp3c5_12770V3.1.p pacid=32954146 transcript=Pp3c5_12770V3.1
locus=Pp3c5_12770 annot-version=v3.3 Physcomitrella_patens
OG0000051
Pp3c5_12820V3.1.p pacid=32954817 transcript=Pp3c5_12820V3.1
locus=Pp3c5_12820 annot-version=v3.3 Physcomitrella_patens
OG0000051
Pp3c5_12750V3.1.p pacid=32954869 transcript=Pp3c5_12750V3.1
locus=Pp3c5_12750 annot-version=v3.3 Physcomitrella_patens
OG0000051
Pp3c8_3840V3.1.p pacid=32963585 transcript=Pp3c8_3840V3.1
locus=Pp3c8_3840 annot-version=v3.3 Physcomitrella_patens
OG0000051
Pp3c8_1680V3.1.p pacid=32964107 transcript=Pp3c8_1680V3.1
locus=Pp3c8_1680 annot-version=v3.3 Physcomitrella_patens
OG0000051
Pp3c8_2010V3.1.p pacid=32964241 transcript=Pp3c8_2010V3.1
locus=Pp3c8_2010 annot-version=v3.3 Physcomitrella_patens
OG0000051
Pp3c8_1440V3.1.p pacid=32965451 transcript=Pp3c8_1440V3.1
locus=Pp3c8_1440 annot-version=v3.3 Physcomitrella_patens
OG0000051
Pp3c8_460V3.1.p pacid=32966015 transcript=Pp3c8_460V3.1
locus=Pp3c8_460 annot-version=v3.3 Physcomitrella_patens
OG0000051

Pp3c1_22810V3.1.p pacid=32968496 transcript=Pp3c1_22810V3.1 locus=Pp3c1_22810 annot-version=v3.3 Physcomitrella_patens OG0000051
Pp3c1_6010V3.1.p pacid=32969050 transcript=Pp3c1_6010V3.1 locus=Pp3c1_6010 annot-version=v3.3 Physcomitrella_patens OG0000051
Pp3c12_24000V3.1.p pacid=32972101 transcript=Pp3c12_24000V3.1 locus=Pp3c12_24000 annot-version=v3.3 Physcomitrella_patens OG0000051
Pp3c12_23790V3.1.p pacid=32974407 transcript=Pp3c12_23790V3.1 locus=Pp3c12_23790 annot-version=v3.3 Physcomitrella_patens OG0000051
Pp3c6_21010V3.1.p pacid=32976454 transcript=Pp3c6_21010V3.1 locus=Pp3c6_21010 annot-version=v3.3 Physcomitrella_patens OG0000051
Pp3c25_760V3.1.p pacid=32979305 transcript=Pp3c25_760V3.1 locus=Pp3c25_760 annot-version=v3.3 Physcomitrella_patens OG0000051
Pp3c25_15080V3.1.p pacid=32979366 transcript=Pp3c25_15080V3.1 locus=Pp3c25_15080 annot-version=v3.3 Physcomitrella_patens OG0000051
Pp3c25_3840V3.1.p pacid=32979548 transcript=Pp3c25_3840V3.1 locus=Pp3c25_3840 annot-version=v3.3 Physcomitrella_patens OG0000051
Pp3c18_170V3.1.p pacid=32981388 transcript=Pp3c18_170V3.1 locus=Pp3c18_170 annot-version=v3.3 Physcomitrella_patens OG0000051
Pp3c18_13190V3.1.p pacid=32981571 transcript=Pp3c18_13190V3.1 locus=Pp3c18_13190 annot-version=v3.3 Physcomitrella_patens OG0000051
Pp3c18_22120V3.1.p pacid=32981758 transcript=Pp3c18_22120V3.1 locus=Pp3c18_22120 annot-version=v3.3 Physcomitrella_patens OG0000051
Pp3c16_19430V3.1.p pacid=32983931 transcript=Pp3c16_19430V3.1 locus=Pp3c16_19430 annot-version=v3.3 Physcomitrella_patens OG0000051
Pp3c16_6660V3.1.p pacid=32984740 transcript=Pp3c16_6660V3.1 locus=Pp3c16_6660 annot-version=v3.3 Physcomitrella_patens OG0000051
Pp3c17_6440V3.1.p pacid=32908174 transcript=Pp3c17_6440V3.1 locus=Pp3c17_6440 annot-version=v3.3 Physcomitrella_patens OG0000051
Pp3c10_18650V3.1.p pacid=32900787 transcript=Pp3c10_18650V3.1 locus=Pp3c10_18650 annot-version=v3.3 Physcomitrella_patens OG0000051
Pp3c21_22110V3.1.p pacid=32914866 transcript=Pp3c21_22110V3.1 locus=Pp3c21_22110 annot-version=v3.3 Physcomitrella_patens OG0000051
Pp3c21_11740V3.1.p pacid=32916856 transcript=Pp3c21_11740V3.1 locus=Pp3c21_11740 annot-version=v3.3 Physcomitrella_patens OG0000051
Pp3c26_2170V3.1.p pacid=32918191 transcript=Pp3c26_2170V3.1 locus=Pp3c26_2170 annot-version=v3.3 Physcomitrella_patens OG0000051

Pp3c10_25030V3.1.p pacid=32901308 transcript=Pp3c10_25030V3.1 locus=Pp3c10_25030 annot-version=v3.3 Physcomitrella_patens OG0000051
Pp3c4_22420V3.1.p pacid=32921440 transcript=Pp3c4_22420V3.1 locus=Pp3c4_22420 annot-version=v3.3 Physcomitrella_patens OG0000051
Pp3c4_10640V3.1.p pacid=32921762 transcript=Pp3c4_10640V3.1 locus=Pp3c4_10640 annot-version=v3.3 Physcomitrella_patens OG0000051
AT2G36700.1 | Pectin lyase-like superfamily protein | Chr2:15384706-15386421 REVERSE LENGTH=333 | 201606 Arabidopsis_thaliana OG0000051
AT2G36710.1 | Pectin lyase-like superfamily protein | Chr2:15389614-15391200 REVERSE LENGTH=407 | 201606 Arabidopsis_thaliana OG0000051
AT2G43050.1 | Plant invertase/pectin methylesterase inhibitor superfamily | Chr2:17902508-17904174 FORWARD LENGTH=518 | 201606 Arabidopsis_thaliana OG0000051
AT2G45220.1 | Plant invertase/pectin methylesterase inhibitor superfamily | Chr2:18644281-18646394 REVERSE LENGTH=511 | 201606 Arabidopsis_thaliana OG0000051
AT1G11370.1 | Pectin lyase-like superfamily protein | Chr1:3828098-3830945 REVERSE LENGTH=288 | 201606 Arabidopsis_thaliana OG0000051
AT2G47030.1 | Plant invertase/pectin methylesterase inhibitor superfamily | Chr2:19324415-19326268 REVERSE LENGTH=588 | 201606 Arabidopsis_thaliana OG0000051
AT2G47040.1 | Plant invertase/pectin methylesterase inhibitor superfamily | Chr2:19328186-19330060 REVERSE LENGTH=595 | 201606 Arabidopsis_thaliana OG0000051
AT2G47280.1 | Pectin lyase-like superfamily protein | Chr2:19416800-19418252 FORWARD LENGTH=336 | 201606 Arabidopsis_thaliana OG0000051
AT1G11580.1 | methylesterase PCR A | Chr1:3888730-3890649 FORWARD LENGTH=557 | 201606 Arabidopsis_thaliana OG0000051
AT2G47550.1 | Plant invertase/pectin methylesterase inhibitor superfamily | Chr2:19509024-19511604 FORWARD LENGTH=560 | 201606 Arabidopsis_thaliana OG0000051
AT1G11590.1 | Plant invertase/pectin methylesterase inhibitor superfamily | Chr1:3892634-3894620 FORWARD LENGTH=524 | 201606 Arabidopsis_thaliana OG0000051
AT3G05610.1 | Plant invertase/pectin methylesterase inhibitor superfamily | Chr3:1625876-1627976 REVERSE LENGTH=669 | 201606 Arabidopsis_thaliana OG0000051
AT3G05620.1 | Plant invertase/pectin methylesterase inhibitor superfamily | Chr3:1629658-1631766 REVERSE LENGTH=543 | 201606 Arabidopsis_thaliana OG0000051
AT3G06830.1 | Plant invertase/pectin methylesterase inhibitor superfamily | Chr3:2154193-2156062 FORWARD LENGTH=568 | 201606 Arabidopsis_thaliana OG0000051
AT3G10710.1 | root hair specific 12 | Chr3:3352289-3354237 FORWARD LENGTH=561 | 201606 Arabidopsis_thaliana OG0000051
AT3G10720.1 | Plant invertase/pectin methylesterase inhibitor superfamily | Chr3:3354639-3355750 REVERSE LENGTH=263 | 201606

Arabidopsis_thaliana   OG0000051
AT3G14300.1 | pectinesterase family protein | Chr3:4766905-4769898 REVERSE LENGTH=968 | 201606   Arabidopsis_thaliana   OG0000051
AT3G14310.1 | pectin methylesterase 3 | Chr3:4772214-4775095 REVERSE LENGTH=592 | 201606   Arabidopsis_thaliana   OG0000051
AT3G17060.1 | Pectin lyase-like superfamily protein | Chr3:5816953-5818458 REVERSE LENGTH=344 | 201606   Arabidopsis_thaliana   OG0000051
AT3G24130.1 | Pectin lyase-like superfamily protein | Chr3:8711663-8713361 REVERSE LENGTH=335 | 201606   Arabidopsis_thaliana   OG0000051
AT3G27980.1 | Plant invertase/pectin methylesterase inhibitor superfamily | Chr3:10393904-10395861 FORWARD LENGTH=497 | 201606   Arabidopsis_thaliana   OG0000051
AT3G29090.1 | pectin methylesterase 31 | Chr3:11073804-11075335 FORWARD LENGTH=317 | 201606   Arabidopsis_thaliana   OG0000051
AT3G43270.1 | Plant invertase/pectin methylesterase inhibitor superfamily | Chr3:15222402-15225124 REVERSE LENGTH=527 | 201606   Arabidopsis_thaliana   OG0000051
AT3G47400.1 | Plant invertase/pectin methylesterase inhibitor superfamily | Chr3:17465629-17467888 FORWARD LENGTH=594 | 201606   Arabidopsis_thaliana   OG0000051
AT3G49220.1 | Plant invertase/pectin methylesterase inhibitor superfamily | Chr3:18249840-18253647 FORWARD LENGTH=598 | 201606   Arabidopsis_thaliana   OG0000051
AT3G59010.1 | pectin methylesterase 61 | Chr3:21803015-21805098 REVERSE LENGTH=529 | 201606   Arabidopsis_thaliana   OG0000051
AT3G60730.1 | Plant invertase/pectin methylesterase inhibitor superfamily | Chr3:22444955-22447226 FORWARD LENGTH=519 | 201606   Arabidopsis_thaliana   OG0000051
AT3G62170.1 | VANGUARD-like protein | Chr3:23016495-23018337 REVERSE LENGTH=588 | 201606   Arabidopsis_thaliana   OG0000051
AT4G00190.1 | pectin methylesterase 38 | Chr4:80433-82040 REVERSE LENGTH=474 | 201606   Arabidopsis_thaliana   OG0000051
AT4G02300.1 | Plant invertase/pectin methylesterase inhibitor superfamily | Chr4:1009366-1013034 REVERSE LENGTH=532 | 201606   Arabidopsis_thaliana   OG0000051
AT4G02320.1 | Plant invertase/pectin methylesterase inhibitor superfamily | Chr4:1022725-1026118 REVERSE LENGTH=518 | 201606   Arabidopsis_thaliana   OG0000051
AT4G02330.1 | Plant invertase/pectin methylesterase inhibitor superfamily | Chr4:1032479-1034928 FORWARD LENGTH=573 | 201606   Arabidopsis_thaliana   OG0000051
AT4G03930.1 | Plant invertase/pectin methylesterase inhibitor superfamily | Chr4:1870422-1872387 FORWARD LENGTH=524 | 201606   Arabidopsis_thaliana   OG0000051
AT4G15980.1 | Plant invertase/pectin methylesterase inhibitor superfamily | Chr4:9057466-9059983 REVERSE LENGTH=701 | 201606   Arabidopsis_thaliana   OG0000051
AT1G02810.1 | Plant invertase/pectin methylesterase inhibitor superfamily | Chr1:618284-620333 FORWARD LENGTH=579 | 201606

Arabidopsis_thaliana   OG0000051
AT4G33220.1 | pectin methylesterase 44 | Chr4:16022506-16026130
FORWARD LENGTH=525 | 201606   Arabidopsis_thaliana
OG0000051
AT4G33230.1 | Plant invertase/pectin methylesterase inhibitor
superfamily | Chr4:16026591-16028754 REVERSE LENGTH=609 | 201606
Arabidopsis_thaliana   OG0000051
AT5G04960.1 | Plant invertase/pectin methylesterase inhibitor
superfamily | Chr5:1461985-1463809 FORWARD LENGTH=564 | 201606
Arabidopsis_thaliana   OG0000051
AT5G04970.1 | Plant invertase/pectin methylesterase inhibitor
superfamily | Chr5:1464262-1467001 REVERSE LENGTH=624 | 201606
Arabidopsis_thaliana   OG0000051
AT5G07410.1 | Pectin lyase-like superfamily protein |
Chr5:2345852-2347276 FORWARD LENGTH=361 | 201606
Arabidopsis_thaliana   OG0000051
AT5G07420.1 | Pectin lyase-like superfamily protein |
Chr5:2349547-2351366 FORWARD LENGTH=361 | 201606
Arabidopsis_thaliana   OG0000051
AT5G07430.1 | Pectin lyase-like superfamily protein |
Chr5:2352549-2354069 FORWARD LENGTH=361 | 201606
Arabidopsis_thaliana   OG0000051
AT5G09760.1 | Plant invertase/pectin methylesterase inhibitor
superfamily | Chr5:3032446-3034364 FORWARD LENGTH=551 | 201606
Arabidopsis_thaliana   OG0000051
AT5G18990.1 | Pectin lyase-like superfamily protein |
Chr5:6340076-6341614 FORWARD LENGTH=330 | 201606
Arabidopsis_thaliana   OG0000051
AT5G19730.1 | Pectin lyase-like superfamily protein |
Chr5:6670562-6673202 FORWARD LENGTH=383 | 201606
Arabidopsis_thaliana   OG0000051
AT5G20860.1 | Plant invertase/pectin methylesterase inhibitor
superfamily | Chr5:7076890-7079077 REVERSE LENGTH=512 | 201606
Arabidopsis_thaliana   OG0000051
AT1G23200.1 | Plant invertase/pectin methylesterase inhibitor
superfamily | Chr1:8227236-8229400 FORWARD LENGTH=554 | 201606
Arabidopsis_thaliana   OG0000051
AT5G26810.1 | Pectin lyase-like superfamily protein |
Chr5:9430952-9432969 FORWARD LENGTH=339 | 201606
Arabidopsis_thaliana   OG0000051
AT5G27870.1 | Plant invertase/pectin methylesterase inhibitor
superfamily | Chr5:9878991-9881806 REVERSE LENGTH=732 | 201606
Arabidopsis_thaliana   OG0000051
AT5G47500.1 | Pectin lyase-like superfamily protein |
Chr5:19271262-19272845 REVERSE LENGTH=362 | 201606
Arabidopsis_thaliana   OG0000051
AT5G49180.1 | Plant invertase/pectin methylesterase inhibitor
superfamily | Chr5:19940783-19942876 REVERSE LENGTH=571 | 201606
Arabidopsis_thaliana   OG0000051
AT5G51490.1 | Plant invertase/pectin methylesterase inhibitor
superfamily | Chr5:20913680-20915606 REVERSE LENGTH=536 | 201606
Arabidopsis_thaliana   OG0000051
AT5G51500.1 | Plant invertase/pectin methylesterase inhibitor
superfamily | Chr5:20917929-20919838 REVERSE LENGTH=540 | 201606

Arabidopsis_thaliana   OG0000051
AT5G53370.1 | pectin methylesterase PCR fragment F |
Chr5:21649683-21651530 REVERSE LENGTH=587 | 201606
Arabidopsis_thaliana   OG0000051
AT5G55590.1 | Pectin lyase-like superfamily protein |
Chr5:22519911-22521699 FORWARD LENGTH=380 | 201606
Arabidopsis_thaliana   OG0000051
AT5G61680.1 | Pectin lyase-like superfamily protein |
Chr5:24786416-24788015 REVERSE LENGTH=364 | 201606
Arabidopsis_thaliana   OG0000051
AT5G64640.1 | Plant invertase/pectin methylesterase inhibitor
superfamily | Chr5:25836820-25839053 FORWARD LENGTH=602 | 201606
Arabidopsis_thaliana   OG0000051
AT1G53830.1 | pectin methylesterase 2 | Chr1:20098562-20100745
FORWARD LENGTH=587 | 201606   Arabidopsis_thaliana
OG0000051
AT1G53840.1 | pectin methylesterase 1 | Chr1:20101533-20103458
FORWARD LENGTH=586 | 201606   Arabidopsis_thaliana
OG0000051
AT1G05310.1 | Pectin lyase-like superfamily protein |
Chr1:1550615-1552434 REVERSE LENGTH=393 | 201606
Arabidopsis_thaliana   OG0000051
AT1G69940.1 | Pectin lyase-like superfamily protein |
Chr1:26343549-26344971 REVERSE LENGTH=361 | 201606
Arabidopsis_thaliana   OG0000051
AT2G19150.1 | Pectin lyase-like superfamily protein |
Chr2:8305100-8307444 FORWARD LENGTH=339 | 201606
Arabidopsis_thaliana   OG0000051
AT2G21610.1 | pectinesterase 11 | Chr2:9245161-9247025 REVERSE
LENGTH=352 | 201606   Arabidopsis_thaliana   OG0000051
AT2G26440.1 | Plant invertase/pectin methylesterase inhibitor
superfamily | Chr2:11247407-11249407 FORWARD LENGTH=547 | 201606
Arabidopsis_thaliana   OG0000051
AT2G26450.1 | Plant invertase/pectin methylesterase inhibitor
superfamily | Chr2:11251279-11253446 FORWARD LENGTH=614 | 201606
Arabidopsis_thaliana   OG0000051
Os01t0234499-00 Similar to Pectinesterase. Oryza_sativa
OG0000051
Os03t0300500-01 Similar to Pectin methylesterase 6 (Fragment).
Oryza_sativa   OG0000051
Os03t0309400-01 Pectin lyase fold domain containing protein.
Oryza_sativa   OG0000051
Os03t0399000-01 Pectin lyase fold domain containing protein.
Oryza_sativa   OG0000051
Os01t0254300-00 Pectinesterase (EC 3.1.1.11) (Fragment).
Oryza_sativa   OG0000051
Os04t0438400-01 Similar to Pectin methylesterase-like protein.
Oryza_sativa   OG0000051
Os04t0458900-01 Similar to Pectin methylesterase-like protein.
Oryza_sativa   OG0000051
Os01t0311800-01 Similar to Pectin methylesterase 8 (Fragment).
Oryza_sativa   OG0000051
Os01t0312500-01 Similar to Pectinesterase. Oryza_sativa
OG0000051

Os04t0553500-01 Pectin lyase fold domain containing protein.
Oryza_sativa OG0000051
Os04t0641200-01 Similar to Pectin methylesterase-like protein.
Oryza_sativa OG0000051
Os05t0361500-01 Similar to Pectinesterase. Oryza_sativa
OG0000051
Os05t0521600-01 Pectin lyase fold/virulence factor domain containing protein. Oryza_sativa OG0000051
Os06t0193200-01 Similar to Pectin methylesterase 8 (Fragment).
Oryza_sativa OG0000051
Os07t0607400-01 Pectin lyase fold/virulence factor domain containing protein. Oryza_sativa OG0000051
Os07t0655600-01 Pectin lyase fold/virulence factor domain containing protein. Oryza_sativa OG0000051
Os07t0675100-01 Similar to Pectin methylesterase isoform alpha (EC 3.1.1.11) (Fragment). Oryza_sativa OG0000051
Os07t0691100-01 Similar to Pectin methylesterase 6 (Fragment).
Oryza_sativa OG0000051
Os01t0634600-00 Pectin lyase fold/virulence factor domain containing protein. Oryza_sativa OG0000051
Os08t0206950-00 Similar to Pectinesterase. Oryza_sativa
OG0000051
Os08t0220400-01 Pectin lyase fold/virulence factor domain containing protein. Oryza_sativa OG0000051
Os08t0450100-01 Similar to Pectinesterase (EC 3.1.1.11) (Fragment).
Oryza_sativa OG0000051
Os08t0450200-01 Similar to Pectin methylesterase (Fragment).
Oryza_sativa OG0000051
Os09t0433700-01 Similar to Pectin methylesterase (Fragment).
Oryza_sativa OG0000051
Os09t0571100-01 Pectin lyase fold/virulence factor domain containing protein. Oryza_sativa OG0000051
Os10t0407000-01 Pectin lyase fold/virulence factor domain containing protein. Oryza_sativa OG0000051
Os11t0172100-00 Similar to Pectinesterase. Oryza_sativa
OG0000051
Os11t0192400-01 Pectin lyase fold/virulence factor domain containing protein. Oryza_sativa OG0000051
Os11t0194000-00 Similar to Pectinesterase. Oryza_sativa
OG0000051
Os01t0743200-01 Pectin lyase fold/virulence factor domain containing protein. Oryza_sativa OG0000051
Os11t0659600-01 Pectinesterase, catalytic domain containing protein.
Oryza_sativa OG0000051
Os11t0683700-01 Pectin lyase fold domain containing protein.
Oryza_sativa OG0000051
Os11t0683800-01 Pectin lyase fold/virulence factor domain containing protein. Oryza_sativa OG0000051
Os12t0563700-01 Pectin lyase fold domain containing protein.
Oryza_sativa OG0000051
Os01t0788400-01 Similar to Pectinesterase (EC 3.1.1.11) (Fragment).
Oryza_sativa OG0000051
Os01t0880300-02 Similar to Pectin methylesterase-like protein.
Oryza_sativa OG0000051

Os02t0288100-01 Similar to Pectinesterase (EC 3.1.1.11) (Fragment).
Oryza_sativa OG0000051
Os02t0783000-01 Similar to Pectin methylesterase 5 (Fragment).
Oryza_sativa OG0000051
g566.t1 Caulerpa_lentillifera OG0000052
g642.t1 Caulerpa_lentillifera OG0000052
g7914.t1 Caulerpa_lentillifera OG0000052
g8134.t1 Caulerpa_lentillifera OG0000052
Cre01.g002300.t1.2 pacid=30788660 transcript=Cre01.g002300.t1.2 locus=Cre01.g002300 ID=Cre01.g002300.t1.2.v5.5 annot-version=v5.5
Chlamydomonas_reinhardtii OG0000052
Cre01.g037400.t1.1 pacid=30789223 transcript=Cre01.g037400.t1.1 locus=Cre01.g037400 ID=Cre01.g037400.t1.1.v5.5 annot-version=v5.5
Chlamydomonas_reinhardtii OG0000052
Cre12.g544150.t1.2 pacid=30791953 transcript=Cre12.g544150.t1.2 locus=Cre12.g544150 ID=Cre12.g544150.t1.2.v5.5 annot-version=v5.5
Chlamydomonas_reinhardtii OG0000052
Cre12.g544114.t1.1 pacid=30792216 transcript=Cre12.g544114.t1.1 locus=Cre12.g544114 ID=Cre12.g544114.t1.1.v5.5 annot-version=v5.5
Chlamydomonas_reinhardtii OG0000052
Cre12.g533800.t1.2 pacid=30792269 transcript=Cre12.g533800.t1.2 locus=Cre12.g533800 ID=Cre12.g533800.t1.2.v5.5 annot-version=v5.5
Chlamydomonas_reinhardtii OG0000052
Cre12.g495951.t2.1 pacid=30792446 transcript=Cre12.g495951.t2.1 locus=Cre12.g495951 ID=Cre12.g495951.t2.1.v5.5 annot-version=v5.5
Chlamydomonas_reinhardtii OG0000052
Cre14.g619300.t1.1 pacid=30776159 transcript=Cre14.g619300.t1.1 locus=Cre14.g619300 ID=Cre14.g619300.t1.1.v5.5 annot-version=v5.5
Chlamydomonas_reinhardtii OG0000052
Cre06.g284900.t1.2 pacid=30778769 transcript=Cre06.g284900.t1.2 locus=Cre06.g284900 ID=Cre06.g284900.t1.2.v5.5 annot-version=v5.5
Chlamydomonas_reinhardtii OG0000052
Cre13.g588100.t1.2 pacid=30783969 transcript=Cre13.g588100.t1.2 locus=Cre13.g588100 ID=Cre13.g588100.t1.2.v5.5 annot-version=v5.5
Chlamydomonas_reinhardtii OG0000052
Vocar.0021s0098.1.p pacid=32895950 transcript=Vocar.0021s0098.1 locus=Vocar.0021s0098 ID=Vocar.0021s0098.1.v2.1 annot-version=v2.1
Volvox_carteri OG0000052
Vocar.0020s0013.1.p pacid=32886724 transcript=Vocar.0020s0013.1 locus=Vocar.0020s0013 ID=Vocar.0020s0013.1.v2.1 annot-version=v2.1
Volvox_carteri OG0000052
Vocar.0007s0128.1.p pacid=32888199 transcript=Vocar.0007s0128.1 locus=Vocar.0007s0128 ID=Vocar.0007s0128.1.v2.1 annot-version=v2.1
Volvox_carteri OG0000052
Vocar.0031s0055.1.p pacid=32890185 transcript=Vocar.0031s0055.1 locus=Vocar.0031s0055 ID=Vocar.0031s0055.1.v2.1 annot-version=v2.1
Volvox_carteri OG0000052
Vocar.0024s0222.1.p pacid=32890356 transcript=Vocar.0024s0222.1 locus=Vocar.0024s0222 ID=Vocar.0024s0222.1.v2.1 annot-version=v2.1
Volvox_carteri OG0000052
Vocar.0002s0004.1.p pacid=32891244 transcript=Vocar.0002s0004.1 locus=Vocar.0002s0004 ID=Vocar.0002s0004.1.v2.1 annot-version=v2.1
Volvox_carteri OG0000052
Vocar.0034s0033.1.p pacid=32893104 transcript=Vocar.0034s0033.1

locus=Vocar.0034s0033 ID=Vocar.0034s0033.1.v2.1 annot-version=v2.1
Volvox_carteri   OG0000052
jgi|ChlNC64A_1|29941|estExt_Genewise1.C_30673
Chlorella_variabilis   OG0000052
jgi|ChlNC64A_1|35742|estExt_Genewise1Plus.C_120139
Chlorella_variabilis   OG0000052
jgi|ChlNC64A_1|13673|gw1.12.363.1 Chlorella_variabilis
OG0000052
jgi|ChlNC64A_1|12885|gw1.2.362.1 Chlorella_variabilis
OG0000052
jgi|ChlNC64A_1|24996|e_gw1.14.299.1   Chlorella_variabilis
OG0000052
jgi|ChlNC64A_1|143107|IGS.gm_5_00436   Chlorella_variabilis
OG0000052
jgi|ChlNC64A_1|22373|e_gw1.8.270.1   Chlorella_variabilis
OG0000052
jgi|ChlNC64A_1|26227|e_gw1.19.151.1   Chlorella_variabilis
OG0000052
16087 pacid=27388567 transcript=16087 locus=e_gw1.9.343.1
ID=16087.2.0.227 annot-version=v2.0   Coccomyxa_subellipsoidea
OG0000052
24264 pacid=27386002 transcript=24264
locus=estExt_Genewise1.C_100235 ID=24264.2.0.227 annot-version=v2.0
Coccomyxa_subellipsoidea OG0000052
11218 pacid=27389505 transcript=11218 locus=e_gw1.1.719.1
ID=11218.2.0.227 annot-version=v2.0   Coccomyxa_subellipsoidea
OG0000052
29609 pacid=27385685 transcript=29609
locus=estExt_Genewise1Plus.C_100204 ID=29609.2.0.227 annot-
version=v2.0   Coccomyxa_subellipsoidea OG0000052
12651 pacid=27390620 transcript=12651 locus=e_gw1.3.480.1
ID=12651.2.0.227 annot-version=v2.0   Coccomyxa_subellipsoidea
OG0000052
47524 pacid=27392391 transcript=47524
locus=estExt_fgenesh1_pg.C_80140 ID=47524.2.0.227 annot-version=v2.0
        Coccomyxa_subellipsoidea OG0000052
18417 pacid=27394949 transcript=18417 locus=e_gw1.15.293.1
ID=18417.2.0.227 annot-version=v2.0   Coccomyxa_subellipsoidea
OG0000052
26133 pacid=27395118 transcript=26133
locus=estExt_Genewise1.C_210127 ID=26133.2.0.227 annot-version=v2.0
Coccomyxa_subellipsoidea OG0000052
19603_e_gw1.10.00.373.1   Ostreococcus_tauri   OG0000052
19795_e_gw1.10.00.346.1   Ostreococcus_tauri   OG0000052
27939_estExt_gwp_GeneWisePlus.C_Chr_03.00010435
Ostreococcus_tauri   OG0000052
29712_estExt_gwp_GeneWisePlus.C_Chr_17.00010099
Ostreococcus_tauri   OG0000052
6672_gw1.07.00.408.1   Ostreococcus_tauri   OG0000052
6824_gw1.06.00.399.1   Ostreococcus_tauri   OG0000052
6901_gw1.08.00.346.1   Ostreococcus_tauri   OG0000052
6913_gw1.10.00.383.1   Ostreococcus_tauri   OG0000052
8685_fgenesh1_pm.C_Chr_09.0001000021   Ostreococcus_tauri
OG0000052

8882_fgenesh1_pm.C_Chr_12.0001000018	Ostreococcus_tauri
OG0000052
32742 pacid=27414090 transcript=32742 locus=eugene.0700010339
ID=32742.2.0.231 annot-version=v2.0	Ostreococcus_lucimarinus
OG0000052
28138 pacid=27415828 transcript=28138 locus=eugene.1600010194
ID=28138.2.0.231 annot-version=v2.0	Ostreococcus_lucimarinus
OG0000052
8538 pacid=27416061 transcript=8538 locus=gwEuk.10.433.1
ID=8538.2.0.231 annot-version=v2.0	Ostreococcus_lucimarinus
OG0000052
38323 pacid=27416196 transcript=38323 locus=e_gwEuk.10.512.1
ID=38323.2.0.231 annot-version=v2.0	Ostreococcus_lucimarinus
OG0000052
37985 pacid=27416199 transcript=37985 locus=e_gwEuk.10.372.1
ID=37985.2.0.231 annot-version=v2.0	Ostreococcus_lucimarinus
OG0000052
8483 pacid=27416314 transcript=8483 locus=gwEuk.10.430.1
ID=8483.2.0.231 annot-version=v2.0	Ostreococcus_lucimarinus
OG0000052
7102 pacid=27417170 transcript=7102 locus=gwEuk.12.307.1
ID=7102.2.0.231 annot-version=v2.0	Ostreococcus_lucimarinus
OG0000052
34076 pacid=27413388 transcript=34076 locus=e_gwEuk.3.674.1
ID=34076.2.0.231 annot-version=v2.0	Ostreococcus_lucimarinus
OG0000052
41188 pacid=27418450 transcript=41188 locus=e_gwEuk.6.522.1
ID=41188.2.0.231 annot-version=v2.0	Ostreococcus_lucimarinus
OG0000052
42852 pacid=27419242 transcript=42852 locus=e_gwEuk.8.482.1
ID=42852.2.0.231 annot-version=v2.0	Ostreococcus_lucimarinus
OG0000052
35139 pacid=27413670 transcript=35139 locus=e_gwEuk.9.510.1
ID=35139.2.0.231 annot-version=v2.0	Ostreococcus_lucimarinus
OG0000052
35671 pacid=27339651 transcript=35671
locus=MicpuC2.estExt_Genewise1Plus.C_110367 ID=35671.3.0.228 annot-
version=v3.0	Micromonas_pusilla	OG0000052
156024 pacid=27341330 transcript=156064 locus=e_gw1.5.1142.1
ID=156064.3.0.228 annot-version=v3.0	Micromonas_pusilla
OG0000052
123350 pacid=27341477 transcript=123390 locus=fgenesh2_kg.
5_#_124_#_4272007:1 ID=123390.3.0.228 annot-version=v3.0
Micromonas_pusilla	OG0000052
58285 pacid=27341707 transcript=58285 locus=MicpuC2.EuGene.
0000050590 ID=58285.3.0.228 annot-version=v3.0
Micromonas_pusilla	OG0000052
182504 pacid=27343524 transcript=182544
locus=estExt_Genewise1Plus.C_16_t20045 ID=182544.3.0.228 annot-
version=v3.0	Micromonas_pusilla	OG0000052
195080 pacid=27339276 transcript=195120
locus=estExt_Genemark1.C_110315 ID=195120.3.0.228 annot-version=v3.0
	Micromonas_pusilla	OG0000052
50452 pacid=27345134 transcript=50452 locus=MicpuC2.EuGene.

0000010676 ID=50452.3.0.228 annot-version=v3.0
Micromonas_pusilla OG0000052
129002 pacid=27345618 transcript=129042 locus=gw1.1.129.1
ID=129042.3.0.228 annot-version=v3.0 Micromonas_pusilla
OG0000052
183545 pacid=27345967 transcript=183585 locus=gm1.666_g
ID=183585.3.0.228 annot-version=v3.0 Micromonas_pusilla
OG0000052
33009 pacid=27346278 transcript=33009
locus=MicpuC2.estExt_Genewise1Plus.C_40487 ID=33009.3.0.228 annot-version=v3.0 Micromonas_pusilla OG0000052
161197 pacid=27339426 transcript=161237 locus=e_gw1.11.106.1
ID=161237.3.0.228 annot-version=v3.0 Micromonas_pusilla
OG0000052
185702 pacid=27346645 transcript=185742 locus=gm1.2823_g
ID=185742.3.0.228 annot-version=v3.0 Micromonas_pusilla
OG0000052
8217 pacid=27347268 transcript=8217 locus=MicpuC2.gw1.3.851.1
ID=8217.3.0.228 annot-version=v3.0 Micromonas_pusilla
OG0000052
107701 pacid=27396904 transcript=107701
locus=est_cluster_kg.Chr_02__65__3200643:1 ID=107701.3.0.229 annot-version=v3.0 Micromonas_sp._RCC299 OG0000052
58982 pacid=27395505 transcript=58982 locus=EuGene.0500010715
ID=58982.3.0.229 annot-version=v3.0 Micromonas_sp._RCC299
OG0000052
96416 pacid=27401987 transcript=96416
locus=fgenesh2_kg.C_Chr_06000031 ID=96416.3.0.229 annot-version=v3.0
Micromonas_sp._RCC299 OG0000052
97605 pacid=27402283 transcript=97605
locus=fgenesh2_pm.C_Chr_06000136 ID=97605.3.0.229 annot-version=v3.0
Micromonas_sp._RCC299 OG0000052
107024 pacid=27403274 transcript=107024
locus=estExt_fgenesh2_pm.C_Chr_080021 ID=107024.3.0.229 annot-version=v3.0 Micromonas_sp._RCC299 OG0000052
84408 pacid=27403291 transcript=84408 locus=e_gw2.08.507.1
ID=84408.3.0.229 annot-version=v3.0 Micromonas_sp._RCC299
OG0000052
60515 pacid=27403390 transcript=60515 locus=EuGene.0800010083
ID=60515.3.0.229 annot-version=v3.0 Micromonas_sp._RCC299
OG0000052
60844 pacid=27403497 transcript=60844 locus=EuGene.0800010412
ID=60844.3.0.229 annot-version=v3.0 Micromonas_sp._RCC299
OG0000052
84244 pacid=27403643 transcript=84244 locus=e_gw2.08.453.1
ID=84244.3.0.229 annot-version=v3.0 Micromonas_sp._RCC299
OG0000052
98302 pacid=27403722 transcript=98302
locus=fgenesh2_pm.C_Chr_14000013 ID=98302.3.0.229 annot-version=v3.0
Micromonas_sp._RCC299 OG0000052
56070 pacid=27396117 transcript=56070 locus=EuGene.0200010447
ID=56070.3.0.229 annot-version=v3.0 Micromonas_sp._RCC299
OG0000052
106992 pacid=27404113 transcript=106992

locus=estExt_fgenesh2_pm.C_Chr_070023 ID=106992.3.0.229 annot-version=v3.0 Micromonas_sp._RCC299 OG0000052
73476 pacid=27404312 transcript=73476 locus=gw2.07.439.1 ID=73476.3.0.229 annot-version=v3.0 Micromonas_sp._RCC299 OG0000052
97666 pacid=27404387 transcript=97666 locus=fgenesh2_pm.C_Chr_07000048 ID=97666.3.0.229 annot-version=v3.0 Micromonas_sp._RCC299 OG0000052
kfl00355_0110_v1.1 Klebsormidium_flaccidum OG0000052
kfl00016_0060_v1.1 Klebsormidium_flaccidum OG0000052
kfl00042_0050_v1.1 Klebsormidium_flaccidum OG0000052
kfl00071_0230_v1.1 Klebsormidium_flaccidum OG0000052
kfl00085_0400_v1.1 Klebsormidium_flaccidum OG0000052
kfl00086_0260_v1.1 Klebsormidium_flaccidum OG0000052
kfl00133_0120_v1.1 Klebsormidium_flaccidum OG0000052
kfl00289_0160_v1.1 Klebsormidium_flaccidum OG0000052
kfl00304_0050_v1.1 Klebsormidium_flaccidum OG0000052
Pp3c7_6420V3.1.p pacid=32926346 transcript=Pp3c7_6420V3.1 locus=Pp3c7_6420 annot-version=v3.3 Physcomitrella_patens OG0000052
Pp3c13_5300V3.1.p pacid=32930151 transcript=Pp3c13_5300V3.1 locus=Pp3c13_5300 annot-version=v3.3 Physcomitrella_patens OG0000052
Pp3c13_22770V3.1.p pacid=32931341 transcript=Pp3c13_22770V3.1 locus=Pp3c13_22770 annot-version=v3.3 Physcomitrella_patens OG0000052
Pp3c13_1510V3.1.p pacid=32931787 transcript=Pp3c13_1510V3.1 locus=Pp3c13_1510 annot-version=v3.3 Physcomitrella_patens OG0000052
Pp3c22_15430V3.1.p pacid=32903634 transcript=Pp3c22_15430V3.1 locus=Pp3c22_15430 annot-version=v3.3 Physcomitrella_patens OG0000052
Pp3c23_2850V3.1.p pacid=32949576 transcript=Pp3c23_2850V3.1 locus=Pp3c23_2850 annot-version=v3.3 Physcomitrella_patens OG0000052
Pp3c5_6120V3.1.p pacid=32953510 transcript=Pp3c5_6120V3.1 locus=Pp3c5_6120 annot-version=v3.3 Physcomitrella_patens OG0000052
Pp3c11_4980V3.1.p pacid=32957526 transcript=Pp3c11_4980V3.1 locus=Pp3c11_4980 annot-version=v3.3 Physcomitrella_patens OG0000052
Pp3c11_24900V3.1.p pacid=32958732 transcript=Pp3c11_24900V3.1 locus=Pp3c11_24900 annot-version=v3.3 Physcomitrella_patens OG0000052
Pp3c8_17390V3.1.p pacid=32964908 transcript=Pp3c8_17390V3.1 locus=Pp3c8_17390 annot-version=v3.3 Physcomitrella_patens OG0000052
Pp3c1_32280V3.1.p pacid=32967543 transcript=Pp3c1_32280V3.1 locus=Pp3c1_32280 annot-version=v3.3 Physcomitrella_patens OG0000052
Pp3c6_27160V3.1.p pacid=32978787 transcript=Pp3c6_27160V3.1 locus=Pp3c6_27160 annot-version=v3.3 Physcomitrella_patens OG0000052
Pp3c24_16740V3.1.p pacid=32909990 transcript=Pp3c24_16740V3.1

locus=Pp3c24_16740 annot-version=v3.3 Physcomitrella_patens OG0000052
Pp3c24_9660V3.1.p pacid=32910114 transcript=Pp3c24_9660V3.1 locus=Pp3c24_9660 annot-version=v3.3 Physcomitrella_patens OG0000052
Pp3c7_8500V3.1.p pacid=32923454 transcript=Pp3c7_8500V3.1 locus=Pp3c7_8500 annot-version=v3.3 Physcomitrella_patens OG0000052
AT2G38730.1 | Cyclophilin-like peptidyl-prolyl cis-trans isomerase family protein | Chr2:16192579-16194038 REVERSE LENGTH=199 | 201606 Arabidopsis_thaliana OG0000052
AT3G22920.1 | Cyclophilin-like peptidyl-prolyl cis-trans isomerase family protein | Chr3:8122713-8123411 REVERSE LENGTH=232 | 201606 Arabidopsis_thaliana OG0000052
AT3G55920.1 | Cyclophilin-like peptidyl-prolyl cis-trans isomerase family protein | Chr3:20743529-20745049 REVERSE LENGTH=228 | 201606 Arabidopsis_thaliana OG0000052
AT3G56070.1 | rotamase cyclophilin 2 | Chr3:20806987-20807517 REVERSE LENGTH=176 | 201606 Arabidopsis_thaliana OG0000052
AT3G62030.1 | rotamase CYP 4 | Chr3:22973708-22975139 FORWARD LENGTH=260 | 201606 Arabidopsis_thaliana OG0000052
AT3G63400.1 | Cyclophilin-like peptidyl-prolyl cis-trans isomerase family protein | Chr3:23412449-23415435 FORWARD LENGTH=570 | 201606 Arabidopsis_thaliana OG0000052
AT4G34870.1 | rotamase cyclophilin 5 | Chr4:16614451-16614969 FORWARD LENGTH=172 | 201606 Arabidopsis_thaliana OG0000052
AT4G34960.1 | Cyclophilin-like peptidyl-prolyl cis-trans isomerase family protein | Chr4:16648865-16650646 FORWARD LENGTH=224 | 201606 Arabidopsis_thaliana OG0000052
AT4G38740.1 | rotamase CYP 1 | Chr4:18083620-18084138 REVERSE LENGTH=172 | 201606 Arabidopsis_thaliana OG0000052
AT5G13120.1 | cyclophilin 20-2 | Chr5:4162714-4164720 REVERSE LENGTH=259 | 201606 Arabidopsis_thaliana OG0000052
AT5G58710.1 | rotamase CYP 7 | Chr5:23717840-23719495 FORWARD LENGTH=204 | 201606 Arabidopsis_thaliana OG0000052
AT2G16600.1 | rotamase CYP 3 | Chr2:7200862-7201383 FORWARD LENGTH=173 | 201606 Arabidopsis_thaliana OG0000052
AT2G21130.1 | Cyclophilin-like peptidyl-prolyl cis-trans isomerase family protein | Chr2:9055619-9056143 REVERSE LENGTH=174 | 201606 Arabidopsis_thaliana OG0000052
AT2G29960.1 | cyclophilin 5 | Chr2:12769183-12770528 REVERSE LENGTH=201 | 201606 Arabidopsis_thaliana OG0000052
Os03t0811600-01 Similar to Peptidyl prolyl isomerase H. Oryza_sativa OG0000052
Os01t0284700-01 Similar to Peptidyl-prolyl cis-trans isomerase. Oryza_sativa OG0000052
Os05t0103200-01 Cyclophilin-like domain containing protein. Oryza_sativa OG0000052
Os06t0708400-01 Cyclophilin, Cold tolerance Oryza_sativa OG0000052
Os06t0708500-01 Similar to Peptidyl-prolyl cis-trans isomerase. Oryza_sativa OG0000052

Os09t0537600-01 Similar to Cyclophilin-like protein (Single domain cyclophilin type peptidyl- prolyl cis-trans isomerase).
Oryza_sativa OG0000052
Os09t0571400-01 Cyclophilin 1. Oryza_sativa OG0000052
Os10t0154700-01 Similar to Cyclophilin Dicyp-2. Oryza_sativa OG0000052
Os02t0121300-01 Cyclophilin, Peptidyl-prolyl cis-trans isomerase, Auxin signal transduction, Lateral root initiation, Stress tolerance
Oryza_sativa OG0000052
CMR272CT pep chromosome:ASM9120v1:18:661264:662019:1 gene:CMR272C transcript:CMR272CT gene_biotype:protein_coding transcript_biotype:protein_coding Cyanidioschyzon_merolae OG0000052
CMP271CT pep chromosome:ASM9120v1:16:674120:675052:1 gene:CMP271C transcript:CMP271CT gene_biotype:protein_coding transcript_biotype:protein_coding Cyanidioschyzon_merolae OG0000052
CMO300CT pep chromosome:ASM9120v1:15:754569:755075:1 gene:CMO300C transcript:CMO300CT gene_biotype:protein_coding transcript_biotype:protein_coding Cyanidioschyzon_merolae OG0000052
evm.model.contig_2165.10 Porphyridium_purpureum OG0000052
evm.model.contig_3395.3 Porphyridium_purpureum OG0000052
evm.model.contig_3403.3 Porphyridium_purpureum OG0000052
evm.model.contig_3418.3 Porphyridium_purpureum OG0000052
evm.model.contig_4404.13 Porphyridium_purpureum OG0000052
evm.model.contig_4604.1 Porphyridium_purpureum OG0000052
evm.model.contig_524.3 Porphyridium_purpureum OG0000052
evm.model.contig_699.9 Porphyridium_purpureum OG0000052
CDF36948 pep supercontig:ASM35022v2:HG001808:73782:74831:-1 gene:CHC_T00008420001 transcript:CDF36948 gene_biotype:protein_coding transcript_biotype:protein_coding description:Peptidyl-prolyl cis-trans isomerase Chondrus_crispus OG0000052
CDF40125 pep supercontig:ASM35022v2:HG002126:330412:331438:-1 gene:CHC_T00006996001 transcript:CDF40125 gene_biotype:protein_coding transcript_biotype:protein_coding Chondrus_crispus OG0000052
CDF38419 pep supercontig:ASM35022v2:HG001932:202012:202827:-1 gene:CHC_T00006154001 transcript:CDF38419 gene_biotype:protein_coding transcript_biotype:protein_coding Chondrus_crispus OG0000052
CDF77398 pep supercontig:ASM35022v2:HG001523:204767:205126:-1 gene:CHC_T00007912001 transcript:CDF77398 gene_biotype:protein_coding transcript_biotype:protein_coding Chondrus_crispus OG0000052
CDF33758 pep supercontig:ASM35022v2:HG001657:144050:144622:1 gene:CHC_T00010100001 transcript:CDF33758 gene_biotype:protein_coding transcript_biotype:protein_coding description:Peptidyl-prolyl cis-trans isomerase Chondrus_crispus OG0000052
CDF32123 pep supercontig:ASM35022v2:HG001459:244132:245356:1 gene:CHC_T00008599001 transcript:CDF32123 gene_biotype:protein_coding transcript_biotype:protein_coding

description:peptidyl-prolyl cis-trans isomerase	Chondrus_crispus OG0000052
g1087.t1 Caulerpa_lentillifera	OG0000053
g5177.t1 Caulerpa_lentillifera	OG0000053
g7644.t1 Caulerpa_lentillifera	OG0000053
g8623.t1 Caulerpa_lentillifera	OG0000053
g8913.t1 Caulerpa_lentillifera	OG0000053
Cre02.g112750.t1.2 pacid=30785823 transcript=Cre02.g112750.t1.2 locus=Cre02.g112750 ID=Cre02.g112750.t1.2.v5.5 annot-version=v5.5 Chlamydomonas_reinhardtii OG0000053
Cre02.g110150.t1.2 pacid=30786240 transcript=Cre02.g110150.t1.2 locus=Cre02.g110150 ID=Cre02.g110150.t1.2.v5.5 annot-version=v5.5 Chlamydomonas_reinhardtii OG0000053
Cre02.g104700.t1.2 pacid=30786376 transcript=Cre02.g104700.t1.2 locus=Cre02.g104700 ID=Cre02.g104700.t1.2.v5.5 annot-version=v5.5 Chlamydomonas_reinhardtii OG0000053
Cre02.g085150.t1.1 pacid=30786461 transcript=Cre02.g085150.t1.1 locus=Cre02.g085150 ID=Cre02.g085150.t1.1.v5.5 annot-version=v5.5 Chlamydomonas_reinhardtii OG0000053
Cre01.g033900.t1.1 pacid=30788409 transcript=Cre01.g033900.t1.1 locus=Cre01.g033900 ID=Cre01.g033900.t1.1.v5.5 annot-version=v5.5 Chlamydomonas_reinhardtii OG0000053
Cre01.g070932.t1.1 pacid=30788643 transcript=Cre01.g070932.t1.1 locus=Cre01.g070932 ID=Cre01.g070932.t1.1.v5.5 annot-version=v5.5 Chlamydomonas_reinhardtii OG0000053
Cre01.g043550.t1.1 pacid=30788943 transcript=Cre01.g043550.t1.1 locus=Cre01.g043550 ID=Cre01.g043550.t1.1.v5.5 annot-version=v5.5 Chlamydomonas_reinhardtii OG0000053
Cre01.g012200.t1.1 pacid=30789471 transcript=Cre01.g012200.t1.1 locus=Cre01.g012200 ID=Cre01.g012200.t1.1.v5.5 annot-version=v5.5 Chlamydomonas_reinhardtii OG0000053
Cre10.g433350.t1.1 pacid=30790155 transcript=Cre10.g433350.t1.1 locus=Cre10.g433350 ID=Cre10.g433350.t1.1.v5.5 annot-version=v5.5 Chlamydomonas_reinhardtii OG0000053
Cre10.g421550.t2.1 pacid=30790606 transcript=Cre10.g421550.t2.1 locus=Cre10.g421550 ID=Cre10.g421550.t2.1.v5.5 annot-version=v5.5 Chlamydomonas_reinhardtii OG0000053
Cre07.g325738.t1.1 pacid=30775112 transcript=Cre07.g325738.t1.1 locus=Cre07.g325738 ID=Cre07.g325738.t1.1.v5.5 annot-version=v5.5 Chlamydomonas_reinhardtii OG0000053
Cre04.g212700.t1.1 pacid=30791083 transcript=Cre04.g212700.t1.1 locus=Cre04.g212700 ID=Cre04.g212700.t1.1.v5.5 annot-version=v5.5 Chlamydomonas_reinhardtii OG0000053
Cre07.g345050.t1.2 pacid=30775147 transcript=Cre07.g345050.t1.2 locus=Cre07.g345050 ID=Cre07.g345050.t1.2.v5.5 annot-version=v5.5 Chlamydomonas_reinhardtii OG0000053
Cre07.g335150.t1.1 pacid=30775323 transcript=Cre07.g335150.t1.1 locus=Cre07.g335150 ID=Cre07.g335150.t1.1.v5.5 annot-version=v5.5 Chlamydomonas_reinhardtii OG0000053
Cre16.g683953.t1.1 pacid=30777197 transcript=Cre16.g683953.t1.1 locus=Cre16.g683953 ID=Cre16.g683953.t1.1.v5.5 annot-version=v5.5 Chlamydomonas_reinhardtii OG0000053
Cre16.g673250.t1.1 pacid=30777414 transcript=Cre16.g673250.t1.1 locus=Cre16.g673250 ID=Cre16.g673250.t1.1.v5.5 annot-version=v5.5

Chlamydomonas_reinhardtii OG0000053
Cre16.g692500.t1.1 pacid=30777567 transcript=Cre16.g692500.t1.1
locus=Cre16.g692500 ID=Cre16.g692500.t1.1.v5.5 annot-version=v5.5
Chlamydomonas_reinhardtii OG0000053
Cre16.g686100.t1.1 pacid=30777585 transcript=Cre16.g686100.t1.1
locus=Cre16.g686100 ID=Cre16.g686100.t1.1.v5.5 annot-version=v5.5
Chlamydomonas_reinhardtii OG0000053
Cre06.g278229.t1.1 pacid=30778862 transcript=Cre06.g278229.t1.1
locus=Cre06.g278229 ID=Cre06.g278229.t1.1.v5.5 annot-version=v5.5
Chlamydomonas_reinhardtii OG0000053
Cre06.g300600.t1.1 pacid=30780087 transcript=Cre06.g300600.t1.1
locus=Cre06.g300600 ID=Cre06.g300600.t1.1.v5.5 annot-version=v5.5
Chlamydomonas_reinhardtii OG0000053
Cre09.g399289.t1.1 pacid=30780920 transcript=Cre09.g399289.t1.1
locus=Cre09.g399289 ID=Cre09.g399289.t1.1.v5.5 annot-version=v5.5
Chlamydomonas_reinhardtii OG0000053
Cre17.g698233.t1.1 pacid=30782131 transcript=Cre17.g698233.t1.1
locus=Cre17.g698233 ID=Cre17.g698233.t1.1.v5.5 annot-version=v5.5
Chlamydomonas_reinhardtii OG0000053
Cre05.g233551.t1.1 pacid=30783356 transcript=Cre05.g233551.t1.1
locus=Cre05.g233551 ID=Cre05.g233551.t1.1.v5.5 annot-version=v5.5
Chlamydomonas_reinhardtii OG0000053
Vocar.0012s0159.1.p pacid=32894892 transcript=Vocar.0012s0159.1
locus=Vocar.0012s0159 ID=Vocar.0012s0159.1.v2.1 annot-version=v2.1
Volvox_carteri OG0000053
Vocar.0011s0018.1.p pacid=32895276 transcript=Vocar.0011s0018.1
locus=Vocar.0011s0018 ID=Vocar.0011s0018.1.v2.1 annot-version=v2.1
Volvox_carteri OG0000053
Vocar.0028s0178.1.p pacid=32895527 transcript=Vocar.0028s0178.1
locus=Vocar.0028s0178 ID=Vocar.0028s0178.1.v2.1 annot-version=v2.1
Volvox_carteri OG0000053
Vocar.0013s0255.1.p pacid=32896409 transcript=Vocar.0013s0255.1
locus=Vocar.0013s0255 ID=Vocar.0013s0255.1.v2.1 annot-version=v2.1
Volvox_carteri OG0000053
Vocar.0035s0051.1.p pacid=32896844 transcript=Vocar.0035s0051.1
locus=Vocar.0035s0051 ID=Vocar.0035s0051.1.v2.1 annot-version=v2.1
Volvox_carteri OG0000053
Vocar.0025s0134.1.p pacid=32898392 transcript=Vocar.0025s0134.1
locus=Vocar.0025s0134 ID=Vocar.0025s0134.1.v2.1 annot-version=v2.1
Volvox_carteri OG0000053
Vocar.0001s0492.1.p pacid=32884700 transcript=Vocar.0001s0492.1
locus=Vocar.0001s0492 ID=Vocar.0001s0492.1.v2.1 annot-version=v2.1
Volvox_carteri OG0000053
Vocar.0018s0197.1.p pacid=32898583 transcript=Vocar.0018s0197.1
locus=Vocar.0018s0197 ID=Vocar.0018s0197.1.v2.1 annot-version=v2.1
Volvox_carteri OG0000053
Vocar.0001s0783.1.p pacid=32884860 transcript=Vocar.0001s0783.1
locus=Vocar.0001s0783 ID=Vocar.0001s0783.1.v2.1 annot-version=v2.1
Volvox_carteri OG0000053
Vocar.0001s0653.1.p pacid=32885344 transcript=Vocar.0001s0653.1
locus=Vocar.0001s0653 ID=Vocar.0001s0653.1.v2.1 annot-version=v2.1
Volvox_carteri OG0000053
Vocar.0001s1587.1.p pacid=32886106 transcript=Vocar.0001s1587.1
locus=Vocar.0001s1587 ID=Vocar.0001s1587.1.v2.1 annot-version=v2.1

Volvox_carteri   OG0000053
Vocar.0005s0477.1.p pacid=32883560 transcript=Vocar.0005s0477.1 locus=Vocar.0005s0477 ID=Vocar.0005s0477.1.v2.1 annot-version=v2.1
Volvox_carteri   OG0000053
Vocar.0005s0367.1.p pacid=32883600 transcript=Vocar.0005s0367.1 locus=Vocar.0005s0367 ID=Vocar.0005s0367.1.v2.1 annot-version=v2.1
Volvox_carteri   OG0000053
Vocar.0003s0172.1.p pacid=32888906 transcript=Vocar.0003s0172.1 locus=Vocar.0003s0172 ID=Vocar.0003s0172.1.v2.1 annot-version=v2.1
Volvox_carteri   OG0000053
Vocar.0003s0435.1.p pacid=32889022 transcript=Vocar.0003s0435.1 locus=Vocar.0003s0435 ID=Vocar.0003s0435.1.v2.1 annot-version=v2.1
Volvox_carteri   OG0000053
Vocar.0002s0311.1.p pacid=32890916 transcript=Vocar.0002s0311.1 locus=Vocar.0002s0311 ID=Vocar.0002s0311.1.v2.1 annot-version=v2.1
Volvox_carteri   OG0000053
Vocar.0002s0008.1.p pacid=32891235 transcript=Vocar.0002s0008.1 locus=Vocar.0002s0008 ID=Vocar.0002s0008.1.v2.1 annot-version=v2.1
Volvox_carteri   OG0000053
Vocar.0022s0024.1.p pacid=32891975 transcript=Vocar.0022s0024.1 locus=Vocar.0022s0024 ID=Vocar.0022s0024.1.v2.1 annot-version=v2.1
Volvox_carteri   OG0000053
Vocar.0042s0081.1.p pacid=32892040 transcript=Vocar.0042s0081.1 locus=Vocar.0042s0081 ID=Vocar.0042s0081.1.v2.1 annot-version=v2.1
Volvox_carteri   OG0000053
Vocar.0042s0082.1.p pacid=32892061 transcript=Vocar.0042s0082.1 locus=Vocar.0042s0082 ID=Vocar.0042s0082.1.v2.1 annot-version=v2.1
Volvox_carteri   OG0000053
jgi|ChlNC64A_1|133755|IGS.gm_10_00046   Chlorella_variabilis OG0000053
jgi|ChlNC64A_1|59250|estExt_fgenesh3_pg.C_300018 Chlorella_variabilis   OG0000053
jgi|ChlNC64A_1|141047|IGS.gm_30_00085   Chlorella_variabilis OG0000053
jgi|ChlNC64A_1|19177|e_gw1.2.707.1   Chlorella_variabilis OG0000053
jgi|ChlNC64A_1|57007|estExt_fgenesh3_pg.C_40020 Chlorella_variabilis   OG0000053
jgi|ChlNC64A_1|143491|IGS.gm_6_00065   Chlorella_variabilis OG0000053
jgi|ChlNC64A_1|17840|gw1.9.419.1 Chlorella_variabilis OG0000053
jgi|ChlNC64A_1|52112|fgenesh3_pg.C_scaffold_9000137 Chlorella_variabilis   OG0000053
jgi|ChlNC64A_1|17960|gw1.9.427.1 Chlorella_variabilis OG0000053
jgi|ChlNC64A_1|145623|IGS.gm_9_00430   Chlorella_variabilis OG0000053
jgi|ChlNC64A_1|138661|IGS.gm_21_00140   Chlorella_variabilis OG0000053
jgi|ChlNC64A_1|133640|IGS.gm_1_00776   Chlorella_variabilis OG0000053
jgi|ChlNC64A_1|133643|IGS.gm_1_00779   Chlorella_variabilis OG0000053

jgi|ChlNC64A_1|28394|e_gw1.34.51.1   Chlorella_variabilis
OG0000053
jgi|ChlNC64A_1|37677|estExt_Genewise1Plus.C_380072
Chlorella_variabilis   OG0000053
jgi|ChlNC64A_1|136317|IGS.gm_16_00040   Chlorella_variabilis
OG0000053
jgi|ChlNC64A_1|138913|IGS.gm_22_00154   Chlorella_variabilis
OG0000053
jgi|ChlNC64A_1|141258|IGS.gm_32_00096   Chlorella_variabilis
OG0000053
jgi|ChlNC64A_1|141274|IGS.gm_32_00112   Chlorella_variabilis
OG0000053
jgi|ChlNC64A_1|58139|estExt_fgenesh3_pg.C_130036
Chlorella_variabilis   OG0000053
jgi|ChlNC64A_1|135230|IGS.gm_13_00158   Chlorella_variabilis
OG0000053
jgi|ChlNC64A_1|135325|IGS.gm_13_00253   Chlorella_variabilis
OG0000053
jgi|ChlNC64A_1|54787|fgenesh3_pg.C_scaffold_25000062
Chlorella_variabilis   OG0000053
jgi|ChlNC64A_1|27387|e_gw1.25.168.1   Chlorella_variabilis
OG0000053
jgi|ChlNC64A_1|139850|IGS.gm_27_00028   Chlorella_variabilis
OG0000053
jgi|ChlNC64A_1|136650|IGS.gm_17_00054   Chlorella_variabilis
OG0000053
jgi|ChlNC64A_1|136680|IGS.gm_17_00084   Chlorella_variabilis
OG0000053
jgi|ChlNC64A_1|136692|IGS.gm_17_00096   Chlorella_variabilis
OG0000053
jgi|ChlNC64A_1|136693|IGS.gm_17_00097   Chlorella_variabilis
OG0000053
jgi|ChlNC64A_1|53768|fgenesh3_pg.C_scaffold_17000101
Chlorella_variabilis   OG0000053
jgi|ChlNC64A_1|140014|IGS.gm_28_00045   Chlorella_variabilis
OG0000053
jgi|ChlNC64A_1|140043|IGS.gm_28_00074   Chlorella_variabilis
OG0000053
61925 pacid=27387119 transcript=61925 locus=Genemark1.2708_g
ID=61925.2.0.227 annot-version=v2.0   Coccomyxa_subellipsoidea
OG0000053
65460 pacid=27387530 transcript=65460 locus=estExt_Genemark1.C_50268
ID=65460.2.0.227 annot-version=v2.0   Coccomyxa_subellipsoidea
OG0000053
55627 pacid=27388952 transcript=55627 locus=Genemark1.571_g
ID=55627.2.0.227 annot-version=v2.0   Coccomyxa_subellipsoidea
OG0000053
64317 pacid=27389345 transcript=64317 locus=estExt_Genemark1.C_10572
ID=64317.2.0.227 annot-version=v2.0   Coccomyxa_subellipsoidea
OG0000053
67762 pacid=27394401 transcript=67762
locus=estExt_Genemark1.C_180179 ID=67762.2.0.227 annot-version=v2.0
Coccomyxa_subellipsoidea OG0000053
47586 pacid=27347916 transcript=47586

locus=MicpuC2.estExt_fgenesh1_pg.C_60304 ID=47586.3.0.228 annot-version=v3.0   Micromonas_pusilla   OG0000053
62070 pacid=27397709 transcript=62070 locus=EuGene.1000010407 ID=62070.3.0.229 annot-version=v3.0   Micromonas_sp._RCC299 OG0000053
52223 pacid=27398581 transcript=52223 locus=est_orfs.Chr_03_2094_3199765:1 ID=52223.3.0.229 annot-version=v3.0   Micromonas_sp._RCC299   OG0000053
105954 pacid=27403391 transcript=105954 locus=estExt_fgenesh2_pg.C_Chr_080215 ID=105954.3.0.229 annot-version=v3.0   Micromonas_sp._RCC299   OG0000053
108680 pacid=27404657 transcript=108680 locus=est_cluster_kg.Chr_07__150__3202459:1 ID=108680.3.0.229 annot-version=v3.0   Micromonas_sp._RCC299   OG0000053
kfl00979_0030_v1.1   Klebsormidium_flaccidum OG0000053
kfl00107_0150_v1.1   Klebsormidium_flaccidum OG0000053
Pp3c3_31350V3.1.p pacid=32940924 transcript=Pp3c3_31350V3.1 locus=Pp3c3_31350 annot-version=v3.3   Physcomitrella_patens OG0000053
Pp3c3_31330V3.1.p pacid=32942788 transcript=Pp3c3_31330V3.1 locus=Pp3c3_31330 annot-version=v3.3   Physcomitrella_patens OG0000053
Pp3c3_23940V3.1.p pacid=32943489 transcript=Pp3c3_23940V3.1 locus=Pp3c3_23940 annot-version=v3.3   Physcomitrella_patens OG0000053
Pp3c3_31380V3.1.p pacid=32944095 transcript=Pp3c3_31380V3.1 locus=Pp3c3_31380 annot-version=v3.3   Physcomitrella_patens OG0000053
Pp3c5_22750V3.1.p pacid=32953096 transcript=Pp3c5_22750V3.1 locus=Pp3c5_22750 annot-version=v3.3   Physcomitrella_patens OG0000053
Pp3c14_18960V3.1.p pacid=32960297 transcript=Pp3c14_18960V3.1 locus=Pp3c14_18960 annot-version=v3.3   Physcomitrella_patens OG0000053
Pp3c12_24350V3.1.p pacid=32975041 transcript=Pp3c12_24350V3.1 locus=Pp3c12_24350 annot-version=v3.3   Physcomitrella_patens OG0000053
Pp3c17_12760V3.1.p pacid=32907146 transcript=Pp3c17_12760V3.1 locus=Pp3c17_12760 annot-version=v3.3   Physcomitrella_patens OG0000053
Pp3c6_6900V3.1.p pacid=32976010 transcript=Pp3c6_6900V3.1 locus=Pp3c6_6900 annot-version=v3.3   Physcomitrella_patens OG0000053
Pp3c25_8610V3.1.p pacid=32979874 transcript=Pp3c25_8610V3.1 locus=Pp3c25_8610 annot-version=v3.3   Physcomitrella_patens OG0000053
Pp3c25_8630V3.1.p pacid=32980810 transcript=Pp3c25_8630V3.1 locus=Pp3c25_8630 annot-version=v3.3   Physcomitrella_patens OG0000053
Pp3c16_7490V3.1.p pacid=32983653 transcript=Pp3c16_7490V3.1 locus=Pp3c16_7490 annot-version=v3.3   Physcomitrella_patens OG0000053
Pp3c16_7480V3.1.p pacid=32984029 transcript=Pp3c16_7480V3.1 locus=Pp3c16_7480 annot-version=v3.3   Physcomitrella_patens

OG0000053
Pp3c16_7540V3.1.p pacid=32985522 transcript=Pp3c16_7540V3.1
locus=Pp3c16_7540 annot-version=v3.3   Physcomitrella_patens
OG0000053
Pp3c4_6000V3.1.p pacid=32919817 transcript=Pp3c4_6000V3.1
locus=Pp3c4_6000 annot-version=v3.3   Physcomitrella_patens
OG0000053
AT2G42200.1 | squamosa promoter binding protein-like 9 |
Chr2:17587601-17589451 FORWARD LENGTH=375 | 201606
Arabidopsis_thaliana   OG0000053
AT1G02065.1 | squamosa promoter binding protein-like 8 |
Chr1:365625-367149 FORWARD LENGTH=333 | 201606
Arabidopsis_thaliana   OG0000053
AT2G47070.1 | squamosa promoter binding protein-like 1 |
Chr2:19337144-19340552 FORWARD LENGTH=881 | 201606
Arabidopsis_thaliana   OG0000053
AT3G15270.1 | squamosa promoter binding protein-like 5 |
Chr3:5140624-5141256 REVERSE LENGTH=181 | 201606
Arabidopsis_thaliana   OG0000053
AT3G57920.1 | squamosa promoter binding protein-like 15 |
Chr3:21444450-21445852 REVERSE LENGTH=354 | 201606
Arabidopsis_thaliana   OG0000053
AT3G60030.1 | squamosa promoter-binding protein-like 12 |
Chr3:22165856-22169410 REVERSE LENGTH=927 | 201606
Arabidopsis_thaliana   OG0000053
AT1G20980.1 | squamosa promoter binding protein-like 14 |
Chr1:7325042-7328933 FORWARD LENGTH=1035 | 201606
Arabidopsis_thaliana   OG0000053
AT5G18830.1 | squamosa promoter binding protein-like 7 |
Chr5:6276116-6280227 FORWARD LENGTH=801 | 201606
Arabidopsis_thaliana   OG0000053
AT5G43270.1 | squamosa promoter binding protein-like 2 |
Chr5:17360527-17362143 REVERSE LENGTH=419 | 201606
Arabidopsis_thaliana   OG0000053
AT5G50570.1 | Squamosa promoter-binding protein-like (SBP domain)
transcription factor family protein | Chr5:20582555-20583791 REVERSE
LENGTH=359 | 201606   Arabidopsis_thaliana   OG0000053
AT5G50670.1 | Squamosa promoter-binding protein-like (SBP domain)
transcription factor family protein | Chr5:20615897-20617133 REVERSE
LENGTH=359 | 201606   Arabidopsis_thaliana   OG0000053
AT1G27360.1 | squamosa promoter-like 11 | Chr1:9502139-9503715
FORWARD LENGTH=393 | 201606   Arabidopsis_thaliana
OG0000053
AT1G27370.1 | squamosa promoter binding protein-like 10 |
Chr1:9505454-9506997 REVERSE LENGTH=396 | 201606
Arabidopsis_thaliana   OG0000053
AT1G53160.1 | squamosa promoter binding protein-like 4 |
Chr1:19806478-19807080 FORWARD LENGTH=174 | 201606
Arabidopsis_thaliana   OG0000053
AT1G69170.1 | Squamosa promoter-binding protein-like (SBP domain)
transcription factor family protein | Chr1:26005626-26007041 FORWARD
LENGTH=405 | 201606   Arabidopsis_thaliana   OG0000053
AT1G76580.1 | Squamosa promoter-binding protein-like (SBP domain)
transcription factor family protein | Chr1:28734600-28738451 FORWARD

LENGTH=1020 | 201606 Arabidopsis_thaliana OG0000053
AT2G33810.1 | squamosa promoter binding protein-like 3 | Chr2:14305342-14305828 FORWARD LENGTH=131 | 201606 Arabidopsis_thaliana OG0000053
Os03t0833300-01 Similar to Squamosa promoter-binding-like protein 6. Oryza_sativa OG0000053
Os01t0292900-01 Similar to Squamosa-promoter binding-like protein 2. Oryza_sativa OG0000053
Os04t0551500-01 SBP domain containing protein. Oryza_sativa OG0000053
Os04t0656500-01 Similar to Squamosa-promoter binding-like protein 3. Oryza_sativa OG0000053
Os05t0408200-01 SBP domain containing protein. Oryza_sativa OG0000053
Os06t0659100-00 Similar to Squamosa-promoter binding protein 1. Oryza_sativa OG0000053
Os06t0663500-00 SBP domain containing protein. Oryza_sativa OG0000053
Os06t0703500-01 Similar to SBP-domain protein 5 (Fragment). Oryza_sativa OG0000053
Os08t0509600-01 Squamosa promter-binding-like transcription activator, Regulation of branching in panicles and vegetative shoots, Semidominant regulator of plant architecture Oryza_sativa OG0000053
Os08t0513700-01 Similar to Squamosa promoter-binding-like protein 15. Oryza_sativa OG0000053
Os08t0531600-01 Transcription factor, Positive regulator of cell proliferation, Control of grain size, shape and quality Oryza_sativa OG0000053
Os09t0491532-01 Similar to Squamosa-promoter binding protein 2. Oryza_sativa OG0000053
Os09t0507100-00 Similar to Squamosa-promoter binding-like protein 11. Oryza_sativa OG0000053
Os11t0496600-00 Hypothetical conserved gene. Oryza_sativa OG0000053
Os01t0922600-01 Similar to SBP-domain protein 4. Oryza_sativa OG0000053
Os02t0139400-01 Similar to Squamosa promoter-binding-like protein 3. Oryza_sativa OG0000053
Os02t0174100-01 Similar to Isoform 2 of Squamosa promoter-binding-like protein 4. Oryza_sativa OG0000053
Os02t0177300-00 Squamosa-promoter binding protein 1. Oryza_sativa OG0000053
Os02t0445200-00 Similar to SLP16. Oryza_sativa OG0000053
g5024.t1 Caulerpa_lentillifera OG0000054
31957_0300010511 Ostreococcus_tauri OG0000054
7153 pacid=27413200 transcript=7153 locus=gwEuk.3.545.1 ID=7153.2.0.231 annot-version=v2.0 Ostreococcus_lucimarinus OG0000054
kfl00687_0090_v1.1 Klebsormidium_flaccidum OG0000054
kfl00026_0560_v1.1 Klebsormidium_flaccidum OG0000054
kfl00043_0080_v1.1 Klebsormidium_flaccidum OG0000054
kfl00006_0850_v1.1 Klebsormidium_flaccidum OG0000054
kfl00153_0220_v1.1 Klebsormidium_flaccidum OG0000054

kfl00218_0110_v1.1	Klebsormidium_flaccidum	OG0000054
kfl00222_0050_v1.1	Klebsormidium_flaccidum	OG0000054
Pp3c13_12640V3.1.p pacid=32932544 transcript=Pp3c13_12640V3.1 locus=Pp3c13_12640 annot-version=v3.3	Physcomitrella_patens	OG0000054
Pp3c2_13250V3.1.p pacid=32933525 transcript=Pp3c2_13250V3.1 locus=Pp3c2_13250 annot-version=v3.3	Physcomitrella_patens	OG0000054
Pp3c22_21630V3.1.p pacid=32903999 transcript=Pp3c22_21630V3.1 locus=Pp3c22_21630 annot-version=v3.3	Physcomitrella_patens	OG0000054
Pp3c3_15470V3.1.p pacid=32944562 transcript=Pp3c3_15470V3.1 locus=Pp3c3_15470 annot-version=v3.3	Physcomitrella_patens	OG0000054
Pp3c5_5360V3.1.p pacid=32955226 transcript=Pp3c5_5360V3.1 locus=Pp3c5_5360 annot-version=v3.3	Physcomitrella_patens	OG0000054
Pp3c11_19970V3.1.p pacid=32957170 transcript=Pp3c11_19970V3.1 locus=Pp3c11_19970 annot-version=v3.3	Physcomitrella_patens	OG0000054
Pp3c11_18480V3.1.p pacid=32958794 transcript=Pp3c11_18480V3.1 locus=Pp3c11_18480 annot-version=v3.3	Physcomitrella_patens	OG0000054
Pp3c11_19400V3.1.p pacid=32959235 transcript=Pp3c11_19400V3.1 locus=Pp3c11_19400 annot-version=v3.3	Physcomitrella_patens	OG0000054
Pp3c14_1310V3.1.p pacid=32960777 transcript=Pp3c14_1310V3.1 locus=Pp3c14_1310 annot-version=v3.3	Physcomitrella_patens	OG0000054
Pp3c14_4360V3.1.p pacid=32962541 transcript=Pp3c14_4360V3.1 locus=Pp3c14_4360 annot-version=v3.3	Physcomitrella_patens	OG0000054
Pp3c1_20910V3.1.p pacid=32969040 transcript=Pp3c1_20910V3.1 locus=Pp3c1_20910 annot-version=v3.3	Physcomitrella_patens	OG0000054
Pp3c1_22300V3.1.p pacid=32971851 transcript=Pp3c1_22300V3.1 locus=Pp3c1_22300 annot-version=v3.3	Physcomitrella_patens	OG0000054
Pp3c18_4130V3.1.p pacid=32981831 transcript=Pp3c18_4130V3.1 locus=Pp3c18_4130 annot-version=v3.3	Physcomitrella_patens	OG0000054
Pp3c9_7690V3.1.p pacid=32912636 transcript=Pp3c9_7690V3.1 locus=Pp3c9_7690 annot-version=v3.3	Physcomitrella_patens	OG0000054
Pp3c10_1490V3.1.p pacid=32901088 transcript=Pp3c10_1490V3.1 locus=Pp3c10_1490 annot-version=v3.3	Physcomitrella_patens	OG0000054
Pp3c10_4990V3.1.p pacid=32901299 transcript=Pp3c10_4990V3.1 locus=Pp3c10_4990 annot-version=v3.3	Physcomitrella_patens	OG0000054
Pp3c26_7990V3.1.p pacid=32918385 transcript=Pp3c26_7990V3.1 locus=Pp3c26_7990 annot-version=v3.3	Physcomitrella_patens	OG0000054
Pp3c7_3160V3.1.p pacid=32923261 transcript=Pp3c7_3160V3.1

locus=Pp3c7_3160 annot-version=v3.3	Physcomitrella_patens
OG0000054
Pp3c7_3040V3.1.p pacid=32923810 transcript=Pp3c7_3040V3.1
locus=Pp3c7_3040 annot-version=v3.3	Physcomitrella_patens
OG0000054
AT2G44030.1 | Galactose oxidase/kelch repeat superfamily protein |
Chr2:18220086-18221228 FORWARD LENGTH=380 | 201606
Arabidopsis_thaliana	OG0000054
AT2G44130.1 | Galactose oxidase/kelch repeat superfamily protein |
Chr2:18253731-18254960 FORWARD LENGTH=409 | 201606
Arabidopsis_thaliana	OG0000054
AT2G44630.1 | Galactose oxidase/kelch repeat superfamily protein |
Chr2:18415829-18416947 REVERSE LENGTH=372 | 201606
Arabidopsis_thaliana	OG0000054
AT2G44700.1 | Galactose oxidase/kelch repeat superfamily protein |
Chr2:18431015-18432121 FORWARD LENGTH=368 | 201606
Arabidopsis_thaliana	OG0000054
AT2G45406.1 | Galactose oxidase/kelch repeat superfamily protein |
Chr2:18710961-18711338 REVERSE LENGTH=125 | 201606
Arabidopsis_thaliana	OG0000054
AT3G06570.1 | Galactose oxidase/kelch repeat superfamily protein |
Chr3:2047369-2048541 REVERSE LENGTH=390 | 201606
Arabidopsis_thaliana	OG0000054
AT3G08810.1 | Galactose oxidase/kelch repeat superfamily protein |
Chr3:2675631-2676662 REVERSE LENGTH=343 | 201606
Arabidopsis_thaliana	OG0000054
AT3G10510.1 | Galactose oxidase/kelch repeat superfamily protein |
Chr3:3274220-3275551 REVERSE LENGTH=371 | 201606
Arabidopsis_thaliana	OG0000054
AT3G17270.1 | F-box/associated interaction domain protein |
Chr3:5902479-5902886 REVERSE LENGTH=135 | 201606
Arabidopsis_thaliana	OG0000054
AT3G24610.1 | Galactose oxidase/kelch repeat superfamily protein |
Chr3:8975366-8976575 REVERSE LENGTH=375 | 201606
Arabidopsis_thaliana	OG0000054
AT3G27150.1 | Galactose oxidase/kelch repeat superfamily protein |
Chr3:10009692-10010960 REVERSE LENGTH=422 | 201606
Arabidopsis_thaliana	OG0000054
AT1G14330.1 | Galactose oxidase/kelch repeat superfamily protein |
Chr1:4890375-4891700 REVERSE LENGTH=441 | 201606
Arabidopsis_thaliana	OG0000054
AT3G27910.1 | Galactose oxidase/kelch repeat superfamily protein |
Chr3:10357232-10358116 REVERSE LENGTH=294 | 201606
Arabidopsis_thaliana	OG0000054
AT3G43710.1 | Galactose oxidase/kelch repeat superfamily protein |
Chr3:15605608-15606744 FORWARD LENGTH=378 | 201606
Arabidopsis_thaliana	OG0000054
AT3G46050.1 | Galactose oxidase/kelch repeat superfamily protein |
Chr3:16916072-16917184 FORWARD LENGTH=370 | 201606
Arabidopsis_thaliana	OG0000054
AT1G15670.1 | Galactose oxidase/kelch repeat superfamily protein |
Chr1:5390119-5391198 FORWARD LENGTH=359 | 201606
Arabidopsis_thaliana	OG0000054
AT1G16250.1 | Galactose oxidase/kelch repeat superfamily protein |

Chr1:5557214-5558697 FORWARD LENGTH=383 | 201606
Arabidopsis_thaliana   OG0000054
AT3G59940.1 | Galactose oxidase/kelch repeat superfamily protein |
Chr3:22143009-22144265 FORWARD LENGTH=418 | 201606
Arabidopsis_thaliana   OG0000054
AT3G61350.1 | SKP1 interacting partner 4 | Chr3:22703203-22704374
FORWARD LENGTH=358 | 201606   Arabidopsis_thaliana
OG0000054
AT3G63220.1 | Galactose oxidase/kelch repeat superfamily protein |
Chr3:23357540-23358577 REVERSE LENGTH=345 | 201606
Arabidopsis_thaliana   OG0000054
AT4G02310.1 | Galactose oxidase/kelch repeat superfamily protein |
Chr4:1014680-1015729 REVERSE LENGTH=349 | 201606
Arabidopsis_thaliana   OG0000054
AT4G05620.1 | Galactose oxidase/kelch repeat superfamily protein |
Chr4:2985051-2985449 REVERSE LENGTH=132 | 201606
Arabidopsis_thaliana   OG0000054
AT4G11745.1 | Galactose oxidase/kelch repeat superfamily protein |
Chr4:7075826-7076680 REVERSE LENGTH=284 | 201606
Arabidopsis_thaliana   OG0000054
AT4G11750.1 | Galactose oxidase/kelch repeat superfamily protein |
Chr4:7077390-7078550 REVERSE LENGTH=386 | 201606
Arabidopsis_thaliana   OG0000054
AT4G11770.1 | Galactose oxidase/kelch repeat superfamily protein |
Chr4:7082279-7083469 REVERSE LENGTH=396 | 201606
Arabidopsis_thaliana   OG0000054
AT4G14905.1 | Galactose oxidase/kelch repeat superfamily protein |
Chr4:8526879-8527997 REVERSE LENGTH=372 | 201606
Arabidopsis_thaliana   OG0000054
AT4G19250.1 | Galactose oxidase/kelch repeat superfamily protein |
Chr4:10531504-10532190 FORWARD LENGTH=228 | 201606
Arabidopsis_thaliana   OG0000054
AT4G19330.1 | kelch repeat F-box protein | Chr4:10557354-10558505
FORWARD LENGTH=383 | 201606   Arabidopsis_thaliana
OG0000054
AT4G19865.1 | Galactose oxidase/kelch repeat superfamily protein |
Chr4:10781184-10782365 REVERSE LENGTH=393 | 201606
Arabidopsis_thaliana   OG0000054
AT4G19870.1 | Galactose oxidase/kelch repeat superfamily protein |
Chr4:10783054-10784256 REVERSE LENGTH=400 | 201606
Arabidopsis_thaliana   OG0000054
AT4G23580.1 | Galactose oxidase/kelch repeat superfamily protein |
Chr4:12304246-12305397 REVERSE LENGTH=383 | 201606
Arabidopsis_thaliana   OG0000054
AT4G25710.1 | Galactose oxidase/kelch repeat superfamily protein |
Chr4:13098359-13099531 REVERSE LENGTH=390 | 201606
Arabidopsis_thaliana   OG0000054
AT1G19460.1 | Galactose oxidase/kelch repeat superfamily protein |
Chr1:6738484-6739734 FORWARD LENGTH=416 | 201606
Arabidopsis_thaliana   OG0000054
AT1G19470.1 | Galactose oxidase/kelch repeat superfamily protein |
Chr1:6741296-6742534 FORWARD LENGTH=412 | 201606
Arabidopsis_thaliana   OG0000054
AT4G29370.1 | Galactose oxidase/kelch repeat superfamily protein |

Chr4:14455602-14456738 FORWARD LENGTH=378 | 201606
Arabidopsis_thaliana OG0000054
AT1G19930.1 | Galactose oxidase/kelch repeat superfamily protein |
Chr1:6917020-6918138 FORWARD LENGTH=344 | 201606
Arabidopsis_thaliana OG0000054
AT4G33900.1 | Galactose oxidase/kelch repeat superfamily protein |
Chr4:16251572-16252711 REVERSE LENGTH=379 | 201606
Arabidopsis_thaliana OG0000054
AT4G34170.1 | Galactose oxidase/kelch repeat superfamily protein |
Chr4:16368087-16369025 FORWARD LENGTH=293 | 201606
Arabidopsis_thaliana OG0000054
AT4G35120.1 | Galactose oxidase/kelch repeat superfamily protein |
Chr4:16716806-16718017 FORWARD LENGTH=389 | 201606
Arabidopsis_thaliana OG0000054
AT4G38940.1 | Galactose oxidase/kelch repeat superfamily protein |
Chr4:18152848-18153960 FORWARD LENGTH=370 | 201606
Arabidopsis_thaliana OG0000054
AT4G39240.1 | Galactose oxidase/kelch repeat superfamily protein |
Chr4:18269599-18270726 REVERSE LENGTH=375 | 201606
Arabidopsis_thaliana OG0000054
AT4G39290.1 | Galactose oxidase/kelch repeat superfamily protein |
Chr4:18285205-18286302 REVERSE LENGTH=365 | 201606
Arabidopsis_thaliana OG0000054
AT4G39550.1 | Galactose oxidase/kelch repeat superfamily protein |
Chr4:18380681-18381859 REVERSE LENGTH=392 | 201606
Arabidopsis_thaliana OG0000054
AT4G39560.1 | Galactose oxidase/kelch repeat superfamily protein |
Chr4:18382940-18383740 REVERSE LENGTH=266 | 201606
Arabidopsis_thaliana OG0000054
AT4G39570.1 | Galactose oxidase/kelch repeat superfamily protein |
Chr4:18384254-18385441 REVERSE LENGTH=395 | 201606
Arabidopsis_thaliana OG0000054
AT4G39580.1 | Galactose oxidase/kelch repeat superfamily protein |
Chr4:18385684-18386811 REVERSE LENGTH=375 | 201606
Arabidopsis_thaliana OG0000054
AT4G39590.1 | Galactose oxidase/kelch repeat superfamily protein |
Chr4:18387515-18388723 REVERSE LENGTH=402 | 201606
Arabidopsis_thaliana OG0000054
AT4G39600.1 | Galactose oxidase/kelch repeat superfamily protein |
Chr4:18389436-18390539 REVERSE LENGTH=367 | 201606
Arabidopsis_thaliana OG0000054
AT4G39753.1 | Galactose oxidase/kelch repeat superfamily protein |
Chr4:18440125-18441297 FORWARD LENGTH=390 | 201606
Arabidopsis_thaliana OG0000054
AT4G39756.1 | Galactose oxidase/kelch repeat superfamily protein |
Chr4:18441756-18442880 FORWARD LENGTH=374 | 201606
Arabidopsis_thaliana OG0000054
AT4G39760.1 | Galactose oxidase/kelch repeat superfamily protein |
Chr4:18443679-18444788 FORWARD LENGTH=369 | 201606
Arabidopsis_thaliana OG0000054
AT5G02980.1 | Galactose oxidase/kelch repeat superfamily protein |
Chr5:697993-699000 FORWARD LENGTH=335 | 201606
Arabidopsis_thaliana OG0000054
AT5G02990.1 | galactose oxidase/kelch repeat protein |

Chr5:700169-700648 FORWARD LENGTH=159 | 201606
Arabidopsis_thaliana OG0000054
AT5G02995.1 | galactose oxidase/kelch repeat protein |
Chr5:701689-702630 FORWARD LENGTH=313 | 201606
Arabidopsis_thaliana OG0000054
AT5G03000.1 | Galactose oxidase/kelch repeat superfamily protein |
Chr5:703268-704437 FORWARD LENGTH=354 | 201606
Arabidopsis_thaliana OG0000054
AT5G03020.1 | Galactose oxidase/kelch repeat superfamily protein |
Chr5:707263-708306 FORWARD LENGTH=347 | 201606
Arabidopsis_thaliana OG0000054
AT1G22040.1 | Galactose oxidase/kelch repeat superfamily protein |
Chr1:7768370-7769797 FORWARD LENGTH=475 | 201606
Arabidopsis_thaliana OG0000054
AT5G28160.1 | Galactose oxidase/kelch repeat superfamily protein |
Chr5:10137926-10139013 FORWARD LENGTH=324 | 201606
Arabidopsis_thaliana OG0000054
AT5G28180.1 | Galactose oxidase/kelch repeat superfamily protein |
Chr5:10162783-10163943 FORWARD LENGTH=352 | 201606
Arabidopsis_thaliana OG0000054
AT5G38670.1 | Galactose oxidase/kelch repeat superfamily protein |
Chr5:15475786-15476871 FORWARD LENGTH=291 | 201606
Arabidopsis_thaliana OG0000054
AT5G38680.1 | Galactose oxidase/kelch repeat superfamily protein |
Chr5:15477324-15479040 FORWARD LENGTH=357 | 201606
Arabidopsis_thaliana OG0000054
AT5G39560.1 | Galactose oxidase/kelch repeat superfamily protein |
Chr5:15841885-15843096 REVERSE LENGTH=403 | 201606
Arabidopsis_thaliana OG0000054
AT5G40680.1 | Galactose oxidase/kelch repeat superfamily protein |
Chr5:16288754-16290001 FORWARD LENGTH=415 | 201606
Arabidopsis_thaliana OG0000054
AT5G48980.1 | Galactose oxidase/kelch repeat superfamily protein |
Chr5:19860724-19861923 FORWARD LENGTH=399 | 201606
Arabidopsis_thaliana OG0000054
AT5G48990.1 | Galactose oxidase/kelch repeat superfamily protein |
Chr5:19862340-19863458 FORWARD LENGTH=372 | 201606
Arabidopsis_thaliana OG0000054
AT5G49000.1 | Galactose oxidase/kelch repeat superfamily protein |
Chr5:19864527-19865645 FORWARD LENGTH=328 | 201606
Arabidopsis_thaliana OG0000054
AT5G51250.1 | Galactose oxidase/kelch repeat superfamily protein |
Chr5:20830743-20831849 FORWARD LENGTH=368 | 201606
Arabidopsis_thaliana OG0000054
AT5G60570.1 | Galactose oxidase/kelch repeat superfamily protein |
Chr5:24348713-24349894 FORWARD LENGTH=393 | 201606
Arabidopsis_thaliana OG0000054
AT1G26930.1 | Galactose oxidase/kelch repeat superfamily protein |
Chr1:9336211-9337476 REVERSE LENGTH=421 | 201606
Arabidopsis_thaliana OG0000054
AT1G27420.1 | Galactose oxidase/kelch repeat superfamily protein |
Chr1:9519108-9520520 FORWARD LENGTH=346 | 201606
Arabidopsis_thaliana OG0000054
AT1G30090.1 | Galactose oxidase/kelch repeat superfamily protein |

Chr1:10559746-10560942 REVERSE LENGTH=398 | 201606
Arabidopsis_thaliana   OG0000054
AT1G60570.1 | Galactose oxidase/kelch repeat superfamily protein |
Chr1:22311836-22312981 FORWARD LENGTH=381 | 201606
Arabidopsis_thaliana   OG0000054
AT1G61540.1 | Galactose oxidase/kelch repeat superfamily protein |
Chr1:22702498-22703706 FORWARD LENGTH=402 | 201606
Arabidopsis_thaliana   OG0000054
AT1G67480.1 | Galactose oxidase/kelch repeat superfamily protein |
Chr1:25277294-25278529 FORWARD LENGTH=376 | 201606
Arabidopsis_thaliana   OG0000054
AT1G74510.1 | Galactose oxidase/kelch repeat superfamily protein |
Chr1:28006065-28007420 FORWARD LENGTH=451 | 201606
Arabidopsis_thaliana   OG0000054
AT1G77880.1 | Galactose oxidase/kelch repeat superfamily protein |
Chr1:29286675-29287067 REVERSE LENGTH=130 | 201606
Arabidopsis_thaliana   OG0000054
AT1G80440.1 | Galactose oxidase/kelch repeat superfamily protein |
Chr1:30241763-30242827 FORWARD LENGTH=354 | 201606
Arabidopsis_thaliana   OG0000054
AT2G02870.1 | Galactose oxidase/kelch repeat superfamily protein |
Chr2:838378-839781 FORWARD LENGTH=467 | 201606
Arabidopsis_thaliana   OG0000054
AT2G03460.1 | Galactose oxidase/kelch repeat superfamily protein |
Chr2:1043729-1044594 REVERSE LENGTH=257 | 201606
Arabidopsis_thaliana   OG0000054
AT2G20380.1 | Galactose oxidase/kelch repeat superfamily protein |
Chr2:8794818-8795980 REVERSE LENGTH=348 | 201606
Arabidopsis_thaliana   OG0000054
AT2G21680.1 | Galactose oxidase/kelch repeat superfamily protein |
Chr2:9267730-9269019 FORWARD LENGTH=429 | 201606
Arabidopsis_thaliana   OG0000054
AT2G21950.1 | SKP1 interacting partner 6 | Chr2:9353526-9354644
REVERSE LENGTH=372 | 201606   Arabidopsis_thaliana
OG0000054
AT2G22030.1 | Galactose oxidase/kelch repeat superfamily protein |
Chr2:9372530-9373681 FORWARD LENGTH=383 | 201606
Arabidopsis_thaliana   OG0000054
AT2G22050.1 | Galactose oxidase/kelch repeat superfamily protein |
Chr2:9376684-9377552 FORWARD LENGTH=259 | 201606
Arabidopsis_thaliana   OG0000054
AT2G24540.1 | Galactose oxidase/kelch repeat superfamily protein |
Chr2:10426414-10427532 FORWARD LENGTH=372 | 201606
Arabidopsis_thaliana   OG0000054
AT2G29600.1 | Galactose oxidase/kelch repeat superfamily protein |
Chr2:12655661-12656908 FORWARD LENGTH=415 | 201606
Arabidopsis_thaliana   OG0000054
AT2G29770.1 | Galactose oxidase/kelch repeat superfamily protein |
Chr2:12715710-12716873 REVERSE LENGTH=387 | 201606
Arabidopsis_thaliana   OG0000054
AT2G29780.1 | Galactose oxidase/kelch repeat superfamily protein |
Chr2:12718132-12719328 REVERSE LENGTH=398 | 201606
Arabidopsis_thaliana   OG0000054
AT2G29800.1 | Galactose oxidase/kelch repeat superfamily protein |

Chr2:12723358-12724602 REVERSE LENGTH=414 | 201606
Arabidopsis_thaliana   OG0000054
AT2G29810.1 | Galactose oxidase/kelch repeat superfamily protein |
Chr2:12726102-12727253 REVERSE LENGTH=383 | 201606
Arabidopsis_thaliana   OG0000054
AT2G29820.1 | Galactose oxidase/kelch repeat superfamily protein |
Chr2:12728362-12729528 REVERSE LENGTH=388 | 201606
Arabidopsis_thaliana   OG0000054
AT2G29830.1 | Galactose oxidase/kelch repeat superfamily protein |
Chr2:12730411-12731562 REVERSE LENGTH=383 | 201606
Arabidopsis_thaliana   OG0000054
AT2G29860.1 | Galactose oxidase/kelch repeat superfamily protein |
Chr2:12737666-12738388 REVERSE LENGTH=240 | 201606
Arabidopsis_thaliana   OG0000054
Os04t0380300-01 Kelch-type beta propeller domain containing protein.
        Oryza_sativa   OG0000054
Os04t0619300-01 Similar to H0114G12.4 protein.   Oryza_sativa
OG0000054
Os06t0594400-02 Kelch-type beta propeller domain containing protein.
        Oryza_sativa   OG0000054
Os06t0655500-01 Kelch-type beta propeller domain containing protein.
        Oryza_sativa   OG0000054
Os07t0153400-01 Kelch-type beta propeller domain containing protein.
        Oryza_sativa   OG0000054
Os07t0673700-01 Kelch-type beta propeller domain containing protein.
        Oryza_sativa   OG0000054
Os10t0363600-01 Kelch-type beta propeller domain containing protein.
        Oryza_sativa   OG0000054
Os10t0388200-01 Kelch-type beta propeller domain containing protein.
        Oryza_sativa   OG0000054
Os11t0246200-00 Kelch-type beta propeller domain containing protein.
        Oryza_sativa   OG0000054
Os11t0602800-01 Galactose oxidase/kelch, beta-propeller domain
containing protein.   Oryza_sativa   OG0000054
Os02t0114600-01 Kelch-type beta propeller domain containing protein.
        Oryza_sativa   OG0000054
Os02t0202900-00 Similar to ring canal kelch.   Oryza_sativa
OG0000054
Os02t0208700-00 Similar to Protein kinase Kelch repeat:Kelch.
Oryza_sativa   OG0000054
Os02t0504900-01 Kelch-type beta propeller domain containing protein.
        Oryza_sativa   OG0000054
Os02t0563000-00 Hypothetical conserved gene.   Oryza_sativa
OG0000054
Os02t0574900-01 Kelch-type beta propeller domain containing protein.
        Oryza_sativa   OG0000054
Os02t0748300-01 Similar to VMP3 protein. Oryza_sativa
OG0000054
Os03t0171600-01 F-box protein containing a Kelch repeat motif,
Reguration of leaf senescence, seed size and grain number
Oryza_sativa   OG0000054
evm.model.contig_2038.4 Porphyridium_purpureum   OG0000054
CDF37341 pep supercontig:ASM35022v2:HG001832:46810:48024:1
gene:CHC_T00005485001 transcript:CDF37341

gene_biotype:protein_coding transcript_biotype:protein_coding
Chondrus_crispus OG0000054
CDF34227 pep supercontig:ASM35022v2:HG001679:15491:15646:1
gene:CHC_T00002928001 transcript:CDF34227
gene_biotype:protein_coding transcript_biotype:protein_coding
Chondrus_crispus OG0000054
g1161.t1 Caulerpa_lentillifera OG0000055
g3080.t1 Caulerpa_lentillifera OG0000055
g345.t1 Caulerpa_lentillifera OG0000055
g537.t1 Caulerpa_lentillifera OG0000055
g5380.t1 Caulerpa_lentillifera OG0000055
g5410.t1 Caulerpa_lentillifera OG0000055
g553.t1 Caulerpa_lentillifera OG0000055
g5931.t1 Caulerpa_lentillifera OG0000055
g6577.t1 Caulerpa_lentillifera OG0000055
g7142.t1 Caulerpa_lentillifera OG0000055
g7384.t1 Caulerpa_lentillifera OG0000055
g7938.t1 Caulerpa_lentillifera OG0000055
g8899.t1 Caulerpa_lentillifera OG0000055
g9071.t1 Caulerpa_lentillifera OG0000055
g9225.t1 Caulerpa_lentillifera OG0000055
Cre03.g206350.t1.1 pacid=30787976 transcript=Cre03.g206350.t1.1
locus=Cre03.g206350 ID=Cre03.g206350.t1.1.v5.5 annot-version=v5.5
Chlamydomonas_reinhardtii OG0000055
Cre08.g358350.t1.1 pacid=30773500 transcript=Cre08.g358350.t1.1
locus=Cre08.g358350 ID=Cre08.g358350.t1.1.v5.5 annot-version=v5.5
Chlamydomonas_reinhardtii OG0000055
Cre07.g330800.t1.2 pacid=30774867 transcript=Cre07.g330800.t1.2
locus=Cre07.g330800 ID=Cre07.g330800.t1.2.v5.5 annot-version=v5.5
Chlamydomonas_reinhardtii OG0000055
Cre10.g459350.t1.1 pacid=30789913 transcript=Cre10.g459350.t1.1
locus=Cre10.g459350 ID=Cre10.g459350.t1.1.v5.5 annot-version=v5.5
Chlamydomonas_reinhardtii OG0000055
Cre04.g223150.t1.1 pacid=30791111 transcript=Cre04.g223150.t1.1
locus=Cre04.g223150 ID=Cre04.g223150.t1.1.v5.5 annot-version=v5.5
Chlamydomonas_reinhardtii OG0000055
Cre12.g541700.t1.2 pacid=30793580 transcript=Cre12.g541700.t1.2
locus=Cre12.g541700 ID=Cre12.g541700.t1.2.v5.5 annot-version=v5.5
Chlamydomonas_reinhardtii OG0000055
Cre11.g467649.t1.1 pacid=30775976 transcript=Cre11.g467649.t1.1
locus=Cre11.g467649 ID=Cre11.g467649.t1.1.v5.5 annot-version=v5.5
Chlamydomonas_reinhardtii OG0000055
Cre14.g633150.t1.1 pacid=30776122 transcript=Cre14.g633150.t1.1
locus=Cre14.g633150 ID=Cre14.g633150.t1.1.v5.5 annot-version=v5.5
Chlamydomonas_reinhardtii OG0000055
Cre14.g626800.t1.1 pacid=30776720 transcript=Cre14.g626800.t1.1
locus=Cre14.g626800 ID=Cre14.g626800.t1.1.v5.5 annot-version=v5.5
Chlamydomonas_reinhardtii OG0000055
Cre14.g614550.t1.1 pacid=30776747 transcript=Cre14.g614550.t1.1
locus=Cre14.g614550 ID=Cre14.g614550.t1.1.v5.5 annot-version=v5.5
Chlamydomonas_reinhardtii OG0000055
Cre16.g661588.t1.1 pacid=30776796 transcript=Cre16.g661588.t1.1
locus=Cre16.g661588 ID=Cre16.g661588.t1.1.v5.5 annot-version=v5.5
Chlamydomonas_reinhardtii OG0000055

Cre16.g680850.t1.1 pacid=30777176 transcript=Cre16.g680850.t1.1 locus=Cre16.g680850 ID=Cre16.g680850.t1.1.v5.5 annot-version=v5.5 Chlamydomonas_reinhardtii OG0000055
Cre19.g750747.t1.1 pacid=30778340 transcript=Cre19.g750747.t1.1 locus=Cre19.g750747 ID=Cre19.g750747.t1.1.v5.5 annot-version=v5.5 Chlamydomonas_reinhardtii OG0000055
Cre19.g750497.t1.1 pacid=30778341 transcript=Cre19.g750497.t1.1 locus=Cre19.g750497 ID=Cre19.g750497.t1.1.v5.5 annot-version=v5.5 Chlamydomonas_reinhardtii OG0000055
Cre19.g750697.t1.1 pacid=30778348 transcript=Cre19.g750697.t1.1 locus=Cre19.g750697 ID=Cre19.g750697.t1.1.v5.5 annot-version=v5.5 Chlamydomonas_reinhardtii OG0000055
Cre06.g258051.t1.1 pacid=30779698 transcript=Cre06.g258051.t1.1 locus=Cre06.g258051 ID=Cre06.g258051.t1.1.v5.5 annot-version=v5.5 Chlamydomonas_reinhardtii OG0000055
Cre06.g309500.t1.1 pacid=30779995 transcript=Cre06.g309500.t1.1 locus=Cre06.g309500 ID=Cre06.g309500.t1.1.v5.5 annot-version=v5.5 Chlamydomonas_reinhardtii OG0000055
Cre09.g387500.t1.2 pacid=30780471 transcript=Cre09.g387500.t1.2 locus=Cre09.g387500 ID=Cre09.g387500.t1.2.v5.5 annot-version=v5.5 Chlamydomonas_reinhardtii OG0000055
Cre17.g728864.t1.2 pacid=30782597 transcript=Cre17.g728864.t1.2 locus=Cre17.g728864 ID=Cre17.g728864.t1.2.v5.5 annot-version=v5.5 Chlamydomonas_reinhardtii OG0000055
Cre17.g739800.t1.1 pacid=30782755 transcript=Cre17.g739800.t1.1 locus=Cre17.g739800 ID=Cre17.g739800.t1.1.v5.5 annot-version=v5.5 Chlamydomonas_reinhardtii OG0000055
Cre15.g639000.t1.2 pacid=30783503 transcript=Cre15.g639000.t1.2 locus=Cre15.g639000 ID=Cre15.g639000.t1.2.v5.5 annot-version=v5.5 Chlamydomonas_reinhardtii OG0000055
Cre15.g639802.t1.1 pacid=30783505 transcript=Cre15.g639802.t1.1 locus=Cre15.g639802 ID=Cre15.g639802.t1.1.v5.5 annot-version=v5.5 Chlamydomonas_reinhardtii OG0000055
Cre15.g639304.t1.2 pacid=30783517 transcript=Cre15.g639304.t1.2 locus=Cre15.g639304 ID=Cre15.g639304.t1.2.v5.5 annot-version=v5.5 Chlamydomonas_reinhardtii OG0000055
Cre15.g638304.t1.1 pacid=30783525 transcript=Cre15.g638304.t1.1 locus=Cre15.g638304 ID=Cre15.g638304.t1.1.v5.5 annot-version=v5.5 Chlamydomonas_reinhardtii OG0000055
Cre15.g638300.t1.1 pacid=30783531 transcript=Cre15.g638300.t1.1 locus=Cre15.g638300 ID=Cre15.g638300.t1.1.v5.5 annot-version=v5.5 Chlamydomonas_reinhardtii OG0000055
Cre15.g638956.t1.1 pacid=30783547 transcript=Cre15.g638956.t1.1 locus=Cre15.g638956 ID=Cre15.g638956.t1.1.v5.5 annot-version=v5.5 Chlamydomonas_reinhardtii OG0000055
Cre15.g638150.t1.1 pacid=30783553 transcript=Cre15.g638150.t1.1 locus=Cre15.g638150 ID=Cre15.g638150.t1.1.v5.5 annot-version=v5.5 Chlamydomonas_reinhardtii OG0000055
Cre15.g638750.t1.2 pacid=30783555 transcript=Cre15.g638750.t1.2 locus=Cre15.g638750 ID=Cre15.g638750.t1.2.v5.5 annot-version=v5.5 Chlamydomonas_reinhardtii OG0000055
Cre15.g638450.t1.2 pacid=30783560 transcript=Cre15.g638450.t1.2 locus=Cre15.g638450 ID=Cre15.g638450.t1.2.v5.5 annot-version=v5.5 Chlamydomonas_reinhardtii OG0000055

Cre15.g638950.t1.1 pacid=30783561 transcript=Cre15.g638950.t1.1 locus=Cre15.g638950 ID=Cre15.g638950.t1.1.v5.5 annot-version=v5.5 Chlamydomonas_reinhardtii OG0000055
Cre15.g639700.t1.1 pacid=30783570 transcript=Cre15.g639700.t1.1 locus=Cre15.g639700 ID=Cre15.g639700.t1.1.v5.5 annot-version=v5.5 Chlamydomonas_reinhardtii OG0000055
Cre15.g639650.t1.1 pacid=30783577 transcript=Cre15.g639650.t1.1 locus=Cre15.g639650 ID=Cre15.g639650.t1.1.v5.5 annot-version=v5.5 Chlamydomonas_reinhardtii OG0000055
Cre15.g634800.t1.1 pacid=30783581 transcript=Cre15.g634800.t1.1 locus=Cre15.g634800 ID=Cre15.g634800.t1.1.v5.5 annot-version=v5.5 Chlamydomonas_reinhardtii OG0000055
Cre15.g638100.t1.1 pacid=30783587 transcript=Cre15.g638100.t1.1 locus=Cre15.g638100 ID=Cre15.g638100.t1.1.v5.5 annot-version=v5.5 Chlamydomonas_reinhardtii OG0000055
Cre15.g638700.t1.1 pacid=30783598 transcript=Cre15.g638700.t1.1 locus=Cre15.g638700 ID=Cre15.g638700.t1.1.v5.5 annot-version=v5.5 Chlamydomonas_reinhardtii OG0000055
Cre15.g639800.t1.1 pacid=30783602 transcript=Cre15.g639800.t1.1 locus=Cre15.g639800 ID=Cre15.g639800.t1.1.v5.5 annot-version=v5.5 Chlamydomonas_reinhardtii OG0000055
Cre15.g638651.t1.1 pacid=30783607 transcript=Cre15.g638651.t1.1 locus=Cre15.g638651 ID=Cre15.g638651.t1.1.v5.5 annot-version=v5.5 Chlamydomonas_reinhardtii OG0000055
Cre15.g638401.t1.1 pacid=30783620 transcript=Cre15.g638401.t1.1 locus=Cre15.g638401 ID=Cre15.g638401.t1.1.v5.5 annot-version=v5.5 Chlamydomonas_reinhardtii OG0000055
Cre15.g638000.t1.1 pacid=30783636 transcript=Cre15.g638000.t1.1 locus=Cre15.g638000 ID=Cre15.g638000.t1.1.v5.5 annot-version=v5.5 Chlamydomonas_reinhardtii OG0000055
Cre15.g639614.t1.2 pacid=30783637 transcript=Cre15.g639614.t1.2 locus=Cre15.g639614 ID=Cre15.g639614.t1.2.v5.5 annot-version=v5.5 Chlamydomonas_reinhardtii OG0000055
Cre15.g639308.t1.2 pacid=30783642 transcript=Cre15.g639308.t1.2 locus=Cre15.g639308 ID=Cre15.g639308.t1.2.v5.5 annot-version=v5.5 Chlamydomonas_reinhardtii OG0000055
Cre15.g638050.t1.1 pacid=30783648 transcript=Cre15.g638050.t1.1 locus=Cre15.g638050 ID=Cre15.g638050.t1.1.v5.5 annot-version=v5.5 Chlamydomonas_reinhardtii OG0000055
Cre15.g640350.t1.2 pacid=30783649 transcript=Cre15.g640350.t1.2 locus=Cre15.g640350 ID=Cre15.g640350.t1.2.v5.5 annot-version=v5.5 Chlamydomonas_reinhardtii OG0000055
Cre15.g637850.t1.1 pacid=30783659 transcript=Cre15.g637850.t1.1 locus=Cre15.g637850 ID=Cre15.g637850.t1.1.v5.5 annot-version=v5.5 Chlamydomonas_reinhardtii OG0000055
Cre15.g639750.t1.1 pacid=30783697 transcript=Cre15.g639750.t1.1 locus=Cre15.g639750 ID=Cre15.g639750.t1.1.v5.5 annot-version=v5.5 Chlamydomonas_reinhardtii OG0000055
Cre15.g639850.t1.2 pacid=30783713 transcript=Cre15.g639850.t1.2 locus=Cre15.g639850 ID=Cre15.g639850.t1.2.v5.5 annot-version=v5.5 Chlamydomonas_reinhardtii OG0000055
Cre15.g638650.t1.1 pacid=30783730 transcript=Cre15.g638650.t1.1 locus=Cre15.g638650 ID=Cre15.g638650.t1.1.v5.5 annot-version=v5.5 Chlamydomonas_reinhardtii OG0000055

Cre15.g638550.t1.1 pacid=30783737 transcript=Cre15.g638550.t1.1 locus=Cre15.g638550 ID=Cre15.g638550.t1.1.v5.5 annot-version=v5.5 Chlamydomonas_reinhardtii OG0000055
Cre15.g638954.t1.1 pacid=30783742 transcript=Cre15.g638954.t1.1 locus=Cre15.g638954 ID=Cre15.g638954.t1.1.v5.5 annot-version=v5.5 Chlamydomonas_reinhardtii OG0000055
Cre15.g638303.t1.1 pacid=30783748 transcript=Cre15.g638303.t1.1 locus=Cre15.g638303 ID=Cre15.g638303.t1.1.v5.5 annot-version=v5.5 Chlamydomonas_reinhardtii OG0000055
Cre15.g640400.t1.1 pacid=30783768 transcript=Cre15.g640400.t1.1 locus=Cre15.g640400 ID=Cre15.g640400.t1.1.v5.5 annot-version=v5.5 Chlamydomonas_reinhardtii OG0000055
Cre15.g640450.t1.2 pacid=30783775 transcript=Cre15.g640450.t1.2 locus=Cre15.g640450 ID=Cre15.g640450.t1.2.v5.5 annot-version=v5.5 Chlamydomonas_reinhardtii OG0000055
Cre15.g639300.t1.1 pacid=30783784 transcript=Cre15.g639300.t1.1 locus=Cre15.g639300 ID=Cre15.g639300.t1.1.v5.5 annot-version=v5.5 Chlamydomonas_reinhardtii OG0000055
Cre13.g566400.t1.2 pacid=30783873 transcript=Cre13.g566400.t1.2 locus=Cre13.g566400 ID=Cre13.g566400.t1.2.v5.5 annot-version=v5.5 Chlamydomonas_reinhardtii OG0000055
Cre13.g588201.t1.1 pacid=30784428 transcript=Cre13.g588201.t1.1 locus=Cre13.g588201 ID=Cre13.g588201.t1.1.v5.5 annot-version=v5.5 Chlamydomonas_reinhardtii OG0000055
Vocar.0030s0031.1.p pacid=32894608 transcript=Vocar.0030s0031.1 locus=Vocar.0030s0031 ID=Vocar.0030s0031.1.v2.1 annot-version=v2.1 Volvox_carteri OG0000055
Vocar.0057s0023.1.p pacid=32894735 transcript=Vocar.0057s0023.1 locus=Vocar.0057s0023 ID=Vocar.0057s0023.1.v2.1 annot-version=v2.1 Volvox_carteri OG0000055
Vocar.0011s0235.1.p pacid=32895375 transcript=Vocar.0011s0235.1 locus=Vocar.0011s0235 ID=Vocar.0011s0235.1.v2.1 annot-version=v2.1 Volvox_carteri OG0000055
Vocar.0021s0202.1.p pacid=32895945 transcript=Vocar.0021s0202.1 locus=Vocar.0021s0202 ID=Vocar.0021s0202.1.v2.1 annot-version=v2.1 Volvox_carteri OG0000055
Vocar.0018s0155.1.p pacid=32898611 transcript=Vocar.0018s0155.1 locus=Vocar.0018s0155 ID=Vocar.0018s0155.1.v2.1 annot-version=v2.1 Volvox_carteri OG0000055
Vocar.0015s0104.1.p pacid=32898834 transcript=Vocar.0015s0104.1 locus=Vocar.0015s0104 ID=Vocar.0015s0104.1.v2.1 annot-version=v2.1 Volvox_carteri OG0000055
Vocar.0001s1520.1.p pacid=32885208 transcript=Vocar.0001s1520.1 locus=Vocar.0001s1520 ID=Vocar.0001s1520.1.v2.1 annot-version=v2.1 Volvox_carteri OG0000055
Vocar.0468s0001.1.p pacid=32886182 transcript=Vocar.0468s0001.1 locus=Vocar.0468s0001 ID=Vocar.0468s0001.1.v2.1 annot-version=v2.1 Volvox_carteri OG0000055
Vocar.0020s0115.1.p pacid=32886784 transcript=Vocar.0020s0115.1 locus=Vocar.0020s0115 ID=Vocar.0020s0115.1.v2.1 annot-version=v2.1 Volvox_carteri OG0000055
Vocar.0020s0010.1.p pacid=32886796 transcript=Vocar.0020s0010.1 locus=Vocar.0020s0010 ID=Vocar.0020s0010.1.v2.1 annot-version=v2.1 Volvox_carteri OG0000055

Vocar.0007s0068.1.p pacid=32888045 transcript=Vocar.0007s0068.1
locus=Vocar.0007s0068 ID=Vocar.0007s0068.1.v2.1 annot-version=v2.1
Volvox_carteri   OG0000055
Vocar.0004s0405.1.p pacid=32889413 transcript=Vocar.0004s0405.1
locus=Vocar.0004s0405 ID=Vocar.0004s0405.1.v2.1 annot-version=v2.1
Volvox_carteri   OG0000055
Vocar.0022s0072.1.p pacid=32891831 transcript=Vocar.0022s0072.1
locus=Vocar.0022s0072 ID=Vocar.0022s0072.1.v2.1 annot-version=v2.1
Volvox_carteri   OG0000055
Vocar.0022s0104.1.p pacid=32891876 transcript=Vocar.0022s0104.1
locus=Vocar.0022s0104 ID=Vocar.0022s0104.1.v2.1 annot-version=v2.1
Volvox_carteri   OG0000055
Vocar.0034s0067.1.p pacid=32893050 transcript=Vocar.0034s0067.1
locus=Vocar.0034s0067 ID=Vocar.0034s0067.1.v2.1 annot-version=v2.1
Volvox_carteri   OG0000055
Vocar.0009s0338.1.p pacid=32893229 transcript=Vocar.0009s0338.1
locus=Vocar.0009s0338 ID=Vocar.0009s0338.1.v2.1 annot-version=v2.1
Volvox_carteri   OG0000055
Vocar.0001s0054.1.p pacid=32884222 transcript=Vocar.0001s0054.1
locus=Vocar.0001s0054 ID=Vocar.0001s0054.1.v2.1 annot-version=v2.1
Volvox_carteri   OG0000055
Vocar.0030s0033.1.p pacid=32894420 transcript=Vocar.0030s0033.1
locus=Vocar.0030s0033 ID=Vocar.0030s0033.1.v2.1 annot-version=v2.1
Volvox_carteri   OG0000055
jgi|ChlNC64A_1|137207|IGS.gm_18_00313   Chlorella_variabilis
OG0000055
jgi|ChlNC64A_1|137973|IGS.gm_2_00440   Chlorella_variabilis
OG0000055
jgi|ChlNC64A_1|49989|fgenesh3_pg.C_scaffold_2000234
Chlorella_variabilis   OG0000055
jgi|ChlNC64A_1|133279|IGS.gm_1_00415   Chlorella_variabilis
OG0000055
jgi|ChlNC64A_1|142412|IGS.gm_4_00553   Chlorella_variabilis
OG0000055
jgi|ChlNC64A_1|143845|IGS.gm_6_00419   Chlorella_variabilis
OG0000055
jgi|ChlNC64A_1|144712|IGS.gm_8_00040   Chlorella_variabilis
OG0000055
jgi|ChlNC64A_1|141313|IGS.gm_33_00023   Chlorella_variabilis
OG0000055
49195 pacid=27387757 transcript=49195
locus=estExt_fgenesh1_pg.C_220107 ID=49195.2.0.227 annot-
version=v2.0   Coccomyxa_subellipsoidea OG0000055
48936 pacid=27388399 transcript=48936
locus=estExt_fgenesh1_pg.C_190114 ID=48936.2.0.227 annot-
version=v2.0   Coccomyxa_subellipsoidea OG0000055
61463 pacid=27389979 transcript=61463 locus=Genemark1.2253_g
ID=61463.2.0.227 annot-version=v2.0   Coccomyxa_subellipsoidea
OG0000055
47154 pacid=27391050 transcript=47154
locus=estExt_fgenesh1_pg.C_60227 ID=47154.2.0.227 annot-version=v2.0
Coccomyxa_subellipsoidea OG0000055
56649 pacid=27391455 transcript=56649 locus=Genemark1.5678_g
ID=56649.2.0.227 annot-version=v2.0   Coccomyxa_subellipsoidea

OG0000055
60280 pacid=27392680 transcript=60280 locus=Genemark1.8596_g
ID=60280.2.0.227 annot-version=v2.0	Coccomyxa_subellipsoidea
OG0000055
11102_fgenesh1_pg.C_Chr_05.0001000237	Ostreococcus_tauri
OG0000055
34655_0900010121 Ostreococcus_tauri	OG0000055
37635_1800010039 Ostreococcus_tauri	OG0000055
9385_fgenesh1_pg.C_Chr_01.0001000151	Ostreococcus_tauri
OG0000055
92987 pacid=27414085 transcript=92987 locus=ost_07_004_099
ID=92987.2.0.231 annot-version=v2.0	Ostreococcus_lucimarinus
OG0000055
31794 pacid=27416842 transcript=31794 locus=eugene.0500010331
ID=31794.2.0.231 annot-version=v2.0	Ostreococcus_lucimarinus
OG0000055
13716 pacid=27418025 transcript=13716
locus=fgenesh1_pg.C_Chr_1000176 ID=13716.2.0.231 annot-version=v2.0
Ostreococcus_lucimarinus	OG0000055
25712 pacid=27418998 transcript=25712
locus=estExt_fgenesh1_pg.C_Chr_170024 ID=25712.2.0.231 annot-
version=v2.0	Ostreococcus_lucimarinus	OG0000055
124209 pacid=27348782 transcript=124249 locus=fgenesh2_kg.
15_#_9_#_4278852:2 ID=124249.3.0.228 annot-version=v3.0
Micromonas_pusilla	OG0000055
54337 pacid=27344850 transcript=54337 locus=MicpuC2.EuGene.
0000170183 ID=54337.3.0.228 annot-version=v3.0
Micromonas_pusilla	OG0000055
193067 pacid=27347138 transcript=193107
locus=estExt_Genemark1.C_3_t20098 ID=193107.3.0.228 annot-
version=v3.0	Micromonas_pusilla	OG0000055
64935 pacid=27397178 transcript=64935 locus=EuGene.1600010296
ID=64935.3.0.229 annot-version=v3.0	Micromonas_sp._RCC299
OG0000055
58198 pacid=27400119 transcript=58198 locus=EuGene.0400010727
ID=58198.3.0.229 annot-version=v3.0	Micromonas_sp._RCC299
OG0000055
100534 pacid=27395831 transcript=100534
locus=fgenesh2_pg.C_Chr_05000227 ID=100534.3.0.229 annot-
version=v3.0	Micromonas_sp._RCC299	OG0000055
61399 pacid=27401386 transcript=61399 locus=EuGene.0900010382
ID=61399.3.0.229 annot-version=v3.0	Micromonas_sp._RCC299
OG0000055
kfl00007_0400_v1.1	Klebsormidium_flaccidum	OG0000055
kfl00324_0180_v1.1	Klebsormidium_flaccidum	OG0000055
Pp3c3_28000V3.1.p pacid=32942174 transcript=Pp3c3_28000V3.1
locus=Pp3c3_28000 annot-version=v3.3	Physcomitrella_patens
OG0000055
Os03t0425000-01 Similar to predicted protein.	Oryza_sativa
OG0000055
evm.model.contig_2095.14	Porphyridium_purpureum	OG0000055
evm.model.contig_2020.24	Porphyridium_purpureum	OG0000055
evm.model.contig_2121.20	Porphyridium_purpureum	OG0000055
evm.model.contig_2142.6	Porphyridium_purpureum	OG0000055

evm.model.contig_2150.9 Porphyridium_purpureum   OG0000055
evm.model.contig_2286.11 Porphyridium_purpureum   OG0000055
evm.model.contig_2316.2 Porphyridium_purpureum   OG0000055
evm.model.contig_2502.4 Porphyridium_purpureum   OG0000055
evm.model.contig_2505.1 Porphyridium_purpureum   OG0000055
evm.model.contig_2707.2 Porphyridium_purpureum   OG0000055
evm.model.contig_3450.24 Porphyridium_purpureum   OG0000055
evm.model.contig_3539.2 Porphyridium_purpureum   OG0000055
evm.model.contig_3551.4 Porphyridium_purpureum   OG0000055
evm.model.contig_4461.5 Porphyridium_purpureum   OG0000055
evm.model.contig_4492.3 Porphyridium_purpureum   OG0000055
evm.model.contig_4495.12 Porphyridium_purpureum   OG0000055
evm.model.contig_522.23 Porphyridium_purpureum   OG0000055
evm.model.contig_568.5   Porphyridium_purpureum   OG0000055
evm.model.contig_570.2   Porphyridium_purpureum   OG0000055
evm.model.contig_596.1   Porphyridium_purpureum   OG0000055
evm.model.contig_620.3   Porphyridium_purpureum   OG0000055
Cre14.g609250.t1.1 pacid=30776728 transcript=Cre14.g609250.t1.1 locus=Cre14.g609250 ID=Cre14.g609250.t1.1.v5.5 annot-version=v5.5 Chlamydomonas_reinhardtii OG0000056
jgi|ChlNC64A_1|134456|IGS.gm_11_00302 Chlorella_variabilis OG0000056
55863 pacid=27385964 transcript=55863 locus=Genemark1.4892_g ID=55863.2.0.227 annot-version=v2.0 Coccomyxa_subellipsoidea OG0000056
43154 pacid=27391465 transcript=43154 locus=fgenesh1_pg.12_#_93 ID=43154.2.0.227 annot-version=v2.0 Coccomyxa_subellipsoidea OG0000056
66858 pacid=27391605 transcript=66858 locus=estExt_Genemark1.C_120087 ID=66858.2.0.227 annot-version=v2.0 Coccomyxa_subellipsoidea OG0000056
43152 pacid=27391697 transcript=43152 locus=fgenesh1_pg.12_#_91 ID=43152.2.0.227 annot-version=v2.0 Coccomyxa_subellipsoidea OG0000056
56731 pacid=27391699 transcript=56731 locus=Genemark1.5760_g ID=56731.2.0.227 annot-version=v2.0 Coccomyxa_subellipsoidea OG0000056
66960 pacid=27391768 transcript=66960 locus=estExt_Genemark1.C_120278 ID=66960.2.0.227 annot-version=v2.0 Coccomyxa_subellipsoidea OG0000056
43198 pacid=27391809 transcript=43198 locus=fgenesh1_pg.12_#_137 ID=43198.2.0.227 annot-version=v2.0 Coccomyxa_subellipsoidea OG0000056
44250 pacid=27394577 transcript=44250 locus=fgenesh1_pg.17_#_5 ID=44250.2.0.227 annot-version=v2.0 Coccomyxa_subellipsoidea OG0000056
kfl00060_0330_v1.1   Klebsormidium_flaccidum OG0000056
kfl00010_0380_v1.1   Klebsormidium_flaccidum OG0000056
Pp3c2_37020V3.1.p pacid=32934201 transcript=Pp3c2_37020V3.1 locus=Pp3c2_37020 annot-version=v3.3 Physcomitrella_patens OG0000056
Pp3c20_4820V3.1.p pacid=32947484 transcript=Pp3c20_4820V3.1 locus=Pp3c20_4820 annot-version=v3.3 Physcomitrella_patens OG0000056

Pp3c5_23290V3.1.p pacid=32952751 transcript=Pp3c5_23290V3.1 locus=Pp3c5_23290 annot-version=v3.3	Physcomitrella_patens OG0000056
Pp3c5_27060V3.1.p pacid=32954704 transcript=Pp3c5_27060V3.1 locus=Pp3c5_27060 annot-version=v3.3	Physcomitrella_patens OG0000056
Pp3c5_27320V3.1.p pacid=32955310 transcript=Pp3c5_27320V3.1 locus=Pp3c5_27320 annot-version=v3.3	Physcomitrella_patens OG0000056
Pp3c5_14230V3.1.p pacid=32955347 transcript=Pp3c5_14230V3.1 locus=Pp3c5_14230 annot-version=v3.3	Physcomitrella_patens OG0000056
[truncated: 522,655 more chars]
